# Polygenic scores for tobacco use provide insights into systemic health risks in a diverse EHR-linked biobank in Los Angeles

**DOI:** 10.1038/s41398-024-02743-z

**Published:** 2024-01-18

**Authors:** Vidhya Venkateswaran, Kristin Boulier, Yi Ding, Ruth Johnson, Arjun Bhattacharya, Bogdan Pasaniuc

**Affiliations:** 1 Department of Pathology and Laboratory Medicine, David Geffen School of Medicine, University of California, Los Angeles, Los Angeles, CA 90095 USA; 2 Department of Oral Biology, School of Dentistry, University of California, Los Angeles, Los Angeles, CA 90095 USA; 3 Office of the Director and National Institute of Dental and Craniofacial Research, National Institutes of Health, Bethesda, MD 20892 USA; 4 Department of Medicine, Division of Cardiology, University of California, Los Angeles, Los Angeles, CA 90095 USA; 5 Bioinformatics Interdepartmental Program, University of California, Los Angeles, Los Angeles, CA 90095 USA; 6 Department of Computer Science, University of California, Los Angeles, Los Angeles, CA 90095 USA; 7 Department of Epidemiology, University of Texas MD Anderson Cancer Center, Houston, TX 77030 USA; 8 Institute for Data Science in Oncology, University of Texas MD Anderson Cancer Center, Houston, TX 77030 USA; 9 Department of Human Genetics, David Geffen School of Medicine, University of California, Los Angeles, Los Angeles, CA 90095 USA; 10 Department of Computational Medicine, David Geffen School of Medicine, University of California, Los Angeles, Los Angeles, CA 90095 USA; 11 Institute of Precision Health, University of California, Los Angeles, Los Angeles, CA 90095 USA

**Keywords:** Addiction, Personalized medicine

## Abstract

Tobacco use is a major risk factor for many diseases and is heavily influenced by environmental factors with significant underlying genetic contributions. Here, we evaluated the predictive performance, risk stratification, and potential systemic health effects of tobacco use disorder (TUD) predisposing germline variants using a European- ancestry-derived polygenic score (PGS) in 24,202 participants from the multi-ancestry, hospital-based UCLA ATLAS biobank. Among genetically inferred ancestry groups (GIAs), TUD-PGS was significantly associated with TUD in European American (EA) (OR: 1.20, CI: [1.16, 1.24]), Hispanic/Latin American (HL) (OR:1.19, CI: [1.11, 1.28]), and East Asian American (EAA) (OR: 1.18, CI: [1.06, 1.31]) GIAs but not in African American (AA) GIA (OR: 1.04, CI: [0.93, 1.17]). Similarly, TUD-PGS offered strong risk stratification across PGS quantiles in EA and HL GIAs and inconsistently in EAA and AA GIAs. In a cross-ancestry phenome-wide association meta-analysis, TUD-PGS was associated with cardiometabolic, respiratory, and psychiatric phecodes (17 phecodes at *P* < 2.7E-05). In individuals with no history of smoking, the top TUD-PGS associations with obesity and alcohol-related disorders (*P* = 3.54E-07, 1.61E-06) persist. Mendelian Randomization (MR) analysis provides evidence of a causal association between adiposity measures and tobacco use. Inconsistent predictive performance of the TUD-PGS across GIAs motivates the inclusion of multiple ancestry populations at all levels of genetic research of tobacco use for equitable clinical translation of TUD-PGS. Phenome associations suggest that TUD-predisposed individuals may require comprehensive tobacco use prevention and management approaches to address underlying addictive tendencies.

## Introduction

Tobacco use causes significant global mortality and morbidity, contributing to several systemic conditions, including cardiometabolic diseases and cancers [1, 2]. Tobacco use has historically been studied as an environmental risk factor for other diseases. However, tobacco use could instead be viewed as a complex psychiatric trait with environmental risk factors [3] and significant genetic contributions [4, 5]. Multi-ancestry genetic studies report an estimated SNP-based heritability (i.e., the proportion of the phenotypic variance explained by genetics) of tobacco use behaviors ranging between 5–18% [4, 5]. Twin and family studies report heritability estimates of 40–56% for cigarette smoking and 72% for nicotine dependence. These family-based heritability estimates vary widely between different tobacco use traits and between males and females [6]. Prevention and management strategies for tobacco use can benefit from precision medicine approaches, with the inclusion of baseline genetic risk to develop individualized preventive and therapeutic strategies for tobacco use. However, these efforts require a thorough understanding of the effects of a genetic predisposition to tobacco use and the impact of tobacco predisposition on the overall systemic health of an individual.

To understand the genetic etiology of tobacco use, researchers use genome-wide association studies (GWAS) to identify single nucleotide polymorphisms (SNPs) associated with tobacco use disorder. GWAS have identified over 2000 loci associated with tobacco use traits, such as smoking behaviors and nicotine dependence [4, 5]. However, single variants rarely capture a large proportion of phenotypic variation for a complex behavioral trait like tobacco use. To capture the overall genetic predisposition to tobacco use, polygenic scores (PGS) aggregate the weighted effects for multiple variants of interest, thus capturing a larger proportion of phenotypic variation than single variants. Polygenic scores have been used in research for disease prediction and to evaluate disease correlations, with the potential for clinical translation to identify high-risk individuals [7]. In particular, tobacco use behaviors have shown genetic correlations with diseases such as schizophrenia and substance use disorders [8–12].

To further contextualize disease-associated genetic variants, phenome-wide association studies (PheWAS) systematically test the association of a single genetic variant across multiple phenotypes [13]. PheWAS potentially identify other traits or disorders upon which the single genetic variant could exert an effect, i.e., horizontal pleiotropic effects of the genetic variant. Generally, PheWAS use phenotypes that are identified using phecodes, or ICD codes that are aggregated into clinically meaningful groupings.

In our analysis, we combined a PGS for tobacco use disorder (TUD) with a PheWAS approach to create a PGS-PheWAS, a powerful way to examine the potential pleiotropic effects of multiple genetic variants that predispose to tobacco use disorder and identify systemic disease risks for individuals with a genetic predisposition to tobacco use [14]. We used a publicly available PGS for tobacco use disorder, developed in European-ancestry individuals in UK Biobank [15] and imputed these scores into the UCLA ATLAS biobank which comprises consented and genotyped UCLA patients representing diverse ancestry groups and phenotypes drawn from their electronic health records [16–20]. We found that the TUD-PGS demonstrated inconsistent predictive performance and risk stratification in non-European ancestry groups within the UCLA ATLAS biobank. In a PGS-PheWAS, we identified several phecodes associated with a genetic predisposition to tobacco use, mainly in cardiometabolic, respiratory, and neuropsychiatric phenotype categories. Next, to separate out the effects of tobacco use behavior from a genetic predisposition to tobacco use, we restricted a PGS-PheWAS to patients with no smoking history and identified persistent associations with obesity and alcohol-related disorders, suggesting shared genetic etiologies for these complex traits. Finally, we used publicly available GWAS summary statistics to perform Mendelian randomization [21] to evaluate the nature of the persistent tobacco use-obesity associations. We found evidence of causality between adiposity measures and tobacco use. Our work underscores the need to expand the diversity of study populations to generalize findings and to equitably translate genetic research to patient care. Further, the potential pleiotropic effects of tobacco-predisposing genetic variants suggest a more comprehensive approach to addressing tobacco use addiction that includes due consideration to other associated behavioral traits.

## Methods

### Study population

All analyses were performed with UCLA ATLAS Biobank data, a biobank embedded within the UCLA Health medical system [16–20]. UCLA Health is a comprehensive healthcare system serving the population in and around the greater Los Angeles area. The UCLA Institute for Precision Health is home to the UCLA ATLAS biobank with >40k participants genotyped, of which 24,202 participants were included in this study. This large-scale collection of genotyped biospecimens is integrated with the UCLA Data Discovery Repository (DDR), containing de-identified patient electronic health records (EHR) which include clinical, procedural, laboratory, prescription, and demographic information.

Final analyses included 24,202 ATLAS participants (7902 cases and 16,283 controls) with complete information on the outcome and covariates including smoking status and insurance information. For ancestry-specific analysis, we included European American (*N* = 15,780), Hispanic/Latin American (*N* = 4412), East Asian American (*N* = 2377), and African American (*N* = 1633) ancestry groups with sufficient sample sizes for analysis.

### Data processing and population stratification

Detailed information on data processing can be found in previous publications [16–20]. Briefly, blood samples were collected from consented participants and genotyped on a custom array [22]. Initial array-level quality control measures included removing strand ambiguous SNPs and variants with >5% missingness and filtering out SNPs that do not pass the Hardy–Weinberg equilibrium test with a p-value set at (“–hwe 0.001”). After restricting to unrelated individuals, the QC-ed genotypes were imputed to the TOPMed Freeze5 reference using the Michigan Imputation Server [23, 24]. The final QC steps were to filter the variants at the threshold of *R*^2^ > 0.90 and minor allele frequency > 1%. All quality control steps were conducted using PLINK 1.9 [25].

We computed the top 10 principal components for the study population using FlashPCA2 software [26]. We then grouped the study population into genetically inferred ancestry groups (GIAs) - European American (EA), Hispanic/Latin American (HL), East Asian American (EAA), African American (AA) - by k-nearest neighbor (KNN) stratification of the principal components, using the continental ancestry populations from 1000 Genomes Project [27, 28] as a reference. To account for differences in population stratification between GIA groups, for the PGS-PheWAS analysis, we conducted individual PGS-PheWAS within each GIA group and then meta-analyzed across GIA groups to obtain cross-ancestry results.

### Polygenic score imputation within UCLA ATLAS biobank

We used a publicly available polygenic score trained on 391,124 European individuals (21,954 cases and 35,7624 controls) from the UK biobank for the trait ‘tobacco use disorder’ from the PGS catalog (PGS002037) [15, 29]. This trait, ‘tobacco use disorder’ was identified using phecode 318.0 which corresponds to ICD codes F17.0, F17.1, F17.2, F17.3, F17.4, F17.9, Z72.0, 305.1, 305.10, 305.11, 305.12, 305.13, 649.0, 649.00, 649.01, 649.02, 649.03, 649.04 and V15.82. This PGS was selected for two reasons: (1) the PGS was trained on the same phecode for TUD that is available in ATLAS and (2) there is a high degree of overlap with ATLAS genotyped variants (800,381 of 847,691 total variants in TUD-PGS overlapping with ATLAS data - 94.4% overlap). The original PGS training analyses were performed using LDpred2 [30] and adjusted for the following covariates: sex, age, birth date, Townsend’s deprivation index, and the first 16 principal components of the genotype matrix. We computed the PGS for each ATLAS participant by multiplying the individual risk allele dosages by their corresponding weights that are provided by the PGS catalog [29]. The PGS was mean-centered and standardized by the standard deviation within each GIA group to generate a PGS Z-score.

We also tested the predictive performance of 16 multi-ancestry PGS from Saunders et al, Nature 2022 [5], trained on European, Admixed, East Asian and African ancestry populations for traits ‘Smoking initiation’, ‘Age of smoking initiation’, ‘Cigarettes smoked per day’ and, ‘Smoking cessation’. We downloaded these PGS (PGS003357- PGS003372) from the PGS Catalog [29] and tested their predictive performance on 4 genetically inferred ancestry groups within ATLAS for phecode 318.0 for tobacco use disorder, since we do not have information on the traits that the PGS were originally trained in.

### Phecodes

ICD9 and ICD10 billing codes were aggregated into clinically meaningful groupings called phecodes using mappings derived from the PheWAS catalog, v1.2 [31]. Cases were defined by the presence of an ICD code tagged by the respective phecode and controls by the absence of the ICD codes. Tobacco use disorder diagnosis was derived from the presence of “tobacco use disorder” phecode (318.00) within an individual’s health record which groups ICD codes (F17.200, F17.201, F17.210, F17.211, F17.220, F17.221, F17.290, F17.291, O99.33, O99.330, O99.331, O99.332, O99.333, O99.334, O99.335, Z87.891) for tobacco use disorder (TUD). For the PheWAS analysis, we used 1847 phecodes, extracted from each individual’s health record as described above, to capture phenotypes across the phenotypic spectrum [31].

### Statistical analysis

All analysis was conducted in either Python 2.6.8 [32] or R 4.2.1 [33].

#### Predictive performance and risk stratification

We analyzed the predictive performance of the standardized TUD-PGS across ancestry groups and risk quantiles using GIA-stratified logistic regression models, with the phecode for TUD as the outcome and with predictors including terms for age, sex, the first five principal components of the genotype matrix, and insurance class.

We include insurance class information as the closest proxy to bias introduced by participation and access to healthcare within the de-identified electronic health records [34]. This insurance class variable consists of the type of insurance used by the patient in their clinical encounters. The classes include “Public”, “Private” or “Self-pay”. Public class includes ‘Medicare’, ‘Medicare Advantage’, ‘Medicare Assigned’, ‘Medi-Cal’, ‘Medicaid’, and ‘Medi-Cal Assigned’. Private class includes ‘International Payor’, ‘Group Health Plan’, ‘Worker’s Comp’, ‘Tricare’, ‘UCLA Managed Care’, ‘Blue Shield’, ’Commercial’, ’Blue Cross’, ‘Package Billing’ and ‘Other’.

Odds ratios were calculated within each GIA, with *P*-values from Wald-type test statistics and a Bonferroni-corrected significance level of 0.0125 = (0.05/4). For risk stratification analysis, we grouped individuals of each GIA group into 5 groups of equal size based on their PGS and compared the quintile with the highest score with the quintile with the lowest scores. This model can be represented as

$${Tobacco}\,{use}\,{disorder}\,{phecode}\,(318.0) \sim {PGS}{\rm{\_}}Z({or})\,{PGS}\,{quintile}+{Age}+{Sex}+{PCs}1-5+{Insurance}\,{Class}$$

#### Phenome-wide association meta-analysis

For the phenome-wide association analysis, we tested the association between the standardized TUD-PGS and 1847 electronic health record-derived phecodes across the phenome. Each GIA-specific PheWAS analysis consisted of logistic regressions across 1847 EHR-derived phecodes, controlling for age, sex, first 5 PCs, and insurance class. For the cross-ancestry meta-analysis, we use the PGS-PheWAS results computed within each GIA group and meta-analyze across these ancestry groups using a random effect, inverse variance weighted model using the metafor (version 3.4) package in R [35]. We use a phenome-wide Bonferroni-corrected p-value significance threshold of 2.7e-05 to adjust for the multiple testing burden (*P* = 0.05/1847 tests for each trait identified by phecodes). The never-smoker analysis followed a similar analysis plan, restricted to individuals of European American GIA with no history of smoking recorded by their provider within their medical records (*n* = 9921).

#### Mendelian randomization

We evaluated causality using Mendelian Randomization (MR) methods to test for and evaluate the causality between tobacco use and obesity [21]. We used summary statistics from GSCAN Consortium GWAS for “Cigarettes Smoked Per Day” (249,752 participants of European Ancestry and 12,003,613 SNPs) [36] and summary statistics from MRC Integrative Epidemiology Unit - the University of Bristol and UKBB GWAS for “Waist Circumference” (462,166 participants of European Ancestry and 9,851,867 SNPs) [37] as the instrumental variables to test the causal association between tobacco use and obesity measures. We performed a second MR analysis to validate the previous analysis using summary statistics for ‘Body Mass Index - BMI’ using summary statistics from UK Biobank [37] (461,460 individuals and 9,851,867 SNPs), using the same ‘Cigarettes smoked per day’ summary statistics from GSCAN as the outcome.

Lastly, GSCAN consortium and UK Biobank have approximately 35% sample overlap and hence we also tested this association using summary statistics for BMI from GIANT consortium (322,154 individuals and 2,554,668 SNPs) [38]. We used the ‘TwoSampleMR’ R package to extract instruments, harmonize and obtain effect sizes from multiple MR methods (MR Egger, Weighted median, Inverse variance weighted, Simple mode, and Weighted mode) [39].

## Results

### Baseline characteristics of included ATLAS Biobank participants

The final analysis included *n* = 24,202 individuals with complete information on all covariates. Within the “TUD” phecode, the study population consisted of 7902 cases and 16,283 controls. The average age of individuals with a TUD phecode was 64.3 years. Participant sex was significantly associated with TUD phecode with 55.1% of the phecode represented by the male sex. Four genetically inferred ancestry groups had sufficient sample size to perform the analyses: European American (EA), Hispanic/Latin American (HL), East Asian American (EAA), and African American ancestry (AA) (*n* = 15,780, 4412, 2377, and 1633, respectively). Table 1 summarizes the demographics of the study sample.

**Table 1:** Baseline characteristics of ATLAS participants included in this study.

		Overall
*n*		24,202
Age, median [Q1,Q3]		61.0 [46.0,72.0]
Sex, *n* (%)	Female	13,277 (54.9)
	Male	10,914 (45.1)
Insurance class, *n* (%)	Private	14,996 (62.0)
	Public	8431 (34.8)
	Self-Pay	775 (3.2)
Tobacco use disorder, *n* (%)	Controls	16,283 (67.3)
	Cases	7902 (32.7)
Genetically Inferred Ancestry, *n* (%)	African American (AA)	1633 (6.7)
	Hispanic/Latin American (HL)	4412 (18.2)
	East Asian American (EAA)	2377 (9.8)
	European American (EA)	15,780 (65.2)

### Prediction and risk stratification of TUD using TUD-PGS across genetically inferred ancestry groups

We first evaluated how well the TUD-PGS predicts TUD across the multi-ancestry study sample within the ATLAS biobank. The TUD-PGS associated significantly with the phecode for TUD within the ATLAS biobank for individuals of European American (EA) GIA (OR:1.20, CI: [1.16, 1.24]), showing an increase in odds of TUD by 20% for each standard deviation increase in the TUD-PGS. Similarly, we observed significant associations between TUD-PGS and TUD among Hispanic/Latin American (HL) GIA (OR:1.19, CI: [1.11, 1.28]), and East Asian American (EAA) GIA groups (OR: 1.18, CI: [1.06, 1.31]). However, the TUD-PGS was not associated with TUD in individuals of African American (AA) GIA group (OR: 1.04, CI: [0.93, 1.17]). Supplementary Table 1 summarizes these associations.

In addition, we used multi-ancestry PGS (PGS003357- PGS003372) and tested their predictive performance in the ancestry group corresponding to their training group. These PGS showed inconsistent albeit significant associations in EA GIA and insignificant associations in non-European GIAs with TUD in ATLAS (Supplementary Table 2).

**Fig. 1:**
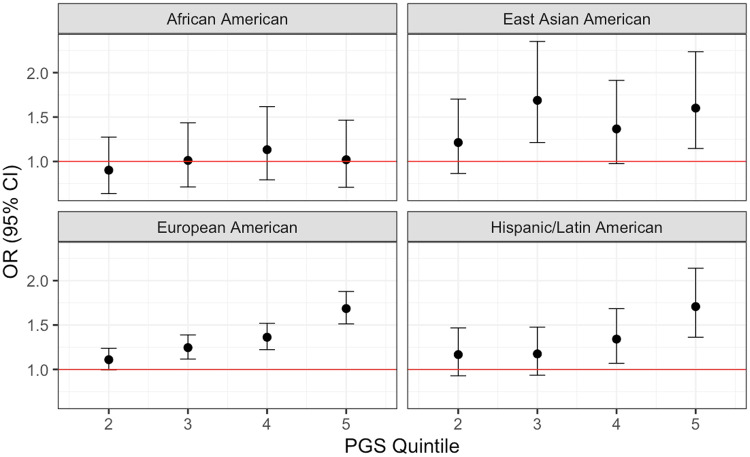
TUD-PGS correlates with TUD phecode in EA, HL, and EAA ancestries across risk quintiles. The X-axis represents the top 4 quintiles grouped according to TUD-PGS. Y axis represents effect sizes represented by odds ratios. The red line indicates OR = 1. Effect sizes between TUD-PGS and TUD phecode vary across PGS quintiles in 4 genetically inferred ancestry groups with strong risk stratification noted in EA and HL and inconsistent risk stratification in AA and EAA groups.

Next, we assessed if the TUD-PGS could stratify individuals by risk for tobacco use disorder. Based on TUD-PGS, we divided the study sample into quintiles and estimated the odds ratio of TUD for each quintile compared to the bottom quintile. When compared to the quintile with the lowest TUD-PGS, the quintile with the highest TUD-PGS demonstrated an OR = 1.69 (CI: [1.51, 1.88]) in EA and 1.71 (CI: [1.36, 2.14]) in HL ancestry groups. The TUD-PGS offered strong risk stratification for individuals of EA GIA and for the top two risk quintiles in HL. Risk stratification was weaker and inconsistent in the EAA, (OR = 1.60, CI = [1.15, 2.24]) and AA ancestry groups (OR = 1.02, CI = [0.71, 1.47]) (Fig. 1, Supplementary Table 3). This TUD-PGS risk stratifies individuals in EA and HL ancestry groups, potentially identifying individuals at a higher risk of tobacco use disorder within these ancestry groups. However, this risk stratification was inconsistent or absent in EAA and AA ancestry groups.

### Systemic comorbidities in TUD-predisposed individuals identified by TUD-PGS- PheWAS

**Fig. 2:**
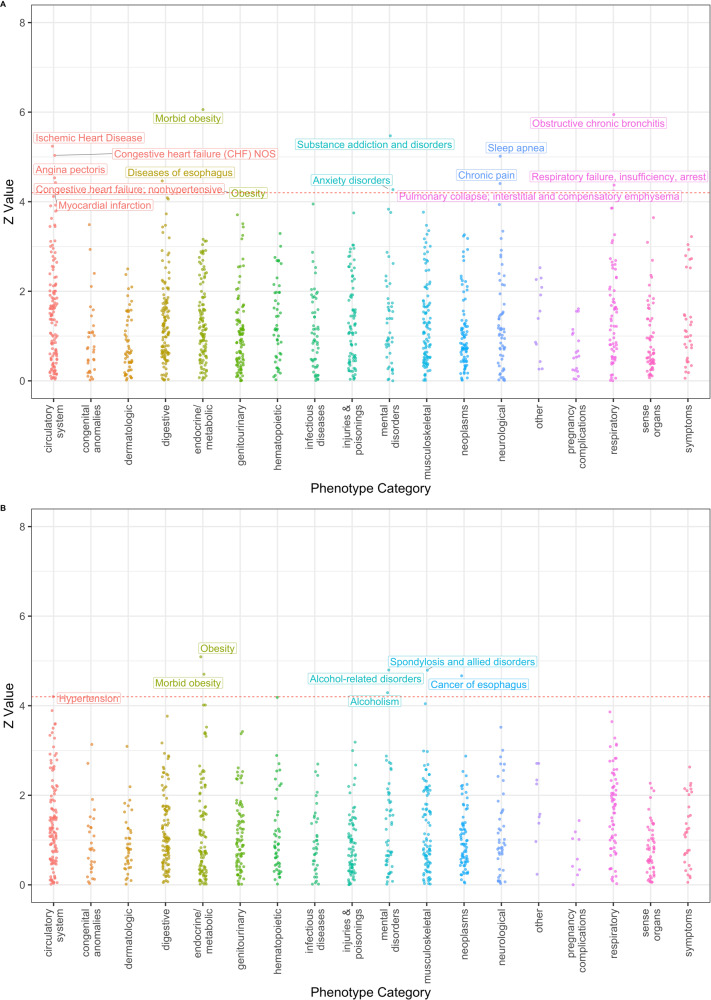
Phenome-wide associations for TUD-PGS. **A** TUD-PGS-PheWAS plot across 1847 phecodes (cross-ancestry meta-analysis). Associations between TUD-PGS and 1847 phecodes across the phenome, meta-analyzed across 4 GIA groups with significant associations labeled. The X-axis represents the *Z* value (beta/SE). Each color represents a phecode category and each dot represents a phecode. Phenome-wide significance is represented by the red dashed line at a *Z* value = 4.2 which corresponds to a *P* value of 2.57e-5 (1847 tests/0.05). Top associations were noted in circulatory, metabolic, mental and respiratory phenotype categories. **B** TUD-PGS-PheWAS plot across 1847 phecodes in never smokers of EA ancestry group. Associations between TUD-PGS and 1847 phecodes across the phenome in never smokers of EA ancestry with significant associations labeled. The X-axis represents the *Z* value (beta/SE). Each color represents a phecode category and each dot represents a phecode. Phenome-wide significance is represented by the red dashed line at a *Z* value = 4.2 which corresponds to a *P* value of 2.57e-5 (1847 tests/0.05). In TUD-PGS-PheWAS restricted to ‘never-smokers’, top associations were obesity and alcohol-related disorders.

Next, we systematically evaluated associations between a genetic predisposition to TUD with 1847 traits or diseases across the phenome. The TUD-PGS captures the genetic predisposition to TUD and the 1847 traits are captured using phecodes extracted from each individual’s electronic health record. In a PheWAS of the TUD-PGS across 1847 phecodes (Supplementary Fig. 1a), meta-analyzed across 4 GIAs, we found 17 significant associations at Bonferroni-adjusted *P* < 0.05 after adjusting for age, sex, first 5 principal components of the genotype matrix, and health insurance information. The top phecodes associated with the TUD-PGS were ‘morbid obesity’, ‘obstructive chronic bronchitis’, ‘substance addiction and disorders’, and ‘ischemic heart disease’ (*P* = 1.38E-09, 2.73E-09, 4.45E-08, 1.61E-07) (Fig. 2a). Phecode categories with multiple associations were circulatory (*n* = 5), respiratory (*n* = 3), neurological (*n* = 2), and metabolic (*n* = 2) phenotypes (Supplementary Table 4). The results of this analysis systematically identify the health risks associated with a genetic predisposition to tobacco use captured by the PGS.

However, it must be noted that these associations may reflect the traits and diseases associated with tobacco use behavior, which lie on the TUD-PGS to trait/disease pathway (Supplementary Fig. 1b). To study the potential pleiotropic effects of germline variants that predispose to TUD, we leveraged the fact that individuals with genetic predisposition to TUD may choose not to engage in tobacco use behaviors. We can thus account for the effect of tobacco use behavior to identify systemic risks of TUD genetic predisposition by stratifying to individuals with no smoking history recorded in their electronic health records. Accordingly, we repeated the PGS-PheWAS association analysis, restricting to “never-smokers” in individuals of EA ancestry, i.e. individuals who reported that they have never smoked tobacco (Supp Fig. 1b). In this analysis, the TUD-PGS demonstrated associations with obesity, alcohol-related disorders, cancer of the esophagus, and hypertension (*P* = 3.54E-07, 1.61E-06, 3.05E-06, 2.62E-05) (Fig. 2b, Supplementary Table 5).

**Fig. 3:**
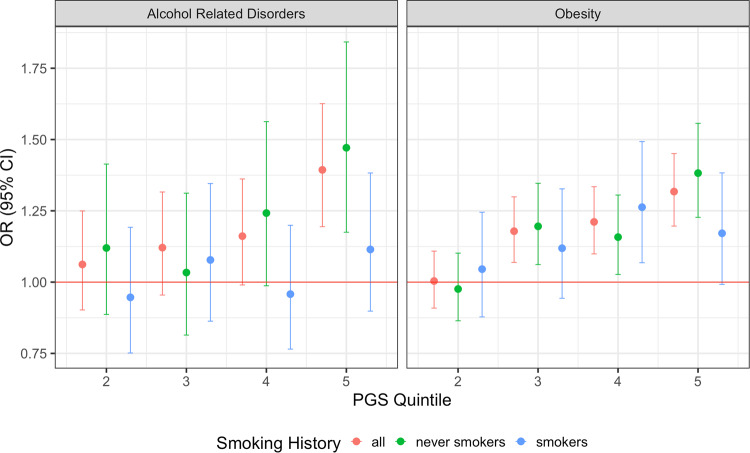
TUD-PGS associations with alcohol-related disorders and obesity among all vs ever vs never-smokers across TUD-PGS quintiles. Associations between TUD-PGS quintiles and Alcohol-related disorders (phecode = 317.0) and Obesity (phecode = 278.1). The X-axis represents the top 4 quintiles grouped according to TUD-PGS. Y axis represents effect sizes represented by odds ratios. The red line indicates OR = 1. TUD-PGS risk stratifies for the phecodes for alcohol-related disorders and obesity in ‘never-smokers’ but not in ‘ever-smokers’.

In an evaluation of the trends of obesity and alcohol-related disorders across quintiles of the TUD-PGS, we observed higher ORs among never-smokers compared to ever-smokers for obesity and alcohol-related disorders. TUD-PGS offered inconsistent risk stratification for obesity and alcohol-related disorders in ever-smokers, or individuals with a history of smoking (Fig. 3). In contrast, a reverse trend is noted in lung cancer, an established trait associated with smoking behavior, which can thus serve as a negative control, where we observed higher ORs in ever-smokers compared to never-smokers. (Supplementary Fig. 2, Supplementary Table 6) We can conclude from this analysis that, individuals predisposed to TUD show associations with obesity and alcohol-related disorder even in the absence of tobacco use behavior.

### Mendelian randomization analysis finds evidence of causality in the association between obesity and tobacco use

To evaluate if the association between obesity and tobacco use can be given a directional and causal interpretation, we performed Mendelian randomization (MR) analysis between quantitative measures of obesity and tobacco use using publicly available GWAS of “waist circumference” [36] and “cigarettes smoked per day” [35]. From the results of multiple MR methods, we observed that the exposure “waist circumference” demonstrated significant positive causal associations with the outcome “cigarettes smoked per day” across all methods used to test this association (MR Egger, Weighted median, Inverse variance weighted, Simple mode, Weighted mode with P = 2.39E-03, 1.50E-32, 1.49E-46, 8.22E-05, 2.05E-08, respectively). A second MR analysis of “body mass index” as the exposure and “cigarettes smoked per day” as the outcome showed similar positive causal associations (MR Egger, Weighted median, Inverse variance weighted, Simple mode, Weighted mode *P* = 2.65E-03, 8.34E-33, 1.17E-45, 8.23E-06, 5.78E-07). An MR analysis of the reverse direction, with “cigarettes smoked per day” as the exposure and “waist circumference” and “body mass index” as outcomes did not show significant causal effects. Supplementary Fig. 3a, b presents the causal effect estimates and confidence intervals. In a subsequent MR analysis in both directions using summary statistics for BMI from GIANT consortium, we find similar results, shown in Supplementary Table 7.

## Discussion

In this study, we examined the predictive performance and risk stratification of a publicly available, European ancestry PGS for tobacco use disorder in a multi-ancestry EHR-linked biobank. Our results demonstrated that this TUD-PGS predicts TUD and risk stratifies European American and Hispanic/Latin GIA groups. However, inconsistent prediction and risk stratification was noted in the East Asian American and African American GIA groups.

Based on these results, we anticipate two issues if TUD-PGS is used clinically to identify individuals at high risk for tobacco use or to predict tobacco use in individuals. First, the risk stratification offered only to certain ancestry populations does not allow for equitable clinical translation of genetic research. Second, the application of these PGS to individual-level clinical decisions must proceed with caution with additional extensive validation with clinical history. At present, we do not recommend interventions solely based on being classified as “high risk” by TUD-PGS due to large uncertainty in imputed polygenic scores at an individual level [40] and inconsistent performance in non-European populations.

Next, we evaluated the potential pleiotropic effects of TUD predisposing variants using the PGS to conduct a phenome-wide association analysis. Additionally, we repeated this analysis in a subgroup of participants without a reported history of smoking behavior, to evaluate the systemic associations of a genetic predisposition to tobacco use in the absence of tobacco use behavior. The PGS-PheWAS cross-ancestry meta-analysis demonstrated significant associations with respiratory and cardiovascular phenotypes, both of which have robust clinical and biological evidence [41, 42]. Other significant associations were in the category of psychiatric disorders, including associations with anxiety disorders and substance addiction disorders. These psychiatric disorder associations have been consistently reported in past genetic studies of smoking and tobacco use [43].

In the PGS-PheWAS analysis of never-smokers, phenotypes associated with tobacco use behaviors, namely, respiratory and cardiovascular phecodes, did not demonstrate statistical significance. Instead, we observed associations with psychiatric phecodes including alcohol-related disorders, and metabolic phecodes with potential behavioral contributions such as obesity. The MR analysis results suggest a causal association between adiposity and tobacco use, in line with other published literature with similar directionality and effect sizes [44]. Together, the associations between tobacco use, obesity, and alcoholism are suggestive of shared genetic architecture between these traits, likely originating from the biological regulation of impulsivity and addictive behaviors [45].

While this TUD-PGS cannot yet be translated clinically, these findings nevertheless have implications for patients with tobacco use disorder. We demonstrate the systemic comorbidities associated with a genetic propensity to TUD. Additionally, we demonstrate that genetically predisposed individuals may be at risk for obesity and alcohol use disorder even when tobacco use behavior is absent. For patients in the TUD high-risk genetic propensity group, these findings would necessitate broadening the focus of the preventive and therapeutic strategy to include a more comprehensive regulation of biological pathways that underlie addiction and impulsivity.

A major strength of this study is that we evaluated TUD-PGS in an information-rich biobank across multiple genetically inferred ancestry groups. The rich phenotypic information available in the biobank allowed us to test associations across the phenome in a hypothesis-free manner, allowing for the discovery of disease associations. Another strength of the paper is that we accounted for possible confounding bias introduced by participation/access to healthcare bias, which can arise from using data from a hospital-based biobank, by using an insurance class variable as a proxy marker for participation and access.

Previous work has shown that PGS accuracy decreases linearly when there is a large difference in genetic ancestry between the training sample and the target sample. These differences in performance lead to bias and imprecision in risk stratification when PGS are applied clinically for complex traits such as TUD. Our results add to these results and motivate more sophisticated computational methods to improve the portability of PGS, especially for complex traits, like TUD, that are influenced greatly by both genetics and the environment and are risk factors for other diseases.

We conclude with limitations and future considerations of our work. Our study included a multi-ancestry sample of patients, but non-European ancestries are represented at smaller sample sizes for most analyses using the UCLA ATLAS biobank. With continued enrollment, we hope to increase the non-European sample sizes and evaluate differential genetic effects in these ancestries. Next, phecodes are derived from ICD codes which are billing codes and, accordingly, may not always capture the full extent of an individual’s disease history. The interpretations of our analyses are within the limitations of these phenotype definitions. We emphasize that the risk of having a phecode in the electronic health record does not accurately reflect the risk of having the disease. Phecode assignments come with biases, including access to healthcare. We have attempted to address this bias introduced by healthcare access by including an insurance class information variable. Nevertheless, this difference must be considered when applying these results to the general population. Lastly, the MR analysis has a partial sample overlap which might offer biased results. However, subsequent analysis with summary statistics from GWAS without sample overlap demonstrates similar results as the original MR analysis, supporting a conclusion of a potential causal association between measures of adiposity and tobacco use.

The results of our study have implications for public health and clinical approaches to the treatment of tobacco use disorder. Future research should strive to improve the prediction and risk stratification of TUD-PGS in all ancestry groups. With consistent performance across ancestry groups and improved individual-level prediction, TUD-PGS can be useful to identify individuals who can benefit from comprehensive preventive and therapeutic strategies to manage their underlying addictive tendencies. Given the growing evidence on health risks associated with obesity and tobacco use, our results suggest possible shared genetic etiology between these two risk factors, strengthening the argument that public health approaches must consider this shared risk while formulating interventions.

### Supplementary information

Supplementary Tables

Supplementary Figures

Supplementary material Part 1

Supplementary material Part 2

## Data Availability

All data produced in the present work are contained in the manuscript.
