# Supplementary material for: Polygenic scores for tobacco use provide insights into systemic health risks in a diverse EHR-linked biobank in Los Angeles
Source: Transl Psychiatry. 2024 Jan 18;14:38. doi: 10.1038/s41398-024-02743-z (PMC10796315; doi:10.1038/s41398-024-02743-z)
Supplement: Supplementary file 1 — Supplementary Tables [file 41398_2024_2743_MOESM1_ESM.pdf]

Supplementary Tables

Supplementary Table 1 - TUD-PGS association with TUD across GIAs

GIA	β	SE	Z	P>|z|	[0.025	0.975]	OR	OR_lower_CI	OR_upper_CI
European American	0.18	0.02	10.44	1.66E-25	0.15	0.22	1.20	1.16	1.24
Hispanic/Latin American	0.17	0.04	4.73	2.24E-06	0.10	0.24	1.19	1.11	1.28
East Asian American	0.17	0.05	3.10	1.93E-03	0.06	0.27	1.18	1.06	1.31
African American	0.04	0.06	0.66	5.07E-01	-0.08	0.16	1.04	0.93	1.17

Supplementary Table 2
Evaluation of predictive performance of 16 multiancestry PGS for phecode for 'tobacco use disorder' (TUD) in ATLAS

Associations of PGS002037 (trained for TUD) for TUD in ATLAS

Trained Phenotype	Training Pop	Testing Pop in ATLAS	PGS	coef	std err	z	P>|z|	[0.025	0.975]	% SNP overlap with ATLAS variants
TUD	European	European American	PGS002037	0.20	0.02	10.44	<0.0001	0.16	0.24	94.4
TUD	European	Admixed American	PGS002037	0.21	0.04	4.99	<0.0001	0.13	0.29	94.4
TUD	European	East Asian American	PGS002037	0.20	0.07	3.01	0.003	0.07	0.33	94.4
TUD	European	African American	PGS002037	0.04	0.08	0.54	0.59	-0.12	0.20	94.4

Associations of the multi-ancestry PGS for TUD - Trained in European ancestry, tested in European ancestry in ATLAS

Trained Phenotype	Training Pop	Testing Pop in ATLAS	PGS	coef	std err	z	P>|z|	[0.025	0.975]	% SNP overlap with ATLAS variants
Smoking initiation (ever smoked	European	European American	PGS003360	-0.34	0.02	-18.64	<0.0001	-0.37	-0.30	93

regularly)										
Age of smoking initiation	European	European American	PGS003364	0.14	0.017	7.87	<0.0001	0.10	0.17	93
Cigarettes smoked per day	European	European American	PGS003368	-0.11	0.018	-6.25	<0.0001	-0.15	-0.08	93
Smoking cessation	European	European American	PGS003372	-0.11	0.019	-5.55	<0.0001	-0.14	-0.07	93

Associations of the multi-ancestry PGS for TUD- Trained in Admixed ancestry, tested in Admixed American ancestry in ATLAS

Smoking initiation	Admixed	Admixed American	PGS003358	-0.09	0.05	-1.85	0.07	-0.19	0.006	92.2
Age of smoking initiation	Admixed	Admixed American	PGS003362	-0.06	0.04	-1.665	0.1	-0.14	0.01	92.7
Cigarettes smoked per day	Admixed	Admixed American	PGS003366	0.01	0.04	0.202	0.84	-0.07	0.08	92
Smoking cessation	Admixed	Admixed American	PGS003370	0.02	0.04	0.53	0.6	-0.06	0.11	92.2

Associations of the multi-ancestry PGS for TUD - Trained in East Asian ancestry, tested in East Asian American ancestry in ATLAS

Smoking initiation	East Asian	East Asian American	PGS003359	-0.22	0.06	-3.85	<0.0001	-0.33	-0.10	94.1
Age of smoking initiation	East Asian	East Asian American	PGS003363	-0.03	0.06	-0.56	0.57	-0.14	0.08	94.2
Cigarettes smoked per day	East Asian	East Asian American	PGS003367	-0.05	0.06	-0.86	0.39	-0.16	0.06	94.2
Smoking cessation	East Asian	East Asian American	PGS003371	-0.08	0.05	-1.54	0.12	-0.18	0.02	94.1

Associations of the multi-ancestry PGS for TUD - Trained in African ancestry, tested in African American ancestry in ATLAS

Smoking initiation	African	African American	PGS003357	-0.08	0.10	-0.74	0.46	-0.28	0.13	85.9
Age of smoking initiation	African	African American	PGS003361	-0.005	0.07	-0.07	0.94	-0.15	0.14	85.9

Cigarettes smoked per day	African	African American	PGS003365	0.09	0.06	1.56	0.12	-0.02	0.20	85.8
Smoking cessation	African	African American	PGS003369	-0.18	0.06	-2.88	0.004	-0.29	-0.06	85.9

Supplementary Table 3 - TUD-PGS association with TUD across quintiles and GIAs

PGS_ Quantile	Coeff	SE	Z	P>|z|	[0.025	0.975]	OR	OR_ lower_CI	OR_ upper_CI	GIA
2	0.10	0.06	1.86	6.27E-02	-0.005	0.21	1.11	0.99	1.24	European American
3	0.22	0.06	3.95	7.70E-05	0.11	0.33	1.25	1.12	1.39	European American
4	0.31	0.06	5.58	2.47E-08	0.20	0.42	1.36	1.22	1.52	European American
5	0.52	0.06	9.45	3.25E-21	0.41	0.63	1.69	1.51	1.88	European American
2	0.15	0.12	1.32	1.86E-01	-0.07	0.38	1.17	0.93	1.47	Hispanic/ Latin American
3	0.16	0.12	1.39	1.66E-01	-0.07	0.39	1.18	0.94	1.48	Hispanic/ Latin American
4	0.29	0.12	2.53	1.13E-02	0.07	0.52	1.34	1.07	1.68	Hispanic/ Latin American
5	0.54	0.12	4.65	3.27E-06	0.31	0.76	1.71	1.36	2.14	Hispanic/ Latin American
2	0.19	0.17	1.12	2.64E-01	-0.15	0.53	1.21	0.86	1.70	East Asian American
3	0.52	0.17	3.1	1.94E-03	0.19	0.85	1.69	1.21	2.35	East Asian American
4	0.31	0.17	1.82	6.87E-02	-0.02	0.65	1.37	0.98	1.91	East Asian American
5	0.47	0.17	2.76	5.77E-03	0.14	0.80	1.60	1.15	2.23	East Asian American
2	-0.10	0.18	-0.59	5.58E-01	-0.45	0.24	0.90	0.63	1.27	African American

3	0.01	0.18	0.06	9.49E-01	-0.34	0.36	1.01	0.71	1.43	African American
4	0.12	0.18	0.69	4.93E-01	-0.23	0.48	1.13	0.79	1.62	African American
5	0.02	0.18	0.10	9.18E-01	-0.34	0.38	1.02	0.71	1.47	African American

Supplementary Table 4 - Significant associations between TUD-PGS and 1847 traits in the PGS-PheWAS cross-ancestry meta-analysis

Phecode	beta	SE	Z	P Value	CI.LB	CI.UB	QEp	Phenotype	Category
278.11	0.12	0.02	6.06	1.38E-09	0.08	0.17	0.46	Morbid obesity	endocrine/metabolic
496.21	0.25	0.04	5.95	2.73E-09	0.17	0.33	0.10	Obstructive chronic bronchitis	respiratory
316	0.12	0.02	5.47	4.45E-08	0.08	0.16	0.56	Substance addiction and disorders	mental disorders
411	0.09	0.02	5.24	1.61E-07	0.05	0.12	0.81	Ischemic Heart Disease	circulatory system
228.1	-0.10	0.02	-5.09	3.49E-07	-0.14	-0.06	0.74	Hemangioma of skin and subcutaneous tissue	neoplasms
428.1	0.12	0.02	5.03	4.80E-07	0.07	0.16	0.33	Congestive heart failure (CHF) NOS	circulatory system
327.3	0.08	0.02	5.02	5.29E-07	0.05	0.11	0.23	Sleep apnea	neurological
228	-0.09	0.02	-4.78	1.74E-06	-0.13	-0.05	0.76	Hemangioma and lymphangioma, any site	neoplasms
411.3	0.12	0.03	4.53	5.83E-06	0.07	0.17	0.71	Angina pectoris	circulatory system
530	0.06	0.01	4.46	8.05E-06	0.03	0.09	0.31	Diseases of esophagus	digestive
428	0.09	0.02	4.42	9.57E-06	0.05	0.14	0.99	Congestive heart failure; nonhypertensive	circulatory system
338.2	0.06	0.01	4.41	1.05E-05	0.03	0.09	0.38	Chronic pain	neurological
509	0.09	0.02	4.37	1.24E-05	0.04817	0.13	0.96	Respiratory failure,	respiratory

								insufficiency, arrest	
278.1	0.11	0.03	4.34	1.42E-05	0.064552	0.17	0.12	Obesity	endocrine/metabolic
411.2	0.11	0.03	4.34	1.45E-05	0.060558	0.16	0.67	Myocardial infarction	circulatory system
300	0.06	0.01	4.27	1.95E-05	0.031596	0.09	0.87	Anxiety disorders	mental disorders
508	0.07	0.02	4.24	2.23E-05	0.035846	0.097	0.53	Pulmonary collapse; interstitial and compensatory emphysema	respiratory

Supplementary Table 5 - Significant associations between TUD-PGS and 1847 traits in the 'never-smoker' PGS-PheWAS in EA ancestry group

Phecode	Coef	SE	Z	P Value	[0.025	0.975]	Phenotype	Category
278.1	0.13	0.03	5.09	3.54E-07	0.08	0.18	Obesity	endocrine/metabolic
317	0.23	0.05	4.8	1.61E-06	0.14	0.32	Alcohol-related disorders	mental disorders
721	0.12	0.03	4.79	1.64E-06	0.074	0.17	Spondylosis and allied disorders	musculoskeletal
278.11	0.16	0.03	4.70	2.56E-06	0.09	0.23	Morbid obesity	endocrine/metabolic
150	0.71	0.15	4.67	3.05E-06	0.41	1.01	Cancer of esophagus	neoplasms
228	-0.12	0.03	-4.58	4.67E-06	-0.17	-0.07	Hemangioma and lymphangioma, any site	neoplasms
317.1	0.23	0.05	4.29	1.78E-05	0.12	0.33	Alcoholism	mental disorders
401	0.09	0.02	4.20	2.62E-05	0.05	0.14	Hypertension	circulatory system

Supplementary Table 6 - Associations between Alcohol-Related Disorders, Obesity, and Lung cancer and PGS quantiles traits in the 'ever-smoker' and 'never-smoker' groups

PGS_Quantile	Coef.	SE	Z	P>|z|	[0.025	0.975]	Phecode	Smoking History	OR	OR_Lower_CI	OR_Upepr_CI	Phenotype
2	0.004	0.05	0.08	9.40E-01	-0.095	0.10	278.1	all	1.00	0.91	1.11	Obesity
3	0.16	0.05	3.31	9.38E-04	0.07	0.26	278.1	all	1.18	1.07	1.30	Obesity

4	0.19	0.05	3.87	1.11E-04	0.09	0.29	278.1	all	1.21	1.1	1.33	Obesity
5	0.28	0.05	5.61	2.02E-08	0.18	0.37	278.1	all	1.32	1.2	1.45	Obesity
2	0.04	0.09	0.50	6.17E-01	-0.13	0.22	278.1	smokers	1.05	0.88	1.25	Obesity
3	0.11	0.09	1.29	1.97E-01	-0.06	0.28	278.1	smokers	1.12	0.94	1.33	Obesity
4	0.23	0.09	2.73	6.34E-03	0.07	0.40	278.1	smokers	1.26	1.07	1.49	Obesity
5	0.16	0.08	1.87	6.22E-02	-0.008	0.32	278.1	smokers	1.17	0.99	1.38	Obesity
2	-0.02	0.06	-0.40	6.93E-01	-0.15	0.1	278.1	never smokers	0.98	0.86	1.10	Obesity
3	0.18	0.06	2.95	3.22E-03	0.06	0.30	278.1	never smokers	1.20	1.06	1.35	Obesity
4	0.15	0.06	2.4	1.67E-02	0.027	0.27	278.1	never smokers	1.16	1.03	1.31	Obesity
5	0.32	0.06	5.33	9.73E-08	0.20	0.44	278.1	never smokers	1.38	1.23	1.56	Obesity
2	0.06	0.08	0.72	4.69E-01	-0.10	0.22	317	all	1.06	0.90	1.25	Alcohol Related Disorders
3	0.11	0.08	1.39	1.63E-01	-0.05	0.27	317	all	1.12	0.95	1.32	Alcohol Related Disorders
4	0.15	0.08	1.84	6.60E-02	-0.009	0.31	317	all	1.16	0.99	1.36	Alcohol Related Disorders
5	0.33	0.08	4.22	2.44E-05	0.18	0.49	317	all	1.39	1.19	1.63	Alcohol Related Disorders
2	-0.05	0.12	-0.47	6.41E-01	-0.29	0.18	317	smokers	0.95	0.75	1.19	Alcohol Related Disorders
3	0.07	0.11	0.66	5.09E-01	-0.15	0.30	317	smokers	1.08	0.86	1.35	Alcohol Related Disorders
4	-0.04	0.11	-0.38	7.08E-01	-0.27	0.18	317	smokers	0.96	0.77	1.20	Alcohol Related Disorders
5	0.11	0.11	0.99	3.24E-01	-0.11	0.32	317	smokers	1.11	0.90	1.38	Alcohol Related Disorders
2	0.11	0.12	0.95	3.42E-01	-0.12	0.35	317	never smokers	1.12	0.88	1.41	Alcohol Related Disorders

3	0.03	0.12	0.27	7.85E-01	-0.21	0.27	317	never smokers	1.03	0.81	1.31	Alcohol Related Disorders
4	0.22	0.12	1.85	6.42E-02	-0.01	0.45	317	never smokers	1.24	0.99	1.56	Alcohol Related Disorders
5	0.39	0.11	3.37	7.64E-04	0.16	0.61	317	never smokers	1.47	1.17	1.84	Alcohol Related Disorders
2	0.08	0.11	0.73	4.65E-01	-0.14	0.31	165.1	all	1.09	0.87	1.36	Lung Cancer
3	0.13	0.11	1.18	2.39E-01	-0.09	0.35	165.1	all	1.14	0.91	1.42	Lung Cancer
4	0.01	0.11	0.11	9.08E-01	-0.21	0.24	165.1	all	1.01	0.81	1.27	Lung Cancer
5	0.07	0.11	0.60	5.50E-01	-0.16	0.30	165.1	all	1.07	0.85	1.34	Lung Cancer
2	0.05	0.16	0.28	7.82E-01	-0.27	0.36	165.1	smokers	1.05	0.76	1.44	Lung Cancer
3	0.24	0.15	1.58	1.15E-01	-0.06	0.55	165.1	smokers	1.28	0.94	1.73	Lung Cancer
4	0.04	0.16	0.24	8.13E-01	-0.28	0.35	165.1	smokers	1.04	0.76	1.42	Lung Cancer
5	0.11	0.16	0.75	4.52E-01	-0.19	0.42	165.1	smokers	1.12	0.83	1.53	Lung Cancer
2	0.08	0.16	0.50	6.18E-01	-0.24	0.40	165.1	never smokers	1.08	0.79	1.49	Lung Cancer
3	-0.08	0.17	-0.47	6.40E-01	-0.41	0.25	165.1	never smokers	0.92	0.66	1.29	Lung Cancer
4	-0.09	0.17	-0.54	5.88E-01	-0.43	0.24	165.1	never smokers	0.91	0.65	1.27	Lung Cancer
5	-0.16	0.18	-0.88	3.81E-01	-0.51	0.19	165.1	never smokers	0.86	0.60	1.21	Lung Cancer

Supplementary Table 7
Mendelian Randomization results between Cigarettes smoked per day (GSCAN) and Body Mass Index (GIANT Consortium)

outcome	exposure	method	nsnp	b	se	pval
Body mass index || id:ieu-a-835	Cigarettes smoked per day || id:ieu-b-142	MR Egger	16	-0.082	0.044	0.083
Body mass index || id:ieu-a-835	Cigarettes smoked per day || id:ieu-b-142	Weighted median	16	-0.046	0.021	0.032

Body mass index || id:ieu-a-835	Cigarettes smoked per day || id:ieu-b-142	Inverse variance weighted	16	-0.014	0.029	0.622
Body mass index || id:ieu-a-835	Cigarettes smoked per day || id:ieu-b-142	Simple mode	16	0.060	0.065	0.372
Body mass index || id:ieu-a-835	Cigarettes smoked per day || id:ieu-b-142	Weighted mode	16	-0.049	0.021	0.0360
Cigarettes smoked per day || id:ieu-b-142	Body mass index || id:ieu-a-835	MR Egger	65	0.23	0.123	0.070
Cigarettes smoked per day || id:ieu-b-142	Body mass index || id:ieu-a-835	Weighted median	65	0.33	0.047	1.29E-12
Cigarettes smoked per day || id:ieu-b-142	Body mass index || id:ieu-a-835	Inverse variance weighted	65	0.26	0.042	2.93E-10
Cigarettes smoked per day || id:ieu-b-142	Body mass index || id:ieu-a-835	Simple mode	65	0.47	0.127	4.44e-04
Cigarettes smoked per day || id:ieu-b-142	Body mass index || id:ieu-a-835	Weighted mode	65	0.45	0.086	1.87E-06
