# Supplementary material for: Polygenic scores for tobacco use provide insights into systemic health risks in a diverse EHR-linked biobank in Los Angeles
Source: Transl Psychiatry. 2024 Jan 18;14:38. doi: 10.1038/s41398-024-02743-z (PMC10796315; doi:10.1038/s41398-024-02743-z)

**Supplementary Figures**

**Supplementary Figures 1A and B**

1. **DAG showing the relationship evaluated in the PGS-PheWAS meta-analysis**
2. **DAG showing the relationship evaluated in the PGS-PheWAS never-smoker analysis**

Effects of germline variants that predispose to tobacco use are captured by the TUD-PGS. Systemic health effects are captured by 1847 phecodes. The star indicates the relationship evaluated in the PGS-PheWAS analysis. The red X in 1B denotes that the effect of tobacco use behavior on those systemic health effects is accounted for in the PGS-PheWAS analysis in never-smokers.


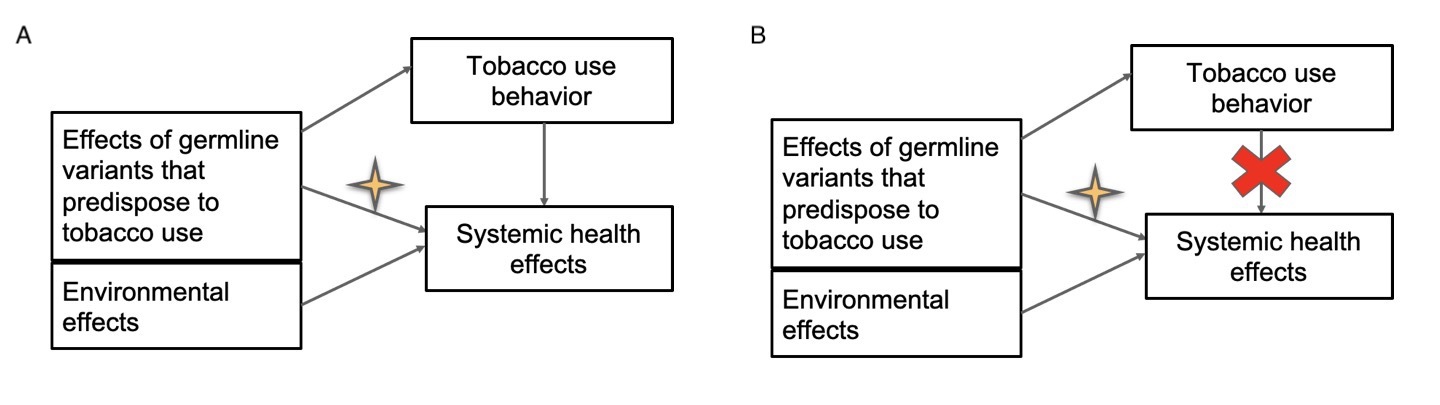


**Supplementary Figure 2**

**TUD-PGS association with Lung Cancer across PGS quintiles among ever-smokers and never smokers**

X-axis represents the top 4 quintiles grouped by TUD-PGS. Y axis represents effect sizes represented by odds ratios. Red line indicates OR =1.


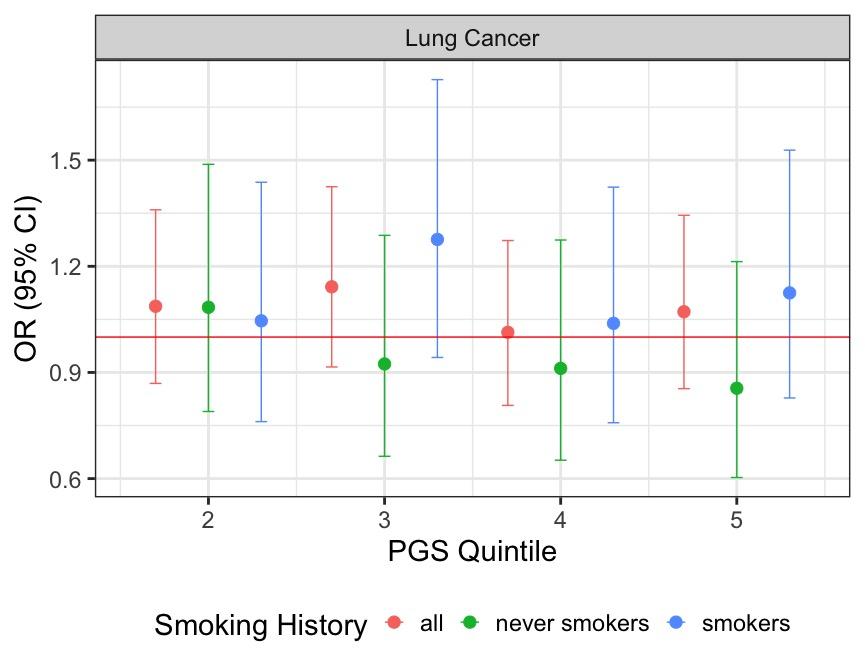


**Supplementary Figures 3A and 3B**

**Mendelian Randomization Between Waist Circumference, BMI, and Cigarettes Smoked Per Day**

1. **MR results between Outcome - cigarettes smoked per day and Exposure - waist circumference and body mass index**

Mendelian Randomization results across multiple MR methods using summary statistics for cigarettes smoked per day from the GSCAN Consortium and for waist circumference from MRC-UBristol and body mass index from UKBB. X axis represents the effect sizes and Y axis represents the MR method used.

1. **MR results between Exposure - cigarettes smoked per day and Outcome - waist circumference and body mass index**

Mendelian Randomization results across multiple MR methods using summary statistics for cigarettes smoked per day from the GSCAN Consortium and for waist circumference from MRC-UBristol and body mass index from UKBB. X axis represents the effect sizes and Y axis represents the MR method used.


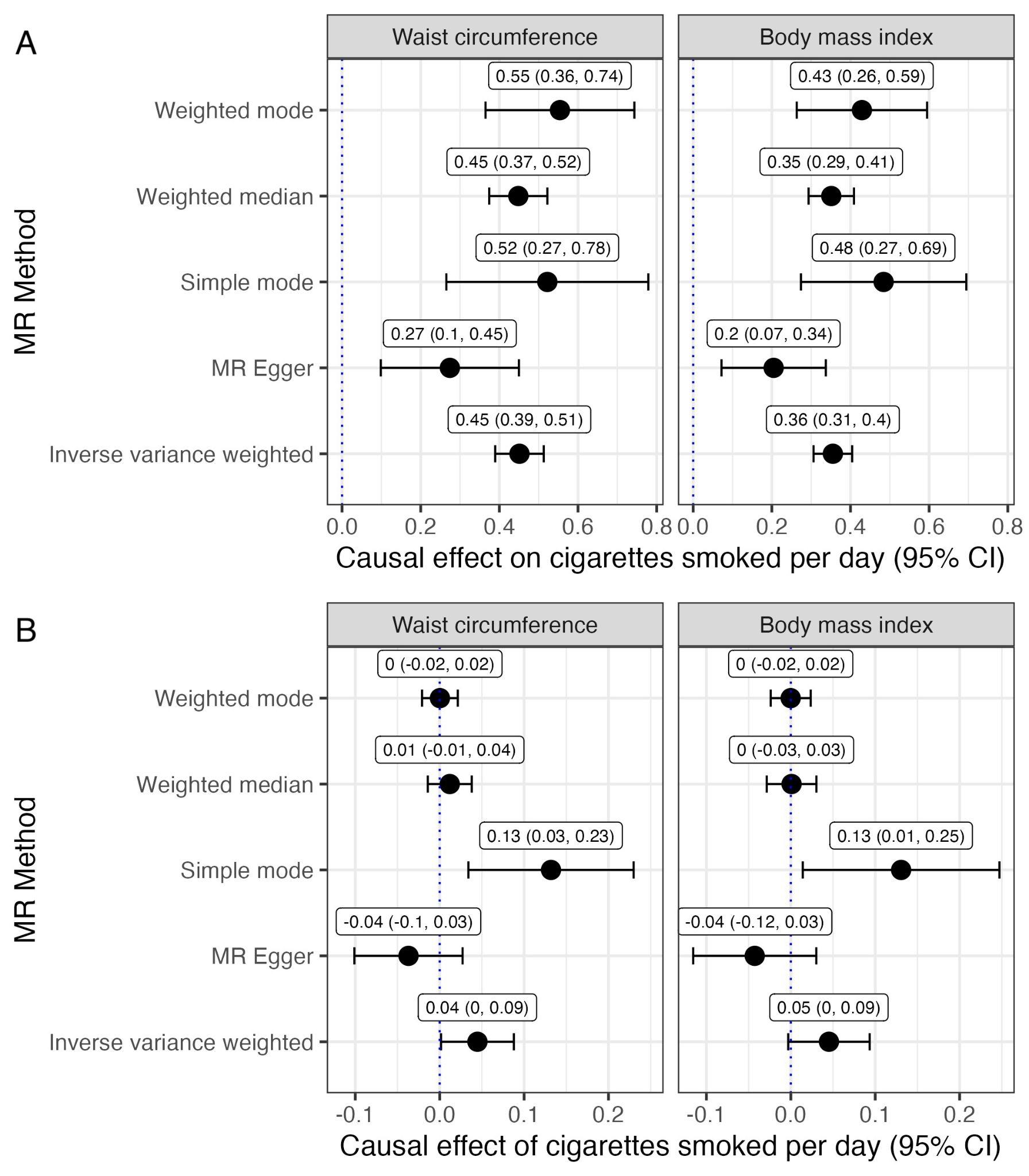

Supplement: Supplementary file 2 — Supplementary Figures [file 41398_2024_2743_MOESM2_ESM.docx]
