# Supplementary material for: Polygenic scores for tobacco use provide insights into systemic health risks in a diverse EHR-linked biobank in Los Angeles
Source: Transl Psychiatry. 2024 Jan 18;14:38. doi: 10.1038/s41398-024-02743-z (PMC10796315; doi:10.1038/s41398-024-02743-z)
Supplement: Supplementary file 3 — Supplementary material Part 1 [file 41398_2024_2743_MOESM3_ESM.pdf]

| | | Overall |
|---|---|---|
| **n** | | 24202 |
| **Age, median [Q1,Q3]** | | 61.0 [46.0,72.0] |
| **Sex, n (%)** | **Female** | 13277 (54.9) |
| | **Male** | 10914 (45.1) |
| **Insurance Class, n (%)** | **private** | 14996 (62.0) |
| | **public** | 8431 (34.8) |
| | **selfpay** | 775 (3.2) |
| **Tobacco Use Disorder, n (%)** | **0** | 16283 (67.3) |
| | **1** | 7902 (32.7) |
| **Genetically Inferred Ancestry, n (%)** | **AFR** | 1633 (6.7) |
| | **AMR** | 4412 (18.2) |
| | **EAS** | 2377 (9.8) |
| | **EUR** | 15780 (65.2) |

| GIA | coef | std err | z | P>\|z\| | [0.025 | 0.975] | or | or_lower_ci | or_upper_ci |
|---|---|---|---|---|---|---|---|---|---|
| European American | 0.183796 | 0.017609 | 10.437893 | 1.66E-25 | 0.149284 | 0.218308 | 1.201770637 | 1.161002667 | 1.243970151 |
| Hispanic/Latin American | 0.17314 | 0.036603 | 4.730157 | 2.24E-06 | 0.101399 | 0.244881 | 1.189032558 | 1.106718134 | 1.277469285 |
| East Asian American | 0.165009 | 0.053223 | 3.100306 | 1.93E-03 | 0.060693 | 0.269324 | 1.179403733 | 1.062572654 | 1.309079214 |
| African American | 0.039451 | 0.059467 | 0.663412 | 5.07E-01 | -0.077102 | 0.156005 | 1.040239526 | 0.9257954177 | 1.168832047 |

| pgs_quantile | coef | std err | z | P>\|z\| | [0.025 | 0.975] | or | or_lower_ci | or_upper_ci | GIA |
|---|---|---|---|---|---|---|---|---|---|---|
| 2 | 0.10407 | 0.055912 | 1.861316 | 6.27E-02 | -0.005516 | 0.213656 | 1.10967813 | 0.9944991852 | 1.238196642 | European American |
| 3 | 0.219436 | 0.055506 | 3.953396 | 7.70E-05 | 0.110647 | 0.328226 | 1.245374141 | 1.117000536 | 1.388502738 | European American |
| 4 | 0.309244 | 0.055464 | 5.57553 | 2.47E-08 | 0.200535 | 0.417952 | 1.362394754 | 1.222056383 | 1.518847768 | European American |
| 5 | 0.521974 | 0.05521 | 9.454349 | 3.25E-21 | 0.413765 | 0.630184 | 1.685351252 | 1.512501647 | 1.877956091 | European American |
| 2 | 0.154497 | 0.116707 | 1.3238 | 1.86E-01 | -0.074245 | 0.383239 | 1.167070777 | 0.9284441971 | 1.467028608 | Hispanic/Latin American |
| 3 | 0.16135 | 0.116472 | 1.385309 | 1.66E-01 | -0.066931 | 0.389631 | 1.175096181 | 0.935259732 | 1.476435889 | Hispanic/Latin American |
| 4 | 0.29403 | 0.116078 | 2.533044 | 1.13E-02 | 0.066522 | 0.521538 | 1.341824158 | 1.068784477 | 1.684616599 | Hispanic/Latin American |
| 5 | 0.535119 | 0.114996 | 4.65337 | 3.27E-06 | 0.309731 | 0.760506 | 1.707651441 | 1.363058402 | 2.139358462 | Hispanic/Latin American |
| 2 | 0.193084 | 0.172924 | 1.116584 | 2.64E-01 | -0.145841 | 0.532009 | 1.21298468 | 0.8642951152 | 1.702348894 | East Asian American |
| 3 | 0.52366 | 0.168958 | 3.099348 | 1.94E-03 | 0.192508 | 0.854811 | 1.688195151 | 1.212286202 | 2.350930013 | East Asian American |
| 4 | 0.312306 | 0.17157 | 1.820279 | 6.87E-02 | -0.023966 | 0.648577 | 1.3665728 | 0.976318904 | 1.912816953 | East Asian American |
| 5 | 0.470507 | 0.170442 | 2.760507 | 5.77E-03 | 0.136446 | 0.804568 | 1.600805596 | 1.146192982 | 2.235730455 | East Asian American |
| 2 | -0.103231 | 0.17638 | -0.585276 | 5.58E-01 | -0.44893 | 0.242468 | 0.9019186062 | 0.6383107789 | 1.274390468 | African American |
| 3 | 0.011374 | 0.178467 | 0.063729 | 9.49E-01 | -0.338415 | 0.361162 | 1.01143893 | 0.7128993733 | 1.434995911 | African American |
| 4 | 0.124504 | 0.181621 | 0.685514 | 4.93E-01 | -0.231467 | 0.480474 | 1.132586551 | 0.7933688762 | 1.616840603 | African American |
| 5 | 0.019169 | 0.18506 | 0.103583 | 9.18E-01 | -0.343542 | 0.38188 | 1.019353905 | 0.7092536919 | 1.46503627 | African American |

| phecode | Coef. | Std.Err. | z | p_value_z | [0.025 | 0.975] | GIA | phenotype | category |
|---|---|---|---|---|---|---|---|---|---|
| 318 | 0.187402 | 0.017607 | 10.64342 | 1.87E-26 | 0.152892 | 0.221911 | EUR | Tobacco use disc | mental disorders |
| 496.1 | 0.243532 | 0.035151 | 6.928172 | 4.26E-12 | 0.174638 | 0.312427 | EUR | Emphysema | respiratory |
| 250.2 | 0.144867 | 0.021819 | 6.639472 | 3.15E-11 | 0.102103 | 0.187632 | EUR | Type 2 diabetes | endocrine/metabolic |
| 317 | 0.204217 | 0.031105 | 6.565495 | 5.19E-11 | 0.143253 | 0.265181 | EUR | Alcohol-related d | mental disorders |
| 278.1 | 0.12472 | 0.02032 | 6.137866 | 8.36E-10 | 0.084894 | 0.164546 | EUR | Obesity | endocrine/metabolic |
| 317.1 | 0.211836 | 0.034615 | 6.119816 | 9.37E-10 | 0.143992 | 0.27968 | EUR | Alcoholism | mental disorders |
| 539 | 0.258798 | 0.045839 | 5.645832 | 1.64E-08 | 0.168956 | 0.348641 | EUR | Bariatric surgery | digestive |
| 496 | 0.135441 | 0.02418 | 5.601477 | 2.13E-08 | 0.08805 | 0.182833 | EUR | Chronic airway o | respiratory |
| 250 | 0.12127 | 0.021849 | 5.550469 | 2.85E-08 | 0.078447 | 0.164092 | EUR | Diabetes mellitus | endocrine/metabolic |
| 496.2 | 0.205749 | 0.037783 | 5.445553 | 5.16E-08 | 0.131696 | 0.279802 | EUR | Chronic bronchiti | respiratory |
| 278.11 | 0.144205 | 0.026573 | 5.426798 | 5.74E-08 | 0.092123 | 0.196287 | EUR | Morbid obesity | endocrine/metabolic |
| 496.21 | 0.243816 | 0.04766 | 5.115705 | 3.13E-07 | 0.150404 | 0.337229 | EUR | Obstructive chror | respiratory |
| 228.1 | -0.10888 | 0.021928 | -4.96559 | 6.85E-07 | -0.15186 | -0.06591 | EUR | Hemangioma of s | neoplasms |
| 296.22 | 0.08698 | 0.017881 | 4.864395 | 1.15E-06 | 0.051934 | 0.122026 | EUR | Major depressive | mental disorders |
| 290.13 | 0.754752 | 0.15706 | 4.805518 | 1.54E-06 | 0.446921 | 1.062583 | EUR | Senile dementia | mental disorders |
| 721.1 | 0.092809 | 0.019361 | 4.793587 | 1.64E-06 | 0.054862 | 0.130756 | EUR | Spondylosis with | musculoskeletal |
| 721 | 0.091465 | 0.019224 | 4.757857 | 1.96E-06 | 0.053787 | 0.129144 | EUR | Spondylosis and | musculoskeletal |
| 285.1 | 0.110043 | 0.023246 | 4.733832 | 2.20E-06 | 0.064482 | 0.155605 | EUR | Acute posthemor | hematopoietic |
| 278 | 0.082167 | 0.017796 | 4.617057 | 3.89E-06 | 0.047287 | 0.117048 | EUR | Overweight, obes | endocrine/metabolic |
| 428.1 | 0.134763 | 0.029323 | 4.595868 | 4.31E-06 | 0.077292 | 0.192234 | EUR | Congestive heart | circulatory system |
| 655 | -0.21619 | 0.047094 | -4.59066 | 4.42E-06 | -0.3085 | -0.12389 | EUR | Known or suspec | pregnancy complications |
| 411 | 0.091821 | 0.020449 | 4.490306 | 7.11E-06 | 0.051742 | 0.1319 | EUR | Ischemic Heart D | circulatory system |
| 440 | 0.116963 | 0.026247 | 4.456231 | 8.34E-06 | 0.06552 | 0.168406 | EUR | Atherosclerosis | circulatory system |
| 764 | 0.107252 | 0.02407 | 4.455822 | 8.36E-06 | 0.060075 | 0.154428 | EUR | Sciatica | symptoms |
| 411.4 | 0.093452 | 0.021176 | 4.413011 | 1.02E-05 | 0.051947 | 0.134957 | EUR | Coronary atheros | circulatory system |
| 331.9 | -0.32715 | 0.074237 | -4.40688 | 1.05E-05 | -0.47265 | -0.18165 | EUR | Cerebral degene | neurological |
| 228 | -0.0929 | 0.021092 | -4.4047 | 1.06E-05 | -0.13424 | -0.05156 | EUR | Hemangioma an | neoplasms |
| 316 | 0.114231 | 0.025956 | 4.400954 | 1.08E-05 | 0.063358 | 0.165103 | EUR | Substance addic | mental disorders |
| 440.21 | 0.557778 | 0.128652 | 4.33554 | 1.45E-05 | 0.305624 | 0.809932 | EUR | Atherosclerosis c | circulatory system |
| 250.22 | 0.158607 | 0.036718 | 4.319596 | 1.56E-05 | 0.086641 | 0.230573 | EUR | Type 2 diabetes v | endocrine/metabolic |
| 260.21 | 1.290743 | 0.301828 | 4.276423 | 1.90E-05 | 0.699172 | 1.882314 | EUR | Kwashiorkor | endocrine/metabolic |
| 150 | 0.39525 | 0.092443 | 4.275601 | 1.91E-05 | 0.214065 | 0.576435 | EUR | Cancer of esopha | neoplasms |
| 512.7 | 0.074879 | 0.017667 | 4.238325 | 2.25E-05 | 0.040252 | 0.109506 | EUR | Shortness of brea | respiratory |
| 338 | 0.069615 | 0.016518 | 4.214554 | 2.50E-05 | 0.037241 | 0.101989 | EUR | Pain | neurological |
| 571.5 | 0.091268 | 0.022131 | 4.124073 | 3.72E-05 | 0.047893 | 0.134643 | EUR | Other chronic no | digestive |
| 694 | -0.08048 | 0.019675 | -4.09027 | 4.31E-05 | -0.11904 | -0.04191 | EUR | Dyschromia and | dermatologic |
| 457.2 | 0.173422 | 0.042565 | 4.07424 | 4.62E-05 | 0.089995 | 0.256848 | EUR | Encounter for lon | circulatory system |

| phecode | Coef. | Std.Err. | z | p_value_z | [0.025 | 0.975] | GIA | phenotype | category |
|---|---|---|---|---|---|---|---|---|---|
| 694.2 | -0.08034 | 0.019819 | -4.05371 | 5.04E-05 | -0.11919 | -0.0415 | EUR | Other dyschromia | dermatologic |
| 401 | 0.075494 | 0.018644 | 4.049171 | 5.14E-05 | 0.038952 | 0.112036 | EUR | Hypertension | circulatory system |
| 599.5 | -0.08897 | 0.021981 | -4.04759 | 5.17E-05 | -0.13205 | -0.04589 | EUR | Frequency of urir | genitourinary |
| 571 | 0.087981 | 0.021857 | 4.025278 | 5.69E-05 | 0.045142 | 0.130821 | EUR | Chronic liver dise | digestive |
| 440.9 | 0.116753 | 0.029103 | 4.011742 | 6.03E-05 | 0.059712 | 0.173793 | EUR | Atherosclerosis c | circulatory system |
| 296.2 | 0.068593 | 0.017287 | 3.967947 | 7.25E-05 | 0.034712 | 0.102474 | EUR | Depression | mental disorders |
| 327.3 | 0.07942 | 0.020024 | 3.966196 | 7.30E-05 | 0.040173 | 0.118666 | EUR | Sleep apnea | neurological |
| 338.2 | 0.065422 | 0.016664 | 3.925985 | 8.64E-05 | 0.032761 | 0.098082 | EUR | Chronic pain | neurological |
| 702 | -0.07152 | 0.01826 | -3.91655 | 8.98E-05 | -0.10731 | -0.03573 | EUR | Degenerative ski | dermatologic |
| 676 | -0.39748 | 0.102524 | -3.87696 | 0.000106 | -0.59842 | -0.19654 | EUR | Other disorders c | pregnancy complications |
| 172.2 | -0.08118 | 0.021018 | -3.86235 | 0.000112 | -0.12237 | -0.03998 | EUR | Other non-epithe | neoplasms |
| 706 | -0.07625 | 0.019864 | -3.83859 | 0.000124 | -0.11518 | -0.03732 | EUR | Diseases of seba | dermatologic |
| 216 | -0.0665 | 0.017358 | -3.831 | 0.000128 | -0.10052 | -0.03248 | EUR | Benign neoplasm | neoplasms |
| 695.22 | 0.761533 | 0.202703 | 3.756887 | 0.000172 | 0.364242 | 1.158824 | EUR | Pemphigus and p | dermatologic |
| 296 | 0.064142 | 0.017122 | 3.746225 | 0.00018 | 0.030584 | 0.0977 | EUR | Mood disorders | mental disorders |
| 571.81 | 0.208666 | 0.055965 | 3.728503 | 0.000193 | 0.098977 | 0.318356 | EUR | Portal hypertensi | digestive |
| 411.3 | 0.120342 | 0.032405 | 3.713707 | 0.000204 | 0.05683 | 0.183854 | EUR | Angina pectoris | circulatory system |
| 250.25 | 0.165618 | 0.044644 | 3.709754 | 0.000207 | 0.078117 | 0.253118 | EUR | Diabetes type 2 v | endocrine/metabolic |
| 530 | 0.061028 | 0.016453 | 3.709307 | 0.000208 | 0.028781 | 0.093275 | EUR | Diseases of esop | digestive |
| 401.1 | 0.068713 | 0.018582 | 3.697783 | 0.000217 | 0.032293 | 0.105134 | EUR | Essential hyperte | circulatory system |
| 457 | 0.076207 | 0.020672 | 3.686497 | 0.000227 | 0.035691 | 0.116723 | EUR | Encounter for lon | circulatory system |
| 452 | 0.096442 | 0.026186 | 3.682961 | 0.000231 | 0.045118 | 0.147765 | EUR | Other venous em | circulatory system |
| 702.1 | -0.07883 | 0.021438 | -3.67719 | 0.000236 | -0.12085 | -0.03681 | EUR | Actinic keratosis | dermatologic |
| 250.3 | 0.126995 | 0.03458 | 3.672465 | 0.00024 | 0.059219 | 0.194771 | EUR | Insulin pump use | endocrine/metabolic |
| 702.2 | -0.06835 | 0.018739 | -3.64718 | 0.000265 | -0.10507 | -0.03162 | EUR | Seborrheic kerat | dermatologic |
| 514.2 | 0.086083 | 0.023627 | 3.643439 | 0.000269 | 0.039775 | 0.13239 | EUR | Solitary pulmona | respiratory |
| 655.1 | -0.22059 | 0.060669 | -3.63595 | 0.000277 | -0.3395 | -0.10168 | EUR | Abnormality in fe | pregnancy complications |
| 110 | -0.08338 | 0.022967 | -3.63059 | 0.000283 | -0.1284 | -0.03837 | EUR | Dermatophytosis | infectious diseases |
| 317.11 | 0.254089 | 0.070033 | 3.628129 | 0.000285 | 0.116827 | 0.391352 | EUR | Alcoholic liver da | mental disorders |
| 335 | 0.314795 | 0.086846 | 3.624727 | 2.89E-04 | 0.144579 | 0.485011 | EUR | Multiple sclerosis | neurological |
| 327.32 | 0.078641 | 0.021748 | 3.616065 | 0.000299 | 0.036016 | 0.121265 | EUR | Obstructive sleep | neurological |
| 411.2 | 0.114062 | 0.03166 | 3.602695 | 0.000315 | 0.052009 | 0.176114 | EUR | Myocardial infarc | circulatory system |
| 110.12 | -0.1515 | 0.042257 | -3.58522 | 0.000337 | -0.23433 | -0.06868 | EUR | Althete's foot | infectious diseases |
| 571.8 | 0.172503 | 0.048483 | 3.558029 | 0.000374 | 0.077478 | 0.267527 | EUR | Liver abscess an | digestive |
| 433.12 | 0.350226 | 0.098535 | 3.554317 | 0.000379 | 0.1571 | 0.543351 | EUR | Cerebral atheros | circulatory system |
| 500.2 | 0.817849 | 0.230956 | 3.541153 | 0.000398 | 0.365184 | 1.270513 | EUR | Pneumoconiosis | respiratory |
| 217.1 | -0.08783 | 0.024876 | -3.53055 | 4.15E-04 | -0.13658 | -0.03907 | EUR | Nevus, non-neop | neoplasms |

| phecode | Coef. | Std.Err. | z | p_value_z | [0.025 | 0.975] | GIA | phenotype | category |
|---|---|---|---|---|---|---|---|---|---|
| 440.22 | 0.26656 | 0.076015 | 3.506665 | 0.000454 | 0.117573 | 0.415547 | EUR | Atherosclerosis o | circulatory system |
| 604.2 | 1.131891 | 0.322909 | 3.505298 | 0.000456 | 0.499002 | 1.764781 | EUR | Vascular disorde | genitourinary |
| 514 | 0.061825 | 0.017686 | 3.495636 | 0.000473 | 0.027161 | 0.09649 | EUR | Abnormal finding | respiratory |
| 428 | 0.093014 | 0.02662 | 3.494163 | 0.000476 | 0.04084 | 0.145188 | EUR | Congestive heart | circulatory system |
| 145.1 | 0.810623 | 0.233249 | 3.475349 | 0.00051 | 0.353463 | 1.267784 | EUR | Cancer of lip | neoplasms |
| 250.24 | 0.128522 | 0.037345 | 3.44146 | 0.000579 | 0.055327 | 0.201718 | EUR | Type 2 diabetes | endocrine/metabolic |
| 530.1 | 0.056587 | 0.016471 | 3.435498 | 0.000591 | 0.024304 | 0.088871 | EUR | Esophagitis, GEF | digestive |
| 611 | -0.08314 | 0.024333 | -3.41675 | 0.000634 | -0.13083 | -0.03545 | EUR | Abnormal finding | genitourinary |
| 510.2 | 0.241905 | 0.070824 | 3.415593 | 0.000636 | 0.103093 | 0.380717 | EUR | Lung transplant | respiratory |
| 754.1 | 0.747279 | 0.22026 | 3.392707 | 0.000692 | 0.315576 | 1.178981 | EUR | Lumbosacral spo | congenital anomalies |
| 274.2 | 0.18949 | 0.0559 | 3.38978 | 0.000699 | 0.079927 | 0.299052 | EUR | Crystal arthropat | endocrine/metabolic |
| 509 | 0.087751 | 0.02591 | 3.386718 | 0.000707 | 0.036968 | 0.138534 | EUR | Respiratory failur | respiratory |
| 872 | 0.486175 | 0.143631 | 3.384882 | 7.12E-04 | 0.204663 | 0.767687 | EUR | Traumatic amput | injuries & poisonings |
| 355.1 | 0.158393 | 0.046823 | 3.382834 | 0.000717 | 0.066623 | 0.250164 | EUR | Chronic pain syn | neurological |
| 731.1 | 0.905732 | 0.269227 | 3.364195 | 0.000768 | 0.378057 | 1.433407 | EUR | Osteitis deformar | musculoskeletal |
| 571.51 | 0.158074 | 0.046997 | 3.363501 | 0.00077 | 0.065962 | 0.250186 | EUR | Cirrhosis of liver | digestive |
| 380.4 | -0.08156 | 0.02426 | -3.36193 | 0.000774 | -0.12911 | -0.03401 | EUR | Impacted cerume | sense organs |
| 364.2 | 0.415667 | 0.1237 | 3.360268 | 0.000779 | 0.173218 | 0.658115 | EUR | Corneal edema | sense organs |
| 217 | -0.08216 | 0.024595 | -3.34058 | 0.000836 | -0.13037 | -0.03396 | EUR | Vascular hamarto | neoplasms |
| 389.1 | -0.08838 | 0.026549 | -3.32905 | 0.000871 | -0.14042 | -0.03635 | EUR | Sensorineural he | sense organs |
| 300 | 0.055253 | 0.016678 | 3.312928 | 0.000923 | 0.022565 | 0.087941 | EUR | Anxiety disorders | mental disorders |
| 394.4 | -1.55734 | 0.472816 | -3.29376 | 0.000989 | -2.48404 | -0.63064 | EUR | Acute rheumatic | circulatory system |
| 401.2 | 0.077276 | 0.023561 | 3.279884 | 0.001038 | 0.031098 | 0.123453 | EUR | Hypertensive hea | circulatory system |
| 977 | 0.068947 | 0.02112 | 3.264489 | 0.001097 | 0.027552 | 0.110342 | EUR | Personal history | injuries & poisonings |
| 78 | -0.08463 | 0.025985 | -3.25692 | 0.001126 | -0.13556 | -0.0337 | EUR | Viral warts & HP\ | infectious diseases |
| 367 | -0.07484 | 0.022998 | -3.25424 | 0.001137 | -0.11992 | -0.02977 | EUR | Disorders of refra | sense organs |
| 274.21 | 0.183765 | 0.05673 | 3.23932 | 0.001198 | 0.072577 | 0.294953 | EUR | Chondrocalcinos | endocrine/metabolic |
| 415.2 | 0.115589 | 0.035829 | 3.226117 | 0.001255 | 0.045365 | 0.185813 | EUR | Chronic pulmona | circulatory system |
| 716 | 0.062491 | 0.019383 | 3.223938 | 0.001264 | 0.0245 | 0.100482 | EUR | Other arthropathi | musculoskeletal |
| 246.7 | -0.12835 | 0.039821 | -3.22305 | 0.001268 | -0.20639 | -0.0503 | EUR | Abnormal results | endocrine/metabolic |
| 521.1 | 0.304934 | 0.094985 | 3.210345 | 0.001326 | 0.118767 | 0.491101 | EUR | Dental caries | digestive |
| 972.6 | 0.529375 | 0.165029 | 3.207766 | 0.001338 | 0.205924 | 0.852827 | EUR | Antihypertensive | injuries & poisonings |
| 379.2 | -0.08861 | 0.027752 | -3.19281 | 0.001409 | -0.143 | -0.03421 | EUR | Disorders of vitre | sense organs |
| 510 | 0.075759 | 0.02382 | 3.180451 | 0.00147 | 0.029072 | 0.122446 | EUR | Other diseases o | respiratory |
| 735 | -0.08003 | 0.025192 | -3.17692 | 0.001489 | -0.12941 | -0.03066 | EUR | Acquired foot def | musculoskeletal |
| 244.5 | -0.44686 | 0.14084 | -3.17282 | 0.00151 | -0.7229 | -0.17082 | EUR | Congenital hypot | endocrine/metabolic |
| 416 | 0.078619 | 0.024801 | 3.170003 | 0.001524 | 0.03001 | 0.127228 | EUR | Cardiomegaly | circulatory system |

| phecode | Coef. | Std.Err. | z | p_value_z | [0.025 | 0.975] | GIA | phenotype | category |
|---|---|---|---|---|---|---|---|---|---|
| 716.9 | 0.061866 | 0.019552 | 3.164119 | 0.001556 | 0.023544 | 0.100188 | EUR | Arthropathy NOS | musculoskeletal |
| 627 | -0.06984 | 0.022125 | -3.1564 | 0.001597 | -0.1132 | -0.02647 | EUR | Menopausal and | genitourinary |
| 285 | 0.053823 | 0.017057 | 3.155449 | 0.001603 | 0.020392 | 0.087255 | EUR | Other anemias | hematopoietic |
| 110.1 | -0.07517 | 0.02398 | -3.13475 | 0.00172 | -0.12217 | -0.02817 | EUR | Dermatophytosis | infectious diseases |
| 503 | 0.097284 | 0.031169 | 3.121145 | 0.001801 | 0.036193 | 0.158374 | EUR | Pulmonary conge | respiratory |
| 965.1 | 0.082282 | 0.026425 | 3.113847 | 0.001847 | 0.030491 | 0.134073 | EUR | Opiates and relat | injuries & poisonings |
| 530.11 | 0.051273 | 0.016508 | 3.106046 | 0.001896 | 0.018919 | 0.083628 | EUR | GERD | digestive |
| 513 | 0.094188 | 0.030349 | 3.103487 | 0.001913 | 0.034705 | 0.153672 | EUR | Respiratory abno | respiratory |
| 300.1 | 0.05225 | 0.016841 | 3.102439 | 0.001919 | 0.019241 | 0.085258 | EUR | Anxiety disorder | mental disorders |
| 1010 | -0.07853 | 0.02532 | -3.10135 | 0.001926 | -0.12815 | -0.0289 | EUR | Other tests | |
| 388 | -0.10068 | 0.032501 | -3.09761 | 0.001951 | -0.16438 | -0.03697 | EUR | Other disorders c | sense organs |
| 361.1 | -0.22622 | 0.073112 | -3.09412 | 0.001974 | -0.36952 | -0.08292 | EUR | Retinal detachme | sense organs |
| 508 | 0.060541 | 0.019595 | 3.089623 | 0.002004 | 0.022136 | 0.098946 | EUR | Pulmonary collap | respiratory |
| 962.2 | 0.642993 | 0.209619 | 3.067433 | 0.002159 | 0.232147 | 1.053839 | EUR | Insulins and antic | injuries & poisonings |
| 457.3 | 0.063663 | 0.020854 | 3.052857 | 0.002267 | 0.022791 | 0.104535 | EUR | Encounter for lon | circulatory system |
| 279.1 | 0.083973 | 0.02751 | 3.052424 | 0.00227 | 0.030054 | 0.137893 | EUR | Immunity deficier | endocrine/metabolic |
| 715.2 | -0.26533 | 0.087131 | -3.04524 | 0.002325 | -0.43611 | -0.09456 | EUR | Ankylosing spond | musculoskeletal |
| 509.1 | 0.082574 | 0.02716 | 3.040212 | 0.002364 | 0.02934 | 0.135807 | EUR | Respiratory failur | respiratory |
| 741.5 | 0.587176 | 0.193335 | 3.037091 | 0.002389 | 0.208246 | 0.966106 | EUR | Hemarthrosis | musculoskeletal |
| 765 | 0.090344 | 0.029832 | 3.028406 | 0.002458 | 0.031874 | 0.148814 | EUR | Cervical radiculiti | symptoms |
| 290.11 | 0.207424 | 0.068501 | 3.028019 | 2.46E-03 | 0.073163 | 0.341684 | EUR | Alzheimer's disea | mental disorders |
| 327.1 | 0.098597 | 0.032568 | 3.027441 | 0.002466 | 0.034766 | 0.162429 | EUR | Hypersomnia | neurological |
| 653 | -0.18663 | 0.061707 | -3.02436 | 0.002492 | -0.30757 | -0.06568 | EUR | Problems associa | pregnancy complications |
| 81.11 | 0.68967 | 0.228611 | 3.016785 | 0.002555 | 0.241601 | 1.13774 | EUR | Acute graft-versu | infectious diseases |
| 381.3 | -0.31433 | 0.104524 | -3.00729 | 2.64E-03 | -0.5192 | -0.10947 | EUR | Mastoiditis & rela | sense organs |
| 286.5 | 0.582473 | 0.194261 | 2.998397 | 0.002714 | 0.201727 | 0.963218 | EUR | Hemorrhagic diso | hematopoietic |
| 604.3 | 0.317803 | 0.106022 | 2.997509 | 0.002722 | 0.110003 | 0.525602 | EUR | Peyronie's diseas | genitourinary |
| 972.2 | 0.383705 | 0.128324 | 2.99012 | 0.002789 | 0.132194 | 0.635216 | EUR | Antilipemic and a | injuries & poisonings |
| 411.1 | 0.161626 | 0.054072 | 2.989086 | 0.002798 | 0.055647 | 0.267605 | EUR | Unstable angina | circulatory system |
| 962.1 | 0.151951 | 0.051034 | 2.977447 | 0.002907 | 0.051926 | 0.251976 | EUR | Adrenal cortical s | injuries & poisonings |
| 743.11 | -0.07759 | 0.026102 | -2.97246 | 0.002954 | -0.12875 | -0.02643 | EUR | Osteoporosis NO | musculoskeletal |
| 351 | 0.058131 | 0.01961 | 2.964284 | 0.003034 | 0.019695 | 0.096566 | EUR | Other peripheral | neurological |
| 415 | 0.085694 | 0.029122 | 2.942636 | 0.003254 | 0.028617 | 0.142771 | EUR | Pulmonary heart | circulatory system |
| 695.41 | 0.367516 | 0.125692 | 2.923933 | 0.003456 | 0.121164 | 0.613869 | EUR | Cutaneous lupus | dermatologic |
| 222 | 0.55323 | 0.189488 | 2.919609 | 0.003505 | 0.181841 | 0.924619 | EUR | Benign neoplasm | neoplasms |
| 573.3 | 0.099332 | 0.034055 | 2.916831 | 0.003536 | 0.032586 | 0.166078 | EUR | Hepatomegaly | digestive |
| 70.3 | 0.164413 | 0.05685 | 2.89206 | 0.003827 | 0.05299 | 0.275837 | EUR | Viral hepatitis C | infectious diseases |

| phecode | Coef. | Std.Err. | z | p_value_z | [0.025 | 0.975] | GIA | phenotype | category |
|---|---|---|---|---|---|---|---|---|---|
| 650 | -0.15989 | 0.05529 | -2.89176 | 0.003831 | -0.26825 | -0.05152 | EUR | Normal delivery | pregnancy complications |
| 279 | 0.077053 | 0.026691 | 2.886816 | 3.89E-03 | 0.024739 | 0.129366 | EUR | Disorders involvi | endocrine/metabolic |
| 706.1 | -0.09216 | 0.032009 | -2.8792 | 0.003987 | -0.1549 | -0.02942 | EUR | Acne | dermatologic |
| 379 | -0.0695 | 0.024207 | -2.87132 | 0.004088 | -0.11695 | -0.02206 | EUR | Other disorders c | sense organs |
| 189.12 | 0.47851 | 0.167453 | 2.857589 | 0.004269 | 0.15031 | 0.806711 | EUR | Malignant neopla | neoplasms |
| 816 | 0.631304 | 0.221069 | 2.855684 | 0.004294 | 0.198016 | 1.064592 | EUR | Cerebral lacerati | injuries & poisonings |
| 738 | 0.086811 | 0.030452 | 2.850738 | 0.004362 | 0.027126 | 0.146497 | EUR | Other acquired m | musculoskeletal |
| 338.1 | 0.04971 | 0.017468 | 2.845765 | 0.00443 | 0.015473 | 0.083947 | EUR | Acute pain | neurological |
| 722.1 | 0.070953 | 0.024943 | 2.844548 | 0.004447 | 0.022065 | 0.119841 | EUR | Displacement of | musculoskeletal |
| 70 | 0.129849 | 0.045654 | 2.844186 | 0.004453 | 0.040368 | 0.21933 | EUR | Viral hepatitis | infectious diseases |
| 601.11 | -0.20806 | 0.073188 | -2.84286 | 0.004471 | -0.35151 | -0.06462 | EUR | Acute prostatitis | genitourinary |
| 401.21 | 0.092986 | 0.032821 | 2.833159 | 0.004609 | 0.028659 | 0.157314 | EUR | Hypertensive hea | circulatory system |
| 360.3 | 0.630305 | 0.222779 | 2.82928 | 0.004665 | 0.193666 | 1.066945 | EUR | Hypotony of eye | sense organs |
| 380 | -0.17787 | 0.063402 | -2.80546 | 5.03E-03 | -0.30214 | -0.05361 | EUR | Disorders of exte | sense organs |
| 253.1 | -0.23407 | 0.083728 | -2.79566 | 0.005179 | -0.39818 | -0.06997 | EUR | Pituitary hyperfur | endocrine/metabolic |
| 187 | 0.285292 | 0.102063 | 2.795246 | 0.005186 | 0.085251 | 0.485332 | EUR | Cancer of other r | neoplasms |
| 428.4 | 0.099447 | 0.035604 | 2.793135 | 0.00522 | 0.029664 | 0.16923 | EUR | Heart failure with | circulatory system |
| 270.21 | 0.60806 | 0.217847 | 2.791219 | 0.005251 | 0.181087 | 1.035033 | EUR | Disorders of urea | endocrine/metabolic |
| 585.3 | 0.064194 | 0.023096 | 2.77948 | 0.005445 | 0.018927 | 0.109461 | EUR | Chronic renal fail | genitourinary |
| 259.8 | 0.650309 | 0.235187 | 2.765072 | 0.005691 | 0.189351 | 1.111267 | EUR | Polyglandular ac | endocrine/metabolic |
| 509.5 | 0.610444 | 0.222155 | 2.747829 | 0.005999 | 0.175028 | 1.045861 | EUR | Respiratory arres | respiratory |
| 286.2 | 0.067351 | 0.024558 | 2.742551 | 0.006096 | 0.019219 | 0.115483 | EUR | Encounter for lon | hematopoietic |
| 740.9 | 0.051215 | 0.018713 | 2.736895 | 0.006202 | 0.014538 | 0.087891 | EUR | Osteoarthrosis N | musculoskeletal |
| 189.4 | 0.269012 | 0.098347 | 2.735349 | 0.006231 | 0.076256 | 0.461768 | EUR | Malignant neopla | neoplasms |
| 585.1 | 0.063944 | 0.02343 | 2.729197 | 0.006349 | 0.018023 | 0.109865 | EUR | Acute renal failur | genitourinary |
| 754 | 0.156269 | 0.057517 | 2.716917 | 0.006589 | 0.043538 | 0.269001 | EUR | Congenital musc | congenital anomalies |
| 362.22 | 0.19682 | 0.07252 | 2.713993 | 0.006648 | 0.054682 | 0.338957 | EUR | Macular degener | sense organs |
| 242.1 | -0.20224 | 0.074556 | -2.71265 | 0.006675 | -0.34837 | -0.05612 | EUR | Graves' disease | endocrine/metabolic |
| 502 | 0.097798 | 0.036268 | 2.696489 | 0.007007 | 0.026713 | 0.168883 | EUR | Postinflammatory | respiratory |
| 256.1 | 0.796416 | 0.295527 | 2.694901 | 0.007041 | 0.217194 | 1.375638 | EUR | Hyperestrogenisi | endocrine/metabolic |
| 874 | 0.529634 | 0.196876 | 2.690183 | 0.007141 | 0.143763 | 0.915505 | EUR | Complication of a | injuries & poisonings |
| 525.2 | 0.929848 | 0.347178 | 2.678302 | 0.0074 | 0.249391 | 1.610304 | EUR | Atrophy of edent | digestive |
| 1012 | 0.240563 | 0.089928 | 2.675058 | 0.007472 | 0.064307 | 0.416819 | EUR | Late effect | |
| 443.9 | 0.094066 | 0.035207 | 2.671783 | 0.007545 | 0.025061 | 0.16307 | EUR | Peripheral vascu | circulatory system |
| 736.3 | 0.581981 | 0.218292 | 2.666063 | 0.007675 | 0.154136 | 1.009826 | EUR | Acquired deformi | musculoskeletal |
| 230 | 0.678293 | 0.255591 | 2.653821 | 0.007959 | 0.177344 | 1.179242 | EUR | Kaposi's sarcoma | neoplasms |
| 303.4 | 0.244677 | 0.092247 | 2.652409 | 0.007992 | 0.063876 | 0.425477 | EUR | Somatoform diso | mental disorders |

| phecode | Coef. | Std.Err. | z | p_value_z | [0.025 | 0.975] | GIA | phenotype | category |
|---|---|---|---|---|---|---|---|---|---|
| 691.1 | 0.624523 | 0.23562 | 2.650553 | 0.008036 | 0.162717 | 1.086329 | EUR | Ichthyosis conge | dermatologic |
| 250.6 | 0.108459 | 0.040945 | 2.648887 | 0.008076 | 0.028208 | 0.188711 | EUR | Polyneuropathy i | endocrine/metabolic |
| 525 | 0.169241 | 0.063891 | 2.648884 | 0.008076 | 0.044016 | 0.294466 | EUR | Other diseases o | digestive |
| 698 | -0.06958 | 0.026325 | -2.64314 | 0.008214 | -0.12118 | -0.01798 | EUR | Pruritus and relat | dermatologic |
| 743.1 | -0.06781 | 0.025663 | -2.64232 | 0.008234 | -0.11811 | -0.01751 | EUR | Osteoporosis | musculoskeletal |
| 717 | -0.24316 | 0.092232 | -2.6364 | 0.008379 | -0.42393 | -0.06239 | EUR | Polymyalgia Rhe | musculoskeletal |
| 512.2 | 0.073652 | 0.027944 | 2.635701 | 0.008396 | 0.018883 | 0.128422 | EUR | Painful respiratio | respiratory |
| 244.2 | -0.07258 | 0.027558 | -2.63363 | 0.008448 | -0.12659 | -0.01857 | EUR | Acquired hypothy | endocrine/metabolic |
| 284.2 | 0.852454 | 0.324062 | 2.63053 | 0.008525 | 0.217305 | 1.487604 | EUR | Constitutional ap | hematopoietic |
| 250.21 | 0.320717 | 0.122062 | 2.627494 | 0.008602 | 0.08148 | 0.559954 | EUR | Type 2 diabetes v | endocrine/metabolic |
| 1015 | 0.068956 | 0.026253 | 2.626638 | 0.008623 | 0.017502 | 0.12041 | EUR | Effects of other e | xternal causes |
| 562 | 0.054078 | 0.020592 | 2.626183 | 8.64E-03 | 0.013719 | 0.094438 | EUR | Diverticulosis and | digestive |
| 149.4 | 0.298206 | 0.113559 | 2.625989 | 0.00864 | 0.075633 | 0.520778 | EUR | Cancer of larynx | neoplasms |
| 440.2 | 0.136498 | 0.052233 | 2.61328 | 0.008968 | 0.034124 | 0.238873 | EUR | Atherosclerosis o | circulatory system |
| 681.5 | 0.094039 | 0.035994 | 2.612607 | 0.008985 | 0.023491 | 0.164586 | EUR | Cellulitis and abs | dermatologic |
| 626.15 | -0.32316 | 0.123923 | -2.60775 | 0.009114 | -0.56605 | -0.08028 | EUR | Infertility, female, | genitourinary |
| 769 | 0.472001 | 0.181084 | 2.606526 | 0.009147 | 0.117082 | 0.826919 | EUR | Nonallopathic les | symptoms |
| 418 | 0.045338 | 0.017416 | 2.603233 | 0.009235 | 0.011203 | 0.079472 | EUR | Nonspecific ches | circulatory system |
| 706.8 | -0.07956 | 0.030668 | -2.59416 | 0.009482 | -0.13967 | -0.01945 | EUR | Other specified d | dermatologic |
| 626.8 | -0.17257 | 0.066567 | -2.59246 | 0.009529 | -0.30304 | -0.0421 | EUR | Infertility, female | genitourinary |
| 276.41 | 0.086027 | 0.033252 | 2.587152 | 0.009677 | 0.020855 | 0.151199 | EUR | Acidosis | endocrine/metabolic |
| 149.3 | 0.502743 | 0.194617 | 2.583237 | 0.009788 | 0.1213 | 0.884186 | EUR | Cancer of hypopl | neoplasms |
| 442.4 | -0.26138 | 0.101718 | -2.5697 | 0.010179 | -0.46075 | -0.06202 | EUR | Arterial dissectio | circulatory system |
| 427.21 | 0.062589 | 0.024445 | 2.560346 | 0.010457 | 0.014677 | 0.110501 | EUR | Atrial fibrillation | circulatory system |
| 362.9 | -0.17797 | 0.069565 | -2.55835 | 0.010517 | -0.31432 | -0.04163 | EUR | Retinal edema | sense organs |
| 385.5 | 0.422335 | 0.165098 | 2.558079 | 0.010525 | 0.098748 | 0.745922 | EUR | Tympanosclerosi | sense organs |
| 643.1 | -0.35297 | 0.138012 | -2.5575 | 0.010543 | -0.62346 | -0.08247 | EUR | Hyperemesis gra | pregnancy complications |
| 611.1 | -0.06874 | 0.026885 | -2.55664 | 0.010569 | -0.12143 | -0.01604 | EUR | Abnormal mamm | genitourinary |
| 724 | 0.068939 | 0.026986 | 2.554577 | 0.010632 | 0.016047 | 0.121832 | EUR | Other and unspe | musculoskeletal |
| 71 | 0.214516 | 0.084067 | 2.551712 | 0.01072 | 0.049747 | 0.379284 | EUR | Human immunod | infectious diseases |
| 695.9 | -0.17863 | 0.070059 | -2.54965 | 0.010783 | -0.31594 | -0.04131 | EUR | Unspecified eryth | dermatologic |
| 277.4 | -0.12223 | 0.047942 | -2.54942 | 0.01079 | -0.21619 | -0.02826 | EUR | Disorders of biliru | endocrine/metabolic |
| 362 | -0.07011 | 0.027534 | -2.5464 | 0.010884 | -0.12408 | -0.01615 | EUR | Other retinal disc | sense organs |
| 561.2 | -0.05802 | 0.022796 | -2.54499 | 0.010928 | -0.1027 | -0.01334 | EUR | Flatulence | digestive |
| 428.3 | 0.092391 | 0.03631 | 2.544504 | 0.010943 | 0.021225 | 0.163557 | EUR | Heart failure with | circulatory system |
| 580.31 | 0.132396 | 0.052117 | 2.540335 | 0.011075 | 0.030247 | 0.234544 | EUR | Nephritis and nep | genitourinary |
| 513.8 | 0.094943 | 0.037398 | 2.538697 | 0.011127 | 0.021644 | 0.168242 | EUR | Disorders of diap | respiratory |

| phecode | Coef. | Std.Err. | z | p_value_z | [0.025 | 0.975] | GIA | phenotype | category |
|---|---|---|---|---|---|---|---|---|---|
| 370.2 | -0.22537 | 0.088799 | -2.538 | 0.011149 | -0.39941 | -0.05133 | EUR | Superficial kerati | sense organs |
| 433.32 | 1.351438 | 0.533265 | 2.534272 | 0.011268 | 0.306258 | 2.396618 | EUR | Moyamoya disea | circulatory system |
| 130 | -0.2703 | 0.106815 | -2.53056 | 0.011388 | -0.47965 | -0.06095 | EUR | Spirochetal infec | infectious diseases |
| 506 | 0.081274 | 0.032122 | 2.530178 | 0.0114 | 0.018316 | 0.144232 | EUR | Empyema and pı | respiratory |
| 366.2 | -0.05854 | 0.023209 | -2.5222 | 0.011662 | -0.10403 | -0.01305 | EUR | Senile cataract | sense organs |
| 974 | 0.500047 | 0.198376 | 2.520696 | 0.011712 | 0.111236 | 0.888857 | EUR | Poisoning by wat | injuries & poisonings |
| 627.4 | -0.25925 | 0.103185 | -2.5125 | 0.011988 | -0.46149 | -0.05701 | EUR | Premenopausal ı | genitourinary |
| 130.1 | -0.34373 | 0.137146 | -2.50631 | 0.0122 | -0.61253 | -0.07493 | EUR | Lyme disease | infectious diseases |
| 789.1 | 0.150861 | 0.06026 | 2.503483 | 0.012298 | 0.032753 | 0.268969 | EUR | Persistent vomitiı | symptoms |
| 567 | 0.116808 | 0.046667 | 2.502994 | 0.012315 | 0.025342 | 0.208275 | EUR | Peritonitis and re | digestive |
| 1013 | 0.070742 | 0.028269 | 2.502402 | 0.012335 | 0.015334 | 0.126149 | EUR | Asphyxia and hypoxemia | |
| 368.3 | -0.29333 | 0.117593 | -2.49445 | 0.012615 | -0.52381 | -0.06285 | EUR | Anisometropia | sense organs |
| 704.12 | -0.21335 | 0.085859 | -2.48494 | 0.012957 | -0.38164 | -0.04507 | EUR | Telogen effluvium | dermatologic |
| 797.1 | 0.145552 | 0.058602 | 2.483747 | 0.013001 | 0.030695 | 0.260409 | EUR | Cardiogenic shoc | symptoms |
| 375.1 | -0.0646 | 0.026023 | -2.4826 | 0.013043 | -0.11561 | -0.0136 | EUR | Dry eyes | sense organs |
| 526.8 | 0.6777 | 0.27325 | 2.480148 | 0.013133 | 0.14214 | 1.213259 | EUR | Exostosis of jaw | digestive |
| 507 | 0.053551 | 0.021612 | 2.477911 | 0.013215 | 0.011194 | 0.095909 | EUR | Pleurisy; pleural | respiratory |
| 521 | 0.234142 | 0.094758 | 2.470942 | 0.013476 | 0.048419 | 0.419864 | EUR | Diseases of hard | digestive |
| 286.1 | -0.26543 | 0.108173 | -2.45372 | 0.014139 | -0.47744 | -0.05341 | EUR | Congenital coagı | hematopoietic |
| 145.4 | 0.557686 | 0.227392 | 2.452535 | 0.014185 | 0.112006 | 1.003365 | EUR | Cancer of the gu | neoplasms |
| 710 | 0.145259 | 0.059367 | 2.44678 | 0.014414 | 0.028901 | 0.261617 | EUR | Osteomyelitis, pe | musculoskeletal |
| 249 | 0.1032 | 0.042265 | 2.441719 | 0.014618 | 0.020362 | 0.186039 | EUR | Secondary diabe | endocrine/metabolic |
| 994 | 0.064847 | 0.02656 | 2.441515 | 0.014626 | 0.01279 | 0.116904 | EUR | Sepsis and SIRS | injuries & poisonings |
| 733.8 | 0.169015 | 0.069348 | 2.437213 | 0.014801 | 0.033096 | 0.304934 | EUR | Malunion and no | musculoskeletal |
| 145.3 | 0.316725 | 0.130137 | 2.433779 | 0.014942 | 0.061661 | 0.571789 | EUR | Cancer of major | neoplasms |
| 327.72 | -0.2515 | 0.103632 | -2.42684 | 0.015231 | -0.45461 | -0.04838 | EUR | Sleep related leg | neurological |
| 426 | 0.047039 | 0.019406 | 2.42391 | 0.015354 | 0.009003 | 0.085075 | EUR | Cardiac conducti | circulatory system |
| 286.7 | 0.097464 | 0.040295 | 2.418765 | 0.015573 | 0.018487 | 0.176441 | EUR | Other and unspe | hematopoietic |
| 763 | 0.055604 | 0.023024 | 2.415077 | 0.015732 | 0.010478 | 0.10073 | EUR | Thoracic or lumb | symptoms |
| 870.8 | 0.495748 | 0.205454 | 2.412939 | 0.015824 | 0.093065 | 0.89843 | EUR | Open wound of g | injuries & poisonings |
| 557.1 | -0.17165 | 0.071149 | -2.41257 | 0.015841 | -0.3111 | -0.0322 | EUR | Celiac disease | digestive |
| 614 | -0.07288 | 0.030257 | -2.40872 | 0.016009 | -0.13218 | -0.01358 | EUR | Inflammatory dis | genitourinary |
| 427.42 | 0.176696 | 0.073421 | 2.406616 | 0.016101 | 0.032794 | 0.320598 | EUR | Cardiac arrest | circulatory system |
| 726.4 | 0.105638 | 0.043919 | 2.405294 | 0.016159 | 0.019558 | 0.191717 | EUR | Calcaneal spur; E | musculoskeletal |
| 740 | 0.043897 | 0.018273 | 2.402296 | 0.016293 | 0.008083 | 0.079712 | EUR | Osteoarthrosis | musculoskeletal |
| 530.2 | 0.084964 | 0.035413 | 2.39927 | 0.016428 | 0.015557 | 0.154372 | EUR | Esophageal blee | digestive |
| 389.4 | -0.07029 | 0.029398 | -2.39094 | 0.016805 | -0.12791 | -0.01267 | EUR | Tinnitus | sense organs |

| phecode | Coef. | Std.Err. | z | p_value_z | [0.025 | 0.975] | GIA | phenotype | category |
|---|---|---|---|---|---|---|---|---|---|
| 368.5 | 0.566866 | 0.237249 | 2.389332 | 0.016879 | 0.101867 | 1.031865 | EUR | Color vision defic | sense organs |
| 912 | -0.10592 | 0.044333 | -2.38915 | 0.016887 | -0.19281 | -0.01903 | EUR | Insect bite | injuries & poisonings |
| 729.7 | 0.495777 | 0.207606 | 2.388071 | 0.016937 | 0.088877 | 0.902677 | EUR | Nontraumatic cor | musculoskeletal |
| 604.1 | 0.475542 | 0.199218 | 2.387049 | 0.016984 | 0.085083 | 0.866002 | EUR | Redundant prepu | genitourinary |
| 624 | -0.10513 | 0.044112 | -2.38333 | 0.017157 | -0.19159 | -0.01868 | EUR | Symptoms involv | genitourinary |
| 370 | -0.13957 | 0.0587 | -2.37764 | 1.74E-02 | -0.25462 | -0.02452 | EUR | Keratitis | sense organs |
| 1011 | 0.07605 | 0.032071 | 2.371309 | 0.017725 | 0.013192 | 0.138908 | EUR | Complications of | surgical and med |
| 782.3 | 0.050444 | 0.021276 | 2.370876 | 0.017746 | 0.008743 | 0.092145 | EUR | Edema | symptoms |
| 475 | -0.04948 | 0.0209 | -2.36763 | 0.017902 | -0.09044 | -0.00852 | EUR | Chronic sinusitis | respiratory |
| 365.5 | 0.411373 | 0.173756 | 2.367531 | 0.017907 | 0.070817 | 0.751928 | EUR | Pseudoexfoliatio | sense organs |
| 433.3 | 0.065678 | 0.02775 | 2.366796 | 0.017943 | 0.01129 | 0.120067 | EUR | Cerebral ischemi | circulatory system |
| 733.4 | 0.163207 | 0.069021 | 2.364598 | 0.01805 | 0.027928 | 0.298485 | EUR | Aseptic necrosis | musculoskeletal |
| 797 | 0.095283 | 0.040321 | 2.363098 | 0.018123 | 0.016255 | 0.17431 | EUR | Shock | symptoms |
| 536.8 | -0.0762 | 0.032253 | -2.36263 | 0.018146 | -0.13941 | -0.01299 | EUR | Dyspepsia and o | digestive |
| 728.1 | 0.432534 | 0.183077 | 2.362575 | 0.018148 | 0.073709 | 0.791359 | EUR | Muscular calcific | musculoskeletal |
| 276.13 | 0.067095 | 0.028412 | 2.361515 | 0.0182 | 0.011409 | 0.122781 | EUR | Hyperpotassemia | endocrine/metabolic |
| 695.21 | 0.549324 | 0.232737 | 2.360283 | 0.018261 | 0.093169 | 1.005479 | EUR | Dermatitis herpet | dermatologic |
| 443.7 | 0.156716 | 0.066435 | 2.358957 | 0.018326 | 0.026507 | 0.286925 | EUR | Peripheral angiop | circulatory system |
| 975 | 0.413489 | 0.17573 | 2.352981 | 0.018624 | 0.069065 | 0.757912 | EUR | Poisoning by age | injuries & poisonings |
| 737.2 | 0.495223 | 0.210485 | 2.352773 | 0.018634 | 0.08268 | 0.907766 | EUR | Lordosis (acquire | musculoskeletal |
| 562.2 | 0.100009 | 0.042525 | 2.351773 | 0.018684 | 0.016662 | 0.183357 | EUR | Diverticulitis | digestive |
| 971 | 0.484342 | 0.20613 | 2.34969 | 0.018789 | 0.080334 | 0.88835 | EUR | Poisoning by dru | injuries & poisonings |
| 614.5 | -0.07399 | 0.031519 | -2.34758 | 0.018896 | -0.13577 | -0.01222 | EUR | Inflammatory dise | genitourinary |
| 149 | 0.134387 | 0.057278 | 2.346223 | 0.018965 | 0.022124 | 0.246651 | EUR | Cancer of larynx, | neoplasms |
| 661 | -0.16362 | 0.069771 | -2.34512 | 0.019021 | -0.30037 | -0.02687 | EUR | Fetal distress and | pregnancy complications |
| 286 | 0.062506 | 0.026708 | 2.34032 | 0.019267 | 0.010159 | 0.114853 | EUR | Coagulation defe | hematopoietic |
| 332 | -0.16313 | 0.06978 | -2.33777 | 0.019399 | -0.29989 | -0.02636 | EUR | Parkinson's disea | neurological |
| 427.2 | 0.056341 | 0.024101 | 2.337749 | 0.0194 | 0.009105 | 0.103578 | EUR | Atrial fibrillation a | circulatory system |
| 402 | -0.0592 | 0.025358 | -2.33443 | 0.019573 | -0.1089 | -0.0095 | EUR | Elevated blood p | circulatory system |
| 855 | 0.299969 | 0.128514 | 2.334142 | 0.019588 | 0.048087 | 0.551851 | EUR | Complication of r | injuries & poisonings |
| 610 | -0.08358 | 0.035929 | -2.32616 | 0.02001 | -0.154 | -0.01316 | EUR | Benign mammary | genitourinary |
| 327 | 0.040696 | 0.017497 | 2.325905 | 0.020024 | 0.006403 | 0.074989 | EUR | Sleep disorders | neurological |
| 255.1 | -0.19562 | 0.084177 | -2.32396 | 0.020128 | -0.36061 | -0.03064 | EUR | Adrenal hyperfun | endocrine/metabolic |
| 225.2 | 0.425338 | 0.183154 | 2.3223 | 0.020217 | 0.066363 | 0.784312 | EUR | Benign neoplasm | neoplasms |
| 149.2 | 0.425165 | 0.183305 | 2.319436 | 0.020371 | 0.065893 | 0.784437 | EUR | Cancer of nasopl | neoplasms |
| 964.1 | 0.315 | 0.135861 | 2.31855 | 0.020419 | 0.048718 | 0.581282 | EUR | Anticoagulants ca | injuries & poisonings |
| 288.2 | 0.052243 | 0.022558 | 2.315926 | 0.020562 | 0.00803 | 0.096456 | EUR | Elevated white bl | hematopoietic |

| phecode | Coef. | Std.Err. | z | p_value_z | [0.025 | 0.975] | GIA | phenotype | category |
|---|---|---|---|---|---|---|---|---|---|
| 750.15 | -0.40735 | 0.176186 | -2.31202 | 0.020776 | -0.75266 | -0.06203 | EUR | Congenital anom | congenital anomalies |
| 117.3 | 1.422169 | 0.617244 | 2.304065 | 0.021219 | 0.212394 | 2.631944 | EUR | Blastomycotic inf | infectious diseases |
| 384.4 | -0.22576 | 0.098099 | -2.30136 | 0.021371 | -0.41803 | -0.03349 | EUR | Perforation of tym | sense organs |
| 288 | 0.04986 | 0.021687 | 2.29905 | 0.021502 | 0.007354 | 0.092365 | EUR | Diseases of white | hematopoietic |
| 756.3 | -0.26034 | 0.11373 | -2.28915 | 0.022071 | -0.48325 | -0.03744 | EUR | Congenital anom | congenital anomalies |
| 149.9 | 0.347393 | 0.151943 | 2.286338 | 0.022234 | 0.04959 | 0.645196 | EUR | Cancer of of nas | neoplasms |
| 577.2 | 0.176983 | 0.077409 | 2.286335 | 0.022235 | 0.025264 | 0.328702 | EUR | Chronic pancreat | digestive |
| 722.9 | 0.088153 | 0.038655 | 2.280531 | 0.022576 | 0.012391 | 0.163914 | EUR | Other and unspe | musculoskeletal |
| 350.5 | -0.24611 | 0.108292 | -2.27269 | 0.023045 | -0.45836 | -0.03387 | EUR | Abnormal reflex | neurological |
| 562.1 | 0.047227 | 0.020789 | 2.271782 | 0.0231 | 0.006482 | 0.087972 | EUR | Diverticulosis | digestive |
| 706.2 | -0.05907 | 0.026019 | -2.27039 | 0.023184 | -0.11007 | -0.00808 | EUR | Sebaceous cyst | dermatologic |
| 636.3 | -0.17603 | 0.07769 | -2.26579 | 0.023464 | -0.3283 | -0.02376 | EUR | Hemorrhage in e | pregnancy complications |
| 960 | 0.045578 | 0.020167 | 2.260053 | 0.023818 | 0.006052 | 0.085105 | EUR | Poisoning by ant | injuries & poisonings |
| 324 | -0.17252 | 0.076384 | -2.25858 | 0.023909 | -0.32223 | -0.02281 | EUR | Other CNS infect | neurological |
| 363.4 | 0.656124 | 0.291135 | 2.253677 | 0.024217 | 0.08551 | 1.226739 | EUR | Choroidal degen | sense organs |
| 715 | 0.074967 | 0.033285 | 2.252309 | 0.024303 | 0.009731 | 0.140204 | EUR | Other inflammato | musculoskeletal |
| 754.2 | 0.146819 | 0.065399 | 2.244959 | 0.024771 | 0.018638 | 0.274999 | EUR | Spondylolisthesis | congenital anomalies |
| 710.12 | 0.209002 | 0.093135 | 2.244071 | 0.024828 | 0.02646 | 0.391544 | EUR | Chronic osteomy | musculoskeletal |
| 349 | -0.0863 | 0.038477 | -2.2429 | 0.024904 | -0.16171 | -0.01089 | EUR | Other and unspe | neurological |
| 627.2 | -0.05197 | 0.023193 | -2.24095 | 0.02503 | -0.09743 | -0.00652 | EUR | Symptomatic me | genitourinary |
| 276.4 | 0.069964 | 0.031247 | 2.239056 | 0.025152 | 0.008721 | 0.131208 | EUR | Acid-base balanc | endocrine/metabolic |
| 599.3 | -0.05116 | 0.022853 | -2.23852 | 0.025187 | -0.09595 | -0.00637 | EUR | Dysuria | genitourinary |
| 306.1 | -0.14751 | 0.06591 | -2.23814 | 0.025212 | -0.2767 | -0.01833 | EUR | Mental disorders | mental disorders |
| 258 | 0.389845 | 0.174481 | 2.234316 | 0.025462 | 0.047869 | 0.731822 | EUR | Iatrogenic endoc | endocrine/metabolic |
| 618.1 | 0.111379 | 0.049927 | 2.230859 | 0.02569 | 0.013525 | 0.209233 | EUR | Prolapse of vagir | genitourinary |
| 738.4 | 0.08101 | 0.036329 | 2.229874 | 0.025756 | 0.009806 | 0.152214 | EUR | Acquired spondy | musculoskeletal |
| 426.7 | 0.046166 | 0.020721 | 2.227954 | 0.025884 | 0.005553 | 0.086779 | EUR | Abnormal electro | circulatory system |
| 613 | -0.06317 | 0.028403 | -2.22415 | 0.026138 | -0.11884 | -0.0075 | EUR | Other nonmalign | genitourinary |
| 254 | 0.499905 | 0.224857 | 2.223214 | 0.026201 | 0.059193 | 0.940616 | EUR | Diseases of thym | endocrine/metabolic |
| 389 | -0.04367 | 0.019642 | -2.22308 | 0.02621 | -0.08216 | -0.00517 | EUR | Hearing loss | sense organs |
| 425.2 | 0.130263 | 0.058613 | 2.222408 | 0.026256 | 0.015383 | 0.245143 | EUR | Secondary/extrin | circulatory system |
| 305.2 | -0.17792 | 0.080092 | -2.22149 | 0.026318 | -0.3349 | -0.02095 | EUR | Eating disorder | mental disorders |
| 621 | 0.235306 | 0.105924 | 2.221462 | 0.02632 | 0.027699 | 0.442914 | EUR | Endometrial hype | genitourinary |
| 961.1 | -0.06438 | 0.029109 | -2.21171 | 0.026987 | -0.12143 | -0.00733 | EUR | Poisoning/allergy | injuries & poisonings |
| 564.1 | -0.06483 | 0.029357 | -2.20837 | 0.027219 | -0.12237 | -0.00729 | EUR | Irritable Bowel Sy | digestive |
| 495.1 | 0.130989 | 0.059405 | 2.204999 | 0.027454 | 0.014556 | 0.247421 | EUR | Chronic obstructi | respiratory |
| 714.2 | -0.29429 | 0.133543 | -2.20374 | 0.027542 | -0.55603 | -0.03256 | EUR | Juvenile rheumat | musculoskeletal |

| phecode | Coef. | Std.Err. | z | p_value_z | [0.025 | 0.975] | GIA | phenotype | category |
|---|---|---|---|---|---|---|---|---|---|
| 286.6 | 0.181476 | 0.082354 | 2.203603 | 0.027552 | 0.020065 | 0.342888 | EUR | Defibrination syn | hematopoietic |
| 189.11 | 0.136093 | 0.061889 | 2.199001 | 0.027878 | 0.014794 | 0.257393 | EUR | Malignant neopla | neoplasms |
| 426.91 | 0.088552 | 0.040283 | 2.198242 | 0.027932 | 0.009599 | 0.167505 | EUR | Cardiac pacemal | circulatory system |
| 573 | 0.052592 | 0.023964 | 2.194646 | 0.028189 | 0.005624 | 0.099559 | EUR | Other disorders c | digestive |
| 509.8 | 0.10197 | 0.04656 | 2.19007 | 0.028519 | 0.010714 | 0.193226 | EUR | Dependence on r | respiratory |
| 634.1 | -0.18429 | 0.08429 | -2.18644 | 0.028783 | -0.3495 | -0.01909 | EUR | Missed abortion/l | pregnancy complications |
| 617 | -0.07174 | 0.032842 | -2.18434 | 0.028937 | -0.13611 | -0.00737 | EUR | Disorders second | genitourinary |
| 433 | 0.049529 | 0.022675 | 2.184334 | 0.028938 | 0.005088 | 0.09397 | EUR | Cerebrovascular | circulatory system |
| 430.1 | 0.205431 | 0.094214 | 2.180485 | 0.029222 | 0.020776 | 0.390086 | EUR | Subarachnoid he | circulatory system |
| 585 | 0.043911 | 0.020147 | 2.179504 | 0.029294 | 0.004423 | 0.083398 | EUR | Renal failure | genitourinary |
| 619 | -0.05082 | 0.023416 | -2.17023 | 0.02999 | -0.09671 | -0.00492 | EUR | Noninflammatory | genitourinary |
| 291.8 | 0.076511 | 0.035285 | 2.168402 | 0.030128 | 0.007355 | 0.145667 | EUR | Alteration of cons | mental disorders |
| 524.3 | 0.457598 | 0.211045 | 2.168251 | 0.03014 | 0.043958 | 0.871238 | EUR | Anomalies of too | digestive |
| 261.4 | -0.04262 | 0.019681 | -2.16577 | 0.030328 | -0.0812 | -0.00405 | EUR | Vitamin D deficie | endocrine/metabolic |
| 580.13 | 0.467325 | 0.216103 | 2.16251 | 0.030579 | 0.043771 | 0.890879 | EUR | Acute glomerulor | genitourinary |
| 704.2 | -0.20379 | 0.094241 | -2.16241 | 0.030587 | -0.3885 | -0.01908 | EUR | Hirsutism | dermatologic |
| 285.3 | 0.655601 | 0.30332 | 2.161418 | 0.030663 | 0.061105 | 1.250097 | EUR | Sideroblastic ane | hematopoietic |
| 713 | 0.251186 | 0.116227 | 2.161175 | 0.030682 | 0.023386 | 0.478987 | EUR | Arthropathy asso | musculoskeletal |
| 724.1 | 0.071232 | 0.032983 | 2.159692 | 0.030797 | 6.59E-03 | 0.135877 | EUR | Disorders of sacr | musculoskeletal |
| 994.2 | 0.058438 | 0.027065 | 2.159174 | 0.030837 | 0.005392 | 0.111485 | EUR | Sepsis | injuries & poisonings |
| 755.3 | 0.480855 | 0.222925 | 2.157027 | 0.031004 | 0.04393 | 0.917781 | EUR | Congenital anom | congenital anomalies |
| 283.1 | 0.344242 | 0.159791 | 2.154331 | 0.031214 | 0.031058 | 0.657426 | EUR | Autoimmune hen | hematopoietic |
| 870.2 | 0.248361 | 0.115521 | 2.149918 | 0.031562 | 0.021944 | 0.474778 | EUR | Open wound of e | injuries & poisonings |
| 283.2 | 0.404453 | 0.188298 | 2.14794 | 0.031719 | 0.035396 | 0.773511 | EUR | Non-autoimmune | hematopoietic |
| 361 | -0.10949 | 0.050981 | -2.14772 | 3.17E-02 | -0.20941 | -0.00957 | EUR | Retinal detachme | sense organs |
| 371.9 | 0.80445 | 0.375044 | 2.144947 | 0.031957 | 0.069377 | 1.539523 | EUR | Chronic inflamma | sense organs |
| 374.2 | 0.248619 | 0.116056 | 2.142233 | 0.032175 | 0.021153 | 0.476084 | EUR | Lagophthalmos | sense organs |
| 394.3 | 0.266016 | 0.124282 | 2.140417 | 3.23E-02 | 0.022427 | 0.509605 | EUR | Aortic valve disea | circulatory system |
| 270.2 | 0.364216 | 0.170253 | 2.139264 | 0.032414 | 0.030526 | 0.697906 | EUR | Disorders of amir | endocrine/metabolic |
| 519.1 | 0.352927 | 0.165344 | 2.134501 | 0.032802 | 0.028859 | 0.676995 | EUR | Tracheostomy cc | respiratory |
| 720 | 0.05012 | 0.023534 | 2.129643 | 0.033201 | 0.003993 | 0.096246 | EUR | Spinal stenosis | musculoskeletal |
| 626.11 | -0.11229 | 0.052823 | -2.12569 | 0.033529 | -0.21582 | -0.00875 | EUR | Absent or infrequ | genitourinary |
| 276.14 | 0.063485 | 0.029925 | 2.121469 | 0.033882 | 0.004833 | 0.122137 | EUR | Hypopotassemia | endocrine/metabolic |
| 610.4 | -0.16081 | 0.075872 | -2.11945 | 0.034053 | -0.30951 | -0.0121 | EUR | Benign neoplasm | genitourinary |
| 155.1 | 0.129914 | 0.061297 | 2.119419 | 0.034055 | 0.009774 | 0.250054 | EUR | Malignant neopla | neoplasms |
| 1006 | 0.485728 | 0.229324 | 2.11808 | 0.034168 | 0.03626 | 0.935195 | EUR | Crushing injury | |
| 770 | 0.042976 | 0.020327 | 2.114252 | 0.034494 | 0.003136 | 0.082815 | EUR | Myalgia and myo | symptoms |

| phecode | Coef. | Std.Err. | z | p_value_z | [0.025 | 0.975] | GIA | phenotype | category |
|---|---|---|---|---|---|---|---|---|---|
| 665 | -0.10812 | 0.05115 | -2.11385 | 0.034528 | -0.20838 | -0.00787 | EUR | Obstetrical/birth t | pregnancy complications |
| 913 | -0.18035 | 0.085336 | -2.11345 | 0.034562 | -0.34761 | -0.0131 | EUR | Toxic effect of ve | injuries & poisonings |
| 187.2 | 0.250811 | 0.118907 | 2.109307 | 0.034918 | 0.017758 | 0.483864 | EUR | Malignant neopla | neoplasms |
| 367.1 | -0.08156 | 0.038788 | -2.10267 | 0.035495 | -0.15758 | -0.00554 | EUR | Myopia | sense organs |
| 199 | -0.04365 | 0.020764 | -2.10218 | 0.035538 | -0.08435 | -0.00295 | EUR | Neoplasm of unc | neoplasms |
| 611.11 | -0.12687 | 0.060369 | -2.10159 | 0.035589 | -0.24519 | -0.00855 | EUR | Mammographic r | genitourinary |
| 614.52 | -0.07707 | 0.03674 | -2.09766 | 0.035935 | -0.14908 | -0.00506 | EUR | Vaginitis and vulv | genitourinary |
| 530.14 | 0.067875 | 0.032394 | 2.095276 | 0.036146 | 0.004383 | 0.131366 | EUR | Reflux esophagit | digestive |
| 522.1 | -3.44781 | 1.647735 | -2.09245 | 0.036398 | -6.67731 | -0.21831 | EUR | Pulpitis and necr | digestive |
| 371 | -0.0502 | 0.024011 | -2.09048 | 0.036574 | -0.09726 | -0.00313 | EUR | Inflammation of t | sense organs |
| 781.2 | -0.14884 | 0.071411 | -2.08432 | 0.037131 | -0.28881 | -0.00888 | EUR | Abnormal postur | symptoms |
| 260.6 | -0.09092 | 0.04368 | -2.08152 | 0.037386 | -0.17653 | -0.00531 | EUR | Anorexia | endocrine/metabolic |
| 337.1 | 0.180545 | 0.08686 | 2.078572 | 3.77E-02 | 0.010302 | 0.350787 | EUR | Peripheral auton | neurological |
| 526.5 | 0.3172 | 0.152734 | 2.076806 | 0.037819 | 0.017846 | 0.616554 | EUR | Inflammatory cor | digestive |
| 870.6 | 0.404945 | 0.195274 | 2.073725 | 0.038105 | 0.022215 | 0.787675 | EUR | Open wound of n | injuries & poisonings |
| 742.1 | 0.358946 | 0.17328 | 2.071476 | 0.038314 | 0.019323 | 0.698569 | EUR | Loose body in joi | musculoskeletal |
| 495.11 | 0.235679 | 0.113824 | 2.070545 | 0.038401 | 0.012587 | 0.458771 | EUR | Chronic obstructi | respiratory |
| 442.2 | 0.262403 | 0.126776 | 2.069812 | 0.03847 | 0.013926 | 0.510881 | EUR | Aneurysm of iliac | circulatory system |
| 389.2 | -0.1513 | 0.07313 | -2.06891 | 0.038554 | -0.29463 | -0.00797 | EUR | Conductive heari | sense organs |
| 327.41 | -0.06266 | 0.030348 | -2.06479 | 0.038943 | -0.12214 | -0.00318 | EUR | Organic or persis | neurological |
| 323 | -0.17728 | 0.085952 | -2.06249 | 0.039161 | -0.34574 | -0.00881 | EUR | Encephalitis | neurological |
| 712 | 0.329768 | 0.159979 | 2.061321 | 0.039272 | 0.016215 | 0.64332 | EUR | Infective connect | musculoskeletal |
| 480.12 | 0.204149 | 0.099147 | 2.059054 | 3.95E-02 | 0.009824 | 0.398474 | EUR | Pseudomonal pn | respiratory |
| 597.8 | -0.24194 | 0.117856 | -2.05281 | 0.040091 | -0.47293 | -0.01094 | EUR | Urethral hypermo | genitourinary |
| 965.3 | 0.470451 | 0.229496 | 2.049928 | 0.040371 | 0.020646 | 0.920255 | EUR | Salicylates causi | injuries & poisonings |
| 800.2 | 0.187429 | 0.091437 | 2.049824 | 0.040382 | 0.008216 | 0.366641 | EUR | Fracture of unspe | injuries & poisonings |
| 440.1 | 0.243381 | 0.118838 | 2.048012 | 0.040559 | 0.010463 | 0.4763 | EUR | Atherosclerosis c | circulatory system |
| 981 | 0.779982 | 0.380879 | 2.047851 | 0.040575 | 0.033474 | 1.52649 | EUR | Toxic effect of (no | injuries & poisonings |
| 428.2 | 0.083056 | 0.04069 | 2.041188 | 0.041232 | 0.003305 | 0.162807 | EUR | Heart failure NOS | circulatory system |
| 512.9 | 0.037695 | 0.018467 | 2.04119 | 0.041232 | 0.0015 | 0.07389 | EUR | Other dyspnea | respiratory |
| 703 | -0.06554 | 0.032201 | -2.03552 | 0.041798 | -0.12866 | -0.00243 | EUR | Diseases of nail, | dermatologic |
| 396 | -0.05751 | 0.028262 | -2.03493 | 0.041858 | -0.1129 | -0.00212 | EUR | Abnormal heart s | circulatory system |
| 379.1 | 0.336818 | 0.165644 | 2.033392 | 0.042013 | 0.012163 | 0.661474 | EUR | Scleritis and epis | sense organs |
| 528.5 | -0.11158 | 0.054885 | -2.03298 | 0.042055 | -0.21915 | -0.00401 | EUR | Diseases of lips | digestive |
| 772.2 | 0.06981 | 0.034393 | 2.029787 | 0.042378 | 0.002401 | 0.137219 | EUR | Spasm of muscle | symptoms |
| 446.2 | 0.769154 | 0.379548 | 2.026499 | 0.042714 | 0.025253 | 1.513054 | EUR | Acute febrile muc | circulatory system |
| 346.2 | 0.249272 | 0.123199 | 2.023323 | 0.04304 | 0.007806 | 0.490738 | EUR | Nonspecific abno | neurological |

| phecode | Coef. | Std.Err. | z | p_value_z | [0.025 | 0.975] | GIA | phenotype | category |
|---|---|---|---|---|---|---|---|---|---|
| 613.8 | -0.08452 | 0.041774 | -2.02321 | 0.043052 | -0.16639 | -0.00264 | EUR | Other specified d | genitourinary |
| 446.6 | 0.498674 | 0.246784 | 2.020692 | 0.043312 | 0.014987 | 0.982362 | EUR | Polyarteritis nodo | circulatory system |
| 736.1 | 0.334036 | 0.165635 | 2.016706 | 0.043726 | 0.009399 | 0.658674 | EUR | Acquired deformi | musculoskeletal |
| 686.4 | -0.22555 | 0.112 | -2.01383 | 0.044028 | -0.44506 | -0.00603 | EUR | Pyogenic granulo | dermatologic |
| 38 | 0.053078 | 0.026375 | 2.012433 | 0.044174 | 0.001384 | 0.104772 | EUR | Septicemia | infectious diseases |
| 601.12 | -0.17031 | 0.084674 | -2.01132 | 0.044291 | -0.33627 | -0.00435 | EUR | Chronic prostatiti | genitourinary |
| 312.3 | 0.368072 | 0.183039 | 2.01089 | 0.044337 | 0.009322 | 0.726822 | EUR | Impulse control d | mental disorders |
| 264.1 | 0.841557 | 0.418558 | 2.010614 | 0.044366 | 0.0212 | 1.661915 | EUR | Short stature | endocrine/metabolic |
| 958.2 | 0.400764 | 0.19934 | 2.010453 | 0.044383 | 0.010065 | 0.791464 | EUR | Traumatic and su | injuries & poisonings |
| 504.1 | 0.118562 | 0.059101 | 2.0061 | 0.044846 | 0.002727 | 0.234396 | EUR | Idiopathic fibrosir | respiratory |
| 442.11 | 0.125498 | 0.062716 | 2.001069 | 0.045385 | 0.002578 | 0.248419 | EUR | Abdominal aortic | circulatory system |
| 429.1 | 0.091679 | 0.045871 | 1.998641 | 0.045647 | 0.001774 | 0.181585 | EUR | Heart transplant/s | circulatory system |
| 454.11 | 0.077587 | 0.038933 | 1.992848 | 0.046278 | 0.00128 | 0.153894 | EUR | Varicose veins of | circulatory system |
| 290.2 | 0.115729 | 0.058277 | 1.985834 | 0.047052 | 0.001508 | 0.229951 | EUR | Delirium due to c | mental disorders |
| 364.5 | -1.54E-01 | 0.077463 | -1.9849 | 0.047156 | -0.30558 | -0.00193 | EUR | Corneal dystroph | sense organs |
| 227.1 | 0.160806 | 0.081062 | 1.983731 | 0.047286 | 0.001927 | 0.319685 | EUR | Benign neoplasm | neoplasms |
| 145.5 | 0.348093 | 0.175853 | 1.979456 | 0.047765 | 0.003428 | 0.692758 | EUR | Cancer of the mo | neoplasms |
| 433.6 | 0.317528 | 0.161039 | 1.971752 | 0.048638 | 0.001898 | 0.633158 | EUR | Acute, but ill-defi | circulatory system |
| 456 | 0.062266 | 0.031585 | 1.971359 | 0.048683 | 0.00036 | 0.124171 | EUR | Chronic venous i | circulatory system |
| 986 | -1.4889 | 0.755643 | -1.97037 | 0.048796 | -2.96993 | -0.00786 | EUR | Toxic effect of ca | injuries & poisonings |
| 260.2 | 0.087934 | 0.044713 | 1.966645 | 0.049224 | 0.000299 | 0.175569 | EUR | severe protein-ca | endocrine/metabolic |
| 348.4 | 0.22065 | 0.112219 | 1.96624 | 0.049271 | 0.000704 | 0.440596 | EUR | Cerebral cysts | neurological |
| 433.31 | 0.058757 | 0.029963 | 1.960992 | 0.04988 | 3.08E-05 | 0.117484 | EUR | Transient cerebra | circulatory system |
| 535.9 | 0.071767 | 0.036697 | 1.955681 | 0.050503 | -0.00016 | 0.143691 | EUR | Gastritis and duo | digestive |
| 359.1 | 0.369191 | 0.188816 | 1.955288 | 0.050549 | -0.00088 | 0.739264 | EUR | Muscular dystrop | neurological |
| 292.5 | 0.132516 | 0.0678 | 1.954514 | 0.05064 | -0.00037 | 0.265401 | EUR | Transient alterati | mental disorders |
| 165.1 | 0.08428 | 0.043155 | 1.952945 | 0.050826 | -0.0003 | 0.168862 | EUR | Cancer of bronch | neoplasms |
| 334.1 | -0.24323 | 0.124644 | -1.9514 | 0.05101 | -0.48753 | 0.001068 | EUR | Spinocerebellar c | neurological |
| 276.6 | 0.069082 | 0.035468 | 1.947742 | 0.051446 | -0.00043 | 0.138597 | EUR | Fluid overload | endocrine/metabolic |
| 79 | -0.03712 | 0.019071 | -1.94626 | 0.051624 | -0.07449 | 0.000261 | EUR | Viral infection | infectious diseases |
| 394.1 | -0.14444 | 0.074227 | -1.94591 | 0.051665 | -0.28992 | 0.001043 | EUR | Mitral valve stenc | circulatory system |
| 430.3 | 0.15026 | 0.077344 | 1.94275 | 0.052046 | -0.00133 | 0.301852 | EUR | Subdural hemorr | circulatory system |
| 645 | -0.1338 | 0.068884 | -1.94234 | 0.052096 | -0.26881 | 0.001214 | EUR | Late pregnancy a | pregnancy complications |
| 316.1 | -0.09995 | 0.051541 | -1.93913 | 0.052485 | -0.20096 | 0.001074 | EUR | Polyneuropathy c | mental disorders |
| 704.11 | 0.247837 | 0.127814 | 1.939036 | 0.052497 | -0.00267 | 0.498348 | EUR | Alopecia Areata | dermatologic |
| 229.1 | 0.737636 | 0.381033 | 1.935885 | 0.052882 | -0.00917 | 1.484447 | EUR | Benign neoplasm | neoplasms |
| 281.1 | 0.130594 | 0.067477 | 1.935392 | 0.052942 | -0.00166 | 0.262845 | EUR | Megaloblastic an | hematopoietic |

| phecode | Coef. | Std.Err. | z | p_value_z | [0.025 | 0.975] | GIA | phenotype | category | |
|---|---|---|---|---|---|---|---|---|---|---|
| 184.11 | -0.13106 | 0.067991 | -1.9276 | 0.053905 | -0.26432 | 0.0022 | EUR | Malignant neopla | neoplasms | |
| 295.3 | 0.103638 | 0.053791 | 1.92666 | 0.054022 | -0.00179 | 0.209067 | EUR | Psychosis | mental disorders | |
| 204.22 | 0.320085 | 0.166148 | 1.926509 | 5.40E-02 | -0.00556 | 0.645729 | EUR | Myeloid leukemia | neoplasms | |
| 334 | -0.09253 | 0.04814 | -1.92212 | 0.05459 | -0.18688 | 0.001822 | EUR | Degenerative dis | neurological | |
| 370.3 | -1.77E-01 | 0.09249 | -1.91883 | 0.055006 | -0.35875 | 0.003805 | EUR | Keratoconjunctivi | sense organs | |
| 250.5 | 0.324394 | 0.169526 | 1.913538 | 0.055679 | -0.00787 | 0.656659 | EUR | Glycosuria or Ace | endocrine/metabolic | |
| 334.21 | 0.504294 | 0.263835 | 1.911403 | 0.055953 | -0.01281 | 1.021401 | EUR | Amyotrophic Late | neurological | |
| 715.3 | 0.239269 | 0.125329 | 1.909121 | 0.056247 | -0.00637 | 0.48491 | EUR | Spinal enthesopa | musculoskeletal | |
| 740.1 | 0.035865 | 0.01881 | 1.906721 | 0.056557 | -0.001 | 0.072732 | EUR | Osteoarthritis; loc | musculoskeletal | |
| 729 | 0.039905 | 0.020931 | 1.906491 | 0.056587 | -0.00112 | 0.080929 | EUR | Other disorders c | musculoskeletal | |
| 270.31 | 0.416329 | 0.218882 | 1.902069 | 0.057162 | -0.01267 | 0.84533 | EUR | Polyclonal hyperg | endocrine/metabolic | |
| 216.1 | -0.04336 | 0.022818 | -1.90015 | 0.057413 | -0.08808 | 0.001365 | EUR | Screening for ma | neoplasms | |
| 585.33 | 0.050599 | 0.026646 | 1.898934 | 0.057573 | -0.00163 | 0.102824 | EUR | Chronic Kidney D | genitourinary | |
| 519.2 | 0.166247 | 0.087614 | 1.897481 | 0.057765 | -0.00547 | 0.337968 | EUR | Respiratory comp | respiratory | |
| 275.1 | 0.153533 | 0.080963 | 1.896336 | 0.057916 | -0.00515 | 0.312217 | EUR | Disorders of iron | hematopoietic | |
| 818.2 | 0.355891 | 0.187735 | 1.895708 | 0.057999 | -0.01206 | 0.723845 | EUR | Subarachnoid he | injuries & poisonings | |
| 740.3 | 0.11094 | 0.058531 | 1.895406 | 0.058039 | -0.00378 | 0.225659 | EUR | Osteoarthrosis in | musculoskeletal | |
| 710.19 | 0.128361 | 0.067815 | 1.892813 | 0.058383 | -0.00455 | 0.261275 | EUR | Unspecified oste | musculoskeletal | |
| 513.32 | 0.212909 | 0.112564 | 1.891446 | 0.058565 | -0.00771 | 0.433531 | EUR | Orthopnea | respiratory | |
| 1009 | 0.057055 | 0.030211 | 1.88857 | 0.058949 | -0.00216 | 0.116267 | EUR | Injury, NOS | | |
| 81.1 | 0.253894 | 0.134501 | 1.88767 | 0.05907 | -0.00972 | 0.517511 | EUR | Graft-versus-hos | infectious diseases | |
| 870.5 | 0.156592 | 0.082983 | 1.887038 | 0.059155 | -0.00605 | 0.319235 | EUR | Open wound of li | injuries & poisonings | |
| 709.5 | 0.27174 | 0.144021 | 1.886804 | 0.059187 | -0.01054 | 0.554016 | EUR | Dermatomyositis | dermatologic | |
| 415.21 | 0.10961 | 0.058133 | 1.885509 | 0.059361 | -0.00433 | 0.223549 | EUR | Primary pulmona | circulatory system | |
| 647 | -0.17018 | 0.090415 | -1.88225 | 0.059802 | -0.34739 | 0.007027 | EUR | Infectious and pa | pregnancy complications | |
| 170.1 | 0.129232 | 0.068713 | 1.880741 | 0.060007 | -0.00544 | 0.263907 | EUR | Bone cancer | neoplasms | |
| 352.1 | 0.147703 | 0.078666 | 1.877609 | 0.060435 | -0.00648 | 0.301885 | EUR | Trigeminal nerve | neurological | |
| 851 | 0.089737 | 0.047837 | 1.875876 | 0.060672 | -0.00402 | 0.183496 | EUR | Complications of | injuries & poisonings | |
| 558 | 0.049111 | 0.026183 | 1.875673 | 0.0607 | -0.00221 | 0.10043 | EUR | Noninfectious ga | digestive | |
| 371.21 | -0.08943 | 0.047717 | -1.87423 | 0.060898 | -0.18296 | 0.004091 | EUR | Allergic conjuncti | sense organs | |
| 368 | -0.04294 | 0.022932 | -1.87258 | 0.061126 | -0.08789 | 0.002004 | EUR | Visual disturbanc | sense organs | |
| 189.1 | 0.110271 | 0.058992 | 1.869265 | 0.061586 | -0.00535 | 0.225892 | EUR | Cancer of kidney | neoplasms | |
| 681.1 | -0.07762 | 0.041579 | -1.86676 | 0.061934 | -0.15911 | 0.003875 | EUR | Cellulitis and abs | dermatologic | |
| 610.2 | -0.1871 | 0.100291 | -1.86561 | 0.062096 | -0.38367 | 0.009463 | EUR | Fibroadenosis of | genitourinary | |
| 385.3 | 0.289625 | 0.155716 | 1.859952 | 0.062892 | -0.01557 | 0.594823 | EUR | Cholesteatoma | sense organs | |
| 198.7 | -0.22846 | 0.122939 | -1.8583 | 0.063127 | -0.46941 | 0.012499 | EUR | Secondary malig | neoplasms | |
| 170 | 0.084805 | 0.045682 | 1.856443 | 0.06339 | -0.00473 | 0.174339 | EUR | Cancer of bone a | neoplasms | |

| phecode | Coef. | Std.Err. | z | p_value_z | [0.025 | 0.975] | GIA | phenotype | category |
|---|---|---|---|---|---|---|---|---|---|
| 159.3 | 0.289593 | 0.156268 | 1.853178 | 0.063857 | -0.01669 | 0.595874 | EUR | Malignant neopla | neoplasms |
| 724.2 | 0.063045 | 0.034114 | 1.848074 | 0.064592 | -0.00382 | 0.129908 | EUR | Disorders of cocc | musculoskeletal |
| 427.5 | -0.06127 | 0.033167 | -1.84719 | 0.064719 | -0.12627 | 0.00374 | EUR | Arrhythmia (cardi | circulatory system |
| 149.5 | 0.134337 | 0.072957 | 1.84131 | 0.065576 | -0.00866 | 0.277331 | EUR | Hx of malignant r | neoplasms |
| 939 | -0.03678 | 0.02005 | -1.83423 | 0.06662 | -0.07607 | 0.002521 | EUR | Atopic/contact de | dermatologic |
| 611.3 | -0.05469 | 0.029927 | -1.82758 | 0.067612 | -0.11335 | 0.003962 | EUR | Lump or mass in | genitourinary |
| 731 | 0.0808 | 0.044237 | 1.826542 | 0.067769 | -0.0059 | 0.167502 | EUR | Osteitis deformar | musculoskeletal |
| 41.12 | 0.127152 | 0.069626 | 1.826231 | 0.067816 | -0.00931 | 0.263616 | EUR | Methicillin resista | infectious diseases |
| 480 | 0.040723 | 0.022308 | 1.825477 | 0.067929 | -0.003 | 0.084446 | EUR | Pneumonia | respiratory |
| 313.2 | -0.29699 | 0.162836 | -1.82384 | 0.068176 | -0.61614 | 0.022166 | EUR | Tics and stutterin | mental disorders |
| 624.2 | 0.299673 | 0.164714 | 1.819353 | 0.068858 | -0.02316 | 0.622508 | EUR | Atrophy of female | genitourinary |
| 756.5 | 0.281182 | 0.154602 | 1.818753 | 0.068949 | -0.02183 | 0.584196 | EUR | Congenital osteo | congenital anomalies |
| 743.12 | -0.06357 | 0.034996 | -1.81637 | 0.069314 | -0.13216 | 0.005025 | EUR | Senile osteoporo | musculoskeletal |
| 592.13 | -0.1788 | 0.098451 | -1.81609 | 0.069356 | -0.37176 | 0.014165 | EUR | Chronic interstitia | genitourinary |
| 733 | -0.03825 | 0.02109 | -1.81372 | 0.069721 | -0.07959 | 0.003084 | EUR | Other disorders c | musculoskeletal |
| 286.11 | 0.413537 | 0.229155 | 1.804616 | 0.071135 | -0.0356 | 0.862673 | EUR | Von willebrand's | hematopoietic |
| 362.23 | -0.11311 | 0.062741 | -1.80272 | 0.071432 | -0.23608 | 0.009866 | EUR | Cystoid macular | sense organs |
| 592.2 | -0.14309 | 0.079428 | -1.80156 | 0.071615 | -0.29877 | 0.012582 | EUR | Urethritis and ure | genitourinary |
| 426.92 | 0.096165 | 0.053384 | 1.8014 | 0.07164 | -0.00846 | 0.200795 | EUR | Cardiac defibrilla | circulatory system |
| 694.3 | -0.34093 | 0.189314 | -1.80089 | 0.07172 | -0.71198 | 0.030115 | EUR | Vascular disorde | dermatologic |
| 278.4 | 0.056209 | 0.031218 | 1.800519 | 0.071779 | -0.00498 | 0.117395 | EUR | Abnormal weight | endocrine/metabolic |
| 327.31 | 0.094639 | 0.052601 | 1.799189 | 0.071989 | -0.00846 | 0.197736 | EUR | Central/nonobstr | neurological |
| 251.8 | 0.472089 | 0.262434 | 1.79889 | 7.20E-02 | -0.04227 | 0.98645 | EUR | Abnormality of se | endocrine/metabolic |
| 635.2 | -0.12811 | 0.071295 | -1.79687 | 0.072356 | -0.26784 | 0.011628 | EUR | Antepartum hem | pregnancy complications |
| 528 | -0.05237 | 0.029179 | -1.79495 | 0.072662 | -0.10956 | 0.004815 | EUR | Diseases of the c | digestive |
| 369.5 | -0.05661 | 0.031543 | -1.7946 | 0.072717 | -0.11843 | 0.005216 | EUR | Conjunctivitis, inf | sense organs |
| 602.3 | 0.29577 | 0.164846 | 1.794226 | 0.072777 | -0.02732 | 0.618862 | EUR | Dysplasia of pros | genitourinary |
| 569 | 0.038674 | 0.021568 | 1.793111 | 0.072955 | -0.0036 | 0.080947 | EUR | Other disorders c | digestive |
| 364.41 | 0.299311 | 0.167173 | 1.790425 | 7.34E-02 | -0.02834 | 0.626964 | EUR | Keratoconus | sense organs |
| 333 | -0.06521 | 0.036482 | -1.78737 | 0.073878 | -0.13671 | 0.006297 | EUR | Extrapyramidal d | neurological |
| 446.5 | 0.238461 | 0.13364 | 1.784352 | 0.074366 | -0.02347 | 0.500391 | EUR | Giant cell arteritis | circulatory system |
| 200 | 0.070692 | 0.039651 | 1.78284 | 0.074612 | -0.00702 | 0.148407 | EUR | Myeloproliferative | neoplasms |
| 362.3 | -0.14548 | 0.081738 | -1.77981 | 0.075107 | -0.30568 | 0.014725 | EUR | Other nondiabeti | sense organs |
| 536.3 | 0.107562 | 0.060443 | 1.77956 | 0.075148 | -0.0109 | 0.226029 | EUR | Gastroparesis | digestive |
| 270.34 | 0.3331 | 0.187225 | 1.779148 | 0.075216 | -0.03385 | 0.700054 | EUR | Alpha-1-antitryps | endocrine/metabolic |
| 270.35 | 0.306721 | 0.172495 | 1.778144 | 0.07538 | -0.03136 | 0.644805 | EUR | Macroglobulinem | endocrine/metabolic |
| 364.1 | -0.17949 | 0.101041 | -1.77644 | 0.07566 | -0.37753 | 0.018543 | EUR | Corneal opacity | sense organs |

| phecode | Coef. | Std.Err. | z | p_value_z | [0.025 | 0.975] | GIA | phenotype | category |
|---|---|---|---|---|---|---|---|---|---|
| 669 | -0.12257 | 0.068999 | -1.77644 | 0.07566 | -0.25781 | 0.012663 | EUR | Complications of | pregnancy complications |
| 427.4 | 0.111787 | 0.062945 | 1.775949 | 0.075741 | -0.01158 | 0.235158 | EUR | Cardiac arrest ar | circulatory system |
| 451.2 | 0.161978 | 0.091232 | 1.775444 | 0.075825 | -0.01683 | 0.340789 | EUR | Phlebitis and thro | circulatory system |
| 716.1 | 0.154499 | 0.087075 | 1.774327 | 0.076009 | -0.01616 | 0.325163 | EUR | Unspecified polya | musculoskeletal |
| 960.2 | 0.042119 | 0.023745 | 1.773834 | 0.076091 | -0.00442 | 0.088658 | EUR | Allergy/adverse e | injuries & poisonings |
| 727.1 | -0.04245 | 0.023999 | -1.76885 | 0.076918 | -8.95E-02 | 0.004586 | EUR | Synovitis and ten | musculoskeletal |
| 270.11 | -0.16915 | 0.095841 | -1.76495 | 0.077572 | -0.357 | 0.01869 | EUR | Disturbances of s | endocrine/metabolic |
| 427.9 | -0.03886 | 0.022037 | -1.76324 | 0.077859 | -0.08205 | 0.004335 | EUR | Palpitations | circulatory system |
| 446.3 | 0.378688 | 0.21491 | 1.762079 | 0.078056 | -0.04253 | 0.799903 | EUR | Hypersensitivity a | circulatory system |
| 291 | 0.054563 | 0.031075 | 1.755849 | 0.079114 | -0.00634 | 0.115468 | EUR | Other specified n | mental disorders |
| 962 | 0.082623 | 0.047067 | 1.755434 | 0.079185 | -0.00963 | 0.174873 | EUR | Poisoning by hor | injuries & poisonings |
| 285.8 | 0.336302 | 0.191601 | 1.755218 | 0.079222 | -0.03923 | 0.711833 | EUR | Hemoglobinuria | hematopoietic |
| 190 | 0.214374 | 0.122185 | 1.754505 | 0.079344 | -0.0251 | 0.453852 | EUR | Cancer of eye | neoplasms |
| 516.1 | 0.100693 | 0.057392 | 1.754462 | 0.079351 | -0.01179 | 0.21318 | EUR | Hemoptysis | respiratory |
| 753 | -0.1928 | 0.109933 | -1.75379 | 0.079466 | -0.40826 | 0.022665 | EUR | Congenital anom | congenital anomalies |
| 528.1 | -0.07874 | 0.044931 | -1.7525 | 0.079688 | -0.1668 | 0.009321 | EUR | Stomatitis and m | digestive |
| 856 | 0.258035 | 0.1474 | 1.750575 | 0.080019 | -0.03086 | 0.546935 | EUR | Vascular complic | injuries & poisonings |
| 750.14 | 0.169482 | 0.096945 | 1.74823 | 0.080424 | -0.02053 | 0.35949 | EUR | Congenital anom | congenital anomalies |
| 609.1 | -0.2551 | 0.146058 | -1.74657 | 0.080712 | -0.54137 | 0.031168 | EUR | Infertility, male | genitourinary |
| 965.2 | 0.583668 | 0.334807 | 1.743298 | 0.081282 | -0.07254 | 1.239878 | EUR | Antirheumatics ca | injuries & poisonings |
| 740.12 | 0.082575 | 0.047408 | 1.741801 | 0.081543 | -0.01034 | 0.175492 | EUR | Osteoarthrosis, lo | musculoskeletal |
| 649 | -0.07971 | 0.045762 | -1.74178 | 0.081547 | -0.1694 | 0.009985 | EUR | Other conditions | pregnancy complications |
| 916 | 0.042359 | 0.024324 | 1.741425 | 0.081609 | -0.00532 | 0.090033 | EUR | Contusion | injuries & poisonings |
| 791 | 0.099537 | 0.0572 | 1.74015 | 0.081833 | -0.01257 | 0.211648 | EUR | Gangrene | symptoms |
| 250.13 | 0.206205 | 0.118544 | 1.739485 | 0.08195 | -0.02614 | 0.438546 | EUR | Type 1 diabetes v | endocrine/metabolic |
| 277.8 | 0.688014 | 0.395727 | 1.738609 | 0.082104 | -0.0876 | 1.463624 | EUR | Carnitine deficier | endocrine/metabolic |
| 1019 | 0.055136 | 0.031746 | 1.736797 | 0.082423 | -0.00708 | 0.117357 | EUR | Other ill-defined and unknown caus | |
| 395.3 | 0.078918 | 0.045539 | 1.732989 | 0.083098 | -0.01034 | 0.168173 | EUR | Nonrheumatic tric | circulatory system |
| 771.1 | 0.037003 | 0.0214 | 1.729158 | 0.083781 | -0.00494 | 0.078946 | EUR | Swelling of limb | symptoms |
| 709.6 | 0.169393 | 0.098146 | 1.725928 | 0.08436 | -0.02297 | 0.361755 | EUR | Other specified d | dermatologic |
| 524 | 0.278313 | 0.161739 | 1.720751 | 0.085296 | -0.03869 | 0.595316 | EUR | Dentofacial anom | digestive |
| 365 | -0.0513 | 0.029821 | -1.72032 | 0.085375 | -0.10975 | 0.007146 | EUR | Glaucoma | sense organs |
| 722.3 | 0.469894 | 0.273733 | 1.716616 | 0.086049 | -0.06661 | 1.006401 | EUR | Schmorl's nodes | musculoskeletal |
| 41.1 | 0.075409 | 0.04393 | 1.716575 | 0.086057 | -0.01069 | 0.16151 | EUR | Staphylococcus i | infectious diseases |
| 364.4 | -0.16339 | 0.095224 | -1.71589 | 0.086183 | -0.35003 | 0.023242 | EUR | Corneal degener | sense organs |
| 766 | 0.052169 | 0.030413 | 1.715372 | 0.086277 | -0.00744 | 0.111776 | EUR | Neuralgia, neuriti | symptoms |
| 165 | 0.072146 | 0.042094 | 1.713944 | 0.086539 | -0.01036 | 0.154648 | EUR | Cancer within the | neoplasms |

| phecode | Coef. | Std.Err. | z | p_value_z | [0.025 | 0.975] | GIA | phenotype | category |
|---|---|---|---|---|---|---|---|---|---|
| 695.7 | 0.090017 | 0.052607 | 1.711124 | 0.087058 | -0.01309 | 0.193124 | EUR | Prurigo and Liche | dermatologic |
| 452.2 | 0.053934 | 0.031677 | 1.702629 | 0.088637 | -0.00815 | 0.11602 | EUR | Deep vein thromb | circulatory system |
| 651 | -0.13049 | 0.076694 | -1.7014 | 0.088868 | -0.2808 | 0.01983 | EUR | Multiple gestation | pregnancy complications |
| 367.2 | -0.05648 | 0.033235 | -1.69932 | 0.089258 | -0.12161 | 0.008662 | EUR | Astigmatism | sense organs |
| 643 | -0.12791 | 0.075371 | -1.69705 | 0.089687 | -0.27563 | 0.019816 | EUR | Excessive vomiti | pregnancy complications |
| 681.2 | 0.112756 | 0.066586 | 1.693385 | 0.090382 | -0.01775 | 0.243262 | EUR | Cellulitis and abs | dermatologic |
| 442 | 0.058207 | 0.034379 | 1.693075 | 9.04E-02 | -0.00918 | 0.12559 | EUR | Other aneurysm | circulatory system |
| 292 | 0.036371 | 0.021513 | 1.69068 | 0.090898 | -0.00579 | 0.078535 | EUR | Neurological disc | mental disorders |
| 433.1 | 0.050704 | 0.030006 | 1.689793 | 0.091068 | -0.00811 | 0.109514 | EUR | Occlusion and st | circulatory system |
| 364 | -0.0854 | 0.050568 | -1.68891 | 9.12E-02 | -0.18452 | 0.013706 | EUR | Corneal opacity a | sense organs |
| 742.8 | -0.10169 | 0.060239 | -1.68805 | 0.091402 | -0.21975 | 0.01638 | EUR | Articular cartilage | musculoskeletal |
| 31 | 0.155259 | 0.092018 | 1.687272 | 0.091551 | -0.02509 | 0.335611 | EUR | Diseases due to | infectious diseases |
| 480.1 | 0.059005 | 0.034971 | 1.687263 | 0.091553 | -0.00954 | 0.127547 | EUR | Bacterial pneumo | respiratory |
| 426.3 | 0.059587 | 0.03537 | 1.68467 | 0.092052 | -0.00974 | 0.12891 | EUR | Bundle branch bl | circulatory system |
| 272.14 | 0.421526 | 0.250273 | 1.684264 | 9.21E-02 | -0.069 | 0.912053 | EUR | Hyperchylomicro | endocrine/metabolic |
| 740.11 | 0.031772 | 0.018867 | 1.683973 | 0.092187 | -0.00521 | 0.068751 | EUR | Osteoarthrosis, lo | musculoskeletal |
| 733.2 | 0.174659 | 0.103731 | 1.683767 | 0.092227 | -0.02865 | 0.377968 | EUR | Cyst of bone | musculoskeletal |
| 805 | 0.072071 | 0.042951 | 1.678009 | 0.093345 | -0.01211 | 0.156253 | EUR | Fracture of vertel | injuries & poisonings |
| 480.3 | 0.140732 | 0.083884 | 1.677696 | 0.093406 | -0.02368 | 0.305141 | EUR | Pneumonia due t | respiratory |
| 760 | 0.028297 | 0.016873 | 1.677114 | 0.09352 | -0.00477 | 0.061367 | EUR | Back pain | symptoms |
| 613.1 | -0.1148 | 0.068495 | -1.676 | 0.093739 | -0.24905 | 0.01945 | EUR | Inflammatory dis | genitourinary |
| 117.1 | 0.432229 | 0.258049 | 1.674987 | 0.093937 | -0.07354 | 0.937995 | EUR | Histoplasmosis | infectious diseases |
| 411.8 | 0.072103 | 0.043088 | 1.673381 | 0.094252 | -0.01235 | 0.156555 | EUR | Other chronic isc | circulatory system |
| 532 | 0.037555 | 0.022471 | 1.671289 | 0.094665 | -0.00649 | 0.081596 | EUR | Dysphagia | digestive |
| 556 | 0.081007 | 0.048478 | 1.671014 | 0.094719 | -0.01401 | 0.176023 | EUR | Ulceration of the | digestive |
| 614.3 | -0.18748 | 0.112228 | -1.67054 | 0.094813 | -0.40744 | 0.032482 | EUR | Pelvic inflammato | genitourinary |
| 528.41 | 0.415335 | 0.24866 | 1.670292 | 0.094862 | -0.07203 | 0.9027 | EUR | Cyst of the saliva | digestive |
| 972.1 | 0.33748 | 0.202313 | 1.668104 | 0.095295 | -0.05905 | 0.734007 | EUR | Cardiac rhythm r | injuries & poisonings |
| 459.7 | 0.064516 | 0.03872 | 1.666239 | 0.095666 | -0.01137 | 0.140405 | EUR | Blood vessel repl | circulatory system |
| 714 | 0.058421 | 0.03513 | 1.662994 | 0.096314 | -0.01043 | 0.127274 | EUR | Rheumatoid arthi | musculoskeletal |
| 724.9 | 0.082767 | 0.049777 | 1.662781 | 0.096356 | -0.01479 | 0.180328 | EUR | Other unspecified | musculoskeletal |
| 705.1 | 0.165727 | 0.099772 | 1.661061 | 0.096701 | -0.02982 | 0.361275 | EUR | Dyshidrosis | dermatologic |
| 701.1 | -0.05821 | 0.035055 | -1.66065 | 0.096784 | -0.12692 | 0.010492 | EUR | Keratoderma, ac | dermatologic |
| 513.3 | 0.083219 | 0.050148 | 1.659462 | 0.097023 | -0.01507 | 0.181508 | EUR | Hypoventilation | respiratory |
| 599.4 | 0.046048 | 0.027764 | 1.658514 | 0.097214 | -0.00837 | 0.100465 | EUR | Urinary incontine | genitourinary |
| 443 | 0.047078 | 0.028409 | 1.65719 | 0.097481 | -0.0086 | 0.102758 | EUR | Peripheral vascu | circulatory system |
| 635 | -0.10395 | 0.062795 | -1.65542 | 0.09784 | -0.22703 | 0.019124 | EUR | Hemorrhage duri | pregnancy complications |

| phecode | Coef. | Std.Err. | z | p_value_z | [0.025 | 0.975] | GIA | phenotype | category |
|---|---|---|---|---|---|---|---|---|---|
| 300.11 | -0.03944 | 0.023841 | -1.65437 | 0.098052 | -0.08617 | 0.007286 | EUR | Generalized anxi | mental disorders |
| 656.2 | 0.742958 | 0.44925 | 1.653775 | 0.098173 | -0.13756 | 1.623472 | EUR | Respiratory cond | pregnancy complications |
| 70.9 | 0.079273 | 0.048052 | 1.649737 | 0.098997 | -0.01491 | 0.173452 | EUR | Hepatitis NOS | infectious diseases |
| 496.3 | 0.066443 | 0.040355 | 1.646467 | 0.099668 | -0.01265 | 0.145537 | EUR | Bronchiectasis | respiratory |
| 286.4 | 0.111855 | 0.067965 | 1.645784 | 0.099808 | -0.02135 | 0.245064 | EUR | Acquired coagula | hematopoietic |
| 226 | -0.22113 | 0.134388 | -1.64546 | 0.099876 | -0.48452 | 0.042266 | EUR | Benign neoplasm | neoplasms |
| 145 | 0.0979 | 0.059658 | 1.641026 | 0.100792 | -0.01903 | 0.214827 | EUR | Cancer of mouth | neoplasms |
| 458 | 0.039713 | 0.024218 | 1.639799 | 0.101047 | -0.00775 | 0.08718 | EUR | Hypotension | circulatory system |
| 250.23 | 0.085629 | 0.052375 | 1.634924 | 0.102065 | -0.01702 | 0.188283 | EUR | Type 2 diabetes \ | endocrine/metabolic |
| 242 | -0.07146 | 0.043722 | -1.63444 | 0.102166 | -0.15715 | 0.014232 | EUR | Thyrotoxicosis wi | endocrine/metabolic |
| 224 | -0.0993 | 0.06077 | -1.63403 | 0.102252 | -0.21841 | 0.019807 | EUR | Benign neoplasm | neoplasms |
| 426.9 | 0.058724 | 0.035955 | 1.633261 | 0.102414 | -0.01175 | 0.129194 | EUR | Cardiac pacemal | circulatory system |
| 272.12 | 0.07114 | 0.043585 | 1.632218 | 0.102634 | -0.01428 | 0.156565 | EUR | Hyperglyceridem | endocrine/metabolic |
| 327.4 | 0.031 | 0.018994 | 1.632112 | 0.102656 | -0.00623 | 0.068228 | EUR | Insomnia | neurological |
| 378.2 | 0.178862 | 0.109628 | 1.63154 | 0.102776 | -0.036 | 0.393729 | EUR | Nystagmus and c | sense organs |
| 369 | -0.04825 | 0.029632 | -1.62825 | 0.103471 | -0.10633 | 0.009829 | EUR | Infection of the e | sense organs |
| 305.21 | -0.2708 | 0.166792 | -1.62359 | 0.104463 | -0.59771 | 0.056104 | EUR | Anorexia nervosa | mental disorders |
| 290 | 0.063162 | 0.038993 | 1.619828 | 0.105269 | -0.01326 | 0.139588 | EUR | Delirium dementi | mental disorders |
| 136 | 0.038024 | 0.023495 | 1.618345 | 0.105588 | -0.00803 | 0.084074 | EUR | Other infectious a | infectious diseases |
| 727.8 | 0.331846 | 0.205088 | 1.618065 | 0.105649 | -0.07012 | 0.73381 | EUR | Plica syndrome | musculoskeletal |
| 618.6 | 0.293479 | 0.181537 | 1.616635 | 0.105957 | -0.06233 | 0.649286 | EUR | Vaginal enteroce | genitourinary |
| 703.1 | -0.06715 | 0.041565 | -1.61565 | 0.10617 | -0.14862 | 0.014312 | EUR | Ingrowing nail | dermatologic |
| 550.4 | 0.069336 | 0.04302 | 1.611719 | 0.107023 | -0.01498 | 0.153654 | EUR | Umbilical hernia | digestive |
| 250.15 | 0.346451 | 0.21542 | 1.608263 | 0.107778 | -0.07576 | 0.768666 | EUR | Diabetes type 1 v | endocrine/metabolic |
| 522.5 | 0.127786 | 0.079462 | 1.608134 | 0.107806 | -0.02796 | 0.28353 | EUR | Periapical absces | digestive |
| 736.5 | 0.493861 | 0.307319 | 1.606997 | 0.108055 | -0.10847 | 1.096196 | EUR | Acquired deformi | musculoskeletal |
| 352.2 | 0.112927 | 0.070298 | 1.606401 | 0.108186 | -0.02485 | 0.250708 | EUR | Facial nerve diso | neurological |
| 560.2 | 0.091035 | 0.056681 | 1.606107 | 0.108251 | -0.02006 | 0.202127 | EUR | Impaction of inte | digestive |
| 803.21 | 0.171135 | 0.106629 | 1.604961 | 0.108502 | -0.03785 | 0.380123 | EUR | Colles' fracture | injuries & poisonings |
| 536 | -0.04613 | 0.028746 | -1.60482 | 0.108534 | -0.10247 | 0.010209 | EUR | Disorders of func | digestive |
| 420.22 | -0.16154 | 0.100841 | -1.6019 | 0.109178 | -0.35918 | 0.036108 | EUR | Chronic pericardi | circulatory system |
| 619.3 | -0.06141 | 0.038351 | -1.60134 | 1.09E-01 | -0.13658 | 0.013754 | EUR | Noninflammatory | genitourinary |
| 793 | -0.10569 | 0.066034 | -1.60053 | 0.109481 | -0.23511 | 0.023735 | EUR | Nonspecific abnc | symptoms |
| 386.21 | 0.407943 | 0.255599 | 1.596031 | 0.110482 | -0.09302 | 0.908908 | EUR | Central origin ver | sense organs |
| 427.6 | 0.046717 | 0.029324 | 1.593129 | 0.111131 | -0.01076 | 0.104191 | EUR | Premature beats | circulatory system |
| 386.9 | -0.03235 | 0.020346 | -1.58999 | 0.111837 | -0.07223 | 0.007527 | EUR | Dizziness and gic | sense organs |
| 751.3 | 0.279238 | 0.175628 | 1.589938 | 0.111849 | -0.06499 | 0.623463 | EUR | Obstructive genit | congenital anomalies |

| phecode | Coef. | Std.Err. | z | p_value_z | [0.025 | 0.975] | GIA | phenotype | category |
|---|---|---|---|---|---|---|---|---|---|
| 370.1 | -0.20195 | 0.127224 | -1.58732 | 0.112441 | -0.4513 | 0.04741 | EUR | Corneal ulcer | sense organs |
| 195.3 | 0.086566 | 0.054552 | 1.586842 | 0.112549 | -0.02035 | 0.193487 | EUR | Malignant neopla | neoplasms |
| 709.4 | 0.330255 | 0.208153 | 1.5866 | 0.112603 | -0.07772 | 0.738228 | EUR | Polymyositis | dermatologic |
| 522 | 0.124822 | 0.078715 | 1.585743 | 0.112798 | -0.02946 | 0.279101 | EUR | Diseases of pulp | digestive |
| 737.1 | -0.11603 | 0.073175 | -1.58566 | 0.112816 | -0.25945 | 0.02739 | EUR | Kyphosis (acquir | musculoskeletal |
| 300.3 | -0.12522 | 0.079024 | -1.58461 | 0.113055 | -0.28011 | 0.029662 | EUR | Obsessive-comp | mental disorders |
| 180 | 0.088172 | 0.055672 | 1.583773 | 0.113245 | -0.02094 | 0.197288 | EUR | Cervical cancer a | neoplasms |
| 736.2 | -0.14884 | 0.094195 | -1.58014 | 0.114075 | -0.33346 | 0.035778 | EUR | Acquired deformi | musculoskeletal |
| 255.12 | 0.233193 | 0.147599 | 1.579913 | 0.114127 | -0.0561 | 0.522482 | EUR | Hyperaldosteroni | endocrine/metabolic |
| 420.21 | -0.151 | 0.095607 | -1.57933 | 0.11426 | -0.33838 | 0.036391 | EUR | Acute pericarditis | circulatory system |
| 361.2 | 0.299991 | 0.190333 | 1.576141 | 0.114993 | -0.07305 | 0.673037 | EUR | Retinoschisis and | sense organs |
| 841 | -0.04368 | 0.027725 | -1.57546 | 0.115149 | -0.09802 | 0.01066 | EUR | Sprains and strai | injuries & poisonings |
| 132.1 | 0.366391 | 0.232654 | 1.574835 | 0.115295 | -0.0896 | 0.822384 | EUR | Pediculosis and p | infectious diseases |
| 1004 | 0.078205 | 0.049706 | 1.57336 | 0.115635 | -0.01922 | 0.175626 | EUR | Other signs and symptoms involvir | |
| 262 | -0.07025 | 0.044675 | -1.57247 | 0.115841 | -0.15781 | 0.017311 | EUR | Mineral deficienc | endocrine/metabolic |
| 480.5 | 0.103671 | 0.066102 | 1.568344 | 0.116801 | -0.02589 | 0.233229 | EUR | Bronchopneumoı | respiratory |
| 270.12 | -0.77918 | 0.498959 | -1.56161 | 0.11838 | -1.75712 | 0.198763 | EUR | Phenylketonuria | endocrine/metabolic |
| 556.1 | 0.081716 | 0.052332 | 1.561508 | 0.118404 | -0.02085 | 0.184284 | EUR | Ulceration of inte | digestive |
| 614.51 | -0.08044 | 0.051541 | -1.56073 | 0.118588 | -0.18146 | 0.020577 | EUR | Cervicitis and en | genitourinary |
| 379.3 | -0.17389 | 0.111497 | -1.55963 | 0.118848 | -0.39242 | 0.044636 | EUR | Aphakia and othe | sense organs |
| 276.1 | 0.031127 | 0.020015 | 1.555223 | 0.119893 | -0.0081 | 0.070355 | EUR | Electrolyte imbala | endocrine/metabolic |
| 241.1 | -0.04335 | 0.0279 | -1.55388 | 0.120212 | -0.09804 | 0.01133 | EUR | Nontoxic uninodu | endocrine/metabolic |
| 210 | -0.13687 | 0.08817 | -1.55235 | 0.120578 | -0.30968 | 0.035939 | EUR | Benign neoplasm | neoplasms |
| 594 | 0.039173 | 0.025244 | 1.551787 | 0.120713 | -0.0103 | 0.088649 | EUR | Urinary calculus | genitourinary |
| 599.7 | 0.270503 | 0.174515 | 1.550027 | 0.121135 | -0.07154 | 0.612545 | EUR | Urethral discharg | genitourinary |
| 609 | -0.14148 | 0.091381 | -1.54824 | 0.121564 | -0.32058 | 0.037624 | EUR | Male infertility an | genitourinary |
| 979 | 0.040581 | 0.026229 | 1.54716 | 0.121825 | -0.01083 | 0.09199 | EUR | Adverse drug eve | injuries & poisonings |
| 420.2 | 0.060647 | 0.039272 | 1.544267 | 0.122524 | -0.01633 | 0.137618 | EUR | Pericarditis | circulatory system |
| 654.1 | 0.137261 | 0.089032 | 1.541709 | 0.123144 | -0.03724 | 0.31176 | EUR | Abnormality of or | pregnancy complications |
| 501 | 0.068162 | 0.044222 | 1.541362 | 0.123229 | -0.01851 | 0.154836 | EUR | Pneumonitis due | respiratory |
| 244.4 | -0.03089 | 0.020092 | -1.53735 | 0.124208 | -0.07027 | 0.008491 | EUR | Hypothyroidism N | endocrine/metabolic |
| 732.1 | 0.247085 | 0.161298 | 1.531856 | 0.125558 | -0.06905 | 0.563224 | EUR | Juvenile osteoch | musculoskeletal |
| 81.12 | 0.282463 | 0.184718 | 1.529163 | 0.126224 | -0.07958 | 0.644503 | EUR | Chronic graft-ver | infectious diseases |
| 732 | 0.208859 | 0.136805 | 1.526687 | 0.126839 | -0.05927 | 0.476992 | EUR | Osteochondropat | musculoskeletal |
| 695.1 | 0.266988 | 0.175324 | 1.522826 | 0.127802 | -0.07664 | 0.610617 | EUR | Toxic erythema | dermatologic |
| 976 | 0.460704 | 0.303029 | 1.520329 | 0.128428 | -0.13322 | 1.05463 | EUR | Poisoning by age | injuries & poisonings |
| 724.8 | 0.074426 | 0.04901 | 1.518589 | 0.128866 | -0.02163 | 0.170483 | EUR | Other symptoms | musculoskeletal |

| phecode | Coef. | Std.Err. | z | p_value_z | [0.025 | 0.975] | GIA | phenotype | category |
|---|---|---|---|---|---|---|---|---|---|
| 741.2 | -0.06129 | 0.040396 | -1.51728 | 0.129196 | -0.14047 | 0.017883 | EUR | Stiffness of joint | musculoskeletal |
| 425.1 | 0.053645 | 0.035425 | 1.514319 | 0.129945 | -0.01579 | 0.123077 | EUR | Primary/intrinsic c | circulatory system |
| 619.4 | -0.04629 | 0.030591 | -1.51323 | 0.130221 | -0.10625 | 0.013666 | EUR | Noninflammatory | genitourinary |
| 282.9 | -0.12007 | 0.079393 | -1.51238 | 0.130437 | -0.27568 | 0.035535 | EUR | Other hereditary | hematopoietic |
| 579.8 | 0.049582 | 0.032787 | 1.512243 | 0.130472 | -0.01468 | 0.113843 | EUR | Nonspecific abnc | digestive |
| 479 | 0.029749 | 0.019686 | 1.511178 | 0.130743 | -0.00883 | 0.068332 | EUR | Other upper resp | respiratory |
| 270.1 | -0.13364 | 0.088673 | -1.50708 | 0.13179 | -0.30743 | 0.040158 | EUR | Disturbances of a | endocrine/metabolic |
| 276.8 | 0.166633 | 0.11072 | 1.504989 | 0.132327 | -0.05037 | 0.383641 | EUR | Polydipsia | endocrine/metabolic |
| 133 | 0.281856 | 0.187488 | 1.50333 | 0.132754 | -0.08561 | 0.649326 | EUR | Arthropod-borne | infectious diseases |
| 184.1 | -0.09437 | 0.062824 | -1.50213 | 0.133064 | -0.2175 | 0.028763 | EUR | Malignant neopla | neoplasms |
| 285.21 | 0.061089 | 0.040726 | 1.500007 | 0.133613 | -0.01873 | 0.140911 | EUR | Anemia in chroni | hematopoietic |
| 180.3 | 0.100122 | 0.066773 | 1.499443 | 0.133759 | -0.03075 | 0.230995 | EUR | Cervical intraepit | neoplasms |
| 272.11 | -0.02897 | 0.019323 | -1.49925 | 0.133808 | -0.06684 | 0.008902 | EUR | Hypercholesterol | endocrine/metabolic |
| 343 | 0.282198 | 0.188769 | 1.49494 | 0.13493 | -0.08778 | 0.652179 | EUR | Infantile cerebral | neurological |
| 420.1 | 0.1792 | 0.119977 | 1.493618 | 0.135276 | -0.05595 | 0.414352 | EUR | Myocarditis | circulatory system |
| 593 | -0.03675 | 0.024628 | -1.49234 | 0.13561 | -0.08502 | 0.011517 | EUR | Hematuria | genitourinary |
| 536.7 | -0.11779 | 0.078974 | -1.49157 | 0.135813 | -0.27258 | 0.036991 | EUR | Complications of | digestive |
| 636 | -0.08651 | 0.058168 | -1.48718 | 0.136966 | -0.20051 | 0.0275 | EUR | Early or threaten | pregnancy complications |
| 276.42 | -0.10411 | 0.070232 | -1.48236 | 0.138244 | -0.24176 | 0.033543 | EUR | Alkalosis | endocrine/metabolic |
| 71.1 | 0.121099 | 0.081747 | 1.481389 | 1.39E-01 | -0.03912 | 0.281321 | EUR | HIV infection, syr | infectious diseases |
| 476 | -0.02781 | 0.018777 | -1.48121 | 0.13855 | -0.06462 | 0.00899 | EUR | Allergic rhinitis | respiratory |
| 276 | 0.02728 | 0.018431 | 1.480092 | 0.138849 | -0.00884 | 0.063404 | EUR | Disorders of fluid | endocrine/metabolic |
| 368.4 | -0.09563 | 0.064614 | -1.48009 | 0.138849 | -0.22228 | 0.031006 | EUR | Visual field defec | sense organs |
| 277.1 | 0.438341 | 0.296518 | 1.478295 | 0.139329 | -0.14282 | 1.019505 | EUR | Disorders of porp | endocrine/metabolic |
| 288.11 | 0.046495 | 0.031458 | 1.477993 | 0.13941 | -0.01516 | 0.108153 | EUR | Neutropenia | hematopoietic |
| 277.6 | 0.327746 | 0.221838 | 1.477407 | 0.139567 | -0.10705 | 0.762541 | EUR | Other deficiencie | endocrine/metabolic |
| 590 | 0.064973 | 0.044017 | 1.476088 | 0.13992 | -0.0213 | 0.151245 | EUR | Pyelonephritis | genitourinary |
| 580.14 | 0.302235 | 0.204934 | 1.474789 | 0.140269 | -0.09943 | 0.703898 | EUR | Chronic glomerul | genitourinary |
| 378.5 | -0.16097 | 0.109427 | -1.47104 | 0.141281 | -0.37545 | 0.053502 | EUR | Paralytic strabisn | sense organs |
| 367.4 | -0.05251 | 0.035734 | -1.4694 | 0.141724 | -0.12255 | 0.01753 | EUR | Presbyopia | sense organs |
| 290.12 | 0.219154 | 0.149418 | 1.466718 | 0.142453 | -0.0737 | 0.512008 | EUR | Dementia with ce | mental disorders |
| 704 | -0.03452 | 0.023553 | -1.46557 | 0.142765 | -0.08068 | 0.011644 | EUR | Diseases of hair | dermatologic |
| 362.2 | -0.0473 | 0.032297 | -1.46465 | 0.143017 | -0.11061 | 0.015997 | EUR | Degeneration of | sense organs |
| 610.1 | -0.05964 | 0.040722 | -1.46465 | 0.143017 | -0.13946 | 0.02017 | EUR | Cystic mastopath | genitourinary |
| 173 | -0.03116 | 0.021289 | -1.46343 | 0.14335 | -0.07288 | 0.010571 | EUR | Neoplasm of unc | neoplasms |
| 727.6 | 0.080068 | 0.054819 | 1.460575 | 0.144132 | -0.02738 | 0.187511 | EUR | Rupture of tendo | musculoskeletal |
| 155 | 0.08094 | 0.055514 | 1.458015 | 0.144836 | -0.02786 | 0.189744 | EUR | Cancer of liver ar | neoplasms |

| phecode | Coef. | Std.Err. | z | p_value_z | [0.025 | 0.975] | GIA | phenotype | category |
|---|---|---|---|---|---|---|---|---|---|
| 455 | -0.02684 | 0.018439 | -1.4557 | 0.145477 | -0.06298 | 0.009298 | EUR | Hemorrhoids | circulatory system |
| 145.2 | 0.110861 | 0.076173 | 1.455385 | 0.145563 | -0.03844 | 0.260158 | EUR | Cancer of tongue | neoplasms |
| 198.2 | 0.066542 | 0.045753 | 1.454397 | 1.46E-01 | -0.02313 | 0.156216 | EUR | Secondary malig | neoplasms |
| 371.2 | -0.06256 | 0.043047 | -1.45331 | 0.146137 | -0.14693 | 0.02181 | EUR | Conjunctivitis, no | sense organs |
| 325 | -0.3912 | 0.269272 | -1.45281 | 0.146276 | -0.91896 | 0.136561 | EUR | Phlebitis and thro | neurological |
| 261.3 | 0.475833 | 0.327663 | 1.452204 | 0.146445 | -0.16637 | 1.118041 | EUR | Vitamin C deficie | endocrine/metabolic |
| 275.11 | 0.216679 | 0.149667 | 1.447738 | 0.14769 | -0.07666 | 0.510022 | EUR | Hereditary hemo | hematopoietic |
| 302.1 | 0.079445 | 0.055094 | 1.441985 | 0.149307 | -0.02854 | 0.187428 | EUR | Decreased libido | mental disorders |
| 401.3 | 0.04382 | 0.03039 | 1.441912 | 0.149327 | -0.01574 | 0.103383 | EUR | Other hypertensi | circulatory system |
| 743 | -0.02876 | 0.019949 | -1.44159 | 0.149419 | -0.06786 | 0.010341 | EUR | Osteoporosis, os | musculoskeletal |
| 772 | -0.05251 | 0.03657 | -1.43595 | 0.151016 | -0.12419 | 0.019163 | EUR | Symptoms of the | symptoms |
| 720.1 | 0.040879 | 0.028509 | 1.433878 | 0.151607 | -0.015 | 0.096756 | EUR | Spinal stenosis o | musculoskeletal |
| 394.2 | -0.06934 | 0.048378 | -1.43327 | 0.151782 | -0.16416 | 0.02548 | EUR | Mitral valve disea | circulatory system |
| 601 | -0.06579 | 0.045925 | -1.43261 | 0.151968 | -0.15581 | 0.024219 | EUR | Inflammatory dis | genitourinary |
| 411.9 | -0.08759 | 0.061172 | -1.43181 | 0.152197 | -0.20748 | 0.032308 | EUR | Other acute and | circulatory system |
| 529.1 | 0.159331 | 0.111832 | 1.424734 | 0.154234 | -0.05986 | 0.378519 | EUR | Glossitis | digestive |
| 743.22 | 0.103022 | 0.072436 | 1.42225 | 0.154954 | -0.03895 | 0.244994 | EUR | Pathologic fractu | musculoskeletal |
| 840 | -0.02946 | 0.020764 | -1.4189 | 0.155929 | -0.07016 | 0.011235 | EUR | Sprains and strai | injuries & poisonings |
| 480.13 | 0.196238 | 0.138619 | 1.415666 | 0.156873 | -0.07545 | 0.467925 | EUR | MRSA pneumoni | respiratory |
| 530.13 | 0.077127 | 0.054482 | 1.415647 | 0.156879 | -0.02966 | 0.18391 | EUR | Barrett's esophag | digestive |
| 527.1 | 0.244228 | 0.172696 | 1.414211 | 0.1573 | -0.09425 | 0.582706 | EUR | Hypertrophy of sa | digestive |
| 735.22 | 0.613241 | 0.433952 | 1.413155 | 0.15761 | -0.23729 | 1.463772 | EUR | Claw toe (acquire | musculoskeletal |
| 1014 | 0.817067 | 0.579813 | 1.409191 | 0.158779 | -0.31935 | 1.95348 | EUR | Effects of heat, cold and air pressu | |
| 444.5 | -0.46476 | 0.330026 | -1.40825 | 0.159056 | -1.1116 | 0.182079 | EUR | Atheroembolism | circulatory system |
| 700 | 0.069059 | 0.049044 | 1.408099 | 0.159102 | -0.02707 | 0.165183 | EUR | Corns and callos | dermatologic |
| 652 | -0.27174 | 0.193489 | -1.40442 | 0.160193 | -0.65097 | 0.107491 | EUR | Malposition and r | pregnancy complications |
| 599 | -0.02414 | 0.017206 | -1.40308 | 0.160594 | -0.05786 | 0.009582 | EUR | Other symptoms/ | genitourinary |
| 941 | 0.074057 | 0.052782 | 1.403062 | 0.160598 | -0.02939 | 0.177508 | EUR | Adverse reaction | injuries & poisonings |
| 359 | 0.073856 | 0.05267 | 1.402236 | 0.160845 | -0.02938 | 0.177088 | EUR | Muscular dystrop | neurological |
| 706.3 | 0.21973 | 0.15675 | 1.401788 | 0.160979 | -0.08749 | 0.526953 | EUR | Seborrhea | dermatologic |
| 526.9 | 0.162077 | 0.115707 | 1.400753 | 0.161288 | -0.0647 | 0.38886 | EUR | Jaw disease NOS | digestive |
| 433.5 | 0.10203 | 0.072883 | 1.399909 | 0.161541 | -0.04082 | 0.244878 | EUR | Cerebral aneurys | circulatory system |
| 200.1 | 0.10014 | 0.071553 | 1.399526 | 0.161655 | -0.0401 | 0.240381 | EUR | Polycythemia ver | neoplasms |
| 475.9 | -0.03852 | 0.027543 | -1.39855 | 0.161947 | -0.0925 | 0.015463 | EUR | Postnasal drip | respiratory |
| 352 | 0.073233 | 0.052458 | 1.396024 | 0.162707 | -0.02958 | 0.176048 | EUR | Disorders of othe | neurological |
| 454.1 | 0.046663 | 0.033436 | 1.395595 | 0.162837 | -0.01887 | 0.112197 | EUR | Varicose veins of | circulatory system |
| 441.2 | 0.221039 | 0.15911 | 1.389218 | 1.65E-01 | -0.09081 | 0.532889 | EUR | Chronic vascular | circulatory system |

| phecode | Coef. | Std.Err. | z | p_value_z | [0.025 | 0.975] | GIA | phenotype | category |
|---|---|---|---|---|---|---|---|---|---|
| 260.3 | 0.076722 | 0.055327 | 1.386712 | 0.16553 | -0.03172 | 0.185161 | EUR | Adult failure to th | endocrine/metabolic |
| 505 | -0.09011 | 0.064992 | -1.38654 | 0.165581 | -0.2175 | 0.037268 | EUR | Other pulmonary | respiratory |
| 636.2 | 0.260843 | 0.188389 | 1.384596 | 0.166176 | -0.10839 | 0.630078 | EUR | Early onset of de | pregnancy complications |
| 274 | 0.044225 | 0.031978 | 1.38299 | 0.166668 | -0.01845 | 0.106902 | EUR | Gout and other c | endocrine/metabolic |
| 270.32 | -0.08319 | 0.060236 | -1.38112 | 0.167243 | -0.20125 | 0.034868 | EUR | Paraproteinemia | endocrine/metabolic |
| 331 | -0.07792 | 0.056449 | -1.38041 | 0.167461 | -0.18856 | 0.032715 | EUR | Other cerebral de | neurological |
| 459 | 0.029801 | 0.021595 | 1.380013 | 0.167583 | -0.01252 | 0.072127 | EUR | Other disorders c | circulatory system |
| 743.13 | -0.06646 | 0.048176 | -1.37944 | 0.167758 | -0.16088 | 0.027967 | EUR | Other specified o | musculoskeletal |
| 282.5 | -0.57802 | 0.419195 | -1.37887 | 0.167935 | -1.39962 | 0.243591 | EUR | Sickle cell anemi | hematopoietic |
| 289.1 | 0.399909 | 0.290119 | 1.378434 | 0.168069 | -0.16871 | 0.968532 | EUR | Myelofibrosis | hematopoietic |
| 90.2 | -0.2158 | 0.156573 | -1.37824 | 0.168129 | -0.52267 | 0.091082 | EUR | Gonococcal infec | infectious diseases |
| 427.41 | 0.122521 | 0.089188 | 1.373727 | 0.169527 | -0.05229 | 0.297327 | EUR | Ventricular fibrilla | circulatory system |
| 174.11 | 0.045656 | 0.033419 | 1.366154 | 0.171891 | -0.01984 | 0.111156 | EUR | Malignant neopla | neoplasms |
| 362.7 | 0.245309 | 0.179839 | 1.364047 | 0.172553 | -0.10717 | 0.597787 | EUR | Hereditary retinal | sense organs |
| 559 | -0.10612 | 0.077851 | -1.3631 | 0.172852 | -0.2587 | 0.046467 | EUR | Ileostomy status | digestive |
| 710.1 | 0.084007 | 0.061705 | 1.361442 | 0.173374 | -0.03693 | 0.204946 | EUR | Osteomyelitis | musculoskeletal |
| 793.2 | -0.0494 | 0.036299 | -1.36098 | 0.173519 | -0.12055 | 0.021742 | EUR | Nonspecific abnc | symptoms |
| 695.2 | 0.191553 | 0.140781 | 1.360646 | 0.173626 | -0.08437 | 0.467478 | EUR | Bullous dermatos | dermatologic |
| 379.4 | 0.121628 | 0.08941 | 1.360343 | 1.74E-01 | -0.05361 | 0.296868 | EUR | Anomalies of pup | sense organs |
| 214 | 0.046264 | 0.034056 | 1.35847 | 0.174315 | -0.02048 | 0.113014 | EUR | Lipoma | neoplasms |
| 381.9 | -0.1038 | 0.07642 | -1.35831 | 1.74E-01 | -0.25358 | 0.045979 | EUR | Otorrhea | sense organs |
| 750.13 | -0.11147 | 0.082076 | -1.35809 | 0.174434 | -0.27233 | 0.049399 | EUR | Congenital anom | congenital anomalies |
| 350.2 | -0.03584 | 0.02639 | -1.3579 | 0.174497 | -0.08756 | 0.015889 | EUR | Abnormality of ga | neurological |
| 442.1 | 0.055843 | 0.04113 | 1.357722 | 0.174552 | -0.02477 | 0.136456 | EUR | Aortic aneurysm | circulatory system |
| 470 | -0.04278 | 0.0316 | -1.35387 | 0.175779 | -0.10472 | 0.019153 | EUR | Septal Deviations | respiratory |
| 472 | -0.03875 | 0.028683 | -1.35115 | 0.176647 | -0.09497 | 0.017462 | EUR | Chronic pharyngi | respiratory |
| 790.9 | 0.166191 | 0.123167 | 1.349314 | 0.177236 | -0.07521 | 0.407594 | EUR | Abnormal arterial | symptoms |
| 735.1 | -0.07772 | 0.057609 | -1.34916 | 0.177285 | -0.19063 | 0.035187 | EUR | Flat foot | musculoskeletal |
| 481 | 0.055125 | 0.040874 | 1.348651 | 0.177449 | -0.02499 | 0.135238 | EUR | Influenza | respiratory |
| 312 | 0.112227 | 0.083266 | 1.347818 | 0.177717 | -0.05097 | 0.275424 | EUR | Conduct disorder | mental disorders |
| 241 | -0.03364 | 0.024962 | -1.34763 | 0.177776 | -0.08256 | 0.015285 | EUR | Nontoxic nodular | endocrine/metabolic |
| 939.1 | 0.161033 | 0.119611 | 1.346307 | 0.178204 | -0.0734 | 0.395466 | EUR | Contact and aller | dermatologic |
| 458.9 | 0.037462 | 0.027861 | 1.344617 | 0.178749 | -0.01714 | 0.092069 | EUR | Hypotension NOS | circulatory system |
| 568 | 0.044718 | 0.033269 | 1.344146 | 0.178901 | -0.02049 | 0.109924 | EUR | Other disorders c | digestive |
| 90 | 0.074429 | 0.055412 | 1.343202 | 0.179207 | -0.03418 | 0.183034 | EUR | Sexually transmit | infectious diseases |
| 520.2 | 0.535834 | 0.400336 | 1.338461 | 0.180746 | -0.24881 | 1.320478 | EUR | Disturbances in t | digestive |
| 803.1 | 0.080671 | 0.060404 | 1.335521 | 0.181706 | -0.03772 | 0.199061 | EUR | Fracture of hume | injuries & poisonings |

| phecode | Coef. | Std.Err. | z | p_value_z | [0.025 | 0.975] | GIA | phenotype | category |
|---|---|---|---|---|---|---|---|---|---|
| 433.2 | 0.045441 | 0.034082 | 1.333259 | 0.182447 | -0.02136 | 0.112241 | EUR | Occlusion of cere | circulatory system |
| 279.2 | -0.14739 | 0.11061 | -1.33255 | 0.182678 | -0.36419 | 0.069398 | EUR | Autoimmune dise | endocrine/metabolic |
| 473 | 0.035262 | 0.026477 | 1.331798 | 0.182926 | -0.01663 | 0.087157 | EUR | Diseases of the l | respiratory |
| 451 | 0.073755 | 0.055511 | 1.32866 | 0.18396 | -0.03504 | 0.182553 | EUR | Phlebitis and thro | circulatory system |
| 753.1 | 0.35721 | 0.268853 | 1.328642 | 0.183966 | -0.16973 | 0.884152 | EUR | Congenital catara | congenital anomalies |
| 224.1 | -0.086 | 0.064807 | -1.32707 | 0.184486 | -0.21302 | 0.041016 | EUR | Benign neoplasm | neoplasms |
| 366 | -0.02863 | 0.021582 | -1.32647 | 0.184683 | -0.07093 | 0.013672 | EUR | Cataract | sense organs |
| 1002 | -0.03524 | 0.026636 | -1.32286 | 0.185883 | -0.08744 | 0.01697 | EUR | Symptoms concerning nutrition, m | |
| 425 | 0.044557 | 0.033745 | 1.320397 | 0.186702 | -0.02158 | 0.110696 | EUR | Cardiomyopathy | circulatory system |
| 281.9 | 0.060225 | 0.045877 | 1.312742 | 0.18927 | -0.02969 | 0.150142 | EUR | Deficiency anemi | hematopoietic |
| 465 | -0.02344 | 0.017868 | -1.31199 | 0.189522 | -0.05846 | 0.011578 | EUR | Acute upper resp | respiratory |
| 964 | 0.153673 | 0.117134 | 1.311937 | 0.189541 | -0.07591 | 0.383251 | EUR | Poisoning by age | injuries & poisonings |
| 261 | -0.02426 | 0.018506 | -1.31078 | 0.189931 | -0.06053 | 0.012014 | EUR | Vitamin deficienc | endocrine/metabolic |
| 395.4 | 0.111048 | 0.08498 | 1.306753 | 0.191297 | -0.05551 | 0.277606 | EUR | Nonrheumatic pu | circulatory system |
| 577.1 | 0.073032 | 0.05593 | 1.305788 | 0.191625 | -0.03659 | 0.182652 | EUR | Acute pancreatiti | digestive |
| 588.1 | 0.317272 | 0.243054 | 1.305357 | 0.191771 | -0.1591 | 0.793648 | EUR | Renal osteodystr | genitourinary |
| 528.11 | -0.09307 | 0.071366 | -1.30408 | 0.192208 | -0.23294 | 0.046808 | EUR | Stomatitis and m | digestive |
| 454 | 0.039051 | 0.029951 | 1.303829 | 0.192292 | -0.01965 | 0.097755 | EUR | Varicose veins | circulatory system |
| 244 | -0.02567 | 0.01971 | -1.30228 | 0.192822 | -0.0643 | 0.012963 | EUR | Hypothyroidism | endocrine/metabolic |
| 79.1 | -0.03839 | 0.029503 | -1.30108 | 0.19323 | -0.09621 | 0.019439 | EUR | Varicella infectior | infectious diseases |
| 911 | -0.10762 | 0.082824 | -1.29932 | 0.193833 | -0.26995 | 0.054717 | EUR | Blister | injuries & poisonings |
| 371.3 | -0.03576 | 0.02754 | -1.29841 | 0.194148 | -0.08974 | 0.018219 | EUR | Inflammation of e | sense organs |
| 41.2 | 0.059513 | 0.045865 | 1.297582 | 0.194431 | -0.03038 | 0.149407 | EUR | Streptococcus in | infectious diseases |
| 346.3 | -0.2254 | 0.174091 | -1.29475 | 0.195405 | -0.56662 | 0.115807 | EUR | Nonspecific abnc | neurological |
| 627.3 | -0.04527 | 0.035043 | -1.29171 | 0.196458 | -0.11395 | 0.023418 | EUR | Postmenopausal | genitourinary |
| 433.21 | 0.044601 | 0.034557 | 1.290652 | 0.196824 | -0.02313 | 0.112332 | EUR | Cerebral artery o | circulatory system |
| 586.4 | -0.04544 | 0.035257 | -1.28874 | 0.197487 | -0.11454 | 0.023665 | EUR | Stricture/obstruct | genitourinary |
| 289.3 | -0.05618 | 0.043623 | -1.28796 | 0.197759 | -0.14168 | 0.029315 | EUR | Personal history | hematopoietic |
| 573.9 | 0.035029 | 0.027256 | 1.285214 | 0.198717 | -0.01839 | 0.08845 | EUR | Abnormal serum | digestive |
| 858 | 0.06486 | 0.050544 | 1.283233 | 0.199411 | -0.0342 | 0.163924 | EUR | Complication of i | injuries & poisonings |
| 303.1 | 0.220202 | 0.171848 | 1.281375 | 0.200062 | -0.11661 | 0.557019 | EUR | Dissociative diso | mental disorders |
| 250.1 | 0.07393 | 0.05793 | 1.276187 | 0.20189 | -0.03961 | 0.187471 | EUR | Type 1 diabetes | endocrine/metabolic |
| 599.6 | 0.10344 | 0.081262 | 1.272925 | 0.203045 | -0.05583 | 0.26271 | EUR | Oliguria and anu | genitourinary |
| 519 | 0.0305 | 0.023984 | 1.271689 | 0.203484 | -0.01651 | 0.077507 | EUR | Other diseases o | respiratory |
| 110.11 | -0.0368 | 0.028935 | -1.27164 | 0.203502 | -0.09351 | 0.019917 | EUR | Dermatophytosis | infectious diseases |
| 521.4 | -1.17886 | 0.928893 | -1.2691 | 0.204406 | -2.99945 | 0.64174 | EUR | Tooth complicatio | digestive |
| 264.2 | 0.177507 | 0.13998 | 1.268084 | 0.204768 | -0.09685 | 0.451863 | EUR | Failure to thrive ( | endocrine/metabolic |

| phecode | Coef. | Std.Err. | z | p_value_z | [0.025 | 0.975] | GIA | phenotype | category |
|---|---|---|---|---|---|---|---|---|---|
| 420.3 | -0.08443 | 0.06664 | -1.26691 | 0.205186 | -0.21504 | 0.046185 | EUR | Endocarditis | circulatory system |
| 550.1 | -0.0452 | 0.035688 | -1.26642 | 2.05E-01 | -0.11514 | 0.024752 | EUR | Inguinal hernia | digestive |
| 586.11 | 0.252845 | 0.199895 | 1.26489 | 0.205911 | -0.13894 | 0.644631 | EUR | Small kidney | genitourinary |
| 134 | 0.173691 | 0.137408 | 1.264049 | 0.206213 | -0.09562 | 0.443006 | EUR | Helminthiases | infectious diseases |
| 199.4 | 0.227401 | 0.180005 | 1.263299 | 0.206482 | -0.1254 | 0.580205 | EUR | Neurofibromatosi | neoplasms |
| 987 | -0.32711 | 0.259013 | -1.26293 | 0.206614 | -0.83477 | 0.180541 | EUR | Toxic effect of oth | injuries & poisonings |
| 110.13 | -0.05542 | 0.043918 | -1.26191 | 0.206982 | -0.1415 | 0.030657 | EUR | Dermatophytosis | infectious diseases |
| 284 | 0.046556 | 0.036917 | 1.261094 | 0.207275 | -0.0258 | 0.118913 | EUR | Aplastic anemia | hematopoietic |
| 572 | 0.043365 | 0.03441 | 1.260255 | 0.207577 | -0.02408 | 0.110807 | EUR | Ascites (non mali | digestive |
| 418.1 | 0.054006 | 0.042918 | 1.258333 | 0.208271 | -0.03011 | 0.138124 | EUR | Precordial pain | circulatory system |
| 755 | 0.082195 | 0.065474 | 1.255387 | 0.209338 | -0.04613 | 0.210522 | EUR | Congenital anom | congenital anomalies |
| 204.3 | 0.566523 | 0.45148 | 1.254813 | 2.10E-01 | -0.31836 | 1.451409 | EUR | Monocytic leuker | neoplasms |
| 365.1 | -0.0596 | 0.04752 | -1.25431 | 0.20973 | -0.15274 | 0.033532 | EUR | Open-angle glau | sense organs |
| 250.12 | 0.142437 | 0.113565 | 1.254232 | 0.209758 | -0.08015 | 0.36502 | EUR | Type 1 diabetes v | endocrine/metabolic |
| 627.5 | -0.09642 | 0.077022 | -1.25191 | 0.210603 | -0.24739 | 0.054536 | EUR | Premature meno | genitourinary |
| 580.3 | 0.05144 | 0.041144 | 1.250246 | 0.21121 | -0.0292 | 0.132082 | EUR | Nephritis and nep | genitourinary |
| 359.2 | 0.067393 | 0.054099 | 1.245733 | 0.212862 | -0.03864 | 0.173425 | EUR | Myopathy | neurological |
| 520.1 | 0.53171 | 0.427585 | 1.243519 | 0.213677 | -0.30634 | 1.369762 | EUR | Hereditary disturl | digestive |
| 626.2 | 0.082555 | 0.066493 | 1.241571 | 0.214395 | -0.04777 | 0.212879 | EUR | Dysmenorrhea | genitourinary |
| 613.9 | -0.10929 | 0.088128 | -1.24017 | 0.214912 | -0.28202 | 0.063434 | EUR | Breast disorder N | genitourinary |
| 346.1 | 0.052418 | 0.042396 | 1.23639 | 0.216314 | -0.03068 | 0.135513 | EUR | Nonspecific abnc | neurological |
| 368.2 | 0.061022 | 0.049451 | 1.233974 | 0.217212 | -0.0359 | 0.157944 | EUR | Diplopia and disc | sense organs |
| 275.2 | 0.267489 | 0.216842 | 1.233562 | 0.217366 | -0.15751 | 0.692492 | EUR | Disorders of copp | endocrine/metabolic |
| 585.32 | 0.057402 | 0.046653 | 1.230396 | 0.218549 | -0.03404 | 0.148841 | EUR | End stage renal c | genitourinary |
| 574.2 | 0.076583 | 0.062286 | 1.229539 | 0.21887 | -0.0455 | 0.19866 | EUR | Calculus of bile d | digestive |
| 622.2 | 0.096216 | 0.078259 | 1.229455 | 0.218901 | -0.05717 | 0.2496 | EUR | Mucous polyp of | genitourinary |
| 300.12 | 0.051334 | 0.04176 | 1.229273 | 0.218969 | -0.03051 | 0.133181 | EUR | Agorophobia, soc | mental disorders |
| 530.9 | -0.05538 | 0.045101 | -1.22789 | 0.219487 | -0.14378 | 0.033017 | EUR | Heartburn | digestive |
| 509.2 | -0.09067 | 0.074022 | -1.22494 | 0.220598 | -0.23575 | 0.054408 | EUR | Respiratory insuf | respiratory |
| 255.11 | -0.14423 | 0.117818 | -1.2242 | 0.220876 | -0.37515 | 0.086686 | EUR | Cushing's syndrc | endocrine/metabolic |
| 592.3 | 0.398195 | 0.325993 | 1.221483 | 0.221903 | -0.24074 | 1.037129 | EUR | Urethral stricture | genitourinary |
| 603.1 | 0.087965 | 0.072143 | 1.219307 | 0.222728 | -0.05343 | 0.229363 | EUR | Hydrocele | genitourinary |
| 634 | -0.064 | 0.052502 | -1.21907 | 0.222819 | -0.16691 | 0.038899 | EUR | Miscarriage; stillb | pregnancy complications |
| 802 | -0.11078 | 0.090898 | -1.21878 | 0.222929 | -0.28894 | 0.067372 | EUR | Fracture of pelvis | injuries & poisonings |
| 759 | 0.080988 | 0.067 | 1.208777 | 0.226749 | -0.05033 | 0.212305 | EUR | Other and unspe | congenital anomalies |
| 292.4 | 0.037103 | 0.030848 | 1.202749 | 0.229073 | -0.02336 | 0.097564 | EUR | Altered mental st | mental disorders |
| 295 | 0.05783 | 0.048131 | 1.201529 | 0.229546 | -0.0365 | 0.152165 | EUR | Schizophrenia ar | mental disorders |

| phecode | Coef. | Std.Err. | z | p_value_z | [0.025 | 0.975] | GIA | phenotype | category |
|---|---|---|---|---|---|---|---|---|---|
| 634.3 | 0.201489 | 0.167736 | 1.201225 | 0.229664 | -0.12727 | 0.530246 | EUR | Ectopic pregnanc | pregnancy complications |
| 353 | 0.064076 | 0.053508 | 1.197511 | 0.231108 | -0.0408 | 0.168949 | EUR | Nerve root and p | neurological |
| 772.1 | -0.08315 | 0.069441 | -1.19743 | 0.23114 | -0.21925 | 0.052951 | EUR | Muscular wasting | symptoms |
| 253.3 | 0.15462 | 0.129132 | 1.197381 | 0.231158 | -0.09847 | 0.407714 | EUR | Diabetes insipidu | endocrine/metabolic |
| 41.11 | 0.082426 | 0.068879 | 1.196676 | 0.231433 | -0.05257 | 0.217427 | EUR | Methicillin sensiti | infectious diseases |
| 255.22 | 0.834262 | 0.697559 | 1.195973 | 0.231707 | -0.53293 | 2.201453 | EUR | Mineralocorticoid | endocrine/metabolic |
| 270.33 | -0.1244 | 0.104066 | -1.19539 | 0.231937 | -0.32837 | 0.079567 | EUR | Amyloidosis | endocrine/metabolic |
| 535 | 0.029455 | 0.024658 | 1.194553 | 0.232262 | -0.01887 | 0.077784 | EUR | Gastritis and duo | digestive |
| 272 | 0.022572 | 0.0189 | 1.194276 | 0.23237 | -0.01447 | 0.059615 | EUR | Disorders of lipoi | endocrine/metabolic |
| 722.6 | 0.023023 | 0.019282 | 1.194007 | 0.232475 | -0.01477 | 0.060815 | EUR | Degeneration of i | musculoskeletal |
| 365.11 | -0.07058 | 0.059195 | -1.19237 | 0.233116 | -0.1866 | 0.045438 | EUR | Primary open ang | sense organs |
| 599.8 | -0.03895 | 0.032729 | -1.18995 | 0.234067 | -0.10309 | 0.025201 | EUR | Other symptoms | genitourinary |
| 447.7 | 0.054063 | 0.045518 | 1.18772 | 0.234944 | -0.03515 | 0.143278 | EUR | Aortic ectasia | circulatory system |
| 287.31 | -0.10495 | 0.088522 | -1.18558 | 0.235787 | -0.27845 | 0.06855 | EUR | Primary thrombo | hematopoietic |
| 654 | -0.05515 | 0.046723 | -1.18044 | 0.237827 | -0.14673 | 0.036422 | EUR | Other and unspe | pregnancy complications |
| 946 | 0.0801 | 0.067876 | 1.180095 | 0.237962 | -0.05293 | 0.213135 | EUR | Anaphylactic sho | injuries & poisonings |
| 333.8 | 0.14326 | 0.121525 | 1.178855 | 0.238456 | -0.09492 | 0.381444 | EUR | Other degenerati | neurological |
| 41.4 | 0.058041 | 0.049258 | 1.178312 | 0.238672 | -0.0385 | 0.154584 | EUR | E. coli | infectious diseases |
| 264.3 | -1.14989 | 0.977821 | -1.17597 | 0.239606 | -3.06638 | 0.766603 | EUR | Delayed milestor | endocrine/metabolic |
| 315.2 | -0.11245 | 0.095911 | -1.17249 | 0.241 | -0.30044 | 0.075527 | EUR | Speech and lang | mental disorders |
| 293 | -0.04786 | 0.040843 | -1.17172 | 0.241308 | -0.12791 | 0.032194 | EUR | Symptoms involv | mental disorders |
| 520 | 0.259314 | 0.221604 | 1.170169 | 0.241933 | -0.17502 | 0.693649 | EUR | Disorders of tootl | digestive |
| 707 | 0.038633 | 0.033041 | 1.169254 | 0.242301 | -0.02613 | 0.103393 | EUR | Chronic ulcer of s | dermatologic |
| 272.1 | 0.022097 | 0.018899 | 1.169218 | 0.242316 | -0.01494 | 0.059138 | EUR | Hyperlipidemia | endocrine/metabolic |
| 459.9 | 0.028424 | 0.024329 | 1.168345 | 0.242668 | -0.01926 | 0.076108 | EUR | Circulatory disea | circulatory system |
| 528.3 | 0.175314 | 0.150082 | 1.16812 | 0.242758 | -0.11884 | 0.469469 | EUR | Cellulitis and abs | digestive |
| 251.1 | 0.065171 | 0.055792 | 1.168104 | 0.242765 | -0.04418 | 0.17452 | EUR | Hypoglycemia | endocrine/metabolic |
| 80 | 0.06182 | 0.052936 | 1.167823 | 0.242878 | -0.04193 | 0.165573 | EUR | Postoperative inf | infectious diseases |
| 550.5 | 0.042153 | 0.0361 | 1.167693 | 0.242931 | -0.0286 | 0.112907 | EUR | Ventral hernia | digestive |
| 722 | 0.0215 | 0.018457 | 1.164903 | 0.244058 | -0.01467 | 0.057674 | EUR | Intervertebral dis | musculoskeletal |
| 366.3 | 0.540138 | 0.464583 | 1.16263 | 0.24498 | -0.37043 | 1.450704 | EUR | Traumatic catara | sense organs |
| 604 | 0.072306 | 0.062213 | 1.162242 | 0.245137 | -0.04963 | 0.194241 | EUR | Disorders of peni | genitourinary |
| 512 | 0.019198 | 0.016579 | 1.157971 | 0.246876 | -0.0133 | 0.051691 | EUR | Other symptoms | respiratory |
| 381 | -0.03257 | 0.028134 | -1.15782 | 0.246938 | -0.08772 | 0.022568 | EUR | Otitis media and | sense organs |
| 281.13 | -0.14699 | 0.127226 | -1.15537 | 0.247938 | -0.39635 | 0.102365 | EUR | Folate-deficiency | hematopoietic |
| 980 | 0.052857 | 0.045804 | 1.153983 | 0.248507 | -0.03692 | 0.142632 | EUR | Encounter for lon | infectious diseases |
| 560.3 | 0.1265 | 0.109679 | 1.153364 | 0.248761 | -0.08847 | 0.341467 | EUR | Peritoneal or inte | digestive |

| phecode | Coef. | Std.Err. | z | p_value_z | [0.025 | 0.975] | GIA | phenotype | category |
|---|---|---|---|---|---|---|---|---|---|
| 530.5 | 0.053859 | 0.046706 | 1.153147 | 0.24885 | -0.03768 | 0.145401 | EUR | Disorders of esop | digestive |
| 530.3 | 0.056324 | 0.048861 | 1.152742 | 0.249016 | -0.03944 | 0.152089 | EUR | Stricture and ster | digestive |
| 381.2 | -0.04255 | 0.037047 | -1.14863 | 2.51E-01 | -0.11516 | 0.030057 | EUR | Eustachian tube | sense organs |
| 711 | 0.089231 | 0.077762 | 1.147489 | 0.25118 | -0.06318 | 0.24164 | EUR | Arthropathy asso | musculoskeletal |
| 686 | -0.03121 | 0.027227 | -1.14631 | 0.251668 | -0.08458 | 0.022154 | EUR | Other local infect | dermatologic |
| 427.8 | 0.047565 | 0.041535 | 1.145177 | 0.252136 | -0.03384 | 0.128972 | EUR | Sinoatrial node d | circulatory system |
| 204.21 | 0.124362 | 0.108608 | 1.145053 | 0.252187 | -0.08851 | 0.33723 | EUR | Myeloid leukemia | neoplasms |
| 300.9 | 0.061825 | 0.053995 | 1.145006 | 0.252207 | -0.044 | 0.167653 | EUR | Posttraumatic str | mental disorders |
| 988 | -0.88915 | 0.776883 | -1.14451 | 0.252411 | -2.41181 | 0.63351 | EUR | Toxic effect of no | injuries & poisonings |
| 256 | 0.073027 | 0.063848 | 1.143764 | 0.252722 | -0.05211 | 0.198165 | EUR | Ovarian dysfunct | endocrine/metabolic |
| 751.22 | -0.11571 | 0.101196 | -1.14347 | 0.252842 | -0.31405 | 0.082625 | EUR | Other specified c | congenital anomalies |
| 949 | -0.02395 | 0.020982 | -1.1413 | 0.253744 | -0.06507 | 0.017177 | EUR | Allergies, other | injuries & poisonings |
| 750.11 | -0.11188 | 0.098204 | -1.13927 | 0.254592 | -0.30436 | 0.080596 | EUR | Esophageal atres | congenital anomalies |
| 752.1 | 0.154103 | 0.135477 | 1.13748 | 0.255338 | -0.11143 | 0.419633 | EUR | Neural tube defe | congenital anomalies |
| 426.31 | 0.0522 | 0.0459 | 1.137249 | 0.255434 | -0.03776 | 0.142163 | EUR | Right bundle bra | circulatory system |
| 446.9 | -0.09888 | 0.087046 | -1.13597 | 0.255969 | -0.26949 | 0.071725 | EUR | Arteritis NOS | circulatory system |
| 283.21 | 0.469128 | 0.414901 | 1.130698 | 2.58E-01 | -0.34406 | 1.282318 | EUR | Hemolytic-uremic | hematopoietic |
| 729.1 | 0.33305 | 0.294589 | 1.130557 | 0.258242 | -0.24433 | 0.910433 | EUR | Rheumatism, uns | musculoskeletal |
| 596.1 | -0.07895 | 0.069989 | -1.12804 | 0.259304 | -0.21613 | 0.058226 | EUR | Bladder neck obs | genitourinary |
| 871.2 | -0.05487 | 0.048644 | -1.12793 | 0.25935 | -0.15021 | 0.040473 | EUR | Open wound of fi | injuries & poisonings |
| 246.2 | -0.10967 | 0.097254 | -1.12768 | 0.259455 | -0.30029 | 0.080943 | EUR | Thyroid cyst | endocrine/metabolic |
| 580 | 0.041697 | 0.037072 | 1.124763 | 0.26069 | -0.03096 | 0.114356 | EUR | Nephritis; nephro | genitourinary |
| 526.1 | 0.274837 | 0.244841 | 1.12251 | 0.261646 | -0.20504 | 0.754717 | EUR | Cysts of the jaws | digestive |
| 526.42 | 0.088561 | 0.079014 | 1.120829 | 0.262361 | -0.0663 | 0.243425 | EUR | Arthralgia/ankylo | digestive |
| 578.1 | 0.079244 | 0.070739 | 1.12023 | 0.262616 | -0.0594 | 0.217891 | EUR | Hematemesis | digestive |
| 289.4 | 0.027017 | 0.02415 | 1.118693 | 0.263271 | -0.02032 | 0.07435 | EUR | Lymphadenitis | hematopoietic |
| 618.2 | 0.086722 | 0.077603 | 1.117509 | 0.263777 | -0.06538 | 0.238822 | EUR | Uterine/Uterovag | genitourinary |
| 362.1 | -0.32871 | 0.295173 | -1.11361 | 0.265446 | -0.90724 | 0.249821 | EUR | Retinopathy of pr | sense organs |
| 426.22 | 0.196106 | 0.176368 | 1.111916 | 0.266174 | -0.14957 | 0.541781 | EUR | Mobitz II AV bloc | circulatory system |
| 180.1 | 0.094423 | 0.085119 | 1.10931 | 0.267296 | -0.07241 | 0.261253 | EUR | Cervical cancer | neoplasms |
| 528.12 | -0.08767 | 0.079045 | -1.10908 | 0.267397 | -0.24259 | 0.067259 | EUR | Oral aphthae | digestive |
| 705.3 | 0.163544 | 0.147523 | 1.108601 | 0.267602 | -0.1256 | 0.452683 | EUR | Hidradenitis | dermatologic |
| 362.26 | -0.05265 | 0.047612 | -1.1059 | 0.268768 | -0.14597 | 0.040663 | EUR | Macular puckerin | sense organs |
| 618 | 0.048075 | 0.043489 | 1.105435 | 0.268971 | -0.03716 | 0.133312 | EUR | Genital prolapse | genitourinary |
| 227.3 | -0.08625 | 0.078056 | -1.10501 | 0.269154 | -0.23924 | 0.066734 | EUR | Benign neoplasm | neoplasms |
| 592.11 | 0.030396 | 0.027554 | 1.10317 | 0.269953 | -0.02361 | 0.084401 | EUR | Acute cystitis | genitourinary |
| 362.31 | -0.13908 | 0.126223 | -1.10189 | 0.270508 | -0.38648 | 0.108308 | EUR | Separation of ret | sense organs |

| phecode | Coef. | Std.Err. | z | p_value_z | [0.025 | 0.975] | GIA | phenotype | category |
|---|---|---|---|---|---|---|---|---|---|
| 610.8 | -0.06644 | 0.060309 | -1.10175 | 0.270573 | -0.18465 | 0.051758 | EUR | Other specified b | genitourinary |
| 245.1 | -0.20502 | 0.186173 | -1.10125 | 0.270787 | -0.56992 | 0.159869 | EUR | Thyroiditis, acute | endocrine/metabolic |
| 184 | -0.06265 | 0.056915 | -1.10067 | 0.271039 | -0.1742 | 0.048907 | EUR | Cancer of other f | neoplasms |
| 327.5 | -0.07352 | 0.066837 | -1.09996 | 0.271352 | -0.20452 | 0.05748 | EUR | Parasomnia | neurological |
| 220 | -0.09446 | 0.086138 | -1.09666 | 0.272791 | -0.26329 | 0.074363 | EUR | Benign neoplasm | neoplasms |
| 281 | 0.042524 | 0.038786 | 1.096395 | 0.272906 | -0.03349 | 0.118543 | EUR | Other deficiency | hematopoietic |
| 302 | 0.054441 | 0.049682 | 1.095789 | 0.273171 | -0.04293 | 0.151817 | EUR | Sexual and gend | mental disorders |
| 593.1 | 0.045255 | 0.041402 | 1.093043 | 0.274375 | -0.03589 | 0.126402 | EUR | Gross hematuria | genitourinary |
| 527.2 | 0.091544 | 0.083799 | 1.09242 | 0.274649 | -0.0727 | 0.255788 | EUR | Sialoadenitis | digestive |
| 504 | 0.050523 | 0.046331 | 1.090481 | 0.275501 | -0.04028 | 0.14133 | EUR | Other alveolar ar | respiratory |
| 189.21 | 0.060797 | 0.055931 | 1.087009 | 0.277033 | -0.04882 | 0.170419 | EUR | Malignant neopla | neoplasms |
| 274.11 | -0.04891 | 0.045133 | -1.08363 | 0.278527 | -0.13737 | 0.039551 | EUR | Gouty arthropath | endocrine/metabolic |
| 259.2 | 0.186367 | 0.172266 | 1.081859 | 0.279315 | -0.15127 | 0.524001 | EUR | Carcinoid syndro | endocrine/metabolic |
| 596.5 | 0.036202 | 0.033615 | 1.076946 | 0.281504 | -0.02968 | 0.102086 | EUR | Functional disord | genitourinary |
| 189 | 0.045363 | 0.042154 | 1.076129 | 0.28187 | -0.03726 | 0.127982 | EUR | Cancer of urinary | neoplasms |
| 208 | -0.01996 | 0.018578 | -1.07419 | 0.282738 | -0.05637 | 0.016456 | EUR | Benign neoplasm | neoplasms |
| 792.1 | -0.05227 | 0.048749 | -1.07226 | 0.283602 | -0.14782 | 0.043274 | EUR | Papanicolaou sm | genitourinary |
| 742.2 | 0.154913 | 0.144833 | 1.069602 | 0.284798 | -0.12895 | 0.43878 | EUR | Pathological, dev | musculoskeletal |
| 601.1 | -0.05904 | 0.05525 | -1.06858 | 0.285261 | -0.16733 | 0.049249 | EUR | Prostatitis | genitourinary |
| 271 | -0.02082 | 0.019536 | -1.06553 | 0.286638 | -0.05911 | 0.017474 | EUR | Disorders of carb | endocrine/metabolic |
| 53 | 0.039276 | 0.036905 | 1.064249 | 0.287216 | -0.03306 | 0.111609 | EUR | Herpes zoster | infectious diseases |
| 729.3 | -0.13639 | 0.12842 | -1.06207 | 0.288202 | -0.38809 | 0.115307 | EUR | Panniculitis | musculoskeletal |
| 871.1 | 0.066511 | 0.063177 | 1.052769 | 0.292447 | -0.05731 | 0.190335 | EUR | Open wound of h | injuries & poisonings |
| 529.6 | -0.09484 | 0.090111 | -1.0525 | 0.292571 | -0.27145 | 0.081772 | EUR | Glossodynia | digestive |
| 794 | -0.10289 | 0.097814 | -1.05194 | 0.292828 | -0.29461 | 0.088818 | EUR | Abnormal results | symptoms |
| 218.2 | 0.237552 | 0.226061 | 1.050829 | 0.293337 | -0.20552 | 0.680623 | EUR | Other benign neo | neoplasms |
| 537.1 | 0.118704 | 0.112963 | 1.050824 | 0.293339 | -0.1027 | 0.340106 | EUR | Lesions of stoma | digestive |
| 605 | -0.03069 | 0.02923 | -1.04988 | 0.293774 | -0.08798 | 0.026602 | EUR | Erectile dysfuncti | genitourinary |
| 747.2 | 0.070017 | 0.066815 | 1.047934 | 0.294669 | -0.06094 | 0.200971 | EUR | Congenital anom | congenital anomalies |
| 260.1 | 0.065443 | 0.062555 | 1.046158 | 0.295488 | -0.05716 | 0.188049 | EUR | Cachexia | endocrine/metabolic |
| 79.2 | 0.119308 | 0.114054 | 1.046064 | 0.295531 | -0.10423 | 0.342849 | EUR | Infectious monon | infectious diseases |
| 117 | 0.050996 | 0.048772 | 1.045607 | 0.295742 | -0.04459 | 0.146587 | EUR | Mycoses | infectious diseases |
| 301 | 0.09729 | 0.09314 | 1.044548 | 0.296232 | -0.08526 | 0.279841 | EUR | Personality disor | mental disorders |
| 796 | -0.0361 | 0.034624 | -1.04272 | 0.297076 | -0.10396 | 0.031758 | EUR | Elevated prostate | genitourinary |
| 272.9 | -0.09176 | 0.088021 | -1.04245 | 2.97E-01 | -0.26428 | 0.080761 | EUR | Unspecified disor | endocrine/metabolic |
| 429.3 | -0.02396 | 0.02301 | -1.04116 | 0.297803 | -0.06905 | 0.021141 | EUR | Symptoms involv | circulatory system |
| 358 | -0.07413 | 0.071228 | -1.04077 | 0.297983 | -0.21374 | 0.065472 | EUR | Myoneural disord | neurological |

| phecode | Coef. | Std.Err. | z | p_value_z | [0.025 | 0.975] | GIA | phenotype | category |
|---|---|---|---|---|---|---|---|---|---|
| 788 | 0.027029 | 0.026048 | 1.037646 | 0.299435 | -0.02402 | 0.078082 | EUR | Syncope and col | symptoms |
| 331.1 | -0.0912 | 0.088166 | -1.03436 | 0.300967 | -0.264 | 0.081607 | EUR | Hydrocephalus | neurological |
| 742.9 | 0.047869 | 0.046321 | 1.033412 | 0.301411 | -0.04292 | 0.138657 | EUR | Other derangeme | musculoskeletal |
| 340.1 | -0.03863 | 0.037408 | -1.03257 | 0.301804 | -0.11194 | 0.034692 | EUR | Migrain with aura | neurological |
| 756.1 | 0.173372 | 0.168124 | 1.031215 | 0.30244 | -0.15615 | 0.502889 | EUR | Congenital anom | congenital anomalies |
| 340 | 0.023604 | 0.022992 | 1.026632 | 0.304594 | -0.02146 | 0.068667 | EUR | Migraine | neurological |
| 509.3 | -0.08547 | 0.083341 | -1.02556 | 0.305098 | -0.24882 | 0.077874 | EUR | Pulmonary insuff | respiratory |
| 850 | 0.050311 | 0.049096 | 1.024753 | 0.30548 | -0.04592 | 0.146538 | EUR | Hemorrhage or h | injuries & poisonings |
| 711.2 | -0.17077 | 0.166781 | -1.02391 | 0.305879 | -0.49765 | 0.156117 | EUR | Reiter's disease | musculoskeletal |
| 202.22 | -0.08472 | 0.082822 | -1.02297 | 0.306323 | -0.24705 | 0.077604 | EUR | Reticulosarcoma | neoplasms |
| 755.61 | 0.157089 | 0.153738 | 1.021796 | 0.306877 | -0.14423 | 0.458411 | EUR | Congenital hip dy | congenital anomalies |
| 425.8 | 0.140685 | 0.137906 | 1.020152 | 0.307656 | -0.12961 | 0.410976 | EUR | Other cardiomyo | circulatory system |
| 610.3 | -0.08178 | 0.08038 | -1.01746 | 3.09E-01 | -0.23933 | 0.075759 | EUR | Fibrosclerosis of | genitourinary |
| 747.13 | 0.074428 | 0.073308 | 1.015273 | 0.309976 | -0.06925 | 0.218109 | EUR | Congenital anom | congenital anomalies |
| 619.5 | -0.05699 | 0.056185 | -1.01426 | 0.310458 | -0.16711 | 0.053134 | EUR | Noninflammatory | genitourinary |
| 527.8 | -0.07767 | 0.076636 | -1.0135 | 0.310822 | -0.22788 | 0.072534 | EUR | Other specified d | digestive |
| 870.3 | -0.04879 | 0.048194 | -1.01242 | 0.311336 | -0.14325 | 0.045666 | EUR | Other open wour | injuries & poisonings |
| 735.3 | -0.04091 | 0.040429 | -1.01194 | 0.311565 | -0.12015 | 0.038327 | EUR | Hallux valgus (Bu | musculoskeletal |
| 275.51 | -0.04727 | 0.046745 | -1.01122 | 0.311911 | -0.13889 | 0.044349 | EUR | Hypocalcemia | endocrine/metabolic |
| 573.2 | 0.070869 | 0.070253 | 1.008777 | 0.313082 | -0.06682 | 0.208562 | EUR | Liver replaced by | digestive |
| 626.14 | 0.059631 | 0.059208 | 1.007142 | 0.313866 | -0.05641 | 0.175677 | EUR | Irregular menstru | genitourinary |
| 348.7 | 0.070214 | 0.069726 | 1.006995 | 0.313937 | -0.06645 | 0.206875 | EUR | Coma | neurological |
| 761 | 0.020179 | 0.020108 | 1.003533 | 0.315604 | -0.01923 | 0.059591 | EUR | Cervicalgia | symptoms |
| 573.5 | -0.0469 | 0.046752 | -1.00324 | 0.315747 | -0.13854 | 0.044729 | EUR | Jaundice (not of | digestive |
| 728 | 0.067276 | 0.067132 | 1.002141 | 0.316275 | -0.0643 | 0.198853 | EUR | Disorders of mus | musculoskeletal |
| 297 | 0.063701 | 0.06358 | 1.001897 | 0.316393 | -0.06091 | 0.188316 | EUR | Suicidal ideation | mental disorders |
| 710.3 | 0.55022 | 0.549627 | 1.00108 | 0.316788 | -0.52703 | 1.627468 | EUR | Osteopathy resul | musculoskeletal |
| 79.9 | -0.04368 | 0.043657 | -1.00046 | 0.317086 | -0.12924 | 0.041889 | EUR | Viremia, NOS | infectious diseases |
| 800.3 | 0.063424 | 0.063465 | 0.999358 | 0.317621 | -0.06096 | 0.187813 | EUR | Fracture of tibia a | injuries & poisonings |
| 286.13 | 0.240924 | 0.241206 | 0.998832 | 0.317876 | -0.23183 | 0.713678 | EUR | Congenital factor | hematopoietic |
| 626.21 | -0.33233 | 0.333254 | -0.99723 | 0.318651 | -0.9855 | 0.320834 | EUR | Mittelschmerz | genitourinary |
| 575.7 | 0.049417 | 0.049666 | 0.994983 | 0.319745 | -0.04793 | 0.146761 | EUR | Other disorders c | digestive |
| 280.2 | 0.036642 | 0.036852 | 0.994306 | 0.320074 | -0.03559 | 0.108872 | EUR | Iron deficiency ar | hematopoietic |
| 448 | -0.05788 | 0.058271 | -0.99328 | 0.320574 | -0.17209 | 0.05633 | EUR | Disease of capilla | circulatory system |
| 571.6 | 0.111876 | 0.112778 | 0.991997 | 0.321199 | -0.10917 | 0.332918 | EUR | Primary biliary cir | digestive |
| 255 | 0.038299 | 0.038687 | 0.989952 | 0.322198 | -0.03753 | 0.114124 | EUR | Disorders of adre | endocrine/metabolic |
| 681.6 | 0.070606 | 0.071377 | 0.989204 | 0.322563 | -0.06929 | 0.210503 | EUR | Cellulitis and abs | dermatologic |

| phecode | Coef. | Std.Err. | z | p_value_z | [0.025 | 0.975] | GIA | phenotype | category |
|---|---|---|---|---|---|---|---|---|---|
| 620 | -0.09969 | 0.10095 | -0.98753 | 0.323382 | -0.29755 | 0.098167 | EUR | Dysplasia of fema | genitourinary |
| 957 | 0.216292 | 0.219196 | 0.986753 | 0.323764 | -0.21332 | 0.645907 | EUR | Injury to other an | injuries & poisonings |
| 384.1 | 0.216305 | 0.219766 | 0.98425 | 0.324992 | -0.21443 | 0.647038 | EUR | Myringitis | sense organs |
| 271.3 | -0.01927 | 0.019603 | -0.98312 | 0.325549 | -0.05769 | 0.019149 | EUR | Intestinal disacch | endocrine/metabolic |
| 573.6 | 0.032154 | 0.032711 | 0.982974 | 0.32562 | -0.03196 | 0.096267 | EUR | Nonspecific eleva | digestive |
| 751.21 | 0.048059 | 0.048974 | 0.981321 | 0.326435 | -0.04793 | 0.144046 | EUR | Cystic kidney dis | congenital anomalies |
| 327.7 | 0.035918 | 0.036639 | 0.980315 | 0.32693 | -0.03589 | 0.10773 | EUR | Sleep related mo | neurological |
| 709.7 | 0.055317 | 0.05645 | 0.979924 | 0.327124 | -0.05532 | 0.165956 | EUR | Unspecified diffus | dermatologic |
| 614.1 | 0.098972 | 0.10111 | 0.978854 | 0.327652 | -0.0992 | 0.297143 | EUR | Pelvic peritoneal | genitourinary |
| 355 | 0.064797 | 0.066317 | 0.977087 | 0.328526 | -0.06518 | 0.194775 | EUR | Complex regiona | neurological |
| 842 | -0.02537 | 0.025963 | -0.97701 | 0.328563 | -0.07625 | 0.02552 | EUR | Other sprains an | injuries & poisonings |
| 781.1 | 0.102728 | 0.105154 | 0.976925 | 0.328606 | -0.10337 | 0.308827 | EUR | Loss of height | symptoms |
| 755.4 | 0.195853 | 0.200778 | 0.97547 | 0.329327 | -0.19767 | 0.589372 | EUR | Congenital anom | congenital anomalies |
| 695 | -0.02329 | 0.023907 | -0.97427 | 0.329923 | -0.07015 | 0.023565 | EUR | Erythematous co | dermatologic |
| 750.1 | -0.05046 | 0.051811 | -0.97391 | 0.330101 | -0.15201 | 0.051089 | EUR | Upper gastrointes | congenital anomalies |
| 713.5 | 0.191018 | 0.196544 | 0.971884 | 0.331108 | -0.1942 | 0.576238 | EUR | Arthropathy asso | musculoskeletal |
| 370.31 | -0.12397 | 0.12757 | -0.97174 | 0.331178 | -0.374 | 0.126068 | EUR | Keratoconjunctivi | sense organs |
| 704.8 | -0.02808 | 0.029 | -0.96824 | 0.332925 | -0.08492 | 0.02876 | EUR | Other specified d | dermatologic |
| 1000 | -0.07586 | 0.078552 | -0.96575 | 0.33417 | -0.22982 | 0.078098 | EUR | Burns | |
| 427.11 | 0.03019 | 0.031287 | 0.964926 | 0.334582 | -0.03113 | 0.091512 | EUR | Paroxysmal supr | circulatory system |
| 8 | 0.028886 | 0.029965 | 0.963975 | 0.335058 | -0.02984 | 0.087616 | EUR | Intestinal infectio | infectious diseases |
| 452.8 | -0.1104 | 0.114706 | -0.9625 | 0.335798 | -0.33522 | 0.114414 | EUR | Postphlebitic syn | circulatory system |
| 473.4 | 0.028451 | 0.02956 | 0.962495 | 0.335801 | -0.02949 | 0.086388 | EUR | Voice disturbance | respiratory |
| 429 | -0.02034 | 0.021139 | -0.96226 | 0.33592 | -0.06177 | 0.021091 | EUR | Ill-defined descrip | circulatory system |
| 198.1 | -0.02962 | 0.030784 | -0.96218 | 0.335959 | -0.08996 | 0.030716 | EUR | Secondary malig | neoplasms |
| 117.2 | 0.138584 | 0.144253 | 0.960706 | 0.3367 | -0.14415 | 0.421315 | EUR | Coccidioidomyco | infectious diseases |
| 415.1 | 0.042318 | 0.044055 | 0.960569 | 0.336769 | -0.04403 | 0.128665 | EUR | Acute pulmonary | circulatory system |
| 320 | -0.07549 | 0.078673 | -0.95952 | 0.337299 | -0.22968 | 0.078708 | EUR | Meningitis | neurological |
| 306.9 | 5.15E-02 | 0.053746 | 0.958717 | 0.337701 | -0.05381 | 0.156866 | EUR | Tension headach | mental disorders |
| 301.2 | 0.121952 | 0.128494 | 0.949088 | 0.342576 | -0.12989 | 0.373794 | EUR | Antisocial/border | mental disorders |
| 803.3 | -0.07066 | 0.07447 | -0.94888 | 0.342684 | -0.21662 | 0.075296 | EUR | Fracture of clavic | injuries & poisonings |
| 947 | 0.038626 | 0.040727 | 0.948411 | 0.34292 | -0.0412 | 0.118449 | EUR | Urticaria | dermatologic |
| 363.3 | 0.099 | 0.104544 | 0.946963 | 0.343658 | -0.1059 | 0.303903 | EUR | Chorioretinal sca | sense organs |
| 297.2 | 0.125421 | 0.132501 | 0.946563 | 0.343861 | -0.13428 | 0.385118 | EUR | Suicide or self-in | mental disorders |
| 447.1 | 0.062589 | 0.066219 | 0.945175 | 0.34457 | -0.0672 | 0.192376 | EUR | Stricture of artery | circulatory system |
| 743.9 | -0.01999 | 0.021173 | -0.94403 | 0.345153 | -0.06149 | 0.02151 | EUR | Osteopenia or ot | musculoskeletal |
| 523.1 | 0.08666 | 0.091877 | 0.943215 | 0.345571 | -0.09342 | 0.266736 | EUR | Gingivitis | digestive |

| phecode | Coef. | Std.Err. | z | p_value_z | [0.025 | 0.975] | GIA | phenotype | category |
|---|---|---|---|---|---|---|---|---|---|
| 364.9 | -0.13421 | 0.142292 | -0.94321 | 0.345574 | -0.4131 | 0.144676 | EUR | Cornea replaced | sense organs |
| 745 | 0.015672 | 0.016653 | 0.941082 | 0.346663 | -0.01697 | 0.04831 | EUR | Pain in joint | musculoskeletal |
| 707.3 | 0.041945 | 0.044609 | 0.940298 | 0.347065 | -0.04549 | 0.129377 | EUR | Chronic ulcer of u | dermatologic |
| 615 | 0.045993 | 0.048951 | 0.939573 | 0.347437 | -0.04995 | 0.141936 | EUR | Endometriosis | genitourinary |
| 722.8 | 0.050814 | 0.054192 | 0.937671 | 0.348413 | -0.0554 | 0.157029 | EUR | Postlaminectomy | musculoskeletal |
| 575.1 | -0.0844 | 0.090131 | -0.93642 | 0.349055 | -0.26105 | 0.092253 | EUR | Cholangitis | digestive |
| 285.2 | 0.025676 | 0.027425 | 0.936204 | 0.349168 | -0.02808 | 0.079428 | EUR | Anemia of chroni | hematopoietic |
| 358.1 | -0.13658 | 0.145924 | -0.93594 | 0.349303 | -0.42258 | 0.149429 | EUR | Myasthenia gravi | neurological |
| 701.2 | -0.02498 | 0.026719 | -0.93487 | 0.349858 | -0.07735 | 0.02739 | EUR | Scar conditions a | dermatologic |
| 752.2 | -0.07771 | 0.083286 | -0.93305 | 0.350794 | -0.24095 | 0.085527 | EUR | Other specified c | congenital anomalies |
| 289 | 0.023838 | 0.025594 | 0.93142 | 0.351637 | -0.02632 | 0.074001 | EUR | Other diseases o | hematopoietic |
| 580.32 | 0.047878 | 0.051591 | 0.928037 | 0.353389 | -0.05324 | 0.148995 | EUR | Nephritis and nep | genitourinary |
| 345.11 | -0.0853 | 0.092003 | -0.92714 | 0.353854 | -0.26562 | 0.095023 | EUR | Generalized conv | neurological |
| 327.71 | 0.044549 | 0.048118 | 0.925844 | 0.354527 | -0.04976 | 0.138858 | EUR | Restless legs syn | neurological |
| 612 | -0.04937 | 0.053368 | -0.92499 | 0.354972 | -0.15397 | 0.055235 | EUR | Breast conditions | genitourinary |
| 701.3 | -0.06611 | 0.071638 | -0.92288 | 0.356071 | -0.20652 | 0.074295 | EUR | Circumscribed sc | dermatologic |
| 989 | 0.161043 | 0.174955 | 0.920483 | 0.357321 | -0.18186 | 0.503949 | EUR | Toxic effect of oth | injuries & poisonings |
| 182 | -0.06306 | 0.06865 | -0.91865 | 0.35828 | -0.19762 | 0.071486 | EUR | Malignant neopla | neoplasms |
| 531.2 | -0.0386 | 0.042023 | -0.91842 | 0.358399 | -0.12096 | 0.043769 | EUR | Gastric ulcer | digestive |
| 715.1 | 0.036207 | 0.039427 | 0.918316 | 0.358454 | -0.04107 | 0.113483 | EUR | Sacroiliitis NEC | musculoskeletal |
| 348 | 0.030367 | 0.033132 | 0.916557 | 0.359375 | -0.03457 | 0.095305 | EUR | Other conditions | neurological |
| 736.4 | 0.13296 | 0.145249 | 0.915397 | 0.359983 | -0.15172 | 0.417643 | EUR | Genu valgum or v | musculoskeletal |
| 523.31 | 0.077374 | 0.084604 | 0.914549 | 0.360429 | -0.08845 | 0.243194 | EUR | Acute periodontit | digestive |
| 930 | -0.03004 | 0.032902 | -0.91294 | 0.361273 | -0.09452 | 0.034449 | EUR | Allergic reaction t | injuries & poisonings |
| 1008 | 0.080193 | 0.087926 | 0.912052 | 0.361742 | -0.09214 | 0.252523 | EUR | Crushing or internal injury to organ | |
| 638 | -0.04656 | 0.051073 | -0.91165 | 0.361954 | -0.14666 | 0.05354 | EUR | Other high-risk pr | pregnancy complications |
| 701.6 | 0.104895 | 0.115229 | 0.910311 | 0.362659 | -0.12095 | 0.33074 | EUR | Acquired acantho | dermatologic |
| 426.25 | -0.0944 | 0.103743 | -0.90993 | 0.36286 | -0.29773 | 0.108934 | EUR | Other heart block | circulatory system |
| 250.42 | 0.016155 | 0.017759 | 0.909683 | 0.36299 | -0.01865 | 0.050961 | EUR | Other abnormal g | endocrine/metabolic |
| 578.2 | 0.030672 | 0.03374 | 0.909067 | 0.363315 | -0.03546 | 0.0968 | EUR | Blood in stool | digestive |
| 441.1 | 0.136413 | 0.15057 | 0.905979 | 0.364947 | -0.1587 | 0.431525 | EUR | Acute vascular in | circulatory system |
| 681.7 | 0.041933 | 0.046341 | 0.904889 | 0.365524 | -0.04889 | 0.13276 | EUR | Cellulitis and abs | dermatologic |
| 531.1 | -0.07621 | 0.084268 | -0.9044 | 0.365783 | -0.24138 | 0.088951 | EUR | Hemorrhage from | digestive |
| 374.3 | 0.043635 | 0.048305 | 0.903312 | 0.366361 | -0.05104 | 0.138311 | EUR | Ptosis of eyelid | sense organs |
| 696.3 | 0.134889 | 0.149345 | 0.903204 | 0.366418 | -0.15782 | 0.4276 | EUR | Pityriasis | dermatologic |
| 613.5 | 0.034415 | 0.038176 | 0.901481 | 0.367333 | -0.04041 | 0.10924 | EUR | Mastodynia | genitourinary |
| 585.31 | 0.037561 | 0.041732 | 0.900059 | 0.368089 | -0.04423 | 0.119355 | EUR | Renal dialysis | genitourinary |

| phecode | Coef. | Std.Err. | z | p_value_z | [0.025 | 0.975] | GIA | phenotype | category |
|---|---|---|---|---|---|---|---|---|---|
| 415.11 | 0.039645 | 0.044069 | 0.899609 | 0.368329 | -0.04673 | 0.126018 | EUR | Pulmonary embo | circulatory system |
| 757 | 0.104315 | 0.116216 | 0.897596 | 0.369401 | -0.12346 | 0.332093 | EUR | Congenital anom | congenital anomalies |
| 609.2 | 0.102983 | 0.114915 | 0.896166 | 0.370164 | -0.12225 | 0.328213 | EUR | Abnormal sperma | genitourinary |
| 800.4 | 0.079727 | 0.089069 | 0.895113 | 0.370727 | -0.09485 | 0.2543 | EUR | Fracture of patell | injuries & poisonings |
| 609.11 | 0.208639 | 0.233975 | 0.891714 | 0.372546 | -0.24994 | 0.667221 | EUR | Azoospermia and | genitourinary |
| 175 | -0.03454 | 0.038753 | -0.89125 | 0.372798 | -0.11049 | 0.041416 | EUR | Acquired absenc | neoplasms |
| 284.1 | 0.033642 | 0.037782 | 0.890417 | 3.73E-01 | -0.04041 | 0.107694 | EUR | Pancytopenia | hematopoietic |
| 526.3 | 0.154017 | 0.173322 | 0.888616 | 0.37421 | -0.18569 | 0.493722 | EUR | Anomalies of jaw | digestive |
| 411.41 | -0.10104 | 0.113943 | -0.88678 | 0.3752 | -0.32436 | 0.122282 | EUR | Aneurysm and di | circulatory system |
| 574.11 | -0.07991 | 0.090142 | -0.8865 | 0.375349 | -0.25659 | 0.096764 | EUR | Cholelithiasis wit | digestive |
| 747.12 | -0.05287 | 0.059789 | -0.88427 | 0.376551 | -0.17005 | 0.064314 | EUR | Valvular heart dis | congenital anomalies |
| 442.3 | 0.11817 | 0.133642 | 0.88423 | 0.376572 | -0.14376 | 0.380103 | EUR | Aneurysm of arte | circulatory system |
| 647.3 | -0.20838 | 0.235934 | -0.88322 | 0.377117 | -0.6708 | 0.25404 | EUR | Major puerperal i | pregnancy complications |
| 586.12 | 0.131813 | 0.14956 | 0.881337 | 0.378135 | -0.16132 | 0.424946 | EUR | Vesicoureteral re | genitourinary |
| 193 | -0.0461 | 0.052377 | -0.88021 | 0.378744 | -0.14876 | 0.056554 | EUR | Thyroid cancer | neoplasms |
| 709.3 | -0.07603 | 0.086551 | -0.87843 | 0.379713 | -0.24566 | 0.093608 | EUR | Systemic scleros | dermatologic |
| 1007 | -0.11454 | 0.130572 | -0.87724 | 0.380359 | -0.37046 | 0.141374 | EUR | Injury to blood vessels | |
| 586.1 | -0.11705 | 0.133807 | -0.87474 | 0.381716 | -0.3793 | 0.145211 | EUR | Anatomical abno | genitourinary |
| 174 | -0.02858 | 0.032739 | -0.87307 | 0.382628 | -0.09275 | 0.035584 | EUR | Breast cancer | neoplasms |
| 751.12 | 0.180398 | 0.207224 | 0.870548 | 0.384001 | -0.22575 | 0.58655 | EUR | Congenital anom | congenital anomalies |
| 915 | 0.034577 | 0.039767 | 0.869489 | 0.384579 | -0.04337 | 0.112519 | EUR | Superficial injury | injuries & poisonings |
| 41 | 0.019279 | 0.022188 | 0.86892 | 0.384891 | -0.02421 | 0.062767 | EUR | Bacterial infectio | infectious diseases |
| 625.1 | -0.04682 | 0.053944 | -0.86797 | 0.385412 | -0.15255 | 0.058906 | EUR | Dyspareunia | genitourinary |
| 441 | 0.065191 | 0.075119 | 0.867842 | 0.385481 | -0.08204 | 0.212421 | EUR | Vascular insuffici | circulatory system |
| 360.2 | -0.11499 | 0.132711 | -0.86648 | 0.386226 | -0.3751 | 0.145117 | EUR | Progressive myo | sense organs |
| 704.1 | -0.03042 | 0.035119 | -0.86627 | 0.386342 | -0.09925 | 0.038409 | EUR | Alopecia | dermatologic |
| 560 | 0.025885 | 0.029973 | 0.863589 | 0.387813 | -0.03286 | 0.084632 | EUR | Intestinal obstruc | digestive |
| 750 | -0.03588 | 0.041666 | -0.86124 | 0.389107 | -0.11755 | 0.045779 | EUR | Digestive conger | congenital anomalies |
| 586.3 | -0.12386 | 0.144084 | -0.85963 | 0.389992 | -0.40626 | 0.15854 | EUR | Vascular disorde | genitourinary |
| 938.2 | -0.02647 | 0.030843 | -0.85817 | 0.390801 | -0.08692 | 0.033983 | EUR | Chronic dermatiti | dermatologic |
| 710.2 | -0.64271 | 0.748949 | -0.85814 | 0.390813 | -2.11062 | 0.825208 | EUR | Periostitis | musculoskeletal |
| 253.5 | 0.220883 | 0.25757 | 0.857564 | 0.391133 | -0.28395 | 0.725711 | EUR | Pituitary dwarfisn | endocrine/metabolic |
| 578.9 | 0.034601 | 0.04048 | 0.854773 | 0.392677 | -0.04474 | 0.11394 | EUR | Hemorrhage of g | digestive |
| 635.3 | -0.08184 | 0.095934 | -0.85304 | 0.393639 | -0.26986 | 0.106192 | EUR | Placenta previa a | pregnancy complications |
| 292.3 | 0.025107 | 0.029545 | 0.849804 | 0.395434 | -0.0328 | 0.083013 | EUR | Memory loss | mental disorders |
| 528.7 | -0.11545 | 0.135926 | -0.84935 | 0.395688 | -0.38186 | 0.150961 | EUR | Sialolithiasis | digestive |
| 480.2 | 0.046315 | 0.054596 | 0.848318 | 0.396261 | -0.06069 | 0.153322 | EUR | Viral pneumonia | respiratory |

| phecode | Coef. | Std.Err. | z | p_value_z | [0.025 | 0.975] | GIA | phenotype | category |
|---|---|---|---|---|---|---|---|---|---|
| 292.11 | -0.05604 | 0.066061 | -0.84824 | 0.396305 | -0.18551 | 0.073442 | EUR | Aphasia | mental disorders |
| 350.6 | -0.0369 | 0.043534 | -0.84763 | 0.396641 | -0.12222 | 0.048424 | EUR | Disturbances of s | neurological |
| 315.3 | 0.140112 | 0.16546 | 0.8468 | 0.397107 | -0.18418 | 0.464408 | EUR | Mental retardatio | mental disorders |
| 368.1 | -0.07961 | 0.094087 | -0.84611 | 0.39749 | -0.26402 | 0.104799 | EUR | Amblyopia | sense organs |
| 368.91 | 0.091465 | 0.10881 | 0.840587 | 0.400579 | -0.1218 | 0.304729 | EUR | Psychophysical v | sense organs |
| 172.3 | -0.03078 | 0.036632 | -0.84032 | 0.40073 | -0.10258 | 0.041015 | EUR | Carcinoma in situ | neoplasms |
| 256.4 | 0.060566 | 0.072108 | 0.839923 | 4.01E-01 | -0.08076 | 0.201895 | EUR | Polycystic ovarie | endocrine/metabolic |
| 592.21 | 0.205139 | 0.244271 | 0.839803 | 0.401019 | -0.27362 | 0.683901 | EUR | Urethral syndrom | genitourinary |
| 159.4 | -0.077 | 0.092111 | -0.83592 | 0.403201 | -0.25753 | 0.103537 | EUR | Malignant neopla | neoplasms |
| 395.6 | 0.035666 | 0.042909 | 0.831205 | 0.405858 | -0.04843 | 0.119765 | EUR | Heart valve repla | circulatory system |
| 613.7 | 0.056882 | 0.068554 | 0.829738 | 0.406687 | -0.07748 | 0.191245 | EUR | Other signs and s | genitourinary |
| 871.3 | -0.08635 | 0.104115 | -0.82937 | 0.406893 | -0.29041 | 0.117711 | EUR | Open wound of fo | injuries & poisonings |
| 519.8 | 0.028029 | 0.033867 | 0.827643 | 0.407873 | -0.03835 | 0.094407 | EUR | Other diseases o | respiratory |
| 619.1 | -0.04118 | 0.049929 | -0.82483 | 0.409468 | -0.13904 | 0.056677 | EUR | Noninflammatory | genitourinary |
| 292.1 | -0.03331 | 0.040536 | -0.82183 | 0.411175 | -0.11276 | 0.046135 | EUR | Aphasia/speech d | mental disorders |
| 726.1 | -0.01751 | 0.021359 | -0.81978 | 0.412343 | -0.05937 | 0.024353 | EUR | Enthesopathy | musculoskeletal |
| 297.1 | 0.057419 | 0.070223 | 0.817672 | 0.413544 | -0.08021 | 0.195053 | EUR | Suicidal ideation | mental disorders |
| 555.21 | 0.045365 | 0.05559 | 0.816067 | 0.414462 | -0.06359 | 0.154321 | EUR | Ulcerative colitis | digestive |
| 840.2 | 0.034622 | 0.042433 | 0.815924 | 0.414544 | -0.04855 | 0.11779 | EUR | Rotator cuff (caps | injuries & poisonings |
| 499 | 0.148523 | 0.182106 | 0.815587 | 0.414736 | -0.2084 | 0.505443 | EUR | Cystic fibrosis | respiratory |
| 512.1 | 0.030145 | 0.036966 | 0.815479 | 0.414798 | -0.04231 | 0.102596 | EUR | Wheezing | respiratory |
| 759.1 | 0.236093 | 0.28975 | 0.814817 | 0.415177 | -0.33181 | 0.803992 | EUR | Anomalies of enc | congenital anomalies |
| 172.11 | 0.03233 | 0.039774 | 0.812839 | 0.41631 | -0.04563 | 0.110286 | EUR | Melanomas of sk | neoplasms |
| 614.31 | -0.18581 | 0.228678 | -0.81252 | 0.416493 | -0.63401 | 0.262395 | EUR | Acute inflammato | genitourinary |
| 174.1 | 0.026887 | 0.033153 | 0.811009 | 0.41736 | -0.03809 | 0.091866 | EUR | Breast cancer [fe | neoplasms |
| 579 | 0.02164 | 0.026696 | 0.810623 | 0.417582 | -0.03068 | 0.073963 | EUR | Other symptoms | digestive |
| 599.9 | 0.024243 | 0.030028 | 0.80735 | 0.419465 | -0.03461 | 0.083098 | EUR | Other abnormalit | genitourinary |
| 798 | -0.01369 | 0.016987 | -0.8058 | 0.420356 | -0.04698 | 0.019606 | EUR | Malaise and fatig | symptoms |
| 695.42 | 0.048942 | 0.060808 | 0.804865 | 0.420898 | -0.07024 | 0.168124 | EUR | Systemic lupus e | dermatologic |
| 530.15 | -0.08168 | 0.101547 | -0.80431 | 0.421215 | -0.28071 | 0.117353 | EUR | Eosinophilic esop | digestive |
| 931 | 0.108835 | 0.136168 | 0.799273 | 0.424132 | -0.15805 | 0.375719 | EUR | Contact dermatiti | dermatologic |
| 474.1 | -0.0797 | 0.099849 | -0.7982 | 0.424755 | -0.2754 | 0.116001 | EUR | Acute tonsillitis | respiratory |
| 443.1 | -0.03928 | 0.049251 | -0.79749 | 0.425164 | -0.13581 | 0.057252 | EUR | Raynaud's syndr | circulatory system |
| 594.1 | 0.021162 | 0.02665 | 0.794062 | 0.427159 | -0.03107 | 0.073396 | EUR | Calculus of kidne | genitourinary |
| 721.8 | 0.044452 | 0.056007 | 0.793678 | 0.427383 | -0.06532 | 0.154223 | EUR | Other allied disor | musculoskeletal |
| 255.13 | 0.387339 | 0.488207 | 0.793391 | 0.42755 | -0.56953 | 1.344207 | EUR | Medulloadrenal h | endocrine/metabolic |
| 790 | -0.02064 | 0.026067 | -0.79191 | 0.428412 | -0.07173 | 0.030448 | EUR | Nonspecific findi | symptoms |

| phecode | Coef. | Std.Err. | z | p_value_z | [0.025 | 0.975] | GIA | phenotype | category |
|---|---|---|---|---|---|---|---|---|---|
| 229 | -0.04524 | 0.057521 | -0.78647 | 0.431591 | -0.15798 | 0.067501 | EUR | Benign neoplasm | neoplasms |
| 191 | 0.040503 | 0.051766 | 0.782408 | 0.433975 | -0.06096 | 0.141963 | EUR | Manlignant and u | neoplasms |
| 426.8 | -0.06501 | 0.083126 | -0.78203 | 0.434196 | -0.22793 | 0.097916 | EUR | Other cardiac co | circulatory system |
| 801.1 | -0.04891 | 0.06256 | -0.78182 | 0.434318 | -0.17153 | 0.073705 | EUR | Fracture of foot | injuries & poisonings |
| 624.1 | 0.109166 | 0.139681 | 0.78154 | 0.434485 | -0.1646 | 0.382936 | EUR | Dystrophy of fem | genitourinary |
| 614.4 | -0.12417 | 0.159201 | -0.77993 | 0.43543 | -0.43619 | 0.187862 | EUR | Inflammatory dis | genitourinary |
| 185 | -0.02727 | 0.034975 | -0.77977 | 0.435529 | -0.09582 | 0.041278 | EUR | Cancer of prosta | neoplasms |
| 255.2 | 0.041748 | 0.053592 | 0.779002 | 0.435979 | -0.06329 | 0.146787 | EUR | Adrenal hypofun | endocrine/metabolic |
| 255.21 | 0.041748 | 0.053592 | 0.779002 | 0.435979 | -0.06329 | 0.146787 | EUR | Glucocorticoid de | endocrine/metabolic |
| 269 | -0.02839 | 0.036447 | -0.7788 | 0.436099 | -0.09982 | 0.043051 | EUR | Proteinuria | endocrine/metabolic |
| 174.3 | -0.13618 | 0.175197 | -0.77727 | 0.436999 | -0.47956 | 0.207204 | EUR | Neoplasm of unc | neoplasms |
| 198.6 | 0.034289 | 0.044116 | 0.777244 | 0.437015 | -0.05218 | 0.120753 | EUR | Secondary malig | neoplasms |
| 465.2 | -0.01873 | 0.024131 | -0.77624 | 0.437607 | -0.06603 | 0.028564 | EUR | Acute pharyngitis | respiratory |
| 696.2 | 0.18219 | 0.234712 | 0.776227 | 0.437615 | -0.27784 | 0.642216 | EUR | Parapsoriasis | dermatologic |
| 288.1 | -0.01914 | 0.02469 | -0.77514 | 0.438259 | -0.06753 | 0.029254 | EUR | Decreased white | hematopoietic |
| 599.2 | -0.01966 | 0.025364 | -0.77506 | 0.438303 | -0.06937 | 0.030054 | EUR | Retention of urin | genitourinary |
| 627.21 | -0.12487 | 0.161498 | -0.77323 | 0.439388 | -0.44141 | 0.191656 | EUR | Symptomatic arti | genitourinary |
| 260 | 0.022931 | 0.029656 | 0.773215 | 0.439395 | -0.03519 | 0.081056 | EUR | Protein-calorie m | endocrine/metabolic |
| 323.2 | 0.175939 | 0.228042 | 0.771519 | 0.440399 | -0.27102 | 0.622892 | EUR | Acute (transverse | neurological |
| 401.22 | 0.021869 | 0.028386 | 0.770407 | 0.441059 | -0.03377 | 0.077505 | EUR | Hypertensive chr | circulatory system |
| 420 | 0.026579 | 0.03453 | 0.769745 | 0.441451 | -0.0411 | 0.094257 | EUR | Carditis | circulatory system |
| 453 | 0.053536 | 0.069953 | 0.765305 | 0.44409 | -0.08357 | 0.190642 | EUR | Chronic venous h | circulatory system |
| 344 | -0.05632 | 0.073689 | -0.76427 | 0.444704 | -0.20075 | 0.088109 | EUR | Other paralytic sy | neurological |
| 368.7 | 0.659196 | 0.86264 | 0.764161 | 0.444772 | -1.03155 | 2.349939 | EUR | Disorders of acc | sense organs |
| 695.4 | 0.045699 | 0.06 | 0.761642 | 0.446274 | -0.0719 | 0.163297 | EUR | Lupus (localized | dermatologic |
| 550.3 | -0.09974 | 0.130985 | -0.76143 | 4.46E-01 | -0.35646 | 0.15699 | EUR | Femoral hernia | digestive |
| 379.5 | -0.06677 | 0.087736 | -0.76105 | 0.446625 | -0.23873 | 0.105188 | EUR | Disorders of iris a | sense organs |
| 384 | -0.06189 | 0.081352 | -0.76072 | 0.446824 | -0.22133 | 0.09756 | EUR | Other disorders c | sense organs |
| 747.11 | 0.035734 | 0.046993 | 0.760412 | 0.447008 | -0.05637 | 0.127839 | EUR | Cardiac shunt/ he | congenital anomalies |
| 575.8 | 0.03644 | 0.047991 | 0.759311 | 0.447666 | -0.05762 | 0.130502 | EUR | Other disorders c | digestive |
| 752.11 | -0.13017 | 0.171908 | -0.75721 | 0.448924 | -0.4671 | 0.206763 | EUR | Spina bifida | congenital anomalies |
| 151 | 0.068319 | 0.090368 | 0.756006 | 0.449645 | -0.1088 | 0.245437 | EUR | Cancer of stoma | neoplasms |
| 250.41 | -0.02222 | 0.029398 | -0.75576 | 0.449792 | -0.07984 | 0.035401 | EUR | Impaired fasting | endocrine/metabolic |
| 430 | 0.040732 | 0.053939 | 0.755149 | 0.45016 | -0.06499 | 0.14645 | EUR | Intracranial hemo | circulatory system |
| 362.27 | -0.04709 | 0.06246 | -0.75389 | 0.450915 | -0.16951 | 0.075331 | EUR | Drusen (degener | sense organs |
| 1100 | -0.04448 | 0.059086 | -0.75272 | 0.451616 | -0.16028 | 0.071332 | EUR | Family history | |
| 535.1 | 0.043838 | 0.058254 | 0.752523 | 0.451737 | -0.07034 | 0.158014 | EUR | Acute gastritis | digestive |

| phecode | Coef. | Std.Err. | z | p_value_z | [0.025 | 0.975] | GIA | phenotype | category |
|---|---|---|---|---|---|---|---|---|---|
| 743.2 | 0.032214 | 0.043009 | 0.749002 | 0.453856 | -0.05208 | 0.116509 | EUR | Pathologic fractu | musculoskeletal |
| 780 | -0.04481 | 0.059834 | -0.74886 | 0.453944 | -0.16208 | 0.072466 | EUR | Hypothermia/Chi | symptoms |
| 573.4 | 0.060348 | 0.080741 | 0.747436 | 0.4548 | -0.0979 | 0.218597 | EUR | Acute and subac | digestive |
| 195 | 0.015064 | 0.020161 | 0.74718 | 0.454955 | -0.02445 | 0.054578 | EUR | Cancer, suspecte | neoplasms |
| 586 | 0.015666 | 0.020969 | 0.747119 | 0.454991 | -0.02543 | 0.056764 | EUR | Other disorders c | genitourinary |
| 286.9 | 0.032755 | 0.044082 | 0.743061 | 0.457445 | -0.05364 | 0.119154 | EUR | Abnormal coagul | hematopoietic |
| 444 | 0.041799 | 0.056323 | 0.742132 | 0.458007 | -0.06859 | 0.152189 | EUR | Arterial embolism | circulatory system |
| 333.2 | -0.07475 | 0.10077 | -0.74175 | 0.458238 | -0.27225 | 0.122759 | EUR | Myoclonus | neurological |
| 984 | 0.183085 | 0.246939 | 0.74142 | 0.458439 | -0.30091 | 0.667076 | EUR | Toxic effect of lea | injuries & poisonings |
| 353.2 | 0.113861 | 0.153781 | 0.740412 | 0.45905 | -0.18754 | 0.415266 | EUR | Nerve root lesion | neurological |
| 300.8 | -0.06214 | 0.084006 | -0.73977 | 0.459441 | -0.22679 | 0.102504 | EUR | Acute reaction to | mental disorders |
| 597 | -0.04171 | 0.05645 | -0.73888 | 0.459983 | -0.15235 | 0.068931 | EUR | Other disorders c | genitourinary |
| 245.2 | -0.02724 | 0.037009 | -0.73608 | 0.461683 | -0.09978 | 0.045295 | EUR | Chronic thyroiditi | endocrine/metabolic |
| 275.5 | -0.02289 | 0.031224 | -0.73317 | 0.463456 | -0.08409 | 0.038306 | EUR | Disorders of calc | endocrine/metabolic |
| 253.4 | -0.08535 | 0.116583 | -0.73207 | 0.464127 | -0.31385 | 0.143152 | EUR | Anterior pituitary | endocrine/metabolic |
| 568.1 | 0.027196 | 0.037208 | 0.730922 | 0.464827 | -0.04573 | 0.100123 | EUR | Peritoneal adhes | digestive |
| 159 | -0.0367 | 0.05029 | -0.72973 | 0.465553 | -0.13527 | 0.061868 | EUR | Malignant neopla | neoplasms |
| 749.2 | 0.148837 | 0.204031 | 0.729483 | 0.465706 | -0.25106 | 0.54873 | EUR | Congenital anom | congenital anomalies |
| 477 | 0.030051 | 0.041229 | 0.728872 | 0.46608 | -0.05076 | 0.110858 | EUR | Epistaxis or throa | respiratory |
| 687.3 | -0.04302 | 0.059123 | -0.72756 | 0.466883 | -0.15889 | 0.072863 | EUR | Changes in skin t | dermatologic |
| 360 | -0.06677 | 0.091835 | -0.72704 | 0.467199 | -0.24676 | 0.113225 | EUR | Disorders of the g | sense organs |
| 426.23 | -0.06202 | 0.085564 | -0.72481 | 0.468568 | -0.22972 | 0.105684 | EUR | Second degree A | circulatory system |
| 722.7 | -0.05939 | 0.082009 | -0.72424 | 0.468916 | -0.22013 | 0.10134 | EUR | Intervertebral dis | musculoskeletal |
| 687.2 | 0.018231 | 0.025173 | 0.724221 | 0.46893 | -0.03111 | 0.067569 | EUR | Localized superfi | dermatologic |
| 601.3 | 0.065993 | 0.091223 | 0.723423 | 0.46942 | -0.1128 | 0.244786 | EUR | Orchitis and epid | genitourinary |
| 772.6 | 0.053392 | 0.073923 | 0.722265 | 0.470131 | -0.09149 | 0.198277 | EUR | Facial weakness | symptoms |
| 195.1 | 0.015154 | 0.021031 | 0.720522 | 0.471203 | -0.02607 | 0.056375 | EUR | Malignant neopla | neoplasms |
| 585.4 | 0.023652 | 0.032827 | 0.720511 | 0.47121 | -0.04069 | 0.087991 | EUR | Chronic kidney d | genitourinary |
| 598.4 | 0.098997 | 0.138095 | 0.716872 | 0.473453 | -0.17167 | 0.369659 | EUR | Other cells and c | genitourinary |
| 253.7 | 0.044161 | 0.061611 | 0.716771 | 0.473516 | -0.07659 | 0.164915 | EUR | Other disorders c | endocrine/metabolic |
| 333.3 | 0.191228 | 0.267001 | 0.716206 | 0.473864 | -0.33208 | 0.71454 | EUR | Tics and choreas | neurological |
| 259.1 | -0.28533 | 0.400559 | -0.71232 | 0.476267 | -1.07041 | 0.499755 | EUR | Nonspecific abnc | endocrine/metabolic |
| 749 | -0.05783 | 0.081308 | -0.71122 | 0.47695 | -0.21719 | 0.101533 | EUR | Congenital anom | congenital anomalies |
| 636.1 | 0.103453 | 0.145474 | 0.711145 | 0.476995 | -0.18167 | 0.388576 | EUR | Threatened prem | pregnancy complications |
| 938.1 | 0.059436 | 0.08377 | 0.709517 | 0.478004 | -0.10475 | 0.223621 | EUR | Acute dermatitis | dermatologic |
| 804 | 0.031919 | 0.045321 | 0.70429 | 0.481252 | -0.05691 | 0.120747 | EUR | Fracture of hand | injuries & poisonings |
| 585.34 | 0.0339 | 0.048287 | 0.702046 | 0.48265 | -0.06074 | 0.12854 | EUR | Chronic Kidney D | genitourinary |

| phecode | Coef. | Std.Err. | z | p_value_z | [0.025 | 0.975] | GIA | phenotype | category |
|---|---|---|---|---|---|---|---|---|---|
| 245 | -0.02473 | 0.035247 | -0.70172 | 0.482852 | -0.09382 | 0.04435 | EUR | Thyroiditis | endocrine/metabolic |
| 201 | 0.066954 | 0.095455 | 0.701415 | 0.483044 | -0.12014 | 0.254043 | EUR | Hodgkin's diseas | neoplasms |
| 366.1 | -0.09631 | 0.137912 | -0.69832 | 0.484976 | -0.36661 | 0.173996 | EUR | Nonsenile Catara | sense organs |
| 527.7 | 0.031932 | 0.045831 | 0.696729 | 0.485972 | -0.0579 | 0.12176 | EUR | Disturbance of sa | digestive |
| 389.3 | 0.063474 | 0.09152 | 0.693557 | 0.48796 | -0.1159 | 0.242851 | EUR | Degenerative and | sense organs |
| 204.11 | -0.09491 | 0.136894 | -0.69335 | 0.488093 | -0.36322 | 0.173393 | EUR | Lymphoid leukem | neoplasms |
| 333.4 | -0.05258 | 0.076316 | -0.68904 | 0.4908 | -0.20216 | 0.096992 | EUR | Torsion dystonia | neurological |
| 426.24 | -0.04182 | 0.060919 | -0.68645 | 0.492427 | -0.16122 | 0.077582 | EUR | Atrioventricular b | circulatory system |
| 755.6 | 0.076909 | 0.112265 | 0.685066 | 0.493303 | -0.14313 | 0.296945 | EUR | Other congenital | congenital anomalies |
| 280 | -0.01627 | 0.023803 | -0.68365 | 0.494194 | -0.06292 | 0.030379 | EUR | Iron deficiency ar | hematopoietic |
| 362.21 | 0.040554 | 0.059332 | 0.683509 | 0.494285 | -0.07573 | 0.156843 | EUR | Macular degener | sense organs |
| 747.1 | -0.0237 | 0.034677 | -0.68337 | 0.494373 | -0.09166 | 0.044268 | EUR | Cardiac congenit | congenital anomalies |
| 337 | 0.040987 | 0.060109 | 0.681872 | 0.49532 | -0.07683 | 0.158799 | EUR | Disorders of the a | neurological |
| 743.21 | -0.04973 | 0.073058 | -0.68066 | 0.496089 | -0.19292 | 0.093463 | EUR | Pathologic fractu | musculoskeletal |
| 578 | -0.01595 | 0.023514 | -0.67813 | 0.497691 | -0.06203 | 0.030141 | EUR | Gastrointestinal h | digestive |
| 277 | -0.01879 | 0.027774 | -0.6765 | 0.498723 | -0.07322 | 0.035646 | EUR | Other disorders c | endocrine/metabolic |
| 531.5 | 0.091859 | 0.135918 | 0.675841 | 0.499142 | -0.17454 | 0.358252 | EUR | Gastrojejunal ulc | digestive |
| 600 | -0.01859 | 0.027593 | -0.6737 | 0.500501 | -0.07267 | 0.035492 | EUR | Hyperplasia of pr | genitourinary |
| 594.2 | -0.04878 | 0.07267 | -0.67123 | 0.502072 | -0.19121 | 0.093653 | EUR | Calculus of lower | genitourinary |
| 246 | -0.01846 | 0.027524 | -0.67061 | 0.502471 | -0.0724 | 0.035489 | EUR | Other disorders c | endocrine/metabolic |
| 132 | 0.065809 | 0.098294 | 0.66951 | 0.50317 | -0.12684 | 0.258462 | EUR | Infestation (lice, r | infectious diseases |
| 251 | 0.148465 | 0.221791 | 0.669392 | 0.503245 | -0.28624 | 0.583166 | EUR | Other disorders c | endocrine/metabolic |
| 529 | 0.035091 | 0.052643 | 0.666595 | 0.505031 | -0.06809 | 0.138269 | EUR | Diseases and oth | digestive |
| 594.3 | -0.03909 | 0.058671 | -0.66629 | 0.505227 | -0.15408 | 0.075901 | EUR | Calculus of urete | genitourinary |
| 523 | 0.038613 | 0.057981 | 0.665949 | 0.505444 | -0.07503 | 0.152254 | EUR | Gingival and peri | digestive |
| 614.32 | 0.106093 | 0.160067 | 0.662805 | 0.507455 | -0.20763 | 0.419818 | EUR | Chronic inflamma | genitourinary |
| 497 | 0.02078 | 0.031382 | 0.66214 | 0.507882 | -0.04073 | 0.082288 | EUR | Bronchitis | respiratory |
| 279.7 | 0.018627 | 0.028142 | 0.661887 | 0.508043 | -0.03653 | 0.073785 | EUR | Other immunolog | endocrine/metabolic |
| 594.8 | -0.05463 | 0.082814 | -0.65973 | 0.509429 | -0.21695 | 0.107677 | EUR | Renal colic | genitourinary |
| 628 | -0.02643 | 0.040087 | -0.65933 | 0.509683 | -0.105 | 0.052139 | EUR | Ovarian cyst | genitourinary |
| 579.2 | 0.027411 | 0.041651 | 0.658118 | 0.510462 | -0.05422 | 0.109046 | EUR | Splenomegaly | digestive |
| 264 | 0.065927 | 0.100294 | 0.657336 | 0.510965 | -0.13065 | 0.262498 | EUR | Lack of normal pl | endocrine/metabolic |
| 807 | -0.04175 | 0.063625 | -0.65625 | 0.511665 | -0.16646 | 0.082949 | EUR | Fracture of ribs | injuries & poisonings |
| 697 | 0.05837 | 0.089101 | 0.655105 | 0.5124 | -0.11626 | 0.233004 | EUR | Sarcoidosis | dermatologic |
| 350.1 | -0.0173 | 0.026419 | -0.65474 | 0.512635 | -0.06908 | 0.034483 | EUR | Abnormal involur | neurological |
| 726.3 | -0.02532 | 0.038727 | -0.65371 | 0.5133 | -0.10122 | 0.050588 | EUR | Bursitis | musculoskeletal |
| 525.1 | 0.089314 | 0.136874 | 0.652528 | 0.51406 | -0.17895 | 0.357583 | EUR | Loss of teeth or e | digestive |

| phecode | Coef. | Std.Err. | z | p_value_z | [0.025 | 0.975] | GIA | phenotype | category |
|---|---|---|---|---|---|---|---|---|---|
| 433.11 | -0.04599 | 0.070557 | -0.65178 | 0.514545 | -0.18428 | 0.092302 | EUR | Occlusion of cere | circulatory system |
| 253 | -0.02672 | 0.040998 | -0.65172 | 0.514584 | -0.10707 | 0.053636 | EUR | Disorders of the p | endocrine/metabolic |
| 483 | 0.020075 | 0.030882 | 0.650046 | 0.515663 | -0.04045 | 0.080603 | EUR | Acute bronchitis a | respiratory |
| 286.12 | -0.10821 | 0.166653 | -0.64933 | 0.516126 | -0.43485 | 0.218422 | EUR | Congenital defici | hematopoietic |
| 279.8 | 0.055824 | 0.085987 | 0.649219 | 0.516197 | -0.11271 | 0.224356 | EUR | Other specified d | endocrine/metabolic |
| 800.1 | 0.035004 | 0.054015 | 0.648046 | 0.516955 | -0.07086 | 0.140871 | EUR | Fracture of neck | injuries & poisonings |
| 172.21 | -0.01658 | 0.025623 | -0.64699 | 0.51764 | -0.0668 | 0.033643 | EUR | Basal cell carcinc | neoplasms |
| 695.3 | -0.02293 | 0.035468 | -0.6464 | 0.518019 | -0.09244 | 0.046589 | EUR | Rosacea | dermatologic |
| 741.1 | -0.10517 | 0.162729 | -0.64628 | 0.518101 | -0.42411 | 0.213776 | EUR | Ankylosis of joint | musculoskeletal |
| 751 | 0.025252 | 0.039287 | 0.642774 | 0.520371 | -0.05175 | 0.102253 | EUR | Genitourinary co | congenital anomalies |
| 53.1 | -0.04772 | 0.074583 | -0.63984 | 0.522274 | -0.1939 | 0.098458 | EUR | Herpes zoster wi | infectious diseases |
| 985 | 0.113713 | 0.178733 | 0.636218 | 0.524634 | -0.2366 | 0.464024 | EUR | Toxic effect of oth | injuries & poisonings |
| 245.21 | -0.02373 | 0.037305 | -0.63609 | 0.524716 | -0.09684 | 0.049387 | EUR | Chronic lymphoc | endocrine/metabolic |
| 427.3 | -0.01415 | 0.022278 | -0.63528 | 5.25E-01 | -0.05782 | 0.029511 | EUR | Other specified c | circulatory system |
| 654.2 | -0.08345 | 0.131461 | -0.63478 | 0.525569 | -0.34111 | 0.174209 | EUR | Rhesus isoimmu | pregnancy complications |
| 523.3 | 0.054038 | 0.085169 | 0.63448 | 0.525767 | -0.11289 | 0.220965 | EUR | Periodontitis (acu | digestive |
| 211 | 0.02161 | 0.034072 | 0.634235 | 0.525927 | -0.04517 | 0.088389 | EUR | Benign neoplasm | neoplasms |
| 585.2 | -0.03794 | 0.059961 | -0.63269 | 0.526938 | -0.15546 | 0.079585 | EUR | Renal failure NO | genitourinary |
| 381.1 | -0.0237 | 0.037467 | -0.63249 | 0.527064 | -0.09713 | 0.049736 | EUR | Otitis media | sense organs |
| 612.2 | -0.03717 | 0.059136 | -0.62856 | 0.529638 | -0.15308 | 0.078734 | EUR | Hypertrophy of b | genitourinary |
| 281.11 | 0.072849 | 0.115942 | 0.628321 | 0.529794 | -0.15439 | 0.300091 | EUR | Pernicious anem | hematopoietic |
| 574 | 0.01875 | 0.030017 | 0.624643 | 0.532206 | -0.04008 | 0.077582 | EUR | Cholelithiasis and | digestive |
| 204.1 | -0.05206 | 0.083466 | -0.62374 | 0.532797 | -0.21565 | 0.11153 | EUR | Lymphoid leuken | neoplasms |
| 642.1 | -0.06013 | 0.097124 | -0.6191 | 0.535848 | -0.25049 | 0.13023 | EUR | Preeclampsia an | pregnancy complications |
| 728.2 | -0.05348 | 0.086509 | -0.61825 | 0.536411 | -0.22304 | 0.11607 | EUR | Laxity of ligamen | musculoskeletal |
| 626.1 | -0.01992 | 0.032341 | -0.61587 | 0.537981 | -0.08331 | 0.04347 | EUR | Irregular menstru | genitourinary |
| 840.3 | 0.030471 | 0.049541 | 0.615078 | 0.538504 | -0.06663 | 0.12757 | EUR | Joint/ligament sp | injuries & poisonings |
| 773 | -0.01026 | 0.016685 | -0.61497 | 0.538573 | -0.04296 | 0.022441 | EUR | Pain in limb | symptoms |
| 732.7 | -0.18053 | 0.29381 | -0.61444 | 0.538923 | -0.75639 | 0.395327 | EUR | Osteochondritis d | musculoskeletal |
| 859 | 0.027382 | 0.04476 | 0.611751 | 0.540702 | -0.06035 | 0.115111 | EUR | Complication due | injuries & poisonings |
| 747 | 0.018862 | 0.031 | 0.608444 | 0.542893 | -0.0419 | 0.07962 | EUR | Cardiac and circu | congenital anomalies |
| 751.11 | 0.073216 | 0.120442 | 0.607891 | 0.54326 | -0.16285 | 0.309278 | EUR | Congenital anom | congenital anomalies |
| 686.5 | -0.0742 | 0.122159 | -0.60744 | 0.543558 | -0.31363 | 0.165223 | EUR | Pyoderma | dermatologic |
| 260.22 | -0.0743 | 0.122998 | -0.60411 | 0.54577 | -0.31538 | 0.166768 | EUR | Nutritional maras | endocrine/metabolic |
| 70.4 | -0.05366 | 0.089115 | -0.60211 | 0.547104 | -0.22832 | 0.121006 | EUR | Chronic hepatitis | infectious diseases |
| 952 | 0.076428 | 0.127312 | 0.600317 | 0.548295 | -0.1731 | 0.325954 | EUR | Spinal cord injury | injuries & poisonings |
| 303 | 0.029198 | 0.049459 | 0.590358 | 0.55495 | -0.06774 | 0.126135 | EUR | Psychogenic and | mental disorders |

| phecode | Coef. | Std.Err. | z | p_value_z | [0.025 | 0.975] | GIA | phenotype | category |
|---|---|---|---|---|---|---|---|---|---|
| 131 | 0.148653 | 0.252171 | 0.589492 | 0.555531 | -0.34559 | 0.642898 | EUR | Protozoan infecti | infectious diseases |
| 289.5 | -0.04354 | 0.07402 | -0.58817 | 0.55642 | -0.18861 | 0.10154 | EUR | Diseases of splee | hematopoietic |
| 687.4 | -0.01247 | 0.021216 | -0.5878 | 0.556666 | -0.05405 | 0.029112 | EUR | Disturbance of sk | dermatologic |
| 386.1 | 0.052819 | 0.090001 | 0.58687 | 0.557291 | -0.12358 | 0.229217 | EUR | Meniere's diseas | sense organs |
| 172.1 | 0.021968 | 0.037496 | 0.585867 | 0.557965 | -0.05152 | 0.095458 | EUR | Melanomas of sk | neoplasms |
| 172 | -0.01179 | 0.020281 | -0.58156 | 0.560863 | -0.05154 | 0.027955 | EUR | Skin cancer | neoplasms |
| 427.12 | 0.021856 | 0.037619 | 0.580986 | 0.56125 | -0.05188 | 0.095588 | EUR | Paroxysmal vent | circulatory system |
| 288.3 | 0.040394 | 0.069527 | 0.580978 | 0.561256 | -0.09588 | 0.176664 | EUR | Eosinophilia | hematopoietic |
| 972 | 0.05246 | 0.090802 | 0.577743 | 0.563438 | -0.12551 | 0.230429 | EUR | Poisoning by age | injuries & poisonings |
| 394 | -0.01971 | 0.034145 | -0.57732 | 0.563723 | -0.08664 | 0.047211 | EUR | Rheumatic disea | circulatory system |
| 372 | 0.023928 | 0.041455 | 0.577193 | 0.563809 | -0.05732 | 0.105178 | EUR | Disorders of conj | sense organs |
| 741.3 | -0.03492 | 0.060574 | -0.57655 | 0.564246 | -0.15365 | 0.083799 | EUR | Difficulty in walkir | musculoskeletal |
| 189.2 | 0.031636 | 0.054911 | 0.576136 | 0.564523 | -0.07599 | 0.13926 | EUR | Cancer of bladde | neoplasms |
| 614.53 | 0.084823 | 0.147324 | 0.575757 | 0.564779 | -0.20393 | 0.373572 | EUR | Cyst or abscess ( | genitourinary |
| 286.8 | -0.02705 | 0.0472 | -0.57301 | 0.566639 | -0.11956 | 0.065464 | EUR | Hypercoagulable | hematopoietic |
| 429.9 | -0.03772 | 0.065885 | -0.57256 | 0.566941 | -0.16685 | 0.091408 | EUR | Cardiac complica | circulatory system |
| 426.4 | 0.084842 | 0.148334 | 0.571966 | 0.567345 | -0.20589 | 0.375573 | EUR | Anomalous atriov | circulatory system |
| 907 | 0.050444 | 0.088222 | 0.571791 | 0.567464 | -0.12247 | 0.223356 | EUR | Injuries to the ne | injuries & poisonings |
| 696.4 | 0.021203 | 0.037187 | 0.570165 | 0.568565 | -0.05168 | 0.094089 | EUR | Psoriasis | dermatologic |
| 705.8 | 0.022704 | 0.03984 | 0.569887 | 0.568754 | -0.05538 | 0.100789 | EUR | Hyperhidrosis | dermatologic |
| 374.1 | 0.040474 | 0.071085 | 0.569372 | 0.569104 | -0.09885 | 0.179797 | EUR | Ectropion or entr | sense organs |
| 212 | -0.0589 | 0.103582 | -0.56859 | 0.569635 | -0.26191 | 0.144121 | EUR | Benign neoplasm | neoplasms |
| 577.3 | 0.027974 | 0.049336 | 0.567007 | 0.570709 | -0.06872 | 0.12467 | EUR | Cyst and pseudo | digestive |
| 362.5 | 0.184528 | 0.325711 | 0.566538 | 0.571028 | -0.45385 | 0.822909 | EUR | Toxic maculopath | sense organs |
| 795 | 0.034799 | 0.061612 | 0.564811 | 0.572202 | -0.08596 | 0.155556 | EUR | Other and nonsp | symptoms |
| 261.41 | 0.089572 | 0.158741 | 0.564266 | 0.572573 | -0.22155 | 0.400698 | EUR | Rickets or osteor | endocrine/metabolic |
| 528.6 | 0.078149 | 0.138932 | 0.5625 | 0.573776 | -0.19415 | 0.350451 | EUR | Leukoplakia of or | digestive |
| 81 | 0.02773 | 0.049308 | 0.562371 | 0.573864 | -0.06891 | 0.124372 | EUR | Infection/inflamm | infectious diseases |
| 172.22 | -0.01815 | 0.032275 | -0.56227 | 0.573933 | -0.08141 | 0.045111 | EUR | Squamous cell ca | neoplasms |
| 293.1 | -0.01523 | 0.027254 | -0.55871 | 0.576359 | -0.06864 | 0.03819 | EUR | Swelling, mass, c | mental disorders |
| 530.12 | 0.02973 | 0.053449 | 0.556243 | 0.578044 | -0.07503 | 0.134488 | EUR | Ulcer of esophag | digestive |
| 646 | -0.02706 | 0.048676 | -0.55599 | 0.578217 | -0.12247 | 0.06834 | EUR | Other complicatic | pregnancy complications |
| 240 | -0.02989 | 0.053761 | -0.55595 | 0.578247 | -0.13526 | 0.075482 | EUR | Simple and unsp | endocrine/metabolic |
| 41.21 | 0.092414 | 0.166461 | 0.555166 | 0.578781 | -0.23384 | 0.418672 | EUR | Rheumatic fever | infectious diseases |
| 414 | -0.01815 | 0.032791 | -0.55338 | 0.580004 | -0.08241 | 0.046123 | EUR | Other forms of ch | circulatory system |
| 242.3 | -0.05367 | 0.097166 | -0.5524 | 0.580677 | -0.24411 | 0.136767 | EUR | Exophthalmos | endocrine/metabolic |
| 674 | -0.06476 | 0.11727 | -0.55221 | 0.580806 | -0.2946 | 0.165087 | EUR | Other complicatic | pregnancy complications |

| phecode | Coef. | Std.Err. | z | p_value_z | [0.025 | 0.975] | GIA | phenotype | category | |
|---|---|---|---|---|---|---|---|---|---|---|
| 174.2 | 0.077564 | 0.141329 | 0.54882 | 0.583129 | -0.19944 | 0.354563 | EUR | Breast cancer [m | neoplasms | |
| 623 | -0.03027 | 0.055228 | -0.54814 | 0.583595 | -0.13852 | 0.077972 | EUR | Hypertrophy of fe | genitourinary | |
| 516 | 0.028456 | 0.051924 | 0.548037 | 0.583667 | -0.07331 | 0.130226 | EUR | Abnormal sputun | respiratory | |
| 394.7 | -0.03151 | 0.057534 | -0.54761 | 0.583961 | -0.14427 | 0.081259 | EUR | Disease of tricus | circulatory system | |
| 414.2 | -0.04931 | 0.090208 | -0.54665 | 0.584619 | -0.22612 | 0.127492 | EUR | ASCVD | circulatory system | |
| 282 | -0.02913 | 0.053303 | -0.54652 | 0.584709 | -0.1336 | 0.07534 | EUR | Hereditary hemo | hematopoietic | |
| 701.4 | 0.027057 | 0.049525 | 0.546323 | 0.584844 | -0.07001 | 0.124123 | EUR | Keloid scar | dermatologic | |
| 382 | -0.01641 | 0.030123 | -0.54483 | 0.585867 | -0.07545 | 0.042628 | EUR | Otalgia | sense organs | |
| 560.4 | 0.022521 | 0.041508 | 0.542574 | 0.587423 | -0.05883 | 0.103875 | EUR | Other intestinal o | digestive | |
| 446 | -0.03665 | 0.067686 | -0.54149 | 0.588171 | -0.16931 | 0.09601 | EUR | Polyarteritis nodo | circulatory system | |
| 727.7 | 0.064497 | 0.119121 | 0.541441 | 0.588203 | -0.16898 | 0.29797 | EUR | Contracture of te | musculoskeletal | |
| 442.8 | 0.042964 | 0.079382 | 0.541238 | 0.588343 | -0.11262 | 0.19855 | EUR | Aneurysm of othe | circulatory system | |
| 480.11 | 0.026045 | 0.048125 | 0.541188 | 0.588378 | -0.06828 | 0.120367 | EUR | Pneumococcal p | respiratory | |
| 300.13 | 0.031326 | 0.05799 | 0.54019 | 0.589066 | -0.08233 | 0.144984 | EUR | Phobia | mental disorders | |
| 555.2 | 0.023436 | 0.043594 | 0.537611 | 0.590846 | -0.06201 | 0.108878 | EUR | Ulcerative colitis | digestive | |
| 202.21 | -0.05778 | 0.107679 | -0.5366 | 0.591541 | -0.26883 | 0.153266 | EUR | Nodular lymphon | neoplasms | |
| 8.7 | 0.089574 | 0.167543 | 0.534634 | 0.592903 | -0.2388 | 0.417953 | EUR | Intestinal infectio | infectious diseases | |
| 750.5 | -0.28695 | 0.538932 | -0.53244 | 0.594418 | -1.34324 | 0.769336 | EUR | Congenital hyper | congenital anomalies | |
| 333.1 | 0.026473 | 0.049978 | 0.529683 | 5.96E-01 | -0.07148 | 0.124428 | EUR | Essential tremor | neurological | |
| 819 | -0.02458 | 0.046413 | -0.5296 | 0.596386 | -0.11555 | 0.066388 | EUR | Skull and face fra | injuries & poisonings | |
| 689 | -0.01005 | 0.019009 | -0.52877 | 0.596967 | -0.04731 | 0.027206 | EUR | Disorder of skin a | dermatologic | |
| 153.2 | -0.02352 | 0.044508 | -0.52854 | 0.597125 | -0.11076 | 0.06371 | EUR | Colon cancer | neoplasms | |
| 711.1 | 0.049373 | 0.093864 | 0.526007 | 0.598884 | -0.1346 | 0.233342 | EUR | Pyogenic arthritis | musculoskeletal | |
| 569.2 | 0.024199 | 0.046034 | 0.525686 | 0.599106 | -0.06603 | 0.114424 | EUR | Gastrointestinal c | digestive | |
| 227.2 | 0.034462 | 0.065762 | 0.524041 | 0.60025 | -0.09443 | 0.163354 | EUR | Benign neoplasm | neoplasms | |
| 303.31 | -0.03827 | 0.073031 | -0.52396 | 0.600308 | -0.1814 | 0.104873 | EUR | Gastrointestinal r | mental disorders | |
| 425.11 | 0.063449 | 0.121333 | 0.52293 | 0.601023 | -0.17436 | 0.301257 | EUR | Hypertrophic obs | circulatory system | |
| 561 | 0.008923 | 0.017083 | 0.522303 | 0.60146 | -0.02456 | 0.042405 | EUR | Symptoms involv | digestive | |
| 242.31 | 0.108553 | 0.209484 | 0.518191 | 0.604325 | -0.30203 | 0.519134 | EUR | Thyrotoxic exoph | endocrine/metabolic | |
| 345.12 | 0.036045 | 0.069581 | 0.518031 | 0.604436 | -0.10033 | 0.172422 | EUR | Partial epilepsy | neurological | |
| 110.2 | -0.0318 | 0.0614 | -0.51788 | 0.604542 | -0.15214 | 0.088544 | EUR | Dermatomycoses | infectious diseases | |
| 809 | 0.009015 | 0.017482 | 0.515677 | 0.60608 | -0.02525 | 0.043278 | EUR | Fracture of unspe | injuries & poisonings | |
| 642 | -0.03461 | 0.067146 | -0.51545 | 0.606239 | -0.16621 | 0.096994 | EUR | Hypertension cor | pregnancy complications | |
| 735.2 | -0.02074 | 0.040373 | -0.51368 | 0.607472 | -0.09987 | 0.058391 | EUR | Acquired toe defo | musculoskeletal | |
| 526 | -0.01872 | 0.036441 | -0.51362 | 0.607518 | -0.09014 | 0.052707 | EUR | Diseases of the ja | digestive | |
| 303.3 | -0.02839 | 0.055326 | -0.51315 | 0.607846 | -0.13683 | 0.080047 | EUR | Psychogenic disc | mental disorders | |
| 170.2 | 0.027667 | 0.053984 | 0.512501 | 0.6083 | -0.07814 | 0.133473 | EUR | Cancer of conne | neoplasms | |

| phecode | Coef. | Std.Err. | z | p_value_z | [0.025 | 0.975] | GIA | phenotype | category |
|---|---|---|---|---|---|---|---|---|---|
| 575.9 | -0.03352 | 0.065432 | -0.51224 | 6.08E-01 | -0.16176 | 0.094727 | EUR | Nonspecific abnc | digestive |
| 198.3 | -0.02666 | 0.05242 | -0.50864 | 6.11E-01 | -0.1294 | 0.076078 | EUR | Secondary malig | neoplasms |
| 593.2 | -0.01711 | 0.033661 | -0.50821 | 0.611308 | -0.08308 | 0.048868 | EUR | Microscopic hem | genitourinary |
| 716.2 | 0.085239 | 0.167836 | 0.507872 | 0.611543 | -0.24371 | 0.414191 | EUR | Unspecified mon | musculoskeletal |
| 795.82 | 0.06265 | 0.123387 | 0.507752 | 0.611628 | -0.17918 | 0.304485 | EUR | Elevated cancer | symptoms |
| 112 | 0.014856 | 0.029264 | 0.507635 | 0.611709 | -0.0425 | 0.072212 | EUR | Candidiasis | infectious diseases |
| 278.3 | 0.054337 | 0.107849 | 0.503824 | 0.614385 | -0.15704 | 0.265718 | EUR | Localized adipos | endocrine/metabolic |
| 134.1 | -0.09198 | 0.183194 | -0.50212 | 0.615586 | -0.45104 | 0.26707 | EUR | Intestinal helmint | infectious diseases |
| 870 | 0.013976 | 0.027854 | 0.501778 | 0.615824 | -0.04062 | 0.068569 | EUR | Open wounds of | injuries & poisonings |
| 961 | 0.02939 | 0.058684 | 0.500824 | 0.616495 | -0.08563 | 0.144408 | EUR | Poisoning by oth | injuries & poisonings |
| 346 | 0.053891 | 0.107664 | 0.500551 | 0.616687 | -0.15713 | 0.264909 | EUR | Abnormal finding | neurological |
| 290.16 | -0.04814 | 0.096222 | -0.50028 | 0.616876 | -0.23673 | 0.140453 | EUR | Vascular dement | mental disorders |
| 280.1 | -0.0127 | 0.025443 | -0.49911 | 0.617698 | -0.06257 | 0.037168 | EUR | Iron deficiency ar | hematopoietic |
| 622 | 0.022502 | 0.045242 | 0.497361 | 0.618935 | -0.06617 | 0.111174 | EUR | Polyp of female g | genitourinary |
| 958.1 | 0.096685 | 0.194543 | 0.496986 | 0.619199 | -0.28461 | 0.477982 | EUR | Postoperative sh | injuries & poisonings |
| 798.1 | 0.017596 | 0.035611 | 0.494129 | 0.621215 | -0.0522 | 0.087392 | EUR | Chronic fatigue s | symptoms |
| 967 | 0.028268 | 0.057452 | 0.492022 | 0.622704 | -0.08434 | 0.140872 | EUR | Adverse effects c | injuries & poisonings |
| 564 | -0.00911 | 0.018833 | -0.48379 | 0.628534 | -0.04602 | 0.027801 | EUR | Functional digest | digestive |
| 658 | 0.072216 | 0.15053 | 0.479747 | 0.631407 | -0.22282 | 0.367249 | EUR | Maternal complic | pregnancy complications |
| 345 | 0.017722 | 0.036977 | 0.479268 | 0.631748 | -0.05475 | 0.090195 | EUR | Epilepsy, recurre | neurological |
| 362.8 | -0.05341 | 0.11177 | -0.4779 | 6.33E-01 | -0.27248 | 0.16565 | EUR | Retinal hemorrha | sense organs |
| 707.2 | 0.022976 | 0.048185 | 0.47684 | 0.633476 | -0.07146 | 0.117417 | EUR | Chronic ulcer of l | dermatologic |
| 870.4 | 0.063824 | 0.133973 | 0.476397 | 0.633792 | -0.19876 | 0.326407 | EUR | Open wound of n | injuries & poisonings |
| 727.5 | 0.025038 | 0.052627 | 0.475769 | 0.634239 | -0.07811 | 0.128185 | EUR | Rupture of synov | musculoskeletal |
| 204.2 | 0.043026 | 0.091006 | 0.472778 | 6.36E-01 | -0.13534 | 0.221395 | EUR | Myeloid leukemia | neoplasms |
| 473.3 | -0.02679 | 0.056822 | -0.47154 | 0.637256 | -0.13816 | 0.084576 | EUR | Paralysis/spasm | respiratory |
| 690 | -0.01419 | 0.030106 | -0.47121 | 0.637489 | -0.07319 | 0.04482 | EUR | Erythematosquar | dermatologic |
| 223 | -0.04777 | 0.101469 | -0.47076 | 0.637813 | -0.24664 | 0.151108 | EUR | Benign neoplasm | neoplasms |
| 425.12 | -0.05011 | 0.107171 | -0.46759 | 0.640078 | -0.26016 | 0.159939 | EUR | Other hypertroph | circulatory system |
| 636.8 | -0.10457 | 0.223878 | -0.46707 | 0.640453 | -0.54336 | 0.334227 | EUR | Cervical incompe | pregnancy complications |
| 792 | -0.01902 | 0.040976 | -0.46406 | 0.642607 | -0.09933 | 0.061296 | EUR | Abnormal Papan | genitourinary |
| 214.1 | 0.020695 | 0.04471 | 0.462884 | 0.643448 | -0.06693 | 0.108325 | EUR | Lipoma of skin ar | neoplasms |
| 259.4 | 0.175446 | 0.379777 | 0.461972 | 0.644101 | -0.5689 | 0.919795 | EUR | Precocious sexua | endocrine/metabolic |
| 577 | 0.014561 | 0.031538 | 0.461699 | 6.44E-01 | -0.04725 | 0.076373 | EUR | Diseases of panc | digestive |
| 550 | 0.008931 | 0.019532 | 0.457242 | 0.647497 | -0.02935 | 0.047213 | EUR | Abdominal hernia | digestive |
| 702.4 | -0.08249 | 0.180834 | -0.45614 | 0.648287 | -0.43691 | 0.271941 | EUR | Degenerative ski | dermatologic |
| 362.29 | 0.025218 | 0.055311 | 0.455934 | 0.648437 | -0.08319 | 0.133627 | EUR | Macular degener | sense organs |

| phecode | Coef. | Std.Err. | z | p_value_z | [0.025 | 0.975] | GIA | phenotype | category |
|---|---|---|---|---|---|---|---|---|---|
| 260.7 | -0.07689 | 0.169082 | -0.45477 | 0.649275 | -0.40829 | 0.254502 | EUR | Polyphagia | endocrine/metabolic |
| 748 | 0.061881 | 0.136594 | 0.453026 | 0.65053 | -0.20584 | 0.3296 | EUR | Anomalies of res | congenital anomalies |
| 473.1 | 0.041557 | 0.091762 | 0.452873 | 0.65064 | -0.13829 | 0.221407 | EUR | Chronic laryngitis | respiratory |
| 591 | -0.00913 | 0.020164 | -0.45268 | 0.650781 | -0.04865 | 0.030393 | EUR | Urinary tract infe | genitourinary |
| 716.8 | 0.175631 | 0.389225 | 0.451231 | 0.651823 | -0.58724 | 0.938498 | EUR | Palindromic rheu | musculoskeletal |
| 345.1 | 0.023387 | 0.05214 | 0.448537 | 0.653766 | -0.07881 | 0.125578 | EUR | Epilepsy | neurological |
| 377.1 | -0.03908 | 0.087264 | -0.44788 | 0.654239 | -0.21012 | 0.131951 | EUR | Optic atrophy | sense organs |
| 626.4 | -0.04339 | 0.097212 | -0.44639 | 0.655313 | -0.23393 | 0.147137 | EUR | Premenstrual ten | genitourinary |
| 250.11 | -0.07149 | 0.160338 | -0.44584 | 0.65571 | -0.38574 | 0.242771 | EUR | Type 1 diabetes | endocrine/metabolic |
| 535.6 | -0.03042 | 0.068351 | -0.44509 | 0.656252 | -0.16439 | 0.103542 | EUR | Duodenitis | digestive |
| 447 | 0.013618 | 0.030603 | 0.445005 | 0.656316 | -0.04636 | 0.073599 | EUR | Other disorders c | circulatory system |
| 626 | -0.01277 | 0.028864 | -0.44225 | 0.658309 | -0.06934 | 0.043808 | EUR | Disorders of men | genitourinary |
| 375.2 | -0.02838 | 0.064235 | -0.44187 | 0.658583 | -0.15428 | 0.097514 | EUR | Epiphora | sense organs |
| 471 | 0.025281 | 0.057366 | 0.440707 | 0.659425 | -0.08715 | 0.137716 | EUR | Nasal polyps | respiratory |
| 960.1 | -0.10626 | 0.241719 | -0.43959 | 0.660232 | -0.58002 | 0.367503 | EUR | Adverse effects c | injuries & poisonings |
| 514.1 | 0.028369 | 0.064545 | 0.439527 | 0.660279 | -0.09814 | 0.154874 | EUR | Abnormal results | respiratory |
| 727.4 | -0.01656 | 0.0377 | -0.43932 | 0.66043 | -0.09045 | 0.057328 | EUR | Ganglion and cys | musculoskeletal |
| 371.33 | 0.054834 | 0.125168 | 0.438088 | 0.661323 | -0.19049 | 0.300159 | EUR | Noninfectious de | sense organs |
| 758.1 | 0.041043 | 0.094231 | 0.435556 | 0.663159 | -0.14365 | 0.225732 | EUR | Chromosomal an | congenital anomalies |
| 564.9 | -0.0103 | 0.023705 | -0.43443 | 0.663973 | -0.05676 | 0.036162 | EUR | Personal history | digestive |
| 668 | -0.14428 | 0.332153 | -0.43438 | 0.664014 | -0.79529 | 0.506728 | EUR | Complications of | pregnancy complications |
| 686.2 | -0.02557 | 0.058969 | -0.43366 | 0.664537 | -0.14115 | 0.090005 | EUR | Impetigo | dermatologic |
| 426.2 | -0.01731 | 0.039949 | -0.43336 | 0.664754 | -0.09561 | 0.060987 | EUR | Atrioventricular [/ | circulatory system |
| 726.2 | 0.019136 | 0.044248 | 0.432473 | 0.665398 | -0.06759 | 0.105861 | EUR | Synoviopathy | musculoskeletal |
| 781 | 0.0103 | 0.023904 | 0.430879 | 0.666557 | -0.03655 | 0.057151 | EUR | Symptoms involv | symptoms |
| 377 | -0.02482 | 0.057724 | -0.42992 | 0.667253 | -0.13795 | 0.08832 | EUR | Disorders of optic | sense organs |
| 221 | -0.07699 | 0.179681 | -0.42847 | 0.668306 | -0.42916 | 0.275179 | EUR | Benign neoplasm | neoplasms |
| 227 | 0.018829 | 0.044021 | 0.42773 | 0.668848 | -0.06745 | 0.105109 | EUR | Benign neoplasm | neoplasms |
| 563 | 0.008207 | 0.019268 | 0.425915 | 0.67017 | -0.02956 | 0.045972 | EUR | Constipation | digestive |
| 276.5 | 0.010426 | 0.024515 | 0.425282 | 0.670631 | -0.03762 | 0.058473 | EUR | Hypovolemia | endocrine/metabolic |
| 771 | -0.01181 | 0.027763 | -0.4252 | 0.670692 | -0.06622 | 0.04261 | EUR | Musculoskeletal | symptoms |
| 686.1 | -0.03097 | 0.073139 | -0.42344 | 0.671976 | -0.17432 | 0.112381 | EUR | Carbuncle and fu | dermatologic |
| 275.3 | 0.016649 | 0.039325 | 0.423364 | 0.67203 | -0.06043 | 0.093724 | EUR | Disorders of mag | endocrine/metabolic |
| 275.53 | 0.019081 | 0.045118 | 0.422917 | 0.672356 | -0.06935 | 0.107511 | EUR | Disorders of phos | endocrine/metabolic |
| 574.1 | 0.013297 | 0.031495 | 0.422208 | 0.672873 | -0.04843 | 0.075026 | EUR | Cholelithiasis | digestive |
| 379.9 | -0.01513 | 0.035896 | -0.42158 | 0.673332 | -0.08549 | 0.055222 | EUR | Pain, swelling or | sense organs |
| 691.3 | 0.074244 | 0.176213 | 0.421331 | 0.673514 | -0.27113 | 0.419615 | EUR | Congenital pigme | dermatologic |

| phecode | Coef. | Std.Err. | z | p_value_z | [0.025 | 0.975] | GIA | phenotype | category |
|---|---|---|---|---|---|---|---|---|---|
| 994.1 | -0.02887 | 0.068709 | -0.42019 | 0.674348 | -0.16354 | 0.105796 | EUR | Systemic inflamm | injuries & poisonings |
| 756 | -0.02672 | 0.063598 | -0.42016 | 0.674367 | -0.15137 | 0.097929 | EUR | Other congenital | congenital anomalies |
| 836 | 0.04894 | 0.116641 | 0.41958 | 0.674792 | -0.17967 | 0.277553 | EUR | Traumatic arthro | injuries & poisonings |
| 289.8 | 0.024601 | 0.058644 | 0.419503 | 0.674849 | -0.09034 | 0.13954 | EUR | Polycythemia, se | hematopoietic |
| 313.1 | 0.017248 | 0.041159 | 0.419042 | 0.675185 | -0.06342 | 0.097918 | EUR | Attention deficit h | mental disorders |
| 459.1 | 0.020899 | 0.050021 | 0.41781 | 0.676086 | -0.07714 | 0.118939 | EUR | Hemorrhage NOS | circulatory system |
| 597.2 | -0.02833 | 0.068225 | -0.4153 | 0.67792 | -0.16205 | 0.105384 | EUR | Urinary complica | genitourinary |
| 750.22 | 0.040593 | 0.09788 | 0.414718 | 0.678348 | -0.15125 | 0.232434 | EUR | Congenital anom | congenital anomalies |
| 569.1 | 0.026307 | 0.063494 | 0.414318 | 0.678641 | -0.09814 | 0.150753 | EUR | Toxic gastroenter | digestive |
| 624.9 | 0.017319 | 0.041923 | 0.41311 | 0.679526 | -0.06485 | 0.099485 | EUR | stress incontinen | genitourinary |
| 250.14 | 0.04724 | 0.114456 | 0.412733 | 0.679802 | -0.17709 | 0.271569 | EUR | Type 1 diabetes | endocrine/metabolic |
| 250.4 | 0.007192 | 0.017448 | 0.412221 | 0.680178 | -0.027 | 0.041389 | EUR | Abnormal glucos | endocrine/metabolic |
| 164 | 0.051295 | 0.12546 | 0.408856 | 0.682645 | -0.1946 | 0.297193 | EUR | Cancer of intrath | neoplasms |
| 690.1 | -0.01237 | 0.030367 | -0.4073 | 0.683788 | -0.07189 | 0.04715 | EUR | Seborrheic derm | dermatologic |
| 965 | 0.014368 | 0.035436 | 0.405473 | 0.68513 | -0.05508 | 0.083821 | EUR | Poisoning by ana | injuries & poisonings |
| 803.2 | -0.0213 | 0.052689 | -0.40425 | 0.686028 | -0.12457 | 0.081969 | EUR | Fracture of radius | injuries & poisonings |
| 427.22 | 0.014134 | 0.035108 | 0.402601 | 0.687242 | -0.05468 | 0.082944 | EUR | Atrial flutter | circulatory system |
| 687 | -0.00876 | 0.021777 | -0.40205 | 0.687647 | -0.05144 | 0.033927 | EUR | Symptoms affect | dermatologic |
| 573.1 | -0.04225 | 0.105176 | -0.40168 | 0.687922 | -0.24839 | 0.163895 | EUR | Chronic passive | digestive |
| 379.51 | 0.071563 | 0.179281 | 0.399168 | 0.689769 | -0.27982 | 0.422948 | EUR | Pigmentary iris d | sense organs |
| 574.12 | -0.02745 | 0.068954 | -0.39809 | 0.690565 | -0.1626 | 0.107698 | EUR | Cholelithiasis wit | digestive |
| 295.1 | -0.04358 | 0.109693 | -0.39726 | 0.691176 | -0.25857 | 0.171418 | EUR | Schizophrenia | mental disorders |
| 601.8 | 0.067975 | 0.172336 | 0.394431 | 0.693263 | -0.2698 | 0.405747 | EUR | Other inflammato | genitourinary |
| 657 | 0.196315 | 0.498503 | 0.393808 | 0.693723 | -0.78073 | 1.173363 | EUR | Infections specifi | pregnancy complications |
| 723.1 | -0.03033 | 0.077061 | -0.39363 | 0.693853 | -0.18137 | 0.120703 | EUR | Torticollis | musculoskeletal |
| 751.1 | -0.03774 | 0.096549 | -0.39091 | 0.695864 | -0.22697 | 0.15149 | EUR | Congenital anom | congenital anomalies |
| 383 | -0.05997 | 0.153614 | -0.39038 | 0.696253 | -0.36105 | 0.241109 | EUR | Otosclerosis | sense organs |
| 612.1 | -0.06155 | 0.158221 | -0.38904 | 0.697249 | -0.37166 | 0.248554 | EUR | Galactorrhea | genitourinary |
| 555.1 | 0.019638 | 0.050529 | 0.388645 | 0.697539 | -0.0794 | 0.118674 | EUR | Regional enteritis | digestive |
| 756.2 | -0.04689 | 0.120705 | -0.38849 | 0.697655 | -0.28347 | 0.189685 | EUR | Pectus and other | congenital anomalies |
| 580.11 | 0.065729 | 0.170232 | 0.386115 | 0.699412 | -0.26792 | 0.399377 | EUR | Proliferative glom | genitourinary |
| 550.2 | 0.009084 | 0.02363 | 0.384438 | 0.700654 | -0.03723 | 0.055397 | EUR | Diaphragmatic he | digestive |
| 252.2 | -0.02833 | 0.074031 | -0.38262 | 0.702005 | -0.17342 | 0.116772 | EUR | Hypoparathyroidi | endocrine/metabolic |
| 735.21 | 0.018796 | 0.049206 | 0.381991 | 0.702468 | -0.07765 | 0.115238 | EUR | Hammer toe (acq | musculoskeletal |
| 625 | -0.01134 | 0.029697 | -0.38199 | 0.70247 | -0.06955 | 0.046861 | EUR | Pain and other sy | genitourinary |
| 876 | -0.02506 | 0.065716 | -0.38129 | 0.702987 | -0.15386 | 0.103744 | EUR | Posttraumatic wo | injuries & poisonings |
| 443.8 | -0.02647 | 0.069534 | -0.38069 | 0.703433 | -0.16276 | 0.109813 | EUR | Other specified p | circulatory system |

| phecode | Coef. | Std.Err. | z | p_value_z | [0.025 | 0.975] | GIA | phenotype | category |
|---|---|---|---|---|---|---|---|---|---|
| 614.33 | -0.05981 | 0.157188 | -0.38047 | 0.703594 | -0.36789 | 0.248276 | EUR | Pelvic inflammato | genitourinary |
| 289.9 | 0.018752 | 0.049288 | 0.380458 | 0.703605 | -0.07785 | 0.115356 | EUR | Abnormality of re | hematopoietic |
| 580.1 | -0.03642 | 0.095946 | -0.37957 | 0.704261 | -0.22447 | 0.151632 | EUR | Glomerulonephri | genitourinary |
| 287.4 | 0.056625 | 0.149311 | 0.379244 | 0.704507 | -0.23602 | 0.34927 | EUR | Qualitative platel | hematopoietic |
| 619.2 | 0.019964 | 0.052751 | 0.378464 | 0.705086 | -0.08343 | 0.123354 | EUR | Disorders of uter | genitourinary |
| 263 | -0.01344 | 0.035526 | -0.37826 | 0.705239 | -0.08307 | 0.056191 | EUR | Other nutritional | endocrine/metabolic |
| 327.6 | 0.029081 | 0.077038 | 0.377494 | 0.705806 | -0.12191 | 0.180073 | EUR | Circadian rhythm | neurological |
| 790.1 | -0.0246 | 0.065191 | -0.37734 | 0.705922 | -0.15237 | 0.103172 | EUR | Elevated sedime | symptoms |
| 90.3 | -0.11022 | 0.292438 | -0.37691 | 0.706243 | -0.68339 | 0.462946 | EUR | Venereal disease | infectious diseases |
| 531.3 | -0.02745 | 0.073029 | -0.37594 | 0.706965 | -0.17059 | 0.115679 | EUR | Duodenal ulcer | digestive |
| 433.8 | -0.022 | 0.05873 | -0.37456 | 0.707989 | -0.13711 | 0.093111 | EUR | Late effects of ce | circulatory system |
| 737 | -0.01201 | 0.032072 | -0.37441 | 0.708096 | -0.07487 | 0.050852 | EUR | Curvature of spin | musculoskeletal |
| 626.13 | -0.01538 | 0.041074 | -0.37438 | 0.70812 | -0.09588 | 0.065126 | EUR | Irregular menstru | genitourinary |
| 218.1 | -0.01299 | 0.034798 | -0.37328 | 0.708939 | -0.08119 | 0.055213 | EUR | Uterine leiomyom | neoplasms |
| 452.1 | -0.20428 | 0.547677 | -0.37299 | 0.709157 | -1.27771 | 0.869151 | EUR | Iatrogenic pulmo | circulatory system |
| 854 | 0.018445 | 0.049608 | 0.371825 | 0.710023 | -0.07878 | 0.115674 | EUR | Complications of | injuries & poisonings |
| 751.2 | 0.016007 | 0.04325 | 0.370107 | 0.711303 | -0.06876 | 0.100776 | EUR | Congenital anom | congenital anomalies |
| 478 | 0.016046 | 0.043363 | 0.370038 | 0.711354 | -0.06894 | 0.101036 | EUR | Throat pain | respiratory |
| 187.1 | 0.076623 | 0.207254 | 0.369704 | 0.711603 | -0.32959 | 0.482834 | EUR | Malignant neopla | neoplasms |
| 264.9 | -0.05504 | 0.14923 | -0.36879 | 0.712281 | -0.34752 | 0.23745 | EUR | Lack of normal p | endocrine/metabolic |
| 287.32 | 0.018888 | 0.051246 | 0.368568 | 0.71245 | -0.08155 | 0.119327 | EUR | Secondary throm | hematopoietic |
| 800 | 0.011796 | 0.032396 | 0.364115 | 0.715772 | -0.0517 | 0.075292 | EUR | Fracture of lower | injuries & poisonings |
| 530.7 | 0.065789 | 0.180846 | 0.363783 | 0.71602 | -0.28866 | 0.42024 | EUR | Gastroesophage | digestive |
| 618.5 | -0.04949 | 0.136512 | -0.3625 | 0.716976 | -0.31705 | 0.218073 | EUR | Prolapse of vagir | genitourinary |
| 244.1 | -0.01386 | 0.03843 | -0.36076 | 0.718276 | -0.08919 | 0.061458 | EUR | Secondary hypot | endocrine/metabolic |
| 772.3 | -0.01195 | 0.033444 | -0.35729 | 0.720877 | -0.0775 | 0.0536 | EUR | Muscle weakness | symptoms |
| 367.8 | -0.02105 | 0.05892 | -0.35723 | 0.720917 | -0.13653 | 0.094434 | EUR | Hypermetropia | sense organs |
| 733.6 | 0.022728 | 0.063775 | 0.356368 | 0.721565 | -0.10227 | 0.147725 | EUR | Costochondritis | musculoskeletal |
| 386.2 | -0.01372 | 0.038569 | -0.35577 | 0.722016 | -0.08932 | 0.061873 | EUR | Peripheral or cen | sense organs |
| 739 | 0.025548 | 0.071988 | 0.35489 | 0.722672 | -0.11555 | 0.166641 | EUR | Contracture of jo | musculoskeletal |
| 990 | -0.00924 | 0.026056 | -0.35456 | 0.722916 | -0.06031 | 0.04183 | EUR | Effects radiation | injuries & poisonings |
| 295.2 | 0.033457 | 0.095656 | 0.349769 | 0.726512 | -0.15402 | 0.220939 | EUR | Paranoid disorde | mental disorders |
| 860 | 0.026864 | 0.076992 | 0.348925 | 0.727146 | -0.12404 | 0.177766 | EUR | Bone marrow or | neoplasms |
| 603 | 0.020716 | 0.059683 | 0.347107 | 0.728511 | -0.09626 | 0.137693 | EUR | Other disorders c | genitourinary |
| 637 | -0.05661 | 0.163424 | -0.3464 | 0.729039 | -0.37692 | 0.263694 | EUR | Short gestation; l | pregnancy complications |
| 782.6 | 0.015072 | 0.043687 | 0.345005 | 0.730091 | -0.07055 | 0.100697 | EUR | Pallor and flushir | symptoms |
| 389.5 | -0.02787 | 0.081058 | -0.34381 | 0.730987 | -0.18674 | 0.131003 | EUR | Disorders of acou | sense organs |

| phecode | Coef. | Std.Err. | z | p_value_z | [0.025 | 0.975] | GIA | phenotype | category |
|---|---|---|---|---|---|---|---|---|---|
| 198.4 | -0.01673 | 0.048655 | -0.34379 | 0.731006 | -0.11209 | 0.078636 | EUR | Secondary malig | neoplasms |
| 465.4 | 0.020892 | 0.060857 | 0.343293 | 0.731378 | -0.09839 | 0.140169 | EUR | Acute laryngitis a | respiratory |
| 426.21 | 0.019553 | 0.05723 | 0.341651 | 0.732613 | -0.09262 | 0.131721 | EUR | First degree AV b | circulatory system |
| 159.2 | 0.035622 | 0.104828 | 0.339814 | 0.733997 | -0.16984 | 0.241081 | EUR | Malignant neopla | neoplasms |
| 498 | -0.02194 | 0.064716 | -0.33903 | 7.35E-01 | -0.14878 | 0.104901 | EUR | Acute bronchosp | respiratory |
| 283 | 0.032118 | 0.094979 | 0.33816 | 0.735243 | -0.15404 | 0.218273 | EUR | Acquired hemoly | hematopoietic |
| 276.11 | -0.01769 | 0.052514 | -0.3369 | 0.736189 | -0.12062 | 0.085233 | EUR | Hyperosmolality a | endocrine/metabolic |
| 639 | 0.227207 | 0.676088 | 0.336061 | 0.736825 | -1.0979 | 1.552315 | EUR | Complications fo | pregnancy complications |
| 627.1 | 0.016648 | 0.0496 | 0.33564 | 0.737142 | -0.08057 | 0.113862 | EUR | Postmenopausal | genitourinary |
| 622.1 | 0.017234 | 0.051414 | 0.335205 | 0.73747 | -0.08353 | 0.118003 | EUR | Polyp of corpus u | genitourinary |
| 365.2 | -0.0244 | 0.072805 | -0.33511 | 0.737544 | -0.16709 | 0.118297 | EUR | Primary angle-clo | sense organs |
| 287.2 | 0.148183 | 0.443001 | 0.334499 | 0.738003 | -0.72008 | 1.01645 | EUR | Allergic purpura | hematopoietic |
| 656 | 0.02111 | 0.063151 | 0.334274 | 0.738173 | -0.10266 | 0.144884 | EUR | Other perinatal c | pregnancy complications |
| 8.5 | -0.01443 | 0.04318 | -0.33409 | 0.738314 | -0.09906 | 0.070206 | EUR | Bacterial enteritis | infectious diseases |
| 270.38 | 0.020732 | 0.062084 | 0.333927 | 0.738435 | -0.10095 | 0.142414 | EUR | Other specified d | endocrine/metabolic |
| 287.3 | 0.008943 | 0.026804 | 0.333631 | 0.738658 | -0.04359 | 0.061478 | EUR | Thrombocytopen | hematopoietic |
| 587 | 0.01994 | 0.059813 | 0.33337 | 0.738855 | -0.09729 | 0.13717 | EUR | Kidney replaced | genitourinary |
| 375 | -0.01994 | 0.060106 | -0.33182 | 0.740024 | -0.13775 | 0.097862 | EUR | Disorders of lacri | sense organs |
| 292.2 | -0.01826 | 0.055052 | -0.33174 | 0.740087 | -0.12616 | 0.089638 | EUR | Mild cognitive im | mental disorders |
| 743.4 | -0.02803 | 0.084659 | -0.33106 | 0.740599 | -0.19396 | 0.137902 | EUR | Stress fracture | musculoskeletal |
| 513.31 | 0.021219 | 0.064097 | 0.33104 | 0.740614 | -0.10441 | 0.146846 | EUR | Apnea | respiratory |
| 790.6 | 0.006137 | 0.018543 | 0.330966 | 0.74067 | -0.03021 | 0.042481 | EUR | Other abnormal b | symptoms |
| 711.3 | 0.088995 | 0.269654 | 0.330036 | 0.741372 | -0.43952 | 0.617507 | EUR | Behcet's syndron | musculoskeletal |
| 742 | -0.01243 | 0.037678 | -0.32996 | 0.741433 | -0.08628 | 0.061415 | EUR | Derangement of j | musculoskeletal |
| 253.11 | -0.07679 | 0.232914 | -0.32969 | 0.741632 | -0.53329 | 0.379713 | EUR | Acromegaly and | endocrine/metabolic |
| 275 | -0.00756 | 0.022966 | -0.32939 | 0.741859 | -0.05258 | 0.037448 | EUR | Disorders of mine | endocrine/metabolic |
| 707.1 | -0.01947 | 0.059359 | -0.32799 | 0.742916 | -0.13581 | 0.096872 | EUR | Decubitus ulcer | dermatologic |
| 709 | 0.012784 | 0.039122 | 0.326779 | 0.743835 | -0.06389 | 0.089461 | EUR | Diffuse diseases | dermatologic |
| 783 | 0.007064 | 0.021824 | 0.323691 | 0.746172 | -0.03571 | 0.049839 | EUR | Fever of unknow | symptoms |
| 592 | -0.00784 | 0.024254 | -0.32343 | 0.746372 | -0.05538 | 0.039693 | EUR | Cystitis and ureth | genitourinary |
| 910 | -0.04563 | 0.142634 | -0.3199 | 0.749042 | -0.32519 | 0.233928 | EUR | Superficial injury, | injuries & poisonings |
| 285.22 | -0.01481 | 0.046518 | -0.31839 | 0.750187 | -0.10598 | 0.076363 | EUR | Anemia in neopla | hematopoietic |
| 157 | -0.02039 | 0.0643 | -0.31717 | 0.751112 | -0.14642 | 0.105632 | EUR | Pancreatic cance | neoplasms |
| 374 | 0.009716 | 0.030659 | 0.316917 | 0.751306 | -0.05037 | 0.069806 | EUR | Other disorders c | sense organs |
| 287 | -0.0077 | 0.024438 | -0.31512 | 0.752674 | -0.0556 | 0.040197 | EUR | Purpura and othe | hematopoietic |
| 339 | -0.00623 | 0.019896 | -0.31328 | 0.754069 | -0.04523 | 0.032763 | EUR | Other headache | neurological |
| 281.12 | -0.02937 | 0.094649 | -0.31029 | 7.56E-01 | -0.21488 | 0.15614 | EUR | Other vitamin B1 | hematopoietic |

| phecode | Coef. | Std.Err. | z | p_value_z | [0.025 | 0.975] | GIA | phenotype | category |
|---|---|---|---|---|---|---|---|---|---|
| 202 | -0.01416 | 0.045916 | -0.30838 | 0.757792 | -0.10415 | 0.075835 | EUR | Cancer of other ly | neoplasms |
| 733.9 | -0.01796 | 0.058602 | -0.30654 | 0.759191 | -0.13282 | 0.096894 | EUR | Chondromalacia | musculoskeletal |
| 259 | -0.01254 | 0.040994 | -0.30592 | 0.759663 | -0.09289 | 0.067805 | EUR | Other endocrine | endocrine/metabolic |
| 187.8 | 0.108502 | 0.3557 | 0.305038 | 0.760337 | -0.58866 | 0.805661 | EUR | Neoplasm of unc | neoplasms |
| 261.1 | 0.058778 | 0.19304 | 0.304485 | 0.760759 | -0.31957 | 0.437129 | EUR | Vitamin A deficie | endocrine/metabolic |
| 363 | -0.02453 | 0.080676 | -0.30404 | 0.761095 | -1.83E-01 | 0.133593 | EUR | Chorioretinal infla | sense organs |
| 279.11 | 0.015374 | 0.050622 | 0.303707 | 0.761351 | -0.08384 | 0.114593 | EUR | Deficiency of hur | endocrine/metabolic |
| 202.2 | -0.01516 | 0.050066 | -0.30283 | 0.762019 | -0.11329 | 0.082966 | EUR | Non-Hodgkins ly | neoplasms |
| 8.52 | 0.016691 | 0.055157 | 0.302609 | 0.762187 | -0.09141 | 0.124797 | EUR | Intestinal infectio | infectious diseases |
| 565 | -0.0073 | 0.024118 | -0.30248 | 0.762286 | -0.05457 | 0.039975 | EUR | Anal and rectal c | digestive |
| 512.3 | -0.02731 | 0.090753 | -0.30089 | 0.763496 | -0.20518 | 0.150566 | EUR | Abnormal chest s | respiratory |
| 306 | 0.005126 | 0.01723 | 0.297533 | 0.76606 | -0.02864 | 0.038897 | EUR | Other mental dis | mental disorders |
| 694.1 | -0.03342 | 0.112739 | -0.29642 | 0.766909 | -0.25438 | 0.187546 | EUR | Vitiligo | dermatologic |
| 292.12 | -0.03306 | 0.112323 | -0.29434 | 0.768499 | -0.25321 | 0.187089 | EUR | Symbolic dysfunc | mental disorders |
| 755.1 | -0.02449 | 0.083289 | -0.29398 | 0.768776 | -0.18773 | 0.138759 | EUR | Congenital defor | congenital anomalies |
| 771.2 | -0.01147 | 0.039045 | -0.29389 | 0.768845 | -0.088 | 0.065052 | EUR | Cramp of limb | symptoms |
| 218 | -0.01014 | 0.034724 | -0.29194 | 0.77033 | -0.07819 | 0.05792 | EUR | Benign neoplasm | neoplasms |
| 540.11 | 0.023827 | 0.081757 | 0.291434 | 0.77072 | -0.13641 | 0.184068 | EUR | Acute appendiciti | digestive |
| 41.9 | -0.01829 | 0.06279 | -0.29133 | 0.770802 | -0.14136 | 0.104774 | EUR | Infection with dru | infectious diseases |
| 756.22 | -0.17061 | 0.590643 | -0.28886 | 0.772692 | -1.32825 | 0.987028 | EUR | Pectus carinatum | congenital anomalies |
| 244.3 | 0.131391 | 0.455621 | 0.288378 | 0.773057 | -0.76161 | 1.024392 | EUR | Iodine hypothyroi | endocrine/metabolic |
| 197 | 0.004887 | 0.016947 | 0.288371 | 0.773062 | -0.02833 | 0.038102 | EUR | Chemotherapy | neoplasms |
| 556.11 | 0.029395 | 0.102343 | 0.28722 | 0.773944 | -0.17119 | 0.229983 | EUR | Angiodysplasia o | digestive |
| 691 | 0.016633 | 0.058029 | 0.286635 | 0.774392 | -0.0971 | 0.130368 | EUR | Congenital anom | dermatologic |
| 614.54 | -0.03975 | 0.138686 | -0.28662 | 0.774403 | -0.31157 | 0.23207 | EUR | Abscess or ulcer | genitourinary |
| 740.2 | 0.009836 | 0.034384 | 0.286055 | 0.774836 | -0.05756 | 0.077227 | EUR | Osteoarthrosis, g | musculoskeletal |
| 430.2 | -0.02223 | 0.077765 | -0.28584 | 0.774999 | -0.17464 | 0.130188 | EUR | Intracerebral hen | circulatory system |
| 41.8 | 0.019251 | 0.067414 | 0.28556 | 0.775215 | -0.11288 | 0.15138 | EUR | H. pylori | infectious diseases |
| 257 | 0.011007 | 0.038707 | 0.284373 | 0.776125 | -0.06486 | 0.086872 | EUR | Testicular dysfun | endocrine/metabolic |
| 608 | -0.01169 | 0.041123 | -0.28429 | 0.776187 | -0.09229 | 0.068909 | EUR | Other disorders c | genitourinary |
| 531.4 | -0.01142 | 0.040288 | -0.28345 | 0.776828 | -0.09038 | 0.067544 | EUR | Peptic ulcer, site | digestive |
| 202.23 | -0.05332 | 0.188815 | -0.28237 | 0.777663 | -0.42339 | 0.316756 | EUR | Lymphosarcoma | neoplasms |
| 368.9 | -0.01379 | 0.048986 | -0.28149 | 0.778337 | -0.1098 | 0.082222 | EUR | Subjective visual | sense organs |
| 736 | 0.013966 | 0.049676 | 0.281143 | 0.778601 | -0.0834 | 0.111328 | EUR | Other acquired d | musculoskeletal |
| 257.1 | 0.010844 | 0.038736 | 0.279933 | 0.779529 | -0.06508 | 0.086766 | EUR | Testicular hypofu | endocrine/metabolic |
| 561.1 | -0.00546 | 0.01951 | -0.27987 | 0.779578 | -0.0437 | 0.032778 | EUR | Diarrhea | digestive |
| 286.3 | -0.02578 | 0.092288 | -0.27935 | 0.779972 | -0.20666 | 0.1551 | EUR | Coagulation defe | hematopoietic |

| phecode | Coef. | Std.Err. | z | p_value_z | [0.025 | 0.975] | GIA | phenotype | category |
|---|---|---|---|---|---|---|---|---|---|
| 364.51 | -0.02954 | 0.105874 | -0.27903 | 0.780223 | -0.23705 | 0.177967 | EUR | Fuchs' dystrophy | sense organs |
| 627.22 | -0.00782 | 0.028055 | -0.27866 | 0.780503 | -0.06281 | 0.047169 | EUR | Need for Hormor | genitourinary |
| 938 | -0.0079 | 0.028503 | -0.27721 | 0.78162 | -0.06377 | 0.047963 | EUR | Dermatitis due to | dermatologic |
| 701 | -0.00536 | 0.019345 | -0.27695 | 0.781815 | -0.04327 | 0.032557 | EUR | Other hypertroph | dermatologic |
| 871 | 0.007645 | 0.027655 | 0.276449 | 0.782203 | -0.04656 | 0.061848 | EUR | Open wounds of | injuries & poisonings |
| 474.2 | 0.017654 | 0.064288 | 0.274603 | 0.783621 | -0.10835 | 0.143657 | EUR | Chronic tonsillitis | respiratory |
| 726 | -0.00501 | 0.018315 | -0.27371 | 0.784306 | -0.04091 | 0.030884 | EUR | Peripheral enthes | musculoskeletal |
| 270 | -0.01054 | 0.038774 | -0.27196 | 0.785653 | -0.08654 | 0.06545 | EUR | Disorders of prot | endocrine/metabolic |
| 741 | -0.00545 | 0.020071 | -0.27134 | 0.78613 | -0.04479 | 0.033893 | EUR | Symptoms and d | musculoskeletal |
| 741.4 | 0.006394 | 0.023635 | 0.270539 | 0.786746 | -0.03993 | 0.052718 | EUR | Joint effusions | musculoskeletal |
| 575.6 | -0.0201 | 0.074428 | -0.27006 | 0.787111 | -0.16598 | 0.125776 | EUR | Cholesterolosis c | digestive |
| 277.2 | -0.19256 | 0.713672 | -0.26981 | 7.87E-01 | -1.59133 | 1.206212 | EUR | Other disorders c | endocrine/metabolic |
| 204 | 0.01375 | 0.051529 | 0.266839 | 0.789593 | -0.08724 | 0.114744 | EUR | Leukemia | neoplasms |
| 696.41 | 0.010114 | 0.038468 | 0.262908 | 0.792622 | -0.06528 | 0.08551 | EUR | Psoriasis vulgaris | dermatologic |
| 701.5 | -0.01675 | 0.064271 | -0.26066 | 0.794357 | -0.14272 | 0.109216 | EUR | Abnormal granula | dermatologic |
| 647.1 | 0.028475 | 0.109656 | 0.259678 | 0.795113 | -0.18645 | 0.243398 | EUR | Infections of geni | pregnancy complications |
| 272.13 | 0.005632 | 0.021729 | 0.25921 | 0.795473 | -0.03696 | 0.048221 | EUR | Mixed hyperlipide | endocrine/metabolic |
| 458.2 | -0.01389 | 0.054099 | -0.25675 | 0.797373 | -0.11992 | 0.092142 | EUR | Iatrogenic hypote | circulatory system |
| 395.1 | -0.00753 | 0.029453 | -0.25568 | 0.798201 | -0.06526 | 0.050196 | EUR | Nonrheumatic mi | circulatory system |
| 427.1 | 0.006599 | 0.025843 | 0.255349 | 0.798454 | -0.04405 | 0.05725 | EUR | Paroxysmal tachy | circulatory system |
| 601.4 | 0.034495 | 0.135612 | 0.254368 | 0.799211 | -0.2313 | 0.30029 | EUR | Balanoposthitis | genitourinary |
| 112.3 | -0.01805 | 0.071075 | -0.2539 | 0.799574 | -0.15735 | 0.121258 | EUR | Candidiasis of sk | infectious diseases |
| 313 | 0.009788 | 0.038602 | 0.253556 | 0.799838 | -0.06587 | 0.085446 | EUR | Pervasive develo | mental disorders |
| 446.7 | 0.080019 | 0.316424 | 0.252887 | 0.800356 | -0.54016 | 0.700198 | EUR | Takayasu's disea | circulatory system |
| 750.21 | -0.0226 | 0.090043 | -0.25095 | 0.801854 | -0.19908 | 0.153885 | EUR | Congenital anom | congenital anomalies |
| 225.1 | 0.013243 | 0.052883 | 0.250414 | 0.802267 | -0.09041 | 0.116892 | EUR | Benign neoplasm | neoplasms |
| 817 | 0.013411 | 0.053673 | 0.249867 | 0.80269 | -0.09179 | 0.118609 | EUR | Concussion | injuries & poisonings |
| 963 | -0.00784 | 0.031377 | -0.24987 | 0.802691 | -0.06934 | 0.053658 | EUR | Poisoning by prir | injuries & poisonings |
| 287.1 | -0.01824 | 0.073566 | -0.2479 | 0.804214 | -0.16242 | 0.12595 | EUR | Spontaneous ecc | hematopoietic |
| 38.3 | 0.01104 | 0.044708 | 0.246935 | 0.804958 | -0.07659 | 0.098665 | EUR | Bacteremia | infectious diseases |
| 535.2 | -0.00787 | 0.032097 | -0.24511 | 0.806374 | -0.07078 | 0.055042 | EUR | Atrophic gastritis | digestive |
| 671 | -0.01737 | 0.071817 | -0.2419 | 0.808858 | -0.15813 | 0.123387 | EUR | Venous/cerebrov | pregnancy complications |
| 290.3 | 0.011898 | 0.049209 | 0.241785 | 0.808946 | -0.08455 | 0.108346 | EUR | Other persistent | mental disorders |
| 871.4 | -0.02385 | 0.098966 | -0.24104 | 0.809528 | -0.21782 | 0.170116 | EUR | Open wound of t | injuries & poisonings |
| 963.1 | -0.00763 | 0.031907 | -0.23916 | 0.810985 | -0.07017 | 0.054906 | EUR | Antineoplastic an | injuries & poisonings |
| 785 | 0.003923 | 0.016426 | 0.238838 | 0.811231 | -0.02827 | 0.036116 | EUR | Abdominal pain | symptoms |
| 580.12 | -0.03204 | 0.134207 | -0.23871 | 0.811327 | -0.29508 | 0.231004 | EUR | Non-proliferative | genitourinary |

| phecode | Coef. | Std.Err. | z | p_value_z | [0.025 | 0.975] | GIA | phenotype | category |
|---|---|---|---|---|---|---|---|---|---|
| 649.1 | -0.02085 | 0.087874 | -0.23723 | 0.812475 | -0.19308 | 0.151383 | EUR | Diabetes or abnc | pregnancy complications |
| 595 | 0.009244 | 0.039057 | 0.236678 | 0.812907 | -0.06731 | 0.085794 | EUR | Hydronephrosis | genitourinary |
| 191.1 | -0.01494 | 0.06328 | -0.23604 | 0.813401 | -0.13896 | 0.10909 | EUR | Cancer of brain a | neoplasms |
| 599.1 | -0.00923 | 0.039178 | -0.23547 | 0.813848 | -0.08601 | 0.067563 | EUR | Urinary obstructi | genitourinary |
| 446.8 | 0.04437 | 0.19005 | 0.233468 | 0.815398 | -0.32812 | 0.416861 | EUR | Thrombotic micro | circulatory system |
| 450 | 0.012203 | 0.052281 | 0.233406 | 0.815446 | -0.09027 | 0.114672 | EUR | Noninfectious dis | circulatory system |
| 341 | 0.033561 | 0.143984 | 0.233091 | 0.815691 | -0.24864 | 0.315765 | EUR | Other demyelinat | neurological |
| 540.1 | 0.015551 | 0.066919 | 0.232379 | 0.816244 | -0.11561 | 0.146709 | EUR | Appendicitis | digestive |
| 575.2 | 0.014348 | 0.061926 | 0.231703 | 0.816768 | -0.10702 | 0.135721 | EUR | Obstruction of bil | digestive |
| 644 | -0.01735 | 0.075278 | -0.23054 | 0.817671 | -0.1649 | 0.130188 | EUR | Anemia during pr | pregnancy complications |
| 194 | -0.0313 | 0.136046 | -0.2301 | 0.818017 | -0.29795 | 0.235342 | EUR | Cancer of other e | neoplasms |
| 714.1 | 0.009794 | 0.042646 | 0.229662 | 0.818354 | -0.07379 | 0.093378 | EUR | Rheumatoid arthi | musculoskeletal |
| 315 | 0.011779 | 0.051603 | 0.228252 | 0.819451 | -0.08936 | 0.112919 | EUR | Develomental de | mental disorders |
| 250.7 | 0.014246 | 0.062431 | 0.228184 | 0.819503 | -0.10812 | 0.136609 | EUR | Diabetic retinopa | endocrine/metabolic |
| 870.1 | -0.0235 | 0.103233 | -0.22763 | 0.819935 | -0.22583 | 0.178835 | EUR | Open wound or la | injuries & poisonings |
| 527 | 0.008553 | 0.037626 | 0.227309 | 0.820183 | -0.06519 | 0.082299 | EUR | Diseases of the s | digestive |
| 589 | 0.012913 | 0.057752 | 0.223593 | 0.823074 | -0.10028 | 0.126104 | EUR | Abnormal results | genitourinary |
| 575 | -0.00678 | 0.030568 | -0.2219 | 0.824393 | -0.06669 | 0.053129 | EUR | Other biliary tract | digestive |
| 656.5 | 0.230435 | 1.040442 | 0.221478 | 0.824721 | -1.80879 | 2.269663 | EUR | Hematological di | pregnancy complications |
| 357 | 0.004738 | 0.021401 | 0.221398 | 0.824783 | -0.03721 | 0.046683 | EUR | Inflammatory and | neurological |
| 500.1 | -0.02728 | 0.124167 | -0.21971 | 0.826094 | -0.27064 | 0.216082 | EUR | Extrinsic allergic | respiratory |
| 323.8 | 0.022132 | 0.100849 | 0.219456 | 0.826295 | -0.17553 | 0.219793 | EUR | Encephalitis, non | neurological |
| 304 | -0.00594 | 0.02724 | -0.21788 | 0.827525 | -0.05933 | 0.047455 | EUR | Adjustment react | mental disorders |
| 588 | 0.009937 | 0.045657 | 0.217653 | 0.827699 | -0.07955 | 0.099424 | EUR | Disorders resultir | genitourinary |
| 204.12 | -0.02223 | 0.102621 | -0.21663 | 0.828497 | -0.22336 | 0.178903 | EUR | Lymphoid leuken | neoplasms |
| 495.2 | 0.007408 | 0.034207 | 0.216558 | 0.828553 | -0.05964 | 0.074453 | EUR | Asthma with exa | respiratory |
| 526.41 | -0.01249 | 0.059054 | -0.21144 | 0.832547 | -0.12823 | 0.103258 | EUR | Temporomandibu | digestive |
| 381.11 | -0.00923 | 0.044125 | -0.20911 | 8.34E-01 | -0.09571 | 0.077256 | EUR | Suppurative and | sense organs |
| 315.1 | 0.035649 | 0.171081 | 0.208375 | 0.834936 | -0.29966 | 0.370961 | EUR | Learning disorde | mental disorders |
| 429.2 | -0.00784 | 0.038165 | -0.20531 | 0.837334 | -0.08264 | 0.066967 | EUR | Abnormal functio | circulatory system |
| 681 | -0.00453 | 0.022173 | -0.2042 | 0.838197 | -0.04799 | 0.038931 | EUR | Superficial celluli | dermatologic |
| 795.81 | 0.020179 | 0.1004 | 0.200981 | 0.840713 | -0.1766 | 0.21696 | EUR | Elevated carcino | symptoms |
| 578.8 | -0.00588 | 0.029584 | -0.1989 | 0.842343 | -0.06387 | 0.052099 | EUR | Hemorrhage of re | digestive |
| 10 | 0.01822 | 0.091817 | 0.198439 | 0.842702 | -0.16174 | 0.198178 | EUR | Tuberculosis | infectious diseases |
| 277.51 | 0.014044 | 0.070844 | 0.198238 | 8.43E-01 | -0.12481 | 0.152895 | EUR | Lipoprotein disor | endocrine/metabolic |
| 70.2 | 0.016788 | 0.084746 | 0.198098 | 0.842968 | -0.14931 | 0.182888 | EUR | Viral hepatitis B | infectious diseases |
| 550.6 | -0.01192 | 0.060281 | -0.19771 | 0.843275 | -0.13007 | 0.106231 | EUR | Incisional hernia | digestive |

| phecode | Coef. | Std.Err. | z | p_value_z | [0.025 | 0.975] | GIA | phenotype | category |
|---|---|---|---|---|---|---|---|---|---|
| 427.61 | -0.00931 | 0.047133 | -0.19742 | 0.843496 | -0.10168 | 0.083074 | EUR | Supraventricular | circulatory system |
| 710.11 | -0.01848 | 0.093649 | -0.1973 | 0.843592 | -0.20203 | 0.165072 | EUR | Acute osteomyeli | musculoskeletal |
| 728.7 | -0.00584 | 0.029693 | -0.19671 | 0.844055 | -0.06404 | 0.052356 | EUR | Fasciitis | musculoskeletal |
| 942 | -0.01499 | 0.077425 | -0.19361 | 0.846483 | -0.16674 | 0.136761 | EUR | Infusion and tran | injuries & poisonings |
| 270.3 | 0.008393 | 0.04337 | 0.193532 | 0.846542 | -0.07661 | 0.093396 | EUR | Disorders of plas | endocrine/metabolic |
| 380.1 | -0.00754 | 0.039215 | -0.19221 | 0.847581 | -0.0844 | 0.069322 | EUR | Otitis externa | sense organs |
| 291.4 | 0.011086 | 0.059184 | 0.18732 | 0.85141 | -0.10491 | 0.127085 | EUR | Specific nonpsyc | mental disorders |
| 38.1 | -0.00991 | 0.052892 | -0.18728 | 0.85144 | -0.11357 | 0.093761 | EUR | Gram negative se | infectious diseases |
| 513.4 | 0.020981 | 0.112364 | 0.186723 | 0.851878 | -0.19925 | 0.241211 | EUR | Hyperventilation | respiratory |
| 474 | 0.010829 | 0.058022 | 0.186643 | 0.85194 | -0.10289 | 0.12455 | EUR | Acute and chroni | respiratory |
| 727.2 | 0.015289 | 0.082333 | 0.185693 | 0.852685 | -0.14608 | 0.176659 | EUR | Bursitis disorders | musculoskeletal |
| 818 | 0.019306 | 0.105084 | 0.183716 | 0.854236 | -0.18666 | 0.225266 | EUR | Intracranial hemo | injuries & poisonings |
| 371.1 | -0.01568 | 0.085826 | -0.18266 | 0.855062 | -0.18389 | 0.152538 | EUR | Uveitis, noninfect | sense organs |
| 523.32 | 0.122144 | 0.681814 | 0.179146 | 0.857823 | -1.21419 | 1.458474 | EUR | Chronic periodon | digestive |
| 378 | 0.007956 | 0.044444 | 0.179015 | 0.857926 | -0.07915 | 0.095065 | EUR | Strabismus and c | sense organs |
| 723 | 0.007445 | 0.041662 | 0.178714 | 0.858163 | -0.07421 | 0.089101 | EUR | Other disorders c | musculoskeletal |
| 8.6 | -0.01355 | 0.075969 | -0.17841 | 0.858399 | -0.16245 | 0.135342 | EUR | Viral Enteritis | infectious diseases |
| 259.3 | 0.198216 | 1.121694 | 0.176711 | 0.859736 | -2.00026 | 2.396696 | EUR | Delay in sexual d | endocrine/metabolic |
| 184.2 | -0.02018 | 0.114899 | -0.17562 | 0.860593 | -0.24538 | 0.205019 | EUR | Cancer of other f | neoplasms |
| 275.6 | -0.00618 | 0.035561 | -0.17378 | 0.862036 | -0.07588 | 0.063518 | EUR | Hypercalcemia | endocrine/metabolic |
| 741.6 | -0.04129 | 0.23881 | -0.1729 | 0.862732 | -0.50935 | 0.426769 | EUR | Villonodular sync | musculoskeletal |
| 395 | 0.00404 | 0.023371 | 0.172859 | 0.862763 | -0.04177 | 0.049847 | EUR | Heart valve disor | circulatory system |
| 198.5 | -0.01142 | 0.066336 | -0.17219 | 0.86329 | -0.14144 | 0.118594 | EUR | Secondary malig | neoplasms |
| 573.7 | 0.006184 | 0.036316 | 0.170289 | 0.864783 | -0.06499 | 0.077361 | EUR | Abnormal results | digestive |
| 596 | 0.004697 | 0.027653 | 0.169839 | 0.865136 | -0.0495 | 0.058895 | EUR | Other disorders c | genitourinary |
| 356 | 0.005589 | 0.033004 | 0.169349 | 0.865522 | -0.0591 | 0.070276 | EUR | Hereditary and id | neurological |
| 656.3 | 0.038957 | 0.232314 | 0.167693 | 0.866825 | -0.41637 | 0.494284 | EUR | Endocrine and m | pregnancy complications |
| 444.1 | 0.014168 | 0.086534 | 0.163732 | 0.869943 | -0.15543 | 0.183771 | EUR | Arterial embolism | circulatory system |
| 580.2 | -0.01214 | 0.074882 | -0.16209 | 0.871237 | -0.1589 | 0.134628 | EUR | Nephrotic syndro | genitourinary |
| 949.1 | 0.03946 | 0.247045 | 0.159727 | 0.873096 | -0.44474 | 0.523659 | EUR | Diaper or napkin | injuries & poisonings |
| 153 | 0.00617 | 0.038997 | 0.158219 | 0.874284 | -0.07026 | 0.082602 | EUR | Colorectal cance | neoplasms |
| 70.1 | -0.02218 | 0.14028 | -0.15812 | 0.87436 | -0.29712 | 0.252761 | EUR | Viral hepatitis A | infectious diseases |
| 705 | -0.01108 | 0.070315 | -0.15751 | 0.874845 | -0.14889 | 0.12674 | EUR | Disorders of swe | dermatologic |
| 752 | -0.01126 | 0.071776 | -0.15684 | 0.875371 | -0.15194 | 0.129421 | EUR | Nervous system | congenital anomalies |
| 695.8 | -0.00653 | 0.041922 | -0.15567 | 0.876289 | -0.08869 | 0.07564 | EUR | Other specified e | dermatologic |
| 530.6 | 0.021696 | 0.143994 | 0.150671 | 8.80E-01 | -0.26053 | 0.303918 | EUR | Diverticulum of e | digestive |
| 362.4 | 0.009034 | 0.060566 | 0.149156 | 0.88143 | -0.10967 | 0.127741 | EUR | Retinal vascular | sense organs |

| phecode | Coef. | Std.Err. | z | p_value_z | [0.025 | 0.975] | GIA | phenotype | category |
|---|---|---|---|---|---|---|---|---|---|
| 735.23 | 0.013672 | 0.092208 | 0.148269 | 0.88213 | -0.16705 | 0.194396 | EUR | Hallux rigidus | musculoskeletal |
| 253.2 | -0.01375 | 0.093125 | -0.1476 | 0.882655 | -0.19627 | 0.168776 | EUR | Pituitary hypofun | endocrine/metabolic |
| 564.8 | -0.00487 | 0.033627 | -0.14486 | 0.884821 | -0.07078 | 0.061036 | EUR | Abnormal finding | digestive |
| 350.3 | -0.00841 | 0.058874 | -0.14292 | 0.88635 | -0.12381 | 0.106976 | EUR | Lack of coordinat | neurological |
| 772.4 | 0.01647 | 0.116066 | 0.1419 | 0.887159 | -0.21101 | 0.243954 | EUR | Rhabdomyolysis | symptoms |
| 603.2 | -0.01296 | 0.091487 | -0.14167 | 0.887344 | -0.19227 | 0.166351 | EUR | Spermatocele | genitourinary |
| 353.1 | 0.009441 | 0.066899 | 0.141127 | 0.88777 | -0.12168 | 0.140562 | EUR | Nerve plexus lesi | neurological |
| 377.3 | -0.01083 | 0.078639 | -0.13774 | 0.890443 | -0.16496 | 0.143297 | EUR | Optic neuritis/neu | sense organs |
| 213 | 0.010784 | 0.078654 | 0.137106 | 0.890947 | -0.14337 | 0.164942 | EUR | Benign neoplasm | neoplasms |
| 686.3 | -0.01799 | 0.13133 | -0.13701 | 0.891021 | -0.2754 | 0.239408 | EUR | Pilonidal cyst | dermatologic |
| 196 | 0.002461 | 0.017987 | 0.136807 | 0.891184 | -0.03279 | 0.037715 | EUR | Radiotherapy | neoplasms |
| 313.3 | 0.019651 | 0.143935 | 0.136531 | 0.891402 | -0.26246 | 0.301758 | EUR | Autism | mental disorders |
| 835 | 0.004799 | 0.03574 | 0.134287 | 0.893175 | -0.06525 | 0.074849 | EUR | Internal deranger | injuries & poisonings |
| 255.3 | -0.02138 | 0.161911 | -0.13206 | 0.894936 | -0.33872 | 0.295958 | EUR | Adrenogenital dis | endocrine/metabolic |
| 300.4 | 0.004737 | 0.036299 | 0.130495 | 0.896175 | -0.06641 | 0.075882 | EUR | Dysthymic disord | mental disorders |
| 348.2 | -0.00692 | 0.053188 | -0.1301 | 0.89649 | -0.11117 | 0.097327 | EUR | Cerebral edema | neurological |
| 153.3 | -0.00631 | 0.050314 | -0.12548 | 0.900142 | -0.10493 | 0.0923 | EUR | Malignant neopla | neoplasms |
| 54 | -0.00361 | 0.028808 | -0.12531 | 0.900281 | -0.06007 | 0.052853 | EUR | Herpes simplex | infectious diseases |
| 158 | -0.00646 | 0.051746 | -0.12483 | 0.900656 | -0.10788 | 0.094962 | EUR | Neoplasm of uns | neoplasms |
| 395.2 | 0.003859 | 0.031049 | 0.124294 | 0.901082 | -0.057 | 0.064715 | EUR | Nonrheumatic ac | circulatory system |
| 8.51 | 0.108744 | 0.881615 | 0.123346 | 0.901833 | -1.61919 | 1.836678 | EUR | Intestinal e.coli | infectious diseases |
| 803 | -0.00426 | 0.034634 | -0.12293 | 0.902165 | -0.07214 | 0.063623 | EUR | Fracture of uppe | injuries & poisonings |
| 38.2 | 0.010417 | 0.085068 | 0.122458 | 0.902536 | -0.15631 | 0.177148 | EUR | Gram positive se | infectious diseases |
| 560.1 | 0.00555 | 0.045924 | 0.120858 | 0.903804 | -0.08446 | 0.095559 | EUR | Paralytic ileus | digestive |
| 716.3 | 0.091541 | 0.760024 | 0.120445 | 0.904131 | -1.39808 | 1.581161 | EUR | Kaschin-Beck dis | musculoskeletal |
| 801 | 0.004461 | 0.037174 | 0.119996 | 0.904486 | -0.0684 | 0.077321 | EUR | Fracture of ankle | injuries & poisonings |
| 840.1 | 0.007853 | 0.066218 | 0.118598 | 0.905594 | -0.12193 | 0.137639 | EUR | Muscle/tendon sp | injuries & poisonings |
| 588.2 | -0.00547 | 0.047327 | -0.11568 | 0.907908 | -0.09823 | 0.087285 | EUR | Secondary hyper | genitourinary |
| 580.4 | -0.00996 | 0.086319 | -0.11537 | 0.908153 | -0.17914 | 0.159224 | EUR | Renal sclerosis, I | genitourinary |
| 342 | 0.00944 | 0.08229 | 0.114716 | 0.90867 | -0.15184 | 0.170725 | EUR | Hemiplegia | neurological |
| 324.1 | -0.1305 | 1.145207 | -0.11395 | 0.909278 | -2.37506 | 2.114069 | EUR | Jakob-Creutzfeld | neurological |
| 386 | -0.00375 | 0.033248 | -0.11265 | 0.910308 | -0.06891 | 0.06142 | EUR | Vertiginous syndı | sense organs |
| 1005 | -0.00198 | 0.017783 | -0.11147 | 0.911242 | -0.03684 | 0.032871 | EUR | Other symptoms | |
| 334.2 | 0.021541 | 0.194001 | 0.111036 | 9.12E-01 | -0.35869 | 0.401776 | EUR | Anterior horn cell | neurological |
| 464 | 0.002578 | 0.023374 | 0.110283 | 0.912185 | -0.04323 | 0.04839 | EUR | Acute sinusitis | respiratory |
| 444.2 | 0.019591 | 0.180497 | 0.10854 | 0.913567 | -0.33418 | 0.373359 | EUR | Embolism and th | circulatory system |
| 446.4 | 0.019435 | 0.180465 | 0.107694 | 0.914239 | -0.33427 | 0.373139 | EUR | Wegener's granu | circulatory system |

| phecode | Coef. | Std.Err. | z | p_value_z | [0.025 | 0.975] | GIA | phenotype | category |
|---|---|---|---|---|---|---|---|---|---|
| 531 | -0.00317 | 0.030732 | -0.10303 | 0.917937 | -0.0634 | 0.057067 | EUR | Peptic ulcer (excl | digestive |
| 495 | 0.00203 | 0.019967 | 0.101661 | 0.919026 | -0.03711 | 0.041165 | EUR | Asthma | respiratory |
| 367.9 | -0.00398 | 0.039256 | -0.10148 | 0.919166 | -0.08092 | 0.072957 | EUR | Blindness and lov | sense organs |
| 301.1 | 0.02957 | 0.295378 | 0.100109 | 0.920258 | -0.54936 | 0.608501 | EUR | Schizoid persona | mental disorders |
| 535.8 | 0.004975 | 0.050096 | 0.099317 | 0.920887 | -0.09321 | 0.103162 | EUR | Other specified g | digestive |
| 574.3 | -0.00602 | 0.061469 | -0.09798 | 0.921946 | -0.1265 | 0.114455 | EUR | Cholecystitis with | digestive |
| 727 | 0.001898 | 0.019711 | 0.096314 | 0.923271 | -0.03673 | 0.040532 | EUR | Other disorders c | musculoskeletal |
| 709.2 | 0.004981 | 0.052165 | 0.095488 | 0.923927 | -0.09726 | 0.107223 | EUR | Sicca syndrome | dermatologic |
| 225 | 0.004907 | 0.051402 | 0.095472 | 0.92394 | -0.09584 | 0.105654 | EUR | Benign neoplasm | neoplasms |
| 958 | -0.00916 | 0.095989 | -0.09542 | 0.92398 | -0.19729 | 0.178976 | EUR | Certain early con | injuries & poisonings |
| 117.4 | -0.00801 | 0.085489 | -0.09371 | 0.925341 | -0.17557 | 0.159544 | EUR | Aspergillosis | infectious diseases |
| 696 | 0.003356 | 0.035987 | 0.093265 | 0.925693 | -0.06718 | 0.07389 | EUR | Psoriasis and rel | dermatologic |
| 369.2 | 0.006468 | 0.071486 | 0.090478 | 0.927907 | -0.13364 | 0.146577 | EUR | Eye infection, vir | sense organs |
| 427 | -0.00147 | 0.016943 | -0.0865 | 0.931071 | -0.03467 | 0.031743 | EUR | Cardiac dysrhyth | circulatory system |
| 789 | -0.00151 | 0.017535 | -0.08613 | 0.931363 | -0.03588 | 0.032858 | EUR | Nausea and vom | symptoms |
| 376 | 0.01228 | 0.14286 | 0.08596 | 0.931498 | -0.26772 | 0.292281 | EUR | Disorders of the | sense organs |
| 736.6 | -0.00775 | 0.092498 | -0.08379 | 0.93322 | -0.18904 | 0.173542 | EUR | Unequal leg leng | musculoskeletal |
| 681.3 | 0.004311 | 0.051521 | 0.083678 | 0.933312 | -0.09667 | 0.105289 | EUR | Cellulitis and abs | dermatologic |
| 362.6 | 0.006707 | 0.081041 | 0.082765 | 0.934038 | -0.15213 | 0.165544 | EUR | Peripheral retinal | sense organs |
| 1001 | -0.00394 | 0.048606 | -0.0811 | 0.935359 | -0.09921 | 0.091323 | EUR | Foreign body injury | |
| 783.1 | -0.00658 | 0.081293 | -0.08094 | 0.93549 | -0.16591 | 0.152751 | EUR | Postprocedural fe | symptoms |
| 620.1 | -0.00941 | 0.11962 | -0.07866 | 0.937303 | -0.24386 | 0.225041 | EUR | Dysplasia of cerv | genitourinary |
| 737.3 | 0.00268 | 0.03413 | 0.078534 | 0.937403 | -0.06421 | 0.069573 | EUR | Kyphoscoliosis a | musculoskeletal |
| 537 | -0.00191 | 0.02437 | -0.07831 | 0.937584 | -0.04967 | 0.045855 | EUR | Other disorders c | digestive |
| 753.2 | 0.012571 | 0.161225 | 0.077974 | 0.937849 | -0.30342 | 0.328567 | EUR | Congenital anom | congenital anomalies |
| 209 | -0.00633 | 0.081387 | -0.07779 | 0.937999 | -0.16585 | 0.153185 | EUR | Neuroendocrine | neoplasms |
| 512.8 | -0.0013 | 0.017338 | -0.07496 | 0.940245 | -0.03528 | 0.032683 | EUR | Cough | respiratory |
| 687.1 | -0.00149 | 0.020069 | -0.07405 | 0.940973 | -0.04082 | 0.037848 | EUR | Rash and other r | dermatologic |
| 385 | 0.007768 | 0.105519 | 0.073616 | 0.941316 | -0.19905 | 0.214582 | EUR | Other disorders c | sense organs |
| 291.1 | -0.01147 | 0.156754 | -0.0732 | 0.941645 | -0.31871 | 0.295757 | EUR | Transient mental | mental disorders |
| 962.3 | -0.00948 | 0.12961 | -0.07311 | 0.941716 | -0.26351 | 0.244555 | EUR | Hormones and sy | injuries & poisonings |
| 983 | -0.03043 | 0.421458 | -0.07221 | 0.942435 | -0.85648 | 0.79561 | EUR | Toxic effect of co | injuries & poisonings |
| 656.8 | 0.066194 | 0.92202 | 0.071792 | 0.942767 | -1.74093 | 1.87332 | EUR | Perinatal jaundic | pregnancy complications |
| 261.2 | -0.00206 | 0.028817 | -0.07141 | 0.943068 | -0.05854 | 0.054423 | EUR | Vitamin B-comple | endocrine/metabolic |
| 557 | -0.00369 | 0.053721 | -0.06873 | 0.945203 | -0.10898 | 0.101599 | EUR | Intestinal malabs | digestive |
| 277.7 | 0.003804 | 0.055452 | 0.068609 | 9.45E-01 | -0.10488 | 0.112488 | EUR | Dysmetabolic syr | endocrine/metabolic |
| 612.3 | 0.013234 | 0.193181 | 0.068507 | 0.945382 | -0.36539 | 0.391862 | EUR | Congenital anom | genitourinary |

| phecode | Coef. | Std.Err. | z | p_value_z | [0.025 | 0.975] | GIA | phenotype | category |
|---|---|---|---|---|---|---|---|---|---|
| 875 | 0.008084 | 0.121902 | 0.066319 | 0.947124 | -0.23084 | 0.247007 | EUR | Non-healing surg | injuries & poisonings |
| 857 | 0.003935 | 0.061749 | 0.063728 | 0.949187 | -0.11709 | 0.124962 | EUR | Mechanical comp | injuries & poisonings |
| 500 | 0.004271 | 0.067061 | 0.063685 | 0.949221 | -0.12717 | 0.135709 | EUR | Lung disease du | respiratory |
| 592.12 | 0.006422 | 0.105809 | 0.060695 | 0.951602 | -0.20096 | 0.213804 | EUR | Chronic cystitis | genitourinary |
| 767 | 0.007226 | 0.123626 | 0.058452 | 0.953388 | -0.23508 | 0.249529 | EUR | Cervicocranial/C | symptoms |
| 348.8 | -0.00248 | 0.042526 | -0.05842 | 0.953415 | -0.08583 | 0.080865 | EUR | Encephalopathy, | neurological |
| 347 | 0.008757 | 0.156586 | 0.055926 | 0.9554 | -0.29814 | 0.315659 | EUR | Cataplexy and na | neurological |
| 277.5 | -0.00316 | 0.056882 | -0.05561 | 9.56E-01 | -0.11465 | 0.108323 | EUR | Other disorders c | endocrine/metabolic |
| 597.1 | -0.00466 | 0.089054 | -0.05232 | 0.958276 | -0.1792 | 0.169883 | EUR | Urethral stricture | genitourinary |
| 274.1 | -0.00185 | 0.036423 | -0.05076 | 0.95952 | -0.07324 | 0.069538 | EUR | Gout | endocrine/metabolic |
| 696.42 | 0.003795 | 0.0749 | 0.050664 | 0.959593 | -0.14301 | 0.150596 | EUR | Psoriatic arthropa | dermatologic |
| 252 | 0.001748 | 0.0345 | 0.050655 | 9.60E-01 | -0.06587 | 0.069367 | EUR | Disorders of para | endocrine/metabolic |
| 966 | -0.01688 | 0.340199 | -0.04963 | 0.960416 | -0.68366 | 0.649894 | EUR | Poisoning by anti | injuries & poisonings |
| 350 | 0.001024 | 0.020925 | 0.048957 | 0.960954 | -0.03999 | 0.042037 | EUR | Abnormal moven | neurological |
| 528.4 | 0.010139 | 0.209388 | 0.048421 | 0.961381 | -0.40025 | 0.420532 | EUR | Cysts of oral soft | digestive |
| 31.1 | -0.05065 | 1.063429 | -0.04763 | 0.962009 | -2.13494 | 2.033629 | EUR | Leprosy | infectious diseases |
| 602 | 0.003582 | 0.07638 | 0.04689 | 0.962601 | -0.14612 | 0.153284 | EUR | Other disorders c | genitourinary |
| 292.6 | 0.004275 | 0.092043 | 0.046451 | 0.962951 | -0.17613 | 0.184677 | EUR | Hallucinations | mental disorders |
| 853 | 0.00367 | 0.07984 | 0.045971 | 0.963333 | -0.15281 | 0.160153 | EUR | Complication of c | injuries & poisonings |
| 348.9 | -0.00175 | 0.038482 | -0.04548 | 0.963728 | -0.07717 | 0.073673 | EUR | Other conditions | neurological |
| 598 | -0.00144 | 0.032728 | -0.04389 | 0.964995 | -0.06558 | 0.062709 | EUR | Abnormal finding | genitourinary |
| 960.3 | 0.043414 | 0.994223 | 0.043666 | 0.96517 | -1.90523 | 1.992056 | EUR | Poisoning by anti | injuries & poisonings |
| 258.1 | 0.005941 | 0.137318 | 0.043266 | 0.96549 | -0.2632 | 0.275079 | EUR | Postablative ova | endocrine/metabolic |
| 830 | 0.00128 | 0.030263 | 0.04228 | 0.966275 | -0.05804 | 0.060594 | EUR | Dislocation | injuries & poisonings |
| 598.9 | -0.00139 | 0.033148 | -0.04189 | 0.966586 | -0.06636 | 0.06358 | EUR | Other nonspecific | genitourinary |
| 790.8 | 0.002738 | 0.066693 | 0.041051 | 0.967255 | -0.12798 | 0.133454 | EUR | Elevated C-react | symptoms |
| 198 | -0.00097 | 0.024412 | -0.03977 | 0.968276 | -0.04882 | 0.046876 | EUR | Secondary malig | neoplasms |
| 374.6 | 0.002404 | 0.060531 | 0.039723 | 0.968314 | -0.11623 | 0.121042 | EUR | Dermatochalasis | sense organs |
| 795.8 | -0.00263 | 0.067896 | -0.03879 | 0.969056 | -0.13571 | 0.13044 | EUR | Abnormal tumor | symptoms |
| 626.12 | 0.001898 | 0.048973 | 0.038754 | 0.969087 | -0.09409 | 0.097884 | EUR | Excessive or freq | genitourinary |
| 427.7 | 0.000977 | 0.026034 | 0.037545 | 0.97005 | -0.05005 | 0.052004 | EUR | Tachycardia NOS | circulatory system |
| 526.4 | -0.00169 | 0.047591 | -0.03545 | 0.971725 | -0.09496 | 0.09159 | EUR | Temporomandibu | digestive |
| 282.8 | 0.002509 | 0.07124 | 0.03522 | 0.971904 | -0.13712 | 0.142138 | EUR | Other hemoglobi | hematopoietic |
| 386.3 | -0.00323 | 0.09751 | -0.03317 | 0.973538 | -0.19435 | 0.187881 | EUR | Labyrinthitis | sense organs |
| 756.21 | -0.00349 | 0.113145 | -0.03082 | 0.975417 | -0.22525 | 0.218273 | EUR | Pectus excavatu | congenital anomalies |
| 656.6 | 0.011731 | 0.401896 | 0.029189 | 0.976714 | -0.77597 | 0.799432 | EUR | Perinatal disorde | pregnancy complications |
| 426.32 | 0.001511 | 0.055051 | 0.027452 | 0.978099 | -0.10639 | 0.109409 | EUR | Left bundle branc | circulatory system |

| phecode | Coef. | Std.Err. | z | p_value_z | [0.025 | 0.975] | GIA | phenotype | category |
|---|---|---|---|---|---|---|---|---|---|
| 286.81 | 0.001438 | 0.052682 | 0.027293 | 0.978226 | -0.10182 | 0.104694 | EUR | Primary hypercoa | hematopoietic |
| 276.12 | -0.00071 | 0.026757 | -0.02644 | 0.978903 | -0.05315 | 0.051735 | EUR | Hyposmolality an | endocrine/metabolic |
| 750.2 | -0.00174 | 0.067821 | -0.02561 | 0.979565 | -0.13466 | 0.131189 | EUR | Lower gastrointes | congenital anomalies |
| 242.2 | -0.00406 | 0.162198 | -0.02504 | 0.98002 | -0.32196 | 0.31384 | EUR | Toxic multinodula | endocrine/metabolic |
| 296.1 | 0.0009 | 0.036243 | 0.024835 | 0.980187 | -0.07013 | 0.071935 | EUR | Bipolar | mental disorders |
| 191.11 | 0.001588 | 0.066423 | 0.023907 | 0.980926 | -0.1286 | 0.131775 | EUR | Cancer of brain | neoplasms |
| 969 | -0.00249 | 0.105279 | -0.02369 | 0.9811 | -0.20884 | 0.203849 | EUR | Poisoning by psy | injuries & poisonings |
| 521.2 | -0.01718 | 0.887224 | -0.01937 | 0.984548 | -1.75611 | 1.721744 | EUR | Dental abrasion, | digestive |
| 519.9 | -0.00062 | 0.03189 | -0.01933 | 0.984574 | -0.06312 | 0.061886 | EUR | Symptoms involv | respiratory |
| 721.2 | -0.00103 | 0.056578 | -0.01819 | 0.985484 | -0.11192 | 0.109862 | EUR | Spondylosis with | musculoskeletal |
| 749.1 | 0.004391 | 0.252302 | 0.017403 | 0.986115 | -0.49011 | 0.498893 | EUR | Cleft palate | congenital anomalies |
| 204.4 | -0.00139 | 0.084897 | -0.01636 | 0.98695 | -0.16778 | 0.165006 | EUR | Multiple myeloma | neoplasms |
| 252.1 | 0.00057 | 0.035867 | 0.015895 | 9.87E-01 | -0.06973 | 0.070869 | EUR | Hyperparathyroic | endocrine/metabolic |
| 994.21 | 0.000791 | 0.051828 | 0.015258 | 0.987827 | -0.10079 | 0.102372 | EUR | Septic shock | injuries & poisonings |
| 818.1 | 0.002592 | 0.172779 | 0.015002 | 0.988031 | -0.33605 | 0.341233 | EUR | Subdural hemorr | injuries & poisonings |
| 555 | 0.000553 | 0.037437 | 0.01478 | 0.988208 | -0.07282 | 0.073929 | EUR | Inflammatory bov | digestive |
| 565.1 | 0.000501 | 0.035221 | 0.014238 | 0.98864 | -0.06853 | 0.069534 | EUR | Anal and rectal p | digestive |
| 215 | -0.00055 | 0.039899 | -0.01384 | 0.988954 | -0.07875 | 0.077647 | EUR | Other benign neo | neoplasms |
| 728.71 | -0.00063 | 0.050641 | -0.0125 | 0.990028 | -0.09989 | 0.098622 | EUR | Contracture of pa | musculoskeletal |
| 586.2 | -0.00036 | 0.030951 | -0.01161 | 0.990734 | -0.06102 | 0.060303 | EUR | Cyst of kidney, ac | genitourinary |
| 973 | -0.00467 | 0.424633 | -0.011 | 0.991225 | -0.83693 | 0.827595 | EUR | Poisoning by age | injuries & poisonings |
| 592.1 | -0.00027 | 0.024812 | -0.01087 | 0.991327 | -0.0489 | 0.048361 | EUR | Cystitis | genitourinary |
| 241.2 | 0.000319 | 0.032047 | 0.009945 | 0.992065 | -0.06249 | 0.06313 | EUR | Nontoxic multino | endocrine/metabolic |
| 271.9 | 0.001296 | 0.136783 | 0.009478 | 0.992438 | -0.26679 | 0.269387 | EUR | Other disorders c | endocrine/metabolic |
| 695.81 | 0.002194 | 0.236912 | 0.009261 | 0.992611 | -0.46215 | 0.466534 | EUR | Erythema nodosu | dermatologic |
| 345.3 | -0.00037 | 0.039831 | -0.0092 | 0.99266 | -0.07843 | 0.0777 | EUR | Convulsions | neurological |
| 823 | -0.0063 | 0.734115 | -0.00858 | 0.993151 | -1.44514 | 1.432538 | EUR | Torus fracture | injuries & poisonings |
| 758 | -0.00074 | 0.090828 | -0.00809 | 0.993543 | -0.17876 | 0.177285 | EUR | Chromosomal an | congenital anomalies |
| 540 | 0.000358 | 0.061481 | 0.005824 | 0.995353 | -0.12014 | 0.120858 | EUR | Appendiceal con | digestive |
| 290.1 | 0.000231 | 0.048799 | 0.00473 | 0.996226 | -0.09541 | 0.095876 | EUR | Dementias | mental disorders |
| 202.24 | -0.00082 | 0.192571 | -0.00427 | 0.996589 | -0.37826 | 0.376609 | EUR | Large cell lympho | neoplasms |
| 149.1 | -0.00038 | 0.093152 | -0.00412 | 0.996713 | -0.18296 | 0.182191 | EUR | Cancer of oropha | neoplasms |
| 458.1 | -7.10E-05 | 0.037839 | -0.00188 | 0.998503 | -0.07423 | 0.074093 | EUR | Orthostatic hypot | circulatory system |
| 378.1 | -8.42E-05 | 0.057695 | -0.00146 | 0.998835 | -0.11316 | 0.112996 | EUR | Strabismus (not s | sense organs |

| phecode | Coef. | Std.Err. | z | p_value_z | [0.025 | 0.975] | GIA | phenotype | category |
|---|---|---|---|---|---|---|---|---|---|
| 8 | 0.039835 | 0.044816 | 8.89E-01 | 0.374083 | -0.048 | 0.127674 | AMR | Intestinal infectio | infectious diseases |
| 8.5 | 0.024453 | 0.059581 | 0.410412 | 0.681503 | -0.09232 | 0.14123 | AMR | Bacterial enteritis | infectious diseases |
| 8.51 | 0.700089 | 0.375609 | 1.863878 | 0.062339 | -0.03609 | 1.436269 | AMR | Intestinal e.coli | infectious diseases |
| 8.52 | 0.020945 | 0.091939 | 0.227818 | 0.819787 | -0.15925 | 0.201143 | AMR | Intestinal infectio | infectious diseases |
| 8.6 | -0.00433 | 0.08289 | -0.05221 | 0.958365 | -0.16679 | 0.158135 | AMR | Viral Enteritis | infectious diseases |
| 8.7 | 0.014552 | 0.362574 | 0.040136 | 0.967984 | -0.69608 | 0.725184 | AMR | Intestinal infectio | infectious diseases |
| 10 | 0.08493 | 0.109739 | 0.773923 | 0.438977 | -0.13016 | 0.300014 | AMR | Tuberculosis | infectious diseases |
| 31 | 0.207694 | 0.195367 | 1.063096 | 0.287738 | -0.17522 | 0.590607 | AMR | Diseases due to | infectious diseases |
| 31.1 | 2.002467 | 1.24296 | 1.611047 | 0.10717 | -0.43369 | 4.438624 | AMR | Leprosy | infectious diseases |
| 38 | 0.04575 | 0.040005 | 1.143593 | 0.252792 | -0.03266 | 0.124159 | AMR | Septicemia | infectious diseases |
| 38.1 | -0.00036 | 0.071852 | -0.00494 | 0.996056 | -0.14118 | 0.140473 | AMR | Gram negative se | infectious diseases |
| 38.2 | -0.13104 | 0.123074 | -1.06E+00 | 0.287014 | -0.37226 | 0.110185 | AMR | Gram positive se | infectious diseases |
| 38.3 | 0.052083 | 0.063365 | 0.821959 | 0.4111 | -0.07211 | 0.176276 | AMR | Bacteremia | infectious diseases |
| 41 | 0.026925 | 0.035524 | 0.757946 | 0.448483 | -0.0427 | 0.096551 | AMR | Bacterial infectio | infectious diseases |
| 41.1 | 0.073774 | 0.071942 | 1.025461 | 0.305146 | -0.06723 | 0.214778 | AMR | Staphylococcus i | infectious diseases |
| 41.11 | 0.03036 | 0.110072 | 0.275822 | 0.782685 | -0.18538 | 0.246097 | AMR | Methicillin sensiti | infectious diseases |
| 41.12 | -0.17889 | 0.118468 | -1.51003 | 0.131037 | -0.41108 | 0.053303 | AMR | Methicillin resista | infectious diseases |
| 41.2 | -0.00765 | 0.068192 | -0.11219 | 0.91067 | -0.1413 | 0.126003 | AMR | Streptococcus in | infectious diseases |
| 41.21 | 0.453378 | 0.27267 | 1.662733 | 0.096366 | -0.08105 | 0.987801 | AMR | Rheumatic fever | infectious diseases |
| 41.4 | 0.113768 | 0.065314 | 1.741874 | 0.08153 | -0.01424 | 0.241781 | AMR | E. coli | infectious diseases |
| 41.8 | 0.020397 | 0.072673 | 0.280671 | 0.778963 | -0.12204 | 0.162835 | AMR | H. pylori | infectious diseases |
| 41.9 | 0.022083 | 0.078953 | 0.279695 | 0.779712 | -0.13266 | 0.176828 | AMR | Infection with dru | infectious diseases |
| 53 | 0.065832 | 0.071396 | 9.22E-01 | 0.356495 | -0.0741 | 0.205766 | AMR | Herpes zoster | infectious diseases |
| 53.1 | 0.098348 | 0.130321 | 0.754666 | 0.45045 | -0.15708 | 0.353772 | AMR | Herpes zoster wi | infectious diseases |
| 54 | 0.014655 | 0.061212 | 0.239407 | 0.81079 | -0.10532 | 0.134629 | AMR | Herpes simplex | infectious diseases |
| 70 | 0.055667 | 0.067366 | 0.826348 | 0.408607 | -0.07637 | 0.187702 | AMR | Viral hepatitis | infectious diseases |
| 70.1 | 0.352488 | 0.254242 | 1.386425 | 0.165617 | -0.14582 | 0.850793 | AMR | Viral hepatitis A | infectious diseases |
| 70.2 | 0.212742 | 0.115083 | 1.848599 | 0.064516 | -0.01282 | 0.438301 | AMR | Viral hepatitis B | infectious diseases |
| 70.3 | 0.049057 | 0.080376 | 0.610347 | 0.541632 | -0.10848 | 0.206591 | AMR | Viral hepatitis C | infectious diseases |
| 70.4 | -0.04349 | 0.120143 | -0.362 | 0.717353 | -0.27897 | 0.191985 | AMR | Chronic hepatitis | infectious diseases |
| 70.9 | 0.043003 | 7.06E-02 | 0.608878 | 0.542605 | -0.09542 | 0.181428 | AMR | Hepatitis NOS | infectious diseases |
| 71 | 0.305371 | 0.133788 | 2.282501 | 0.02246 | 0.043152 | 0.567591 | AMR | Human immunod | infectious diseases |
| 71.1 | 0.337181 | 0.136034 | 2.478654 | 0.013188 | 0.070559 | 0.603802 | AMR | HIV infection, syr | infectious diseases |
| 78 | -0.05382 | 0.058149 | -9.26E-01 | 0.354701 | -0.16779 | 0.060153 | AMR | Viral warts & HP\ | infectious diseases |
| 79 | 0.017153 | 0.032778 | 0.523318 | 0.600753 | -0.04709 | 0.081398 | AMR | Viral infection | infectious diseases |
| 79.1 | -0.02737 | 0.077686 | -3.52E-01 | 0.724608 | -0.17963 | 0.124893 | AMR | Varicella infectior | infectious diseases |
| 79.2 | 0.016889 | 0.170594 | 0.099003 | 0.921136 | -0.31747 | 0.351247 | AMR | Infectious monon | infectious diseases |

| | | | | | | | | | |
|---|---|---|---|---|---|---|---|---|---|
| 79.9 | -0.07459 | 0.06456 | -1.15535 | 0.247948 | -0.20112 | 0.051946 | AMR | Viremia, NOS | infectious diseases |
| 80 | -0.04237 | 0.091712 | -0.462 | 0.644085 | -0.22212 | 0.137381 | AMR | Postoperative inf | infectious diseases |
| 81 | 0.060213 | 0.075087 | 0.801903 | 0.422609 | -0.08696 | 0.20738 | AMR | Infection/inflamm | infectious diseases |
| 81.1 | -0.03344 | 0.189299 | -0.17665 | 0.859782 | -0.40446 | 0.337579 | AMR | Graft-versus-hos | infectious diseases |
| 81.11 | 0.342215 | 0.359789 | 0.951157 | 0.341525 | -0.36296 | 1.047388 | AMR | Acute graft-versu | infectious diseases |
| 81.12 | 0.48483 | 0.279473 | 1.7348 | 0.082776 | -0.06293 | 1.032587 | AMR | Chronic graft-ver | infectious diseases |
| 90 | 0.023437 | 0.092613 | 0.253065 | 0.800218 | -0.15808 | 0.204955 | AMR | Sexually transmit | infectious diseases |
| 90.2 | 0.109867 | 0.268094 | 4.10E-01 | 0.681947 | -0.41559 | 0.63532 | AMR | Gonococcal infec | infectious diseases |
| 90.3 | 0.456387 | 0.447611 | 1.019605 | 0.307916 | -0.42092 | 1.333689 | AMR | Venereal disease | infectious diseases |
| 110 | 0.069523 | 0.043706 | 1.59068 | 0.111682 | -0.01614 | 0.155185 | AMR | Dermatophytosis | infectious diseases |
| 110.1 | 0.064249 | 0.044626 | 1.439702 | 0.149952 | -0.02322 | 0.151715 | AMR | Dermatophytosis | infectious diseases |
| 110.11 | 0.09787 | 0.052601 | 1.860611 | 0.062799 | -0.00523 | 0.200965 | AMR | Dermatophytosis | infectious diseases |
| 110.12 | 0.093794 | 0.079567 | 1.18E+00 | 0.238478 | -0.06215 | 0.249742 | AMR | Althete's foot | infectious diseases |
| 110.13 | 0.026144 | 0.089305 | 0.292747 | 0.769716 | -0.14889 | 0.201179 | AMR | Dermatophytosis | infectious diseases |
| 110.2 | 0.009532 | 0.134247 | 0.071002 | 0.943396 | -0.25359 | 0.27265 | AMR | Dermatomycoses | infectious diseases |
| 112 | 0.082558 | 0.051119 | 1.615027 | 0.106305 | -0.01763 | 0.182749 | AMR | Candidiasis | infectious diseases |
| 112.3 | 0.268586 | 0.138084 | 1.945096 | 0.051763 | -0.00205 | 0.539225 | AMR | Candidiasis of sk | infectious diseases |
| 117 | 0.049617 | 0.080789 | 0.614153 | 0.539115 | -0.10873 | 0.20796 | AMR | Mycoses | infectious diseases |
| 117.1 | 0.383187 | 0.530812 | 7.22E-01 | 0.470364 | -0.65719 | 1.42356 | AMR | Histoplasmosis | infectious diseases |
| 117.2 | -0.13851 | 0.175278 | -0.79025 | 0.42938 | -0.48205 | 0.205024 | AMR | Coccidioidomyco | infectious diseases |
| 117.3 | 0.00186 | 1.029821 | 0.001806 | 0.998559 | -2.01655 | 2.020272 | AMR | Blastomycotic inf | infectious diseases |
| 117.4 | 0.144038 | 0.140399 | 1.025923 | 0.304928 | -0.13114 | 0.419215 | AMR | Aspergillosis | infectious diseases |
| 130 | 0.277626 | 0.243743 | 1.13901 | 0.254699 | -0.2001 | 0.755353 | AMR | Spirochetal infec | infectious diseases |
| 130.1 | -0.11878 | 0.492659 | -0.24109 | 0.809484 | -1.08437 | 0.846817 | AMR | Lyme disease | infectious diseases |
| 131 | 0.375079 | 0.210497 | 1.781874 | 0.07477 | -0.03749 | 0.787646 | AMR | Protozoan infecti | infectious diseases |
| 132 | -0.21073 | 0.159793 | -1.31879 | 0.187238 | -0.52392 | 0.102455 | AMR | Infestation (lice, r | infectious diseases |
| 132.1 | 0.590533 | 0.553487 | 1.066932 | 0.286002 | -0.49428 | 1.675348 | AMR | Pediculosis and p | infectious diseases |
| 133 | 0.571477 | 0.218678 | 2.613321 | 0.008967 | 0.142875 | 1.000078 | AMR | Arthropod-borne | infectious diseases |
| 134 | -0.03847 | 1.74E-01 | -0.22163 | 0.824603 | -0.37872 | 0.301769 | AMR | Helminthiases | infectious diseases |
| 134.1 | 0.156606 | 0.199848 | 7.84E-01 | 0.43326 | -0.23509 | 0.5483 | AMR | Intestinal helmint | infectious diseases |
| 136 | 0.060407 | 0.038815 | 1.556259 | 0.119647 | -0.01567 | 0.136483 | AMR | Other infectious a | infectious diseases |
| 145 | 0.043641 | 0.139177 | 0.313565 | 0.753851 | -0.22914 | 0.316423 | AMR | Cancer of mouth | neoplasms |
| 145.1 | 0.600769 | 0.523722 | 1.147113 | 0.251335 | -0.42571 | 1.627245 | AMR | Cancer of lip | neoplasms |
| 145.2 | 0.416702 | 0.204028 | 2.042371 | 0.041115 | 0.016813 | 0.81659 | AMR | Cancer of tongue | neoplasms |
| 145.3 | 0.245062 | 0.215411 | 1.137646 | 0.255268 | -0.17714 | 0.66726 | AMR | Cancer of major s | neoplasms |
| 145.4 | 0.100908 | 0.378037 | 0.266927 | 0.789525 | -0.64003 | 0.841847 | AMR | Cancer of the gu | neoplasms |
| 145.5 | 0.189503 | 0.328076 | 0.577621 | 0.56352 | -0.45351 | 0.83252 | AMR | Cancer of the mo | neoplasms |
| 149 | -0.06072 | 0.135264 | -0.4489 | 0.653505 | -0.32583 | 0.204393 | AMR | Cancer of larynx, | neoplasms |

| | | | | | | | | | | |
|---|---|---|---|---|---|---|---|---|---|---|
| 149.1 | 0.173142 | 0.25445 | 0.680457 | 0.496215 | -0.32557 | 0.671855 | AMR | Cancer of orophа | neoplasms | |
| 149.2 | -0.03095 | 0.31578 | -0.09802 | 0.921913 | -0.64987 | 0.587963 | AMR | Cancer of nasopl | neoplasms | |
| 149.3 | 0.870574 | 0.458705 | 1.90E+00 | 0.05771 | -0.02847 | 1.769619 | AMR | Cancer of hypopl | neoplasms | |
| 149.4 | 0.411866 | 0.281522 | 1.463 | 0.143467 | -0.13991 | 0.963639 | AMR | Cancer of larynx | neoplasms | |
| 149.5 | -0.34088 | 0.165814 | -2.05582 | 0.0398 | -0.66587 | -0.01589 | AMR | Hx of malignant r | neoplasms | |
| 149.9 | 0.661176 | 0.246636 | 2.68078 | 0.007345 | 0.177779 | 1.144573 | AMR | Cancer of of nasa | neoplasms | |
| 150 | -0.203 | 0.259643 | -0.78183 | 0.434315 | -0.71189 | 0.305894 | AMR | Cancer of esopha | neoplasms | |
| 151 | -0.12368 | 0.176066 | -0.70245 | 0.482397 | -0.46876 | 0.221405 | AMR | Cancer of stoma | neoplasms | |
| 153 | 0.040821 | 0.083459 | 0.489112 | 0.624762 | -0.12276 | 0.204397 | AMR | Colorectal cance | neoplasms | |
| 153.2 | 0.044856 | 1.02E-01 | 0.441858 | 0.658592 | -0.15411 | 0.243825 | AMR | Colon cancer | neoplasms | |
| 153.3 | -0.04605 | 1.03E-01 | -0.44682 | 0.655004 | -0.24807 | 0.155961 | AMR | Malignant neopla | neoplasms | |
| 155 | 0.05897 | 0.074743 | 0.788975 | 0.430127 | -0.08752 | 0.205464 | AMR | Cancer of liver ar | neoplasms | |
| 155.1 | 0.06553 | 7.86E-02 | 0.833254 | 0.404701 | -0.08861 | 0.219669 | AMR | Malignant neopla | neoplasms | |
| 157 | 0.236325 | 0.153993 | 1.534649 | 0.12487 | -0.0655 | 0.538146 | AMR | Pancreatic cance | neoplasms | |
| 158 | 0.2606 | 0.105934 | 2.460013 | 0.013893 | 0.052972 | 0.468227 | AMR | Neoplasm of uns | neoplasms | |
| 159 | -0.05031 | 0.109081 | -0.46124 | 0.644629 | -0.26411 | 0.163483 | AMR | Malignant neopla | neoplasms | |
| 159.2 | 0.573979 | 0.270698 | 2.12037 | 0.033975 | 0.043422 | 1.104537 | AMR | Malignant neopla | neoplasms | |
| 159.3 | -0.01845 | 0.236546 | -0.07799 | 0.937835 | -0.48207 | 0.445173 | AMR | Malignant neopla | neoplasms | |
| 159.4 | -0.09164 | 0.173781 | -0.52736 | 0.597947 | -0.43225 | 0.24896 | AMR | Malignant neopla | neoplasms | |
| 164 | 0.623468 | 0.274848 | 2.268413 | 0.023304 | 0.084776 | 1.16216 | AMR | Cancer of intrath | neoplasms | |
| 165 | -0.05648 | 0.105468 | -0.53551 | 0.592297 | -0.26319 | 0.150234 | AMR | Cancer within the | neoplasms | |
| 165.1 | -0.0899 | 1.09E-01 | -0.82408 | 0.409897 | -0.30372 | 0.123917 | AMR | Cancer of bronch | neoplasms | |
| 170 | -0.24118 | 9.68E-02 | -2.4903 | 0.012764 | -0.431 | -0.05136 | AMR | Cancer of bone a | neoplasms | |
| 170.1 | -0.30946 | 1.37E-01 | -2.25503 | 0.024132 | -0.57844 | -0.04049 | AMR | Bone cancer | neoplasms | |
| 170.2 | -0.19667 | 0.115768 | -1.70E+00 | 0.089346 | -0.42358 | 0.030228 | AMR | Cancer of conne | neoplasms | |
| 172 | -0.07295 | 0.075542 | -9.66E-01 | 0.334224 | -0.22101 | 0.075113 | AMR | Skin cancer | neoplasms | |
| 172.1 | 0.177003 | 0.160065 | 1.11E+00 | 0.268805 | -0.13672 | 0.490724 | AMR | Melanomas of sk | neoplasms | |
| 172.11 | -0.03438 | 0.185932 | -1.85E-01 | 0.853304 | -0.3988 | 0.33004 | AMR | Melanomas of sk | neoplasms | |
| 172.2 | 0.00257 | 0.081087 | 0.031691 | 0.974719 | -0.15636 | 0.161497 | AMR | Other non-epithe | neoplasms | |
| 172.21 | 0.065236 | 0.120778 | 0.540131 | 0.589107 | -0.17148 | 0.301957 | AMR | Basal cell carcino | neoplasms | |
| 172.22 | -0.129 | 0.125142 | -1.03082 | 0.302624 | -0.37427 | 0.116274 | AMR | Squamous cell ca | neoplasms | |
| 172.3 | -0.19063 | 0.155752 | -1.22395 | 0.220969 | -0.4959 | 0.114635 | AMR | Carcinoma in situ | neoplasms | |
| 173 | -0.15006 | 0.063066 | -2.37937 | 0.017342 | -0.27366 | -0.02645 | AMR | Neoplasm of unc | neoplasms | |
| 174 | -0.01483 | 0.076096 | -0.19495 | 0.845435 | -0.16398 | 0.13431 | AMR | Breast cancer | neoplasms | |
| 174.1 | -0.01691 | 0.077445 | -0.21838 | 0.827134 | -0.1687 | 0.134878 | AMR | Breast cancer [fe | neoplasms | |
| 174.11 | -0.02037 | 0.077989 | -2.61E-01 | 0.793903 | -0.17323 | 0.132482 | AMR | Malignant neopla | neoplasms | |
| 174.2 | -1.59923 | 0.50028 | -3.19667 | 0.00139 | -2.57976 | -0.6187 | AMR | Breast cancer [m | neoplasms | |
| 174.3 | 0.44326 | 0.448186 | 9.89E-01 | 0.322659 | -0.43517 | 1.321689 | AMR | Neoplasm of unc | neoplasms | |

| | | | | | | | | | | |
|---|---|---|---|---|---|---|---|---|---|---|
| 175 | -0.07359 | 0.091582 | -0.80357 | 0.421648 | -0.25309 | 0.105905 | AMR | Acquired absenc | neoplasms | |
| 180 | 0.005401 | 0.092618 | 0.058316 | 0.953497 | -0.17613 | 0.186929 | AMR | Cervical cancer a | neoplasms | |
| 180.1 | -0.03459 | 0.163193 | -0.21195 | 0.832146 | -0.35444 | 0.285263 | AMR | Cervical cancer | neoplasms | |
| 180.3 | -0.01567 | 0.105767 | -0.14818 | 0.882203 | -0.22297 | 0.191628 | AMR | Cervical intraepit | neoplasms | |
| 182 | 0.003892 | 0.127916 | 0.030424 | 0.975729 | -0.24682 | 0.254602 | AMR | Malignant neopla | neoplasms | |
| 184 | 0.004856 | 0.11823 | 0.041076 | 0.967236 | -0.22687 | 0.236583 | AMR | Cancer of other f | neoplasms | |
| 184.1 | 0.073084 | 0.128675 | 0.56797 | 0.570056 | -0.17912 | 0.325283 | AMR | Malignant neopla | neoplasms | |
| 184.11 | 0.099747 | 0.136568 | 0.730383 | 0.465156 | -0.16792 | 0.367415 | AMR | Malignant neopla | neoplasms | |
| 184.2 | 0.015755 | 0.23195 | 6.79E-02 | 0.945847 | -0.43886 | 0.470369 | AMR | Cancer of other f | neoplasms | |
| 185 | -0.0741 | 0.103889 | -0.71331 | 0.475656 | -0.27772 | 0.129514 | AMR | Cancer of prosta | neoplasms | |
| 187 | 0.233411 | 0.168537 | 1.38E+00 | 0.166075 | -0.09691 | 0.563737 | AMR | Cancer of other r | neoplasms | |
| 187.1 | 1.545403 | 0.403969 | 3.825546 | 0.00013 | 0.753638 | 2.337169 | AMR | Malignant neopla | neoplasms | |
| 187.2 | 0.421925 | 0.214745 | 1.964774 | 0.04944 | 0.001033 | 0.842818 | AMR | Malignant neopla | neoplasms | |
| 187.8 | 1.167779 | 0.512103 | 2.28036 | 0.022586 | 0.164076 | 2.171483 | AMR | Neoplasm of unc | neoplasms | |
| 189 | -0.02505 | 0.091881 | -0.27268 | 0.785099 | -0.20514 | 0.15503 | AMR | Cancer of urinary | neoplasms | |
| 189.1 | -0.10439 | 0.102916 | -1.01432 | 0.310431 | -0.3061 | 0.097322 | AMR | Cancer of kidney | neoplasms | |
| 189.11 | -0.02196 | 0.106364 | -0.20645 | 0.836443 | -0.23043 | 0.186511 | AMR | Malignant neopla | neoplasms | |
| 189.12 | -0.04071 | 0.262917 | -0.15486 | 0.876935 | -0.55602 | 0.474594 | AMR | Malignant neopla | neoplasms | |
| 189.2 | 0.031351 | 1.81E-01 | 0.173515 | 0.862246 | -0.32278 | 0.385478 | AMR | Cancer of bladde | neoplasms | |
| 189.21 | 0.399848 | 0.18728 | 2.14E+00 | 0.032759 | 0.032785 | 0.766911 | AMR | Malignant neopla | neoplasms | |
| 189.4 | 0.362867 | 0.268704 | 1.35043 | 0.176878 | -0.16378 | 0.889518 | AMR | Malignant neopla | neoplasms | |
| 190 | 0.508314 | 0.253668 | 2.00E+00 | 0.045086 | 0.011134 | 1.005495 | AMR | Cancer of eye | neoplasms | |
| 191 | 0.113893 | 0.098775 | 1.153054 | 0.248888 | -0.0797 | 0.307489 | AMR | Manlignant and u | neoplasms | |
| 191.1 | 0.070759 | 0.117902 | 0.60015 | 0.548406 | -0.16032 | 0.301842 | AMR | Cancer of brain a | neoplasms | |
| 191.11 | 0.008799 | 0.129324 | 0.068042 | 0.945752 | -0.24467 | 0.262269 | AMR | Cancer of brain | neoplasms | |
| 193 | -0.08895 | 0.104581 | -0.85052 | 0.395035 | -0.29392 | 0.116026 | AMR | Thyroid cancer | neoplasms | |
| 194 | 0.407798 | 0.198444 | 2.054975 | 0.039881 | 0.018854 | 0.796741 | AMR | Cancer of other e | neoplasms | |
| 195 | -0.09029 | 0.043113 | -2.09433 | 0.036231 | -0.17479 | -0.00579 | AMR | Cancer, suspecte | neoplasms | |
| 195.1 | -0.10378 | 0.045355 | -2.28814 | 0.022129 | -0.19267 | -0.01488 | AMR | Malignant neopla | neoplasms | |
| 195.3 | -0.20612 | 0.137439 | -1.49973 | 0.133685 | -0.4755 | 0.063255 | AMR | Malignant neopla | neoplasms | |
| 196 | 0.035866 | 0.034187 | 1.05E+00 | 0.294131 | -0.03114 | 0.102871 | AMR | Radiotherapy | neoplasms | |
| 197 | 0.023292 | 0.032409 | 0.718701 | 0.472325 | -0.04023 | 0.086812 | AMR | Chemotherapy | neoplasms | |
| 198 | -0.11191 | 0.052726 | -2.12E+00 | 0.033797 | -0.21525 | -0.00857 | AMR | Secondary malig | neoplasms | |
| 198.1 | -0.11535 | 0.068946 | -1.67306 | 0.094315 | -0.25048 | 0.019781 | AMR | Secondary malig | neoplasms | |
| 198.2 | -0.16538 | 0.102446 | -1.6143 | 0.106463 | -0.36617 | 0.035412 | AMR | Secondary malig | neoplasms | |
| 198.3 | -0.04003 | 0.107488 | -0.37244 | 0.709562 | -0.25071 | 0.170639 | AMR | Secondary malig | neoplasms | |
| 198.4 | -0.17885 | 0.109286 | -1.6365 | 0.101734 | -0.39304 | 0.03535 | AMR | Secondary malig | neoplasms | |
| 198.5 | -0.08447 | 0.136574 | -0.61847 | 0.536265 | -0.35215 | 0.183213 | AMR | Secondary malig | neoplasms | |

| | | | | | | | | | | |
|---|---|---|---|---|---|---|---|---|---|---|
| 198.6 | -0.09537 | 0.100694 | -0.94709 | 0.343591 | -0.29272 | 0.10199 | AMR | Secondary malig | neoplasms | |
| 198.7 | 0.761977 | 0.209093 | 3.644203 | 0.000268 | 0.352162 | 1.171791 | AMR | Secondary malig | neoplasms | |
| 199 | -0.02564 | 0.049707 | -0.51588 | 0.605941 | -0.12307 | 0.071782 | AMR | Neoplasm of unc | neoplasms | |
| 199.4 | 0.256429 | 0.243023 | 1.06E+00 | 0.29135 | -0.21989 | 0.732746 | AMR | Neurofibromatosi | neoplasms | |
| 200 | 0.071566 | 0.080904 | 8.85E-01 | 0.376382 | -0.087 | 0.230135 | AMR | Myeloproliferative | neoplasms | |
| 200.1 | -0.02182 | 0.175181 | -1.25E-01 | 0.900856 | -0.36517 | 0.321524 | AMR | Polycythemia ver | neoplasms | |
| 201 | 0.416577 | 0.244754 | 1.702027 | 0.08875 | -0.06313 | 0.896286 | AMR | Hodgkin's diseas | neoplasms | |
| 202 | -0.18576 | 0.097436 | -1.90651 | 0.056584 | -0.37673 | 0.005208 | AMR | Cancer of other l | neoplasms | |
| 202.2 | -0.14753 | 0.103812 | -1.42111 | 0.155284 | -0.351 | 0.055939 | AMR | Non-Hodgkins ly | neoplasms | |
| 202.21 | -0.58507 | 0.235056 | -2.48906 | 0.012808 | -1.04577 | -0.12437 | AMR | Nodular lymphon | neoplasms | |
| 202.22 | -0.19602 | 0.161068 | -1.21698 | 0.223614 | -0.5117 | 0.119672 | AMR | Reticulosarcoma | neoplasms | |
| 202.23 | 0.784995 | 0.467227 | 1.680115 | 0.092935 | -0.13075 | 1.700743 | AMR | Lymphosarcoma | neoplasms | |
| 202.24 | -0.00757 | 0.285414 | -0.02652 | 0.978844 | -0.56697 | 0.551833 | AMR | Large cell lymph | neoplasms | |
| 204 | -0.02684 | 0.097684 | -0.27478 | 0.783488 | -0.2183 | 0.164615 | AMR | Leukemia | neoplasms | |
| 204.1 | -0.10899 | 0.159384 | -6.84E-01 | 0.494093 | -0.42138 | 0.203398 | AMR | Lymphoid leuken | neoplasms | |
| 204.11 | -0.03545 | 0.176731 | -0.20056 | 0.84104 | -0.38183 | 0.31094 | AMR | Lymphoid leuken | neoplasms | |
| 204.12 | 0.046823 | 0.290016 | 1.61E-01 | 0.87174 | -0.5216 | 0.615245 | AMR | Lymphoid leuken | neoplasms | |
| 204.2 | 0.079685 | 0.154331 | 0.516322 | 0.60563 | -0.2228 | 0.382168 | AMR | Myeloid leukemia | neoplasms | |
| 204.21 | 0.052478 | 0.170941 | 0.306992 | 0.75885 | -0.28256 | 0.387516 | AMR | Myeloid leukemia | neoplasms | |
| 204.22 | -0.2486 | 0.312593 | -0.79528 | 0.426452 | -0.86127 | 0.364074 | AMR | Myeloid leukemia | neoplasms | |
| 204.3 | 0.399159 | 0.69414 | 0.575041 | 0.565263 | -0.96133 | 1.759647 | AMR | Monocytic leuker | neoplasms | |
| 204.4 | -0.09863 | 0.159226 | -0.61944 | 0.535628 | -0.41071 | 0.213447 | AMR | Multiple myeloma | neoplasms | |
| 208 | 0.064384 | 0.04298 | 1.498 | 0.134133 | -0.01986 | 0.148623 | AMR | Benign neoplasm | neoplasms | |
| 209 | 0.12929 | 0.159627 | 0.80995 | 0.417969 | -0.18357 | 0.442153 | AMR | Neuroendocrine t | neoplasms | |
| 210 | 0.111261 | 0.184996 | 0.601426 | 0.547556 | -0.25132 | 0.473847 | AMR | Benign neoplasm | neoplasms | |
| 211 | 0.046534 | 0.06639 | 7.01E-01 | 0.483358 | -0.08359 | 0.176656 | AMR | Benign neoplasm | neoplasms | |
| 212 | 0.339816 | 0.199151 | 1.706325 | 0.087948 | -0.05051 | 0.730144 | AMR | Benign neoplasm | neoplasms | |
| 213 | -0.00168 | 0.171487 | -0.00979 | 0.99219 | -0.33779 | 0.334431 | AMR | Benign neoplasm | neoplasms | |
| 214 | 0.039095 | 0.077366 | 0.505332 | 0.613326 | -0.11254 | 0.190729 | AMR | Lipoma | neoplasms | |
| 214.1 | 0.028173 | 0.100894 | 0.279234 | 0.780065 | -0.16958 | 0.225921 | AMR | Lipoma of skin ar | neoplasms | |
| 215 | 0.195242 | 0.073054 | 2.672571 | 0.007527 | 0.052059 | 0.338426 | AMR | Other benign neo | neoplasms | |
| 216 | -0.03122 | 0.042237 | -0.73926 | 0.45975 | -0.11401 | 0.051559 | AMR | Benign neoplasm | neoplasms | |
| 216.1 | 0.017051 | 0.058575 | 0.291095 | 0.770979 | -0.09775 | 0.131856 | AMR | Screening for ma | neoplasms | |
| 217 | 0.054409 | 0.077668 | 0.700533 | 0.483595 | -0.09782 | 0.206636 | AMR | Vascular hamarto | neoplasms | |
| 217.1 | 0.028853 | 0.079772 | 0.361696 | 0.717579 | -0.1275 | 0.185204 | AMR | Nevus, non-neop | neoplasms | |
| 218 | 0.034897 | 0.05888 | 0.592678 | 0.553397 | -0.08051 | 0.150299 | AMR | Benign neoplasm | neoplasms | |
| 218.1 | 0.034473 | 0.058946 | 5.85E-01 | 0.558671 | -0.08106 | 0.150006 | AMR | Uterine leiomyon | neoplasms | |
| 218.2 | 0.362137 | 0.346831 | 1.044129 | 0.296426 | -0.31764 | 1.041914 | AMR | Other benign neo | neoplasms | |

| | | | | | | | | | | |
|---|---|---|---|---|---|---|---|---|---|---|
| 220 | -0.05379 | 0.155389 | -3.46E-01 | 0.729208 | -0.35835 | 0.250765 | AMR | Benign neoplasm | neoplasms | |
| 221 | 0.039647 | 0.305811 | 0.129646 | 0.896847 | -0.55973 | 0.639025 | AMR | Benign neoplasm | neoplasms | |
| 222 | 0.633158 | 0.401696 | 1.576213 | 0.114977 | -0.15415 | 1.420467 | AMR | Benign neoplasm | neoplasms | |
| 223 | 0.239403 | 0.20824 | 1.149647 | 0.250289 | -0.16874 | 0.647546 | AMR | Benign neoplasm | neoplasms | |
| 224 | 0.30239 | 0.168405 | 1.795614 | 0.072556 | -0.02768 | 0.632456 | AMR | Benign neoplasm | neoplasms | |
| 224.1 | -0.10048 | 0.197048 | -0.50992 | 0.610111 | -0.48668 | 0.285729 | AMR | Benign neoplasm | neoplasms | |
| 225 | 0.159965 | 0.102317 | 1.563431 | 0.117951 | -0.04057 | 0.360502 | AMR | Benign neoplasm | neoplasms | |
| 225.1 | 0.136357 | 0.105447 | 1.293137 | 0.195964 | -0.07031 | 0.343028 | AMR | Benign neoplasm | neoplasms | |
| 225.2 | 0.975886 | 0.297688 | 3.278217 | 0.001045 | 0.392428 | 1.559344 | AMR | Benign neoplasm | neoplasms | |
| 226 | 0.275792 | 0.34074 | 8.09E-01 | 0.41829 | -0.39205 | 0.94363 | AMR | Benign neoplasm | neoplasms | |
| 227 | 0.060623 | 0.093332 | 0.649547 | 0.515985 | -0.1223 | 0.24355 | AMR | Benign neoplasm | neoplasms | |
| 227.1 | 0.321472 | 0.172482 | 1.863805 | 0.062349 | -0.01659 | 0.65953 | AMR | Benign neoplasm | neoplasms | |
| 227.2 | -0.19057 | 0.172526 | -1.10459 | 0.269337 | -0.52872 | 0.147574 | AMR | Benign neoplasm | neoplasms | |
| 227.3 | 0.066666 | 0.142547 | 0.467681 | 0.640013 | -0.21272 | 0.346053 | AMR | Benign neoplasm | neoplasms | |
| 228 | -0.12339 | 0.058863 | -2.09627 | 0.036058 | -0.23876 | -0.00802 | AMR | Hemangioma and | neoplasms | |
| 228.1 | -0.10574 | 0.06766 | -1.56274 | 0.118113 | -0.23835 | 0.026876 | AMR | Hemangioma of s | neoplasms | |
| 229 | -0.01393 | 0.123018 | -0.11323 | 0.909848 | -0.25504 | 0.227181 | AMR | Benign neoplasm | neoplasms | |
| 229.1 | 0.918677 | 8.90E-01 | 1.032077 | 0.302036 | -0.82593 | 2.663288 | AMR | Benign neoplasm | neoplasms | |
| 230 | 0.857333 | 0.519807 | 1.649328 | 0.09908 | -0.16147 | 1.876137 | AMR | Kaposi's sarcoma | neoplasms | |
| 240 | -0.14602 | 0.112108 | -1.30247 | 0.192756 | -0.36575 | 0.073711 | AMR | Simple and unsp | endocrine/metabolic | |
| 241 | -0.09593 | 0.055839 | -1.72E+00 | 0.085807 | -0.20537 | 0.013514 | AMR | Nontoxic nodular | endocrine/metabolic | |
| 241.1 | -0.09767 | 0.062953 | -1.55E+00 | 0.1208 | -0.22105 | 0.025719 | AMR | Nontoxic uninodu | endocrine/metabolic | |
| 241.2 | -0.12529 | 0.080067 | -1.56E+00 | 0.117635 | -0.28221 | 0.031641 | AMR | Nontoxic multino | endocrine/metabolic | |
| 242 | 0.038835 | 0.097347 | 0.398932 | 0.689943 | -0.15196 | 0.229631 | AMR | Thyrotoxicosis wi | endocrine/metabolic | |
| 242.1 | 0.041441 | 0.184361 | 0.224783 | 0.822148 | -0.3199 | 0.402781 | AMR | Graves' disease | endocrine/metabolic | |
| 242.2 | 0.286718 | 0.350009 | 0.819173 | 0.412688 | -0.39929 | 0.972723 | AMR | Toxic multinodula | endocrine/metabolic | |
| 242.3 | -0.11292 | 0.214337 | -0.52685 | 0.598296 | -0.53302 | 0.307169 | AMR | Exophthalmos | endocrine/metabolic | |
| 242.31 | 0.12071 | 0.470227 | 0.256706 | 0.797406 | -0.80092 | 1.042338 | AMR | Thyrotoxic exoph | endocrine/metabolic | |
| 244 | -0.04512 | 0.041495 | -1.08734 | 0.276885 | -0.12645 | 0.036209 | AMR | Hypothyroidism | endocrine/metabolic | |
| 244.1 | 0.035276 | 0.084734 | 0.41631 | 0.677183 | -0.1308 | 0.201352 | AMR | Secondary hypot | endocrine/metabolic | |
| 244.2 | -0.03092 | 0.063152 | -0.48957 | 0.624442 | -0.15469 | 0.092858 | AMR | Acquired hypothy | endocrine/metabolic | |
| 244.4 | -0.08556 | 0.042724 | -2.00E+00 | 0.045217 | -0.1693 | -0.00182 | AMR | Hypothyroidism N | endocrine/metabolic | |
| 244.5 | 0.385112 | 0.274394 | 1.4035 | 0.160468 | -0.15269 | 0.922915 | AMR | Congenital hypot | endocrine/metabolic | |
| 245 | 0.124512 | 0.082263 | 1.51358 | 0.130133 | -0.03672 | 0.285745 | AMR | Thyroiditis | endocrine/metabolic | |
| 245.1 | 0.499803 | 0.461913 | 1.082027 | 0.279241 | -0.40553 | 1.405137 | AMR | Thyroiditis, acute | endocrine/metabolic | |
| 245.2 | 0.123699 | 0.090127 | 1.372505 | 0.169906 | -0.05295 | 0.300345 | AMR | Chronic thyroiditi | endocrine/metabolic | |
| 245.21 | 0.132767 | 0.09047 | 1.467516 | 0.142236 | -0.04455 | 0.310085 | AMR | Chronic lymphoc | endocrine/metabolic | |
| 246 | -0.0176 | 0.061497 | -0.28616 | 0.774759 | -0.13813 | 0.102934 | AMR | Other disorders c | endocrine/metabolic | |

| | | | | | | | | | |
|---|---|---|---|---|---|---|---|---|---|
| 246.2 | 0.141149 | 0.232952 | 0.605914 | 0.544572 | -0.31543 | 0.597726 | AMR | Thyroid cyst | endocrine/metabolic |
| 246.7 | -0.17449 | 0.085683 | -2.0364 | 0.041711 | -0.34242 | -0.00655 | AMR | Abnormal results | endocrine/metabolic |
| 249 | 0.043142 | 0.053902 | 0.800388 | 0.423486 | -0.0625 | 0.148788 | AMR | Secondary diabe | endocrine/metabolic |
| 250 | 0.035955 | 0.035433 | 1.014744 | 0.310228 | -0.03349 | 0.105403 | AMR | Diabetes mellitus | endocrine/metabolic |
| 250.1 | 0.079455 | 0.087306 | 9.10E-01 | 0.362785 | -0.09166 | 0.250572 | AMR | Type 1 diabetes | endocrine/metabolic |
| 250.11 | 0.039976 | 0.339624 | 0.117706 | 0.906301 | -0.62568 | 0.705627 | AMR | Type 1 diabetes | endocrine/metabolic |
| 250.12 | 0.312668 | 0.179145 | 1.75E+00 | 0.080927 | -0.03845 | 0.663786 | AMR | Type 1 diabetes | endocrine/metabolic |
| 250.13 | 0.554286 | 0.204741 | 2.707248 | 0.006784 | 0.153 | 0.955571 | AMR | Type 1 diabetes | endocrine/metabolic |
| 250.14 | 0.037429 | 0.163081 | 0.22951 | 0.818472 | -0.2822 | 0.357061 | AMR | Type 1 diabetes | endocrine/metabolic |
| 250.15 | 0.761822 | 0.244625 | 3.114248 | 0.001844 | 0.282366 | 1.241278 | AMR | Diabetes type 1 | endocrine/metabolic |
| 250.2 | 0.05119 | 0.035978 | 1.422813 | 0.15479 | -0.01933 | 0.121707 | AMR | Type 2 diabetes | endocrine/metabolic |
| 250.21 | -0.09609 | 0.176135 | -0.54554 | 0.58538 | -0.44131 | 0.249129 | AMR | Type 2 diabetes | endocrine/metabolic |
| 250.22 | 0.104648 | 0.048101 | 2.175609 | 0.029585 | 0.010373 | 0.198924 | AMR | Type 2 diabetes | endocrine/metabolic |
| 250.23 | 0.066369 | 0.06234 | 1.064632 | 0.287043 | -0.05581 | 0.188553 | AMR | Type 2 diabetes | endocrine/metabolic |
| 250.24 | 0.046508 | 0.054256 | 0.857202 | 0.391333 | -0.05983 | 0.152848 | AMR | Type 2 diabetes | endocrine/metabolic |
| 250.25 | 0.020131 | 0.061797 | 3.26E-01 | 0.744607 | -0.10099 | 0.14125 | AMR | Diabetes type 2 | endocrine/metabolic |
| 250.3 | 0.026454 | 0.045067 | 0.587 | 0.557204 | -0.06187 | 0.114783 | AMR | Insulin pump use | endocrine/metabolic |
| 250.4 | 0.050032 | 0.032638 | 1.532938 | 0.125291 | -0.01394 | 0.114002 | AMR | Abnormal glucos | endocrine/metabolic |
| 250.41 | -0.00066 | 0.062451 | -0.01062 | 0.991525 | -0.12307 | 0.121739 | AMR | Impaired fasting | endocrine/metabolic |
| 250.42 | 0.044275 | 0.032964 | 1.343143 | 0.179226 | -0.02033 | 0.108882 | AMR | Other abnormal | endocrine/metabolic |
| 250.5 | 0.219663 | 0.185482 | 1.18E+00 | 0.2363 | -0.14387 | 0.583201 | AMR | Glycosuria or Ac | endocrine/metabolic |
| 250.6 | 0.085699 | 0.060461 | 1.417435 | 0.156356 | -0.0328 | 0.204199 | AMR | Polyneuropathy i | endocrine/metabolic |
| 250.7 | 0.071628 | 0.069666 | 1.028166 | 0.303872 | -0.06491 | 0.208171 | AMR | Diabetic retinopa | endocrine/metabolic |
| 251 | 0.251675 | 0.269531 | 0.933752 | 0.350432 | -0.2766 | 0.779945 | AMR | Other disorders c | endocrine/metabolic |
| 251.1 | 0.101041 | 0.077984 | 1.295653 | 0.195095 | -0.05181 | 0.253887 | AMR | Hypoglycemia | endocrine/metabolic |
| 251.8 | 0.244085 | 0.420625 | 0.580292 | 0.561718 | -0.58032 | 1.068494 | AMR | Abnormality of se | endocrine/metabolic |
| 252 | 0.057343 | 0.060001 | 9.56E-01 | 0.339228 | -0.06026 | 0.174944 | AMR | Disorders of para | endocrine/metabolic |
| 252.1 | 0.056185 | 0.062248 | 0.902601 | 0.366738 | -0.06582 | 0.178188 | AMR | Hyperparathyroic | endocrine/metabolic |
| 252.2 | 0.079355 | 0.132897 | 0.597112 | 0.550433 | -0.18112 | 0.339828 | AMR | Hypoparathyroidi | endocrine/metabolic |
| 253 | 0.030401 | 0.069886 | 0.435004 | 0.663559 | -0.10657 | 0.167376 | AMR | Disorders of the | endocrine/metabolic |
| 253.1 | -0.03903 | 0.125114 | -0.31195 | 0.755076 | -0.28425 | 0.20619 | AMR | Pituitary hyperfur | endocrine/metabolic |
| 253.11 | 0.204673 | 0.33753 | 6.06E-01 | 0.544259 | -0.45687 | 0.86622 | AMR | Acromegaly and | endocrine/metabolic |
| 253.2 | -0.32266 | 0.175525 | -1.83826 | 0.066024 | -0.66668 | 0.021362 | AMR | Pituitary hypofun | endocrine/metabolic |
| 253.3 | 0.063844 | 0.215618 | 0.296096 | 0.767157 | -0.35876 | 0.486447 | AMR | Diabetes insipidu | endocrine/metabolic |
| 253.4 | -0.25179 | 2.75E-01 | -0.91434 | 0.360536 | -0.79152 | 0.28794 | AMR | Anterior pituitary | endocrine/metabolic |
| 253.5 | 0.508792 | 0.361067 | 1.409132 | 0.158796 | -0.19889 | 1.216471 | AMR | Pituitary dwarfisn | endocrine/metabolic |
| 253.7 | 0.132019 | 0.103628 | 1.273974 | 0.202673 | -0.07109 | 0.335125 | AMR | Other disorders c | endocrine/metabolic |
| 254 | -0.22225 | 0.494892 | -4.49E-01 | 0.653371 | -1.19222 | 0.747722 | AMR | Diseases of thym | endocrine/metabolic |

| | | | | | | | | | | |
|---|---|---|---|---|---|---|---|---|---|---|
| 255 | 0.029111 | 0.07049 | 4.13E-01 | 0.679619 | -0.10905 | 0.16727 | AMR | | Disorders of adre | endocrine/metabolic |
| 255.1 | 0.006584 | 0.163956 | 0.040159 | 0.967966 | -0.31476 | 0.327932 | AMR | | Adrenal hyperfun | endocrine/metabolic |
| 255.11 | 0.011974 | 0.200775 | 0.05964 | 0.952442 | -0.38154 | 0.405485 | AMR | | Cushing's syndrc | endocrine/metabolic |
| 255.12 | -0.04052 | 0.325592 | -0.12444 | 0.900969 | -0.67866 | 0.597632 | AMR | | Hyperaldosteroni | endocrine/metabolic |
| 255.13 | 0.906135 | 0.97753 | 0.926964 | 0.353945 | -1.00979 | 2.822058 | AMR | | Medulloadrenal h | endocrine/metabolic |
| 255.2 | -0.004 | 0.093974 | -0.04257 | 0.966045 | -0.18819 | 0.180185 | AMR | | Adrenal hypofunc | endocrine/metabolic |
| 255.21 | -0.004 | 0.093974 | -0.04257 | 0.966045 | -0.18819 | 0.180185 | AMR | | Glucocorticoid de | endocrine/metabolic |
| 255.22 | 1.168005 | 0.693179 | 1.684999 | 0.091989 | -0.1906 | 2.526611 | AMR | | Mineralocorticoid | endocrine/metabolic |
| 255.3 | 0.36138 | 0.55244 | 0.654151 | 0.513014 | -0.72138 | 1.444142 | AMR | | Adrenogenital dis | endocrine/metabolic |
| 256 | -0.01179 | 0.09862 | -0.11953 | 0.904855 | -0.20508 | 0.181504 | AMR | | Ovarian dysfunct | endocrine/metabolic |
| 256.1 | 0.812293 | 0.633961 | 1.28E+00 | 0.200089 | -0.43025 | 2.054835 | AMR | | Hyperestrogenisı | endocrine/metabolic |
| 256.4 | -0.02392 | 0.104499 | -2.29E-01 | 0.818972 | -0.22873 | 0.180897 | AMR | | Polycystic ovarie | endocrine/metabolic |
| 257 | 0.049047 | 0.099129 | 4.95E-01 | 0.620752 | -0.14524 | 0.243336 | AMR | | Testicular dysfun | endocrine/metabolic |
| 257.1 | 0.049047 | 0.099129 | 0.494784 | 0.620752 | -0.14524 | 0.243336 | AMR | | Testicular hypofu | endocrine/metabolic |
| 258 | 0.366155 | 0.33012 | 1.109159 | 0.267362 | -0.28087 | 1.013179 | AMR | | Iatrogenic endoc | endocrine/metabolic |
| 258.1 | -0.20999 | 0.219537 | -9.56E-01 | 0.338823 | -0.64027 | 0.220299 | AMR | | Postablative ovaı | endocrine/metabolic |
| 259 | -0.00322 | 0.085444 | -0.03766 | 0.969956 | -0.17069 | 0.164249 | AMR | | Other endocrine | endocrine/metabolic |
| 259.1 | 0.070801 | 0.594664 | 0.11906 | 0.905228 | -1.09472 | 1.23632 | AMR | | Nonspecific abnc | endocrine/metabolic |
| 259.2 | 0.48638 | 0.680356 | 0.71489 | 0.474677 | -0.84709 | 1.819854 | AMR | | Carcinoid syndro | endocrine/metabolic |
| 259.3 | 0.337253 | 0.63319 | 0.532625 | 0.594293 | -0.90378 | 1.578282 | AMR | | Delay in sexual d | endocrine/metabolic |
| 259.8 | 0.00626 | 0.43419 | 0.014418 | 0.988497 | -0.84474 | 0.857258 | AMR | | Polyglandular ac | endocrine/metabolic |
| 260 | 0.122926 | 0.047546 | 2.59E+00 | 0.009726 | 0.029737 | 0.216114 | AMR | | Protein-calorie m | endocrine/metabolic |
| 260.1 | 0.145224 | 0.09572 | 1.517173 | 0.129223 | -0.04238 | 0.332832 | AMR | | Cachexia | endocrine/metabolic |
| 260.2 | 0.122451 | 0.067216 | 1.821747 | 0.068493 | -0.00929 | 0.254193 | AMR | | severe protein-ca | endocrine/metabolic |
| 260.21 | 0.395367 | 0.323607 | 1.221751 | 0.221802 | -0.23889 | 1.029624 | AMR | | Kwashiorkor | endocrine/metabolic |
| 260.22 | 0.135898 | 0.15229 | 0.892363 | 0.372198 | -0.16258 | 0.43438 | AMR | | Nutritional maras | endocrine/metabolic |
| 260.3 | 0.053458 | 0.099361 | 0.538017 | 0.590565 | -0.14129 | 0.248201 | AMR | | Adult failure to th | endocrine/metabolic |
| 260.6 | -0.0173 | 0.075927 | -0.2278 | 0.819805 | -0.16611 | 0.131518 | AMR | | Anorexia | endocrine/metabolic |
| 260.7 | -0.22224 | 0.259223 | -0.85732 | 0.391269 | -0.73031 | 0.285831 | AMR | | Polyphagia | endocrine/metabolic |
| 261 | 0.042847 | 3.40E-02 | 1.258504 | 0.20821 | -0.02388 | 0.109576 | AMR | | Vitamin deficienc | endocrine/metabolic |
| 261.1 | 0.350994 | 0.260516 | 1.3473 | 0.177883 | -0.15961 | 0.861597 | AMR | | Vitamin A deficie | endocrine/metabolic |
| 261.2 | 0.09871 | 0.057751 | 1.709248 | 0.087405 | -0.01448 | 0.2119 | AMR | | Vitamin B-comple | endocrine/metabolic |
| 261.4 | 0.029231 | 0.035158 | 8.31E-01 | 0.405734 | -0.03968 | 0.09814 | AMR | | Vitamin D deficie | endocrine/metabolic |
| 261.41 | 0.322411 | 0.439605 | 0.733411 | 0.463308 | -0.5392 | 1.184021 | AMR | | Rickets or osteor | endocrine/metabolic |
| 262 | 0.038502 | 0.07623 | 0.505071 | 0.613509 | -0.11091 | 0.187909 | AMR | | Mineral deficienc | endocrine/metabolic |
| 263 | -0.00688 | 0.064853 | -0.1061 | 0.915501 | -0.13399 | 0.120229 | AMR | | Other nutritional | endocrine/metabolic |
| 264 | 0.119548 | 0.115234 | 1.03744 | 0.299531 | -0.10631 | 0.345402 | AMR | | Lack of normal p | endocrine/metabolic |
| 264.1 | 0.251253 | 0.230114 | 1.091867 | 0.274891 | -0.19976 | 0.702268 | AMR | | Short stature | endocrine/metabolic |

| | | | | | | | | | |
|---|---|---|---|---|---|---|---|---|---|
| 264.2 | 0.039452 | 0.174251 | 0.226411 | 0.820882 | -0.30207 | 0.380979 | AMR | Failure to thrive ( | endocrine/metabolic |
| 264.3 | 0.641502 | 0.484314 | 1.324557 | 0.185318 | -0.30774 | 1.59074 | AMR | Delayed milestor | endocrine/metabolic |
| 264.9 | 0.221628 | 0.146191 | 1.516018 | 0.129515 | -0.0649 | 0.508157 | AMR | Lack of normal pl | endocrine/metabolic |
| 269 | 0.110136 | 0.055341 | 1.990145 | 0.046575 | 0.00167 | 0.218602 | AMR | Proteinuria | endocrine/metabolic |
| 270 | 0.100643 | 0.065696 | 1.531945 | 0.125536 | -0.02812 | 0.229405 | AMR | Disorders of prot | endocrine/metabolic |
| 270.1 | -0.0569 | 0.213491 | -2.67E-01 | 0.789851 | -0.47533 | 0.361538 | AMR | Disturbances of a | endocrine/metabolic |
| 270.11 | 0.265475 | 0.317906 | 8.35E-01 | 0.403677 | -0.35761 | 0.888559 | AMR | Disturbances of s | endocrine/metabolic |
| 270.12 | -1.04788 | 0.97158 | -1.08E+00 | 0.280797 | -2.95214 | 0.856384 | AMR | Phenylketonuria | endocrine/metabolic |
| 270.2 | 0.231214 | 0.125178 | 1.847074 | 0.064736 | -0.01413 | 0.476559 | AMR | Disorders of amir | endocrine/metabolic |
| 270.21 | 0.27724 | 0.134301 | 2.064317 | 0.038988 | 0.014015 | 0.540465 | AMR | Disorders of urea | endocrine/metabolic |
| 270.3 | 0.090205 | 0.075609 | 1.19E+00 | 0.232849 | -0.05799 | 0.238395 | AMR | Disorders of plas | endocrine/metabolic |
| 270.31 | 1.475889 | 0.545507 | 2.705537 | 0.006819 | 0.406715 | 2.545063 | AMR | Polyclonal hyper | endocrine/metabolic |
| 270.32 | 0.009727 | 0.130552 | 0.074508 | 0.940606 | -0.24615 | 0.265605 | AMR | Paraproteinemia | endocrine/metabolic |
| 270.33 | -0.24857 | 0.188305 | -1.32006 | 0.186817 | -0.61764 | 0.120498 | AMR | Amyloidosis | endocrine/metabolic |
| 270.34 | 0.203632 | 0.282771 | 0.720132 | 0.471443 | -0.35059 | 0.757853 | AMR | Alpha-1-antitryps | endocrine/metabolic |
| 270.35 | 0.857615 | 0.668358 | 1.283167 | 0.199433 | -0.45234 | 2.167573 | AMR | Macroglobulinem | endocrine/metabolic |
| 270.38 | 0.083541 | 0.089569 | 9.33E-01 | 0.350974 | -0.09201 | 0.259093 | AMR | Other specified d | endocrine/metabolic |
| 271 | 0.053001 | 0.03711 | 1.428216 | 0.15323 | -0.01973 | 0.125736 | AMR | Disorders of carb | endocrine/metabolic |
| 271.3 | 0.051415 | 0.037246 | 1.380421 | 0.167457 | -0.02159 | 0.124415 | AMR | Intestinal disacch | endocrine/metabolic |
| 271.9 | 0.816954 | 0.29219 | 2.79597 | 0.005174 | 0.244272 | 1.389635 | AMR | Other disorders c | endocrine/metabolic |
| 272 | 0.043406 | 0.034847 | 1.245602 | 0.212911 | -0.02489 | 0.111704 | AMR | Disorders of lipoi | endocrine/metabolic |
| 272.1 | 0.043872 | 0.034892 | 1.257348 | 0.208628 | -0.02452 | 0.112259 | AMR | Hyperlipidemia | endocrine/metabolic |
| 272.11 | 0.005619 | 0.041681 | 0.134819 | 0.892755 | -0.07607 | 0.087313 | AMR | Hypercholesterol | endocrine/metabolic |
| 272.12 | 0.093842 | 0.068196 | 1.376051 | 0.168806 | -0.03982 | 0.227504 | AMR | Hyperglyceridem | endocrine/metabolic |
| 272.13 | -0.00144 | 0.045073 | -0.03192 | 0.974536 | -0.08978 | 0.086902 | AMR | Mixed hyperlipide | endocrine/metabolic |
| 272.14 | 0.353602 | 0.503678 | 0.702041 | 0.482654 | -0.63359 | 1.340793 | AMR | Hyperchylomicro | endocrine/metabolic |
| 272.9 | -0.21352 | 0.182854 | -1.16768 | 0.242934 | -0.5719 | 0.144871 | AMR | Unspecified disor | endocrine/metabolic |
| 274 | 0.006823 | 0.071573 | 9.53E-02 | 0.92405 | -0.13346 | 0.147105 | AMR | Gout and other c | endocrine/metabolic |
| 274.1 | 0.020137 | 0.079523 | 0.253226 | 0.800093 | -0.13572 | 0.176 | AMR | Gout | endocrine/metabolic |
| 274.11 | 0.021488 | 0.097336 | 2.21E-01 | 0.825282 | -0.16929 | 0.212263 | AMR | Gouty arthropath | endocrine/metabolic |
| 274.2 | -0.08781 | 0.126428 | -0.69453 | 0.48735 | -0.3356 | 0.159986 | AMR | Crystal arthropat | endocrine/metabolic |
| 274.21 | -0.1131 | 0.132737 | -0.8521 | 0.39416 | -0.37326 | 0.147055 | AMR | Chondrocalcinos | endocrine/metabolic |
| 275 | 0.071571 | 0.0387 | 1.849392 | 0.064401 | -0.00428 | 0.147422 | AMR | Disorders of mine | endocrine/metabolic |
| 275.1 | -0.02489 | 0.151265 | -0.16452 | 0.869321 | -0.32136 | 0.271588 | AMR | Disorders of iron | hematopoietic |
| 275.11 | 0.346748 | 0.387853 | 0.894019 | 0.371312 | -0.41343 | 1.106925 | AMR | Hereditary hemo | hematopoietic |
| 275.2 | 0.543927 | 0.462247 | 1.176703 | 0.239314 | -0.36206 | 1.449914 | AMR | Disorders of copp | endocrine/metabolic |
| 275.3 | 0.036629 | 0.05285 | 0.693078 | 0.488261 | -0.06695 | 0.140213 | AMR | Disorders of mag | endocrine/metabolic |
| 275.5 | 0.097041 | 0.044895 | 2.161497 | 0.030657 | 0.009048 | 0.185034 | AMR | Disorders of calc | endocrine/metabolic |

| | | | | | | | | | |
|---|---|---|---|---|---|---|---|---|---|
| 275.51 | 0.110708 | 0.061808 | 1.79E+00 | 0.073269 | -0.01043 | 0.231849 | AMR | Hypocalcemia | endocrine/metabolic |
| 275.53 | 0.078483 | 0.052842 | 1.49E+00 | 0.137479 | -0.02508 | 0.18205 | AMR | Disorders of phos | endocrine/metabolic |
| 275.6 | 0.029123 | 0.06996 | 0.416286 | 0.677201 | -0.108 | 0.166242 | AMR | Hypercalcemia | endocrine/metabolic |
| 276 | 0.03278 | 0.033826 | 0.969084 | 0.332503 | -0.03352 | 0.099078 | AMR | Disorders of fluid | endocrine/metabolic |
| 276.1 | 0.08577 | 0.035527 | 2.414238 | 0.015768 | 0.016139 | 0.155401 | AMR | Electrolyte imbala | endocrine/metabolic |
| 276.11 | 0.061829 | 0.072416 | 0.8538 | 0.393216 | -0.0801 | 0.203762 | AMR | Hyperosmolality a | endocrine/metabolic |
| 276.12 | 0.054201 | 0.04209 | 1.287741 | 0.197836 | -0.02829 | 0.136696 | AMR | Hyposmolality an | endocrine/metabolic |
| 276.13 | 0.100278 | 0.045221 | 2.217511 | 0.026588 | 0.011647 | 0.18891 | AMR | Hyperpotassemia | endocrine/metabolic |
| 276.14 | 0.122131 | 0.046371 | 2.633781 | 0.008444 | 0.031245 | 0.213016 | AMR | Hypopotassemia | endocrine/metabolic |
| 276.4 | 0.068385 | 0.043138 | 1.585247 | 0.11291 | -0.01616 | 0.152934 | AMR | Acid-base balanc | endocrine/metabolic |
| 276.41 | 0.070705 | 0.045551 | 1.552213 | 0.120611 | -0.01857 | 0.159983 | AMR | Acidosis | endocrine/metabolic |
| 276.42 | -0.00911 | 0.083731 | -1.09E-01 | 0.913344 | -0.17322 | 0.154998 | AMR | Alkalosis | endocrine/metabolic |
| 276.5 | -0.04105 | 0.040839 | -1.01E+00 | 0.314779 | -0.1211 | 0.038989 | AMR | Hypovolemia | endocrine/metabolic |
| 276.6 | 0.114469 | 0.049086 | 2.332014 | 0.0197 | 0.018262 | 0.210676 | AMR | Fluid overload | endocrine/metabolic |
| 276.8 | 0.077526 | 0.166202 | 0.46646 | 0.640886 | -0.24822 | 0.403275 | AMR | Polydipsia | endocrine/metabolic |
| 277 | 0.03681 | 0.04915 | 0.748938 | 0.453895 | -0.05952 | 0.133141 | AMR | Other disorders c | endocrine/metabolic |
| 277.1 | 0.278067 | 0.69124 | 0.402273 | 0.687483 | -1.07674 | 1.632872 | AMR | Disorders of porp | endocrine/metabolic |
| 277.2 | 1.418407 | 1.705858 | 0.831492 | 0.405696 | -1.92501 | 4.761828 | AMR | Other disorders c | endocrine/metabolic |
| 277.4 | 0.078345 | 0.083821 | 0.934669 | 0.349959 | -0.08594 | 0.242631 | AMR | Disorders of biliru | endocrine/metabolic |
| 277.5 | -0.00924 | 0.106713 | -0.08656 | 0.93102 | -0.21839 | 0.199916 | AMR | Other disorders c | endocrine/metabolic |
| 277.51 | 0.120041 | 0.147593 | 0.813325 | 0.416032 | -0.16924 | 0.409318 | AMR | Lipoprotein disor | endocrine/metabolic |
| 277.6 | 0.196727 | 0.339572 | 0.579337 | 0.562362 | -0.46882 | 0.862276 | AMR | Other deficiencie | endocrine/metabolic |
| 277.7 | 0.068235 | 0.087419 | 7.81E-01 | 0.435065 | -0.1031 | 0.239572 | AMR | Dysmetabolic syr | endocrine/metabolic |
| 277.8 | 0.797659 | 0.495989 | 1.608218 | 0.107787 | -0.17446 | 1.76978 | AMR | Carnitine deficier | endocrine/metabolic |
| 278 | 0.148449 | 0.031471 | 4.72E+00 | 2.39E-06 | 0.086767 | 0.210131 | AMR | Overweight, obes | endocrine/metabolic |
| 278.1 | 0.149203 | 0.032894 | 4.535824 | 5.74E-06 | 0.084731 | 0.213675 | AMR | Obesity | endocrine/metabolic |
| 278.11 | 0.098564 | 0.039072 | 2.522634 | 0.011648 | 0.021984 | 0.175143 | AMR | Morbid obesity | endocrine/metabolic |
| 278.3 | 0.301684 | 0.165927 | 1.818176 | 0.069037 | -0.02353 | 0.626895 | AMR | Localized adipos | endocrine/metabolic |
| 278.4 | 0.050833 | 0.058936 | 0.862516 | 0.388404 | -0.06468 | 0.166345 | AMR | Abnormal weight | endocrine/metabolic |
| 279 | 0.061279 | 0.040807 | 1.501679 | 0.13318 | -0.0187 | 0.14126 | AMR | Disorders involvi | endocrine/metabolic |
| 279.1 | 0.04736 | 0.041363 | 1.144981 | 0.252217 | -0.03371 | 0.12843 | AMR | Immunity deficier | endocrine/metabolic |
| 279.11 | -0.04225 | 0.118342 | -0.357 | 0.72109 | -0.2742 | 0.189698 | AMR | Deficiency of hur | endocrine/metabolic |
| 279.2 | 0.085571 | 0.205128 | 0.417159 | 0.676562 | -0.31647 | 0.487614 | AMR | Autoimmune dise | endocrine/metabolic |
| 279.7 | 0.106059 | 0.04997 | 2.12E+00 | 0.033798 | 0.00812 | 0.203998 | AMR | Other immunolog | endocrine/metabolic |
| 279.8 | 0.031443 | 0.1579 | 0.199131 | 0.84216 | -0.27804 | 0.340921 | AMR | Other specified d | endocrine/metabolic |
| 280 | 0.035742 | 0.039147 | 0.913019 | 0.361232 | -0.04098 | 0.112469 | AMR | Iron deficiency ar | hematopoietic |
| 280.1 | 0.052854 | 0.041349 | 1.28E+00 | 0.201164 | -0.02819 | 0.133897 | AMR | Iron deficiency ar | hematopoietic |
| 280.2 | -0.06356 | 0.057657 | -1.10243 | 0.270277 | -0.17657 | 0.049443 | AMR | Iron deficiency ar | hematopoietic |

| | | | | | | | | | | |
|---|---|---|---|---|---|---|---|---|---|---|
| 281 | 0.016457 | 0.069392 | 0.237163 | 0.81253 | -0.11955 | 0.152463 | AMR | Other deficiency | hematopoietic | |
| 281.1 | 0.124777 | 0.130077 | 0.959257 | 0.337429 | -0.13017 | 0.379724 | AMR | Megaloblastic an | hematopoietic | |
| 281.11 | 0.803457 | 0.249457 | 3.22082 | 0.001278 | 0.31453 | 1.292384 | AMR | Pernicious anem | hematopoietic | |
| 281.12 | 0.316769 | 0.187662 | 1.69E+00 | 9.14E-02 | -0.05104 | 0.684581 | AMR | Other vitamin B1 | hematopoietic | |
| 281.13 | 0.313271 | 0.216023 | 1.45E+00 | 1.47E-01 | -0.11013 | 0.736668 | AMR | Folate-deficiency | hematopoietic | |
| 281.9 | -0.00044 | 0.079161 | -0.00553 | 0.995589 | -0.15559 | 0.154715 | AMR | Deficiency anemi | hematopoietic | |
| 282 | 0.057841 | 0.10824 | 5.34E-01 | 0.593078 | -0.1543 | 0.269987 | AMR | Hereditary hemol | hematopoietic | |
| 282.5 | 0.41996 | 0.337792 | 1.24325 | 0.213776 | -0.2421 | 1.082021 | AMR | Sickle cell anemi | hematopoietic | |
| 282.8 | -0.03622 | 0.154454 | -0.2345 | 0.814599 | -0.33894 | 0.266505 | AMR | Other hemoglobi | hematopoietic | |
| 282.9 | 0.100253 | 0.159657 | 0.627927 | 0.530052 | -0.21267 | 0.413175 | AMR | Other hereditary | hematopoietic | |
| 283 | 0.098043 | 0.1494 | 0.65625 | 0.511664 | -0.19477 | 0.390862 | AMR | Acquired hemoly | hematopoietic | |
| 283.1 | 0.694391 | 0.243758 | 2.848687 | 0.00439 | 0.216633 | 1.172148 | AMR | Autoimmune hen | hematopoietic | |
| 283.2 | 0.381194 | 0.293867 | 1.297167 | 0.194574 | -0.19477 | 0.957162 | AMR | Non-autoimmune | hematopoietic | |
| 283.21 | 0.501717 | 0.497311 | 1.00886 | 0.313042 | -0.47299 | 1.476428 | AMR | Hemolytic-uremic | hematopoietic | |
| 284 | 0.057465 | 0.054854 | 1.047602 | 0.294822 | -0.05005 | 0.164977 | AMR | Aplastic anemia | hematopoietic | |
| 284.1 | 0.06089 | 0.055839 | 1.090452 | 0.275514 | -0.04855 | 0.170334 | AMR | Pancytopenia | hematopoietic | |
| 284.2 | 0.773937 | 5.31E-01 | 1.458772 | 0.144628 | -0.2659 | 1.813775 | AMR | Constitutional ap | hematopoietic | |
| 285 | 0.062406 | 0.031921 | 1.96E+00 | 0.050582 | -0.00016 | 0.124971 | AMR | Other anemias | hematopoietic | |
| 285.1 | 0.019474 | 0.040493 | 0.480928 | 0.630568 | -0.05989 | 0.098839 | AMR | Acute posthemor | hematopoietic | |
| 285.2 | 0.044218 | 0.040493 | 1.091985 | 0.274839 | -0.03515 | 0.123582 | AMR | Anemia of chroni | hematopoietic | |
| 285.21 | 0.053479 | 0.050203 | 1.065254 | 0.286761 | -0.04492 | 0.151876 | AMR | Anemia in chroni | hematopoietic | |
| 285.22 | 0.017357 | 0.090331 | 0.192147 | 0.847627 | -0.15969 | 0.194403 | AMR | Anemia in neopla | hematopoietic | |
| 285.3 | 0.738374 | 0.458652 | 1.61E+00 | 0.107424 | -0.16057 | 1.637315 | AMR | Sideroblastic ane | hematopoietic | |
| 285.8 | 0.536816 | 0.310016 | 1.731575 | 0.083349 | -0.0708 | 1.144435 | AMR | Hemoglobinuria | hematopoietic | |
| 286 | 0.069038 | 0.043308 | 1.594122 | 0.110909 | -0.01584 | 0.153919 | AMR | Coagulation defe | hematopoietic | |
| 286.1 | 0.258529 | 0.185158 | 1.39626 | 0.162636 | -0.10437 | 0.621433 | AMR | Congenital coagu | hematopoietic | |
| 286.11 | 0.518362 | 0.350664 | 1.478229 | 0.139346 | -0.16893 | 1.205651 | AMR | Von willebrand's | hematopoietic | |
| 286.12 | 0.150613 | 0.234655 | 0.641848 | 0.520972 | -0.3093 | 0.610529 | AMR | Congenital defici | hematopoietic | |
| 286.13 | 0.729088 | 0.323485 | 2.25E+00 | 0.024205 | 0.095069 | 1.363106 | AMR | Congenital factor | hematopoietic | |
| 286.2 | 0.106192 | 0.048164 | 2.204808 | 0.027468 | 0.011793 | 0.200591 | AMR | Encounter for lon | hematopoietic | |
| 286.3 | 0.106769 | 0.196943 | 0.542133 | 0.587727 | -0.27923 | 0.49277 | AMR | Coagulation defe | hematopoietic | |
| 286.4 | 0.059479 | 0.073627 | 0.807845 | 0.41918 | -0.08483 | 0.203785 | AMR | Acquired coagula | hematopoietic | |
| 286.5 | 0.407724 | 0.284845 | 1.431389 | 0.152319 | -0.15056 | 0.966009 | AMR | Hemorrhagic disc | hematopoietic | |
| 286.6 | 0.056377 | 0.104351 | 5.40E-01 | 0.589014 | -0.14815 | 0.260901 | AMR | Defibrination syn | hematopoietic | |
| 286.7 | 0.112923 | 0.057015 | 1.980605 | 0.047636 | 0.001177 | 0.22467 | AMR | Other and unspe | hematopoietic | |
| 286.8 | 0.01016 | 0.089663 | 0.113317 | 0.909779 | -0.16558 | 0.185896 | AMR | Hypercoagulable | hematopoietic | |
| 286.81 | 0.012573 | 0.098631 | 0.12748 | 0.89856 | -0.18074 | 0.205887 | AMR | Primary hypercoa | hematopoietic | |
| 286.9 | 0.024304 | 0.078153 | 0.310976 | 0.755819 | -0.12887 | 0.177481 | AMR | Abnormal coagul | hematopoietic | |

| | | | | | | | | | | |
|---|---|---|---|---|---|---|---|---|---|---|
| 287 | 0.08801 | 0.042321 | 2.079588 | 0.037563 | 0.005063 | 0.170957 | AMR | Purpura and othe | hematopoietic | |
| 287.1 | 0.004241 | 0.156413 | 2.71E-02 | 0.97837 | -0.30232 | 0.310804 | AMR | Spontaneous ecc | hematopoietic | |
| 287.2 | -0.07708 | 1.121283 | -0.06874 | 0.945195 | -2.27475 | 2.120596 | AMR | Allergic purpura | hematopoietic | |
| 287.3 | 0.067687 | 0.043324 | 1.56E+00 | 0.118209 | -0.01723 | 0.1526 | AMR | Thrombocytopen | hematopoietic | |
| 287.31 | -0.15698 | 0.168751 | -0.93023 | 0.352254 | -0.48772 | 0.17377 | AMR | Primary thrombo | hematopoietic | |
| 287.32 | -0.01644 | 0.066338 | -0.24779 | 0.8043 | -0.14646 | 0.113583 | AMR | Secondary throm | hematopoietic | |
| 287.4 | 0.41201 | 0.212971 | 1.93458 | 0.053042 | -0.00541 | 0.829426 | AMR | Qualitative platel | hematopoietic | |
| 288 | 0.037399 | 0.03852 | 0.970886 | 0.331605 | -0.0381 | 0.112896 | AMR | Diseases of white | hematopoietic | |
| 288.1 | 0.013782 | 0.04322 | 0.318866 | 0.749828 | -0.07093 | 0.098492 | AMR | Decreased white | hematopoietic | |
| 288.11 | 0.064347 | 0.051078 | 1.259783 | 0.207748 | -0.03576 | 0.164457 | AMR | Neutropenia | hematopoietic | |
| 288.2 | 0.029038 | 0.039581 | 0.733631 | 0.463174 | -0.04854 | 0.106614 | AMR | Elevated white bl | hematopoietic | |
| 288.3 | 0.244733 | 0.139182 | 1.758371 | 0.078684 | -0.02806 | 0.517523 | AMR | Eosinophilia | hematopoietic | |
| 289 | -0.02295 | 0.04975 | -4.61E-01 | 0.644554 | -0.12046 | 0.074557 | AMR | Other diseases o | hematopoietic | |
| 289.1 | 0.766423 | 0.406691 | 1.884535 | 0.059493 | -0.03068 | 1.563522 | AMR | Myelofibrosis | hematopoietic | |
| 289.3 | -0.03205 | 0.080865 | -3.96E-01 | 0.691812 | -0.19055 | 0.126438 | AMR | Personal history | hematopoietic | |
| 289.4 | -0.01374 | 0.04747 | -0.28953 | 0.772178 | -0.10678 | 0.079296 | AMR | Lymphadenitis | hematopoietic | |
| 289.5 | 0.114172 | 0.127392 | 0.896226 | 0.370132 | -0.13551 | 0.363856 | AMR | Diseases of sple | hematopoietic | |
| 289.8 | 0.058439 | 0.112281 | 0.520469 | 0.602737 | -0.16163 | 0.278505 | AMR | Polycythemia, se | hematopoietic | |
| 289.9 | 0.06421 | 0.096047 | 0.668528 | 0.503797 | -0.12404 | 0.252458 | AMR | Abnormality of re | hematopoietic | |
| 290 | -0.07232 | 0.077441 | -0.93389 | 0.350359 | -0.2241 | 0.07946 | AMR | Delirium dementi | mental disorders | |
| 290.1 | -0.15354 | 0.11016 | -1.39384 | 0.163366 | -0.36945 | 0.062364 | AMR | Dementias | mental disorders | |
| 290.11 | -0.18466 | 0.18608 | -0.99238 | 0.321013 | -0.54937 | 0.180049 | AMR | Alzheimer's disea | mental disorders | |
| 290.12 | 0.628132 | 0.447896 | 1.402407 | 0.160794 | -0.24973 | 1.505992 | AMR | Dementia with ce | mental disorders | |
| 290.13 | 0.31811 | 0.314178 | 1.01E+00 | 0.311293 | -0.29767 | 0.933887 | AMR | Senile dementia | mental disorders | |
| 290.16 | -0.12282 | 0.189835 | -0.64696 | 0.517656 | -0.49489 | 0.249253 | AMR | Vascular dement | mental disorders | |
| 290.2 | 0.027677 | 0.106425 | 0.260061 | 0.794817 | -0.18091 | 0.236266 | AMR | Delirium due to c | mental disorders | |
| 290.3 | -0.11665 | 0.116959 | -0.99736 | 0.318592 | -0.34588 | 0.112585 | AMR | Other persistent | mental disorders | |
| 291 | 0.062819 | 0.06017 | 1.044017 | 0.296477 | -0.05511 | 0.18075 | AMR | Other specified n | mental disorders | |
| 291.1 | -0.3684 | 0.293726 | -1.25422 | 0.209761 | -0.94409 | 0.207294 | AMR | Transient mental | mental disorders | |
| 291.4 | -0.08364 | 0.142878 | -0.58539 | 0.558286 | -0.36367 | 0.196397 | AMR | Specific nonpsyc | mental disorders | |
| 291.8 | 0.103627 | 0.06528 | 1.58743 | 0.112415 | -0.02432 | 0.231574 | AMR | Alteration of cons | mental disorders | |
| 292 | 0.057313 | 0.040471 | 1.416139 | 0.156735 | -0.02201 | 0.136636 | AMR | Neurological disc | mental disorders | |
| 292.1 | 0.005365 | 0.079409 | 0.067557 | 0.946138 | -0.15027 | 0.161004 | AMR | Aphasia/speech | mental disorders | |
| 292.11 | -0.03066 | 0.12601 | -0.24332 | 0.807756 | -0.27764 | 0.216313 | AMR | Aphasia | mental disorders | |
| 292.12 | -0.24637 | 0.274882 | -8.96E-01 | 0.37011 | -0.78513 | 0.292391 | AMR | Symbolic dysfunc | mental disorders | |
| 292.2 | -0.02993 | 0.162443 | -0.18423 | 0.85383 | -0.34831 | 0.288456 | AMR | Mild cognitive im | mental disorders | |
| 292.3 | 0.011692 | 0.063442 | 0.184287 | 0.853788 | -0.11265 | 0.136035 | AMR | Memory loss | mental disorders | |
| 292.4 | 0.102022 | 0.053357 | 1.91206 | 0.055868 | -0.00256 | 0.206599 | AMR | Altered mental st | mental disorders | |

| | | | | | | | | | | |
|---|---|---|---|---|---|---|---|---|---|---|
| 292.5 | -0.02905 | 0.119344 | -0.24345 | 0.807658 | -0.26296 | 0.204855 | AMR | Transient alterati | mental disorders | |
| 292.6 | 0.124227 | 0.154173 | 0.805765 | 0.420379 | -0.17795 | 0.426401 | AMR | Hallucinations | mental disorders | |
| 293 | 0.049112 | 0.080723 | 0.608394 | 0.542926 | -0.1091 | 0.207327 | AMR | Symptoms involv | mental disorders | |
| 293.1 | 0.042695 | 0.053463 | 0.798599 | 0.424523 | -0.06209 | 0.147481 | AMR | Swelling, mass, ( | mental disorders | |
| 295 | -0.09422 | 8.52E-02 | -1.10619 | 0.268646 | -0.26116 | 0.072721 | AMR | Schizophrenia ar | mental disorders | |
| 295.1 | 0.038221 | 0.18841 | 0.202859 | 0.839245 | -0.33106 | 0.407498 | AMR | Schizophrenia | mental disorders | |
| 295.2 | 0.133127 | 1.72E-01 | 0.774529 | 0.438618 | -0.20375 | 0.470008 | AMR | Paranoid disorde | mental disorders | |
| 295.3 | -0.18144 | 0.098097 | -1.85E+00 | 0.064368 | -0.37371 | 0.010824 | AMR | Psychosis | mental disorders | |
| 296 | 0.001222 | 0.033286 | 0.036724 | 0.970705 | -0.06402 | 0.066462 | AMR | Mood disorders | mental disorders | |
| 296.1 | 0.017054 | 0.073013 | 0.233579 | 0.815312 | -0.12605 | 0.160158 | AMR | Bipolar | mental disorders | |
| 296.2 | 0.00364 | 0.033554 | 0.108471 | 0.913622 | -0.06213 | 0.069404 | AMR | Depression | mental disorders | |
| 296.22 | 0.01291 | 0.034942 | 0.369465 | 0.711781 | -0.05557 | 0.081394 | AMR | Major depressive | mental disorders | |
| 297 | 0.012583 | 0.110527 | 0.113849 | 0.909358 | -0.20405 | 0.229213 | AMR | Suicidal ideation | mental disorders | |
| 297.1 | -0.05958 | 0.118318 | -0.50359 | 0.61455 | -0.29148 | 0.172316 | AMR | Suicidal ideation | mental disorders | |
| 297.2 | -0.30959 | 0.245159 | -1.26282 | 0.206653 | -0.7901 | 0.17091 | AMR | Suicide or self-in | mental disorders | |
| 300 | 0.050397 | 3.15E-02 | 1.599693 | 0.109667 | -0.01135 | 0.112143 | AMR | Anxiety disorders | mental disorders | |
| 300.1 | 0.037602 | 0.032 | 1.175038 | 0.239979 | -0.02512 | 0.100321 | AMR | Anxiety disorder | mental disorders | |
| 300.11 | 0.012 | 0.047441 | 0.252952 | 0.800305 | -0.08098 | 0.104983 | AMR | Generalized anxi | mental disorders | |
| 300.12 | 0.104661 | 0.072027 | 1.45E+00 | 0.146199 | -0.03651 | 0.245831 | AMR | Agorophobia, soc | mental disorders | |
| 300.13 | 0.03731 | 0.133957 | 0.278524 | 0.78061 | -0.22524 | 0.299862 | AMR | Phobia | mental disorders | |
| 300.3 | -0.24351 | 0.226193 | -1.07655 | 0.281679 | -0.68684 | 0.199821 | AMR | Obsessive-comp | mental disorders | |
| 300.4 | -0.01056 | 0.071552 | -0.14765 | 0.882618 | -0.1508 | 0.129674 | AMR | Dysthymic disord | mental disorders | |
| 300.8 | -0.01575 | 0.12889 | -0.12219 | 0.902751 | -0.26837 | 0.236871 | AMR | Acute reaction to | mental disorders | |
| 300.9 | 0.062482 | 0.101909 | 0.613114 | 0.539801 | -0.13726 | 0.26222 | AMR | Posttraumatic str | mental disorders | |
| 301 | 0.010712 | 0.193117 | 0.05547 | 0.955764 | -0.36779 | 0.389215 | AMR | Personality disor | mental disorders | |
| 301.2 | -0.16838 | 0.252901 | -0.66579 | 0.505548 | -0.66405 | 0.327299 | AMR | Antisocial/border | mental disorders | |
| 302 | 0.025355 | 0.099692 | 0.254335 | 0.799237 | -0.17004 | 0.220747 | AMR | Sexual and gend | mental disorders | |
| 302.1 | -0.01954 | 0.115994 | -0.16847 | 0.866217 | -0.24689 | 0.207804 | AMR | Decreased libido | mental disorders | |
| 303 | 0.03506 | 0.091526 | 0.383055 | 0.701679 | -0.14433 | 0.214448 | AMR | Psychogenic and | mental disorders | |
| 303.1 | -0.13938 | 0.401254 | -3.47E-01 | 0.728324 | -0.92582 | 0.647066 | AMR | Dissociative diso | mental disorders | |
| 303.3 | 0.04639 | 0.105361 | 0.4403 | 0.65972 | -0.16011 | 0.252893 | AMR | Psychogenic disc | mental disorders | |
| 303.31 | -0.01299 | 0.144564 | -0.08983 | 0.928425 | -0.29633 | 0.270355 | AMR | Gastrointestinal r | mental disorders | |
| 303.4 | 0.024585 | 0.153166 | 0.16051 | 0.872479 | -0.27561 | 0.324784 | AMR | Somatoform diso | mental disorders | |
| 304 | 0.044207 | 0.050991 | 0.866961 | 0.385963 | -0.05573 | 0.144148 | AMR | Adjustment react | mental disorders | |
| 305.2 | -0.01965 | 0.161481 | -0.12166 | 0.90317 | -0.33614 | 0.296851 | AMR | Eating disorder | mental disorders | |
| 305.21 | -0.07174 | 0.371647 | -0.19302 | 0.846941 | -0.80015 | 0.656677 | AMR | Anorexia nervosa | mental disorders | |
| 306 | 0.027447 | 0.032389 | 0.847415 | 0.396764 | -0.03603 | 0.090929 | AMR | Other mental dis | mental disorders | |
| 306.1 | 0.016972 | 0.106885 | 0.158784 | 0.873839 | -0.19252 | 0.226463 | AMR | Mental disorders | mental disorders | |

| | | | | | | | | | | |
|---|---|---|---|---|---|---|---|---|---|---|
| 306.9 | -0.0114 | 0.0764 | -0.14917 | 0.881418 | -0.16114 | 0.138345 | AMR | Tension headach | mental disorders | |
| 312 | 0.189688 | 1.63E-01 | 1.161655 | 0.245376 | -0.13036 | 0.509733 | AMR | Conduct disorder | mental disorders | |
| 312.3 | 0.501893 | 0.439244 | 1.142631 | 0.253192 | -0.35901 | 1.362795 | AMR | Impulse control d | mental disorders | |
| 313 | -0.08151 | 0.099298 | -0.82086 | 0.411725 | -0.27613 | 0.11311 | AMR | Pervasive develo | mental disorders | |
| 313.1 | -0.17081 | 0.112749 | -1.51499 | 0.129775 | -0.3918 | 0.050171 | AMR | Attention deficit h | mental disorders | |
| 313.2 | -0.29527 | 0.34457 | -0.85691 | 0.391495 | -0.97061 | 0.380079 | AMR | Tics and stutterin | mental disorders | |
| 313.3 | -0.01223 | 0.210487 | -0.05812 | 0.95365 | -0.42478 | 0.400313 | AMR | Autism | mental disorders | |
| 315 | 0.103456 | 0.080614 | 1.283345 | 0.199371 | -0.05455 | 0.261457 | AMR | Develomental de | mental disorders | |
| 315.1 | -0.14902 | 0.261115 | -0.57072 | 0.568192 | -0.6608 | 0.362754 | AMR | Learning disorde | mental disorders | |
| 315.2 | 0.273173 | 0.171665 | 1.591314 | 0.111539 | -0.06328 | 0.60963 | AMR | Speech and lang | mental disorders | |
| 315.3 | 0.089374 | 1.71E-01 | 0.522539 | 0.601295 | -0.24586 | 0.424604 | AMR | Mental retardatio | mental disorders | |
| 316 | 0.108416 | 0.052199 | 2.07695 | 0.037806 | 0.006107 | 0.210725 | AMR | Substance addic | mental disorders | |
| 316.1 | 0.011674 | 1.13E-01 | 0.103317 | 0.917711 | -0.20979 | 0.233143 | AMR | Polyneuropathy d | mental disorders | |
| 317 | 0.068426 | 0.053338 | 1.28E+00 | 0.199534 | -0.03611 | 0.172966 | AMR | Alcohol-related d | mental disorders | |
| 317.1 | 0.089253 | 0.055653 | 1.603747 | 0.10877 | -0.01982 | 0.19833 | AMR | Alcoholism | mental disorders | |
| 317.11 | 0.024962 | 0.076868 | 0.324734 | 0.745382 | -0.1257 | 0.175621 | AMR | Alcoholic liver da | mental disorders | |
| 318 | 0.142447 | 0.03658 | 3.894177 | 9.85E-05 | 0.070753 | 0.214142 | AMR | Tobacco use disc | mental disorders | |
| 320 | -0.1355 | 0.129609 | -1.04545 | 0.295813 | -0.38953 | 0.118528 | AMR | Meningitis | neurological | |
| 323 | 0.234709 | 0.151922 | 1.544928 | 0.122364 | -0.06305 | 0.532471 | AMR | Encephalitis | neurological | |
| 323.2 | 0.823281 | 0.471722 | 1.745268 | 0.080938 | -0.10128 | 1.747838 | AMR | Acute (transverse | neurological | |
| 323.8 | 0.232648 | 0.176708 | 1.316566 | 0.187984 | -0.11369 | 0.578989 | AMR | Encephalitis, non | neurological | |
| 324 | 0.006133 | 1.18E-01 | 0.051928 | 0.958586 | -0.22536 | 0.23763 | AMR | Other CNS infect | neurological | |
| 325 | -0.10611 | 0.342837 | -0.3095 | 0.756941 | -0.77806 | 0.565841 | AMR | Phlebitis and thro | neurological | |
| 327 | 0.04709 | 0.034978 | 1.346273 | 0.178214 | -0.02147 | 0.115645 | AMR | Sleep disorders | neurological | |
| 327.1 | 0.086893 | 0.065935 | 1.32E+00 | 0.187546 | -0.04234 | 0.216123 | AMR | Hypersomnia | neurological | |
| 327.3 | 0.084384 | 0.038574 | 2.187599 | 0.028699 | 0.008781 | 0.159987 | AMR | Sleep apnea | neurological | |
| 327.31 | 0.04434 | 0.119339 | 0.371548 | 0.710229 | -0.18956 | 0.27824 | AMR | Central/nonobstr | neurological | |
| 327.32 | 0.086012 | 0.041898 | 2.052911 | 0.040081 | 0.003894 | 0.16813 | AMR | Obstructive sleep | neurological | |
| 327.4 | 0.048443 | 0.0382 | 1.268166 | 0.204739 | -0.02643 | 0.123313 | AMR | Insomnia | neurological | |
| 327.41 | 0.065062 | 0.064748 | 1.004844 | 0.314972 | -0.06184 | 0.191966 | AMR | Organic or persis | neurological | |
| 327.5 | -0.1051 | 0.160088 | -0.65651 | 0.511499 | -0.41887 | 0.208669 | AMR | Parasomnia | neurological | |
| 327.6 | -0.05403 | 1.73E-01 | -0.31181 | 0.755186 | -0.39362 | 0.285567 | AMR | Circadian rhythm | neurological | |
| 327.7 | 0.033582 | 0.090798 | 0.369848 | 0.711495 | -0.14438 | 0.211543 | AMR | Sleep related mo | neurological | |
| 327.71 | -0.04197 | 0.125008 | -3.36E-01 | 7.37E-01 | -0.28698 | 0.203041 | AMR | Restless legs syr | neurological | |
| 327.72 | 0.396751 | 0.295385 | 1.343165 | 0.179218 | -0.18219 | 0.975695 | AMR | Sleep related leg | neurological | |
| 331 | 0.106529 | 0.102278 | 1.04E+00 | 0.297613 | -0.09393 | 0.306991 | AMR | Other cerebral de | neurological | |
| 331.1 | 0.023476 | 0.130713 | 0.1796 | 0.857466 | -0.23272 | 0.279669 | AMR | Hydrocephalus | neurological | |
| 331.9 | 0.335456 | 0.207872 | 1.613764 | 0.106579 | -0.07197 | 0.742877 | AMR | Cerebral degene | neurological | |

| | | | | | | | | | | |
|---|---|---|---|---|---|---|---|---|---|---|
| 332 | -0.53879 | 0.260327 | -2.06965 | 0.038485 | -1.04902 | -0.02856 | AMR | Parkinson's disea | neurological | |
| 333 | -0.03121 | 0.086359 | -0.36134 | 0.717843 | -0.20047 | 0.138055 | AMR | Extrapyramidal d | neurological | |
| 333.1 | -0.29919 | 0.140782 | -2.12521 | 0.033569 | -0.57512 | -0.02326 | AMR | Essential tremor | neurological | |
| 333.2 | -0.01223 | 0.202239 | -0.06049 | 0.951766 | -0.40861 | 0.384148 | AMR | Myoclonus | neurological | |
| 333.3 | 0.442026 | 1.03278 | 0.427997 | 0.668653 | -1.58218 | 2.466238 | AMR | Tics and choreas | neurological | |
| 333.4 | 0.304624 | 0.169587 | 1.796271 | 0.072451 | -0.02776 | 0.637008 | AMR | Torsion dystonia | neurological | |
| 333.8 | 0.373751 | 0.247622 | 1.50936 | 0.131207 | -0.11158 | 0.859082 | AMR | Other degenerati | neurological | |
| 334 | 0.178902 | 0.096169 | 1.860279 | 0.062846 | -0.00959 | 0.36739 | AMR | Degenerative dis | neurological | |
| 334.1 | 0.40516 | 0.330015 | 1.23E+00 | 0.219558 | -0.24166 | 1.051978 | AMR | Spinocerebellar ( | neurological | |
| 334.2 | 0.468637 | 0.19782 | 2.369013 | 0.017836 | 0.080918 | 0.856357 | AMR | Anterior horn cell | neurological | |
| 334.21 | 0.649564 | 0.219055 | 2.965293 | 0.003024 | 0.220223 | 1.078904 | AMR | Amyotrophic Late | neurological | |
| 335 | -0.064 | 0.18741 | -0.34149 | 0.732735 | -0.43132 | 0.303319 | AMR | Multiple sclerosis | neurological | |
| 337 | 0.142432 | 0.122074 | 1.166772 | 0.243303 | -0.09683 | 0.381693 | AMR | Disorders of the a | neurological | |
| 337.1 | 0.236066 | 0.147463 | 1.600851 | 0.10941 | -0.05296 | 0.525088 | AMR | Peripheral auton | neurological | |
| 338 | 0.074021 | 0.031429 | 2.355162 | 0.018515 | 0.012421 | 0.135621 | AMR | Pain | neurological | |
| 338.1 | 0.089965 | 0.032541 | 2.764644 | 0.005698 | 0.026185 | 0.153744 | AMR | Acute pain | neurological | |
| 338.2 | 0.078684 | 0.032869 | 2.393863 | 0.016672 | 0.014262 | 0.143107 | AMR | Chronic pain | neurological | |
| 339 | 0.049753 | 0.035065 | 1.418895 | 0.15593 | -0.01897 | 0.118478 | AMR | Other headache | neurological | |
| 340 | 0.081657 | 0.044849 | 1.820695 | 0.068653 | -0.00625 | 0.16956 | AMR | Migraine | neurological | |
| 340.1 | 0.114731 | 0.074985 | 1.53E+00 | 0.126001 | -0.03224 | 0.261698 | AMR | Migrain with aura | neurological | |
| 341 | 0.009546 | 0.225341 | 4.24E-02 | 0.966209 | -0.43211 | 0.451207 | AMR | Other demyelinat | neurological | |
| 342 | 0.061735 | 0.133502 | 0.462424 | 0.643777 | -0.19993 | 0.323395 | AMR | Hemiplegia | neurological | |
| 343 | 0.168954 | 0.190843 | 0.885301 | 0.375994 | -0.20509 | 0.542999 | AMR | Infantile cerebral | neurological | |
| 344 | 0.033998 | 0.117208 | 0.290065 | 0.771767 | -0.19573 | 0.263721 | AMR | Other paralytic sy | neurological | |
| 345 | 0.074467 | 0.059217 | 1.257524 | 0.208564 | -0.0416 | 0.190531 | AMR | Epilepsy, recurre | neurological | |
| 345.1 | 0.06451 | 0.082888 | 0.778281 | 0.436403 | -0.09795 | 0.226967 | AMR | Epilepsy | neurological | |
| 345.11 | 0.037315 | 0.129987 | 0.287064 | 0.774063 | -0.21746 | 0.292084 | AMR | Generalized conv | neurological | |
| 345.12 | -0.06248 | 0.118737 | -0.52621 | 0.59874 | -0.2952 | 0.17024 | AMR | Partial epilepsy | neurological | |
| 345.3 | 0.09905 | 0.061518 | 1.610104 | 0.107375 | -0.02152 | 0.219622 | AMR | Convulsions | neurological | |
| 346 | 0.414914 | 0.184756 | 2.245741 | 0.024721 | 0.052799 | 0.777028 | AMR | Abnormal finding | neurological | |
| 346.1 | 0.041121 | 0.08154 | 0.504305 | 0.614047 | -0.11869 | 0.200936 | AMR | Nonspecific abnc | neurological | |
| 346.2 | 0.437951 | 0.187499 | 2.335752 | 0.019504 | 0.07046 | 0.805442 | AMR | Nonspecific abnc | neurological | |
| 346.3 | 0.008363 | 0.210747 | 0.039683 | 0.968346 | -0.40469 | 0.421419 | AMR | Nonspecific abnc | neurological | |
| 347 | 0.522333 | 0.432346 | 1.208136 | 0.226995 | -0.32505 | 1.369717 | AMR | Cataplexy and na | neurological | |
| 348 | 0.070934 | 0.050775 | 1.397028 | 0.162405 | -0.02858 | 0.170451 | AMR | Other conditions | neurological | |
| 348.2 | -0.06269 | 0.078532 | -0.79827 | 0.424716 | -0.21661 | 0.09123 | AMR | Cerebral edema | neurological | |
| 348.4 | -0.14878 | 0.193892 | -0.76733 | 0.442885 | -0.5288 | 0.231243 | AMR | Cerebral cysts | neurological | |
| 348.7 | 0.104223 | 0.101708 | 1.024725 | 0.305493 | -0.09512 | 0.303566 | AMR | Coma | neurological | |

| | | | | | | | | | | |
|---|---|---|---|---|---|---|---|---|---|---|
| 348.8 | 0.106626 | 6.19E-02 | 1.723849 | 0.084735 | -0.0146 | 0.227857 | AMR | Encephalopathy, | neurological | |
| 348.9 | 0.03965 | 0.065853 | 0.602105 | 0.547104 | -0.08942 | 0.168719 | AMR | Other conditions | neurological | |
| 349 | 0.025548 | 0.073513 | 0.347524 | 0.728198 | -0.11854 | 0.16963 | AMR | Other and unspe | neurological | |
| 350 | 0.081378 | 0.043513 | 1.87E+00 | 0.061459 | -0.00391 | 0.166663 | AMR | Abnormal moven | neurological | |
| 350.1 | 0.095643 | 0.054641 | 1.750392 | 0.080051 | -0.01145 | 0.202738 | AMR | Abnormal involur | neurological | |
| 350.2 | 0.133228 | 0.057603 | 2.312857 | 0.02073 | 0.020328 | 0.246128 | AMR | Abnormality of ga | neurological | |
| 350.3 | -0.19805 | 0.129284 | -1.53188 | 0.125553 | -0.45144 | 0.055345 | AMR | Lack of coordinat | neurological | |
| 350.5 | -0.05643 | 0.207132 | -0.27245 | 0.785275 | -0.46241 | 0.349538 | AMR | Abnormal reflex | neurological | |
| 350.6 | -0.16173 | 0.096417 | -1.67745 | 0.093454 | -0.35071 | 0.027239 | AMR | Disturbances of s | neurological | |
| 351 | 0.015605 | 0.03967 | 0.393375 | 0.694042 | -0.06215 | 0.093357 | AMR | Other peripheral | neurological | |
| 352 | 0.208051 | 0.087221 | 2.385343 | 0.017063 | 0.037102 | 0.379 | AMR | Disorders of othe | neurological | |
| 352.1 | 0.439077 | 0.154891 | 2.834756 | 0.004586 | 0.135497 | 0.742657 | AMR | Trigeminal nerve | neurological | |
| 352.2 | 0.124425 | 0.102503 | 1.21386 | 0.224801 | -0.07648 | 0.325328 | AMR | Facial nerve disc | neurological | |
| 353 | -0.05489 | 0.116315 | -0.47187 | 0.63702 | -0.28286 | 0.173087 | AMR | Nerve root and p | neurological | |
| 353.1 | -0.14247 | 0.15035 | -9.48E-01 | 0.343351 | -0.43715 | 0.152214 | AMR | Nerve plexus lesi | neurological | |
| 353.2 | 0.32628 | 0.435685 | 0.748889 | 0.453924 | -0.52765 | 1.180207 | AMR | Nerve root lesion | neurological | |
| 355 | -0.0184 | 0.145268 | -0.12666 | 0.899213 | -0.30312 | 0.266322 | AMR | Complex regiona | neurological | |
| 355.1 | 0.188669 | 0.096347 | 1.958211 | 0.050205 | -0.00017 | 0.377506 | AMR | Chronic pain syn | neurological | |
| 356 | 0.017253 | 0.070965 | 0.243116 | 0.807916 | -0.12184 | 0.156341 | AMR | Hereditary and id | neurological | |
| 357 | 0.042722 | 0.043639 | 0.978995 | 0.327582 | -0.04281 | 0.128253 | AMR | Inflammatory and | neurological | |
| 358 | -0.16257 | 0.148756 | -1.09288 | 0.274448 | -0.45413 | 0.128985 | AMR | Myoneural disorc | neurological | |
| 358.1 | -0.40679 | 0.294514 | -1.38121 | 0.167213 | -0.98402 | 0.17045 | AMR | Myasthenia gravi | neurological | |
| 359 | -0.02267 | 0.084611 | -0.26792 | 0.788758 | -0.1885 | 0.143165 | AMR | Muscular dystrop | neurological | |
| 359.1 | -0.36326 | 0.315744 | -1.15049 | 0.249941 | -0.98211 | 0.255585 | AMR | Muscular dystrop | neurological | |
| 359.2 | 0.01075 | 0.086403 | 0.124419 | 0.900983 | -0.1586 | 0.180097 | AMR | Myopathy | neurological | |
| 360 | -0.18603 | 0.178427 | -1.04E+00 | 0.297127 | -0.53574 | 0.16368 | AMR | Disorders of the g | sense organs | |
| 360.2 | -0.00555 | 0.225796 | -0.02459 | 0.980384 | -0.4481 | 0.437001 | AMR | Progressive myo | sense organs | |
| 360.3 | 0.185873 | 0.370179 | 0.502117 | 0.615586 | -0.53966 | 0.911411 | AMR | Hypotony of eye | sense organs | |
| 361 | -0.08823 | 0.107727 | -0.81904 | 0.412763 | -0.29937 | 0.122908 | AMR | Retinal detachme | sense organs | |
| 361.1 | 0.017666 | 0.187488 | 0.094226 | 0.92493 | -0.3498 | 0.385136 | AMR | Retinal detachme | sense organs | |
| 361.2 | 0.662141 | 0.387731 | 1.71E+00 | 0.087686 | -0.0978 | 1.42208 | AMR | Retinoschisis and | sense organs | |
| 362 | -0.05421 | 0.058867 | -0.92085 | 0.357128 | -0.16958 | 0.061169 | AMR | Other retinal disc | sense organs | |
| 362.2 | -0.13378 | 0.077162 | -1.73375 | 0.082963 | -0.28501 | 0.017455 | AMR | Degeneration of | sense organs | |
| 362.21 | -0.06836 | 0.168737 | -0.40512 | 0.685386 | -0.39908 | 0.262358 | AMR | Macular degener | sense organs | |
| 362.22 | 0.619427 | 0.254427 | 2.434598 | 0.014908 | 0.12076 | 1.118095 | AMR | Macular degener | sense organs | |
| 362.23 | -0.15322 | 0.160746 | -0.95317 | 0.340504 | -0.46828 | 0.161838 | AMR | Cystoid macular | sense organs | |
| 362.26 | -0.10951 | 0.114862 | -9.53E-01 | 0.340368 | -0.33464 | 0.115611 | AMR | Macular puckerin | sense organs | |
| 362.27 | -0.19963 | 0.145231 | -1.37455 | 0.169272 | -0.48428 | 0.085021 | AMR | Drusen (degener | sense organs | |

| | | | | | | | | | | |
|---|---|---|---|---|---|---|---|---|---|---|
| 362.29 | -0.06478 | 0.145783 | -4.44E-01 | 0.656772 | -0.35051 | 0.220948 | AMR | Macular degener | sense organs | |
| 362.3 | 0.065426 | 0.147886 | 4.42E-01 | 0.658192 | -0.22442 | 0.355277 | AMR | Other nondiabeti | sense organs | |
| 362.31 | 0.760628 | 0.208317 | 3.651293 | 0.000261 | 0.352333 | 1.168923 | AMR | Separation of ret | sense organs | |
| 362.4 | -0.07996 | 0.142182 | -5.62E-01 | 0.573858 | -0.35863 | 0.198711 | AMR | Retinal vascular | sense organs | |
| 362.5 | 0.208974 | 0.42342 | 0.493539 | 0.621632 | -0.62091 | 1.038863 | AMR | Toxic maculopath | sense organs | |
| 362.6 | 0.016354 | 0.156883 | 0.104241 | 0.916978 | -0.29113 | 0.32384 | AMR | Peripheral retinal | sense organs | |
| 362.7 | -0.05579 | 0.272503 | -0.20472 | 0.837791 | -0.58988 | 0.47831 | AMR | Hereditary retinal | sense organs | |
| 362.8 | -0.12948 | 0.188147 | -0.68819 | 0.49133 | -0.49824 | 0.239279 | AMR | Retinal hemorrha | sense organs | |
| 362.9 | 0.078321 | 0.145241 | 0.539249 | 0.589715 | -0.20635 | 0.362989 | AMR | Retinal edema | sense organs | |
| 363 | 0.033664 | 0.152693 | 2.20E-01 | 0.825508 | -0.26561 | 0.332937 | AMR | Chorioretinal infla | sense organs | |
| 363.3 | 0.139768 | 0.183267 | 0.762645 | 0.445675 | -0.21943 | 0.498965 | AMR | Chorioretinal sca | sense organs | |
| 363.4 | -1.60267 | 1.490631 | -1.08E+00 | 0.282302 | -4.52425 | 1.318914 | AMR | Choroidal degen | sense organs | |
| 364 | -0.09275 | 0.106913 | -0.86751 | 0.385663 | -0.30229 | 0.116797 | AMR | Corneal opacity a | sense organs | |
| 364.1 | 0.165522 | 0.18607 | 0.889569 | 0.373697 | -0.19917 | 0.530213 | AMR | Corneal opacity | sense organs | |
| 364.2 | 0.722863 | 0.334624 | 2.160225 | 0.030755 | 0.067012 | 1.378714 | AMR | Corneal edema | sense organs | |
| 364.4 | 0.047944 | 0.169173 | 0.283401 | 0.77687 | -0.28363 | 0.379516 | AMR | Corneal degener | sense organs | |
| 364.41 | 0.293179 | 0.238943 | 1.226985 | 0.219828 | -0.17514 | 0.761498 | AMR | Keratoconus | sense organs | |
| 364.5 | 0.511134 | 0.222059 | 2.301798 | 0.021347 | 0.075907 | 0.946361 | AMR | Corneal dystroph | sense organs | |
| 364.51 | 0.124531 | 0.363501 | 0.342588 | 0.731909 | -0.58792 | 0.83698 | AMR | Fuchs' dystrophy | sense organs | |
| 364.9 | 0.266367 | 0.256755 | 1.037437 | 0.299532 | -0.23686 | 0.769597 | AMR | Cornea replaced | sense organs | |
| 365 | -0.04583 | 0.056671 | -8.09E-01 | 0.418639 | -0.15691 | 0.065238 | AMR | Glaucoma | sense organs | |
| 365.1 | -0.03108 | 0.096335 | -0.32259 | 0.747009 | -0.21989 | 0.157737 | AMR | Open-angle glau | sense organs | |
| 365.11 | -0.09054 | 0.117737 | -0.769 | 0.441891 | -0.3213 | 0.14022 | AMR | Primary open an | sense organs | |
| 365.2 | -0.21421 | 0.126799 | -1.68937 | 0.091148 | -0.46273 | 0.034311 | AMR | Primary angle-clo | sense organs | |
| 365.5 | 0.786626 | 0.350586 | 2.243747 | 0.024849 | 0.09949 | 1.473761 | AMR | Pseudoexfoliatio | sense organs | |
| 366 | -0.07461 | 0.047237 | -1.5795 | 0.114221 | -0.16719 | 0.017972 | AMR | Cataract | sense organs | |
| 366.1 | 0.030422 | 0.221873 | 0.137116 | 0.890939 | -0.40444 | 0.465286 | AMR | Nonsenile Catara | sense organs | |
| 366.2 | 0.046471 | 0.051259 | 0.906578 | 0.36463 | -0.054 | 0.146937 | AMR | Senile cataract | sense organs | |
| 367 | -0.06703 | 0.045929 | -1.45938 | 0.144462 | -0.15705 | 0.022992 | AMR | Disorders of refra | sense organs | |
| 367.1 | -0.02436 | 0.087872 | -0.27728 | 0.781569 | -0.19659 | 0.147861 | AMR | Myopia | sense organs | |
| 367.2 | -0.05089 | 0.076717 | -0.66332 | 0.507128 | -0.20125 | 0.099475 | AMR | Astigmatism | sense organs | |
| 367.4 | -0.02277 | 0.075905 | -3.00E-01 | 0.764179 | -0.17154 | 0.125999 | AMR | Presbyopia | sense organs | |
| 367.8 | -0.08295 | 0.117948 | -0.70327 | 0.481885 | -0.31412 | 0.148224 | AMR | Hypermetropia | sense organs | |
| 367.9 | -0.16806 | 0.07209 | -2.33E+00 | 0.019741 | -0.30935 | -0.02677 | AMR | Blindness and lo | sense organs | |
| 368 | 0.038386 | 0.043072 | 0.891193 | 0.372825 | -0.04603 | 0.122806 | AMR | Visual disturbanc | sense organs | |
| 368.1 | 0.279133 | 0.169646 | 1.645383 | 0.099891 | -0.05337 | 0.611632 | AMR | Amblyopia | sense organs | |
| 368.2 | 0.0137 | 0.10572 | 1.30E-01 | 0.896892 | -0.19351 | 0.220907 | AMR | Diplopia and disc | sense organs | |
| 368.3 | 0.208485 | 0.270961 | 0.76943 | 0.441638 | -0.32259 | 0.739558 | AMR | Anisometropia | sense organs | |

| | | | | | | | | | | |
|---|---|---|---|---|---|---|---|---|---|---|
| 368.4 | 0.096956 | 0.114063 | 0.850017 | 0.395316 | -0.1266 | 0.320516 | AMR | Visual field defec | sense organs | |
| 368.5 | 1.823889 | 1.367657 | 1.333586 | 0.182339 | -0.85667 | 4.504447 | AMR | Color vision defic | sense organs | |
| 368.7 | -2.04839 | 0.931868 | -2.19816 | 0.027938 | -3.87482 | -0.22197 | AMR | Disorders of acco | sense organs | |
| 368.9 | 0.029891 | 0.089225 | 0.335008 | 0.737619 | -0.14499 | 0.20477 | AMR | Subjective visual | sense organs | |
| 368.91 | 0.337165 | 0.188146 | 1.792036 | 0.073127 | -0.03159 | 0.705925 | AMR | Psychophysical v | sense organs | |
| 369 | 0.014611 | 0.055778 | 0.261949 | 0.793361 | -0.09471 | 0.123934 | AMR | Infection of the e | sense organs | |
| 369.2 | -0.00791 | 0.132122 | -0.05989 | 0.952242 | -0.26687 | 0.251041 | AMR | Eye infection, vira | sense organs | |
| 369.5 | -0.00738 | 0.060043 | -0.12284 | 0.902231 | -0.12506 | 0.110307 | AMR | Conjunctivitis, inf | sense organs | |
| 370 | 0.100171 | 0.110422 | 0.907163 | 0.364321 | -0.11625 | 0.316595 | AMR | Keratitis | sense organs | |
| 370.1 | 0.206375 | 0.205459 | 1.004459 | 0.315158 | -0.19632 | 0.609066 | AMR | Corneal ulcer | sense organs | |
| 370.2 | 0.344607 | 0.215405 | 1.59981 | 0.109641 | -0.07758 | 0.766793 | AMR | Superficial kerati | sense organs | |
| 370.3 | 0.318829 | 0.165268 | 1.929161 | 0.053711 | -0.00509 | 0.642748 | AMR | Keratoconjunctivi | sense organs | |
| 370.31 | 0.309866 | 2.17E-01 | 1.429886 | 0.15275 | -0.11487 | 0.734604 | AMR | Keratoconjunctivi | sense organs | |
| 371 | -0.08571 | 0.049259 | -1.73996 | 0.081865 | -0.18226 | 0.010837 | AMR | Inflammation of t | sense organs | |
| 371.1 | 0.086296 | 1.59E-01 | 0.541783 | 0.587968 | -0.22589 | 0.398484 | AMR | Uveitis, noninfect | sense organs | |
| 371.2 | -0.0119 | 0.084108 | -1.41E-01 | 0.887493 | -0.17675 | 0.15295 | AMR | Conjunctivitis, no | sense organs | |
| 371.21 | -0.0396 | 0.091716 | -0.43172 | 0.665942 | -0.21936 | 0.140165 | AMR | Allergic conjuncti | sense organs | |
| 371.3 | -0.08533 | 0.057192 | -1.49207 | 0.135681 | -0.19743 | 0.02676 | AMR | Inflammation of e | sense organs | |
| 371.33 | 0.221482 | 0.228897 | 0.967605 | 0.333242 | -0.22715 | 0.670111 | AMR | Noninfectious de | sense organs | |
| 371.9 | 0.230075 | 0.368194 | 0.624875 | 0.532053 | -0.49157 | 0.951722 | AMR | Chronic inflamma | sense organs | |
| 372 | 0.077004 | 0.06268 | 1.23E+00 | 0.219254 | -0.04585 | 0.199855 | AMR | Disorders of conj | sense organs | |
| 374 | -0.02943 | 0.062818 | -0.46848 | 0.639441 | -0.15255 | 0.093692 | AMR | Other disorders o | sense organs | |
| 374.1 | -0.01393 | 0.160148 | -0.08699 | 0.93068 | -0.32782 | 0.299954 | AMR | Ectropion or entr | sense organs | |
| 374.2 | 0.065567 | 1.97E-01 | 0.332752 | 0.739321 | -0.32063 | 0.451764 | AMR | Lagophthalmos | sense organs | |
| 374.3 | -0.07881 | 0.096518 | -0.81651 | 0.41421 | -0.26798 | 0.110364 | AMR | Ptosis of eyelid | sense organs | |
| 374.6 | -0.17426 | 0.122528 | -1.42219 | 0.154972 | -0.41441 | 0.065893 | AMR | Dermatochalasis | sense organs | |
| 375 | 0.096779 | 0.107065 | 9.04E-01 | 0.366034 | -0.11306 | 0.306622 | AMR | Disorders of lacri | sense organs | |
| 375.1 | 0.024073 | 0.051135 | 0.470773 | 0.637803 | -0.07615 | 0.124295 | AMR | Dry eyes | sense organs | |
| 375.2 | 0.178354 | 0.114639 | 1.555781 | 0.11976 | -0.04634 | 0.403043 | AMR | Epiphora | sense organs | |
| 376 | -0.08091 | 0.209187 | -0.38679 | 0.698913 | -0.49091 | 0.329088 | AMR | Disorders of the o | sense organs | |
| 377 | -0.04701 | 0.098438 | -0.4776 | 0.632936 | -0.23995 | 0.145921 | AMR | Disorders of optic | sense organs | |
| 377.1 | 0.066184 | 0.140775 | 0.470138 | 0.638256 | -0.20973 | 0.342097 | AMR | Optic atrophy | sense organs | |
| 377.3 | -0.12425 | 0.153457 | -0.80965 | 0.418143 | -0.42502 | 0.176524 | AMR | Optic neuritis/neu | sense organs | |
| 378 | -0.00442 | 0.085643 | -0.05166 | 0.958798 | -0.17228 | 0.163432 | AMR | Strabismus and o | sense organs | |
| 378.1 | 0.041717 | 0.114815 | 0.363346 | 0.716347 | -0.18332 | 0.26675 | AMR | Strabismus (not s | sense organs | |
| 378.2 | 0.029259 | 0.180403 | 0.162186 | 0.871159 | -0.32432 | 0.382841 | AMR | Nystagmus and o | sense organs | |
| 378.5 | 0.188591 | 0.197146 | 0.956608 | 0.338765 | -0.19781 | 0.574989 | AMR | Paralytic strabism | sense organs | |
| 379 | -0.05237 | 0.051235 | -1.02E+00 | 0.306675 | -0.15279 | 0.048045 | AMR | Other disorders o | sense organs | |

| | | | | | | | | | | |
|---|---|---|---|---|---|---|---|---|---|---|
| 379.1 | -0.3368 | 0.22133 | -1.52171 | 0.128081 | -0.7706 | 0.096998 | AMR | Scleritis and epis | sense organs | |
| 379.2 | -0.02902 | 0.063709 | -4.56E-01 | 0.648731 | -0.15389 | 0.095846 | AMR | Disorders of vitre | sense organs | |
| 379.3 | 0.144429 | 0.23823 | 6.06E-01 | 0.544343 | -0.32249 | 0.61135 | AMR | Aphakia and othe | sense organs | |
| 379.4 | 0.227254 | 0.175358 | 1.295943 | 0.194995 | -0.11644 | 0.570949 | AMR | Anomalies of pup | sense organs | |
| 379.5 | -0.00891 | 0.164246 | -5.43E-02 | 0.956718 | -0.33083 | 0.313002 | AMR | Disorders of iris a | sense organs | |
| 379.51 | 0.059066 | 0.346055 | 0.170685 | 0.864472 | -0.61919 | 0.737322 | AMR | Pigmentary iris d | sense organs | |
| 379.9 | -0.02636 | 0.058554 | -0.45012 | 0.652624 | -0.14112 | 0.088407 | AMR | Pain, swelling or | sense organs | |
| 380 | 0.027968 | 0.146563 | 0.190826 | 0.848662 | -0.25929 | 0.315226 | AMR | Disorders of exte | sense organs | |
| 380.1 | -0.07449 | 0.078416 | -0.94989 | 0.342166 | -0.22818 | 0.079205 | AMR | Otitis externa | sense organs | |
| 380.4 | 0.059788 | 0.054282 | 1.101425 | 0.270712 | -0.0466 | 0.166179 | AMR | Impacted cerume | sense organs | |
| 381 | 0.114424 | 0.057127 | 2.00E+00 | 0.045178 | 0.002458 | 0.226391 | AMR | Otitis media and | sense organs | |
| 381.1 | 0.169826 | 0.067352 | 2.521462 | 0.011687 | 0.037818 | 0.301834 | AMR | Otitis media | sense organs | |
| 381.11 | 0.169612 | 0.078874 | 2.15E+00 | 0.031521 | 0.015023 | 0.324202 | AMR | Suppurative and | sense organs | |
| 381.2 | 0.04605 | 0.090853 | 5.07E-01 | 0.612252 | -0.13202 | 0.22412 | AMR | Eustachian tube | sense organs | |
| 381.3 | 0.502249 | 0.197907 | 2.537798 | 0.011155 | 0.114358 | 0.890141 | AMR | Mastoiditis & rela | sense organs | |
| 381.9 | 0.015093 | 0.141009 | 1.07E-01 | 0.914763 | -0.26128 | 0.291464 | AMR | Otorrhea | sense organs | |
| 382 | 0.047521 | 0.055087 | 0.862654 | 0.388328 | -0.06045 | 0.155488 | AMR | Otalgia | sense organs | |
| 383 | 0.008785 | 0.306803 | 0.028635 | 0.977156 | -0.59254 | 0.610108 | AMR | Otosclerosis | sense organs | |
| 384 | 0.061544 | 0.152206 | 0.404347 | 0.685957 | -0.23677 | 0.359862 | AMR | Other disorders c | sense organs | |
| 384.1 | 0.403563 | 0.44896 | 0.898884 | 0.368715 | -0.47638 | 1.283509 | AMR | Myringitis | sense organs | |
| 384.4 | 0.113583 | 0.186451 | 0.609185 | 0.542401 | -0.25185 | 0.479021 | AMR | Perforation of tyn | sense organs | |
| 385 | 0.21828 | 0.180957 | 1.21E+00 | 0.22772 | -0.13639 | 0.57295 | AMR | Other disorders c | sense organs | |
| 385.3 | 0.552679 | 0.374749 | 1.4748 | 0.140266 | -0.18181 | 1.287174 | AMR | Cholesteatoma | sense organs | |
| 385.5 | 0.499663 | 0.229874 | 2.173639 | 0.029732 | 0.049118 | 0.950208 | AMR | Tympanosclerosi | sense organs | |
| 386 | 0.096868 | 0.066351 | 1.459944 | 0.144305 | -0.03318 | 0.226913 | AMR | Vertiginous syndr | sense organs | |
| 386.1 | 0.23285 | 0.184181 | 1.264248 | 0.206141 | -0.12814 | 0.593837 | AMR | Meniere's diseas | sense organs | |
| 386.2 | 0.005754 | 0.075679 | 7.60E-02 | 0.939399 | -0.14257 | 0.154082 | AMR | Peripheral or cen | sense organs | |
| 386.21 | 1.697494 | 0.571533 | 2.970069 | 0.002977 | 0.577309 | 2.817679 | AMR | Central origin ver | sense organs | |
| 386.3 | 0.256132 | 0.228477 | 1.121043 | 0.262269 | -0.19167 | 0.703938 | AMR | Labyrinthitis | sense organs | |
| 386.9 | 0.051586 | 0.03751 | 1.375253 | 0.169053 | -0.02193 | 0.125105 | AMR | Dizziness and gic | sense organs | |
| 388 | 0.125526 | 0.072088 | 1.741281 | 0.081634 | -0.01576 | 0.266817 | AMR | Other disorders c | sense organs | |
| 389 | 0.098878 | 0.042104 | 2.348414 | 0.018854 | 0.016355 | 0.1814 | AMR | Hearing loss | sense organs | |
| 389.1 | 0.112845 | 0.059008 | 1.91E+00 | 0.055829 | -0.00281 | 0.2285 | AMR | Sensorineural he | sense organs | |
| 389.2 | 0.005699 | 0.130313 | 0.043732 | 0.965118 | -0.24971 | 0.261109 | AMR | Conductive heari | sense organs | |
| 389.3 | 0.150294 | 0.227515 | 0.660591 | 0.508875 | -0.29563 | 0.596215 | AMR | Degenerative anc | sense organs | |
| 389.4 | 0.096412 | 0.058821 | 1.639068 | 0.101199 | -0.01888 | 0.211699 | AMR | Tinnitus | sense organs | |
| 389.5 | 0.148548 | 0.169697 | 0.875372 | 0.381371 | -0.18405 | 0.481148 | AMR | Disorders of acou | sense organs | |
| 394 | 0.004537 | 0.060316 | 7.52E-02 | 0.940044 | -0.11368 | 0.122755 | AMR | Rheumatic disea | circulatory system | |

| | | | | | | | | | |
|---|---|---|---|---|---|---|---|---|---|
| 394.1 | -0.19565 | 0.160578 | -1.21843 | 0.223059 | -0.51038 | 0.119073 | AMR | Mitral valve stenc | circulatory system |
| 394.2 | -0.08971 | 0.093185 | -0.96273 | 0.335685 | -0.27235 | 0.092928 | AMR | Mitral valve disea | circulatory system |
| 394.3 | 0.220329 | 0.239999 | 0.918039 | 0.358599 | -0.25006 | 0.690719 | AMR | Aortic valve disea | circulatory system |
| 394.4 | 0.128008 | 0.356497 | 0.359072 | 0.719541 | -0.57071 | 0.82673 | AMR | Acute rheumatic | circulatory system |
| 394.7 | 0.07286 | 0.087125 | 0.836274 | 0.403001 | -0.0979 | 0.243622 | AMR | Disease of tricus | circulatory system |
| 395 | -0.08559 | 0.046144 | -1.85E+00 | 0.063606 | -0.17604 | 0.004847 | AMR | Heart valve disor | circulatory system |
| 395.1 | -0.06788 | 0.061936 | -1.09597 | 0.273093 | -0.18927 | 0.053512 | AMR | Nonrheumatic mi | circulatory system |
| 395.2 | -0.10305 | 0.064014 | -1.60973 | 0.107457 | -0.22851 | 0.02242 | AMR | Nonrheumatic ac | circulatory system |
| 395.3 | 0.042259 | 0.069734 | 0.605997 | 0.544517 | -0.09442 | 0.178936 | AMR | Nonrheumatic tri | circulatory system |
| 395.4 | -0.05452 | 0.129185 | -0.42202 | 0.673009 | -0.30772 | 0.198679 | AMR | Nonrheumatic pu | circulatory system |
| 395.6 | -0.00107 | 0.086992 | -0.01227 | 0.990211 | -0.17157 | 0.169434 | AMR | Heart valve repla | circulatory system |
| 396 | 0.044778 | 0.056527 | 0.792146 | 0.428276 | -0.06601 | 0.15557 | AMR | Abnormal heart s | circulatory system |
| 401 | 0.131688 | 0.035629 | 3.696073 | 0.000219 | 0.061856 | 0.20152 | AMR | Hypertension | circulatory system |
| 401.1 | 0.117612 | 0.03557 | 3.306498 | 0.000945 | 0.047896 | 0.187328 | AMR | Essential hyperte | circulatory system |
| 401.2 | 0.070363 | 0.040372 | 1.742886 | 0.081353 | -0.00876 | 0.14949 | AMR | Hypertensive hea | circulatory system |
| 401.21 | 0.099771 | 0.062094 | 1.606771 | 0.108105 | -0.02193 | 0.221474 | AMR | Hypertensive hea | circulatory system |
| 401.22 | 0.043863 | 0.044504 | 9.86E-01 | 0.324334 | -0.04336 | 0.131088 | AMR | Hypertensive chr | circulatory system |
| 401.3 | 0.041386 | 0.046176 | 0.896263 | 0.370112 | -0.04912 | 0.131888 | AMR | Other hypertensi | circulatory system |
| 402 | 0.078283 | 0.054857 | 1.427031 | 0.153571 | -0.02924 | 0.1858 | AMR | Elevated blood p | circulatory system |
| 411 | 0.076 | 0.040444 | 1.879114 | 0.060229 | -0.00327 | 0.155269 | AMR | Ischemic Heart D | circulatory system |
| 411.1 | 0.241025 | 0.103306 | 2.333116 | 0.019642 | 0.038549 | 0.443501 | AMR | Unstable angina | circulatory system |
| 411.2 | 0.144066 | 0.060259 | 2.390795 | 0.016812 | 0.025961 | 0.26217 | AMR | Myocardial infarc | circulatory system |
| 411.3 | 0.09227 | 0.064277 | 1.435508 | 0.151143 | -0.03371 | 0.218251 | AMR | Angina pectoris | circulatory system |
| 411.4 | 0.064691 | 0.042741 | 1.513562 | 0.130137 | -0.01908 | 0.148463 | AMR | Coronary atheros | circulatory system |
| 411.41 | -0.13889 | 0.228243 | -0.60851 | 0.542852 | -0.58623 | 0.308461 | AMR | Aneurysm and di | circulatory system |
| 411.8 | 0.180177 | 0.076864 | 2.344105 | 0.019073 | 0.029527 | 0.330828 | AMR | Other chronic isc | circulatory system |
| 411.9 | -0.10612 | 0.125817 | -0.84343 | 0.398985 | -0.35272 | 0.140479 | AMR | Other acute and | circulatory system |
| 414 | -0.02347 | 0.062659 | -3.75E-01 | 0.708001 | -0.14628 | 0.099341 | AMR | Other forms of ch | circulatory system |
| 414.2 | -0.02892 | 0.234088 | -0.12353 | 0.901687 | -0.48772 | 0.429888 | AMR | ASCVD | circulatory system |
| 415 | 0.070715 | 0.048835 | 1.448058 | 0.147601 | -0.025 | 0.16643 | AMR | Pulmonary heart | circulatory system |
| 415.1 | 0.05989 | 0.077726 | 0.77053 | 0.440985 | -0.09245 | 0.212231 | AMR | Acute pulmonary | circulatory system |
| 415.11 | 0.05989 | 0.077726 | 0.77053 | 0.440985 | -0.09245 | 0.212231 | AMR | Pulmonary embo | circulatory system |
| 415.2 | 0.04421 | 0.055332 | 0.798987 | 0.424298 | -0.06424 | 0.152659 | AMR | Chronic pulmona | circulatory system |
| 415.21 | 0.05743 | 0.087321 | 0.657693 | 0.510736 | -0.11372 | 0.228575 | AMR | Primary pulmona | circulatory system |
| 416 | 0.090985 | 0.042086 | 2.161874 | 0.030628 | 0.008498 | 0.173473 | AMR | Cardiomegaly | circulatory system |
| 418 | 0.081381 | 0.032464 | 2.506824 | 0.012182 | 0.017753 | 0.145009 | AMR | Nonspecific ches | circulatory system |
| 418.1 | 0.072188 | 0.078119 | 0.924088 | 0.355441 | -0.08092 | 0.225298 | AMR | Precordial pain | circulatory system |
| 420 | 0.022852 | 0.056062 | 0.407621 | 0.683552 | -0.08703 | 0.132732 | AMR | Carditis | circulatory system |

| | | | | | | | | | |
|---|---|---|---|---|---|---|---|---|---|
| 420.1 | 0.272617 | 0.158578 | 1.72E+00 | 0.085589 | -0.03819 | 0.583424 | AMR | Myocarditis | circulatory system |
| 420.2 | -0.01433 | 0.063612 | -2.25E-01 | 0.821731 | -0.13901 | 0.110345 | AMR | Pericarditis | circulatory system |
| 420.21 | 0.196534 | 0.152631 | 1.287639 | 0.197872 | -0.10262 | 0.495685 | AMR | Acute pericarditis | circulatory system |
| 420.22 | 0.010438 | 0.172244 | 0.060598 | 0.951679 | -0.32716 | 0.34803 | AMR | Chronic pericardi | circulatory system |
| 420.3 | 0.061202 | 0.106022 | 0.577257 | 0.563766 | -0.1466 | 0.269001 | AMR | Endocarditis | circulatory system |
| 425 | 0.045343 | 0.058924 | 0.769517 | 0.441587 | -0.07015 | 0.160832 | AMR | Cardiomyopathy | circulatory system |
| 425.1 | 0.06038 | 0.061469 | 0.982283 | 0.32596 | -0.0601 | 0.180858 | AMR | Primary/intrinsic | circulatory system |
| 425.11 | 0.005734 | 0.215688 | 0.026587 | 0.978789 | -0.41701 | 0.428474 | AMR | Hypertrophic obs | circulatory system |
| 425.12 | -0.24996 | 0.182813 | -1.36731 | 0.171527 | -0.60827 | 0.108344 | AMR | Other hypertroph | circulatory system |
| 425.2 | 0.035547 | 0.09938 | 0.357683 | 0.72058 | -0.15923 | 0.230328 | AMR | Secondary/extrin | circulatory system |
| 425.8 | 0.095444 | 0.208651 | 0.457431 | 0.647361 | -0.31351 | 0.504393 | AMR | Other cardiomyo | circulatory system |
| 426 | 0.059708 | 0.037678 | 1.58E+00 | 0.113042 | -0.01414 | 0.133556 | AMR | Cardiac conducti | circulatory system |
| 426.2 | 0.021272 | 0.087696 | 0.242563 | 0.808344 | -0.15061 | 0.193152 | AMR | Atrioventricular [/ | circulatory system |
| 426.21 | -0.0073 | 0.131451 | -0.05554 | 0.955706 | -0.26494 | 0.250338 | AMR | First degree AV b | circulatory system |
| 426.22 | 0.720995 | 0.34523 | 2.088447 | 0.036758 | 0.044356 | 1.397634 | AMR | Mobitz II AV block | circulatory system |
| 426.23 | -0.25629 | 0.185618 | -1.38075 | 0.167355 | -0.6201 | 0.107512 | AMR | Second degree A | circulatory system |
| 426.24 | 0.09445 | 0.122784 | 0.769237 | 0.441752 | -0.1462 | 0.335103 | AMR | Atrioventricular b | circulatory system |
| 426.25 | 0.193749 | 0.228587 | 0.847591 | 0.396666 | -0.25427 | 0.641771 | AMR | Other heart block | circulatory system |
| 426.3 | 0.041569 | 0.071182 | 0.583982 | 0.559232 | -0.09795 | 0.181084 | AMR | Bundle branch bl | circulatory system |
| 426.31 | -0.01018 | 0.090056 | -0.11299 | 0.910035 | -0.18668 | 0.16633 | AMR | Right bundle bra | circulatory system |
| 426.32 | 0.074427 | 0.110986 | 0.670595 | 0.502479 | -0.1431 | 0.291956 | AMR | Left bundle branc | circulatory system |
| 426.4 | -1.16382 | 0.450611 | -2.58276 | 0.009801 | -2.047 | -0.28064 | AMR | Anomalous atriov | circulatory system |
| 426.7 | 0.051318 | 0.039782 | 1.29E+00 | 0.197051 | -0.02665 | 0.129289 | AMR | Abnormal electro | circulatory system |
| 426.8 | 0.0511 | 0.140288 | 0.364251 | 0.71567 | -0.22386 | 0.32606 | AMR | Other cardiac co | circulatory system |
| 426.9 | 0.000998 | 0.070962 | 0.014061 | 0.988781 | -0.13809 | 0.140081 | AMR | Cardiac pacemal | circulatory system |
| 426.91 | 0.067142 | 0.078698 | 0.853167 | 0.393567 | -0.0871 | 0.221387 | AMR | Cardiac pacemal | circulatory system |
| 426.92 | 0.066452 | 0.089547 | 0.74209 | 0.458033 | -0.10906 | 0.241962 | AMR | Cardiac defibrilla | circulatory system |
| 427 | 0.034858 | 0.03193 | 1.091691 | 0.274969 | -0.02772 | 0.097439 | AMR | Cardiac dysrhyth | circulatory system |
| 427.1 | -0.0111 | 0.051489 | -0.21564 | 0.829267 | -0.11202 | 0.089814 | AMR | Paroxysmal tach | circulatory system |
| 427.11 | -0.07088 | 0.066947 | -1.05881 | 0.289688 | -0.2021 | 0.06033 | AMR | Paroxysmal supr | circulatory system |
| 427.12 | 0.039177 | 0.068153 | 0.574841 | 0.565399 | -0.0944 | 0.172755 | AMR | Paroxysmal vent | circulatory system |
| 427.2 | 0.07034 | 0.053051 | 1.325897 | 0.184874 | -0.03364 | 0.174318 | AMR | Atrial fibrillation a | circulatory system |
| 427.21 | 0.070369 | 5.46E-02 | 1.287727 | 0.197841 | -0.03673 | 0.177472 | AMR | Atrial fibrillation | circulatory system |
| 427.22 | 0.063529 | 0.078952 | 0.804654 | 0.421019 | -0.09121 | 0.218271 | AMR | Atrial flutter | circulatory system |
| 427.3 | 0.033674 | 0.043831 | 0.768278 | 0.442322 | -0.05223 | 0.119581 | AMR | Other specified c | circulatory system |
| 427.4 | 0.019337 | 0.084667 | 0.228389 | 0.819344 | -0.14661 | 0.185281 | AMR | Cardiac arrest ar | circulatory system |
| 427.41 | -0.09507 | 0.146319 | -0.64974 | 0.515858 | -0.38185 | 0.191711 | AMR | Ventricular fibrilla | circulatory system |
| 427.42 | -0.00091 | 0.099528 | -0.00909 | 0.992744 | -0.19598 | 0.194166 | AMR | Cardiac arrest | circulatory system |

| | | | | | | | | | |
|---|---|---|---|---|---|---|---|---|---|
| 427.5 | 0.090274 | 0.064831 | 1.392455 | 0.163785 | -0.03679 | 0.217341 | AMR | Arrhythmia (cardi | circulatory system |
| 427.6 | 0.03473 | 0.066 | 0.526211 | 0.598742 | -0.09463 | 0.164089 | AMR | Premature beats | circulatory system |
| 427.61 | -0.08145 | 0.113697 | -0.71639 | 0.473749 | -0.30429 | 0.141391 | AMR | Supraventricular | circulatory system |
| 427.7 | 0.034434 | 4.29E-02 | 0.802097 | 0.422497 | -0.04971 | 0.118574 | AMR | Tachycardia NOS | circulatory system |
| 427.8 | -0.01476 | 0.088767 | -0.16632 | 0.867908 | -0.18874 | 0.159216 | AMR | Sinoatrial node d | circulatory system |
| 427.9 | -0.02376 | 4.33E-02 | -0.54823 | 0.583535 | -0.10869 | 0.061176 | AMR | Palpitations | circulatory system |
| 428 | 0.102856 | 0.048225 | 2.13E+00 | 0.032937 | 0.008337 | 0.197375 | AMR | Congestive heart | circulatory system |
| 428.1 | 0.087289 | 0.051217 | 1.704287 | 0.088327 | -0.0131 | 0.187672 | AMR | Congestive heart | circulatory system |
| 428.2 | 0.09584 | 0.067447 | 1.420976 | 0.155324 | -0.03635 | 0.228033 | AMR | Heart failure NOS | circulatory system |
| 428.3 | 0.115813 | 0.061472 | 1.884008 | 0.059564 | -0.00467 | 0.236296 | AMR | Heart failure with | circulatory system |
| 428.4 | 0.07081 | 0.06872 | 1.030422 | 0.302812 | -0.06388 | 0.205498 | AMR | Heart failure with | circulatory system |
| 429 | 0.036336 | 0.039267 | 0.925368 | 0.354775 | -0.04063 | 0.113298 | AMR | Ill-defined descrip | circulatory system |
| 429.1 | -0.01929 | 0.07024 | -0.27456 | 0.783651 | -0.15695 | 0.118383 | AMR | Heart transplant/s | circulatory system |
| 429.2 | -0.01674 | 0.084095 | -0.19909 | 0.842194 | -0.18156 | 0.14808 | AMR | Abnormal functio | circulatory system |
| 429.3 | 0.03577 | 0.041575 | 0.860377 | 0.389582 | -0.04572 | 0.117255 | AMR | Symptoms involv | circulatory system |
| 429.9 | 0.090344 | 0.129287 | 0.698789 | 0.484684 | -0.16305 | 0.343741 | AMR | Cardiac complica | circulatory system |
| 430 | -0.07607 | 0.083579 | -0.91015 | 0.362744 | -0.23988 | 0.087743 | AMR | Intracranial hemo | circulatory system |
| 430.1 | -0.10402 | 0.130589 | -0.79652 | 0.425732 | -0.35997 | 0.151934 | AMR | Subarachnoid he | circulatory system |
| 430.2 | -0.03418 | 0.116232 | -0.29405 | 0.768718 | -0.26199 | 0.193632 | AMR | Intracerebral hen | circulatory system |
| 430.3 | -0.11262 | 0.131837 | -0.85423 | 0.392976 | -0.37102 | 0.145777 | AMR | Subdural hemorr | circulatory system |
| 433 | 0.028309 | 0.044968 | 0.629521 | 0.529008 | -0.05983 | 0.116445 | AMR | Cerebrovascular | circulatory system |
| 433.1 | 0.037544 | 0.065906 | 0.569664 | 0.568906 | -0.09163 | 0.166717 | AMR | Occlusion and st | circulatory system |
| 433.11 | 0.215675 | 0.140348 | 1.536712 | 0.124364 | -0.0594 | 0.490753 | AMR | Occlusion of cere | circulatory system |
| 433.12 | 0.220471 | 0.183426 | 1.201963 | 0.229378 | -0.13904 | 0.579979 | AMR | Cerebral atheros | circulatory system |
| 433.2 | 0.014953 | 0.060883 | 0.245602 | 0.80599 | -0.10438 | 0.134281 | AMR | Occlusion of cere | circulatory system |
| 433.21 | 0.049146 | 6.20E-02 | 0.793185 | 0.42767 | -0.07229 | 0.170586 | AMR | Cerebral artery o | circulatory system |
| 433.3 | -0.03203 | 5.32E-02 | -0.60207 | 0.54713 | -0.13631 | 0.072244 | AMR | Cerebral ischemi | circulatory system |
| 433.31 | -0.07246 | 5.62E-02 | -1.29034 | 0.196932 | -0.18251 | 0.0376 | AMR | Transient cerebra | circulatory system |
| 433.32 | 0.23407 | 0.369808 | 6.33E-01 | 0.526766 | -0.49074 | 0.958881 | AMR | Moyamoya disea | circulatory system |
| 433.5 | 0.060961 | 0.115622 | 0.527245 | 0.598023 | -0.16565 | 0.287577 | AMR | Cerebral aneurys | circulatory system |
| 433.6 | 0.746981 | 0.235905 | 3.166442 | 0.001543 | 0.284615 | 1.209347 | AMR | Acute, but ill-defi | circulatory system |
| 433.8 | 0.004644 | 0.09353 | 0.049653 | 0.960399 | -0.17867 | 0.18796 | AMR | Late effects of ce | circulatory system |
| 440 | -0.00223 | 0.052193 | -0.04281 | 0.965855 | -0.10453 | 0.100063 | AMR | Atherosclerosis | circulatory system |
| 440.1 | -0.00413 | 0.164564 | -0.02509 | 0.979979 | -0.32667 | 0.31841 | AMR | Atherosclerosis c | circulatory system |
| 440.2 | -0.0473 | 0.090654 | -0.52181 | 0.601799 | -0.22498 | 0.130374 | AMR | Atherosclerosis c | circulatory system |
| 440.21 | 0.122492 | 0.159489 | 0.768024 | 0.442473 | -0.1901 | 0.435085 | AMR | Atherosclerosis c | circulatory system |
| 440.22 | -0.08764 | 0.151285 | -0.57933 | 0.562369 | -0.38416 | 0.20887 | AMR | Atherosclerosis c | circulatory system |
| 440.9 | 0.053744 | 0.059247 | 0.907107 | 0.36435 | -0.06238 | 0.169866 | AMR | Atherosclerosis c | circulatory system |

| | | | | | | | | | |
|---|---|---|---|---|---|---|---|---|---|
| 441 | 0.163951 | 0.126522 | 1.295831 | 0.195034 | -0.08403 | 0.41193 | AMR | Vascular insuffici | circulatory system |
| 441.1 | -0.00548 | 0.232795 | -2.36E-02 | 0.981206 | -0.46175 | 0.450786 | AMR | Acute vascular in | circulatory system |
| 441.2 | 0.225604 | 0.229028 | 0.985049 | 0.3246 | -0.22328 | 0.674492 | AMR | Chronic vascular | circulatory system |
| 442 | -0.04787 | 0.073342 | -0.65268 | 0.513963 | -0.19162 | 0.095879 | AMR | Other aneurysm | circulatory system |
| 442.1 | -0.04591 | 0.107041 | -0.42894 | 0.667968 | -0.25571 | 0.163883 | AMR | Aortic aneurysm | circulatory system |
| 442.11 | -0.108 | 0.166412 | -0.64898 | 0.516352 | -0.43416 | 0.218164 | AMR | Abdominal aortic | circulatory system |
| 442.2 | 0.870376 | 0.276494 | 3.147903 | 0.001644 | 0.328458 | 1.412294 | AMR | Aneurysm of iliac | circulatory system |
| 442.3 | -0.36875 | 0.230132 | -1.60233 | 0.109082 | -0.8198 | 0.082303 | AMR | Aneurysm of arte | circulatory system |
| 442.4 | 0.379984 | 0.250788 | 1.515162 | 0.129731 | -0.11155 | 0.871519 | AMR | Arterial dissectio | circulatory system |
| 442.8 | 0.085001 | 1.31E-01 | 0.646602 | 0.517889 | -0.17265 | 0.342654 | AMR | Aneurysm of othe | circulatory system |
| 443 | 0.031502 | 0.055765 | 0.564893 | 0.572147 | -0.0778 | 0.1408 | AMR | Peripheral vascu | circulatory system |
| 443.1 | 0.104983 | 0.111361 | 0.942728 | 0.34582 | -0.11328 | 0.323247 | AMR | Raynaud's syndr | circulatory system |
| 443.7 | 0.06001 | 0.096919 | 6.19E-01 | 0.5358 | -0.12995 | 0.249968 | AMR | Peripheral angio | circulatory system |
| 443.8 | -0.17356 | 0.136247 | -1.27387 | 0.202711 | -0.4406 | 0.093479 | AMR | Other specified p | circulatory system |
| 443.9 | -0.02541 | 0.068328 | -0.37184 | 0.710016 | -0.15933 | 0.108514 | AMR | Peripheral vascu | circulatory system |
| 444 | 0.046072 | 0.090443 | 0.509404 | 0.610469 | -0.13119 | 0.223338 | AMR | Arterial embolism | circulatory system |
| 444.1 | 0.080504 | 0.135644 | 0.593497 | 0.552848 | -0.18535 | 0.346361 | AMR | Arterial embolism | circulatory system |
| 444.2 | -0.27222 | 0.29855 | -0.91181 | 0.361867 | -0.85737 | 0.312925 | AMR | Embolism and th | circulatory system |
| 444.5 | 0.791858 | 0.524907 | 1.508569 | 0.131409 | -0.23694 | 1.820656 | AMR | Atheroembolism | circulatory system |
| 446 | 0.044382 | 0.113079 | 0.392487 | 0.694698 | -0.17725 | 0.266013 | AMR | Polyarteritis nodo | circulatory system |
| 446.1 | -0.80773 | 1.07E+00 | -0.7531 | 0.451387 | -2.90986 | 1.2944 | AMR | Thromboangiitis | circulatory system |
| 446.2 | -0.59115 | 0.75994 | -0.77789 | 0.436634 | -2.08061 | 0.898305 | AMR | Acute febrile muc | circulatory system |
| 446.3 | 0.772964 | 0.334889 | 2.308119 | 0.020993 | 0.116593 | 1.429335 | AMR | Hypersensitivity a | circulatory system |
| 446.4 | 0.47456 | 0.287045 | 1.65E+00 | 0.098279 | -0.08804 | 1.037158 | AMR | Wegener's granu | circulatory system |
| 446.5 | 0.43134 | 0.225355 | 1.914045 | 0.055614 | -0.01035 | 0.873027 | AMR | Giant cell arteritis | circulatory system |
| 446.6 | 0.313493 | 0.328891 | 0.953183 | 0.340497 | -0.33112 | 0.958107 | AMR | Polyarteritis nodo | circulatory system |
| 446.7 | 0.448825 | 0.580576 | 0.773069 | 0.439482 | -0.68908 | 1.586732 | AMR | Takayasu's disea | circulatory system |
| 446.8 | 0.558316 | 0.247697 | 2.254026 | 0.024195 | 0.072838 | 1.043795 | AMR | Thrombotic micro | circulatory system |
| 446.9 | 0.028542 | 0.154831 | 0.18434 | 0.853747 | -0.27492 | 0.332006 | AMR | Arteritis NOS | circulatory system |
| 447 | -0.02683 | 0.06364 | -0.42158 | 0.673334 | -0.15156 | 0.097903 | AMR | Other disorders c | circulatory system |
| 447.1 | -0.19311 | 0.115049 | -1.67852 | 0.093245 | -0.41861 | 0.032379 | AMR | Stricture of artery | circulatory system |
| 447.7 | -0.02157 | 1.27E-01 | -0.16978 | 0.865183 | -0.27053 | 0.227399 | AMR | Aortic ectasia | circulatory system |
| 448 | 0.08716 | 0.173307 | 0.502926 | 0.615016 | -0.25251 | 0.426835 | AMR | Disease of capilla | circulatory system |
| 450 | 0.049145 | 1.21E-01 | 0.407468 | 0.683664 | -0.18725 | 0.285537 | AMR | Noninfectious dis | circulatory system |
| 451 | 0.078891 | 0.101733 | 7.75E-01 | 0.438063 | -0.1205 | 0.278284 | AMR | Phlebitis and thro | circulatory system |
| 451.2 | 0.025474 | 0.169151 | 0.1506 | 0.880291 | -0.30606 | 0.357004 | AMR | Phlebitis and thro | circulatory system |
| 452 | 0.073409 | 0.045303 | 1.620399 | 0.105147 | -0.01538 | 0.162202 | AMR | Other venous em | circulatory system |
| 452.1 | 0.871959 | 0.582467 | 1.49701 | 0.134391 | -0.26966 | 2.013573 | AMR | Iatrogenic pulmo | circulatory system |

| | | | | | | | | | | |
|---|---|---|---|---|---|---|---|---|---|---|
| 452.2 | 0.070932 | 0.055324 | 1.28211 | 0.199804 | -0.0375 | 0.179365 | AMR | Deep vein thromb | circulatory system | |
| 452.8 | 0.133871 | 0.212407 | 0.630259 | 0.528525 | -0.28244 | 0.550181 | AMR | Postphlebitic syn | circulatory system | |
| 453 | 0.046822 | 0.158832 | 0.294789 | 0.768155 | -0.26448 | 0.358126 | AMR | Chronic venous h | circulatory system | |
| 454 | 0.062701 | 0.057925 | 1.082443 | 0.279056 | -0.05083 | 0.176232 | AMR | Varicose veins | circulatory system | |
| 454.1 | 0.123218 | 7.18E-02 | 1.716648 | 0.086043 | -0.01746 | 0.263901 | AMR | Varicose veins of | circulatory system | |
| 454.11 | 0.103379 | 0.084046 | 1.230021 | 0.218689 | -0.06135 | 0.268107 | AMR | Varicose veins of | circulatory system | |
| 455 | -0.01668 | 0.038462 | -0.43363 | 0.66456 | -0.09206 | 0.058706 | AMR | Hemorrhoids | circulatory system | |
| 456 | 0.100785 | 0.060433 | 1.67E+00 | 0.095372 | -0.01766 | 0.219232 | AMR | Chronic venous i | circulatory system | |
| 457 | 0.051909 | 0.039573 | 1.311727 | 0.189612 | -0.02565 | 0.12947 | AMR | Encounter for lon | circulatory system | |
| 457.2 | 0.069279 | 0.077342 | 0.895749 | 0.370387 | -0.08231 | 0.220866 | AMR | Encounter for lon | circulatory system | |
| 457.3 | 0.053122 | 0.039944 | 1.329908 | 0.183549 | -0.02517 | 0.13141 | AMR | Encounter for lon | circulatory system | |
| 458 | 0.040735 | 0.042254 | 0.964052 | 0.33502 | -0.04208 | 0.12355 | AMR | Hypotension | circulatory system | |
| 458.1 | 0.108517 | 0.077677 | 1.397041 | 0.162401 | -0.04373 | 0.260761 | AMR | Orthostatic hypot | circulatory system | |
| 458.2 | -0.00536 | 0.081005 | -0.06616 | 0.94725 | -0.16413 | 0.153407 | AMR | Iatrogenic hypote | circulatory system | |
| 458.9 | 0.025139 | 0.046439 | 0.541332 | 0.588279 | -0.06588 | 0.116157 | AMR | Hypotension NOS | circulatory system | |
| 459 | 0.006842 | 3.99E-02 | 0.171449 | 0.86387 | -0.07137 | 0.085058 | AMR | Other disorders c | circulatory system | |
| 459.1 | 0.056538 | 0.086027 | 0.657217 | 0.511041 | -0.11207 | 0.225149 | AMR | Hemorrhage NOS | circulatory system | |
| 459.7 | 0.031894 | 6.18E-02 | 0.516333 | 0.605622 | -0.08917 | 0.152962 | AMR | Blood vessel repl | circulatory system | |
| 459.9 | 0.01785 | 0.04655 | 3.83E-01 | 0.701373 | -0.07339 | 0.109087 | AMR | Circulatory disea | circulatory system | |
| 464 | -0.03195 | 0.048543 | -0.65825 | 0.510379 | -0.1271 | 0.06319 | AMR | Acute sinusitis | respiratory | |
| 465 | 0.021119 | 0.033925 | 0.622521 | 0.5336 | -0.04537 | 0.087612 | AMR | Acute upper resp | respiratory | |
| 465.2 | 0.008242 | 0.043281 | 0.190425 | 0.848976 | -0.07659 | 0.09307 | AMR | Acute pharyngitis | respiratory | |
| 465.4 | 0.255134 | 0.127213 | 2.005571 | 0.044902 | 0.005802 | 0.504466 | AMR | Acute laryngitis a | respiratory | |
| 470 | -0.00524 | 0.069837 | -0.07503 | 0.940193 | -0.14212 | 0.131638 | AMR | Septal Deviations | respiratory | |
| 471 | -0.04002 | 0.134585 | -0.29737 | 0.766188 | -0.3038 | 0.223761 | AMR | Nasal polyps | respiratory | |
| 472 | 0.00662 | 0.064511 | 0.102623 | 0.918262 | -0.11982 | 0.133059 | AMR | Chronic pharyngi | respiratory | |
| 473 | -0.02623 | 0.058979 | -0.44479 | 0.656471 | -0.14183 | 0.089364 | AMR | Diseases of the l | respiratory | |
| 473.1 | 0.030226 | 0.257232 | 0.117505 | 0.90646 | -0.47394 | 0.534391 | AMR | Chronic laryngitis | respiratory | |
| 473.3 | 0.046603 | 0.11274 | 0.41337 | 0.679335 | -0.17436 | 0.26757 | AMR | Paralysis/spasm | respiratory | |
| 473.4 | -0.01926 | 0.068178 | -2.83E-01 | 0.777529 | -0.15289 | 0.114364 | AMR | Voice disturbanc | respiratory | |
| 474 | 0.039295 | 0.098265 | 0.399885 | 0.689241 | -0.1533 | 0.23189 | AMR | Acute and chroni | respiratory | |
| 474.1 | -0.14119 | 0.162244 | -0.87023 | 0.384175 | -0.45918 | 0.176803 | AMR | Acute tonsillitis | respiratory | |
| 474.2 | 0.060446 | 0.109674 | 0.551144 | 0.581535 | -0.15451 | 0.275403 | AMR | Chronic tonsillitis | respiratory | |
| 475 | -0.00514 | 0.045001 | -0.11425 | 0.90904 | -0.09334 | 0.08306 | AMR | Chronic sinusitis | respiratory | |
| 475.9 | -0.02437 | 0.061819 | -0.39425 | 0.693397 | -0.14553 | 0.09679 | AMR | Postnasal drip | respiratory | |
| 476 | 0.03197 | 0.039486 | 0.809651 | 0.418141 | -0.04542 | 0.10936 | AMR | Allergic rhinitis | respiratory | |
| 477 | -0.02436 | 0.067579 | -0.36051 | 0.718468 | -0.15682 | 0.10809 | AMR | Epistaxis or throa | respiratory | |
| 478 | 0.032649 | 0.088003 | 0.371006 | 0.710633 | -0.13983 | 0.205131 | AMR | Throat pain | respiratory | |

| | | | | | | | | | | | |
|---|---|---|---|---|---|---|---|---|---|---|---|
| 479 | 0.005737 | 0.038279 | 0.149879 | 0.88086 | -0.06929 | 0.080762 | AMR | | Other upper resp | respiratory | |
| 480 | 0.047759 | 0.037348 | 1.278768 | 0.200979 | -0.02544 | 0.12096 | AMR | | Pneumonia | respiratory | |
| 480.1 | 0.07038 | 0.058485 | 1.20E+00 | 0.22883 | -0.04425 | 0.185008 | AMR | | Bacterial pneumo | respiratory | |
| 480.11 | 0.005881 | 0.086939 | 0.067649 | 0.946065 | -0.16452 | 0.176278 | AMR | | Pneumococcal p | respiratory | |
| 480.12 | -0.03508 | 0.158044 | -0.22198 | 0.824331 | -0.34484 | 0.274679 | AMR | | Pseudomonal pn | respiratory | |
| 480.13 | 0.070607 | 0.155544 | 0.453939 | 0.649872 | -0.23425 | 0.375467 | AMR | | MRSA pneumoni | respiratory | |
| 480.2 | 0.101539 | 0.056267 | 1.804594 | 0.071138 | -0.00874 | 0.211821 | AMR | | Viral pneumonia | respiratory | |
| 480.3 | -0.03106 | 0.13052 | -0.23796 | 0.811912 | -0.28687 | 0.224755 | AMR | | Pneumonia due t | respiratory | |
| 480.5 | 0.144456 | 0.120196 | 1.201835 | 0.229427 | -0.09112 | 0.380036 | AMR | | Bronchopneumo | respiratory | |
| 481 | 0.028021 | 0.066458 | 0.421631 | 0.673294 | -0.10223 | 0.158277 | AMR | | Influenza | respiratory | |
| 483 | 0.072606 | 0.061225 | 1.1859 | 0.235662 | -0.04739 | 0.192604 | AMR | | Acute bronchitis | respiratory | |
| 495 | 0.001918 | 0.039344 | 0.048742 | 0.961125 | -0.07519 | 0.07903 | AMR | | Asthma | respiratory | |
| 495.1 | -0.14263 | 0.149725 | -0.95262 | 0.340783 | -0.43609 | 0.150825 | AMR | | Chronic obstructi | respiratory | |
| 495.11 | -0.40976 | 0.294911 | -1.39E+00 | 0.164706 | -0.98777 | 0.168261 | AMR | | Chronic obstructi | respiratory | |
| 495.2 | 0.037798 | 0.065528 | 0.576827 | 0.564057 | -0.09063 | 0.16623 | AMR | | Asthma with exa | respiratory | |
| 496 | -0.03793 | 0.051435 | -0.73749 | 0.460822 | -0.13874 | 0.062878 | AMR | | Chronic airway o | respiratory | |
| 496.1 | -0.09395 | 0.08904 | -1.05517 | 0.291349 | -0.26847 | 0.080564 | AMR | | Emphysema | respiratory | |
| 496.2 | 0.029915 | 0.08966 | 0.333654 | 0.738641 | -0.14581 | 0.205645 | AMR | | Chronic bronchiti | respiratory | |
| 496.21 | 0.088181 | 0.125213 | 0.70425 | 0.481277 | -0.15723 | 0.333594 | AMR | | Obstructive chro | respiratory | |
| 496.3 | 0.022346 | 0.073411 | 0.304399 | 0.760824 | -0.12154 | 0.166229 | AMR | | Bronchiectasis | respiratory | |
| 497 | -0.09972 | 0.06554 | -1.52158 | 0.128115 | -0.22818 | 0.028732 | AMR | | Bronchitis | respiratory | |
| 498 | -0.00714 | 0.133256 | -0.0536 | 0.957257 | -0.26832 | 0.254035 | AMR | | Acute bronchosp | respiratory | |
| 499 | 0.159297 | 0.316318 | 0.503598 | 0.614544 | -0.46067 | 0.779269 | AMR | | Cystic fibrosis | respiratory | |
| 500 | 0.214669 | 0.130081 | 1.650275 | 0.098887 | -0.04028 | 0.469623 | AMR | | Lung disease du | respiratory | |
| 500.1 | 0.198791 | 0.214133 | 9.28E-01 | 0.353225 | -0.2209 | 0.618483 | AMR | | Extrinsic allergic | respiratory | |
| 500.2 | 0.521379 | 0.45105 | 1.155923 | 0.247713 | -0.36266 | 1.40542 | AMR | | Pneumoconiosis | respiratory | |
| 501 | 0.079783 | 0.076063 | 1.0489 | 0.294224 | -0.0693 | 0.228864 | AMR | | Pneumonitis due | respiratory | |
| 502 | 0.04289 | 0.063566 | 0.674728 | 0.499849 | -0.0817 | 0.167478 | AMR | | Postinflammatory | respiratory | |
| 503 | 0.05944 | 0.046828 | 1.269313 | 0.204329 | -0.03234 | 0.151221 | AMR | | Pulmonary conge | respiratory | |
| 504 | 0.086962 | 0.080639 | 1.08E+00 | 0.28085 | -0.07109 | 0.245011 | AMR | | Other alveolar ar | respiratory | |
| 504.1 | 0.047846 | 0.107939 | 0.44327 | 0.65757 | -0.16371 | 0.259403 | AMR | | Idiopathic fibrosir | respiratory | |
| 505 | 0.048301 | 0.104231 | 0.463406 | 0.643074 | -0.15599 | 0.252591 | AMR | | Other pulmonary | respiratory | |
| 506 | 0.006222 | 0.060964 | 0.102061 | 0.918709 | -0.11327 | 0.125709 | AMR | | Empyema and pr | respiratory | |
| 507 | 0.081102 | 0.038769 | 2.091922 | 0.036445 | 0.005116 | 0.157088 | AMR | | Pleurisy; pleural | respiratory | |
| 508 | 0.098849 | 3.54E-02 | 2.792718 | 0.005227 | 0.029475 | 0.168222 | AMR | | Pulmonary collap | respiratory | |
| 509 | 0.083078 | 0.040118 | 2.07E+00 | 0.038373 | 0.004448 | 0.161707 | AMR | | Respiratory failur | respiratory | |
| 509.1 | 0.083093 | 0.041135 | 2.019982 | 0.043385 | 0.002469 | 0.163716 | AMR | | Respiratory failur | respiratory | |
| 509.2 | 0.178283 | 0.08574 | 2.079348 | 0.037585 | 0.010236 | 0.34633 | AMR | | Respiratory insuf | respiratory | |

| | | | | | | | | | | |
|---|---|---|---|---|---|---|---|---|---|---|
| 509.3 | 0.145489 | 0.09478 | 1.535017 | 0.12478 | -0.04028 | 0.331255 | AMR | Pulmonary insuff | respiratory | |
| 509.5 | 0.355705 | 0.305116 | 1.165802 | 0.243695 | -0.24231 | 0.953721 | AMR | Respiratory arres | respiratory | |
| 509.8 | 0.124243 | 0.068384 | 1.816831 | 0.069243 | -0.00979 | 0.258274 | AMR | Dependence on r | respiratory | |
| 510 | 0.05027 | 0.044907 | 1.119425 | 0.262959 | -0.03775 | 0.138287 | AMR | Other diseases o | respiratory | |
| 510.2 | 0.03318 | 0.096198 | 0.344916 | 0.730158 | -0.15536 | 0.221725 | AMR | Lung transplant | respiratory | |
| 512 | 0.017381 | 3.15E-02 | 0.551725 | 0.581137 | -0.04436 | 0.079128 | AMR | Other symptoms | respiratory | |
| 512.1 | 0.033614 | 0.067884 | 0.49517 | 0.62048 | -0.09944 | 0.166665 | AMR | Wheezing | respiratory | |
| 512.2 | 0.03953 | 0.052029 | 0.759767 | 0.447394 | -0.06245 | 0.141506 | AMR | Painful respiratio | respiratory | |
| 512.3 | 0.029027 | 0.183538 | 1.58E-01 | 0.874337 | -0.3307 | 0.388754 | AMR | Abnormal chest s | respiratory | |
| 512.7 | 0.031892 | 0.032623 | 0.977597 | 0.328274 | -0.03205 | 0.095833 | AMR | Shortness of brea | respiratory | |
| 512.8 | -0.00185 | 0.032721 | -0.05652 | 0.954928 | -0.06598 | 0.062283 | AMR | Cough | respiratory | |
| 512.9 | -0.0021 | 0.034078 | -0.06173 | 0.950776 | -0.0689 | 0.064688 | AMR | Other dyspnea | respiratory | |
| 513 | -0.03989 | 0.0502 | -0.79467 | 0.426803 | -0.13828 | 0.058498 | AMR | Respiratory abnc | respiratory | |
| 513.3 | -0.07653 | 0.082927 | -9.23E-01 | 0.356114 | -0.23906 | 0.08601 | AMR | Hypoventilation | respiratory | |
| 513.31 | 0.092537 | 0.125471 | 0.737515 | 0.460809 | -0.15338 | 0.338455 | AMR | Apnea | respiratory | |
| 513.32 | -0.32968 | 0.150089 | -2.19659 | 0.028049 | -0.62385 | -0.03552 | AMR | Orthopnea | respiratory | |
| 513.4 | -0.19977 | 1.47E-01 | -1.35743 | 0.174644 | -0.48822 | 0.088674 | AMR | Hyperventilation | respiratory | |
| 513.8 | 0.012786 | 0.062524 | 0.2045 | 0.837963 | -0.10976 | 0.135331 | AMR | Disorders of diap | respiratory | |
| 514 | 0.046435 | 0.033532 | 1.384807 | 0.166111 | -0.01929 | 0.112156 | AMR | Abnormal finding | respiratory | |
| 514.1 | -0.06908 | 0.11826 | -5.84E-01 | 0.55915 | -0.30086 | 0.16271 | AMR | Abnormal results | respiratory | |
| 514.2 | -0.07261 | 0.049591 | -1.46412 | 0.143161 | -0.1698 | 0.024589 | AMR | Solitary pulmona | respiratory | |
| 516 | 0.116528 | 0.092789 | 1.25584 | 0.209174 | -0.06533 | 0.298391 | AMR | Abnormal sputun | respiratory | |
| 516.1 | 0.161825 | 0.09898 | 1.634916 | 0.102067 | -0.03217 | 0.355822 | AMR | Hemoptysis | respiratory | |
| 519 | 0.015189 | 0.043595 | 0.348421 | 0.727524 | -0.07025 | 0.100633 | AMR | Other diseases o | respiratory | |
| 519.1 | -0.33565 | 0.207779 | -1.61543 | 0.106217 | -0.74289 | 0.071586 | AMR | Tracheostomy cc | respiratory | |
| 519.2 | 0.152741 | 0.122929 | 1.242514 | 0.214047 | -0.0882 | 0.393676 | AMR | Respiratory comp | respiratory | |
| 519.8 | 0.012603 | 0.055908 | 0.225419 | 0.821654 | -0.09697 | 0.12218 | AMR | Other diseases o | respiratory | |
| 519.9 | -0.03342 | 0.062718 | -0.53284 | 0.594144 | -0.15634 | 0.089506 | AMR | Symptoms involv | respiratory | |
| 520 | 0.315763 | 2.03E-01 | 1.558529 | 0.119108 | -0.08133 | 0.712857 | AMR | Disorders of tootl | digestive | |
| 520.1 | 0.062946 | 0.426012 | 0.147757 | 0.882535 | -0.77202 | 0.897915 | AMR | Hereditary disturl | digestive | |
| 520.2 | 0.3666 | 0.2719 | 1.35E+00 | 0.177565 | -0.16631 | 0.899513 | AMR | Disturbances in t | digestive | |
| 521 | 0.251052 | 0.094121 | 2.667344 | 0.007645 | 0.066579 | 0.435525 | AMR | Diseases of hard | digestive | |
| 521.1 | 0.290273 | 0.095761 | 3.031222 | 0.002436 | 0.102585 | 0.477961 | AMR | Dental caries | digestive | |
| 521.2 | 0.133038 | 1.056234 | 0.125955 | 0.899767 | -1.93714 | 2.203218 | AMR | Dental abrasion, | digestive | |
| 522 | 0.140434 | 0.127822 | 1.09867 | 0.271912 | -0.11009 | 0.390961 | AMR | Diseases of pulp | digestive | |
| 522.5 | 0.14148 | 0.129971 | 1.088551 | 0.276352 | -0.11326 | 0.396218 | AMR | Periapical absce: | digestive | |
| 523 | 0.151109 | 0.079922 | 1.890699 | 0.058665 | -0.00554 | 0.307754 | AMR | Gingival and peri | digestive | |
| 523.1 | 0.145176 | 0.10867 | 1.335936 | 0.18157 | -0.06781 | 0.358166 | AMR | Gingivitis | digestive | |

| | | | | | | | | | | | |
|---|---|---|---|---|---|---|---|---|---|---|---|
| 523.3 | 0.176733 | 1.17E-01 | 1.50958 | 0.131151 | -0.05273 | 0.406193 | AMR | | Periodontitis (acu | digestive | |
| 523.31 | 0.201059 | 0.119389 | 1.684065 | 0.092169 | -0.03294 | 0.435056 | AMR | | Acute periodontit | digestive | |
| 523.32 | 0.503224 | 0.574621 | 0.875749 | 0.381166 | -0.62301 | 1.629462 | AMR | | Chronic periodon | digestive | |
| 524 | 0.322457 | 0.225497 | 1.43E+00 | 0.152722 | -0.11951 | 0.764423 | AMR | | Dentofacial anom | digestive | |
| 524.3 | 0.491367 | 0.26078 | 1.884219 | 0.059535 | -0.01975 | 1.002488 | AMR | | Anomalies of too | digestive | |
| 525 | 0.129808 | 0.097596 | 1.330063 | 0.183497 | -0.06148 | 0.321092 | AMR | | Other diseases o | digestive | |
| 525.1 | 0.545759 | 0.220003 | 2.480692 | 0.013113 | 0.114562 | 0.976956 | AMR | | Loss of teeth or e | digestive | |
| 526 | 0.020206 | 0.070961 | 0.284746 | 0.775839 | -0.11887 | 0.159286 | AMR | | Diseases of the ja | digestive | |
| 526.1 | 0.398999 | 0.311282 | 1.28179 | 0.199916 | -0.2111 | 1.009101 | AMR | | Cysts of the jaws | digestive | |
| 526.3 | 0.277117 | 0.279889 | 0.990095 | 0.322128 | -0.27146 | 0.82569 | AMR | | Anomalies of jaw | digestive | |
| 526.4 | -0.00966 | 0.091431 | -0.10562 | 0.915881 | -0.18886 | 0.169545 | AMR | | Temporomandibu | digestive | |
| 526.41 | 0.040915 | 1.18E-01 | 0.347053 | 0.728551 | -0.19015 | 0.271978 | AMR | | Temporomandibu | digestive | |
| 526.42 | -0.10981 | 0.146525 | -0.74944 | 0.453595 | -0.397 | 0.177373 | AMR | | Arthralgia/ankylo | digestive | |
| 526.5 | 0.533253 | 0.240611 | 2.216247 | 0.026675 | 0.061664 | 1.004842 | AMR | | Inflammatory cor | digestive | |
| 526.8 | 0.88956 | 0.490535 | 1.81E+00 | 0.069762 | -0.07187 | 1.850991 | AMR | | Exostosis of jaw | digestive | |
| 526.9 | -0.55254 | 0.248647 | -2.22E+00 | 0.026271 | -1.03988 | -0.0652 | AMR | | Jaw disease NOS | digestive | |
| 527 | 0.003128 | 0.075306 | 0.041534 | 0.96687 | -0.14447 | 0.150725 | AMR | | Diseases of the s | digestive | |
| 527.1 | 0.448439 | 0.34978 | 1.282059 | 0.199822 | -0.23712 | 1.133996 | AMR | | Hypertrophy of sa | digestive | |
| 527.2 | 0.070351 | 0.159583 | 0.440842 | 0.659327 | -0.24243 | 0.383128 | AMR | | Sialoadenitis | digestive | |
| 527.7 | 0.008722 | 0.09402 | 0.092772 | 0.926084 | -0.17555 | 0.192998 | AMR | | Disturbance of sa | digestive | |
| 527.8 | -0.03714 | 0.15647 | -0.23734 | 0.812393 | -0.34381 | 0.269539 | AMR | | Other specified d | digestive | |
| 528 | 0.012008 | 0.057654 | 0.208269 | 0.835019 | -0.10099 | 0.125007 | AMR | | Diseases of the o | digestive | |
| 528.1 | -0.02334 | 0.084176 | -0.27726 | 0.781581 | -0.18832 | 0.141644 | AMR | | Stomatitis and m | digestive | |
| 528.11 | -0.01597 | 0.119567 | -0.13358 | 0.893733 | -0.25032 | 0.218376 | AMR | | Stomatitis and m | digestive | |
| 528.12 | 0.067176 | 0.143316 | 0.468727 | 0.639265 | -0.21372 | 0.348071 | AMR | | Oral aphthae | digestive | |
| 528.3 | 0.845186 | 0.227759 | 3.71E+00 | 0.000207 | 0.398786 | 1.291587 | AMR | | Cellulitis and abs | digestive | |
| 528.4 | 0.187283 | 0.38941 | 0.480942 | 0.630558 | -0.57595 | 0.950513 | AMR | | Cysts of oral soft | digestive | |
| 528.41 | 0.270873 | 0.418223 | 0.647676 | 0.517194 | -0.54883 | 1.090575 | AMR | | Cyst of the saliva | digestive | |
| 528.5 | 0.189907 | 0.127416 | 1.490445 | 0.136107 | -0.05982 | 0.439637 | AMR | | Diseases of lips | digestive | |
| 528.6 | 0.507335 | 0.33126 | 1.531533 | 0.125638 | -0.14192 | 1.156592 | AMR | | Leukoplakia of or | digestive | |
| 528.7 | 0.460386 | 0.291163 | 1.581199 | 0.113833 | -0.11028 | 1.031054 | AMR | | Sialolithiasis | digestive | |
| 529 | -0.11674 | 0.102002 | -1.14447 | 0.252427 | -0.31666 | 0.083182 | AMR | | Diseases and oth | digestive | |
| 529.1 | -0.20732 | 0.214923 | -0.96461 | 0.334741 | -0.62856 | 0.213925 | AMR | | Glossitis | digestive | |
| 529.6 | 0.209051 | 0.186564 | 1.120535 | 0.262486 | -0.15661 | 0.574709 | AMR | | Glossodynia | digestive | |
| 530 | 0.01681 | 0.031694 | 0.530376 | 0.595851 | -0.04531 | 0.078929 | AMR | | Diseases of esop | digestive | |
| 530.1 | 0.013107 | 0.031654 | 0.414066 | 0.678826 | -0.04893 | 0.075148 | AMR | | Esophagitis, GEF | digestive | |
| 530.11 | 0.00474 | 0.031733 | 1.49E-01 | 0.881271 | -0.05746 | 0.066934 | AMR | | GERD | digestive | |
| 530.12 | -0.13844 | 0.104385 | -1.32625 | 0.184757 | -0.34303 | 0.06615 | AMR | | Ulcer of esophag | digestive | |

| | | | | | | | | | | |
|---|---|---|---|---|---|---|---|---|---|---|
| 530.13 | -0.04104 | 0.132654 | -0.30935 | 0.757059 | -0.30103 | 0.218962 | AMR | Barrett's esophag | digestive | |
| 530.14 | 0.017863 | 0.066325 | 0.269323 | 0.787681 | -0.11213 | 0.147858 | AMR | Reflux esophagit | digestive | |
| 530.15 | 0.261229 | 0.247081 | 1.05726 | 0.290393 | -0.22304 | 0.745499 | AMR | Eosinophilic esop | digestive | |
| 530.2 | 0.114315 | 0.056613 | 2.019227 | 0.043464 | 0.003355 | 0.225274 | AMR | Esophageal blee | digestive | |
| 530.3 | 0.007216 | 0.124871 | 0.057788 | 0.953917 | -0.23753 | 0.251958 | AMR | Stricture and ster | digestive | |
| 530.5 | 0.127129 | 0.094382 | 1.346961 | 0.177993 | -0.05786 | 0.312114 | AMR | Disorders of esop | digestive | |
| 530.6 | 0.52578 | 2.70E-01 | 1.945698 | 0.051691 | -0.00385 | 1.055415 | AMR | Diverticulum of e | digestive | |
| 530.7 | 0.33043 | 0.241487 | 1.368311 | 0.171215 | -0.14288 | 0.803736 | AMR | Gastroesophagea | digestive | |
| 530.9 | 0.004882 | 0.090477 | 0.053955 | 0.956971 | -0.17245 | 0.182214 | AMR | Heartburn | digestive | |
| 531 | -0.07191 | 0.05806 | -1.24E+00 | 0.215526 | -0.1857 | 0.041887 | AMR | Peptic ulcer (excl | digestive | |
| 531.1 | -0.15608 | 0.130834 | -1.19293 | 0.232898 | -0.41251 | 0.100355 | AMR | Hemorrhage fron | digestive | |
| 531.2 | -0.01703 | 0.083456 | -0.20402 | 0.83834 | -0.1806 | 0.146545 | AMR | Gastric ulcer | digestive | |
| 531.3 | 0.036401 | 0.145984 | 0.249351 | 0.803089 | -0.24972 | 0.322525 | AMR | Duodenal ulcer | digestive | |
| 531.4 | -0.03041 | 0.074524 | -0.40809 | 0.683205 | -0.17648 | 0.115652 | AMR | Peptic ulcer, site | digestive | |
| 531.5 | 0.102606 | 0.220531 | 0.46527 | 0.641738 | -0.32963 | 0.534839 | AMR | Gastrojejunal ulc | digestive | |
| 532 | 0.055454 | 0.043284 | 1.281162 | 0.200137 | -0.02938 | 0.140289 | AMR | Dysphagia | digestive | |
| 535 | 0.00985 | 4.27E-02 | 0.230782 | 0.817484 | -0.0738 | 0.093503 | AMR | Gastritis and duo | digestive | |
| 535.1 | -0.01269 | 1.13E-01 | -0.11213 | 0.91072 | -0.23452 | 0.209142 | AMR | Acute gastritis | digestive | |
| 535.2 | 0.023722 | 0.053527 | 0.443182 | 0.657634 | -0.08119 | 0.128632 | AMR | Atrophic gastritis | digestive | |
| 535.6 | 0.069684 | 1.28E-01 | 0.545649 | 0.585307 | -0.18062 | 0.319988 | AMR | Duodenitis | digestive | |
| 535.8 | 0.025008 | 0.085613 | 2.92E-01 | 0.770202 | -0.14279 | 0.192807 | AMR | Other specified g | digestive | |
| 535.9 | -0.04492 | 0.058592 | -0.76668 | 0.443269 | -0.15976 | 0.069917 | AMR | Gastritis and duo | digestive | |
| 536 | -0.03465 | 0.04968 | -0.6975 | 0.485488 | -0.13202 | 0.062719 | AMR | Disorders of func | digestive | |
| 536.3 | -0.15287 | 0.084982 | -1.79887 | 0.072039 | -0.31944 | 0.01369 | AMR | Gastroparesis | digestive | |
| 536.7 | 0.035755 | 0.132124 | 0.270616 | 0.786687 | -0.2232 | 0.294714 | AMR | Complications of | digestive | |
| 536.8 | 0.000168 | 0.057984 | 0.002903 | 0.997684 | -0.11348 | 0.113814 | AMR | Dyspepsia and o | digestive | |
| 537 | 0.038211 | 0.043418 | 0.880065 | 0.378824 | -0.04689 | 0.123309 | AMR | Other disorders c | digestive | |
| 537.1 | 0.033095 | 0.156739 | 0.211145 | 0.832774 | -0.27411 | 0.340298 | AMR | Lesions of stoma | digestive | |
| 539 | 0.122421 | 6.79E-02 | 1.801802 | 0.071577 | -0.01075 | 0.255589 | AMR | Bariatric surgery | digestive | |
| 540 | -0.01102 | 0.103601 | -0.10641 | 0.915257 | -0.21408 | 0.19203 | AMR | Appendiceal con | digestive | |
| 540.1 | 0.040244 | 0.108756 | 0.370042 | 0.711351 | -0.17291 | 0.253401 | AMR | Appendicitis | digestive | |
| 540.11 | -0.06552 | 0.170937 | -3.83E-01 | 0.701479 | -0.40056 | 0.269506 | AMR | Acute appendiciti | digestive | |
| 550 | -0.04652 | 0.039564 | -1.17591 | 0.23963 | -0.12407 | 0.03102 | AMR | Abdominal hernia | digestive | |
| 550.1 | -0.14321 | 0.079411 | -1.80339 | 0.071327 | -0.29885 | 0.012434 | AMR | Inguinal hernia | digestive | |
| 550.2 | -0.01734 | 0.051119 | -0.33919 | 0.73447 | -0.11753 | 0.082853 | AMR | Diaphragmatic he | digestive | |
| 550.3 | 0.617445 | 0.291654 | 2.117044 | 0.034256 | 0.045813 | 1.189076 | AMR | Femoral hernia | digestive | |
| 550.4 | -0.01972 | 0.076951 | -0.25623 | 0.797774 | -0.17054 | 0.131104 | AMR | Umbilical hernia | digestive | |
| 550.5 | 0.058706 | 0.067405 | 0.870944 | 0.383784 | -0.07341 | 0.190817 | AMR | Ventral hernia | digestive | |

| | | | | | | | | | | |
|---|---|---|---|---|---|---|---|---|---|---|
| 550.6 | -0.04576 | 0.107424 | -0.42598 | 0.670124 | -0.25631 | 0.164787 | AMR | Incisional hernia | digestive | |
| 555 | -0.02728 | 0.093421 | -0.292 | 0.770283 | -0.21038 | 0.155823 | AMR | Inflammatory bov | digestive | |
| 555.1 | -0.09376 | 0.142662 | -0.65724 | 0.511025 | -0.37338 | 0.185848 | AMR | Regional enteritis | digestive | |
| 555.2 | -0.05023 | 0.102611 | -0.48952 | 0.624471 | -0.25135 | 0.150884 | AMR | Ulcerative colitis | digestive | |
| 555.21 | -0.00746 | 0.130086 | -5.74E-02 | 0.95427 | -0.26242 | 0.247504 | AMR | Ulcerative colitis | digestive | |
| 556 | -0.01314 | 0.098206 | -0.13377 | 0.893581 | -0.20562 | 0.179342 | AMR | Ulceration of the | digestive | |
| 556.1 | -0.03153 | 0.104896 | -0.30059 | 0.763726 | -0.23712 | 0.174062 | AMR | Ulceration of inte | digestive | |
| 556.11 | -0.00255 | 0.155431 | -0.01642 | 0.986899 | -0.30719 | 0.302087 | AMR | Angiodysplasia o | digestive | |
| 557 | -0.05829 | 0.104584 | -0.55739 | 0.577262 | -0.26327 | 0.146687 | AMR | Intestinal malabs | digestive | |
| 557.1 | -0.2847 | 0.18141 | -1.56938 | 0.116559 | -0.64026 | 0.070855 | AMR | Celiac disease | digestive | |
| 558 | -0.05808 | 0.05043 | -1.15162 | 0.249476 | -0.15692 | 0.040765 | AMR | Noninfectious ga | digestive | |
| 559 | 0.006282 | 0.134292 | 0.046777 | 0.962691 | -0.25693 | 0.269489 | AMR | Ileostomy status | digestive | |
| 560 | -0.03726 | 0.0521 | -0.71513 | 0.474529 | -0.13937 | 0.064856 | AMR | Intestinal obstruc | digestive | |
| 560.1 | -0.00299 | 0.067196 | -0.04455 | 0.964466 | -0.1347 | 0.128709 | AMR | Paralytic ileus | digestive | |
| 560.2 | 0.037119 | 0.106784 | 0.347612 | 0.728132 | -0.17217 | 0.246412 | AMR | Impaction of intes | digestive | |
| 560.3 | -0.13223 | 0.227588 | -5.81E-01 | 0.561233 | -0.5783 | 0.313833 | AMR | Peritoneal or inte | digestive | |
| 560.4 | -0.12961 | 0.079947 | -1.62121 | 0.104972 | -0.28631 | 0.027082 | AMR | Other intestinal o | digestive | |
| 561 | 0.004209 | 0.03182 | 0.13228 | 0.894763 | -0.05816 | 0.066576 | AMR | Symptoms involv | digestive | |
| 561.1 | -0.00603 | 0.035425 | -0.17024 | 0.864823 | -0.07546 | 0.0634 | AMR | Diarrhea | digestive | |
| 561.2 | 0.046627 | 0.040233 | 1.15891 | 0.246493 | -0.03223 | 0.125482 | AMR | Flatulence | digestive | |
| 562 | 0.019507 | 0.044372 | 0.439631 | 0.660205 | -0.06746 | 0.106475 | AMR | Diverticulosis and | digestive | |
| 562.1 | 0.017315 | 0.045193 | 0.383129 | 0.701624 | -0.07126 | 0.105892 | AMR | Diverticulosis | digestive | |
| 562.2 | 0.127812 | 0.08313 | 1.537493 | 0.124173 | -0.03512 | 0.290745 | AMR | Diverticulitis | digestive | |
| 563 | 0.024595 | 3.50E-02 | 0.702621 | 0.482292 | -0.04401 | 0.093205 | AMR | Constipation | digestive | |
| 564 | 0.004352 | 0.036654 | 0.118738 | 0.905483 | -0.06749 | 0.076192 | AMR | Functional digest | digestive | |
| 564.1 | 0.012423 | 0.070021 | 0.177411 | 0.859186 | -0.12482 | 0.149662 | AMR | Irritable Bowel Sy | digestive | |
| 564.8 | -0.06311 | 0.060703 | -1.04E+00 | 0.298495 | -0.18209 | 0.055865 | AMR | Abnormal finding | digestive | |
| 564.9 | 0.007536 | 0.043967 | 0.171409 | 0.863902 | -0.07864 | 0.09371 | AMR | Personal history | digestive | |
| 565 | -0.03388 | 0.050512 | -0.67066 | 0.502436 | -0.13288 | 0.065125 | AMR | Anal and rectal c | digestive | |
| 565.1 | -0.13645 | 0.085806 | -1.59017 | 0.111795 | -0.30462 | 0.03173 | AMR | Anal and rectal p | digestive | |
| 567 | 0.066387 | 0.068576 | 0.968079 | 0.333005 | -0.06802 | 0.200793 | AMR | Peritonitis and re | digestive | |
| 568 | 0.039583 | 0.054548 | 0.725654 | 0.468051 | -0.06733 | 0.146495 | AMR | Other disorders c | digestive | |
| 568.1 | 0.031589 | 0.061701 | 0.511973 | 0.60867 | -0.08934 | 0.15252 | AMR | Peritoneal adhes | digestive | |
| 569 | 0.016914 | 0.039758 | 0.425415 | 0.670534 | -0.06101 | 0.094839 | AMR | Other disorders c | digestive | |
| 569.1 | 0.046036 | 0.110579 | 0.416315 | 0.67718 | -0.1707 | 0.262767 | AMR | Toxic gastroenter | digestive | |
| 569.2 | 0.044551 | 0.073149 | 0.609039 | 0.542498 | -0.09882 | 0.187921 | AMR | Gastrointestinal c | digestive | |
| 571 | 0.029121 | 0.035014 | 0.831702 | 0.405577 | -0.0395 | 0.097746 | AMR | Chronic liver dise | digestive | |
| 571.5 | 0.033781 | 0.035278 | 9.58E-01 | 0.338283 | -0.03536 | 0.102926 | AMR | Other chronic no | digestive | |

| | | | | | | | | | | |
|---|---|---|---|---|---|---|---|---|---|---|
| 571.51 | 0.018271 | 0.054215 | 0.337019 | 0.736103 | -0.08799 | 0.124531 | AMR | Cirrhosis of liver | digestive | |
| 571.6 | 0.130834 | 0.136711 | 0.957007 | 0.338564 | -0.13712 | 0.398783 | AMR | Primary biliary ci | digestive | |
| 571.8 | 0.042527 | 0.056223 | 0.756406 | 0.449406 | -0.06767 | 0.152722 | AMR | Liver abscess an | digestive | |
| 571.81 | 0.070461 | 0.060673 | 1.161335 | 0.245506 | -0.04845 | 0.189377 | AMR | Portal hypertensi | digestive | |
| 572 | 0.069404 | 0.047786 | 1.45E+00 | 0.14639 | -0.02425 | 0.163063 | AMR | Ascites (non mali | digestive | |
| 573 | 0.033719 | 0.039597 | 0.851546 | 0.394466 | -0.04389 | 0.111328 | AMR | Other disorders c | digestive | |
| 573.1 | 0.050986 | 0.119922 | 0.425163 | 0.670718 | -0.18406 | 0.28603 | AMR | Chronic passive c | digestive | |
| 573.2 | 0.100684 | 6.96E-02 | 1.446534 | 0.148027 | -0.03574 | 0.237105 | AMR | Liver replaced by | digestive | |
| 573.3 | -0.06765 | 0.053683 | -1.26016 | 0.20761 | -0.17287 | 0.037567 | AMR | Hepatomegaly | digestive | |
| 573.4 | 0.05391 | 0.082348 | 0.654657 | 0.512688 | -0.10749 | 0.21531 | AMR | Acute and subac | digestive | |
| 573.5 | 0.043715 | 0.071032 | 6.15E-01 | 0.53827 | -0.09551 | 0.182936 | AMR | Jaundice (not of | digestive | |
| 573.6 | 0.07249 | 0.049598 | 1.461543 | 0.143867 | -0.02472 | 0.169701 | AMR | Nonspecific eleva | digestive | |
| 573.7 | 0.004831 | 0.052926 | 0.091282 | 0.927269 | -0.0989 | 0.108564 | AMR | Abnormal results | digestive | |
| 573.9 | -0.009 | 0.045184 | -0.19923 | 0.842086 | -0.09756 | 0.079557 | AMR | Abnormal serum | digestive | |
| 574 | 0.066733 | 0.046694 | 1.429165 | 0.152957 | -0.02479 | 0.158252 | AMR | Cholelithiasis and | digestive | |
| 574.1 | 0.040299 | 0.05014 | 8.04E-01 | 0.421547 | -0.05797 | 0.138571 | AMR | Cholelithiasis | digestive | |
| 574.11 | 0.014922 | 0.137382 | 0.108617 | 0.913506 | -0.25434 | 0.284186 | AMR | Cholelithiasis wit | digestive | |
| 574.12 | 0.095783 | 0.104406 | 0.917402 | 0.358932 | -0.10885 | 0.300415 | AMR | Cholelithiasis wit | digestive | |
| 574.2 | -0.01376 | 0.08588 | -0.16018 | 0.872741 | -0.18208 | 0.154565 | AMR | Calculus of bile d | digestive | |
| 574.3 | 0.103813 | 0.092169 | 1.126338 | 0.260023 | -0.07683 | 0.284461 | AMR | Cholecystitis with | digestive | |
| 575 | 0.067062 | 0.046625 | 1.438324 | 0.150342 | -0.02432 | 0.158446 | AMR | Other biliary tract | digestive | |
| 575.1 | 0.086935 | 0.117086 | 7.42E-01 | 0.45779 | -0.14255 | 0.31642 | AMR | Cholangitis | digestive | |
| 575.2 | 0.028651 | 0.082732 | 3.46E-01 | 0.729108 | -0.1335 | 0.190802 | AMR | Obstruction of bil | digestive | |
| 575.6 | 0.039518 | 0.115255 | 0.342871 | 0.731695 | -0.18638 | 0.265414 | AMR | Cholesterolosis c | digestive | |
| 575.7 | 0.104416 | 0.074754 | 1.396796 | 0.162475 | -0.0421 | 0.250932 | AMR | Other disorders c | digestive | |
| 575.8 | 0.110934 | 0.072386 | 1.532524 | 0.125393 | -0.03094 | 0.252809 | AMR | Other disorders c | digestive | |
| 575.9 | -0.0497 | 0.096527 | -0.51492 | 0.60661 | -0.23889 | 0.139486 | AMR | Nonspecific abnc | digestive | |
| 577 | 0.036376 | 0.057416 | 0.633549 | 0.526375 | -0.07616 | 0.148908 | AMR | Diseases of panc | digestive | |
| 577.1 | 0.025505 | 0.089026 | 0.286487 | 0.774506 | -0.14898 | 0.199993 | AMR | Acute pancreatiti | digestive | |
| 577.2 | 0.228892 | 0.138657 | 1.650784 | 0.098783 | -0.04287 | 0.500654 | AMR | Chronic pancreat | digestive | |
| 577.3 | 0.064146 | 0.095583 | 0.671103 | 0.502155 | -0.12319 | 0.251485 | AMR | Cyst and pseudo | digestive | |
| 578 | -0.01118 | 0.041441 | -0.26972 | 0.787379 | -0.0924 | 0.070045 | AMR | Gastrointestinal h | digestive | |
| 578.1 | 0.018759 | 0.096536 | 1.94E-01 | 0.845923 | -0.17045 | 0.207966 | AMR | Hematemesis | digestive | |
| 578.2 | 0.018565 | 0.053674 | 3.46E-01 | 0.729423 | -0.08663 | 0.123765 | AMR | Blood in stool | digestive | |
| 578.8 | -0.04637 | 0.054769 | -8.47E-01 | 0.397165 | -0.15372 | 0.060973 | AMR | Hemorrhage of re | digestive | |
| 578.9 | 0.034806 | 0.062591 | 5.56E-01 | 0.578156 | -0.08787 | 0.157483 | AMR | Hemorrhage of g | digestive | |
| 579 | 0.01667 | 0.044566 | 0.374057 | 0.708362 | -0.07068 | 0.104018 | AMR | Other symptoms | digestive | |
| 579.2 | 0.012242 | 0.059861 | 2.05E-01 | 0.837957 | -0.10508 | 0.129567 | AMR | Splenomegaly | digestive | |

| | | | | | | | | | | |
|---|---|---|---|---|---|---|---|---|---|---|
| 579.8 | -0.03036 | 0.066238 | -0.4583 | 0.64674 | -0.16018 | 0.099467 | AMR | Nonspecific abnc | digestive | |
| 580 | 0.086927 | 0.049304 | 1.763081 | 0.077887 | -0.00971 | 0.183561 | AMR | Nephritis; nephrc | genitourinary | |
| 580.1 | 0.023822 | 0.115076 | 0.207008 | 0.836004 | -0.20172 | 0.249367 | AMR | Glomerulonephri | genitourinary | |
| 580.11 | 0.283223 | 0.198632 | 1.425866 | 0.153907 | -0.10609 | 0.672535 | AMR | Proliferative glom | genitourinary | |
| 580.12 | 0.071783 | 0.147614 | 0.486288 | 0.626763 | -0.21754 | 0.361101 | AMR | Non-proliferative | genitourinary | |
| 580.13 | 0.784803 | 0.304827 | 2.57E+00 | 0.010036 | 0.187353 | 1.382254 | AMR | Acute glomerulor | genitourinary | |
| 580.14 | 0.1712 | 0.26632 | 6.43E-01 | 0.520331 | -0.35078 | 0.693176 | AMR | Chronic glomerul | genitourinary | |
| 580.2 | 0.122659 | 0.085549 | 1.43E+00 | 0.151633 | -0.04501 | 0.290331 | AMR | Nephrotic syndro | genitourinary | |
| 580.3 | 0.093785 | 0.053079 | 1.77E+00 | 0.077245 | -0.01025 | 0.197818 | AMR | Nephritis and nep | genitourinary | |
| 580.31 | 0.111057 | 0.061893 | 1.794338 | 0.072759 | -0.01025 | 0.232366 | AMR | Nephritis and nep | genitourinary | |
| 580.32 | 0.132752 | 0.069428 | 1.91E+00 | 0.055867 | -0.00333 | 0.268829 | AMR | Nephritis and nep | genitourinary | |
| 580.4 | 0.008022 | 0.097452 | 0.082322 | 0.934391 | -0.18298 | 0.199025 | AMR | Renal sclerosis, I | genitourinary | |
| 585 | 0.058609 | 0.0357 | 1.641689 | 0.100654 | -0.01136 | 0.12858 | AMR | Renal failure | genitourinary | |
| 585.1 | 0.058637 | 0.038662 | 1.516666 | 0.129351 | -0.01714 | 0.134414 | AMR | Acute renal failur | genitourinary | |
| 585.2 | 0.056719 | 0.076054 | 0.74577 | 0.455806 | -0.09234 | 0.205783 | AMR | Renal failure NOS | genitourinary | |
| 585.3 | 0.057713 | 0.038878 | 1.48446 | 0.137687 | -0.01849 | 0.133912 | AMR | Chronic renal fail | genitourinary | |
| 585.31 | 0.045296 | 0.048911 | 9.26E-01 | 0.354396 | -0.05057 | 0.14116 | AMR | Renal dialysis | genitourinary | |
| 585.32 | 0.023742 | 0.050741 | 0.46791 | 0.639849 | -0.07571 | 0.123192 | AMR | End stage renal c | genitourinary | |
| 585.33 | 0.044552 | 0.047032 | 0.947277 | 0.343497 | -0.04763 | 0.136734 | AMR | Chronic Kidney D | genitourinary | |
| 585.34 | -0.04082 | 0.069197 | -0.58992 | 0.555247 | -0.17644 | 0.094803 | AMR | Chronic Kidney D | genitourinary | |
| 585.4 | 0.042327 | 0.053808 | 0.786618 | 0.431505 | -0.06314 | 0.147789 | AMR | Chronic kidney d | genitourinary | |
| 586 | -0.02217 | 0.037683 | -5.88E-01 | 0.556269 | -0.09603 | 0.051684 | AMR | Other disorders c | genitourinary | |
| 586.1 | -0.01771 | 0.142065 | -0.12464 | 0.90081 | -0.29615 | 0.260735 | AMR | Anatomical abno | genitourinary | |
| 586.11 | 0.102182 | 0.202576 | 0.504413 | 0.613971 | -0.29486 | 0.499224 | AMR | Small kidney | genitourinary | |
| 586.12 | -0.06676 | 1.75E-01 | -0.38132 | 0.702966 | -0.40989 | 0.276377 | AMR | Vesicoureteral re | genitourinary | |
| 586.2 | 0.043882 | 0.063393 | 0.69222 | 0.488799 | -0.08037 | 0.168131 | AMR | Cyst of kidney, ac | genitourinary | |
| 586.3 | 0.144865 | 0.171984 | 0.84232 | 0.399609 | -0.19222 | 0.481947 | AMR | Vascular disorde | genitourinary | |
| 586.4 | -0.05199 | 0.077859 | -6.68E-01 | 0.504304 | -0.20459 | 0.100612 | AMR | Stricture/obstruct | genitourinary | |
| 587 | 0.031416 | 0.060806 | 0.516661 | 0.605393 | -0.08776 | 0.150593 | AMR | Kidney replaced | genitourinary | |
| 588 | 0.024802 | 0.057456 | 0.431665 | 0.665985 | -0.08781 | 0.137413 | AMR | Disorders resultir | genitourinary | |
| 588.1 | -0.2945 | 0.191931 | -1.53441 | 0.124928 | -0.67068 | 0.081677 | AMR | Renal osteodystr | genitourinary | |
| 588.2 | 0.035329 | 0.058499 | 0.60392 | 0.545897 | -0.07933 | 0.149984 | AMR | Secondary hyper | genitourinary | |
| 589 | -0.02597 | 0.086853 | -0.29902 | 0.764922 | -0.1962 | 0.144258 | AMR | Abnormal results | genitourinary | |
| 590 | 0.080221 | 0.062492 | 1.283703 | 0.199246 | -0.04226 | 0.202704 | AMR | Pyelonephritis | genitourinary | |
| 591 | 0.03353 | 0.036223 | 0.925639 | 0.354634 | -0.03747 | 0.104526 | AMR | Urinary tract infe | genitourinary | |
| 592 | 0.052995 | 4.45E-02 | 1.189792 | 0.234128 | -0.0343 | 0.140294 | AMR | Cystitis and ureth | genitourinary | |
| 592.1 | 0.053291 | 0.045163 | 1.179969 | 0.238013 | -0.03523 | 0.141809 | AMR | Cystitis | genitourinary | |
| 592.11 | 0.048235 | 0.048435 | 0.995882 | 0.319307 | -0.0467 | 0.143166 | AMR | Acute cystitis | genitourinary | |

| | | | | | | | | | | |
|---|---|---|---|---|---|---|---|---|---|---|
| 592.12 | 0.663138 | 0.260505 | 2.55E+00 | 0.010909 | 0.152559 | 1.173718 | AMR | Chronic cystitis | genitourinary | |
| 592.13 | 0.161096 | 0.211565 | 0.761451 | 0.446388 | -0.25356 | 0.575757 | AMR | Chronic interstitia | genitourinary | |
| 592.2 | -0.00199 | 0.162214 | -0.01228 | 0.990199 | -0.31993 | 0.31594 | AMR | Urethritis and ure | genitourinary | |
| 592.3 | 0.915542 | 0.869863 | 1.052513 | 0.292564 | -0.78936 | 2.620442 | AMR | Urethral stricture | genitourinary | |
| 593 | 0.051746 | 0.046676 | 1.108639 | 0.267586 | -0.03974 | 0.143229 | AMR | Hematuria | genitourinary | |
| 593.1 | 0.050668 | 0.086101 | 5.88E-01 | 0.556218 | -0.11809 | 0.219423 | AMR | Gross hematuria | genitourinary | |
| 593.2 | 0.186339 | 0.068513 | 2.719745 | 0.006533 | 0.052055 | 0.320623 | AMR | Microscopic hem | genitourinary | |
| 594 | 0.044781 | 0.051747 | 0.865386 | 0.386827 | -0.05664 | 0.146204 | AMR | Urinary calculus | genitourinary | |
| 594.1 | 0.027722 | 0.05642 | 0.491357 | 0.623174 | -0.08286 | 0.138303 | AMR | Calculus of kidne | genitourinary | |
| 594.2 | 0.035578 | 0.174646 | 0.203717 | 0.838574 | -0.30672 | 0.377878 | AMR | Calculus of lower | genitourinary | |
| 594.3 | 0.153161 | 0.144067 | 1.063122 | 0.287727 | -0.12921 | 0.435528 | AMR | Calculus of urete | genitourinary | |
| 594.8 | 0.08059 | 0.156017 | 5.17E-01 | 0.605472 | -0.2252 | 0.386379 | AMR | Renal colic | genitourinary | |
| 595 | 0.084741 | 0.066536 | 1.273606 | 0.202803 | -0.04567 | 0.21515 | AMR | Hydronephrosis | genitourinary | |
| 596 | 0.075477 | 0.05487 | 1.375557 | 0.168959 | -0.03207 | 0.183021 | AMR | Other disorders c | genitourinary | |
| 596.1 | -0.12125 | 0.162826 | -0.74466 | 0.456479 | -0.44038 | 0.197884 | AMR | Bladder neck obs | genitourinary | |
| 596.5 | 0.113928 | 0.065333 | 1.74382 | 0.081191 | -0.01412 | 0.241978 | AMR | Functional disord | genitourinary | |
| 597 | -0.07495 | 0.112606 | -0.66555 | 0.505697 | -0.29565 | 0.145758 | AMR | Other disorders c | genitourinary | |
| 597.1 | -0.02835 | 0.157064 | -0.18049 | 0.856767 | -0.33619 | 0.279491 | AMR | Urethral stricture | genitourinary | |
| 597.2 | 0.020263 | 0.110292 | 0.183718 | 0.854235 | -0.19591 | 0.23643 | AMR | Urinary complica | genitourinary | |
| 597.8 | 0.603144 | 2.46E-01 | 2.456166 | 0.014043 | 0.121849 | 1.084439 | AMR | Urethral hypermo | genitourinary | |
| 598 | 0.079515 | 0.058638 | 1.356044 | 0.175085 | -0.03541 | 0.194443 | AMR | Abnormal finding | genitourinary | |
| 598.4 | -0.05107 | 0.219218 | -0.23295 | 0.815798 | -0.48073 | 0.378592 | AMR | Other cells and c | genitourinary | |
| 598.9 | 0.091596 | 0.05922 | 1.55E+00 | 0.121938 | -0.02447 | 0.207665 | AMR | Other nonspecific | genitourinary | |
| 599 | 0.011428 | 0.032462 | 0.352049 | 0.724801 | -0.0522 | 0.075052 | AMR | Other symptoms/ | genitourinary | |
| 599.1 | -0.04145 | 0.093236 | -0.44453 | 0.656661 | -0.22418 | 0.141293 | AMR | Urinary obstructio | genitourinary | |
| 599.2 | 0.044805 | 0.051073 | 0.877277 | 0.380336 | -0.0553 | 0.144906 | AMR | Retention of urin | genitourinary | |
| 599.3 | 0.033982 | 0.0404 | 0.841139 | 0.40027 | -0.0452 | 0.113165 | AMR | Dysuria | genitourinary | |
| 599.4 | 0.017861 | 0.052269 | 0.341707 | 0.732571 | -0.08458 | 0.120306 | AMR | Urinary incontine | genitourinary | |
| 599.5 | 0.002922 | 0.044565 | 0.065562 | 0.947727 | -0.08442 | 0.090267 | AMR | Frequency of urir | genitourinary | |
| 599.6 | 0.011813 | 0.086325 | 0.136846 | 0.891153 | -0.15738 | 0.181008 | AMR | Oliguria and anu | genitourinary | |
| 599.7 | 0.25535 | 3.21E-01 | 0.794682 | 0.426798 | -0.37443 | 0.885133 | AMR | Urethral discharg | genitourinary | |
| 599.8 | 0.002277 | 0.067744 | 0.033609 | 0.973189 | -0.1305 | 0.135053 | AMR | Other symptoms | genitourinary | |
| 599.9 | -0.00135 | 0.058591 | -0.023 | 0.981649 | -0.11618 | 0.113489 | AMR | Other abnormalit | genitourinary | |
| 600 | -0.11202 | 0.067049 | -1.67E+00 | 0.094792 | -0.24343 | 0.019399 | AMR | Hyperplasia of pr | genitourinary | |
| 601 | 0.03856 | 0.09469 | 0.407223 | 0.683844 | -0.14703 | 0.224148 | AMR | Inflammatory dis | genitourinary | |
| 601.1 | -0.01889 | 0.132895 | -0.14212 | 0.886982 | -0.27936 | 0.241581 | AMR | Prostatitis | genitourinary | |
| 601.11 | 0.099355 | 0.158831 | 0.625542 | 0.531616 | -0.21195 | 0.410658 | AMR | Acute prostatitis | genitourinary | |
| 601.12 | 0.062861 | 0.190426 | 0.330106 | 0.74132 | -0.31037 | 0.436089 | AMR | Chronic prostatiti | genitourinary | |

| | | | | | | | | | | |
|---|---|---|---|---|---|---|---|---|---|---|
| 601.3 | 0.522225 | 0.176647 | 2.956321 | 0.003113 | 0.176003 | 0.868447 | AMR | Orchitis and epid | genitourinary | |
| 601.4 | 0.035032 | 0.165931 | 0.211123 | 0.832791 | -0.29019 | 0.360251 | AMR | Balanoposthitis | genitourinary | |
| 601.8 | 0.367182 | 0.223468 | 1.643106 | 0.100361 | -0.07081 | 0.805172 | AMR | Other inflammato | genitourinary | |
| 602 | 0.556534 | 0.195469 | 2.84717 | 0.004411 | 0.173421 | 0.939646 | AMR | Other disorders c | genitourinary | |
| 602.3 | 0.135618 | 0.380067 | 0.356828 | 0.721221 | -0.6093 | 0.880536 | AMR | Dysplasia of pros | genitourinary | |
| 603 | 0.101871 | 0.126264 | 0.806805 | 0.419779 | -0.1456 | 0.349344 | AMR | Other disorders c | genitourinary | |
| 603.1 | 0.152578 | 0.143426 | 1.06E+00 | 0.287414 | -0.12853 | 0.433688 | AMR | Hydrocele | genitourinary | |
| 603.2 | 0.082349 | 0.184914 | 0.445336 | 0.656077 | -0.28008 | 0.444774 | AMR | Spermatocele | genitourinary | |
| 604 | 0.020645 | 0.106423 | 0.193988 | 0.846185 | -0.18794 | 0.229229 | AMR | Disorders of peni | genitourinary | |
| 604.1 | 0.22271 | 0.197088 | 1.130004 | 0.258475 | -0.16358 | 0.608996 | AMR | Redundant prepu | genitourinary | |
| 604.2 | 0.429137 | 0.521149 | 0.823444 | 0.410256 | -0.5923 | 1.450571 | AMR | Vascular disorde | genitourinary | |
| 604.3 | 0.495366 | 0.282412 | 1.754056 | 0.079421 | -0.05815 | 1.048884 | AMR | Peyronie's diseas | genitourinary | |
| 605 | 0.054371 | 0.069747 | 0.779544 | 0.43566 | -0.08233 | 0.191072 | AMR | Erectile dysfuncti | genitourinary | |
| 608 | 0.066637 | 0.078118 | 0.853031 | 0.393642 | -0.08647 | 0.219745 | AMR | Other disorders c | genitourinary | |
| 609 | 0.267709 | 0.207617 | 1.289437 | 0.197246 | -0.13921 | 0.674631 | AMR | Male infertility an | genitourinary | |
| 609.1 | -0.20408 | 0.304679 | -0.66982 | 0.502974 | -0.80124 | 0.393081 | AMR | Infertility, male | genitourinary | |
| 609.11 | 0.377249 | 0.49156 | 0.767452 | 0.442813 | -0.58619 | 1.340688 | AMR | Azoospermia anc | genitourinary | |
| 609.2 | 0.436185 | 0.259029 | 1.68E+00 | 0.092196 | -0.0715 | 0.943872 | AMR | Abnormal sperma | genitourinary | |
| 610 | 0.078228 | 0.077757 | 1.006058 | 0.314388 | -0.07417 | 0.230628 | AMR | Benign mammary | genitourinary | |
| 610.1 | -0.00927 | 0.089203 | -0.10394 | 0.917213 | -0.18411 | 0.165562 | AMR | Cystic mastopath | genitourinary | |
| 610.2 | 0.267724 | 0.223933 | 1.195556 | 0.23187 | -0.17118 | 0.706625 | AMR | Fibroadenosis of | genitourinary | |
| 610.3 | -0.05255 | 0.184459 | -0.28491 | 0.775712 | -0.41409 | 0.308978 | AMR | Fibrosclerosis of | genitourinary | |
| 610.4 | -0.02193 | 0.161893 | -1.35E-01 | 0.892264 | -0.33923 | 0.295378 | AMR | Benign neoplasm | genitourinary | |
| 610.8 | -0.04329 | 0.12954 | -0.33414 | 0.738271 | -0.29718 | 0.210608 | AMR | Other specified b | genitourinary | |
| 611 | -0.05268 | 0.05 | -1.05352 | 0.292102 | -0.15067 | 0.045322 | AMR | Abnormal finding | genitourinary | |
| 611.1 | -0.05677 | 0.055557 | -1.02185 | 0.306851 | -0.16566 | 0.052119 | AMR | Abnormal mamm | genitourinary | |
| 611.11 | 0.156759 | 0.139439 | 1.124212 | 0.260923 | -0.11654 | 0.430053 | AMR | Mammographic r | genitourinary | |
| 611.3 | 0.025867 | 0.0645 | 0.40104 | 0.68839 | -0.10055 | 0.152284 | AMR | Lump or mass in | genitourinary | |
| 612 | 0.04217 | 0.110821 | 3.81E-01 | 0.703555 | -0.17504 | 0.259376 | AMR | Breast conditions | genitourinary | |
| 612.1 | -0.26229 | 0.201651 | -1.30069 | 0.193364 | -0.65752 | 0.132943 | AMR | Galactorrhea | genitourinary | |
| 612.2 | 0.046834 | 0.132578 | 0.353256 | 0.723896 | -0.21301 | 0.306682 | AMR | Hypertrophy of b | genitourinary | |
| 612.3 | 0.439491 | 0.44824 | 0.980481 | 0.326849 | -0.43904 | 1.318024 | AMR | Congenital anom | genitourinary | |
| 613 | 0.042829 | 0.055538 | 0.771167 | 0.440608 | -0.06602 | 0.151682 | AMR | Other nonmaligna | genitourinary | |
| 613.1 | -0.10764 | 0.153411 | -7.02E-01 | 0.482903 | -0.40832 | 0.193041 | AMR | Inflammatory disc | genitourinary | |
| 613.5 | 0.093637 | 0.067666 | 1.383812 | 0.166416 | -0.03899 | 0.226261 | AMR | Mastodynia | genitourinary | |
| 613.7 | 0.348102 | 0.141557 | 2.45909 | 0.013929 | 0.070655 | 0.625548 | AMR | Other signs and s | genitourinary | |
| 613.8 | -0.01436 | 0.088421 | -0.16245 | 0.870954 | -0.18767 | 0.158938 | AMR | Other specified d | genitourinary | |
| 613.9 | -0.11324 | 0.180123 | -0.62868 | 0.529556 | -0.46628 | 0.239794 | AMR | Breast disorder N | genitourinary | |

| | | | | | | | | | | |
|---|---|---|---|---|---|---|---|---|---|---|
| 614 | -0.02578 | 0.0518 | -0.49763 | 0.618744 | -0.1273 | 0.075749 | AMR | Inflammatory dis | genitourinary | |
| 614.1 | -0.15565 | 0.141386 | -1.10E+00 | 0.270954 | -0.43276 | 0.121464 | AMR | Pelvic peritoneal | genitourinary | |
| 614.3 | -0.16055 | 0.152742 | -1.0511 | 0.293211 | -0.45992 | 0.138821 | AMR | Pelvic inflammato | genitourinary | |
| 614.31 | 0.374306 | 0.40591 | 0.92214 | 0.356456 | -0.42126 | 1.169875 | AMR | Acute inflammato | genitourinary | |
| 614.32 | 0.070944 | 0.180488 | 0.39307 | 0.694268 | -0.28281 | 0.424695 | AMR | Chronic inflamma | genitourinary | |
| 614.33 | -0.37756 | 0.20081 | -1.8802 | 0.060081 | -0.77114 | 0.016017 | AMR | Pelvic inflammato | genitourinary | |
| 614.4 | -0.18015 | 0.210326 | -0.85651 | 0.391714 | -0.59238 | 0.232085 | AMR | Inflammatory dis | genitourinary | |
| 614.5 | -0.00978 | 0.054483 | -0.17943 | 0.857601 | -0.11656 | 0.097009 | AMR | Inflammatory dis | genitourinary | |
| 614.51 | 0.035021 | 0.095479 | 0.366799 | 0.713769 | -0.15211 | 0.222156 | AMR | Cervicitis and en | genitourinary | |
| 614.52 | -0.00399 | 0.061205 | -0.06511 | 0.948087 | -0.12395 | 0.115976 | AMR | Vaginitis and vulv | genitourinary | |
| 614.53 | 0.01372 | 0.229448 | 0.059794 | 0.95232 | -0.43599 | 0.463429 | AMR | Cyst or abscess | genitourinary | |
| 614.54 | 0.262254 | 0.184641 | 1.420345 | 0.155507 | -0.09964 | 0.624144 | AMR | Abscess or ulcera | genitourinary | |
| 615 | 0.010803 | 0.087721 | 1.23E-01 | 0.901989 | -0.16113 | 0.182733 | AMR | Endometriosis | genitourinary | |
| 617 | 0.063676 | 0.053405 | 1.19E+00 | 0.233131 | -0.041 | 0.168348 | AMR | Disorders second | genitourinary | |
| 618 | 0.096593 | 0.085361 | 1.131593 | 0.257806 | -0.07071 | 0.263897 | AMR | Genital prolapse | genitourinary | |
| 618.1 | 0.095658 | 0.097299 | 0.983136 | 0.325541 | -0.09504 | 0.28636 | AMR | Prolapse of vagin | genitourinary | |
| 618.2 | 0.066118 | 0.138939 | 0.47588 | 0.63416 | -0.2062 | 0.338434 | AMR | Uterine/Uterovag | genitourinary | |
| 618.5 | 0.668479 | 0.223462 | 2.991465 | 0.002776 | 0.230501 | 1.106456 | AMR | Prolapse of vagin | genitourinary | |
| 618.6 | 0.338138 | 0.365364 | 0.925484 | 0.354714 | -0.37796 | 1.054237 | AMR | Vaginal enteroce | genitourinary | |
| 619 | -0.02451 | 0.042307 | -0.57926 | 0.562411 | -0.10743 | 0.058414 | AMR | Noninflammatory | genitourinary | |
| 619.1 | 0.030649 | 0.081819 | 0.374596 | 0.707961 | -0.12971 | 0.191011 | AMR | Noninflammatory | genitourinary | |
| 619.2 | 0.042519 | 0.099581 | 0.426976 | 0.669396 | -0.15266 | 0.237695 | AMR | Disorders of uter | genitourinary | |
| 619.3 | -0.10377 | 0.088714 | -1.16971 | 0.242119 | -0.27765 | 0.070107 | AMR | Noninflammatory | genitourinary | |
| 619.4 | 0.073391 | 0.051148 | 1.43E+00 | 0.151326 | -0.02686 | 0.17364 | AMR | Noninflammatory | genitourinary | |
| 619.5 | -0.01364 | 0.104737 | -0.13024 | 0.896375 | -0.21892 | 0.19164 | AMR | Noninflammatory | genitourinary | |
| 620 | 0.041519 | 0.165917 | 0.250239 | 0.802403 | -0.28367 | 0.36671 | AMR | Dysplasia of fema | genitourinary | |
| 620.1 | 2.67E-01 | 0.195934 | 1.361934 | 0.173219 | -0.11717 | 0.650871 | AMR | Dysplasia of cerv | genitourinary | |
| 621 | 0.475265 | 0.225771 | 2.105075 | 0.035285 | 0.032762 | 0.917767 | AMR | Endometrial hype | genitourinary | |
| 622 | -0.14653 | 0.085582 | -1.71219 | 0.086862 | -0.31427 | 0.021205 | AMR | Polyp of female g | genitourinary | |
| 622.1 | -0.1396 | 0.094106 | -1.48346 | 0.137951 | -0.32405 | 0.044842 | AMR | Polyp of corpus u | genitourinary | |
| 622.2 | -0.2377 | 0.162446 | -1.46325 | 0.143398 | -0.55609 | 0.080688 | AMR | Mucous polyp of | genitourinary | |
| 623 | -0.02573 | 1.06E-01 | -0.24274 | 0.808206 | -0.23344 | 0.181987 | AMR | Hypertrophy of fe | genitourinary | |
| 624 | 0.051154 | 0.073028 | 0.70047 | 0.483634 | -0.09198 | 0.194287 | AMR | Symptoms involv | genitourinary | |
| 624.1 | 0.143037 | 0.312633 | 0.457524 | 0.647295 | -0.46971 | 0.755787 | AMR | Dystrophy of fem | genitourinary | |
| 624.2 | 0.393039 | 0.511042 | 7.69E-01 | 0.441839 | -0.60859 | 1.394663 | AMR | Atrophy of female | genitourinary | |
| 624.9 | 0.028753 | 0.07191 | 0.39985 | 0.689267 | -0.11219 | 0.169694 | AMR | stress incontinen | genitourinary | |
| 625 | 0.071236 | 0.052152 | 1.365937 | 0.171959 | -0.03098 | 0.173452 | AMR | Pain and other sy | genitourinary | |
| 625.1 | -0.06309 | 0.10775 | -0.58553 | 0.558188 | -0.27428 | 0.148094 | AMR | Dyspareunia | genitourinary | |

| | | | | | | | | | | |
|---|---|---|---|---|---|---|---|---|---|---|
| 626 | 0.066161 | 0.046332 | 1.427977 | 0.153298 | -0.02465 | 0.15697 | AMR | Disorders of men | genitourinary | |
| 626.1 | 0.106695 | 0.050726 | 2.10E+00 | 0.035433 | 0.007275 | 0.206115 | AMR | Irregular menstru | genitourinary | |
| 626.11 | 0.109194 | 0.082733 | 1.319827 | 0.186893 | -0.05296 | 0.271348 | AMR | Absent or infrequ | genitourinary | |
| 626.12 | 0.069257 | 0.069081 | 1.002553 | 0.316077 | -0.06614 | 0.204652 | AMR | Excessive or freq | genitourinary | |
| 626.13 | 0.101662 | 0.063268 | 1.606857 | 0.108086 | -0.02234 | 0.225664 | AMR | Irregular menstru | genitourinary | |
| 626.14 | -0.00758 | 0.087275 | -0.08687 | 0.930771 | -0.17864 | 0.163474 | AMR | Irregular menstru | genitourinary | |
| 626.15 | -0.15345 | 0.1991 | -0.77071 | 0.440878 | -0.54368 | 0.236781 | AMR | Infertility, female, | genitourinary | |
| 626.2 | 0.039617 | 0.102058 | 3.88E-01 | 0.697884 | -0.16041 | 0.239646 | AMR | Dysmenorrhea | genitourinary | |
| 626.21 | -0.48674 | 0.382898 | -1.2712 | 0.203658 | -1.2372 | 0.263727 | AMR | Mittelschmerz | genitourinary | |
| 626.4 | -0.48665 | 0.22821 | -2.13248 | 0.032967 | -0.93394 | -0.03937 | AMR | Premenstrual ten | genitourinary | |
| 626.8 | -0.18196 | 0.12908 | -1.40966 | 0.15864 | -0.43495 | 0.071033 | AMR | Infertility, female | genitourinary | |
| 627 | -0.0051 | 0.045019 | -0.11318 | 0.909888 | -0.09333 | 0.083141 | AMR | Menopausal and | genitourinary | |
| 627.1 | 0.057513 | 0.096971 | 5.93E-01 | 0.553116 | -0.13255 | 0.247573 | AMR | Postmenopausal | genitourinary | |
| 627.2 | -0.02796 | 0.050404 | -0.55468 | 0.579115 | -0.12675 | 0.070832 | AMR | Symptomatic me | genitourinary | |
| 627.21 | -0.27033 | 0.302752 | -0.89291 | 0.371905 | -0.86371 | 0.323052 | AMR | Symptomatic arti | genitourinary | |
| 627.22 | 0.00555 | 0.06572 | 0.084457 | 0.932693 | -0.12326 | 0.134359 | AMR | Need for Hormor | genitourinary | |
| 627.3 | 0.050793 | 0.079821 | 0.63633 | 0.524561 | -0.10565 | 0.207239 | AMR | Postmenopausal | genitourinary | |
| 627.4 | -0.43067 | 0.160401 | -2.68495 | 0.007254 | -0.74505 | -0.11629 | AMR | Premenopausal r | genitourinary | |
| 627.5 | -0.15685 | 0.166187 | -9.44E-01 | 0.345261 | -0.48257 | 0.168869 | AMR | Premature meno | genitourinary | |
| 628 | 0.106765 | 0.069041 | 1.54641 | 0.122006 | -0.02855 | 0.242083 | AMR | Ovarian cyst | genitourinary | |
| 634 | -0.02515 | 0.090777 | -0.27701 | 0.781774 | -0.20307 | 0.152774 | AMR | Miscarriage; stillb | pregnancy complications | |
| 634.1 | 0.049743 | 0.157612 | 0.315606 | 0.752302 | -0.25917 | 0.358657 | AMR | Missed abortion/I | pregnancy complications | |
| 634.3 | -0.26617 | 0.254718 | -1.04497 | 0.296038 | -0.76541 | 0.233066 | AMR | Ectopic pregnanc | pregnancy complications | |
| 635 | 0.025354 | 0.101336 | 0.250195 | 0.802436 | -0.17326 | 0.223969 | AMR | Hemorrhage duri | pregnancy complications | |
| 635.2 | -0.01418 | 0.113259 | -0.12522 | 0.900353 | -0.23617 | 0.207803 | AMR | Antepartum hem | pregnancy complications | |
| 635.3 | 0.026612 | 0.172439 | 0.154329 | 0.87735 | -0.31136 | 0.364586 | AMR | Placenta previa a | pregnancy complications | |
| 636 | 0.063704 | 0.090022 | 0.707652 | 0.479161 | -0.11274 | 0.240144 | AMR | Early or threaten | pregnancy complications | |
| 636.1 | 0.112524 | 0.229237 | 0.490864 | 0.623523 | -0.33677 | 0.561821 | AMR | Threatened prem | pregnancy complications | |
| 636.2 | -0.23903 | 0.261123 | -0.91541 | 0.359977 | -0.75082 | 0.272757 | AMR | Early onset of de | pregnancy complications | |
| 636.3 | -0.01256 | 0.118697 | -1.06E-01 | 0.915722 | -0.2452 | 0.220081 | AMR | Hemorrhage in e | pregnancy complications | |
| 636.8 | -0.31117 | 0.262812 | -1.18401 | 0.236409 | -0.82627 | 0.20393 | AMR | Cervical incompe | pregnancy complications | |
| 637 | -0.09194 | 0.223994 | -0.41047 | 0.681464 | -0.53096 | 0.347079 | AMR | Short gestation; l | pregnancy complications | |
| 638 | 0.076522 | 0.084298 | 0.907755 | 0.364008 | -0.0887 | 0.241744 | AMR | Other high-risk p | pregnancy complications | |
| 639 | -0.04943 | 0.746744 | -0.0662 | 0.947219 | -1.51303 | 1.414157 | AMR | Complications fo | pregnancy complications | |
| 642 | -0.00766 | 0.110259 | -6.95E-02 | 0.944603 | -0.22376 | 0.208442 | AMR | Hypertension cor | pregnancy complications | |
| 642.1 | -0.02698 | 0.16133 | -0.16722 | 0.8672 | -0.34318 | 0.289225 | AMR | Preeclampsia an | pregnancy complications | |
| 643 | 0.449816 | 0.145307 | 3.095623 | 0.001964 | 0.165019 | 0.734613 | AMR | Excessive vomiti | pregnancy complications | |
| 643.1 | 0.386423 | 0.245966 | 1.571045 | 0.116172 | -0.09566 | 0.868507 | AMR | Hyperemesis gra | pregnancy complications | |

| | | | | | | | | | | |
|---|---|---|---|---|---|---|---|---|---|---|
| 644 | 0.179237 | 0.113919 | 1.57337 | 0.115633 | -0.04404 | 0.402514 | AMR | Anemia during pr | pregnancy complications | |
| 645 | -0.00061 | 0.137709 | -0.00442 | 0.996471 | -0.27051 | 0.269297 | AMR | Late pregnancy a | pregnancy complications | |
| 646 | 0.030398 | 0.076532 | 3.97E-01 | 0.69123 | -0.1196 | 0.180398 | AMR | Other complicatio | pregnancy complications | |
| 647 | 0.241424 | 0.125816 | 1.91886 | 0.055002 | -0.00517 | 0.488019 | AMR | Infectious and pa | pregnancy complications | |
| 647.1 | 0.239881 | 0.15805 | 1.517754 | 0.129077 | -0.06989 | 0.549654 | AMR | Infections of geni | pregnancy complications | |
| 647.3 | -0.13193 | 0.275935 | -0.47813 | 0.632558 | -0.67275 | 0.40889 | AMR | Major puerperal i | pregnancy complications | |
| 649 | 0.061925 | 0.071459 | 0.866589 | 0.386167 | -0.07813 | 0.201982 | AMR | Other conditions | pregnancy complications | |
| 649.1 | 0.281724 | 0.11261 | 2.50E+00 | 0.012358 | 0.061012 | 0.502435 | AMR | Diabetes or abno | pregnancy complications | |
| 650 | 0.008394 | 0.093706 | 0.08958 | 0.928621 | -0.17527 | 0.192055 | AMR | Normal delivery | pregnancy complications | |
| 651 | -0.04871 | 0.162663 | -0.29944 | 0.764603 | -0.36752 | 0.270105 | AMR | Multiple gestatior | pregnancy complications | |
| 652 | 0.304909 | 0.314151 | 0.970582 | 0.331757 | -0.31082 | 0.920633 | AMR | Malposition and r | pregnancy complications | |
| 653 | 0.136754 | 0.104208 | 1.312315 | 0.189414 | -0.06749 | 0.340999 | AMR | Problems associa | pregnancy complications | |
| 654 | 0.071088 | 0.083406 | 0.852306 | 0.394044 | -0.09239 | 0.234561 | AMR | Other and unspe | pregnancy complications | |
| 654.1 | -0.09663 | 0.138766 | -6.96E-01 | 0.486229 | -0.3686 | 0.175351 | AMR | Abnormality of or | pregnancy complications | |
| 654.2 | 0.271279 | 0.375761 | 7.22E-01 | 0.470328 | -0.4652 | 1.007757 | AMR | Rhesus isoimmu | pregnancy complications | |
| 655 | 0.108634 | 0.077667 | 1.398718 | 0.161897 | -0.04359 | 0.260859 | AMR | Known or suspec | pregnancy complications | |
| 655.1 | 0.193042 | 0.104952 | 1.839334 | 0.065866 | -0.01266 | 0.398745 | AMR | Abnormality in fe | pregnancy complications | |
| 656 | 0.109286 | 0.086492 | 1.263538 | 0.206396 | -0.06024 | 0.278808 | AMR | Other perinatal c | pregnancy complications | |
| 656.1 | -1.28396 | 0.780605 | -1.64E+00 | 0.100005 | -2.81392 | 0.245997 | AMR | Isoimmunization | pregnancy complications | |
| 656.2 | 0.368831 | 0.401453 | 0.91874 | 0.358232 | -0.418 | 1.155665 | AMR | Respiratory cond | pregnancy complications | |
| 656.3 | 0.025851 | 0.281499 | 0.091834 | 0.92683 | -0.52588 | 0.577579 | AMR | Endocrine and m | pregnancy complications | |
| 656.4 | 0.841544 | 0.532129 | 1.581466 | 0.113772 | -0.20141 | 1.884497 | AMR | Hemorrhage of fe | pregnancy complications | |
| 656.5 | 5.158746 | | | | | | AMR | Hematological di | pregnancy complications | |
| 656.6 | 0.235599 | 0.466533 | 0.505001 | 0.613558 | -0.67879 | 1.149987 | AMR | Perinatal disorde | pregnancy complications | |
| 656.7 | -0.22698 | 0.573243 | -3.96E-01 | 0.692142 | -1.35051 | 0.896561 | AMR | Conditions involv | pregnancy complications | |
| 656.8 | 0.735795 | 1.837743 | 0.40038 | 0.688877 | -2.86612 | 4.337705 | AMR | Perinatal jaundic | pregnancy complications | |
| 656.9 | 1.418407 | 1.705858 | 0.831492 | 0.405696 | -1.92501 | 4.761828 | AMR | Neonatal bradyca | pregnancy complications | |
| 657 | 0.175568 | 0.493515 | 0.355751 | 0.722027 | -0.7917 | 1.142839 | AMR | Infections specifi | pregnancy complications | |
| 658 | 0.506831 | 0.274811 | 1.844286 | 0.065141 | -0.03179 | 1.045452 | AMR | Maternal complic | pregnancy complications | |
| 661 | 0.217245 | 0.113403 | 1.92E+00 | 0.055405 | -0.00502 | 0.439512 | AMR | Fetal distress and | pregnancy complications | |
| 665 | 0.054901 | 0.092617 | 0.592775 | 0.553332 | -0.12662 | 0.236426 | AMR | Obstetrical/birth t | pregnancy complications | |
| 668 | 1.057598 | 0.78611 | 1.345357 | 0.17851 | -0.48315 | 2.598345 | AMR | Complications of | pregnancy complications | |
| 669 | 0.077168 | 0.123468 | 0.625001 | 0.53197 | -0.16483 | 0.319161 | AMR | Complications of | pregnancy complications | |
| 671 | 0.034256 | 0.128088 | 0.267438 | 0.789132 | -0.21679 | 0.285304 | AMR | Venous/cerebrov | pregnancy complications | |
| 674 | -0.05356 | 0.183665 | -0.29162 | 0.770578 | -0.41354 | 0.306416 | AMR | Other complicatio | pregnancy complications | |
| 676 | 0.067219 | 0.18743 | 3.59E-01 | 0.719867 | -0.30014 | 0.434575 | AMR | Other disorders o | pregnancy complications | |
| 681 | -0.03629 | 0.042568 | -0.85252 | 0.393927 | -0.11972 | 0.047142 | AMR | Superficial celluli | dermatologic | |
| 681.1 | 0.057413 | 0.082941 | 6.92E-01 | 0.488803 | -0.10515 | 0.219975 | AMR | Cellulitis and abs | dermatologic | |

| | | | | | | | | | | |
|---|---|---|---|---|---|---|---|---|---|---|
| 681.2 | 0.126716 | 0.126852 | 0.998926 | 0.31783 | -0.12191 | 0.375343 | AMR | Cellulitis and abs | dermatologic | |
| 681.3 | 0.011095 | 0.100208 | 0.110716 | 0.911842 | -0.18531 | 0.207498 | AMR | Cellulitis and abs | dermatologic | |
| 681.5 | -0.13958 | 0.071386 | -1.96E+00 | 0.050548 | -0.27949 | 0.000333 | AMR | Cellulitis and abs | dermatologic | |
| 681.6 | -0.08523 | 0.133131 | -0.64018 | 0.522057 | -0.34616 | 0.175705 | AMR | Cellulitis and abs | dermatologic | |
| 681.7 | -0.00751 | 0.075212 | -0.09986 | 0.920457 | -0.15492 | 0.139903 | AMR | Cellulitis and abs | dermatologic | |
| 686 | 0.028461 | 0.055355 | 0.51416 | 0.60714 | -0.08003 | 0.136956 | AMR | Other local infect | dermatologic | |
| 686.1 | -0.36066 | 0.157974 | -2.28303 | 0.022429 | -0.67028 | -0.05104 | AMR | Carbuncle and fu | dermatologic | |
| 686.2 | 0.135505 | 0.130992 | 1.034452 | 0.300925 | -0.12123 | 0.392245 | AMR | Impetigo | dermatologic | |
| 686.3 | -0.4317 | 0.243339 | -1.77E+00 | 0.076052 | -0.90864 | 0.045236 | AMR | Pilonidal cyst | dermatologic | |
| 686.4 | -0.18153 | 0.208102 | -8.72E-01 | 0.383039 | -0.5894 | 0.226343 | AMR | Pyogenic granulo | dermatologic | |
| 686.5 | -0.0065 | 0.228531 | -2.84E-02 | 0.97732 | -0.45441 | 0.441416 | AMR | Pyoderma | dermatologic | |
| 687 | -0.03089 | 0.04273 | -0.72281 | 0.469794 | -0.11463 | 0.052863 | AMR | Symptoms affect | dermatologic | |
| 687.1 | 0.074671 | 0.038808 | 1.924101 | 0.054342 | -0.00139 | 0.150734 | AMR | Rash and other r | dermatologic | |
| 687.2 | -0.01857 | 0.047579 | -3.90E-01 | 0.696366 | -0.11182 | 0.074686 | AMR | Localized superfi | dermatologic | |
| 687.3 | -0.19728 | 0.138261 | -1.42689 | 0.153612 | -0.46827 | 0.073703 | AMR | Changes in skin t | dermatologic | |
| 687.4 | 0.042438 | 0.041229 | 1.029319 | 0.30333 | -0.03837 | 0.123246 | AMR | Disturbance of sk | dermatologic | |
| 689 | 0.034226 | 0.042911 | 0.797599 | 0.425103 | -0.04988 | 0.11833 | AMR | Disorder of skin a | dermatologic | |
| 690 | -0.07439 | 0.073744 | -1.00873 | 0.313104 | -0.21892 | 0.070147 | AMR | Erythematosquar | dermatologic | |
| 690.1 | -0.07439 | 0.073744 | -1.00873 | 0.313104 | -0.21892 | 0.070147 | AMR | Seborrheic derma | dermatologic | |
| 691 | -0.02084 | 0.121958 | -1.71E-01 | 0.86435 | -0.25987 | 0.218199 | AMR | Congenital anom | dermatologic | |
| 691.1 | 0.549004 | 0.459044 | 1.20E+00 | 0.231707 | -0.35071 | 1.448715 | AMR | Ichthyosis conge | dermatologic | |
| 691.3 | -0.29576 | 0.360075 | -8.21E-01 | 0.411427 | -1.00149 | 0.409973 | AMR | Congenital pigme | dermatologic | |
| 694 | 0.000459 | 0.047515 | 0.009667 | 0.992287 | -0.09267 | 0.093586 | AMR | Dyschromia and | dermatologic | |
| 694.1 | 0.186286 | 0.174741 | 1.066069 | 0.286392 | -0.1562 | 0.528772 | AMR | Vitiligo | dermatologic | |
| 694.2 | -0.01586 | 0.048919 | -3.24E-01 | 0.745781 | -0.11174 | 0.080019 | AMR | Other dyschromia | dermatologic | |
| 694.3 | 0.527184 | 0.348237 | 1.513868 | 0.130059 | -0.15535 | 1.209716 | AMR | Vascular disorde | dermatologic | |
| 695 | 0.091359 | 0.050171 | 1.820931 | 0.068617 | -0.00698 | 0.189693 | AMR | Erythematous co | dermatologic | |
| 695.1 | 0.120889 | 0.263617 | 0.458577 | 0.646538 | -0.39579 | 0.637569 | AMR | Toxic erythema | dermatologic | |
| 695.2 | 0.628743 | 0.421287 | 1.492433 | 0.135586 | -0.19696 | 1.454451 | AMR | Bullous dermatos | dermatologic | |
| 695.21 | 1.063442 | 1.137365 | 0.935005 | 0.349786 | -1.16575 | 3.292637 | AMR | Dermatitis herpet | dermatologic | |
| 695.22 | 0.555183 | 0.691093 | 8.03E-01 | 0.421778 | -0.79933 | 1.9097 | AMR | Pemphigus and p | dermatologic | |
| 695.3 | 0.107384 | 0.089081 | 1.21E+00 | 0.228024 | -0.06721 | 0.28198 | AMR | Rosacea | dermatologic | |
| 695.4 | 0.143337 | 0.088373 | 1.62E+00 | 0.104815 | -0.02987 | 0.316545 | AMR | Lupus (localized | dermatologic | |
| 695.41 | 0.265428 | 0.144849 | 1.832446 | 0.066885 | -0.01847 | 0.549327 | AMR | Cutaneous lupus | dermatologic | |
| 695.42 | 0.128592 | 0.089641 | 1.434521 | 0.151424 | -0.0471 | 0.304284 | AMR | Systemic lupus e | dermatologic | |
| 695.7 | 0.010118 | 0.121392 | 8.33E-02 | 0.933575 | -0.22781 | 0.248041 | AMR | Prurigo and Liche | dermatologic | |
| 695.8 | 0.030053 | 0.090855 | 0.330778 | 0.740812 | -0.14802 | 0.208126 | AMR | Other specified e | dermatologic | |
| 695.81 | 0.159255 | 0.236576 | 0.673167 | 0.500841 | -0.30443 | 0.622936 | AMR | Erythema nodosu | dermatologic | |

| | | | | | | | | | | |
|---|---|---|---|---|---|---|---|---|---|---|
| 695.9 | -0.05423 | 0.158548 | -0.34204 | 0.732319 | -0.36498 | 0.256519 | AMR | Unspecified eryth | dermatologic | |
| 696 | 0.011834 | 0.090137 | 0.13129 | 0.895546 | -0.16483 | 0.1885 | AMR | Psoriasis and rel | dermatologic | |
| 696.2 | 0.795669 | 0.590283 | 1.347945 | 0.177676 | -0.36126 | 1.952602 | AMR | Parapsoriasis | dermatologic | |
| 696.3 | 0.129712 | 0.19509 | 6.65E-01 | 0.506124 | -0.25266 | 0.512081 | AMR | Pityriasis | dermatologic | |
| 696.4 | -0.02225 | 0.096859 | -0.22971 | 0.818317 | -0.21209 | 0.16759 | AMR | Psoriasis | dermatologic | |
| 696.41 | -0.08131 | 0.100404 | -0.80988 | 0.418012 | -0.2781 | 0.115474 | AMR | Psoriasis vulgaris | dermatologic | |
| 696.42 | 0.153428 | 0.183323 | 0.836928 | 0.402633 | -0.20588 | 0.512734 | AMR | Psoriatic arthropa | dermatologic | |
| 697 | 0.680157 | 0.218459 | 3.113439 | 0.001849 | 0.251987 | 1.108328 | AMR | Sarcoidosis | dermatologic | |
| 698 | 0.091384 | 0.046317 | 1.973003 | 0.048495 | 0.000604 | 0.182164 | AMR | Pruritus and relat | dermatologic | |
| 700 | 0.208367 | 0.126482 | 1.647403 | 0.099475 | -0.03953 | 0.456267 | AMR | Corns and callos | dermatologic | |
| 701 | -0.02478 | 0.040606 | -0.61029 | 0.541667 | -0.10437 | 0.054805 | AMR | Other hypertroph | dermatologic | |
| 701.1 | 0.084313 | 8.15E-02 | 1.03495 | 0.300692 | -0.07536 | 0.243983 | AMR | Keratoderma, ac | dermatologic | |
| 701.2 | -0.0554 | 0.068714 | -0.80625 | 0.4201 | -0.19008 | 0.079276 | AMR | Scar conditions a | dermatologic | |
| 701.3 | -0.06544 | 1.51E-01 | -0.43425 | 0.664109 | -0.36078 | 0.229911 | AMR | Circumscribed sc | dermatologic | |
| 701.4 | -0.09942 | 0.103266 | -0.96272 | 0.33569 | -0.30181 | 0.102982 | AMR | Keloid scar | dermatologic | |
| 701.5 | -0.04766 | 0.131565 | -0.36224 | 0.717172 | -0.30552 | 0.210204 | AMR | Abnormal granula | dermatologic | |
| 701.6 | -0.23121 | 0.146397 | -1.57932 | 0.114262 | -0.51814 | 0.055725 | AMR | Acquired acantho | dermatologic | |
| 702 | -0.06302 | 0.048286 | -1.3051 | 0.191858 | -0.15766 | 0.031621 | AMR | Degenerative ski | dermatologic | |
| 702.1 | -0.11339 | 0.092429 | -1.22677 | 0.219911 | -0.29455 | 0.067769 | AMR | Actinic keratosis | dermatologic | |
| 702.2 | -0.01929 | 0.050632 | -0.38105 | 0.703168 | -0.11853 | 0.079944 | AMR | Seborrheic kerato | dermatologic | |
| 702.4 | 0.46567 | 0.22922 | 2.031541 | 0.0422 | 0.016407 | 0.914933 | AMR | Degenerative ski | dermatologic | |
| 703 | 0.060013 | 0.064044 | 0.937056 | 0.34873 | -0.06551 | 0.185536 | AMR | Diseases of nail, | dermatologic | |
| 703.1 | 0.039306 | 7.73E-02 | 0.508287 | 0.611252 | -0.11226 | 0.190871 | AMR | Ingrowing nail | dermatologic | |
| 704 | 0.02623 | 0.049769 | 0.527047 | 0.598161 | -0.07131 | 0.123775 | AMR | Diseases of hair | dermatologic | |
| 704.1 | 0.004292 | 0.067565 | 0.063518 | 0.949354 | -0.12813 | 0.136716 | AMR | Alopecia | dermatologic | |
| 704.11 | 0.017345 | 0.222051 | 7.81E-02 | 0.937737 | -0.41787 | 0.452557 | AMR | Alopecia Areata | dermatologic | |
| 704.12 | 0.065748 | 0.179686 | 3.66E-01 | 0.714436 | -0.28643 | 0.417927 | AMR | Telogen effluvium | dermatologic | |
| 704.2 | -0.19666 | 0.161183 | -1.22E+00 | 0.222433 | -0.51257 | 0.119256 | AMR | Hirsutism | dermatologic | |
| 704.8 | 0.083281 | 0.068248 | 1.220274 | 0.222361 | -0.05048 | 0.217045 | AMR | Other specified d | dermatologic | |
| 705 | 0.081592 | 0.117184 | 0.696275 | 0.486257 | -0.14808 | 0.311267 | AMR | Disorders of swe | dermatologic | |
| 705.1 | 0.280058 | 0.188167 | 1.488351 | 0.136658 | -0.08874 | 0.648859 | AMR | Dyshidrosis | dermatologic | |
| 705.3 | 0.031652 | 0.169199 | 0.187072 | 0.851604 | -0.29997 | 0.363276 | AMR | Hidradenitis | dermatologic | |
| 705.8 | 0.137694 | 0.081304 | 1.693564 | 0.090348 | -0.02166 | 0.297046 | AMR | Hyperhidrosis | dermatologic | |
| 706 | -0.03836 | 0.044273 | -0.86653 | 0.386199 | -0.12514 | 0.048409 | AMR | Diseases of seba | dermatologic | |
| 706.1 | -0.06128 | 0.065578 | -0.9344 | 0.350098 | -0.18981 | 0.067255 | AMR | Acne | dermatologic | |
| 706.2 | 0.00059 | 0.065494 | 0.009006 | 0.992814 | -0.12778 | 0.128955 | AMR | Sebaceous cyst | dermatologic | |
| 706.3 | -0.32253 | 0.349041 | -9.24E-01 | 0.355464 | -1.00664 | 0.361579 | AMR | Seborrhea | dermatologic | |
| 706.8 | 0.048301 | 0.07485 | 6.45E-01 | 0.518726 | -0.0984 | 0.195004 | AMR | Other specified d | dermatologic | |

| | | | | | | | | | | |
|---|---|---|---|---|---|---|---|---|---|---|
| 707 | 0.028217 | 0.063948 | 0.441252 | 0.65903 | -0.09712 | 0.153554 | AMR | Chronic ulcer of s | dermatologic | |
| 707.1 | 0.066134 | 0.097336 | 0.679442 | 0.496858 | -0.12464 | 0.25691 | AMR | Decubitus ulcer | dermatologic | |
| 707.2 | -0.01301 | 0.089463 | -0.14543 | 0.884368 | -0.18836 | 0.162334 | AMR | Chronic ulcer of l | dermatologic | |
| 707.3 | 0.01813 | 0.095284 | 0.190277 | 0.849092 | -0.16862 | 0.204883 | AMR | Chronic ulcer of u | dermatologic | |
| 709 | 0.143733 | 0.068703 | 2.092077 | 0.036432 | 0.009077 | 0.278388 | AMR | Diffuse diseases | dermatologic | |
| 709.2 | 0.078019 | 0.094808 | 0.822917 | 0.410555 | -0.1078 | 0.263839 | AMR | Sicca syndrome | dermatologic | |
| 709.3 | 0.151502 | 0.133571 | 1.13424 | 0.256694 | -0.11029 | 0.413297 | AMR | Systemic scleros | dermatologic | |
| 709.4 | 0.31003 | 0.271216 | 1.143111 | 0.252993 | -0.22154 | 0.841604 | AMR | Polymyositis | dermatologic | |
| 709.5 | 0.155248 | 0.189974 | 0.817206 | 0.413811 | -0.21709 | 0.527589 | AMR | Dermatomyositis | dermatologic | |
| 709.6 | 0.037816 | 0.16246 | 2.33E-01 | 0.815939 | -0.2806 | 0.356233 | AMR | Other specified d | dermatologic | |
| 709.7 | 0.099441 | 0.10749 | 0.925116 | 0.354906 | -0.11124 | 0.310117 | AMR | Unspecified diffu | dermatologic | |
| 710 | 0.09997 | 0.093518 | 1.068996 | 0.285071 | -0.08332 | 0.283261 | AMR | Osteomyelitis, pe | musculoskeletal | |
| 710.1 | 0.10587 | 0.094747 | 1.117394 | 0.263826 | -0.07983 | 0.29157 | AMR | Osteomyelitis | musculoskeletal | |
| 710.11 | -0.05971 | 0.139324 | -0.42855 | 0.66825 | -0.33278 | 0.213362 | AMR | Acute osteomyeli | musculoskeletal | |
| 710.12 | 0.104056 | 0.128643 | 0.80888 | 0.418584 | -0.14808 | 0.356192 | AMR | Chronic osteomy | musculoskeletal | |
| 710.19 | 0.077917 | 0.104583 | 0.745026 | 0.456256 | -0.12706 | 0.282895 | AMR | Unspecified oste | musculoskeletal | |
| 710.2 | 1.186052 | 0.534527 | 2.218881 | 0.026495 | 0.138398 | 2.233705 | AMR | Periostitis | musculoskeletal | |
| 710.3 | 1.420006 | 1.02313 | 1.387904 | 0.165166 | -0.58529 | 3.425303 | AMR | Osteopathy resul | musculoskeletal | |
| 711 | -0.11838 | 0.138316 | -0.85585 | 0.392082 | -0.38947 | 0.152717 | AMR | Arthropathy asso | musculoskeletal | |
| 711.1 | -0.01962 | 0.157035 | -0.12493 | 0.900578 | -0.3274 | 0.288165 | AMR | Pyogenic arthritis | musculoskeletal | |
| 711.2 | -0.75433 | 0.372501 | -2.03E+00 | 0.042862 | -1.48442 | -0.02425 | AMR | Reiter's disease | musculoskeletal | |
| 711.3 | 0.401323 | 0.527138 | 7.61E-01 | 0.446463 | -0.63185 | 1.434494 | AMR | Behcet's syndron | musculoskeletal | |
| 712 | 0.894229 | 0.228341 | 3.916197 | 9.00E-05 | 0.446688 | 1.341769 | AMR | Infective connect | musculoskeletal | |
| 713 | -0.25854 | 0.216311 | -1.19521 | 0.232006 | -0.6825 | 0.165425 | AMR | Arthropathy asso | musculoskeletal | |
| 713.5 | -0.01408 | 0.234279 | -0.06008 | 0.952091 | -0.47325 | 0.445103 | AMR | Arthropathy asso | musculoskeletal | |
| 714 | 0.016825 | 0.06543 | 0.257152 | 0.797062 | -0.11141 | 0.145065 | AMR | Rheumatoid arth | musculoskeletal | |
| 714.1 | -0.00076 | 0.076377 | -0.00996 | 0.992053 | -0.15046 | 0.148935 | AMR | Rheumatoid arth | musculoskeletal | |
| 714.2 | 0.314674 | 0.272111 | 1.156418 | 0.24751 | -0.21865 | 0.848001 | AMR | Juvenile rheumat | musculoskeletal | |
| 715 | 0.040389 | 0.074063 | 0.545339 | 0.58552 | -0.10477 | 0.18555 | AMR | Other inflammatc | musculoskeletal | |
| 715.1 | 0.030018 | 0.088626 | 0.338707 | 0.734831 | -0.14368 | 0.203721 | AMR | Sacroiliitis NEC | musculoskeletal | |
| 715.2 | 0.379042 | 0.260802 | 1.453372 | 0.14612 | -0.13212 | 0.890205 | AMR | Ankylosing spond | musculoskeletal | |
| 715.3 | 0.275633 | 0.30014 | 9.18E-01 | 0.358437 | -0.31263 | 0.863898 | AMR | Spinal enthesopa | musculoskeletal | |
| 716 | 0.003209 | 0.04197 | 0.076458 | 0.939055 | -0.07905 | 0.085468 | AMR | Other arthropathi | musculoskeletal | |
| 716.1 | 0.100084 | 0.167977 | 0.595823 | 0.551293 | -0.22914 | 0.429312 | AMR | Unspecified polya | musculoskeletal | |
| 716.2 | 0.056219 | 0.28636 | 0.196322 | 0.844358 | -0.50504 | 0.617474 | AMR | Unspecified mon | musculoskeletal | |
| 716.9 | 0.018309 | 0.042598 | 0.429811 | 0.667333 | -0.06518 | 0.101799 | AMR | Arthropathy NOS | musculoskeletal | |
| 717 | -0.07041 | 0.236208 | -2.98E-01 | 0.765635 | -0.53337 | 0.392547 | AMR | Polymyalgia Rhe | musculoskeletal | |
| 720 | 0.012189 | 0.05657 | 0.215468 | 0.829403 | -0.09869 | 0.123065 | AMR | Spinal stenosis | musculoskeletal | |

| | | | | | | | | | | |
|---|---|---|---|---|---|---|---|---|---|---|
| 720.1 | 0.00239 | 0.07265 | 0.0329 | 0.973755 | -0.14 | 0.144782 | AMR | Spinal stenosis o | musculoskeletal | |
| 721 | 0.0238 | 0.042178 | 0.564264 | 0.572575 | -0.05887 | 0.106468 | AMR | Spondylosis and | musculoskeletal | |
| 721.1 | 0.02937 | 0.042648 | 0.688658 | 0.491038 | -0.05422 | 0.112957 | AMR | Spondylosis with | musculoskeletal | |
| 721.2 | -0.02112 | 0.134977 | -0.15649 | 0.87565 | -0.28567 | 0.243428 | AMR | Spondylosis with | musculoskeletal | |
| 721.8 | -0.00296 | 0.115483 | -2.56E-02 | 0.979541 | -0.2293 | 0.22338 | AMR | Other allied disor | musculoskeletal | |
| 722 | 0.017444 | 0.040242 | 4.33E-01 | 0.664672 | -0.06143 | 0.096317 | AMR | Intervertebral dis | musculoskeletal | |
| 722.1 | -0.01641 | 0.055552 | -0.29546 | 0.767643 | -0.12529 | 0.092467 | AMR | Displacement of | musculoskeletal | |
| 722.3 | 0.881659 | 0.597218 | 1.476278 | 0.139869 | -0.28887 | 2.052184 | AMR | Schmorl's nodes | musculoskeletal | |
| 722.6 | 0.028416 | 0.042992 | 0.660963 | 0.508636 | -0.05585 | 0.112679 | AMR | Degeneration of i | musculoskeletal | |
| 722.7 | 0.195246 | 0.186684 | 1.05E+00 | 0.295624 | -0.17065 | 0.56114 | AMR | Intervertebral dis | musculoskeletal | |
| 722.8 | -0.08486 | 0.130909 | -0.64823 | 0.516835 | -0.34144 | 0.171717 | AMR | Postlaminectomy | musculoskeletal | |
| 722.9 | 0.034289 | 0.094516 | 0.362785 | 0.716765 | -0.15096 | 0.219537 | AMR | Other and unspe | musculoskeletal | |
| 723 | 0.100606 | 0.082745 | 1.215865 | 0.224036 | -0.06157 | 0.262783 | AMR | Other disorders c | musculoskeletal | |
| 723.1 | 0.341922 | 0.145923 | 2.343161 | 0.019121 | 0.055917 | 0.627927 | AMR | Torticollis | musculoskeletal | |
| 724 | -0.00308 | 0.057354 | -0.05373 | 0.957149 | -0.11549 | 0.109329 | AMR | Other and unspe | musculoskeletal | |
| 724.1 | -0.05882 | 0.069766 | -8.43E-01 | 0.399208 | -0.19555 | 0.077924 | AMR | Disorders of sacr | musculoskeletal | |
| 724.2 | -0.04863 | 0.072179 | -6.74E-01 | 0.500438 | -0.1901 | 0.092834 | AMR | Disorders of cocc | musculoskeletal | |
| 724.8 | 0.085783 | 0.104956 | 0.817327 | 0.413741 | -0.11993 | 0.291492 | AMR | Other symptoms | musculoskeletal | |
| 724.9 | 0.007273 | 0.109181 | 0.066615 | 0.946888 | -0.20672 | 0.221263 | AMR | Other unspecified | musculoskeletal | |
| 726 | 0.039739 | 0.039475 | 1.006695 | 0.314081 | -0.03763 | 0.117107 | AMR | Peripheral enthes | musculoskeletal | |
| 726.1 | 0.039209 | 0.047409 | 8.27E-01 | 0.408213 | -0.05371 | 0.132128 | AMR | Enthesopathy | musculoskeletal | |
| 726.2 | -0.01875 | 0.102143 | -0.18354 | 0.854371 | -0.21895 | 0.18145 | AMR | Synoviopathy | musculoskeletal | |
| 726.3 | 0.01918 | 0.089987 | 0.213141 | 0.831217 | -0.15719 | 0.195552 | AMR | Bursitis | musculoskeletal | |
| 726.4 | 0.127754 | 0.08603 | 1.484988 | 0.137547 | -0.04086 | 0.29637 | AMR | Calcaneal spur; E | musculoskeletal | |
| 727 | 0.010996 | 0.042867 | 0.256524 | 0.797546 | -0.07302 | 0.095014 | AMR | Other disorders c | musculoskeletal | |
| 727.1 | -0.00317 | 0.05207 | -0.06089 | 0.951449 | -0.10523 | 0.098885 | AMR | Synovitis and ten | musculoskeletal | |
| 727.2 | 0.188009 | 0.198799 | 9.46E-01 | 0.34429 | -0.20163 | 0.577648 | AMR | Bursitis disorders | musculoskeletal | |
| 727.4 | 0.041356 | 0.098985 | 0.417802 | 0.676092 | -0.15265 | 0.235362 | AMR | Ganglion and cys | musculoskeletal | |
| 727.5 | 0.117655 | 0.119491 | 0.984629 | 0.324806 | -0.11654 | 0.351853 | AMR | Rupture of synov | musculoskeletal | |
| 727.6 | -0.19273 | 0.122221 | -1.57692 | 0.114814 | -0.43228 | 0.046816 | AMR | Rupture of tendo | musculoskeletal | |
| 727.7 | -0.08717 | 0.254148 | -0.34299 | 0.731605 | -0.58529 | 0.41095 | AMR | Contracture of te | musculoskeletal | |
| 727.8 | -1.52166 | 0.478952 | -3.18E+00 | 0.001488 | -2.46039 | -0.58293 | AMR | Plica syndrome | musculoskeletal | |
| 728 | 0.135751 | 0.16681 | 0.813805 | 0.415757 | -0.19119 | 0.462693 | AMR | Disorders of mus | musculoskeletal | |
| 728.1 | -0.20566 | 0.329402 | -0.62435 | 0.532397 | -0.85128 | 0.439954 | AMR | Muscular calcifica | musculoskeletal | |
| 728.2 | 0.226726 | 0.217005 | 1.044796 | 0.296117 | -0.1986 | 0.652047 | AMR | Laxity of ligamen | musculoskeletal | |
| 728.7 | -0.12779 | 0.065613 | -1.94756 | 0.051467 | -0.25638 | 0.000814 | AMR | Fasciitis | musculoskeletal | |
| 728.71 | 0.081527 | 0.178649 | 0.456352 | 0.648137 | -0.26862 | 0.431673 | AMR | Contracture of pa | musculoskeletal | |
| 729 | 0.100543 | 0.038651 | 2.60E+00 | 0.009288 | 0.024788 | 0.176298 | AMR | Other disorders c | musculoskeletal | |

| | | | | | | | | | | |
|---|---|---|---|---|---|---|---|---|---|---|
| 729.1 | 0.500829 | 0.643514 | 0.778272 | 0.436409 | -0.76044 | 1.762094 | AMR | Rheumatism, uns | musculoskeletal | |
| 729.3 | 0.161152 | 0.281958 | 0.571546 | 0.56763 | -0.39147 | 0.713778 | AMR | Panniculitis | musculoskeletal | |
| 729.7 | 0.897917 | 0.266038 | 3.375139 | 0.000738 | 0.376491 | 1.419343 | AMR | Nontraumatic cor | musculoskeletal | |
| 731 | 0.044844 | 0.102408 | 0.437897 | 0.661461 | -0.15587 | 0.245559 | AMR | Osteitis deformar | musculoskeletal | |
| 731.1 | 0.746058 | 0.477635 | 1.561983 | 0.118292 | -0.19009 | 1.682206 | AMR | Osteitis deformar | musculoskeletal | |
| 732 | -0.09884 | 0.262533 | -0.37649 | 0.706554 | -0.6134 | 0.415715 | AMR | Osteochondropat | musculoskeletal | |
| 732.1 | 0.246695 | 0.360152 | 0.684975 | 0.49336 | -0.45919 | 0.95258 | AMR | Juvenile osteoch | musculoskeletal | |
| 732.7 | -0.09935 | 0.446771 | -0.22237 | 0.824023 | -0.975 | 0.776304 | AMR | Osteochondritis c | musculoskeletal | |
| 733 | 0.039966 | 0.043315 | 0.922679 | 0.356174 | -0.04493 | 0.124861 | AMR | Other disorders c | musculoskeletal | |
| 733.2 | 0.035299 | 2.13E-01 | 0.165584 | 0.868485 | -0.38252 | 0.453118 | AMR | Cyst of bone | musculoskeletal | |
| 733.4 | 0.120377 | 0.128331 | 9.38E-01 | 0.348232 | -0.13115 | 0.371901 | AMR | Aseptic necrosis | musculoskeletal | |
| 733.6 | 0.432767 | 0.102216 | 4.23E+00 | 2.30E-05 | 0.232427 | 0.633107 | AMR | Costochondritis | musculoskeletal | |
| 733.8 | -0.15527 | 0.1469 | -1.05697 | 0.290526 | -0.44319 | 0.13265 | AMR | Malunion and no | musculoskeletal | |
| 733.9 | 0.037071 | 0.141178 | 0.262584 | 0.792871 | -0.23963 | 0.313775 | AMR | Chondromalacia | musculoskeletal | |
| 735 | 0.048274 | 0.053157 | 0.908144 | 0.363802 | -0.05591 | 0.15246 | AMR | Acquired foot def | musculoskeletal | |
| 735.1 | -0.03632 | 0.120562 | -3.01E-01 | 0.763214 | -0.27262 | 0.199976 | AMR | Flat foot | musculoskeletal | |
| 735.2 | 0.106709 | 0.099322 | 1.074377 | 0.282654 | -0.08796 | 0.301377 | AMR | Acquired toe defo | musculoskeletal | |
| 735.21 | 0.085335 | 0.112304 | 0.759853 | 0.447343 | -0.13478 | 0.305448 | AMR | Hammer toe (acc | musculoskeletal | |
| 735.23 | -0.14847 | 0.29584 | -0.50186 | 0.615764 | -0.72831 | 0.431365 | AMR | Hallux rigidus | musculoskeletal | |
| 735.3 | 0.04478 | 0.091596 | 0.488887 | 0.624922 | -0.13474 | 0.224305 | AMR | Hallux valgus (Bu | musculoskeletal | |
| 736 | -0.04009 | 0.107614 | -0.37257 | 0.709467 | -0.25101 | 0.170826 | AMR | Other acquired d | musculoskeletal | |
| 736.1 | 0.714045 | 0.29738 | 2.40E+00 | 0.016345 | 0.131191 | 1.296898 | AMR | Acquired deformi | musculoskeletal | |
| 736.2 | 0.572342 | 0.256276 | 2.23E+00 | 0.025529 | 0.070051 | 1.074633 | AMR | Acquired deformi | musculoskeletal | |
| 736.3 | 0.038021 | 0.283941 | 0.133904 | 0.893478 | -0.51849 | 0.594535 | AMR | Acquired deformi | musculoskeletal | |
| 736.4 | 0.453205 | 0.204572 | 2.215385 | 0.026734 | 0.052252 | 0.854158 | AMR | Genu valgum or v | musculoskeletal | |
| 736.5 | 0.110359 | 0.486517 | 0.226835 | 0.820552 | -0.8432 | 1.063915 | AMR | Acquired deformi | musculoskeletal | |
| 736.6 | 0.016306 | 0.211173 | 0.077217 | 0.938451 | -0.39758 | 0.430197 | AMR | Unequal leg leng | musculoskeletal | |
| 737 | 0.005699 | 0.07411 | 0.076902 | 0.938701 | -0.13955 | 0.150952 | AMR | Curvature of spin | musculoskeletal | |
| 737.1 | 0.289088 | 0.162026 | 1.78E+00 | 7.44E-02 | -0.02848 | 0.606654 | AMR | Kyphosis (acquir | musculoskeletal | |
| 737.2 | 0.28999 | 0.406189 | 0.713927 | 0.475272 | -0.50613 | 1.086106 | AMR | Lordosis (acquire | musculoskeletal | |
| 737.3 | 0.034033 | 0.08044 | 0.423089 | 0.67223 | -0.12363 | 0.191692 | AMR | Kyphoscoliosis a | musculoskeletal | |
| 738 | 0.006015 | 0.067768 | 0.088753 | 0.929278 | -0.12681 | 0.138837 | AMR | Other acquired m | musculoskeletal | |
| 738.4 | -0.00159 | 0.083551 | -1.90E-02 | 0.984838 | -0.16534 | 0.162169 | AMR | Acquired spondy | musculoskeletal | |
| 739 | 0.040156 | 0.151248 | 0.265501 | 0.790624 | -0.25628 | 0.336597 | AMR | Contracture of joi | musculoskeletal | |
| 740 | 0.052525 | 0.037167 | 1.413232 | 0.157588 | -0.02032 | 0.125371 | AMR | Osteoarthrosis | musculoskeletal | |
| 740.1 | 0.079507 | 0.040318 | 1.972012 | 0.048608 | 0.000486 | 0.158528 | AMR | Osteoarthritis; loc | musculoskeletal | |
| 740.11 | 0.075706 | 0.04034 | 1.876686 | 0.060561 | -0.00336 | 0.154772 | AMR | Osteoarthrosis, lo | musculoskeletal | |
| 740.12 | 0.113318 | 0.106345 | 1.06557 | 0.286618 | -0.09511 | 0.321752 | AMR | Osteoarthrosis, lo | musculoskeletal | |

| | | | | | | | | | | |
|---|---|---|---|---|---|---|---|---|---|---|
| 740.2 | 0.043023 | 0.079537 | 0.54092 | 0.588563 | -0.11287 | 0.198913 | AMR | Osteoarthrosis, g | musculoskeletal | |
| 740.3 | -0.10741 | 0.141823 | -0.75739 | 0.448816 | -0.38538 | 0.170552 | AMR | Osteoarthrosis in | musculoskeletal | |
| 740.9 | 0.038819 | 3.98E-02 | 0.975566 | 0.329279 | -0.03917 | 0.116807 | AMR | Osteoarthrosis N | musculoskeletal | |
| 741 | -0.01221 | 0.040874 | -0.29875 | 0.765131 | -0.09232 | 0.0679 | AMR | Symptoms and d | musculoskeletal | |
| 741.1 | 0.413862 | 0.309275 | 1.338168 | 0.180842 | -0.19231 | 1.02003 | AMR | Ankylosis of joint | musculoskeletal | |
| 741.2 | 0.05678 | 0.098902 | 5.74E-01 | 0.5659 | -0.13706 | 0.250624 | AMR | Stiffness of joint | musculoskeletal | |
| 741.3 | 0.118367 | 0.137624 | 0.860072 | 0.389749 | -0.15137 | 0.388106 | AMR | Difficulty in walki | musculoskeletal | |
| 741.4 | -0.00699 | 0.046593 | -0.15012 | 0.88067 | -0.09831 | 0.084325 | AMR | Joint effusions | musculoskeletal | |
| 741.5 | 0.374699 | 0.408221 | 0.917882 | 0.358681 | -0.4254 | 1.174798 | AMR | Hemarthrosis | musculoskeletal | |
| 741.6 | 0.457323 | 0.471504 | 0.969923 | 0.332085 | -0.46681 | 1.381453 | AMR | Villonodular sync | musculoskeletal | |
| 742 | 0.010623 | 0.086495 | 0.122816 | 0.902253 | -0.1589 | 0.18015 | AMR | Derangement of | musculoskeletal | |
| 742.2 | -0.28912 | 0.275029 | -1.05124 | 0.293149 | -0.82817 | 0.249926 | AMR | Pathological, dev | musculoskeletal | |
| 742.8 | -0.23893 | 0.140658 | -1.69867 | 0.089382 | -0.51462 | 0.036754 | AMR | Articular cartilage | musculoskeletal | |
| 742.9 | 0.15986 | 0.103512 | 1.544367 | 0.122499 | -0.04302 | 0.362739 | AMR | Other derangeme | musculoskeletal | |
| 743 | -0.02001 | 0.040238 | -0.49733 | 0.618956 | -0.09888 | 0.058853 | AMR | Osteoporosis, os | musculoskeletal | |
| 743.1 | 0.028414 | 0.052996 | 0.536164 | 0.591845 | -0.07546 | 0.132285 | AMR | Osteoporosis | musculoskeletal | |
| 743.11 | -0.00856 | 0.054462 | -0.15709 | 0.875171 | -0.1153 | 0.098187 | AMR | Osteoporosis NC | musculoskeletal | |
| 743.12 | 0.054381 | 0.088555 | 0.614092 | 0.539154 | -0.11918 | 0.227945 | AMR | Senile osteoporo | musculoskeletal | |
| 743.13 | 0.056846 | 0.094186 | 0.60355 | 0.546143 | -0.12775 | 0.241447 | AMR | Other specified o | musculoskeletal | |
| 743.2 | -0.00092 | 0.095677 | -0.0096 | 0.99234 | -0.18844 | 0.186604 | AMR | Pathologic fractu | musculoskeletal | |
| 743.21 | -0.08261 | 0.172249 | -0.47961 | 0.631508 | -0.42021 | 0.25499 | AMR | Pathologic fractu | musculoskeletal | |
| 743.22 | -0.03521 | 0.183702 | -0.19168 | 0.847994 | -0.39526 | 0.324837 | AMR | Pathologic fractu | musculoskeletal | |
| 743.4 | -0.05074 | 0.173966 | -0.29167 | 0.770536 | -0.39171 | 0.290225 | AMR | Stress fracture | musculoskeletal | |
| 743.9 | -0.05718 | 0.044064 | -1.29762 | 0.194417 | -0.14354 | 0.029186 | AMR | Osteopenia or ot | musculoskeletal | |
| 745 | 0.099647 | 0.032364 | 3.078974 | 0.002077 | 0.036215 | 0.163079 | AMR | Pain in joint | musculoskeletal | |
| 747 | -0.05829 | 0.054108 | -1.07736 | 0.281319 | -0.16434 | 0.047756 | AMR | Cardiac and circu | congenital anomalies | |
| 747.1 | -0.05944 | 6.04E-02 | -0.98429 | 0.324975 | -0.17779 | 0.058918 | AMR | Cardiac congenit | congenital anomalies | |
| 747.11 | -0.04612 | 0.076113 | -6.06E-01 | 0.544593 | -0.1953 | 0.103064 | AMR | Cardiac shunt/ he | congenital anomalies | |
| 747.12 | -0.06289 | 0.103386 | -0.60833 | 0.542971 | -0.26552 | 0.13974 | AMR | Valvular heart dis | congenital anomalies | |
| 747.13 | -0.04837 | 0.110692 | -0.43697 | 0.662133 | -0.26532 | 0.168583 | AMR | Congenital anom | congenital anomalies | |
| 747.2 | -0.11243 | 0.10768 | -1.04407 | 0.296451 | -0.32347 | 0.098623 | AMR | Congenital anom | congenital anomalies | |
| 748 | 0.447381 | 0.186281 | 2.401649 | 0.016321 | 0.082277 | 0.812485 | AMR | Anomalies of res | congenital anomalies | |
| 749 | 0.207238 | 0.142428 | 1.46E+00 | 0.145658 | -0.07192 | 0.486391 | AMR | Congenital anom | congenital anomalies | |
| 749.1 | 0.51108 | 0.339239 | 1.506551 | 0.131926 | -0.15382 | 1.175976 | AMR | Cleft palate | congenital anomalies | |
| 749.2 | 0.320666 | 0.238383 | 1.345169 | 0.178571 | -0.14656 | 0.787888 | AMR | Congenital anom | congenital anomalies | |
| 750 | -0.01735 | 0.081508 | -0.21288 | 0.831422 | -0.1771 | 0.142401 | AMR | Digestive conger | congenital anomalies | |
| 750.1 | 0.013733 | 0.114312 | 0.120132 | 0.904379 | -0.21031 | 0.23778 | AMR | Upper gastrointe: | congenital anomalies | |
| 750.11 | 0.530286 | 0.238421 | 2.224157 | 0.026138 | 0.062989 | 0.997583 | AMR | Esophageal atres | congenital anomalies | |

| | | | | | | | | | |
|---|---|---|---|---|---|---|---|---|---|
| 750.13 | 0.043173 | 0.153953 | 2.80E-01 | 0.779148 | -0.25857 | 0.344915 | AMR | Congenital anom | congenital anomalies |
| 750.14 | 0.066876 | 0.217436 | 0.307565 | 0.758414 | -0.35929 | 0.493043 | AMR | Congenital anom | congenital anomalies |
| 750.15 | 0.020997 | 0.480693 | 0.04368 | 0.96516 | -0.92115 | 0.963138 | AMR | Congenital anom | congenital anomalies |
| 750.2 | -0.04754 | 0.113695 | -0.4181 | 0.675871 | -0.27037 | 0.175302 | AMR | Lower gastrointe | congenital anomalies |
| 750.21 | -0.07075 | 0.174302 | -0.40591 | 0.684811 | -0.41237 | 0.270874 | AMR | Congenital anom | congenital anomalies |
| 750.22 | -0.13809 | 0.151416 | -9.12E-01 | 0.361756 | -0.43486 | 0.158674 | AMR | Congenital anom | congenital anomalies |
| 751 | 0.079025 | 0.070769 | 1.116671 | 0.264135 | -0.05968 | 0.21773 | AMR | Genitourinary co | congenital anomalies |
| 751.1 | 0.013744 | 0.169828 | 0.080932 | 0.935496 | -0.31911 | 0.3466 | AMR | Congenital anom | congenital anomalies |
| 751.11 | 0.03437 | 0.224079 | 0.153382 | 0.878097 | -0.40482 | 0.473557 | AMR | Congenital anom | congenital anomalies |
| 751.12 | -0.00036 | 0.254991 | -0.00141 | 0.998871 | -0.50013 | 0.499413 | AMR | Congenital anom | congenital anomalies |
| 751.2 | 0.062522 | 0.07601 | 0.822546 | 0.410766 | -0.08646 | 0.211499 | AMR | Congenital anom | congenital anomalies |
| 751.21 | 0.091954 | 0.091316 | 1.01E+00 | 0.31394 | -0.08702 | 0.270931 | AMR | Cystic kidney dis | congenital anomalies |
| 751.22 | 0.007893 | 0.1394 | 0.05662 | 0.954848 | -0.26533 | 0.281112 | AMR | Other specified c | congenital anomalies |
| 751.3 | -0.00767 | 0.240374 | -0.0319 | 0.974548 | -0.47879 | 0.463456 | AMR | Obstructive genit | congenital anomalies |
| 752 | -0.0633 | 0.117556 | -0.53847 | 0.590251 | -0.29371 | 0.167105 | AMR | Nervous system | congenital anomalies |
| 752.1 | 0.119442 | 0.179923 | 0.663852 | 0.506785 | -0.2332 | 0.472084 | AMR | Neural tube defe | congenital anomalies |
| 752.11 | 0.022829 | 0.190216 | 0.120014 | 0.904472 | -0.34999 | 0.395645 | AMR | Spina bifida | congenital anomalies |
| 752.2 | -0.03573 | 0.144793 | -0.24677 | 0.805086 | -0.31952 | 0.248058 | AMR | Other specified c | congenital anomalies |
| 753 | 0.264339 | 0.21446 | 1.232578 | 0.217733 | -0.156 | 0.684673 | AMR | Congenital anom | congenital anomalies |
| 753.1 | 0.643576 | 0.517521 | 1.243575 | 0.213656 | -0.37075 | 1.657899 | AMR | Congenital catara | congenital anomalies |
| 753.2 | 0.016793 | 0.374973 | 0.044785 | 0.964279 | -0.71814 | 0.751728 | AMR | Congenital anom | congenital anomalies |
| 754 | 0.125019 | 0.134293 | 0.930939 | 0.351885 | -0.13819 | 0.388228 | AMR | Congenital musc | congenital anomalies |
| 754.1 | 0.499266 | 0.415266 | 1.20E+00 | 0.229255 | -0.31464 | 1.313172 | AMR | Lumbosacral spo | congenital anomalies |
| 754.2 | 0.010267 | 0.170323 | 0.060279 | 0.951933 | -0.32356 | 0.344093 | AMR | Spondylolisthesis | congenital anomalies |
| 755 | 0.07139 | 0.127474 | 0.560035 | 0.575455 | -0.17845 | 0.321233 | AMR | Congenital anom | congenital anomalies |
| 755.1 | 0.176835 | 0.169931 | 1.040625 | 0.298049 | -0.15622 | 0.509894 | AMR | Congenital defor | congenital anomalies |
| 755.3 | -0.09373 | 0.372547 | -0.25159 | 0.801359 | -0.82391 | 0.63645 | AMR | Congenital anom | congenital anomalies |
| 755.4 | 0.141157 | 0.327544 | 0.430955 | 0.666501 | -0.50082 | 0.783132 | AMR | Congenital anom | congenital anomalies |
| 755.6 | -0.28366 | 0.202603 | -1.40007 | 0.161493 | -0.68075 | 0.113437 | AMR | Other congenital | congenital anomalies |
| 755.61 | -0.59843 | 0.248674 | -2.4065 | 0.016106 | -1.08583 | -0.11104 | AMR | Congenital hip dy | congenital anomalies |
| 756 | 0.09978 | 0.130889 | 0.762327 | 0.445865 | -0.15676 | 0.356317 | AMR | Other congenital | congenital anomalies |
| 756.1 | -0.04729 | 0.285739 | -0.16551 | 0.868544 | -0.60733 | 0.512746 | AMR | Congenital anom | congenital anomalies |
| 756.2 | -0.09662 | 2.08E-01 | -0.46363 | 0.642913 | -0.50506 | 0.311825 | AMR | Pectus and other | congenital anomalies |
| 756.21 | 0.009807 | 0.217244 | 4.51E-02 | 0.963995 | -0.41598 | 0.435598 | AMR | Pectus excavatu | congenital anomalies |
| 756.22 | 0.722251 | 0.685458 | 1.053676 | 0.292031 | -0.62122 | 2.065723 | AMR | Pectus carinatum | congenital anomalies |
| 756.3 | 0.430867 | 0.316194 | 1.362666 | 0.172988 | -0.18886 | 1.050596 | AMR | Congenital anom | congenital anomalies |
| 756.5 | 0.193552 | 0.289265 | 0.669117 | 0.503421 | -0.3734 | 0.760501 | AMR | Congenital osteo | congenital anomalies |
| 757 | 0.422691 | 0.242974 | 1.739655 | 0.08192 | -0.05353 | 0.898912 | AMR | Congenital anom | congenital anomalies |

| | | | | | | | | | | |
|---|---|---|---|---|---|---|---|---|---|---|
| 758 | 0.236338 | 0.126963 | 1.861467 | 0.062678 | -0.01251 | 0.485181 | AMR | Chromosomal an | congenital anomalies | |
| 758.1 | 0.188358 | 0.133126 | 1.414884 | 0.157103 | -0.07256 | 0.449281 | AMR | Chromosomal an | congenital anomalies | |
| 759 | 0.03808 | 0.103259 | 0.36878 | 0.712292 | -0.1643 | 0.240464 | AMR | Other and unspe | congenital anomalies | |
| 759.1 | 0.124887 | 0.316982 | 0.393987 | 0.693591 | -0.49639 | 0.746159 | AMR | Anomalies of enc | congenital anomalies | |
| 760 | 0.084807 | 0.032604 | 2.601139 | 0.009291 | 0.020905 | 0.148709 | AMR | Back pain | symptoms | |
| 761 | 0.117736 | 0.039292 | 2.996405 | 0.002732 | 0.040724 | 0.194747 | AMR | Cervicalgia | symptoms | |
| 763 | 0.054415 | 0.050836 | 1.07E+00 | 0.284442 | -0.04522 | 0.154053 | AMR | Thoracic or lumb | symptoms | |
| 764 | 0.045568 | 0.047959 | 0.95013 | 0.342046 | -0.04843 | 0.139566 | AMR | Sciatica | symptoms | |
| 765 | 0.039025 | 0.064993 | 0.600456 | 0.548202 | -0.08836 | 0.166409 | AMR | Cervical radiculiti | symptoms | |
| 766 | 0.022699 | 0.064278 | 0.353145 | 0.72398 | -0.10328 | 0.148681 | AMR | Neuralgia, neuriti | symptoms | |
| 767 | 0.296545 | 0.230566 | 1.286157 | 0.198388 | -0.15536 | 0.748446 | AMR | Cervicocranial/Cc | symptoms | |
| 769 | 0.537936 | 0.422804 | 1.272306 | 0.203264 | -0.29074 | 1.366616 | AMR | Nonallopathic les | symptoms | |
| 770 | 0.048974 | 0.039934 | 1.226378 | 0.220056 | -0.02929 | 0.127242 | AMR | Myalgia and myo | symptoms | |
| 771 | 0.010469 | 0.053776 | 0.194677 | 0.845646 | -0.09493 | 0.115869 | AMR | Musculoskeletal : | symptoms | |
| 771.1 | 0.099332 | 3.98E-02 | 2.495722 | 0.01257 | 0.021324 | 0.177339 | AMR | Swelling of limb | symptoms | |
| 771.2 | 0.136304 | 0.07676 | 1.775728 | 0.075778 | -0.01414 | 0.286751 | AMR | Cramp of limb | symptoms | |
| 772 | 0.079399 | 0.077081 | 1.030071 | 0.302977 | -0.07168 | 0.230475 | AMR | Symptoms of the | symptoms | |
| 772.1 | -0.01136 | 0.140153 | -8.11E-02 | 0.935399 | -0.28606 | 0.263336 | AMR | Muscular wasting | symptoms | |
| 772.2 | 0.005791 | 0.06561 | 0.088267 | 0.929664 | -0.1228 | 0.134384 | AMR | Spasm of muscle | symptoms | |
| 772.3 | 0.028158 | 0.06974 | 0.403764 | 0.686386 | -0.10853 | 0.164846 | AMR | Muscle weakness | symptoms | |
| 772.4 | 0.372107 | 0.200743 | 1.853653 | 0.063789 | -0.02134 | 0.765556 | AMR | Rhabdomyolysis | symptoms | |
| 772.6 | 0.023397 | 0.106162 | 0.220391 | 0.825566 | -0.18468 | 0.231471 | AMR | Facial weakness | symptoms | |
| 773 | 0.053456 | 0.032498 | 1.644897 | 0.099991 | -0.01024 | 0.117152 | AMR | Pain in limb | symptoms | |
| 780 | 0.000695 | 0.088234 | 0.007878 | 0.993714 | -0.17224 | 0.17363 | AMR | Hypothermia/Chi | symptoms | |
| 781 | 0.036766 | 0.046042 | 0.798546 | 0.424554 | -0.05347 | 0.127006 | AMR | Symptoms involv | symptoms | |
| 781.1 | 0.316359 | 2.24E-01 | 1.411568 | 0.158077 | -0.12291 | 0.755623 | AMR | Loss of height | symptoms | |
| 781.2 | 0.309857 | 0.163245 | 1.898104 | 0.057682 | -0.0101 | 0.629812 | AMR | Abnormal postur | symptoms | |
| 782.3 | 0.02421 | 0.039916 | 0.606509 | 0.544177 | -0.05403 | 0.102445 | AMR | Edema | symptoms | |
| 782.6 | -0.06697 | 0.091126 | -7.35E-01 | 0.462378 | -0.24557 | 0.111631 | AMR | Pallor and flushir | symptoms | |
| 783 | 0.022112 | 0.035777 | 0.618061 | 0.536535 | -0.04801 | 0.092234 | AMR | Fever of unknow | symptoms | |
| 783.1 | -0.12725 | 0.136066 | -0.93524 | 0.349664 | -0.39394 | 0.13943 | AMR | Postprocedural fe | symptoms | |
| 785 | 0.044185 | 0.031203 | 1.416081 | 0.156752 | -0.01697 | 0.105341 | AMR | Abdominal pain | symptoms | |
| 788 | 0.030604 | 0.05237 | 0.584379 | 0.558965 | -0.07204 | 0.133248 | AMR | Syncope and col | symptoms | |
| 789 | 0.044004 | 0.031969 | 1.376489 | 0.16867 | -0.01865 | 0.106662 | AMR | Nausea and vom | symptoms | |
| 789.1 | 0.132514 | 0.095318 | 1.390238 | 0.164457 | -0.0543 | 0.319333 | AMR | Persistent vomitii | symptoms | |
| 790 | 0.075691 | 0.05073 | 1.492025 | 0.135693 | -0.02374 | 0.175121 | AMR | Nonspecific findi | symptoms | |
| 790.1 | -0.00113 | 0.112234 | -0.01011 | 0.991937 | -0.22111 | 0.218841 | AMR | Elevated sedime | symptoms | |
| 790.6 | 0.055599 | 0.034708 | 1.601876 | 0.109183 | -0.01243 | 0.123626 | AMR | Other abnormal b | symptoms | |

| | | | | | | | | | |
|---|---|---|---|---|---|---|---|---|---|
| 790.8 | -0.00759 | 0.130417 | -0.05822 | 0.953571 | -0.26321 | 0.248019 | AMR | Elevated C-react | symptoms |
| 790.9 | 0.526249 | 0.26831 | 1.96E+00 | 0.049839 | 0.00037 | 1.052128 | AMR | Abnormal arterial | symptoms |
| 791 | 0.01087 | 0.097546 | 0.111434 | 0.911272 | -0.18032 | 0.202057 | AMR | Gangrene | symptoms |
| 792 | 0.008683 | 0.070101 | 0.123867 | 0.901421 | -0.12871 | 0.146079 | AMR | Abnormal Papan | genitourinary |
| 792.1 | 0.038103 | 0.084728 | 0.449705 | 0.652923 | -0.12796 | 0.204166 | AMR | Papanicolaou sm | genitourinary |
| 793 | -0.05906 | 0.132181 | -0.44678 | 0.655031 | -0.31813 | 0.200014 | AMR | Nonspecific abnc | symptoms |
| 793.2 | 0.055229 | 0.071571 | 0.771672 | 0.440309 | -0.08505 | 0.195505 | AMR | Nonspecific abnc | symptoms |
| 794 | -0.18085 | 0.163307 | -1.10745 | 0.268098 | -0.50093 | 0.139221 | AMR | Abnormal results | symptoms |
| 795 | 0.090008 | 0.104757 | 0.859207 | 0.390226 | -0.11531 | 0.295329 | AMR | Other and nonsp | symptoms |
| 795.8 | -0.07118 | 0.141957 | -0.50143 | 0.616066 | -0.34941 | 0.207048 | AMR | Abnormal tumor | symptoms |
| 795.81 | 0.505891 | 0.238547 | 2.120715 | 0.033946 | 0.038347 | 0.973435 | AMR | Elevated carcino | symptoms |
| 795.82 | -0.10273 | 0.191199 | -0.53727 | 0.59108 | -0.47747 | 0.272017 | AMR | Elevated cancer | symptoms |
| 796 | 0.016005 | 0.10175 | 1.57E-01 | 0.875014 | -0.18342 | 0.215431 | AMR | Elevated prostate | genitourinary |
| 797 | 0.0744 | 0.05562 | 1.337653 | 0.18101 | -0.03461 | 0.183413 | AMR | Shock | symptoms |
| 797.1 | -0.00374 | 0.082647 | -0.04521 | 0.963937 | -0.16572 | 0.158248 | AMR | Cardiogenic shoc | symptoms |
| 798 | 0.002662 | 0.032708 | 0.081383 | 0.935137 | -0.06144 | 0.066768 | AMR | Malaise and fatig | symptoms |
| 798.1 | -0.00765 | 0.079099 | -0.0967 | 0.922969 | -0.16268 | 0.147382 | AMR | Chronic fatigue s | symptoms |
| 800 | 0.066485 | 0.069409 | 0.957874 | 0.338126 | -0.06955 | 0.202525 | AMR | Fracture of lower | injuries & poisonings |
| 800.1 | -0.12288 | 0.139337 | -0.88188 | 0.37784 | -0.39598 | 0.150217 | AMR | Fracture of neck | injuries & poisonings |
| 800.2 | -0.02836 | 0.193724 | -0.14638 | 0.883618 | -0.40805 | 0.351334 | AMR | Fracture of unspe | injuries & poisonings |
| 800.3 | 0.101789 | 0.12467 | 0.816464 | 0.414235 | -0.14256 | 0.346138 | AMR | Fracture of tibia a | injuries & poisonings |
| 800.4 | -0.13806 | 0.267905 | -0.51532 | 0.606329 | -0.66314 | 0.387028 | AMR | Fracture of patell | injuries & poisonings |
| 801 | 0.186936 | 0.073621 | 2.539179 | 0.011111 | 0.042642 | 0.33123 | AMR | Fracture of ankle | injuries & poisonings |
| 801.1 | 0.289874 | 0.126381 | 2.29E+00 | 0.02181 | 0.042173 | 0.537576 | AMR | Fracture of foot | injuries & poisonings |
| 802 | 0.375983 | 0.196934 | 1.909177 | 0.056239 | -0.01 | 0.761967 | AMR | Fracture of pelvis | injuries & poisonings |
| 803 | 0.002641 | 0.074468 | 0.035463 | 0.97171 | -0.14331 | 0.148595 | AMR | Fracture of upper | injuries & poisonings |
| 803.1 | 0.121553 | 0.132465 | 0.917622 | 0.358817 | -0.13807 | 0.381179 | AMR | Fracture of hume | injuries & poisonings |
| 803.2 | 0.064063 | 0.115635 | 0.554012 | 0.57957 | -0.16258 | 0.290704 | AMR | Fracture of radius | injuries & poisonings |
| 803.21 | -0.06066 | 0.27808 | -0.21814 | 0.82732 | -0.60569 | 0.484367 | AMR | Colles' fracture | injuries & poisonings |
| 803.3 | 0.005675 | 0.180994 | 0.031356 | 0.974986 | -0.34907 | 0.360418 | AMR | Fracture of clavic | injuries & poisonings |
| 804 | -0.17731 | 0.102504 | -1.72975 | 0.083674 | -0.37821 | 0.023597 | AMR | Fracture of hand | injuries & poisonings |
| 805 | 0.043751 | 0.096779 | 0.452068 | 0.65122 | -0.14593 | 0.233434 | AMR | Fracture of vertel | injuries & poisonings |
| 807 | -0.16883 | 0.162914 | -1.0363 | 0.300062 | -0.48813 | 0.150478 | AMR | Fracture of ribs | injuries & poisonings |
| 809 | 0.03829 | 0.03327 | 1.150881 | 0.249781 | -0.02692 | 0.103498 | AMR | Fracture of unspe | injuries & poisonings |
| 816 | 0.125754 | 0.357032 | 3.52E-01 | 0.724672 | -0.57402 | 0.825524 | AMR | Cerebral laceratio | injuries & poisonings |
| 817 | 0.185507 | 0.134015 | 1.384226 | 0.166289 | -0.07716 | 0.448171 | AMR | Concussion | injuries & poisonings |
| 818 | -0.09032 | 0.187622 | -0.48138 | 0.630246 | -0.45805 | 0.277415 | AMR | Intracranial hemo | injuries & poisonings |
| 818.1 | 0.590758 | 0.477168 | 1.23805 | 0.215697 | -0.34447 | 1.52599 | AMR | Subdural hemorr | injuries & poisonings |

| | | | | | | | | | |
|---|---|---|---|---|---|---|---|---|---|
| 818.2 | 0.822878 | 0.30531 | 2.695218 | 0.007034 | 0.224481 | 1.421276 | AMR | Subarachnoid he | injuries & poisonings |
| 819 | -0.04383 | 0.103948 | -0.42161 | 0.673308 | -0.24756 | 0.159909 | AMR | Skull and face fra | injuries & poisonings |
| 823 | -1.54628 | 1.249803 | -1.23722 | 0.216005 | -3.99585 | 0.903285 | AMR | Torus fracture | injuries & poisonings |
| 830 | 0.002834 | 0.06283 | 0.0451 | 0.964027 | -0.12031 | 0.125978 | AMR | Dislocation | injuries & poisonings |
| 835 | 0.16387 | 0.080327 | 2.040028 | 0.041348 | 0.006431 | 0.321308 | AMR | Internal deranger | injuries & poisonings |
| 836 | 0.197437 | 0.341074 | 0.578869 | 0.562678 | -0.47106 | 0.86593 | AMR | Traumatic arthro | injuries & poisonings |
| 840 | 0.024028 | 0.04348 | 0.552614 | 0.580528 | -0.06119 | 0.109247 | AMR | Sprains and strai | injuries & poisonings |
| 840.1 | 0.104479 | 0.134561 | 7.76E-01 | 0.437484 | -0.15925 | 0.368213 | AMR | Muscle/tendon sp | injuries & poisonings |
| 840.2 | -0.01457 | 0.095348 | -0.15284 | 0.878526 | -0.20145 | 0.172305 | AMR | Rotator cuff (cap | injuries & poisonings |
| 840.3 | -0.01393 | 0.111059 | -0.12546 | 0.90016 | -0.23161 | 0.203739 | AMR | Joint/ligament sp | injuries & poisonings |
| 841 | 0.014694 | 0.053931 | 0.272461 | 0.785268 | -0.09101 | 0.120397 | AMR | Sprains and strai | injuries & poisonings |
| 842 | 0.041996 | 0.052501 | 0.799897 | 0.42377 | -0.06091 | 0.144897 | AMR | Other sprains an | injuries & poisonings |
| 850 | 0.026583 | 0.078351 | 0.339278 | 0.7344 | -0.12698 | 0.180148 | AMR | Hemorrhage or h | injuries & poisonings |
| 851 | 0.10347 | 0.054333 | 1.904374 | 0.056862 | -0.00302 | 0.209961 | AMR | Complications of | injuries & poisonings |
| 853 | -0.02326 | 0.133599 | -0.17409 | 0.861791 | -0.28511 | 0.23859 | AMR | Complication of c | injuries & poisonings |
| 854 | 0.078492 | 7.03E-02 | 1.115775 | 0.264518 | -0.05939 | 0.21637 | AMR | Complications of | injuries & poisonings |
| 855 | 0.70312 | 0.397199 | 1.770197 | 0.076694 | -0.07538 | 1.481616 | AMR | Complication of r | injuries & poisonings |
| 856 | 0.490823 | 2.04E-01 | 2.41166 | 0.01588 | 0.09193 | 0.889716 | AMR | Vascular complic | injuries & poisonings |
| 857 | -0.11456 | 0.103687 | -1.10E+00 | 0.2692 | -0.31779 | 0.088659 | AMR | Mechanical comp | injuries & poisonings |
| 858 | 0.160117 | 0.113456 | 1.411266 | 0.158166 | -0.06225 | 0.382487 | AMR | Complication of i | injuries & poisonings |
| 859 | 0.058154 | 0.076813 | 0.757093 | 0.448994 | -0.0924 | 0.208705 | AMR | Complication due | injuries & poisonings |
| 860 | -0.11109 | 0.1319 | -0.84223 | 0.399661 | -0.36961 | 0.14743 | AMR | Bone marrow or s | neoplasms |
| 870 | -0.01024 | 0.059824 | -0.17125 | 0.864028 | -0.1275 | 0.107009 | AMR | Open wounds of | injuries & poisonings |
| 870.1 | 0.021942 | 0.216273 | 1.01E-01 | 0.919188 | -0.40195 | 0.44583 | AMR | Open wound or la | injuries & poisonings |
| 870.2 | 0.756303 | 0.246635 | 3.066485 | 0.002166 | 0.272907 | 1.239699 | AMR | Open wound of e | injuries & poisonings |
| 870.3 | -0.04543 | 0.120937 | -0.37567 | 0.707162 | -0.28246 | 0.191599 | AMR | Other open wour | injuries & poisonings |
| 870.4 | -0.16731 | 0.354127 | -0.47247 | 0.636594 | -0.86139 | 0.526762 | AMR | Open wound of n | injuries & poisonings |
| 870.5 | -0.27987 | 0.18559 | -1.508 | 0.131554 | -0.64362 | 0.08388 | AMR | Open wound of li | injuries & poisonings |
| 870.6 | 0.533059 | 0.379931 | 1.403044 | 0.160604 | -0.21159 | 1.27771 | AMR | Open wound of n | injuries & poisonings |
| 870.8 | 0.790897 | 0.426739 | 1.85E+00 | 0.063832 | -0.0455 | 1.627291 | AMR | Open wound of g | injuries & poisonings |
| 871 | 0.008612 | 0.061661 | 0.139659 | 0.888929 | -0.11224 | 0.129466 | AMR | Open wounds of | injuries & poisonings |
| 871.1 | 0.004967 | 0.158835 | 0.031273 | 0.975052 | -0.30634 | 0.316278 | AMR | Open wound of h | injuries & poisonings |
| 871.2 | 0.054148 | 0.11558 | 0.468489 | 0.639435 | -0.17238 | 0.280681 | AMR | Open wound of fi | injuries & poisonings |
| 871.3 | 0.029971 | 0.163099 | 0.18376 | 0.854202 | -0.2897 | 0.34964 | AMR | Open wound of fo | injuries & poisonings |
| 871.4 | 0.105504 | 0.203601 | 5.18E-01 | 0.604323 | -0.29355 | 0.504554 | AMR | Open wound of to | injuries & poisonings |
| 872 | 0.174588 | 0.171505 | 1.017974 | 0.30869 | -0.16156 | 0.510733 | AMR | Traumatic amput | injuries & poisonings |
| 874 | 0.556587 | 0.250571 | 2.22128 | 0.026332 | 0.065478 | 1.047696 | AMR | Complication of a | injuries & poisonings |
| 875 | 0.230592 | 0.206992 | 1.114012 | 0.265274 | -0.17511 | 0.636289 | AMR | Non-healing surg | injuries & poisonings |

| | | | | | | | | | |
|---|---|---|---|---|---|---|---|---|---|
| 876 | 0.117028 | 0.130421 | 0.897311 | 0.369553 | -0.13859 | 0.372647 | AMR | Posttraumatic wo | injuries & poisonings |
| 907 | 0.133757 | 0.180154 | 0.742456 | 0.457811 | -0.21934 | 0.486853 | AMR | Injuries to the ne | injuries & poisonings |
| 910 | -0.20358 | 0.262563 | -7.75E-01 | 0.438133 | -0.71819 | 0.311036 | AMR | Superficial injury, | injuries & poisonings |
| 911 | -0.14251 | 0.147455 | -0.96643 | 0.333827 | -0.43151 | 0.146501 | AMR | Blister | injuries & poisonings |
| 912 | -0.09547 | 0.108795 | -0.87748 | 0.380229 | -0.3087 | 0.11777 | AMR | Insect bite | injuries & poisonings |
| 913 | 0.512639 | 0.22216 | 2.30752 | 0.021026 | 0.077213 | 0.948064 | AMR | Toxic effect of ve | injuries & poisonings |
| 915 | -0.02171 | 0.08689 | -0.2499 | 0.802664 | -0.19202 | 0.148588 | AMR | Superficial injury | injuries & poisonings |
| 916 | 0.009408 | 0.049531 | 1.90E-01 | 0.849361 | -0.08767 | 0.106486 | AMR | Contusion | injuries & poisonings |
| 930 | -0.06821 | 0.064821 | -1.05222 | 0.292697 | -0.19525 | 0.058841 | AMR | Allergic reaction t | injuries & poisonings |
| 931 | -0.7567 | 0.613976 | -1.23245 | 0.21778 | -1.96007 | 0.446674 | AMR | Contact dermatiti | dermatologic |
| 938 | 0.048465 | 9.30E-02 | 0.521057 | 0.602327 | -0.13384 | 0.230766 | AMR | Dermatitis due to | dermatologic |
| 938.1 | 0.159356 | 0.175865 | 0.906126 | 0.364869 | -0.18533 | 0.504044 | AMR | Acute dermatitis | dermatologic |
| 938.2 | 0.050352 | 0.122125 | 0.412298 | 0.680121 | -0.18901 | 0.289713 | AMR | Chronic dermatiti | dermatologic |
| 939 | 0.019088 | 0.042142 | 4.53E-01 | 0.650587 | -0.06351 | 0.101686 | AMR | Atopic/contact de | dermatologic |
| 939.1 | -0.06369 | 0.255437 | -0.24935 | 0.803088 | -0.56434 | 0.436952 | AMR | Contact and aller | dermatologic |
| 941 | 0.09247 | 0.069064 | 1.338912 | 0.180599 | -0.04289 | 0.227832 | AMR | Adverse reaction | injuries & poisonings |
| 942 | -0.0335 | 0.127816 | -2.62E-01 | 0.793242 | -0.28402 | 0.217013 | AMR | Infusion and tran | injuries & poisonings |
| 946 | 0.112476 | 0.131621 | 0.854542 | 0.392805 | -0.1455 | 0.370449 | AMR | Anaphylactic sho | injuries & poisonings |
| 947 | 0.058235 | 0.077602 | 7.50E-01 | 0.452997 | -0.09386 | 0.210332 | AMR | Urticaria | dermatologic |
| 949 | -0.02755 | 0.043484 | -0.63354 | 0.526378 | -0.11277 | 0.057677 | AMR | Allergies, other | injuries & poisonings |
| 949.1 | 0.106599 | 0.253418 | 0.420644 | 0.674015 | -0.39009 | 0.603288 | AMR | Diaper or napkin | injuries & poisonings |
| 952 | 0.371262 | 0.213027 | 1.742791 | 0.08137 | -0.04626 | 0.788788 | AMR | Spinal cord injury | injuries & poisonings |
| 957 | 1.022332 | 0.48378 | 2.113218 | 0.034582 | 0.074141 | 1.970522 | AMR | Injury to other an | injuries & poisonings |
| 958 | 0.126987 | 0.146692 | 0.86567 | 0.386671 | -0.16052 | 0.414499 | AMR | Certain early con | injuries & poisonings |
| 958.1 | 0.568913 | 0.22328 | 2.55E+00 | 0.010835 | 0.131292 | 1.006534 | AMR | Postoperative sh | injuries & poisonings |
| 958.2 | 0.370005 | 0.279043 | 1.325982 | 0.184846 | -0.17691 | 0.916919 | AMR | Traumatic and su | injuries & poisonings |
| 960 | -0.02673 | 0.039967 | -0.66868 | 0.503702 | -0.10506 | 0.051609 | AMR | Poisoning by anti | injuries & poisonings |
| 960.1 | 0.169284 | 0.43297 | 0.390982 | 0.69581 | -0.67932 | 1.017889 | AMR | Adverse effects c | injuries & poisonings |
| 960.2 | -0.042 | 0.050514 | -0.83145 | 0.405721 | -0.141 | 0.057006 | AMR | Allergy/adverse e | injuries & poisonings |
| 960.3 | 0.272026 | 0.691866 | 3.93E-01 | 0.694188 | -1.08401 | 1.628058 | AMR | Poisoning by anti | injuries & poisonings |
| 961 | 0.040608 | 0.093888 | 0.432513 | 0.665369 | -0.14341 | 0.224625 | AMR | Poisoning by oth | injuries & poisonings |
| 961.1 | -0.02658 | 0.070689 | -0.37597 | 0.706936 | -0.16513 | 0.111971 | AMR | Poisoning/allergy | injuries & poisonings |
| 962 | 0.176144 | 0.062564 | 2.815409 | 0.004872 | 0.05352 | 0.298768 | AMR | Poisoning by hor | injuries & poisonings |
| 962.1 | 0.176868 | 0.064543 | 2.740299 | 0.006138 | 0.050365 | 0.303371 | AMR | Adrenal cortical s | injuries & poisonings |
| 962.2 | 0.657906 | 0.268028 | 2.454617 | 0.014103 | 0.132581 | 1.183232 | AMR | Insulins and antic | injuries & poisonings |
| 962.3 | 0.633371 | 0.276887 | 2.29E+00 | 0.022169 | 0.090681 | 1.17606 | AMR | Hormones and sy | injuries & poisonings |
| 963 | -0.05317 | 0.061654 | -0.86237 | 0.388486 | -0.17401 | 0.067671 | AMR | Poisoning by prir | injuries & poisonings |
| 963.1 | -0.05636 | 0.061856 | -0.91108 | 0.362255 | -0.17759 | 0.06488 | AMR | Antineoplastic an | injuries & poisonings |

| | | | | | | | | | |
|---|---|---|---|---|---|---|---|---|---|
| 964 | 0.015278 | 0.153435 | 0.099575 | 0.920682 | -0.28545 | 0.316006 | AMR | Poisoning by age | injuries & poisonings |
| 964.1 | 0.112378 | 0.189227 | 0.593882 | 0.552591 | -0.2585 | 0.483256 | AMR | Anticoagulants ca | injuries & poisonings |
| 965 | -0.12788 | 0.061516 | -2.08E+00 | 0.037637 | -0.24845 | -0.00731 | AMR | Poisoning by ana | injuries & poisonings |
| 965.1 | 0.070837 | 0.050249 | 1.409716 | 0.158624 | -0.02765 | 0.169325 | AMR | Opiates and relat | injuries & poisonings |
| 965.2 | -0.9286 | 1.436743 | -0.64632 | 0.518071 | -3.74456 | 1.887367 | AMR | Antirheumatics ca | injuries & poisonings |
| 965.3 | 0.359721 | 0.349916 | 1.028022 | 0.303939 | -0.3261 | 1.045544 | AMR | Salicylates causi | injuries & poisonings |
| 966 | 0.867412 | 0.470535 | 1.843459 | 0.065262 | -0.05482 | 1.789644 | AMR | Poisoning by anti | injuries & poisonings |
| 967 | 0.016195 | 0.106872 | 0.151539 | 0.879551 | -0.19327 | 0.225661 | AMR | Adverse effects c | injuries & poisonings |
| 969 | 0.253369 | 0.187178 | 1.35E+00 | 0.175856 | -0.11349 | 0.620231 | AMR | Poisoning by psy | injuries & poisonings |
| 971 | -0.05338 | 0.559567 | -0.0954 | 0.923997 | -1.15011 | 1.043348 | AMR | Poisoning by dru | injuries & poisonings |
| 972 | -0.26289 | 0.192311 | -1.36698 | 0.171631 | -0.63981 | 0.114037 | AMR | Poisoning by age | injuries & poisonings |
| 972.1 | 0.23549 | 0.359645 | 0.654785 | 0.512606 | -0.4694 | 0.940382 | AMR | Cardiac rhythm re | injuries & poisonings |
| 972.2 | 0.367845 | 0.272299 | 1.350886 | 0.176732 | -0.16585 | 0.90154 | AMR | Antilipemic and a | injuries & poisonings |
| 972.6 | 0.000368 | 0.302356 | 1.22E-03 | 0.99903 | -0.59224 | 0.592974 | AMR | Antihypertensive | injuries & poisonings |
| 974 | -0.02869 | 0.331361 | -0.08658 | 0.931003 | -0.67815 | 0.620765 | AMR | Poisoning by wat | injuries & poisonings |
| 975 | 0.643179 | 0.612836 | 1.049512 | 0.293943 | -0.55796 | 1.844316 | AMR | Poisoning by age | injuries & poisonings |
| 976 | 0.428779 | 5.22E-01 | 0.822141 | 0.410997 | -0.59342 | 1.450977 | AMR | Poisoning by age | injuries & poisonings |
| 977 | 0.03841 | 0.040455 | 0.949456 | 0.342389 | -0.04088 | 0.1177 | AMR | Personal history | injuries & poisonings |
| 979 | 0.000525 | 0.049005 | 0.010706 | 0.991458 | -0.09552 | 0.096572 | AMR | Adverse drug eve | injuries & poisonings |
| 980 | -0.0281 | 0.057429 | -4.89E-01 | 0.624646 | -0.14066 | 0.084461 | AMR | Encounter for lon | infectious diseases |
| 981 | 0.5023 | 0.477998 | 1.050841 | 0.293332 | -0.43456 | 1.439159 | AMR | Toxic effect of (no | injuries & poisonings |
| 983 | 0.16926 | 0.629266 | 0.268981 | 0.787945 | -1.06408 | 1.402599 | AMR | Toxic effect of co | injuries & poisonings |
| 984 | 0.953589 | 0.640117 | 1.48971 | 0.1363 | -0.30102 | 2.208196 | AMR | Toxic effect of lea | injuries & poisonings |
| 985 | 0.554992 | 0.6821 | 0.813651 | 0.415845 | -0.7819 | 1.891884 | AMR | Toxic effect of oth | injuries & poisonings |
| 987 | -0.12616 | 1.069299 | -0.11798 | 0.906083 | -2.22195 | 1.969631 | AMR | Toxic effect of oth | injuries & poisonings |
| 988 | 0.766383 | 0.561413 | 1.365096 | 0.172223 | -0.33397 | 1.866733 | AMR | Toxic effect of no | injuries & poisonings |
| 989 | 0.135755 | 0.425916 | 0.318736 | 0.749927 | -0.69903 | 0.970535 | AMR | Toxic effect of oth | injuries & poisonings |
| 990 | -0.08841 | 0.059053 | -1.49709 | 0.134371 | -0.20415 | 0.027334 | AMR | Effects radiation | injuries & poisonings |
| 994 | 0.054807 | 0.039841 | 1.375645 | 0.168932 | -0.02328 | 0.132895 | AMR | Sepsis and SIRS | injuries & poisonings |
| 994.1 | 0.014551 | 0.086741 | 0.167754 | 0.866777 | -0.15546 | 0.18456 | AMR | Systemic inflamm | injuries & poisonings |
| 994.2 | 0.064744 | 0.04057 | 1.60E+00 | 0.110522 | -0.01477 | 0.14426 | AMR | Sepsis | injuries & poisonings |
| 994.21 | 0.127149 | 0.068308 | 1.861413 | 0.062686 | -0.00673 | 0.26103 | AMR | Septic shock | injuries & poisonings |
| 1000 | 0.229104 | 0.134613 | 1.701938 | 0.088767 | -0.03473 | 0.492941 | AMR | Burns | |
| 1001 | 0.037588 | 0.103078 | 0.364657 | 0.715367 | -0.16444 | 0.239616 | AMR | Foreign body injury | |
| 1002 | -0.05113 | 0.049929 | -1.02401 | 0.305832 | -0.14899 | 0.046731 | AMR | Symptoms concerning nutrition, me | |
| 1004 | 0.15562 | 0.085918 | 1.81E+00 | 0.070101 | -0.01278 | 0.324016 | AMR | Other signs and symptoms involvin | |
| 1005 | 0.027208 | 0.03382 | 0.80452 | 0.421097 | -0.03908 | 0.093494 | AMR | Other symptoms | |
| 1006 | 0.840808 | 0.641931 | 1.30981 | 0.19026 | -0.41735 | 2.09897 | AMR | Crushing injury | |

| | | | | | | | | | |
|---|---|---|---|---|---|---|---|---|---|
| 1007 | 0.204334 | 0.268769 | 0.76026 | 0.447099 | -0.32244 | 0.731113 | AMR | Injury to blood vessels | |
| 1008 | 0.146837 | 0.129434 | 1.13445 | 0.256606 | -0.10685 | 0.400523 | AMR | Crushing or internal injury to organ | |
| 1009 | 0.017353 | 0.060078 | 0.288844 | 0.7727 | -0.1004 | 0.135104 | AMR | Injury, NOS | |
| 1010 | 0.022395 | 0.043445 | 5.15E-01 | 0.606221 | -0.06276 | 0.107547 | AMR | Other tests | |
| 1011 | 0.028762 | 0.054466 | 0.52807 | 0.597451 | -0.07799 | 0.135513 | AMR | Complications of surgical and med | |
| 1012 | 0.528775 | 0.197549 | 2.676674 | 0.007436 | 0.141585 | 0.915964 | AMR | Late effect | |
| 1013 | 0.134379 | 0.047369 | 2.836844 | 0.004556 | 0.041537 | 0.227221 | AMR | Asphyxia and hypoxemia | |
| 1014 | 0.341071 | 0.735364 | 0.463812 | 0.642782 | -1.10022 | 1.782357 | AMR | Effects of heat, cold and air pressu | |
| 1015 | 0.05981 | 0.051939 | 1.151551 | 0.249505 | -0.04199 | 0.161609 | AMR | Effects of other external causes | |
| 1019 | -0.05947 | 0.05575 | -1.06673 | 0.286093 | -0.16874 | 0.049797 | AMR | Other ill-defined and unknown cau | |
| 1100 | 0.056033 | 0.14432 | 0.388252 | 0.697829 | -0.22683 | 0.338896 | AMR | Family history | |

| phecode | Coef. | Std.Err. | z | p_value_z | [0.025 | 0.975] | GIA | phenotype | category |
|---|---|---|---|---|---|---|---|---|---|
| 8 | 0.051805 | 0.07309 | 0.708789 | 0.478455 | -0.09145 | 0.195058 | EAS | Intestinal infectio | infectious diseases |
| 8.5 | 0.044846 | 0.090372 | 0.496239 | 0.619726 | -0.13228 | 0.221973 | EAS | Bacterial enteritis | infectious diseases |
| 8.52 | -0.08517 | 0.165009 | -0.51614 | 0.605754 | -0.40858 | 0.238244 | EAS | Intestinal infectio | infectious diseases |
| 8.6 | 0.202728 | 0.175077 | 1.157935 | 0.246891 | -0.14042 | 0.545872 | EAS | Viral Enteritis | infectious diseases |
| 10 | 0.083837 | 0.106836 | 0.784723 | 0.432616 | -0.12556 | 0.293232 | EAS | Tuberculosis | infectious diseases |
| 31 | 0.326173 | 0.197562 | 1.650989 | 0.098741 | -0.06104 | 0.713387 | EAS | Diseases due to | infectious diseases |
| 38 | -0.0128 | 0.069403 | -0.18445 | 0.85366 | -0.14883 | 0.123226 | EAS | Septicemia | infectious diseases |
| 38.1 | -0.00502 | 0.146464 | -0.03426 | 0.972669 | -0.29208 | 0.282046 | EAS | Gram negative s | infectious diseases |
| 38.2 | -0.45399 | 0.289123 | -1.57023 | 0.116362 | -1.02066 | 0.112682 | EAS | Gram positive se | infectious diseases |
| 38.3 | -0.05091 | 0.119605 | -0.42561 | 0.67039 | -0.28533 | 0.183516 | EAS | Bacteremia | infectious diseases |
| 41 | -0.04128 | 0.055376 | -0.7454 | 0.456029 | -0.14981 | 0.067258 | EAS | Bacterial infectio | infectious diseases |
| 41.1 | -0.13154 | 0.140086 | -0.93902 | 0.347718 | -0.40611 | 0.143019 | EAS | Staphylococcus i | infectious diseases |
| 41.11 | -0.05269 | 0.198266 | -0.26576 | 0.790425 | -0.44129 | 0.335903 | EAS | Methicillin sensiti | infectious diseases |
| 41.12 | -0.30139 | 0.223479 | -1.34861 | 0.177463 | -0.7394 | 0.136625 | EAS | Methicillin resista | infectious diseases |
| 41.2 | -0.17088 | 0.110466 | -1.54686 | 0.121896 | -0.38738 | 0.045633 | EAS | Streptococcus in | infectious diseases |
| 41.21 | 0.002667 | 0.394966 | 0.006752 | 0.994613 | -0.77145 | 0.776785 | EAS | Rheumatic fever | infectious diseases |
| 41.4 | 0.059211 | 0.13185 | 0.449078 | 0.653375 | -0.19921 | 0.317632 | EAS | E. coli | infectious diseases |
| 41.8 | 0.099854 | 0.100297 | 0.995576 | 0.319456 | -0.09673 | 0.296433 | EAS | H. pylori | infectious diseases |
| 41.9 | -0.07391 | 0.175741 | -0.42054 | 0.674089 | -0.41835 | 0.27054 | EAS | Infection with dru | infectious diseases |
| 53 | -0.02393 | 0.088409 | -0.27069 | 0.786628 | -0.19721 | 0.149346 | EAS | Herpes zoster | infectious diseases |
| 53.1 | 0.138772 | 0.180427 | 0.769134 | 0.441814 | -0.21486 | 0.492402 | EAS | Herpes zoster wi | infectious diseases |
| 54 | 0.031455 | 0.087004 | 0.361537 | 0.717698 | -0.13907 | 0.20198 | EAS | Herpes simplex | infectious diseases |
| 70 | -0.07975 | 0.068905 | -1.15744 | 0.247092 | -0.21481 | 0.055298 | EAS | Viral hepatitis | infectious diseases |
| 70.1 | -0.02481 | 0.341774 | -0.07259 | 0.942135 | -0.69467 | 0.645057 | EAS | Viral hepatitis A | infectious diseases |
| 70.2 | -0.03807 | 0.076693 | -0.49635 | 0.619646 | -0.18838 | 0.112249 | EAS | Viral hepatitis B | infectious diseases |
| 70.3 | -0.01512 | 0.114986 | -0.13154 | 0.89535 | -0.24049 | 0.210243 | EAS | Viral hepatitis C | infectious diseases |
| 70.4 | -0.66605 | 0.23244 | -2.86549 | 0.004164 | -1.12163 | -0.21048 | EAS | Chronic hepatitis | infectious diseases |
| 70.9 | 0.055585 | 0.100408 | 0.553593 | 0.579858 | -0.14121 | 0.252382 | EAS | Hepatitis NOS | infectious diseases |
| 71 | 0.265592 | 0.414952 | 0.640056 | 0.522136 | -0.5477 | 1.078883 | EAS | Human immunod | infectious diseases |
| 71.1 | 0.265592 | 0.414952 | 0.640056 | 0.522136 | -0.5477 | 1.078883 | EAS | HIV infection, syr | infectious diseases |
| 78 | -0.08676 | 0.073025 | -1.18806 | 0.23481 | -0.22988 | 0.056368 | EAS | Viral warts & HP\ | infectious diseases |
| 79 | 0.130518 | 0.051711 | 2.523981 | 0.011603 | 0.029166 | 0.231871 | EAS | Viral infection | infectious diseases |
| 79.1 | 0.158498 | 0.093301 | 1.698791 | 0.089359 | -0.02437 | 0.341364 | EAS | Varicella infectior | infectious diseases |
| 79.2 | -0.45886 | 0.343397 | -1.33625 | 0.181467 | -1.13191 | 0.214181 | EAS | Infectious monon | infectious diseases |
| 79.9 | 0.064618 | 0.125492 | 0.514921 | 0.606608 | -0.18134 | 0.310578 | EAS | Viremia, NOS | infectious diseases |
| 80 | -0.15441 | 0.16861 | -0.91578 | 0.359782 | -0.48488 | 0.17606 | EAS | Postoperative inf | infectious diseases |
| 81 | 0.063852 | 0.150179 | 0.42517 | 0.670713 | -0.23049 | 0.358198 | EAS | Infection/inflamm | infectious diseases |

| phecode | Coef. | Std.Err. | z | p_value_z | [0.025 | 0.975] | GIA | phenotype | category |
|---|---|---|---|---|---|---|---|---|---|
| 81.1 | -0.08986 | 0.426012 | -0.21093 | 0.832944 | -0.92483 | 0.745111 | EAS | Graft-versus-hos | infectious diseases |
| 81.11 | -0.60235 | 0.617154 | -0.97602 | 0.329054 | -1.81195 | 0.607244 | EAS | Acute graft-versu | infectious diseases |
| 81.12 | -1.17386 | 0.877599 | -1.33758 | 0.181033 | -2.89392 | 0.546201 | EAS | Chronic graft-ver | infectious diseases |
| 90 | 0.025699 | 0.18624 | 0.137989 | 0.890249 | -0.33932 | 0.390723 | EAS | Sexually transmit | infectious diseases |
| 90.2 | 0.162399 | 0.455143 | 0.356808 | 0.721235 | -0.72967 | 1.054463 | EAS | Gonococcal infec | infectious diseases |
| 90.3 | -0.49071 | 0.64899 | -0.75611 | 0.449581 | -1.76271 | 0.781286 | EAS | Venereal disease | infectious diseases |
| 110 | -0.06522 | 0.070551 | -0.92449 | 0.355233 | -0.2035 | 0.073054 | EAS | Dermatophytosis | infectious diseases |
| 110.1 | -0.06501 | 0.072934 | -0.89139 | 0.372719 | -0.20796 | 0.077935 | EAS | Dermatophytosis | infectious diseases |
| 110.11 | -0.07287 | 0.092373 | -0.78885 | 0.430201 | -0.25392 | 0.10818 | EAS | Dermatophytosis | infectious diseases |
| 110.12 | -0.10743 | 0.138402 | -0.7762 | 0.437629 | -0.37869 | 0.163835 | EAS | Althete's foot | infectious diseases |
| 110.13 | -0.1115 | 0.14442 | -0.77207 | 0.440074 | -0.39456 | 0.171556 | EAS | Dermatophytosis | infectious diseases |
| 110.2 | 0.008168 | 0.215438 | 0.037911 | 0.969758 | -0.41408 | 0.430418 | EAS | Dermatomycoses | infectious diseases |
| 112 | 0.072102 | 0.092076 | 0.783076 | 0.433582 | -0.10836 | 0.252568 | EAS | Candidiasis | infectious diseases |
| 112.3 | -0.28095 | 0.304425 | -0.92288 | 0.35607 | -0.87761 | 0.315714 | EAS | Candidiasis of sk | infectious diseases |
| 117 | -0.1015 | 0.151308 | -0.67083 | 0.502327 | -0.39806 | 0.195055 | EAS | Mycoses | infectious diseases |
| 117.1 | -0.25631 | 1.031327 | -0.24853 | 0.803726 | -2.27768 | 1.765049 | EAS | Histoplasmosis | infectious diseases |
| 117.2 | 0.66496 | 0.549608 | 1.209881 | 0.226325 | -0.41225 | 1.742171 | EAS | Coccidioidomyco | infectious diseases |
| 117.4 | -0.0843 | 0.241718 | -0.34875 | 0.727279 | -0.55806 | 0.38946 | EAS | Aspergillosis | infectious diseases |
| 130 | -0.19993 | 0.440083 | -0.4543 | 0.649614 | -1.06248 | 0.662618 | EAS | Spirochetal infec | infectious diseases |
| 130.1 | -0.12637 | 0.597309 | -0.21156 | 0.83245 | -1.29707 | 1.044337 | EAS | Lyme disease | infectious diseases |
| 131 | -6.96751 | 7.47937 | -0.93156 | 0.351562 | -21.6268 | 7.691787 | EAS | Protozoan infecti | infectious diseases |
| 132 | 0.154489 | 0.307163 | 0.502955 | 0.614996 | -0.44754 | 0.756518 | EAS | Infestation (lice, r | infectious diseases |
| 133 | -2.63133 | 1.81687 | -1.44828 | 0.14754 | -6.19233 | 0.92967 | EAS | Arthropod-borne | infectious diseases |
| 134 | 0.226979 | 0.273631 | 0.829506 | 0.406818 | -0.30933 | 0.763286 | EAS | Helminthiases | infectious diseases |
| 134.1 | 0.215349 | 0.354017 | 0.608302 | 0.542987 | -0.47851 | 0.909209 | EAS | Intestinal helmint | infectious diseases |
| 136 | -0.00487 | 0.060307 | -0.08071 | 0.935676 | -0.12307 | 0.113333 | EAS | Other infectious a | infectious diseases |
| 145 | -0.29115 | 0.184103 | -1.58147 | 0.11377 | -0.65199 | 0.069682 | EAS | Cancer of mouth | neoplasms |
| 145.1 | -1.43224 | 0.726982 | -1.97011 | 0.048826 | -2.85709 | -0.00738 | EAS | Cancer of lip | neoplasms |
| 145.2 | 0.04044 | 0.295201 | 0.136992 | 0.891037 | -0.53814 | 0.619023 | EAS | Cancer of tongue | neoplasms |
| 145.3 | -0.1652 | 0.284512 | -0.58065 | 0.561477 | -0.72283 | 0.392431 | EAS | Cancer of major s | neoplasms |
| 145.4 | -6.75297 | 21.29764 | -0.31708 | 0.751186 | -48.4956 | 34.98964 | EAS | Cancer of the gu | neoplasms |
| 145.5 | -1.13255 | 0.646233 | -1.75255 | 0.07968 | -2.39915 | 0.134039 | EAS | Cancer of the mo | neoplasms |
| 149 | -0.3303 | 0.17468 | -1.89087 | 0.058642 | -0.67266 | 0.012069 | EAS | Cancer of larynx, | neoplasms |
| 149.1 | -0.00553 | 0.552816 | -0.01001 | 0.992013 | -1.08903 | 1.077966 | EAS | Cancer of orophа | neoplasms |
| 149.2 | -0.2263 | 0.310559 | -0.7287 | 0.466187 | -0.83499 | 0.382381 | EAS | Cancer of nasopl | neoplasms |
| 149.3 | 0.535551 | 0.95731 | 0.559433 | 0.575866 | -1.34074 | 2.411843 | EAS | Cancer of hypopl | neoplasms |
| 149.4 | 0.045906 | 0.499899 | 0.091831 | 0.926832 | -0.93388 | 1.02569 | EAS | Cancer of larynx | neoplasms |

| phecode | Coef. | Std.Err. | z | p_value_z | [0.025 | 0.975] | GIA | phenotype | category | |
|---|---|---|---|---|---|---|---|---|---|---|
| 149.5 | -0.44964 | 0.203105 | -2.21385 | 0.026839 | -0.84772 | -0.05157 | EAS | Hx of malignant r | neoplasms | |
| 149.9 | -0.12748 | 0.333468 | -0.38229 | 0.702243 | -0.78107 | 0.526102 | EAS | Cancer of of nasa | neoplasms | |
| 150 | 0.490476 | 0.295274 | 1.661085 | 0.096696 | -0.08825 | 1.069203 | EAS | Cancer of esopha | neoplasms | |
| 151 | 0.049289 | 0.158784 | 0.310414 | 0.756246 | -0.26192 | 0.3605 | EAS | Cancer of stomac | neoplasms | |
| 153 | 0.112967 | 0.103428 | 1.092227 | 0.274733 | -0.08975 | 0.315682 | EAS | Colorectal cance | neoplasms | |
| 153.2 | 0.130186 | 0.123532 | 1.053867 | 0.291944 | -0.11193 | 0.372305 | EAS | Colon cancer | neoplasms | |
| 153.3 | 0.258394 | 0.132167 | 1.95506 | 0.050576 | -0.00065 | 0.517436 | EAS | Malignant neopla | neoplasms | |
| 155 | 0.015982 | 0.085772 | 0.186332 | 0.852184 | -0.15213 | 0.184093 | EAS | Cancer of liver ar | neoplasms | |
| 155.1 | 0.071002 | 0.088328 | 0.803847 | 0.421485 | -0.10212 | 0.244122 | EAS | Malignant neopla | neoplasms | |
| 157 | -0.0289 | 0.168746 | -0.17129 | 0.863994 | -0.35964 | 0.301832 | EAS | Pancreatic cance | neoplasms | |
| 158 | -0.17155 | 0.126836 | -1.35256 | 0.176195 | -0.42015 | 0.07704 | EAS | Neoplasm of uns | neoplasms | |
| 159 | 0.029242 | 0.121352 | 0.240965 | 0.809582 | -0.2086 | 0.267087 | EAS | Malignant neopla | neoplasms | |
| 159.2 | 0.015357 | 0.262042 | 0.058606 | 0.953266 | -0.49824 | 0.528951 | EAS | Malignant neopla | neoplasms | |
| 159.3 | -0.43709 | 0.252689 | -1.72974 | 0.083677 | -0.93235 | 0.058176 | EAS | Malignant neopla | neoplasms | |
| 159.4 | 0.345841 | 0.252617 | 1.369034 | 0.170989 | -0.14928 | 0.840961 | EAS | Malignant neopla | neoplasms | |
| 164 | -0.30404 | 0.343561 | -0.88496 | 0.376176 | -0.97741 | 0.369327 | EAS | Cancer of intrath | neoplasms | |
| 165 | -0.19617 | 0.101832 | -1.92643 | 0.054051 | -0.39576 | 0.003415 | EAS | Cancer within the | neoplasms | |
| 165.1 | -0.19577 | 0.104009 | -1.88228 | 0.059798 | -0.39963 | 0.00808 | EAS | Cancer of bronch | neoplasms | |
| 170 | 0.112011 | 0.124877 | 0.896976 | 0.369732 | -0.13274 | 0.356766 | EAS | Cancer of bone a | neoplasms | |
| 170.1 | 0.113288 | 0.176844 | 0.640614 | 0.521773 | -0.23332 | 0.459895 | EAS | Bone cancer | neoplasms | |
| 170.2 | 0.029645 | 0.14849 | 0.199647 | 0.841757 | -0.26139 | 0.32068 | EAS | Cancer of conne | neoplasms | |
| 172 | -0.33819 | 0.134869 | -2.50755 | 0.012157 | -0.60253 | -0.07385 | EAS | Skin cancer | neoplasms | |
| 172.1 | -0.77917 | 0.351537 | -2.21648 | 0.026659 | -1.46817 | -0.09017 | EAS | Melanomas of sk | neoplasms | |
| 172.11 | -0.77917 | 0.351537 | -2.21648 | 0.026659 | -1.46817 | -0.09017 | EAS | Melanomas of sk | neoplasms | |
| 172.2 | -0.31726 | 0.145926 | -2.17415 | 0.029694 | -0.60327 | -0.03126 | EAS | Other non-epithe | neoplasms | |
| 172.21 | -0.10738 | 0.235265 | -0.45641 | 0.648092 | -0.56849 | 0.353732 | EAS | Basal cell carcinc | neoplasms | |
| 172.22 | -0.54286 | 0.243168 | -2.23244 | 0.025586 | -1.01946 | -0.06626 | EAS | Squamous cell ca | neoplasms | |
| 172.3 | -0.3688 | 0.329828 | -1.11817 | 0.263495 | -1.01525 | 0.277648 | EAS | Carcinoma in situ | neoplasms | |
| 173 | -0.11227 | 0.079081 | -1.41969 | 0.155698 | -0.26727 | 0.042725 | EAS | Neoplasm of unc | neoplasms | |
| 174 | 0.019155 | 0.083302 | 0.229944 | 0.818136 | -0.14411 | 0.182424 | EAS | Breast cancer | neoplasms | |
| 174.1 | 0.029095 | 0.08295 | 0.35075 | 0.725776 | -0.13348 | 0.191673 | EAS | Breast cancer [fe | neoplasms | |
| 174.11 | 0.029095 | 0.08295 | 0.35075 | 0.725776 | -0.13348 | 0.191673 | EAS | Malignant neopla | neoplasms | |
| 174.2 | 0.260328 | 0.358555 | 0.726048 | 0.467809 | -0.44243 | 0.963083 | EAS | Breast cancer [m | neoplasms | |
| 174.3 | -1.02407 | 0.680918 | -1.50396 | 0.132591 | -2.35865 | 0.310501 | EAS | Neoplasm of unc | neoplasms | |
| 175 | -0.0481 | 0.102572 | -0.46898 | 0.639085 | -0.24914 | 0.152934 | EAS | Acquired absenc | neoplasms | |
| 180 | -0.28814 | 0.130509 | -2.20782 | 0.027257 | -0.54393 | -0.03235 | EAS | Cervical cancer a | neoplasms | |
| 180.1 | -0.22899 | 0.259259 | -0.88325 | 0.377101 | -0.73713 | 0.279148 | EAS | Cervical cancer | neoplasms | |

| phecode | Coef. | Std.Err. | z | p_value_z | [0.025 | 0.975] | GIA | phenotype | category | |
|---|---|---|---|---|---|---|---|---|---|---|
| 180.3 | -0.3926 | 0.142566 | -2.75381 | 0.005891 | -0.67202 | -0.11318 | EAS | Cervical intraepit | neoplasms | |
| 182 | -0.29467 | 0.184031 | -1.60121 | 0.109329 | -0.65537 | 0.066021 | EAS | Malignant neopla | neoplasms | |
| 184 | 0.097297 | 0.150607 | 0.646032 | 0.518259 | -0.19789 | 0.392482 | EAS | Cancer of other f | neoplasms | |
| 184.1 | 0.06378 | 0.16089 | 0.396418 | 0.691797 | -0.25156 | 0.379118 | EAS | Malignant neopla | neoplasms | |
| 184.11 | 0.211775 | 0.17102 | 1.238307 | 0.215602 | -0.12342 | 0.546968 | EAS | Malignant neopla | neoplasms | |
| 184.2 | 0.271062 | 0.314235 | 0.86261 | 0.388352 | -0.34483 | 0.886951 | EAS | Cancer of other f | neoplasms | |
| 185 | 0.004729 | 0.127698 | 0.037034 | 0.970458 | -0.24555 | 0.255012 | EAS | Cancer of prostat | neoplasms | |
| 187 | -0.48906 | 0.388264 | -1.25962 | 0.207808 | -1.25005 | 0.27192 | EAS | Cancer of other r | neoplasms | |
| 187.1 | -1.57336 | 0.80569 | -1.95281 | 0.050842 | -3.15248 | 0.005761 | EAS | Malignant neopla | neoplasms | |
| 187.2 | -0.10918 | 0.480835 | -0.22706 | 0.820378 | -1.0516 | 0.833241 | EAS | Malignant neopla | neoplasms | |
| 187.8 | -0.79011 | 1.08083 | -0.73102 | 0.464767 | -2.9085 | 1.32828 | EAS | Neoplasm of unc | neoplasms | |
| 189 | 0.136789 | 0.124376 | 1.099803 | 0.271418 | -0.10698 | 0.380561 | EAS | Cancer of urinary | neoplasms | |
| 189.1 | 0.109404 | 0.161171 | 0.67881 | 0.497258 | -0.20649 | 0.425294 | EAS | Cancer of kidney | neoplasms | |
| 189.11 | 0.19822 | 0.167172 | 1.185727 | 0.23573 | -0.12943 | 0.52587 | EAS | Malignant neopla | neoplasms | |
| 189.12 | -0.46304 | 0.377079 | -1.22795 | 0.219465 | -1.2021 | 0.276026 | EAS | Malignant neopla | neoplasms | |
| 189.2 | 0.233974 | 0.178864 | 1.30811 | 0.190836 | -0.11659 | 0.58454 | EAS | Cancer of bladde | neoplasms | |
| 189.21 | 0.253183 | 0.18825 | 1.344931 | 0.178648 | -0.11578 | 0.622146 | EAS | Malignant neopla | neoplasms | |
| 189.4 | 0.225264 | 0.235415 | 0.956882 | 0.338627 | -0.23614 | 0.686668 | EAS | Malignant neopla | neoplasms | |
| 190 | -0.08098 | 0.370532 | -0.21855 | 0.827003 | -0.80721 | 0.64525 | EAS | Cancer of eye | neoplasms | |
| 191 | -0.0915 | 0.127036 | -0.72023 | 0.471382 | -0.34048 | 0.15749 | EAS | Manlignant and u | neoplasms | |
| 191.1 | -0.09769 | 0.187173 | -0.52195 | 0.601706 | -0.46455 | 0.269157 | EAS | Cancer of brain a | neoplasms | |
| 191.11 | -0.13802 | 0.196291 | -0.70312 | 0.481983 | -0.52274 | 0.246708 | EAS | Cancer of brain | neoplasms | |
| 193 | 0.127247 | 0.122433 | 1.039315 | 0.298658 | -0.11272 | 0.367211 | EAS | Thyroid cancer | neoplasms | |
| 194 | -0.07579 | 0.301313 | -0.25152 | 0.801411 | -0.66635 | 0.514775 | EAS | Cancer of other e | neoplasms | |
| 195 | -0.00805 | 0.056161 | -0.14333 | 0.886031 | -0.11812 | 0.102024 | EAS | Cancer, suspecte | neoplasms | |
| 195.1 | 0.062862 | 0.057633 | 1.090732 | 0.275391 | -0.0501 | 0.175821 | EAS | Malignant neopla | neoplasms | |
| 195.3 | -0.51103 | 0.20375 | -2.50814 | 0.012137 | -0.91037 | -0.11169 | EAS | Malignant neopla | neoplasms | |
| 196 | 0.059175 | 0.046928 | 1.260974 | 0.207318 | -0.0328 | 0.151152 | EAS | Radiotherapy | neoplasms | |
| 197 | 0.054428 | 0.043242 | 1.258686 | 0.208144 | -0.03032 | 0.13918 | EAS | Chemotherapy | neoplasms | |
| 198 | 0.06763 | 0.065653 | 1.030106 | 0.30296 | -0.06105 | 0.196308 | EAS | Secondary malig | neoplasms | |
| 198.1 | 0.012399 | 0.086447 | 0.143431 | 0.88595 | -0.15703 | 0.181833 | EAS | Secondary malig | neoplasms | |
| 198.2 | -0.06866 | 0.112987 | -0.60766 | 0.543415 | -0.29011 | 0.152793 | EAS | Secondary malig | neoplasms | |
| 198.3 | 0.036358 | 0.138538 | 0.262441 | 0.792982 | -0.23517 | 0.307887 | EAS | Secondary malig | neoplasms | |
| 198.4 | 0.063063 | 0.119879 | 0.526054 | 0.598851 | -0.1719 | 0.298021 | EAS | Secondary malig | neoplasms | |
| 198.5 | 0.1004 | 0.163599 | 0.613699 | 0.539414 | -0.22025 | 0.421048 | EAS | Secondary malig | neoplasms | |
| 198.6 | -0.00378 | 0.123822 | -0.0305 | 0.97567 | -0.24646 | 0.238911 | EAS | Secondary malig | neoplasms | |
| 198.7 | 0.130786 | 0.368284 | 0.355122 | 0.722498 | -0.59104 | 0.85261 | EAS | Secondary malig | neoplasms | |

| phecode | Coef. | Std.Err. | z | p_value_z | [0.025 | 0.975] | GIA | phenotype | category | |
|---|---|---|---|---|---|---|---|---|---|---|
| 199 | 0.076844 | 0.066357 | 1.158036 | 0.246849 | -0.05321 | 0.206902 | EAS | Neoplasm of unc | neoplasms | |
| 199.4 | -0.21559 | 0.351274 | -0.61375 | 0.539383 | -0.90408 | 0.472892 | EAS | Neurofibromatosi | neoplasms | |
| 200 | -0.08476 | 0.109211 | -0.77613 | 0.43767 | -0.29881 | 0.129287 | EAS | Myeloproliferative | neoplasms | |
| 200.1 | -0.09522 | 0.227982 | -0.41765 | 0.676206 | -0.54205 | 0.351621 | EAS | Polycythemia ver | neoplasms | |
| 201 | 0.552545 | 0.49587 | 1.114293 | 0.265153 | -0.41934 | 1.524433 | EAS | Hodgkin's diseas | neoplasms | |
| 202 | 0.057922 | 0.142192 | 0.407352 | 0.683749 | -0.22077 | 0.336615 | EAS | Cancer of other l | neoplasms | |
| 202.2 | 0.035344 | 0.149339 | 0.236671 | 0.812912 | -0.25735 | 0.328043 | EAS | Non-Hodgkins ly | neoplasms | |
| 202.21 | 0.390193 | 0.290261 | 1.344283 | 0.178857 | -0.17871 | 0.959095 | EAS | Nodular lymphon | neoplasms | |
| 202.22 | -0.39603 | 0.243133 | -1.62884 | 0.103346 | -0.87256 | 0.080506 | EAS | Reticulosarcoma | neoplasms | |
| 202.23 | 0.8769 | 0.625999 | 1.4008 | 0.161274 | -0.35004 | 2.103836 | EAS | Lymphosarcoma | neoplasms | |
| 202.24 | -0.8693 | 0.608185 | -1.42933 | 0.152909 | -2.06132 | 0.322722 | EAS | Large cell lymph | neoplasms | |
| 204 | 0.336074 | 0.176964 | 1.899109 | 0.05755 | -0.01077 | 0.682917 | EAS | Leukemia | neoplasms | |
| 204.1 | 0.196141 | 0.361307 | 0.542864 | 0.587223 | -0.51201 | 0.904289 | EAS | Lymphoid leuken | neoplasms | |
| 204.11 | 0.229272 | 0.499252 | 0.459231 | 0.646068 | -0.74924 | 1.207787 | EAS | Lymphoid leuken | neoplasms | |
| 204.12 | 0.286268 | 0.700413 | 0.408713 | 0.682751 | -1.08652 | 1.659052 | EAS | Lymphoid leuken | neoplasms | |
| 204.2 | 0.235405 | 0.280905 | 0.838024 | 0.402017 | -0.31516 | 0.78597 | EAS | Myeloid leukemia | neoplasms | |
| 204.21 | 0.29259 | 0.29681 | 0.985782 | 0.32424 | -0.28915 | 0.874328 | EAS | Myeloid leukemia | neoplasms | |
| 204.22 | -1.17951 | 1.167661 | -1.01015 | 0.312424 | -3.46809 | 1.109061 | EAS | Myeloid leukemia | neoplasms | |
| 204.3 | -1.17951 | 1.167661 | -1.01015 | 0.312424 | -3.46809 | 1.109061 | EAS | Monocytic leuker | neoplasms | |
| 204.4 | 0.998794 | 0.236421 | 4.224636 | 2.39E-05 | 0.535417 | 1.462172 | EAS | Multiple myeloma | neoplasms | |
| 208 | 0.0443 | 0.050738 | 0.873101 | 0.382608 | -0.05515 | 0.143744 | EAS | Benign neoplasm | neoplasms | |
| 209 | -0.47991 | 0.284267 | -1.68824 | 0.091365 | -1.03707 | 0.077242 | EAS | Neuroendocrine t | neoplasms | |
| 210 | 0.350712 | 0.287496 | 1.219885 | 0.222508 | -0.21277 | 0.914192 | EAS | Benign neoplasm | neoplasms | |
| 211 | -0.05977 | 0.085832 | -0.69637 | 0.4862 | -0.228 | 0.108458 | EAS | Benign neoplasm | neoplasms | |
| 212 | -0.04059 | 0.249094 | -0.16295 | 0.870557 | -0.52881 | 0.447626 | EAS | Benign neoplasm | neoplasms | |
| 213 | 0.346444 | 0.236346 | 1.465835 | 0.142693 | -0.11679 | 0.809673 | EAS | Benign neoplasm | neoplasms | |
| 214 | 0.009029 | 0.108205 | 0.083442 | 0.9335 | -0.20305 | 0.221108 | EAS | Lipoma | neoplasms | |
| 214.1 | 0.044856 | 0.136144 | 0.329475 | 0.741796 | -0.22198 | 0.311692 | EAS | Lipoma of skin ar | neoplasms | |
| 215 | 0.026077 | 0.085909 | 0.303546 | 0.761474 | -0.1423 | 0.194457 | EAS | Other benign nec | neoplasms | |
| 216 | 0.027449 | 0.055328 | 0.496111 | 0.619816 | -0.08099 | 0.135891 | EAS | Benign neoplasm | neoplasms | |
| 216.1 | 0.059066 | 0.075733 | 0.779924 | 0.435436 | -0.08937 | 0.207501 | EAS | Screening for ma | neoplasms | |
| 217 | -0.13852 | 0.114075 | -1.21431 | 0.22463 | -0.3621 | 0.085061 | EAS | Vascular hamartc | neoplasms | |
| 217.1 | -0.12605 | 0.120532 | -1.04582 | 0.295645 | -0.36229 | 0.110184 | EAS | Nevus, non-neop | neoplasms | |
| 218 | 0.036894 | 0.069897 | 0.527825 | 0.597621 | -0.1001 | 0.17389 | EAS | Benign neoplasm | neoplasms | |
| 218.1 | 0.034505 | 0.069952 | 0.493266 | 0.621825 | -0.1026 | 0.171609 | EAS | Uterine leiomyom | neoplasms | |
| 218.2 | -0.55159 | 0.483733 | -1.14028 | 0.254169 | -1.49969 | 0.396508 | EAS | Other benign nec | neoplasms | |
| 220 | -0.51946 | 0.219154 | -2.37029 | 0.017774 | -0.94899 | -0.08992 | EAS | Benign neoplasm | neoplasms | |

| phecode | Coef. | Std.Err. | z | p_value_z | [0.025 | 0.975] | GIA | phenotype | category |
|---|---|---|---|---|---|---|---|---|---|
| 221 | -0.22953 | 0.602437 | -0.381 | 0.703203 | -1.41028 | 0.951226 | EAS | Benign neoplasm | neoplasms |
| 223 | 0.197339 | 0.224397 | 0.879419 | 0.379174 | -0.24247 | 0.63715 | EAS | Benign neoplasm | neoplasms |
| 224 | -0.2655 | 0.229189 | -1.15842 | 0.246691 | -0.7147 | 0.183705 | EAS | Benign neoplasm | neoplasms |
| 224.1 | -0.14443 | 0.236245 | -0.61136 | 0.54096 | -0.60746 | 0.318601 | EAS | Benign neoplasm | neoplasms |
| 225 | 0.076025 | 0.122455 | 0.620838 | 0.534707 | -0.16398 | 0.316033 | EAS | Benign neoplasm | neoplasms |
| 225.1 | 0.029609 | 0.129244 | 0.229093 | 0.818797 | -0.2237 | 0.282921 | EAS | Benign neoplasm | neoplasms |
| 225.2 | 0.373913 | 0.317852 | 1.176376 | 0.239445 | -0.24906 | 0.996891 | EAS | Benign neoplasm | neoplasms |
| 226 | -0.13911 | 0.336863 | -0.41297 | 0.679628 | -0.79935 | 0.521125 | EAS | Benign neoplasm | neoplasms |
| 227 | -0.08394 | 0.123113 | -0.68178 | 0.495376 | -0.32523 | 0.15736 | EAS | Benign neoplasm | neoplasms |
| 227.1 | -0.14254 | 0.222345 | -0.64106 | 0.521481 | -0.57832 | 0.29325 | EAS | Benign neoplasm | neoplasms |
| 227.2 | -0.24242 | 0.231366 | -1.04779 | 0.294737 | -0.69589 | 0.211046 | EAS | Benign neoplasm | neoplasms |
| 227.3 | 0.065789 | 0.195167 | 0.337093 | 0.736047 | -0.31673 | 0.448309 | EAS | Benign neoplasm | neoplasms |
| 228 | -0.03664 | 0.077198 | -0.47456 | 0.635099 | -0.18794 | 0.11467 | EAS | Hemangioma and | neoplasms |
| 228.1 | -0.02867 | 0.097999 | -0.29254 | 0.769872 | -0.22074 | 0.163406 | EAS | Hemangioma of s | neoplasms |
| 229 | -0.1897 | 0.136399 | -1.39079 | 0.164288 | -0.45704 | 0.077634 | EAS | Benign neoplasm | neoplasms |
| 229.1 | -2.69788 | 1.717745 | -1.5706 | 0.116277 | -6.0646 | 0.668837 | EAS | Benign neoplasm | neoplasms |
| 240 | 0.019496 | 0.110264 | 0.176814 | 0.859655 | -0.19662 | 0.235609 | EAS | Simple and unsp | endocrine/metabolic |
| 241 | 0.050949 | 0.058738 | 0.86738 | 0.385734 | -0.06418 | 0.166074 | EAS | Nontoxic nodular | endocrine/metabolic |
| 241.1 | 0.025838 | 0.065043 | 0.397239 | 0.691191 | -0.10164 | 0.153319 | EAS | Nontoxic uninodu | endocrine/metabolic |
| 241.2 | 0.019882 | 0.079638 | 0.249649 | 0.802859 | -0.13621 | 0.17597 | EAS | Nontoxic multino | endocrine/metabolic |
| 242 | -0.07442 | 0.095996 | -0.77523 | 0.438205 | -0.26257 | 0.113731 | EAS | Thyrotoxicosis wi | endocrine/metabolic |
| 242.1 | -0.01455 | 0.150027 | -0.097 | 0.92273 | -0.3086 | 0.279495 | EAS | Graves' disease | endocrine/metabolic |
| 242.2 | -0.45517 | 0.705 | -0.64564 | 0.518515 | -1.83695 | 0.926601 | EAS | Toxic multinodula | endocrine/metabolic |
| 242.3 | 0.113178 | 0.205557 | 0.550589 | 0.581915 | -0.28971 | 0.516063 | EAS | Exophthalmos | endocrine/metabolic |
| 242.31 | 0.609264 | 0.372811 | 1.634244 | 0.102208 | -0.12143 | 1.33996 | EAS | Thyrotoxic exoph | endocrine/metabolic |
| 244 | 0.017211 | 0.056872 | 0.302623 | 0.762177 | -0.09426 | 0.128679 | EAS | Hypothyroidism | endocrine/metabolic |
| 244.1 | -0.02514 | 0.096997 | -0.25921 | 0.79547 | -0.21525 | 0.164967 | EAS | Secondary hypot | endocrine/metabolic |
| 244.2 | -0.0897 | 0.088354 | -1.01524 | 0.309992 | -0.26287 | 0.08347 | EAS | Acquired hypothy | endocrine/metabolic |
| 244.3 | -1.65657 | 0.852077 | -1.94415 | 0.051877 | -3.32661 | 0.013473 | EAS | Iodine hypothyroi | endocrine/metabolic |
| 244.4 | -0.00814 | 0.059205 | -0.13747 | 0.890658 | -0.12418 | 0.107901 | EAS | Hypothyroidism N | endocrine/metabolic |
| 244.5 | -0.23056 | 1.005688 | -0.22925 | 0.818672 | -2.20167 | 1.740556 | EAS | Congenital hypot | endocrine/metabolic |
| 245 | 0.041161 | 0.105434 | 0.390393 | 0.696246 | -0.16549 | 0.247809 | EAS | Thyroiditis | endocrine/metabolic |
| 245.1 | 0.16683 | 0.324905 | 0.513475 | 0.607619 | -0.46997 | 0.803632 | EAS | Thyroiditis, acute | endocrine/metabolic |
| 245.2 | 0.023988 | 0.112506 | 0.213215 | 0.83116 | -0.19652 | 0.244495 | EAS | Chronic thyroiditi | endocrine/metabolic |
| 245.21 | 0.019319 | 0.115159 | 0.167756 | 0.866775 | -0.20639 | 0.245026 | EAS | Chronic lymphoc | endocrine/metabolic |
| 246 | -0.01331 | 0.07214 | -0.18455 | 0.853583 | -0.1547 | 0.128078 | EAS | Other disorders c | endocrine/metabolic |
| 246.2 | -0.00212 | 0.163048 | -0.01299 | 0.989636 | -0.32169 | 0.31745 | EAS | Thyroid cyst | endocrine/metabolic |

| phecode | Coef. | Std.Err. | z | p_value_z | [0.025 | 0.975] | GIA | phenotype | category |
|---|---|---|---|---|---|---|---|---|---|
| 246.7 | -0.0297 | 0.098739 | -0.30082 | 0.76355 | -0.22323 | 0.163823 | EAS | Abnormal results | endocrine/metabolic |
| 249 | -0.00227 | 0.084602 | -0.02679 | 0.978629 | -0.16808 | 0.16355 | EAS | Secondary diabe | endocrine/metabolic |
| 250 | -0.04152 | 0.048324 | -0.85927 | 0.39019 | -0.13624 | 0.05319 | EAS | Diabetes mellitus | endocrine/metabolic |
| 250.1 | -0.07464 | 0.128632 | -0.58027 | 0.561734 | -0.32676 | 0.177474 | EAS | Type 1 diabetes | endocrine/metabolic |
| 250.11 | 0.278018 | 0.922193 | 0.301475 | 0.763052 | -1.52945 | 2.085483 | EAS | Type 1 diabetes v | endocrine/metabolic |
| 250.12 | 0.508322 | 0.291834 | 1.741819 | 0.08154 | -0.06366 | 1.080305 | EAS | Type 1 diabetes v | endocrine/metabolic |
| 250.13 | 0.354612 | 0.375843 | 0.94351 | 0.34542 | -0.38203 | 1.091252 | EAS | Type 1 diabetes v | endocrine/metabolic |
| 250.14 | 0.188512 | 0.435404 | 0.432959 | 0.665044 | -0.66486 | 1.041889 | EAS | Type 1 diabetes v | endocrine/metabolic |
| 250.15 | -0.04931 | 1.251105 | -0.03941 | 0.96856 | -2.50143 | 2.402809 | EAS | Diabetes type 1 v | endocrine/metabolic |
| 250.2 | -0.03219 | 0.04877 | -0.65998 | 0.509264 | -0.12777 | 0.0634 | EAS | Type 2 diabetes | endocrine/metabolic |
| 250.21 | 0.177248 | 0.318614 | 0.556309 | 0.578 | -0.44722 | 0.801719 | EAS | Type 2 diabetes v | endocrine/metabolic |
| 250.22 | -0.03861 | 0.073548 | -0.52501 | 0.599577 | -0.18276 | 0.105538 | EAS | Type 2 diabetes v | endocrine/metabolic |
| 250.23 | 0.085123 | 0.094421 | 0.901529 | 0.367307 | -0.09994 | 0.270186 | EAS | Type 2 diabetes v | endocrine/metabolic |
| 250.24 | 0.029101 | 0.084666 | 0.343717 | 0.731059 | -0.13684 | 0.195045 | EAS | Type 2 diabetes v | endocrine/metabolic |
| 250.25 | 0.049504 | 0.089272 | 0.554528 | 0.579218 | -0.12547 | 0.224473 | EAS | Diabetes type 2 v | endocrine/metabolic |
| 250.3 | -0.05779 | 0.074764 | -0.77298 | 0.439532 | -0.20433 | 0.088743 | EAS | Insulin pump use | endocrine/metabolic |
| 250.4 | 0.011945 | 0.043613 | 0.273889 | 0.78417 | -0.07353 | 0.097425 | EAS | Abnormal glucos | endocrine/metabolic |
| 250.41 | 0.079361 | 0.06258 | 1.268144 | 0.204747 | -0.04329 | 0.202015 | EAS | Impaired fasting g | endocrine/metabolic |
| 250.42 | -0.01399 | 0.043668 | -0.32039 | 0.748671 | -0.09958 | 0.071597 | EAS | Other abnormal g | endocrine/metabolic |
| 250.5 | -0.62726 | 0.371953 | -1.68639 | 0.091721 | -1.35627 | 0.101758 | EAS | Glycosuria or Ac | endocrine/metabolic |
| 250.6 | 0.100387 | 0.101142 | 0.99254 | 0.320934 | -0.09785 | 0.298622 | EAS | Polyneuropathy i | endocrine/metabolic |
| 250.7 | 0.183577 | 0.10991 | 1.670258 | 0.094868 | -0.03184 | 0.398996 | EAS | Diabetic retinopa | endocrine/metabolic |
| 251 | -0.2669 | 0.479253 | -0.55691 | 0.577587 | -1.20622 | 0.672417 | EAS | Other disorders o | endocrine/metabolic |
| 251.1 | -0.03743 | 0.139188 | -0.26894 | 0.787973 | -0.31024 | 0.235369 | EAS | Hypoglycemia | endocrine/metabolic |
| 251.8 | -0.5386 | 0.585372 | -0.92009 | 0.357524 | -1.68591 | 0.608712 | EAS | Abnormality of se | endocrine/metabolic |
| 252 | -0.06287 | 0.103838 | -0.60545 | 0.544881 | -0.26639 | 0.140651 | EAS | Disorders of para | endocrine/metabolic |
| 252.1 | -0.10322 | 0.110247 | -0.93624 | 0.349149 | -0.3193 | 0.112862 | EAS | Hyperparathyroic | endocrine/metabolic |
| 252.2 | 0.00454 | 0.207027 | 0.021928 | 0.982505 | -0.40123 | 0.410306 | EAS | Hypoparathyroidi | endocrine/metabolic |
| 253 | 0.068682 | 0.111779 | 0.614443 | 0.538923 | -0.1504 | 0.287766 | EAS | Disorders of the p | endocrine/metabolic |
| 253.1 | 0.024968 | 0.202471 | 0.123319 | 0.901855 | -0.37187 | 0.421805 | EAS | Pituitary hyperfur | endocrine/metabolic |
| 253.11 | -0.48225 | 0.775764 | -0.62164 | 0.534179 | -2.00271 | 1.038224 | EAS | Acromegaly and | endocrine/metabolic |
| 253.2 | 0.238604 | 0.403706 | 0.591035 | 0.554497 | -0.55264 | 1.029854 | EAS | Pituitary hypofun | endocrine/metabolic |
| 253.3 | -0.07944 | 0.319366 | -0.24873 | 0.803567 | -0.70538 | 0.546508 | EAS | Diabetes insipidu | endocrine/metabolic |
| 253.4 | -0.02914 | 0.503533 | -0.05786 | 0.953859 | -1.01604 | 0.957771 | EAS | Anterior pituitary | endocrine/metabolic |
| 253.7 | 0.081383 | 0.167006 | 0.487303 | 0.626044 | -0.24594 | 0.408709 | EAS | Other disorders o | endocrine/metabolic |
| 254 | -0.15463 | 0.725938 | -0.21301 | 0.831321 | -1.57744 | 1.268183 | EAS | Diseases of thym | endocrine/metabolic |
| 255 | 0.052739 | 0.105268 | 0.500996 | 0.616374 | -0.15358 | 0.25906 | EAS | Disorders of adre | endocrine/metabolic |

| phecode | Coef. | Std.Err. | z | p_value_z | [0.025 | 0.975] | GIA | phenotype | category |
|---|---|---|---|---|---|---|---|---|---|
| 255.1 | 0.537748 | 0.227795 | 2.360668 | 0.018242 | 0.091278 | 0.984218 | EAS | Adrenal hyperfun | endocrine/metabolic |
| 255.11 | 0.654239 | 0.35977 | 1.818495 | 0.068988 | -0.0509 | 1.359375 | EAS | Cushing's syndro | endocrine/metabolic |
| 255.12 | 0.515207 | 0.286621 | 1.797519 | 0.072253 | -0.04656 | 1.076974 | EAS | Hyperaldosteroni | endocrine/metabolic |
| 255.2 | 0.05444 | 0.173965 | 0.312936 | 0.754329 | -0.28653 | 0.395406 | EAS | Adrenal hypofun | endocrine/metabolic |
| 255.21 | 0.05444 | 0.173965 | 0.312936 | 0.754329 | -0.28653 | 0.395406 | EAS | Glucocorticoid de | endocrine/metabolic |
| 255.22 | -0.76043 | 1.144924 | -0.66418 | 0.506577 | -3.00444 | 1.483577 | EAS | Mineralocorticoid | endocrine/metabolic |
| 255.3 | -0.01148 | 1.130705 | -0.01015 | 0.9919 | -2.22762 | 2.204663 | EAS | Adrenogenital dis | endocrine/metabolic |
| 256 | -0.12565 | 0.16149 | -0.77809 | 0.436513 | -0.44217 | 0.19086 | EAS | Ovarian dysfunct | endocrine/metabolic |
| 256.1 | -2.15004 | 1.44482 | -1.4881 | 0.136724 | -4.98184 | 0.681754 | EAS | Hyperestrogenisı | endocrine/metabolic |
| 256.4 | -0.2721 | 0.175002 | -1.55487 | 0.119977 | -0.6151 | 0.070892 | EAS | Polycystic ovarie | endocrine/metabolic |
| 257 | 0.075734 | 0.17483 | 0.433184 | 0.664881 | -0.26693 | 0.418395 | EAS | Testicular dysfun | endocrine/metabolic |
| 257.1 | 0.075734 | 0.17483 | 0.433184 | 0.664881 | -0.26693 | 0.418395 | EAS | Testicular hypofu | endocrine/metabolic |
| 258 | -0.16407 | 0.368696 | -0.44499 | 0.656324 | -0.8867 | 0.558564 | EAS | Iatrogenic endoc | endocrine/metabolic |
| 258.1 | 0.045664 | 0.382104 | 0.119507 | 0.904874 | -0.70325 | 0.794574 | EAS | Postablative ova | endocrine/metabolic |
| 259 | -0.02808 | 0.147985 | -0.18976 | 0.849499 | -0.31813 | 0.261963 | EAS | Other endocrine | endocrine/metabolic |
| 259.2 | -0.04326 | 1.011954 | -0.04275 | 0.965904 | -2.02665 | 1.940137 | EAS | Carcinoid syndro | endocrine/metabolic |
| 259.8 | -0.85528 | 0.789302 | -1.08359 | 0.278548 | -2.40228 | 0.691725 | EAS | Polyglandular ac | endocrine/metabolic |
| 260 | -0.03098 | 0.071958 | -0.43048 | 0.666846 | -0.17201 | 0.110058 | EAS | Protein-calorie m | endocrine/metabolic |
| 260.1 | -0.16124 | 0.152239 | -1.05911 | 0.289551 | -0.45962 | 0.137145 | EAS | Cachexia | endocrine/metabolic |
| 260.2 | -0.08481 | 0.110424 | -0.76803 | 0.442469 | -0.30123 | 0.131618 | EAS | severe protein-ca | endocrine/metabolic |
| 260.21 | -0.5345 | 0.70633 | -0.75673 | 0.449213 | -1.91888 | 0.849881 | EAS | Kwashiorkor | endocrine/metabolic |
| 260.22 | -0.88867 | 0.357099 | -2.48858 | 0.012825 | -1.58857 | -0.18877 | EAS | Nutritional maras | endocrine/metabolic |
| 260.3 | 0.235591 | 0.141744 | 1.662092 | 0.096494 | -0.04222 | 0.513403 | EAS | Adult failure to th | endocrine/metabolic |
| 260.6 | -0.18352 | 0.103893 | -1.7664 | 0.077329 | -0.38714 | 0.02011 | EAS | Anorexia | endocrine/metabolic |
| 260.7 | -0.82799 | 0.610433 | -1.3564 | 0.174972 | -2.02442 | 0.368436 | EAS | Polyphagia | endocrine/metabolic |
| 261 | -0.09836 | 0.046391 | -2.12027 | 0.033983 | -0.18929 | -0.00744 | EAS | Vitamin deficienc | endocrine/metabolic |
| 261.1 | -0.43345 | 0.719003 | -0.60284 | 0.546614 | -1.84267 | 0.975775 | EAS | Vitamin A deficie | endocrine/metabolic |
| 261.2 | -0.10793 | 0.093896 | -1.14948 | 0.250358 | -0.29196 | 0.076101 | EAS | Vitamin B-comple | endocrine/metabolic |
| 261.3 | -0.46711 | 1.055527 | -0.44254 | 0.658098 | -2.53591 | 1.601681 | EAS | Vitamin C deficie | endocrine/metabolic |
| 261.4 | -0.0801 | 0.047669 | -1.6804 | 0.09288 | -0.17353 | 0.013327 | EAS | Vitamin D deficie | endocrine/metabolic |
| 261.41 | 0.332481 | 0.323721 | 1.027062 | 0.304391 | -0.302 | 0.966962 | EAS | Rickets or osteor | endocrine/metabolic |
| 262 | 0.000917 | 0.129676 | 0.00707 | 0.994359 | -0.25324 | 0.255078 | EAS | Mineral deficienc | endocrine/metabolic |
| 263 | -0.03507 | 0.092397 | -0.37961 | 0.704234 | -0.21617 | 0.14602 | EAS | Other nutritional | endocrine/metabolic |
| 264 | 0.296882 | 0.281514 | 1.05459 | 0.291613 | -0.25488 | 0.84864 | EAS | Lack of normal p | endocrine/metabolic |
| 264.2 | 0.401722 | 0.288942 | 1.390321 | 0.164431 | -0.16459 | 0.968039 | EAS | Failure to thrive ( | endocrine/metabolic |
| 264.3 | -1.03047 | 1.158431 | -0.88954 | 0.373714 | -3.30095 | 1.240014 | EAS | Delayed milestor | endocrine/metabolic |
| 264.9 | 0.449145 | 0.698747 | 0.642787 | 0.520363 | -0.92037 | 1.818664 | EAS | Lack of normal p | endocrine/metabolic |

| phecode | Coef. | Std.Err. | z | p_value_z | [0.025 | 0.975] | GIA | phenotype | category |
|---|---|---|---|---|---|---|---|---|---|
| 269 | 0.038712 | 0.076053 | 0.509011 | 0.610745 | -0.11035 | 0.187772 | EAS | Proteinuria | endocrine/metabolic |
| 270 | 0.007738 | 0.116355 | 0.066499 | 0.94698 | -0.22031 | 0.235788 | EAS | Disorders of prot | endocrine/metabolic |
| 270.1 | 0.036534 | 0.305749 | 0.119489 | 0.904888 | -0.56272 | 0.635791 | EAS | Disturbances of a | endocrine/metabolic |
| 270.11 | 0.09292 | 0.348941 | 0.266292 | 0.790014 | -0.59099 | 0.776832 | EAS | Disturbances of s | endocrine/metabolic |
| 270.12 | 0.576542 | 1.090025 | 0.528925 | 0.596857 | -1.55987 | 2.712952 | EAS | Phenylketonuria | endocrine/metabolic |
| 270.2 | -0.15471 | 0.571886 | -0.27052 | 0.78676 | -1.27558 | 0.96617 | EAS | Disorders of amir | endocrine/metabolic |
| 270.21 | -0.15471 | 0.571886 | -0.27052 | 0.78676 | -1.27558 | 0.96617 | EAS | Disorders of urea | endocrine/metabolic |
| 270.3 | -0.02035 | 0.12614 | -0.16136 | 0.871806 | -0.26758 | 0.226875 | EAS | Disorders of plas | endocrine/metabolic |
| 270.31 | -1.31572 | 0.507294 | -2.5936 | 0.009498 | -2.31 | -0.32144 | EAS | Polyclonal hyper | endocrine/metabolic |
| 270.32 | 0.303218 | 0.214184 | 1.415692 | 0.156866 | -0.11657 | 0.72301 | EAS | Paraproteinemia | endocrine/metabolic |
| 270.33 | 0.249198 | 0.26891 | 0.926698 | 0.354083 | -0.27786 | 0.776253 | EAS | Amyloidosis | endocrine/metabolic |
| 270.34 | 0.865861 | 1.10803 | 0.781442 | 0.434543 | -1.30584 | 3.037559 | EAS | Alpha-1-antitryps | endocrine/metabolic |
| 270.35 | -0.09783 | 0.992759 | -0.09854 | 0.921501 | -2.0436 | 1.847942 | EAS | Macroglobulinem | endocrine/metabolic |
| 270.38 | -0.08287 | 0.154817 | -0.53529 | 0.59245 | -0.38631 | 0.220563 | EAS | Other specified d | endocrine/metabolic |
| 271 | -0.00749 | 0.046595 | -0.16065 | 0.872371 | -0.09881 | 0.08384 | EAS | Disorders of carb | endocrine/metabolic |
| 271.3 | -0.0028 | 0.046658 | -0.05996 | 0.952189 | -0.09425 | 0.088651 | EAS | Intestinal disacch | endocrine/metabolic |
| 271.9 | -0.71955 | 0.456388 | -1.57662 | 0.114882 | -1.61406 | 0.174952 | EAS | Other disorders c | endocrine/metabolic |
| 272 | -0.06408 | 0.049189 | -1.30272 | 0.192669 | -0.16049 | 0.032329 | EAS | Disorders of lipoi | endocrine/metabolic |
| 272.1 | -0.06407 | 0.04921 | -1.30192 | 0.192944 | -0.16052 | 0.032383 | EAS | Hyperlipidemia | endocrine/metabolic |
| 272.11 | -0.02671 | 0.051083 | -0.52278 | 0.601127 | -0.12683 | 0.073416 | EAS | Hypercholesterol | endocrine/metabolic |
| 272.12 | -0.0623 | 0.092259 | -0.67524 | 0.499525 | -0.24312 | 0.118527 | EAS | Hyperglyceridem | endocrine/metabolic |
| 272.13 | -0.07058 | 0.057503 | -1.22745 | 0.219653 | -0.18329 | 0.042122 | EAS | Mixed hyperlipide | endocrine/metabolic |
| 272.14 | -0.70827 | 0.736906 | -0.96113 | 0.336485 | -2.15258 | 0.736044 | EAS | Hyperchylomicro | endocrine/metabolic |
| 272.9 | -0.27468 | 0.203113 | -1.35235 | 0.176264 | -0.67277 | 0.123415 | EAS | Unspecified disor | endocrine/metabolic |
| 274 | 0.105719 | 0.081735 | 1.293436 | 0.19586 | -0.05448 | 0.265918 | EAS | Gout and other c | endocrine/metabolic |
| 274.1 | 0.097714 | 0.084696 | 1.15371 | 0.248619 | -0.06829 | 0.263715 | EAS | Gout | endocrine/metabolic |
| 274.11 | 0.079981 | 0.105601 | 0.75739 | 0.448816 | -0.12699 | 0.286956 | EAS | Gouty arthropath | endocrine/metabolic |
| 274.2 | 0.153459 | 0.231236 | 0.663647 | 0.506916 | -0.29976 | 0.606673 | EAS | Crystal arthropat | endocrine/metabolic |
| 274.21 | 0.153459 | 0.231236 | 0.663647 | 0.506916 | -0.29976 | 0.606673 | EAS | Chondrocalcinos | endocrine/metabolic |
| 275 | -0.06927 | 0.059303 | -1.16804 | 0.242792 | -0.1855 | 0.046964 | EAS | Disorders of mine | endocrine/metabolic |
| 275.1 | 0.157616 | 0.254909 | 0.618324 | 0.536362 | -0.342 | 0.657228 | EAS | Disorders of iron | hematopoietic |
| 275.11 | 0.775019 | 0.501501 | 1.545399 | 0.12225 | -0.2079 | 1.757943 | EAS | Hereditary hemo | hematopoietic |
| 275.2 | -0.53808 | 0.619145 | -0.86908 | 0.384805 | -1.75159 | 0.675418 | EAS | Disorders of copp | endocrine/metabolic |
| 275.3 | -0.02357 | 0.092127 | -0.25588 | 0.798043 | -0.20414 | 0.156992 | EAS | Disorders of mag | endocrine/metabolic |
| 275.5 | 0.057466 | 0.074526 | 0.771089 | 0.440654 | -0.0886 | 0.203534 | EAS | Disorders of calc | endocrine/metabolic |
| 275.51 | 0.041145 | 0.098397 | 0.418153 | 0.675836 | -0.15171 | 0.233999 | EAS | Hypocalcemia | endocrine/metabolic |
| 275.53 | 0.082986 | 0.097067 | 0.854937 | 0.392586 | -0.10726 | 0.273233 | EAS | Disorders of phos | endocrine/metabolic |

| phecode | Coef. | Std.Err. | z | p_value_z | [0.025 | 0.975] | GIA | phenotype | category |
|---|---|---|---|---|---|---|---|---|---|
| 275.6 | -0.21056 | 0.102443 | -2.05539 | 0.039841 | -0.41135 | -0.00978 | EAS | Hypercalcemia | endocrine/metabolic |
| 276 | 0.022591 | 0.048259 | 0.468129 | 0.639692 | -0.07199 | 0.117176 | EAS | Disorders of fluid | endocrine/metabolic |
| 276.1 | 0.008232 | 0.050578 | 0.162751 | 0.870714 | -0.0909 | 0.107363 | EAS | Electrolyte imbala | endocrine/metabolic |
| 276.11 | -0.03192 | 0.128909 | -0.24761 | 0.804438 | -0.28457 | 0.220737 | EAS | Hyperosmolality a | endocrine/metabolic |
| 276.12 | -0.00413 | 0.066728 | -0.06186 | 0.950677 | -0.13491 | 0.126657 | EAS | Hyposmolality an | endocrine/metabolic |
| 276.13 | 0.010549 | 0.075652 | 0.139435 | 0.889106 | -0.13773 | 0.158824 | EAS | Hyperpotassemia | endocrine/metabolic |
| 276.14 | 0.050726 | 0.069381 | 0.731119 | 0.464707 | -0.08526 | 0.186711 | EAS | Hypopotassemia | endocrine/metabolic |
| 276.4 | 0.044408 | 0.074194 | 0.598529 | 0.549487 | -0.10101 | 0.189826 | EAS | Acid-base balanc | endocrine/metabolic |
| 276.41 | 0.053883 | 0.07879 | 0.683884 | 0.494049 | -0.10054 | 0.208308 | EAS | Acidosis | endocrine/metabolic |
| 276.42 | 0.100541 | 0.172376 | 0.583264 | 0.559716 | -0.23731 | 0.438393 | EAS | Alkalosis | endocrine/metabolic |
| 276.5 | -0.04933 | 0.070066 | -0.704 | 0.481431 | -0.18665 | 0.088 | EAS | Hypovolemia | endocrine/metabolic |
| 276.6 | 0.095834 | 0.090969 | 1.053475 | 0.292123 | -0.08246 | 0.27413 | EAS | Fluid overload | endocrine/metabolic |
| 276.8 | 0.075191 | 0.386876 | 0.194355 | 0.845898 | -0.68307 | 0.833455 | EAS | Polydipsia | endocrine/metabolic |
| 277 | -0.03395 | 0.072214 | -0.47017 | 0.638231 | -0.17549 | 0.107583 | EAS | Other disorders c | endocrine/metabolic |
| 277.1 | -0.48836 | 1.092024 | -0.4472 | 0.654728 | -2.62869 | 1.651971 | EAS | Disorders of porp | endocrine/metabolic |
| 277.4 | -0.21537 | 0.123714 | -1.74084 | 0.081712 | -0.45784 | 0.027109 | EAS | Disorders of biliru | endocrine/metabolic |
| 277.5 | 0.053807 | 0.172069 | 0.312705 | 0.754505 | -0.28344 | 0.391055 | EAS | Other disorders c | endocrine/metabolic |
| 277.51 | -0.23838 | 0.214622 | -1.1107 | 0.266698 | -0.65903 | 0.182271 | EAS | Lipoprotein disor | endocrine/metabolic |
| 277.6 | -0.51543 | 0.501563 | -1.02765 | 0.304113 | -1.49848 | 0.467613 | EAS | Other deficiencie | endocrine/metabolic |
| 277.7 | -0.01814 | 0.139686 | -0.12985 | 0.896681 | -0.29192 | 0.255642 | EAS | Dysmetabolic syr | endocrine/metabolic |
| 278 | 0.053484 | 0.054847 | 0.975137 | 0.329492 | -0.05402 | 0.160983 | EAS | Overweight, obes | endocrine/metabolic |
| 278.1 | 0.178901 | 0.074937 | 2.387348 | 0.01697 | 0.032027 | 0.325775 | EAS | Obesity | endocrine/metabolic |
| 278.11 | 0.217376 | 0.124257 | 1.749403 | 0.080221 | -0.02616 | 0.460916 | EAS | Morbid obesity | endocrine/metabolic |
| 278.3 | -0.28117 | 0.446134 | -0.63023 | 0.528546 | -1.15557 | 0.593241 | EAS | Localized adipos | endocrine/metabolic |
| 278.4 | -0.09156 | 0.104433 | -0.87676 | 0.380615 | -0.29625 | 0.113122 | EAS | Abnormal weight | endocrine/metabolic |
| 279 | -0.00598 | 0.069744 | -0.08568 | 0.931723 | -0.14267 | 0.13072 | EAS | Disorders involvi | endocrine/metabolic |
| 279.1 | 0.012379 | 0.072326 | 0.171155 | 0.864102 | -0.12938 | 0.154134 | EAS | Immunity deficier | endocrine/metabolic |
| 279.11 | 0.087514 | 0.225008 | 0.388937 | 0.697323 | -0.35349 | 0.528522 | EAS | Deficiency of hur | endocrine/metabolic |
| 279.2 | -0.48845 | 0.284874 | -1.7146 | 0.086419 | -1.04679 | 0.069898 | EAS | Autoimmune dise | endocrine/metabolic |
| 279.7 | -0.04965 | 0.071593 | -0.69353 | 0.487979 | -0.18997 | 0.090668 | EAS | Other immunolog | endocrine/metabolic |
| 279.8 | -0.02672 | 0.261351 | -0.10225 | 0.918559 | -0.53896 | 0.485516 | EAS | Other specified d | endocrine/metabolic |
| 280 | -0.03635 | 0.062897 | -0.57786 | 0.563362 | -0.15962 | 0.086931 | EAS | Iron deficiency ar | hematopoietic |
| 280.1 | 0.049968 | 0.068075 | 0.734012 | 0.462941 | -0.08346 | 0.183393 | EAS | Iron deficiency ar | hematopoietic |
| 280.2 | -0.10155 | 0.096552 | -1.05181 | 0.292885 | -0.29079 | 0.087684 | EAS | Iron deficiency ar | hematopoietic |
| 281 | -0.10777 | 0.110571 | -0.97471 | 0.329703 | -0.32449 | 0.10894 | EAS | Other deficiency | hematopoietic |
| 281.1 | -0.32007 | 0.213701 | -1.49775 | 0.134199 | -0.73892 | 0.098776 | EAS | Megaloblastic an | hematopoietic |
| 281.11 | -1.25682 | 0.458421 | -2.74163 | 0.006114 | -2.15531 | -0.35833 | EAS | Pernicious anem | hematopoietic |

| phecode | Coef. | Std.Err. | z | p_value_z | [0.025 | 0.975] | GIA | phenotype | category |
|---|---|---|---|---|---|---|---|---|---|
| 281.12 | -0.38132 | 0.283318 | -1.34592 | 0.178327 | -0.93662 | 0.173968 | EAS | Other vitamin B1 | hematopoietic |
| 281.13 | 0.112474 | 0.446405 | 0.251955 | 0.801076 | -0.76246 | 0.987412 | EAS | Folate-deficiency | hematopoietic |
| 281.9 | -0.05923 | 0.126512 | -0.4682 | 0.63964 | -0.30719 | 0.188726 | EAS | Deficiency anemi | hematopoietic |
| 282 | 0.179819 | 0.117744 | 1.5272 | 0.126711 | -0.05096 | 0.410593 | EAS | Hereditary hemol | hematopoietic |
| 282.5 | 0.301218 | 0.541671 | 0.55609 | 0.578149 | -0.76044 | 1.362874 | EAS | Sickle cell anemi | hematopoietic |
| 282.8 | 0.028642 | 0.13947 | 0.205364 | 0.837287 | -0.24471 | 0.301998 | EAS | Other hemoglobi | hematopoietic |
| 282.9 | 0.393056 | 0.228596 | 1.719433 | 0.085536 | -0.05498 | 0.841097 | EAS | Other hereditary | hematopoietic |
| 283 | -0.09786 | 0.242568 | -0.40345 | 0.686616 | -0.57329 | 0.377559 | EAS | Acquired hemoly | hematopoietic |
| 283.1 | -0.51715 | 0.520438 | -0.99368 | 0.320377 | -1.53719 | 0.502889 | EAS | Autoimmune hen | hematopoietic |
| 283.2 | 0.328738 | 0.552904 | 0.594566 | 0.552134 | -0.75493 | 1.41241 | EAS | Non-autoimmune | hematopoietic |
| 283.21 | 0.502671 | 0.668137 | 0.752347 | 0.451842 | -0.80685 | 1.812195 | EAS | Hemolytic-uremic | hematopoietic |
| 284 | -0.08191 | 0.094499 | -0.86681 | 0.386048 | -0.26713 | 0.103302 | EAS | Aplastic anemia | hematopoietic |
| 284.1 | -0.08076 | 0.095546 | -0.84522 | 0.39799 | -0.26802 | 0.10651 | EAS | Pancytopenia | hematopoietic |
| 284.2 | 0.05942 | 0.487894 | 0.121788 | 0.903067 | -0.89684 | 1.015674 | EAS | Constitutional ap | hematopoietic |
| 285 | 0.012624 | 0.043137 | 0.292643 | 0.769795 | -0.07192 | 0.09717 | EAS | Other anemias | hematopoietic |
| 285.1 | 0.116713 | 0.06079 | 1.91993 | 0.054867 | -0.00243 | 0.235859 | EAS | Acute posthemor | hematopoietic |
| 285.2 | 0.038391 | 0.064084 | 0.599065 | 0.549129 | -0.08721 | 0.163994 | EAS | Anemia of chroni | hematopoietic |
| 285.21 | 0.082937 | 0.086997 | 0.953335 | 0.34042 | -0.08757 | 0.253448 | EAS | Anemia in chroni | hematopoietic |
| 285.22 | -0.10547 | 0.112798 | -0.93504 | 0.349766 | -0.32655 | 0.115609 | EAS | Anemia in neopla | hematopoietic |
| 285.8 | -0.9133 | 0.630831 | -1.44778 | 0.147679 | -2.14971 | 0.323103 | EAS | Hemoglobinuria | hematopoietic |
| 286 | 0.081268 | 0.074217 | 1.094996 | 0.273519 | -0.0642 | 0.226731 | EAS | Coagulation defe | hematopoietic |
| 286.1 | -0.01336 | 0.269826 | -0.04951 | 0.960511 | -0.54221 | 0.51549 | EAS | Congenital coagu | hematopoietic |
| 286.11 | 0.061093 | 0.319281 | 0.191346 | 0.848255 | -0.56469 | 0.686872 | EAS | Von willebrand's | hematopoietic |
| 286.12 | 0.187069 | 0.579866 | 0.322607 | 0.746993 | -0.94945 | 1.323586 | EAS | Congenital defici | hematopoietic |
| 286.13 | -0.45638 | 1.134829 | -0.40216 | 0.687569 | -2.6806 | 1.767845 | EAS | Congenital factor | hematopoietic |
| 286.2 | -0.0306 | 0.079727 | -0.38379 | 0.701132 | -0.18686 | 0.125664 | EAS | Encounter for lon | hematopoietic |
| 286.3 | -0.02551 | 0.169414 | -0.1506 | 0.880294 | -0.35756 | 0.306533 | EAS | Coagulation defe | hematopoietic |
| 286.4 | -0.02689 | 0.158324 | -0.16982 | 0.865151 | -0.3372 | 0.283423 | EAS | Acquired coagula | hematopoietic |
| 286.6 | 0.134787 | 0.207579 | 0.649329 | 0.516126 | -0.27206 | 0.541635 | EAS | Defibrination syn | hematopoietic |
| 286.7 | 0.034747 | 0.101248 | 0.343189 | 0.731456 | -0.1637 | 0.23319 | EAS | Other and unspe | hematopoietic |
| 286.8 | 0.134085 | 0.177447 | 0.755632 | 0.44987 | -0.21371 | 0.481875 | EAS | Hypercoagulable | hematopoietic |
| 286.81 | 0.021792 | 0.199256 | 0.109365 | 0.912913 | -0.36874 | 0.412327 | EAS | Primary hypercoa | hematopoietic |
| 286.9 | -0.00063 | 0.135632 | -0.00464 | 0.996301 | -0.26646 | 0.265206 | EAS | Abnormal coagul | hematopoietic |
| 287 | 0.084485 | 0.061963 | 1.363473 | 0.172733 | -0.03696 | 0.205929 | EAS | Purpura and othe | hematopoietic |
| 287.1 | 0.019345 | 0.216998 | 0.089149 | 0.928964 | -0.40596 | 0.444653 | EAS | Spontaneous ecc | hematopoietic |
| 287.3 | 0.095998 | 0.063781 | 1.505118 | 0.132294 | -0.02901 | 0.221006 | EAS | Thrombocytopen | hematopoietic |
| 287.31 | -0.20279 | 0.251217 | -0.80722 | 0.41954 | -0.69516 | 0.289589 | EAS | Primary thrombo | hematopoietic |

| phecode | Coef. | Std.Err. | z | p_value_z | [0.025 | 0.975] | GIA | phenotype | category | |
|---|---|---|---|---|---|---|---|---|---|---|
| 287.32 | -0.0823 | 0.113828 | -0.72298 | 0.469692 | -0.30539 | 0.140803 | EAS | Secondary throm | hematopoietic | |
| 287.4 | -0.19014 | 0.293023 | -0.64887 | 0.51642 | -0.76445 | 0.38418 | EAS | Qualitative platel | hematopoietic | |
| 288 | 0.06362 | 0.063666 | 0.999273 | 0.317663 | -0.06116 | 0.188403 | EAS | Diseases of white | hematopoietic | |
| 288.1 | -0.0669 | 0.058017 | -1.15309 | 0.248872 | -0.18061 | 0.046812 | EAS | Decreased white | hematopoietic | |
| 288.11 | -0.06051 | 0.07737 | -0.78213 | 0.43414 | -0.21216 | 0.091129 | EAS | Neutropenia | hematopoietic | |
| 288.2 | 0.102487 | 0.06559 | 1.56254 | 0.118161 | -0.02607 | 0.231042 | EAS | Elevated white bl | hematopoietic | |
| 288.3 | -0.10798 | 0.180713 | -0.59755 | 0.550143 | -0.46218 | 0.246207 | EAS | Eosinophilia | hematopoietic | |
| 289 | 0.063813 | 0.063102 | 1.011273 | 0.311886 | -0.05986 | 0.18749 | EAS | Other diseases o | hematopoietic | |
| 289.3 | 0.117544 | 0.106242 | 1.106382 | 0.268561 | -0.09069 | 0.325775 | EAS | Personal history | hematopoietic | |
| 289.4 | -0.09697 | 0.066599 | -1.45604 | 0.145382 | -0.2275 | 0.033561 | EAS | Lymphadenitis | hematopoietic | |
| 289.5 | -0.02454 | 0.226874 | -0.10817 | 0.913863 | -0.46921 | 0.420125 | EAS | Diseases of sple | hematopoietic | |
| 289.8 | 0.019314 | 0.154562 | 0.124959 | 0.900556 | -0.28362 | 0.32225 | EAS | Polycythemia, se | hematopoietic | |
| 289.9 | 0.002045 | 0.124172 | 0.016471 | 0.986859 | -0.24133 | 0.245419 | EAS | Abnormality of re | hematopoietic | |
| 290 | 0.028689 | 0.119794 | 0.239484 | 0.810731 | -0.2061 | 0.263482 | EAS | Delirium dementi | mental disorders | |
| 290.1 | -0.04478 | 0.155184 | -0.28858 | 0.7729 | -0.34894 | 0.259372 | EAS | Dementias | mental disorders | |
| 290.11 | 0.0717 | 0.199754 | 0.358939 | 0.719641 | -0.31981 | 0.463211 | EAS | Alzheimer's disea | mental disorders | |
| 290.12 | 0.090997 | 0.473598 | 0.19214 | 0.847633 | -0.83724 | 1.019233 | EAS | Dementia with ce | mental disorders | |
| 290.13 | 0.14919 | 0.355437 | 0.419737 | 0.674677 | -0.54745 | 0.845834 | EAS | Senile dementia | mental disorders | |
| 290.16 | 0.04774 | 0.261019 | 0.1829 | 0.854877 | -0.46385 | 0.559329 | EAS | Vascular dement | mental disorders | |
| 290.2 | -0.01918 | 0.167751 | -0.11436 | 0.908951 | -0.34797 | 0.309601 | EAS | Delirium due to c | mental disorders | |
| 290.3 | -0.01143 | 0.161127 | -0.07094 | 0.943449 | -0.32723 | 0.304373 | EAS | Other persistent | mental disorders | |
| 291 | -0.01117 | 0.095394 | -0.11708 | 0.906798 | -0.19814 | 0.1758 | EAS | Other specified n | mental disorders | |
| 291.1 | -0.44953 | 0.416429 | -1.0795 | 0.280367 | -1.26572 | 0.366653 | EAS | Transient mental | mental disorders | |
| 291.4 | -0.02245 | 0.186874 | -0.12016 | 0.904357 | -0.38872 | 0.343811 | EAS | Specific nonpsyc | mental disorders | |
| 291.8 | 0.05087 | 0.111864 | 0.454749 | 0.64929 | -0.16838 | 0.270119 | EAS | Alteration of cons | mental disorders | |
| 292 | -0.05686 | 0.060446 | -0.94068 | 0.346869 | -0.17533 | 0.061611 | EAS | Neurological disc | mental disorders | |
| 292.1 | 0.012932 | 0.118388 | 0.109236 | 0.913015 | -0.2191 | 0.244969 | EAS | Aphasia/speech | mental disorders | |
| 292.11 | -0.14948 | 0.201036 | -0.74355 | 0.457147 | -0.5435 | 0.244543 | EAS | Aphasia | mental disorders | |
| 292.12 | 0.24948 | 0.302647 | 0.824326 | 0.409755 | -0.3437 | 0.842657 | EAS | Symbolic dysfunc | mental disorders | |
| 292.2 | 0.099864 | 0.19362 | 0.515772 | 0.606013 | -0.27962 | 0.479352 | EAS | Mild cognitive im | mental disorders | |
| 292.3 | -0.12747 | 0.08617 | -1.47926 | 0.139072 | -0.29636 | 0.041423 | EAS | Memory loss | mental disorders | |
| 292.4 | -0.10363 | 0.089076 | -1.16344 | 0.244652 | -0.27822 | 0.070952 | EAS | Altered mental st | mental disorders | |
| 292.5 | -0.11036 | 0.225885 | -0.48857 | 0.625149 | -0.55309 | 0.332367 | EAS | Transient alterati | mental disorders | |
| 292.6 | 0.660728 | 0.31143 | 2.121596 | 0.033872 | 0.050337 | 1.271119 | EAS | Hallucinations | mental disorders | |
| 293 | 0.118482 | 0.106696 | 1.110469 | 0.266797 | -0.09064 | 0.327602 | EAS | Symptoms involv | mental disorders | |
| 293.1 | -0.06614 | 0.071794 | -0.92119 | 0.356952 | -0.20685 | 0.074577 | EAS | Swelling, mass, c | mental disorders | |
| 295 | -0.05853 | 0.173304 | -0.33771 | 0.735578 | -0.3982 | 0.281142 | EAS | Schizophrenia ar | mental disorders | |

| phecode | Coef. | Std.Err. | z | p_value_z | [0.025 | 0.975] | GIA | phenotype | category | |
|---|---|---|---|---|---|---|---|---|---|---|
| 295.1 | -0.02117 | 0.268348 | -0.0789 | 0.937114 | -0.54712 | 0.50478 | EAS | Schizophrenia | mental disorders | |
| 295.2 | 0.182398 | 0.318264 | 0.573102 | 0.566575 | -0.44139 | 0.806184 | EAS | Paranoid disorde | mental disorders | |
| 295.3 | -0.11554 | 0.202443 | -0.57072 | 0.568193 | -0.51232 | 0.281243 | EAS | Psychosis | mental disorders | |
| 296 | 0.076228 | 0.052763 | 1.444717 | 0.148537 | -0.02719 | 0.179641 | EAS | Mood disorders | mental disorders | |
| 296.1 | 0.060262 | 0.14784 | 0.407619 | 0.683553 | -0.2295 | 0.350024 | EAS | Bipolar | mental disorders | |
| 296.2 | 0.081716 | 0.05472 | 1.493344 | 0.135347 | -0.02553 | 0.188966 | EAS | Depression | mental disorders | |
| 296.22 | 0.058325 | 0.058438 | 0.998072 | 0.318244 | -0.05621 | 0.172861 | EAS | Major depressive | mental disorders | |
| 297 | -0.08664 | 0.191559 | -0.4523 | 0.651055 | -0.46209 | 0.288807 | EAS | Suicidal ideation | mental disorders | |
| 297.1 | -0.13421 | 0.211084 | -0.6358 | 0.524904 | -0.54792 | 0.279509 | EAS | Suicidal ideation | mental disorders | |
| 297.2 | 0.575658 | 0.552624 | 1.041681 | 0.297559 | -0.50746 | 1.658781 | EAS | Suicide or self-in | mental disorders | |
| 300 | 0.073431 | 0.048544 | 1.512676 | 0.130362 | -0.02171 | 0.168575 | EAS | Anxiety disorders | mental disorders | |
| 300.1 | 0.060036 | 0.050498 | 1.188885 | 0.234485 | -0.03894 | 0.159011 | EAS | Anxiety disorder | mental disorders | |
| 300.11 | -0.07685 | 0.086698 | -0.88645 | 0.375376 | -0.24678 | 0.093071 | EAS | Generalized anxi | mental disorders | |
| 300.12 | -0.12973 | 0.143537 | -0.90383 | 0.366084 | -0.41106 | 0.151594 | EAS | Agorophobia, so | mental disorders | |
| 300.13 | 0.211472 | 0.219839 | 0.961942 | 0.336079 | -0.2194 | 0.642349 | EAS | Phobia | mental disorders | |
| 300.3 | -0.0675 | 0.38448 | -0.17555 | 0.860647 | -0.82106 | 0.686072 | EAS | Obsessive-comp | mental disorders | |
| 300.4 | 0.079138 | 0.129137 | 0.612823 | 0.539994 | -0.17397 | 0.332242 | EAS | Dysthymic disord | mental disorders | |
| 300.8 | -0.08656 | 0.218241 | -0.39662 | 0.691644 | -0.5143 | 0.341185 | EAS | Acute reaction to | mental disorders | |
| 300.9 | -0.1865 | 0.216486 | -0.86148 | 0.388971 | -0.6108 | 0.237805 | EAS | Posttraumatic str | mental disorders | |
| 301 | 0.211753 | 0.379178 | 0.558451 | 0.576536 | -0.53142 | 0.954928 | EAS | Personality disor | mental disorders | |
| 301.2 | 0.006862 | 0.4709 | 0.014571 | 0.988374 | -0.91609 | 0.929809 | EAS | Antisocial/border | mental disorders | |
| 302 | 0.027474 | 0.205158 | 0.133914 | 0.89347 | -0.37463 | 0.429575 | EAS | Sexual and gend | mental disorders | |
| 302.1 | 0.17171 | 0.269406 | 0.637365 | 0.523887 | -0.35632 | 0.699735 | EAS | Decreased libido | mental disorders | |
| 303 | 0.008579 | 0.127146 | 0.067473 | 0.946205 | -0.24062 | 0.25778 | EAS | Psychogenic and | mental disorders | |
| 303.1 | 0.798671 | 0.698693 | 1.143092 | 0.253 | -0.57074 | 2.168085 | EAS | Dissociative diso | mental disorders | |
| 303.3 | 0.124891 | 0.130791 | 0.954893 | 0.339632 | -0.13145 | 0.381237 | EAS | Psychogenic dis | mental disorders | |
| 303.31 | 0.113617 | 0.151943 | 0.747757 | 0.454607 | -0.18419 | 0.411421 | EAS | Gastrointestinal | mental disorders | |
| 303.4 | -1.37193 | 0.407668 | -3.36531 | 0.000765 | -2.17094 | -0.57291 | EAS | Somatoform diso | mental disorders | |
| 304 | -0.06853 | 0.078085 | -0.87759 | 0.380168 | -0.22157 | 0.084518 | EAS | Adjustment react | mental disorders | |
| 305.2 | -0.1342 | 0.278757 | -0.48141 | 0.630226 | -0.68055 | 0.412157 | EAS | Eating disorder | mental disorders | |
| 305.21 | -0.88579 | 0.815389 | -1.08634 | 0.277326 | -2.48393 | 0.71234 | EAS | Anorexia nervosa | mental disorders | |
| 306 | 0.019272 | 0.0434 | 0.44406 | 0.656999 | -0.06579 | 0.104336 | EAS | Other mental dis | mental disorders | |
| 306.1 | 0.094921 | 0.156618 | 0.606067 | 0.54447 | -0.21204 | 0.401886 | EAS | Mental disorders | mental disorders | |
| 306.9 | -0.11253 | 0.133856 | -0.84068 | 0.400527 | -0.37488 | 0.149822 | EAS | Tension headach | mental disorders | |
| 312 | 0.4102 | 0.24715 | 1.659718 | 0.096971 | -0.07421 | 0.894605 | EAS | Conduct disorder | mental disorders | |
| 312.3 | 0.059786 | 0.594986 | 0.100483 | 0.919961 | -1.10636 | 1.225936 | EAS | Impulse control d | mental disorders | |
| 313 | 0.184341 | 0.166106 | 1.109781 | 0.267093 | -0.14122 | 0.509902 | EAS | Pervasive develo | mental disorders | |

| phecode | Coef. | Std.Err. | z | p_value_z | [0.025 | 0.975] | GIA | phenotype | category |
|---|---|---|---|---|---|---|---|---|---|
| 313.1 | 0.127196 | 0.180594 | 0.704319 | 0.481234 | -0.22676 | 0.481153 | EAS | Attention deficit h | mental disorders |
| 313.2 | 0.167807 | 0.575991 | 0.291336 | 0.770795 | -0.96111 | 1.296728 | EAS | Tics and stutterin | mental disorders |
| 313.3 | -0.53736 | 0.715727 | -0.75079 | 0.45278 | -1.94016 | 0.865439 | EAS | Autism | mental disorders |
| 315 | -0.43597 | 0.17902 | -2.4353 | 0.01488 | -0.78684 | -0.08509 | EAS | Develomental de | mental disorders |
| 315.1 | 1.122433 | 1.045562 | 1.073521 | 0.283037 | -0.92683 | 3.171697 | EAS | Learning disorde | mental disorders |
| 315.2 | -0.60047 | 0.306924 | -1.9564 | 0.050418 | -1.20203 | 0.001094 | EAS | Speech and lang | mental disorders |
| 315.3 | -1.03047 | 1.158431 | -0.88954 | 0.373714 | -3.30095 | 1.240014 | EAS | Mental retardatio | mental disorders |
| 316 | 0.259914 | 0.10079 | 2.578783 | 0.009915 | 0.06237 | 0.457458 | EAS | Substance addic | mental disorders |
| 316.1 | 0.077616 | 0.147203 | 0.527273 | 0.598004 | -0.2109 | 0.366129 | EAS | Polyneuropathy ( | mental disorders |
| 317 | -0.04711 | 0.115331 | -0.40843 | 0.682954 | -0.27315 | 0.17894 | EAS | Alcohol-related d | mental disorders |
| 317.1 | -0.06072 | 0.127174 | -0.47747 | 0.633029 | -0.30998 | 0.188536 | EAS | Alcoholism | mental disorders |
| 317.11 | -0.43644 | 0.214197 | -2.03756 | 0.041594 | -0.85626 | -0.01662 | EAS | Alcoholic liver da | mental disorders |
| 318 | 0.199433 | 0.051943 | 3.839449 | 0.000123 | 0.097626 | 0.301239 | EAS | Tobacco use disc | mental disorders |
| 320 | -0.21039 | 0.221171 | -0.95124 | 0.341485 | -0.64387 | 0.223102 | EAS | Meningitis | neurological |
| 323 | -0.53491 | 0.316928 | -1.6878 | 0.091449 | -1.15608 | 0.086255 | EAS | Encephalitis | neurological |
| 323.8 | -0.76269 | 0.435322 | -1.75202 | 0.07977 | -1.61591 | 0.090521 | EAS | Encephalitis, non | neurological |
| 324 | -0.48292 | 0.255789 | -1.88798 | 0.059029 | -0.98426 | 0.018413 | EAS | Other CNS infect | neurological |
| 325 | 0.862336 | 0.972447 | 0.88677 | 0.375203 | -1.04362 | 2.768296 | EAS | Phlebitis and thro | neurological |
| 327 | -0.00123 | 0.049821 | -0.02462 | 0.980361 | -0.09887 | 0.096422 | EAS | Sleep disorders | neurological |
| 327.1 | 0.038414 | 0.101507 | 0.378432 | 0.70511 | -0.16054 | 0.237364 | EAS | Hypersomnia | neurological |
| 327.3 | 0.176691 | 0.058899 | 2.999892 | 0.002701 | 0.061251 | 0.292132 | EAS | Sleep apnea | neurological |
| 327.31 | 0.081876 | 0.179493 | 0.456153 | 0.64828 | -0.26992 | 0.433676 | EAS | Central/nonobstr | neurological |
| 327.32 | 0.209388 | 0.065436 | 3.199868 | 0.001375 | 0.081135 | 0.337641 | EAS | Obstructive sleep | neurological |
| 327.4 | -0.06232 | 0.055815 | -1.11659 | 0.264172 | -0.17172 | 0.047073 | EAS | Insomnia | neurological |
| 327.41 | 0.005055 | 0.093661 | 0.053968 | 0.956961 | -0.17852 | 0.188627 | EAS | Organic or persis | neurological |
| 327.5 | 0.548008 | 0.233751 | 2.344406 | 0.019057 | 0.089864 | 1.006153 | EAS | Parasomnia | neurological |
| 327.6 | 0.289098 | 0.217776 | 1.327501 | 0.184343 | -0.13774 | 0.715931 | EAS | Circadian rhythm | neurological |
| 327.7 | -0.02562 | 0.118043 | -0.21706 | 0.828165 | -0.25698 | 0.205737 | EAS | Sleep related mo | neurological |
| 327.71 | 0.131306 | 0.192322 | 0.682742 | 4.95E-01 | -0.24564 | 0.50825 | EAS | Restless legs syr | neurological |
| 327.72 | -0.1403 | 0.237319 | -0.59121 | 0.554382 | -0.60544 | 0.324833 | EAS | Sleep related leg | neurological |
| 331 | -0.18379 | 0.157929 | -1.16374 | 0.244529 | -0.49332 | 0.125747 | EAS | Other cerebral de | neurological |
| 331.1 | -0.32456 | 0.237789 | -1.36491 | 0.172282 | -0.79062 | 0.141498 | EAS | Hydrocephalus | neurological |
| 331.9 | -0.282 | 0.209512 | -1.34597 | 0.178311 | -0.69263 | 0.128638 | EAS | Cerebral degene | neurological |
| 332 | 0.495071 | 0.253347 | 1.954126 | 0.050686 | -0.00148 | 0.991621 | EAS | Parkinson's disea | neurological |
| 333 | -0.06812 | 0.13749 | -0.49543 | 0.620297 | -0.33759 | 0.201358 | EAS | Extrapyramidal d | neurological |
| 333.1 | -0.34912 | 0.228906 | -1.52516 | 0.127218 | -0.79777 | 0.099528 | EAS | Essential tremor | neurological |
| 333.2 | 0.053053 | 0.28831 | 0.184015 | 0.854002 | -0.51202 | 0.61813 | EAS | Myoclonus | neurological |

| phecode | Coef. | Std.Err. | z | p_value_z | [0.025 | 0.975] | GIA | phenotype | category | |
|---|---|---|---|---|---|---|---|---|---|---|
| 333.3 | -3.39722 | 2.593581 | -1.30986 | 0.190245 | -8.48054 | 1.68611 | EAS | Tics and choreas | neurological | |
| 333.4 | 0.426167 | 0.248694 | 1.713617 | 0.086599 | -0.06127 | 0.913598 | EAS | Torsion dystonia | neurological | |
| 333.8 | 0.425882 | 0.479449 | 0.888274 | 0.374394 | -0.51382 | 1.365584 | EAS | Other degenerati | neurological | |
| 334 | 0.25774 | 0.130255 | 1.97873 | 0.047846 | 0.002444 | 0.513036 | EAS | Degenerative dis | neurological | |
| 334.1 | 0.580112 | 0.556838 | 1.041795 | 0.297507 | -0.51127 | 1.671495 | EAS | Spinocerebellar ( | neurological | |
| 334.2 | 0.366089 | 0.397963 | 0.919908 | 0.357621 | -0.4139 | 1.146082 | EAS | Anterior horn cell | neurological | |
| 334.21 | 0.366089 | 0.397963 | 0.919908 | 0.357621 | -0.4139 | 1.146082 | EAS | Amyotrophic Late | neurological | |
| 335 | -0.12954 | 0.360963 | -0.35887 | 0.719696 | -0.83701 | 0.577938 | EAS | Multiple sclerosis | neurological | |
| 337 | 0.161773 | 0.171973 | 0.940689 | 0.346864 | -0.17529 | 0.498834 | EAS | Disorders of the a | neurological | |
| 337.1 | 0.168454 | 0.204568 | 0.823462 | 0.410245 | -0.23249 | 0.5694 | EAS | Peripheral autono | neurological | |
| 338 | -0.0356 | 0.042288 | -0.84188 | 0.399854 | -0.11848 | 0.047281 | EAS | Pain | neurological | |
| 338.1 | -0.03806 | 0.04769 | -0.79816 | 0.424776 | -0.13153 | 0.055406 | EAS | Acute pain | neurological | |
| 338.2 | -0.01414 | 0.04564 | -0.30985 | 0.756677 | -0.10359 | 0.075311 | EAS | Chronic pain | neurological | |
| 339 | -0.14611 | 0.05391 | -2.71026 | 0.006723 | -0.25177 | -0.04045 | EAS | Other headache | neurological | |
| 340 | -0.10903 | 0.073927 | -1.47478 | 0.140273 | -0.25392 | 0.035868 | EAS | Migraine | neurological | |
| 340.1 | -0.10014 | 0.13688 | -0.7316 | 0.464412 | -0.36842 | 0.168138 | EAS | Migrain with aura | neurological | |
| 341 | -0.06763 | 0.477688 | -0.14157 | 0.887416 | -1.00388 | 0.868624 | EAS | Other demyelinat | neurological | |
| 342 | -0.35401 | 0.194706 | -1.81816 | 0.069039 | -0.73562 | 0.02761 | EAS | Hemiplegia | neurological | |
| 343 | 0.156163 | 0.462879 | 0.337372 | 0.735836 | -0.75106 | 1.06339 | EAS | Infantile cerebral | neurological | |
| 344 | 0.42857 | 0.180345 | 2.376388 | 0.017483 | 0.0751 | 0.78204 | EAS | Other paralytic sy | neurological | |
| 345 | -0.09207 | 0.111579 | -0.82512 | 0.409304 | -0.31076 | 0.126625 | EAS | Epilepsy, recurre | neurological | |
| 345.1 | 0.056378 | 0.173079 | 0.325734 | 0.744626 | -0.28285 | 0.395607 | EAS | Epilepsy | neurological | |
| 345.11 | -0.25206 | 0.34848 | -0.72331 | 0.46949 | -0.93507 | 0.430949 | EAS | Generalized conv | neurological | |
| 345.12 | 0.55012 | 0.277568 | 1.981925 | 0.047488 | 0.006096 | 1.094144 | EAS | Partial epilepsy | neurological | |
| 345.3 | -0.10076 | 0.119726 | -0.84163 | 0.399998 | -0.33542 | 0.133894 | EAS | Convulsions | neurological | |
| 346 | -0.12247 | 0.262514 | -0.46653 | 0.640839 | -0.63699 | 0.392048 | EAS | Abnormal finding | neurological | |
| 346.1 | 0.050329 | 0.117517 | 0.428272 | 0.668453 | -0.18 | 0.280658 | EAS | Nonspecific abno | neurological | |
| 346.2 | -0.26996 | 0.310604 | -0.86914 | 0.384768 | -0.87873 | 0.338813 | EAS | Nonspecific abno | neurological | |
| 346.3 | -1.08388 | 0.545827 | -1.98576 | 0.04706 | -2.15368 | -0.01408 | EAS | Nonspecific abno | neurological | |
| 347 | 0.71948 | 0.480924 | 1.496036 | 0.134644 | -0.22311 | 1.662075 | EAS | Cataplexy and na | neurological | |
| 348 | -0.02328 | 0.086314 | -0.26969 | 0.787397 | -0.19245 | 0.145894 | EAS | Other conditions | neurological | |
| 348.2 | -0.06253 | 0.123639 | -0.50577 | 0.613019 | -0.30486 | 0.179796 | EAS | Cerebral edema | neurological | |
| 348.4 | 0.346139 | 0.29862 | 1.159126 | 0.246405 | -0.23915 | 0.931424 | EAS | Cerebral cysts | neurological | |
| 348.7 | -0.05975 | 0.172 | -0.34736 | 0.72832 | -0.39686 | 0.277368 | EAS | Coma | neurological | |
| 348.8 | -0.03977 | 0.116695 | -0.34079 | 0.733264 | -0.26849 | 0.18895 | EAS | Encephalopathy, | neurological | |
| 348.9 | -0.03509 | 0.099323 | -0.35334 | 0.723837 | -0.22976 | 0.159575 | EAS | Other conditions | neurological | |
| 349 | 0.006816 | 0.125265 | 0.054411 | 0.956608 | -0.2387 | 0.252331 | EAS | Other and unspe | neurological | |

| phecode | Coef. | Std.Err. | z | p_value_z | [0.025 | 0.975] | GIA | phenotype | category | |
|---|---|---|---|---|---|---|---|---|---|---|
| 350 | -0.06425 | 0.063609 | -1.01004 | 0.312476 | -0.18892 | 0.060423 | EAS | Abnormal moven | neurological | |
| 350.1 | 0.011741 | 0.080286 | 0.146239 | 0.883733 | -0.14562 | 0.169099 | EAS | Abnormal involur | neurological | |
| 350.2 | -0.00223 | 0.082903 | -0.02685 | 0.97858 | -0.16471 | 0.160262 | EAS | Abnormality of ga | neurological | |
| 350.3 | -0.04629 | 0.22429 | -0.20639 | 0.83649 | -0.48589 | 0.393309 | EAS | Lack of coordinat | neurological | |
| 350.5 | 0.252774 | 0.342602 | 0.737806 | 0.460633 | -0.41871 | 0.924261 | EAS | Abnormal reflex | neurological | |
| 350.6 | -0.00502 | 0.116252 | -0.04322 | 0.965523 | -0.23288 | 0.222825 | EAS | Disturbances of s | neurological | |
| 351 | 0.09203 | 0.057152 | 1.61027 | 0.107339 | -0.01999 | 0.204046 | EAS | Other peripheral | neurological | |
| 352 | 0.167409 | 0.125802 | 1.330732 | 0.183277 | -0.07916 | 0.413977 | EAS | Disorders of othe | neurological | |
| 352.1 | 0.248793 | 0.201074 | 1.237319 | 0.215969 | -0.14531 | 0.642891 | EAS | Trigeminal nerve | neurological | |
| 352.2 | 0.187028 | 0.168486 | 1.110053 | 0.266976 | -0.1432 | 0.517255 | EAS | Facial nerve diso | neurological | |
| 353 | 0.005376 | 0.191525 | 0.028071 | 0.977606 | -0.37001 | 0.380758 | EAS | Nerve root and p | neurological | |
| 353.1 | 0.111885 | 0.280424 | 0.398986 | 0.689904 | -0.43774 | 0.661505 | EAS | Nerve plexus lesi | neurological | |
| 353.2 | -0.38907 | 0.443191 | -0.87788 | 0.380008 | -1.25771 | 0.479569 | EAS | Nerve root lesion | neurological | |
| 355 | -0.38042 | 0.313605 | -1.21306 | 0.225106 | -0.99508 | 0.234232 | EAS | Complex regiona | neurological | |
| 355.1 | 0.594099 | 0.253465 | 2.343912 | 0.019083 | 0.097317 | 1.090881 | EAS | Chronic pain syn | neurological | |
| 356 | 0.044778 | 0.127965 | 0.349923 | 0.726396 | -0.20603 | 0.295584 | EAS | Hereditary and id | neurological | |
| 357 | 0.028641 | 0.067117 | 0.426737 | 0.669571 | -0.10291 | 0.160189 | EAS | Inflammatory and | neurological | |
| 358 | 0.016533 | 0.240644 | 0.068702 | 0.945227 | -0.45512 | 0.488186 | EAS | Myoneural disord | neurological | |
| 358.1 | 0.227701 | 0.502726 | 0.452933 | 0.650597 | -0.75762 | 1.213026 | EAS | Myasthenia gravi | neurological | |
| 359 | -0.07504 | 0.156399 | -0.47983 | 0.631348 | -0.38158 | 0.231491 | EAS | Muscular dystrop | neurological | |
| 359.1 | -0.67047 | 0.458121 | -1.46353 | 0.143322 | -1.56838 | 0.227427 | EAS | Muscular dystrop | neurological | |
| 359.2 | -0.01126 | 0.16285 | -0.06915 | 0.944866 | -0.33044 | 0.307919 | EAS | Myopathy | neurological | |
| 360 | -0.1462 | 0.150618 | -0.97064 | 0.331727 | -0.4414 | 0.14901 | EAS | Disorders of the g | sense organs | |
| 360.2 | -0.03501 | 0.166761 | -0.20993 | 0.833723 | -0.36185 | 0.291838 | EAS | Progressive myo | sense organs | |
| 360.3 | 0.160038 | 0.453898 | 0.352586 | 0.724399 | -0.72959 | 1.049662 | EAS | Hypotony of eye | sense organs | |
| 361 | -0.04805 | 0.128108 | -0.37504 | 0.707629 | -0.29913 | 0.203041 | EAS | Retinal detachme | sense organs | |
| 361.1 | -0.16207 | 0.199766 | -0.81129 | 0.4172 | -0.5536 | 0.229466 | EAS | Retinal detachme | sense organs | |
| 361.2 | 0.669569 | 0.563565 | 1.188096 | 0.234796 | -0.435 | 1.774137 | EAS | Retinoschisis and | sense organs | |
| 362 | -0.01003 | 0.065253 | -0.15371 | 0.877838 | -0.13792 | 0.117863 | EAS | Other retinal diso | sense organs | |
| 362.2 | -0.00764 | 0.078682 | -0.09716 | 0.922599 | -0.16186 | 0.146569 | EAS | Degeneration of i | sense organs | |
| 362.21 | 0.026413 | 0.19051 | 0.138646 | 0.88973 | -0.34698 | 0.399806 | EAS | Macular degener | sense organs | |
| 362.22 | -0.18262 | 0.248491 | -0.73492 | 0.462386 | -0.66965 | 0.304411 | EAS | Macular degener | sense organs | |
| 362.23 | 0.200082 | 0.139711 | 1.43212 | 0.152109 | -0.07375 | 0.47391 | EAS | Cystoid macular | sense organs | |
| 362.26 | -0.0372 | 0.104174 | -0.35707 | 0.721042 | -0.24137 | 0.16698 | EAS | Macular puckerin | sense organs | |
| 362.27 | -0.18488 | 0.135315 | -1.36631 | 0.171841 | -0.45009 | 0.08033 | EAS | Drusen (degener | sense organs | |
| 362.29 | 0.114801 | 0.156274 | 0.734618 | 0.462572 | -0.19149 | 0.421092 | EAS | Macular degener | sense organs | |
| 362.3 | 0.28085 | 0.165981 | 1.692058 | 0.090635 | -0.04447 | 0.606168 | EAS | Other nondiabeti | sense organs | |

| phecode | Coef. | Std.Err. | z | p_value_z | [0.025 | 0.975] | GIA | phenotype | category | |
|---|---|---|---|---|---|---|---|---|---|---|
| 362.31 | 0.384076 | 0.245629 | 1.56364 | 0.117902 | -0.09735 | 0.865501 | EAS | Separation of reti | sense organs | |
| 362.4 | 0.031068 | 0.14814 | 0.209717 | 0.833888 | -0.25928 | 0.321416 | EAS | Retinal vascular ( | sense organs | |
| 362.6 | 0.144295 | 0.159794 | 0.903004 | 0.366524 | -0.1689 | 0.457486 | EAS | Peripheral retinal | sense organs | |
| 362.7 | -0.06427 | 0.324084 | -0.19831 | 0.842804 | -0.69946 | 0.570924 | EAS | Hereditary retinal | sense organs | |
| 362.8 | 0.083909 | 0.200616 | 0.418256 | 0.67576 | -0.30929 | 0.477109 | EAS | Retinal hemorrha | sense organs | |
| 362.9 | 0.198267 | 0.171337 | 1.157174 | 0.247201 | -0.13755 | 0.534082 | EAS | Retinal edema | sense organs | |
| 363 | 0.419867 | 0.197671 | 2.124066 | 0.033665 | 0.032438 | 0.807296 | EAS | Chorioretinal infla | sense organs | |
| 363.3 | 0.443164 | 0.26321 | 1.683688 | 0.092242 | -0.07272 | 0.959047 | EAS | Chorioretinal sca | sense organs | |
| 364 | 0.154975 | 0.148349 | 1.044663 | 0.296179 | -0.13578 | 0.445733 | EAS | Corneal opacity a | sense organs | |
| 364.1 | 0.193207 | 0.237818 | 0.812416 | 0.416553 | -0.27291 | 0.659322 | EAS | Corneal opacity | sense organs | |
| 364.2 | -0.46801 | 0.401275 | -1.16631 | 0.24349 | -1.2545 | 0.318475 | EAS | Corneal edema | sense organs | |
| 364.4 | 0.065749 | 0.21279 | 0.308986 | 0.757333 | -0.35131 | 0.482809 | EAS | Corneal degener | sense organs | |
| 364.41 | 0.314921 | 0.425796 | 0.739605 | 0.459539 | -0.51962 | 1.149467 | EAS | Keratoconus | sense organs | |
| 364.5 | 0.132551 | 0.352059 | 0.376502 | 0.706544 | -0.55747 | 0.822575 | EAS | Corneal dystroph | sense organs | |
| 364.51 | -0.51141 | 1.035769 | -0.49375 | 0.62148 | -2.54149 | 1.518656 | EAS | Fuchs' dystrophy | sense organs | |
| 364.9 | 0.13603 | 0.411918 | 0.330234 | 0.741223 | -0.67132 | 0.943375 | EAS | Cornea replaced | sense organs | |
| 365 | 0.108362 | 0.063452 | 1.707786 | 0.087676 | -0.016 | 0.232725 | EAS | Glaucoma | sense organs | |
| 365.1 | 0.084872 | 0.093172 | 0.91092 | 0.362337 | -0.09774 | 0.267486 | EAS | Open-angle glau | sense organs | |
| 365.11 | 0.149055 | 0.114242 | 1.30473 | 0.191985 | -0.07486 | 0.372966 | EAS | Primary open an | sense organs | |
| 365.2 | -0.0545 | 0.150901 | -0.36115 | 0.717988 | -0.35026 | 0.241263 | EAS | Primary angle-clo | sense organs | |
| 365.5 | 0.223396 | 0.684168 | 0.326522 | 0.744029 | -1.11755 | 1.564341 | EAS | Pseudoexfoliation | sense organs | |
| 366 | 0.02436 | 0.056356 | 0.432252 | 0.665558 | -0.0861 | 0.134817 | EAS | Cataract | sense organs | |
| 366.1 | -0.21831 | 0.30299 | -0.72053 | 0.471198 | -0.81216 | 0.375535 | EAS | Nonsenile Catara | sense organs | |
| 366.2 | 0.065704 | 0.059154 | 1.110725 | 0.266687 | -0.05024 | 0.181644 | EAS | Senile cataract | sense organs | |
| 366.3 | -0.70436 | 1.143926 | -0.61574 | 0.538067 | -2.94642 | 1.537694 | EAS | Traumatic catara | sense organs | |
| 367 | -0.07883 | 0.057273 | -1.37635 | 0.168712 | -0.19108 | 0.033425 | EAS | Disorders of refra | sense organs | |
| 367.1 | -0.15103 | 0.078923 | -1.91364 | 0.055666 | -0.30572 | 0.003656 | EAS | Myopia | sense organs | |
| 367.2 | 0.045504 | 0.084076 | 0.541225 | 0.588353 | -0.11928 | 0.21029 | EAS | Astigmatism | sense organs | |
| 367.4 | -0.06901 | 0.090804 | -0.76004 | 0.447232 | -0.24699 | 0.108958 | EAS | Presbyopia | sense organs | |
| 367.8 | 0.141498 | 0.242569 | 0.583332 | 0.55967 | -0.33393 | 0.616925 | EAS | Hypermetropia | sense organs | |
| 367.9 | -0.08988 | 0.103697 | -0.86674 | 0.386083 | -0.29312 | 0.113363 | EAS | Blindness and lov | sense organs | |
| 368 | -0.10052 | 0.060148 | -1.67117 | 0.094688 | -0.21841 | 0.01737 | EAS | Visual disturbanc | sense organs | |
| 368.1 | 0.589954 | 0.352102 | 1.675519 | 0.093832 | -0.10015 | 1.280062 | EAS | Amblyopia | sense organs | |
| 368.2 | -0.1584 | 0.140035 | -1.13111 | 0.258008 | -0.43286 | 0.116069 | EAS | Diplopia and disc | sense organs | |
| 368.3 | 0.265768 | 0.250871 | 1.059382 | 0.289426 | -0.22593 | 0.757466 | EAS | Anisometropia | sense organs | |
| 368.4 | -0.2146 | 0.149581 | -1.43465 | 0.151388 | -0.50777 | 0.078578 | EAS | Visual field defec | sense organs | |
| 368.5 | -1.79088 | 1.464732 | -1.22267 | 0.221455 | -4.6617 | 1.079938 | EAS | Color vision defic | sense organs | |

| phecode | Coef. | Std.Err. | z | p_value_z | [0.025 | 0.975] | GIA | phenotype | category | |
|---|---|---|---|---|---|---|---|---|---|---|
| 368.7 | -0.54839 | 1.043577 | -0.52549 | 0.599244 | -2.59376 | 1.496986 | EAS | Disorders of acco | sense organs | |
| 368.9 | 0.001697 | 0.139359 | 0.012177 | 0.990284 | -0.27144 | 0.274835 | EAS | Subjective visual | sense organs | |
| 368.91 | 0.22775 | 0.319744 | 0.71229 | 0.476285 | -0.39894 | 0.854437 | EAS | Psychophysical v | sense organs | |
| 369 | 0.056493 | 0.077352 | 0.730343 | 0.46518 | -0.09511 | 0.2081 | EAS | Infection of the e | sense organs | |
| 369.2 | -0.18155 | 0.158312 | -1.14678 | 0.251474 | -0.49183 | 0.128737 | EAS | Eye infection, vira | sense organs | |
| 369.5 | 0.078282 | 0.086143 | 0.908748 | 0.363483 | -0.09055 | 0.24712 | EAS | Conjunctivitis, inf | sense organs | |
| 370 | 0.151912 | 0.130814 | 1.161285 | 0.245526 | -0.10448 | 0.408303 | EAS | Keratitis | sense organs | |
| 370.1 | 0.718106 | 0.282453 | 2.542388 | 0.01101 | 0.164508 | 1.271704 | EAS | Corneal ulcer | sense organs | |
| 370.2 | 0.209026 | 0.244639 | 0.854429 | 0.392867 | -0.27046 | 0.68851 | EAS | Superficial kerati | sense organs | |
| 370.3 | 0.199894 | 0.195118 | 1.024474 | 0.305611 | -0.18253 | 0.582319 | EAS | Keratoconjunctivi | sense organs | |
| 370.31 | 0.217873 | 0.270841 | 0.804433 | 0.421147 | -0.31296 | 0.748711 | EAS | Keratoconjunctivi | sense organs | |
| 371 | -0.00212 | 0.063845 | -0.03323 | 0.973493 | -0.12726 | 0.123013 | EAS | Inflammation of t | sense organs | |
| 371.1 | 0.090846 | 0.229201 | 0.39636 | 0.691839 | -0.35838 | 0.540071 | EAS | Uveitis, noninfect | sense organs | |
| 371.2 | 0.015962 | 0.100076 | 0.159495 | 0.873279 | -0.18018 | 0.212107 | EAS | Conjunctivitis, no | sense organs | |
| 371.21 | -0.0203 | 0.106145 | -0.19122 | 0.848352 | -0.22834 | 0.187744 | EAS | Allergic conjuncti | sense organs | |
| 371.3 | -0.00641 | 0.077999 | -0.08215 | 0.93453 | -0.15928 | 0.146468 | EAS | Inflammation of e | sense organs | |
| 371.33 | -0.38979 | 0.300647 | -1.2965 | 0.194802 | -0.97905 | 0.199467 | EAS | Noninfectious de | sense organs | |
| 371.9 | 0.465053 | 1.020827 | 0.455565 | 0.648703 | -1.53573 | 2.465837 | EAS | Chronic inflamma | sense organs | |
| 372 | -0.15417 | 0.088949 | -1.73324 | 0.083053 | -0.32851 | 0.020167 | EAS | Disorders of conj | sense organs | |
| 374 | -0.02771 | 0.073232 | -0.37843 | 0.705109 | -0.17125 | 0.115819 | EAS | Other disorders c | sense organs | |
| 374.1 | 0.024709 | 0.160008 | 0.154424 | 0.877275 | -0.2889 | 0.338318 | EAS | Ectropion or entr | sense organs | |
| 374.2 | 0.02414 | 0.310613 | 0.077719 | 0.938052 | -0.58465 | 0.632931 | EAS | Lagophthalmos | sense organs | |
| 374.3 | 0.045954 | 0.111936 | 0.410537 | 0.681412 | -0.17344 | 0.265343 | EAS | Ptosis of eyelid | sense organs | |
| 374.6 | -0.06553 | 0.117026 | -0.55995 | 0.575515 | -0.29489 | 0.163838 | EAS | Dermatochalasis | sense organs | |
| 375 | 0.045136 | 0.146511 | 0.308071 | 0.758028 | -0.24202 | 0.332292 | EAS | Disorders of lacri | sense organs | |
| 375.1 | -0.10707 | 0.059493 | -1.79972 | 0.071905 | -0.22367 | 0.009534 | EAS | Dry eyes | sense organs | |
| 375.2 | 0.040167 | 0.15291 | 0.262686 | 0.792793 | -0.25953 | 0.339866 | EAS | Epiphora | sense organs | |
| 376 | -0.70682 | 0.378039 | -1.86971 | 0.061524 | -1.44777 | 0.034119 | EAS | Disorders of the c | sense organs | |
| 377 | -0.08873 | 0.122282 | -0.72563 | 0.468066 | -0.3284 | 0.150937 | EAS | Disorders of optic | sense organs | |
| 377.1 | -0.13118 | 0.150426 | -0.87205 | 0.383182 | -0.42601 | 0.16365 | EAS | Optic atrophy | sense organs | |
| 377.3 | -0.03602 | 0.208297 | -0.17295 | 0.86269 | -0.44428 | 0.372229 | EAS | Optic neuritis/neu | sense organs | |
| 378 | -0.26992 | 0.121288 | -2.22543 | 0.026052 | -0.50764 | -0.0322 | EAS | Strabismus and c | sense organs | |
| 378.1 | -0.14158 | 0.151662 | -0.93353 | 0.350546 | -0.43883 | 0.155671 | EAS | Strabismus (not s | sense organs | |
| 378.2 | -0.07634 | 0.319201 | -0.23916 | 0.810983 | -0.70196 | 0.549283 | EAS | Nystagmus and c | sense organs | |
| 378.5 | -0.51245 | 0.266354 | -1.92395 | 0.054361 | -1.0345 | 0.009592 | EAS | Paralytic strabisn | sense organs | |
| 379 | 0.034589 | 0.060464 | 0.572062 | 0.56728 | -0.08392 | 0.153096 | EAS | Other disorders c | sense organs | |
| 379.1 | 0.184946 | 0.245793 | 0.752446 | 0.451783 | -0.2968 | 0.66669 | EAS | Scleritis and epis | sense organs | |

| phecode | Coef. | Std.Err. | z | p_value_z | [0.025 | 0.975] | GIA | phenotype | category |
|---|---|---|---|---|---|---|---|---|---|
| 379.2 | -0.00404 | 0.069353 | -0.05823 | 0.953566 | -0.13997 | 0.131891 | EAS | Disorders of vitre | sense organs |
| 379.3 | 0.485853 | 0.354963 | 1.368743 | 0.17108 | -0.20986 | 1.181568 | EAS | Aphakia and othe | sense organs |
| 379.4 | -0.61736 | 0.313383 | -1.96997 | 0.048841 | -1.23157 | -0.00314 | EAS | Anomalies of pup | sense organs |
| 379.5 | 0.353693 | 0.234952 | 1.505381 | 0.132226 | -0.10681 | 0.814191 | EAS | Disorders of iris a | sense organs |
| 379.51 | -0.42282 | 0.717255 | -0.5895 | 0.555529 | -1.82861 | 0.982975 | EAS | Pigmentary iris d | sense organs |
| 379.9 | -0.0622 | 0.088775 | -0.70069 | 0.483494 | -0.2362 | 0.111792 | EAS | Pain, swelling or | sense organs |
| 380 | -0.07207 | 0.217372 | -0.33153 | 0.740244 | -0.49811 | 0.353977 | EAS | Disorders of exte | sense organs |
| 380.1 | -0.0728 | 0.123586 | -0.58908 | 0.555811 | -0.31502 | 0.169422 | EAS | Otitis externa | sense organs |
| 380.4 | -0.07434 | 0.07631 | -0.97414 | 0.329985 | -0.2239 | 0.075228 | EAS | Impacted cerume | sense organs |
| 381 | 0.100266 | 0.083092 | 1.206686 | 0.227553 | -0.06259 | 0.263122 | EAS | Otitis media and | sense organs |
| 381.1 | 0.200023 | 0.109708 | 1.823229 | 0.068269 | -0.015 | 0.415046 | EAS | Otitis media | sense organs |
| 381.11 | 0.198356 | 0.129439 | 1.532436 | 0.125415 | -0.05534 | 0.452051 | EAS | Suppurative and | sense organs |
| 381.2 | 0.010189 | 0.112982 | 0.09018 | 0.928145 | -0.21125 | 0.231629 | EAS | Eustachian tube | sense organs |
| 381.3 | 0.347196 | 0.327506 | 1.060124 | 0.289088 | -0.2947 | 0.989095 | EAS | Mastoiditis & rela | sense organs |
| 381.9 | 0.098065 | 0.203891 | 0.480965 | 0.630541 | -0.30156 | 0.497685 | EAS | Otorrhea | sense organs |
| 382 | -0.01372 | 0.095426 | -0.14383 | 0.885637 | -0.20076 | 0.173307 | EAS | Otalgia | sense organs |
| 383 | 0.686837 | 0.473326 | 1.451087 | 0.146756 | -0.24086 | 1.614538 | EAS | Otosclerosis | sense organs |
| 384 | 0.218869 | 0.174871 | 1.251606 | 0.210714 | -0.12387 | 0.56161 | EAS | Other disorders c | sense organs |
| 384.1 | 0.361243 | 0.346418 | 1.042797 | 0.297042 | -0.31772 | 1.04021 | EAS | Myringitis | sense organs |
| 384.4 | 0.140128 | 0.1943 | 0.72119 | 0.470793 | -0.24069 | 0.520949 | EAS | Perforation of tyn | sense organs |
| 385 | 0.227807 | 0.263527 | 0.864455 | 0.387338 | -0.2887 | 0.744311 | EAS | Other disorders c | sense organs |
| 385.3 | -0.59397 | 0.471102 | -1.26081 | 0.207377 | -1.51732 | 0.329373 | EAS | Cholesteatoma | sense organs |
| 385.5 | 0.672672 | 0.389517 | 1.72694 | 0.084178 | -0.09077 | 1.436111 | EAS | Tympanosclerosi | sense organs |
| 386 | -0.10386 | 0.092364 | -1.12451 | 0.260796 | -0.28489 | 0.077166 | EAS | Vertiginous syndı | sense organs |
| 386.1 | 0.205425 | 0.24598 | 0.835126 | 0.403647 | -0.27669 | 0.687537 | EAS | Meniere's diseas | sense organs |
| 386.2 | -0.1317 | 0.110928 | -1.18726 | 0.235126 | -0.34912 | 0.085715 | EAS | Peripheral or cen | sense organs |
| 386.21 | 0.294016 | 0.661261 | 0.444629 | 0.656588 | -1.00203 | 1.590064 | EAS | Central origin ver | sense organs |
| 386.3 | 0.019445 | 0.243722 | 0.079785 | 0.936409 | -0.45824 | 0.497131 | EAS | Labyrinthitis | sense organs |
| 386.9 | 0.005563 | 0.053018 | 0.10493 | 0.916431 | -0.09835 | 0.109476 | EAS | Dizziness and gi | sense organs |
| 388 | -0.00849 | 0.095555 | -0.08886 | 0.929197 | -0.19577 | 0.178794 | EAS | Other disorders c | sense organs |
| 389 | -0.02917 | 0.055435 | -0.52611 | 0.598811 | -0.13782 | 0.079486 | EAS | Hearing loss | sense organs |
| 389.1 | -0.0451 | 0.074388 | -0.60623 | 0.544361 | -0.19089 | 0.100701 | EAS | Sensorineural he | sense organs |
| 389.2 | -0.26906 | 0.188102 | -1.43039 | 0.152606 | -0.63773 | 0.099614 | EAS | Conductive heari | sense organs |
| 389.3 | -0.01345 | 0.283527 | -0.04745 | 0.962157 | -0.56916 | 0.54225 | EAS | Degenerative an | sense organs |
| 389.4 | -0.07154 | 0.08491 | -0.84258 | 0.399464 | -0.23797 | 0.094878 | EAS | Tinnitus | sense organs |
| 389.5 | 0.494971 | 0.198345 | 2.495507 | 0.012578 | 0.106222 | 0.883719 | EAS | Disorders of acou | sense organs |
| 394 | 0.01169 | 0.084854 | 0.137767 | 0.890425 | -0.15462 | 0.178001 | EAS | Rheumatic disea | circulatory system |

| phecode | Coef. | Std.Err. | z | p_value_z | [0.025 | 0.975] | GIA | phenotype | category |
|---|---|---|---|---|---|---|---|---|---|
| 394.1 | -0.03903 | 0.246631 | -0.15826 | 0.874249 | -0.52242 | 0.444355 | EAS | Mitral valve stenc | circulatory system |
| 394.2 | -0.13878 | 0.133492 | -1.03965 | 0.298503 | -0.40042 | 0.122854 | EAS | Mitral valve disea | circulatory system |
| 394.3 | -0.59732 | 0.331297 | -1.80297 | 0.071393 | -1.24665 | 0.052012 | EAS | Aortic valve disea | circulatory system |
| 394.4 | 0.757299 | 0.547208 | 1.383931 | 0.16638 | -0.31521 | 1.829807 | EAS | Acute rheumatic | circulatory system |
| 394.7 | 0.013729 | 0.126117 | 0.108859 | 0.913314 | -0.23346 | 0.260914 | EAS | Disease of tricus | circulatory system |
| 395 | 0.117071 | 0.062805 | 1.864046 | 0.062315 | -0.00602 | 0.240167 | EAS | Heart valve disor | circulatory system |
| 395.1 | -0.06103 | 0.084341 | -0.72362 | 0.469297 | -0.22634 | 0.104275 | EAS | Nonrheumatic mi | circulatory system |
| 395.2 | 0.133898 | 0.085919 | 1.55843 | 0.119131 | -0.0345 | 0.302295 | EAS | Nonrheumatic ac | circulatory system |
| 395.3 | -0.02086 | 0.111218 | -0.18756 | 0.851218 | -0.23884 | 0.197123 | EAS | Nonrheumatic tri | circulatory system |
| 395.4 | 0.323608 | 0.188284 | 1.718722 | 0.085665 | -0.04542 | 0.692638 | EAS | Nonrheumatic pu | circulatory system |
| 395.6 | 0.107107 | 0.129019 | 0.830161 | 0.406448 | -0.14577 | 0.35998 | EAS | Heart valve repla | circulatory system |
| 396 | 0.021957 | 0.072764 | 0.301762 | 0.762834 | -0.12066 | 0.164571 | EAS | Abnormal heart s | circulatory system |
| 401 | -0.04968 | 0.049982 | -0.99385 | 0.320296 | -0.14764 | 0.048289 | EAS | Hypertension | circulatory system |
| 401.1 | -0.04371 | 0.049788 | -0.87787 | 0.380012 | -0.14129 | 0.053875 | EAS | Essential hyperte | circulatory system |
| 401.2 | 0.033264 | 0.061349 | 0.542208 | 0.587675 | -0.08698 | 0.153505 | EAS | Hypertensive hea | circulatory system |
| 401.21 | 0.069532 | 0.096203 | 0.722766 | 0.469824 | -0.11902 | 0.258086 | EAS | Hypertensive hea | circulatory system |
| 401.22 | -0.01569 | 0.068704 | -0.2284 | 0.819332 | -0.15035 | 0.118965 | EAS | Hypertensive chr | circulatory system |
| 401.3 | 0.026738 | 0.069744 | 0.383372 | 0.701444 | -0.10996 | 0.163433 | EAS | Other hypertensi | circulatory system |
| 402 | -0.22234 | 0.071006 | -3.13125 | 0.001741 | -0.36151 | -0.08317 | EAS | Elevated blood p | circulatory system |
| 411 | 0.11713 | 0.056399 | 2.076824 | 0.037818 | 0.006591 | 0.22767 | EAS | Ischemic Heart D | circulatory system |
| 411.1 | -0.12797 | 0.156995 | -0.81513 | 0.414998 | -0.43568 | 0.179733 | EAS | Unstable angina | circulatory system |
| 411.2 | 0.112307 | 0.0882 | 1.273329 | 0.202901 | -0.06056 | 0.285176 | EAS | Myocardial infarc | circulatory system |
| 411.3 | 0.205281 | 0.087004 | 2.359456 | 0.018302 | 0.034757 | 0.375806 | EAS | Angina pectoris | circulatory system |
| 411.4 | 0.209234 | 0.059685 | 3.505605 | 0.000456 | 0.092252 | 0.326215 | EAS | Coronary atheros | circulatory system |
| 411.41 | -0.19122 | 0.330428 | -0.5787 | 0.562789 | -0.83885 | 0.456408 | EAS | Aneurysm and di | circulatory system |
| 411.8 | 0.119185 | 0.10006 | 1.191138 | 0.2336 | -0.07693 | 0.315299 | EAS | Other chronic isc | circulatory system |
| 411.9 | -0.19347 | 0.175544 | -1.1021 | 0.270417 | -0.53753 | 0.150592 | EAS | Other acute and | circulatory system |
| 414 | 0.076967 | 0.098533 | 0.781137 | 0.434722 | -0.11615 | 0.270088 | EAS | Other forms of ch | circulatory system |
| 414.2 | 0.327928 | 0.22745 | 1.44176 | 0.14937 | -0.11787 | 0.773722 | EAS | ASCVD | circulatory system |
| 415 | 0.003616 | 0.082073 | 0.04406 | 0.964856 | -0.15724 | 0.164476 | EAS | Pulmonary heart | circulatory system |
| 415.1 | -0.07713 | 0.143147 | -0.53884 | 0.589996 | -0.3577 | 0.203429 | EAS | Acute pulmonary | circulatory system |
| 415.11 | -0.07713 | 0.143147 | -0.53884 | 0.589996 | -0.3577 | 0.203429 | EAS | Pulmonary embo | circulatory system |
| 415.2 | -0.01692 | 0.096907 | -0.17458 | 0.861412 | -0.20685 | 0.173016 | EAS | Chronic pulmona | circulatory system |
| 415.21 | -0.21073 | 0.17054 | -1.23564 | 0.216591 | -0.54498 | 0.123526 | EAS | Primary pulmona | circulatory system |
| 416 | 0.133616 | 0.062702 | 2.130979 | 0.033091 | 0.010723 | 0.256508 | EAS | Cardiomegaly | circulatory system |
| 418 | 0.018652 | 0.045438 | 0.410487 | 0.681449 | -0.0704 | 0.107707 | EAS | Nonspecific ches | circulatory system |
| 418.1 | -0.02506 | 0.110641 | -0.22647 | 0.820839 | -0.24191 | 0.191796 | EAS | Precordial pain | circulatory system |

| phecode | Coef. | Std.Err. | z | p_value_z | [0.025 | 0.975] | GIA | phenotype | category |
|---|---|---|---|---|---|---|---|---|---|
| 420 | -0.11692 | 0.089206 | -1.31066 | 0.189973 | -0.29176 | 0.057922 | EAS | Carditis | circulatory system |
| 420.1 | 0.226392 | 0.29658 | 0.763341 | 0.44526 | -0.35489 | 0.807679 | EAS | Myocarditis | circulatory system |
| 420.2 | -0.09143 | 0.10017 | -0.91277 | 0.361363 | -0.28776 | 0.104897 | EAS | Pericarditis | circulatory system |
| 420.21 | -0.1049 | 0.305544 | -0.34333 | 0.731348 | -0.70376 | 0.493952 | EAS | Acute pericarditis | circulatory system |
| 420.22 | -0.29978 | 0.264247 | -1.13449 | 0.25659 | -0.8177 | 0.218129 | EAS | Chronic pericardi | circulatory system |
| 420.3 | -0.25125 | 0.17855 | -1.40716 | 0.15938 | -0.6012 | 0.098703 | EAS | Endocarditis | circulatory system |
| 425 | 0.052723 | 0.089837 | 0.586868 | 0.557293 | -0.12336 | 0.2288 | EAS | Cardiomyopathy | circulatory system |
| 425.1 | 0.049305 | 0.094955 | 0.519244 | 0.60359 | -0.1368 | 0.235412 | EAS | Primary/intrinsic | circulatory system |
| 425.11 | 0.121959 | 0.272076 | 0.448252 | 0.653971 | -0.4113 | 0.655217 | EAS | Hypertrophic obs | circulatory system |
| 425.12 | -0.27975 | 0.231199 | -1.21 | 0.226279 | -0.73289 | 0.173391 | EAS | Other hypertroph | circulatory system |
| 425.2 | -0.03653 | 0.157212 | -0.23233 | 0.81628 | -0.34466 | 0.271605 | EAS | Secondary/extrin | circulatory system |
| 425.8 | 0.300394 | 0.364493 | 0.82414 | 0.40986 | -0.414 | 1.014787 | EAS | Other cardiomyo | circulatory system |
| 426 | 0.035848 | 0.051893 | 0.690803 | 0.48969 | -0.06586 | 0.137556 | EAS | Cardiac conducti | circulatory system |
| 426.2 | 0.10743 | 0.113265 | 0.948482 | 0.342884 | -0.11457 | 0.329424 | EAS | Atrioventricular [ | circulatory system |
| 426.21 | 0.069693 | 0.156362 | 0.445717 | 0.655802 | -0.23677 | 0.376157 | EAS | First degree AV b | circulatory system |
| 426.22 | -0.44392 | 0.601663 | -0.73782 | 0.460625 | -1.62316 | 0.73532 | EAS | Mobitz II AV bloc | circulatory system |
| 426.23 | 0.576451 | 0.242838 | 2.373814 | 0.017605 | 0.100498 | 1.052404 | EAS | Second degree A | circulatory system |
| 426.24 | 0.238645 | 0.181529 | 1.31464 | 0.188631 | -0.11715 | 0.594436 | EAS | Atrioventricular b | circulatory system |
| 426.25 | -0.1986 | 0.278936 | -0.71198 | 0.47648 | -0.7453 | 0.348109 | EAS | Other heart block | circulatory system |
| 426.3 | 0.135816 | 0.106146 | 1.279517 | 0.200715 | -0.07223 | 0.343859 | EAS | Bundle branch bl | circulatory system |
| 426.31 | 0.106394 | 0.119216 | 0.89245 | 0.372152 | -0.12726 | 0.340052 | EAS | Right bundle bra | circulatory system |
| 426.32 | 0.177455 | 0.224215 | 0.791452 | 0.42868 | -0.262 | 0.616908 | EAS | Left bundle branc | circulatory system |
| 426.4 | -0.26107 | 0.437552 | -0.59665 | 0.550742 | -1.11865 | 0.596522 | EAS | Anomalous atrio | circulatory system |
| 426.7 | 0.032772 | 0.054666 | 0.599493 | 0.548844 | -0.07437 | 0.139915 | EAS | Abnormal electro | circulatory system |
| 426.8 | 0.419203 | 0.234284 | 1.789293 | 0.073568 | -0.03999 | 0.878391 | EAS | Other cardiac co | circulatory system |
| 426.9 | 0.10012 | 0.098765 | 1.013717 | 0.310718 | -0.09346 | 0.293697 | EAS | Cardiac pacemal | circulatory system |
| 426.91 | 0.136171 | 0.110482 | 1.232517 | 0.217756 | -0.08037 | 0.352711 | EAS | Cardiac pacemal | circulatory system |
| 426.92 | 0.315581 | 0.141421 | 2.231491 | 0.025649 | 0.0384 | 0.592761 | EAS | Cardiac defibrilla | circulatory system |
| 427 | 0.008172 | 0.044456 | 0.183813 | 0.85416 | -0.07896 | 0.095304 | EAS | Cardiac dysrhyth | circulatory system |
| 427.1 | -0.02762 | 0.079728 | -0.34641 | 0.729037 | -0.18388 | 0.128646 | EAS | Paroxysmal tach | circulatory system |
| 427.11 | -0.00717 | 0.099051 | -0.07243 | 0.94226 | -0.20131 | 0.186962 | EAS | Paroxysmal supr | circulatory system |
| 427.12 | 0.025253 | 0.116098 | 0.217517 | 0.827805 | -0.2023 | 0.252802 | EAS | Paroxysmal vent | circulatory system |
| 427.2 | 0.01794 | 0.074498 | 0.240808 | 0.809704 | -0.12807 | 0.163952 | EAS | Atrial fibrillation a | circulatory system |
| 427.21 | 0.004858 | 0.076402 | 0.06358 | 0.949304 | -0.14489 | 0.154604 | EAS | Atrial fibrillation | circulatory system |
| 427.22 | -0.01744 | 0.110703 | -0.15753 | 0.87483 | -0.23441 | 0.199536 | EAS | Atrial flutter | circulatory system |
| 427.3 | 0.008102 | 0.06626 | 0.122281 | 0.902677 | -0.12177 | 0.13797 | EAS | Other specified c | circulatory system |
| 427.4 | 0.120753 | 0.152212 | 0.793322 | 0.42759 | -0.17758 | 0.419082 | EAS | Cardiac arrest ar | circulatory system |

| phecode | Coef. | Std.Err. | z | p_value_z | [0.025 | 0.975] | GIA | phenotype | category |
|---|---|---|---|---|---|---|---|---|---|
| 427.41 | 0.370527 | 0.219598 | 1.687295 | 0.091547 | -0.05988 | 0.800932 | EAS | Ventricular fibrilla | circulatory system |
| 427.42 | 0.027182 | 0.202122 | 0.134484 | 0.89302 | -0.36897 | 0.423335 | EAS | Cardiac arrest | circulatory system |
| 427.5 | -0.01842 | 0.094752 | -0.19439 | 0.845871 | -0.20413 | 0.167291 | EAS | Arrhythmia (cardi | circulatory system |
| 427.6 | 0.077265 | 0.08609 | 0.89749 | 0.369458 | -0.09147 | 0.245998 | EAS | Premature beats | circulatory system |
| 427.61 | 0.111824 | 0.155678 | 0.718306 | 0.472568 | -0.1933 | 0.416947 | EAS | Supraventricular | circulatory system |
| 427.7 | -0.03963 | 0.070603 | -0.56137 | 0.574544 | -0.17801 | 0.098744 | EAS | Tachycardia NOS | circulatory system |
| 427.8 | -0.1287 | 0.120548 | -1.06758 | 0.285708 | -0.36497 | 0.107575 | EAS | Sinoatrial node d | circulatory system |
| 427.9 | 0.070395 | 0.059687 | 1.179389 | 0.238243 | -0.04659 | 0.18738 | EAS | Palpitations | circulatory system |
| 428 | 0.077615 | 0.075633 | 1.026204 | 0.304796 | -0.07062 | 0.225852 | EAS | Congestive heart | circulatory system |
| 428.1 | 0.175759 | 0.08054 | 2.182245 | 0.029091 | 0.017903 | 0.333614 | EAS | Congestive heart | circulatory system |
| 428.2 | 0.048161 | 0.109257 | 0.440809 | 0.659351 | -0.16598 | 0.262301 | EAS | Heart failure NOS | circulatory system |
| 428.3 | 0.161077 | 0.10013 | 1.608685 | 0.107685 | -0.03517 | 0.357328 | EAS | Heart failure with | circulatory system |
| 428.4 | 0.116066 | 0.104462 | 1.111087 | 0.266531 | -0.08868 | 0.320808 | EAS | Heart failure with | circulatory system |
| 429 | 0.139607 | 0.057814 | 2.414763 | 0.015745 | 0.026294 | 0.25292 | EAS | Ill-defined descri | circulatory system |
| 429.1 | 0.116749 | 0.115571 | 1.010191 | 0.312404 | -0.10977 | 0.343265 | EAS | Heart transplant/ | circulatory system |
| 429.2 | 0.116199 | 0.107305 | 1.082886 | 0.278859 | -0.09411 | 0.326512 | EAS | Abnormal functio | circulatory system |
| 429.3 | 0.115455 | 0.063883 | 1.807292 | 0.070717 | -0.00975 | 0.240663 | EAS | Symptoms involv | circulatory system |
| 429.9 | 0.394081 | 0.178074 | 2.213023 | 0.026896 | 0.045063 | 0.7431 | EAS | Cardiac complica | circulatory system |
| 430 | -0.10563 | 0.12489 | -0.84577 | 0.397681 | -0.35041 | 0.139152 | EAS | Intracranial hem | circulatory system |
| 430.1 | 0.031315 | 0.19129 | 0.163707 | 0.869962 | -0.34361 | 0.406236 | EAS | Subarachnoid he | circulatory system |
| 430.2 | -0.26648 | 0.17669 | -1.50817 | 0.13151 | -0.61279 | 0.079827 | EAS | Intracerebral hen | circulatory system |
| 430.3 | -0.00325 | 0.180578 | -0.01801 | 0.985628 | -0.35718 | 0.350673 | EAS | Subdural hemorr | circulatory system |
| 433 | 0.008411 | 0.06165 | 0.136426 | 0.891484 | -0.11242 | 0.129243 | EAS | Cerebrovascular | circulatory system |
| 433.1 | 0.047792 | 0.082803 | 0.577181 | 0.563817 | -0.1145 | 0.210084 | EAS | Occlusion and st | circulatory system |
| 433.11 | 0.058609 | 0.183508 | 0.31938 | 0.749439 | -0.30106 | 0.418279 | EAS | Occlusion of cere | circulatory system |
| 433.12 | 0.191513 | 0.214709 | 0.891969 | 0.37241 | -0.22931 | 0.612335 | EAS | Cerebral atheros | circulatory system |
| 433.2 | -0.04498 | 0.089255 | -0.50396 | 0.614293 | -0.21992 | 0.129956 | EAS | Occlusion of cere | circulatory system |
| 433.21 | -0.02894 | 0.089978 | -0.3216 | 0.747759 | -0.20529 | 0.147418 | EAS | Cerebral artery o | circulatory system |
| 433.3 | 0.039176 | 0.075467 | 0.519115 | 0.603681 | -0.10874 | 0.18709 | EAS | Cerebral ischemi | circulatory system |
| 433.31 | 0.107572 | 0.082046 | 1.311121 | 0.189817 | -0.05324 | 0.26838 | EAS | Transient cerebra | circulatory system |
| 433.32 | 0.581256 | 0.316235 | 1.838049 | 0.066055 | -0.03855 | 1.201065 | EAS | Moyamoya disea | circulatory system |
| 433.5 | -0.15279 | 0.158774 | -0.96229 | 0.335905 | -0.46398 | 0.158405 | EAS | Cerebral aneurys | circulatory system |
| 433.6 | -0.32648 | 0.635978 | -0.51336 | 0.607702 | -1.57298 | 0.92001 | EAS | Acute, but ill-defi | circulatory system |
| 433.8 | -0.21899 | 0.144571 | -1.51476 | 0.129832 | -0.50235 | 0.064363 | EAS | Late effects of ce | circulatory system |
| 440 | 0.046204 | 0.066734 | 0.69236 | 0.488711 | -0.08459 | 0.177001 | EAS | Atherosclerosis | circulatory system |
| 440.1 | -0.55297 | 0.249453 | -2.21675 | 0.02664 | -1.04189 | -0.06406 | EAS | Atherosclerosis c | circulatory system |
| 440.2 | 0.29553 | 0.151155 | 1.95515 | 0.050565 | -0.00073 | 0.591788 | EAS | Atherosclerosis c | circulatory system |

| phecode | Coef. | Std.Err. | z | p_value_z | [0.025 | 0.975] | GIA | phenotype | category |
|---|---|---|---|---|---|---|---|---|---|
| 440.21 | 0.484135 | 0.268287 | 1.804542 | 0.071146 | -0.0417 | 1.009968 | EAS | Atherosclerosis c | circulatory system |
| 440.22 | 0.328956 | 0.219919 | 1.495808 | 0.134704 | -0.10208 | 0.759989 | EAS | Atherosclerosis c | circulatory system |
| 440.9 | 0.029311 | 0.071629 | 0.409205 | 0.682389 | -0.11108 | 0.169702 | EAS | Atherosclerosis c | circulatory system |
| 441 | 0.022183 | 0.179691 | 0.123454 | 0.901748 | -0.33 | 0.374371 | EAS | Vascular insuffici | circulatory system |
| 441.1 | -0.30569 | 0.309752 | -0.98688 | 0.323704 | -0.91279 | 0.301416 | EAS | Acute vascular in | circulatory system |
| 441.2 | 0.299303 | 0.267311 | 1.119681 | 0.26285 | -0.22462 | 0.823222 | EAS | Chronic vascular | circulatory system |
| 442 | -0.09696 | 0.096079 | -1.00916 | 0.3129 | -0.28527 | 0.091352 | EAS | Other aneurysm | circulatory system |
| 442.1 | -0.01342 | 0.119408 | -0.11242 | 0.910487 | -0.24746 | 0.220611 | EAS | Aortic aneurysm | circulatory system |
| 442.11 | 0.128056 | 0.178508 | 0.717369 | 0.473147 | -0.22181 | 0.477924 | EAS | Abdominal aortic | circulatory system |
| 442.2 | -0.02038 | 0.285657 | -0.07135 | 0.943116 | -0.58026 | 0.539495 | EAS | Aneurysm of iliac | circulatory system |
| 442.3 | -0.12145 | 0.324104 | -0.37473 | 0.707865 | -0.75668 | 0.513782 | EAS | Aneurysm of arte | circulatory system |
| 442.4 | 0.326061 | 0.266084 | 1.225407 | 0.220422 | -0.19545 | 0.847576 | EAS | Arterial dissectio | circulatory system |
| 442.8 | -0.13156 | 0.199929 | -0.65803 | 0.51052 | -0.52341 | 0.260295 | EAS | Aneurysm of othe | circulatory system |
| 443 | 0.029029 | 0.087808 | 0.330595 | 0.740951 | -0.14307 | 0.201129 | EAS | Peripheral vascu | circulatory system |
| 443.1 | 0.075155 | 0.187237 | 0.401389 | 0.688133 | -0.29182 | 0.442134 | EAS | Raynaud's syndr | circulatory system |
| 443.7 | 0.12673 | 0.157266 | 0.805831 | 0.42034 | -0.18151 | 0.434965 | EAS | Peripheral angio | circulatory system |
| 443.8 | 0.435476 | 0.212768 | 2.046713 | 0.040686 | 0.018457 | 0.852494 | EAS | Other specified p | circulatory system |
| 443.9 | 0.014963 | 0.108403 | 0.13803 | 0.890217 | -0.1975 | 0.22743 | EAS | Peripheral vascu | circulatory system |
| 444 | -0.04216 | 0.190325 | -0.2215 | 0.824705 | -0.41519 | 0.330874 | EAS | Arterial embolism | circulatory system |
| 444.1 | -0.03797 | 0.268174 | -0.14157 | 0.88742 | -0.56358 | 0.487647 | EAS | Arterial embolism | circulatory system |
| 444.2 | -0.74601 | 0.470014 | -1.58721 | 0.112465 | -1.66722 | 0.175199 | EAS | Embolism and th | circulatory system |
| 446 | 0.13129 | 0.180757 | 0.726338 | 0.467631 | -0.22299 | 0.485567 | EAS | Polyarteritis nodo | circulatory system |
| 446.2 | 0.675044 | 1.069951 | 0.630911 | 0.528099 | -1.42202 | 2.77211 | EAS | Acute febrile muc | circulatory system |
| 446.3 | 0.80094 | 0.690209 | 1.16043 | 0.245874 | -0.55185 | 2.153725 | EAS | Hypersensitivity a | circulatory system |
| 446.4 | -0.52657 | 0.406782 | -1.29448 | 0.195499 | -1.32385 | 0.270706 | EAS | Wegener's granu | circulatory system |
| 446.5 | 0.226099 | 0.460191 | 0.491316 | 0.623203 | -0.67586 | 1.128057 | EAS | Giant cell arteritis | circulatory system |
| 446.6 | 0.232611 | 0.977754 | 0.237903 | 0.811956 | -1.68375 | 2.148974 | EAS | Polyarteritis nodo | circulatory system |
| 446.7 | 0.690416 | 0.778613 | 0.886726 | 0.375227 | -0.83564 | 2.21647 | EAS | Takayasu's disea | circulatory system |
| 446.8 | 0.374135 | 0.392973 | 0.952062 | 0.341065 | -0.39608 | 1.144348 | EAS | Thrombotic micro | circulatory system |
| 446.9 | 0.001422 | 0.243647 | 0.005835 | 0.995345 | -0.47612 | 0.478962 | EAS | Arteritis NOS | circulatory system |
| 447 | -0.05846 | 0.083082 | -0.7036 | 0.48168 | -0.22129 | 0.104381 | EAS | Other disorders c | circulatory system |
| 447.1 | -0.07329 | 0.150679 | -0.48638 | 0.626695 | -0.36861 | 0.222037 | EAS | Stricture of artery | circulatory system |
| 447.7 | 0.0021 | 0.137605 | 0.015259 | 0.987826 | -0.2676 | 0.2718 | EAS | Aortic ectasia | circulatory system |
| 448 | -0.93599 | 0.305688 | -3.0619 | 0.002199 | -1.53512 | -0.33685 | EAS | Disease of capilla | circulatory system |
| 450 | -0.15609 | 0.166953 | -0.93492 | 0.349828 | -0.48331 | 0.171134 | EAS | Noninfectious dis | circulatory system |
| 451 | -0.16659 | 0.256605 | -0.64919 | 0.516215 | -0.66952 | 0.336351 | EAS | Phlebitis and thro | circulatory system |
| 451.2 | -1.09271 | 0.608758 | -1.79498 | 0.072657 | -2.28585 | 0.100434 | EAS | Phlebitis and thro | circulatory system |

| phecode | Coef. | Std.Err. | z | p_value_z | [0.025 | 0.975] | GIA | phenotype | category |
|---|---|---|---|---|---|---|---|---|---|
| 452 | 0.016811 | 0.082184 | 0.204558 | 0.837917 | -0.14427 | 0.177888 | EAS | Other venous em | circulatory system |
| 452.2 | -0.02357 | 0.108855 | -0.21652 | 0.828582 | -0.23692 | 0.189782 | EAS | Deep vein thromb | circulatory system |
| 452.8 | 0.124224 | 0.438161 | 0.283513 | 0.776784 | -0.73456 | 0.983005 | EAS | Postphlebitic syn | circulatory system |
| 453 | -0.4471 | 0.3327 | -1.34387 | 0.178992 | -1.09918 | 0.204976 | EAS | Chronic venous h | circulatory system |
| 454 | 0.035098 | 0.104338 | 0.336391 | 0.736576 | -0.1694 | 0.239598 | EAS | Varicose veins | circulatory system |
| 454.1 | 0.242903 | 0.125947 | 1.928616 | 0.053779 | -0.00395 | 0.489754 | EAS | Varicose veins of | circulatory system |
| 454.11 | 0.273567 | 0.147422 | 1.855668 | 0.063501 | -0.01538 | 0.562509 | EAS | Varicose veins of | circulatory system |
| 455 | -0.06248 | 0.04891 | -1.27741 | 0.201457 | -0.15834 | 0.033384 | EAS | Hemorrhoids | circulatory system |
| 456 | 0.033228 | 0.119147 | 0.278886 | 0.780333 | -0.2003 | 0.266753 | EAS | Chronic venous i | circulatory system |
| 457 | 0.099058 | 0.057271 | 1.729642 | 0.083694 | -0.01319 | 0.211306 | EAS | Encounter for lon | circulatory system |
| 457.2 | 0.234205 | 0.119725 | 1.956192 | 0.050442 | -0.00045 | 0.468862 | EAS | Encounter for lon | circulatory system |
| 457.3 | 0.068517 | 0.056971 | 1.202661 | 0.229108 | -0.04314 | 0.180178 | EAS | Encounter for lon | circulatory system |
| 458 | -0.05728 | 0.065716 | -0.87163 | 0.383408 | -0.18608 | 0.07152 | EAS | Hypotension | circulatory system |
| 458.1 | -0.01978 | 0.120172 | -0.16457 | 0.869282 | -0.25531 | 0.215756 | EAS | Orthostatic hypot | circulatory system |
| 458.2 | -0.03893 | 0.131654 | -0.29569 | 0.767463 | -0.29697 | 0.219107 | EAS | Iatrogenic hypote | circulatory system |
| 458.9 | -0.03894 | 0.075299 | -0.51716 | 0.605042 | -0.18653 | 0.108641 | EAS | Hypotension NOS | circulatory system |
| 459 | -0.07492 | 0.062351 | -1.20165 | 0.229499 | -0.19713 | 0.047281 | EAS | Other disorders c | circulatory system |
| 459.1 | -0.12447 | 0.14712 | -0.84604 | 0.397528 | -0.41282 | 0.16388 | EAS | Hemorrhage NOS | circulatory system |
| 459.7 | 0.041054 | 0.108234 | 0.379308 | 0.704459 | -0.17108 | 0.253189 | EAS | Blood vessel repl | circulatory system |
| 459.9 | -0.07904 | 0.073101 | -1.08126 | 0.279582 | -0.22232 | 0.064234 | EAS | Circulatory disea | circulatory system |
| 464 | 0.039079 | 0.079507 | 0.491517 | 0.623061 | -0.11675 | 0.19491 | EAS | Acute sinusitis | respiratory |
| 465 | 0.008251 | 0.047583 | 0.173398 | 0.862339 | -0.08501 | 0.101511 | EAS | Acute upper resp | respiratory |
| 465.2 | 0.017845 | 0.065103 | 0.27411 | 0.784 | -0.10975 | 0.145445 | EAS | Acute pharyngitis | respiratory |
| 465.4 | 0.146404 | 0.1809 | 0.80931 | 0.418337 | -0.20815 | 0.500962 | EAS | Acute laryngitis a | respiratory |
| 470 | -0.15268 | 0.097645 | -1.56362 | 0.117906 | -0.34406 | 0.038701 | EAS | Septal Deviations | respiratory |
| 471 | 0.028972 | 0.165902 | 0.174634 | 0.861367 | -0.29619 | 0.354134 | EAS | Nasal polyps | respiratory |
| 472 | 0.048866 | 0.080635 | 0.606012 | 0.544507 | -0.10918 | 0.206908 | EAS | Chronic pharyngi | respiratory |
| 473 | 0.043948 | 0.074012 | 0.593792 | 0.552651 | -0.10111 | 0.189008 | EAS | Diseases of the la | respiratory |
| 473.1 | -0.19556 | 0.224143 | -0.87247 | 0.38295 | -0.63487 | 0.243754 | EAS | Chronic laryngitis | respiratory |
| 473.3 | -0.05518 | 0.174054 | -0.31702 | 0.751229 | -0.39632 | 0.285961 | EAS | Paralysis/spasm | respiratory |
| 473.4 | 0.04339 | 0.084865 | 0.511282 | 0.609153 | -0.12294 | 0.209722 | EAS | Voice disturbanc | respiratory |
| 474 | -0.10136 | 0.135292 | -0.74916 | 0.453759 | -0.36652 | 0.163811 | EAS | Acute and chroni | respiratory |
| 474.1 | -0.18232 | 0.248881 | -0.73254 | 0.463838 | -0.67011 | 0.305482 | EAS | Acute tonsillitis | respiratory |
| 474.2 | -0.0976 | 0.145319 | -0.67161 | 0.501832 | -0.38242 | 0.187222 | EAS | Chronic tonsillitis | respiratory |
| 475 | -0.03688 | 0.061207 | -0.60251 | 0.546835 | -0.15684 | 0.083086 | EAS | Chronic sinusitis | respiratory |
| 475.9 | 0.007905 | 0.07659 | 0.103214 | 0.917793 | -0.14221 | 0.158019 | EAS | Postnasal drip | respiratory |
| 476 | -0.04841 | 0.050099 | -0.96627 | 0.333909 | -0.1466 | 0.049783 | EAS | Allergic rhinitis | respiratory |

| phecode | Coef. | Std.Err. | z | p_value_z | [0.025 | 0.975] | GIA | phenotype | category | |
|---|---|---|---|---|---|---|---|---|---|---|
| 477 | -0.16376 | 0.099345 | -1.64845 | 0.09926 | -0.35848 | 0.030947 | EAS | Epistaxis or throa | respiratory | |
| 478 | -0.04442 | 0.122126 | -0.36372 | 0.71607 | -0.28378 | 0.194943 | EAS | Throat pain | respiratory | |
| 479 | -0.07109 | 0.0555 | -1.28087 | 0.200238 | -0.17987 | 0.037689 | EAS | Other upper resp | respiratory | |
| 480 | 0.077467 | 0.060155 | 1.287793 | 0.197818 | -0.04043 | 0.195369 | EAS | Pneumonia | respiratory | |
| 480.1 | 0.165983 | 0.097015 | 1.710906 | 0.087099 | -0.02416 | 0.356129 | EAS | Bacterial pneumo | respiratory | |
| 480.11 | 0.045668 | 0.14257 | 0.320322 | 0.748724 | -0.23376 | 0.325099 | EAS | Pneumococcal p | respiratory | |
| 480.12 | 0.221352 | 0.406782 | 0.544154 | 0.586336 | -0.57593 | 1.01863 | EAS | Pseudomonal pn | respiratory | |
| 480.13 | 0.160933 | 0.48839 | 0.329518 | 0.741764 | -0.79629 | 1.118159 | EAS | MRSA pneumoni | respiratory | |
| 480.2 | 0.181504 | 0.118424 | 1.532655 | 0.125361 | -0.0506 | 0.413611 | EAS | Viral pneumonia | respiratory | |
| 480.3 | -0.35371 | 0.239809 | -1.47495 | 0.140227 | -0.82372 | 0.116311 | EAS | Pneumonia due t | respiratory | |
| 480.5 | -0.03069 | 0.174413 | -0.17596 | 0.860322 | -0.37253 | 0.311152 | EAS | Bronchopneumo | respiratory | |
| 481 | 0.283078 | 0.107141 | 2.642114 | 0.008239 | 0.073086 | 0.493071 | EAS | Influenza | respiratory | |
| 483 | -0.02547 | 0.097068 | -0.26237 | 0.793039 | -0.21572 | 0.164782 | EAS | Acute bronchitis | respiratory | |
| 495 | 0.11149 | 0.055825 | 1.997116 | 0.045813 | 0.002074 | 0.220905 | EAS | Asthma | respiratory | |
| 495.1 | 0.16407 | 0.201591 | 0.813872 | 0.415718 | -0.23104 | 0.559181 | EAS | Chronic obstructi | respiratory | |
| 495.11 | 0.409802 | 0.679839 | 0.602793 | 0.546647 | -0.92266 | 1.742261 | EAS | Chronic obstructi | respiratory | |
| 495.2 | 0.109186 | 0.107702 | 1.013772 | 0.310692 | -0.10191 | 0.320278 | EAS | Asthma with exa | respiratory | |
| 496 | 0.095581 | 0.071849 | 1.330305 | 0.183418 | -0.04524 | 0.236401 | EAS | Chronic airway o | respiratory | |
| 496.1 | 0.167588 | 0.116062 | 1.443954 | 0.148752 | -0.05989 | 0.395065 | EAS | Emphysema | respiratory | |
| 496.2 | 0.273968 | 0.143688 | 1.906686 | 0.056561 | -0.00766 | 0.555592 | EAS | Chronic bronchiti | respiratory | |
| 496.21 | 0.681984 | 0.202164 | 3.373418 | 0.000742 | 0.285749 | 1.078218 | EAS | Obstructive chro | respiratory | |
| 496.3 | -0.06366 | 0.097069 | -0.65585 | 0.511922 | -0.25391 | 0.126589 | EAS | Bronchiectasis | respiratory | |
| 497 | 0.058832 | 0.092458 | 0.636304 | 0.524579 | -0.12238 | 0.240047 | EAS | Bronchitis | respiratory | |
| 498 | -0.15223 | 0.224468 | -0.67819 | 0.49765 | -0.59218 | 0.287717 | EAS | Acute bronchosp | respiratory | |
| 499 | -0.2808 | 0.615983 | -0.45585 | 0.648496 | -1.4881 | 0.926507 | EAS | Cystic fibrosis | respiratory | |
| 500 | 0.093059 | 0.187166 | 0.4972 | 0.619048 | -0.27378 | 0.459897 | EAS | Lung disease du | respiratory | |
| 500.1 | 1.36473 | 1.321973 | 1.032343 | 0.301911 | -1.22629 | 3.955749 | EAS | Extrinsic allergic | respiratory | |
| 500.2 | -0.56987 | 0.425378 | -1.33968 | 0.18035 | -1.40359 | 0.263856 | EAS | Pneumoconiosis | respiratory | |
| 501 | -0.0238 | 0.118751 | -0.20045 | 0.84113 | -0.25655 | 0.208944 | EAS | Pneumonitis due | respiratory | |
| 502 | 0.048572 | 0.100274 | 0.484396 | 0.628105 | -0.14796 | 0.245105 | EAS | Postinflammatory | respiratory | |
| 503 | 0.097455 | 0.079039 | 1.233003 | 0.217575 | -0.05746 | 0.252368 | EAS | Pulmonary conge | respiratory | |
| 504 | 0.06583 | 0.137678 | 0.478149 | 0.632544 | -0.20401 | 0.335674 | EAS | Other alveolar ar | respiratory | |
| 504.1 | 0.152634 | 0.179466 | 0.850488 | 0.395054 | -0.19911 | 0.504382 | EAS | Idiopathic fibrosi | respiratory | |
| 505 | -0.06643 | 0.185814 | -0.35752 | 0.720705 | -0.43062 | 0.297757 | EAS | Other pulmonary | respiratory | |
| 506 | -0.12324 | 0.084804 | -1.45323 | 0.146159 | -0.28945 | 0.042973 | EAS | Empyema and pr | respiratory | |
| 507 | -0.04128 | 0.056045 | -0.73654 | 0.461403 | -0.15113 | 0.068567 | EAS | Pleurisy; pleural | respiratory | |
| 508 | 0.015501 | 0.051696 | 0.299859 | 0.764285 | -0.08582 | 0.116823 | EAS | Pulmonary colla | respiratory | |

| phecode | Coef. | Std.Err. | z | p_value_z | [0.025 | 0.975] | GIA | phenotype | category | |
|---|---|---|---|---|---|---|---|---|---|---|
| 509 | 0.067171 | 0.068868 | 0.975358 | 0.329383 | -0.06781 | 0.202151 | EAS | Respiratory failur | respiratory | |
| 509.1 | 0.058632 | 0.072495 | 0.808769 | 0.418648 | -0.08346 | 0.200719 | EAS | Respiratory failur | respiratory | |
| 509.2 | 0.027828 | 0.173739 | 0.160172 | 0.872745 | -0.31269 | 0.36835 | EAS | Respiratory insuf | respiratory | |
| 509.3 | -0.26768 | 0.171277 | -1.56288 | 0.118081 | -0.60338 | 0.068011 | EAS | Pulmonary insuff | respiratory | |
| 509.5 | -0.97319 | 1.208231 | -0.80547 | 0.420548 | -3.34128 | 1.394895 | EAS | Respiratory arres | respiratory | |
| 509.8 | 0.238214 | 0.130595 | 1.824073 | 0.068141 | -0.01775 | 0.494176 | EAS | Dependence on r | respiratory | |
| 510 | -0.01172 | 0.063412 | -0.18479 | 0.853395 | -0.136 | 0.112567 | EAS | Other diseases o | respiratory | |
| 510.2 | -0.06398 | 0.222882 | -0.28707 | 0.774056 | -0.50083 | 0.372858 | EAS | Lung transplant | respiratory | |
| 512 | -0.00565 | 0.04249 | -0.13307 | 0.894141 | -0.08893 | 0.077625 | EAS | Other symptoms | respiratory | |
| 512.1 | 0.296761 | 0.111135 | 2.670269 | 0.007579 | 0.07894 | 0.514582 | EAS | Wheezing | respiratory | |
| 512.2 | -0.01834 | 0.080779 | -0.22705 | 0.820387 | -0.17666 | 0.139983 | EAS | Painful respiratio | respiratory | |
| 512.3 | -0.03455 | 0.223331 | -0.15469 | 0.877068 | -0.47227 | 0.403174 | EAS | Abnormal chest s | respiratory | |
| 512.7 | -0.03716 | 0.046136 | -0.80539 | 0.420597 | -0.12758 | 0.053267 | EAS | Shortness of brea | respiratory | |
| 512.8 | -0.01564 | 0.044732 | -0.34974 | 0.72653 | -0.10332 | 0.072028 | EAS | Cough | respiratory | |
| 512.9 | -0.01492 | 0.049528 | -0.30124 | 0.763229 | -0.11199 | 0.082154 | EAS | Other dyspnea | respiratory | |
| 513 | 0.215376 | 0.091001 | 2.366761 | 0.017945 | 0.037019 | 0.393734 | EAS | Respiratory abnc | respiratory | |
| 513.3 | 0.248738 | 0.155685 | 1.597697 | 0.11011 | -0.0564 | 0.553876 | EAS | Hypoventilation | respiratory | |
| 513.31 | 0.180237 | 0.204457 | 0.881537 | 0.378027 | -0.22049 | 0.580965 | EAS | Apnea | respiratory | |
| 513.32 | 0.222203 | 0.258599 | 0.859256 | 0.390199 | -0.28464 | 0.729047 | EAS | Orthopnea | respiratory | |
| 513.4 | 0.924828 | 0.288711 | 3.2033 | 0.001359 | 0.358965 | 1.490691 | EAS | Hyperventilation | respiratory | |
| 513.8 | 0.039279 | 0.1166 | 0.336869 | 0.736216 | -0.18925 | 0.267811 | EAS | Disorders of diap | respiratory | |
| 514 | 0.090521 | 0.046569 | 1.943817 | 0.051918 | -0.00075 | 0.181793 | EAS | Abnormal finding | respiratory | |
| 514.1 | 0.318213 | 0.17265 | 1.843111 | 0.065313 | -0.02017 | 0.656601 | EAS | Abnormal results | respiratory | |
| 514.2 | 0.015778 | 0.063004 | 0.25043 | 0.802255 | -0.10771 | 0.139264 | EAS | Solitary pulmona | respiratory | |
| 516 | 0.008521 | 0.113708 | 0.074936 | 0.940265 | -0.21434 | 0.231384 | EAS | Abnormal sputun | respiratory | |
| 516.1 | 0.004731 | 0.126122 | 0.037511 | 0.970078 | -0.24246 | 0.251927 | EAS | Hemoptysis | respiratory | |
| 519 | 0.110858 | 0.064836 | 1.709807 | 0.087302 | -0.01622 | 0.237935 | EAS | Other diseases o | respiratory | |
| 519.1 | -0.24112 | 0.441206 | -0.54651 | 0.584716 | -1.10587 | 0.623625 | EAS | Tracheostomy cc | respiratory | |
| 519.2 | 0.089495 | 0.278926 | 0.320854 | 0.748321 | -0.45719 | 0.63618 | EAS | Respiratory comp | respiratory | |
| 519.8 | 0.15516 | 0.086433 | 1.795147 | 0.07263 | -0.01425 | 0.324566 | EAS | Other diseases o | respiratory | |
| 519.9 | 0.038522 | 0.087847 | 0.438517 | 0.661012 | -0.13365 | 0.210699 | EAS | Symptoms involv | respiratory | |
| 520 | -0.1525 | 0.567501 | -0.26872 | 0.788142 | -1.26478 | 0.959781 | EAS | Disorders of tootl | digestive | |
| 520.1 | -0.69405 | 1.090903 | -0.63622 | 0.524635 | -2.83218 | 1.444079 | EAS | Hereditary disturl | digestive | |
| 520.2 | -0.08695 | 0.92086 | -0.09442 | 0.924773 | -1.8918 | 1.717902 | EAS | Disturbances in t | digestive | |
| 521 | -0.06374 | 0.248772 | -0.25624 | 0.797769 | -0.55133 | 0.423839 | EAS | Diseases of hard | digestive | |
| 521.1 | 0.023997 | 0.264859 | 0.090602 | 0.927809 | -0.49512 | 0.543111 | EAS | Dental caries | digestive | |
| 521.2 | -0.9979 | 1.110023 | -0.89899 | 0.368656 | -3.17351 | 1.177702 | EAS | Dental abrasion, | digestive | |

| phecode | Coef. | Std.Err. | z | p_value_z | [0.025 | 0.975] | GIA | phenotype | category | |
|---|---|---|---|---|---|---|---|---|---|---|
| 522 | -0.04433 | 0.219758 | -0.2017 | 0.840149 | -0.47504 | 0.386392 | EAS | Diseases of pulp | digestive | |
| 522.5 | -0.04433 | 0.219758 | -0.2017 | 0.840149 | -0.47504 | 0.386392 | EAS | Periapical absces | digestive | |
| 523 | 0.019462 | 0.163904 | 0.11874 | 0.905481 | -0.30178 | 0.340708 | EAS | Gingival and peri | digestive | |
| 523.1 | -0.3222 | 0.353711 | -0.91092 | 0.362336 | -1.01547 | 0.371058 | EAS | Gingivitis | digestive | |
| 523.3 | -0.0361 | 0.25529 | -0.14139 | 0.887561 | -0.53646 | 0.464264 | EAS | Periodontitis (acu | digestive | |
| 523.31 | -0.0361 | 0.25529 | -0.14139 | 0.887561 | -0.53646 | 0.464264 | EAS | Acute periodontit | digestive | |
| 524 | -0.42608 | 0.472612 | -0.90154 | 0.3673 | -1.35238 | 0.500223 | EAS | Dentofacial anom | digestive | |
| 524.3 | -0.75357 | 0.50328 | -1.49732 | 0.13431 | -1.73998 | 0.232839 | EAS | Anomalies of too | digestive | |
| 525 | -0.07351 | 0.19544 | -0.37611 | 0.706833 | -0.45656 | 0.309547 | EAS | Other diseases o | digestive | |
| 525.1 | 0.356284 | 0.436548 | 0.816138 | 0.414421 | -0.49933 | 1.211902 | EAS | Loss of teeth or e | digestive | |
| 525.2 | 0.282192 | 1.018829 | 0.276977 | 0.781798 | -1.71468 | 2.27906 | EAS | Atrophy of edent | digestive | |
| 526 | 0.06651 | 0.09628 | 0.690795 | 0.489695 | -0.1222 | 0.255216 | EAS | Diseases of the j | digestive | |
| 526.1 | -0.52856 | 1.130193 | -0.46767 | 0.640018 | -2.7437 | 1.686577 | EAS | Cysts of the jaws | digestive | |
| 526.3 | 0.126189 | 0.437464 | 0.288455 | 0.772998 | -0.73122 | 0.983602 | EAS | Anomalies of jaw | digestive | |
| 526.4 | 0.160512 | 0.120049 | 1.337048 | 0.181207 | -0.07478 | 0.395804 | EAS | Temporomandibu | digestive | |
| 526.41 | 0.187172 | 0.161514 | 1.158859 | 0.246514 | -0.12939 | 0.503735 | EAS | Temporomandibu | digestive | |
| 526.42 | 0.309226 | 0.189405 | 1.632621 | 0.102549 | -0.062 | 0.680453 | EAS | Arthralgia/ankylo | digestive | |
| 526.5 | 0.339109 | 0.350365 | 0.967873 | 0.333108 | -0.34759 | 1.025812 | EAS | Inflammatory cor | digestive | |
| 526.8 | 0.075662 | 0.976526 | 0.077481 | 0.938241 | -1.83829 | 1.989619 | EAS | Exostosis of jaw | digestive | |
| 526.9 | -0.62396 | 0.381372 | -1.63608 | 0.101822 | -1.37143 | 0.123518 | EAS | Jaw disease NOS | digestive | |
| 527 | 0.035923 | 0.10612 | 0.338515 | 0.734975 | -0.17207 | 0.243915 | EAS | Diseases of the s | digestive | |
| 527.1 | 0.59177 | 0.464761 | 1.273279 | 0.202919 | -0.31914 | 1.502685 | EAS | Hypertrophy of sa | digestive | |
| 527.2 | 0.05097 | 0.249339 | 0.204419 | 0.838026 | -0.43773 | 0.539664 | EAS | Sialoadenitis | digestive | |
| 527.7 | 0.079471 | 0.139727 | 0.568755 | 0.569522 | -0.19439 | 0.353331 | EAS | Disturbance of sa | digestive | |
| 527.8 | -0.05952 | 0.189607 | -0.31391 | 0.753589 | -0.43114 | 0.312104 | EAS | Other specified d | digestive | |
| 528 | 0.020315 | 0.081965 | 0.24785 | 0.804251 | -0.14033 | 0.180964 | EAS | Diseases of the c | digestive | |
| 528.1 | 0.019062 | 0.125656 | 0.151702 | 0.879422 | -0.22722 | 0.265343 | EAS | Stomatitis and m | digestive | |
| 528.11 | -0.03541 | 0.204547 | -0.17311 | 0.862562 | -0.43631 | 0.365495 | EAS | Stomatitis and m | digestive | |
| 528.12 | 0.149667 | 0.212973 | 0.70275 | 0.482212 | -0.26775 | 0.567087 | EAS | Oral aphthae | digestive | |
| 528.3 | -0.10781 | 0.551392 | -0.19552 | 0.844984 | -1.18852 | 0.972899 | EAS | Cellulitis and abs | digestive | |
| 528.4 | -0.02811 | 0.371222 | -0.07572 | 0.939642 | -0.75569 | 0.699474 | EAS | Cysts of oral soft | digestive | |
| 528.41 | -0.02811 | 0.371222 | -0.07572 | 0.939642 | -0.75569 | 0.699474 | EAS | Cyst of the saliva | digestive | |
| 528.5 | -0.20408 | 0.158097 | -1.29084 | 0.196758 | -0.51394 | 0.105786 | EAS | Diseases of lips | digestive | |
| 528.6 | 0.028299 | 0.397176 | 0.071251 | 0.943198 | -0.75015 | 0.80675 | EAS | Leukoplakia of or | digestive | |
| 528.7 | -0.4195 | 0.495884 | -0.84597 | 0.397571 | -1.39142 | 0.552413 | EAS | Sialolithiasis | digestive | |
| 529 | -0.04365 | 0.145727 | -0.29954 | 0.764528 | -0.32927 | 0.241968 | EAS | Diseases and oth | digestive | |
| 529.1 | 0.238318 | 0.226777 | 1.05089 | 0.293309 | -0.20616 | 0.682794 | EAS | Glossitis | digestive | |

| phecode | Coef. | Std.Err. | z | p_value_z | [0.025 | 0.975] | GIA | phenotype | category | |
|---|---|---|---|---|---|---|---|---|---|---|
| 529.6 | -0.42915 | 0.384934 | -1.11486 | 0.264909 | -1.1836 | 0.325308 | EAS | Glossodynia | digestive | |
| 530 | 0.105124 | 0.043287 | 2.428517 | 0.015161 | 0.020282 | 0.189966 | EAS | Diseases of esop | digestive | |
| 530.1 | 0.09356 | 0.0437 | 2.140965 | 0.032277 | 0.00791 | 0.179209 | EAS | Esophagitis, GEF | digestive | |
| 530.11 | 0.076429 | 0.043989 | 1.737479 | 0.082303 | -0.00979 | 0.162646 | EAS | GERD | digestive | |
| 530.12 | 0.146083 | 0.157981 | 0.924691 | 0.355127 | -0.16355 | 0.45572 | EAS | Ulcer of esophag | digestive | |
| 530.13 | -0.21566 | 0.189504 | -1.13804 | 0.255103 | -0.58708 | 0.155758 | EAS | Barrett's esophag | digestive | |
| 530.14 | 0.120237 | 0.10106 | 1.189757 | 0.234142 | -0.07784 | 0.318311 | EAS | Reflux esophagit | digestive | |
| 530.15 | 0.119374 | 0.409879 | 0.291243 | 0.770866 | -0.68397 | 0.922723 | EAS | Eosinophilic esop | digestive | |
| 530.2 | 0.172271 | 0.095434 | 1.805135 | 0.071054 | -0.01478 | 0.359319 | EAS | Esophageal blee | digestive | |
| 530.3 | -0.03855 | 0.1554 | -0.24807 | 0.804083 | -0.34313 | 0.266029 | EAS | Stricture and ster | digestive | |
| 530.5 | 0.064219 | 0.144439 | 0.444612 | 0.6566 | -0.21888 | 0.347315 | EAS | Disorders of esop | digestive | |
| 530.6 | 0.143495 | 0.489187 | 0.293332 | 0.769268 | -0.81529 | 1.102284 | EAS | Diverticulum of e | digestive | |
| 530.7 | -0.04114 | 0.431236 | -0.09539 | 0.924004 | -0.88634 | 0.80407 | EAS | Gastroesophage | digestive | |
| 530.9 | 0.156251 | 0.122691 | 1.273533 | 0.202829 | -0.08422 | 0.39672 | EAS | Heartburn | digestive | |
| 531 | 0.065163 | 0.077378 | 0.842141 | 0.399709 | -0.08649 | 0.216821 | EAS | Peptic ulcer (excl | digestive | |
| 531.1 | -0.03882 | 0.188385 | -0.20608 | 0.836725 | -0.40805 | 0.330405 | EAS | Hemorrhage fron | digestive | |
| 531.2 | 0.032034 | 0.102139 | 0.313632 | 0.7538 | -0.16815 | 0.232223 | EAS | Gastric ulcer | digestive | |
| 531.3 | -0.03652 | 0.173672 | -0.21028 | 0.833448 | -0.37691 | 0.30387 | EAS | Duodenal ulcer | digestive | |
| 531.4 | 0.06298 | 0.101113 | 0.622873 | 0.533368 | -0.1352 | 0.261158 | EAS | Peptic ulcer, site | digestive | |
| 532 | -0.02483 | 0.062696 | -0.39607 | 0.692056 | -0.14771 | 0.09805 | EAS | Dysphagia | digestive | |
| 535 | 0.125149 | 0.060145 | 2.080782 | 0.037454 | 0.007267 | 0.243031 | EAS | Gastritis and duo | digestive | |
| 535.1 | 0.423977 | 0.15122 | 2.803708 | 0.005052 | 0.127591 | 0.720363 | EAS | Acute gastritis | digestive | |
| 535.2 | 0.084092 | 0.071157 | 1.181782 | 0.237292 | -0.05537 | 0.223557 | EAS | Atrophic gastritis | digestive | |
| 535.6 | -0.02046 | 0.214289 | -0.09548 | 0.923931 | -0.44046 | 0.399537 | EAS | Duodenitis | digestive | |
| 535.8 | 0.199613 | 0.117514 | 1.698631 | 0.089389 | -0.03071 | 0.429936 | EAS | Other specified g | digestive | |
| 535.9 | 0.201184 | 0.090245 | 2.229313 | 0.025793 | 0.024307 | 0.378061 | EAS | Gastritis and duo | digestive | |
| 536 | 0.052105 | 0.067707 | 0.769558 | 0.441562 | -0.0806 | 0.184809 | EAS | Disorders of func | digestive | |
| 536.3 | 0.135855 | 0.156439 | 0.868419 | 0.385165 | -0.17076 | 0.44247 | EAS | Gastroparesis | digestive | |
| 536.7 | -0.07474 | 0.255263 | -0.29281 | 0.769669 | -0.57505 | 0.425562 | EAS | Complications of | digestive | |
| 536.8 | 0.043285 | 0.074519 | 0.580864 | 0.561332 | -0.10277 | 0.18934 | EAS | Dyspepsia and o | digestive | |
| 537 | 0.124385 | 0.060201 | 2.066172 | 0.038812 | 0.006394 | 0.242376 | EAS | Other disorders c | digestive | |
| 537.1 | -0.15142 | 0.319562 | -0.47383 | 0.635621 | -0.77775 | 0.474912 | EAS | Lesions of stoma | digestive | |
| 539 | 0.115856 | 0.22353 | 0.5183 | 0.604249 | -0.32226 | 0.553967 | EAS | Bariatric surgery | digestive | |
| 540 | -0.01595 | 0.182252 | -0.08754 | 0.930245 | -0.37316 | 0.341255 | EAS | Appendiceal con | digestive | |
| 540.1 | 0.082397 | 0.182738 | 0.450902 | 0.65206 | -0.27576 | 0.440557 | EAS | Appendicitis | digestive | |
| 540.11 | 0.111401 | 0.230536 | 0.483225 | 0.628936 | -0.34044 | 0.563243 | EAS | Acute appendicit | digestive | |
| 550 | 0.002971 | 0.0649 | 0.045786 | 0.963481 | -0.12423 | 0.130172 | EAS | Abdominal hernia | digestive | |

| phecode | Coef. | Std.Err. | z | p_value_z | [0.025 | 0.975] | GIA | phenotype | category | |
|---|---|---|---|---|---|---|---|---|---|---|
| 550.1 | -0.08866 | 0.135699 | -0.65333 | 0.513544 | -0.35462 | 0.177308 | EAS | Inguinal hernia | digestive | |
| 550.2 | 0.014859 | 0.076235 | 0.194912 | 0.845462 | -0.13456 | 0.164278 | EAS | Diaphragmatic he | digestive | |
| 550.4 | 0.165226 | 0.214724 | 0.769484 | 0.441606 | -0.25562 | 0.586077 | EAS | Umbilical hernia | digestive | |
| 550.5 | 0.213243 | 0.162535 | 1.311982 | 0.189526 | -0.10532 | 0.531805 | EAS | Ventral hernia | digestive | |
| 550.6 | 0.079132 | 0.265783 | 0.297733 | 0.765907 | -0.44179 | 0.600058 | EAS | Incisional hernia | digestive | |
| 555 | -0.03729 | 0.141001 | -0.26448 | 0.791406 | -0.31365 | 0.239065 | EAS | Inflammatory bov | digestive | |
| 555.1 | -0.13655 | 0.238156 | -0.57335 | 0.566408 | -0.60332 | 0.330231 | EAS | Regional enteritis | digestive | |
| 555.2 | -0.00405 | 0.164662 | -0.02458 | 0.980393 | -0.32678 | 0.318685 | EAS | Ulcerative colitis | digestive | |
| 555.21 | -0.09073 | 0.255485 | -0.35514 | 0.722485 | -0.59148 | 0.410009 | EAS | Ulcerative colitis | digestive | |
| 556 | -0.19725 | 0.157754 | -1.25035 | 0.21117 | -0.50644 | 0.111944 | EAS | Ulceration of the | digestive | |
| 556.1 | -0.17972 | 0.185229 | -0.97028 | 0.331907 | -0.54277 | 0.183319 | EAS | Ulceration of inte | digestive | |
| 556.11 | -0.15911 | 0.264868 | -0.6007 | 0.548041 | -0.67824 | 0.360027 | EAS | Angiodysplasia o | digestive | |
| 557 | -0.00968 | 0.191389 | -0.0506 | 0.959645 | -0.3848 | 0.365431 | EAS | Intestinal malabs | digestive | |
| 557.1 | 0.393453 | 0.394225 | 0.998041 | 0.318259 | -0.37921 | 1.166121 | EAS | Celiac disease | digestive | |
| 558 | -0.06656 | 0.08077 | -0.82412 | 0.409871 | -0.22487 | 0.091742 | EAS | Noninfectious ga | digestive | |
| 559 | 0.272423 | 0.307757 | 0.885189 | 0.376055 | -0.33077 | 0.875616 | EAS | Ileostomy status | digestive | |
| 560 | 0.096145 | 0.091946 | 1.045669 | 0.295714 | -0.08407 | 0.276357 | EAS | Intestinal obstruc | digestive | |
| 560.1 | 0.253111 | 0.119161 | 2.124106 | 0.033661 | 0.019559 | 0.486662 | EAS | Paralytic ileus | digestive | |
| 560.2 | -0.2851 | 0.182494 | -1.56225 | 0.118228 | -0.64278 | 0.07258 | EAS | Impaction of intes | digestive | |
| 560.3 | 0.444862 | 0.337696 | 1.317347 | 0.187722 | -0.21701 | 1.106734 | EAS | Peritoneal or inte | digestive | |
| 560.4 | 0.168102 | 0.140497 | 1.196481 | 0.231509 | -0.10727 | 0.443471 | EAS | Other intestinal o | digestive | |
| 561 | -0.01072 | 0.045538 | -0.23551 | 0.813816 | -0.09998 | 0.078528 | EAS | Symptoms involv | digestive | |
| 561.1 | -0.10044 | 0.055933 | -1.79569 | 0.072544 | -0.21007 | 0.009188 | EAS | Diarrhea | digestive | |
| 561.2 | 0.045527 | 0.060753 | 0.749387 | 0.453624 | -0.07355 | 0.164601 | EAS | Flatulence | digestive | |
| 562 | 0.089413 | 0.06542 | 1.366756 | 0.171702 | -0.03881 | 0.217633 | EAS | Diverticulosis and | digestive | |
| 562.1 | 0.07523 | 0.066269 | 1.135224 | 0.256281 | -0.05465 | 0.205114 | EAS | Diverticulosis | digestive | |
| 562.2 | -0.04611 | 0.178077 | -0.25894 | 0.795681 | -0.39514 | 0.302913 | EAS | Diverticulitis | digestive | |
| 563 | -0.00061 | 0.052707 | -0.01163 | 0.990717 | -0.10392 | 0.102692 | EAS | Constipation | digestive | |
| 564 | 0.065568 | 0.054626 | 1.20031 | 0.230019 | -0.0415 | 0.172633 | EAS | Functional digest | digestive | |
| 564.1 | 0.003651 | 0.116834 | 0.031251 | 0.975069 | -0.22534 | 0.232642 | EAS | Irritable Bowel Sy | digestive | |
| 564.8 | 0.032394 | 0.091972 | 0.352217 | 0.724676 | -0.14787 | 0.212656 | EAS | Abnormal finding | digestive | |
| 564.9 | 0.113814 | 0.068447 | 1.662801 | 0.096352 | -0.02034 | 0.247968 | EAS | Personal history | digestive | |
| 565 | -0.06227 | 0.071157 | -0.87511 | 0.381513 | -0.20174 | 0.077195 | EAS | Anal and rectal c | digestive | |
| 565.1 | -0.02913 | 0.098919 | -0.29445 | 0.768413 | -0.223 | 0.164751 | EAS | Anal and rectal p | digestive | |
| 567 | -0.12899 | 0.152168 | -0.84767 | 0.39662 | -0.42723 | 0.169256 | EAS | Peritonitis and re | digestive | |
| 568 | 0.192212 | 0.084581 | 2.27251 | 0.023056 | 0.026436 | 0.357988 | EAS | Other disorders c | digestive | |
| 568.1 | 0.151081 | 0.096153 | 1.571263 | 0.116122 | -0.03737 | 0.339537 | EAS | Peritoneal adhes | digestive | |

| phecode | Coef. | Std.Err. | z | p_value_z | [0.025 | 0.975] | GIA | phenotype | category | |
|---|---|---|---|---|---|---|---|---|---|---|
| 569 | 0.020124 | 0.062468 | 0.322148 | 0.74734 | -0.10231 | 0.142558 | EAS | Other disorders c | digestive | |
| 569.1 | -0.07527 | 0.165078 | -0.45598 | 0.648402 | -0.39882 | 0.248274 | EAS | Toxic gastroenter | digestive | |
| 569.2 | 0.211283 | 0.138533 | 1.525145 | 0.127223 | -0.06024 | 0.482802 | EAS | Gastrointestinal c | digestive | |
| 571 | -0.00442 | 0.051478 | -0.08585 | 0.931583 | -0.10532 | 0.096476 | EAS | Chronic liver dise | digestive | |
| 571.5 | -0.00861 | 0.052119 | -0.16527 | 0.868731 | -0.11077 | 0.093538 | EAS | Other chronic no | digestive | |
| 571.51 | -0.00619 | 0.094812 | -0.06534 | 0.947904 | -0.19202 | 0.179633 | EAS | Cirrhosis of liver | digestive | |
| 571.6 | -0.05852 | 0.356559 | -0.16412 | 0.869634 | -0.75736 | 0.640323 | EAS | Primary biliary ci | digestive | |
| 571.8 | 0.060898 | 0.106326 | 0.572748 | 0.566815 | -0.1475 | 0.269293 | EAS | Liver abscess an | digestive | |
| 571.81 | 0.087816 | 0.120767 | 0.727152 | 0.467133 | -0.14888 | 0.324514 | EAS | Portal hypertensi | digestive | |
| 572 | -0.03006 | 0.076896 | -0.39093 | 0.695852 | -0.18077 | 0.120653 | EAS | Ascites (non mali | digestive | |
| 573 | 0.011457 | 0.054411 | 0.210561 | 0.83323 | -0.09519 | 0.1181 | EAS | Other disorders c | digestive | |
| 573.1 | 0.070008 | 0.214563 | 0.326283 | 0.744211 | -0.35053 | 0.490543 | EAS | Chronic passive | digestive | |
| 573.2 | 0.004448 | 0.132838 | 0.033485 | 0.973288 | -0.25591 | 0.264805 | EAS | Liver replaced by | digestive | |
| 573.3 | 0.197711 | 0.095514 | 2.069968 | 0.038455 | 0.010507 | 0.384915 | EAS | Hepatomegaly | digestive | |
| 573.4 | 0.038499 | 0.186344 | 0.206601 | 0.836321 | -0.32673 | 0.403727 | EAS | Acute and subac | digestive | |
| 573.5 | -0.05059 | 0.112754 | -0.44864 | 0.653688 | -0.27158 | 0.170407 | EAS | Jaundice (not of | digestive | |
| 573.6 | -0.09049 | 0.074387 | -1.21646 | 0.223811 | -0.23628 | 0.055307 | EAS | Nonspecific eleva | digestive | |
| 573.7 | -0.07651 | 0.084334 | -0.90719 | 0.364306 | -0.2418 | 0.088785 | EAS | Abnormal results | digestive | |
| 573.9 | 0.061585 | 0.06893 | 0.893446 | 0.371619 | -0.07352 | 0.196685 | EAS | Abnormal serum | digestive | |
| 574 | 0.112235 | 0.070297 | 1.596574 | 0.110361 | -0.02555 | 0.250014 | EAS | Cholelithiasis and | digestive | |
| 574.1 | 0.10119 | 0.074579 | 1.356819 | 0.174839 | -0.04498 | 0.247361 | EAS | Cholelithiasis | digestive | |
| 574.11 | -0.01961 | 0.216493 | -0.09056 | 0.927843 | -0.44392 | 0.404713 | EAS | Cholelithiasis wit | digestive | |
| 574.12 | 0.203806 | 0.158699 | 1.28423 | 0.199062 | -0.10724 | 0.514851 | EAS | Cholelithiasis wit | digestive | |
| 574.2 | 0.306678 | 0.146614 | 2.091739 | 0.036462 | 0.01932 | 0.594035 | EAS | Calculus of bile c | digestive | |
| 574.3 | 0.248958 | 0.142753 | 1.743972 | 0.081164 | -0.03083 | 0.528749 | EAS | Cholecystitis with | digestive | |
| 575 | 0.029008 | 0.067965 | 0.426811 | 0.669517 | -0.1042 | 0.162217 | EAS | Other biliary tract | digestive | |
| 575.1 | -0.03102 | 0.203987 | -0.15209 | 0.879118 | -0.43083 | 0.368783 | EAS | Cholangitis | digestive | |
| 575.2 | -0.14537 | 0.13252 | -1.09698 | 0.272648 | -0.40511 | 0.114362 | EAS | Obstruction of bil | digestive | |
| 575.6 | 0.167801 | 0.122818 | 1.366257 | 0.171858 | -0.07292 | 0.40852 | EAS | Cholesterolosis c | digestive | |
| 575.7 | -0.04801 | 0.109092 | -0.44007 | 0.659884 | -0.26183 | 0.165808 | EAS | Other disorders c | digestive | |
| 575.8 | 0.077428 | 0.108477 | 0.713775 | 0.475366 | -0.13518 | 0.29004 | EAS | Other disorders c | digestive | |
| 575.9 | -0.18136 | 0.148138 | -1.2243 | 0.22084 | -0.47171 | 0.10898 | EAS | Nonspecific abnc | digestive | |
| 577 | 0.038286 | 0.084098 | 0.455256 | 0.648925 | -0.12654 | 0.203116 | EAS | Diseases of panc | digestive | |
| 577.1 | 0.023214 | 0.151721 | 0.153002 | 0.878397 | -0.27415 | 0.320582 | EAS | Acute pancreatiti | digestive | |
| 577.2 | -0.02639 | 0.265176 | -0.09951 | 0.920733 | -0.54612 | 0.493348 | EAS | Chronic pancreat | digestive | |
| 577.3 | -0.08652 | 0.129662 | -0.66729 | 0.504584 | -0.34066 | 0.167611 | EAS | Cyst and pseudo | digestive | |
| 578 | -0.10499 | 0.061084 | -1.71887 | 0.085638 | -0.22472 | 0.014727 | EAS | Gastrointestinal h | digestive | |

| phecode | Coef. | Std.Err. | z | p_value_z | [0.025 | 0.975] | GIA | phenotype | category | |
|---|---|---|---|---|---|---|---|---|---|---|
| 578.1 | 0.224444 | 0.173135 | 1.296353 | 0.194854 | -0.11489 | 0.563783 | EAS | Hematemesis | digestive | |
| 578.2 | -0.12097 | 0.086897 | -1.39211 | 0.163888 | -0.29129 | 0.049345 | EAS | Blood in stool | digestive | |
| 578.8 | -0.18186 | 0.079082 | -2.2997 | 0.021465 | -0.33686 | -0.02687 | EAS | Hemorrhage of re | digestive | |
| 578.9 | -0.0977 | 0.100353 | -0.97353 | 0.33029 | -0.29438 | 0.098992 | EAS | Hemorrhage of g | digestive | |
| 579 | 0.060771 | 0.075437 | 0.80558 | 0.420485 | -0.08708 | 0.208625 | EAS | Other symptoms | digestive | |
| 579.2 | 0.063996 | 0.132395 | 0.483369 | 0.628834 | -0.19549 | 0.323485 | EAS | Splenomegaly | digestive | |
| 579.8 | -0.16114 | 0.093862 | -1.71677 | 0.086022 | -0.34511 | 0.022827 | EAS | Nonspecific abno | digestive | |
| 580 | 0.053583 | 0.077026 | 0.69564 | 0.486655 | -0.09739 | 0.204551 | EAS | Nephritis; nephro | genitourinary | |
| 580.1 | -0.1131 | 0.177453 | -0.63734 | 0.523902 | -0.4609 | 0.234704 | EAS | Glomerulonephrit | genitourinary | |
| 580.11 | 0.28678 | 0.298445 | 0.960914 | 0.336595 | -0.29816 | 0.871721 | EAS | Proliferative glom | genitourinary | |
| 580.12 | -0.08508 | 0.198913 | -0.42771 | 0.668862 | -0.47494 | 0.304785 | EAS | Non-proliferative | genitourinary | |
| 580.13 | 0.118037 | 1.024693 | 0.115193 | 0.908292 | -1.89032 | 2.126399 | EAS | Acute glomerulon | genitourinary | |
| 580.14 | -0.19163 | 0.42616 | -0.44967 | 0.65295 | -1.02689 | 0.643627 | EAS | Chronic glomerul | genitourinary | |
| 580.2 | 0.020961 | 0.125509 | 0.167011 | 0.867362 | -0.22503 | 0.266954 | EAS | Nephrotic syndro | genitourinary | |
| 580.3 | 0.07482 | 0.081707 | 0.915708 | 0.35982 | -0.08532 | 0.234963 | EAS | Nephritis and nep | genitourinary | |
| 580.31 | 0.088174 | 0.095862 | 0.919807 | 0.357674 | -0.09971 | 0.27606 | EAS | Nephritis and nep | genitourinary | |
| 580.32 | 0.042709 | 0.106462 | 0.401168 | 0.688297 | -0.16595 | 0.251371 | EAS | Nephritis and nep | genitourinary | |
| 580.4 | 0.202598 | 0.190028 | 1.066147 | 0.286357 | -0.16985 | 0.575046 | EAS | Renal sclerosis, I | genitourinary | |
| 585 | 0.007477 | 0.053382 | 0.140066 | 0.888608 | -0.09715 | 0.112103 | EAS | Renal failure | genitourinary | |
| 585.1 | 0.066868 | 0.061662 | 1.084425 | 0.278176 | -0.05399 | 0.187723 | EAS | Acute renal failur | genitourinary | |
| 585.2 | 0.123432 | 0.132704 | 0.93013 | 0.352304 | -0.13666 | 0.383528 | EAS | Renal failure NOS | genitourinary | |
| 585.3 | 0.060834 | 0.059197 | 1.027658 | 0.304111 | -0.05519 | 0.176858 | EAS | Chronic renal fail | genitourinary | |
| 585.31 | 0.083846 | 0.089755 | 0.934171 | 0.350216 | -0.09207 | 0.259763 | EAS | Renal dialysis | genitourinary | |
| 585.32 | 0.128492 | 0.088373 | 1.453978 | 0.145952 | -0.04472 | 0.3017 | EAS | End stage renal d | genitourinary | |
| 585.33 | 0.066547 | 0.07199 | 0.924381 | 0.355288 | -0.07455 | 0.207645 | EAS | Chronic Kidney D | genitourinary | |
| 585.34 | -0.08556 | 0.107871 | -0.79315 | 0.42769 | -0.29698 | 0.125866 | EAS | Chronic Kidney D | genitourinary | |
| 585.4 | -0.05424 | 0.086991 | -0.62352 | 0.532941 | -0.22474 | 0.116258 | EAS | Chronic kidney d | genitourinary | |
| 586 | 0.058391 | 0.054105 | 1.079226 | 0.280487 | -0.04765 | 0.164434 | EAS | Other disorders o | genitourinary | |
| 586.1 | -0.16703 | 0.302722 | -0.55177 | 0.581108 | -0.76036 | 0.426292 | EAS | Anatomical abno | genitourinary | |
| 586.11 | -0.19423 | 0.35317 | -0.54995 | 0.582354 | -0.88643 | 0.497975 | EAS | Small kidney | genitourinary | |
| 586.12 | -0.40423 | 0.756997 | -0.534 | 0.593345 | -1.88792 | 1.079454 | EAS | Vesicoureteral re | genitourinary | |
| 586.2 | 0.08223 | 0.078527 | 1.047153 | 0.295029 | -0.07168 | 0.236141 | EAS | Cyst of kidney, ac | genitourinary | |
| 586.3 | 0.354426 | 0.394493 | 0.898434 | 0.368954 | -0.41877 | 1.127617 | EAS | Vascular disorder | genitourinary | |
| 586.4 | 0.02435 | 0.115105 | 0.211543 | 0.832464 | -0.20125 | 0.249951 | EAS | Stricture/obstruct | genitourinary | |
| 587 | 0.114484 | 0.108531 | 1.054847 | 0.291495 | -0.09823 | 0.327202 | EAS | Kidney replaced | genitourinary | |
| 588 | 0.09554 | 0.098374 | 0.971189 | 0.331454 | -0.09727 | 0.288349 | EAS | Disorders resultin | genitourinary | |
| 588.1 | 0.106295 | 0.39493 | 0.269149 | 0.787815 | -0.66775 | 0.880344 | EAS | Renal osteodystr | genitourinary | |

| phecode | Coef. | Std.Err. | z | p_value_z | [0.025 | 0.975] | GIA | phenotype | category | |
|---|---|---|---|---|---|---|---|---|---|---|
| 588.2 | 0.111907 | 0.099919 | 1.119974 | 0.262725 | -0.08393 | 0.307746 | EAS | Secondary hyper | genitourinary | |
| 589 | -0.05368 | 0.146986 | -0.36522 | 0.714946 | -0.34177 | 0.234404 | EAS | Abnormal results | genitourinary | |
| 590 | -0.27284 | 0.129467 | -2.10745 | 0.035079 | -0.52659 | -0.01909 | EAS | Pyelonephritis | genitourinary | |
| 591 | 0.030289 | 0.054849 | 0.55222 | 0.580797 | -0.07721 | 0.137792 | EAS | Urinary tract infe | genitourinary | |
| 592 | -0.0131 | 0.069888 | -0.1875 | 0.851265 | -0.15008 | 0.123874 | EAS | Cystitis and ureth | genitourinary | |
| 592.1 | -0.02313 | 0.070869 | -0.32635 | 0.744162 | -0.16203 | 0.115773 | EAS | Cystitis | genitourinary | |
| 592.11 | -0.06178 | 0.079693 | -0.77527 | 0.438178 | -0.21798 | 0.094411 | EAS | Acute cystitis | genitourinary | |
| 592.12 | 0.051569 | 0.30228 | 0.1706 | 0.864538 | -0.54089 | 0.644028 | EAS | Chronic cystitis | genitourinary | |
| 592.13 | -0.20875 | 0.440242 | -0.47417 | 0.635381 | -1.07161 | 0.65411 | EAS | Chronic interstitia | genitourinary | |
| 592.2 | 0.444026 | 0.284589 | 1.560235 | 0.118704 | -0.11376 | 1.00181 | EAS | Urethritis and ure | genitourinary | |
| 592.21 | 1.628783 | 0.708486 | 2.298961 | 0.021507 | 0.240175 | 3.017391 | EAS | Urethral syndrom | genitourinary | |
| 593 | 0.08262 | 0.064258 | 1.285743 | 0.198533 | -0.04332 | 0.208563 | EAS | Hematuria | genitourinary | |
| 593.1 | 0.037311 | 0.109841 | 0.339679 | 0.734098 | -0.17797 | 0.252594 | EAS | Gross hematuria | genitourinary | |
| 593.2 | 0.044605 | 0.080463 | 0.554358 | 0.579334 | -0.1131 | 0.202309 | EAS | Microscopic hem | genitourinary | |
| 594 | 0.140894 | 0.075645 | 1.86258 | 0.062521 | -0.00737 | 0.289155 | EAS | Urinary calculus | genitourinary | |
| 594.1 | 0.154259 | 0.07851 | 1.964836 | 0.049433 | 0.000382 | 0.308135 | EAS | Calculus of kidne | genitourinary | |
| 594.2 | 0.311096 | 0.265782 | 1.170492 | 0.241803 | -0.20983 | 0.832019 | EAS | Calculus of lower | genitourinary | |
| 594.3 | 0.353788 | 0.23143 | 1.528706 | 0.126337 | -0.09981 | 0.807382 | EAS | Calculus of urete | genitourinary | |
| 594.8 | 0.360773 | 0.261457 | 1.379859 | 0.16763 | -0.15167 | 0.873219 | EAS | Renal colic | genitourinary | |
| 595 | 0.301116 | 0.114144 | 2.638023 | 0.008339 | 0.077397 | 0.524835 | EAS | Hydronephrosis | genitourinary | |
| 596 | 0.022183 | 0.084178 | 0.26352 | 0.79215 | -0.1428 | 0.18717 | EAS | Other disorders c | genitourinary | |
| 596.1 | 0.183086 | 0.20757 | 0.882046 | 0.377752 | -0.22374 | 0.589915 | EAS | Bladder neck obs | genitourinary | |
| 596.5 | -0.05239 | 0.108882 | -0.48112 | 0.630432 | -0.26579 | 0.16102 | EAS | Functional disord | genitourinary | |
| 597 | 0.122476 | 0.211697 | 0.578542 | 0.562898 | -0.29244 | 0.537395 | EAS | Other disorders c | genitourinary | |
| 597.1 | 0.126226 | 0.303839 | 0.415436 | 0.677823 | -0.46929 | 0.72174 | EAS | Urethral stricture | genitourinary | |
| 597.2 | -0.11407 | 0.177322 | -0.64328 | 0.520042 | -0.46161 | 0.233477 | EAS | Urinary complica | genitourinary | |
| 597.8 | -0.50536 | 0.739248 | -0.68361 | 0.494223 | -1.95425 | 0.943544 | EAS | Urethral hypermo | genitourinary | |
| 598 | -0.24281 | 0.094205 | -2.57752 | 0.009951 | -0.42745 | -0.05818 | EAS | Abnormal finding | genitourinary | |
| 598.4 | -0.88877 | 0.431943 | -2.0576 | 0.039628 | -1.73536 | -0.04217 | EAS | Other cells and c | genitourinary | |
| 598.9 | -0.24997 | 0.094795 | -2.63696 | 0.008365 | -0.43576 | -0.06418 | EAS | Other nonspecifi | genitourinary | |
| 599 | -0.00807 | 0.045579 | -0.17701 | 0.8595 | -0.0974 | 0.081266 | EAS | Other symptoms/ | genitourinary | |
| 599.1 | -0.03986 | 0.135173 | -0.29492 | 0.768058 | -0.3048 | 0.225069 | EAS | Urinary obstructi | genitourinary | |
| 599.2 | 0.083728 | 0.076887 | 1.088969 | 0.276168 | -0.06697 | 0.234425 | EAS | Retention of urin | genitourinary | |
| 599.3 | 0.021277 | 0.061854 | 0.343997 | 0.730848 | -0.09995 | 0.142508 | EAS | Dysuria | genitourinary | |
| 599.4 | 0.053199 | 0.082494 | 0.644881 | 0.519004 | -0.10849 | 0.214885 | EAS | Urinary incontine | genitourinary | |
| 599.5 | -9.71E-05 | 0.06277 | -0.00155 | 0.998766 | -0.12312 | 0.122931 | EAS | Frequency of urir | genitourinary | |
| 599.6 | -0.02282 | 0.182672 | -0.12491 | 0.900596 | -0.38085 | 0.335213 | EAS | Oliguria and anui | genitourinary | |

| phecode | Coef. | Std.Err. | z | p_value_z | [0.025 | 0.975] | GIA | phenotype | category | |
|---|---|---|---|---|---|---|---|---|---|---|
| 599.7 | 0.192335 | 0.710087 | 0.27086 | 0.786498 | -1.19941 | 1.58408 | EAS | Urethral discharg | genitourinary | |
| 599.8 | -0.10333 | 0.101762 | -1.01537 | 0.309928 | -0.30278 | 0.096123 | EAS | Other symptoms | genitourinary | |
| 599.9 | -0.07294 | 0.097705 | -0.74656 | 0.45533 | -0.26444 | 0.118556 | EAS | Other abnormalit | genitourinary | |
| 600 | -0.08269 | 0.087764 | -0.94219 | 0.346093 | -0.2547 | 0.089323 | EAS | Hyperplasia of pr | genitourinary | |
| 601 | 0.099177 | 0.172239 | 0.575807 | 0.564745 | -0.23841 | 0.436759 | EAS | Inflammatory dis | genitourinary | |
| 601.1 | 0.226494 | 0.215942 | 1.048865 | 0.29424 | -0.19674 | 0.649733 | EAS | Prostatitis | genitourinary | |
| 601.11 | 0.240205 | 0.282292 | 0.850911 | 0.394819 | -0.31308 | 0.793488 | EAS | Acute prostatitis | genitourinary | |
| 601.12 | 0.340354 | 0.338393 | 1.005794 | 0.314514 | -0.32288 | 1.003593 | EAS | Chronic prostatiti | genitourinary | |
| 601.3 | -0.86685 | 1.312488 | -0.66046 | 0.508956 | -3.43928 | 1.705579 | EAS | Orchitis and epid | genitourinary | |
| 601.4 | -0.53199 | 0.32942 | -1.61494 | 0.106323 | -1.17764 | 0.113656 | EAS | Balanoposthitis | genitourinary | |
| 601.8 | 0.304765 | 0.469852 | 0.648641 | 0.51657 | -0.61613 | 1.225658 | EAS | Other inflammato | genitourinary | |
| 602 | 0.089985 | 0.212907 | 0.422648 | 0.672552 | -0.32731 | 0.507275 | EAS | Other disorders c | genitourinary | |
| 602.3 | 0.255312 | 0.523804 | 0.487418 | 0.625962 | -0.77133 | 1.281949 | EAS | Dysplasia of pros | genitourinary | |
| 603 | 0.011313 | 0.215678 | 0.052454 | 0.958167 | -0.41141 | 0.434034 | EAS | Other disorders c | genitourinary | |
| 603.1 | -0.08487 | 0.261137 | -0.325 | 0.745181 | -0.59669 | 0.426949 | EAS | Hydrocele | genitourinary | |
| 603.2 | 0.524988 | 0.38673 | 1.357507 | 0.17462 | -0.23299 | 1.282964 | EAS | Spermatocele | genitourinary | |
| 604 | 0.111628 | 0.207189 | 0.538774 | 0.590043 | -0.29445 | 0.517711 | EAS | Disorders of peni | genitourinary | |
| 604.1 | 0.070697 | 0.413955 | 0.170785 | 0.864393 | -0.74064 | 0.882034 | EAS | Redundant prepu | genitourinary | |
| 604.3 | 0.91691 | 0.693436 | 1.32227 | 0.186078 | -0.4422 | 2.27602 | EAS | Peyronie's diseas | genitourinary | |
| 605 | 0.095941 | 0.104522 | 0.917898 | 0.358672 | -0.10892 | 0.3008 | EAS | Erectile dysfuncti | genitourinary | |
| 608 | 0.072333 | 0.132118 | 0.547486 | 0.584045 | -0.18661 | 0.331279 | EAS | Other disorders c | genitourinary | |
| 609 | 0.126727 | 0.240052 | 0.527915 | 0.597559 | -0.34377 | 0.597221 | EAS | Male infertility an | genitourinary | |
| 609.1 | 0.20237 | 0.298951 | 0.676935 | 0.498447 | -0.38356 | 0.788303 | EAS | Infertility, male | genitourinary | |
| 609.11 | 0.115689 | 0.408176 | 0.283428 | 0.776849 | -0.68432 | 0.915699 | EAS | Azoospermia and | genitourinary | |
| 609.2 | 0.100375 | 0.467913 | 0.214517 | 0.830144 | -0.81672 | 1.017468 | EAS | Abnormal sperma | genitourinary | |
| 610 | -0.03948 | 0.084541 | -0.46701 | 0.640494 | -0.20518 | 0.126216 | EAS | Benign mammar | genitourinary | |
| 610.1 | -0.00154 | 0.100479 | -0.01529 | 0.9878 | -0.19847 | 0.195399 | EAS | Cystic mastopath | genitourinary | |
| 610.2 | 0.180501 | 0.168232 | 1.072925 | 0.283305 | -0.14923 | 0.51023 | EAS | Fibroadenosis of | genitourinary | |
| 610.3 | 0.10076 | 0.204807 | 0.491975 | 0.622737 | -0.30065 | 0.502175 | EAS | Fibrosclerosis of | genitourinary | |
| 610.4 | -0.15753 | 0.135275 | -1.16453 | 0.244211 | -0.42267 | 0.107603 | EAS | Benign neoplasm | genitourinary | |
| 610.8 | 0.056237 | 0.129558 | 0.434067 | 0.66424 | -0.19769 | 0.310167 | EAS | Other specified b | genitourinary | |
| 611 | -0.0457 | 0.058357 | -0.78306 | 0.433593 | -0.16008 | 0.068681 | EAS | Abnormal finding | genitourinary | |
| 611.1 | -0.02654 | 0.063589 | -0.41744 | 0.676358 | -0.15118 | 0.098088 | EAS | Abnormal mamm | genitourinary | |
| 611.11 | 0.011695 | 0.119336 | 0.098001 | 0.921932 | -0.2222 | 0.245588 | EAS | Mammographic r | genitourinary | |
| 611.3 | -0.04182 | 0.074764 | -0.55931 | 0.575949 | -0.18835 | 0.104718 | EAS | Lump or mass in | genitourinary | |
| 612 | 0.06206 | 0.162603 | 0.381666 | 0.702709 | -0.25664 | 0.380756 | EAS | Breast conditions | genitourinary | |
| 612.1 | 0.098538 | 0.411155 | 0.239661 | 0.810593 | -0.70731 | 0.904388 | EAS | Galactorrhea | genitourinary | |

| phecode | Coef. | Std.Err. | z | p_value_z | [0.025 | 0.975] | GIA | phenotype | category | |
|---|---|---|---|---|---|---|---|---|---|---|
| 612.2 | -0.01376 | 0.205451 | -0.06697 | 0.946607 | -0.41643 | 0.388917 | EAS | Hypertrophy of b | genitourinary | |
| 612.3 | 0.419 | 0.324332 | 1.291885 | 0.196397 | -0.21668 | 1.05468 | EAS | Congenital anom | genitourinary | |
| 613 | 0.008265 | 0.069704 | 0.118574 | 0.905613 | -0.12835 | 0.144883 | EAS | Other nonmaligna | genitourinary | |
| 613.1 | 0.235405 | 0.182631 | 1.288971 | 0.197408 | -0.12254 | 0.593355 | EAS | Inflammatory dis | genitourinary | |
| 613.5 | -0.04721 | 0.094479 | -0.49968 | 0.617299 | -0.23239 | 0.137966 | EAS | Mastodynia | genitourinary | |
| 613.7 | 0.170961 | 0.148191 | 1.15365 | 0.248644 | -0.11949 | 0.461411 | EAS | Other signs and s | genitourinary | |
| 613.8 | -0.10786 | 0.110249 | -0.9783 | 0.327927 | -0.32394 | 0.108228 | EAS | Other specified d | genitourinary | |
| 613.9 | 0.223815 | 0.207313 | 1.0796 | 0.28032 | -0.18251 | 0.63014 | EAS | Breast disorder N | genitourinary | |
| 614 | 0.028536 | 0.071012 | 0.401853 | 0.687792 | -0.11064 | 0.167716 | EAS | Inflammatory dis | genitourinary | |
| 614.1 | 0.267766 | 0.220739 | 1.213045 | 0.225113 | -0.16487 | 0.700407 | EAS | Pelvic peritoneal | genitourinary | |
| 614.3 | -0.01924 | 0.227208 | -0.08469 | 0.932506 | -0.46456 | 0.426077 | EAS | Pelvic inflammato | genitourinary | |
| 614.31 | 0.375029 | 0.566491 | 0.662022 | 0.507957 | -0.73527 | 1.485331 | EAS | Acute inflammato | genitourinary | |
| 614.32 | 0.021532 | 0.275576 | 0.078134 | 0.937721 | -0.51859 | 0.561652 | EAS | Chronic inflamma | genitourinary | |
| 614.33 | -0.00685 | 0.381229 | -0.01796 | 0.985672 | -0.75404 | 0.740349 | EAS | Pelvic inflammato | genitourinary | |
| 614.4 | 0.001981 | 0.237078 | 0.008357 | 0.993332 | -0.46268 | 0.466645 | EAS | Inflammatory dis | genitourinary | |
| 614.5 | -0.01156 | 0.076644 | -0.15083 | 0.880108 | -0.16178 | 0.13866 | EAS | Inflammatory dis | genitourinary | |
| 614.51 | -0.14522 | 0.116218 | -1.24956 | 0.211462 | -0.373 | 0.082562 | EAS | Cervicitis and en | genitourinary | |
| 614.52 | 0.040909 | 0.094355 | 0.433563 | 0.664606 | -0.14402 | 0.225842 | EAS | Vaginitis and vulv | genitourinary | |
| 614.53 | 0.615827 | 0.576382 | 1.068437 | 0.285324 | -0.51386 | 1.745515 | EAS | Cyst or abscess | genitourinary | |
| 614.54 | -0.05722 | 0.336583 | -0.16999 | 0.865018 | -0.71691 | 0.602474 | EAS | Abscess or ulcera | genitourinary | |
| 615 | 0.215122 | 0.119008 | 1.807619 | 0.070666 | -0.01813 | 0.448374 | EAS | Endometriosis | genitourinary | |
| 617 | -0.03688 | 0.076732 | -0.48066 | 0.630755 | -0.18728 | 0.11351 | EAS | Disorders second | genitourinary | |
| 618 | 0.06602 | 0.136963 | 0.482026 | 0.629788 | -0.20242 | 0.334463 | EAS | Genital prolapse | genitourinary | |
| 618.1 | 0.194956 | 0.160452 | 1.215043 | 0.22435 | -0.11952 | 0.509436 | EAS | Prolapse of vagin | genitourinary | |
| 618.2 | 0.182475 | 0.236157 | 0.772687 | 0.439707 | -0.28038 | 0.645334 | EAS | Uterine/Uterovag | genitourinary | |
| 619 | -0.04421 | 0.055859 | -0.79152 | 0.428642 | -0.15369 | 0.065268 | EAS | Noninflammatory | genitourinary | |
| 619.1 | 0.072697 | 0.11764 | 0.617959 | 0.536602 | -0.15787 | 0.303267 | EAS | Noninflammatory | genitourinary | |
| 619.2 | -0.05016 | 0.108525 | -0.46223 | 0.643915 | -0.26287 | 0.162541 | EAS | Disorders of uter | genitourinary | |
| 619.3 | -0.0561 | 0.084367 | -0.6649 | 0.506115 | -0.22145 | 0.109261 | EAS | Noninflammatory | genitourinary | |
| 619.4 | -0.11728 | 0.070728 | -1.65821 | 0.097275 | -0.25591 | 0.021342 | EAS | Noninflammatory | genitourinary | |
| 619.5 | 0.259421 | 0.17329 | 1.497035 | 0.134384 | -0.08022 | 0.599063 | EAS | Noninflammatory | genitourinary | |
| 620 | -0.02221 | 0.204817 | -0.10842 | 0.913666 | -0.42364 | 0.379229 | EAS | Dysplasia of fema | genitourinary | |
| 620.1 | -0.00459 | 0.234362 | -0.01959 | 0.984372 | -0.46393 | 0.454751 | EAS | Dysplasia of cerv | genitourinary | |
| 621 | -0.60963 | 0.224236 | -2.71869 | 0.006554 | -1.04912 | -0.17013 | EAS | Endometrial hype | genitourinary | |
| 622 | -0.04034 | 0.094554 | -0.42666 | 0.669626 | -0.22567 | 0.14498 | EAS | Polyp of female g | genitourinary | |
| 622.1 | -0.02423 | 0.11026 | -0.21977 | 0.826048 | -0.24034 | 0.191874 | EAS | Polyp of corpus u | genitourinary | |
| 622.2 | 0.038445 | 0.142312 | 0.270146 | 0.787048 | -0.24048 | 0.317372 | EAS | Mucous polyp of | genitourinary | |

| phecode | Coef. | Std.Err. | z | p_value_z | [0.025 | 0.975] | GIA | phenotype | category |
|---|---|---|---|---|---|---|---|---|---|
| 623 | 0.080029 | 0.133229 | 0.600687 | 0.548049 | -0.1811 | 0.341153 | EAS | Hypertrophy of fe | genitourinary |
| 624 | -0.08781 | 0.098706 | -0.88959 | 0.373689 | -0.28127 | 0.105653 | EAS | Symptoms involv | genitourinary |
| 624.1 | -0.34108 | 0.511872 | -0.66633 | 0.505197 | -1.34433 | 0.662172 | EAS | Dystrophy of fem | genitourinary |
| 624.2 | -0.99772 | 0.636726 | -1.56695 | 0.117127 | -2.24567 | 0.250244 | EAS | Atrophy of female | genitourinary |
| 624.9 | 0.0595 | 0.117105 | 0.508094 | 0.611388 | -0.17002 | 0.289021 | EAS | stress incontinen | genitourinary |
| 625 | -0.14187 | 0.074777 | -1.8972 | 0.057801 | -0.28843 | 0.004693 | EAS | Pain and other sy | genitourinary |
| 625.1 | -0.25233 | 0.158375 | -1.59327 | 0.1111 | -0.56274 | 0.058075 | EAS | Dyspareunia | genitourinary |
| 626 | -0.0288 | 0.064373 | -0.4474 | 0.654586 | -0.15497 | 0.097368 | EAS | Disorders of men | genitourinary |
| 626.1 | -0.08715 | 0.069585 | -1.25243 | 0.210412 | -0.22353 | 0.049233 | EAS | Irregular menstru | genitourinary |
| 626.11 | -0.16994 | 0.099951 | -1.70022 | 0.08909 | -0.36584 | 0.025962 | EAS | Absent or infrequ | genitourinary |
| 626.12 | -0.02304 | 0.101528 | -0.22688 | 0.820514 | -0.22203 | 0.175956 | EAS | Excessive or frec | genitourinary |
| 626.13 | -0.06958 | 0.080692 | -0.86228 | 0.388534 | -0.22773 | 0.088574 | EAS | Irregular menstru | genitourinary |
| 626.14 | -0.08643 | 0.140541 | -0.61495 | 0.538587 | -0.36188 | 0.189029 | EAS | Irregular menstru | genitourinary |
| 626.15 | 0.302368 | 0.224857 | 1.344714 | 0.178718 | -0.13834 | 0.743079 | EAS | Infertility, female, | genitourinary |
| 626.2 | 0.00054 | 0.148961 | 0.003624 | 0.997108 | -0.29142 | 0.292498 | EAS | Dysmenorrhea | genitourinary |
| 626.4 | -0.11987 | 0.297804 | -0.40251 | 0.687307 | -0.70355 | 0.463815 | EAS | Premenstrual ten | genitourinary |
| 626.8 | 0.142179 | 0.124935 | 1.138023 | 0.255111 | -0.10269 | 0.387048 | EAS | Infertility, female | genitourinary |
| 627 | 0.079961 | 0.057551 | 1.389408 | 0.164709 | -0.03284 | 0.192758 | EAS | Menopausal and | genitourinary |
| 627.1 | 0.049408 | 0.124863 | 0.3957 | 0.692326 | -0.19532 | 0.294136 | EAS | Postmenopausal | genitourinary |
| 627.2 | 0.014755 | 0.063589 | 0.232044 | 0.816504 | -0.10988 | 0.139387 | EAS | Symptomatic me | genitourinary |
| 627.21 | 0.066903 | 0.399001 | 0.167676 | 0.866838 | -0.71512 | 0.848931 | EAS | Symptomatic arti | genitourinary |
| 627.22 | 0.10953 | 0.09097 | 1.204024 | 0.22858 | -0.06877 | 0.287827 | EAS | Need for Hormor | genitourinary |
| 627.3 | -0.00971 | 0.096709 | -0.10036 | 0.920056 | -0.19925 | 0.17984 | EAS | Postmenopausal | genitourinary |
| 627.4 | 0.335317 | 0.21927 | 1.529247 | 0.126203 | -0.09444 | 0.765078 | EAS | Premenopausal r | genitourinary |
| 627.5 | -0.04645 | 0.184884 | -0.25124 | 0.801629 | -0.40882 | 0.315915 | EAS | Premature meno | genitourinary |
| 628 | 0.007367 | 0.098437 | 0.074836 | 0.940346 | -0.18557 | 0.2003 | EAS | Ovarian cyst | genitourinary |
| 634 | 0.074501 | 0.107775 | 0.691268 | 0.489397 | -0.13673 | 0.285735 | EAS | Miscarriage; stillb | pregnancy complications |
| 634.1 | -0.05046 | 0.152538 | -0.33078 | 0.740811 | -0.34943 | 0.248512 | EAS | Missed abortion/l | pregnancy complications |
| 634.3 | 0.58283 | 0.339297 | 1.717757 | 0.085841 | -0.08218 | 1.247839 | EAS | Ectopic pregnanc | pregnancy complications |
| 635 | -0.0513 | 0.11392 | -0.45029 | 0.6525 | -0.27458 | 0.171982 | EAS | Hemorrhage duri | pregnancy complications |
| 635.2 | -0.21783 | 0.129529 | -1.68174 | 0.092618 | -0.47171 | 0.036037 | EAS | Antepartum hem | pregnancy complications |
| 635.3 | 0.035935 | 0.154563 | 0.232493 | 0.816155 | -0.267 | 0.338874 | EAS | Placenta previa a | pregnancy complications |
| 636 | -0.05446 | 0.102567 | -0.53093 | 0.595464 | -0.25548 | 0.146571 | EAS | Early or threaten | pregnancy complications |
| 636.1 | 0.134444 | 0.242621 | 0.55413 | 0.57949 | -0.34109 | 0.609973 | EAS | Threatened prem | pregnancy complications |
| 636.2 | 0.004579 | 0.284028 | 0.016123 | 0.987137 | -0.5521 | 0.561263 | EAS | Early onset of de | pregnancy complications |
| 636.3 | -0.09965 | 0.138468 | -0.7197 | 0.47171 | -0.37105 | 0.171736 | EAS | Hemorrhage in e | pregnancy complications |
| 636.8 | -0.69593 | 0.347097 | -2.00499 | 0.044964 | -1.37623 | -0.01563 | EAS | Cervical incompe | pregnancy complications |

| phecode | Coef. | Std.Err. | z | p_value_z | [0.025 | 0.975] | GIA | phenotype | category |
|---|---|---|---|---|---|---|---|---|---|
| 637 | 0.049443 | 0.274083 | 0.180393 | 0.856844 | -0.48775 | 0.586634 | EAS | Short gestation; l | pregnancy complications |
| 638 | 0.104431 | 0.10213 | 1.022532 | 0.306529 | -0.09574 | 0.304602 | EAS | Other high-risk p | pregnancy complications |
| 639 | 0.147686 | 1.040198 | 0.141979 | 0.887097 | -1.89107 | 2.186438 | EAS | Complications fo | pregnancy complications |
| 642 | 0.25717 | 0.127685 | 2.0141 | 0.043999 | 0.006912 | 0.507428 | EAS | Hypertension cor | pregnancy complications |
| 642.1 | 0.272026 | 0.169417 | 1.605658 | 0.108349 | -0.06003 | 0.604077 | EAS | Preeclampsia an | pregnancy complications |
| 643 | -0.22063 | 0.150794 | -1.46315 | 0.143427 | -0.51619 | 0.074917 | EAS | Excessive vomiti | pregnancy complications |
| 643.1 | -0.34088 | 0.269172 | -1.26639 | 0.205375 | -0.86844 | 0.186692 | EAS | Hyperemesis gra | pregnancy complications |
| 644 | -0.1825 | 0.133521 | -1.36681 | 0.171685 | -0.44419 | 0.079199 | EAS | Anemia during pr | pregnancy complications |
| 645 | -0.31753 | 0.163241 | -1.94514 | 0.051758 | -0.63747 | 0.002419 | EAS | Late pregnancy a | pregnancy complications |
| 646 | -0.0779 | 0.10004 | -0.7787 | 0.436159 | -0.27398 | 0.118175 | EAS | Other complicatio | pregnancy complications |
| 647 | 0.048663 | 0.174744 | 0.278481 | 0.780643 | -0.29383 | 0.391155 | EAS | Infectious and pa | pregnancy complications |
| 647.1 | 0.229153 | 0.216613 | 1.057888 | 0.290107 | -0.1954 | 0.653707 | EAS | Infections of geni | pregnancy complications |
| 647.3 | -0.58651 | 0.369071 | -1.58917 | 0.112023 | -1.30988 | 0.136851 | EAS | Major puerperal i | pregnancy complications |
| 649 | -0.03958 | 0.089248 | -0.44344 | 0.657449 | -0.2145 | 0.135347 | EAS | Other conditions | pregnancy complications |
| 649.1 | -0.0814 | 0.119784 | -0.67958 | 0.496773 | -0.31617 | 0.15337 | EAS | Diabetes or abno | pregnancy complications |
| 650 | -0.09002 | 0.108561 | -0.82922 | 0.406978 | -0.3028 | 0.122755 | EAS | Normal delivery | pregnancy complications |
| 651 | 0.045359 | 0.148739 | 0.304954 | 0.760401 | -0.24616 | 0.336882 | EAS | Multiple gestatior | pregnancy complications |
| 652 | -0.22908 | 0.431555 | -0.53082 | 0.595546 | -1.07491 | 0.616756 | EAS | Malposition and r | pregnancy complications |
| 653 | -0.17546 | 0.108688 | -1.61431 | 0.10646 | -0.38848 | 0.037568 | EAS | Problems associa | pregnancy complications |
| 654 | -0.03659 | 0.088647 | -0.41276 | 0.679781 | -0.21033 | 0.137155 | EAS | Other and unspe | pregnancy complications |
| 654.1 | 0.239771 | 0.147982 | 1.620267 | 0.105175 | -0.05027 | 0.52981 | EAS | Abnormality of or | pregnancy complications |
| 655 | 0.043169 | 0.090554 | 0.476722 | 0.63356 | -0.13431 | 0.220651 | EAS | Known or suspec | pregnancy complications |
| 655.1 | -0.13865 | 0.113967 | -1.2166 | 0.223755 | -0.36202 | 0.084719 | EAS | Abnormality in fe | pregnancy complications |
| 656 | -0.09711 | 0.123458 | -0.78659 | 0.431521 | -0.33908 | 0.144863 | EAS | Other perinatal c | pregnancy complications |
| 656.3 | -0.32843 | 0.339276 | -0.96803 | 0.333028 | -0.9934 | 0.336538 | EAS | Endocrine and m | pregnancy complications |
| 656.5 | -0.10831 | 1.153093 | -0.09393 | 0.925165 | -2.36833 | 2.151711 | EAS | Hematological dis | pregnancy complications |
| 656.6 | 1.634996 | 1.538979 | 1.06239 | 0.288059 | -1.38135 | 4.651341 | EAS | Perinatal disorde | pregnancy complications |
| 658 | -0.09623 | 0.373699 | -0.2575 | 0.796797 | -0.82866 | 0.636211 | EAS | Maternal complic | pregnancy complications |
| 661 | -0.26346 | 0.133949 | -1.96685 | 0.049201 | -0.52599 | -0.00092 | EAS | Fetal distress and | pregnancy complications |
| 665 | -0.19937 | 0.099904 | -1.99566 | 0.045971 | -0.39518 | -0.00357 | EAS | Obstetrical/birth t | pregnancy complications |
| 668 | 2.798582 | 2.42963 | 1.151855 | 0.249381 | -1.96341 | 7.56057 | EAS | Complications of | pregnancy complications |
| 669 | -0.11008 | 0.118093 | -0.93217 | 0.351251 | -0.34154 | 0.121376 | EAS | Complications of | pregnancy complications |
| 671 | -0.19236 | 0.205238 | -0.93728 | 0.348617 | -0.59462 | 0.209895 | EAS | Venous/cerebrov | pregnancy complications |
| 674 | -0.07906 | 0.218063 | -0.36258 | 0.716922 | -0.50646 | 0.348331 | EAS | Other complicatio | pregnancy complications |
| 676 | -0.27018 | 0.175988 | -1.5352 | 0.124735 | -0.61511 | 0.074753 | EAS | Other disorders o | pregnancy complications |
| 681 | -0.02472 | 0.070706 | -0.34967 | 0.726583 | -0.16331 | 0.113857 | EAS | Superficial celluli | dermatologic |
| 681.1 | -0.02399 | 0.133733 | -0.17937 | 0.857646 | -0.2861 | 0.238125 | EAS | Cellulitis and abs | dermatologic |

| phecode | Coef. | Std.Err. | z | p_value_z | [0.025 | 0.975] | GIA | phenotype | category | |
|---|---|---|---|---|---|---|---|---|---|---|
| 681.2 | 0.03502 | 0.207469 | 0.168797 | 0.865956 | -0.37161 | 0.441652 | EAS | Cellulitis and abs | dermatologic | |
| 681.3 | 0.298562 | 0.187729 | 1.590389 | 0.111747 | -0.06938 | 0.666505 | EAS | Cellulitis and abs | dermatologic | |
| 681.5 | 0.062739 | 0.135751 | 0.462161 | 0.643966 | -0.20333 | 0.328805 | EAS | Cellulitis and abs | dermatologic | |
| 681.6 | 0.202849 | 0.260743 | 0.777966 | 0.436589 | -0.3082 | 0.713897 | EAS | Cellulitis and abs | dermatologic | |
| 681.7 | 0.049194 | 0.144125 | 0.34133 | 0.732856 | -0.23329 | 0.331674 | EAS | Cellulitis and abs | dermatologic | |
| 686 | -0.14129 | 0.085609 | -1.65038 | 0.098866 | -0.30908 | 0.026503 | EAS | Other local infect | dermatologic | |
| 686.1 | 0.07485 | 0.205893 | 0.363536 | 0.716204 | -0.32869 | 0.478393 | EAS | Carbuncle and fu | dermatologic | |
| 686.2 | 0.294715 | 0.171116 | 1.722312 | 0.085013 | -0.04067 | 0.630095 | EAS | Impetigo | dermatologic | |
| 686.3 | 0.28276 | 0.514833 | 0.549226 | 0.58285 | -0.72629 | 1.291815 | EAS | Pilonidal cyst | dermatologic | |
| 686.4 | 0.138369 | 0.276663 | 0.500135 | 0.61698 | -0.40388 | 0.680618 | EAS | Pyogenic granulo | dermatologic | |
| 686.5 | -0.13341 | 0.382602 | -0.34869 | 0.727321 | -0.8833 | 0.616476 | EAS | Pyoderma | dermatologic | |
| 687 | -0.07784 | 0.060509 | -1.28646 | 0.198284 | -0.19644 | 0.040754 | EAS | Symptoms affect | dermatologic | |
| 687.1 | -0.04348 | 0.051013 | -0.85239 | 0.393997 | -0.14347 | 0.056501 | EAS | Rash and other r | dermatologic | |
| 687.2 | -0.08322 | 0.070318 | -1.1835 | 0.236611 | -0.22104 | 0.0546 | EAS | Localized superfi | dermatologic | |
| 687.3 | -0.16374 | 0.189647 | -0.86341 | 0.387913 | -0.53544 | 0.207958 | EAS | Changes in skin t | dermatologic | |
| 687.4 | 0.006051 | 0.060008 | 0.100842 | 0.919676 | -0.11156 | 0.123665 | EAS | Disturbance of sk | dermatologic | |
| 689 | -0.00506 | 0.058314 | -0.08672 | 0.930891 | -0.11935 | 0.109235 | EAS | Disorder of skin a | dermatologic | |
| 690 | -0.02799 | 0.098863 | -0.28313 | 0.777076 | -0.22176 | 0.165777 | EAS | Erythematosquar | dermatologic | |
| 690.1 | 0.002267 | 0.098937 | 0.022918 | 0.981716 | -0.19165 | 0.19618 | EAS | Seborrheic derma | dermatologic | |
| 691 | -0.00054 | 0.156196 | -0.00347 | 0.99723 | -0.30668 | 0.305596 | EAS | Congenital anom | dermatologic | |
| 691.1 | -0.04642 | 0.412765 | -0.11247 | 0.910454 | -0.85543 | 0.762582 | EAS | Ichthyosis conge | dermatologic | |
| 691.3 | 0.058768 | 0.507341 | 0.115835 | 0.907783 | -0.9356 | 1.053138 | EAS | Congenital pigme | dermatologic | |
| 694 | -0.07691 | 0.060599 | -1.26921 | 0.204367 | -0.19569 | 0.041859 | EAS | Dyschromia and | dermatologic | |
| 694.1 | 0.237221 | 0.290121 | 0.817663 | 0.41355 | -0.33141 | 0.805849 | EAS | Vitiligo | dermatologic | |
| 694.2 | -0.08556 | 0.061198 | -1.39814 | 0.162071 | -0.20551 | 0.034383 | EAS | Other dyschromia | dermatologic | |
| 694.3 | -0.23815 | 0.450311 | -0.52886 | 0.596905 | -1.12074 | 0.644444 | EAS | Vascular disorder | dermatologic | |
| 695 | -0.0749 | 0.075131 | -0.99686 | 0.318832 | -0.22215 | 0.072359 | EAS | Erythematous co | dermatologic | |
| 695.1 | 0.079401 | 0.352033 | 0.225549 | 0.821552 | -0.61057 | 0.769373 | EAS | Toxic erythema | dermatologic | |
| 695.2 | -0.38016 | 0.592569 | -0.64155 | 0.521163 | -1.54158 | 0.781248 | EAS | Bullous dermatos | dermatologic | |
| 695.22 | -0.38016 | 0.592569 | -0.64155 | 0.521163 | -1.54158 | 0.781248 | EAS | Pemphigus and p | dermatologic | |
| 695.3 | 0.069896 | 0.158 | 0.44238 | 0.658214 | -0.23978 | 0.379571 | EAS | Rosacea | dermatologic | |
| 695.4 | 0.008812 | 0.164145 | 0.053683 | 0.957188 | -0.31291 | 0.330529 | EAS | Lupus (localized | dermatologic | |
| 695.41 | -0.0969 | 0.305862 | -0.31682 | 0.751383 | -0.69638 | 0.502576 | EAS | Cutaneous lupus | dermatologic | |
| 695.42 | -0.00312 | 0.166493 | -0.01875 | 0.985042 | -0.32944 | 0.323199 | EAS | Systemic lupus e | dermatologic | |
| 695.7 | 0.105135 | 0.128856 | 0.815914 | 0.414549 | -0.14742 | 0.357688 | EAS | Prurigo and Liche | dermatologic | |
| 695.8 | -0.27518 | 0.160809 | -1.71124 | 0.087038 | -0.59036 | 0.039998 | EAS | Other specified e | dermatologic | |
| 695.81 | 0.488185 | 0.695271 | 0.702151 | 0.482585 | -0.87452 | 1.85089 | EAS | Erythema nodosu | dermatologic | |

| phecode | Coef. | Std.Err. | z | p_value_z | [0.025 | 0.975] | GIA | phenotype | category | |
|---|---|---|---|---|---|---|---|---|---|---|
| 695.9 | 0.053854 | 0.193485 | 0.27834 | 0.780752 | -0.32537 | 0.433078 | EAS | Unspecified eryth | dermatologic | |
| 696 | -0.05093 | 0.111133 | -0.4583 | 0.646737 | -0.26875 | 0.166884 | EAS | Psoriasis and rel | dermatologic | |
| 696.2 | 0.255264 | 0.389655 | 0.655102 | 0.512402 | -0.50845 | 1.018973 | EAS | Parapsoriasis | dermatologic | |
| 696.3 | -0.27196 | 0.316831 | -0.85838 | 0.390684 | -0.89294 | 0.349017 | EAS | Pityriasis | dermatologic | |
| 696.4 | -0.00925 | 0.116828 | -0.07921 | 0.936868 | -0.23823 | 0.219725 | EAS | Psoriasis | dermatologic | |
| 696.41 | -0.04233 | 0.119338 | -0.35467 | 0.722839 | -0.27622 | 0.191573 | EAS | Psoriasis vulgaris | dermatologic | |
| 696.42 | 0.293248 | 0.260485 | 1.125779 | 0.260259 | -0.21729 | 0.803789 | EAS | Psoriatic arthropa | dermatologic | |
| 697 | 0.27751 | 0.319068 | 0.86975 | 0.384437 | -0.34785 | 0.902872 | EAS | Sarcoidosis | dermatologic | |
| 698 | -0.13902 | 0.063945 | -2.17405 | 0.029701 | -0.26435 | -0.01369 | EAS | Pruritus and relat | dermatologic | |
| 700 | -0.1193 | 0.155646 | -0.76651 | 0.443372 | -0.42436 | 0.185756 | EAS | Corns and callos | dermatologic | |
| 701 | 0.076157 | 0.055139 | 1.381176 | 0.167225 | -0.03191 | 0.184227 | EAS | Other hypertroph | dermatologic | |
| 701.1 | -0.02581 | 0.105172 | -0.24541 | 0.806136 | -0.23194 | 0.180323 | EAS | Keratoderma, ac | dermatologic | |
| 701.2 | 0.107929 | 0.094796 | 1.138535 | 0.254897 | -0.07787 | 0.293726 | EAS | Scar conditions a | dermatologic | |
| 701.3 | -0.3454 | 0.293726 | -1.17594 | 0.239621 | -0.92109 | 0.230289 | EAS | Circumscribed sc | dermatologic | |
| 701.4 | -0.02672 | 0.102266 | -0.26128 | 0.793879 | -0.22716 | 0.173718 | EAS | Keloid scar | dermatologic | |
| 701.5 | -0.0466 | 0.228468 | -0.20396 | 0.838383 | -0.49439 | 0.401191 | EAS | Abnormal granula | dermatologic | |
| 701.6 | 0.090543 | 0.356486 | 0.253989 | 0.799504 | -0.60816 | 0.789244 | EAS | Acquired acantho | dermatologic | |
| 702 | 0.013557 | 0.060195 | 0.225225 | 0.821804 | -0.10442 | 0.131538 | EAS | Degenerative ski | dermatologic | |
| 702.1 | -0.08213 | 0.142498 | -0.57634 | 0.564386 | -0.36142 | 0.197164 | EAS | Actinic keratosis | dermatologic | |
| 702.2 | -0.00064 | 0.061507 | -0.01037 | 0.991724 | -0.12119 | 0.119913 | EAS | Seborrheic kerato | dermatologic | |
| 702.4 | 0.42566 | 0.686639 | 0.619918 | 0.535312 | -0.92013 | 1.771447 | EAS | Degenerative ski | dermatologic | |
| 703 | -0.11448 | 0.099122 | -1.15495 | 0.248112 | -0.30876 | 0.079795 | EAS | Diseases of nail, | dermatologic | |
| 703.1 | 0.171802 | 0.134966 | 1.272921 | 0.203046 | -0.09273 | 0.436331 | EAS | Ingrowing nail | dermatologic | |
| 704 | 0.029046 | 0.068688 | 0.422868 | 0.672392 | -0.10558 | 0.163671 | EAS | Diseases of hair | dermatologic | |
| 704.1 | 0.068688 | 0.090426 | 0.759602 | 0.447492 | -0.10854 | 0.24592 | EAS | Alopecia | dermatologic | |
| 704.11 | 0.213246 | 0.230523 | 0.925052 | 0.354939 | -0.23857 | 0.665063 | EAS | Alopecia Areata | dermatologic | |
| 704.12 | 0.12256 | 0.207774 | 0.58987 | 0.555278 | -0.28467 | 0.52979 | EAS | Telogen effluvium | dermatologic | |
| 704.2 | 0.138051 | 0.38338 | 0.36009 | 0.71878 | -0.61336 | 0.889462 | EAS | Hirsutism | dermatologic | |
| 704.8 | -0.01877 | 0.097414 | -0.19269 | 0.847201 | -0.2097 | 0.172157 | EAS | Other specified d | dermatologic | |
| 705 | 0.128662 | 0.168252 | 0.764696 | 0.444453 | -0.20111 | 0.45843 | EAS | Disorders of swe | dermatologic | |
| 705.1 | -0.05301 | 0.212671 | -0.24925 | 0.803169 | -0.46984 | 0.36382 | EAS | Dyshidrosis | dermatologic | |
| 705.3 | 0.377923 | 0.4008 | 0.94292 | 0.345722 | -0.40763 | 1.163477 | EAS | Hidradenitis | dermatologic | |
| 705.8 | 0.131397 | 0.140233 | 0.936987 | 0.348765 | -0.14346 | 0.406249 | EAS | Hyperhidrosis | dermatologic | |
| 706 | 0.054901 | 0.056266 | 0.975734 | 0.329197 | -0.05538 | 0.16518 | EAS | Diseases of seba | dermatologic | |
| 706.1 | 0.03112 | 0.083517 | 0.372615 | 0.709435 | -0.13257 | 0.19481 | EAS | Acne | dermatologic | |
| 706.2 | 0.114424 | 0.08118 | 1.4095 | 0.158687 | -0.04469 | 0.273534 | EAS | Sebaceous cyst | dermatologic | |
| 706.3 | -0.2778 | 0.55359 | -0.50181 | 0.615802 | -1.36281 | 0.80722 | EAS | Seborrhea | dermatologic | |

| phecode | Coef. | Std.Err. | z | p_value_z | [0.025 | 0.975] | GIA | phenotype | category | |
|---|---|---|---|---|---|---|---|---|---|---|
| 706.8 | 0.043978 | 0.098318 | 0.447299 | 0.654659 | -0.14872 | 0.236678 | EAS | Other specified d | dermatologic | |
| 707 | -0.08026 | 0.114549 | -0.70066 | 0.483515 | -0.30477 | 0.144251 | EAS | Chronic ulcer of s | dermatologic | |
| 707.1 | -0.11469 | 0.188562 | -0.60822 | 0.543044 | -0.48426 | 0.254889 | EAS | Decubitus ulcer | dermatologic | |
| 707.2 | 0.138266 | 0.177962 | 0.77694 | 0.437194 | -0.21053 | 0.487064 | EAS | Chronic ulcer of l | dermatologic | |
| 707.3 | -0.18642 | 0.166757 | -1.11792 | 0.263602 | -0.51326 | 0.140417 | EAS | Chronic ulcer of u | dermatologic | |
| 709 | -0.04705 | 0.102404 | -0.4595 | 0.645873 | -0.24776 | 0.153653 | EAS | Diffuse diseases | dermatologic | |
| 709.2 | 0.079555 | 0.131602 | 0.604517 | 0.5455 | -0.17838 | 0.33749 | EAS | Sicca syndrome | dermatologic | |
| 709.3 | -0.02912 | 0.255012 | -0.11418 | 0.909092 | -0.52893 | 0.470696 | EAS | Systemic scleros | dermatologic | |
| 709.4 | -0.5963 | 0.444622 | -1.34114 | 0.179873 | -1.46775 | 0.275141 | EAS | Polymyositis | dermatologic | |
| 709.5 | -0.40883 | 0.297117 | -1.37598 | 0.168829 | -0.99117 | 0.173513 | EAS | Dermatomyositis | dermatologic | |
| 709.6 | -0.51157 | 0.268086 | -1.90821 | 0.056363 | -1.037 | 0.013873 | EAS | Other specified d | dermatologic | |
| 709.7 | -0.20147 | 0.161131 | -1.25033 | 0.211178 | -0.51728 | 0.114344 | EAS | Unspecified diffu | dermatologic | |
| 710 | 0.000828 | 0.204243 | 0.004053 | 0.996766 | -0.39948 | 0.401137 | EAS | Osteomyelitis, pe | musculoskeletal | |
| 710.1 | 0.000828 | 0.204243 | 0.004053 | 0.996766 | -0.39948 | 0.401137 | EAS | Osteomyelitis | musculoskeletal | |
| 710.11 | 0.185303 | 0.307828 | 0.601969 | 0.547195 | -0.41803 | 0.788635 | EAS | Acute osteomyeli | musculoskeletal | |
| 710.12 | -0.14494 | 0.26429 | -0.54841 | 0.58341 | -0.66294 | 0.37306 | EAS | Chronic osteomy | musculoskeletal | |
| 710.19 | -0.01527 | 0.238953 | -0.06388 | 0.949062 | -0.4836 | 0.453073 | EAS | Unspecified oste | musculoskeletal | |
| 711 | 0.1856 | 0.262577 | 0.706841 | 0.479665 | -0.32904 | 0.700241 | EAS | Arthropathy asso | musculoskeletal | |
| 711.1 | 0.300477 | 0.3033 | 0.990694 | 0.321835 | -0.29398 | 0.894934 | EAS | Pyogenic arthritis | musculoskeletal | |
| 711.2 | -0.22256 | 0.715271 | -0.31116 | 0.755679 | -1.62447 | 1.179341 | EAS | Reiter's disease | musculoskeletal | |
| 712 | 0.004641 | 0.393169 | 0.011805 | 0.990581 | -0.76596 | 0.775239 | EAS | Infective connect | musculoskeletal | |
| 713 | -0.26938 | 0.449431 | -0.59939 | 0.548916 | -1.15025 | 0.611487 | EAS | Arthropathy asso | musculoskeletal | |
| 713.5 | -0.13522 | 0.712729 | -0.18972 | 0.849526 | -1.53215 | 1.261702 | EAS | Arthropathy asso | musculoskeletal | |
| 714 | 0.019216 | 0.103018 | 0.186533 | 0.852027 | -0.1827 | 0.221128 | EAS | Rheumatoid arthi | musculoskeletal | |
| 714.1 | 0.077157 | 0.124677 | 0.618856 | 0.536011 | -0.16721 | 0.32152 | EAS | Rheumatoid arthi | musculoskeletal | |
| 714.2 | -0.05922 | 0.576396 | -0.10273 | 0.918175 | -1.18893 | 1.0705 | EAS | Juvenile rheumat | musculoskeletal | |
| 715 | -0.01685 | 0.113472 | -0.14847 | 0.881972 | -0.23925 | 0.205554 | EAS | Other inflammato | musculoskeletal | |
| 715.1 | -0.01266 | 0.13448 | -0.09413 | 0.925007 | -0.27623 | 0.250917 | EAS | Sacroiliitis NEC | musculoskeletal | |
| 715.2 | -0.44315 | 0.294343 | -1.50556 | 0.13218 | -1.02005 | 0.13375 | EAS | Ankylosing spond | musculoskeletal | |
| 715.3 | -0.02051 | 0.31535 | -0.06504 | 0.948142 | -0.63859 | 0.597565 | EAS | Spinal enthesopa | musculoskeletal | |
| 716 | 0.096122 | 0.062494 | 1.538093 | 0.124026 | -0.02636 | 0.218609 | EAS | Other arthropathi | musculoskeletal | |
| 716.1 | 0.192746 | 0.266655 | 0.722829 | 0.469785 | -0.32989 | 0.71538 | EAS | Unspecified polya | musculoskeletal | |
| 716.2 | 0.424211 | 0.553462 | 0.766468 | 0.443398 | -0.66055 | 1.508975 | EAS | Unspecified mon | musculoskeletal | |
| 716.8 | 0.328417 | 1.071758 | 0.306428 | 0.759279 | -1.77219 | 2.429023 | EAS | Palindromic rheu | musculoskeletal | |
| 716.9 | 0.126767 | 0.062772 | 2.019478 | 0.043438 | 0.003736 | 0.249798 | EAS | Arthropathy NOS | musculoskeletal | |
| 717 | -0.55389 | 0.448765 | -1.23426 | 0.217108 | -1.43345 | 0.325672 | EAS | Polymyalgia Rhe | musculoskeletal | |
| 720 | 0.174908 | 0.074049 | 2.362053 | 0.018174 | 0.029774 | 0.320041 | EAS | Spinal stenosis | musculoskeletal | |

| phecode | Coef. | Std.Err. | z | p_value_z | [0.025 | 0.975] | GIA | phenotype | category | |
|---|---|---|---|---|---|---|---|---|---|---|
| 720.1 | 0.180877 | 0.099642 | 1.815271 | 0.069482 | -0.01442 | 0.376172 | EAS | Spinal stenosis o | musculoskeletal | |
| 721 | 0.049199 | 0.058562 | 0.840116 | 0.400843 | -0.06558 | 0.163979 | EAS | Spondylosis and | musculoskeletal | |
| 721.1 | 0.044386 | 0.059427 | 0.746902 | 0.455123 | -0.07209 | 0.16086 | EAS | Spondylosis with | musculoskeletal | |
| 721.2 | 0.656081 | 0.179976 | 3.645385 | 0.000267 | 0.303335 | 1.008827 | EAS | Spondylosis with | musculoskeletal | |
| 721.8 | 0.154669 | 0.149312 | 1.035881 | 0.300258 | -0.13798 | 0.447315 | EAS | Other allied disor | musculoskeletal | |
| 722 | 0.069614 | 0.054345 | 1.280967 | 0.200205 | -0.0369 | 0.176128 | EAS | Intervertebral dis | musculoskeletal | |
| 722.1 | 0.073605 | 0.075374 | 0.976534 | 0.3288 | -0.07413 | 0.221335 | EAS | Displacement of | musculoskeletal | |
| 722.3 | -0.49128 | 0.980342 | -0.50113 | 0.616279 | -2.41271 | 1.430154 | EAS | Schmorl's nodes | musculoskeletal | |
| 722.6 | 0.106265 | 0.057884 | 1.835835 | 0.066382 | -0.00719 | 0.219715 | EAS | Degeneration of i | musculoskeletal | |
| 722.7 | 0.114786 | 0.243677 | 0.471059 | 0.637598 | -0.36281 | 0.592384 | EAS | Intervertebral dis | musculoskeletal | |
| 722.8 | 0.362108 | 0.201776 | 1.794606 | 0.072716 | -0.03337 | 0.757581 | EAS | Postlaminectomy | musculoskeletal | |
| 722.9 | -0.08943 | 0.127334 | -0.70233 | 0.482472 | -0.339 | 0.160139 | EAS | Other and unspe | musculoskeletal | |
| 723 | -0.00405 | 0.136519 | -0.02963 | 0.976362 | -0.27162 | 0.263528 | EAS | Other disorders o | musculoskeletal | |
| 723.1 | -0.22313 | 0.252754 | -0.8828 | 0.377342 | -0.71852 | 0.272257 | EAS | Torticollis | musculoskeletal | |
| 724 | -0.08487 | 0.08321 | -1.0199 | 0.307775 | -0.24795 | 0.078222 | EAS | Other and unspe | musculoskeletal | |
| 724.1 | -0.13471 | 0.102312 | -1.31663 | 0.187961 | -0.33524 | 0.065821 | EAS | Disorders of sacr | musculoskeletal | |
| 724.2 | -0.13701 | 0.10491 | -1.30597 | 0.191562 | -0.34263 | 0.06861 | EAS | Disorders of cocc | musculoskeletal | |
| 724.8 | 0.028988 | 0.164847 | 0.175848 | 0.860414 | -0.29411 | 0.352082 | EAS | Other symptoms | musculoskeletal | |
| 724.9 | 0.026419 | 0.160683 | 0.164415 | 0.869405 | -0.28851 | 0.341351 | EAS | Other unspecified | musculoskeletal | |
| 726 | 0.06441 | 0.052193 | 1.23407 | 0.217177 | -0.03789 | 0.166706 | EAS | Peripheral enthes | musculoskeletal | |
| 726.1 | 0.117704 | 0.062255 | 1.890674 | 0.058668 | -0.00431 | 0.239721 | EAS | Enthesopathy | musculoskeletal | |
| 726.2 | 0.052062 | 0.159156 | 0.327111 | 0.743584 | -0.25988 | 0.364002 | EAS | Synoviopathy | musculoskeletal | |
| 726.3 | -0.04257 | 0.126966 | -0.33532 | 0.737387 | -0.29142 | 0.206275 | EAS | Bursitis | musculoskeletal | |
| 726.4 | 0.091902 | 0.13873 | 0.66245 | 0.507683 | -0.18 | 0.363807 | EAS | Calcaneal spur; E | musculoskeletal | |
| 727 | 0.104126 | 0.055922 | 1.861978 | 0.062606 | -0.00548 | 0.213731 | EAS | Other disorders c | musculoskeletal | |
| 727.1 | 0.124247 | 0.066348 | 1.872671 | 0.061114 | -0.00579 | 0.254287 | EAS | Synovitis and ten | musculoskeletal | |
| 727.2 | 0.301462 | 0.328242 | 0.918412 | 0.358403 | -0.34188 | 0.944805 | EAS | Bursitis disorders | musculoskeletal | |
| 727.4 | 0.196733 | 0.106483 | 1.847554 | 0.064667 | -0.01197 | 0.405436 | EAS | Ganglion and cys | musculoskeletal | |
| 727.5 | -0.072 | 0.220886 | -0.32598 | 0.744439 | -0.50493 | 0.360924 | EAS | Rupture of synov | musculoskeletal | |
| 727.6 | -0.11437 | 0.230457 | -0.49629 | 0.619691 | -0.56606 | 0.337314 | EAS | Rupture of tendo | musculoskeletal | |
| 727.7 | -0.1335 | 0.370336 | -0.36048 | 0.718489 | -0.85934 | 0.592348 | EAS | Contracture of te | musculoskeletal | |
| 727.8 | 0.023099 | 0.647896 | 0.035652 | 0.97156 | -1.24675 | 1.292951 | EAS | Plica syndrome | musculoskeletal | |
| 728 | -0.30858 | 0.342612 | -0.90067 | 0.367762 | -0.98009 | 0.362925 | EAS | Disorders of mus | musculoskeletal | |
| 728.1 | 0.212378 | 0.501306 | 0.423649 | 0.671822 | -0.77016 | 1.19492 | EAS | Muscular calcifica | musculoskeletal | |
| 728.2 | -0.15577 | 0.272228 | -0.57222 | 0.567176 | -0.68933 | 0.377783 | EAS | Laxity of ligamen | musculoskeletal | |
| 728.7 | 0.055645 | 0.099799 | 0.557574 | 0.577136 | -0.13996 | 0.251248 | EAS | Fasciitis | musculoskeletal | |
| 728.71 | 0.767385 | 0.349898 | 2.193167 | 0.028295 | 0.081597 | 1.453173 | EAS | Contracture of pa | musculoskeletal | |

| phecode | Coef. | Std.Err. | z | p_value_z | [0.025 | 0.975] | GIA | phenotype | category |
|---|---|---|---|---|---|---|---|---|---|
| 729 | 0.064721 | 0.060103 | 1.076839 | 0.281552 | -0.05308 | 0.18252 | EAS | Other disorders c | musculoskeletal |
| 729.1 | -0.19171 | 0.467582 | -0.41001 | 0.681797 | -1.10816 | 0.724729 | EAS | Rheumatism, uns | musculoskeletal |
| 729.3 | 0.360478 | 0.362336 | 0.994873 | 0.319798 | -0.34969 | 1.070643 | EAS | Panniculitis | musculoskeletal |
| 729.7 | 0.325176 | 0.551015 | 0.590139 | 0.555097 | -0.75479 | 1.405144 | EAS | Nontraumatic cor | musculoskeletal |
| 731 | 0.092904 | 0.153757 | 0.604225 | 0.545694 | -0.20845 | 0.394263 | EAS | Osteitis deformar | musculoskeletal |
| 731.1 | -1.74362 | 0.898536 | -1.94051 | 0.052318 | -3.50471 | 0.017482 | EAS | Osteitis deformar | musculoskeletal |
| 732 | 0.078395 | 0.493339 | 0.158906 | 0.873743 | -0.88853 | 1.045321 | EAS | Osteochondropat | musculoskeletal |
| 732.1 | 0.111028 | 0.571542 | 0.194261 | 0.845972 | -1.00917 | 1.23123 | EAS | Juvenile osteoch | musculoskeletal |
| 732.7 | -0.62718 | 1.072544 | -0.58476 | 0.55871 | -2.72933 | 1.474967 | EAS | Osteochondritis c | musculoskeletal |
| 733 | 0.009639 | 0.057227 | 0.168433 | 0.866243 | -0.10252 | 0.121802 | EAS | Other disorders c | musculoskeletal |
| 733.2 | 0.04594 | 0.336632 | 0.136471 | 0.891449 | -0.61385 | 0.705726 | EAS | Cyst of bone | musculoskeletal |
| 733.4 | -0.4875 | 0.290778 | -1.67653 | 0.093635 | -1.05741 | 0.082418 | EAS | Aseptic necrosis | musculoskeletal |
| 733.6 | 0.044642 | 0.154614 | 0.288734 | 0.772785 | -0.2584 | 0.347681 | EAS | Costochondritis | musculoskeletal |
| 733.8 | 0.074645 | 0.26303 | 0.283789 | 0.776572 | -0.44088 | 0.590173 | EAS | Malunion and no | musculoskeletal |
| 733.9 | 0.0749 | 0.166573 | 0.449655 | 0.652959 | -0.25158 | 0.401377 | EAS | Chondromalacia | musculoskeletal |
| 735 | -0.12611 | 0.067734 | -1.86181 | 0.06263 | -0.25886 | 0.006648 | EAS | Acquired foot def | musculoskeletal |
| 735.1 | -0.15717 | 0.148307 | -1.05979 | 0.289241 | -0.44785 | 0.133502 | EAS | Flat foot | musculoskeletal |
| 735.2 | -0.27317 | 0.173442 | -1.575 | 0.115257 | -0.61311 | 0.066769 | EAS | Acquired toe defo | musculoskeletal |
| 735.21 | -0.11686 | 0.237863 | -0.4913 | 0.623217 | -0.58307 | 0.349342 | EAS | Hammer toe (acq | musculoskeletal |
| 735.23 | -1.00512 | 0.44223 | -2.27285 | 0.023035 | -1.87188 | -0.13837 | EAS | Hallux rigidus | musculoskeletal |
| 735.3 | -0.11925 | 0.095355 | -1.25055 | 0.2111 | -0.30614 | 0.067647 | EAS | Hallux valgus (Bu | musculoskeletal |
| 736 | 0.316087 | 0.178697 | 1.768848 | 0.076919 | -0.03415 | 0.666327 | EAS | Other acquired d | musculoskeletal |
| 736.1 | 0.010356 | 0.402595 | 0.025723 | 0.979478 | -0.77872 | 0.799428 | EAS | Acquired deformi | musculoskeletal |
| 736.2 | 0.524717 | 0.40708 | 1.288977 | 0.197406 | -0.27315 | 1.322579 | EAS | Acquired deformi | musculoskeletal |
| 736.3 | 0.282192 | 1.018829 | 0.276977 | 0.781798 | -1.71468 | 2.27906 | EAS | Acquired deformi | musculoskeletal |
| 736.4 | 0.70832 | 0.533037 | 1.32884 | 0.183901 | -0.33641 | 1.753053 | EAS | Genu valgum or v | musculoskeletal |
| 736.6 | 0.604032 | 0.312024 | 1.935853 | 0.052886 | -0.00752 | 1.215588 | EAS | Unequal leg leng | musculoskeletal |
| 737 | -0.11405 | 0.10254 | -1.11227 | 0.266022 | -0.31503 | 0.086923 | EAS | Curvature of spin | musculoskeletal |
| 737.1 | -0.47094 | 0.238115 | -1.9778 | 0.047951 | -0.93764 | -0.00425 | EAS | Kyphosis (acquir | musculoskeletal |
| 737.3 | -0.05923 | 0.114375 | -0.51783 | 0.604575 | -0.2834 | 0.164944 | EAS | Kyphoscoliosis a | musculoskeletal |
| 738 | 0.084418 | 0.097014 | 0.870164 | 0.384211 | -0.10573 | 0.274562 | EAS | Other acquired m | musculoskeletal |
| 738.4 | 0.371984 | 0.126327 | 2.944613 | 0.003234 | 0.124388 | 0.61958 | EAS | Acquired spondy | musculoskeletal |
| 739 | -0.13261 | 0.268524 | -0.49384 | 0.62142 | -0.6589 | 0.39369 | EAS | Contracture of joi | musculoskeletal |
| 740 | 0.103567 | 0.05241 | 1.976102 | 0.048143 | 0.000846 | 0.206289 | EAS | Osteoarthrosis | musculoskeletal |
| 740.1 | 0.108032 | 0.056126 | 1.924813 | 0.054253 | -0.00197 | 0.218037 | EAS | Osteoarthritis; loc | musculoskeletal |
| 740.11 | 0.115689 | 0.056142 | 2.060634 | 0.039338 | 0.005652 | 0.225726 | EAS | Osteoarthrosis, lo | musculoskeletal |
| 740.12 | 0.046837 | 0.201395 | 0.232564 | 0.8161 | -0.34789 | 0.441565 | EAS | Osteoarthrosis, lo | musculoskeletal |

| phecode | Coef. | Std.Err. | z | p_value_z | [0.025 | 0.975] | GIA | phenotype | category |
|---|---|---|---|---|---|---|---|---|---|
| 740.2 | 0.149585 | 0.111806 | 1.337894 | 0.180931 | -0.06955 | 0.36872 | EAS | Osteoarthrosis, g | musculoskeletal |
| 740.3 | 0.162344 | 0.190068 | 0.854135 | 0.39303 | -0.21018 | 0.534871 | EAS | Osteoarthrosis in | musculoskeletal |
| 740.9 | 0.135456 | 0.057286 | 2.364559 | 0.018052 | 0.023178 | 0.247734 | EAS | Osteoarthrosis N | musculoskeletal |
| 741 | 0.069134 | 0.057506 | 1.202205 | 0.229284 | -0.04358 | 0.181844 | EAS | Symptoms and d | musculoskeletal |
| 741.1 | 0.30603 | 0.679692 | 0.450248 | 0.652532 | -1.02614 | 1.638203 | EAS | Ankylosis of joint | musculoskeletal |
| 741.2 | 0.183588 | 0.116287 | 1.578749 | 0.114394 | -0.04433 | 0.411507 | EAS | Stiffness of joint | musculoskeletal |
| 741.3 | -0.22209 | 0.288952 | -0.76862 | 0.442119 | -0.78843 | 0.344241 | EAS | Difficulty in walki | musculoskeletal |
| 741.4 | -0.06125 | 0.068987 | -0.88789 | 0.374598 | -0.19647 | 0.073959 | EAS | Joint effusions | musculoskeletal |
| 741.5 | -0.60778 | 0.828754 | -0.73336 | 0.463336 | -2.23211 | 1.016549 | EAS | Hemarthrosis | musculoskeletal |
| 742 | -0.09048 | 0.12372 | -0.73135 | 0.464563 | -0.33297 | 0.152004 | EAS | Derangement of | musculoskeletal |
| 742.1 | 0.419641 | 1.03832 | 0.404154 | 0.686099 | -1.61543 | 2.45471 | EAS | Loose body in joi | musculoskeletal |
| 742.2 | -0.38756 | 0.380222 | -1.0193 | 0.308061 | -1.13278 | 0.357662 | EAS | Pathological, dev | musculoskeletal |
| 742.8 | -0.24149 | 0.204942 | -1.17834 | 0.238659 | -0.64317 | 0.160186 | EAS | Articular cartilage | musculoskeletal |
| 742.9 | 0.014063 | 0.153274 | 0.091751 | 0.926896 | -0.28635 | 0.314475 | EAS | Other derangeme | musculoskeletal |
| 743 | 0.020493 | 0.053725 | 0.381436 | 0.70288 | -0.08481 | 0.125791 | EAS | Osteoporosis, os | musculoskeletal |
| 743.1 | -0.0382 | 0.070522 | -0.54163 | 0.588075 | -0.17642 | 0.100024 | EAS | Osteoporosis | musculoskeletal |
| 743.11 | -0.02506 | 0.071455 | -0.35069 | 0.725822 | -0.16511 | 0.114991 | EAS | Osteoporosis NC | musculoskeletal |
| 743.12 | -0.26069 | 0.096442 | -2.70305 | 0.006871 | -0.44971 | -0.07166 | EAS | Senile osteoporo | musculoskeletal |
| 743.13 | -0.11894 | 0.131409 | -0.90509 | 0.365417 | -0.37649 | 0.138619 | EAS | Other specified o | musculoskeletal |
| 743.2 | -0.00886 | 0.130386 | -0.06792 | 0.945851 | -0.26441 | 0.246697 | EAS | Pathologic fractu | musculoskeletal |
| 743.21 | 0.007546 | 0.244068 | 0.030919 | 0.975334 | -0.47082 | 0.485911 | EAS | Pathologic fractu | musculoskeletal |
| 743.22 | -0.22728 | 0.211167 | -1.07628 | 0.2818 | -0.64116 | 0.186604 | EAS | Pathologic fractu | musculoskeletal |
| 743.4 | 0.351876 | 0.229486 | 1.533324 | 0.125196 | -0.09791 | 0.801659 | EAS | Stress fracture | musculoskeletal |
| 743.9 | 0.028903 | 0.055905 | 0.516996 | 0.605159 | -0.08067 | 0.138474 | EAS | Osteopenia or ot | musculoskeletal |
| 745 | -0.04158 | 0.043464 | -0.95661 | 0.338766 | -0.12677 | 0.04361 | EAS | Pain in joint | musculoskeletal |
| 747 | -0.06092 | 0.086268 | -0.70617 | 0.480081 | -0.23 | 0.108162 | EAS | Cardiac and circu | congenital anomalies |
| 747.1 | 0.032018 | 0.100652 | 0.318109 | 0.750402 | -0.16526 | 0.229293 | EAS | Cardiac congenit | congenital anomalies |
| 747.11 | -0.00831 | 0.143236 | -0.058 | 0.953748 | -0.28904 | 0.272429 | EAS | Cardiac shunt/ he | congenital anomalies |
| 747.12 | 0.027191 | 0.187937 | 0.144683 | 0.884961 | -0.34116 | 0.395541 | EAS | Valvular heart dis | congenital anomalies |
| 747.13 | 0.091595 | 0.178967 | 0.511797 | 0.608793 | -0.25917 | 0.442364 | EAS | Congenital anom | congenital anomalies |
| 747.2 | -0.28885 | 0.157754 | -1.83099 | 0.067101 | -0.59804 | 0.020345 | EAS | Congenital anom | congenital anomalies |
| 748 | -0.37155 | 0.328136 | -1.13231 | 0.257504 | -1.01469 | 0.271583 | EAS | Anomalies of res | congenital anomalies |
| 749 | -0.07221 | 0.245315 | -0.29435 | 0.768492 | -0.55302 | 0.408601 | EAS | Congenital anom | congenital anomalies |
| 749.1 | 1.285422 | 1.05766 | 1.215345 | 0.224234 | -0.78755 | 3.358396 | EAS | Cleft palate | congenital anomalies |
| 749.2 | 0.280811 | 0.63776 | 0.440309 | 0.659714 | -0.96918 | 1.530798 | EAS | Congenital anom | congenital anomalies |
| 750 | -0.08285 | 0.119019 | -0.69613 | 0.48635 | -0.31612 | 0.15042 | EAS | Digestive conger | congenital anomalies |
| 750.1 | 0.065417 | 0.143774 | 0.454998 | 0.649111 | -0.21638 | 0.34721 | EAS | Upper gastrointe | congenital anomalies |

| phecode | Coef. | Std.Err. | z | p_value_z | [0.025 | 0.975] | GIA | phenotype | category |
|---|---|---|---|---|---|---|---|---|---|
| 750.11 | -0.6723 | 0.337961 | -1.98928 | 0.04667 | -1.33469 | -0.00991 | EAS | Esophageal atres | congenital anomalies |
| 750.13 | -0.13275 | 0.219882 | -0.60374 | 0.546014 | -0.56371 | 0.298208 | EAS | Congenital anom | congenital anomalies |
| 750.14 | 0.493576 | 0.274725 | 1.79662 | 0.072396 | -0.04487 | 1.032027 | EAS | Congenital anom | congenital anomalies |
| 750.15 | 0.68608 | 0.285066 | 2.406743 | 0.016095 | 0.127362 | 1.244798 | EAS | Congenital anom | congenital anomalies |
| 750.2 | -0.35049 | 0.202049 | -1.73468 | 0.082798 | -0.7465 | 0.045519 | EAS | Lower gastrointe: | congenital anomalies |
| 750.21 | -0.69457 | 0.288763 | -2.40533 | 0.016158 | -1.26053 | -0.1286 | EAS | Congenital anom | congenital anomalies |
| 750.22 | 0.077608 | 0.260131 | 0.298342 | 0.765442 | -0.43224 | 0.587456 | EAS | Congenital anom | congenital anomalies |
| 751 | 0.020208 | 0.111277 | 0.181604 | 0.855893 | -0.19789 | 0.238307 | EAS | Genitourinary co | congenital anomalies |
| 751.1 | -0.30814 | 0.280594 | -1.09819 | 0.272122 | -0.8581 | 0.241808 | EAS | Congenital anom | congenital anomalies |
| 751.11 | -0.20356 | 0.349091 | -0.5831 | 0.559826 | -0.88776 | 0.480651 | EAS | Congenital anom | congenital anomalies |
| 751.12 | -0.78425 | 0.664178 | -1.18078 | 0.237689 | -2.08601 | 0.517515 | EAS | Congenital anom | congenital anomalies |
| 751.2 | 0.086834 | 0.12367 | 0.702145 | 0.482589 | -0.15555 | 0.329222 | EAS | Congenital anom | congenital anomalies |
| 751.21 | 0.122853 | 0.133036 | 0.923454 | 0.355771 | -0.13789 | 0.383599 | EAS | Cystic kidney dis | congenital anomalies |
| 751.22 | -0.40674 | 0.48849 | -0.83264 | 0.405045 | -1.36416 | 0.550684 | EAS | Other specified c | congenital anomalies |
| 751.3 | -0.89371 | 0.620613 | -1.44005 | 0.149854 | -2.11009 | 0.322666 | EAS | Obstructive genit | congenital anomalies |
| 752 | -0.10478 | 0.255466 | -0.41014 | 0.681707 | -0.60548 | 0.395928 | EAS | Nervous system | congenital anomalies |
| 752.1 | -0.59252 | 1.10965 | -0.53397 | 0.593364 | -2.76739 | 1.582356 | EAS | Neural tube defe | congenital anomalies |
| 752.11 | -0.59252 | 1.10965 | -0.53397 | 0.593364 | -2.76739 | 1.582356 | EAS | Spina bifida | congenital anomalies |
| 752.2 | 0.021352 | 0.284304 | 0.075104 | 0.940132 | -0.53587 | 0.578579 | EAS | Other specified c | congenital anomalies |
| 753 | -0.18892 | 0.273241 | -0.69142 | 0.4893 | -0.72447 | 0.346617 | EAS | Congenital anom | congenital anomalies |
| 753.1 | -0.66823 | 0.453422 | -1.47375 | 0.14055 | -1.55692 | 0.220462 | EAS | Congenital catara | congenital anomalies |
| 753.2 | 0.116685 | 0.476981 | 0.244633 | 0.806741 | -0.81818 | 1.051551 | EAS | Congenital anom | congenital anomalies |
| 754 | 0.226051 | 0.245842 | 0.919497 | 0.357836 | -0.25579 | 0.707893 | EAS | Congenital musc | congenital anomalies |
| 754.2 | 0.678911 | 0.321291 | 2.113072 | 0.034595 | 0.049192 | 1.30863 | EAS | Spondylolisthesis | congenital anomalies |
| 755 | -0.14702 | 0.289721 | -0.50746 | 0.611834 | -0.71486 | 0.420821 | EAS | Congenital anom | congenital anomalies |
| 755.1 | 0.564939 | 0.288465 | 1.958433 | 0.050179 | -0.00044 | 1.13032 | EAS | Congenital defor | congenital anomalies |
| 755.6 | -1.49638 | 0.686206 | -2.18065 | 0.029209 | -2.84132 | -0.15144 | EAS | Other congenital | congenital anomalies |
| 755.61 | -2.76182 | 1.817873 | -1.51926 | 0.128698 | -6.32478 | 0.801149 | EAS | Congenital hip dy | congenital anomalies |
| 756 | -0.3313 | 0.209235 | -1.5834 | 0.11333 | -0.7414 | 0.07879 | EAS | Other congenital | congenital anomalies |
| 756.1 | 2.077231 | 0.89821 | 2.312634 | 0.020743 | 0.316772 | 3.837689 | EAS | Congenital anom | congenital anomalies |
| 756.2 | -0.48722 | 0.292791 | -1.66406 | 0.0961 | -1.06108 | 0.086638 | EAS | Pectus and other | congenital anomalies |
| 756.21 | -0.63062 | 0.314847 | -2.00295 | 0.045183 | -1.24771 | -0.01353 | EAS | Pectus excavatu | congenital anomalies |
| 756.3 | -0.98626 | 0.825907 | -1.19415 | 0.232419 | -2.60501 | 0.632491 | EAS | Congenital anom | congenital anomalies |
| 756.5 | -0.89425 | 0.373152 | -2.39647 | 0.016554 | -1.62561 | -0.16288 | EAS | Congenital osteo | congenital anomalies |
| 757 | -0.5294 | 0.318934 | -1.6599 | 0.096934 | -1.1545 | 0.0957 | EAS | Congenital anom | congenital anomalies |
| 758 | -0.27105 | 0.251735 | -1.07673 | 0.281602 | -0.76444 | 0.222341 | EAS | Chromosomal an | congenital anomalies |
| 758.1 | -0.31298 | 0.264037 | -1.18538 | 0.235867 | -0.83049 | 0.204519 | EAS | Chromosomal an | congenital anomalies |

| phecode | Coef. | Std.Err. | z | p_value_z | [0.025 | 0.975] | GIA | phenotype | category |
|---|---|---|---|---|---|---|---|---|---|
| 759 | -0.37808 | 0.205611 | -1.83882 | 0.065942 | -0.78107 | 0.024909 | EAS | Other and unspe | congenital anomalies |
| 759.1 | -1.03407 | 0.754325 | -1.37086 | 0.170419 | -2.51252 | 0.444376 | EAS | Anomalies of enc | congenital anomalies |
| 760 | 0.026642 | 0.045966 | 0.579598 | 0.562186 | -0.06345 | 0.116734 | EAS | Back pain | symptoms |
| 761 | -0.06957 | 0.054101 | -1.28591 | 0.198474 | -0.17561 | 0.036467 | EAS | Cervicalgia | symptoms |
| 763 | 0.135326 | 0.076479 | 1.769446 | 0.076819 | -0.01457 | 0.285223 | EAS | Thoracic or lumb | symptoms |
| 764 | -0.00529 | 0.07447 | -0.07107 | 0.943346 | -0.15125 | 0.140666 | EAS | Sciatica | symptoms |
| 765 | 0.109155 | 0.085342 | 1.279038 | 0.200884 | -0.05811 | 0.276422 | EAS | Cervical radiculiti | symptoms |
| 766 | 0.03798 | 0.102124 | 0.3719 | 0.709967 | -0.16218 | 0.23814 | EAS | Neuralgia, neuriti | symptoms |
| 767 | 0.206241 | 0.634562 | 0.325013 | 0.745171 | -1.03748 | 1.449959 | EAS | Cervicocranial/C | symptoms |
| 769 | 0.105761 | 0.502413 | 0.210507 | 0.833272 | -0.87895 | 1.090473 | EAS | Nonallopathic les | symptoms |
| 770 | -0.08447 | 0.058415 | -1.4461 | 0.148148 | -0.19897 | 0.030017 | EAS | Myalgia and myo | symptoms |
| 771 | -0.01059 | 0.081955 | -0.12916 | 0.897231 | -0.17121 | 0.150043 | EAS | Musculoskeletal | symptoms |
| 771.1 | 0.059385 | 0.061196 | 0.9704 | 0.331847 | -0.06056 | 0.179327 | EAS | Swelling of limb | symptoms |
| 771.2 | 0.113921 | 0.110417 | 1.031738 | 0.302195 | -0.10249 | 0.330334 | EAS | Cramp of limb | symptoms |
| 772 | -0.01533 | 0.112886 | -0.13584 | 0.891947 | -0.23659 | 0.205919 | EAS | Symptoms of the | symptoms |
| 772.1 | -0.04533 | 0.201258 | -0.22526 | 0.821779 | -0.43979 | 0.349124 | EAS | Muscular wasting | symptoms |
| 772.2 | -0.02431 | 0.104946 | -0.23164 | 0.816819 | -0.23 | 0.181381 | EAS | Spasm of muscle | symptoms |
| 772.3 | -0.10565 | 0.10478 | -1.00828 | 0.313318 | -0.31101 | 0.099717 | EAS | Muscle weakness | symptoms |
| 772.4 | 0.043193 | 0.266758 | 0.161918 | 0.871371 | -0.47964 | 0.566029 | EAS | Rhabdomyolysis | symptoms |
| 772.6 | -0.03808 | 0.161343 | -0.23602 | 0.813416 | -0.35431 | 0.278146 | EAS | Facial weakness | symptoms |
| 773 | 0.066978 | 0.044746 | 1.496845 | 0.134433 | -0.02072 | 0.154678 | EAS | Pain in limb | symptoms |
| 780 | 0.05132 | 0.15922 | 0.322323 | 0.747208 | -0.26074 | 0.363385 | EAS | Hypothermia/Chi | symptoms |
| 781 | 0.060054 | 0.071127 | 0.844313 | 0.398494 | -0.07935 | 0.199461 | EAS | Symptoms involv | symptoms |
| 781.1 | -0.77346 | 0.426623 | -1.81298 | 0.069834 | -1.60963 | 0.062705 | EAS | Loss of height | symptoms |
| 781.2 | 0.399829 | 0.212777 | 1.879095 | 0.060232 | -0.01721 | 0.816864 | EAS | Abnormal postur | symptoms |
| 782.3 | 0.10223 | 0.059225 | 1.726139 | 0.084322 | -0.01385 | 0.218309 | EAS | Edema | symptoms |
| 782.6 | 0.067737 | 0.128984 | 0.52516 | 0.599472 | -0.18507 | 0.320542 | EAS | Pallor and flushir | symptoms |
| 783 | 0.070127 | 0.055604 | 1.26118 | 0.207244 | -0.03886 | 0.179108 | EAS | Fever of unknow | symptoms |
| 783.1 | 0.043361 | 0.184447 | 0.235084 | 0.814144 | -0.31815 | 0.404871 | EAS | Postprocedural fe | symptoms |
| 785 | -0.0002 | 0.0421 | -0.00471 | 0.996244 | -0.08271 | 0.082316 | EAS | Abdominal pain | symptoms |
| 788 | -0.10794 | 0.076616 | -1.40891 | 0.15886 | -0.25811 | 0.042219 | EAS | Syncope and coll | symptoms |
| 789 | -0.06065 | 0.047367 | -1.28041 | 0.2004 | -0.15349 | 0.032188 | EAS | Nausea and vom | symptoms |
| 789.1 | 0.186082 | 0.202431 | 0.919235 | 0.357973 | -0.21068 | 0.58284 | EAS | Persistent vomiti | symptoms |
| 790 | -0.00881 | 0.067986 | -0.12963 | 0.896855 | -0.14206 | 0.124436 | EAS | Nonspecific findi | symptoms |
| 790.1 | -0.09899 | 0.166612 | -0.59411 | 0.552439 | -0.42554 | 0.227568 | EAS | Elevated sedime | symptoms |
| 790.6 | -0.0369 | 0.047663 | -0.77425 | 0.438781 | -0.13032 | 0.056515 | EAS | Other abnormal b | symptoms |
| 790.8 | -0.01939 | 0.221112 | -0.08771 | 0.930108 | -0.45277 | 0.413979 | EAS | Elevated C-react | symptoms |

| phecode | Coef. | Std.Err. | z | p_value_z | [0.025 | 0.975] | GIA | phenotype | category |
|---|---|---|---|---|---|---|---|---|---|
| 790.9 | -0.54522 | 0.404321 | -1.34847 | 0.177507 | -1.33767 | 0.24724 | EAS | Abnormal arterial | symptoms |
| 791 | 0.101846 | 0.201318 | 0.505899 | 0.612928 | -0.29273 | 0.496422 | EAS | Gangrene | symptoms |
| 792 | -0.04026 | 0.093418 | -0.43094 | 0.666513 | -0.22335 | 0.142839 | EAS | Abnormal Papan | genitourinary |
| 792.1 | -0.03901 | 0.107975 | -0.3613 | 0.717872 | -0.25064 | 0.172616 | EAS | Papanicolaou sm | genitourinary |
| 793 | -0.07713 | 0.176817 | -0.43623 | 0.662672 | -0.42369 | 0.269423 | EAS | Nonspecific abnc | symptoms |
| 793.2 | -0.10543 | 0.089806 | -1.17402 | 0.240389 | -0.28145 | 0.070582 | EAS | Nonspecific abnc | symptoms |
| 794 | -0.13385 | 0.272708 | -0.49082 | 0.623555 | -0.66835 | 0.400648 | EAS | Abnormal results | symptoms |
| 795 | -0.18825 | 0.150826 | -1.24815 | 0.211975 | -0.48387 | 0.10736 | EAS | Other and nonsp | symptoms |
| 795.8 | 0.239986 | 0.133628 | 1.795925 | 0.072506 | -0.02192 | 0.501893 | EAS | Abnormal tumor | symptoms |
| 795.81 | 0.168795 | 0.210235 | 0.802887 | 0.42204 | -0.24326 | 0.580847 | EAS | Elevated carcino | symptoms |
| 795.82 | 0.281952 | 0.232961 | 1.210294 | 0.226166 | -0.17464 | 0.738547 | EAS | Elevated cancer | symptoms |
| 796 | -0.07946 | 0.117491 | -0.67629 | 0.498858 | -0.30974 | 0.15082 | EAS | Elevated prostate | genitourinary |
| 797 | 0.044574 | 0.098738 | 0.45144 | 0.651672 | -0.14895 | 0.238097 | EAS | Shock | symptoms |
| 797.1 | 0.239246 | 0.170293 | 1.404911 | 0.160048 | -0.09452 | 0.573014 | EAS | Cardiogenic shoc | symptoms |
| 798 | -0.03057 | 0.047247 | -0.64705 | 0.517599 | -0.12317 | 0.062031 | EAS | Malaise and fatig | symptoms |
| 798.1 | 0.074421 | 0.142508 | 0.522223 | 0.601515 | -0.20489 | 0.353732 | EAS | Chronic fatigue s | symptoms |
| 800 | -0.01749 | 0.114342 | -0.15294 | 0.878443 | -0.24159 | 0.206618 | EAS | Fracture of lower | injuries & poisonings |
| 800.1 | 0.129611 | 0.20335 | 0.637378 | 0.523879 | -0.26895 | 0.528169 | EAS | Fracture of neck | injuries & poisonings |
| 800.2 | -0.90715 | 0.38245 | -2.37195 | 0.017695 | -1.65674 | -0.15756 | EAS | Fracture of unspe | injuries & poisonings |
| 800.3 | -0.42561 | 0.224218 | -1.89817 | 0.057673 | -0.86506 | 0.013855 | EAS | Fracture of tibia a | injuries & poisonings |
| 800.4 | -0.22597 | 0.324776 | -0.69577 | 0.486572 | -0.86252 | 0.410579 | EAS | Fracture of patell | injuries & poisonings |
| 801 | -0.07313 | 0.140648 | -0.51998 | 0.603075 | -0.3488 | 0.20253 | EAS | Fracture of ankle | injuries & poisonings |
| 801.1 | -0.05946 | 0.214933 | -0.27665 | 0.782046 | -0.48072 | 0.361798 | EAS | Fracture of foot | injuries & poisonings |
| 802 | -0.79621 | 0.352363 | -2.25964 | 0.023844 | -1.48683 | -0.10559 | EAS | Fracture of pelvis | injuries & poisonings |
| 803 | 0.081317 | 0.128901 | 0.630845 | 0.528142 | -0.17133 | 0.333959 | EAS | Fracture of upper | injuries & poisonings |
| 803.1 | 0.39438 | 0.271134 | 1.454557 | 0.145792 | -0.13703 | 0.925794 | EAS | Fracture of hume | injuries & poisonings |
| 803.2 | 0.01508 | 0.186062 | 0.081049 | 0.935403 | -0.34959 | 0.379754 | EAS | Fracture of radius | injuries & poisonings |
| 803.21 | 0.650244 | 0.680619 | 0.955372 | 0.33939 | -0.68374 | 1.984232 | EAS | Colles' fracture | injuries & poisonings |
| 803.3 | -0.39701 | 0.289935 | -1.3693 | 0.170905 | -0.96527 | 0.171253 | EAS | Fracture of clavic | injuries & poisonings |
| 804 | 0.05748 | 0.160542 | 0.358035 | 0.720317 | -0.25718 | 0.372137 | EAS | Fracture of hand | injuries & poisonings |
| 805 | -0.0003 | 0.143444 | -0.00208 | 0.998339 | -0.28144 | 0.280846 | EAS | Fracture of vertel | injuries & poisonings |
| 807 | 0.371379 | 0.215891 | 1.720212 | 0.085394 | -0.05176 | 0.794518 | EAS | Fracture of ribs | injuries & poisonings |
| 809 | 0.05389 | 0.045872 | 1.174803 | 0.240074 | -0.03602 | 0.143797 | EAS | Fracture of unspe | injuries & poisonings |
| 816 | 0.284621 | 0.741654 | 0.383765 | 0.701153 | -1.16899 | 1.738235 | EAS | Cerebral laceratio | injuries & poisonings |
| 817 | 0.069152 | 0.237954 | 0.290608 | 0.771351 | -0.39723 | 0.535534 | EAS | Concussion | injuries & poisonings |
| 818 | -0.10509 | 0.265566 | -0.39574 | 0.692298 | -0.6256 | 0.415406 | EAS | Intracranial hemo | injuries & poisonings |
| 818.1 | 0.587252 | 0.609549 | 0.96342 | 0.335337 | -0.60744 | 1.781947 | EAS | Subdural hemorr | injuries & poisonings |

| phecode | Coef. | Std.Err. | z | p_value_z | [0.025 | 0.975] | GIA | phenotype | category |
|---|---|---|---|---|---|---|---|---|---|
| 818.2 | 0.213485 | 0.348052 | 0.613371 | 0.539631 | -0.46868 | 0.895655 | EAS | Subarachnoid he | injuries & poisonings |
| 819 | -0.01348 | 0.146693 | -0.09187 | 0.926801 | -0.30099 | 0.274036 | EAS | Skull and face fra | injuries & poisonings |
| 823 | -1.45902 | 1.228595 | -1.18755 | 0.23501 | -3.86702 | 0.948981 | EAS | Torus fracture | injuries & poisonings |
| 830 | -0.0288 | 0.101608 | -0.28341 | 0.776866 | -0.22794 | 0.170352 | EAS | Dislocation | injuries & poisonings |
| 835 | -0.02018 | 0.110976 | -0.18187 | 0.855686 | -0.23769 | 0.197326 | EAS | Internal deranger | injuries & poisonings |
| 836 | -0.45106 | 0.78943 | -0.57137 | 0.567747 | -1.99831 | 1.096195 | EAS | Traumatic arthro | injuries & poisonings |
| 840 | -0.10279 | 0.060925 | -1.68708 | 0.091589 | -0.2222 | 0.016626 | EAS | Sprains and strai | injuries & poisonings |
| 840.1 | -0.02469 | 0.222696 | -0.11085 | 0.911732 | -0.46116 | 0.411789 | EAS | Muscle/tendon sp | injuries & poisonings |
| 840.2 | 0.077212 | 0.145968 | 0.528966 | 0.596829 | -0.20888 | 0.363305 | EAS | Rotator cuff (caps | injuries & poisonings |
| 840.3 | -0.23073 | 0.158957 | -1.45149 | 0.146642 | -0.54228 | 0.080825 | EAS | Joint/ligament sp | injuries & poisonings |
| 841 | -0.13683 | 0.07916 | -1.72855 | 0.083889 | -0.29198 | 0.018318 | EAS | Sprains and strai | injuries & poisonings |
| 842 | -0.16277 | 0.075692 | -2.15048 | 0.031517 | -0.31113 | -0.01442 | EAS | Other sprains and | injuries & poisonings |
| 850 | -0.01478 | 0.139893 | -0.10562 | 0.915885 | -0.28896 | 0.25941 | EAS | Hemorrhage or h | injuries & poisonings |
| 851 | -0.05513 | 0.107148 | -0.51451 | 0.606895 | -0.26514 | 0.154878 | EAS | Complications of | injuries & poisonings |
| 853 | 0.250853 | 0.279737 | 0.896748 | 0.369854 | -0.29742 | 0.799127 | EAS | Complication of c | injuries & poisonings |
| 854 | 0.034822 | 0.127567 | 0.272971 | 0.784876 | -0.21521 | 0.28485 | EAS | Complications of | injuries & poisonings |
| 855 | -0.2288 | 0.511102 | -0.44765 | 0.654405 | -1.23054 | 0.772946 | EAS | Complication of r | injuries & poisonings |
| 856 | 0.003388 | 0.423487 | 0.008001 | 0.993616 | -0.82663 | 0.833407 | EAS | Vascular complic | injuries & poisonings |
| 857 | 0.187666 | 0.195636 | 0.959263 | 0.337426 | -0.19577 | 0.571106 | EAS | Mechanical comp | injuries & poisonings |
| 858 | -0.23402 | 0.238026 | -0.98318 | 0.325517 | -0.70054 | 0.232499 | EAS | Complication of i | injuries & poisonings |
| 859 | -0.23689 | 0.13624 | -1.73875 | 0.082079 | -0.50391 | 0.030138 | EAS | Complication due | injuries & poisonings |
| 860 | 0.228049 | 0.213164 | 1.069828 | 0.284697 | -0.18974 | 0.645842 | EAS | Bone marrow or s | neoplasms |
| 870 | -0.12036 | 0.089465 | -1.34538 | 0.178503 | -0.29571 | 0.054984 | EAS | Open wounds of | injuries & poisonings |
| 870.1 | 0.810942 | 0.466274 | 1.739196 | 0.082 | -0.10294 | 1.724823 | EAS | Open wound or la | injuries & poisonings |
| 870.2 | -0.44701 | 0.691492 | -0.64644 | 0.517992 | -1.80231 | 0.908289 | EAS | Open wound of e | injuries & poisonings |
| 870.3 | -0.00543 | 0.167929 | -0.03234 | 0.974205 | -0.33457 | 0.323705 | EAS | Other open wour | injuries & poisonings |
| 870.4 | 0.852854 | 0.722595 | 1.180265 | 0.237895 | -0.56341 | 2.269115 | EAS | Open wound of n | injuries & poisonings |
| 870.5 | -0.09731 | 0.230155 | -0.4228 | 0.67244 | -0.54841 | 0.353786 | EAS | Open wound of li | injuries & poisonings |
| 870.8 | -0.72472 | 0.695938 | -1.04136 | 0.29771 | -2.08873 | 0.639293 | EAS | Open wound of g | injuries & poisonings |
| 871 | -0.09557 | 0.088529 | -1.07948 | 0.280375 | -0.26908 | 0.077949 | EAS | Open wounds of | injuries & poisonings |
| 871.1 | 0.08399 | 0.243484 | 0.34495 | 0.730132 | -0.39323 | 0.56121 | EAS | Open wound of h | injuries & poisonings |
| 871.2 | -0.13316 | 0.14404 | -0.92444 | 0.355258 | -0.41547 | 0.149157 | EAS | Open wound of fi | injuries & poisonings |
| 871.3 | 0.171649 | 0.387966 | 0.442432 | 0.658177 | -0.58875 | 0.932049 | EAS | Open wound of fo | injuries & poisonings |
| 871.4 | -0.12603 | 0.401728 | -0.31371 | 0.753741 | -0.9134 | 0.661346 | EAS | Open wound of to | injuries & poisonings |
| 872 | -0.07211 | 0.453853 | -0.15889 | 0.873752 | -0.96165 | 0.817421 | EAS | Traumatic amput | injuries & poisonings |
| 874 | -0.13846 | 0.711578 | -0.19459 | 0.845717 | -1.53313 | 1.256204 | EAS | Complication of a | injuries & poisonings |
| 875 | 0.025858 | 0.386637 | 0.066879 | 0.946678 | -0.73194 | 0.783652 | EAS | Non-healing surg | injuries & poisonings |

| phecode | Coef. | Std.Err. | z | p_value_z | [0.025 | 0.975] | GIA | phenotype | category |
|---|---|---|---|---|---|---|---|---|---|
| 876 | 0.018906 | 0.250568 | 0.075451 | 0.939856 | -0.4722 | 0.51001 | EAS | Posttraumatic wo | injuries & poisonings |
| 907 | 0.68739 | 0.362982 | 1.893727 | 0.058261 | -0.02404 | 1.398822 | EAS | Injuries to the ne | injuries & poisonings |
| 910 | -0.11349 | 0.510286 | -0.2224 | 0.824 | -1.11363 | 0.886652 | EAS | Superficial injury, | injuries & poisonings |
| 911 | 0.134935 | 0.219883 | 0.613667 | 0.539435 | -0.29603 | 0.565898 | EAS | Blister | injuries & poisonings |
| 912 | 0.153189 | 0.111038 | 1.379605 | 0.167708 | -0.06444 | 0.370819 | EAS | Insect bite | injuries & poisonings |
| 913 | 0.04249 | 0.423706 | 0.100282 | 0.92012 | -0.78796 | 0.872938 | EAS | Toxic effect of ve | injuries & poisonings |
| 915 | -0.29699 | 0.141526 | -2.0985 | 0.035861 | -0.57438 | -0.01961 | EAS | Superficial injury | injuries & poisonings |
| 916 | -0.09476 | 0.075623 | -1.25303 | 0.210196 | -0.24298 | 0.053461 | EAS | Contusion | injuries & poisonings |
| 930 | 0.112297 | 0.093863 | 1.196385 | 0.231546 | -0.07167 | 0.296265 | EAS | Allergic reaction t | injuries & poisonings |
| 931 | -0.78214 | 0.67062 | -1.16629 | 0.243497 | -2.09653 | 0.532254 | EAS | Contact dermatiti | dermatologic |
| 938 | 0.096614 | 0.139835 | 0.690919 | 0.489617 | -0.17746 | 0.370685 | EAS | Dermatitis due to | dermatologic |
| 938.1 | 0.058244 | 0.223652 | 0.260421 | 0.794539 | -0.38011 | 0.496593 | EAS | Acute dermatitis | dermatologic |
| 938.2 | 0.17 | 0.198785 | 0.855196 | 0.392443 | -0.21961 | 0.55961 | EAS | Chronic dermatiti | dermatologic |
| 939 | 0.008416 | 0.049354 | 0.170522 | 0.8646 | -0.08832 | 0.105147 | EAS | Atopic/contact de | dermatologic |
| 939.1 | -0.03896 | 0.272846 | -0.14277 | 0.886468 | -0.57372 | 0.495812 | EAS | Contact and aller | dermatologic |
| 941 | -0.17466 | 0.129317 | -1.35061 | 0.176822 | -0.42811 | 0.078801 | EAS | Adverse reaction | injuries & poisonings |
| 942 | -0.18278 | 0.217081 | -0.84197 | 0.399804 | -0.60825 | 0.242696 | EAS | Infusion and tran | injuries & poisonings |
| 946 | -0.02394 | 0.167827 | -0.14264 | 0.886573 | -0.35287 | 0.304996 | EAS | Anaphylactic sho | injuries & poisonings |
| 947 | 0.002948 | 0.084539 | 0.034866 | 0.972187 | -0.16275 | 0.16864 | EAS | Urticaria | dermatologic |
| 949 | 0.040286 | 0.058176 | 0.692476 | 0.488638 | -0.07374 | 0.154309 | EAS | Allergies, other | injuries & poisonings |
| 949.1 | 0.192553 | 0.521867 | 0.368969 | 0.712151 | -0.83029 | 1.215393 | EAS | Diaper or napkin | injuries & poisonings |
| 952 | 0.174571 | 0.436625 | 0.399819 | 0.68929 | -0.6812 | 1.030341 | EAS | Spinal cord injury | injuries & poisonings |
| 957 | 0.082267 | 1.086506 | 0.075717 | 0.939644 | -2.04724 | 2.211779 | EAS | Injury to other an | injuries & poisonings |
| 958 | -0.34415 | 0.368102 | -0.93493 | 0.349823 | -1.06562 | 0.377316 | EAS | Certain early con | injuries & poisonings |
| 958.1 | 1.316333 | 1.055647 | 1.246945 | 0.212418 | -0.7527 | 3.385363 | EAS | Postoperative sh | injuries & poisonings |
| 958.2 | -0.76236 | 0.591875 | -1.28804 | 0.197731 | -1.92241 | 0.397694 | EAS | Traumatic and su | injuries & poisonings |
| 960 | -0.01935 | 0.060155 | -0.3217 | 0.747683 | -0.13725 | 0.09855 | EAS | Poisoning by anti | injuries & poisonings |
| 960.1 | 0.569535 | 0.71584 | 0.795617 | 0.426255 | -0.83349 | 1.972556 | EAS | Adverse effects c | injuries & poisonings |
| 960.2 | -0.08474 | 0.07347 | -1.15345 | 0.248725 | -0.22874 | 0.059255 | EAS | Allergy/adverse e | injuries & poisonings |
| 961 | 0.012111 | 0.160777 | 0.075325 | 0.939956 | -0.30301 | 0.327227 | EAS | Poisoning by oth | injuries & poisonings |
| 961.1 | -0.19082 | 0.098649 | -1.93436 | 0.053068 | -0.38417 | 0.002525 | EAS | Poisoning/allergy | injuries & poisonings |
| 962 | 0.110835 | 0.12525 | 0.884914 | 0.376203 | -0.13465 | 0.35632 | EAS | Poisoning by hor | injuries & poisonings |
| 962.1 | 0.118081 | 0.134302 | 0.879221 | 0.379281 | -0.14515 | 0.381308 | EAS | Adrenal cortical s | injuries & poisonings |
| 962.2 | 0.170788 | 0.690012 | 0.247514 | 0.80451 | -1.18161 | 1.523187 | EAS | Insulins and antic | injuries & poisonings |
| 962.3 | 0.060583 | 0.336379 | 0.180103 | 0.857072 | -0.59871 | 0.719874 | EAS | Hormones and sy | injuries & poisonings |
| 963 | -0.0452 | 0.082483 | -0.54805 | 0.583657 | -0.20687 | 0.116459 | EAS | Poisoning by prir | injuries & poisonings |
| 963.1 | -0.04554 | 0.083969 | -0.54228 | 0.587624 | -0.21011 | 0.119042 | EAS | Antineoplastic an | injuries & poisonings |

| phecode | Coef. | Std.Err. | z | p_value_z | [0.025 | 0.975] | GIA | phenotype | category |
|---|---|---|---|---|---|---|---|---|---|
| 964 | 0.354584 | 0.244749 | 1.448768 | 0.147403 | -0.12511 | 0.834283 | EAS | Poisoning by age | injuries & poisonings |
| 964.1 | 0.281458 | 0.305191 | 0.922238 | 0.356405 | -0.3167 | 0.879621 | EAS | Anticoagulants ca | injuries & poisonings |
| 965 | 0.130177 | 0.093962 | 1.385429 | 0.165921 | -0.05398 | 0.314338 | EAS | Poisoning by ana | injuries & poisonings |
| 965.1 | 0.199461 | 0.093424 | 2.135009 | 0.03276 | 0.016353 | 0.38257 | EAS | Opiates and relat | injuries & poisonings |
| 965.2 | -0.13596 | 0.464997 | -0.29239 | 0.769989 | -1.04734 | 0.775418 | EAS | Antirheumatics ca | injuries & poisonings |
| 965.3 | -0.35926 | 1.080129 | -0.33261 | 0.739428 | -2.47628 | 1.757752 | EAS | Salicylates causi | injuries & poisonings |
| 967 | 0.086595 | 0.192117 | 0.450741 | 0.652176 | -0.28995 | 0.463138 | EAS | Adverse effects c | injuries & poisonings |
| 969 | 0.709369 | 0.466222 | 1.521526 | 0.128128 | -0.20441 | 1.623148 | EAS | Poisoning by psy | injuries & poisonings |
| 971 | -0.61875 | 0.555816 | -1.11322 | 0.265614 | -1.70813 | 0.470634 | EAS | Poisoning by dru | injuries & poisonings |
| 972 | -0.10247 | 0.225175 | -0.45506 | 0.649064 | -0.5438 | 0.338866 | EAS | Poisoning by age | injuries & poisonings |
| 972.1 | -0.36792 | 0.463456 | -0.79387 | 0.42727 | -1.27628 | 0.540433 | EAS | Cardiac rhythm r | injuries & poisonings |
| 972.2 | -0.25984 | 0.415037 | -0.62607 | 0.531266 | -1.0733 | 0.553613 | EAS | Antilipemic and a | injuries & poisonings |
| 972.6 | -0.04491 | 0.291476 | -0.15407 | 0.877552 | -0.61619 | 0.526373 | EAS | Antihypertensive | injuries & poisonings |
| 974 | 0.069523 | 0.685797 | 0.101376 | 0.919252 | -1.27461 | 1.41366 | EAS | Poisoning by wat | injuries & poisonings |
| 975 | 0.938262 | 1.496822 | 0.626836 | 0.530767 | -1.99546 | 3.87198 | EAS | Poisoning by age | injuries & poisonings |
| 976 | -0.3448 | 0.404596 | -0.8522 | 0.394103 | -1.13779 | 0.448197 | EAS | Poisoning by age | injuries & poisonings |
| 977 | -0.04821 | 0.061071 | -0.78938 | 0.429888 | -0.16791 | 0.071489 | EAS | Personal history | injuries & poisonings |
| 979 | 0.058456 | 0.073059 | 0.800122 | 0.42364 | -0.08474 | 0.201648 | EAS | Adverse drug eve | injuries & poisonings |
| 980 | -1.47E-05 | 0.117667 | -0.00012 | 0.999901 | -0.23064 | 0.230609 | EAS | Encounter for lon | infectious diseases |
| 981 | 0.265268 | 0.982705 | 0.269936 | 0.787209 | -1.6608 | 2.191334 | EAS | Toxic effect of (no | injuries & poisonings |
| 984 | 0.391831 | 0.797582 | 0.491274 | 0.623233 | -1.1714 | 1.955064 | EAS | Toxic effect of lea | injuries & poisonings |
| 985 | 0.668444 | 0.617777 | 1.082015 | 0.279246 | -0.54238 | 1.879266 | EAS | Toxic effect of oth | injuries & poisonings |
| 987 | -0.40954 | 0.74912 | -0.54669 | 0.584593 | -1.87778 | 1.058714 | EAS | Toxic effect of oth | injuries & poisonings |
| 988 | 0.867706 | 0.958877 | 0.904919 | 0.365508 | -1.01166 | 2.747071 | EAS | Toxic effect of no | injuries & poisonings |
| 989 | 0.433812 | 0.339638 | 1.277279 | 0.201504 | -0.23187 | 1.099489 | EAS | Toxic effect of oth | injuries & poisonings |
| 990 | 0.040196 | 0.075544 | 0.532084 | 0.594668 | -0.10787 | 0.188258 | EAS | Effects radiation | injuries & poisonings |
| 994 | -0.0274 | 0.069005 | -0.39708 | 0.691307 | -0.16265 | 0.107847 | EAS | Sepsis and SIRS | injuries & poisonings |
| 994.1 | -0.00065 | 0.134714 | -0.00485 | 0.99613 | -0.26469 | 0.263382 | EAS | Systemic inflamm | injuries & poisonings |
| 994.2 | -0.02232 | 0.071541 | -0.31201 | 0.755037 | -0.16254 | 0.117896 | EAS | Sepsis | injuries & poisonings |
| 994.21 | -0.05177 | 0.12628 | -0.41 | 0.681807 | -0.29928 | 0.19573 | EAS | Septic shock | injuries & poisonings |
| 1000 | 0.126964 | 0.221995 | 0.571923 | 0.567374 | -0.30814 | 0.562065 | EAS | Burns | |
| 1001 | 0.091293 | 0.148962 | 0.61286 | 0.539969 | -0.20067 | 0.383252 | EAS | Foreign body injury | |
| 1002 | 0.069837 | 0.067658 | 1.032215 | 0.301971 | -0.06277 | 0.202444 | EAS | Symptoms concerning nutrition, me | |
| 1004 | -0.04226 | 0.15778 | -0.26784 | 0.788825 | -0.3515 | 0.266985 | EAS | Other signs and symptoms involvin | |
| 1005 | -0.02542 | 0.046684 | -0.54458 | 0.586044 | -0.11692 | 0.066076 | EAS | Other symptoms | |
| 1006 | 0.376792 | 0.5683 | 0.663015 | 0.507321 | -0.73706 | 1.49064 | EAS | Crushing injury | |
| 1007 | -0.02951 | 0.354302 | -0.08329 | 0.933617 | -0.72393 | 0.664907 | EAS | Injury to blood vessels | |

| phecode | Coef. | Std.Err. | z | p_value_z | [0.025 | 0.975] | GIA | phenotype | category |
|---|---|---|---|---|---|---|---|---|---|
| 1008 | -0.2024 | 0.200459 | -1.00969 | 0.312645 | -0.59529 | 0.190492 | EAS | Crushing or internal injury to organ | |
| 1009 | -0.01419 | 0.096642 | -0.1468 | 0.88329 | -0.2036 | 0.175228 | EAS | Injury, NOS | |
| 1010 | -0.03447 | 0.063596 | -0.54209 | 0.587756 | -0.15912 | 0.090171 | EAS | Other tests | |
| 1011 | 0.064826 | 0.093605 | 0.692551 | 0.488591 | -0.11864 | 0.248289 | EAS | Complications of surgical and med | |
| 1012 | -0.28823 | 0.350671 | -0.82195 | 0.411106 | -0.97554 | 0.399069 | EAS | Late effect | |
| 1013 | 0.102522 | 0.079241 | 1.293799 | 0.195735 | -0.05279 | 0.257832 | EAS | Asphyxia and hypoxemia | |
| 1015 | 0.017732 | 0.08245 | 0.215062 | 0.829719 | -0.14387 | 0.179332 | EAS | Effects of other external causes | |
| 1019 | 0.106771 | 0.089909 | 1.187546 | 0.235012 | -0.06945 | 0.28299 | EAS | Other ill-defined and unknown cau | |
| 1100 | -0.36303 | 0.163197 | -2.22448 | 0.026116 | -0.68289 | -0.04317 | EAS | Family history | |

| phecode | Coef. | Std.Err. | z | p_value_z | [0.025 | 0.975] | GIA | phenotype | category |
|---|---|---|---|---|---|---|---|---|---|
| 8 | 0.157178 | 0.086235 | 1.822675 | 0.068353 | -0.01184 | 0.326194 | AFR | Intestinal infectio | infectious diseases |
| 8.5 | 0.252622 | 0.105653 | 2.391054 | 0.0168 | 0.045546 | 0.459699 | AFR | Bacterial enteritis | infectious diseases |
| 8.52 | 0.244803 | 0.15036 | 1.628117 | 0.1035 | -0.0499 | 0.539502 | AFR | Intestinal infectio | infectious diseases |
| 8.6 | 0.007232 | 0.215361 | 0.033583 | 0.97321 | -0.41487 | 0.429332 | AFR | Viral Enteritis | infectious diseases |
| 8.7 | 0.40748 | 0.485448 | 0.839388 | 0.401252 | -0.54398 | 1.358941 | AFR | Intestinal infectio | infectious diseases |
| 10 | 0.249889 | 0.238429 | 1.048066 | 0.294608 | -0.21742 | 0.717202 | AFR | Tuberculosis | infectious diseases |
| 31 | -0.46008 | 0.292192 | -1.57458 | 0.115353 | -1.03277 | 0.112605 | AFR | Diseases due to | infectious diseases |
| 38 | 0.093075 | 0.073709 | 1.262733 | 0.206685 | -0.05139 | 0.237542 | AFR | Septicemia | infectious diseases |
| 38.1 | 0.138019 | 0.14243 | 0.969032 | 0.332529 | -0.14114 | 0.417177 | AFR | Gram negative se | infectious diseases |
| 38.2 | 0.232822 | 0.203907 | 1.141808 | 0.253534 | -0.16683 | 0.632472 | AFR | Gram positive se | infectious diseases |
| 38.3 | 0.124496 | 0.115402 | 1.078803 | 0.280676 | -0.10169 | 0.35068 | AFR | Bacteremia | infectious diseases |
| 41 | 0.101029 | 0.063696 | 1.586111 | 0.112714 | -0.02381 | 0.225872 | AFR | Bacterial infectio | infectious diseases |
| 41.1 | 0.18122 | 0.121569 | 1.490669 | 0.136049 | -0.05705 | 0.419491 | AFR | Staphylococcus i | infectious diseases |
| 41.11 | 0.264798 | 0.178719 | 1.481649 | 0.138434 | -0.08548 | 0.61508 | AFR | Methicillin sensiti | infectious diseases |
| 41.12 | 0.078972 | 0.184029 | 0.429127 | 0.667831 | -0.28172 | 0.439661 | AFR | Methicillin resista | infectious diseases |
| 41.2 | 0.12488 | 0.141311 | 0.883721 | 0.376847 | -0.15209 | 0.401845 | AFR | Streptococcus in | infectious diseases |
| 41.21 | -0.35224 | 0.542635 | -0.64913 | 0.516255 | -1.41579 | 0.711305 | AFR | Rheumatic fever | infectious diseases |
| 41.4 | -0.00303 | 0.129136 | -0.02347 | 0.981276 | -0.25613 | 0.25007 | AFR | E. coli | infectious diseases |
| 41.8 | 0.317992 | 0.132709 | 2.396161 | 0.016568 | 0.057887 | 0.578098 | AFR | H. pylori | infectious diseases |
| 41.9 | 0.118267 | 0.162992 | 0.725602 | 0.468083 | -0.20119 | 0.437726 | AFR | Infection with dru | infectious diseases |
| 53 | 0.228748 | 0.124763 | 1.833467 | 0.066733 | -0.01578 | 0.473279 | AFR | Herpes zoster | infectious diseases |
| 53.1 | 0.192527 | 0.212911 | 0.904261 | 0.365857 | -0.22477 | 0.609824 | AFR | Herpes zoster wi | infectious diseases |
| 54 | -0.00469 | 0.103126 | -0.04552 | 0.963693 | -0.20682 | 0.197429 | AFR | Herpes simplex | infectious diseases |
| 70 | 0.145033 | 0.110227 | 1.315761 | 0.188254 | -0.07101 | 0.361074 | AFR | Viral hepatitis | infectious diseases |
| 70.1 | 0.007014 | 0.455604 | 0.015394 | 0.987718 | -0.88595 | 0.89998 | AFR | Viral hepatitis A | infectious diseases |
| 70.2 | 0.197122 | 0.19645 | 1.003418 | 0.315659 | -0.18791 | 0.582158 | AFR | Viral hepatitis B | infectious diseases |
| 70.3 | 0.237541 | 0.129305 | 1.83706 | 0.066201 | -0.01589 | 0.490973 | AFR | Viral hepatitis C | infectious diseases |
| 70.4 | 0.245636 | 0.204791 | 1.199443 | 0.230356 | -0.15575 | 0.64702 | AFR | Chronic hepatitis | infectious diseases |
| 70.9 | 0.111935 | 0.150905 | 0.741758 | 0.458234 | -0.18383 | 0.407705 | AFR | Hepatitis NOS | infectious diseases |
| 71 | 0.414689 | 0.195562 | 2.120495 | 0.033964 | 0.031394 | 0.797983 | AFR | Human immunod | infectious diseases |
| 71.1 | 0.414689 | 0.195562 | 2.120495 | 0.033964 | 0.031394 | 0.797983 | AFR | HIV infection, syr | infectious diseases |
| 78 | -0.05828 | 0.102927 | -0.56621 | 0.571251 | -0.26001 | 0.143455 | AFR | Viral warts & HP\ | infectious diseases |
| 79 | -0.01241 | 0.0581 | -0.21352 | 0.830924 | -0.12628 | 0.101469 | AFR | Viral infection | infectious diseases |
| 79.1 | -0.16448 | 0.133688 | -1.23034 | 0.218568 | -0.42651 | 0.097542 | AFR | Varicella infectior | infectious diseases |
| 79.2 | -0.47445 | 0.364535 | -1.30152 | 0.19308 | -1.18892 | 0.240025 | AFR | Infectious monon | infectious diseases |
| 79.9 | -0.0047 | 0.109128 | -0.04309 | 0.965632 | -0.21859 | 0.209185 | AFR | Viremia, NOS | infectious diseases |
| 80 | -0.08285 | 0.179079 | -0.46262 | 0.643634 | -0.43384 | 0.268143 | AFR | Postoperative inf | infectious diseases |

| | | | | | | | | | |
|---|---|---|---|---|---|---|---|---|---|
| 81 | 0.094166 | 0.133005 | 0.707993 | 0.478949 | -0.16652 | 0.354851 | AFR | Infection/inflamm | infectious diseases |
| 81.1 | -0.39002 | 0.347396 | -1.12269 | 0.261569 | -1.0709 | 0.290865 | AFR | Graft-versus-hos | infectious diseases |
| 81.11 | -0.44984 | 0.611132 | -0.73608 | 0.461682 | -1.64764 | 0.747955 | AFR | Acute graft-versu | infectious diseases |
| 81.12 | -0.26218 | 0.692611 | -0.37854 | 0.705032 | -1.61967 | 1.095315 | AFR | Chronic graft-ver | infectious diseases |
| 90 | 0.177167 | 0.162039 | 1.093363 | 0.274234 | -0.14042 | 0.494758 | AFR | Sexually transmit | infectious diseases |
| 90.2 | 0.08797 | 0.334046 | 0.263347 | 0.792283 | -0.56675 | 0.742688 | AFR | Gonococcal infec | infectious diseases |
| 90.3 | -0.68974 | 0.62797 | -1.09836 | 0.272048 | -1.92053 | 0.541062 | AFR | Venereal disease | infectious diseases |
| 110 | 0.125804 | 0.0682 | 1.844634 | 0.065091 | -0.00787 | 0.259473 | AFR | Dermatophytosis | infectious diseases |
| 110.1 | 0.103491 | 0.069991 | 1.47863 | 0.139239 | -0.03369 | 0.24067 | AFR | Dermatophytosis | infectious diseases |
| 110.11 | 0.105462 | 0.080556 | 1.309179 | 0.190474 | -0.05242 | 0.263348 | AFR | Dermatophytosis | infectious diseases |
| 110.12 | -0.0233 | 0.116112 | -0.20068 | 0.840949 | -0.25088 | 0.204274 | AFR | Althete's foot | infectious diseases |
| 110.13 | -0.03853 | 0.1533 | -0.25132 | 0.801563 | -0.33899 | 0.261934 | AFR | Dermatophytosis | infectious diseases |
| 110.2 | 0.026752 | 0.197052 | 0.135761 | 0.89201 | -0.35946 | 0.412966 | AFR | Dermatomycoses | infectious diseases |
| 112 | -0.04783 | 0.077878 | -0.6142 | 0.53908 | -0.20047 | 0.104804 | AFR | Candidiasis | infectious diseases |
| 112.3 | 0.264807 | 0.180259 | 1.469032 | 0.141824 | -0.08849 | 0.618108 | AFR | Candidiasis of sk | infectious diseases |
| 117 | -0.05648 | 0.138523 | -0.40771 | 0.683484 | -0.32798 | 0.215022 | AFR | Mycoses | infectious diseases |
| 117.1 | -0.68127 | 0.620743 | -1.0975 | 0.272423 | -1.8979 | 0.535368 | AFR | Histoplasmosis | infectious diseases |
| 117.2 | -0.84746 | 0.343507 | -2.46709 | 0.013622 | -1.52073 | -0.1742 | AFR | Coccidioidomyco | infectious diseases |
| 117.4 | -0.22071 | 0.275105 | -0.80228 | 0.422391 | -0.75991 | 0.318485 | AFR | Aspergillosis | infectious diseases |
| 130 | -0.03328 | 0.819138 | -0.04063 | 0.967593 | -1.63876 | 1.572202 | AFR | Spirochetal infec | infectious diseases |
| 131 | -0.46602 | 0.345006 | -1.35075 | 0.176777 | -1.14222 | 0.210184 | AFR | Protozoan infecti | infectious diseases |
| 132 | 0.13766 | 0.329593 | 0.417668 | 0.67619 | -0.50833 | 0.78365 | AFR | Infestation (lice, r | infectious diseases |
| 133 | 0.531561 | 0.561257 | 0.94709 | 0.343593 | -0.56848 | 1.631603 | AFR | Arthropod-borne | infectious diseases |
| 134 | -0.21565 | 0.711956 | -0.3029 | 0.761966 | -1.61106 | 1.179757 | AFR | Helminthiases | infectious diseases |
| 134.1 | -0.21565 | 0.711956 | -0.3029 | 0.761966 | -1.61106 | 1.179757 | AFR | Intestinal helmint | infectious diseases |
| 136 | 0.019287 | 0.071253 | 0.27068 | 0.786637 | -0.12037 | 0.158941 | AFR | Other infectious a | infectious diseases |
| 145 | 0.162227 | 0.288361 | 0.562584 | 0.573718 | -0.40295 | 0.727404 | AFR | Cancer of mouth | neoplasms |
| 145.2 | 0.311324 | 0.352827 | 0.882371 | 0.377576 | -0.3802 | 1.002852 | AFR | Cancer of tongue | neoplasms |
| 145.3 | -0.05847 | 0.681031 | -0.08585 | 0.931586 | -1.39326 | 1.27633 | AFR | Cancer of major s | neoplasms |
| 145.4 | 0.14018 | 0.880998 | 0.159115 | 0.873579 | -1.58654 | 1.866904 | AFR | Cancer of the gu | neoplasms |
| 145.5 | 2.049256 | 1.071125 | 1.913182 | 0.055725 | -0.05011 | 4.148622 | AFR | Cancer of the mo | neoplasms |
| 149 | 0.055688 | 0.255813 | 0.217692 | 0.827669 | -0.4457 | 0.557072 | AFR | Cancer of larynx, | neoplasms |
| 149.1 | 0.176714 | 0.338025 | 0.522783 | 0.601125 | -0.4858 | 0.839231 | AFR | Cancer of oropha | neoplasms |
| 149.2 | 0.490375 | 0.506316 | 0.968516 | 0.332787 | -0.50199 | 1.482737 | AFR | Cancer of nasopl | neoplasms |
| 149.3 | 0.316821 | 0.809485 | 0.391386 | 0.695512 | -1.26974 | 1.903383 | AFR | Cancer of hypopl | neoplasms |
| 149.4 | -0.03262 | 0.580685 | -0.05618 | 0.955196 | -1.17075 | 1.105496 | AFR | Cancer of larynx | neoplasms |
| 149.5 | 0.042162 | 0.343824 | 0.122627 | 0.902402 | -0.63172 | 0.716044 | AFR | Hx of malignant r | neoplasms |
| 149.9 | -0.85557 | 0.550348 | -1.55459 | 0.120043 | -1.93423 | 0.223094 | AFR | Cancer of of nasa | neoplasms |

| | | | | | | | | | | |
|---|---|---|---|---|---|---|---|---|---|---|
| 150 | 0.551193 | 0.349042 | 1.579158 | 0.1143 | -0.13292 | 1.235303 | AFR | Cancer of esopha | neoplasms | |
| 151 | 0.247481 | 0.275007 | 0.899908 | 0.368169 | -0.29152 | 0.786484 | AFR | Cancer of stoma | neoplasms | |
| 153 | 0.234446 | 0.135761 | 1.726904 | 0.084185 | -0.03164 | 0.500533 | AFR | Colorectal cance | neoplasms | |
| 153.2 | 0.220543 | 0.151011 | 1.460443 | 0.144168 | -0.07543 | 0.51652 | AFR | Colon cancer | neoplasms | |
| 153.3 | 0.288952 | 0.181284 | 1.593915 | 0.110955 | -0.06636 | 0.644263 | AFR | Malignant neopla | neoplasms | |
| 155 | 0.044266 | 0.180135 | 0.245737 | 0.805885 | -0.30879 | 0.397324 | AFR | Cancer of liver ar | neoplasms | |
| 155.1 | 0.080867 | 0.189213 | 0.427389 | 0.669096 | -0.28998 | 0.451717 | AFR | Malignant neopla | neoplasms | |
| 157 | -0.26555 | 0.265706 | -0.99943 | 0.317589 | -0.78633 | 0.255221 | AFR | Pancreatic cance | neoplasms | |
| 158 | 0.096779 | 0.174193 | 0.555585 | 0.578494 | -0.24463 | 0.438192 | AFR | Neoplasm of uns | neoplasms | |
| 159 | 0.313144 | 0.205535 | 1.523556 | 0.12762 | -0.0897 | 0.715985 | AFR | Malignant neopla | neoplasms | |
| 159.2 | 0.189901 | 0.299269 | 0.634551 | 0.525721 | -0.39665 | 0.776457 | AFR | Malignant neopla | neoplasms | |
| 159.3 | 0.302531 | 0.425924 | 0.710293 | 0.477522 | -0.53226 | 1.137327 | AFR | Malignant neopla | neoplasms | |
| 159.4 | -0.002 | 0.42483 | -0.00472 | 0.996238 | -0.83465 | 0.830648 | AFR | Malignant neopla | neoplasms | |
| 164 | 0.311085 | 0.50871 | 0.611517 | 0.540857 | -0.68597 | 1.308138 | AFR | Cancer of intrath | neoplasms | |
| 165 | -0.21765 | 0.1898 | -1.14675 | 0.251484 | -0.58966 | 0.154348 | AFR | Cancer within the | neoplasms | |
| 165.1 | -0.24988 | 0.195633 | -1.27729 | 0.201501 | -0.63331 | 0.133554 | AFR | Cancer of bronch | neoplasms | |
| 170 | 0.075304 | 0.16598 | 0.453695 | 0.650048 | -0.25001 | 0.400619 | AFR | Cancer of bone a | neoplasms | |
| 170.1 | 0.192137 | 0.231063 | 0.831536 | 0.405671 | -0.26074 | 0.645012 | AFR | Bone cancer | neoplasms | |
| 170.2 | 0.057848 | 0.20326 | 0.284602 | 0.775949 | -0.34053 | 0.456231 | AFR | Cancer of conne | neoplasms | |
| 172 | 0.159748 | 0.180279 | 0.886114 | 0.375556 | -0.19359 | 0.513088 | AFR | Skin cancer | neoplasms | |
| 172.1 | 1.272921 | 0.482752 | 2.636803 | 0.008369 | 0.326745 | 2.219096 | AFR | Melanomas of sk | neoplasms | |
| 172.11 | 1.887253 | 0.66049 | 2.857354 | 0.004272 | 0.592717 | 3.181789 | AFR | Melanomas of sk | neoplasms | |
| 172.2 | -0.03257 | 0.198268 | -0.16425 | 0.869533 | -0.42116 | 0.356032 | AFR | Other non-epithe | neoplasms | |
| 172.21 | -0.11631 | 0.437105 | -0.2661 | 0.790166 | -0.97302 | 0.740399 | AFR | Basal cell carcin | neoplasms | |
| 172.22 | -0.11658 | 0.367278 | -0.31743 | 0.750921 | -0.83643 | 0.603268 | AFR | Squamous cell ca | neoplasms | |
| 172.3 | -0.87239 | 0.516188 | -1.69007 | 0.091014 | -1.8841 | 0.139315 | AFR | Carcinoma in situ | neoplasms | |
| 173 | 0.161921 | 0.106225 | 1.524321 | 0.127428 | -0.04628 | 0.370117 | AFR | Neoplasm of unc | neoplasms | |
| 174 | 0.015593 | 0.10795 | 0.144451 | 0.885145 | -0.19598 | 0.227171 | AFR | Breast cancer | neoplasms | |
| 174.1 | 0.007072 | 0.108579 | 0.065129 | 0.948071 | -0.20574 | 0.219882 | AFR | Breast cancer [fe | neoplasms | |
| 174.11 | 0.020728 | 0.108917 | 0.190314 | 0.849063 | -0.19274 | 0.234202 | AFR | Malignant neopla | neoplasms | |
| 174.2 | -0.54742 | 0.628912 | -0.87043 | 0.384067 | -1.78007 | 0.685223 | AFR | Breast cancer [m | neoplasms | |
| 174.3 | 0.647564 | 0.811623 | 0.797863 | 0.42495 | -0.94319 | 2.238317 | AFR | Neoplasm of unc | neoplasms | |
| 175 | -0.0533 | 0.128942 | -0.41336 | 0.679346 | -0.30602 | 0.199423 | AFR | Acquired absenc | neoplasms | |
| 180 | 0.098387 | 0.16838 | 0.584313 | 0.55901 | -0.23163 | 0.428405 | AFR | Cervical cancer a | neoplasms | |
| 180.1 | 0.273089 | 0.364037 | 0.750167 | 0.453154 | -0.44041 | 0.986589 | AFR | Cervical cancer | neoplasms | |
| 180.3 | 0.077611 | 0.18309 | 0.423894 | 0.671643 | -0.28124 | 0.43646 | AFR | Cervical intraepit | neoplasms | |
| 182 | 0.008949 | 0.248586 | 0.036001 | 0.971281 | -0.47827 | 0.49617 | AFR | Malignant neopla | neoplasms | |
| 184 | 0.269115 | 0.203257 | 1.324015 | 0.185498 | -0.12926 | 0.667492 | AFR | Cancer of other f | neoplasms | |

| | | | | | | | | | | |
|---|---|---|---|---|---|---|---|---|---|---|
| 184.1 | 0.349393 | 0.230533 | 1.515591 | 0.129623 | -0.10244 | 0.801229 | AFR | Malignant neopla | neoplasms | |
| 184.11 | 0.312622 | 0.265521 | 1.177393 | 0.239039 | -0.20779 | 0.833033 | AFR | Malignant neopla | neoplasms | |
| 184.2 | 0.138587 | 0.321353 | 0.431261 | 0.666279 | -0.49125 | 0.768428 | AFR | Cancer of other f | neoplasms | |
| 185 | 0.106007 | 0.128186 | 0.826975 | 0.408251 | -0.14523 | 0.357247 | AFR | Cancer of prostat | neoplasms | |
| 187 | 0.213947 | 0.646637 | 0.330861 | 0.740749 | -1.05344 | 1.481333 | AFR | Cancer of other r | neoplasms | |
| 187.1 | 0.23586 | 0.815619 | 0.289179 | 0.772445 | -1.36272 | 1.834444 | AFR | Malignant neopla | neoplasms | |
| 187.8 | 0.483804 | 1.085121 | 0.445853 | 0.655704 | -1.64299 | 2.610603 | AFR | Neoplasm of unc | neoplasms | |
| 189 | 0.018528 | 0.146914 | 0.126112 | 0.899643 | -0.26942 | 0.306473 | AFR | Cancer of urinary | neoplasms | |
| 189.1 | 0.058072 | 0.168054 | 0.345553 | 0.729679 | -0.27131 | 0.387452 | AFR | Cancer of kidney | neoplasms | |
| 189.11 | 0.074136 | 0.178455 | 0.415431 | 0.677826 | -0.27563 | 0.423902 | AFR | Malignant neopla | neoplasms | |
| 189.12 | -0.4173 | 0.342611 | -1.218 | 0.223225 | -1.0888 | 0.254206 | AFR | Malignant neopla | neoplasms | |
| 189.2 | -0.35374 | 0.276709 | -1.27839 | 0.201113 | -0.89608 | 0.188599 | AFR | Cancer of bladde | neoplasms | |
| 189.21 | -0.48601 | 0.256033 | -1.89822 | 0.057667 | -0.98783 | 0.015808 | AFR | Malignant neopla | neoplasms | |
| 189.4 | -0.41474 | 0.440107 | -0.94236 | 0.346006 | -1.27734 | 0.447853 | AFR | Malignant neopla | neoplasms | |
| 190 | 21.75516 | 4612.168 | 0.004717 | 0.996236 | -9017.93 | 9061.438 | AFR | Cancer of eye | neoplasms | |
| 191 | 0.090942 | 0.222909 | 0.407976 | 0.683291 | -0.34595 | 0.527836 | AFR | Manlignant and u | neoplasms | |
| 191.1 | 0.21283 | 0.281926 | 0.754914 | 0.450301 | -0.33974 | 0.765395 | AFR | Cancer of brain a | neoplasms | |
| 191.11 | 0.005973 | 0.29358 | 0.020345 | 0.983768 | -0.56943 | 0.581379 | AFR | Cancer of brain | neoplasms | |
| 193 | -0.24469 | 0.187245 | -1.3068 | 0.191281 | -0.61168 | 0.122302 | AFR | Thyroid cancer | neoplasms | |
| 194 | 0.734192 | 0.797607 | 0.920494 | 0.357315 | -0.82909 | 2.297473 | AFR | Cancer of other e | neoplasms | |
| 195 | -0.03584 | 0.077047 | -0.46523 | 0.641768 | -0.18685 | 0.115165 | AFR | Cancer, suspecte | neoplasms | |
| 195.1 | 0.015294 | 0.08164 | 0.187333 | 0.851399 | -0.14472 | 0.175306 | AFR | Malignant neopla | neoplasms | |
| 195.3 | 0.032736 | 0.240344 | 0.136203 | 0.891661 | -0.43833 | 0.503801 | AFR | Malignant neopla | neoplasms | |
| 196 | 0.063296 | 0.056985 | 1.110739 | 0.266681 | -0.04839 | 0.174985 | AFR | Radiotherapy | neoplasms | |
| 197 | 0.066444 | 0.054951 | 1.209156 | 0.226603 | -0.04126 | 0.174145 | AFR | Chemotherapy | neoplasms | |
| 198 | 0.037225 | 0.093344 | 0.398796 | 0.690044 | -0.14573 | 0.220175 | AFR | Secondary malig | neoplasms | |
| 198.1 | 0.035298 | 0.115964 | 0.304383 | 0.760836 | -0.19199 | 0.262584 | AFR | Secondary malig | neoplasms | |
| 198.2 | 0.005926 | 0.168898 | 0.035084 | 0.972013 | -0.32511 | 0.336959 | AFR | Secondary malig | neoplasms | |
| 198.3 | 0.071376 | 0.191741 | 0.37225 | 0.709706 | -0.30443 | 0.447182 | AFR | Secondary malig | neoplasms | |
| 198.4 | 0.214149 | 0.177871 | 1.20396 | 0.228605 | -0.13447 | 0.56277 | AFR | Secondary malig | neoplasms | |
| 198.5 | -0.05001 | 0.252861 | -0.19778 | 0.843219 | -0.54561 | 0.445588 | AFR | Secondary malig | neoplasms | |
| 198.6 | -0.35135 | 0.170041 | -2.06627 | 0.038803 | -0.68463 | -0.01808 | AFR | Secondary malig | neoplasms | |
| 198.7 | 0.248193 | 0.467654 | 0.530719 | 0.595614 | -0.66839 | 1.164779 | AFR | Secondary malig | neoplasms | |
| 199 | 0.026159 | 0.089777 | 0.291376 | 0.770764 | -0.1498 | 0.202119 | AFR | Neoplasm of unc | neoplasms | |
| 200 | -0.09415 | 0.114271 | -0.82393 | 0.409981 | -0.31812 | 0.129816 | AFR | Myeloproliferative | neoplasms | |
| 200.1 | 0.035162 | 0.272838 | 0.128874 | 0.897458 | -0.49959 | 0.569914 | AFR | Polycythemia ver | neoplasms | |
| 201 | 0.055961 | 0.545052 | 0.102671 | 0.918224 | -1.01232 | 1.124242 | AFR | Hodgkin's diseas | neoplasms | |
| 202 | -0.20131 | 0.191243 | -1.05264 | 0.292506 | -0.57614 | 0.173519 | AFR | Cancer of other l | neoplasms | |

| | | | | | | | | | | |
|---|---|---|---|---|---|---|---|---|---|---|
| 202.2 | -0.29724 | 0.215436 | -1.37971 | 0.167675 | -0.71949 | 0.125007 | AFR | | Non-Hodgkins lyı | neoplasms |
| 202.21 | -0.03502 | 0.436742 | -0.08018 | 0.936096 | -0.89102 | 0.820981 | AFR | | Nodular lymphon | neoplasms |
| 202.22 | -0.65358 | 0.474035 | -1.37875 | 0.167971 | -1.58267 | 0.275515 | AFR | | Reticulosarcoma | neoplasms |
| 202.23 | -0.40563 | 0.53024 | -0.76499 | 0.444277 | -1.44488 | 0.633622 | AFR | | Lymphosarcoma | neoplasms |
| 202.24 | 0.725692 | 1.392658 | 0.521084 | 0.602308 | -2.00387 | 3.455251 | AFR | | Large cell lymphc | neoplasms |
| 204 | -0.09248 | 0.173553 | -0.53288 | 0.594118 | -0.43264 | 0.247675 | AFR | | Leukemia | neoplasms |
| 204.1 | -0.15388 | 0.338204 | -0.45501 | 0.649105 | -0.81675 | 0.508982 | AFR | | Lymphoid leuken | neoplasms |
| 204.11 | 0.134245 | 0.668588 | 0.200789 | 0.840863 | -1.17616 | 1.444654 | AFR | | Lymphoid leuken | neoplasms |
| 204.12 | 0.476015 | 0.5356 | 0.888751 | 0.374137 | -0.57374 | 1.525771 | AFR | | Lymphoid leuken | neoplasms |
| 204.2 | 0.321294 | 0.332638 | 0.965898 | 0.334095 | -0.33066 | 0.973252 | AFR | | Myeloid leukemia | neoplasms |
| 204.21 | 0.201289 | 0.329188 | 0.611471 | 0.540888 | -0.44391 | 0.846485 | AFR | | Myeloid leukemia | neoplasms |
| 204.22 | 0.912622 | 0.90524 | 1.008155 | 0.31338 | -0.86162 | 2.68686 | AFR | | Myeloid leukemia | neoplasms |
| 204.3 | 0.547588 | 1.069226 | 0.512135 | 0.608557 | -1.54806 | 2.643231 | AFR | | Monocytic leuker | neoplasms |
| 204.4 | -0.08669 | 0.229825 | -0.37719 | 0.706031 | -0.53714 | 0.36376 | AFR | | Multiple myeloma | neoplasms |
| 208 | 0.054474 | 0.06393 | 0.852094 | 0.394162 | -0.07083 | 0.179775 | AFR | | Benign neoplasm | neoplasms |
| 209 | 0.075301 | 0.292498 | 0.257441 | 0.796839 | -0.49798 | 0.648585 | AFR | | Neuroendocrine t | neoplasms |
| 210 | -0.16966 | 0.355102 | -0.47779 | 0.6328 | -0.86565 | 0.526323 | AFR | | Benign neoplasm | neoplasms |
| 211 | 0.037651 | 0.138743 | 0.271374 | 0.786103 | -0.23428 | 0.309582 | AFR | | Benign neoplasm | neoplasms |
| 212 | -0.54788 | 0.258763 | -2.11729 | 0.034235 | -1.05504 | -0.04071 | AFR | | Benign neoplasm | neoplasms |
| 213 | 0.255869 | 0.308484 | 0.82944 | 0.406856 | -0.34875 | 0.860486 | AFR | | Benign neoplasm | neoplasms |
| 214 | 0.20806 | 0.118689 | 1.752987 | 0.079604 | -0.02457 | 0.440686 | AFR | | Lipoma | neoplasms |
| 214.1 | 0.119735 | 0.145528 | 0.822762 | 0.410644 | -0.16549 | 0.404964 | AFR | | Lipoma of skin ar | neoplasms |
| 215 | 0.07349 | 0.090191 | 0.814833 | 0.415168 | -0.10328 | 0.250261 | AFR | | Other benign nec | neoplasms |
| 216 | -0.01054 | 0.084829 | -0.12428 | 0.901094 | -0.1768 | 0.15572 | AFR | | Benign neoplasm | neoplasms |
| 216.1 | 0.171653 | 0.098651 | 1.740007 | 0.081858 | -0.0217 | 0.365004 | AFR | | Screening for ma | neoplasms |
| 217 | -0.13825 | 0.171316 | -0.80696 | 0.419689 | -0.47402 | 0.197528 | AFR | | Vascular hamartc | neoplasms |
| 217.1 | -0.10088 | 0.18492 | -0.54555 | 0.585374 | -0.46332 | 0.261553 | AFR | | Nevus, non-neop | neoplasms |
| 218 | 0.114507 | 0.079087 | 1.447872 | 0.147653 | -0.0405 | 0.269514 | AFR | | Benign neoplasm | neoplasms |
| 218.1 | 0.114507 | 0.079087 | 1.447872 | 0.147653 | -0.0405 | 0.269514 | AFR | | Uterine leiomyon | neoplasms |
| 218.2 | -0.35625 | 0.542274 | -0.65696 | 0.511208 | -1.41909 | 0.706587 | AFR | | Other benign nec | neoplasms |
| 220 | -0.47053 | 0.259127 | -1.81583 | 0.069397 | -0.97841 | 0.03735 | AFR | | Benign neoplasm | neoplasms |
| 221 | 0.128481 | 0.746542 | 0.172102 | 0.863358 | -1.33471 | 1.591677 | AFR | | Benign neoplasm | neoplasms |
| 222 | -0.39858 | 1.188463 | -0.33537 | 0.737345 | -2.72792 | 1.930768 | AFR | | Benign neoplasm | neoplasms |
| 223 | 0.596332 | 0.307281 | 1.940669 | 0.052298 | -0.00593 | 1.198592 | AFR | | Benign neoplasm | neoplasms |
| 224 | -0.07182 | 0.287529 | -0.24979 | 0.802749 | -0.63537 | 0.491725 | AFR | | Benign neoplasm | neoplasms |
| 224.1 | 0.000489 | 0.358832 | 0.001362 | 0.998913 | -0.70281 | 0.703787 | AFR | | Benign neoplasm | neoplasms |
| 225 | 0.205277 | 0.212358 | 0.966657 | 0.333715 | -0.21094 | 0.62149 | AFR | | Benign neoplasm | neoplasms |
| 225.1 | 0.205277 | 0.212358 | 0.966657 | 0.333715 | -0.21094 | 0.62149 | AFR | | Benign neoplasm | neoplasms |

| | | | | | | | | | |
|---|---|---|---|---|---|---|---|---|---|
| 226 | 0.034023 | 0.332438 | 0.102344 | 0.918484 | -0.61754 | 0.685589 | AFR | Benign neoplasm | neoplasms |
| 227 | -0.12661 | 0.140396 | -0.90184 | 0.367142 | -0.40178 | 0.148556 | AFR | Benign neoplasm | neoplasms |
| 227.1 | 0.002906 | 0.216591 | 0.013419 | 0.989294 | -0.4216 | 0.427417 | AFR | Benign neoplasm | neoplasms |
| 227.2 | -0.24926 | 0.215594 | -1.15617 | 0.247612 | -0.67182 | 0.173293 | AFR | Benign neoplasm | neoplasms |
| 227.3 | -0.2023 | 0.261373 | -0.774 | 0.438928 | -0.71458 | 0.309977 | AFR | Benign neoplasm | neoplasms |
| 228 | -0.01444 | 0.126435 | -0.11425 | 0.909041 | -0.26225 | 0.233362 | AFR | Hemangioma and | neoplasms |
| 228.1 | 0.028806 | 0.16904 | 0.170407 | 0.86469 | -0.30251 | 0.360118 | AFR | Hemangioma of s | neoplasms |
| 229 | -0.15469 | 0.187768 | -0.82383 | 0.410038 | -0.52271 | 0.21333 | AFR | Benign neoplasm | neoplasms |
| 230 | -0.84901 | 0.633216 | -1.3408 | 0.179987 | -2.09009 | 0.392067 | AFR | Kaposi's sarcoma | neoplasms |
| 240 | -0.13389 | 0.119708 | -1.11847 | 0.263366 | -0.36851 | 0.100734 | AFR | Simple and unsp | endocrine/metabolic |
| 241 | -0.12592 | 0.072541 | -1.73578 | 0.082602 | -0.26809 | 0.016262 | AFR | Nontoxic nodular | endocrine/metabolic |
| 241.1 | -0.09615 | 0.080698 | -1.19153 | 0.233446 | -0.25432 | 0.062011 | AFR | Nontoxic uninodu | endocrine/metabolic |
| 241.2 | -0.10213 | 0.087427 | -1.16814 | 0.242751 | -0.27348 | 0.069227 | AFR | Nontoxic multino | endocrine/metabolic |
| 242 | -0.15981 | 0.119012 | -1.3428 | 0.179336 | -0.39307 | 0.07345 | AFR | Thyrotoxicosis w | endocrine/metabolic |
| 242.1 | -0.10548 | 0.189106 | -0.55779 | 0.576988 | -0.47612 | 0.265159 | AFR | Graves' disease | endocrine/metabolic |
| 242.2 | 0.475133 | 0.342968 | 1.385355 | 0.165944 | -0.19707 | 1.147339 | AFR | Toxic multinodula | endocrine/metabolic |
| 242.3 | -0.09008 | 0.222794 | -0.40432 | 0.685974 | -0.52675 | 0.346587 | AFR | Exophthalmos | endocrine/metabolic |
| 242.31 | -0.13311 | 0.409763 | -0.32485 | 0.745294 | -0.93623 | 0.670009 | AFR | Thyrotoxic exoph | endocrine/metabolic |
| 244 | -0.1686 | 0.073733 | -2.2866 | 0.022219 | -0.31311 | -0.02408 | AFR | Hypothyroidism | endocrine/metabolic |
| 244.1 | -0.22343 | 0.128253 | -1.74207 | 0.081496 | -0.4748 | 0.027946 | AFR | Secondary hypot | endocrine/metabolic |
| 244.2 | 0.022262 | 0.110426 | 0.201605 | 0.840226 | -0.19417 | 0.238694 | AFR | Acquired hypothy | endocrine/metabolic |
| 244.3 | -0.17197 | 1.024355 | -0.16788 | 0.86668 | -2.17966 | 1.835733 | AFR | Iodine hypothyroi | endocrine/metabolic |
| 244.4 | -0.20714 | 0.076954 | -2.6918 | 0.007107 | -0.35797 | -0.05632 | AFR | Hypothyroidism N | endocrine/metabolic |
| 244.5 | -0.38394 | 0.426519 | -0.90017 | 0.368031 | -1.2199 | 0.452024 | AFR | Congenital hypot | endocrine/metabolic |
| 245 | 0.017482 | 0.156504 | 0.111702 | 0.91106 | -0.28926 | 0.324224 | AFR | Thyroiditis | endocrine/metabolic |
| 245.1 | -0.99274 | 0.627326 | -1.5825 | 0.113536 | -2.22228 | 0.236795 | AFR | Thyroiditis, acute | endocrine/metabolic |
| 245.2 | 0.001375 | 0.176744 | 0.007781 | 0.993791 | -0.34504 | 0.347788 | AFR | Chronic thyroiditi | endocrine/metabolic |
| 245.21 | 0.001375 | 0.176744 | 0.007781 | 0.993791 | -0.34504 | 0.347788 | AFR | Chronic lymphoc | endocrine/metabolic |
| 246 | -0.25521 | 0.092215 | -2.76752 | 0.005648 | -0.43594 | -0.07447 | AFR | Other disorders c | endocrine/metabolic |
| 246.2 | 0.089193 | 0.2501 | 0.35663 | 0.721369 | -0.40099 | 0.579381 | AFR | Thyroid cyst | endocrine/metabolic |
| 246.7 | -0.12644 | 0.129887 | -0.9735 | 0.330305 | -0.38102 | 0.128129 | AFR | Abnormal results | endocrine/metabolic |
| 249 | 0.164112 | 0.091772 | 1.788247 | 0.073736 | -0.01576 | 0.343983 | AFR | Secondary diabe | endocrine/metabolic |
| 250 | 0.013574 | 0.057896 | 0.234458 | 0.81463 | -0.0999 | 0.127049 | AFR | Diabetes mellitus | endocrine/metabolic |
| 250.1 | 0.29661 | 0.136886 | 2.166836 | 0.030247 | 0.028318 | 0.564901 | AFR | Type 1 diabetes | endocrine/metabolic |
| 250.11 | 0.095078 | 0.444436 | 0.213929 | 0.830603 | -0.776 | 0.966156 | AFR | Type 1 diabetes w | endocrine/metabolic |
| 250.12 | 0.010803 | 0.228961 | 0.047184 | 0.962366 | -0.43795 | 0.459558 | AFR | Type 1 diabetes w | endocrine/metabolic |
| 250.13 | -0.01325 | 0.282467 | -0.04692 | 0.962576 | -0.56688 | 0.540371 | AFR | Type 1 diabetes w | endocrine/metabolic |
| 250.14 | -0.00962 | 0.286141 | -0.03362 | 0.973182 | -0.57044 | 0.551206 | AFR | Type 1 diabetes w | endocrine/metabolic |

| | | | | | | | | | |
|---|---|---|---|---|---|---|---|---|---|
| 250.15 | -0.59063 | 0.494669 | -1.19399 | 0.23248 | -1.56016 | 0.378901 | AFR | Diabetes type 1 v | endocrine/metabolic |
| 250.2 | 0.015165 | 0.058414 | 0.259609 | 0.795165 | -0.09932 | 0.129654 | AFR | Type 2 diabetes | endocrine/metabolic |
| 250.21 | 0.477077 | 0.262438 | 1.817865 | 0.069085 | -0.03729 | 0.991446 | AFR | Type 2 diabetes v | endocrine/metabolic |
| 250.22 | 0.06371 | 0.079008 | 0.806378 | 0.420025 | -0.09114 | 0.218563 | AFR | Type 2 diabetes v | endocrine/metabolic |
| 250.23 | 0.145277 | 0.102565 | 1.416435 | 0.156648 | -0.05575 | 0.346302 | AFR | Type 2 diabetes v | endocrine/metabolic |
| 250.24 | 0.068645 | 0.089207 | 0.769506 | 0.441593 | -0.1062 | 0.243488 | AFR | Type 2 diabetes v | endocrine/metabolic |
| 250.25 | 0.105638 | 0.089403 | 1.181597 | 0.237366 | -0.06959 | 0.280865 | AFR | Diabetes type 2 v | endocrine/metabolic |
| 250.3 | 0.032036 | 0.078971 | 0.405665 | 0.684988 | -0.12274 | 0.186815 | AFR | Insulin pump use | endocrine/metabolic |
| 250.4 | -0.05328 | 0.055445 | -0.96092 | 0.336591 | -0.16195 | 0.055392 | AFR | Abnormal glucos | endocrine/metabolic |
| 250.41 | -0.02235 | 0.095752 | -0.23339 | 0.815457 | -0.21002 | 0.165322 | AFR | Impaired fasting | endocrine/metabolic |
| 250.42 | -0.06309 | 0.055542 | -1.13589 | 0.256003 | -0.17195 | 0.045771 | AFR | Other abnormal g | endocrine/metabolic |
| 250.5 | 0.456321 | 0.388739 | 1.173849 | 0.240455 | -0.30559 | 1.218236 | AFR | Glycosuria or Ace | endocrine/metabolic |
| 250.6 | 0.052934 | 0.097851 | 0.540969 | 0.588529 | -0.13885 | 0.244719 | AFR | Polyneuropathy i | endocrine/metabolic |
| 250.7 | 0.073372 | 0.122166 | 0.600588 | 0.548115 | -0.16607 | 0.312814 | AFR | Diabetic retinopa | endocrine/metabolic |
| 251 | 0.651047 | 0.80292 | 0.81085 | 0.417452 | -0.92265 | 2.224741 | AFR | Other disorders c | endocrine/metabolic |
| 251.1 | 0.059595 | 0.135857 | 0.438659 | 0.660909 | -0.20668 | 0.325869 | AFR | Hypoglycemia | endocrine/metabolic |
| 251.8 | 0.718779 | 1.02796 | 0.699229 | 0.484409 | -1.29599 | 2.733544 | AFR | Abnormality of se | endocrine/metabolic |
| 252 | -0.04233 | 0.091027 | -0.46497 | 0.64195 | -0.22074 | 0.136085 | AFR | Disorders of para | endocrine/metabolic |
| 252.1 | -0.06058 | 0.093944 | -0.64488 | 0.519002 | -0.24471 | 0.123543 | AFR | Hyperparathyroid | endocrine/metabolic |
| 252.2 | 0.147743 | 0.246644 | 0.599011 | 0.549165 | -0.33567 | 0.631156 | AFR | Hypoparathyroidi | endocrine/metabolic |
| 253 | -0.11756 | 0.150235 | -0.78253 | 0.433903 | -0.41202 | 0.176892 | AFR | Disorders of the | endocrine/metabolic |
| 253.1 | -0.85569 | 0.280138 | -3.05452 | 0.002254 | -1.40475 | -0.30663 | AFR | Pituitary hyperfur | endocrine/metabolic |
| 253.2 | -0.58898 | 0.42664 | -1.38052 | 0.167427 | -1.42518 | 0.247215 | AFR | Pituitary hypofun | endocrine/metabolic |
| 253.3 | -0.13293 | 0.366231 | -0.36296 | 0.716637 | -0.85073 | 0.584874 | AFR | Diabetes insipidu | endocrine/metabolic |
| 253.5 | -14.0839 | 727.6309 | -0.01936 | 0.984557 | -1440.21 | 1412.046 | AFR | Pituitary dwarfisn | endocrine/metabolic |
| 253.7 | 0.306102 | 0.24705 | 1.23903 | 0.215334 | -0.17811 | 0.790311 | AFR | Other disorders c | endocrine/metabolic |
| 254 | 0.077482 | 0.746129 | 0.103845 | 0.917292 | -1.3849 | 1.539868 | AFR | Diseases of thym | endocrine/metabolic |
| 255 | 0.11684 | 0.11543 | 1.012214 | 0.311436 | -0.1094 | 0.343078 | AFR | Disorders of adre | endocrine/metabolic |
| 255.1 | 0.247522 | 0.214421 | 1.154377 | 0.248346 | -0.17273 | 0.667779 | AFR | Adrenal hyperfun | endocrine/metabolic |
| 255.11 | -0.07148 | 0.481555 | -0.14844 | 0.881999 | -1.01531 | 0.87235 | AFR | Cushing's syndrc | endocrine/metabolic |
| 255.12 | 0.287138 | 0.243432 | 1.179543 | 0.238182 | -0.18998 | 0.764256 | AFR | Hyperaldosteroni | endocrine/metabolic |
| 255.13 | 1.043982 | 1.082611 | 0.964318 | 0.334886 | -1.0779 | 3.165861 | AFR | Medulloadrenal h | endocrine/metabolic |
| 255.2 | -0.2215 | 0.182521 | -1.21354 | 0.224924 | -0.57923 | 0.136238 | AFR | Adrenal hypofunc | endocrine/metabolic |
| 255.21 | -0.2215 | 0.182521 | -1.21354 | 0.224924 | -0.57923 | 0.136238 | AFR | Glucocorticoid de | endocrine/metabolic |
| 255.22 | 1.265363 | 0.60202 | 2.101861 | 0.035565 | 0.085425 | 2.445301 | AFR | Mineralocorticoid | endocrine/metabolic |
| 255.3 | -0.26067 | 0.506427 | -0.51472 | 0.606748 | -1.25325 | 0.73191 | AFR | Adrenogenital dis | endocrine/metabolic |
| 256 | 0.071743 | 0.212089 | 0.33827 | 0.735159 | -0.34394 | 0.487431 | AFR | Ovarian dysfunct | endocrine/metabolic |
| 256.1 | 0.923913 | 1.259696 | 0.733441 | 0.46329 | -1.54505 | 3.392872 | AFR | Hyperestrogenisr | endocrine/metabolic |

| | | | | | | | | | |
|---|---|---|---|---|---|---|---|---|---|
| 256.4 | -0.02387 | 0.241285 | -0.09892 | 0.921202 | -0.49678 | 0.449043 | AFR | Polycystic ovarie | endocrine/metabolic |
| 257 | 0.362006 | 0.166768 | 2.170722 | 0.029952 | 0.035148 | 0.688865 | AFR | Testicular dysfun | endocrine/metabolic |
| 257.1 | 0.362006 | 0.166768 | 2.170722 | 0.029952 | 0.035148 | 0.688865 | AFR | Testicular hypofu | endocrine/metabolic |
| 258 | 0.254954 | 0.678076 | 0.375997 | 0.706919 | -1.07405 | 1.583959 | AFR | Iatrogenic endoc | endocrine/metabolic |
| 258.1 | 0.058295 | 0.403103 | 0.144617 | 0.885013 | -0.73177 | 0.848362 | AFR | Postablative ova | endocrine/metabolic |
| 259 | 0.189564 | 0.129689 | 1.461685 | 0.143828 | -0.06462 | 0.44375 | AFR | Other endocrine | endocrine/metabolic |
| 259.2 | 0.373393 | 0.66079 | 0.565071 | 0.572025 | -0.92173 | 1.668517 | AFR | Carcinoid syndro | endocrine/metabolic |
| 259.4 | -0.90556 | 0.792145 | -1.14318 | 0.252965 | -2.45814 | 0.647013 | AFR | Precocious sexu | endocrine/metabolic |
| 259.8 | -0.51188 | 0.687053 | -0.74504 | 0.456248 | -1.85848 | 0.834718 | AFR | Polyglandular ac | endocrine/metabolic |
| 260 | 0.092205 | 0.081759 | 1.127759 | 0.259422 | -0.06804 | 0.25245 | AFR | Protein-calorie m | endocrine/metabolic |
| 260.1 | 0.08161 | 0.169627 | 0.481115 | 0.630435 | -0.25085 | 0.414074 | AFR | Cachexia | endocrine/metabolic |
| 260.2 | 0.090518 | 0.112546 | 0.804271 | 0.421241 | -0.13007 | 0.311104 | AFR | severe protein-ca | endocrine/metabolic |
| 260.21 | 0.130278 | 1.196798 | 0.108856 | 0.913317 | -2.2154 | 2.475959 | AFR | Kwashiorkor | endocrine/metabolic |
| 260.22 | 0.180361 | 0.33957 | 0.531145 | 0.595318 | -0.48518 | 0.845907 | AFR | Nutritional maras | endocrine/metabolic |
| 260.3 | 0.015289 | 0.136368 | 0.112116 | 0.910732 | -0.25199 | 0.282566 | AFR | Adult failure to th | endocrine/metabolic |
| 260.6 | -0.08899 | 0.120754 | -0.73692 | 0.461169 | -0.32566 | 0.147688 | AFR | Anorexia | endocrine/metabolic |
| 260.7 | -0.42068 | 0.543262 | -0.77436 | 0.438719 | -1.48545 | 0.644095 | AFR | Polyphagia | endocrine/metabolic |
| 261 | -0.0716 | 0.056245 | -1.27301 | 0.203014 | -0.18184 | 0.038638 | AFR | Vitamin deficienc | endocrine/metabolic |
| 261.1 | 0.055126 | 0.436666 | 0.126243 | 0.899539 | -0.80072 | 0.910977 | AFR | Vitamin A deficie | endocrine/metabolic |
| 261.2 | -0.09066 | 0.090288 | -1.00407 | 0.315345 | -0.26762 | 0.086306 | AFR | Vitamin B-comple | endocrine/metabolic |
| 261.4 | -0.05042 | 0.056607 | -0.89065 | 0.373118 | -0.16136 | 0.060531 | AFR | Vitamin D deficie | endocrine/metabolic |
| 261.41 | -0.34781 | 0.539427 | -0.64477 | 0.519074 | -1.40507 | 0.709449 | AFR | Rickets or osteor | endocrine/metabolic |
| 262 | 0.323005 | 0.132877 | 2.430853 | 0.015063 | 0.06257 | 0.583439 | AFR | Mineral deficienc | endocrine/metabolic |
| 263 | -0.09391 | 0.095835 | -0.97994 | 0.327118 | -0.28175 | 0.093921 | AFR | Other nutritional | endocrine/metabolic |
| 264 | 0.411716 | 0.271897 | 1.514236 | 0.129966 | -0.12119 | 0.944623 | AFR | Lack of normal p | endocrine/metabolic |
| 264.1 | -0.27685 | 0.756585 | -0.36592 | 0.714426 | -1.75973 | 1.206031 | AFR | Short stature | endocrine/metabolic |
| 264.2 | 0.305392 | 0.383572 | 0.79618 | 0.425928 | -0.44639 | 1.057179 | AFR | Failure to thrive ( | endocrine/metabolic |
| 264.9 | 0.876081 | 0.468143 | 1.871397 | 0.06129 | -0.04146 | 1.793624 | AFR | Lack of normal p | endocrine/metabolic |
| 269 | 9.27E-05 | 0.082955 | 0.001117 | 0.999108 | -0.1625 | 0.162682 | AFR | Proteinuria | endocrine/metabolic |
| 270 | 0.01642 | 0.107294 | 0.153037 | 0.878369 | -0.19387 | 0.226712 | AFR | Disorders of prot | endocrine/metabolic |
| 270.1 | -0.31305 | 0.263689 | -1.18719 | 0.235152 | -0.82987 | 0.203772 | AFR | Disturbances of a | endocrine/metabolic |
| 270.11 | -0.54048 | 0.288721 | -1.872 | 0.061207 | -1.10637 | 0.025397 | AFR | Disturbances of s | endocrine/metabolic |
| 270.2 | 0.509476 | 0.409963 | 1.242735 | 0.213965 | -0.29404 | 1.312989 | AFR | Disorders of ami | endocrine/metabolic |
| 270.21 | 0.166457 | 0.534541 | 0.311401 | 0.755496 | -0.88122 | 1.214138 | AFR | Disorders of urea | endocrine/metabolic |
| 270.3 | 0.015969 | 0.116618 | 0.136931 | 0.891085 | -0.2126 | 0.244535 | AFR | Disorders of plas | endocrine/metabolic |
| 270.31 | 0.167507 | 0.384749 | 0.435367 | 0.663296 | -0.58659 | 0.921602 | AFR | Polyclonal hyper | endocrine/metabolic |
| 270.32 | 0.18214 | 0.154694 | 1.177423 | 0.239027 | -0.12105 | 0.485335 | AFR | Paraproteinemia | endocrine/metabolic |
| 270.33 | 0.077291 | 0.271562 | 0.284615 | 0.775939 | -0.45496 | 0.609541 | AFR | Amyloidosis | endocrine/metabolic |

| | | | | | | | | | |
|---|---|---|---|---|---|---|---|---|---|
| 270.34 | -1.47756 | 0.959954 | -1.5392 | 0.123755 | -3.35904 | 0.403913 | AFR | Alpha-1-antitryps | endocrine/metabolic |
| 270.38 | -0.00312 | 0.166488 | -0.01874 | 0.985045 | -0.32943 | 0.32319 | AFR | Other specified d | endocrine/metabolic |
| 271 | -0.04387 | 0.058553 | -0.7492 | 0.45374 | -0.15863 | 0.070894 | AFR | Disorders of carb | endocrine/metabolic |
| 271.3 | -0.04769 | 0.058718 | -0.8122 | 0.416678 | -0.16278 | 0.067395 | AFR | Intestinal disacch | endocrine/metabolic |
| 271.9 | 0.514247 | 0.51335 | 1.001748 | 0.316465 | -0.4919 | 1.520395 | AFR | Other disorders c | endocrine/metabolic |
| 272 | -0.01462 | 0.061104 | -0.23922 | 0.810936 | -0.13438 | 0.105145 | AFR | Disorders of lipoi | endocrine/metabolic |
| 272.1 | -0.0247 | 0.06117 | -0.40385 | 0.686321 | -0.14459 | 0.095187 | AFR | Hyperlipidemia | endocrine/metabolic |
| 272.11 | 0.012309 | 0.063912 | 0.192586 | 0.847283 | -0.11296 | 0.137574 | AFR | Hypercholesterol | endocrine/metabolic |
| 272.12 | 0.152392 | 0.170546 | 0.893557 | 0.371559 | -0.18187 | 0.486656 | AFR | Hyperglyceridem | endocrine/metabolic |
| 272.13 | -0.06959 | 0.071045 | -0.97951 | 0.327326 | -0.20883 | 0.069656 | AFR | Mixed hyperlipide | endocrine/metabolic |
| 272.14 | -0.42577 | 0.427742 | -0.99538 | 0.319552 | -1.26413 | 0.412594 | AFR | Hyperchylomicro | endocrine/metabolic |
| 272.9 | -0.15527 | 0.278102 | -0.55834 | 0.576615 | -0.70035 | 0.389796 | AFR | Unspecified disor | endocrine/metabolic |
| 274 | -0.07512 | 0.093005 | -0.80772 | 0.41925 | -0.25741 | 0.107165 | AFR | Gout and other c | endocrine/metabolic |
| 274.1 | -0.01964 | 0.097581 | -0.2013 | 0.840461 | -0.2109 | 0.171612 | AFR | Gout | endocrine/metabolic |
| 274.11 | -0.03245 | 0.113598 | -0.28564 | 0.77515 | -0.2551 | 0.1902 | AFR | Gouty arthropath | endocrine/metabolic |
| 274.2 | 0.162946 | 0.176997 | 0.920612 | 0.357253 | -0.18396 | 0.509854 | AFR | Crystal arthropat | endocrine/metabolic |
| 274.21 | 0.132556 | 0.179636 | 0.737914 | 0.460567 | -0.21952 | 0.484635 | AFR | Chondrocalcinos | endocrine/metabolic |
| 275 | 0.049663 | 0.062144 | 0.799168 | 0.424193 | -0.07214 | 0.171463 | AFR | Disorders of mine | endocrine/metabolic |
| 275.1 | -0.65606 | 0.263746 | -2.48748 | 0.012865 | -1.173 | -0.13913 | AFR | Disorders of iron | hematopoietic |
| 275.11 | -0.20434 | 0.965209 | -0.2117 | 0.832339 | -2.09611 | 1.687437 | AFR | Hereditary hemo | hematopoietic |
| 275.2 | 0.374381 | 0.645543 | 0.579947 | 0.56195 | -0.89086 | 1.639622 | AFR | Disorders of copp | endocrine/metabolic |
| 275.3 | 0.045868 | 0.092556 | 0.495569 | 0.620199 | -0.13554 | 0.227274 | AFR | Disorders of mag | endocrine/metabolic |
| 275.5 | -0.00232 | 0.078387 | -0.02964 | 0.97635 | -0.15596 | 0.151312 | AFR | Disorders of calc | endocrine/metabolic |
| 275.51 | -0.07039 | 0.115337 | -0.61029 | 0.541667 | -0.29645 | 0.155667 | AFR | Hypocalcemia | endocrine/metabolic |
| 275.53 | -0.08802 | 0.098494 | -0.89366 | 0.371506 | -0.28106 | 0.105025 | AFR | Disorders of phos | endocrine/metabolic |
| 275.6 | 0.030688 | 0.092729 | 0.330944 | 0.740687 | -0.15106 | 0.212433 | AFR | Hypercalcemia | endocrine/metabolic |
| 276 | 0.03953 | 0.055778 | 0.708697 | 0.478513 | -0.06979 | 0.148853 | AFR | Disorders of fluid | endocrine/metabolic |
| 276.1 | 0.054237 | 0.057976 | 0.935512 | 0.349525 | -0.05939 | 0.167868 | AFR | Electrolyte imbala | endocrine/metabolic |
| 276.11 | -0.05451 | 0.112152 | -0.48607 | 0.626918 | -0.27433 | 0.165301 | AFR | Hyperosmolality a | endocrine/metabolic |
| 276.12 | 0.094647 | 0.082845 | 1.142461 | 0.253263 | -0.06773 | 0.257019 | AFR | Hyposmolality an | endocrine/metabolic |
| 276.13 | -0.01918 | 0.079012 | -0.2427 | 0.808239 | -0.17404 | 0.135684 | AFR | Hyperpotassemia | endocrine/metabolic |
| 276.14 | 0.002669 | 0.072283 | 0.036919 | 0.97055 | -0.139 | 0.144341 | AFR | Hypopotassemia | endocrine/metabolic |
| 276.4 | -0.00167 | 0.075654 | -0.02201 | 0.98244 | -0.14994 | 0.146615 | AFR | Acid-base balanc | endocrine/metabolic |
| 276.41 | 0.053079 | 0.079324 | 0.66914 | 0.503406 | -0.10239 | 0.208551 | AFR | Acidosis | endocrine/metabolic |
| 276.42 | 0.103418 | 0.160383 | 0.644816 | 0.519046 | -0.21093 | 0.417763 | AFR | Alkalosis | endocrine/metabolic |
| 276.5 | 0.063289 | 0.072857 | 0.868669 | 0.385028 | -0.07951 | 0.206086 | AFR | Hypovolemia | endocrine/metabolic |
| 276.6 | 0.015529 | 0.091306 | 0.170078 | 0.864949 | -0.16343 | 0.194486 | AFR | Fluid overload | endocrine/metabolic |
| 276.8 | -0.42188 | 0.37249 | -1.13259 | 0.257388 | -1.15194 | 0.30819 | AFR | Polydipsia | endocrine/metabolic |

| | | | | | | | | | | |
|---|---|---|---|---|---|---|---|---|---|---|
| 277 | -0.06457 | 0.073233 | -0.88165 | 0.377966 | -0.2081 | 0.078968 | AFR | | Other disorders c | endocrine/metabolic |
| 277.1 | -0.45521 | 1.013792 | -0.44902 | 0.653416 | -2.44221 | 1.531782 | AFR | | Disorders of porp | endocrine/metabolic |
| 277.4 | -0.03195 | 0.176486 | -0.18104 | 0.85634 | -0.37786 | 0.313957 | AFR | | Disorders of biliru | endocrine/metabolic |
| 277.5 | 0.015089 | 0.19599 | 0.076987 | 0.938634 | -0.36904 | 0.399222 | AFR | | Other disorders c | endocrine/metabolic |
| 277.51 | 0.049475 | 0.243712 | 0.203007 | 0.83913 | -0.42819 | 0.527141 | AFR | | Lipoprotein disor | endocrine/metabolic |
| 277.6 | -0.34354 | 0.522888 | -0.657 | 0.51118 | -1.36838 | 0.681304 | AFR | | Other deficiencie | endocrine/metabolic |
| 277.7 | 0.269948 | 0.150756 | 1.790629 | 0.073353 | -0.02553 | 0.565425 | AFR | | Dysmetabolic syr | endocrine/metabolic |
| 278 | -0.01898 | 0.054279 | -0.34964 | 0.726612 | -0.12536 | 0.087407 | AFR | | Overweight, obes | endocrine/metabolic |
| 278.1 | 0.003434 | 0.055514 | 0.061857 | 0.950677 | -0.10537 | 0.11224 | AFR | | Obesity | endocrine/metabolic |
| 278.11 | 0.059165 | 0.063716 | 0.928566 | 0.353114 | -0.06572 | 0.184046 | AFR | | Morbid obesity | endocrine/metabolic |
| 278.3 | -0.19938 | 0.257295 | -0.77489 | 0.438404 | -0.70367 | 0.304913 | AFR | | Localized adipos | endocrine/metabolic |
| 278.4 | -0.02078 | 0.095736 | -0.21702 | 0.828191 | -0.20842 | 0.166862 | AFR | | Abnormal weight | endocrine/metabolic |
| 279 | -0.02892 | 0.078101 | -0.37028 | 0.711177 | -0.18199 | 0.124157 | AFR | | Disorders involvi | endocrine/metabolic |
| 279.1 | -0.02183 | 0.080699 | -0.27048 | 0.786788 | -0.18 | 0.13634 | AFR | | Immunity deficier | endocrine/metabolic |
| 279.11 | -0.0771 | 0.283563 | -0.27188 | 0.785712 | -0.63287 | 0.478678 | AFR | | Deficiency of hur | endocrine/metabolic |
| 279.2 | -0.21809 | 0.412904 | -0.52819 | 0.597368 | -1.02737 | 0.591186 | AFR | | Autoimmune dise | endocrine/metabolic |
| 279.7 | 0.03327 | 0.083442 | 0.398715 | 0.690103 | -0.13027 | 0.196814 | AFR | | Other immunolog | endocrine/metabolic |
| 279.8 | -0.11089 | 0.266109 | -0.41671 | 0.676893 | -0.63245 | 0.410675 | AFR | | Other specified d | endocrine/metabolic |
| 280 | 0.058353 | 0.06151 | 0.948677 | 0.342785 | -0.0622 | 0.178911 | AFR | | Iron deficiency ar | hematopoietic |
| 280.1 | 0.067796 | 0.064073 | 1.058111 | 0.290005 | -0.05778 | 0.193377 | AFR | | Iron deficiency ar | hematopoietic |
| 280.2 | 0.069676 | 0.086791 | 0.802794 | 0.422094 | -0.10043 | 0.239783 | AFR | | Iron deficiency ar | hematopoietic |
| 281 | 0.130366 | 0.118609 | 1.099127 | 0.271713 | -0.1021 | 0.362836 | AFR | | Other deficiency | hematopoietic |
| 281.1 | -0.19033 | 0.180488 | -1.05452 | 0.291644 | -0.54408 | 0.163422 | AFR | | Megaloblastic an | hematopoietic |
| 281.11 | -0.6169 | 0.270367 | -2.28172 | 0.022506 | -1.14681 | -0.08699 | AFR | | Pernicious anem | hematopoietic |
| 281.12 | -0.17756 | 0.351975 | -0.50447 | 0.613931 | -0.86742 | 0.512298 | AFR | | Other vitamin B1 | hematopoietic |
| 281.13 | 0.029081 | 0.254145 | 0.114426 | 0.9089 | -0.46903 | 0.527197 | AFR | | Folate-deficiency | hematopoietic |
| 281.9 | 0.339189 | 0.152913 | 2.218185 | 0.026542 | 0.039485 | 0.638893 | AFR | | Deficiency anemi | hematopoietic |
| 282 | 0.170511 | 0.101409 | 1.681416 | 0.092682 | -0.02825 | 0.36927 | AFR | | Hereditary hemol | hematopoietic |
| 282.5 | 0.15538 | 0.155087 | 1.001891 | 0.316396 | -0.14858 | 0.459345 | AFR | | Sickle cell anemi | hematopoietic |
| 282.8 | 0.227075 | 0.15842 | 1.433377 | 0.15175 | -0.08342 | 0.537573 | AFR | | Other hemoglobi | hematopoietic |
| 282.9 | 0.008042 | 0.164427 | 0.048908 | 0.960993 | -0.31423 | 0.330314 | AFR | | Other hereditary | hematopoietic |
| 283 | -0.21449 | 0.234246 | -0.91566 | 0.359848 | -0.6736 | 0.244625 | AFR | | Acquired hemoly | hematopoietic |
| 283.1 | -0.80606 | 0.338336 | -2.38243 | 0.017199 | -1.46919 | -0.14293 | AFR | | Autoimmune hen | hematopoietic |
| 283.2 | -0.44053 | 0.465006 | -0.94737 | 0.34345 | -1.35193 | 0.470861 | AFR | | Non-autoimmune | hematopoietic |
| 283.21 | -0.22548 | 1.155875 | -0.19508 | 0.845333 | -2.49096 | 2.039989 | AFR | | Hemolytic-uremic | hematopoietic |
| 284 | -0.04681 | 0.112777 | -0.41507 | 0.678088 | -0.26785 | 0.174228 | AFR | | Aplastic anemia | hematopoietic |
| 284.1 | -0.07779 | 0.118569 | -0.65605 | 0.511794 | -0.31018 | 0.154605 | AFR | | Pancytopenia | hematopoietic |
| 285 | -0.08823 | 0.054391 | -1.62205 | 0.104792 | -0.19483 | 0.018379 | AFR | | Other anemias | hematopoietic |

| | | | | | | | | | | |
|---|---|---|---|---|---|---|---|---|---|---|
| 285.1 | -0.00561 | 0.068402 | -0.08199 | 0.934651 | -0.13967 | 0.128456 | AFR | Acute posthemor | hematopoietic | |
| 285.2 | 0.017794 | 0.069295 | 0.256784 | 0.797346 | -0.11802 | 0.15361 | AFR | Anemia of chroni | hematopoietic | |
| 285.21 | -0.04943 | 0.081855 | -0.60388 | 0.545926 | -0.20986 | 0.111003 | AFR | Anemia in chroni | hematopoietic | |
| 285.22 | -0.03723 | 0.167305 | -0.22254 | 0.823894 | -0.36514 | 0.290681 | AFR | Anemia in neopla | hematopoietic | |
| 285.8 | -0.21981 | 0.309344 | -0.71058 | 0.477347 | -0.82611 | 0.38649 | AFR | Hemoglobinuria | hematopoietic | |
| 286 | -0.04452 | 0.077206 | -0.5766 | 0.564208 | -0.19584 | 0.106804 | AFR | Coagulation defe | hematopoietic | |
| 286.1 | -0.07432 | 0.346686 | -0.21437 | 0.830256 | -0.75381 | 0.605172 | AFR | Congenital coagu | hematopoietic | |
| 286.11 | -0.5203 | 0.96168 | -0.54103 | 0.588488 | -2.40516 | 1.364562 | AFR | Von willebrand's | hematopoietic | |
| 286.12 | 0.148429 | 0.500944 | 0.296299 | 0.767002 | -0.8334 | 1.130261 | AFR | Congenital defici | hematopoietic | |
| 286.13 | 0.324953 | 0.571133 | 0.568962 | 0.569382 | -0.79445 | 1.444353 | AFR | Congenital factor | hematopoietic | |
| 286.2 | -0.05912 | 0.076065 | -0.7772 | 0.437041 | -0.2082 | 0.089968 | AFR | Encounter for lon | hematopoietic | |
| 286.3 | -0.35826 | 0.377492 | -0.94905 | 0.342596 | -1.09813 | 0.381612 | AFR | Coagulation defe | hematopoietic | |
| 286.4 | 0.038926 | 0.1821 | 0.213761 | 0.830733 | -0.31798 | 0.395835 | AFR | Acquired coagula | hematopoietic | |
| 286.5 | 0.262967 | 0.434724 | 0.604906 | 0.545241 | -0.58908 | 1.11501 | AFR | Hemorrhagic diso | hematopoietic | |
| 286.6 | -0.18137 | 0.279054 | -0.64995 | 0.515727 | -0.72831 | 0.365566 | AFR | Defibrination syn | hematopoietic | |
| 286.7 | 0.013983 | 0.123834 | 0.112917 | 0.910097 | -0.22873 | 0.256693 | AFR | Other and unspe | hematopoietic | |
| 286.8 | 0.004286 | 0.143399 | 0.029886 | 0.976158 | -0.27677 | 0.285343 | AFR | Hypercoagulable | hematopoietic | |
| 286.81 | -0.00631 | 0.152858 | -0.04129 | 0.967065 | -0.30591 | 0.293285 | AFR | Primary hypercoa | hematopoietic | |
| 286.9 | -0.09854 | 0.10611 | -0.92865 | 0.35307 | -0.30651 | 0.109433 | AFR | Abnormal coagul | hematopoietic | |
| 287 | -0.00363 | 0.082209 | -0.04417 | 0.964769 | -0.16476 | 0.157495 | AFR | Purpura and othe | hematopoietic | |
| 287.1 | -0.85101 | 0.28106 | -3.02786 | 0.002463 | -1.40188 | -0.30014 | AFR | Spontaneous ecc | hematopoietic | |
| 287.3 | -0.00814 | 0.083987 | -0.09697 | 0.922747 | -0.17276 | 0.156467 | AFR | Thrombocytopen | hematopoietic | |
| 287.31 | -0.02745 | 0.364468 | -0.07532 | 0.93996 | -0.7418 | 0.686892 | AFR | Primary thrombo | hematopoietic | |
| 287.32 | -0.15811 | 0.154056 | -1.02629 | 0.304755 | -0.46005 | 0.143838 | AFR | Secondary throm | hematopoietic | |
| 287.4 | 0.322815 | 0.393988 | 0.819352 | 0.412586 | -0.44939 | 1.095017 | AFR | Qualitative platel | hematopoietic | |
| 288 | 0.029751 | 0.066984 | 0.444154 | 0.656931 | -0.10154 | 0.161038 | AFR | Diseases of white | hematopoietic | |
| 288.1 | 0.030417 | 0.068519 | 0.443922 | 0.657099 | -0.10388 | 0.164713 | AFR | Decreased white | hematopoietic | |
| 288.11 | -0.1118 | 0.088895 | -1.25762 | 0.20853 | -0.28603 | 0.062435 | AFR | Neutropenia | hematopoietic | |
| 288.2 | -0.00342 | 0.070218 | -0.0487 | 0.961156 | -0.14104 | 0.134205 | AFR | Elevated white bl | hematopoietic | |
| 288.3 | 0.185515 | 0.210922 | 0.879546 | 0.379105 | -0.22788 | 0.598914 | AFR | Eosinophilia | hematopoietic | |
| 289 | -0.07302 | 0.074513 | -0.97996 | 0.327108 | -0.21906 | 0.073023 | AFR | Other diseases o | hematopoietic | |
| 289.1 | -0.21358 | 0.657902 | -0.32464 | 0.745452 | -1.50305 | 1.075881 | AFR | Myelofibrosis | hematopoietic | |
| 289.3 | -0.13975 | 0.101682 | -1.37442 | 0.16931 | -0.33905 | 0.059539 | AFR | Personal history | hematopoietic | |
| 289.4 | 0.047476 | 0.077574 | 0.612008 | 0.540533 | -0.10457 | 0.199518 | AFR | Lymphadenitis | hematopoietic | |
| 289.5 | 0.190855 | 0.235332 | 0.811001 | 0.417365 | -0.27039 | 0.652097 | AFR | Diseases of splee | hematopoietic | |
| 289.8 | 0.267122 | 0.223462 | 1.195378 | 0.231939 | -0.17086 | 0.7051 | AFR | Polycythemia, se | hematopoietic | |
| 289.9 | -0.01959 | 0.140678 | -0.13927 | 0.889239 | -0.29532 | 0.256133 | AFR | Abnormality of re | hematopoietic | |
| 290 | 0.048742 | 0.11493 | 0.424099 | 0.671493 | -0.17652 | 0.274001 | AFR | Delirium dementi | mental disorders | |

| | | | | | | | | | | |
|---|---|---|---|---|---|---|---|---|---|---|
| 290.1 | 0.201271 | 0.14489 | 1.389127 | 0.164794 | -0.08271 | 0.485251 | AFR | Dementias | mental disorders | |
| 290.11 | 0.040904 | 0.229384 | 0.17832 | 0.858472 | -0.40868 | 0.490489 | AFR | Alzheimer's disea | mental disorders | |
| 290.12 | -0.0183 | 0.475163 | -0.03851 | 0.969281 | -0.9496 | 0.913004 | AFR | Dementia with ce | mental disorders | |
| 290.13 | 0.062829 | 0.357562 | 0.175714 | 0.860519 | -0.63798 | 0.763637 | AFR | Senile dementia | mental disorders | |
| 290.16 | 0.03249 | 0.215788 | 0.150566 | 0.880318 | -0.39045 | 0.455427 | AFR | Vascular dement | mental disorders | |
| 290.2 | -0.01727 | 0.160771 | -0.10741 | 0.914462 | -0.33237 | 0.297836 | AFR | Delirium due to c | mental disorders | |
| 290.3 | -0.05492 | 0.154599 | -0.35527 | 0.722387 | -0.35793 | 0.248084 | AFR | Other persistent | mental disorders | |
| 291 | -0.09964 | 0.093939 | -1.06074 | 0.288809 | -0.28376 | 0.084472 | AFR | Other specified n | mental disorders | |
| 291.1 | 1.84168 | 1.461889 | 1.259795 | 0.207743 | -1.02357 | 4.70693 | AFR | Transient mental | mental disorders | |
| 291.4 | -0.24156 | 0.190019 | -1.27126 | 0.203635 | -0.614 | 0.130866 | AFR | Specific nonpsyc | mental disorders | |
| 291.8 | -0.0864 | 0.102269 | -0.84479 | 0.398227 | -0.28684 | 0.114048 | AFR | Alteration of cons | mental disorders | |
| 292 | 0.101027 | 0.066771 | 1.513034 | 0.130271 | -0.02984 | 0.231897 | AFR | Neurological disc | mental disorders | |
| 292.1 | 0.056527 | 0.115306 | 0.490233 | 0.623969 | -0.16947 | 0.282522 | AFR | Aphasia/speech | mental disorders | |
| 292.11 | 0.030415 | 0.175751 | 0.173059 | 0.862605 | -0.31405 | 0.374881 | AFR | Aphasia | mental disorders | |
| 292.12 | -0.55677 | 0.333477 | -1.66959 | 0.095 | -1.21037 | 0.096833 | AFR | Symbolic dysfunc | mental disorders | |
| 292.2 | 0.176556 | 0.199876 | 0.883328 | 0.377059 | -0.21519 | 0.568307 | AFR | Mild cognitive im | mental disorders | |
| 292.3 | 0.097218 | 0.096101 | 1.01162 | 0.31172 | -0.09114 | 0.285573 | AFR | Memory loss | mental disorders | |
| 292.4 | 0.070834 | 0.088691 | 0.79866 | 0.424487 | -0.103 | 0.244664 | AFR | Altered mental st | mental disorders | |
| 292.5 | 0.0016 | 0.203505 | 0.007862 | 0.993727 | -0.39726 | 0.400463 | AFR | Transient alterati | mental disorders | |
| 292.6 | 0.497589 | 0.250579 | 1.985755 | 0.047061 | 0.006463 | 0.988714 | AFR | Hallucinations | mental disorders | |
| 293 | 0.008804 | 0.13446 | 0.065479 | 0.947792 | -0.25473 | 0.272342 | AFR | Symptoms involv | mental disorders | |
| 293.1 | 0.004583 | 0.08506 | 0.053881 | 0.95703 | -0.16213 | 0.171298 | AFR | Swelling, mass, | mental disorders | |
| 295 | 0.097939 | 0.143406 | 0.682953 | 0.494637 | -0.18313 | 0.379009 | AFR | Schizophrenia ar | mental disorders | |
| 295.1 | 0.05503 | 0.211787 | 0.259838 | 0.794989 | -0.36007 | 0.470126 | AFR | Schizophrenia | mental disorders | |
| 295.2 | 0.141277 | 0.230986 | 0.611628 | 0.540784 | -0.31145 | 0.594001 | AFR | Paranoid disorde | mental disorders | |
| 295.3 | 0.177229 | 0.181614 | 0.975855 | 0.329136 | -0.17873 | 0.533187 | AFR | Psychosis | mental disorders | |
| 296 | 0.048414 | 0.058627 | 0.825792 | 0.408922 | -0.06649 | 0.163321 | AFR | Mood disorders | mental disorders | |
| 296.1 | -0.14679 | 0.123006 | -1.19333 | 0.23274 | -0.38787 | 0.0943 | AFR | Bipolar | mental disorders | |
| 296.2 | 0.06578 | 0.05977 | 1.100544 | 0.271095 | -0.05137 | 0.182927 | AFR | Depression | mental disorders | |
| 296.22 | 0.088462 | 0.062232 | 1.421483 | 0.155176 | -0.03351 | 0.210435 | AFR | Major depressive | mental disorders | |
| 297 | 0.010877 | 0.207445 | 0.052433 | 0.958184 | -0.39571 | 0.417462 | AFR | Suicidal ideation | mental disorders | |
| 297.1 | 0.053595 | 0.216878 | 0.247123 | 0.804813 | -0.37148 | 0.478669 | AFR | Suicidal ideation | mental disorders | |
| 297.2 | -0.61495 | 0.456979 | -1.3457 | 0.178401 | -1.51062 | 0.280708 | AFR | Suicide or self-in | mental disorders | |
| 300 | 0.099195 | 0.055998 | 1.771381 | 0.076497 | -0.01056 | 0.208949 | AFR | Anxiety disorders | mental disorders | |
| 300.1 | 0.086609 | 0.056903 | 1.522055 | 0.127995 | -0.02492 | 0.198136 | AFR | Anxiety disorder | mental disorders | |
| 300.11 | -0.02725 | 0.086845 | -0.31376 | 0.753703 | -0.19746 | 0.142965 | AFR | Generalized anxi | mental disorders | |
| 300.12 | 0.114876 | 0.145983 | 0.786915 | 0.431332 | -0.17125 | 0.400997 | AFR | Agorophobia, soc | mental disorders | |
| 300.13 | 0.027768 | 0.212411 | 0.130725 | 0.895993 | -0.38855 | 0.444086 | AFR | Phobia | mental disorders | |

| | | | | | | | | | | |
|---|---|---|---|---|---|---|---|---|---|---|
| 300.3 | -0.06283 | 0.696809 | -0.09017 | 0.928152 | -1.42855 | 1.30289 | AFR | Obsessive-comp | mental disorders | |
| 300.4 | 0.177828 | 0.126923 | 1.401065 | 0.161195 | -0.07094 | 0.426593 | AFR | Dysthymic disord | mental disorders | |
| 300.8 | -0.04664 | 0.223631 | -0.20854 | 0.834806 | -0.48495 | 0.391673 | AFR | Acute reaction to | mental disorders | |
| 300.9 | -0.05905 | 0.145546 | -0.40572 | 0.684952 | -0.34432 | 0.226215 | AFR | Posttraumatic str | mental disorders | |
| 301 | -0.23451 | 0.295116 | -0.79464 | 0.426822 | -0.81293 | 0.343906 | AFR | Personality disor | mental disorders | |
| 301.1 | -0.33446 | 0.996895 | -0.3355 | 0.73725 | -2.28833 | 1.619423 | AFR | Schizoid persona | mental disorders | |
| 301.2 | -0.46516 | 0.316694 | -1.46881 | 0.141884 | -1.08587 | 0.155545 | AFR | Antisocial/border | mental disorders | |
| 302 | 0.275871 | 0.236137 | 1.168268 | 0.242699 | -0.18695 | 0.73869 | AFR | Sexual and gend | mental disorders | |
| 302.1 | 0.124053 | 0.25315 | 0.490037 | 0.624107 | -0.37211 | 0.620218 | AFR | Decreased libido | mental disorders | |
| 303 | 0.187696 | 0.167337 | 1.121669 | 0.262003 | -0.14028 | 0.51567 | AFR | Psychogenic and | mental disorders | |
| 303.1 | 0.536765 | 0.737637 | 0.727681 | 0.466809 | -0.90898 | 1.982507 | AFR | Dissociative diso | mental disorders | |
| 303.3 | 0.132798 | 0.206187 | 0.644068 | 0.519532 | -0.27132 | 0.536918 | AFR | Psychogenic diso | mental disorders | |
| 303.31 | 0.155513 | 0.247195 | 0.62911 | 0.529277 | -0.32898 | 0.640007 | AFR | Gastrointestinal r | mental disorders | |
| 303.4 | -0.00136 | 0.26505 | -0.00512 | 0.995911 | -0.52085 | 0.518131 | AFR | Somatoform diso | mental disorders | |
| 304 | -0.04669 | 0.080365 | -0.58103 | 0.561223 | -0.20421 | 0.110818 | AFR | Adjustment react | mental disorders | |
| 305.2 | -0.07261 | 0.300573 | -0.24156 | 0.809123 | -0.66172 | 0.516507 | AFR | Eating disorder | mental disorders | |
| 305.21 | -0.23858 | 0.59096 | -0.40371 | 0.686422 | -1.39684 | 0.919681 | AFR | Anorexia nervosa | mental disorders | |
| 306 | 0.03107 | 0.054765 | 0.567324 | 0.570494 | -0.07627 | 0.138408 | AFR | Other mental diso | mental disorders | |
| 306.1 | 0.211717 | 0.267637 | 0.791059 | 0.428909 | -0.31284 | 0.736275 | AFR | Mental disorders | mental disorders | |
| 306.9 | -0.1422 | 0.133943 | -1.06167 | 0.288386 | -0.40473 | 0.12032 | AFR | Tension headach | mental disorders | |
| 312 | -0.27229 | 0.283846 | -0.95927 | 0.337421 | -0.82861 | 0.284042 | AFR | Conduct disorder | mental disorders | |
| 313 | 0.095588 | 0.183697 | 0.520355 | 0.602816 | -0.26445 | 0.455628 | AFR | Pervasive develo | mental disorders | |
| 313.1 | -0.0028 | 0.207549 | -0.01349 | 0.98924 | -0.40959 | 0.40399 | AFR | Attention deficit h | mental disorders | |
| 313.2 | 0.160743 | 0.593656 | 0.270768 | 0.786569 | -1.0028 | 1.324288 | AFR | Tics and stutterin | mental disorders | |
| 313.3 | 0.805246 | 0.668148 | 1.205192 | 0.228129 | -0.5043 | 2.114792 | AFR | Autism | mental disorders | |
| 315 | 0.116471 | 0.155875 | 0.747204 | 0.45494 | -0.18904 | 0.421981 | AFR | Develomental de | mental disorders | |
| 315.1 | 1.282981 | 0.677081 | 1.894869 | 0.05811 | -0.04407 | 2.610036 | AFR | Learning disorde | mental disorders | |
| 315.2 | 0.050004 | 0.292037 | 0.171226 | 0.864046 | -0.52238 | 0.622387 | AFR | Speech and lang | mental disorders | |
| 315.3 | 0.529992 | 0.530447 | 0.999142 | 0.317726 | -0.50967 | 1.56965 | AFR | Mental retardatio | mental disorders | |
| 316 | 0.10686 | 0.082443 | 1.296165 | 0.194919 | -0.05473 | 0.268445 | AFR | Substance addic | mental disorders | |
| 316.1 | -0.03774 | 0.205939 | -0.18325 | 0.854601 | -0.44137 | 0.365895 | AFR | Polyneuropathy d | mental disorders | |
| 317 | -0.0124 | 0.1028 | -0.12064 | 0.903976 | -0.21389 | 0.189083 | AFR | Alcohol-related d | mental disorders | |
| 317.1 | -0.00869 | 0.114525 | -0.07587 | 0.939526 | -0.23315 | 0.215776 | AFR | Alcoholism | mental disorders | |
| 317.11 | 0.234445 | 0.332585 | 0.704918 | 0.480861 | -0.41741 | 0.886299 | AFR | Alcoholic liver da | mental disorders | |
| 318 | 0.004973 | 0.059434 | 0.083673 | 0.933316 | -0.11151 | 0.121461 | AFR | Tobacco use diso | mental disorders | |
| 320 | 0.272692 | 0.269543 | 1.011681 | 0.31169 | -0.2556 | 0.800987 | AFR | Meningitis | neurological | |
| 323 | 0.105739 | 0.276268 | 0.38274 | 0.701912 | -0.43574 | 0.647213 | AFR | Encephalitis | neurological | |
| 323.2 | 0.382964 | 0.685151 | 0.558948 | 0.576197 | -0.95991 | 1.725835 | AFR | Acute (transverse | neurological | |

| | | | | | | | | | | |
|---|---|---|---|---|---|---|---|---|---|---|
| 323.8 | 0.096327 | 0.288629 | 0.333739 | 0.738577 | -0.46938 | 0.66203 | AFR | Encephalitis, non | neurological | |
| 324 | 0.143248 | 0.312908 | 0.457796 | 0.647099 | -0.47004 | 0.756535 | AFR | Other CNS infect | neurological | |
| 325 | 0.594807 | 0.597829 | 0.994946 | 0.319763 | -0.57692 | 1.76653 | AFR | Phlebitis and thro | neurological | |
| 327 | -0.0307 | 0.059128 | -0.51925 | 0.603586 | -0.14659 | 0.085186 | AFR | Sleep disorders | neurological | |
| 327.1 | 0.009755 | 0.100227 | 0.097331 | 0.922464 | -0.18669 | 0.206197 | AFR | Hypersomnia | neurological | |
| 327.3 | 0.00048 | 0.062357 | 0.0077 | 0.993856 | -0.12174 | 0.122697 | AFR | Sleep apnea | neurological | |
| 327.31 | -0.25899 | 0.193505 | -1.33841 | 0.180764 | -0.63825 | 0.120274 | AFR | Central/nonobstru | neurological | |
| 327.32 | -0.03882 | 0.066827 | -0.58098 | 0.561257 | -0.1698 | 0.092153 | AFR | Obstructive sleep | neurological | |
| 327.4 | -0.02543 | 0.066443 | -0.38271 | 0.701932 | -0.15565 | 0.104797 | AFR | Insomnia | neurological | |
| 327.41 | -0.01427 | 0.108423 | -0.13166 | 0.895254 | -0.22678 | 0.19823 | AFR | Organic or persis | neurological | |
| 327.5 | -0.249 | 0.227635 | -1.09384 | 0.274024 | -0.69515 | 0.19716 | AFR | Parasomnia | neurological | |
| 327.6 | -0.32651 | 0.237802 | -1.37305 | 0.169735 | -0.7926 | 0.139568 | AFR | Circadian rhythm | neurological | |
| 327.7 | 0.159995 | 0.141066 | 1.134179 | 0.25672 | -0.11649 | 0.43648 | AFR | Sleep related mo | neurological | |
| 327.71 | 0.145365 | 0.197947 | 0.734363 | 0.462728 | -0.2426 | 0.533334 | AFR | Restless legs syr | neurological | |
| 327.72 | 0.338334 | 0.310419 | 1.089925 | 0.275746 | -0.27008 | 0.946745 | AFR | Sleep related leg | neurological | |
| 331 | 0.025996 | 0.16122 | 0.161245 | 0.8719 | -0.28999 | 0.341981 | AFR | Other cerebral de | neurological | |
| 331.1 | -0.09918 | 0.302037 | -0.32838 | 0.742622 | -0.69117 | 0.492798 | AFR | Hydrocephalus | neurological | |
| 331.9 | 0.040344 | 0.224712 | 0.179536 | 0.857517 | -0.40008 | 0.480771 | AFR | Cerebral degene | neurological | |
| 332 | 0.015787 | 0.332607 | 0.047465 | 0.962143 | -0.63611 | 0.667684 | AFR | Parkinson's disea | neurological | |
| 333 | 0.016451 | 0.128162 | 0.128363 | 0.897862 | -0.23474 | 0.267645 | AFR | Extrapyramidal d | neurological | |
| 333.1 | -0.04918 | 0.189845 | -0.25907 | 0.795579 | -0.42127 | 0.322906 | AFR | Essential tremor | neurological | |
| 333.2 | -0.09595 | 0.230338 | -0.41656 | 0.676998 | -0.5474 | 0.355503 | AFR | Myoclonus | neurological | |
| 333.3 | 0.22793 | 0.749147 | 0.304253 | 0.760935 | -1.24037 | 1.696231 | AFR | Tics and choreas | neurological | |
| 333.4 | 0.428911 | 0.314524 | 1.363679 | 0.172669 | -0.18755 | 1.045367 | AFR | Torsion dystonia | neurological | |
| 333.8 | -0.23305 | 0.454554 | -0.5127 | 0.608164 | -1.12396 | 0.657862 | AFR | Other degenerati | neurological | |
| 334 | -0.17491 | 0.149177 | -1.17253 | 0.240984 | -0.4673 | 0.117467 | AFR | Degenerative dis | neurological | |
| 334.1 | -0.55231 | 0.643533 | -0.85825 | 0.390753 | -1.81361 | 0.708988 | AFR | Spinocerebellar ( | neurological | |
| 334.2 | -0.45594 | 0.528724 | -0.86234 | 0.3885 | -1.49222 | 0.580339 | AFR | Anterior horn cell | neurological | |
| 334.21 | -1.52321 | 1.068736 | -1.42524 | 0.154087 | -3.61789 | 0.571477 | AFR | Amyotrophic Late | neurological | |
| 335 | 0.108184 | 0.223307 | 0.48446 | 0.628059 | -0.32949 | 0.545858 | AFR | Multiple sclerosis | neurological | |
| 337 | 0.140115 | 0.169683 | 0.825744 | 0.408949 | -0.19246 | 0.472688 | AFR | Disorders of the a | neurological | |
| 337.1 | 0.207665 | 0.200692 | 1.034746 | 0.300788 | -0.18568 | 0.601014 | AFR | Peripheral auton | neurological | |
| 338 | 0.12266 | 0.0559 | 2.19428 | 0.028215 | 0.013098 | 0.232221 | AFR | Pain | neurological | |
| 338.1 | 0.077666 | 0.055204 | 1.406903 | 0.159456 | -0.03053 | 0.185864 | AFR | Acute pain | neurological | |
| 338.2 | 0.067256 | 0.055372 | 1.21462 | 0.224511 | -0.04127 | 0.175782 | AFR | Chronic pain | neurological | |
| 339 | 0.013436 | 0.059714 | 0.225 | 0.821979 | -0.1036 | 0.130474 | AFR | Other headache | neurological | |
| 340 | -0.0703 | 0.078755 | -0.89267 | 0.372036 | -0.22466 | 0.084055 | AFR | Migraine | neurological | |
| 340.1 | -0.14786 | 0.150253 | -0.98409 | 0.325073 | -0.44235 | 0.146628 | AFR | Migrain with aura | neurological | |

| | | | | | | | | | | | |
|---|---|---|---|---|---|---|---|---|---|---|---|
| 341 | -0.16022 | 0.353892 | -0.45273 | 0.650741 | -0.85383 | 0.533397 | AFR | | Other demyelinat | neurological | |
| 342 | -0.17132 | 0.191027 | -0.89686 | 0.369795 | -0.54573 | 0.203083 | AFR | | Hemiplegia | neurological | |
| 343 | 0.403558 | 0.396459 | 1.017906 | 0.308723 | -0.37349 | 1.180604 | AFR | | Infantile cerebral | neurological | |
| 344 | 0.288691 | 0.188708 | 1.529829 | 0.126059 | -0.08117 | 0.658552 | AFR | | Other paralytic sy | neurological | |
| 345 | -0.01657 | 0.101263 | -0.16367 | 0.869993 | -0.21505 | 0.181899 | AFR | | Epilepsy, recurre | neurological | |
| 345.1 | 0.115291 | 0.154547 | 0.745996 | 0.45567 | -0.18761 | 0.418197 | AFR | | Epilepsy | neurological | |
| 345.11 | 0.192777 | 0.259438 | 0.743056 | 0.457448 | -0.31571 | 0.701266 | AFR | | Generalized conv | neurological | |
| 345.12 | 0.215056 | 0.245377 | 0.876432 | 0.380795 | -0.26587 | 0.695985 | AFR | | Partial epilepsy | neurological | |
| 345.3 | -0.02573 | 0.108145 | -0.23788 | 0.811976 | -0.23769 | 0.186236 | AFR | | Convulsions | neurological | |
| 346 | -0.06671 | 0.321247 | -0.20765 | 0.835501 | -0.69634 | 0.562924 | AFR | | Abnormal finding | neurological | |
| 346.1 | 0.017723 | 0.131345 | 0.134936 | 0.892663 | -0.23971 | 0.275154 | AFR | | Nonspecific abnc | neurological | |
| 346.2 | -0.22554 | 0.334895 | -0.67345 | 0.50066 | -0.88192 | 0.430846 | AFR | | Nonspecific abnc | neurological | |
| 346.3 | 0.000565 | 0.270569 | 0.002087 | 0.998335 | -0.52974 | 0.530869 | AFR | | Nonspecific abnc | neurological | |
| 347 | 0.255305 | 0.519173 | 0.491754 | 0.622893 | -0.76226 | 1.272867 | AFR | | Cataplexy and na | neurological | |
| 348 | -0.03306 | 0.095948 | -0.34455 | 0.730431 | -0.22111 | 0.154995 | AFR | | Other conditions | neurological | |
| 348.2 | -0.09827 | 0.176024 | -0.55826 | 0.576668 | -0.44327 | 0.246734 | AFR | | Cerebral edema | neurological | |
| 348.4 | -0.25428 | 0.530379 | -0.47943 | 0.631632 | -1.2938 | 0.785243 | AFR | | Cerebral cysts | neurological | |
| 348.7 | 0.115268 | 0.166241 | 0.69338 | 0.488071 | -0.21056 | 0.441096 | AFR | | Coma | neurological | |
| 348.8 | -0.04521 | 0.107097 | -0.42218 | 0.672894 | -0.25512 | 0.164692 | AFR | | Encephalopathy, | neurological | |
| 348.9 | -0.14668 | 0.11801 | -1.24297 | 0.213879 | -0.37798 | 0.084612 | AFR | | Other conditions | neurological | |
| 349 | -0.0424 | 0.105482 | -0.40198 | 0.687702 | -0.24914 | 0.16434 | AFR | | Other and unspe | neurological | |
| 350 | -0.06023 | 0.065369 | -0.92137 | 0.356857 | -0.18835 | 0.067892 | AFR | | Abnormal moven | neurological | |
| 350.1 | -0.05738 | 0.080601 | -0.71191 | 0.476518 | -0.21536 | 0.100594 | AFR | | Abnormal involur | neurological | |
| 350.2 | -0.07687 | 0.082509 | -0.93171 | 0.351486 | -0.23859 | 0.08484 | AFR | | Abnormality of ga | neurological | |
| 350.3 | 0.011553 | 0.202837 | 0.056957 | 0.95458 | -0.386 | 0.409106 | AFR | | Lack of coordinat | neurological | |
| 350.5 | -0.02737 | 0.361378 | -0.07575 | 0.93962 | -0.73566 | 0.680915 | AFR | | Abnormal reflex | neurological | |
| 350.6 | 0.101956 | 0.157051 | 0.649187 | 0.516218 | -0.20586 | 0.40977 | AFR | | Disturbances of s | neurological | |
| 351 | -0.04208 | 0.062989 | -0.66809 | 0.504079 | -0.16554 | 0.081374 | AFR | | Other peripheral | neurological | |
| 352 | 0.029635 | 0.168742 | 0.175625 | 0.860589 | -0.30109 | 0.360364 | AFR | | Disorders of othe | neurological | |
| 352.1 | 0.263058 | 0.290017 | 0.907043 | 0.364384 | -0.30537 | 0.831481 | AFR | | Trigeminal nerve | neurological | |
| 352.2 | -0.03976 | 0.205597 | -0.19337 | 0.846667 | -0.44272 | 0.363205 | AFR | | Facial nerve diso | neurological | |
| 353 | 0.187193 | 0.185373 | 1.009814 | 0.312584 | -0.17613 | 0.550518 | AFR | | Nerve root and p | neurological | |
| 353.1 | 0.286942 | 0.227339 | 1.262177 | 0.206885 | -0.15863 | 0.732519 | AFR | | Nerve plexus lesi | neurological | |
| 353.2 | 0.160443 | 0.434487 | 0.369269 | 0.711927 | -0.69114 | 1.012022 | AFR | | Nerve root lesion | neurological | |
| 355 | 0.099777 | 0.203636 | 0.489978 | 0.62415 | -0.29934 | 0.498897 | AFR | | Complex regiona | neurological | |
| 355.1 | -0.049 | 0.135094 | -0.36269 | 0.716835 | -0.31378 | 0.215782 | AFR | | Chronic pain syn | neurological | |
| 356 | -0.01729 | 0.103444 | -0.1671 | 0.867293 | -0.22003 | 0.185461 | AFR | | Hereditary and id | neurological | |
| 357 | 0.091247 | 0.068379 | 1.334438 | 0.18206 | -0.04277 | 0.225267 | AFR | | Inflammatory and | neurological | |

| | | | | | | | | | | |
|---|---|---|---|---|---|---|---|---|---|---|
| 358 | -0.25123 | 0.193809 | -1.29628 | 0.19488 | -0.63109 | 0.128628 | AFR | Myoneural disord | neurological | |
| 358.1 | -0.3686 | 0.319833 | -1.15246 | 0.249132 | -0.99546 | 0.258266 | AFR | Myasthenia gravi | neurological | |
| 359 | -0.1064 | 0.133251 | -0.79852 | 0.424572 | -0.36757 | 0.154764 | AFR | Muscular dystrop | neurological | |
| 359.1 | -0.56812 | 0.382725 | -1.48441 | 0.137701 | -1.31825 | 0.182008 | AFR | Muscular dystrop | neurological | |
| 359.2 | -0.11942 | 0.137807 | -0.86655 | 0.386188 | -0.38951 | 0.15068 | AFR | Myopathy | neurological | |
| 360 | -0.16388 | 0.309884 | -0.52883 | 0.596922 | -0.77124 | 0.443486 | AFR | Disorders of the | sense organs | |
| 360.2 | -0.49027 | 0.356923 | -1.3736 | 0.169567 | -1.18983 | 0.209288 | AFR | Progressive myo | sense organs | |
| 360.3 | 0.45244 | 0.685956 | 0.659575 | 0.509526 | -0.89201 | 1.796889 | AFR | Hypotony of eye | sense organs | |
| 361 | 0.131632 | 0.172061 | 0.765031 | 0.444253 | -0.2056 | 0.468865 | AFR | Retinal detachme | sense organs | |
| 361.1 | 0.342476 | 0.25242 | 1.356773 | 0.174853 | -0.15226 | 0.837209 | AFR | Retinal detachme | sense organs | |
| 361.2 | -0.79599 | 0.336573 | -2.36497 | 0.018031 | -1.45566 | -0.13631 | AFR | Retinoschisis and | sense organs | |
| 362 | 0.053158 | 0.084266 | 0.630839 | 0.528146 | -0.112 | 0.218317 | AFR | Other retinal disc | sense organs | |
| 362.1 | 0.275037 | 1.271499 | 0.216309 | 0.828747 | -2.21706 | 2.767129 | AFR | Retinopathy of pr | sense organs | |
| 362.2 | 0.020389 | 0.109905 | 0.185515 | 0.852825 | -0.19502 | 0.235798 | AFR | Degeneration of | sense organs | |
| 362.21 | 0.238015 | 0.318717 | 0.746792 | 0.455189 | -0.38666 | 0.862688 | AFR | Macular degener | sense organs | |
| 362.22 | -0.10814 | 0.34725 | -0.31141 | 0.755491 | -0.78873 | 0.572461 | AFR | Macular degener | sense organs | |
| 362.23 | -0.02186 | 0.174858 | -0.12501 | 0.900516 | -0.36457 | 0.320856 | AFR | Cystoid macular | sense organs | |
| 362.26 | 0.170158 | 0.180202 | 0.944264 | 0.345035 | -0.18303 | 0.523348 | AFR | Macular puckerin | sense organs | |
| 362.27 | -0.02995 | 0.198611 | -0.15082 | 0.880118 | -0.41922 | 0.359316 | AFR | Drusen (degener | sense organs | |
| 362.29 | 0.155524 | 0.229621 | 0.677307 | 0.498211 | -0.29453 | 0.605573 | AFR | Macular degener | sense organs | |
| 362.3 | 0.172445 | 0.196023 | 0.87972 | 0.379011 | -0.21175 | 0.556643 | AFR | Other nondiabeti | sense organs | |
| 362.31 | 0.327314 | 0.422861 | 0.774047 | 0.438903 | -0.50148 | 1.156107 | AFR | Separation of ret | sense organs | |
| 362.4 | 0.33618 | 0.16994 | 1.978228 | 0.047903 | 0.003104 | 0.669257 | AFR | Retinal vascular | sense organs | |
| 362.5 | 1.543727 | 0.851791 | 1.81233 | 0.069935 | -0.12575 | 3.213207 | AFR | Toxic maculopath | sense organs | |
| 362.6 | 0.005702 | 0.195929 | 0.029101 | 0.976784 | -0.37831 | 0.389716 | AFR | Peripheral retinal | sense organs | |
| 362.7 | -0.73192 | 0.275924 | -2.65262 | 0.007987 | -1.27272 | -0.19112 | AFR | Hereditary retinal | sense organs | |
| 362.8 | 0.154291 | 0.255225 | 0.604528 | 0.545492 | -0.34594 | 0.654523 | AFR | Retinal hemorrha | sense organs | |
| 362.9 | 0.246263 | 0.192476 | 1.279451 | 0.200738 | -0.13098 | 0.623509 | AFR | Retinal edema | sense organs | |
| 363 | 0.480351 | 0.234777 | 2.04599 | 0.040757 | 0.020197 | 0.940505 | AFR | Chorioretinal infla | sense organs | |
| 363.3 | 0.564034 | 0.25227 | 2.235829 | 0.025363 | 0.069593 | 1.058475 | AFR | Chorioretinal sca | sense organs | |
| 363.4 | -0.21628 | 0.973924 | -0.22207 | 0.824259 | -2.12514 | 1.692575 | AFR | Choroidal degen | sense organs | |
| 364 | 0.085367 | 0.150802 | 0.566089 | 0.571333 | -0.2102 | 0.380934 | AFR | Corneal opacity a | sense organs | |
| 364.1 | -0.17708 | 0.264118 | -0.67048 | 0.502554 | -0.69475 | 0.340577 | AFR | Corneal opacity | sense organs | |
| 364.2 | -0.31663 | 0.315536 | -1.00347 | 0.315635 | -0.93507 | 0.301809 | AFR | Corneal edema | sense organs | |
| 364.4 | 0.351311 | 0.24098 | 1.457846 | 0.144883 | -0.121 | 0.823623 | AFR | Corneal degener | sense organs | |
| 364.41 | 0.157461 | 0.291682 | 0.539838 | 0.589309 | -0.41423 | 0.729148 | AFR | Keratoconus | sense organs | |
| 364.5 | -0.04708 | 0.268087 | -0.1756 | 0.86061 | -0.57252 | 0.478365 | AFR | Corneal dystroph | sense organs | |
| 364.51 | 0.028477 | 0.278083 | 0.102406 | 0.918435 | -0.51656 | 0.57351 | AFR | Fuchs' dystrophy | sense organs | |

| | | | | | | | | | | |
|---|---|---|---|---|---|---|---|---|---|---|
| 364.9 | -0.06289 | 0.37692 | -0.16686 | 0.867477 | -0.80164 | 0.675855 | AFR | Cornea replaced | sense organs | |
| 365 | 0.014829 | 0.075934 | 0.195287 | 0.845169 | -0.134 | 0.163657 | AFR | Glaucoma | sense organs | |
| 365.1 | -0.08344 | 0.108949 | -0.76586 | 0.443759 | -0.29697 | 0.130096 | AFR | Open-angle glau | sense organs | |
| 365.11 | -0.10262 | 0.122517 | -0.8376 | 0.402253 | -0.34275 | 0.137509 | AFR | Primary open an | sense organs | |
| 365.2 | -0.08252 | 0.174353 | -0.47331 | 0.635992 | -0.42425 | 0.259203 | AFR | Primary angle-cl | sense organs | |
| 365.5 | 0.111021 | 0.980302 | 0.113252 | 0.909831 | -1.81034 | 2.032378 | AFR | Pseudoexfoliatio | sense organs | |
| 366 | -0.10973 | 0.069015 | -1.58996 | 0.111844 | -0.245 | 0.025536 | AFR | Cataract | sense organs | |
| 366.1 | 1.011498 | 0.392301 | 2.578376 | 0.009927 | 0.242603 | 1.780394 | AFR | Nonsenile Catara | sense organs | |
| 366.2 | 0.036235 | 0.069944 | 0.518056 | 0.604419 | -0.10085 | 0.173324 | AFR | Senile cataract | sense organs | |
| 366.3 | 0.697388 | 1.246279 | 0.559577 | 0.575768 | -1.74527 | 3.140049 | AFR | Traumatic catara | sense organs | |
| 367 | -0.00783 | 0.071033 | -0.11025 | 0.912215 | -0.14705 | 0.131391 | AFR | Disorders of refr | sense organs | |
| 367.1 | 0.031951 | 0.129505 | 0.246716 | 0.805128 | -0.22187 | 0.285777 | AFR | Myopia | sense organs | |
| 367.2 | -0.04719 | 0.113198 | -0.41692 | 0.676734 | -0.26906 | 0.174669 | AFR | Astigmatism | sense organs | |
| 367.4 | 0.001124 | 0.100191 | 0.011216 | 0.991051 | -0.19525 | 0.197494 | AFR | Presbyopia | sense organs | |
| 367.8 | 0.186763 | 0.182169 | 1.025215 | 0.305262 | -0.17028 | 0.543808 | AFR | Hypermetropia | sense organs | |
| 367.9 | -0.08988 | 0.11341 | -0.79255 | 0.428039 | -0.31216 | 0.132396 | AFR | Blindness and lo | sense organs | |
| 368 | 0.024962 | 0.071303 | 0.350084 | 0.726276 | -0.11479 | 0.164714 | AFR | Visual disturbanc | sense organs | |
| 368.1 | 0.088208 | 0.327897 | 0.269012 | 0.787921 | -0.55446 | 0.730873 | AFR | Amblyopia | sense organs | |
| 368.2 | -0.05384 | 0.16864 | -0.31927 | 0.749525 | -0.38437 | 0.276688 | AFR | Diplopia and disc | sense organs | |
| 368.3 | -0.36433 | 0.521371 | -0.69879 | 0.484685 | -1.3862 | 0.657541 | AFR | Anisometropia | sense organs | |
| 368.4 | 0.200133 | 0.200496 | 0.99819 | 0.318187 | -0.19283 | 0.593097 | AFR | Visual field defe | sense organs | |
| 368.9 | 0.21154 | 0.160493 | 1.318065 | 0.187482 | -0.10302 | 0.5261 | AFR | Subjective visual | sense organs | |
| 368.91 | -0.01522 | 0.352988 | -0.04312 | 0.965606 | -0.70707 | 0.676624 | AFR | Psychophysical v | sense organs | |
| 369 | 0.076467 | 0.09202 | 0.830979 | 0.405985 | -0.10389 | 0.256823 | AFR | Infection of the e | sense organs | |
| 369.2 | -0.04846 | 0.230997 | -0.2098 | 0.833824 | -0.50121 | 0.404283 | AFR | Eye infection, vir | sense organs | |
| 369.5 | 0.094302 | 0.097077 | 0.971413 | 0.331343 | -0.09597 | 0.284569 | AFR | Conjunctivitis, inf | sense organs | |
| 370 | -0.25939 | 0.191897 | -1.35172 | 0.176464 | -0.6355 | 0.11672 | AFR | Keratitis | sense organs | |
| 370.1 | -0.8529 | 0.317989 | -2.68216 | 0.007315 | -1.47614 | -0.22965 | AFR | Corneal ulcer | sense organs | |
| 370.2 | 0.15225 | 0.412812 | 0.368811 | 0.712269 | -0.65685 | 0.961347 | AFR | Superficial kerati | sense organs | |
| 370.3 | -0.34292 | 0.287186 | -1.19407 | 0.23245 | -0.90579 | 0.219954 | AFR | Keratoconjunctiv | sense organs | |
| 370.31 | -0.11486 | 0.48171 | -0.23845 | 0.811532 | -1.059 | 0.82927 | AFR | Keratoconjunctiv | sense organs | |
| 371 | -0.04621 | 0.07612 | -0.60709 | 0.54379 | -0.19541 | 0.102981 | AFR | Inflammation of t | sense organs | |
| 371.1 | 0.034832 | 0.15243 | 0.228509 | 0.819251 | -0.26393 | 0.333588 | AFR | Uveitis, noninfect | sense organs | |
| 371.2 | 0.064873 | 0.126962 | 0.510966 | 0.609375 | -0.18397 | 0.313715 | AFR | Conjunctivitis, no | sense organs | |
| 371.21 | -0.00435 | 0.136699 | -0.03181 | 0.974627 | -0.27227 | 0.263578 | AFR | Allergic conjuncti | sense organs | |
| 371.3 | -0.06637 | 0.097183 | -0.68298 | 0.494622 | -0.25685 | 0.124102 | AFR | Inflammation of e | sense organs | |
| 371.33 | 0.236393 | 0.615404 | 0.384127 | 0.700884 | -0.96978 | 1.442564 | AFR | Noninfectious de | sense organs | |
| 371.9 | -0.5971 | 0.854416 | -0.69885 | 0.484649 | -2.27173 | 1.077519 | AFR | Chronic inflamma | sense organs | |

| | | | | | | | | | | |
|---|---|---|---|---|---|---|---|---|---|---|
| 372 | 0.02348 | 0.095122 | 0.246846 | 0.805027 | -0.16295 | 0.209915 | AFR | Disorders of conj | sense organs | |
| 374 | -0.13723 | 0.100249 | -1.36894 | 0.171019 | -0.33372 | 0.05925 | AFR | Other disorders c | sense organs | |
| 374.1 | -0.10489 | 0.29202 | -0.35919 | 0.719456 | -0.67724 | 0.467459 | AFR | Ectropion or entr | sense organs | |
| 374.2 | -0.21433 | 0.437624 | -0.48975 | 0.62431 | -1.07205 | 0.643401 | AFR | Lagophthalmos | sense organs | |
| 374.3 | -0.17359 | 0.147513 | -1.17676 | 0.239293 | -0.46271 | 0.115533 | AFR | Ptosis of eyelid | sense organs | |
| 374.6 | -0.52111 | 0.219142 | -2.37794 | 0.017409 | -0.95062 | -0.0916 | AFR | Dermatochalasis | sense organs | |
| 375 | 0.097448 | 0.178856 | 0.54484 | 0.585863 | -0.2531 | 0.447999 | AFR | Disorders of lacri | sense organs | |
| 375.1 | -0.007 | 0.077012 | -0.09096 | 0.927526 | -0.15795 | 0.143936 | AFR | Dry eyes | sense organs | |
| 375.2 | 0.097448 | 0.178856 | 0.54484 | 0.585863 | -0.2531 | 0.447999 | AFR | Epiphora | sense organs | |
| 376 | 0.10355 | 0.383353 | 0.270117 | 0.78707 | -0.64781 | 0.854908 | AFR | Disorders of the ( | sense organs | |
| 377 | -0.00321 | 0.14447 | -0.02219 | 0.982298 | -0.28636 | 0.27995 | AFR | Disorders of optic | sense organs | |
| 377.1 | 0.241581 | 0.200062 | 1.20753 | 0.227228 | -0.15053 | 0.633694 | AFR | Optic atrophy | sense organs | |
| 377.3 | -0.10258 | 0.209432 | -0.48981 | 0.624268 | -0.51306 | 0.307897 | AFR | Optic neuritis/neu | sense organs | |
| 378 | -0.11037 | 0.145979 | -0.75604 | 0.449626 | -0.39648 | 0.175748 | AFR | Strabismus and c | sense organs | |
| 378.1 | -0.1878 | 0.177116 | -1.06034 | 0.28899 | -0.53494 | 0.159338 | AFR | Strabismus (not s | sense organs | |
| 378.2 | 0.938001 | 0.446519 | 2.100699 | 0.035667 | 0.062841 | 1.813162 | AFR | Nystagmus and c | sense organs | |
| 378.5 | -0.26191 | 0.347692 | -0.75329 | 0.451278 | -0.94337 | 0.419552 | AFR | Paralytic strabisn | sense organs | |
| 379 | 0.060122 | 0.074005 | 0.812411 | 0.416556 | -0.08492 | 0.205169 | AFR | Other disorders c | sense organs | |
| 379.1 | -0.43472 | 0.268166 | -1.6211 | 0.104996 | -0.96032 | 0.090871 | AFR | Scleritis and epis | sense organs | |
| 379.2 | 0.07725 | 0.085582 | 0.902653 | 0.36671 | -0.09049 | 0.244987 | AFR | Disorders of vitre | sense organs | |
| 379.3 | -0.20386 | 0.479884 | -0.42482 | 0.670971 | -1.14442 | 0.736693 | AFR | Aphakia and othe | sense organs | |
| 379.4 | 0.159663 | 0.259455 | 0.615379 | 0.538305 | -0.34886 | 0.668185 | AFR | Anomalies of pup | sense organs | |
| 379.5 | 0.155585 | 0.209759 | 0.741731 | 0.45825 | -0.25554 | 0.566705 | AFR | Disorders of iris a | sense organs | |
| 379.51 | -0.43534 | 0.646931 | -0.67293 | 0.500991 | -1.7033 | 0.832621 | AFR | Pigmentary iris d | sense organs | |
| 379.9 | 0.09747 | 0.094944 | 1.026606 | 0.304606 | -0.08862 | 0.283557 | AFR | Pain, swelling or | sense organs | |
| 380 | 0.029136 | 0.272877 | 0.106775 | 0.914967 | -0.50569 | 0.563966 | AFR | Disorders of exte | sense organs | |
| 380.1 | -0.19116 | 0.141972 | -1.3465 | 0.178142 | -0.46942 | 0.087095 | AFR | Otitis externa | sense organs | |
| 380.4 | -0.137 | 0.075911 | -1.80476 | 0.071112 | -0.28578 | 0.011781 | AFR | Impacted cerume | sense organs | |
| 381 | -0.05698 | 0.10577 | -0.5387 | 0.590091 | -0.26428 | 0.150327 | AFR | Otitis media and | sense organs | |
| 381.1 | -0.15949 | 0.136365 | -1.1696 | 0.24216 | -0.42676 | 0.107777 | AFR | Otitis media | sense organs | |
| 381.11 | -0.12696 | 0.166367 | -0.76313 | 0.445387 | -0.45303 | 0.199113 | AFR | Suppurative and | sense organs | |
| 381.2 | 0.130136 | 0.160827 | 0.809165 | 0.41842 | -0.18508 | 0.445352 | AFR | Eustachian tube | sense organs | |
| 381.3 | -0.10765 | 0.3669 | -0.2934 | 0.769213 | -0.82676 | 0.61146 | AFR | Mastoiditis & rela | sense organs | |
| 381.9 | 0.352426 | 0.291951 | 1.207142 | 0.227378 | -0.21979 | 0.924639 | AFR | Otorrhea | sense organs | |
| 382 | -0.13379 | 0.097153 | -1.37714 | 0.168469 | -0.32421 | 0.056623 | AFR | Otalgia | sense organs | |
| 383 | 0.165659 | 0.627172 | 0.264137 | 0.791675 | -1.06358 | 1.394894 | AFR | Otosclerosis | sense organs | |
| 384 | 0.106316 | 0.411341 | 0.258461 | 0.796051 | -0.6999 | 0.91253 | AFR | Other disorders c | sense organs | |
| 384.4 | 0.321968 | 0.482083 | 0.667868 | 0.504218 | -0.6229 | 1.266834 | AFR | Perforation of tyn | sense organs | |

| | | | | | | | | | |
|---|---|---|---|---|---|---|---|---|---|
| 385 | 0.095073 | 0.366947 | 0.259091 | 0.795565 | -0.62413 | 0.814276 | AFR | Other disorders c | sense organs |
| 385.3 | 0.332892 | 0.593761 | 0.56065 | 0.575036 | -0.83086 | 1.496643 | AFR | Cholesteatoma | sense organs |
| 385.5 | -0.3789 | 0.970734 | -0.39032 | 0.696298 | -2.2815 | 1.523704 | AFR | Tympanosclerosi | sense organs |
| 386 | 0.142777 | 0.117104 | 1.219236 | 0.222755 | -0.08674 | 0.372296 | AFR | Vertiginous syndı | sense organs |
| 386.1 | 0.245205 | 0.39649 | 0.618439 | 0.536286 | -0.5319 | 1.022312 | AFR | Meniere's diseas | sense organs |
| 386.2 | 0.114015 | 0.136549 | 0.834978 | 0.40373 | -0.15362 | 0.381646 | AFR | Peripheral or cen | sense organs |
| 386.21 | -0.04818 | 1.119681 | -0.04303 | 0.965678 | -2.24271 | 2.146355 | AFR | Central origin ver | sense organs |
| 386.3 | 0.194262 | 0.272498 | 0.712895 | 0.47591 | -0.33982 | 0.728348 | AFR | Labyrinthitis | sense organs |
| 386.9 | 0.031111 | 0.062466 | 0.498048 | 0.61845 | -0.09132 | 0.153541 | AFR | Dizziness and gi | sense organs |
| 388 | -0.02882 | 0.116479 | -0.24739 | 0.804603 | -0.25711 | 0.199479 | AFR | Other disorders c | sense organs |
| 389 | -0.04049 | 0.07204 | -0.56209 | 0.574051 | -0.18169 | 0.100702 | AFR | Hearing loss | sense organs |
| 389.1 | -0.0297 | 0.102586 | -0.28952 | 0.77218 | -0.23077 | 0.171364 | AFR | Sensorineural he | sense organs |
| 389.2 | 0.061453 | 0.260254 | 0.236126 | 0.813335 | -0.44864 | 0.571542 | AFR | Conductive heari | sense organs |
| 389.3 | 0.199438 | 0.37427 | 0.532871 | 0.594123 | -0.53412 | 0.932994 | AFR | Degenerative an | sense organs |
| 389.4 | -0.05404 | 0.105703 | -0.51121 | 0.609207 | -0.26121 | 0.153138 | AFR | Tinnitus | sense organs |
| 389.5 | -0.18541 | 0.334752 | -0.55387 | 0.579669 | -0.84151 | 0.470694 | AFR | Disorders of acou | sense organs |
| 394 | -0.01785 | 0.093951 | -0.19 | 0.849312 | -0.20199 | 0.16629 | AFR | Rheumatic disea | circulatory system |
| 394.1 | -0.05305 | 0.261214 | -0.2031 | 0.839055 | -0.56502 | 0.458916 | AFR | Mitral valve stenc | circulatory system |
| 394.2 | -0.12916 | 0.157732 | -0.81884 | 0.412876 | -0.43831 | 0.179991 | AFR | Mitral valve disea | circulatory system |
| 394.3 | 0.58324 | 0.774252 | 0.753295 | 0.451273 | -0.93427 | 2.100746 | AFR | Aortic valve disea | circulatory system |
| 394.4 | 0.074249 | 0.564478 | 0.131537 | 0.895351 | -1.03211 | 1.180606 | AFR | Acute rheumatic | circulatory system |
| 394.7 | 0.201392 | 0.142141 | 1.416848 | 0.156527 | -0.0772 | 0.479984 | AFR | Disease of tricus | circulatory system |
| 395 | -0.00454 | 0.073819 | -0.06155 | 0.950922 | -0.14923 | 0.140139 | AFR | Heart valve disor | circulatory system |
| 395.1 | -0.0332 | 0.095141 | -0.34895 | 0.727126 | -0.21967 | 0.153273 | AFR | Nonrheumatic mi | circulatory system |
| 395.2 | -0.0652 | 0.109999 | -0.59278 | 0.553332 | -0.2808 | 0.150389 | AFR | Nonrheumatic ac | circulatory system |
| 395.3 | 0.105434 | 0.113205 | 0.931362 | 0.351666 | -0.11644 | 0.327311 | AFR | Nonrheumatic tri | circulatory system |
| 395.4 | 0.237539 | 0.246854 | 0.962266 | 0.335916 | -0.24629 | 0.721365 | AFR | Nonrheumatic pu | circulatory system |
| 395.6 | -0.20099 | 0.172027 | -1.16836 | 0.242661 | -0.53816 | 0.136177 | AFR | Heart valve repla | circulatory system |
| 396 | 0.097648 | 0.08157 | 1.197108 | 0.231264 | -0.06223 | 0.257521 | AFR | Abnormal heart s | circulatory system |
| 401 | 0.059722 | 0.068628 | 0.870226 | 0.384177 | -0.07479 | 0.194232 | AFR | Hypertension | circulatory system |
| 401.1 | 0.046746 | 0.0677 | 0.690489 | 0.489887 | -0.08594 | 0.179434 | AFR | Essential hyperte | circulatory system |
| 401.2 | 0.086235 | 0.064104 | 1.345236 | 0.178549 | -0.03941 | 0.211876 | AFR | Hypertensive hea | circulatory system |
| 401.21 | 0.215453 | 0.088107 | 2.445344 | 0.014471 | 0.042766 | 0.38814 | AFR | Hypertensive hea | circulatory system |
| 401.22 | 0.06851 | 0.069355 | 0.987808 | 0.323247 | -0.06742 | 0.204444 | AFR | Hypertensive chr | circulatory system |
| 401.3 | 0.115845 | 0.070153 | 1.651317 | 0.098674 | -0.02165 | 0.253342 | AFR | Other hypertensi | circulatory system |
| 402 | -0.15962 | 0.07903 | -2.01968 | 0.043417 | -0.31451 | -0.00472 | AFR | Elevated blood p | circulatory system |
| 411 | 0.040263 | 0.062926 | 0.639848 | 0.522272 | -0.08307 | 0.163597 | AFR | Ischemic Heart D | circulatory system |
| 411.1 | 0.134405 | 0.148581 | 0.904591 | 0.365682 | -0.15681 | 0.425618 | AFR | Unstable angina | circulatory system |

| | | | | | | | | | |
|---|---|---|---|---|---|---|---|---|---|
| 411.2 | 0.01605 | 0.08521 | 0.188352 | 0.850601 | -0.15096 | 0.183059 | AFR | Myocardial infarc | circulatory system |
| 411.3 | 0.071581 | 0.101389 | 0.705999 | 0.480189 | -0.12714 | 0.270301 | AFR | Angina pectoris | circulatory system |
| 411.4 | -0.0032 | 0.067894 | -0.04716 | 0.962388 | -0.13627 | 0.129867 | AFR | Coronary atheros | circulatory system |
| 411.41 | -0.50564 | 0.318994 | -1.5851 | 0.112944 | -1.13085 | 0.11958 | AFR | Aneurysm and di | circulatory system |
| 411.8 | 0.187851 | 0.112068 | 1.676229 | 0.093693 | -0.0318 | 0.4075 | AFR | Other chronic isc | circulatory system |
| 411.9 | 0.142955 | 0.144939 | 0.986309 | 0.323982 | -0.14112 | 0.42703 | AFR | Other acute and | circulatory system |
| 414 | -0.00328 | 0.09582 | -0.03423 | 0.972693 | -0.19108 | 0.184524 | AFR | Other forms of ch | circulatory system |
| 414.2 | -0.55375 | 0.338002 | -1.6383 | 0.101359 | -1.21622 | 0.108723 | AFR | ASCVD | circulatory system |
| 415 | -0.07692 | 0.074868 | -1.02735 | 0.304256 | -0.22365 | 0.069823 | AFR | Pulmonary heart | circulatory system |
| 415.1 | -0.11949 | 0.106069 | -1.12652 | 0.259947 | -0.32738 | 0.088403 | AFR | Acute pulmonary | circulatory system |
| 415.11 | -0.11949 | 0.106069 | -1.12652 | 0.259947 | -0.32738 | 0.088403 | AFR | Pulmonary embo | circulatory system |
| 415.2 | -0.09044 | 0.086102 | -1.05037 | 0.293546 | -0.2592 | 0.078318 | AFR | Chronic pulmona | circulatory system |
| 415.21 | -0.15301 | 0.140169 | -1.09157 | 0.27502 | -0.42773 | 0.121721 | AFR | Primary pulmona | circulatory system |
| 416 | 0.015348 | 0.065939 | 0.232767 | 0.815942 | -0.11389 | 0.144586 | AFR | Cardiomegaly | circulatory system |
| 418 | 0.101297 | 0.054615 | 1.854734 | 0.063634 | -0.00575 | 0.208341 | AFR | Nonspecific ches | circulatory system |
| 418.1 | 0.18171 | 0.107889 | 1.684233 | 0.092137 | -0.02975 | 0.393169 | AFR | Precordial pain | circulatory system |
| 420 | 0.026531 | 0.089272 | 0.297193 | 0.766319 | -0.14844 | 0.201501 | AFR | Carditis | circulatory system |
| 420.1 | 0.34634 | 0.333835 | 1.037459 | 0.299522 | -0.30796 | 1.000645 | AFR | Myocarditis | circulatory system |
| 420.2 | 0.067406 | 0.098256 | 0.686023 | 0.492698 | -0.12517 | 0.259983 | AFR | Pericarditis | circulatory system |
| 420.21 | 0.083154 | 0.23715 | 0.350638 | 0.72586 | -0.38165 | 0.547959 | AFR | Acute pericarditis | circulatory system |
| 420.22 | 0.269147 | 0.354917 | 0.758339 | 0.448248 | -0.42648 | 0.964772 | AFR | Chronic pericardi | circulatory system |
| 420.3 | -0.1617 | 0.17507 | -0.92362 | 0.355685 | -0.50483 | 0.181433 | AFR | Endocarditis | circulatory system |
| 425 | 0.125383 | 0.086052 | 1.457059 | 0.1451 | -0.04328 | 0.294042 | AFR | Cardiomyopathy | circulatory system |
| 425.1 | 0.148639 | 0.08843 | 1.680862 | 0.09279 | -0.02468 | 0.32196 | AFR | Primary/intrinsic | circulatory system |
| 425.11 | -0.05947 | 0.260736 | -0.22809 | 0.819575 | -0.57051 | 0.451562 | AFR | Hypertrophic obs | circulatory system |
| 425.12 | -0.23012 | 0.204629 | -1.12455 | 0.260779 | -0.63118 | 0.17095 | AFR | Other hypertroph | circulatory system |
| 425.2 | 0.07553 | 0.144149 | 0.523973 | 0.600297 | -0.207 | 0.358058 | AFR | Secondary/extrin | circulatory system |
| 425.8 | 0.2999 | 0.377894 | 0.793608 | 0.427424 | -0.44076 | 1.040559 | AFR | Other cardiomyo | circulatory system |
| 426 | 0.03469 | 0.058395 | 0.59406 | 0.552472 | -0.07976 | 0.149143 | AFR | Cardiac conducti | circulatory system |
| 426.2 | 0.032115 | 0.136912 | 0.23457 | 0.814543 | -0.23623 | 0.300458 | AFR | Atrioventricular [A | circulatory system |
| 426.21 | 0.188111 | 0.173054 | 1.087008 | 0.277033 | -0.15107 | 0.52729 | AFR | First degree AV b | circulatory system |
| 426.22 | -0.25596 | 0.600064 | -0.42656 | 0.669702 | -1.43207 | 0.920142 | AFR | Mobitz II AV block | circulatory system |
| 426.23 | -0.34023 | 0.274329 | -1.24023 | 0.214891 | -0.87791 | 0.197444 | AFR | Second degree A | circulatory system |
| 426.24 | 0.014429 | 0.258691 | 0.055776 | 0.95552 | -0.4926 | 0.521454 | AFR | Atrioventricular b | circulatory system |
| 426.25 | -1.33299 | 0.474077 | -2.81176 | 0.004927 | -2.26216 | -0.40381 | AFR | Other heart block | circulatory system |
| 426.3 | -0.17229 | 0.126165 | -1.3656 | 0.172065 | -0.41957 | 0.074988 | AFR | Bundle branch bl | circulatory system |
| 426.31 | -0.12817 | 0.177767 | -0.72099 | 0.470914 | -0.47658 | 0.220248 | AFR | Right bundle bra | circulatory system |
| 426.32 | -0.20865 | 0.17341 | -1.20322 | 0.22889 | -0.54853 | 0.131227 | AFR | Left bundle branc | circulatory system |

| | | | | | | | | | |
|---|---|---|---|---|---|---|---|---|---|
| 426.4 | 0.360064 | 0.681484 | 0.528352 | 0.597255 | -0.97562 | 1.695749 | AFR | Anomalous atriov | circulatory system |
| 426.7 | 0.036056 | 0.059799 | 0.602955 | 0.546539 | -0.08115 | 0.153261 | AFR | Abnormal electro | circulatory system |
| 426.8 | -0.10919 | 0.221387 | -0.49322 | 0.621856 | -0.5431 | 0.324718 | AFR | Other cardiac co | circulatory system |
| 426.9 | 0.20765 | 0.112566 | 1.844689 | 0.065083 | -0.01298 | 0.428276 | AFR | Cardiac pacemal | circulatory system |
| 426.91 | 0.101424 | 0.12626 | 0.803297 | 0.421803 | -0.14604 | 0.348889 | AFR | Cardiac pacemal | circulatory system |
| 426.92 | 0.184521 | 0.145969 | 1.264109 | 0.206191 | -0.10157 | 0.470616 | AFR | Cardiac defibrilla | circulatory system |
| 427 | 0.045855 | 0.055343 | 0.828555 | 0.407356 | -0.06262 | 0.154326 | AFR | Cardiac dysrhyth | circulatory system |
| 427.1 | -0.07087 | 0.081286 | -0.87182 | 0.383307 | -0.23018 | 0.088451 | AFR | Paroxysmal tachy | circulatory system |
| 427.11 | -0.14163 | 0.104763 | -1.35186 | 0.17642 | -0.34696 | 0.063707 | AFR | Paroxysmal supr | circulatory system |
| 427.12 | -0.05534 | 0.105399 | -0.52504 | 0.599554 | -0.26192 | 0.151239 | AFR | Paroxysmal vent | circulatory system |
| 427.2 | 0.110782 | 0.083596 | 1.325207 | 0.185103 | -0.05306 | 0.274627 | AFR | Atrial fibrillation a | circulatory system |
| 427.21 | 0.120446 | 0.084921 | 1.418328 | 0.156095 | -0.046 | 0.286888 | AFR | Atrial fibrillation | circulatory system |
| 427.22 | -0.07811 | 0.133791 | -0.58385 | 0.559319 | -0.34034 | 0.184112 | AFR | Atrial flutter | circulatory system |
| 427.3 | 0.039389 | 0.071875 | 0.548023 | 0.583676 | -0.10148 | 0.18026 | AFR | Other specified c | circulatory system |
| 427.4 | 0.105604 | 0.170913 | 0.61788 | 0.536654 | -0.22938 | 0.440588 | AFR | Cardiac arrest ar | circulatory system |
| 427.41 | 0.343508 | 0.27393 | 1.253998 | 0.209843 | -0.19339 | 0.880401 | AFR | Ventricular fibrilla | circulatory system |
| 427.42 | 0.134975 | 0.190325 | 0.709179 | 0.478213 | -0.23806 | 0.508005 | AFR | Cardiac arrest | circulatory system |
| 427.5 | 0.00402 | 0.103856 | 0.038707 | 0.969124 | -0.19953 | 0.207574 | AFR | Arrhythmia (cardi | circulatory system |
| 427.6 | 0.07122 | 0.091512 | 0.778258 | 0.436417 | -0.10814 | 0.250581 | AFR | Premature beats | circulatory system |
| 427.61 | 0.136644 | 0.165056 | 0.827869 | 0.407745 | -0.18686 | 0.460147 | AFR | Supraventricular | circulatory system |
| 427.7 | -0.01044 | 0.070983 | -0.14711 | 0.883046 | -0.14957 | 0.128681 | AFR | Tachycardia NOS | circulatory system |
| 427.8 | 0.078954 | 0.143924 | 0.548582 | 0.583293 | -0.20313 | 0.36104 | AFR | Sinoatrial node d | circulatory system |
| 427.9 | 0.104264 | 0.071854 | 1.451054 | 0.146765 | -0.03657 | 0.245095 | AFR | Palpitations | circulatory system |
| 428 | 0.096419 | 0.070549 | 1.366691 | 0.171722 | -0.04185 | 0.234692 | AFR | Congestive heart | circulatory system |
| 428.1 | 0.004505 | 0.075952 | 0.059317 | 0.9527 | -0.14436 | 0.153369 | AFR | Congestive heart | circulatory system |
| 428.2 | 0.218521 | 0.095218 | 2.294942 | 0.021736 | 0.031896 | 0.405145 | AFR | Heart failure NOS | circulatory system |
| 428.3 | 0.152961 | 0.093629 | 1.633699 | 0.102322 | -0.03055 | 0.336471 | AFR | Heart failure with | circulatory system |
| 428.4 | 0.12678 | 0.088081 | 1.439355 | 0.15005 | -0.04586 | 0.299415 | AFR | Heart failure with | circulatory system |
| 429 | 0.041631 | 0.063777 | 0.652751 | 0.513917 | -0.08337 | 0.166632 | AFR | Ill-defined descri | circulatory system |
| 429.1 | 0.025914 | 0.140789 | 0.184063 | 0.853964 | -0.25003 | 0.301856 | AFR | Heart transplant/ | circulatory system |
| 429.2 | 0.108614 | 0.117025 | 0.928125 | 0.353343 | -0.12075 | 0.337978 | AFR | Abnormal functio | circulatory system |
| 429.3 | 0.058325 | 0.067541 | 0.863552 | 0.387834 | -0.07405 | 0.190703 | AFR | Symptoms involv | circulatory system |
| 429.9 | -0.19198 | 0.236821 | -0.81066 | 0.417562 | -0.65614 | 0.27218 | AFR | Cardiac complica | circulatory system |
| 430 | 0.004573 | 0.157144 | 0.029101 | 0.976784 | -0.30342 | 0.31257 | AFR | Intracranial hemo | circulatory system |
| 430.1 | -0.24113 | 0.254274 | -0.94829 | 0.342979 | -0.73949 | 0.257241 | AFR | Subarachnoid he | circulatory system |
| 430.2 | 0.067214 | 0.224144 | 0.29987 | 0.764276 | -0.3721 | 0.506529 | AFR | Intracerebral hen | circulatory system |
| 430.3 | -0.37448 | 0.242404 | -1.54487 | 0.122377 | -0.84959 | 0.10062 | AFR | Subdural hemorr | circulatory system |
| 433 | 0.062566 | 0.070769 | 0.884088 | 0.376649 | -0.07614 | 0.201271 | AFR | Cerebrovascular | circulatory system |

| | | | | | | | | | |
|---|---|---|---|---|---|---|---|---|---|
| 433.1 | -0.11906 | 0.09787 | -1.21656 | 0.223772 | -0.31089 | 0.072757 | AFR | Occlusion and st | circulatory system |
| 433.11 | -0.10972 | 0.195987 | -0.55981 | 0.575607 | -0.49384 | 0.274411 | AFR | Occlusion of cere | circulatory system |
| 433.12 | 0.054905 | 0.226015 | 0.242926 | 0.808063 | -0.38808 | 0.497885 | AFR | Cerebral atheros | circulatory system |
| 433.2 | 0.200348 | 0.094026 | 2.130769 | 0.033108 | 0.01606 | 0.384636 | AFR | Occlusion of cere | circulatory system |
| 433.21 | 0.192777 | 0.095294 | 2.022974 | 0.043076 | 0.006005 | 0.37955 | AFR | Cerebral artery o | circulatory system |
| 433.3 | 0.104566 | 0.078669 | 1.329193 | 0.183784 | -0.04962 | 0.258755 | AFR | Cerebral ischemi | circulatory system |
| 433.31 | 0.136604 | 0.083683 | 1.632402 | 0.102595 | -0.02741 | 0.30062 | AFR | Transient cerebra | circulatory system |
| 433.32 | 125.9114 | | | | | | AFR | Moyamoya disea | circulatory system |
| 433.5 | -0.07519 | 0.181821 | -0.41357 | 0.679192 | -0.43156 | 0.281167 | AFR | Cerebral aneurys | circulatory system |
| 433.6 | 0.139623 | 0.349078 | 0.399977 | 0.689174 | -0.54456 | 0.823802 | AFR | Acute, but ill-defi | circulatory system |
| 433.8 | 0.142767 | 0.139849 | 1.020865 | 0.307318 | -0.13133 | 0.416865 | AFR | Late effects of ce | circulatory system |
| 440 | -0.06563 | 0.079384 | -0.82675 | 0.40838 | -0.22122 | 0.08996 | AFR | Atherosclerosis | circulatory system |
| 440.1 | 0.046704 | 0.287269 | 0.16258 | 0.870849 | -0.51633 | 0.60974 | AFR | Atherosclerosis c | circulatory system |
| 440.2 | 0.105165 | 0.131065 | 0.80239 | 0.422327 | -0.15172 | 0.362048 | AFR | Atherosclerosis c | circulatory system |
| 440.21 | -0.25634 | 0.275087 | -0.93184 | 0.351419 | -0.7955 | 0.282824 | AFR | Atherosclerosis c | circulatory system |
| 440.22 | 0.220786 | 0.205252 | 1.075683 | 0.282069 | -0.1815 | 0.623072 | AFR | Atherosclerosis c | circulatory system |
| 440.9 | -0.06726 | 0.089514 | -0.75141 | 0.452409 | -0.24271 | 0.108183 | AFR | Atherosclerosis c | circulatory system |
| 441 | 0.039486 | 0.195982 | 0.201475 | 0.840327 | -0.34463 | 0.423604 | AFR | Vascular insuffici | circulatory system |
| 441.1 | -0.04013 | 0.340522 | -0.11783 | 0.906199 | -0.70754 | 0.627286 | AFR | Acute vascular in | circulatory system |
| 441.2 | -0.21824 | 0.295135 | -0.73947 | 0.45962 | -0.7967 | 0.36021 | AFR | Chronic vascular | circulatory system |
| 442 | -0.00202 | 0.123117 | -0.0164 | 0.986918 | -0.24332 | 0.239286 | AFR | Other aneurysm | circulatory system |
| 442.1 | 0.023953 | 0.168485 | 0.142167 | 0.886948 | -0.30627 | 0.354177 | AFR | Aortic aneurysm | circulatory system |
| 442.11 | 0.218994 | 0.242577 | 0.902781 | 0.366642 | -0.25645 | 0.694437 | AFR | Abdominal aortic | circulatory system |
| 442.2 | 0.465758 | 0.439427 | 1.05992 | 0.289181 | -0.3955 | 1.327019 | AFR | Aneurysm of iliac | circulatory system |
| 442.3 | 0.083351 | 0.328214 | 0.253953 | 0.799532 | -0.55994 | 0.726638 | AFR | Aneurysm of arte | circulatory system |
| 442.4 | -0.05229 | 0.340519 | -0.15357 | 0.877948 | -0.7197 | 0.615111 | AFR | Arterial dissectio | circulatory system |
| 442.8 | 0.076368 | 0.233796 | 0.326646 | 0.743936 | -0.38186 | 0.5346 | AFR | Aneurysm of othe | circulatory system |
| 443 | -0.07043 | 0.083273 | -0.84578 | 0.397673 | -0.23364 | 0.092782 | AFR | Peripheral vascu | circulatory system |
| 443.1 | -0.04705 | 0.168204 | -0.27975 | 0.779673 | -0.37673 | 0.282619 | AFR | Raynaud's syndr | circulatory system |
| 443.7 | -0.06617 | 0.127055 | -0.5208 | 0.602504 | -0.31519 | 0.182853 | AFR | Peripheral angio | circulatory system |
| 443.8 | 0.030809 | 0.200068 | 0.153994 | 0.877614 | -0.36132 | 0.422936 | AFR | Other specified p | circulatory system |
| 443.9 | -0.02002 | 0.099975 | -0.20026 | 0.841277 | -0.21597 | 0.175926 | AFR | Peripheral vascu | circulatory system |
| 444 | -0.10067 | 0.153169 | -0.65722 | 0.511041 | -0.40087 | 0.19954 | AFR | Arterial embolism | circulatory system |
| 444.1 | -0.13053 | 0.237846 | -0.54879 | 0.583147 | -0.5967 | 0.335641 | AFR | Arterial embolism | circulatory system |
| 444.2 | -0.46003 | 0.517212 | -0.88944 | 0.373766 | -1.47375 | 0.553688 | AFR | Embolism and th | circulatory system |
| 446 | -0.03883 | 0.175949 | -0.22067 | 0.825352 | -0.38368 | 0.306028 | AFR | Polyarteritis nodo | circulatory system |
| 446.2 | -0.71305 | 0.663469 | -1.07473 | 0.282495 | -2.01343 | 0.587325 | AFR | Acute febrile muc | circulatory system |
| 446.3 | 0.141352 | 0.507767 | 0.27838 | 0.780721 | -0.85385 | 1.136556 | AFR | Hypersensitivity a | circulatory system |

| | | | | | | | | | |
|---|---|---|---|---|---|---|---|---|---|
| 446.4 | 0.238011 | 0.59271 | 0.401564 | 0.688005 | -0.92368 | 1.399702 | AFR | Wegener's granu | circulatory system |
| 446.5 | 0.275876 | 0.400498 | 0.688832 | 0.490929 | -0.50909 | 1.060837 | AFR | Giant cell arteritis | circulatory system |
| 446.6 | 0.012001 | 0.711274 | 0.016873 | 0.986538 | -1.38207 | 1.406072 | AFR | Polyarteritis nodo | circulatory system |
| 446.8 | -0.07161 | 0.332482 | -0.21539 | 0.82946 | -0.72327 | 0.580038 | AFR | Thrombotic micro | circulatory system |
| 446.9 | -0.20448 | 0.272205 | -0.75121 | 0.452527 | -0.73799 | 0.329029 | AFR | Arteritis NOS | circulatory system |
| 447 | -0.06598 | 0.093496 | -0.7057 | 0.480372 | -0.24923 | 0.117268 | AFR | Other disorders c | circulatory system |
| 447.1 | -0.21509 | 0.165416 | -1.30029 | 0.193502 | -0.5393 | 0.109121 | AFR | Stricture of artery | circulatory system |
| 447.7 | -0.14681 | 0.195836 | -0.74967 | 0.453456 | -0.53064 | 0.23702 | AFR | Aortic ectasia | circulatory system |
| 448 | -0.49676 | 0.359795 | -1.38067 | 0.16738 | -1.20194 | 0.208427 | AFR | Disease of capilla | circulatory system |
| 450 | -0.10198 | 0.161024 | -0.63333 | 0.526519 | -0.41758 | 0.213621 | AFR | Noninfectious dis | circulatory system |
| 451 | -0.04856 | 0.16076 | -0.30208 | 0.76259 | -0.36365 | 0.266521 | AFR | Phlebitis and thro | circulatory system |
| 451.2 | -0.17241 | 0.24718 | -0.69752 | 0.48548 | -0.65688 | 0.312052 | AFR | Phlebitis and thro | circulatory system |
| 452 | -0.14822 | 0.074232 | -1.99668 | 0.04586 | -0.29371 | -0.00273 | AFR | Other venous em | circulatory system |
| 452.1 | -0.19438 | 1.027492 | -0.18918 | 0.84995 | -2.20823 | 1.819464 | AFR | Iatrogenic pulmo | circulatory system |
| 452.2 | -0.09616 | 0.082218 | -1.16958 | 0.242171 | -0.2573 | 0.064984 | AFR | Deep vein thromb | circulatory system |
| 452.8 | -0.23673 | 0.387128 | -0.6115 | 0.540867 | -0.99549 | 0.522028 | AFR | Postphlebitic syn | circulatory system |
| 453 | -0.22394 | 0.217004 | -1.03196 | 0.30209 | -0.64926 | 0.20138 | AFR | Chronic venous h | circulatory system |
| 454 | -0.0091 | 0.113529 | -0.08013 | 0.936137 | -0.23161 | 0.213417 | AFR | Varicose veins | circulatory system |
| 454.1 | -0.04416 | 0.123249 | -0.35833 | 0.720094 | -0.28573 | 0.197399 | AFR | Varicose veins of | circulatory system |
| 454.11 | -0.03696 | 0.141584 | -0.26106 | 0.794044 | -0.31446 | 0.240537 | AFR | Varicose veins of | circulatory system |
| 455 | 0.038274 | 0.060533 | 0.632286 | 0.5272 | -0.08037 | 0.156917 | AFR | Hemorrhoids | circulatory system |
| 456 | -0.11765 | 0.088407 | -1.33076 | 0.183269 | -0.29092 | 0.055626 | AFR | Chronic venous i | circulatory system |
| 457 | 0.013491 | 0.061811 | 0.218264 | 0.827224 | -0.10766 | 0.134638 | AFR | Encounter for lon | circulatory system |
| 457.2 | -0.08593 | 0.119449 | -0.71939 | 0.471903 | -0.32004 | 0.148185 | AFR | Encounter for lon | circulatory system |
| 457.3 | 0.018781 | 0.062217 | 0.301862 | 0.762757 | -0.10316 | 0.140725 | AFR | Encounter for lon | circulatory system |
| 458 | 0.085933 | 0.074852 | 1.148042 | 0.250951 | -0.06077 | 0.23264 | AFR | Hypotension | circulatory system |
| 458.1 | -0.12571 | 0.134591 | -0.934 | 0.350302 | -0.3895 | 0.138085 | AFR | Orthostatic hypot | circulatory system |
| 458.2 | 0.022547 | 0.132331 | 0.17038 | 0.864711 | -0.23682 | 0.28191 | AFR | Iatrogenic hypote | circulatory system |
| 458.9 | 0.141443 | 0.083882 | 1.686227 | 0.091752 | -0.02296 | 0.305848 | AFR | Hypotension NOS | circulatory system |
| 459 | -0.00984 | 0.067671 | -0.14548 | 0.884334 | -0.14248 | 0.122787 | AFR | Other disorders c | circulatory system |
| 459.1 | -0.2254 | 0.163327 | -1.38006 | 0.167567 | -0.54552 | 0.094714 | AFR | Hemorrhage NOS | circulatory system |
| 459.7 | -0.0627 | 0.11175 | -0.56104 | 0.574772 | -0.28172 | 0.15633 | AFR | Blood vessel repl | circulatory system |
| 459.9 | 0.025379 | 0.075602 | 0.335694 | 0.737102 | -0.1228 | 0.173557 | AFR | Circulatory disea | circulatory system |
| 464 | -0.04715 | 0.084922 | -0.55518 | 0.578772 | -0.21359 | 0.119297 | AFR | Acute sinusitis | respiratory |
| 465 | 0.045915 | 0.058302 | 0.787536 | 0.430968 | -0.06835 | 0.160183 | AFR | Acute upper resp | respiratory |
| 465.2 | -0.00167 | 0.077484 | -0.02156 | 0.982795 | -0.15354 | 0.150194 | AFR | Acute pharyngitis | respiratory |
| 465.4 | 0.212758 | 0.186109 | 1.14319 | 0.25296 | -0.15201 | 0.577524 | AFR | Acute laryngitis a | respiratory |
| 470 | -0.06207 | 0.123726 | -0.50169 | 0.615884 | -0.30457 | 0.180427 | AFR | Septal Deviations | respiratory |

| | | | | | | | | | | | |
|---|---|---|---|---|---|---|---|---|---|---|---|
| 471 | 0.061081 | 0.194552 | 0.313955 | 0.753555 | -0.32023 | 0.442396 | AFR | | Nasal polyps | respiratory | |
| 472 | -0.00056 | 0.094454 | -0.00595 | 0.995255 | -0.18569 | 0.184564 | AFR | | Chronic pharyngi | respiratory | |
| 473 | -0.06403 | 0.091583 | -0.69917 | 0.484445 | -0.24353 | 0.115467 | AFR | | Diseases of the la | respiratory | |
| 473.1 | 0.458894 | 0.377439 | 1.215809 | 0.224058 | -0.28087 | 1.198661 | AFR | | Chronic laryngitis | respiratory | |
| 473.3 | -0.11633 | 0.199493 | -0.58312 | 0.559811 | -0.50733 | 0.27467 | AFR | | Paralysis/spasm | respiratory | |
| 473.4 | -0.00372 | 0.103946 | -0.03583 | 0.97142 | -0.20745 | 0.200006 | AFR | | Voice disturbance | respiratory | |
| 474 | -0.06307 | 0.204896 | -0.30781 | 0.758228 | -0.46466 | 0.33852 | AFR | | Acute and chroni | respiratory | |
| 474.1 | 2.69E-02 | 0.277705 | 0.096745 | 0.922929 | -0.51743 | 0.571158 | AFR | | Acute tonsillitis | respiratory | |
| 474.2 | -0.31369 | 0.260255 | -1.2053 | 0.228087 | -0.82378 | 0.196404 | AFR | | Chronic tonsillitis | respiratory | |
| 475 | -0.03189 | 0.069737 | -0.45732 | 0.647443 | -0.16857 | 0.10479 | AFR | | Chronic sinusitis | respiratory | |
| 475.9 | -0.12451 | 0.086398 | -1.44112 | 0.149551 | -0.29385 | 0.044827 | AFR | | Postnasal drip | respiratory | |
| 476 | -0.09 | 0.062432 | -1.44153 | 0.149434 | -0.21236 | 0.032367 | AFR | | Allergic rhinitis | respiratory | |
| 477 | 0.220392 | 0.116107 | 1.89818 | 0.057672 | -0.00717 | 0.447957 | AFR | | Epistaxis or throa | respiratory | |
| 478 | 0.027699 | 0.139752 | 0.198199 | 0.842889 | -0.24621 | 0.301607 | AFR | | Throat pain | respiratory | |
| 479 | -0.0109 | 0.065507 | -0.16643 | 0.867816 | -0.13929 | 0.117489 | AFR | | Other upper resp | respiratory | |
| 480 | 0.066008 | 0.065525 | 1.007375 | 0.313755 | -0.06242 | 0.194434 | AFR | | Pneumonia | respiratory | |
| 480.1 | -0.04001 | 0.108781 | -0.3678 | 0.713025 | -0.25322 | 0.173197 | AFR | | Bacterial pneumo | respiratory | |
| 480.11 | 0.155323 | 0.156037 | 0.995425 | 0.31953 | -0.1505 | 0.46115 | AFR | | Pneumococcal p | respiratory | |
| 480.12 | -0.27406 | 0.318248 | -0.86116 | 0.389148 | -0.89782 | 0.349691 | AFR | | Pseudomonal pn | respiratory | |
| 480.13 | -0.65641 | 0.459938 | -1.42718 | 0.153529 | -1.55787 | 0.245048 | AFR | | MRSA pneumoni | respiratory | |
| 480.2 | 0.221083 | 0.115819 | 1.908862 | 0.05628 | -0.00592 | 0.448084 | AFR | | Viral pneumonia | respiratory | |
| 480.3 | -0.34051 | 0.257943 | -1.32011 | 0.186799 | -0.84607 | 0.165046 | AFR | | Pneumonia due t | respiratory | |
| 480.5 | 0.011591 | 0.249196 | 0.046513 | 0.962902 | -0.47682 | 0.500006 | AFR | | Bronchopneumo | respiratory | |
| 481 | -0.00073 | 0.126856 | -0.00575 | 0.995415 | -0.24936 | 0.247904 | AFR | | Influenza | respiratory | |
| 483 | 0.070409 | 0.103977 | 0.677163 | 0.498303 | -0.13338 | 0.274199 | AFR | | Acute bronchitis a | respiratory | |
| 495 | 0.091517 | 0.062411 | 1.466375 | 0.142546 | -0.03081 | 0.21384 | AFR | | Asthma | respiratory | |
| 495.1 | 0.187402 | 0.156274 | 1.199189 | 0.230455 | -0.11889 | 0.493693 | AFR | | Chronic obstructi | respiratory | |
| 495.11 | 0.461915 | 0.297892 | 1.550616 | 0.120994 | -0.12194 | 1.045772 | AFR | | Chronic obstructi | respiratory | |
| 495.2 | 0.078172 | 0.098237 | 0.79575 | 0.426178 | -0.11437 | 0.270714 | AFR | | Asthma with exa | respiratory | |
| 496 | 0.185335 | 0.074028 | 2.503574 | 0.012295 | 0.040242 | 0.330427 | AFR | | Chronic airway o | respiratory | |
| 496.1 | 0.185169 | 0.112081 | 1.652096 | 0.098515 | -0.03451 | 0.404844 | AFR | | Emphysema | respiratory | |
| 496.2 | 0.229101 | 0.118604 | 1.931648 | 0.053403 | -0.00336 | 0.461561 | AFR | | Chronic bronchiti | respiratory | |
| 496.21 | 0.2657 | 0.140329 | 1.893413 | 0.058303 | -0.00934 | 0.54074 | AFR | | Obstructive chro | respiratory | |
| 496.3 | 0.053623 | 0.120503 | 0.444992 | 0.656326 | -0.18256 | 0.289804 | AFR | | Bronchiectasis | respiratory | |
| 497 | 0.175601 | 0.103226 | 1.701129 | 0.088919 | -0.02672 | 0.37792 | AFR | | Bronchitis | respiratory | |
| 498 | 0.087643 | 0.222394 | 0.394092 | 0.693513 | -0.34824 | 0.523527 | AFR | | Acute bronchosp | respiratory | |
| 499 | -0.06903 | 1.191288 | -0.05794 | 0.953793 | -2.40391 | 2.265853 | AFR | | Cystic fibrosis | respiratory | |
| 500 | -0.36406 | 0.249711 | -1.45792 | 0.144862 | -0.85349 | 0.125365 | AFR | | Lung disease du | respiratory | |

| | | | | | | | | | | |
|---|---|---|---|---|---|---|---|---|---|---|
| 500.1 | -0.52697 | 0.439307 | -1.19954 | 0.230316 | -1.38799 | 0.334058 | AFR | Extrinsic allergic | respiratory | |
| 500.2 | -0.47139 | 0.646153 | -0.72954 | 0.465671 | -1.73783 | 0.795042 | AFR | Pneumoconiosis | respiratory | |
| 501 | -0.01402 | 0.126985 | -0.11045 | 0.912056 | -0.26291 | 0.234861 | AFR | Pneumonitis due | respiratory | |
| 502 | 0.018853 | 0.11419 | 0.165101 | 0.868864 | -0.20495 | 0.24266 | AFR | Postinflammatory | respiratory | |
| 503 | -0.03195 | 0.082536 | -0.38705 | 0.698721 | -0.19371 | 0.129822 | AFR | Pulmonary conge | respiratory | |
| 504 | 0.016186 | 0.140542 | 0.115167 | 0.908313 | -0.25927 | 0.291644 | AFR | Other alveolar ar | respiratory | |
| 504.1 | -0.02976 | 0.183525 | -0.16217 | 0.871175 | -0.38946 | 0.32994 | AFR | Idiopathic fibrosir | respiratory | |
| 505 | 0.148637 | 0.174152 | 0.853489 | 0.393388 | -0.19269 | 0.489968 | AFR | Other pulmonary | respiratory | |
| 506 | -0.10618 | 0.118263 | -0.89787 | 0.369256 | -0.33798 | 0.125607 | AFR | Empyema and pr | respiratory | |
| 507 | -0.04313 | 0.066459 | -0.64903 | 0.516318 | -0.17339 | 0.087124 | AFR | Pleurisy; pleural | respiratory | |
| 508 | 0.101259 | 0.060771 | 1.666233 | 0.095667 | -0.01785 | 0.220368 | AFR | Pulmonary collap | respiratory | |
| 509 | 0.12166 | 0.073967 | 1.644773 | 0.100017 | -0.02331 | 0.266633 | AFR | Respiratory failur | respiratory | |
| 509.1 | 0.074917 | 0.075352 | 0.994226 | 0.320113 | -0.07277 | 0.222604 | AFR | Respiratory failur | respiratory | |
| 509.2 | 0.275844 | 0.185029 | 1.490815 | 0.13601 | -0.08681 | 0.638494 | AFR | Respiratory insuf | respiratory | |
| 509.3 | 0.215533 | 0.226018 | 0.95361 | 0.340281 | -0.22745 | 0.658519 | AFR | Pulmonary insuff | respiratory | |
| 509.5 | -0.17034 | 0.668612 | -0.25476 | 0.798906 | -1.48079 | 1.140117 | AFR | Respiratory arres | respiratory | |
| 509.8 | -0.01146 | 0.128459 | -0.08924 | 0.92889 | -0.26324 | 0.240311 | AFR | Dependence on | respiratory | |
| 510 | 0.038063 | 0.073917 | 0.514944 | 0.606592 | -0.10681 | 0.182938 | AFR | Other diseases o | respiratory | |
| 510.2 | -0.18066 | 0.191669 | -0.94255 | 0.345911 | -0.55632 | 0.195007 | AFR | Lung transplant | respiratory | |
| 512 | 0.057976 | 0.055928 | 1.036613 | 0.299916 | -0.05164 | 0.167593 | AFR | Other symptoms | respiratory | |
| 512.1 | 0.005763 | 0.098189 | 0.05869 | 0.953199 | -0.18668 | 0.198209 | AFR | Wheezing | respiratory | |
| 512.2 | 0.034945 | 0.089419 | 0.390796 | 0.695948 | -0.14031 | 0.210203 | AFR | Painful respiratio | respiratory | |
| 512.3 | 0.417876 | 0.293944 | 1.421615 | 0.155138 | -0.15824 | 0.993996 | AFR | Abnormal chest s | respiratory | |
| 512.7 | 0.063013 | 0.054791 | 1.150075 | 0.250113 | -0.04437 | 0.170401 | AFR | Shortness of brea | respiratory | |
| 512.8 | 0.027481 | 0.055254 | 0.497352 | 0.618941 | -0.08082 | 0.135777 | AFR | Cough | respiratory | |
| 512.9 | 0.063709 | 0.056487 | 1.12784 | 0.259388 | -0.047 | 0.174422 | AFR | Other dyspnea | respiratory | |
| 513 | 0.06571 | 0.089356 | 0.735377 | 0.46211 | -0.10942 | 0.240845 | AFR | Respiratory abnc | respiratory | |
| 513.3 | -0.10323 | 0.145366 | -0.71015 | 0.477612 | -0.38814 | 0.18168 | AFR | Hypoventilation | respiratory | |
| 513.31 | -0.25025 | 0.216002 | -1.15855 | 0.24664 | -0.67361 | 0.173107 | AFR | Apnea | respiratory | |
| 513.32 | -0.04122 | 0.266376 | -0.15473 | 0.877035 | -0.5633 | 0.480871 | AFR | Orthopnea | respiratory | |
| 513.4 | 0.084919 | 0.277962 | 0.305507 | 0.75998 | -0.45988 | 0.629716 | AFR | Hyperventilation | respiratory | |
| 513.8 | 0.157933 | 0.112557 | 1.403137 | 0.160576 | -0.06267 | 0.37854 | AFR | Disorders of diap | respiratory | |
| 514 | -0.00223 | 0.05682 | -0.0393 | 0.968648 | -0.1136 | 0.109131 | AFR | Abnormal finding | respiratory | |
| 514.1 | -0.05323 | 0.164825 | -0.32292 | 0.746754 | -0.37628 | 0.269825 | AFR | Abnormal results | respiratory | |
| 514.2 | 0.082482 | 0.082663 | 0.997812 | 0.318371 | -0.07953 | 0.244499 | AFR | Solitary pulmona | respiratory | |
| 516 | 0.111716 | 0.139492 | 0.800875 | 0.423204 | -0.16168 | 0.385116 | AFR | Abnormal sputun | respiratory | |
| 516.1 | 0.113197 | 0.149457 | 0.75739 | 0.448816 | -0.17973 | 0.406128 | AFR | Hemoptysis | respiratory | |
| 519 | -0.00136 | 0.074411 | -0.01824 | 0.985448 | -0.1472 | 0.144485 | AFR | Other diseases o | respiratory | |

| | | | | | | | | | | |
|---|---|---|---|---|---|---|---|---|---|---|
| 519.1 | -0.61371 | 0.344968 | -1.77903 | 0.075235 | -1.28983 | 0.062417 | AFR | Tracheostomy cc | respiratory | |
| 519.2 | 0.28924 | 0.254648 | 1.135845 | 0.256021 | -0.20986 | 0.78834 | AFR | Respiratory comp | respiratory | |
| 519.8 | 0.12173 | 0.099065 | 1.228798 | 0.219148 | -0.07243 | 0.315894 | AFR | Other diseases o | respiratory | |
| 519.9 | -0.08754 | 0.102839 | -0.85121 | 0.394655 | -0.2891 | 0.114024 | AFR | Symptoms involv | respiratory | |
| 520 | 0.178822 | 0.43028 | 0.415594 | 0.677707 | -0.66451 | 1.022156 | AFR | Disorders of tootl | digestive | |
| 520.1 | -0.31501 | 0.519029 | -0.60692 | 0.543906 | -1.33229 | 0.702271 | AFR | Hereditary disturl | digestive | |
| 520.2 | 1.547683 | 0.81686 | 1.894673 | 0.058136 | -0.05333 | 3.148699 | AFR | Disturbances in t | digestive | |
| 521 | 0.048536 | 0.164279 | 0.295449 | 0.767651 | -0.27345 | 0.370518 | AFR | Diseases of hard | digestive | |
| 521.1 | 0.040421 | 0.16538 | 0.24441 | 0.806913 | -0.28372 | 0.36456 | AFR | Dental caries | digestive | |
| 521.2 | 3.995919 | 2.051091 | 1.948192 | 0.051392 | -0.02415 | 8.015984 | AFR | Dental abrasion, | digestive | |
| 522 | 0.033626 | 0.18047 | 0.186328 | 0.852188 | -0.32009 | 0.387341 | AFR | Diseases of pulp | digestive | |
| 522.5 | 0.033626 | 0.18047 | 0.186328 | 0.852188 | -0.32009 | 0.387341 | AFR | Periapical absces | digestive | |
| 523 | 0.014749 | 0.140963 | 0.104628 | 0.916671 | -0.26153 | 0.291031 | AFR | Gingival and peri | digestive | |
| 523.1 | 0.026908 | 0.223287 | 0.120509 | 0.90408 | -0.41073 | 0.464543 | AFR | Gingivitis | digestive | |
| 523.3 | 0.062803 | 0.188362 | 0.333417 | 0.73882 | -0.30638 | 0.431986 | AFR | Periodontitis (acu | digestive | |
| 523.31 | 0.062803 | 0.188362 | 0.333417 | 0.73882 | -0.30638 | 0.431986 | AFR | Acute periodontit | digestive | |
| 524 | -0.82484 | 0.387927 | -2.12629 | 0.033479 | -1.58517 | -0.06452 | AFR | Dentofacial anom | digestive | |
| 524.3 | -0.77213 | 0.501075 | -1.54096 | 0.123327 | -1.75422 | 0.209953 | AFR | Anomalies of too | digestive | |
| 525 | 0.153284 | 0.157111 | 0.975643 | 0.329241 | -0.15465 | 0.461215 | AFR | Other diseases o | digestive | |
| 525.1 | 0.006615 | 0.34682 | 0.019073 | 0.984783 | -0.67314 | 0.68637 | AFR | Loss of teeth or e | digestive | |
| 525.2 | -0.30808 | 0.945264 | -0.32592 | 0.744486 | -2.16076 | 1.544604 | AFR | Atrophy of edent | digestive | |
| 526 | 0.015441 | 0.134513 | 0.114794 | 0.908609 | -0.2482 | 0.279082 | AFR | Diseases of the j | digestive | |
| 526.1 | -0.30808 | 0.945264 | -0.32592 | 0.744486 | -2.16076 | 1.544604 | AFR | Cysts of the jaws | digestive | |
| 526.3 | -0.07578 | 0.81976 | -0.09244 | 0.926351 | -1.68248 | 1.530923 | AFR | Anomalies of jaw | digestive | |
| 526.4 | 0.026662 | 0.170631 | 0.156257 | 0.875831 | -0.30777 | 0.361092 | AFR | Temporomandibu | digestive | |
| 526.41 | 0.206298 | 0.244584 | 0.843468 | 0.398967 | -0.27308 | 0.685674 | AFR | Temporomandibu | digestive | |
| 526.42 | -0.09464 | 0.267115 | -0.35432 | 0.723097 | -0.61818 | 0.428891 | AFR | Arthralgia/ankylo | digestive | |
| 526.5 | 0.446389 | 0.556525 | 0.8021 | 0.422495 | -0.64438 | 1.537158 | AFR | Inflammatory cor | digestive | |
| 526.8 | -0.31991 | 0.467155 | -0.68481 | 0.493464 | -1.23552 | 0.595695 | AFR | Exostosis of jaw | digestive | |
| 526.9 | -0.37593 | 0.373438 | -1.00668 | 0.314088 | -1.10786 | 0.355992 | AFR | Jaw disease NOS | digestive | |
| 527 | 0.185248 | 0.129633 | 1.429023 | 0.152998 | -0.06883 | 0.439324 | AFR | Diseases of the s | digestive | |
| 527.1 | 0.091338 | 0.347649 | 0.262732 | 0.792758 | -0.59004 | 0.772717 | AFR | Hypertrophy of sa | digestive | |
| 527.2 | -0.03158 | 0.224403 | -0.14073 | 0.888086 | -0.4714 | 0.408243 | AFR | Sialoadenitis | digestive | |
| 527.7 | 0.225782 | 0.180999 | 1.247421 | 0.212243 | -0.12897 | 0.580533 | AFR | Disturbance of sa | digestive | |
| 527.8 | 0.192865 | 0.26244 | 0.734892 | 0.462405 | -0.32151 | 0.707238 | AFR | Other specified d | digestive | |
| 528 | -0.04871 | 0.106913 | -0.4556 | 0.648677 | -0.25826 | 0.160837 | AFR | Diseases of the c | digestive | |
| 528.1 | 0.085692 | 0.16802 | 0.510008 | 0.610046 | -0.24362 | 0.415004 | AFR | Stomatitis and m | digestive | |
| 528.11 | 0.011036 | 0.279154 | 0.039533 | 0.968466 | -0.5361 | 0.558167 | AFR | Stomatitis and m | digestive | |

| | | | | | | | | | | |
|---|---|---|---|---|---|---|---|---|---|---|
| 528.12 | 0.023833 | 0.29826 | 0.079906 | 0.936312 | -0.56075 | 0.608411 | AFR | Oral aphthae | digestive | |
| 528.3 | 0.151312 | 0.379275 | 0.398949 | 0.689931 | -0.59205 | 0.894677 | AFR | Cellulitis and abs | digestive | |
| 528.4 | -0.36549 | 0.585974 | -0.62373 | 0.532804 | -1.51398 | 0.782997 | AFR | Cysts of oral soft | digestive | |
| 528.41 | -0.36549 | 0.585974 | -0.62373 | 0.532804 | -1.51398 | 0.782997 | AFR | Cyst of the saliva | digestive | |
| 528.5 | -0.12204 | 0.227887 | -0.53553 | 0.592284 | -0.56869 | 0.324611 | AFR | Diseases of lips | digestive | |
| 528.6 | 0.071854 | 0.424931 | 0.169095 | 0.865722 | -0.761 | 0.904703 | AFR | Leukoplakia of or | digestive | |
| 528.7 | -0.38321 | 0.319078 | -1.20099 | 0.229755 | -1.00859 | 0.242172 | AFR | Sialolithiasis | digestive | |
| 529 | 0.144383 | 0.192704 | 0.749248 | 0.453707 | -0.23331 | 0.522077 | AFR | Diseases and oth | digestive | |
| 529.1 | 0.007254 | 0.385246 | 0.01883 | 0.984977 | -0.74781 | 0.762322 | AFR | Glossitis | digestive | |
| 529.6 | 0.314132 | 0.346971 | 0.905354 | 0.365278 | -0.36592 | 0.994183 | AFR | Glossodynia | digestive | |
| 530 | 0.103904 | 0.054795 | 1.896231 | 0.057929 | -0.00349 | 0.2113 | AFR | Diseases of esop | digestive | |
| 530.1 | 0.094645 | 0.054894 | 1.724134 | 0.084684 | -0.01295 | 0.202237 | AFR | Esophagitis, GEF | digestive | |
| 530.11 | 0.076964 | 0.054984 | 1.399747 | 0.161589 | -0.0308 | 0.184731 | AFR | GERD | digestive | |
| 530.12 | 0.316085 | 0.172356 | 1.833909 | 0.066668 | -0.02173 | 0.653897 | AFR | Ulcer of esophag | digestive | |
| 530.13 | 0.159717 | 0.341988 | 0.467026 | 0.640481 | -0.51057 | 0.830001 | AFR | Barrett's esophag | digestive | |
| 530.14 | 0.159674 | 0.112951 | 1.413655 | 0.157463 | -0.06171 | 0.381055 | AFR | Reflux esophagit | digestive | |
| 530.15 | 0.132312 | 0.747935 | 0.176903 | 0.859585 | -1.33361 | 1.598238 | AFR | Eosinophilic esop | digestive | |
| 530.2 | 0.012292 | 0.123437 | 0.09958 | 0.920678 | -0.22964 | 0.254223 | AFR | Esophageal blee | digestive | |
| 530.3 | 0.268143 | 0.190069 | 1.410761 | 0.158315 | -0.10439 | 0.640672 | AFR | Stricture and ster | digestive | |
| 530.5 | 0.242253 | 0.148076 | 1.636 | 0.10184 | -0.04797 | 0.532477 | AFR | Disorders of esop | digestive | |
| 530.6 | 0.659855 | 0.820931 | 0.803789 | 0.421519 | -0.94914 | 2.268851 | AFR | Diverticulum of e | digestive | |
| 530.7 | 0.310351 | 0.393549 | 0.788597 | 0.430347 | -0.46099 | 1.081693 | AFR | Gastroesophagea | digestive | |
| 530.9 | 0.029414 | 0.151934 | 0.193599 | 0.84649 | -0.26837 | 0.3272 | AFR | Heartburn | digestive | |
| 531 | 0.122207 | 0.098013 | 1.246839 | 0.212457 | -0.0699 | 0.314309 | AFR | Peptic ulcer (excl | digestive | |
| 531.1 | -0.10625 | 0.207309 | -0.5125 | 0.608304 | -0.51256 | 0.300074 | AFR | Hemorrhage fron | digestive | |
| 531.2 | 0.118316 | 0.136918 | 0.864133 | 0.387515 | -0.15004 | 0.386671 | AFR | Gastric ulcer | digestive | |
| 531.3 | 0.161536 | 0.254297 | 0.635227 | 0.52528 | -0.33688 | 0.659949 | AFR | Duodenal ulcer | digestive | |
| 531.4 | 0.04178 | 0.12214 | 0.342069 | 0.732299 | -0.19761 | 0.281171 | AFR | Peptic ulcer, site | digestive | |
| 531.5 | -1.38861 | 0.400387 | -3.46815 | 0.000524 | -2.17335 | -0.60386 | AFR | Gastrojejunal ulc | digestive | |
| 532 | 0.000396 | 0.072108 | 0.005485 | 0.995624 | -0.14093 | 0.141725 | AFR | Dysphagia | digestive | |
| 535 | 0.013137 | 0.074653 | 0.175979 | 0.86031 | -0.13318 | 0.159455 | AFR | Gastritis and duo | digestive | |
| 535.1 | -0.06735 | 0.208204 | -0.3235 | 0.74632 | -0.47543 | 0.340719 | AFR | Acute gastritis | digestive | |
| 535.2 | 0.067612 | 0.094352 | 0.716598 | 0.473622 | -0.11731 | 0.252539 | AFR | Atrophic gastritis | digestive | |
| 535.6 | -0.14952 | 0.267861 | -0.5582 | 0.576711 | -0.67452 | 0.375479 | AFR | Duodenitis | digestive | |
| 535.8 | -0.00854 | 0.142891 | -0.05978 | 0.952328 | -0.2886 | 0.271518 | AFR | Other specified g | digestive | |
| 535.9 | 0.117154 | 0.10705 | 1.094382 | 0.273787 | -0.09266 | 0.326968 | AFR | Gastritis and duo | digestive | |
| 536 | 0.098211 | 0.080236 | 1.22402 | 0.220945 | -0.05905 | 0.255471 | AFR | Disorders of func | digestive | |
| 536.3 | 0.10615 | 0.137733 | 0.770698 | 0.440886 | -0.1638 | 0.376101 | AFR | Gastroparesis | digestive | |

| | | | | | | | | | | | |
|---|---|---|---|---|---|---|---|---|---|---|---|
| 536.7 | 0.062685 | 0.216379 | 0.289699 | 0.772047 | -0.36141 | 0.48678 | AFR | | Complications of | digestive | |
| 536.8 | 0.039355 | 0.092975 | 0.423283 | 0.672089 | -0.14287 | 0.221582 | AFR | | Dyspepsia and o | digestive | |
| 537 | 0.148926 | 0.077442 | 1.923053 | 0.054473 | -0.00286 | 0.30071 | AFR | | Other disorders c | digestive | |
| 537.1 | -0.21208 | 0.262872 | -0.8068 | 0.419784 | -0.7273 | 0.303136 | AFR | | Lesions of stoma | digestive | |
| 539 | 0.133687 | 0.114105 | 1.171613 | 0.241353 | -0.08996 | 0.357329 | AFR | | Bariatric surgery | digestive | |
| 540 | 0.104947 | 0.227393 | 0.461522 | 0.644424 | -0.34074 | 0.550629 | AFR | | Appendiceal con | digestive | |
| 540.1 | 0.15629 | 0.247439 | 0.63163 | 0.527628 | -0.32868 | 0.641262 | AFR | | Appendicitis | digestive | |
| 540.11 | -0.31999 | 0.531873 | -0.60162 | 0.547424 | -1.36244 | 0.722464 | AFR | | Acute appendiciti | digestive | |
| 550 | 0.030348 | 0.065062 | 0.466453 | 0.640891 | -0.09717 | 0.157868 | AFR | | Abdominal hernia | digestive | |
| 550.1 | -0.0269 | 0.150551 | -0.17865 | 0.858212 | -0.32197 | 0.268178 | AFR | | Inguinal hernia | digestive | |
| 550.2 | 0.13239 | 0.079232 | 1.670927 | 0.094736 | -0.0229 | 0.287682 | AFR | | Diaphragmatic he | digestive | |
| 550.3 | -0.0635 | 0.498103 | -0.12749 | 0.898551 | -1.03977 | 0.91276 | AFR | | Femoral hernia | digestive | |
| 550.4 | 0.04205 | 0.131155 | 0.320614 | 0.748503 | -0.21501 | 0.299108 | AFR | | Umbilical hernia | digestive | |
| 550.5 | -0.13131 | 0.113378 | -1.15817 | 0.246793 | -0.35353 | 0.090905 | AFR | | Ventral hernia | digestive | |
| 550.6 | -0.08593 | 0.177528 | -0.48403 | 0.628362 | -0.43388 | 0.262018 | AFR | | Incisional hernia | digestive | |
| 555 | -0.06001 | 0.150084 | -0.39982 | 0.689292 | -0.35417 | 0.234154 | AFR | | Inflammatory bov | digestive | |
| 555.1 | -0.01378 | 0.213144 | -0.06466 | 0.948444 | -0.43154 | 0.403972 | AFR | | Regional enteritis | digestive | |
| 555.2 | -0.00962 | 0.168001 | -0.05723 | 0.954359 | -0.33889 | 0.31966 | AFR | | Ulcerative colitis | digestive | |
| 555.21 | -0.01293 | 0.218853 | -0.05908 | 0.952885 | -0.44188 | 0.416014 | AFR | | Ulcerative colitis | digestive | |
| 556 | 0.033755 | 0.144944 | 0.232881 | 0.815854 | -0.25033 | 0.317839 | AFR | | Ulceration of the | digestive | |
| 556.1 | 0.004226 | 0.153699 | 0.027495 | 0.978065 | -0.29702 | 0.30547 | AFR | | Ulceration of inte | digestive | |
| 556.11 | -0.08282 | 0.253623 | -0.32657 | 0.743995 | -0.57992 | 0.414267 | AFR | | Angiodysplasia o | digestive | |
| 557 | 0.223479 | 0.19086 | 1.170907 | 0.241636 | -0.1506 | 0.597557 | AFR | | Intestinal malabs | digestive | |
| 557.1 | 0.548653 | 0.375968 | 1.459306 | 0.144481 | -0.18823 | 1.285537 | AFR | | Celiac disease | digestive | |
| 558 | 0.001348 | 0.084562 | 0.015939 | 0.987283 | -0.16439 | 0.167086 | AFR | | Noninfectious ga | digestive | |
| 559 | 0.448796 | 0.275551 | 1.628725 | 0.103371 | -0.09127 | 0.988865 | AFR | | Ileostomy status | digestive | |
| 560 | 0.042262 | 0.089406 | 0.472699 | 0.636428 | -0.13297 | 0.217494 | AFR | | Intestinal obstruc | digestive | |
| 560.1 | -0.00341 | 0.120162 | -0.02838 | 0.977363 | -0.23892 | 0.232104 | AFR | | Paralytic ileus | digestive | |
| 560.2 | 0.176138 | 0.171068 | 1.029637 | 0.30318 | -0.15915 | 0.511425 | AFR | | Impaction of intes | digestive | |
| 560.3 | 0.037665 | 0.304415 | 0.123729 | 0.90153 | -0.55898 | 0.634307 | AFR | | Peritoneal or inte | digestive | |
| 560.4 | 0.071198 | 0.127933 | 0.55653 | 0.577849 | -0.17954 | 0.321941 | AFR | | Other intestinal o | digestive | |
| 561 | 0.082155 | 0.055019 | 1.493204 | 0.135384 | -0.02568 | 0.18999 | AFR | | Symptoms involv | digestive | |
| 561.1 | 0.02351 | 0.062163 | 0.378209 | 0.705276 | -0.09833 | 0.145347 | AFR | | Diarrhea | digestive | |
| 561.2 | 0.091018 | 0.067888 | 1.340705 | 0.180016 | -0.04204 | 0.224075 | AFR | | Flatulence | digestive | |
| 562 | 0.016549 | 0.068339 | 0.242166 | 0.808652 | -0.11739 | 0.15049 | AFR | | Diverticulosis and | digestive | |
| 562.1 | 0.013004 | 0.069125 | 0.188119 | 0.850783 | -0.12248 | 0.148486 | AFR | | Diverticulosis | digestive | |
| 562.2 | 0.052273 | 0.151518 | 0.344994 | 0.730099 | -0.2447 | 0.349242 | AFR | | Diverticulitis | digestive | |
| 563 | 0.141943 | 0.059269 | 2.394899 | 0.016625 | 0.025778 | 0.258108 | AFR | | Constipation | digestive | |

| | | | | | | | | | | |
|---|---|---|---|---|---|---|---|---|---|---|
| 564 | 0.0813 | 0.063671 | 1.276869 | 0.201649 | -0.04349 | 0.206094 | AFR | Functional digest | digestive | |
| 564.1 | 0.109566 | 0.117218 | 0.934717 | 0.349934 | -0.12018 | 0.33931 | AFR | Irritable Bowel Sy | digestive | |
| 564.8 | 0.039908 | 0.112118 | 0.355944 | 0.721883 | -0.17984 | 0.259654 | AFR | Abnormal finding | digestive | |
| 564.9 | 0.09139 | 0.077513 | 1.179025 | 0.238388 | -0.06053 | 0.243312 | AFR | Personal history | digestive | |
| 565 | -0.13403 | 0.081316 | -1.64821 | 0.09931 | -0.2934 | 0.025351 | AFR | Anal and rectal c | digestive | |
| 565.1 | -0.31884 | 0.122706 | -2.59838 | 0.009366 | -0.55934 | -0.07834 | AFR | Anal and rectal p | digestive | |
| 567 | -0.03597 | 0.154591 | -0.23269 | 0.816004 | -0.33896 | 0.267022 | AFR | Peritonitis and re | digestive | |
| 568 | -0.12824 | 0.09602 | -1.33558 | 0.181688 | -0.31644 | 0.059954 | AFR | Other disorders c | digestive | |
| 568.1 | -0.12962 | 0.11102 | -1.16756 | 0.242983 | -0.34722 | 0.087972 | AFR | Peritoneal adhes | digestive | |
| 569 | 0.048071 | 0.066558 | 0.722242 | 0.470146 | -0.08238 | 0.178521 | AFR | Other disorders c | digestive | |
| 569.1 | 0.422554 | 0.214407 | 1.970806 | 0.048746 | 0.002325 | 0.842784 | AFR | Toxic gastroenter | digestive | |
| 569.2 | 0.176743 | 0.147242 | 1.200357 | 0.230001 | -0.11185 | 0.465333 | AFR | Gastrointestinal c | digestive | |
| 571 | 0.049703 | 0.068741 | 0.723046 | 0.469652 | -0.08503 | 0.184433 | AFR | Chronic liver dise | digestive | |
| 571.5 | 0.067367 | 0.069999 | 0.962399 | 0.335849 | -0.06983 | 0.204563 | AFR | Other chronic no | digestive | |
| 571.51 | 0.083491 | 0.138999 | 0.60066 | 0.548066 | -0.18894 | 0.355924 | AFR | Cirrhosis of liver | digestive | |
| 571.6 | -0.29685 | 0.478626 | -0.62021 | 0.535117 | -1.23494 | 0.64124 | AFR | Primary biliary ci | digestive | |
| 571.8 | -0.00092 | 0.146111 | -0.00633 | 0.994952 | -0.2873 | 0.285447 | AFR | Liver abscess an | digestive | |
| 571.81 | 0.213125 | 0.181825 | 1.172142 | 0.24114 | -0.14325 | 0.569496 | AFR | Portal hypertensi | digestive | |
| 572 | -0.00542 | 0.102645 | -0.05278 | 0.957905 | -0.2066 | 0.195762 | AFR | Ascites (non mal | digestive | |
| 573 | 0.021999 | 0.076147 | 0.288896 | 0.772661 | -0.12725 | 0.171245 | AFR | Other disorders c | digestive | |
| 573.1 | 0.416379 | 0.258362 | 1.611607 | 0.107047 | -0.09 | 0.922759 | AFR | Chronic passive | digestive | |
| 573.2 | 0.161924 | 0.182913 | 0.88525 | 0.376022 | -0.19658 | 0.520427 | AFR | Liver replaced by | digestive | |
| 573.3 | 0.018614 | 0.103623 | 0.179635 | 0.857439 | -0.18448 | 0.221711 | AFR | Hepatomegaly | digestive | |
| 573.4 | 0.108773 | 0.233177 | 0.466481 | 0.640872 | -0.34825 | 0.565791 | AFR | Acute and subac | digestive | |
| 573.5 | -0.03958 | 0.149578 | -0.26462 | 0.791302 | -0.33275 | 0.253586 | AFR | Jaundice (not of | digestive | |
| 573.6 | 0.064744 | 0.0984 | 0.657974 | 0.510555 | -0.12812 | 0.257604 | AFR | Nonspecific eleva | digestive | |
| 573.7 | -0.07449 | 0.10455 | -0.71248 | 0.476166 | -0.2794 | 0.130424 | AFR | Abnormal results | digestive | |
| 573.9 | -0.0436 | 0.076452 | -0.57036 | 0.568436 | -0.19345 | 0.106238 | AFR | Abnormal serum | digestive | |
| 574 | 0.200649 | 0.091823 | 2.185177 | 0.028876 | 0.02068 | 0.380619 | AFR | Cholelithiasis and | digestive | |
| 574.1 | 0.190274 | 0.09676 | 1.966458 | 0.049246 | 0.000628 | 0.379919 | AFR | Cholelithiasis | digestive | |
| 574.11 | -0.36967 | 0.280122 | -1.31967 | 0.186946 | -0.9187 | 0.179361 | AFR | Cholelithiasis wit | digestive | |
| 574.12 | 0.012833 | 0.19616 | 0.06542 | 0.94784 | -0.37163 | 0.3973 | AFR | Cholelithiasis wit | digestive | |
| 574.2 | 0.197091 | 0.173741 | 1.134399 | 0.256627 | -0.14343 | 0.537617 | AFR | Calculus of bile d | digestive | |
| 574.3 | 0.07007 | 0.182315 | 0.384332 | 0.700732 | -0.28726 | 0.4274 | AFR | Cholecystitis with | digestive | |
| 575 | -0.03393 | 0.094447 | -0.35922 | 0.719431 | -0.21904 | 0.151185 | AFR | Other biliary tract | digestive | |
| 575.1 | -0.14861 | 0.253495 | -0.58624 | 0.557716 | -0.64545 | 0.348233 | AFR | Cholangitis | digestive | |
| 575.2 | -0.08679 | 0.181631 | -0.47784 | 0.632764 | -0.44278 | 0.269199 | AFR | Obstruction of bil | digestive | |
| 575.6 | 0.07124 | 0.258217 | 0.275892 | 0.782631 | -0.43486 | 0.577337 | AFR | Cholesterolosis c | digestive | |

| | | | | | | | | | | |
|---|---|---|---|---|---|---|---|---|---|---|
| 575.7 | 0.108003 | 0.128356 | 0.841434 | 0.400105 | -0.14357 | 0.359576 | AFR | Other disorders c | digestive | |
| 575.8 | -0.0928 | 0.142782 | -0.64992 | 0.515745 | -0.37264 | 0.187051 | AFR | Other disorders c | digestive | |
| 575.9 | -0.024 | 0.196689 | -0.12203 | 0.902878 | -0.40951 | 0.361503 | AFR | Nonspecific abnc | digestive | |
| 577 | -0.03773 | 0.10122 | -0.37271 | 0.709361 | -0.23611 | 0.160661 | AFR | Diseases of panc | digestive | |
| 577.1 | -0.04152 | 0.158281 | -0.26234 | 0.79306 | -0.35175 | 0.268702 | AFR | Acute pancreatiti | digestive | |
| 577.2 | 0.056076 | 0.230708 | 0.243061 | 0.807958 | -0.3961 | 0.508256 | AFR | Chronic pancreat | digestive | |
| 577.3 | -0.0009 | 0.182892 | -0.00491 | 0.99608 | -0.35936 | 0.357564 | AFR | Cyst and pseudo | digestive | |
| 578 | 0.084275 | 0.072436 | 1.163445 | 0.244649 | -0.0577 | 0.226246 | AFR | Gastrointestinal h | digestive | |
| 578.1 | 0.141699 | 0.167379 | 0.846577 | 0.397231 | -0.18636 | 0.469756 | AFR | Hematemesis | digestive | |
| 578.2 | 0.154786 | 0.093135 | 1.661953 | 0.096522 | -0.02776 | 0.337328 | AFR | Blood in stool | digestive | |
| 578.8 | 0.068872 | 0.092313 | 0.746072 | 0.455624 | -0.11206 | 0.249802 | AFR | Hemorrhage of re | digestive | |
| 578.9 | -0.0142 | 0.101773 | -0.13957 | 0.888997 | -0.21368 | 0.185267 | AFR | Hemorrhage of g | digestive | |
| 579 | -0.10959 | 0.08937 | -1.2262 | 0.220124 | -0.28475 | 0.065577 | AFR | Other symptoms | digestive | |
| 579.2 | -0.13392 | 0.198419 | -0.67492 | 0.499725 | -0.52281 | 0.254977 | AFR | Splenomegaly | digestive | |
| 579.8 | -0.00647 | 0.099389 | -0.06507 | 0.94812 | -0.20127 | 0.188331 | AFR | Nonspecific abnc | digestive | |
| 580 | 0.139223 | 0.07903 | 1.761631 | 0.078132 | -0.01567 | 0.294119 | AFR | Nephritis; nephro | genitourinary | |
| 580.1 | -0.01474 | 0.190686 | -0.07732 | 0.938366 | -0.38848 | 0.358993 | AFR | Glomerulonephrit | genitourinary | |
| 580.11 | -0.15689 | 0.265355 | -0.59124 | 0.55436 | -0.67697 | 0.363198 | AFR | Proliferative glom | genitourinary | |
| 580.12 | 0.030181 | 0.21442 | 0.140756 | 0.888063 | -0.39008 | 0.450437 | AFR | Non-proliferative | genitourinary | |
| 580.13 | -0.03485 | 0.978695 | -0.03561 | 0.97159 | -1.95306 | 1.883353 | AFR | Acute glomerulor | genitourinary | |
| 580.14 | -0.20783 | 0.54154 | -0.38377 | 0.701145 | -1.26923 | 0.853569 | AFR | Chronic glomerul | genitourinary | |
| 580.2 | -0.00574 | 0.139184 | -0.04127 | 0.96708 | -0.27854 | 0.267051 | AFR | Nephrotic syndro | genitourinary | |
| 580.3 | 0.107134 | 0.084693 | 1.264973 | 0.205881 | -0.05886 | 0.273129 | AFR | Nephritis and ne| | genitourinary | |
| 580.31 | 0.140618 | 0.09901 | 1.42023 | 0.155541 | -0.05344 | 0.334674 | AFR | Nephritis and ne| | genitourinary | |
| 580.32 | 0.017473 | 0.106382 | 0.164245 | 0.869539 | -0.19103 | 0.225977 | AFR | Nephritis and ne| | genitourinary | |
| 580.4 | 0.145444 | 0.148902 | 0.976776 | 0.32868 | -0.1464 | 0.437285 | AFR | Renal sclerosis, I | genitourinary | |
| 585 | 0.011508 | 0.06019 | 0.191203 | 0.848367 | -0.10646 | 0.129478 | AFR | Renal failure | genitourinary | |
| 585.1 | 0.060607 | 0.06391 | 0.948304 | 0.342975 | -0.06466 | 0.185869 | AFR | Acute renal failur | genitourinary | |
| 585.2 | -0.00589 | 0.120834 | -0.04873 | 0.961134 | -0.24272 | 0.230942 | AFR | Renal failure NO: | genitourinary | |
| 585.3 | 0.016552 | 0.0633 | 0.261487 | 0.793717 | -0.10751 | 0.140619 | AFR | Chronic renal fail | genitourinary | |
| 585.31 | 0.021281 | 0.088354 | 0.240862 | 0.809662 | -0.15189 | 0.194452 | AFR | Renal dialysis | genitourinary | |
| 585.32 | 0.022794 | 0.089811 | 0.253799 | 0.799651 | -0.15323 | 0.19882 | AFR | End stage renal ( | genitourinary | |
| 585.33 | 0.001307 | 0.07008 | 0.018649 | 0.985121 | -0.13605 | 0.138661 | AFR | Chronic Kidney D | genitourinary | |
| 585.34 | -0.01793 | 0.099276 | -0.18062 | 0.856664 | -0.21251 | 0.176647 | AFR | Chronic Kidney D | genitourinary | |
| 585.4 | 0.106008 | 0.087076 | 1.217426 | 0.223442 | -0.06466 | 0.276673 | AFR | Chronic kidney d | genitourinary | |
| 586 | 0.055136 | 0.062923 | 0.876252 | 0.380893 | -0.06819 | 0.178463 | AFR | Other disorders c | genitourinary | |
| 586.1 | 0.241251 | 0.310742 | 0.776369 | 0.437531 | -0.36779 | 0.850294 | AFR | Anatomical abno | genitourinary | |
| 586.11 | 0.263318 | 0.347792 | 0.757114 | 0.448981 | -0.41834 | 0.944977 | AFR | Small kidney | genitourinary | |

| | | | | | | | | | | |
|---|---|---|---|---|---|---|---|---|---|---|
| 586.12 | 0.159461 | 0.509083 | 0.313231 | 0.754105 | -0.83832 | 1.157245 | AFR | Vesicoureteral re | genitourinary | |
| 586.2 | 0.066617 | 0.089966 | 0.740462 | 0.45902 | -0.10971 | 0.242948 | AFR | Cyst of kidney, ac | genitourinary | |
| 586.3 | 0.200199 | 0.361448 | 0.553882 | 0.57966 | -0.50823 | 0.908624 | AFR | Vascular disorder | genitourinary | |
| 586.4 | -0.04698 | 0.145916 | -0.32195 | 0.747492 | -0.33297 | 0.239013 | AFR | Stricture/obstruct | genitourinary | |
| 587 | 0.05676 | 0.118408 | 0.47936 | 0.631683 | -0.17532 | 0.288836 | AFR | Kidney replaced | genitourinary | |
| 588 | 0.079079 | 0.08566 | 0.923177 | 0.355915 | -0.08881 | 0.24697 | AFR | Disorders resultir | genitourinary | |
| 588.1 | 0.023326 | 0.272647 | 0.085554 | 0.931821 | -0.51105 | 0.557705 | AFR | Renal osteodystr | genitourinary | |
| 588.2 | 0.074887 | 0.086694 | 0.863806 | 0.387695 | -0.09503 | 0.244804 | AFR | Secondary hyper | genitourinary | |
| 589 | 0.032289 | 0.148091 | 0.218038 | 0.8274 | -0.25796 | 0.322543 | AFR | Abnormal results | genitourinary | |
| 590 | 0.100325 | 0.14145 | 0.709261 | 0.478162 | -0.17691 | 0.377562 | AFR | Pyelonephritis | genitourinary | |
| 591 | 0.034687 | 0.062143 | 0.558171 | 0.576728 | -0.08711 | 0.156485 | AFR | Urinary tract infe | genitourinary | |
| 592 | -0.07802 | 0.076827 | -1.01547 | 0.309883 | -0.22859 | 0.072563 | AFR | Cystitis and ureth | genitourinary | |
| 592.1 | -0.07657 | 0.078186 | -0.97938 | 0.327393 | -0.22982 | 0.076669 | AFR | Cystitis | genitourinary | |
| 592.11 | -0.06912 | 0.083569 | -0.82712 | 0.408171 | -0.23291 | 0.094671 | AFR | Acute cystitis | genitourinary | |
| 592.12 | -0.46928 | 0.377426 | -1.24337 | 0.213732 | -1.20902 | 0.270461 | AFR | Chronic cystitis | genitourinary | |
| 592.13 | 0.562923 | 0.46259 | 1.216894 | 0.223645 | -0.34374 | 1.469582 | AFR | Chronic interstitia | genitourinary | |
| 592.2 | -0.29673 | 0.278637 | -1.06494 | 0.286903 | -0.84285 | 0.249387 | AFR | Urethritis and ure | genitourinary | |
| 592.3 | -0.60708 | 0.984245 | -0.61679 | 0.53737 | -2.53616 | 1.322008 | AFR | Urethral stricture | genitourinary | |
| 593 | -0.04206 | 0.077626 | -0.54182 | 0.587943 | -0.1942 | 0.110085 | AFR | Hematuria | genitourinary | |
| 593.1 | -0.01073 | 0.124414 | -0.08627 | 0.931252 | -0.25458 | 0.233113 | AFR | Gross hematuria | genitourinary | |
| 593.2 | 0.058694 | 0.10615 | 0.552933 | 0.580309 | -0.14936 | 0.266745 | AFR | Microscopic hem | genitourinary | |
| 594 | -0.05202 | 0.096682 | -0.5381 | 0.590506 | -0.24152 | 0.137468 | AFR | Urinary calculus | genitourinary | |
| 594.1 | -0.04701 | 0.103188 | -0.45559 | 0.648684 | -0.24926 | 0.155233 | AFR | Calculus of kidne | genitourinary | |
| 594.2 | 0.025046 | 0.303394 | 0.082552 | 0.934208 | -0.5696 | 0.619688 | AFR | Calculus of lower | genitourinary | |
| 594.3 | -0.01367 | 0.255464 | -0.05352 | 0.957317 | -0.51437 | 0.487027 | AFR | Calculus of urete | genitourinary | |
| 594.8 | 0.159029 | 0.343103 | 0.463502 | 0.643005 | -0.51344 | 0.831499 | AFR | Renal colic | genitourinary | |
| 595 | 0.132712 | 0.12938 | 1.025749 | 0.30501 | -0.12087 | 0.386293 | AFR | Hydronephrosis | genitourinary | |
| 596 | -0.13206 | 0.091094 | -1.44971 | 0.14714 | -0.3106 | 0.046481 | AFR | Other disorders c | genitourinary | |
| 596.1 | -0.22259 | 0.236562 | -0.94094 | 0.346737 | -0.68624 | 0.241062 | AFR | Bladder neck obs | genitourinary | |
| 596.5 | -0.0695 | 0.110618 | -0.62829 | 0.529815 | -0.28631 | 0.147308 | AFR | Functional disord | genitourinary | |
| 597 | 0.000486 | 0.201067 | 0.002418 | 0.99807 | -0.3936 | 0.39457 | AFR | Other disorders c | genitourinary | |
| 597.1 | -0.27571 | 0.298336 | -0.92415 | 0.355406 | -0.86044 | 0.309019 | AFR | Urethral stricture | genitourinary | |
| 597.2 | -0.28239 | 0.210193 | -1.34347 | 0.17912 | -0.69436 | 0.129583 | AFR | Urinary complica | genitourinary | |
| 597.8 | 0.001649 | 0.436488 | 0.003777 | 0.996986 | -0.85385 | 0.85715 | AFR | Urethral hypermo | genitourinary | |
| 598 | -0.04397 | 0.093847 | -0.46857 | 0.639379 | -0.22791 | 0.139963 | AFR | Abnormal finding | genitourinary | |
| 598.4 | -0.27559 | 0.269155 | -1.0239 | 0.305884 | -0.80312 | 0.251947 | AFR | Other cells and c | genitourinary | |
| 598.9 | -0.02045 | 0.096901 | -0.21101 | 0.832879 | -0.21037 | 0.169475 | AFR | Other nonspecific | genitourinary | |
| 599 | 0.023383 | 0.055197 | 0.423633 | 0.671833 | -0.0848 | 0.131568 | AFR | Other symptoms/ | genitourinary | |

| | | | | | | | | | | |
|---|---|---|---|---|---|---|---|---|---|---|
| 599.1 | -0.15505 | 0.16449 | -0.9426 | 0.345888 | -0.47744 | 0.167347 | AFR | Urinary obstructio | genitourinary | |
| 599.2 | -0.04136 | 0.085421 | -0.48421 | 0.628239 | -0.20878 | 0.12606 | AFR | Retention of urine | genitourinary | |
| 599.3 | -0.04652 | 0.072396 | -0.64258 | 0.520498 | -0.18841 | 0.095374 | AFR | Dysuria | genitourinary | |
| 599.4 | 0.006283 | 0.084304 | 0.074528 | 0.94059 | -0.15895 | 0.171515 | AFR | Urinary incontine | genitourinary | |
| 599.5 | 0.044395 | 0.070418 | 0.630453 | 0.528399 | -0.09362 | 0.182411 | AFR | Frequency of urir | genitourinary | |
| 599.6 | -0.01233 | 0.166514 | -0.07407 | 0.940955 | -0.3387 | 0.314028 | AFR | Oliguria and anu | genitourinary | |
| 599.7 | -0.22807 | 0.47593 | -0.47921 | 0.631792 | -1.16087 | 0.704736 | AFR | Urethral discharg | genitourinary | |
| 599.8 | -0.13653 | 0.116573 | -1.17116 | 0.241534 | -0.365 | 0.091953 | AFR | Other symptoms | genitourinary | |
| 599.9 | -0.00832 | 0.104677 | -0.07949 | 0.93664 | -0.21348 | 0.196843 | AFR | Other abnormalit | genitourinary | |
| 600 | 0.006194 | 0.1141 | 0.054282 | 0.95671 | -0.21744 | 0.229825 | AFR | Hyperplasia of pr | genitourinary | |
| 601 | 0.161796 | 0.176925 | 0.91449 | 0.360459 | -0.18497 | 0.508562 | AFR | Inflammatory dis | genitourinary | |
| 601.1 | 0.060191 | 0.256029 | 0.235096 | 0.814134 | -0.44162 | 0.562 | AFR | Prostatitis | genitourinary | |
| 601.11 | -0.23053 | 0.350337 | -0.65802 | 0.510522 | -0.91718 | 0.456118 | AFR | Acute prostatitis | genitourinary | |
| 601.12 | 0.500721 | 0.337337 | 1.484332 | 0.137721 | -0.16045 | 1.16189 | AFR | Chronic prostatiti | genitourinary | |
| 601.3 | -0.06229 | 0.318454 | -0.19561 | 0.844918 | -0.68645 | 0.561866 | AFR | Orchitis and epid | genitourinary | |
| 601.4 | 0.511907 | 0.344423 | 1.486275 | 0.137206 | -0.16315 | 1.186963 | AFR | Balanoposthitis | genitourinary | |
| 601.8 | -0.47846 | 0.520024 | -0.92008 | 0.35753 | -1.49769 | 0.540764 | AFR | Other inflammato | genitourinary | |
| 602 | -0.39001 | 0.296919 | -1.31351 | 0.18901 | -0.97196 | 0.191943 | AFR | Other disorders c | genitourinary | |
| 602.3 | -0.30232 | 0.47794 | -0.63254 | 0.527034 | -1.23906 | 0.634429 | AFR | Dysplasia of pros | genitourinary | |
| 603 | -0.10888 | 0.218059 | -0.49932 | 0.617556 | -0.53627 | 0.318508 | AFR | Other disorders c | genitourinary | |
| 603.1 | -0.23539 | 0.289663 | -0.81263 | 0.41643 | -0.80312 | 0.33234 | AFR | Hydrocele | genitourinary | |
| 603.2 | -0.10887 | 0.343067 | -0.31734 | 0.750984 | -0.78127 | 0.56353 | AFR | Spermatocele | genitourinary | |
| 604 | -0.01402 | 0.215157 | -0.06517 | 0.948036 | -0.43572 | 0.407678 | AFR | Disorders of peni | genitourinary | |
| 604.1 | -0.63493 | 0.539494 | -1.1769 | 0.239236 | -1.69232 | 0.422459 | AFR | Redundant prepu | genitourinary | |
| 604.3 | 0.421796 | 0.423956 | 0.994904 | 0.319783 | -0.40914 | 1.252735 | AFR | Peyronie's diseas | genitourinary | |
| 605 | -0.14152 | 0.104141 | -1.35895 | 0.174162 | -0.34563 | 0.06259 | AFR | Erectile dysfuncti | genitourinary | |
| 608 | 0.087902 | 0.159132 | 0.552382 | 0.580687 | -0.22399 | 0.399794 | AFR | Other disorders c | genitourinary | |
| 609 | 0.380879 | 0.605963 | 0.628551 | 0.529643 | -0.80679 | 1.568545 | AFR | Male infertility an | genitourinary | |
| 609.1 | 0.971503 | 0.642062 | 1.513098 | 0.130255 | -0.28692 | 2.22992 | AFR | Infertility, male | genitourinary | |
| 609.2 | 0.681742 | 0.799773 | 0.85242 | 0.393981 | -0.88578 | 2.249267 | AFR | Abnormal sperma | genitourinary | |
| 610 | 0.232078 | 0.112665 | 2.059897 | 0.039408 | 0.011259 | 0.452897 | AFR | Benign mammar | genitourinary | |
| 610.1 | 0.208843 | 0.128432 | 1.626102 | 0.103928 | -0.04288 | 0.460564 | AFR | Cystic mastopath | genitourinary | |
| 610.2 | -0.32457 | 0.372602 | -0.87108 | 0.38371 | -1.05485 | 0.40572 | AFR | Fibroadenosis of | genitourinary | |
| 610.3 | 0.58246 | 0.269124 | 2.164284 | 0.030443 | 0.054987 | 1.109933 | AFR | Fibrosclerosis of | genitourinary | |
| 610.4 | 0.086446 | 0.228314 | 0.378628 | 0.704964 | -0.36104 | 0.533934 | AFR | Benign neoplasm | genitourinary | |
| 610.8 | 0.100423 | 0.213043 | 0.471372 | 0.637375 | -0.31713 | 0.517979 | AFR | Other specified b | genitourinary | |
| 611 | 0.080162 | 0.072715 | 1.102403 | 0.270286 | -0.06236 | 0.222681 | AFR | Abnormal finding | genitourinary | |
| 611.1 | 0.132885 | 0.082522 | 1.610299 | 0.107333 | -0.02886 | 0.294625 | AFR | Abnormal mamm | genitourinary | |

| | | | | | | | | | | | |
|---|---|---|---|---|---|---|---|---|---|---|---|
| 611.11 | 0.129419 | 0.164931 | 0.784688 | 0.432636 | -0.19384 | 0.452677 | AFR | | Mammographic r | genitourinary | |
| 611.3 | 0.138629 | 0.088812 | 1.560932 | 0.11854 | -0.03544 | 0.312698 | AFR | | Lump or mass in | genitourinary | |
| 612 | -0.01528 | 0.156455 | -0.09764 | 0.922218 | -0.32192 | 0.29137 | AFR | | Breast conditions | genitourinary | |
| 612.1 | 0.084543 | 0.529709 | 0.159602 | 0.873194 | -0.95367 | 1.122752 | AFR | | Galactorrhea | genitourinary | |
| 612.2 | -0.07877 | 0.167001 | -0.4717 | 0.63714 | -0.40609 | 0.248541 | AFR | | Hypertrophy of b | genitourinary | |
| 612.3 | 0.129741 | 0.596551 | 0.217485 | 0.82783 | -1.03948 | 1.29896 | AFR | | Congenital anom | genitourinary | |
| 613 | 0.057873 | 0.082377 | 0.702533 | 0.482347 | -0.10358 | 0.219329 | AFR | | Other nonmaligna | genitourinary | |
| 613.1 | -0.06817 | 0.183062 | -0.37238 | 0.709606 | -0.42696 | 0.290625 | AFR | | Inflammatory dis | genitourinary | |
| 613.5 | 0.062257 | 0.105281 | 0.591345 | 0.55429 | -0.14409 | 0.268604 | AFR | | Mastodynia | genitourinary | |
| 613.7 | 0.106519 | 0.15338 | 0.694475 | 0.487384 | -0.1941 | 0.407138 | AFR | | Other signs and s | genitourinary | |
| 613.8 | 0.173554 | 0.130966 | 1.325181 | 0.185111 | -0.08314 | 0.430244 | AFR | | Other specified d | genitourinary | |
| 613.9 | 0.032991 | 0.294493 | 0.112026 | 0.910803 | -0.5442 | 0.610186 | AFR | | Breast disorder N | genitourinary | |
| 614 | -0.01623 | 0.085541 | -0.18969 | 0.849555 | -0.18388 | 0.151432 | AFR | | Inflammatory dis | genitourinary | |
| 614.1 | 0.042148 | 0.24857 | 0.16956 | 0.865356 | -0.44504 | 0.529336 | AFR | | Pelvic peritoneal | genitourinary | |
| 614.3 | 0.142073 | 0.268633 | 0.528874 | 0.596893 | -0.38444 | 0.668584 | AFR | | Pelvic inflammat | genitourinary | |
| 614.31 | 0.116457 | 0.536995 | 0.216868 | 0.828311 | -0.93603 | 1.168949 | AFR | | Acute inflammat | genitourinary | |
| 614.32 | 0.213438 | 0.389844 | 0.547495 | 0.584039 | -0.55064 | 0.977518 | AFR | | Chronic inflamma | genitourinary | |
| 614.33 | 0.959851 | 0.427753 | 2.243939 | 0.024836 | 0.121471 | 1.798231 | AFR | | Pelvic inflammat | genitourinary | |
| 614.4 | 0.630906 | 0.329993 | 1.911878 | 0.055892 | -0.01587 | 1.277681 | AFR | | Inflammatory dis | genitourinary | |
| 614.5 | -0.06577 | 0.089884 | -0.73168 | 0.464362 | -0.24193 | 0.110402 | AFR | | Inflammatory dis | genitourinary | |
| 614.51 | -0.19108 | 0.207803 | -0.91955 | 0.357809 | -0.59837 | 0.216201 | AFR | | Cervicitis and en | genitourinary | |
| 614.52 | -0.06092 | 0.097249 | -0.62645 | 0.531019 | -0.25153 | 0.129683 | AFR | | Vaginitis and vulv | genitourinary | |
| 614.53 | 0.317585 | 0.31881 | 0.996157 | 0.319174 | -0.30727 | 0.942442 | AFR | | Cyst or abscess | genitourinary | |
| 614.54 | 0.14345 | 0.317296 | 0.452103 | 0.651195 | -0.47844 | 0.765339 | AFR | | Abscess or ulcer | genitourinary | |
| 615 | -0.23803 | 0.142926 | -1.66539 | 0.095835 | -0.51816 | 0.042103 | AFR | | Endometriosis | genitourinary | |
| 617 | 0.017036 | 0.08858 | 0.192317 | 0.847494 | -0.15658 | 0.19065 | AFR | | Disorders second | genitourinary | |
| 618 | -0.11255 | 0.156954 | -0.71712 | 0.4733 | -0.42018 | 0.195069 | AFR | | Genital prolapse | genitourinary | |
| 618.1 | -0.21361 | 0.182549 | -1.17016 | 0.241936 | -0.5714 | 0.144177 | AFR | | Prolapse of vagir | genitourinary | |
| 618.2 | -0.04844 | 0.290403 | -0.1668 | 0.867527 | -0.61762 | 0.520739 | AFR | | Uterine/Uterovag | genitourinary | |
| 618.5 | -0.07328 | 0.554049 | -0.13226 | 0.894777 | -1.15919 | 1.012636 | AFR | | Prolapse of vagir | genitourinary | |
| 618.6 | -0.02103 | 1.034104 | -0.02034 | 0.983772 | -2.04784 | 2.005773 | AFR | | Vaginal enteroce | genitourinary | |
| 619 | -0.07881 | 0.069498 | -1.13393 | 0.256822 | -0.21502 | 0.057407 | AFR | | Noninflammatory | genitourinary | |
| 619.1 | -0.04375 | 0.142169 | -0.30774 | 0.758277 | -0.3224 | 0.234895 | AFR | | Noninflammatory | genitourinary | |
| 619.2 | 0.194756 | 0.157186 | 1.239016 | 0.21534 | -0.11332 | 0.502836 | AFR | | Disorders of uter | genitourinary | |
| 619.3 | -0.10671 | 0.13313 | -0.80155 | 0.422814 | -0.36764 | 0.15422 | AFR | | Noninflammatory | genitourinary | |
| 619.4 | 0.012414 | 0.084878 | 0.14626 | 0.883716 | -0.15394 | 0.178773 | AFR | | Noninflammatory | genitourinary | |
| 619.5 | -0.08626 | 0.192918 | -0.44715 | 0.654767 | -0.46438 | 0.29185 | AFR | | Noninflammatory | genitourinary | |
| 620 | -0.05619 | 0.270245 | -0.20791 | 0.835297 | -0.58586 | 0.473483 | AFR | | Dysplasia of fem | genitourinary | |

| | | | | | | | | | | |
|---|---|---|---|---|---|---|---|---|---|---|
| 620.1 | 0.042646 | 0.333279 | 0.127958 | 0.898182 | -0.61057 | 0.695861 | AFR | Dysplasia of cerv | genitourinary | |
| 621 | -0.5122 | 0.261246 | -1.96061 | 0.049925 | -1.02423 | -0.00017 | AFR | Endometrial hype | genitourinary | |
| 622 | -0.16337 | 0.133583 | -1.223 | 0.22133 | -0.42519 | 0.098446 | AFR | Polyp of female g | genitourinary | |
| 622.1 | -0.09449 | 0.156715 | -0.60297 | 0.54653 | -0.40165 | 0.212662 | AFR | Polyp of corpus u | genitourinary | |
| 622.2 | -0.30975 | 0.249655 | -1.24071 | 0.214714 | -0.79906 | 0.179566 | AFR | Mucous polyp of | genitourinary | |
| 623 | -0.08646 | 0.149028 | -0.58016 | 0.561807 | -0.37855 | 0.20563 | AFR | Hypertrophy of fe | genitourinary | |
| 624 | -0.01673 | 0.111521 | -0.14999 | 0.880773 | -0.2353 | 0.201849 | AFR | Symptoms involv | genitourinary | |
| 624.1 | -0.32285 | 0.729367 | -0.44265 | 0.658019 | -1.75239 | 1.106679 | AFR | Dystrophy of fem | genitourinary | |
| 624.9 | 0.125268 | 0.141534 | 0.885076 | 0.376115 | -0.15213 | 0.402669 | AFR | stress incontinen | genitourinary | |
| 625 | -0.03694 | 0.086572 | -0.42675 | 0.669565 | -0.20662 | 0.132733 | AFR | Pain and other sy | genitourinary | |
| 625.1 | 0.090792 | 0.208968 | 0.434477 | 0.663942 | -0.31878 | 0.500363 | AFR | Dyspareunia | genitourinary | |
| 626 | -0.02974 | 0.081679 | -0.3641 | 0.715787 | -0.18983 | 0.130349 | AFR | Disorders of men | genitourinary | |
| 626.1 | -0.05752 | 0.087738 | -0.65561 | 0.512077 | -0.22948 | 0.114441 | AFR | Irregular menstru | genitourinary | |
| 626.11 | 0.111397 | 0.170367 | 0.653864 | 0.5132 | -0.22252 | 0.445311 | AFR | Absent or infrequ | genitourinary | |
| 626.12 | -0.09352 | 0.108203 | -0.86431 | 0.387418 | -0.30559 | 0.118553 | AFR | Excessive or freq | genitourinary | |
| 626.13 | 0.107342 | 0.109272 | 0.982344 | 0.32593 | -0.10683 | 0.321511 | AFR | Irregular menstru | genitourinary | |
| 626.14 | -0.21679 | 0.142191 | -1.52462 | 0.127355 | -0.49548 | 0.061903 | AFR | Irregular menstru | genitourinary | |
| 626.15 | -0.13064 | 0.372615 | -0.3506 | 0.725888 | -0.86095 | 0.599673 | AFR | Infertility, female, | genitourinary | |
| 626.2 | -0.02821 | 0.163553 | -0.17251 | 0.86304 | -0.34877 | 0.292345 | AFR | Dysmenorrhea | genitourinary | |
| 626.21 | -0.12602 | 0.72928 | -0.17281 | 0.862803 | -1.55539 | 1.303337 | AFR | Mittelschmerz | genitourinary | |
| 626.4 | -0.19189 | 0.384137 | -0.49954 | 0.617397 | -0.94479 | 0.561001 | AFR | Premenstrual ten | genitourinary | |
| 626.8 | 0.245021 | 0.25824 | 0.948814 | 0.342715 | -0.26112 | 0.751162 | AFR | Infertility, female | genitourinary | |
| 627 | -0.06858 | 0.070869 | -0.96767 | 0.333207 | -0.20748 | 0.070322 | AFR | Menopausal and | genitourinary | |
| 627.1 | 0.092662 | 0.132107 | 0.701417 | 0.483043 | -0.16626 | 0.351588 | AFR | Postmenopausal | genitourinary | |
| 627.2 | -0.02017 | 0.077937 | -0.25884 | 0.795756 | -0.17293 | 0.13258 | AFR | Symptomatic me | genitourinary | |
| 627.21 | 0.058295 | 0.403103 | 0.144617 | 0.885013 | -0.73177 | 0.848362 | AFR | Symptomatic arti | genitourinary | |
| 627.22 | -0.12292 | 0.115602 | -1.06333 | 0.287633 | -0.3495 | 0.103653 | AFR | Need for Hormon | genitourinary | |
| 627.3 | 0.002216 | 0.127221 | 0.017419 | 0.986102 | -0.24713 | 0.251564 | AFR | Postmenopausal | genitourinary | |
| 627.4 | -0.02535 | 0.243764 | -0.10401 | 0.917158 | -0.50312 | 0.452413 | AFR | Premenopausal r | genitourinary | |
| 627.5 | 0.101404 | 0.267627 | 0.378902 | 0.704761 | -0.42314 | 0.625945 | AFR | Premature meno | genitourinary | |
| 628 | -0.13972 | 0.135567 | -1.03063 | 0.302712 | -0.40543 | 0.125986 | AFR | Ovarian cyst | genitourinary | |
| 634 | -0.05108 | 0.178731 | -0.28576 | 0.775059 | -0.40138 | 0.299232 | AFR | Miscarriage; stillb | pregnancy complications | |
| 634.1 | -0.00379 | 0.26773 | -0.01417 | 0.988691 | -0.52854 | 0.520947 | AFR | Missed abortion/I | pregnancy complications | |
| 634.3 | 0.191657 | 0.385372 | 0.497329 | 0.618957 | -0.56366 | 0.946973 | AFR | Ectopic pregnanc | pregnancy complications | |
| 635 | 0.340567 | 0.211323 | 1.611593 | 0.10705 | -0.07362 | 0.754754 | AFR | Hemorrhage duri | pregnancy complications | |
| 635.2 | 0.440719 | 0.252643 | 1.744435 | 0.081083 | -0.05445 | 0.93589 | AFR | Antepartum hem | pregnancy complications | |
| 635.3 | 0.18491 | 0.314091 | 0.588714 | 0.556053 | -0.4307 | 0.800517 | AFR | Placenta previa a | pregnancy complications | |
| 636 | -0.02287 | 0.182103 | -0.12556 | 0.900077 | -0.37978 | 0.334049 | AFR | Early or threaten | pregnancy complications | |

| | | | | | | | | | |
|---|---|---|---|---|---|---|---|---|---|
| 636.1 | -0.27833 | 0.297776 | -0.93471 | 0.349939 | -0.86196 | 0.305297 | AFR | Threatened prem | pregnancy complications |
| 636.2 | -0.65376 | 0.477221 | -1.36992 | 0.170711 | -1.58909 | 0.28158 | AFR | Early onset of de | pregnancy complications |
| 636.3 | 0.016808 | 0.235682 | 0.071314 | 0.943147 | -0.44512 | 0.478737 | AFR | Hemorrhage in e | pregnancy complications |
| 636.8 | -0.20667 | 0.422673 | -0.48895 | 0.624878 | -1.03509 | 0.621759 | AFR | Cervical incompe | pregnancy complications |
| 637 | 0.276108 | 0.38442 | 0.718244 | 0.472607 | -0.47734 | 1.029557 | AFR | Short gestation; l | pregnancy complications |
| 638 | -0.01921 | 0.173771 | -0.11058 | 0.911953 | -0.3598 | 0.32137 | AFR | Other high-risk p | pregnancy complications |
| 642 | 0.391937 | 0.213547 | 1.835365 | 0.066452 | -0.02661 | 0.810481 | AFR | Hypertension cor | pregnancy complications |
| 642.1 | 0.850623 | 0.332459 | 2.558579 | 0.01051 | 0.199015 | 1.502231 | AFR | Preeclampsia an | pregnancy complications |
| 643 | 0.070693 | 0.261985 | 0.269836 | 0.787287 | -0.44279 | 0.584174 | AFR | Excessive vomiti | pregnancy complications |
| 643.1 | 0.228657 | 0.456664 | 0.500712 | 0.616574 | -0.66639 | 1.123701 | AFR | Hyperemesis gra | pregnancy complications |
| 644 | 0.019385 | 0.205184 | 0.094476 | 0.924731 | -0.38277 | 0.421538 | AFR | Anemia during pr | pregnancy complications |
| 645 | 0.283623 | 0.287439 | 0.986724 | 0.323778 | -0.27975 | 0.846993 | AFR | Late pregnancy a | pregnancy complications |
| 646 | -0.06765 | 0.144583 | -0.46789 | 0.639863 | -0.35103 | 0.215728 | AFR | Other complicatio | pregnancy complications |
| 647 | -0.42029 | 0.261027 | -1.61015 | 0.107365 | -0.9319 | 0.09131 | AFR | Infectious and pa | pregnancy complications |
| 647.1 | 0.257225 | 0.326621 | 0.787534 | 0.430969 | -0.38294 | 0.897391 | AFR | Infections of geni | pregnancy complications |
| 647.3 | 14.53095 | 75.78794 | 0.191732 | 0.847952 | -134.011 | 163.0726 | AFR | Major puerperal i | pregnancy complications |
| 649 | 0.027409 | 0.144748 | 0.189354 | 0.849815 | -0.25629 | 0.31111 | AFR | Other conditions | pregnancy complications |
| 649.1 | -0.03271 | 0.244081 | -0.13402 | 0.893388 | -0.5111 | 0.445678 | AFR | Diabetes or abno | pregnancy complications |
| 650 | -0.17131 | 0.219376 | -0.7809 | 0.434859 | -0.60128 | 0.258657 | AFR | Normal delivery | pregnancy complications |
| 651 | -0.41812 | 0.252482 | -1.65605 | 0.097711 | -0.91298 | 0.076732 | AFR | Multiple gestatior | pregnancy complications |
| 652 | 0.341593 | 0.764577 | 0.446774 | 0.655038 | -1.15695 | 1.840136 | AFR | Malposition and r | pregnancy complications |
| 653 | -0.38397 | 0.216049 | -1.77721 | 0.075533 | -0.80741 | 0.039483 | AFR | Problems associa | pregnancy complications |
| 654 | 0.144719 | 0.167904 | 0.861918 | 0.388733 | -0.18437 | 0.473805 | AFR | Other and unspe | pregnancy complications |
| 654.1 | 0.518952 | 0.224535 | 2.311234 | 0.02082 | 0.078872 | 0.959032 | AFR | Abnormality of or | pregnancy complications |
| 654.2 | -0.98267 | 0.49697 | -1.97732 | 0.048005 | -1.95671 | -0.00863 | AFR | Rhesus isoimmu | pregnancy complications |
| 655 | -0.032 | 0.169753 | -0.18853 | 0.850459 | -0.36471 | 0.300706 | AFR | Known or suspec | pregnancy complications |
| 655.1 | -0.39029 | 0.23167 | -1.68468 | 0.092051 | -0.84435 | 0.063776 | AFR | Abnormality in fe | pregnancy complications |
| 656 | -0.21955 | 0.16223 | -1.35334 | 0.175946 | -0.53752 | 0.098412 | AFR | Other perinatal c | pregnancy complications |
| 656.1 | -0.17426 | 1.006964 | -0.17305 | 0.86261 | -2.14787 | 1.799356 | AFR | Isoimmunization | pregnancy complications |
| 656.2 | 0.061129 | 0.523852 | 0.116691 | 0.907105 | -0.9656 | 1.087861 | AFR | Respiratory cond | pregnancy complications |
| 656.26 | 0.461797 | 1.182883 | 0.390399 | 0.696241 | -1.85661 | 2.780205 | AFR | Transitory tachyp | pregnancy complications |
| 656.3 | -42.278 | | | | | | AFR | Endocrine and m | pregnancy complications |
| 656.8 | 0.730504 | 0.977571 | 0.747265 | 0.454904 | -1.1855 | 2.646508 | AFR | Perinatal jaundic | pregnancy complications |
| 658 | 0.288281 | 0.793217 | 0.363433 | 0.716281 | -1.2664 | 1.842958 | AFR | Maternal complic | pregnancy complications |
| 661 | 0.252499 | 0.320762 | 0.787186 | 0.431173 | -0.37618 | 0.881181 | AFR | Fetal distress and | pregnancy complications |
| 665 | -0.10238 | 0.215623 | -0.47481 | 0.63492 | -0.52499 | 0.320233 | AFR | Obstetrical/birth t | pregnancy complications |
| 668 | -0.92776 | 0.765761 | -1.21156 | 0.225682 | -2.42863 | 0.5731 | AFR | Complications of | pregnancy complications |
| 669 | -0.11571 | 0.239876 | -0.48237 | 0.629545 | -0.58586 | 0.354439 | AFR | Complications of | pregnancy complications |

| | | | | | | | | | | |
|---|---|---|---|---|---|---|---|---|---|---|
| 671 | -0.30905 | 0.238999 | -1.2931 | 0.195977 | -0.77748 | 0.15938 | AFR | | Venous/cerebrov | pregnancy complications |
| 674 | 0.31471 | 0.361502 | 0.870563 | 0.383993 | -0.39382 | 1.02324 | AFR | | Other complicatio | pregnancy complications |
| 676 | -0.56647 | 0.387311 | -1.46257 | 0.143586 | -1.32558 | 0.192648 | AFR | | Other disorders c | pregnancy complications |
| 681 | 0.002834 | 0.072799 | 0.038935 | 0.968942 | -0.13985 | 0.145517 | AFR | | Superficial celluli | dermatologic |
| 681.1 | 0.146344 | 0.13629 | 1.073767 | 0.282927 | -0.12078 | 0.413468 | AFR | | Cellulitis and abs | dermatologic |
| 681.2 | 0.18273 | 0.20935 | 0.872841 | 0.38275 | -0.22759 | 0.593049 | AFR | | Cellulitis and abs | dermatologic |
| 681.3 | -0.02903 | 0.142377 | -0.20392 | 0.838415 | -0.30809 | 0.250019 | AFR | | Cellulitis and abs | dermatologic |
| 681.5 | 0.123386 | 0.117926 | 1.046298 | 0.295423 | -0.10775 | 0.354518 | AFR | | Cellulitis and abs | dermatologic |
| 681.6 | -0.08714 | 0.227578 | -0.38291 | 0.701788 | -0.53319 | 0.358903 | AFR | | Cellulitis and abs | dermatologic |
| 681.7 | -0.1127 | 0.128214 | -0.87902 | 0.379388 | -0.364 | 0.138591 | AFR | | Cellulitis and abs | dermatologic |
| 686 | 0.070439 | 0.09719 | 0.724752 | 0.468604 | -0.12005 | 0.260927 | AFR | | Other local infect | dermatologic |
| 686.1 | 0.174612 | 0.170431 | 1.024529 | 0.305586 | -0.15943 | 0.508651 | AFR | | Carbuncle and fu | dermatologic |
| 686.2 | -0.19292 | 0.242158 | -0.79669 | 0.425632 | -0.66754 | 0.281696 | AFR | | Impetigo | dermatologic |
| 686.3 | 0.432349 | 0.371108 | 1.165022 | 0.24401 | -0.29501 | 1.159708 | AFR | | Pilonidal cyst | dermatologic |
| 686.4 | 0.465335 | 0.338077 | 1.376418 | 0.168692 | -0.19728 | 1.127953 | AFR | | Pyogenic granulo | dermatologic |
| 686.5 | 0.939345 | 0.518575 | 1.811397 | 0.070079 | -0.07704 | 1.955733 | AFR | | Pyoderma | dermatologic |
| 687 | -0.02507 | 0.065711 | -0.38148 | 0.70285 | -0.15386 | 0.103724 | AFR | | Symptoms affect | dermatologic |
| 687.1 | -0.03936 | 0.065536 | -0.60055 | 0.548142 | -0.16781 | 0.089091 | AFR | | Rash and other n | dermatologic |
| 687.2 | -0.07169 | 0.069643 | -1.02935 | 0.303317 | -0.20818 | 0.064811 | AFR | | Localized superfi | dermatologic |
| 687.3 | 0.169041 | 0.228362 | 0.740233 | 0.459159 | -0.27854 | 0.616624 | AFR | | Changes in skin t | dermatologic |
| 687.4 | 0.003331 | 0.063809 | 0.052205 | 0.958365 | -0.12173 | 0.128395 | AFR | | Disturbance of sk | dermatologic |
| 689 | -0.05816 | 0.070506 | -0.82492 | 0.409416 | -0.19635 | 0.080027 | AFR | | Disorder of skin a | dermatologic |
| 690 | 0.072448 | 0.113252 | 0.639706 | 0.522363 | -0.14952 | 0.294417 | AFR | | Erythematosquar | dermatologic |
| 690.1 | 0.096367 | 0.115254 | 0.836126 | 0.403084 | -0.12953 | 0.322262 | AFR | | Seborrheic derma | dermatologic |
| 691 | 0.263491 | 0.184777 | 1.425996 | 0.153869 | -0.09866 | 0.625647 | AFR | | Congenital anom | dermatologic |
| 691.1 | -0.29353 | 0.655918 | -0.44751 | 0.654506 | -1.5791 | 0.992045 | AFR | | Ichthyosis conge | dermatologic |
| 691.3 | 0.027168 | 0.478728 | 0.056751 | 0.954743 | -0.91112 | 0.965458 | AFR | | Congenital pigme | dermatologic |
| 694 | -0.03548 | 0.073898 | -0.48015 | 0.631122 | -0.18032 | 0.109355 | AFR | | Dyschromia and | dermatologic |
| 694.1 | 0.057069 | 0.269943 | 0.211413 | 0.832565 | -0.47201 | 0.586147 | AFR | | Vitiligo | dermatologic |
| 694.2 | -0.03109 | 0.075813 | -0.41007 | 0.681751 | -0.17968 | 0.117502 | AFR | | Other dyschromia | dermatologic |
| 694.3 | -0.22161 | 0.478863 | -0.46279 | 0.643517 | -1.16017 | 0.716943 | AFR | | Vascular disorder | dermatologic |
| 695 | -0.04662 | 0.080429 | -0.57961 | 0.562176 | -0.20426 | 0.111021 | AFR | | Erythematous co | dermatologic |
| 695.1 | 0.15799 | 0.359178 | 0.439865 | 0.660035 | -0.54599 | 0.861965 | AFR | | Toxic erythema | dermatologic |
| 695.2 | -0.3258 | 0.359805 | -0.9055 | 0.365199 | -1.03101 | 0.379401 | AFR | | Bullous dermatos | dermatologic |
| 695.22 | -0.17434 | 0.561836 | -0.31031 | 0.756323 | -1.27552 | 0.926833 | AFR | | Pemphigus and p | dermatologic |
| 695.3 | -0.2911 | 0.222296 | -1.30952 | 0.190359 | -0.72679 | 0.144592 | AFR | | Rosacea | dermatologic |
| 695.4 | 0.065165 | 0.13746 | 0.474063 | 0.635455 | -0.20425 | 0.334582 | AFR | | Lupus (localized | dermatologic |
| 695.41 | -0.0289 | 0.220273 | -0.13121 | 0.895609 | -0.46063 | 0.402824 | AFR | | Cutaneous lupus | dermatologic |

| | | | | | | | | | | |
|---|---|---|---|---|---|---|---|---|---|---|
| 695.42 | 0.044474 | 0.141042 | 0.315324 | 0.752516 | -0.23196 | 0.320911 | AFR | Systemic lupus e | dermatologic | |
| 695.7 | 0.09483 | 0.144951 | 0.654225 | 0.512967 | -0.18927 | 0.378929 | AFR | Prurigo and Liche | dermatologic | |
| 695.8 | -0.00153 | 0.144121 | -0.01062 | 0.991528 | -0.284 | 0.280942 | AFR | Other specified e | dermatologic | |
| 695.81 | -0.16563 | 0.397477 | -0.41671 | 0.676891 | -0.94467 | 0.613409 | AFR | Erythema nodosu | dermatologic | |
| 695.9 | -0.33682 | 0.259522 | -1.29784 | 0.194344 | -0.84547 | 0.171837 | AFR | Unspecified eryth | dermatologic | |
| 696 | 0.017913 | 0.170356 | 0.105149 | 0.916258 | -0.31598 | 0.351804 | AFR | Psoriasis and rel | dermatologic | |
| 696.2 | -0.2671 | 0.720111 | -0.37091 | 0.710706 | -1.67849 | 1.144297 | AFR | Parapsoriasis | dermatologic | |
| 696.3 | -0.11335 | 0.465533 | -0.24349 | 0.80763 | -1.02578 | 0.799078 | AFR | Pityriasis | dermatologic | |
| 696.4 | 0.058541 | 0.188731 | 0.310182 | 0.756422 | -0.31137 | 0.428447 | AFR | Psoriasis | dermatologic | |
| 696.41 | 0.099667 | 0.195152 | 0.510715 | 0.60955 | -0.28282 | 0.482159 | AFR | Psoriasis vulgaris | dermatologic | |
| 696.42 | -0.46656 | 0.368977 | -1.26448 | 0.20606 | -1.18974 | 0.25662 | AFR | Psoriatic arthropa | dermatologic | |
| 697 | 0.064405 | 0.192776 | 0.334093 | 0.738309 | -0.31343 | 0.44224 | AFR | Sarcoidosis | dermatologic | |
| 698 | 0.107826 | 0.074635 | 1.444717 | 0.148537 | -0.03846 | 0.254107 | AFR | Pruritus and relat | dermatologic | |
| 700 | -0.09158 | 0.14327 | -0.63922 | 0.522677 | -0.37239 | 0.189222 | AFR | Corns and callos | dermatologic | |
| 701 | -0.01651 | 0.064658 | -0.25531 | 0.798482 | -0.14323 | 0.110219 | AFR | Other hypertroph | dermatologic | |
| 701.1 | -0.00185 | 0.096714 | -0.01915 | 0.984719 | -0.19141 | 0.187703 | AFR | Keratoderma, ac | dermatologic | |
| 701.2 | 0.124966 | 0.118314 | 1.056219 | 0.290868 | -0.10693 | 0.356857 | AFR | Scar conditions a | dermatologic | |
| 701.3 | 0.058156 | 0.21967 | 0.264743 | 0.791208 | -0.37239 | 0.4887 | AFR | Circumscribed sc | dermatologic | |
| 701.4 | 0.059981 | 0.115232 | 0.520527 | 0.602696 | -0.16587 | 0.285832 | AFR | Keloid scar | dermatologic | |
| 701.5 | 0.054861 | 0.270265 | 0.20299 | 0.839143 | -0.47485 | 0.584571 | AFR | Abnormal granula | dermatologic | |
| 701.6 | 0.260746 | 0.278773 | 0.935333 | 0.349617 | -0.28564 | 0.807132 | AFR | Acquired acantho | dermatologic | |
| 702 | -0.00205 | 0.083502 | -0.02456 | 0.980405 | -0.16571 | 0.16161 | AFR | Degenerative ski | dermatologic | |
| 702.1 | 0.161729 | 0.264994 | 0.610313 | 0.541654 | -0.35765 | 0.681108 | AFR | Actinic keratosis | dermatologic | |
| 702.2 | -0.03825 | 0.087289 | -0.43824 | 0.661211 | -0.20934 | 0.132829 | AFR | Seborrheic kerato | dermatologic | |
| 702.4 | 0.051595 | 0.31188 | 0.165432 | 0.868604 | -0.55968 | 0.662869 | AFR | Degenerative ski | dermatologic | |
| 703 | 0.126182 | 0.08305 | 1.519349 | 0.128675 | -0.03659 | 0.288957 | AFR | Diseases of nail, | dermatologic | |
| 703.1 | 0.064526 | 0.142065 | 0.454196 | 0.649687 | -0.21392 | 0.342969 | AFR | Ingrowing nail | dermatologic | |
| 704 | 0.003788 | 0.079608 | 0.047588 | 0.962045 | -0.15224 | 0.159817 | AFR | Diseases of hair | dermatologic | |
| 704.1 | -0.02079 | 0.101402 | -0.20498 | 0.837589 | -0.21953 | 0.177958 | AFR | Alopecia | dermatologic | |
| 704.11 | 0.216535 | 0.328558 | 0.659048 | 0.509865 | -0.42743 | 0.860497 | AFR | Alopecia Areata | dermatologic | |
| 704.12 | -0.34608 | 0.251402 | -1.37659 | 0.168638 | -0.83882 | 0.146661 | AFR | Telogen effluvium | dermatologic | |
| 704.2 | 0.547931 | 0.206798 | 2.649597 | 0.008059 | 0.142615 | 0.953247 | AFR | Hirsutism | dermatologic | |
| 704.8 | 0.112252 | 0.109654 | 1.023687 | 0.305983 | -0.10267 | 0.32717 | AFR | Other specified d | dermatologic | |
| 705 | -0.01114 | 0.167807 | -0.0664 | 0.94706 | -0.34004 | 0.317753 | AFR | Disorders of swe | dermatologic | |
| 705.1 | -0.1682 | 0.310407 | -0.54186 | 0.587915 | -0.77658 | 0.44019 | AFR | Dyshidrosis | dermatologic | |
| 705.3 | 0.319352 | 0.251832 | 1.268116 | 0.204757 | -0.17423 | 0.812934 | AFR | Hidradenitis | dermatologic | |
| 705.8 | 0.105768 | 0.124901 | 0.846811 | 0.397101 | -0.13903 | 0.35057 | AFR | Hyperhidrosis | dermatologic | |
| 706 | 0.139063 | 0.072955 | 1.906137 | 0.056632 | -0.00393 | 0.282053 | AFR | Diseases of seba | dermatologic | |

| | | | | | | | | | | | |
|---|---|---|---|---|---|---|---|---|---|---|---|
| 706.1 | 0.222466 | 0.115373 | 1.928233 | 0.053826 | -0.00366 | 0.448593 | AFR | | Acne | dermatologic | |
| 706.2 | 0.021168 | 0.10431 | 0.202929 | 0.83919 | -0.18328 | 0.225612 | AFR | | Sebaceous cyst | dermatologic | |
| 706.8 | 0.166915 | 0.114291 | 1.460443 | 0.144168 | -0.05709 | 0.39092 | AFR | | Other specified d | dermatologic | |
| 707 | -0.03009 | 0.098114 | -0.30667 | 0.759091 | -0.22239 | 0.162212 | AFR | | Chronic ulcer of s | dermatologic | |
| 707.1 | -0.04669 | 0.143526 | -0.32529 | 0.744961 | -0.32799 | 0.234618 | AFR | | Decubitus ulcer | dermatologic | |
| 707.2 | 0.070945 | 0.144113 | 0.492284 | 0.622519 | -0.21151 | 0.353401 | AFR | | Chronic ulcer of l | dermatologic | |
| 707.3 | -0.01984 | 0.142608 | -0.13915 | 0.889335 | -0.29935 | 0.259664 | AFR | | Chronic ulcer of u | dermatologic | |
| 709 | -0.04264 | 0.108478 | -0.39311 | 0.69424 | -0.25526 | 0.169969 | AFR | | Diffuse diseases | dermatologic | |
| 709.2 | -0.26885 | 0.166399 | -1.61569 | 0.106161 | -0.59499 | 0.057287 | AFR | | Sicca syndrome | dermatologic | |
| 709.3 | 0.083374 | 0.177841 | 0.468814 | 0.639203 | -0.26519 | 0.431936 | AFR | | Systemic scleros | dermatologic | |
| 709.4 | -0.25417 | 0.389827 | -0.65202 | 0.514389 | -1.01822 | 0.509872 | AFR | | Polymyositis | dermatologic | |
| 709.5 | -0.03445 | 0.295392 | -0.11664 | 0.907148 | -0.61341 | 0.544504 | AFR | | Dermatomyositis | dermatologic | |
| 709.6 | -0.06775 | 0.219537 | -0.30861 | 0.757618 | -0.49804 | 0.362534 | AFR | | Other specified d | dermatologic | |
| 709.7 | 0.090465 | 0.16232 | 0.557328 | 0.577303 | -0.22768 | 0.408607 | AFR | | Unspecified diffu | dermatologic | |
| 710 | 0.23598 | 0.160599 | 1.469368 | 0.141733 | -0.07879 | 0.550749 | AFR | | Osteomyelitis, pe | musculoskeletal | |
| 710.1 | 0.23598 | 0.160599 | 1.469368 | 0.141733 | -0.07879 | 0.550749 | AFR | | Osteomyelitis | musculoskeletal | |
| 710.11 | 0.305656 | 0.216632 | 1.410944 | 0.158261 | -0.11894 | 0.730246 | AFR | | Acute osteomyeli | musculoskeletal | |
| 710.12 | 0.098018 | 0.214288 | 0.457411 | 0.647375 | -0.32198 | 0.518013 | AFR | | Chronic osteomy | musculoskeletal | |
| 710.19 | 0.218301 | 0.176166 | 1.239182 | 0.215278 | -0.12698 | 0.56358 | AFR | | Unspecified oste | musculoskeletal | |
| 711 | -0.1433 | 0.225383 | -0.6358 | 0.524908 | -0.58504 | 0.298445 | AFR | | Arthropathy asso | musculoskeletal | |
| 711.1 | -0.28323 | 0.236008 | -1.2001 | 0.230101 | -0.7458 | 0.179334 | AFR | | Pyogenic arthritis | musculoskeletal | |
| 711.2 | 0.025228 | 0.699345 | 0.036074 | 0.971224 | -1.34546 | 1.395919 | AFR | | Reiter's disease | musculoskeletal | |
| 712 | -0.8791 | 0.453072 | -1.9403 | 0.052343 | -1.7671 | 0.008909 | AFR | | Infective connect | musculoskeletal | |
| 713 | -0.38235 | 0.330462 | -1.15701 | 0.247267 | -1.03004 | 0.265345 | AFR | | Arthropathy asso | musculoskeletal | |
| 713.5 | 0.161119 | 0.461364 | 0.349223 | 0.726922 | -0.74314 | 1.065375 | AFR | | Arthropathy asso | musculoskeletal | |
| 714 | 0.050092 | 0.102449 | 0.488945 | 0.624881 | -0.1507 | 0.250889 | AFR | | Rheumatoid arth | musculoskeletal | |
| 714.1 | 0.097265 | 0.120832 | 0.804963 | 0.420841 | -0.13956 | 0.334091 | AFR | | Rheumatoid arth | musculoskeletal | |
| 714.2 | -0.69575 | 0.662541 | -1.05012 | 0.293664 | -1.9943 | 0.60281 | AFR | | Juvenile rheumat | musculoskeletal | |
| 715 | 0.161938 | 0.108958 | 1.486236 | 0.137217 | -0.05162 | 0.375492 | AFR | | Other inflammato | musculoskeletal | |
| 715.1 | 0.260029 | 0.130725 | 1.989122 | 0.046688 | 0.003812 | 0.516246 | AFR | | Sacroiliitis NEC | musculoskeletal | |
| 715.2 | 0.483771 | 0.444788 | 1.087642 | 0.276753 | -0.388 | 1.355539 | AFR | | Ankylosing spond | musculoskeletal | |
| 715.3 | -0.01857 | 0.440816 | -0.04212 | 0.966403 | -0.88255 | 0.845417 | AFR | | Spinal enthesopa | musculoskeletal | |
| 716 | -0.01288 | 0.063754 | -0.20196 | 0.839946 | -0.13783 | 0.11208 | AFR | | Other arthropathi | musculoskeletal | |
| 716.1 | -0.2221 | 0.226729 | -0.97957 | 0.327297 | -0.66648 | 0.222284 | AFR | | Unspecified polya | musculoskeletal | |
| 716.2 | -0.32978 | 0.528249 | -0.62428 | 0.532444 | -1.36512 | 0.705574 | AFR | | Unspecified mon | musculoskeletal | |
| 716.9 | -0.005 | 0.064731 | -0.07723 | 0.938443 | -0.13187 | 0.121872 | AFR | | Arthropathy NOS | musculoskeletal | |
| 717 | 0.294746 | 0.322066 | 0.915171 | 0.360102 | -0.33649 | 0.925985 | AFR | | Polymyalgia Rhe | musculoskeletal | |
| 720 | -0.0837 | 0.077805 | -1.07573 | 0.282048 | -0.23619 | 0.068798 | AFR | | Spinal stenosis | musculoskeletal | |

| | | | | | | | | | | |
|---|---|---|---|---|---|---|---|---|---|---|
| 720.1 | 0.031119 | 0.093145 | 0.334092 | 0.73831 | -0.15144 | 0.21368 | AFR | Spinal stenosis o | musculoskeletal | |
| 721 | 0.04581 | 0.064781 | 0.707153 | 0.479471 | -0.08116 | 0.172779 | AFR | Spondylosis and | musculoskeletal | |
| 721.1 | 0.031976 | 0.065589 | 0.487517 | 0.625892 | -0.09658 | 0.160527 | AFR | Spondylosis with | musculoskeletal | |
| 721.2 | -0.04919 | 0.170763 | -0.28805 | 0.773306 | -0.38388 | 0.2855 | AFR | Spondylosis with | musculoskeletal | |
| 721.8 | 0.048951 | 0.183092 | 0.267356 | 0.789195 | -0.3099 | 0.407804 | AFR | Other allied disor | musculoskeletal | |
| 722 | -0.03408 | 0.062799 | -0.5427 | 0.587333 | -0.15717 | 0.089003 | AFR | Intervertebral dis | musculoskeletal | |
| 722.1 | -0.02182 | 0.082767 | -0.26363 | 0.792068 | -0.18404 | 0.1404 | AFR | Displacement of | musculoskeletal | |
| 722.3 | -0.77356 | 0.977741 | -0.79117 | 0.428844 | -2.6899 | 1.142775 | AFR | Schmorl's nodes | musculoskeletal | |
| 722.6 | -0.01504 | 0.06532 | -0.23031 | 0.817848 | -0.14307 | 0.112981 | AFR | Degeneration of i | musculoskeletal | |
| 722.7 | -0.11128 | 0.249488 | -0.44603 | 0.655576 | -0.60027 | 0.377708 | AFR | Intervertebral dis | musculoskeletal | |
| 722.8 | 0.233979 | 0.193895 | 1.20673 | 0.227536 | -0.14605 | 0.614007 | AFR | Postlaminectomy | musculoskeletal | |
| 722.9 | -0.11422 | 0.145179 | -0.78672 | 0.431445 | -0.39876 | 0.17033 | AFR | Other and unspe | musculoskeletal | |
| 723 | -0.20131 | 0.156051 | -1.29005 | 0.197033 | -0.50717 | 0.104541 | AFR | Other disorders c | musculoskeletal | |
| 723.1 | -0.03528 | 0.292188 | -0.12073 | 0.903905 | -0.60795 | 0.537401 | AFR | Torticollis | musculoskeletal | |
| 724 | -0.00902 | 0.083052 | -0.10863 | 0.913499 | -0.1718 | 0.153758 | AFR | Other and unspe | musculoskeletal | |
| 724.1 | 0.040469 | 0.104236 | 0.38824 | 0.697839 | -0.16383 | 0.244768 | AFR | Disorders of sacr | musculoskeletal | |
| 724.2 | 0.021652 | 0.109094 | 0.19847 | 0.842677 | -0.19217 | 0.235473 | AFR | Disorders of cocc | musculoskeletal | |
| 724.8 | -0.05509 | 0.138484 | -0.39779 | 0.690785 | -0.32651 | 0.216337 | AFR | Other symptoms | musculoskeletal | |
| 724.9 | 0.099827 | 0.139835 | 0.713891 | 0.475294 | -0.17424 | 0.373897 | AFR | Other unspecified | musculoskeletal | |
| 726 | 0.07077 | 0.060529 | 1.169192 | 0.242326 | -0.04786 | 0.189404 | AFR | Peripheral enthes | musculoskeletal | |
| 726.1 | -0.02126 | 0.070264 | -0.30255 | 0.762232 | -0.15897 | 0.116456 | AFR | Enthesopathy | musculoskeletal | |
| 726.2 | 0.076304 | 0.157609 | 0.484136 | 0.62829 | -0.2326 | 0.385213 | AFR | Synoviopathy | musculoskeletal | |
| 726.3 | -0.03226 | 0.11931 | -0.27035 | 0.786888 | -0.2661 | 0.201588 | AFR | Bursitis | musculoskeletal | |
| 726.4 | -0.02825 | 0.129455 | -0.21825 | 0.827236 | -0.28198 | 0.225474 | AFR | Calcaneal spur; E | musculoskeletal | |
| 727 | 0.076711 | 0.065224 | 1.176122 | 0.239546 | -0.05113 | 0.204547 | AFR | Other disorders c | musculoskeletal | |
| 727.1 | 0.002039 | 0.077968 | 0.026151 | 0.979137 | -0.15078 | 0.154853 | AFR | Synovitis and ten | musculoskeletal | |
| 727.2 | 0.146222 | 0.236586 | 0.618049 | 0.536543 | -0.31748 | 0.609921 | AFR | Bursitis disorders | musculoskeletal | |
| 727.4 | -0.09945 | 0.139935 | -0.71065 | 0.477301 | -0.37371 | 0.174823 | AFR | Ganglion and cys | musculoskeletal | |
| 727.5 | -0.11388 | 0.164193 | -0.69355 | 0.487962 | -0.43569 | 0.207936 | AFR | Rupture of synov | musculoskeletal | |
| 727.6 | 0.189584 | 0.181159 | 1.046506 | 0.295327 | -0.16548 | 0.54465 | AFR | Rupture of tendo | musculoskeletal | |
| 727.7 | 0.494313 | 0.295556 | 1.672487 | 0.094428 | -0.08497 | 1.073592 | AFR | Contracture of te | musculoskeletal | |
| 727.8 | 1.426345 | 1.109369 | 1.285726 | 0.198539 | -0.74798 | 3.600668 | AFR | Plica syndrome | musculoskeletal | |
| 728 | -0.08322 | 0.234255 | -0.35526 | 0.722394 | -0.54235 | 0.375909 | AFR | Disorders of mus | musculoskeletal | |
| 728.1 | 0.272271 | 0.481727 | 0.565198 | 0.571939 | -0.6719 | 1.216438 | AFR | Muscular calcifica | musculoskeletal | |
| 728.2 | -0.12297 | 0.436818 | -0.28152 | 0.778313 | -0.97912 | 0.733176 | AFR | Laxity of ligamen | musculoskeletal | |
| 728.7 | 0.041841 | 0.103466 | 0.404398 | 0.68592 | -0.16095 | 0.244631 | AFR | Fasciitis | musculoskeletal | |
| 728.71 | -0.42984 | 0.27825 | -1.54478 | 0.122399 | -0.9752 | 0.115524 | AFR | Contracture of pa | musculoskeletal | |
| 729 | -0.02186 | 0.060747 | -0.35978 | 0.719011 | -0.14092 | 0.097207 | AFR | Other disorders c | musculoskeletal | |

| | | | | | | | | | | |
|---|---|---|---|---|---|---|---|---|---|---|
| 729.3 | 0.191647 | 0.357422 | 0.536194 | 0.591825 | -0.50889 | 0.892181 | AFR | Panniculitis | musculoskeletal | |
| 731 | 0.122673 | 0.120118 | 1.021266 | 0.307129 | -0.11275 | 0.3581 | AFR | Osteitis deformar | musculoskeletal | |
| 731.1 | 0.186782 | 0.425218 | 0.439263 | 0.660471 | -0.64663 | 1.020193 | AFR | Osteitis deformar | musculoskeletal | |
| 732 | 0.024529 | 1.306171 | 0.018779 | 0.985017 | -2.53552 | 2.584577 | AFR | Osteochondropat | musculoskeletal | |
| 732.1 | 0.024529 | 1.306171 | 0.018779 | 0.985017 | -2.53552 | 2.584577 | AFR | Juvenile osteoch | musculoskeletal | |
| 733 | 0.014916 | 0.069136 | 0.215751 | 0.829182 | -0.12059 | 0.15042 | AFR | Other disorders c | musculoskeletal | |
| 733.2 | -0.26861 | 0.341537 | -0.78648 | 0.431586 | -0.93801 | 0.400787 | AFR | Cyst of bone | musculoskeletal | |
| 733.4 | -0.09324 | 0.164262 | -0.56764 | 0.570282 | -0.41519 | 0.228707 | AFR | Aseptic necrosis | musculoskeletal | |
| 733.6 | 0.033728 | 0.161596 | 0.208718 | 0.834669 | -0.28299 | 0.350449 | AFR | Costochondritis | musculoskeletal | |
| 733.8 | -0.0264 | 0.281277 | -0.09387 | 0.925214 | -0.5777 | 0.52489 | AFR | Malunion and no | musculoskeletal | |
| 733.9 | 0.434704 | 0.198045 | 2.194976 | 0.028165 | 0.046543 | 0.822864 | AFR | Chondromalacia | musculoskeletal | |
| 735 | -0.01518 | 0.073718 | -0.2059 | 0.836865 | -0.15966 | 0.129306 | AFR | Acquired foot def | musculoskeletal | |
| 735.1 | 0.196028 | 0.151449 | 1.294346 | 0.195546 | -0.10081 | 0.492862 | AFR | Flat foot | musculoskeletal | |
| 735.2 | -0.17361 | 0.128359 | -1.35253 | 0.176205 | -0.42519 | 0.07797 | AFR | Acquired toe defo | musculoskeletal | |
| 735.21 | -0.08959 | 0.150185 | -0.59653 | 0.550818 | -0.38395 | 0.204767 | AFR | Hammer toe (acc | musculoskeletal | |
| 735.22 | -1.079 | 0.87698 | -1.23036 | 0.218562 | -2.79785 | 0.639848 | AFR | Claw toe (acquire | musculoskeletal | |
| 735.23 | -0.36014 | 0.323233 | -1.11417 | 0.265207 | -0.99366 | 0.273389 | AFR | Hallux rigidus | musculoskeletal | |
| 735.3 | -0.16019 | 0.117511 | -1.36319 | 0.172822 | -0.39051 | 0.070127 | AFR | Hallux valgus (Bu | musculoskeletal | |
| 736 | 0.158798 | 0.152879 | 1.038721 | 0.298935 | -0.14084 | 0.458435 | AFR | Other acquired d | musculoskeletal | |
| 736.1 | 0.35151 | 0.561483 | 0.626039 | 0.531289 | -0.74898 | 1.451998 | AFR | Acquired deformi | musculoskeletal | |
| 736.2 | 0.263612 | 0.33962 | 0.776199 | 0.437632 | -0.40203 | 0.929255 | AFR | Acquired deformi | musculoskeletal | |
| 736.3 | 0.258998 | 1.086632 | 0.238349 | 0.81161 | -1.87076 | 2.388757 | AFR | Acquired deformi | musculoskeletal | |
| 736.4 | 0.051089 | 0.294064 | 0.173735 | 0.862074 | -0.52527 | 0.627444 | AFR | Genu valgum or v | musculoskeletal | |
| 736.5 | 0.528659 | 0.521915 | 1.012921 | 0.311098 | -0.49428 | 1.551593 | AFR | Acquired deformi | musculoskeletal | |
| 736.6 | 0.072812 | 0.282371 | 0.257858 | 0.796517 | -0.48063 | 0.626248 | AFR | Unequal leg leng | musculoskeletal | |
| 737 | -0.03613 | 0.118284 | -0.30549 | 0.759993 | -0.26797 | 0.195698 | AFR | Curvature of spin | musculoskeletal | |
| 737.1 | 0.181463 | 0.320221 | 0.566679 | 0.570932 | -0.44616 | 0.809085 | AFR | Kyphosis (acquir | musculoskeletal | |
| 737.2 | -0.33014 | 0.419599 | -0.78679 | 0.431404 | -1.15254 | 0.492262 | AFR | Lordosis (acquire | musculoskeletal | |
| 737.3 | -0.08432 | 0.124483 | -0.67739 | 0.498156 | -0.32831 | 0.159658 | AFR | Kyphoscoliosis a | musculoskeletal | |
| 738 | -0.00979 | 0.108391 | -0.09028 | 0.928062 | -0.22223 | 0.202656 | AFR | Other acquired m | musculoskeletal | |
| 738.4 | -0.02911 | 0.124269 | -0.23426 | 0.814781 | -0.27268 | 0.214452 | AFR | Acquired spondy | musculoskeletal | |
| 739 | 0.113231 | 0.254454 | 0.444995 | 0.656324 | -0.38549 | 0.611952 | AFR | Contracture of joi | musculoskeletal | |
| 740 | 0.032229 | 0.061229 | 0.526356 | 0.598641 | -0.08778 | 0.152236 | AFR | Osteoarthrosis | musculoskeletal | |
| 740.1 | 0.017464 | 0.061755 | 0.28279 | 0.777338 | -0.10357 | 0.138502 | AFR | Osteoarthritis; loc | musculoskeletal | |
| 740.11 | 0.015688 | 0.061964 | 0.25318 | 0.800129 | -0.10576 | 0.137135 | AFR | Osteoarthrosis, lo | musculoskeletal | |
| 740.12 | -0.11284 | 0.138038 | -0.81747 | 0.413659 | -0.38339 | 0.157708 | AFR | Osteoarthrosis, lo | musculoskeletal | |
| 740.2 | 0.007759 | 0.102839 | 0.075451 | 0.939856 | -0.1938 | 0.20932 | AFR | Osteoarthrosis, g | musculoskeletal | |
| 740.3 | -0.02806 | 0.174369 | -0.1609 | 0.872175 | -0.36981 | 0.313702 | AFR | Osteoarthrosis in | musculoskeletal | |

| | | | | | | | | | | |
|---|---|---|---|---|---|---|---|---|---|---|
| 740.9 | 0.034626 | 0.062662 | 0.552581 | 0.580551 | -0.08819 | 0.157441 | AFR | | Osteoarthrosis N | musculoskeletal |
| 741 | 0.132097 | 0.064303 | 2.054275 | 0.039949 | 0.006064 | 0.258129 | AFR | | Symptoms and d | musculoskeletal |
| 741.1 | 0.02478 | 0.347548 | 0.071299 | 0.94316 | -0.6564 | 0.70596 | AFR | | Ankylosis of joint | musculoskeletal |
| 741.2 | 0.176772 | 0.151507 | 1.166756 | 0.243309 | -0.12018 | 0.47372 | AFR | | Stiffness of joint | musculoskeletal |
| 741.3 | 0.134178 | 0.18721 | 0.716724 | 0.473544 | -0.23275 | 0.501103 | AFR | | Difficulty in walki | musculoskeletal |
| 741.4 | 0.115571 | 0.071122 | 1.624972 | 0.104168 | -0.02383 | 0.254967 | AFR | | Joint effusions | musculoskeletal |
| 741.5 | 0.534301 | 0.744724 | 0.717449 | 0.473097 | -0.92533 | 1.993933 | AFR | | Hemarthrosis | musculoskeletal |
| 741.6 | 0.644809 | 0.837624 | 0.769807 | 0.441414 | -0.9969 | 2.286522 | AFR | | Villonodular sync | musculoskeletal |
| 742 | 0.063728 | 0.121595 | 0.524098 | 0.60021 | -0.17459 | 0.30205 | AFR | | Derangement of | musculoskeletal |
| 742.1 | 0.265865 | 0.609008 | 0.436553 | 0.662435 | -0.92777 | 1.459499 | AFR | | Loose body in joi | musculoskeletal |
| 742.2 | 0.504693 | 0.990486 | 0.50954 | 0.610374 | -1.43662 | 2.44601 | AFR | | Pathological, dev | musculoskeletal |
| 742.8 | 0.284457 | 0.247286 | 1.150319 | 0.250013 | -0.20021 | 0.769128 | AFR | | Articular cartilage | musculoskeletal |
| 742.9 | 0.021104 | 0.137371 | 0.153631 | 0.8779 | -0.24814 | 0.290347 | AFR | | Other derangeme | musculoskeletal |
| 743 | -0.05528 | 0.064791 | -0.85327 | 0.393508 | -0.18227 | 0.071704 | AFR | | Osteoporosis, os | musculoskeletal |
| 743.1 | -0.12669 | 0.090509 | -1.39973 | 0.161593 | -0.30408 | 0.050706 | AFR | | Osteoporosis | musculoskeletal |
| 743.11 | -0.1355 | 0.093343 | -1.45161 | 0.14661 | -0.31845 | 0.047451 | AFR | | Osteoporosis NC | musculoskeletal |
| 743.12 | 0.024205 | 0.136454 | 0.177386 | 0.859205 | -0.24324 | 0.291649 | AFR | | Senile osteoporo | musculoskeletal |
| 743.13 | 0.010152 | 0.158985 | 0.063857 | 0.949084 | -0.30145 | 0.321757 | AFR | | Other specified o | musculoskeletal |
| 743.2 | -0.07118 | 0.168901 | -0.4214 | 0.673461 | -0.40222 | 0.259865 | AFR | | Pathologic fractu | musculoskeletal |
| 743.21 | -0.63878 | 0.305574 | -2.09041 | 0.036581 | -1.23769 | -0.03986 | AFR | | Pathologic fractu | musculoskeletal |
| 743.22 | 0.075651 | 0.28003 | 0.270152 | 0.787043 | -0.4732 | 0.6245 | AFR | | Pathologic fractu | musculoskeletal |
| 743.4 | -0.18555 | 0.264905 | -0.70044 | 0.483652 | -0.70475 | 0.333654 | AFR | | Stress fracture | musculoskeletal |
| 743.9 | 0.015199 | 0.070044 | 0.216992 | 0.828215 | -0.12208 | 0.152482 | AFR | | Osteopenia or ot | musculoskeletal |
| 745 | 0.085673 | 0.056972 | 1.503778 | 0.132639 | -0.02599 | 0.197337 | AFR | | Pain in joint | musculoskeletal |
| 747 | -0.04923 | 0.109365 | -0.45011 | 0.652629 | -0.26358 | 0.165125 | AFR | | Cardiac and circu | congenital anomalies |
| 747.1 | -0.09222 | 0.121829 | -0.75697 | 0.449068 | -0.331 | 0.14656 | AFR | | Cardiac congenit | congenital anomalies |
| 747.11 | -0.06529 | 0.152918 | -0.42695 | 0.669415 | -0.365 | 0.234425 | AFR | | Cardiac shunt/ he | congenital anomalies |
| 747.12 | -0.33824 | 0.267387 | -1.26497 | 0.205884 | -0.86231 | 0.185834 | AFR | | Valvular heart dis | congenital anomalies |
| 747.13 | -0.18557 | 0.206295 | -0.89955 | 0.368361 | -0.5899 | 0.218758 | AFR | | Congenital anom | congenital anomalies |
| 747.2 | 0.075159 | 0.231529 | 0.32462 | 0.745469 | -0.37863 | 0.528948 | AFR | | Congenital anom | congenital anomalies |
| 748 | 0.16321 | 0.422704 | 0.386109 | 0.699416 | -0.66527 | 0.991695 | AFR | | Anomalies of res | congenital anomalies |
| 749 | -0.15886 | 0.368283 | -0.43136 | 0.666206 | -0.88068 | 0.562959 | AFR | | Congenital anom | congenital anomalies |
| 749.2 | -0.72154 | 0.539567 | -1.33726 | 0.181138 | -1.77907 | 0.335991 | AFR | | Congenital anom | congenital anomalies |
| 750 | 0.185258 | 0.15567 | 1.19007 | 0.234019 | -0.11985 | 0.490365 | AFR | | Digestive conger | congenital anomalies |
| 750.1 | -0.11208 | 0.211586 | -0.52969 | 0.596325 | -0.52678 | 0.302626 | AFR | | Upper gastrointe | congenital anomalies |
| 750.11 | -0.05171 | 0.35347 | -0.1463 | 0.883684 | -0.7445 | 0.641075 | AFR | | Esophageal atres | congenital anomalies |
| 750.13 | -0.24679 | 0.276402 | -0.89286 | 0.371934 | -0.78852 | 0.29495 | AFR | | Congenital anom | congenital anomalies |
| 750.14 | -0.78068 | 0.942871 | -0.82798 | 0.407683 | -2.62867 | 1.067316 | AFR | | Congenital anom | congenital anomalies |

| | | | | | | | | | |
|---|---|---|---|---|---|---|---|---|---|
| 750.15 | 0.011122 | 0.729726 | 0.015242 | 0.987839 | -1.41911 | 1.441358 | AFR | Congenital anom | congenital anomalies |
| 750.2 | 0.489189 | 0.225783 | 2.166636 | 0.030263 | 0.046663 | 0.931714 | AFR | Lower gastrointes | congenital anomalies |
| 750.21 | 0.287004 | 0.281271 | 1.02038 | 0.307548 | -0.26428 | 0.838286 | AFR | Congenital anom | congenital anomalies |
| 750.22 | 0.153237 | 0.296938 | 0.516057 | 0.605815 | -0.42875 | 0.735225 | AFR | Congenital anom | congenital anomalies |
| 750.5 | -0.15092 | 1.112754 | -0.13563 | 0.892117 | -2.33188 | 2.03004 | AFR | Congenital hyper | congenital anomalies |
| 751 | 0.01275 | 0.119015 | 0.107131 | 0.914685 | -0.22051 | 0.246015 | AFR | Genitourinary co | congenital anomalies |
| 751.1 | -0.09529 | 0.444592 | -0.21434 | 0.830281 | -0.96668 | 0.77609 | AFR | Congenital anom | congenital anomalies |
| 751.11 | -0.0738 | 0.755649 | -0.09767 | 0.922197 | -1.55485 | 1.407244 | AFR | Congenital anom | congenital anomalies |
| 751.12 | 0.322192 | 0.806173 | 0.399656 | 0.68941 | -1.25788 | 1.902262 | AFR | Congenital anom | congenital anomalies |
| 751.2 | 0.066527 | 0.126179 | 0.52724 | 0.598027 | -0.18078 | 0.313834 | AFR | Congenital anom | congenital anomalies |
| 751.21 | 0.044085 | 0.140972 | 0.312722 | 0.754492 | -0.23222 | 0.320385 | AFR | Cystic kidney dis | congenital anomalies |
| 751.22 | 0.416179 | 0.404452 | 1.028994 | 0.303483 | -0.37653 | 1.208891 | AFR | Other specified c | congenital anomalies |
| 751.3 | 0.082905 | 0.47984 | 0.172777 | 0.862827 | -0.85756 | 1.023375 | AFR | Obstructive genit | congenital anomalies |
| 752 | 0.050731 | 0.293551 | 0.172819 | 0.862794 | -0.52462 | 0.62608 | AFR | Nervous system | congenital anomalies |
| 752.1 | -0.36907 | 0.51151 | -0.72154 | 0.470579 | -1.37162 | 0.633468 | AFR | Neural tube defe | congenital anomalies |
| 752.11 | 0.943789 | 1.428044 | 0.660897 | 0.508679 | -1.85512 | 3.742703 | AFR | Spina bifida | congenital anomalies |
| 752.2 | 0.467062 | 0.405096 | 1.152966 | 0.248924 | -0.32691 | 1.261035 | AFR | Other specified c | congenital anomalies |
| 753 | -0.00276 | 0.290834 | -0.00949 | 0.992428 | -0.57278 | 0.567265 | AFR | Congenital anom | congenital anomalies |
| 753.1 | -0.60708 | 0.984245 | -0.61679 | 0.53737 | -2.53616 | 1.322008 | AFR | Congenital catara | congenital anomalies |
| 753.2 | 0.092785 | 0.492315 | 0.188467 | 0.85051 | -0.87213 | 1.057704 | AFR | Congenital anom | congenital anomalies |
| 754 | 0.026935 | 0.205236 | 0.131238 | 0.895587 | -0.37532 | 0.429191 | AFR | Congenital musc | congenital anomalies |
| 754.1 | 0.128429 | 0.741258 | 0.173258 | 0.862449 | -1.32441 | 1.581268 | AFR | Lumbosacral spo | congenital anomalies |
| 754.2 | 0.006081 | 0.227907 | 0.02668 | 0.978715 | -0.44061 | 0.452769 | AFR | Spondylolisthesis | congenital anomalies |
| 755 | 0.019514 | 0.252689 | 0.077224 | 0.938445 | -0.47575 | 0.514776 | AFR | Congenital anom | congenital anomalies |
| 755.1 | -0.41348 | 0.300502 | -1.37595 | 0.168836 | -1.00245 | 0.175496 | AFR | Congenital defor | congenital anomalies |
| 755.4 | 0.611646 | 0.461773 | 1.324562 | 0.185316 | -0.29341 | 1.516704 | AFR | Congenital anom | congenital anomalies |
| 755.6 | -0.12238 | 0.47946 | -0.25525 | 0.798526 | -1.06211 | 0.817341 | AFR | Other congenital | congenital anomalies |
| 755.61 | 0.625971 | 0.606041 | 1.032886 | 0.301657 | -0.56185 | 1.81379 | AFR | Congenital hip dy | congenital anomalies |
| 756 | 0.24431 | 0.250125 | 0.976754 | 0.328691 | -0.24593 | 0.734546 | AFR | Other congenital | congenital anomalies |
| 756.1 | 0.709304 | 0.415749 | 1.706086 | 0.087992 | -0.10555 | 1.524158 | AFR | Congenital anom | congenital anomalies |
| 756.2 | 0.601866 | 0.557307 | 1.079953 | 0.280163 | -0.49044 | 1.694168 | AFR | Pectus and other | congenital anomalies |
| 756.21 | 0.031979 | 0.570936 | 0.056012 | 0.955333 | -1.08703 | 1.150993 | AFR | Pectus excavatu | congenital anomalies |
| 756.3 | -0.68912 | 0.698246 | -0.98693 | 0.323678 | -2.05766 | 0.67942 | AFR | Congenital anom | congenital anomalies |
| 756.5 | -0.31834 | 0.330488 | -0.96324 | 0.335429 | -0.96608 | 0.329407 | AFR | Congenital osteo | congenital anomalies |
| 757 | 0.418977 | 0.280133 | 1.495633 | 0.134749 | -0.13007 | 0.968028 | AFR | Congenital anom | congenital anomalies |
| 758 | -0.18179 | 0.315154 | -0.57683 | 0.564052 | -0.79948 | 0.435899 | AFR | Chromosomal an | congenital anomalies |
| 758.1 | -0.18698 | 0.319393 | -0.58541 | 0.558272 | -0.81297 | 0.439023 | AFR | Chromosomal an | congenital anomalies |
| 759 | -0.08836 | 0.273305 | -0.3233 | 0.746471 | -0.62403 | 0.447309 | AFR | Other and unspe | congenital anomalies |

| | | | | | | | | | | |
|---|---|---|---|---|---|---|---|---|---|---|
| 759.1 | 0.804332 | 0.799181 | 1.006445 | 0.314201 | -0.76203 | 2.370699 | AFR | | Anomalies of end | congenital anomalies |
| 760 | 0.017676 | 0.055399 | 0.319062 | 0.74968 | -0.0909 | 0.126255 | AFR | | Back pain | symptoms |
| 761 | -0.07153 | 0.06297 | -1.13591 | 0.255995 | -0.19495 | 0.051891 | AFR | | Cervicalgia | symptoms |
| 763 | -0.03272 | 0.076163 | -0.42963 | 0.667468 | -0.182 | 0.116555 | AFR | | Thoracic or lumb | symptoms |
| 764 | -0.06336 | 0.073416 | -0.86307 | 0.388102 | -0.20726 | 0.08053 | AFR | | Sciatica | symptoms |
| 765 | -0.02788 | 0.091042 | -0.30622 | 0.759438 | -0.20632 | 0.15056 | AFR | | Cervical radiculiti | symptoms |
| 766 | -0.11109 | 0.09729 | -1.14183 | 0.253525 | -0.30177 | 0.079596 | AFR | | Neuralgia, neuriti | symptoms |
| 767 | 0.536629 | 0.76857 | 0.698217 | 0.485041 | -0.96974 | 2.042999 | AFR | | Cervicocranial/Ce | symptoms |
| 769 | 1.007369 | 1.030073 | 0.977959 | 0.328095 | -1.01154 | 3.026276 | AFR | | Nonallopathic les | symptoms |
| 770 | -0.08097 | 0.063504 | -1.2751 | 0.202274 | -0.20544 | 0.043492 | AFR | | Myalgia and myo | symptoms |
| 771 | -0.015 | 0.086436 | -0.17351 | 0.862247 | -0.18441 | 0.154414 | AFR | | Musculoskeletal s | symptoms |
| 771.1 | -0.00233 | 0.061098 | -0.03811 | 0.969603 | -0.12208 | 0.117422 | AFR | | Swelling of limb | symptoms |
| 771.2 | 0.022879 | 0.112361 | 0.203621 | 0.838649 | -0.19734 | 0.243103 | AFR | | Cramp of limb | symptoms |
| 772 | 0.15888 | 0.112612 | 1.410861 | 0.158286 | -0.06184 | 0.379596 | AFR | | Symptoms of the | symptoms |
| 772.1 | 0.081701 | 0.191852 | 0.425855 | 0.670213 | -0.29432 | 0.457724 | AFR | | Muscular wasting | symptoms |
| 772.2 | -0.03357 | 0.097301 | -0.34498 | 0.730112 | -0.22427 | 0.15714 | AFR | | Spasm of muscle | symptoms |
| 772.3 | 0.003895 | 0.10156 | 0.038352 | 0.969407 | -0.19516 | 0.202948 | AFR | | Muscle weakness | symptoms |
| 772.4 | -0.14156 | 0.243864 | -0.58051 | 0.561574 | -0.61953 | 0.3364 | AFR | | Rhabdomyolysis | symptoms |
| 772.6 | -0.04659 | 0.205436 | -0.22679 | 0.820588 | -0.44924 | 0.356057 | AFR | | Facial weakness | symptoms |
| 773 | -0.03591 | 0.055085 | -0.65198 | 0.514415 | -0.14388 | 0.072051 | AFR | | Pain in limb | symptoms |
| 780 | 0.132213 | 0.14391 | 0.918718 | 0.358243 | -0.14985 | 0.414271 | AFR | | Hypothermia/Chi | symptoms |
| 781 | -0.13819 | 0.073381 | -1.88322 | 0.05967 | -0.28202 | 0.005631 | AFR | | Symptoms involv | symptoms |
| 781.1 | -0.57276 | 0.397184 | -1.44204 | 0.14929 | -1.35122 | 0.20571 | AFR | | Loss of height | symptoms |
| 781.2 | -0.56753 | 0.261027 | -2.1742 | 0.02969 | -1.07913 | -0.05592 | AFR | | Abnormal posture | symptoms |
| 782.3 | -0.02858 | 0.060793 | -0.47005 | 0.638322 | -0.14773 | 0.090577 | AFR | | Edema | symptoms |
| 782.6 | -0.30874 | 0.126669 | -2.43738 | 0.014794 | -0.55701 | -0.06047 | AFR | | Pallor and flushin | symptoms |
| 783 | 0.093808 | 0.064891 | 1.445615 | 0.148285 | -0.03338 | 0.220992 | AFR | | Fever of unknow | symptoms |
| 783.1 | -0.37303 | 0.292958 | -1.27333 | 0.202902 | -0.94722 | 0.201156 | AFR | | Postprocedural fe | symptoms |
| 785 | 0.067571 | 0.054288 | 1.244679 | 0.21325 | -0.03883 | 0.173973 | AFR | | Abdominal pain | symptoms |
| 788 | -0.14174 | 0.081712 | -1.73464 | 0.082804 | -0.30189 | 0.018412 | AFR | | Syncope and coll | symptoms |
| 789 | 0.009181 | 0.055312 | 0.165977 | 0.868175 | -0.09923 | 0.117591 | AFR | | Nausea and vom | symptoms |
| 789.1 | 0.20466 | 0.167165 | 1.2243 | 0.220839 | -0.12298 | 0.532298 | AFR | | Persistent vomiti | symptoms |
| 790 | 0.060491 | 0.076214 | 0.793702 | 0.427369 | -0.08888 | 0.209867 | AFR | | Nonspecific findir | symptoms |
| 790.1 | 0.196755 | 0.157594 | 1.24849 | 0.211852 | -0.11212 | 0.505633 | AFR | | Elevated sedime | symptoms |
| 790.6 | 0.03906 | 0.057539 | 0.678835 | 0.497243 | -0.07372 | 0.151834 | AFR | | Other abnormal b | symptoms |
| 790.8 | -0.26702 | 0.200391 | -1.33251 | 0.182694 | -0.65978 | 0.125737 | AFR | | Elevated C-react | symptoms |
| 790.9 | 1.320288 | 0.554468 | 2.38118 | 0.017257 | 0.233551 | 2.407025 | AFR | | Abnormal arterial | symptoms |
| 791 | -0.05496 | 0.154775 | -0.35509 | 0.722525 | -0.35831 | 0.248395 | AFR | | Gangrene | symptoms |

| | | | | | | | | | |
|---|---|---|---|---|---|---|---|---|---|
| 792 | -0.12091 | 0.112732 | -1.07251 | 0.283492 | -0.34186 | 0.100045 | AFR | Abnormal Papan | genitourinary |
| 792.1 | -0.03828 | 0.132561 | -0.2888 | 0.772737 | -0.2981 | 0.221532 | AFR | Papanicolaou sm | genitourinary |
| 793 | 0.067319 | 0.187531 | 0.358976 | 0.719613 | -0.30024 | 0.434874 | AFR | Nonspecific abnc | symptoms |
| 793.2 | -0.08043 | 0.105156 | -0.76491 | 0.444326 | -0.28654 | 0.125667 | AFR | Nonspecific abnc | symptoms |
| 794 | -0.17528 | 0.359273 | -0.48788 | 0.625633 | -0.87945 | 0.528879 | AFR | Abnormal results | symptoms |
| 795 | -0.00898 | 0.175333 | -0.05122 | 0.959152 | -0.35263 | 0.334667 | AFR | Other and nonsp | symptoms |
| 795.8 | 0.256188 | 0.242324 | 1.057211 | 0.290415 | -0.21876 | 0.731134 | AFR | Abnormal tumor | symptoms |
| 795.81 | 0.333676 | 0.342541 | 0.974118 | 0.329998 | -0.33769 | 1.005045 | AFR | Elevated carcino | symptoms |
| 795.82 | 0.711136 | 0.412299 | 1.724806 | 0.084562 | -0.09696 | 1.519227 | AFR | Elevated cancer | symptoms |
| 796 | -0.22802 | 0.138076 | -1.65141 | 0.098655 | -0.49864 | 0.042604 | AFR | Elevated prostate | genitourinary |
| 797 | 0.045849 | 0.123113 | 0.372417 | 0.709583 | -0.19545 | 0.287147 | AFR | Shock | symptoms |
| 797.1 | -0.13039 | 0.169811 | -0.76783 | 0.442591 | -0.46321 | 0.202438 | AFR | Cardiogenic shoc | symptoms |
| 798 | 0.06951 | 0.055635 | 1.249386 | 0.211524 | -0.03953 | 0.178553 | AFR | Malaise and fatig | symptoms |
| 798.1 | -0.09922 | 0.124313 | -0.79814 | 0.424789 | -0.34287 | 0.144429 | AFR | Chronic fatigue s | symptoms |
| 800 | -0.05356 | 0.115571 | -0.46343 | 0.643053 | -0.28007 | 0.172956 | AFR | Fracture of lower | injuries & poisonings |
| 800.1 | -0.09962 | 0.251683 | -0.39581 | 0.692245 | -0.59291 | 0.393671 | AFR | Fracture of neck | injuries & poisonings |
| 800.2 | 0.118158 | 0.326945 | 0.361401 | 0.7178 | -0.52264 | 0.758958 | AFR | Fracture of unspe | injuries & poisonings |
| 800.3 | -0.23813 | 0.194276 | -1.22573 | 0.220301 | -0.6189 | 0.142645 | AFR | Fracture of tibia a | injuries & poisonings |
| 800.4 | 0.700656 | 0.407697 | 1.718569 | 0.085693 | -0.09842 | 1.499728 | AFR | Fracture of patell | injuries & poisonings |
| 801 | -0.06931 | 0.124783 | -0.55545 | 0.578587 | -0.31388 | 0.175259 | AFR | Fracture of ankle | injuries & poisonings |
| 801.1 | -0.05508 | 0.235268 | -0.23411 | 0.814901 | -0.51619 | 0.406039 | AFR | Fracture of foot | injuries & poisonings |
| 802 | 0.044696 | 0.454812 | 0.098275 | 0.921714 | -0.84672 | 0.936111 | AFR | Fracture of pelvis | injuries & poisonings |
| 803 | -0.05626 | 0.144893 | -0.38826 | 0.697821 | -0.34024 | 0.227729 | AFR | Fracture of upper | injuries & poisonings |
| 803.1 | 0.115653 | 0.226914 | 0.509678 | 0.610277 | -0.32909 | 0.560397 | AFR | Fracture of hume | injuries & poisonings |
| 803.2 | -0.05719 | 0.241136 | -0.23716 | 0.812532 | -0.52981 | 0.415429 | AFR | Fracture of radius | injuries & poisonings |
| 803.21 | 0.147182 | 0.617875 | 0.238207 | 0.81172 | -1.06383 | 1.36E+00 | AFR | Colles' fracture | injuries & poisonings |
| 803.3 | 0.343351 | 0.50076 | 0.68566 | 0.492927 | -0.63812 | 1.324824 | AFR | Fracture of clavic | injuries & poisonings |
| 804 | -0.12937 | 0.182803 | -0.70768 | 0.479146 | -0.48765 | 0.228922 | AFR | Fracture of hand | injuries & poisonings |
| 805 | -0.16931 | 0.169232 | -1.00045 | 0.317092 | -0.501 | 0.16238 | AFR | Fracture of vertel | injuries & poisonings |
| 807 | -0.0964 | 0.364327 | -0.26459 | 0.791327 | -0.81047 | 0.617672 | AFR | Fracture of ribs | injuries & poisonings |
| 809 | 0.040444 | 0.056306 | 0.71829 | 0.472579 | -0.06991 | 0.150801 | AFR | Fracture of unspe | injuries & poisonings |
| 816 | -0.98894 | 0.793008 | -1.24707 | 0.212372 | -2.5432 | 0.565331 | AFR | Cerebral laceratio | injuries & poisonings |
| 817 | 0.105722 | 0.209523 | 0.504582 | 0.613852 | -0.30494 | 0.516379 | AFR | Concussion | injuries & poisonings |
| 818 | 0.731056 | 0.518262 | 1.41059 | 0.158365 | -0.28472 | 1.746831 | AFR | Intracranial hemc | injuries & poisonings |
| 818.1 | 2.051041 | 1.096632 | 1.870308 | 0.061441 | -0.09832 | 4.200401 | AFR | Subdural hemorr | injuries & poisonings |
| 818.2 | 0.294815 | 0.678902 | 0.434253 | 0.664105 | -1.03581 | 1.625439 | AFR | Subarachnoid he | injuries & poisonings |
| 819 | -0.05279 | 0.153694 | -0.34347 | 0.731248 | -0.35402 | 0.248447 | AFR | Skull and face fra | injuries & poisonings |
| 830 | 0.002379 | 0.112346 | 0.021172 | 0.983108 | -0.21782 | 0.222572 | AFR | Dislocation | injuries & poisonings |

| | | | | | | | | | |
|---|---|---|---|---|---|---|---|---|---|
| 835 | 0.183702 | 0.123901 | 1.482659 | 0.138165 | -0.05914 | 0.426543 | AFR | Internal deranger | injuries & poisonings |
| 836 | 0.425413 | 0.363449 | 1.17049 | 0.241804 | -0.28693 | 1.137759 | AFR | Traumatic arthro| | injuries & poisonings |
| 840 | 0.037293 | 0.067336 | 0.55383 | 0.579695 | -0.09468 | 0.16927 | AFR | Sprains and strai | injuries & poisonings |
| 840.1 | 0.002797 | 0.173966 | 0.016075 | 0.987174 | -0.33817 | 0.343764 | AFR | Muscle/tendon sp | injuries & poisonings |
| 840.2 | 0.021441 | 0.136564 | 0.157005 | 0.875241 | -0.24622 | 0.289102 | AFR | Rotator cuff (caps | injuries & poisonings |
| 840.3 | -0.18758 | 0.188297 | -0.99618 | 0.319163 | -0.55663 | 0.181478 | AFR | Joint/ligament sp | injuries & poisonings |
| 841 | 0.000841 | 0.077655 | 0.010828 | 0.991361 | -0.15136 | 0.153043 | AFR | Sprains and strai | injuries & poisonings |
| 842 | -0.09464 | 0.078048 | -1.21257 | 0.225293 | -0.24761 | 0.058332 | AFR | Other sprains and | injuries & poisonings |
| 850 | 0.291961 | 0.149473 | 1.953271 | 0.050787 | -0.001 | 0.584923 | AFR | Hemorrhage or h | injuries & poisonings |
| 851 | 0.074532 | 0.107834 | 0.691172 | 0.489458 | -0.13682 | 0.285883 | AFR | Complications of | injuries & poisonings |
| 853 | -0.02605 | 0.290391 | -0.08971 | 0.928514 | -0.59521 | 0.543103 | AFR | Complication of c | injuries & poisonings |
| 854 | 0.21496 | 0.114619 | 1.87544 | 0.060732 | -0.00969 | 0.439609 | AFR | Complications of | injuries & poisonings |
| 855 | 0.023736 | 0.481194 | 0.049327 | 0.960658 | -0.91939 | 0.96686 | AFR | Complication of r | injuries & poisonings |
| 856 | -0.30138 | 0.371623 | -0.81098 | 0.417378 | -1.02975 | 0.426989 | AFR | Vascular complic | injuries & poisonings |
| 857 | -0.05782 | 0.176845 | -0.32696 | 0.743701 | -0.40443 | 0.288788 | AFR | Mechanical comp | injuries & poisonings |
| 858 | 0.079287 | 0.179454 | 0.441824 | 0.658616 | -0.27244 | 0.431011 | AFR | Complication of i | injuries & poisonings |
| 859 | 0.028946 | 0.130157 | 0.222395 | 0.824007 | -0.22616 | 0.284049 | AFR | Complication due | injuries & poisonings |
| 860 | 0.291461 | 0.279324 | 1.043451 | 0.29674 | -0.256 | 0.838927 | AFR | Bone marrow or s | neoplasms |
| 870 | 0.106156 | 0.105185 | 1.00923 | 0.312864 | -0.1 | 0.312316 | AFR | Open wounds of | injuries & poisonings |
| 870.1 | -0.16985 | 0.357617 | -0.47494 | 0.634831 | -0.87076 | 0.53107 | AFR | Open wound or la | injuries & poisonings |
| 870.2 | 0.817046 | 0.421862 | 1.93676 | 0.052775 | -0.00979 | 1.64388 | AFR | Open wound of e | injuries & poisonings |
| 870.3 | -0.09133 | 0.230455 | -0.39631 | 0.691876 | -0.54302 | 0.360352 | AFR | Other open wour | injuries & poisonings |
| 870.4 | 0.607608 | 0.989894 | 0.613812 | 0.53934 | -1.33255 | 2.547765 | AFR | Open wound of n | injuries & poisonings |
| 870.5 | -0.05474 | 0.300603 | -0.18209 | 0.855515 | -0.64391 | 0.534435 | AFR | Open wound of li | injuries & poisonings |
| 870.6 | 0.859238 | 1.311365 | 0.655224 | 0.512323 | -1.71099 | 3.429466 | AFR | Open wound of n | injuries & poisonings |
| 870.8 | 0.227365 | 0.517976 | 0.438948 | 0.660699 | -0.78785 | 1.242579 | AFR | Open wound of g | injuries & poisonings |
| 871 | 0.088175 | 0.095979 | 0.918682 | 0.358262 | -0.09994 | 0.276291 | AFR | Open wounds of | injuries & poisonings |
| 871.1 | 0.395717 | 0.287733 | 1.375294 | 0.16904 | -0.16823 | 0.959663 | AFR | Open wound of h | injuries & poisonings |
| 871.2 | 0.065351 | 0.167853 | 0.389335 | 0.697028 | -0.26364 | 0.394338 | AFR | Open wound of fi | injuries & poisonings |
| 871.3 | -0.38228 | 0.239895 | -1.59355 | 0.111038 | -0.85247 | 0.087902 | AFR | Open wound of fo | injuries & poisonings |
| 871.4 | -0.30435 | 0.284847 | -1.06846 | 0.285314 | -0.86264 | 0.253943 | AFR | Open wound of to | injuries & poisonings |
| 872 | -0.0309 | 0.299401 | -0.10321 | 0.917798 | -0.61772 | 0.555914 | AFR | Traumatic amput | injuries & poisonings |
| 874 | -0.20315 | 0.394398 | -0.51508 | 0.6065 | -0.97615 | 0.569861 | AFR | Complication of a | injuries & poisonings |
| 875 | -0.00566 | 0.369791 | -0.01531 | 0.987781 | -0.73044 | 0.719114 | AFR | Non-healing surg | injuries & poisonings |
| 876 | -0.15586 | 0.260541 | -0.59823 | 0.54969 | -0.66651 | 0.354788 | AFR | Posttraumatic wo | injuries & poisonings |
| 907 | -0.14761 | 0.311796 | -0.47341 | 0.635924 | -0.75872 | 0.463503 | AFR | Injuries to the ne | injuries & poisonings |
| 910 | 0.249403 | 0.549655 | 0.453745 | 0.650013 | -0.8279 | 1.326708 | AFR | Superficial injury, | injuries & poisonings |
| 911 | -0.10588 | 0.243115 | -0.43551 | 0.663194 | -0.58237 | 0.370618 | AFR | Blister | injuries & poisonings |

| | | | | | | | | | | |
|---|---|---|---|---|---|---|---|---|---|---|
| 912 | -0.10841 | 0.143642 | -0.75469 | 0.450435 | -0.38994 | 0.173128 | AFR | | Insect bite | injuries & poisonings |
| 913 | -0.43337 | 0.326463 | -1.32748 | 0.18435 | -1.07323 | 0.206482 | AFR | | Toxic effect of ve | injuries & poisonings |
| 915 | -0.06379 | 0.139267 | -0.45804 | 0.646924 | -0.33675 | 0.209169 | AFR | | Superficial injury | injuries & poisonings |
| 916 | -0.06541 | 0.079955 | -0.81812 | 0.413288 | -0.22212 | 0.091296 | AFR | | Contusion | injuries & poisonings |
| 930 | -0.04478 | 0.093989 | -0.47639 | 0.633799 | -0.22899 | 0.139441 | AFR | | Allergic reaction t | injuries & poisonings |
| 938 | 0.226852 | 0.255868 | 0.886594 | 0.375297 | -0.27464 | 0.728345 | AFR | | Dermatitis due to | dermatologic |
| 938.1 | 0.202353 | 0.456977 | 0.442807 | 0.657905 | -0.69331 | 1.098012 | AFR | | Acute dermatitis | dermatologic |
| 938.2 | 0.004719 | 0.471369 | 0.010011 | 0.992012 | -0.91915 | 0.928584 | AFR | | Chronic dermatiti | dermatologic |
| 939 | -0.05071 | 0.065547 | -0.77368 | 0.439118 | -0.17918 | 0.077757 | AFR | | Atopic/contact de | dermatologic |
| 939.1 | -0.28238 | 0.349602 | -0.80771 | 0.419259 | -0.96758 | 0.402831 | AFR | | Contact and aller | dermatologic |
| 941 | -0.10685 | 0.133439 | -0.80077 | 0.423265 | -0.36839 | 0.154682 | AFR | | Adverse reaction | injuries & poisonings |
| 942 | -0.3172 | 0.25263 | -1.2556 | 0.209261 | -0.81235 | 0.177943 | AFR | | Infusion and tran | injuries & poisonings |
| 946 | -0.04054 | 0.162305 | -0.24977 | 0.802767 | -0.35865 | 0.277573 | AFR | | Anaphylactic sho | injuries & poisonings |
| 947 | 0.135127 | 0.125143 | 1.079775 | 0.280242 | -0.11015 | 0.380403 | AFR | | Urticaria | dermatologic |
| 949 | -0.03434 | 0.068677 | -0.50002 | 0.617063 | -0.16894 | 0.100265 | AFR | | Allergies, other | injuries & poisonings |
| 949.1 | -42.278 | | | | | | AFR | | Diaper or napkin | injuries & poisonings |
| 952 | 0.041089 | 0.51947 | 0.079099 | 0.936954 | -0.97705 | 1.059232 | AFR | | Spinal cord injury | injuries & poisonings |
| 957 | -0.99299 | 0.963672 | -1.03042 | 0.302813 | -2.88175 | 0.895776 | AFR | | Injury to other an | injuries & poisonings |
| 958 | 0.039045 | 0.304718 | 0.128134 | 0.898043 | -0.55819 | 0.636281 | AFR | | Certain early con | injuries & poisonings |
| 958.1 | -0.12506 | 0.760398 | -0.16447 | 0.869363 | -1.61541 | 1.365291 | AFR | | Postoperative sh | injuries & poisonings |
| 958.2 | 0.801698 | 0.63354 | 1.265426 | 0.205719 | -0.44002 | 2.043414 | AFR | | Traumatic and su | injuries & poisonings |
| 960 | -0.00924 | 0.068 | -0.1359 | 0.891898 | -0.14252 | 0.124037 | AFR | | Poisoning by anti | injuries & poisonings |
| 960.1 | -0.54951 | 0.428748 | -1.28167 | 0.199957 | -1.38985 | 0.290816 | AFR | | Adverse effects o | injuries & poisonings |
| 960.2 | -0.08001 | 0.078823 | -1.01502 | 0.310098 | -0.2345 | 0.074483 | AFR | | Allergy/adverse e | injuries & poisonings |
| 960.3 | 0.605189 | 0.971067 | 0.623221 | 0.53314 | -1.29807 | 2.508444 | AFR | | Poisoning by anti | injuries & poisonings |
| 961 | -0.10813 | 0.148557 | -0.7279 | 0.466675 | -0.3993 | 0.183032 | AFR | | Poisoning by othe | injuries & poisonings |
| 961.1 | 0.03565 | 0.097845 | 0.364348 | 0.715598 | -0.15612 | 0.227422 | AFR | | Poisoning/allergy | injuries & poisonings |
| 962 | -0.02787 | 0.126467 | -0.22036 | 0.825593 | -0.27574 | 0.220002 | AFR | | Poisoning by hor | injuries & poisonings |
| 962.1 | -0.10568 | 0.135708 | -0.77872 | 0.436145 | -0.37166 | 0.160305 | AFR | | Adrenal cortical s | injuries & poisonings |
| 962.2 | -0.20472 | 0.358886 | -0.57042 | 0.568393 | -0.90812 | 0.498688 | AFR | | Insulins and antic | injuries & poisonings |
| 962.3 | 0.335804 | 0.388581 | 0.86418 | 0.387489 | -0.4258 | 1.09741 | AFR | | Hormones and sy | injuries & poisonings |
| 963 | -0.0572 | 0.116115 | -0.49257 | 0.622314 | -0.28478 | 0.170386 | AFR | | Poisoning by prir | injuries & poisonings |
| 963.1 | -0.0095 | 0.119231 | -0.07968 | 0.936495 | -0.24319 | 0.224189 | AFR | | Antineoplastic an | injuries & poisonings |
| 964 | 0.289226 | 0.232974 | 1.241448 | 0.21444 | -0.1674 | 0.745847 | AFR | | Poisoning by age | injuries & poisonings |
| 964.1 | 0.521749 | 0.269126 | 1.938677 | 0.052541 | -0.00573 | 1.049227 | AFR | | Anticoagulants ca | injuries & poisonings |
| 965 | 0.109527 | 0.094455 | 1.159568 | 0.246225 | -0.0756 | 0.294655 | AFR | | Poisoning by ana | injuries & poisonings |
| 965.1 | -0.09134 | 0.076773 | -1.18974 | 0.234148 | -0.24181 | 0.059132 | AFR | | Opiates and relat | injuries & poisonings |
| 965.2 | -0.50111 | 0.875562 | -0.57233 | 0.567097 | -2.21718 | 1.214957 | AFR | | Antirheumatics ca | injuries & poisonings |

| | | | | | | | | | |
|---|---|---|---|---|---|---|---|---|---|
| 965.3 | -0.0888 | 0.67355 | -0.13184 | 0.895111 | -1.40893 | 1.231332 | AFR | Salicylates causi | injuries & poisonings |
| 966 | -0.72865 | 0.840961 | -0.86645 | 0.386245 | -2.3769 | 0.919606 | AFR | Poisoning by anti | injuries & poisonings |
| 967 | -0.2262 | 0.171172 | -1.32146 | 0.186348 | -0.56169 | 0.109294 | AFR | Adverse effects c | injuries & poisonings |
| 969 | 0.664132 | 0.366646 | 1.811372 | 0.070083 | -0.05448 | 1.382746 | AFR | Poisoning by psy | injuries & poisonings |
| 972 | 0.294795 | 0.221913 | 1.328425 | 0.184038 | -0.14015 | 0.729736 | AFR | Poisoning by age | injuries & poisonings |
| 972.1 | -0.24779 | 0.488744 | -0.507 | 0.612154 | -1.20571 | 0.710126 | AFR | Cardiac rhythm r | injuries & poisonings |
| 972.2 | -0.07558 | 0.480097 | -0.15743 | 0.874907 | -1.01655 | 0.865392 | AFR | Antilipemic and a | injuries & poisonings |
| 972.6 | 0.572317 | 0.31446 | 1.819998 | 0.068759 | -0.04401 | 1.188648 | AFR | Antihypertensive | injuries & poisonings |
| 973 | 0.228862 | 0.581012 | 0.393902 | 0.693653 | -0.9099 | 1.367623 | AFR | Poisoning by age | injuries & poisonings |
| 974 | -0.44677 | 0.334636 | -1.3351 | 0.181845 | -1.10264 | 0.209103 | AFR | Poisoning by wat | injuries & poisonings |
| 975 | 1.246293 | 1.001397 | 1.244554 | 0.213296 | -0.71641 | 3.208995 | AFR | Poisoning by age | injuries & poisonings |
| 976 | 0.195006 | 1.057032 | 0.184485 | 0.853633 | -1.87674 | 2.26675 | AFR | Poisoning by age | injuries & poisonings |
| 977 | 0.062912 | 0.063279 | 0.994211 | 0.32012 | -0.06111 | 0.186936 | AFR | Personal history | injuries & poisonings |
| 979 | -0.06701 | 0.079489 | -0.84302 | 0.399217 | -0.22281 | 0.088785 | AFR | Adverse drug eve | injuries & poisonings |
| 980 | -0.02699 | 0.117833 | -0.22903 | 0.818849 | -0.25794 | 0.203962 | AFR | Encounter for lon | infectious diseases |
| 981 | 0.714499 | 1.082848 | 0.659833 | 0.509361 | -1.40784 | 2.836841 | AFR | Toxic effect of (no | injuries & poisonings |
| 983 | -2.51541 | 1.893701 | -1.32831 | 0.184077 | -6.227 | 1.196171 | AFR | Toxic effect of co | injuries & poisonings |
| 984 | -0.50208 | 0.747505 | -0.67168 | 0.50179 | -1.96717 | 0.963002 | AFR | Toxic effect of lea | injuries & poisonings |
| 987 | -0.25881 | 0.717673 | -0.36063 | 0.718378 | -1.66543 | 1.1478 | AFR | Toxic effect of oth | injuries & poisonings |
| 988 | -0.02889 | 0.731579 | -0.03949 | 0.968503 | -1.46276 | 1.404981 | AFR | Toxic effect of no | injuries & poisonings |
| 989 | -0.5852 | 0.686605 | -0.85231 | 0.394043 | -1.93092 | 0.760521 | AFR | Toxic effect of oth | injuries & poisonings |
| 990 | 0.087244 | 0.09637 | 0.905304 | 0.365304 | -0.10164 | 0.276126 | AFR | Effects radiation | injuries & poisonings |
| 994 | 0.093666 | 0.073972 | 1.266223 | 0.205433 | -0.05132 | 0.238649 | AFR | Sepsis and SIRS | injuries & poisonings |
| 994.1 | 0.119169 | 0.155244 | 0.767622 | 0.442712 | -0.1851 | 0.423441 | AFR | Systemic inflamm | injuries & poisonings |
| 994.2 | 0.092288 | 0.075948 | 1.215136 | 0.224314 | -0.05657 | 0.241143 | AFR | Sepsis | injuries & poisonings |
| 994.21 | 0.12595 | 0.13924 | 0.904554 | 0.365702 | -0.14695 | 0.398854 | AFR | Septic shock | injuries & poisonings |
| 1000 | -0.17608 | 0.230942 | -0.76242 | 0.445807 | -0.62871 | 0.276563 | AFR | Burns | |
| 1001 | 0.021087 | 0.1989 | 0.106017 | 0.915569 | -0.36875 | 0.410923 | AFR | Foreign body injury | |
| 1002 | -0.0276 | 0.076759 | -0.35961 | 0.719139 | -0.17805 | 0.122842 | AFR | Symptoms concerning nutrition, me | |
| 1004 | 0.163633 | 0.165473 | 0.988881 | 0.322722 | -0.16069 | 0.487955 | AFR | Other signs and symptoms involvir | |
| 1005 | -0.03167 | 0.058251 | -0.54375 | 0.586616 | -0.14584 | 0.082497 | AFR | Other symptoms | |
| 1006 | -0.40922 | 0.540096 | -0.75768 | 0.448642 | -1.46779 | 0.649348 | AFR | Crushing injury | |
| 1007 | -0.75952 | 0.37884 | -2.00485 | 0.04498 | -1.50203 | -0.017 | AFR | Injury to blood vessels | |
| 1008 | -0.09283 | 0.237708 | -0.39052 | 0.696153 | -0.55873 | 0.37307 | AFR | Crushing or internal injury to organ | |
| 1009 | -0.03121 | 0.093309 | -0.33444 | 0.738051 | -0.21409 | 0.151676 | AFR | Injury, NOS | |
| 1010 | -0.13658 | 0.091957 | -1.48522 | 0.137485 | -0.31681 | 0.043656 | AFR | Other tests | |
| 1011 | -0.01931 | 0.092768 | -0.20819 | 0.835084 | -0.20113 | 0.162509 | AFR | Complications of surgical and med | |
| 1012 | -0.00055 | 0.305946 | -0.00179 | 0.998574 | -0.60019 | 0.599097 | AFR | Late effect | |

| | | | | | | | | | |
|---|---|---|---|---|---|---|---|---|---|
| 1013 | -0.08661 | 0.085613 | -1.01164 | 0.311711 | -0.25441 | 0.081189 | AFR | Asphyxia and hypoxemia | |
| 1015 | -0.13376 | 0.084219 | -1.5882 | 0.112241 | -0.29882 | 0.03131 | AFR | Effects of other external causes | |
| 1019 | -0.05958 | 0.098486 | -0.60493 | 0.545223 | -0.25261 | 0.133452 | AFR | Other ill-defined and unknown cau | |
| 1100 | -0.33568 | 0.246316 | -1.36278 | 0.172951 | -0.81845 | 0.147095 | AFR | Family history | |

| phecode | beta | se | zval | pval | ci.lb | ci.ub | QEp | phenotype | category | minuslogp |
|---|---|---|---|---|---|---|---|---|---|---|
| 278.11 | 0.124753 | 0.020594 | 6.057777 | 1.38E-09 | 0.08439 | 0.165117 | 0.457425 | Morbid obesity | endocrine/metab | 8.860121 |
| 496.21 | 0.247105 | 0.041549 | 5.94736 | 2.73E-09 | 0.165671 | 0.328539 | 0.099598 | Obstructive chronic bronchitis | respiratory | 8.563837 |
| 316 | 0.119539 | 0.021846 | 5.471826 | 4.45E-08 | 0.076721 | 0.162357 | 0.561966 | Substance addiction and disorders | mental disorders | 7.35164 |
| 411 | 0.087693 | 0.016737 | 5.239381 | 1.61E-07 | 0.054888 | 0.120497 | 0.809747 | Ischemic Heart Disease | circulatory syster | 6.793174 |
| 228.1 | -0.10319 | 0.020256 | -5.0946 | 3.49E-07 | -0.1429 | -0.06349 | 0.739434 | Hemangioma of skin and subcutaneous tissue | neoplasms | 6.457175 |
| 428.1 | 0.116398 | 0.023122 | 5.034058 | 4.80E-07 | 0.07108 | 0.161717 | 0.33011 | Congestive heart failure (CHF) NOS | circulatory syster | 6.318759 |
| 327.3 | 0.082408 | 0.016431 | 5.015518 | 5.29E-07 | 0.050205 | 0.114612 | 0.229539 | Sleep apnea | neurological | 6.276544 |
| 228 | -0.09089 | 0.019011 | -4.78109 | 1.74E-06 | -0.12816 | -0.05363 | 0.759395 | Hemangioma and lymphangioma, any site | neoplasms | 5.759451 |
| 411.3 | 0.120119 | 0.026502 | 4.532384 | 5.83E-06 | 0.068175 | 0.172063 | 0.711392 | Angina pectoris | circulatory syster | 5.234331 |
| 530 | 0.059912 | 0.013421 | 4.463863 | 8.05E-06 | 0.033606 | 0.086217 | 0.309335 | Diseases of esophagus | digestive | 5.094204 |
| 428 | 0.094017 | 0.021239 | 4.426709 | 9.57E-06 | 0.05239 | 0.135644 | 0.993774 | Congestive heart failure; nonhypertensive | circulatory syster | 5.019088 |
| 338.2 | 0.060631 | 0.013762 | 4.405719 | 1.05E-05 | 0.033658 | 0.087604 | 0.379067 | Chronic pain | neurological | 4.978811 |
| 509 | 0.087334 | 0.019982 | 4.370651 | 1.24E-05 | 0.04817 | 0.126498 | 0.957643 | Respiratory failure, insufficiency, arrest | respiratory | 4.906578 |
| 278.1 | 0.117703 | 0.027118 | 4.340346 | 1.42E-05 | 0.064552 | 0.170854 | 0.119816 | Obesity | endocrine/metab | 4.847712 |
| 411.2 | 0.110514 | 0.025488 | 4.335923 | 1.45E-05 | 0.060558 | 0.16047 | 0.670323 | Myocardial infarction | circulatory syster | 4.838632 |
| 300 | 0.058402 | 0.013677 | 4.270183 | 1.95E-05 | 0.031596 | 0.085207 | 0.866894 | Anxiety disorders | mental disorders | 4.709965 |
| 508 | 0.066654 | 0.015719 | 4.240444 | 2.23E-05 | 0.035846 | 0.097462 | 0.526493 | Pulmonary collapse; interstitial and compensa | respiratory | 4.651695 |
| 416 | 0.081019 | 0.019336 | 4.190086 | 2.79E-05 | 0.043121 | 0.118916 | 0.623463 | Cardiomegaly | circulatory syster | 4.554396 |
| 457 | 0.069165 | 0.016795 | 4.118272 | 3.82E-05 | 0.036248 | 0.102083 | 0.707901 | Encounter for long-term (current) use of antico | circulatory syster | 4.417937 |
| 694.2 | -0.07032 | 0.017138 | -4.10311 | 4.08E-05 | -0.10391 | -0.03673 | 0.609564 | Other dyschromia | dermatologic | 4.38934 |
| 521.1 | 0.248508 | 0.060779 | 4.088733 | 4.34E-05 | 0.129384 | 0.367632 | 0.416178 | Dental caries | digestive | 4.36251 |
| 530.1 | 0.054528 | 0.013436 | 4.058456 | 4.94E-05 | 0.028195 | 0.080862 | 0.382497 | Esophagitis, GERD and related diseases | digestive | 4.306273 |
| 514 | 0.057647 | 0.014349 | 4.017597 | 5.88E-05 | 0.029524 | 0.085769 | 0.620078 | Abnormal findings examination of lungs | respiratory | 4.230623 |
| 172.2 | -0.08002 | 0.020081 | -3.9846 | 6.76E-05 | -0.11938 | -0.04066 | 0.290846 | Other non-epithelial cancer of skin | neoplasms | 4.170053 |
| 71 | 0.260768 | 0.066036 | 3.948903 | 7.85E-05 | 0.131341 | 0.390195 | 0.793157 | Human immunodeficiency virus [HIV] disease | infectious diseas | 4.10513 |
| 355.1 | 0.156311 | 0.039709 | 3.936435 | 8.27E-05 | 0.078483 | 0.234139 | 0.144263 | Chronic pain syndrome | neurological | 4.082494 |
| 401.2 | 0.072572 | 0.018493 | 3.924243 | 8.70E-05 | 0.036326 | 0.108818 | 0.919152 | Hypertensive heart and/or renal disease | circulatory syster | 4.060481 |
| 401.21 | 0.103517 | 0.026495 | 3.907049 | 9.34E-05 | 0.051588 | 0.155446 | 0.605096 | Hypertensive heart disease | circulatory syster | 4.029653 |
| 496.2 | 0.178816 | 0.046345 | 3.858398 | 0.000114 | 0.087982 | 0.26965 | 0.283022 | Chronic bronchitis | respiratory | 3.942589 |
| 509.1 | 0.080154 | 0.020793 | 3.854909 | 0.000116 | 0.039401 | 0.120907 | 0.991107 | Respiratory failure | respiratory | 3.936393 |
| 702.1 | -0.07916 | 0.0206 | -3.84274 | 0.000122 | -0.11954 | -0.03879 | 0.809921 | Actinic keratosis | dermatologic | 3.914827 |
| 702 | -0.06188 | 0.016122 | -3.8383 | 0.000124 | -0.09348 | -0.03028 | 0.500524 | Degenerative skin conditions and other derma | dermatologic | 3.90696 |
| 318 | 0.143569 | 0.037459 | 3.832666 | 0.000127 | 0.07015 | 0.216987 | 0.022547 | Tobacco use disorder | mental disorders | 3.897011 |
| 428.3 | 0.108526 | 0.028433 | 3.816878 | 0.000135 | 0.052798 | 0.164255 | 0.870333 | Heart failure with reduced EF [Systolic or coml | circulatory syster | 3.869181 |
| 418 | 0.053242 | 0.014051 | 3.789267 | 0.000151 | 0.025703 | 0.08078 | 0.510421 | Nonspecific chest pain | circulatory syster | 3.820759 |
| 246.7 | -0.1241 | 0.032814 | -3.78187 | 0.000156 | -0.18842 | -0.05978 | 0.735884 | Abnormal results of function study of thyroid | endocrine/metab | 3.807829 |
| 729.7 | 0.623835 | 0.165642 | 3.766166 | 0.000166 | 0.299183 | 0.948488 | 0.419899 | Nontraumatic compartment syndrome | musculoskeletal | 3.780486 |
| 300.1 | 0.052127 | 0.013863 | 3.76016 | 0.00017 | 0.024956 | 0.079298 | 0.89693 | Anxiety disorder | mental disorders | 3.77005 |
| 962.1 | 0.13847 | 0.036927 | 3.749836 | 0.000177 | 0.066094 | 0.210845 | 0.297736 | Adrenal cortical steroids causing adverse effec | injuries & poisoni | 3.752149 |
| 539 | 0.188359 | 0.050516 | 3.728705 | 0.000192 | 0.08935 | 0.287369 | 0.333171 | Bariatric surgery | digestive | 3.715644 |
| 367 | -0.06934 | 0.018674 | -3.71339 | 0.000204 | -0.10594 | -0.03274 | 0.840593 | Disorders of refraction and accommodation; bl | sense organs | 3.689311 |
| 604.3 | 0.354917 | 0.095719 | 3.707895 | 0.000209 | 0.167311 | 0.542524 | 0.788774 | Peyronie's disease | genitourinary | 3.679877 |
| 385.5 | 0.458107 | 0.125725 | 3.643715 | 0.000269 | 0.21169 | 0.704524 | 0.770676 | Tympanosclerosis and middle ear disease rela | sense organs | 3.570682 |
| 433.12 | 0.275044 | 7.58E-02 | 3.62793 | 0.000286 | 0.126453 | 0.423635 | 0.621328 | Cerebral atherosclerosis | circulatory syster | 3.544087 |
| 78 | -0.07919 | 0.02204 | -3.59303 | 0.000327 | -0.12239 | -0.03599 | 0.962603 | Viral warts & HPV | infectious diseas | 3.485648 |
| 580.31 | 0.121035 | 0.034502 | 3.508 | 0.000451 | 0.053411 | 0.188658 | 0.972583 | Nephritis and nephropathy in diseases classifi | genitourinary | 3.345353 |
| 754.1 | 0.65644 | 1.88E-01 | 3.487873 | 0.000487 | 0.287562 | 1.025318 | 0.663405 | Lumbosacral spondylolysis, congenital | congenital anoma | 3.312579 |
| 457.3 | 0.05888 | 0.016921 | 3.479708 | 0.000502 | 0.025715 | 0.092044 | 0.915057 | Encounter for long-term (current) use of aspiri | circulatory syster | 3.299331 |

| phecode | beta | se | zval | pval | ci.lb | ci.ub | QEp | phenotype | category | minuslogp |
|---|---|---|---|---|---|---|---|---|---|---|
| 530.11 | 0.046788 | 0.013472 | 3.473106 | 0.000514 | 0.020384 | 0.073192 | 0.460156 | GERD | digestive | 3.288638 |
| 740.9 | 0.054581 | 0.01572 | 3.471961 | 0.000517 | 0.023769 | 0.085392 | 0.515647 | Osteoarthrosis NOS | musculoskeletal | 3.286785 |
| 411.1 | 0.150995 | 0.043794 | 3.447829 | 0.000565 | 0.06516 | 0.23683 | 0.26494 | Unstable angina (intermediate coronary syndro | circulatory syster | 3.247865 |
| 428.4 | 0.098613 | 0.028616 | 3.446046 | 0.000569 | 0.042526 | 0.1547 | 0.961076 | Heart failure with preserved EF [Diastolic hear | circulatory syster | 3.244999 |
| 585.1 | 0.062744 | 0.018262 | 3.435739 | 0.000591 | 0.026951 | 0.098537 | 0.99928 | Acute renal failure | genitourinary | 3.228457 |
| 530.2 | 0.09584 | 0.027898 | 3.43536 | 0.000592 | 0.041161 | 0.15052 | 0.729051 | Esophageal bleeding (varices/hemorrhage) | digestive | 3.227849 |
| 716.9 | 0.055446 | 0.016533 | 3.353706 | 0.000797 | 0.023042 | 0.087849 | 0.386905 | Arthropathy NOS | musculoskeletal | 3.098339 |
| 334.21 | 0.513248 | 0.153584 | 3.341813 | 0.000832 | 0.212229 | 0.814266 | 0.245104 | Amyotrophic Lateral Sclerosis | neurological | 3.079704 |
| 521 | 0.198887 | 0.060034 | 3.312925 | 0.000923 | 0.081223 | 0.316552 | 0.494061 | Diseases of hard tissues of teeth | digestive | 3.034677 |
| 286.5 | 0.495328 | 0.150559 | 3.289938 | 0.001002 | 0.200239 | 0.790418 | 0.747695 | Hemorrhagic disorder due to intrinsic circulatin | hematopoietic | 2.999092 |
| 740 | 0.049612 | 0.015163 | 3.271969 | 0.001068 | 0.019894 | 0.07933 | 0.742379 | Osteoarthrosis | musculoskeletal | 2.971424 |
| 503 | 0.077085 | 0.023621 | 3.263359 | 0.001101 | 0.030788 | 0.123382 | 0.498574 | Pulmonary congestion and hypostasis | respiratory | 2.958212 |
| 225.2 | 0.555809 | 0.170368 | 3.262409 | 0.001105 | 0.221895 | 0.889724 | 0.245545 | Benign neoplasm of spinal cord, meninges | neoplasms | 2.956758 |
| 585.3 | 0.058614 | 0.018045 | 3.248311 | 0.001161 | 0.023248 | 0.093981 | 0.918483 | Chronic renal failure [CKD] | genitourinary | 2.935196 |
| 222 | 0.548093 | 0.169623 | 3.231244 | 0.001233 | 0.215638 | 0.880548 | 0.711744 | Benign neoplasm of male genital organs | neoplasms | 2.909203 |
| 789.1 | 0.152578 | 0.04737 | 3.22096 | 0.001278 | 0.059734 | 0.245423 | 0.982343 | Persistent vomiting | symptoms | 2.893598 |
| 526.5 | 0.377711 | 0.11825 | 3.194185 | 0.001402 | 0.145946 | 0.609476 | 0.895923 | Inflammatory conditions of jaw | digestive | 2.853171 |
| 149.3 | 0.547165 | 0.172077 | 3.179778 | 0.001474 | 0.209901 | 0.884429 | 0.889459 | Cancer of hypopharynx | neoplasms | 2.831538 |
| 327.1 | 0.085908 | 0.027023 | 3.179066 | 0.001478 | 0.032944 | 0.138872 | 0.813772 | Hypersomnia | neurological | 2.830471 |
| 721 | 0.067873 | 0.021405 | 3.170817 | 0.00152 | 0.025919 | 0.109827 | 0.45682 | Spondylosis and allied disorders | musculoskeletal | 2.818125 |
| 580.13 | 0.542235 | 0.171068 | 3.169705 | 0.001526 | 0.206948 | 0.877521 | 0.735686 | Acute glomerulonephritis, NOS | genitourinary | 2.816463 |
| 250.6 | 0.096508 | 0.030539 | 3.160156 | 0.001577 | 0.036653 | 0.156363 | 0.956814 | Polyneuropathy in diabetes | endocrine/metab | 2.80221 |
| 217.1 | -0.0785 | 0.02493 | -3.14873 | 0.00164 | -0.12736 | -0.02964 | 0.547968 | Nevus, non-neoplastic | neoplasms | 2.785203 |
| 510 | 0.060408 | 0.019281 | 3.133115 | 0.00173 | 0.022619 | 0.098198 | 0.603799 | Other diseases of lung | respiratory | 2.762051 |
| 411.8 | 0.111064 | 0.035532 | 3.125763 | 0.001773 | 0.041423 | 0.180705 | 0.552748 | Other chronic ischemic heart disease, unspeci | circulatory syster | 2.751182 |
| 276.41 | 0.075596 | 0.024208 | 3.12275 | 0.001792 | 0.028149 | 0.123044 | 0.966209 | Acidosis | endocrine/metab | 2.746735 |
| 276.14 | 0.070039 | 0.022468 | 3.117247 | 0.001825 | 0.026002 | 0.114077 | 0.520982 | Hypopotassemia | endocrine/metab | 2.738622 |
| 428.2 | 0.097624 | 0.031344 | 3.114609 | 0.001842 | 0.036191 | 0.159057 | 0.583702 | Heart failure NOS | circulatory syster | 2.734736 |
| 365.5 | 0.46401 | 0.150016 | 3.093078 | 0.001981 | 0.169985 | 0.758035 | 0.754943 | Pseudoexfoliation glaucoma | sense organs | 2.703133 |
| 721.1 | 0.067593 | 0.021885 | 3.088616 | 0.002011 | 0.0247 | 0.110487 | 0.44317 | Spondylosis without myelopathy | musculoskeletal | 2.696608 |
| 509.8 | 0.109369 | 0.035481 | 3.082491 | 0.002053 | 0.039828 | 0.17891 | 0.586919 | Dependence on respirator [Ventilator] or suppl | respiratory | 2.687662 |
| 276.6 | 0.079867 | 0.026254 | 3.04203 | 0.00235 | 0.028409 | 0.131325 | 0.773042 | Fluid overload | endocrine/metab | 2.628953 |
| 765 | 0.075619 | 0.024858 | 3.042063 | 0.00235 | 0.026899 | 0.12434 | 0.570862 | Cervical radiculitis | symptoms | 2.629001 |
| 962 | 0.105171 | 0.034691 | 3.031623 | 0.002432 | 0.037177 | 0.173165 | 0.453108 | Poisoning by hormones and synthetic substitu | injuries & poisoni | 2.613959 |
| 818.2 | 0.430635 | 0.142097 | 3.030577 | 0.002441 | 0.152131 | 0.70914 | 0.524437 | Subarachnoid hemorrhage (injury) | injuries & poisoni | 2.612455 |
| 427.21 | 0.062909 | 0.020766 | 3.029386 | 0.002451 | 0.022208 | 0.10361 | 0.787897 | Atrial fibrillation | circulatory syster | 2.610743 |
| 286.7 | 0.091198 | 0.030341 | 3.005764 | 0.002649 | 0.031731 | 0.150666 | 0.832893 | Other and unspecified coagulation defects | hematopoietic | 2.576894 |
| 741.5 | 0.500814 | 0.166636 | 3.005436 | 0.002652 | 0.174213 | 0.827415 | 0.55468 | Hemarthrosis | musculoskeletal | 2.576425 |
| 426 | 0.047324 | 0.015763 | 3.002148 | 0.002681 | 0.016428 | 0.07822 | 0.976947 | Cardiac conduction disorders | circulatory syster | 2.571733 |
| 702.2 | -0.05493 | 0.018382 | -2.98826 | 0.002806 | -0.09096 | -0.0189 | 0.613342 | Seborrheic keratosis | dermatologic | 2.55196 |
| 743.11 | -0.06487 | 0.021742 | -2.98357 | 0.002849 | -0.10748 | -0.02225 | 0.53403 | Osteoporosis NOS | musculoskeletal | 2.545292 |
| 740.1 | 0.047039 | 0.01577 | 2.982859 | 0.002856 | 0.016131 | 0.077947 | 0.491461 | Osteoarthritis; localized | musculoskeletal | 2.544288 |
| 874 | 0.417904 | 0.141237 | 2.958884 | 0.003088 | 0.141085 | 0.694723 | 0.293405 | Complication of amputation stump | injuries & poisoni | 2.510386 |
| 411.4 | 0.092125 | 0.031147 | 2.957738 | 0.003099 | 0.031078 | 0.153171 | 0.100627 | Coronary atherosclerosis | circulatory syster | 2.508771 |
| 964.1 | 0.285329 | 0.096833 | 2.946591 | 0.003213 | 0.095539 | 0.475119 | 0.646997 | Anticoagulants causing adverse effects | injuries & poisoni | 2.493093 |
| 754 | 0.147102 | 0.050125 | 2.934725 | 0.003338 | 0.048859 | 0.245344 | 0.919245 | Congenital musculoskeletal deformities of spir | congenital anoma | 2.476457 |
| 189.4 | 0.247508 | 0.08439 | 2.932912 | 0.003358 | 0.082107 | 0.412909 | 0.474334 | Malignant neoplasm of other urinary organs | neoplasms | 2.47392 |
| 769 | 0.457913 | 0.156185 | 2.931857 | 0.003369 | 0.151795 | 0.764031 | 0.84523 | Nonallopathic lesions NEC | symptoms | 2.472445 |

| phecode | beta | se | zval | pval | ci.lb | ci.ub | QEp | phenotype | category | minuslogp |
|---|---|---|---|---|---|---|---|---|---|---|
| 337.1 | 0.193472 | 0.066335 | 2.91658 | 0.003539 | 0.063457 | 0.323486 | 0.988606 | Peripheral autonomic neuropathy | neurological | 2.451129 |
| 250.24 | 0.087892 | 0.030191 | 2.911237 | 0.0036 | 0.02872 | 0.147065 | 0.516522 | Type 2 diabetes with neurological manifestatio | endocrine/metab | 2.443697 |
| 525 | 0.1425 | 0.048991 | 2.908728 | 0.003629 | 0.04648 | 0.23852 | 0.701233 | Other diseases of the teeth and supporting str | digestive | 2.44021 |
| 279.1 | 0.061164 | 0.021079 | 2.901632 | 0.003712 | 0.01985 | 0.102479 | 0.510328 | Immunity deficiency | endocrine/metab | 2.430363 |
| 270.2 | 0.278399 | 0.096527 | 2.884149 | 0.003925 | 0.089209 | 0.467589 | 0.732123 | Disorders of amino-acid metabolism | endocrine/metab | 2.406191 |
| 276.13 | 0.063472 | 0.022018 | 2.882716 | 0.003943 | 0.020317 | 0.106627 | 0.51977 | Hyperpotassemia | endocrine/metab | 2.404215 |
| 149.4 | 0.292475 | 0.101466 | 2.882484 | 0.003946 | 0.093605 | 0.491346 | 0.863968 | Cancer of larynx | neoplasms | 2.403895 |
| 427.2 | 0.058776 | 0.020412 | 2.87955 | 0.003982 | 0.01877 | 0.098782 | 0.862522 | Atrial fibrillation and flutter | circulatory syster | 2.399851 |
| 710 | 0.134141 | 0.046584 | 2.879566 | 0.003982 | 0.042839 | 0.225443 | 0.802042 | Osteomyelitis, periostitis, and other infections | musculoskeletal | 2.399874 |
| 972.2 | 0.313261 | 0.108877 | 2.877196 | 0.004012 | 0.099866 | 0.526656 | 0.406627 | Antilipemic and antiarteriosclerotic drugs caus | injuries & poisoni | 2.39661 |
| 296.22 | 0.064099 | 0.022335 | 2.869882 | 0.004106 | 0.020323 | 0.107875 | 0.29743 | Major depressive disorder | mental disorders | 2.386554 |
| 133 | 0.394782 | 0.137579 | 2.869488 | 0.004111 | 0.125132 | 0.664433 | 0.27822 | Arthropod-borne diseases | infectious diseas | 2.386014 |
| 446.3 | 0.47395 | 0.165418 | 2.865174 | 0.004168 | 0.149738 | 0.798163 | 0.648734 | Hypersensitivity angiitis | circulatory syster | 2.380093 |
| 571.81 | 0.139793 | 0.048946 | 2.856074 | 0.004289 | 0.043861 | 0.235726 | 0.367107 | Portal hypertension | digestive | 2.367629 |
| 279 | 0.058552 | 0.020525 | 2.852719 | 0.004335 | 0.018324 | 0.09878 | 0.458293 | Disorders involving the immune mechanism | endocrine/metab | 2.363042 |
| 270.21 | 0.344566 | 0.12131 | 2.840367 | 0.004506 | 0.106802 | 0.58233 | 0.459917 | Disorders of urea cycle metabolism | endocrine/metab | 2.346192 |
| 736.1 | 0.375288 | 0.132338 | 2.83583 | 0.004571 | 0.11591 | 0.634666 | 0.535283 | Acquired deformities of forearm | musculoskeletal | 2.340019 |
| 694 | -0.06171 | 0.021888 | -2.81937 | 0.004812 | -0.10461 | -0.01881 | 0.441462 | Dyschromia and Vitiligo | dermatologic | 2.317699 |
| 562 | 0.048846 | 0.017371 | 2.811947 | 0.004924 | 0.0148 | 0.082893 | 0.774751 | Diverticulosis and diverticulitis | digestive | 2.307659 |
| 352.1 | 0.239886 | 0.085341 | 2.810921 | 0.00494 | 0.072621 | 0.40715 | 0.410443 | Trigeminal nerve disorders [CN5] | neurological | 2.306274 |
| 763 | 0.054814 | 0.019613 | 2.79474 | 0.005194 | 0.016373 | 0.093255 | 0.488006 | Thoracic or lumbosacral neuritis or radiculitis, | symptoms | 2.284486 |
| 994 | 0.056475 | 0.020243 | 2.789833 | 0.005274 | 0.016799 | 0.09615 | 0.608145 | Sepsis and SIRS | injuries & poisoni | 2.277899 |
| 426.92 | 0.116098 | 0.041792 | 2.777957 | 0.00547 | 0.034186 | 0.198009 | 0.447722 | Cardiac defibrillator in situ | circulatory syster | 2.261998 |
| 187 | 0.234419 | 0.084446 | 2.775952 | 0.005504 | 0.068907 | 0.399931 | 0.293134 | Cancer of other male genital organs | neoplasms | 2.259319 |
| 296.2 | 0.053693 | 0.019348 | 2.775177 | 0.005517 | 0.015772 | 0.091614 | 0.361319 | Depression | mental disorders | 2.258284 |
| 975 | 0.459429 | 0.165547 | 2.775215 | 0.005517 | 0.134963 | 0.783896 | 0.830731 | Poisoning by agents primarily acting on the sm | injuries & poisoni | 2.258334 |
| 351 | 0.046508 | 0.016807 | 2.767128 | 0.005655 | 0.013566 | 0.079449 | 0.311888 | Other peripheral nerve disorders | neurological | 2.247547 |
| 256.1 | 0.710579 | 0.257792 | 2.7564 | 0.005844 | 0.205315 | 1.215843 | 0.25518 | Hyperestrogenism | endocrine/metab | 2.233279 |
| 286 | 0.057662 | 0.020923 | 2.755953 | 0.005852 | 0.016654 | 0.098669 | 0.58186 | Coagulation defects | hematopoietic | 2.232685 |
| 426.91 | 0.089909 | 0.032927 | 2.730582 | 0.006322 | 0.025374 | 0.154444 | 0.965844 | Cardiac pacemaker in situ | circulatory syster | 2.199128 |
| 771.1 | 0.047469 | 0.017396 | 2.728687 | 0.006359 | 0.013373 | 0.081565 | 0.450614 | Swelling of limb | symptoms | 2.196632 |
| 480.2 | 0.09663 | 0.035417 | 2.728331 | 0.006366 | 0.027214 | 0.166047 | 0.470747 | Viral pneumonia | respiratory | 2.196162 |
| 604.2 | 0.899564 | 3.31E-01 | 2.721049 | 0.006508 | 0.251611 | 1.547518 | 0.251684 | Vascular disorders of penis | genitourinary | 2.186585 |
| 726.4 | 0.09817 | 0.036151 | 2.715553 | 0.006617 | 0.027315 | 0.169024 | 0.776381 | Calcaneal spur; Exostosis NOS | musculoskeletal | 2.179371 |
| 797 | 0.081539 | 0.030057 | 2.712789 | 0.006672 | 0.022628 | 0.14045 | 0.949003 | Shock | symptoms | 2.175747 |
| 994.2 | 0.055842 | 0.020666 | 2.702113 | 0.00689 | 0.015337 | 0.096346 | 0.686594 | Sepsis | injuries & poisoni | 2.161779 |
| 729 | 0.049377 | 0.018323 | 2.694796 | 0.007043 | 0.013465 | 0.08529 | 0.334517 | Other disorders of soft tissues | musculoskeletal | 2.152232 |
| 426.7 | 0.045032 | 0.016724 | 2.692602 | 0.00709 | 0.012253 | 0.077811 | 0.991742 | Abnormal electrocardiogram [ECG] [EKG] | circulatory syster | 2.149373 |
| 360.3 | 0.458808 | 0.170435 | 2.691978 | 0.007103 | 0.124761 | 0.792856 | 0.666307 | Hypotony of eye | sense organs | 2.148561 |
| 249 | 0.078959 | 0.029338 | 2.691366 | 0.007116 | 0.021458 | 0.136461 | 0.465735 | Secondary diabetes mellitus | endocrine/metab | 2.147763 |
| 285.3 | 0.680787 | 0.252999 | 2.690868 | 0.007127 | 0.184918 | 1.176656 | 0.880346 | Sideroblastic anemia | hematopoietic | 2.147116 |
| 480 | 0.04724 | 0.01758 | 2.687147 | 0.007207 | 0.012784 | 0.081697 | 0.936066 | Pneumonia | respiratory | 2.142274 |
| 187.2 | 0.273073 | 0.101673 | 2.685804 | 0.007236 | 0.073798 | 0.472347 | 0.563406 | Malignant neoplasm of testis | neoplasms | 2.140528 |
| 288 | 0.046959 | 0.017488 | 2.685189 | 0.007249 | 0.012683 | 0.081235 | 0.975303 | Diseases of white blood cells | hematopoietic | 2.139729 |
| 562.2 | 0.096562 | 0.035973 | 2.684318 | 0.007268 | 0.026057 | 0.167067 | 0.831404 | Diverticulitis | digestive | 2.138598 |
| 244.2 | -0.06356 | 0.023718 | -2.67987 | 0.007365 | -0.11005 | -0.01707 | 0.785331 | Acquired hypothyroidism | endocrine/metab | 2.132826 |
| 284.2 | 0.643421 | 0.240633 | 2.67387 | 0.007498 | 0.171789 | 1.115053 | 0.384955 | Constitutional aplastic anemia | hematopoietic | 2.125045 |
| 738 | 0.069285 | 0.025928 | 2.672179 | 0.007536 | 0.018467 | 0.120104 | 0.6238 | Other acquired musculoskeletal deformity | musculoskeletal | 2.122857 |

| phecode | beta | se | zval | pval | ci.lb | ci.ub | QEp | phenotype | category | minuslogp |
|---|---|---|---|---|---|---|---|---|---|---|
| 71.1 | 0.23939 | 0.089837 | 2.664718 | 0.007705 | 0.063313 | 0.415467 | 0.366469 | HIV infection, symptomatic | infectious diseas | 2.113211 |
| 577.2 | 0.167203 | 0.063006 | 2.653769 | 0.00796 | 0.043714 | 0.290691 | 0.806358 | Chronic pancreatitis | digestive | 2.099096 |
| 276.4 | 0.060575 | 0.022834 | 2.652869 | 0.007981 | 0.015822 | 0.105329 | 0.83808 | Acid-base balance disorder | endocrine/metab | 2.097938 |
| 502 | 0.07707 | 0.029063 | 2.651793 | 0.008007 | 0.020107 | 0.134034 | 0.811785 | Postinflammatory pulmonary fibrosis | respiratory | 2.096554 |
| 446.5 | 0.284316 | 0.107434 | 2.646423 | 0.008135 | 0.073749 | 0.494883 | 0.905566 | Giant cell arteritis | circulatory syster | 2.089652 |
| 740.11 | 0.046243 | 0.0176 | 2.627399 | 0.008604 | 0.011747 | 0.080739 | 0.410975 | Osteoarthrosis, localized, primary | musculoskeletal | 2.065298 |
| 296 | 0.048861 | 0.018646 | 2.620543 | 0.008779 | 0.012317 | 0.085406 | 0.385115 | Mood disorders | mental disorders | 2.056556 |
| 288.2 | 0.047497 | 0.018141 | 2.618252 | 0.008838 | 0.011942 | 0.083052 | 0.684473 | Elevated white blood cell count | hematopoietic | 2.053638 |
| 276.1 | 0.041491 | 0.015857 | 2.616575 | 0.008882 | 0.010412 | 0.072571 | 0.512089 | Electrolyte imbalance | endocrine/metab | 2.051505 |
| 870.8 | 0.441433 | 0.1691 | 2.610493 | 0.009041 | 0.110004 | 0.772862 | 0.293433 | Open wound of genital organs | injuries & poisoni | 2.043775 |
| 509.5 | 0.446331 | 0.171686 | 2.599692 | 0.009331 | 0.109832 | 0.782829 | 0.41292 | Respiratory arrest | respiratory | 2.030084 |
| 352 | 0.112715 | 0.043361 | 2.59945 | 0.009337 | 0.027729 | 0.197702 | 0.533945 | Disorders of other cranial nerves | neurological | 2.029778 |
| 371 | -0.05104 | 0.019747 | -2.58467 | 0.009747 | -0.08974 | -0.01234 | 0.780035 | Inflammation of the eye | sense organs | 2.011117 |
| 513.8 | 0.077015 | 0.029837 | 2.581188 | 0.009846 | 0.018535 | 0.135494 | 0.592004 | Disorders of diaphragm | respiratory | 2.006736 |
| 585 | 0.04128 | 0.016064 | 2.569712 | 0.010178 | 0.009795 | 0.072764 | 0.825835 | Renal failure | genitourinary | 1.992325 |
| 580.3 | 0.072735 | 0.028457 | 2.555958 | 0.01059 | 0.01696 | 0.12851 | 0.898541 | Nephritis and nephropathy without mention of | genitourinary | 1.975121 |
| 454.11 | 0.08534 | 0.033389 | 2.55592 | 0.010591 | 0.019898 | 0.150782 | 0.482186 | Varicose veins of lower extremity, symptomtic | circulatory syster | 1.975073 |
| 870.6 | 0.439086 | 0.172173 | 2.550253 | 0.010764 | 0.101632 | 0.77654 | 0.907376 | Open wound of neck | injuries & poisoni | 1.968007 |
| 255.22 | 0.921425 | 0.361354 | 2.549923 | 0.010775 | 0.213184 | 1.629666 | 0.452879 | Mineralocorticoid deficiency | endocrine/metab | 1.967596 |
| 977 | 0.050318 | 0.019754 | 2.547258 | 0.010857 | 0.011601 | 0.089034 | 0.324699 | Personal history of allergy to medicinal agents | injuries & poisoni | 1.964278 |
| 420.1 | 0.223583 | 0.087848 | 2.545107 | 0.010924 | 0.051404 | 0.395762 | 0.946818 | Myocarditis | circulatory syster | 1.961602 |
| 580 | 0.066379 | 0.026103 | 2.542976 | 0.010991 | 0.015218 | 0.11754 | 0.683624 | Nephritis; nephrosis; renal sclerosis | genitourinary | 1.958952 |
| 782.3 | 0.043638 | 0.017173 | 2.541108 | 0.01105 | 0.00998 | 0.077295 | 0.435296 | Edema | symptoms | 1.956631 |
| 619 | -0.04703 | 0.018538 | -2.53713 | 0.011177 | -0.08337 | -0.0107 | 0.914211 | Noninflammatory female genital disorders | genitourinary | 1.951691 |
| 70.3 | 0.115678 | 0.045681 | 2.53E+00 | 0.011333 | 0.026144 | 0.205212 | 0.308492 | Viral hepatitis C | infectious diseas | 1.945668 |
| 1013 | 0.075301 | 0.029811 | 2.525924 | 0.011539 | 0.016872 | 0.13373 | 0.152931 | Asphyxia and hypoxemia | other | 1.937815 |
| 530.14 | 0.068578 | 0.027151 | 2.525836 | 0.011542 | 0.015364 | 0.121792 | 0.682985 | Reflux esophagitis | digestive | 1.937706 |
| 760 | 0.038832 | 0.015395 | 2.52234 | 0.011658 | 0.008658 | 0.069007 | 0.459852 | Back pain | symptoms | 1.933387 |
| 855 | 0.291149 | 0.115444 | 2.52198 | 0.01167 | 0.064882 | 0.517416 | 0.489141 | Complication of nervous system device, implai | injuries & poisoni | 1.932943 |
| 260.6 | -0.08589 | 0.034123 | -2.51692 | 0.011839 | -0.15277 | -0.01901 | 0.634034 | Anorexia | endocrine/metab | 1.926701 |
| 512.2 | 0.057294 | 0.022772 | 2.515959 | 0.011871 | 0.012661 | 0.101927 | 0.705913 | Painful respiration | respiratory | 1.925516 |
| 367.1 | -0.07858 | 0.031398 | -2.50267 | 0.012326 | -0.14012 | -0.01704 | 0.581218 | Myopia | sense organs | 1.909177 |
| 695.21 | 0.569986 | 0.228012 | 2.499806 | 0.012426 | 0.123091 | 1.016882 | 0.657875 | Dermatitis herpetiformis | dermatologic | 1.905664 |
| 695.9 | -0.14773 | 0.059227 | -2.49424 | 0.012623 | -0.26381 | -0.03164 | 0.540139 | Unspecified erythematous condition | dermatologic | 1.898848 |
| 519.2 | 0.16632 | 0.066708 | 2.493259 | 0.012658 | 0.035575 | 0.297065 | 0.956018 | Respiratory complications | respiratory | 1.897647 |
| 250.13 | 0.271574 | 1.09E-01 | 2.48905 | 0.012808 | 0.057727 | 0.485421 | 0.35192 | Type 1 diabetes with ophthalmic manifestation | endocrine/metab | 1.892503 |
| 250.23 | 0.086422 | 3.47E-02 | 2.488429 | 0.012831 | 0.018353 | 0.154491 | 0.933308 | Type 2 diabetes with ophthalmic manifestation | endocrine/metab | 1.891743 |
| 433.32 | 0.602501 | 0.242436 | 2.4852 | 0.012948 | 0.127336 | 1.077667 | 0.226962 | Moyamoya disease | circulatory syster | 1.887801 |
| 715 | 0.070067 | 0.02832 | 2.474103 | 0.013357 | 0.014561 | 0.125574 | 0.686914 | Other inflammatory spondylopathies | musculoskeletal | 1.874288 |
| 130.1 | -0.31817 | 0.129004 | -2.46634 | 0.01365 | -0.57101 | -0.06532 | 0.860001 | Lyme disease | infectious diseas | 1.864859 |
| 851 | 0.079963 | 0.032464 | 2.463115 | 0.013774 | 0.016334 | 0.143592 | 0.610354 | Complications of transplants and reattached lii | injuries & poisoni | 1.860953 |
| 716 | 0.0477 | 0.019383 | 2.460941 | 0.013857 | 0.00971 | 0.085689 | 0.365681 | Other arthropathies | musculoskeletal | 1.858321 |
| 961.1 | -0.06121 | 0.025163 | -2.43241 | 0.014999 | -0.11052 | -0.01189 | 0.398143 | Poisoning/allergy of sulfonamides | injuries & poisoni | 1.823941 |
| 562.1 | 0.042465 | 0.017567 | 2.417313 | 0.015636 | 0.008034 | 0.076896 | 0.852272 | Diverticulosis | digestive | 1.805887 |
| 278 | 0.077169 | 0.031957 | 2.414743 | 0.015746 | 0.014534 | 0.139804 | 0.044549 | Overweight, obesity and other hyperalimentati | endocrine/metab | 1.802821 |
| 38 | 0.048639 | 0.020186 | 2.41E+00 | 0.015975 | 0.009074 | 0.088204 | 0.75765 | Septicemia | infectious diseas | 1.796555 |
| 754.2 | 0.139207 | 0.058011 | 2.399678 | 0.01641 | 0.025508 | 0.252906 | 0.289807 | Spondylolisthesis, congenital | congenital anoma | 1.784904 |
| 260.2 | 0.080751 | 0.033697 | 2.396365 | 0.016559 | 0.014705 | 0.146796 | 0.446005 | severe protein-calorie malnutrition | endocrine/metab | 1.780977 |

| phecode | beta | se | zval | pval | ci.lb | ci.ub | QEp | phenotype | category | minuslogp |
|---|---|---|---|---|---|---|---|---|---|---|
| 338.1 | 0.050239 | 0.020971 | 2.395627 | 0.016592 | 0.009136 | 0.091342 | 0.16051 | Acute pain | neurological | 1.780102 |
| 433.6 | 0.379864 | 1.59E-01 | 2.390337 | 0.016833 | 0.068393 | 0.691335 | 0.233218 | Acute, but ill-defined cerebrovascular disease | circulatory syster | 1.77384 |
| 695.41 | 0.225861 | 0.095137 | 2.374068 | 0.017593 | 0.039396 | 0.412325 | 0.28817 | Cutaneous lupus erythematosus | dermatologic | 1.754653 |
| 277.8 | 0.730662 | 0.309334 | 2.362047 | 0.018174 | 0.124378 | 1.336946 | 0.862807 | Carnitine deficiencies | endocrine/metab | 1.740541 |
| 368.5 | 0.544117 | 0.230837 | 2.35715 | 0.018416 | 0.091685 | 0.996549 | 0.180314 | Color vision deficiencies | sense organs | 1.734809 |
| 327 | 0.033973 | 0.014475 | 2.347018 | 0.018924 | 0.005603 | 0.062344 | 0.575757 | Sleep disorders | neurological | 1.722979 |
| 856 | 0.259165 | 0.110429 | 2.346896 | 0.018931 | 0.042728 | 0.475602 | 0.269172 | Vascular complications of surgery and medica | injuries & poisoni | 1.722837 |
| 291.8 | 0.06722 | 0.028723 | 2.34029 | 0.019269 | 0.010924 | 0.123516 | 0.447397 | Alteration of consciousness | mental disorders | 1.715146 |
| 155.1 | 0.0968 | 0.041376 | 2.339515 | 0.019309 | 0.015704 | 0.177896 | 0.909458 | Malignant neoplasm of liver, primary | neoplasms | 1.714245 |
| 255.12 | 0.254065 | 0.108861 | 2.333843 | 1.96E-02 | 0.040701 | 0.467429 | 0.639789 | Hyperaldosteronism | endocrine/metab | 1.707744 |
| 981 | 0.64287 | 0.275676 | 2.331972 | 0.019702 | 0.102554 | 1.183185 | 0.946751 | Toxic effect of (non-ethyl) alcohol and petroleu | injuries & poisoni | 1.705486 |
| 618.1 | 0.097191 | 0.041681 | 2.331774 | 0.019713 | 0.015498 | 0.178885 | 0.34061 | Prolapse of vaginal walls | genitourinary | 1.705257 |
| 433 | 0.043083 | 0.018562 | 2.320962 | 0.020289 | 0.006701 | 0.079464 | 0.900804 | Cerebrovascular disease | circulatory syster | 1.692742 |
| 475 | -0.04061 | 0.017526 | -2.31719 | 0.020493 | -0.07496 | -0.00626 | 0.844502 | Chronic sinusitis | respiratory | 1.688388 |
| 516.1 | 0.102187 | 0.044138 | 2.315166 | 0.020604 | 0.015678 | 0.188696 | 0.809424 | Hemoptysis | respiratory | 1.686052 |
| 364.41 | 0.275382 | 0.119042 | 2.313321 | 0.020705 | 0.042064 | 0.5087 | 0.977895 | Keratoconus | sense organs | 1.683925 |
| 362 | -0.05193 | 0.022455 | -2.31263 | 0.020743 | -0.09594 | -0.00792 | 0.492699 | Other retinal disorders | sense organs | 1.68313 |
| 425.1 | 0.063988 | 0.027732 | 2.307387 | 0.021033 | 0.009635 | 0.118341 | 0.794245 | Primary/intrinsic cardiomyopathies | circulatory syster | 1.677093 |
| 250.25 | 0.096035 | 0.041673 | 2.304479 | 0.021196 | 0.014357 | 0.177713 | 0.249357 | Diabetes type 2 with peripheral circulatory disc | endocrine/metab | 1.673751 |
| 480.1 | 0.063873 | 0.027755 | 2.3013 | 0.021375 | 0.009474 | 0.118272 | 0.561782 | Bacterial pneumonia | respiratory | 1.670101 |
| 285.1 | 0.069607 | 0.030284 | 2.298473 | 0.021535 | 0.010251 | 0.128963 | 0.121784 | Acute posthemorrhagic anemia | hematopoietic | 1.666858 |
| 1004 | 0.092418 | 0.040262 | 2.295436 | 0.021708 | 0.013507 | 0.171329 | 0.673826 | Other signs and symptoms involving emotiona | other | 1.663377 |
| 571.5 | 0.055783 | 0.024373 | 2.28869 | 0.022097 | 0.008012 | 0.103553 | 0.241997 | Other chronic nonalcoholic liver disease | digestive | 1.65566 |
| 190 | 0.241285 | 0.105522 | 2.286573 | 0.022221 | 0.034465 | 0.448105 | 0.59064 | Cancer of eye | neoplasms | 1.653241 |
| 250.12 | 0.193252 | 0.084663 | 2.282604 | 0.022454 | 0.027316 | 0.359189 | 0.485292 | Type 1 diabetes with renal manifestations | endocrine/metab | 1.648712 |
| 870.2 | 0.439375 | 0.193284 | 2.273212 | 0.023013 | 0.060546 | 0.818203 | 0.109578 | Open wound of ear | injuries & poisoni | 1.638019 |
| 1011 | 0.057668 | 0.025485 | 2.262809 | 0.023647 | 0.007718 | 0.107618 | 0.728046 | Complications of surgical and medical procedu | other | 1.626215 |
| 573 | 0.041791 | 0.018604 | 2.246389 | 0.024679 | 0.005329 | 0.078254 | 0.891133 | Other disorders of liver | digestive | 1.60767 |
| 327.32 | 0.083588 | 0.03724 | 2.244581 | 0.024795 | 0.010599 | 0.156576 | 0.069079 | Obstructive sleep apnea | neurological | 1.605635 |
| 145.3 | 0.229383 | 0.10254 | 2.237013 | 0.025285 | 0.028409 | 0.430357 | 0.464932 | Cancer of major salivary glands | neoplasms | 1.597129 |
| 312.3 | 0.36339 | 0.16253 | 2.23583 | 0.025363 | 0.044837 | 0.681944 | 0.835079 | Impulse control disorder | mental disorders | 1.595801 |
| 958.2 | 0.338108 | 0.151877 | 2.226198 | 0.026001 | 0.040435 | 0.635781 | 0.250422 | Traumatic and surgical subcutaneous emphys | injuries & poisoni | 1.585011 |
| 394.1 | -0.14012 | 0.063072 | -2.22156 | 0.026313 | -0.26374 | -0.0165 | 0.939811 | Mitral valve stenosis and aortic valve stenosis | circulatory syster | 1.579832 |
| 520.2 | 0.468683 | 0.211083 | 2.220378 | 0.026393 | 0.054969 | 0.882397 | 0.516754 | Disturbances in tooth eruption | digestive | 1.578509 |
| 145.4 | 0.421943 | 0.190252 | 2.217817 | 0.026567 | 0.049057 | 0.794829 | 0.730724 | Cancer of the gums | neoplasms | 1.575652 |
| 710.19 | 0.116567 | 0.052807 | 2.207424 | 0.027284 | 0.013068 | 0.220067 | 0.848338 | Unspecified osteomyelitis | musculoskeletal | 1.564085 |
| 567 | 0.08017 | 0.036349 | 2.205543 | 0.027416 | 0.008927 | 0.151413 | 0.374897 | Peritonitis and retroperitoneal infections | digestive | 1.561996 |
| 250.22 | 0.08955 | 0.040642 | 2.203391 | 0.027567 | 0.009893 | 0.169206 | 0.102725 | Type 2 diabetes with renal manifestations | endocrine/metab | 1.559608 |
| 585.33 | 0.046304 | 0.021049 | 2.199867 | 0.027816 | 0.00505 | 0.087559 | 0.914763 | Chronic Kidney Disease, Stage III | genitourinary | 1.5557 |
| 676 | -0.26537 | 0.120699 | -2.19863 | 0.027905 | -0.50194 | -0.02881 | 0.154788 | Other disorders of the breast associated with c | pregnancy compl | 1.554325 |
| 571 | 0.052065 | 0.023916 | 2.177025 | 0.029479 | 0.005191 | 0.098939 | 0.262384 | Chronic liver disease and cirrhosis | digestive | 1.530492 |
| 619.3 | -0.06857 | 0.031562 | -2.17272 | 0.029802 | -0.13043 | -0.00671 | 0.960739 | Noninflammatory disorders of cervix | genitourinary | 1.52576 |
| 650 | -0.1077 | 0.049581 | -2.17221 | 0.02984 | -0.20488 | -0.01052 | 0.473154 | Normal delivery | pregnancy compl | 1.525204 |
| 614.5 | -0.05367 | 0.024712 | -2.17174 | 0.029875 | -0.1021 | -0.00523 | 0.709158 | Inflammatory disease of cervix, vagina, and vu | genitourinary | 1.524688 |
| 290.13 | 0.413834 | 0.190572 | 2.171541 | 0.02989 | 0.040321 | 0.787347 | 0.147497 | Senile dementia | mental disorders | 1.52447 |
| 401.3 | 0.048911 | 0.022585 | 2.165695 | 0.030335 | 0.004646 | 0.093176 | 0.785281 | Other hypertensive complications | circulatory syster | 1.518063 |
| 731 | 0.08078 | 0.03732 | 2.164495 | 0.030426 | 0.007633 | 0.153927 | 0.968965 | Osteitis deformans and osteopathies associate | musculoskeletal | 1.516749 |
| 258 | 0.3009 | 0.139272 | 2.160515 | 0.030733 | 0.027931 | 0.573869 | 0.594706 | Iatrogenic endocrine disorders | endocrine/metab | 1.512398 |

| phecode | beta | se | zval | pval | ci.lb | ci.ub | QEp | phenotype | category | minuslogp |
|---|---|---|---|---|---|---|---|---|---|---|
| 742.8 | -0.11248 | 0.052251 | -2.1526 | 0.03135 | -0.21489 | -0.01007 | 0.282365 | Articular cartilage disorder | musculoskeletal | 1.503756 |
| 227.1 | 0.14276 | 0.066333 | 2.152186 | 0.031383 | 0.012751 | 0.27277 | 0.363756 | Benign neoplasm of adrenal gland | neoplasms | 1.50331 |
| 283.2 | 0.311647 | 0.144825 | 2.151879 | 0.031407 | 0.027794 | 0.595499 | 0.404696 | Non-autoimmune hemolytic anemias | hematopoietic | 1.502975 |
| 338 | 0.056918 | 0.026455 | 2.151519 | 0.031435 | 0.005068 | 0.108769 | 0.076536 | Pain | neurological | 1.502583 |
| 742.1 | 0.353674 | 0.164559 | 2.149232 | 0.031616 | 0.031146 | 0.676203 | 0.987207 | Loose body in joint | musculoskeletal | 1.500093 |
| 454.1 | 0.066147 | 0.030821 | 2.146164 | 0.03186 | 0.005739 | 0.126555 | 0.291309 | Varicose veins of lower extremity | circulatory syster | 1.496755 |
| 457.2 | 0.11559 | 0.053895 | 2.14475 | 0.031973 | 0.009959 | 0.221222 | 0.130433 | Encounter for long-term (current) use of antipl | circulatory syster | 1.495219 |
| 363.3 | 0.220992 | 0.103378 | 2.137701 | 0.032541 | 0.018374 | 0.423609 | 0.266289 | Chorioretinal scars | sense organs | 1.487569 |
| 352.2 | 0.113187 | 0.052969 | 2.136843 | 0.032611 | 0.009369 | 0.217004 | 0.859599 | Facial nerve disorders [CN7] | neurological | 1.486639 |
| 426.9 | 0.062654 | 0.029444 | 2.127901 | 0.033345 | 0.004945 | 0.120364 | 0.46278 | Cardiac pacemaker/device in situ | circulatory syster | 1.476966 |
| 286.13 | 0.384164 | 0.180815 | 2.124622 | 0.033618 | 0.029773 | 0.738556 | 0.562309 | Congenital factor VIII disorder | hematopoietic | 1.473426 |
| 446.6 | 0.395763 | 0.186705 | 2.119719 | 0.03403 | 0.029827 | 0.761698 | 0.906558 | Polyarteritis nodosa | circulatory syster | 1.468142 |
| 610.4 | -0.12503 | 0.059162 | -2.11339 | 0.034567 | -0.24099 | -0.00908 | 0.672241 | Benign neoplasm of breast | genitourinary | 1.461337 |
| 292 | 0.036913 | 0.017498 | 2.109515 | 0.0349 | 0.002617 | 0.071208 | 0.310105 | Neurological disorders | mental disorders | 1.457172 |
| 750.14 | 0.176916 | 0.083941 | 2.107629 | 0.035063 | 0.012395 | 0.341437 | 0.453635 | Congenital anomalies of esophagus | congenital anoma | 1.45515 |
| 189.11 | 0.103229 | 0.04899 | 2.107121 | 0.035107 | 0.007209 | 0.199249 | 0.568942 | Malignant neoplasm of kidney, except pelvis | neoplasms | 1.454605 |
| 305.2 | -0.14218 | 0.067701 | -2.10011 | 0.035719 | -0.27487 | -0.00949 | 0.842452 | Eating disorder | mental disorders | 1.447101 |
| 717 | -0.18994 | 0.090479 | -2.09924 | 0.035796 | -0.36727 | -0.0126 | 0.320609 | Polymyalgia Rheumatica | musculoskeletal | 1.446167 |
| 736.4 | 0.238383 | 0.11368 | 2.096967 | 0.035997 | 0.015574 | 0.461192 | 0.421725 | Genu valgum or varum (acquired) | musculoskeletal | 1.443739 |
| 681.2 | 0.114728 | 0.054739 | 2.095913 | 0.03609 | 0.007442 | 0.222014 | 0.966844 | Cellulitis and abscess of face/neck | dermatologic | 1.442614 |
| 495.1 | 0.107326 | 0.051231 | 2.094924 | 0.036178 | 0.006914 | 0.207738 | 0.349409 | Chronic obstructive asthma | respiratory | 1.441559 |
| 394.2 | -0.08288 | 0.039567 | -2.09459 | 0.036207 | -0.16043 | -0.00533 | 0.951326 | Mitral valve disease | circulatory syster | 1.441206 |
| 522.1 | -3.44781 | 1.647735 | -2.09245 | 0.036398 | -6.67731 | -0.21831 | 1 | Pulpitis and necrosis of tooth pulp | digestive | 1.438924 |
| 710.12 | 0.143727 | 0.068712 | 2.091733 | 0.036462 | 0.009054 | 0.278399 | 0.609556 | Chronic osteomyelitis | musculoskeletal | 1.438155 |
| 1006 | 0.394769 | 0.189104 | 2.087576 | 0.036836 | 0.024132 | 0.765407 | 0.414192 | Crushing injury | other | 1.433727 |
| 627.2 | -0.04027 | 0.019372 | -2.07849 | 0.037664 | -0.07823 | -0.0023 | 0.769976 | Symptomatic menopause | genitourinary | 1.424071 |
| 527.1 | 0.281093 | 0.135324 | 2.077183 | 0.037785 | 0.015863 | 0.546323 | 0.796598 | Hypertrophy of salivary gland | digestive | 1.422684 |
| 574 | 0.067006 | 0.032301 | 2.074438 | 0.038039 | 0.003698 | 0.130315 | 0.196665 | Cholelithiasis and cholecystitis | digestive | 1.419775 |
| 244.4 | -0.06217 | 0.029971 | -2.07422 | 0.038059 | -0.12091 | -0.00342 | 0.10154 | Hypothyroidism NOS | endocrine/metab | 1.419544 |
| 645 | -0.11724 | 0.056536 | -2.07378 | 0.0381 | -0.22805 | -0.00643 | 0.238128 | Late pregnancy and failed induction | pregnancy compl | 1.419076 |
| 965.3 | 0.376186 | 0.181923 | 2.067833 | 0.038656 | 0.019624 | 0.732747 | 0.77439 | Salicylates causing adverse effects in therape | injuries & poisoni | 1.412787 |
| 594 | 0.043721 | 0.021205 | 2.06179 | 0.039228 | 0.002159 | 0.085283 | 0.446434 | Urinary calculus | genitourinary | 1.406407 |
| 530.5 | 0.079926 | 0.038801 | 2.059871 | 0.039411 | 0.003877 | 0.155975 | 0.62035 | Disorders of esophageal motility | digestive | 1.404384 |
| 710.1 | 0.098508 | 0.047849 | 2.058743 | 0.039519 | 0.004726 | 0.19229 | 0.795755 | Osteomyelitis | musculoskeletal | 1.403195 |
| 704.11 | 0.196589 | 0.095531 | 2.057859 | 0.039604 | 0.009352 | 0.383826 | 0.844371 | Alopecia Areata | dermatologic | 1.402264 |
| 136 | 0.037811 | 0.01842 | 2.052689 | 0.040103 | 0.001708 | 0.073914 | 0.823631 | Other infectious and parasitic diseases | infectious diseas | 1.396825 |
| 481 | 0.065667 | 0.03207 | 2.047599 | 0.040599 | 0.00281 | 0.128523 | 0.188712 | Influenza | respiratory | 1.391482 |
| 571.8 | 0.094508 | 0.046197 | 2.045755 | 0.04078 | 0.003963 | 0.185052 | 0.275764 | Liver abscess and sequelae of chronic liver dis | digestive | 1.389548 |
| 41.1 | 0.070947 | 0.034716 | 2.04E+00 | 0.04099 | 0.002905 | 0.13899 | 0.403496 | Staphylococcus infections | infectious diseas | 1.387319 |
| 613.7 | 0.138076 | 0.067597 | 2.042634 | 0.041089 | 0.005588 | 0.270563 | 0.3118 | Other signs and symptoms in breast | genitourinary | 1.386277 |
| 389.2 | -0.11995 | 0.058834 | -2.03873 | 0.041477 | -0.23526 | -0.00463 | 0.526513 | Conductive hearing loss | sense organs | 1.382192 |
| 150 | 0.307323 | 0.151402 | 2.029846 | 0.042372 | 0.01058 | 0.604066 | 0.148936 | Cancer of esophagus | neoplasms | 1.372919 |
| 241.1 | -0.04618 | 0.022781 | -2.02731 | 0.04263 | -0.09083 | -0.00153 | 0.514693 | Nontoxic uninodular goiter | endocrine/metab | 1.370282 |
| 361 | -0.08523 | 0.042047 | -2.02711 | 0.042651 | -0.16764 | -0.00282 | 0.593418 | Retinal detachments and defects | sense organs | 1.370073 |
| 504.1 | 0.09681 | 0.048065 | 2.014173 | 0.043991 | 0.002606 | 0.191015 | 0.822139 | Idiopathic fibrosing alveolitis | respiratory | 1.356633 |
| 202.22 | -0.15048 | 0.074883 | -2.00951 | 0.044483 | -0.29725 | -0.00371 | 0.415875 | Reticulosarcoma | neoplasms | 1.35181 |
| 442.2 | 0.367495 | 0.182941 | 2.008822 | 0.044556 | 0.008938 | 0.726053 | 0.127256 | Aneurysm of iliac artery | circulatory syster | 1.351093 |
| 425 | 0.053085 | 0.026489 | 2.004006 | 0.045069 | 0.001167 | 0.105003 | 0.852568 | Cardiomyopathy | circulatory syster | 1.346118 |

| phecode | beta | se | zval | pval | ci.lb | ci.ub | QEp | phenotype | category | minuslogp |
|---|---|---|---|---|---|---|---|---|---|---|
| 427.6 | 0.049326 | 0.024642 | 2.001689 | 0.045318 | 0.001028 | 0.097623 | 0.974401 | Premature beats | circulatory syster | 1.343728 |
| 270.35 | 0.329042 | 0.164707 | 1.997735 | 0.045745 | 0.006221 | 0.651862 | 0.661305 | Macroglobulinemia | endocrine/metab | 1.339653 |
| 636.3 | -0.11391 | 0.05709 | -1.99535 | 0.046005 | -0.22581 | -0.00202 | 0.639909 | Hemorrhage in early pregnancy | pregnancy compl | 1.337194 |
| 425.2 | 0.090812 | 0.0456 | 1.99149 | 0.046427 | 0.001438 | 0.180186 | 0.698607 | Secondary/extrinsic cardiomyopathies | circulatory syster | 1.333229 |
| 550.1 | -0.0616 | 0.030977 | -1.98871 | 0.046734 | -0.12232 | -0.00089 | 0.714887 | Inguinal hernia | digestive | 1.33037 |
| 145.2 | 0.160613 | 0.08078 | 1.988294 | 0.046779 | 0.002289 | 0.318938 | 0.508867 | Cancer of tongue | neoplasms | 1.329948 |
| 31 | 0.147249 | 0.074204 | 1.98E+00 | 0.047213 | 0.001812 | 0.292686 | 0.154791 | Diseases due to other mycobacteria | infectious diseas | 1.325943 |
| 275.11 | 0.263409 | 0.133227 | 1.977139 | 0.048026 | 0.002288 | 0.52453 | 0.701035 | Hereditary hemochromatosis | hematopoietic | 1.318524 |
| 289.1 | 0.439369 | 0.222297 | 1.976494 | 0.048099 | 0.003675 | 0.875064 | 0.438189 | Myelofibrosis | hematopoietic | 1.317865 |
| 366 | -0.03535 | 0.017911 | -1.97359 | 0.048428 | -0.07045 | -0.00024 | 0.380685 | Cataract | sense organs | 1.314899 |
| 286.6 | 0.118891 | 0.060266 | 1.972761 | 0.048523 | 0.000771 | 0.23701 | 0.551903 | Defibrination syndrome | hematopoietic | 1.314054 |
| 986 | -1.4889 | 0.755643 | -1.97037 | 0.048795 | -2.96993 | -0.00787 | 1 | Toxic effect of carbon monoxide | injuries & poisoni | 1.311621 |
| 286.11 | 0.318582 | 0.162079 | 1.96559 | 0.049346 | 0.000912 | 0.636252 | 0.591806 | Von willebrand's disease | hematopoietic | 1.306748 |
| 443.7 | 0.096813 | 0.049434 | 1.958422 | 0.050181 | -7.62E-05 | 0.193702 | 0.450942 | Peripheral angiopathy in diseases classified el | circulatory syster | 1.299465 |
| 244 | -0.03238 | 1.66E-02 | -1.94839 | 0.051368 | -0.06496 | 0.000192 | 0.222905 | Hypothyroidism | endocrine/metab | 1.289306 |
| 41.4 | 0.070433 | 0.036178 | 1.95E+00 | 0.051549 | -0.00047 | 0.14134 | 0.841229 | E. coli | infectious diseas | 1.287778 |
| 636.8 | -0.27986 | 0.143848 | -1.94551 | 0.051714 | -0.56179 | 0.002079 | 0.5531 | Cervical incompetence | pregnancy compl | 1.286394 |
| 343 | 0.238239 | 0.122582 | 1.943503 | 0.051955 | -0.00202 | 0.478496 | 0.942023 | Infantile cerebral palsy | neurological | 1.284369 |
| 276 | 0.028753 | 0.014795 | 1.943464 | 0.05196 | -0.00024 | 0.057751 | 0.994743 | Disorders of fluid, electrolyte, and acid-base ba | endocrine/metab | 1.28433 |
| 442.11 | 0.105533 | 0.054334 | 1.942293 | 0.052102 | -0.00096 | 0.212027 | 0.576041 | Abdominal aortic aneurysm | circulatory syster | 1.283149 |
| 614 | -0.04684 | 0.024142 | -1.94034 | 0.052338 | -0.09416 | 0.000474 | 0.539966 | Inflammatory diseases of female pelvic organs | genitourinary | 1.281182 |
| 569 | 0.03365 | 0.017503 | 1.9226 | 0.05453 | -0.00065 | 0.067955 | 0.955205 | Other disorders of intestine | digestive | 1.263362 |
| 803.1 | 0.100657 | 0.052408 | 1.920643 | 0.054777 | -0.00206 | 0.203374 | 0.726216 | Fracture of humerus | injuries & poisoni | 1.261404 |
| 220 | -0.20692 | 0.107742 | -1.92051 | 0.054793 | -0.41809 | 0.004251 | 0.159999 | Benign neoplasm of ovary | neoplasms | 1.261271 |
| 272.9 | -0.13712 | 0.071402 | -1.92036 | 0.054812 | -0.27706 | 0.002828 | 0.824685 | Unspecified disorder of lipoid metabolism | endocrine/metab | 1.261125 |
| 962.2 | 0.416444 | 0.217071 | 1.918468 | 0.055052 | -0.00901 | 0.841896 | 0.18041 | Insulins and antidiabetic agents causing adver | injuries & poisoni | 1.259229 |
| 1012 | 0.228052 | 0.11892 | 1.917689 | 0.05515 | -0.00503 | 0.461132 | 0.170197 | Late effect | other | 1.258451 |
| 70.9 | 0.068727 | 0.035879 | 1.92E+00 | 0.055425 | -0.00159 | 0.139049 | 0.963737 | Hepatitis NOS | infectious diseas | 1.256296 |
| 496 | 0.092047 | 0.048063 | 1.915137 | 0.055475 | -0.00215 | 0.186248 | 0.015078 | Chronic airway obstruction | respiratory | 1.255903 |
| 440.22 | 0.181915 | 0.095247 | 1.909937 | 0.056141 | -0.00476 | 0.368595 | 0.193591 | Atherosclerosis of native arteries of the extrem | circulatory syster | 1.250717 |
| 695.7 | 0.082043 | 0.043152 | 1.901229 | 0.057272 | -0.00253 | 0.16662 | 0.93735 | Prurigo and Lichen | dermatologic | 1.242057 |
| 604.1 | 0.257694 | 0.135817 | 1.897355 | 0.057781 | -0.0085 | 0.523891 | 0.244105 | Redundant prepuce and phimosis/BXO | genitourinary | 1.238214 |
| 715.3 | 0.199971 | 0.105429 | 1.896749 | 0.057861 | -0.00666 | 0.406608 | 0.826277 | Spinal enthesopathy | musculoskeletal | 1.237613 |
| 385.3 | 0.25398 | 0.133989 | 1.895536 | 0.058021 | -0.00863 | 0.516593 | 0.267452 | Cholesteatoma | sense organs | 1.236412 |
| 8 | 0.042849 | 0.022743 | 1.88E+00 | 0.059564 | -0.00173 | 0.087425 | 0.573575 | Intestinal infection | infectious diseas | 1.225013 |
| 964 | 0.153493 | 0.08152 | 1.882892 | 0.059715 | -0.00628 | 0.313268 | 0.609305 | Poisoning by agents primarily affecting blood c | injuries & poisoni | 1.223917 |
| 375.1 | -0.04749 | 0.025224 | -1.88269 | 0.059743 | -0.09693 | 0.001949 | 0.30266 | Dry eyes | sense organs | 1.223716 |
| 250.1 | 0.080615 | 4.29E-02 | 1.878465 | 0.060318 | -0.0035 | 0.164728 | 0.265805 | Type 1 diabetes | endocrine/metab | 1.219556 |
| 371.3 | -0.04292 | 0.022975 | -1.86827 | 0.061724 | -0.08795 | 0.002107 | 0.826683 | Inflammation of eyelids | sense organs | 1.209545 |
| 260.3 | 0.08095 | 0.043375 | 1.866284 | 0.062002 | -0.00406 | 0.165964 | 0.681234 | Adult failure to thrive | endocrine/metab | 1.207597 |
| 242.1 | -0.12589 | 6.75E-02 | -1.86612 | 0.062025 | -0.25812 | 0.006331 | 0.494312 | Graves' disease | endocrine/metab | 1.207432 |
| 132.1 | 0.400047 | 0.214477 | 1.865226 | 0.06215 | -0.02032 | 0.820414 | 0.708908 | Pediculosis and phthirus infestation | infectious diseas | 1.206561 |
| 972.6 | 0.307464 | 0.164917 | 1.864358 | 0.062271 | -0.01577 | 0.630695 | 0.182947 | Antihypertensive agents causing adverse effec | injuries & poisoni | 1.205711 |
| 525.2 | 0.678582 | 0.364684 | 1.860743 | 0.06278 | -0.03618 | 1.393349 | 0.421017 | Atrophy of edentulous alveolar ridge | digestive | 1.202175 |
| 272.12 | 0.062182 | 0.033458 | 1.8585 | 0.063098 | -0.00339 | 0.127759 | 0.501486 | Hyperglyceridemia | endocrine/metab | 1.199984 |
| 433.21 | 0.050928 | 0.027422 | 1.857226 | 0.063279 | -0.00282 | 0.104674 | 0.385803 | Cerebral artery occlusion, with cerebral infarct | circulatory syster | 1.19874 |
| 216 | -0.04197 | 0.022651 | -1.85288 | 0.063899 | -0.08636 | 0.002425 | 0.35672 | Benign neoplasm of skin | neoplasms | 1.194506 |
| 242 | -0.06517 | 0.035185 | -1.85224 | 0.063992 | -0.13413 | 0.00379 | 0.614105 | Thyrotoxicosis with or without goiter | endocrine/metab | 1.193875 |

| phecode | beta | se | zval | pval | ci.lb | ci.ub | QEp | phenotype | category | minuslogp |
|---|---|---|---|---|---|---|---|---|---|---|
| 418.1 | 0.062574 | 0.033818 | 1.850295 | 0.064271 | -0.00371 | 0.128857 | 0.593055 | Precordial pain | circulatory syster | 1.191985 |
| 300.3 | -0.13473 | 0.072835 | -1.84982 | 0.064339 | -0.27749 | 0.008022 | 0.962457 | Obsessive-compulsive disorders | mental disorders | 1.191525 |
| 580.32 | 0.066917 | 0.03628 | 1.844484 | 0.065113 | -0.00419 | 0.138024 | 0.7284 | Nephritis and nephropathy with pathological le | genitourinary | 1.186335 |
| 520 | 0.252847 | 0.137185 | 1.843114 | 0.065312 | -0.01603 | 0.521724 | 0.887986 | Disorders of tooth development | digestive | 1.185004 |
| 81.12 | 0.27289 | 0.148269 | 1.840509 | 0.065694 | -0.01771 | 0.563492 | 0.273335 | Chronic graft-versus-host disease | infectious diseas | 1.182477 |
| 386.21 | 0.655126 | 0.356602 | 1.837137 | 0.06619 | -0.0438 | 1.354052 | 0.189301 | Central origin vertigo | sense organs | 1.17921 |
| 312 | 0.126574 | 0.068922 | 1.836483 | 0.066286 | -0.00851 | 0.261659 | 0.324518 | Conduct disorders | mental disorders | 1.178576 |
| 427.4 | 0.084679 | 0.046162 | 1.834409 | 0.066593 | -0.0058 | 0.175154 | 0.836938 | Cardiac arrest and ventricular fibrillation | circulatory syster | 1.17657 |
| 618.6 | 0.294522 | 0.160602 | 1.83386 | 0.066675 | -0.02025 | 0.609297 | 0.947718 | Vaginal enterocele, congenital or acquired | genitourinary | 1.176038 |
| 395.3 | 0.062932 | 0.034366 | 1.831217 | 0.067068 | -0.00442 | 0.130288 | 0.820674 | Nonrheumatic tricuspid valve disorders | circulatory syster | 1.173484 |
| 229.1 | 0.627404 | 0.343218 | 1.828007 | 0.067549 | -0.04529 | 1.300098 | 0.139576 | Benign neoplasm of lymph nodes | neoplasms | 1.170384 |
| 611 | -0.05022 | 0.027512 | -1.82525 | 0.067963 | -0.10414 | 0.003706 | 0.195977 | Abnormal findings on mammogram or breast e | genitourinary | 1.167724 |
| 512.9 | 0.027159 | 0.014883 | 1.824816 | 0.068029 | -0.00201 | 0.05633 | 0.531323 | Other dyspnea | respiratory | 1.167307 |
| 270.11 | -0.15477 | 0.084835 | -1.82435 | 0.068099 | -0.32104 | 0.011504 | 0.255212 | Disturbances of sulphur-bearing amino-acid m | endocrine/metab | 1.166862 |
| 362.2 | -0.04913 | 0.027008 | -1.81927 | 0.068871 | -0.10207 | 0.0038 | 0.596642 | Degeneration of macula and posterior pole of | sense organs | 1.161965 |
| 573.2 | 0.081184 | 0.044919 | 1.807342 | 0.070709 | -0.00686 | 0.169224 | 0.889856 | Liver replaced by transplant | digestive | 1.150526 |
| 214 | 0.052258 | 0.029041 | 1.799449 | 0.071948 | -0.00466 | 0.109178 | 0.584386 | Lipoma | neoplasms | 1.142983 |
| 872 | 0.248023 | 0.137886 | 1.798761 | 0.072056 | -0.02223 | 0.518274 | 0.25055 | Traumatic amputation | injuries & poisoni | 1.142327 |
| 743.1 | -0.04838 | 0.026898 | -1.79859 | 0.072083 | -0.1011 | 0.004341 | 0.333985 | Osteoporosis | musculoskeletal | 1.142166 |
| 532 | 0.033008 | 0.018378 | 1.796092 | 0.07248 | -0.00301 | 0.069027 | 0.713654 | Dysphagia | digestive | 1.139783 |
| 374.2 | 0.166778 | 0.093009 | 1.793135 | 0.072951 | -0.01552 | 0.349072 | 0.630173 | Lagophthalmos | sense organs | 1.136967 |
| 720.1 | 0.044358 | 0.024743 | 1.792783 | 0.073008 | -0.00414 | 0.092853 | 0.522952 | Spinal stenosis of lumbar region | musculoskeletal | 1.136632 |
| 286.2 | 0.050916 | 0.028451 | 1.789567 | 0.073524 | -0.00485 | 0.106679 | 0.191039 | Encounter for long-term (current) use of antico | hematopoietic | 1.133573 |
| 425.12 | -0.14144 | 0.079162 | -1.78678 | 0.073973 | -0.2966 | 0.013709 | 0.653895 | Other hypertrophic cardiomyopathy | circulatory syster | 1.130929 |
| 458 | 0.034593 | 0.019388 | 1.784252 | 0.074383 | -0.00341 | 0.072592 | 0.476964 | Hypotension | circulatory syster | 1.128528 |
| 305.21 | -0.258 | 0.145014 | -1.77917 | 0.075211 | -0.54223 | 0.026217 | 0.83725 | Anorexia nervosa | mental disorders | 1.123717 |
| 691.1 | 0.37548 | 0.211188 | 1.777943 | 0.075413 | -0.03844 | 0.789401 | 0.348706 | Ichthyosis congenita | dermatologic | 1.122552 |
| 736.5 | 0.413118 | 0.232595 | 1.776123 | 0.075713 | -0.04276 | 0.868996 | 0.776742 | Acquired deformities of knee | musculoskeletal | 1.120832 |
| 217 | -0.06502 | 0.036772 | -1.76824 | 0.077021 | -0.13709 | 0.00705 | 0.34711 | Vascular hamartomas and non-neoplastic nevi | neoplasms | 1.113391 |
| 480.5 | 0.094806 | 0.053679 | 1.766182 | 0.077365 | -0.0104 | 0.200014 | 0.845191 | Bronchopneumonia and lung abscess | respiratory | 1.111454 |
| 313.2 | -0.24478 | 0.138691 | -1.76493 | 0.077575 | -0.51661 | 0.027049 | 0.776111 | Tics and stuttering | mental disorders | 1.110277 |
| 333 | -0.05583 | 0.031635 | -1.76469 | 0.077617 | -0.11783 | 0.006178 | 0.924685 | Extrapyramidal disease and abnormal movem | neurological | 1.110045 |
| 8.51 | 0.609241 | 0.345554 | 1.76E+00 | 0.077886 | -0.06803 | 1.286515 | 0.537181 | Intestinal e.coli | infectious diseas | 1.108538 |
| 535 | 0.034295 | 0.019468 | 1.761594 | 0.078138 | -0.00386 | 0.072452 | 0.435414 | Gastritis and duodenitis | digestive | 1.107138 |
| 264 | 0.123824 | 7.06E-02 | 1.754935 | 0.079271 | -0.01447 | 0.262115 | 0.607642 | Lack of normal physiological development | endocrine/metab | 1.100888 |
| 522.5 | 0.106802 | 0.060973 | 1.751619 | 0.079839 | -0.0127 | 0.226307 | 0.854651 | Periapical abscess | digestive | 1.097783 |
| 512.7 | 0.041716 | 0.023875 | 1.747285 | 0.080588 | -0.00508 | 0.088511 | 0.122864 | Shortness of breath | respiratory | 1.09373 |
| 522 | 0.105308 | 0.060411 | 1.74318 | 0.081302 | -0.0131 | 0.223712 | 0.85939 | Diseases of pulp and periapical tissues | digestive | 1.089898 |
| 651 | -0.1062 | 0.061006 | -1.74075 | 0.081727 | -0.22577 | 0.013373 | 0.42519 | Multiple gestation | pregnancy compl | 1.087634 |
| 440.2 | 0.102913 | 0.059125 | 1.740587 | 0.081756 | -0.01297 | 0.218796 | 0.18929 | Atherosclerosis of the extremities | circulatory syster | 1.08748 |
| 378.2 | 0.153279 | 0.088133 | 1.739184 | 0.082002 | -0.01946 | 0.326017 | 0.24745 | Nystagmus and other irregular eye movement | sense organs | 1.086173 |
| 371.21 | -0.06571 | 0.037787 | -1.73893 | 0.082047 | -0.13977 | 0.008352 | 0.870223 | Allergic conjunctivitis | sense organs | 1.085939 |
| 292.4 | 0.042755 | 0.024593 | 1.738507 | 0.082122 | -0.00545 | 0.090956 | 0.254186 | Altered mental status | mental disorders | 1.085543 |
| 722.3 | 0.407032 | 0.234173 | 1.738168 | 0.082181 | -0.05194 | 0.866002 | 0.394414 | Schmorl's nodes | musculoskeletal | 1.085228 |
| 290.12 | 0.226238 | 0.130562 | 1.732807 | 0.08313 | -0.02966 | 0.482134 | 0.764109 | Dementia with cerebral degenerations | mental disorders | 1.080243 |
| 568 | 0.044807 | 0.025925 | 1.728292 | 0.083936 | -0.00601 | 0.09562 | 0.098136 | Other disorders of peritoneum | digestive | 1.076052 |
| 470 | -0.04648 | 0.026951 | -1.72447 | 0.084623 | -0.0993 | 0.006347 | 0.668219 | Septal Deviations/Turbinate Hypertrophy | respiratory | 1.07251 |
| 614.51 | -0.07134 | 0.041404 | -1.72307 | 0.084876 | -0.15249 | 0.009808 | 0.570696 | Cervicitis and endocervicitis | genitourinary | 1.071214 |

| phecode | beta | se | zval | pval | ci.lb | ci.ub | QEp | phenotype | category | minuslogp |
|---|---|---|---|---|---|---|---|---|---|---|
| 285.21 | 0.04811 | 0.027939 | 1.721933 | 0.085082 | -0.00665 | 0.102869 | 0.638443 | Anemia in chronic kidney disease | hematopoietic | 1.070164 |
| 401.1 | 0.055499 | 0.032241 | 1.721358 | 0.085186 | -0.00769 | 0.118691 | 0.069911 | Essential hypertension | circulatory syster | 1.069633 |
| 722.6 | 0.028036 | 0.016314 | 1.718501 | 0.085705 | -0.00394 | 0.060012 | 0.506978 | Degeneration of intervertebral disc | musculoskeletal | 1.066992 |
| 614.52 | -0.04895 | 0.028562 | -1.71392 | 0.086544 | -0.10493 | 0.007027 | 0.5626 | Vaginitis and vulvovaginitis | genitourinary | 1.062764 |
| 333.8 | 0.177239 | 0.103578 | 1.711173 | 0.087049 | -0.02577 | 0.380247 | 0.616755 | Other degenerative diseases of the basal gang | neurological | 1.060235 |
| 574.2 | 0.084367 | 0.049372 | 1.708803 | 0.087487 | -0.0124 | 0.181135 | 0.257383 | Calculus of bile duct | digestive | 1.058054 |
| 705.1 | 0.134256 | 0.078765 | 1.704513 | 0.088285 | -0.02012 | 0.288632 | 0.489057 | Dyshidrosis | dermatologic | 1.054112 |
| 270.34 | 0.259728 | 0.152617 | 1.701834 | 0.088786 | -0.03939 | 0.558852 | 0.287698 | Alpha-1-antitrypsin deficiency | endocrine/metab | 1.051653 |
| 264.2 | 0.167853 | 0.098656 | 1.7014 | 0.088868 | -0.02551 | 0.361214 | 0.721681 | Failure to thrive (childhood) | endocrine/metab | 1.051255 |
| 635.2 | -0.09198 | 0.054067 | -1.70114 | 0.088916 | -0.19794 | 0.013994 | 0.105992 | Antepartum hemorrhage, abruptio placentae, a | pregnancy compl | 1.051018 |
| 740.12 | 0.068786 | 0.040472 | 1.699569 | 0.089212 | -0.01054 | 0.14811 | 0.571769 | Osteoarthrosis, localized, secondary | musculoskeletal | 1.049576 |
| 599.4 | 0.038431 | 0.02264 | 1.697463 | 0.089609 | -0.00594 | 0.082804 | 0.938667 | Urinary incontinence | genitourinary | 1.047648 |
| 368.91 | 0.148298 | 0.087531 | 1.694222 | 0.090223 | -0.02326 | 0.319856 | 0.669218 | Psychophysical visual disturbances | sense organs | 1.044682 |
| 270.1 | -0.12834 | 0.075767 | -1.69384 | 0.090295 | -0.27684 | 0.020163 | 0.826149 | Disturbances of amino-acid transport | endocrine/metab | 1.044337 |
| 433.3 | 0.045635 | 0.027 | 1.690213 | 0.090987 | -0.00728 | 0.098553 | 0.36225 | Cerebral ischemia | circulatory syster | 1.04102 |
| 519 | 0.032477 | 0.019306 | 1.682286 | 0.092513 | -0.00536 | 0.070316 | 0.607931 | Other diseases of respiratory system, not else | respiratory | 1.033795 |
| 595 | 0.098855 | 0.058783 | 1.681682 | 0.092631 | -0.01636 | 0.214068 | 0.086479 | Hydronephrosis | genitourinary | 1.033245 |
| 426.3 | 0.049673 | 0.02955 | 1.680981 | 0.092767 | -0.00824 | 0.107591 | 0.27866 | Bundle branch block | circulatory syster | 1.032608 |
| 358 | -0.0991 | 0.059112 | -1.67647 | 0.093645 | -0.21496 | 0.016758 | 0.764542 | Myoneural disorders | neurological | 1.028514 |
| 264.1 | 0.350712 | 2.09E-01 | 1.676275 | 0.093684 | -0.05935 | 0.760778 | 0.324826 | Short stature | endocrine/metab | 1.028333 |
| 429.1 | 0.061043 | 0.036458 | 1.674322 | 0.094067 | -0.01041 | 0.1325 | 0.562482 | Heart transplant/surgery | circulatory syster | 1.026562 |
| 380 | -0.11554 | 0.069084 | -1.67247 | 0.094432 | -0.25094 | 0.019861 | 0.54415 | Disorders of external ear | sense organs | 1.024883 |
| 724.9 | 0.069529 | 0.041618 | 1.670651 | 0.094791 | -0.01204 | 0.151098 | 0.91563 | Other unspecified back disorders | musculoskeletal | 1.023235 |
| 475.9 | -0.03847 | 0.023037 | -1.66986 | 0.094948 | -0.08362 | 0.006683 | 0.703103 | Postnasal drip | respiratory | 1.022516 |
| 732.1 | 0.23604 | 0.141715 | 1.665602 | 0.095793 | -0.04172 | 0.513796 | 0.994165 | Juvenile osteochondrosis | musculoskeletal | 1.018667 |
| 427.42 | 0.103395 | 0.062114 | 1.664608 | 0.095991 | -0.01835 | 0.225136 | 0.521773 | Cardiac arrest | circulatory syster | 1.01777 |
| 440.9 | 0.061228 | 0.036796 | 1.663993 | 0.096114 | -0.01089 | 0.133346 | 0.174338 | Atherosclerosis of aorta | circulatory syster | 1.017214 |
| 665 | -0.08821 | 0.053118 | -1.66057 | 0.0968 | -0.19231 | 0.015903 | 0.287108 | Obstetrical/birth trauma | pregnancy compl | 1.014126 |
| 300.12 | 0.056526 | 0.034108 | 1.657267 | 0.097466 | -0.01032 | 0.123377 | 0.511441 | Agorophobia, social phobia, and panic disorde | mental disorders | 1.011149 |
| 756.1 | 0.223844 | 0.135281 | 1.654655 | 0.097995 | -0.0413 | 0.488991 | 0.08536 | Congenital anomalies of abdominal wall; diaph | congenital anoma | 1.008798 |
| 585.32 | 0.049891 | 0.030153 | 1.654593 | 0.098007 | -0.00921 | 0.108989 | 0.759342 | End stage renal disease | genitourinary | 1.008743 |
| 362.27 | -0.08442 | 0.051051 | -1.65361 | 0.098206 | -0.18448 | 0.015639 | 0.656468 | Drusen (degenerative) of retina | sense organs | 1.00786 |
| 841 | -0.03728 | 0.022562 | -1.65238 | 0.098458 | -0.0815 | 0.00694 | 0.422756 | Sprains and strains of back and neck | injuries & poisoni | 1.006751 |
| 722.1 | 0.044825 | 0.027142 | 1.65149 | 0.098639 | -0.00837 | 0.098023 | 0.396159 | Displacement of intervertebral disc | musculoskeletal | 1.005953 |
| 714 | 0.046905 | 0.028474 | 1.647276 | 0.099501 | -0.0089 | 0.102713 | 0.941886 | Rheumatoid arthritis and other inflammatory p | musculoskeletal | 1.002171 |
| 290.2 | 0.076965 | 0.04678 | 1.645244 | 0.099919 | -0.01472 | 0.168652 | 0.722261 | Delirium due to conditions classified elsewhere | mental disorders | 1.00035 |
| 496.1 | 0.131758 | 0.080086 | 1.645208 | 0.099927 | -0.02521 | 0.288725 | 0.00585 | Emphysema | respiratory | 1.000317 |
| 523.31 | 0.103127 | 0.062821 | 1.641605 | 0.100672 | -0.02 | 0.226253 | 0.774959 | Acute periodontitis | digestive | 0.997092 |
| 574.1 | 0.045103 | 0.027514 | 1.639294 | 0.101152 | -0.00882 | 0.09903 | 0.28342 | Cholelithiasis | digestive | 0.995025 |
| 401 | 0.060714 | 0.037068 | 1.637906 | 0.101441 | -0.01194 | 0.133367 | 0.03218 | Hypertension | circulatory syster | 0.993785 |
| 287.31 | -0.11996 | 0.073303 | -1.63653 | 0.101728 | -0.26363 | 0.023709 | 0.969138 | Primary thrombocytopenia | hematopoietic | 0.992559 |
| 519.8 | 0.043181 | 0.026467 | 1.631496 | 0.102786 | -0.00869 | 0.095055 | 0.422436 | Other diseases of respiratory system, NEC | respiratory | 0.988067 |
| 384.1 | 0.279153 | 0.1715 | 1.62771 | 0.103586 | -0.05698 | 0.615287 | 0.8982 | Myringitis | sense organs | 0.984697 |
| 454 | 0.040915 | 0.02514 | 1.627493 | 0.103632 | -0.00836 | 0.090189 | 0.951843 | Varicose veins | circulatory syster | 0.984504 |
| 324 | -0.12465 | 0.076632 | -1.62667 | 0.103808 | -0.27485 | 0.025541 | 0.230165 | Other CNS infection and poliomyelitis | neurological | 0.98377 |
| 286.4 | 0.074921 | 0.046077 | 1.625985 | 0.103953 | -0.01539 | 0.165231 | 0.851405 | Acquired coagulation factor deficiency | hematopoietic | 0.983164 |
| 433.2 | 0.043942 | 0.027046 | 1.624715 | 0.104223 | -0.00907 | 0.096951 | 0.26274 | Occlusion of cerebral arteries | circulatory syster | 0.982035 |
| 358.1 | -0.19116 | 0.117669 | -1.62457 | 0.104254 | -0.42179 | 0.039465 | 0.641852 | Myasthenia gravis | neurological | 0.981909 |

| phecode | beta | se | zval | pval | ci.lb | ci.ub | QEp | phenotype | category | minuslogp |
|---|---|---|---|---|---|---|---|---|---|---|
| 794 | -0.12679 | 0.078276 | -1.61976 | 0.105284 | -0.28021 | 0.02663 | 0.979476 | Abnormal results of other function studies (bla | symptoms | 0.977638 |
| 614.3 | -0.12973 | 0.080196 | -1.61761 | 0.105746 | -0.28691 | 0.027455 | 0.667186 | Pelvic inflammatory disease (PID) | genitourinary | 0.975734 |
| 528.3 | 0.345563 | 0.213701 | 1.617036 | 0.105871 | -0.07328 | 0.764409 | 0.07097 | Cellulitis and abscess of oral soft tissues | digestive | 0.975225 |
| 525.1 | 0.23266 | 0.144064 | 1.614976 | 0.106316 | -0.0497 | 0.51502 | 0.312975 | Loss of teeth or edentulism | digestive | 0.973402 |
| 455 | -0.02458 | 0.015235 | -1.61329 | 0.106681 | -0.05444 | 0.005282 | 0.628999 | Hemorrhoids | circulatory syster | 0.971911 |
| 371.9 | 0.412341 | 0.255604 | 1.613199 | 0.106701 | -0.08863 | 0.913316 | 0.434168 | Chronic inflammatory disorders of orbit | sense organs | 0.97183 |
| 395.4 | 0.103438 | 0.064148 | 1.612485 | 0.106856 | -0.02229 | 0.229167 | 0.366795 | Nonrheumatic pulmonary valve disorders | circulatory syster | 0.971199 |
| 501 | 0.056402 | 0.034983 | 1.612284 | 0.1069 | -0.01216 | 0.124967 | 0.81845 | Pneumonitis due to inhalation of food or vomit | respiratory | 0.971022 |
| 656.2 | 0.418308 | 0.259906 | 1.609461 | 0.107516 | -0.0911 | 0.927713 | 0.605832 | Respiratory conditions of fetus and newborn | pregnancy compl | 0.968528 |
| 458.9 | 0.035331 | 0.02199 | 1.606659 | 0.108129 | -0.00777 | 0.078431 | 0.452747 | Hypotension NOS | circulatory syster | 0.966057 |
| 441.2 | 0.175099 | 0.109083 | 1.605196 | 0.108451 | -0.0387 | 0.388897 | 0.547055 | Chronic vascular insufficiency of intestine | circulatory syster | 0.964767 |
| 512.1 | 0.047625 | 0.029705 | 1.603264 | 0.108876 | -0.0106 | 0.105846 | 0.14024 | Wheezing | respiratory | 0.963067 |
| 669 | -0.08386 | 0.052362 | -1.60162 | 0.10924 | -0.18649 | 0.018764 | 0.555434 | Complications of labor and delivery NEC | pregnancy compl | 0.96162 |
| 711.2 | -0.27394 | 0.171197 | -1.60012 | 0.109572 | -0.60948 | 0.061604 | 0.529104 | Reiter's disease | musculoskeletal | 0.960301 |
| 411.41 | -0.14903 | 0.093167 | -1.59959 | 0.109689 | -0.33163 | 0.033575 | 0.694925 | Aneurysm and dissection of heart | circulatory syster | 0.959835 |
| 251.1 | 0.065678 | 4.11E-02 | 1.597335 | 0.110191 | -0.01491 | 0.146267 | 0.859846 | Hypoglycemia | endocrine/metab | 0.957854 |
| 465.4 | 0.11707 | 0.073343 | 1.596191 | 0.110446 | -0.02668 | 0.26082 | 0.323248 | Acute laryngitis and tracheitis | respiratory | 0.95685 |
| 283.21 | 0.444754 | 0.279061 | 1.593752 | 0.110992 | -0.1022 | 0.991703 | 0.948314 | Hemolytic-uremic syndrome | hematopoietic | 0.95471 |
| 53 | 0.047581 | 0.029899 | 1.59E+00 | 0.111523 | -0.01102 | 0.106183 | 0.410689 | Herpes zoster | infectious diseas | 0.952635 |
| 250.21 | 0.208268 | 0.130996 | 1.589884 | 0.111861 | -0.04848 | 0.465015 | 0.180887 | Type 2 diabetes with ketoacidosis | endocrine/metab | 0.951321 |
| 292.6 | 0.223694 | 0.140765 | 1.589136 | 0.11203 | -0.0522 | 0.499588 | 0.076917 | Hallucinations | mental disorders | 0.950667 |
| 602.3 | 0.220894 | 0.139017 | 1.588976 | 0.112066 | -0.05157 | 0.493361 | 0.691672 | Dysplasia of prostate | genitourinary | 0.950526 |
| 858 | 0.06983 | 0.043956 | 1.588625 | 0.112145 | -0.01632 | 0.155982 | 0.517273 | Complication of internal orthopedic device | injuries & poisoni | 0.95022 |
| 958.1 | 0.323928 | 0.20398 | 1.588036 | 0.112278 | -0.07587 | 0.723721 | 0.286068 | Postoperative shock | injuries & poisoni | 0.949704 |
| 537 | 0.055508 | 0.034957 | 1.587892 | 0.112311 | -0.01301 | 0.124023 | 0.087246 | Other disorders of stomach and duodenum | digestive | 0.949579 |
| 751.21 | 0.062492 | 0.039415 | 1.58547 | 0.11286 | -0.01476 | 0.139744 | 0.937365 | Cystic kidney disease | congenital anoma | 0.947461 |
| 367.2 | -0.04404 | 0.027791 | -1.5848 | 0.113012 | -0.09851 | 0.010426 | 0.733137 | Astigmatism | sense organs | 0.946877 |
| 278.4 | 0.040698 | 0.025693 | 1.583962 | 0.113202 | -0.00966 | 0.091056 | 0.513924 | Abnormal weight gain | endocrine/metab | 0.946144 |
| 965.1 | 0.064137 | 0.040616 | 1.579122 | 0.114308 | -0.01547 | 0.143742 | 0.088127 | Opiates and related narcotics causing adverse | injuries & poisoni | 0.941923 |
| 705.8 | 0.063765 | 0.04039 | 1.578728 | 0.114398 | -0.0154 | 0.142929 | 0.541247 | Hyperhidrosis | dermatologic | 0.94158 |
| 705.3 | 0.155614 | 0.098594 | 1.578341 | 0.114487 | -0.03763 | 0.348855 | 0.736258 | Hidradenitis | dermatologic | 0.941243 |
| 647.3 | -0.25441 | 0.16129 | -1.57734 | 0.114718 | -0.57053 | 0.061714 | 0.78122 | Major puerperal infection | pregnancy compl | 0.94037 |
| 253.7 | 0.077937 | 4.95E-02 | 1.575796 | 0.115073 | -0.019 | 0.174875 | 0.699384 | Other disorders of neurohypophysis | endocrine/metab | 0.939027 |
| 716.1 | 0.1111 | 0.070561 | 1.574518 | 0.115368 | -0.0272 | 0.249398 | 0.474176 | Unspecified polyarthropathy or polyarthritis | musculoskeletal | 0.937916 |
| 523 | 0.067599 | 0.042971 | 1.573141 | 0.115686 | -0.01662 | 0.151821 | 0.666543 | Gingival and periodontal diseases | digestive | 0.936719 |
| 300.11 | -0.03155 | 0.020124 | -1.56754 | 0.116989 | -0.07099 | 0.007897 | 0.746374 | Generalized anxiety disorder | mental disorders | 0.931854 |
| 578.1 | 0.080793 | 0.051557 | 1.567056 | 0.117102 | -0.02026 | 0.181844 | 0.744806 | Hematemesis | digestive | 0.931437 |
| 476 | -0.02438 | 0.015571 | -1.56549 | 0.117467 | -0.05489 | 0.006142 | 0.333328 | Allergic rhinitis | respiratory | 0.930082 |
| 291 | 0.03995 | 0.025532 | 1.564696 | 0.117654 | -0.01009 | 0.089993 | 0.413581 | Other specified nonpsychotic and/or transient | mental disorders | 0.929393 |
| 695.4 | 0.070228 | 0.044908 | 1.563841 | 0.117855 | -0.01779 | 0.158246 | 0.802977 | Lupus (localized and systemic) | dermatologic | 0.928652 |
| 803.21 | 0.151961 | 0.097284 | 1.562038 | 0.118279 | -0.03871 | 0.342633 | 0.7643 | Colles' fracture | injuries & poisoni | 0.927092 |
| 572 | 0.039715 | 0.025429 | 1.561823 | 0.11833 | -0.01012 | 0.089554 | 0.702263 | Ascites (non malignant) | digestive | 0.926906 |
| 260 | 0.051216 | 0.032838 | 1.559645 | 0.118844 | -0.01315 | 0.115577 | 0.196236 | Protein-calorie malnutrition | endocrine/metab | 0.925023 |
| 155 | 0.060212 | 0.038627 | 1.558824 | 0.119038 | -0.01549 | 0.135919 | 0.937452 | Cancer of liver and intrahepatic bile duct | neoplasms | 0.924314 |
| 1009 | 0.039016 | 0.025042 | 1.558013 | 0.11923 | -0.01007 | 0.088097 | 0.715861 | Injury, NOS | other | 0.923614 |
| 250.7 | 0.062911 | 4.04E-02 | 1.55683 | 0.119511 | -0.01629 | 0.142112 | 0.607152 | Diabetic retinopathy | endocrine/metab | 0.922593 |
| 279.2 | -0.13996 | 0.089915 | -1.55656 | 0.119574 | -0.31619 | 0.036272 | 0.432528 | Autoimmune disease NEC | endocrine/metab | 0.922363 |
| 626.21 | -0.36991 | 0.237656 | -1.55648 | 0.119595 | -0.8357 | 0.095891 | 0.896885 | Mittelschmerz | genitourinary | 0.922287 |

| phecode | beta | se | zval | pval | ci.lb | ci.ub | QEp | phenotype | category | minuslogp |
|---|---|---|---|---|---|---|---|---|---|---|
| 654.1 | 0.15669 | 0.100751 | 1.555217 | 0.119894 | -0.04078 | 0.354159 | 0.101808 | Abnormality of organs and soft tissues of pelvi | pregnancy compl | 0.921201 |
| 367.4 | -0.04529 | 0.029141 | -1.55414 | 0.120151 | -0.1024 | 0.011826 | 0.937817 | Presbyopia | sense organs | 0.920271 |
| 609.2 | 0.172076 | 0.110743 | 1.553835 | 0.120224 | -0.04498 | 0.389127 | 0.609805 | Abnormal spermatozoa | genitourinary | 0.920009 |
| 647.1 | 0.1252 | 0.080613 | 1.553094 | 0.120401 | -0.0328 | 0.283198 | 0.637323 | Infections of genitourinary tract during pregnar | pregnancy compl | 0.919371 |
| 695.1 | 0.19589 | 0.126246 | 1.551652 | 0.120746 | -0.05155 | 0.443328 | 0.947168 | Toxic erythema | dermatologic | 0.918129 |
| 317.1 | 0.095224 | 0.061532 | 1.547544 | 0.121732 | -0.02538 | 0.215824 | 0.029136 | Alcoholism | mental disorders | 0.914595 |
| 697 | 0.232443 | 1.50E-01 | 1.546696 | 0.121937 | -0.06211 | 0.526993 | 0.063156 | Sarcoidosis | dermatologic | 0.913866 |
| 270.12 | -0.63446 | 0.411077 | -1.54342 | 0.122729 | -1.44016 | 0.171231 | 0.47249 | Phenylketonuria [PKU] | endocrine/metab | 0.911054 |
| 571.51 | 0.073688 | 0.047874 | 1.539194 | 0.123757 | -0.02014 | 0.16752 | 0.182521 | Cirrhosis of liver without mention of alcohol | digestive | 0.907431 |
| 710.3 | 0.745013 | 0.484185 | 1.538695 | 0.123879 | -0.20397 | 1.693999 | 0.453916 | Osteopathy resulting from poliomyelitis | musculoskeletal | 0.907003 |
| 337 | 0.075744 | 0.049241 | 1.538215 | 0.123996 | -0.02077 | 0.172255 | 0.794731 | Disorders of the autonomic nervous system | neurological | 0.906592 |
| 452.2 | 0.039013 | 0.025367 | 1.537967 | 0.124057 | -0.0107 | 0.088731 | 0.309492 | Deep vein thrombosis [DVT] | circulatory syster | 0.90638 |
| 599.8 | -0.04229 | 0.027507 | -1.53741 | 0.124194 | -0.0962 | 0.011624 | 0.692334 | Other symptoms involving urinary system | genitourinary | 0.905899 |
| 1010 | -0.0479 | 0.031189 | -1.5357 | 0.124613 | -0.10903 | 0.013233 | 0.177117 | Other tests | other | 0.904436 |
| 599.7 | 0.219352 | 0.142948 | 1.534491 | 0.124909 | -0.06082 | 0.499525 | 0.805191 | Urethral discharge | genitourinary | 0.903406 |
| 854 | 0.056119 | 0.036606 | 1.533053 | 0.125263 | -0.01563 | 0.127866 | 0.452883 | Complications of cardiac/vascular device, impl | injuries & poisoni | 0.902178 |
| 360 | -0.10716 | 0.069932 | -1.53233 | 0.125442 | -0.24422 | 0.029906 | 0.92119 | Disorders of the globe | sense organs | 0.901557 |
| 504 | 0.056986 | 0.03719 | 1.532304 | 0.125447 | -0.0159 | 0.129876 | 0.969834 | Other alveolar and parietoalveolar pneumonop | respiratory | 0.901538 |
| 575.7 | 0.056565 | 0.037035 | 1.527338 | 0.126677 | -0.01602 | 0.129153 | 0.679974 | Other disorders of gallbladder | digestive | 0.897303 |
| 90 | 0.067679 | 0.044316 | 1.527179 | 0.126716 | -0.01918 | 0.154537 | 0.861288 | Sexually transmitted infections (not HIV or hep | infectious diseas | 0.897167 |
| 241 | -0.0421 | 0.027639 | -1.52308 | 0.127739 | -0.09627 | 0.012075 | 0.181849 | Nontoxic nodular goiter | endocrine/metab | 0.893675 |
| 742.9 | 0.059465 | 0.039075 | 1.521825 | 0.128053 | -0.01712 | 0.13605 | 0.76043 | Other derangement of joint | musculoskeletal | 0.892611 |
| 585.31 | 0.0431 | 0.028347 | 1.520431 | 0.128403 | -0.01246 | 0.09866 | 0.962509 | Renal dialysis | genitourinary | 0.891426 |
| 350.5 | -0.15016 | 0.098779 | -1.52019 | 0.128463 | -0.34377 | 0.04344 | 0.481223 | Abnormal reflex | neurological | 0.891222 |
| 41.8 | 0.063794 | 0.042049 | 1.52E+00 | 0.129233 | -0.01862 | 0.146208 | 0.204281 | H. pylori | infectious diseas | 0.888627 |
| 285.2 | 0.031013 | 0.02045 | 1.516552 | 0.12938 | -0.00907 | 0.071095 | 0.978574 | Anemia of chronic disease | hematopoietic | 0.888134 |
| 253.5 | 0.317981 | 0.209685 | 1.516468 | 0.129401 | -0.09299 | 0.728957 | 0.809864 | Pituitary dwarfism | endocrine/metab | 0.888062 |
| 724.8 | 0.06202 | 0.04096 | 1.514158 | 0.129986 | -0.01826 | 0.1423 | 0.83251 | Other symptoms referable to back | musculoskeletal | 0.886104 |
| 367.9 | -0.07175 | 0.047399 | -1.51378 | 0.130081 | -0.16465 | 0.021149 | 0.228181 | Blindness and low vision | sense organs | 0.885787 |
| 793 | -0.08119 | 0.053684 | -1.51234 | 0.130448 | -0.18641 | 0.02403 | 0.851058 | Nonspecific abnormal findings on radiological | symptoms | 0.884563 |
| 323.2 | 0.32003 | 0.212115 | 1.508755 | 0.131361 | -0.09571 | 0.735767 | 0.462924 | Acute (transverse) myelitis | neurological | 0.881532 |
| 969 | 0.244038 | 0.161835 | 1.507948 | 0.131568 | -0.07315 | 0.561229 | 0.12937 | Poisoning by psychotropic agents | injuries & poisoni | 0.88085 |
| 459.7 | 0.045571 | 0.03023 | 1.507476 | 0.131689 | -0.01368 | 0.10482 | 0.7461 | Blood vessel replaced | circulatory syster | 0.880452 |
| 224.1 | -0.08858 | 0.058769 | -1.50732 | 0.131728 | -0.20377 | 0.026601 | 0.988976 | Benign neoplasm of eye, uveal | neoplasms | 0.880323 |
| 736.3 | 0.341684 | 0.22761 | 1.50118 | 0.133309 | -0.10442 | 0.787791 | 0.507329 | Acquired deformities of hip | musculoskeletal | 0.87514 |
| 251.8 | 0.305477 | 2.04E-01 | 1.497664 | 0.134221 | -0.09429 | 0.705248 | 0.446167 | Abnormality of secretion of glucagon or gastrir | endocrine/metab | 0.872181 |
| 634.1 | -0.10375 | 0.069354 | -1.49602 | 0.134649 | -0.23969 | 0.032177 | 0.549135 | Missed abortion/Hydatidiform mole | pregnancy compl | 0.870796 |
| 253.1 | -0.21852 | 0.146312 | -1.49352 | 0.135302 | -0.50529 | 0.068247 | 0.036617 | Pituitary hyperfunction | endocrine/metab | 0.868695 |
| 433.1 | 0.037384 | 0.02508 | 1.490572 | 0.136074 | -0.01177 | 0.086541 | 0.428791 | Occlusion and stenosis of precerebral arteries | circulatory syster | 0.866225 |
| 327.31 | 0.067314 | 0.045205 | 1.489082 | 0.136466 | -0.02129 | 0.155913 | 0.36804 | Central/nonobstructive sleep apnea | neurological | 0.864976 |
| 743 | -0.02441 | 0.016406 | -1.48799 | 0.136753 | -0.05657 | 0.007743 | 0.804866 | Osteoporosis, osteopenia and pathological fra | musculoskeletal | 0.864063 |
| 204.21 | 0.125504 | 0.084643 | 1.48275 | 0.138141 | -0.04039 | 0.291402 | 0.907212 | Myeloid leukemia, acute | neoplasms | 0.859677 |
| 531.1 | -0.09341 | 0.063159 | -1.47895 | 0.139155 | -0.2172 | 0.03038 | 0.948593 | Hemorrhage from gastrointestinal ulcer | digestive | 0.856502 |
| 795.81 | 0.179061 | 0.121099 | 1.478632 | 0.139239 | -0.05829 | 0.41641 | 0.256317 | Elevated carcinoembryonic antigen [CEA] | symptoms | 0.85624 |
| 526.1 | 0.274839 | 0.186004 | 1.477592 | 0.139517 | -0.08972 | 0.639401 | 0.79044 | Cysts of the jaws | digestive | 0.855373 |
| 327.4 | 0.023302 | 0.015814 | 1.473456 | 0.140628 | -0.00769 | 0.054297 | 0.322244 | Insomnia | neurological | 0.851928 |
| 440.21 | 0.257902 | 0.175091 | 1.472964 | 0.140761 | -0.08527 | 0.601074 | 0.021289 | Atherosclerosis of native arteries of the extrem | circulatory syster | 0.851519 |
| 425.8 | 0.154871 | 0.105362 | 1.469895 | 0.14159 | -0.05163 | 0.361376 | 0.940575 | Other cardiomyopathy | circulatory syster | 0.848967 |

| phecode | beta | se | zval | pval | ci.lb | ci.ub | QEp | phenotype | category | minuslogp |
|---|---|---|---|---|---|---|---|---|---|---|
| 735.3 | -0.04862 | 0.033088 | -1.46935 | 0.141739 | -0.11347 | 0.016234 | 0.470569 | Hallux valgus (Bunion) | musculoskeletal | 0.848511 |
| 939 | -0.02412 | 0.016453 | -1.46586 | 0.142685 | -0.05637 | 0.00813 | 0.562283 | Atopic/contact dermatitis due to other or unspe | dermatologic | 0.84562 |
| 274.2 | 0.114097 | 0.078042 | 1.462004 | 0.14374 | -0.03886 | 0.267056 | 0.257647 | Crystal arthropathies | endocrine/metab | 0.842422 |
| 411.9 | -0.07207 | 0.049376 | -1.45959 | 0.144404 | -0.16884 | 0.024706 | 0.420741 | Other acute and subacute forms of ischemic h | circulatory syster | 0.840421 |
| 415 | 0.047087 | 0.032341 | 1.455938 | 0.14541 | -0.0163 | 0.110475 | 0.200048 | Pulmonary heart disease | circulatory syster | 0.837406 |
| 791 | 0.066611 | 4.58E-02 | 1.455029 | 0.145661 | -0.02312 | 0.156337 | 0.727832 | Gangrene | symptoms | 0.836656 |
| 809 | 0.020631 | 0.01419 | 1.453893 | 0.145976 | -0.00718 | 0.048444 | 0.71194 | Fracture of unspecified bones | injuries & poisoni | 0.835718 |
| 526.8 | 0.428185 | 0.294719 | 1.452862 | 0.146262 | -0.14945 | 1.005823 | 0.228817 | Exostosis of jaw | digestive | 0.834868 |
| 755.4 | 0.232953 | 0.160505 | 1.451378 | 0.146675 | -0.08163 | 0.547536 | 0.67529 | Congenital anomalies of upper limb, including | congenital anoma | 0.833645 |
| 952 | 0.150667 | 0.103872 | 1.45051 | 0.146916 | -0.05292 | 0.354253 | 0.691569 | Spinal cord injury without evidence of spinal bo | injuries & poisoni | 0.83293 |
| 513 | 0.070848 | 0.048846 | 1.450453 | 0.146932 | -0.02489 | 0.166584 | 0.046397 | Respiratory abnormalities | respiratory | 0.832883 |
| 443 | 0.033835 | 0.023347 | 1.449181 | 0.147287 | -0.01193 | 0.079595 | 0.617164 | Peripheral vascular disease | circulatory syster | 0.831836 |
| 281.9 | 0.053178 | 0.036772 | 1.446149 | 0.148135 | -0.01889 | 0.12525 | 0.189412 | Deficiency anemias | hematopoietic | 0.829341 |
| 850 | 0.055692 | 0.03853 | 1.445416 | 0.148341 | -0.01983 | 0.13121 | 0.406926 | Hemorrhage or hematoma complicating a proc | injuries & poisoni | 0.828739 |
| 565.1 | -0.09193 | 0.063801 | -1.44089 | 0.149615 | -0.21698 | 0.033117 | 0.051295 | Anal and rectal polyp | digestive | 0.825025 |
| 287.3 | 0.033146 | 0.023036 | 1.43885 | 0.150193 | -0.012 | 0.078296 | 0.44884 | Thrombocytopenia | hematopoietic | 0.82335 |
| 333.4 | 0.201145 | 0.140098 | 1.435748 | 0.151074 | -0.07344 | 0.475732 | 0.050455 | Torsion dystonia | neurological | 0.82081 |
| 41.11 | 0.076642 | 0.053447 | 1.43E+00 | 0.151583 | -0.02811 | 0.181397 | 0.632993 | Methicillin sensitive Staphylococcus aureus | infectious diseas | 0.819351 |
| 550.4 | 0.050901 | 0.0356 | 1.429784 | 0.152779 | -0.01887 | 0.120676 | 0.725833 | Umbilical hernia | digestive | 0.815936 |
| 695.42 | 0.06517 | 0.045585 | 1.429641 | 0.15282 | -0.02417 | 0.154515 | 0.858637 | Systemic lupus erythematosus | dermatologic | 0.81582 |
| 528 | -0.03455 | 0.024171 | -1.42931 | 0.152914 | -0.08192 | 0.012826 | 0.68442 | Diseases of the oral soft tissues, excluding les | digestive | 0.815553 |
| 261.4 | -0.03153 | 0.022064 | -1.42888 | 0.153039 | -0.07477 | 0.011718 | 0.217447 | Vitamin D deficiency | endocrine/metab | 0.815197 |
| 796 | -0.04384 | 0.030778 | -1.42452 | 0.154297 | -0.10417 | 0.01648 | 0.518851 | Elevated prostate specific antigen [PSA] | genitourinary | 0.811644 |
| 420.22 | -0.11464 | 0.08051 | -1.42391 | 0.154471 | -0.27244 | 0.043158 | 0.492925 | Chronic pericarditis | circulatory syster | 0.811152 |
| 772.2 | 0.041358 | 0.029068 | 1.422777 | 0.154801 | -0.01562 | 0.098331 | 0.580773 | Spasm of muscle | symptoms | 0.810227 |
| 117.3 | 0.951237 | 0.668649 | 1.422626 | 0.154845 | -0.35929 | 2.261766 | 0.236823 | Blastomycotic infection | infectious diseas | 0.810104 |
| 575.8 | 0.051407 | 0.036296 | 1.416325 | 0.15668 | -0.01973 | 0.122546 | 0.603872 | Other disorders of biliary tract | digestive | 0.804985 |
| 569.2 | 0.051395 | 0.036345 | 1.414085 | 0.157337 | -0.01984 | 0.122631 | 0.49093 | Gastrointestinal complications | digestive | 0.803169 |
| 987 | -0.31883 | 0.226433 | -1.40806 | 0.159112 | -0.76263 | 0.124968 | 0.996612 | Toxic effect of other gases, fumes, or vapors | injuries & poisoni | 0.798296 |
| 535.9 | 0.06858 | 0.048799 | 1.405353 | 0.159916 | -0.02706 | 0.164225 | 0.109597 | Gastritis and duodenitis, NOS | digestive | 0.796107 |
| 374.6 | -0.11082 | 0.078906 | -1.40445 | 0.160185 | -0.26547 | 0.043834 | 0.095293 | Dermatochalasis | sense organs | 0.795378 |
| 528.1 | -0.05176 | 0.03688 | -1.40345 | 0.160483 | -0.12404 | 0.020524 | 0.691192 | Stomatitis and mucositis | digestive | 0.794572 |
| 349 | -0.05041 | 0.035919 | -1.40334 | 0.160516 | -0.12081 | 0.019994 | 0.54813 | Other and unspecified disorders of the nervous | neurological | 0.794481 |
| 255.13 | 0.568327 | 0.405045 | 1.403122 | 0.16058 | -0.22555 | 1.3622 | 0.79856 | Medulloadrenal hyperfunction | endocrine/metab | 0.794307 |
| 911 | -0.09262 | 0.066028 | -1.40266 | 0.160718 | -0.22203 | 0.036798 | 0.747913 | Blister | injuries & poisoni | 0.793936 |
| 600 | -0.03603 | 0.025716 | -1.40123 | 0.161146 | -0.08644 | 0.014368 | 0.552372 | Hyperplasia of prostate | genitourinary | 0.792781 |
| 613.8 | -0.05362 | 0.038315 | -1.39934 | 0.161711 | -0.12871 | 0.02148 | 0.26395 | Other specified disorders of breast | genitourinary | 0.79126 |
| 715.1 | 0.04709 | 0.03366 | 1.398973 | 0.161821 | -0.01888 | 0.113063 | 0.397202 | Sacroiliitis NEC | musculoskeletal | 0.790965 |
| 255 | 0.043468 | 0.031088 | 1.398228 | 0.162044 | -0.01746 | 0.1044 | 0.925187 | Disorders of adrenal glands | endocrine/metab | 0.790366 |
| 805 | 0.051631 | 0.036952 | 1.397243 | 0.162341 | -0.02079 | 0.124056 | 0.558285 | Fracture of vertebral column without mention c | injuries & poisoni | 0.789573 |
| 302.1 | 0.067002 | 0.048048 | 1.394481 | 0.163172 | -0.02717 | 0.161175 | 0.847192 | Decreased libido | mental disorders | 0.787353 |
| 1014 | 0.63459 | 0.455307 | 1.393763 | 0.163389 | -0.2578 | 1.526976 | 0.611245 | Effects of heat, cold and air pressure | other | 0.786776 |
| 362.31 | 0.309689 | 0.222218 | 1.393627 | 0.16343 | -0.12585 | 0.745227 | 0.00189 | Separation of retinal layers | sense organs | 0.786668 |
| 303.1 | 0.209588 | 0.150826 | 1.389604 | 0.164649 | -0.08602 | 0.505201 | 0.644123 | Dissociative disorder | mental disorders | 0.78344 |
| 275.2 | 0.249683 | 0.179734 | 1.389184 | 0.164777 | -0.10259 | 0.601955 | 0.558393 | Disorders of copper metabolism | endocrine/metab | 0.783104 |
| 627.5 | -0.08821 | 0.063501 | -1.38912 | 0.164796 | -0.21267 | 0.036249 | 0.864955 | Premature menopause and other ovarian failu | genitourinary | 0.783054 |
| 317 | 0.083394 | 0.060127 | 1.386953 | 0.165456 | -0.03445 | 0.201242 | 0.012925 | Alcohol-related disorders | mental disorders | 0.781317 |
| 512 | 0.018619 | 0.01346 | 1.383261 | 0.166585 | -0.00776 | 0.045 | 0.843669 | Other symptoms of respiratory system | respiratory | 0.778364 |

| phecode | beta | se | zval | pval | ci.lb | ci.ub | QEp | phenotype | category | minuslogp |
|---|---|---|---|---|---|---|---|---|---|---|
| 346.1 | 0.047805 | 0.034562 | 1.38316 | 0.166616 | -0.01994 | 0.115546 | 0.995025 | Nonspecific abnormal findings on radiological a | neurological | 0.778284 |
| 386.1 | 0.104309 | 0.075416 | 1.383114 | 0.16663 | -0.0435 | 0.252122 | 0.774748 | Meniere's disease | sense organs | 0.778247 |
| 379.4 | 0.102329 | 0.073995 | 1.382917 | 0.16669 | -0.0427 | 0.247357 | 0.117754 | Anomalies of pupillary function | sense organs | 0.77809 |
| 348.7 | 0.071624 | 0.051824 | 1.382066 | 0.166952 | -0.02995 | 0.173196 | 0.860089 | Coma | neurological | 0.77741 |
| 750.13 | -0.09225 | 0.066754 | -1.38193 | 0.166993 | -0.22309 | 0.038586 | 0.758974 | Congenital anomalies of mouth/tongue | congenital anoma | 0.777301 |
| 722 | 0.021426 | 0.015532 | 1.379465 | 0.167751 | -0.00902 | 0.051868 | 0.664553 | Intervertebral disc disorders | musculoskeletal | 0.775334 |
| 766 | 0.035286 | 0.02561 | 1.377836 | 0.168254 | -0.01491 | 0.085481 | 0.455595 | Neuralgia, neuritis, and radiculitis NOS | symptoms | 0.774035 |
| 586.4 | -0.04167 | 0.030263 | -1.37697 | 0.16852 | -0.10099 | 0.017643 | 0.948515 | Stricture/obstruction of ureter | genitourinary | 0.773348 |
| 793.2 | -0.04027 | 0.029253 | -1.37652 | 0.168659 | -0.0976 | 0.017067 | 0.472409 | Nonspecific abnormal findings on radiological a | symptoms | 0.77299 |
| 427.41 | 0.118587 | 0.086208 | 1.375599 | 0.168946 | -0.05038 | 0.287551 | 0.249117 | Ventricular fibrillation and flutter | circulatory syster | 0.772253 |
| 229 | -0.06478 | 0.047118 | -1.37479 | 0.169197 | -0.15713 | 0.027572 | 0.716279 | Benign neoplasm of unspecified sites | neoplasms | 0.771607 |
| 420.3 | -0.07067 | 0.051425 | -1.37429 | 0.169351 | -0.17146 | 0.030118 | 0.410027 | Endocarditis | circulatory syster | 0.771212 |
| 446.8 | 0.213844 | 0.156038 | 1.370462 | 0.170543 | -0.09198 | 0.519672 | 0.304756 | Thrombotic microangiopathy | circulatory syster | 0.768167 |
| 618 | 0.049516 | 0.036277 | 1.364952 | 0.172268 | -0.02159 | 0.120617 | 0.708825 | Genital prolapse | genitourinary | 0.763795 |
| 441 | 0.07924 | 5.81E-02 | 1.364889 | 0.172288 | -0.03455 | 0.193028 | 0.890634 | Vascular insufficiency of intestine | circulatory syster | 0.763745 |
| 456 | 0.046209 | 0.033882 | 1.363846 | 0.172616 | -0.0202 | 0.112616 | 0.215497 | Chronic venous insufficiency [CVI] | circulatory syster | 0.762919 |
| 274 | 0.035947 | 0.026386 | 1.362354 | 0.173086 | -0.01577 | 0.087662 | 0.495983 | Gout and other crystal arthropathies | endocrine/metab | 0.761738 |
| 700 | 0.056927 | 0.041952 | 1.356971 | 0.174791 | -0.0253 | 0.139151 | 0.277991 | Corns and callosities | dermatologic | 0.757482 |
| 596.5 | 0.037835 | 0.027893 | 1.356431 | 0.174962 | -0.01683 | 0.092503 | 0.393625 | Functional disorders of bladder | genitourinary | 0.757056 |
| 523.3 | 0.0848 | 0.062691 | 1.352655 | 0.176166 | -0.03807 | 0.207673 | 0.804693 | Periodontitis (acute or chronic) | digestive | 0.754078 |
| 260.1 | 0.064282 | 0.047552 | 1.351826 | 0.176431 | -0.02892 | 0.157481 | 0.404079 | Cachexia | endocrine/metab | 0.753426 |
| 613.1 | -0.07612 | 0.056317 | -1.35171 | 0.176468 | -0.1865 | 0.034255 | 0.351471 | Inflammatory disease of breast | genitourinary | 0.753334 |
| 215 | 0.064375 | 0.047636 | 1.351385 | 0.176572 | -0.02899 | 0.15774 | 0.128206 | Other benign neoplasm of connective and othe | neoplasms | 0.753078 |
| 523.1 | 0.088774 | 0.065768 | 1.349812 | 0.177076 | -0.04013 | 0.217676 | 0.637674 | Gingivitis | digestive | 0.75184 |
| 560.2 | 0.062719 | 0.046468 | 1.349736 | 0.177101 | -0.02836 | 0.153793 | 0.223327 | Impaction of intestine | digestive | 0.751779 |
| 316.1 | -0.06194 | 0.04597 | -1.34732 | 0.177877 | -0.15204 | 0.028163 | 0.598455 | Polyneuropathy due to drugs | mental disorders | 0.749881 |
| 947 | 0.043176 | 0.032063 | 1.346596 | 0.17811 | -0.01967 | 0.106019 | 0.845527 | Urticaria | dermatologic | 0.749311 |
| 380.4 | -0.05438 | 0.040385 | -1.34643 | 0.178165 | -0.13353 | 0.024778 | 0.083363 | Impacted cerumen | sense organs | 0.749178 |
| 201 | 0.174991 | 0.129995 | 1.346139 | 0.178258 | -0.07979 | 0.429776 | 0.466599 | Hodgkin's disease | neoplasms | 0.748952 |
| 496.3 | 0.042974 | 0.032032 | 1.341584 | 0.179731 | -0.01981 | 0.105756 | 0.652208 | Bronchiectasis | respiratory | 0.745377 |
| 363 | 0.169705 | 0.12652 | 1.34133 | 0.179813 | -0.07827 | 0.41768 | 0.054633 | Chorioretinal inflammations, scars, and other c | sense organs | 0.745178 |
| 198.1 | -0.03477 | 0.026049 | -1.33468 | 0.181981 | -0.08582 | 0.016288 | 0.560712 | Secondary malignancy of lymph nodes | neoplasms | 0.739974 |
| 613.5 | 0.040109 | 0.030058 | 1.334362 | 0.182085 | -0.0188 | 0.099022 | 0.671591 | Mastodynia | genitourinary | 0.739725 |
| 626.4 | -0.1628 | 0.12228 | -1.33136 | 0.183071 | -0.40246 | 0.076866 | 0.356793 | Premenstrual tension syndromes | genitourinary | 0.73738 |
| 625.1 | -0.0599 | 0.045058 | -1.32942 | 0.183709 | -0.14821 | 0.028411 | 0.560869 | Dyspareunia | genitourinary | 0.735871 |
| 594.1 | 0.029889 | 0.022494 | 1.328779 | 0.183921 | -0.0142 | 0.073976 | 0.365634 | Calculus of kidney | genitourinary | 0.735369 |
| 250.2 | 0.053924 | 0.040583 | 1.328752 | 0.18393 | -0.02562 | 0.133465 | 0.001529 | Type 2 diabetes | endocrine/metab | 0.735348 |
| 426.22 | 0.225252 | 0.171357 | 1.314517 | 0.188672 | -0.1106 | 0.561106 | 0.264786 | Mobitz II AV block | circulatory syster | 0.724292 |
| 783 | 0.022391 | 0.017046 | 1.313627 | 0.188972 | -0.01102 | 0.0558 | 0.485945 | Fever of unknown origin | symptoms | 0.723603 |
| 733.2 | 0.114167 | 0.086916 | 1.313534 | 0.189003 | -0.05618 | 0.284518 | 0.620536 | Cyst of bone | musculoskeletal | 0.723531 |
| 272.11 | -0.02101 | 0.01605 | -1.30932 | 0.190426 | -0.05247 | 0.010443 | 0.834565 | Hypercholesterolemia | endocrine/metab | 0.720274 |
| 733.6 | 0.141262 | 0.10793 | 1.308825 | 0.190594 | -0.07028 | 0.352801 | 0.006693 | Costochondritis | musculoskeletal | 0.719892 |
| 618.2 | 0.082873 | 0.063546 | 1.304148 | 0.192183 | -0.04167 | 0.207421 | 0.940377 | Uterine/Uterovaginal prolapse | genitourinary | 0.716285 |
| 259.2 | 0.208149 | 0.159873 | 1.301971 | 0.192926 | -0.1052 | 0.521494 | 0.958615 | Carcinoid syndrome | endocrine/metab | 0.714608 |
| 320 | -0.08151 | 0.062617 | -1.30177 | 0.192996 | -0.20424 | 0.041214 | 0.522993 | Meningitis | neurological | 0.714452 |
| 840 | -0.02248 | 0.017322 | -1.29807 | 0.194262 | -0.05643 | 0.011465 | 0.285904 | Sprains and strains | injuries & poisoni | 0.711613 |
| 550.5 | 0.039088 | 0.030137 | 1.297025 | 0.194623 | -0.01998 | 0.098155 | 0.32092 | Ventral hernia | digestive | 0.710807 |
| 722.8 | 0.061044 | 0.047152 | 1.294626 | 0.195449 | -0.03137 | 0.15346 | 0.230877 | Postlaminectomy syndrome | musculoskeletal | 0.708966 |

| phecode | beta | se | zval | pval | ci.lb | ci.ub | QEp | phenotype | category | minuslogp |
|---|---|---|---|---|---|---|---|---|---|---|
| 401.22 | 0.0278 | 0.021488 | 1.293717 | 0.195763 | -0.01432 | 0.069916 | 0.820799 | Hypertensive chronic kidney disease | circulatory syster | 0.708269 |
| 738.4 | 0.091183 | 0.070574 | 1.292035 | 0.196345 | -0.04714 | 0.229505 | 0.069778 | Acquired spondylolisthesis | musculoskeletal | 0.70698 |
| 402 | -0.08247 | 0.063872 | -1.29113 | 0.196658 | -0.20765 | 0.042719 | 0.004281 | Elevated blood pressure reading without diagn | circulatory syster | 0.706289 |
| 302 | 0.055179 | 0.042739 | 1.291047 | 0.196688 | -0.02859 | 0.138946 | 0.805748 | Sexual and gender identity disorders | mental disorders | 0.706223 |
| 871.1 | 0.072251 | 0.055978 | 1.290699 | 0.196808 | -0.03746 | 0.181966 | 0.692968 | Open wound of hand except finger(s) | injuries & poisoni | 0.705957 |
| 175 | -0.04206 | 0.032611 | -1.2898 | 0.197119 | -0.10598 | 0.021855 | 0.982694 | Acquired absence of breast | neoplasms | 0.705272 |
| 260.7 | -0.17243 | 0.133711 | -1.28954 | 0.197209 | -0.4345 | 0.089643 | 0.632865 | Polyphagia | endocrine/metab | 0.705074 |
| 172.3 | -0.12458 | 0.096644 | -1.28911 | 0.19736 | -0.314 | 0.064834 | 0.20742 | Carcinoma in situ of skin | neoplasms | 0.704742 |
| 199.4 | 0.171969 | 0.133752 | 1.285731 | 0.198537 | -0.09018 | 0.434118 | 0.488489 | Neurofibromatosis | neoplasms | 0.702159 |
| 1100 | -0.1176 | 0.091825 | -1.28071 | 0.200295 | -0.29757 | 0.062372 | 0.149417 | Family history | other | 0.698331 |
| 653 | -0.12273 | 0.095949 | -1.27908 | 0.200867 | -0.31078 | 0.06533 | 0.029945 | Problems associated with amniotic cavity and | pregnancy compl | 0.697091 |
| 728.1 | 0.233665 | 0.182715 | 1.278846 | 0.200951 | -0.12445 | 0.59178 | 0.409651 | Muscular calcification and ossification | musculoskeletal | 0.696909 |
| 346.3 | -0.15 | 0.117424 | -1.27744 | 0.201445 | -0.38015 | 0.080144 | 0.262629 | Nonspecific abnormal findings in cerebrospina | neurological | 0.695843 |
| 289.3 | -0.04345 | 0.034026 | -1.27685 | 0.201656 | -0.11014 | 0.023244 | 0.347884 | Personal history of diseases of blood and bloo | hematopoietic | 0.695389 |
| 818.1 | 0.372366 | 0.292012 | 1.275176 | 0.202247 | -0.19997 | 0.944699 | 0.164666 | Subdural hemorrhage (injury) | injuries & poisoni | 0.694118 |
| 365.2 | -0.0703 | 0.055244 | -1.27251 | 0.203192 | -0.17857 | 0.037978 | 0.636612 | Primary angle-closure glaucoma | sense organs | 0.692093 |
| 530.3 | 0.054124 | 0.042559 | 1.27172 | 0.203472 | -0.02929 | 0.137539 | 0.622213 | Stricture and stenosis of esophagus | digestive | 0.691494 |
| 563 | 0.020981 | 0.016505 | 1.271214 | 0.203652 | -0.01137 | 0.05333 | 0.188816 | Constipation | digestive | 0.69111 |
| 1002 | -0.02711 | 0.021326 | -1.27121 | 0.203655 | -0.06891 | 0.014688 | 0.497773 | Symptoms concerning nutrition, metabolism, a | other | 0.691106 |
| 196 | 0.021153 | 0.016643 | 1.270993 | 0.203731 | -0.01147 | 0.053773 | 0.489718 | Radiotherapy | neoplasms | 0.690942 |
| 521.4 | -1.17886 | 0.928893 | -1.2691 | 0.204405 | -2.99946 | 0.641737 | 1 | Tooth complications likely association with oth | digestive | 0.689509 |
| 526.3 | 0.174561 | 0.137663 | 1.268025 | 0.204789 | -0.09525 | 0.444376 | 0.96847 | Anomalies of jaw size/symmetry | digestive | 0.688693 |
| 735 | -0.04647 | 0.03673 | -1.26519 | 0.205802 | -0.11846 | 0.025519 | 0.106698 | Acquired foot deformities | musculoskeletal | 0.686551 |
| 979 | 0.026879 | 0.021245 | 1.265184 | 0.205805 | -0.01476 | 0.068518 | 0.543053 | Adverse drug events and drug allergies | injuries & poisoni | 0.686543 |
| 592.3 | 0.368103 | 0.29156 | 1.262531 | 0.206758 | -0.20334 | 0.93955 | 0.500009 | Urethral stricture due to infecton | genitourinary | 0.684538 |
| 246 | -0.04968 | 3.93E-02 | -1.26253 | 0.206759 | -0.1268 | 0.027442 | 0.101275 | Other disorders of thyroid | endocrine/metab | 0.684535 |
| 261.3 | 0.392954 | 0.312932 | 1.255716 | 0.209219 | -0.22038 | 1.006289 | 0.393562 | Vitamin C deficiencies | endocrine/metab | 0.679399 |
| 448 | -0.27665 | 0.220855 | -1.25265 | 0.210331 | -0.70952 | 0.156213 | 0.016503 | Disease of capillaries | circulatory syster | 0.677096 |
| 172.22 | -0.11795 | 0.0942 | -1.25214 | 0.21052 | -0.30258 | 0.066677 | 0.154572 | Squamous cell carcinoma | neoplasms | 0.676706 |
| 797.1 | 0.076932 | 0.061494 | 1.251051 | 0.210916 | -0.04359 | 0.197459 | 0.200341 | Cardiogenic shock | symptoms | 0.67589 |
| 451 | 0.057152 | 0.045882 | 1.245634 | 0.212899 | -0.03277 | 0.147079 | 0.722545 | Phlebitis and thrombophlebitis | circulatory syster | 0.671827 |
| 740.3 | 0.069582 | 0.055862 | 1.2456 | 0.212911 | -0.03991 | 0.179071 | 0.459016 | Osteoarthrosis involving more than one site, b | musculoskeletal | 0.671801 |
| 362.5 | 0.307206 | 0.247069 | 1.243399 | 0.213721 | -0.17704 | 0.791452 | 0.316156 | Toxic maculopathy of retina | sense organs | 0.670153 |
| 696.2 | 0.22876 | 0.184 | 1.243263 | 0.213771 | -0.13187 | 0.589394 | 0.696065 | Parapsoriasis | dermatologic | 0.670051 |
| 274.21 | 0.101327 | 0.081625 | 1.241372 | 0.214468 | -0.05866 | 0.26131 | 0.237208 | Chondrocalcinosis | endocrine/metab | 0.668637 |
| 197 | 0.017039 | 0.013736 | 1.240425 | 0.214818 | -0.00988 | 0.043961 | 0.550428 | Chemotherapy | neoplasms | 0.667929 |
| 526.9 | -0.27228 | 0.219613 | -1.2398 | 0.215051 | -0.70271 | 0.158158 | 0.015093 | Jaw disease NOS | digestive | 0.667459 |
| 603.1 | 0.075818 | 0.061161 | 1.23964 | 0.215108 | -0.04406 | 0.195691 | 0.604608 | Hydrocele | genitourinary | 0.667342 |
| 835 | 0.063163 | 0.051013 | 1.238194 | 0.215644 | -0.03682 | 0.163146 | 0.173759 | Internal derangement of knee | injuries & poisoni | 0.666262 |
| 288.3 | 0.070135 | 0.056651 | 1.238017 | 0.21571 | -0.0409 | 0.181169 | 0.387429 | Eosinophilia | hematopoietic | 0.66613 |
| 735.23 | -0.22547 | 0.18212 | -1.23801 | 0.215712 | -0.58242 | 0.131482 | 0.104195 | Hallux rigidus | musculoskeletal | 0.666126 |
| 528.41 | 0.218266 | 0.176614 | 1.235837 | 0.216519 | -0.12789 | 0.564422 | 0.556612 | Cyst of the salivary gland | digestive | 0.664503 |
| 240 | -0.05142 | 0.041609 | -1.23578 | 0.216539 | -0.13297 | 0.030133 | 0.623527 | Simple and unspecified goiter | endocrine/metab | 0.664463 |
| 348 | 0.031434 | 0.025468 | 1.234233 | 0.217116 | -0.01848 | 0.081351 | 0.691562 | Other conditions of brain | neurological | 0.663308 |
| 573.6 | 0.030661 | 0.024849 | 1.233888 | 0.217245 | -0.01804 | 0.079366 | 0.322603 | Nonspecific elevation of levels of transaminase | digestive | 0.663051 |
| 474.1 | -0.09529 | 0.077289 | -1.23295 | 0.217594 | -0.24678 | 0.05619 | 0.936019 | Acute tonsillitis | respiratory | 0.662353 |
| 41 | 0.021186 | 0.017195 | 1.23E+00 | 0.217898 | -0.01251 | 0.054887 | 0.410947 | Bacterial infection NOS | infectious diseas | 0.661747 |
| 755 | 0.068617 | 0.055694 | 1.232027 | 0.217939 | -0.04054 | 0.177776 | 0.888326 | Congenital anomalies of limbs | congenital anoma | 0.661665 |

| phecode | beta | se | zval | pval | ci.lb | ci.ub | QEp | phenotype | category | minuslogp |
|---|---|---|---|---|---|---|---|---|---|---|
| 516 | 0.049513 | 0.040298 | 1.228659 | 0.2192 | -0.02947 | 0.128495 | 0.79765 | Abnormal sputum | respiratory | 0.65916 |
| 79.9 | -0.04068 | 0.033112 | -1.22863 | 0.21921 | -0.10558 | 0.024216 | 0.778675 | Viremia, NOS | infectious diseas | 0.659139 |
| 530.7 | 0.158843 | 0.129578 | 1.22585 | 0.220255 | -0.09512 | 0.41281 | 0.769139 | Gastroesophageal laceration-hemorrhage syn | digestive | 0.657074 |
| 507 | 0.032715 | 0.026828 | 1.219432 | 0.22268 | -0.01987 | 0.085298 | 0.161522 | Pleurisy; pleural effusion | respiratory | 0.652319 |
| 687.3 | -0.06212 | 0.05094 | -1.21943 | 0.222683 | -0.16196 | 0.037723 | 0.498906 | Changes in skin texture | dermatologic | 0.652313 |
| 989 | 0.174158 | 0.142897 | 1.218765 | 0.222933 | -0.10592 | 0.454231 | 0.610298 | Toxic effect of other substances, chiefly nonme | injuries & poisoni | 0.651825 |
| 495 | 0.03155 | 0.02593 | 1.216721 | 0.22371 | -0.01927 | 0.082373 | 0.171921 | Asthma | respiratory | 0.650314 |
| 223 | 0.155377 | 0.127839 | 1.215406 | 0.224211 | -0.09518 | 0.405938 | 0.153543 | Benign neoplasm of kidney and other urinary c | neoplasms | 0.649342 |
| 182 | -0.06802 | 0.055988 | -1.21486 | 0.224419 | -0.17775 | 0.041717 | 0.58623 | Malignant neoplasm of uterus | neoplasms | 0.648941 |
| 260.21 | 0.509131 | 0.419336 | 1.214138 | 0.224695 | -0.31275 | 1.331014 | 0.046492 | Kwashiorkor | endocrine/metab | 0.648406 |
| 596.1 | -0.07217 | 0.05945 | -1.21389 | 0.224788 | -0.18869 | 0.044354 | 0.568926 | Bladder neck obstruction | genitourinary | 0.648227 |
| 745 | 0.037522 | 0.030926 | 1.213286 | 0.225021 | -0.02309 | 0.098136 | 0.028869 | Pain in joint | musculoskeletal | 0.647778 |
| 174.11 | 0.033741 | 0.027848 | 1.211593 | 0.225668 | -0.02084 | 0.088323 | 0.890472 | Malignant neoplasm of female breast | neoplasms | 0.64653 |
| 593.1 | 0.04116 | 0.033986 | 1.211095 | 0.225859 | -0.02545 | 0.107771 | 0.978047 | Gross hematuria | genitourinary | 0.646163 |
| 350.6 | -0.04415 | 0.036521 | -1.20903 | 0.22665 | -0.11573 | 0.027425 | 0.476435 | Disturbances of sensation of smell and taste | neurological | 0.644645 |
| 747.12 | -0.05928 | 0.049053 | -1.20841 | 0.226891 | -0.15542 | 0.036866 | 0.726084 | Valvular heart disease/ heart chambers | congenital anoma | 0.644182 |
| 276.8 | 0.105277 | 0.08715 | 1.207998 | 0.227048 | -0.06553 | 0.276088 | 0.504168 | Polydipsia | endocrine/metab | 0.643882 |
| 277.1 | 0.308616 | 0.255839 | 1.206292 | 0.227705 | -0.19282 | 0.810051 | 0.730642 | Disorders of porphyrin metabolism | endocrine/metab | 0.642627 |
| 110.13 | -0.04418 | 0.036902 | -1.19712 | 0.231261 | -0.1165 | 0.028151 | 0.824405 | Dermatophytosis of the body | infectious diseas | 0.635897 |
| 10 | 0.068287 | 0.057086 | 1.20E+00 | 0.231614 | -0.0436 | 0.180174 | 0.820199 | Tuberculosis | infectious diseas | 0.635235 |
| 743.21 | -0.0757 | 0.063431 | -1.19345 | 0.232692 | -0.20002 | 0.048621 | 0.303074 | Pathologic fracture of vertebrae | musculoskeletal | 0.633218 |
| 226 | -0.1318 | 0.110536 | -1.19233 | 0.233133 | -0.34844 | 0.084852 | 0.547472 | Benign neoplasm of thyroid glands | neoplasms | 0.632396 |
| 333.1 | -0.12088 | 0.101453 | -1.1915 | 0.233457 | -0.31973 | 0.077963 | 0.075988 | Essential tremor | neurological | 0.631792 |
| 962.3 | 0.201034 | 0.168897 | 1.190276 | 0.233938 | -0.13 | 0.532067 | 0.189025 | Hormones and synthetic substitutes causing a | injuries & poisoni | 0.630899 |
| 574.3 | 0.066077 | 0.05559 | 1.188648 | 0.234578 | -0.04288 | 0.175031 | 0.373727 | Cholecystitis without cholelithiasis | digestive | 0.629712 |
| 732 | 0.138529 | 0.117335 | 1.180628 | 0.23775 | -0.09144 | 0.368501 | 0.776049 | Osteochondropathies | musculoskeletal | 0.623879 |
| 334.2 | 0.198122 | 0.168099 | 1.178601 | 0.238557 | -0.13135 | 0.527591 | 0.22207 | Anterior horn cell disease | neurological | 0.622408 |
| 327.7 | 0.037472 | 0.03181 | 1.177972 | 0.238808 | -0.02488 | 0.099819 | 0.790683 | Sleep related movement disorders | neurological | 0.621951 |
| 565 | -0.02374 | 0.020163 | -1.17743 | 0.239024 | -0.06326 | 0.015778 | 0.450914 | Anal and rectal conditions | digestive | 0.621559 |
| 159.2 | 0.131914 | 0.112151 | 1.176224 | 0.239505 | -0.0879 | 0.351726 | 0.303491 | Malignant neoplasm of small intestine, includir | neoplasms | 0.620685 |
| 601.8 | 0.149424 | 0.127079 | 1.17583 | 0.239663 | -0.09965 | 0.398494 | 0.43346 | Other inflammatory disorders of male genital o | genitourinary | 0.620399 |
| 362.26 | -0.04644 | 0.039535 | -1.17458 | 0.240165 | -0.12392 | 0.03105 | 0.621239 | Macular puckering of retina | sense organs | 0.619491 |
| 530.6 | 0.212081 | 0.180645 | 1.174024 | 0.240385 | -0.14198 | 0.566138 | 0.374153 | Diverticulum of esophagus, acquired | digestive | 0.619092 |
| 250.3 | 0.047494 | 0.040507 | 1.172472 | 0.241008 | -0.0319 | 0.126886 | 0.078609 | Insulin pump user | endocrine/metab | 0.617969 |
| 225 | 0.059377 | 0.050721 | 1.17065 | 0.24174 | -0.04003 | 0.158788 | 0.475093 | Benign neoplasm of brain and other parts of n | neoplasms | 0.616652 |
| 331.1 | -0.07963 | 0.06807 | -1.16982 | 0.242072 | -0.21304 | 0.053784 | 0.635917 | Hydrocephalus | neurological | 0.616056 |
| 571.6 | 0.097259 | 0.08323 | 1.16856 | 0.242581 | -0.06587 | 0.260386 | 0.814313 | Primary biliary cirrhosis | digestive | 0.615143 |
| 279.7 | 0.032232 | 0.02764 | 1.166146 | 0.243555 | -0.02194 | 0.086404 | 0.293469 | Other immunological findings | endocrine/metab | 0.613402 |
| 282 | 0.068353 | 0.058656 | 1.165318 | 0.24389 | -0.04661 | 0.183316 | 0.188985 | Hereditary hemolytic anemias | hematopoietic | 0.612806 |
| 586.11 | 0.14369 | 0.123392 | 1.164505 | 0.244219 | -0.09815 | 0.385534 | 0.711644 | Small kidney | genitourinary | 0.61222 |
| 624 | -0.05114 | 0.044026 | -1.16164 | 0.245384 | -0.13743 | 0.035147 | 0.309282 | Symptoms involving female genital tract | genitourinary | 0.610155 |
| 335 | 0.139252 | 0.120246 | 1.158058 | 0.24684 | -0.09643 | 0.374931 | 0.19718 | Multiple sclerosis | neurological | 0.607584 |
| 112 | 0.027034 | 0.023356 | 1.157486 | 0.247074 | -0.01874 | 0.07281 | 0.472296 | Candidiasis | infectious diseas | 0.607173 |
| 287 | 0.035665 | 0.030849 | 1.156124 | 0.24763 | -0.0248 | 0.096127 | 0.170058 | Purpura and other hemorrhagic conditions | hematopoietic | 0.606196 |
| 704.12 | -0.1126 | 0.097498 | -1.15486 | 0.248146 | -0.30369 | 0.078495 | 0.230164 | Telogen effluvium | dermatologic | 0.605293 |
| 528.11 | -0.06632 | 0.057446 | -1.15441 | 0.248333 | -0.17891 | 0.046276 | 0.936615 | Stomatitis and mucositis (ulcerative) | digestive | 0.604966 |
| 593.2 | 0.058703 | 0.050924 | 1.152752 | 0.249012 | -0.04111 | 0.158512 | 0.063978 | Microscopic hematuria | genitourinary | 0.603779 |
| 604 | 0.058234 | 0.050536 | 1.152319 | 0.24919 | -0.04082 | 0.157283 | 0.949353 | Disorders of penis | genitourinary | 0.603469 |

| phecode | beta | se | zval | pval | ci.lb | ci.ub | QEp | phenotype | category | minuslogp |
|---|---|---|---|---|---|---|---|---|---|---|
| 81 | 0.043855 | 0.038082 | 1.151579 | 0.249494 | -0.03078 | 0.118494 | 0.957141 | Infection/inflammation of internal prosthetic de | infectious diseas | 0.60294 |
| 946 | 0.061709 | 0.053587 | 1.151562 | 0.249501 | -0.04332 | 0.166737 | 0.83037 | Anaphylactic shock NOS | injuries & poisoni | 0.602928 |
| 727 | 0.026298 | 0.022845 | 1.151175 | 0.24966 | -0.01848 | 0.071072 | 0.275616 | Other disorders of synovium, tendon, and burs | musculoskeletal | 0.602651 |
| 270.33 | -0.09465 | 0.082227 | -1.15113 | 0.249678 | -0.25582 | 0.066507 | 0.42585 | Amyloidosis | endocrine/metab | 0.602621 |
| 70 | 0.059782 | 0.051968 | 1.15E+00 | 0.249991 | -0.04207 | 0.161637 | 0.073499 | Viral hepatitis | infectious diseas | 0.602075 |
| 344 | 0.127032 | 0.110451 | 1.150127 | 0.250091 | -0.08945 | 0.343512 | 0.04422 | Other paralytic syndromes | neurological | 0.601901 |
| 658 | 0.152792 | 0.133072 | 1.148192 | 0.250889 | -0.10802 | 0.413608 | 0.490553 | Maternal complication of pregnancy affecting f | pregnancy compl | 0.600518 |
| 580.14 | 0.167564 | 0.146136 | 1.146635 | 0.251533 | -0.11886 | 0.453985 | 0.654195 | Chronic glomerulonephritis, NOS | genitourinary | 0.599406 |
| 189 | 0.040725 | 0.035529 | 1.14625 | 0.251692 | -0.02891 | 0.110361 | 0.766475 | Cancer of urinary organs (incl. kidney and blac | neoplasms | 0.599131 |
| 735.1 | -0.05339 | 0.046674 | -1.14379 | 0.252711 | -0.14487 | 0.038095 | 0.333927 | Flat foot | musculoskeletal | 0.597375 |
| 275.53 | 0.035155 | 0.030739 | 1.143645 | 0.252771 | -0.02509 | 0.095402 | 0.456434 | Disorders of phosphorus metabolism | endocrine/metab | 0.597273 |
| 364 | -0.05085 | 0.04449 | -1.14303 | 0.253026 | -0.13805 | 0.036345 | 0.340008 | Corneal opacity and other disorders of cornea | sense organs | 0.596835 |
| 444.2 | -0.19135 | 0.167779 | -1.1405 | 0.254076 | -0.52019 | 0.137488 | 0.388905 | Embolism and thrombosis of abdominal aorta | circulatory syster | 0.595036 |
| 264.9 | 0.174431 | 0.152955 | 1.140408 | 0.254116 | -0.12535 | 0.474216 | 0.196882 | Lack of normal physiological development, uns | endocrine/metab | 0.594967 |
| 284 | 0.032152 | 0.028209 | 1.139793 | 0.254373 | -0.02314 | 0.087441 | 0.510169 | Aplastic anemia | hematopoietic | 0.59453 |
| 347 | 0.217824 | 0.191161 | 1.139482 | 0.254502 | -0.15684 | 0.592492 | 0.394234 | Cataplexy and narcolepsy | neurological | 0.594308 |
| 609.11 | 0.213564 | 0.187622 | 1.138265 | 0.25501 | -0.15417 | 0.581297 | 0.919053 | Azoospermia and oligospermia | genitourinary | 0.593443 |
| 483 | 0.029214 | 0.025701 | 1.136698 | 0.255665 | -0.02116 | 0.079586 | 0.785719 | Acute bronchitis and bronchiolitis | respiratory | 0.592329 |
| 743.13 | -0.04479 | 0.039496 | -1.13405 | 0.256775 | -0.1222 | 0.03262 | 0.613933 | Other specified osteoporosis | musculoskeletal | 0.590448 |
| 580.11 | 0.122396 | 0.108285 | 1.130307 | 0.258347 | -0.08984 | 0.334631 | 0.53639 | Proliferative glomerulonephritis | genitourinary | 0.587796 |
| 287.4 | 0.140005 | 0.123934 | 1.129674 | 0.258614 | -0.1029 | 0.382912 | 0.330514 | Qualitative platelet defects | hematopoietic | 0.587348 |
| 701.3 | -0.06844 | 0.060743 | -1.12679 | 0.259831 | -0.1875 | 0.050609 | 0.747572 | Circumscribed scleroderma | dermatologic | 0.585309 |
| 153 | 0.041873 | 0.037163 | 1.126763 | 0.259843 | -0.03096 | 0.114711 | 0.350729 | Colorectal cancer | neoplasms | 0.585289 |
| 577.1 | 0.048971 | 0.04347 | 1.126554 | 0.259931 | -0.03623 | 0.13417 | 0.894088 | Acute pancreatitis | digestive | 0.585142 |
| 250 | 0.041614 | 0.036984 | 1.125185 | 0.260511 | -0.03087 | 0.114101 | 0.005491 | Diabetes mellitus | endocrine/metab | 0.584174 |
| 764 | 0.042733 | 0.038071 | 1.122471 | 0.261662 | -0.03188 | 0.11735 | 0.075715 | Sciatica | symptoms | 0.582259 |
| 842 | -0.04194 | 0.037406 | -1.12124 | 0.262184 | -0.11526 | 0.031373 | 0.128551 | Other sprains and strains | injuries & poisoni | 0.581393 |
| 949 | -0.01945 | 0.017387 | -1.11885 | 0.263203 | -0.05353 | 0.014625 | 0.757312 | Allergies, other | injuries & poisoni | 0.579709 |
| 938.1 | 0.07893 | 0.070779 | 1.11516 | 0.264782 | -0.05979 | 0.217653 | 0.951395 | Acute dermatitis due to solar radiation | dermatologic | 0.577112 |
| 560.3 | 0.10058 | 0.090537 | 1.110927 | 0.2666 | -0.07687 | 0.27803 | 0.535034 | Peritoneal or intestinal adhesions | digestive | 0.57414 |
| 687 | -0.01976 | 0.017786 | -1.11085 | 0.266631 | -0.05462 | 0.015102 | 0.740836 | Symptoms affecting skin | dermatologic | 0.57409 |
| 823 | -0.65133 | 0.588281 | -1.10718 | 0.268216 | -1.80434 | 0.501676 | 0.424333 | Torus fracture | injuries & poisoni | 0.571515 |
| 870.3 | -0.04704 | 0.042517 | -1.10647 | 0.268524 | -0.13037 | 0.036288 | 0.991858 | Other open wound of head and face | injuries & poisoni | 0.571018 |
| 585.4 | 0.028217 | 0.025504 | 1.106393 | 0.268556 | -0.02177 | 0.078204 | 0.618273 | Chronic kidney disease, Stage I or II | genitourinary | 0.570964 |
| 785 | 0.014678 | 0.013319 | 1.101985 | 0.270468 | -0.01143 | 0.040783 | 0.494173 | Abdominal pain | symptoms | 0.567884 |
| 204.3 | 0.375065 | 0.341205 | 1.099237 | 0.271665 | -0.29368 | 1.043815 | 0.576654 | Monocytic leukemia | neoplasms | 0.565966 |
| 751 | 0.034709 | 0.03164 | 1.097006 | 0.272639 | -0.0273 | 0.096722 | 0.918649 | Genitourinary congenital anomalies | congenital anoma | 0.564412 |
| 592.21 | 0.761626 | 0.694666 | 1.096391 | 0.272908 | -0.5999 | 2.123147 | 0.057475 | Urethral syndrome | genitourinary | 0.563984 |
| 747.1 | -0.03071 | 0.028041 | -1.09514 | 0.273457 | -0.08567 | 0.024251 | 0.82291 | Cardiac congenital anomalies | congenital anoma | 0.563111 |
| 277.4 | -0.07198 | 0.065875 | -1.09271 | 0.274521 | -0.2011 | 0.05713 | 0.132698 | Disorders of bilirubin excretion | endocrine/metab | 0.561425 |
| 353 | 0.049874 | 0.045665 | 1.092161 | 0.274762 | -0.03963 | 0.139376 | 0.685872 | Nerve root and plexus disorders | neurological | 0.561043 |
| 720 | 0.041027 | 0.037628 | 1.090351 | 0.275558 | -0.03272 | 0.114776 | 0.102417 | Spinal stenosis | musculoskeletal | 0.559786 |
| 573.4 | 0.058371 | 0.053602 | 1.088985 | 0.27616 | -0.04669 | 0.163429 | 0.996005 | Acute and subacute necrosis of liver | digestive | 0.558839 |
| 575.9 | -0.05347 | 0.049248 | -1.08582 | 0.27756 | -0.15 | 0.04305 | 0.834526 | Nonspecific abnormal findings on radiological | digestive | 0.556644 |
| 736.2 | 0.225397 | 0.207724 | 1.085078 | 0.277887 | -0.18174 | 0.63253 | 0.02129 | Acquired deformities of finger | musculoskeletal | 0.556132 |
| 528.6 | 0.127056 | 0.117203 | 1.084061 | 0.278338 | -0.10266 | 0.35677 | 0.677553 | Leukoplakia of oral mucosa | digestive | 0.555428 |
| 527.2 | 0.073485 | 0.067789 | 1.084036 | 0.278349 | -0.05938 | 0.206348 | 0.964807 | Sialoadenitis | digestive | 0.55541 |
| 200.1 | 0.067115 | 0.061949 | 1.083391 | 0.278635 | -0.0543 | 0.188532 | 0.803311 | Polycythemia vera | neoplasms | 0.554965 |

| phecode | beta | se | zval | pval | ci.lb | ci.ub | QEp | phenotype | category | minuslogp |
|---|---|---|---|---|---|---|---|---|---|---|
| 985 | 0.1803 | 0.166498 | 1.082892 | 0.278856 | -0.14603 | 0.50663 | 0.587164 | Toxic effect of other metals | injuries & poisoni | 0.554619 |
| 202 | -0.06018 | 0.055695 | -1.0806 | 0.279874 | -0.16935 | 0.048976 | 0.292194 | Cancer of other lymphoid, histiocytic tissue | neoplasms | 0.553038 |
| 743.9 | -0.01887 | 0.017489 | -1.07897 | 0.280603 | -0.05315 | 0.015408 | 0.631279 | Osteopenia or other disorder of bone and carti | musculoskeletal | 0.551908 |
| 378.5 | -0.14353 | 0.133083 | -1.07847 | 0.280823 | -0.40436 | 0.117312 | 0.179917 | Paralytic strabismus | sense organs | 0.551567 |
| 749.2 | 0.156384 | 0.145076 | 1.077943 | 0.281059 | -0.12796 | 0.440728 | 0.367348 | Congenital anomalies of skull and face bones | congenital anoma | 0.551203 |
| 379.2 | -0.03796 | 0.035287 | -1.07579 | 0.282021 | -0.10712 | 0.0312 | 0.210349 | Disorders of vitreous body | sense organs | 0.549719 |
| 957 | 0.314883 | 0.292829 | 1.075314 | 0.282234 | -0.25905 | 0.888818 | 0.239305 | Injury to other and unspecified nerves | injuries & poisoni | 0.549391 |
| 362.22 | 0.161902 | 0.150563 | 1.075309 | 0.282237 | -0.1332 | 0.457 | 0.119562 | Macular degeneration, wet | sense organs | 0.549387 |
| 752.1 | 0.113256 | 1.05E-01 | 1.074488 | 0.282604 | -0.09333 | 0.319844 | 0.708873 | Neural tube defects | congenital anoma | 0.548822 |
| 907 | 0.080723 | 0.075133 | 1.074404 | 0.282642 | -0.06653 | 0.227981 | 0.316363 | Injuries to the nervous system | injuries & poisoni | 0.548764 |
| 315.3 | 0.123826 | 0.115452 | 1.072539 | 0.283478 | -0.10245 | 0.350107 | 0.652729 | Mental retardation | mental disorders | 0.547481 |
| 41.21 | 0.139083 | 0.129809 | 1.07E+00 | 0.283972 | -0.11534 | 0.393504 | 0.503703 | Rheumatic fever / chorea | infectious diseas | 0.546725 |
| 360.2 | -0.09771 | 0.09121 | -1.07129 | 0.28404 | -0.27648 | 0.081056 | 0.674315 | Progressive myopia | sense organs | 0.546621 |
| 984 | 0.227333 | 0.212232 | 1.071152 | 0.284101 | -0.18863 | 0.6433 | 0.509831 | Toxic effect of lead and its compounds (includi | injuries & poisoni | 0.546527 |
| 642 | 0.097514 | 0.091059 | 1.070882 | 0.284223 | -0.08096 | 0.275987 | 0.070961 | Hypertension complicating pregnancy, childbir | pregnancy compl | 0.546341 |
| 749.1 | 0.2687 | 0.251211 | 1.069618 | 0.284791 | -0.22367 | 0.761066 | 0.289255 | Cleft palate | congenital anoma | 0.545473 |
| 972.1 | 0.171878 | 0.160692 | 1.069609 | 0.284795 | -0.14307 | 0.486829 | 0.424581 | Cardiac rhythm regulators causing adverse eff | injuries & poisoni | 0.545467 |
| 726 | 0.022904 | 0.021425 | 1.069045 | 0.28505 | -0.01909 | 0.064895 | 0.346796 | Peripheral enthesopathies and allied syndrom | musculoskeletal | 0.54508 |
| 529.1 | 0.098299 | 0.091955 | 1.068993 | 0.285073 | -0.08193 | 0.278527 | 0.430813 | Glossitis | digestive | 0.545045 |
| 429 | 0.035897 | 0.033614 | 1.067925 | 0.285554 | -0.02998 | 0.101778 | 0.050873 | Ill-defined descriptions and complications of he | circulatory syster | 0.544312 |
| 601.3 | 0.181102 | 0.169661 | 1.067434 | 0.285776 | -0.15143 | 0.513632 | 0.096681 | Orchitis and epididymitis | genitourinary | 0.543974 |
| 626.2 | 0.052999 | 0.049713 | 1.066106 | 0.286376 | -0.04444 | 0.150434 | 0.899781 | Dysmenorrhea | genitourinary | 0.543064 |
| 204.2 | 0.078225 | 0.073633 | 1.062359 | 0.288073 | -0.06609 | 0.222544 | 0.802039 | Myeloid leukemia | neoplasms | 0.540498 |
| 480.13 | 0.104615 | 0.098888 | 1.057915 | 0.290094 | -0.0892 | 0.298431 | 0.356683 | MRSA pneumonia | respiratory | 0.537461 |
| 257 | 0.053731 | 0.050803 | 1.057644 | 0.290218 | -0.04584 | 0.153302 | 0.230758 | Testicular dysfunction | endocrine/metab | 0.537276 |
| 803.3 | -0.07022 | 0.066412 | -1.05739 | 0.290335 | -0.20039 | 0.059942 | 0.5462 | Fracture of clavicle or scapula | injuries & poisoni | 0.5371 |
| 257.1 | 0.053788 | 0.050924 | 1.056244 | 0.290857 | -0.04602 | 0.153598 | 0.23034 | Testicular hypofunction | endocrine/metab | 0.536321 |
| 433.31 | 0.046086 | 0.043633 | 1.056237 | 0.29086 | -0.03943 | 0.131605 | 0.095401 | Transient cerebral ischemia | circulatory syster | 0.536316 |
| 994.21 | 0.047293 | 0.04485 | 1.054474 | 0.291666 | -0.04061 | 0.135197 | 0.37671 | Septic shock | injuries & poisoni | 0.535114 |
| 737.2 | 0.250745 | 0.237891 | 1.054035 | 0.291867 | -0.21551 | 0.717003 | 0.212352 | Lordosis (acquired) | musculoskeletal | 0.534815 |
| 743.12 | -0.06597 | 0.062696 | -1.05223 | 0.292695 | -0.18885 | 0.056911 | 0.095199 | Senile osteoporosis | musculoskeletal | 0.533584 |
| 527.7 | 0.040467 | 0.038606 | 1.048202 | 0.294546 | -0.0352 | 0.116133 | 0.735111 | Disturbance of salivary secretion | digestive | 0.530847 |
| 587 | 0.03942 | 0.03763 | 1.047553 | 0.294845 | -0.03433 | 0.113173 | 0.891101 | Kidney replaced by transpant | genitourinary | 0.530407 |
| 134 | 0.103973 | 0.099346 | 1.046574 | 0.295296 | -0.09074 | 0.298687 | 0.721732 | Helminthiases | infectious diseas | 0.529742 |
| 357 | 0.018653 | 0.017833 | 1.045961 | 0.295579 | -0.0163 | 0.053606 | 0.598495 | Inflammatory and toxic neuropathy | neurological | 0.529327 |
| 253.2 | -0.13057 | 0.125076 | -1.04393 | 0.296518 | -0.37572 | 0.114574 | 0.216236 | Pituitary hypofunction | endocrine/metab | 0.527949 |
| 536.7 | -0.06545 | 0.062705 | -1.04379 | 0.296582 | -0.18835 | 0.057449 | 0.710707 | Complications of gastrostomy, colostomy and | digestive | 0.527856 |
| 242.31 | 0.163378 | 0.157211 | 1.039226 | 0.2987 | -0.14475 | 0.471506 | 0.566058 | Thyrotoxic exophthalmos | endocrine/metab | 0.524765 |
| 202.2 | -0.04633 | 0.044587 | -1.03907 | 0.298771 | -0.13372 | 0.04106 | 0.393104 | Non-Hodgkins lymphoma | neoplasms | 0.524661 |
| 642.1 | 0.163742 | 0.157612 | 1.038897 | 0.298853 | -0.14517 | 0.472655 | 0.028899 | Preeclampsia and eclampsia | pregnancy compl | 0.524543 |
| 272 | 0.015884 | 0.015291 | 1.038777 | 0.298908 | -0.01409 | 0.045853 | 0.302938 | Disorders of lipoid metabolism | endocrine/metab | 0.524462 |
| 526.42 | 0.065811 | 0.063423 | 1.037643 | 0.299436 | -0.0585 | 0.190118 | 0.316645 | Arthralgia/ankylosis of temporomandibular join | digestive | 0.523696 |
| 612.3 | 0.156268 | 0.150602 | 1.037622 | 0.299446 | -0.13891 | 0.451443 | 0.65811 | Congenital anomalies of breast | genitourinary | 0.523681 |
| 614.53 | 0.117394 | 0.113288 | 1.036247 | 0.300087 | -0.10465 | 0.339435 | 0.706672 | Cyst or abscess of Bartholin's gland | genitourinary | 0.522753 |
| 362.1 | -0.29784 | 0.287527 | -1.03586 | 0.300269 | -0.86138 | 0.265706 | 0.6437 | Retinopathy of prematurity | sense organs | 0.52249 |
| 200 | 0.037943 | 0.036742 | 1.032677 | 0.301755 | -0.03407 | 0.109957 | 0.331171 | Myeloproliferative disease | neoplasms | 0.520346 |
| 816 | 0.27599 | 0.267282 | 1.032582 | 0.3018 | -0.24787 | 0.799853 | 0.188969 | Cerebral laceration and contusion | injuries & poisoni | 0.520281 |
| 442.1 | 0.036881 | 0.03572 | 1.032515 | 0.301831 | -0.03313 | 0.10689 | 0.802676 | Aortic aneurysm | circulatory syster | 0.520236 |

| phecode | beta | se | zval | pval | ci.lb | ci.ub | QEp | phenotype | category | minuslogp |
|---|---|---|---|---|---|---|---|---|---|---|
| 721.8 | 0.047703 | 0.046202 | 1.032483 | 0.301846 | -0.04285 | 0.138257 | 0.871062 | Other allied disorders of spine | musculoskeletal | 0.520215 |
| 695.22 | 0.32556 | 0.315553 | 1.03171 | 0.302208 | -0.29291 | 0.944033 | 0.156797 | Pemphigus and pemphigoid | dermatologic | 0.519694 |
| 187.8 | 0.394416 | 0.38238 | 1.031477 | 0.302317 | -0.35503 | 1.143866 | 0.247767 | Neoplasm of uncertain behavior of male genita | neoplasms | 0.519537 |
| 588 | 0.032213 | 0.031277 | 1.029928 | 0.303044 | -0.02909 | 0.093514 | 0.808894 | Disorders resulting from impaired renal functio | genitourinary | 0.518495 |
| 371.2 | -0.03538 | 0.034444 | -1.02727 | 0.304292 | -0.10289 | 0.032126 | 0.714155 | Conjunctivitis, noninfectious | sense organs | 0.51671 |
| 380.1 | -0.03369 | 0.032827 | -1.02641 | 0.304696 | -0.09803 | 0.030646 | 0.562961 | Otitis externa | sense organs | 0.516133 |
| 254 | 0.247766 | 0.241613 | 1.025465 | 0.305144 | -0.22579 | 0.72132 | 0.49853 | Diseases of thymus gland | endocrine/metab | 0.515495 |
| 573.9 | 0.021757 | 0.021236 | 1.024553 | 0.305574 | -0.01986 | 0.063379 | 0.62255 | Abnormal serum enzyme levels | digestive | 0.514883 |
| 446.9 | -0.07153 | 0.070008 | -1.02178 | 0.306886 | -0.20875 | 0.065681 | 0.838756 | Arteritis NOS | circulatory syster | 0.513023 |
| 282.5 | 0.132534 | 0.129718 | 1.021706 | 0.30692 | -0.12171 | 0.386776 | 0.293824 | Sickle cell anemia | hematopoietic | 0.512975 |
| 253.4 | -0.10716 | 1.05E-01 | -1.0208 | 0.307349 | -0.31291 | 0.098591 | 0.845521 | Anterior pituitary disorders | endocrine/metab | 0.512368 |
| 622 | -0.05147 | 0.05047 | -1.01981 | 0.307818 | -0.15039 | 0.047449 | 0.239871 | Polyp of female genital organs | genitourinary | 0.511705 |
| 426.31 | 0.038505 | 0.037798 | 1.018706 | 0.308342 | -0.03558 | 0.112586 | 0.662871 | Right bundle branch block | circulatory syster | 0.510967 |
| 345.1 | 0.041963 | 0.041217 | 1.018104 | 0.308628 | -0.03882 | 0.122747 | 0.933348 | Epilepsy | neurological | 0.510564 |
| 686 | -0.02327 | 0.022859 | -1.01776 | 0.308791 | -0.06807 | 0.021538 | 0.285219 | Other local infections of skin and subcutaneou | dermatologic | 0.510335 |
| 327.71 | 0.043422 | 0.0427 | 1.016906 | 0.309198 | -0.04027 | 0.127113 | 0.815474 | Restless legs syndrome | neurological | 0.509763 |
| 225.1 | 0.044043 | 0.043456 | 1.013528 | 0.310808 | -0.04113 | 0.129215 | 0.638141 | Benign neoplasm of brain, cranial nerves, mer | neoplasms | 0.507508 |
| 771.2 | 0.045583 | 0.045057 | 1.011682 | 0.31169 | -0.04273 | 0.133893 | 0.305593 | Cramp of limb | symptoms | 0.506277 |
| 751.2 | 0.034975 | 0.034588 | 1.011188 | 0.311926 | -0.03282 | 0.102767 | 0.905062 | Congenital anomalies of urinary system | congenital anoma | 0.505948 |
| 1007 | -0.1077 | 0.106952 | -1.00704 | 0.313916 | -0.31733 | 0.101917 | 0.225155 | Injury to blood vessels | other | 0.503186 |
| 636 | -0.04323 | 0.042966 | -1.00616 | 0.314337 | -0.12744 | 0.040981 | 0.574668 | Early or threatened labor; hemorrhage in early | pregnancy compl | 0.502604 |
| 281 | 0.031342 | 0.031234 | 1.003459 | 0.315639 | -0.02988 | 0.09256 | 0.491957 | Other deficiency anemia | hematopoietic | 0.500809 |
| 440 | 0.041363 | 0.041258 | 1.002541 | 0.316082 | -0.0395 | 0.122228 | 0.044716 | Atherosclerosis | circulatory syster | 0.5002 |
| 251 | 0.158262 | 0.158116 | 1.000923 | 0.316864 | -0.15164 | 0.468163 | 0.732519 | Other disorders of pancreatic internal secretion | endocrine/metab | 0.499127 |
| 193 | -0.0425 | 0.042598 | -0.99779 | 0.318383 | -0.12599 | 0.040986 | 0.349004 | Thyroid cancer | neoplasms | 0.497051 |
| 443.9 | 0.040145 | 0.040288 | 0.996463 | 0.319025 | -0.03882 | 0.119108 | 0.346786 | Peripheral vascular disease, unspecified | circulatory syster | 0.496175 |
| 381.11 | 0.06466 | 0.064924 | 0.995939 | 0.31928 | -0.06259 | 0.191909 | 0.091752 | Suppurative and unspecified otitis media | sense organs | 0.495829 |
| 261 | -0.02927 | 0.029416 | -0.99502 | 0.319728 | -0.08692 | 0.028384 | 0.067597 | Vitamin deficiency | endocrine/metab | 0.49522 |
| 871.3 | -0.08021 | 0.080618 | -0.99494 | 0.319763 | -0.23822 | 0.077798 | 0.481321 | Open wound of foot except toe(s) alone | injuries & poisoni | 0.495171 |
| 8.5 | 0.043897 | 0.044167 | 9.94E-01 | 0.320273 | -0.04267 | 0.130462 | 0.137359 | Bacterial enteritis | infectious diseas | 0.49448 |
| 709.7 | 0.045374 | 0.045812 | 0.990439 | 0.321959 | -0.04442 | 0.135164 | 0.43886 | Unspecified diffuse connective tissue disease | dermatologic | 0.492199 |
| 272.1 | 0.015075 | 0.015248 | 0.988608 | 0.322855 | -0.01481 | 0.044961 | 0.280568 | Hyperlipidemia | endocrine/metab | 0.490993 |
| 795.82 | 0.091043 | 0.092314 | 0.986239 | 0.324016 | -0.08989 | 0.271975 | 0.259999 | Elevated cancer antigen 125 [CA 125] | symptoms | 0.489434 |
| 365.11 | -0.04409 | 0.044717 | -0.98599 | 0.32414 | -0.13174 | 0.043554 | 0.328299 | Primary open angle glaucoma | sense organs | 0.489267 |
| 250.5 | 0.164317 | 0.166683 | 0.985804 | 0.32423 | -0.16238 | 0.491009 | 0.115259 | Glycosuria or Acetonuria | endocrine/metab | 0.489147 |
| 365.1 | -0.03599 | 0.036514 | -0.9857 | 0.324281 | -0.10756 | 0.035574 | 0.547507 | Open-angle glaucoma | sense organs | 0.489078 |
| 568.1 | 0.028755 | 0.029184 | 0.985296 | 0.324479 | -0.02844 | 0.085955 | 0.300915 | Peritoneal adhesions (postoperative) (postinfe | digestive | 0.488814 |
| 790.9 | 0.299958 | 0.305477 | 0.981935 | 0.326132 | -0.29877 | 0.898681 | 0.029136 | Abnormal arterial blood gases | symptoms | 0.486607 |
| 366.3 | 0.39947 | 0.406856 | 0.981846 | 0.326176 | -0.39795 | 1.196892 | 0.582764 | Traumatic cataract | sense organs | 0.486548 |
| 755.3 | 0.271093 | 0.276633 | 0.979972 | 0.3271 | -0.2711 | 0.813284 | 0.185679 | Congenital anomaly of fingers/toes | congenital anoma | 0.48532 |
| 153.3 | 0.075975 | 0.077623 | 0.97877 | 0.327693 | -0.07616 | 0.228112 | 0.107215 | Malignant neoplasm of rectum, rectosigmoid ju | neoplasms | 0.484532 |
| 173 | -0.04971 | 0.050797 | -0.97862 | 0.327768 | -0.14927 | 0.049849 | 0.053461 | Neoplasm of uncertain behavior of skin | neoplasms | 0.484433 |
| 573.3 | 0.054794 | 0.056026 | 0.97802 | 0.328064 | -0.05501 | 0.164603 | 0.026434 | Hepatomegaly | digestive | 0.484041 |
| 574.11 | -0.06745 | 0.068985 | -0.97774 | 0.328203 | -0.20266 | 0.067759 | 0.661333 | Cholelithiasis with acute cholecystitis | digestive | 0.483857 |
| 930 | -0.02615 | 0.026838 | -0.97431 | 0.329901 | -0.07875 | 0.026453 | 0.448811 | Allergic reaction to food | injuries & poisoni | 0.481616 |
| 535.1 | 0.09177 | 0.094296 | 0.973214 | 0.330447 | -0.09305 | 0.276588 | 0.084162 | Acute gastritis | digestive | 0.480898 |
| 191 | 0.041144 | 0.042342 | 0.971703 | 0.331198 | -0.04185 | 0.124134 | 0.640776 | Manlignant and unknown neoplasms of brain a | neoplasms | 0.479912 |
| 378 | -0.05622 | 0.057969 | -0.96981 | 0.33214 | -0.16984 | 0.057398 | 0.169941 | Strabismus and other disorders of binocular ey | sense organs | 0.478678 |

| phecode | beta | se | zval | pval | ci.lb | ci.ub | QEp | phenotype | category | minuslogp |
|---|---|---|---|---|---|---|---|---|---|---|
| 578.8 | -0.0351 | 0.036194 | -0.96966 | 0.332214 | -0.10603 | 0.035843 | 0.136379 | Hemorrhage of rectum and anus | digestive | 0.478582 |
| 729.1 | 0.225279 | 0.232422 | 0.969268 | 0.332412 | -0.23026 | 0.680817 | 0.573357 | Rheumatism, unspecified and fibrositis | musculoskeletal | 0.478324 |
| 420.2 | 0.029831 | 0.030842 | 0.967229 | 0.33343 | -0.03062 | 0.09028 | 0.438758 | Pericarditis | circulatory syster | 0.476996 |
| 871.2 | -0.03998 | 0.041481 | -0.96377 | 0.33516 | -0.12128 | 0.041323 | 0.666392 | Open wound of finger(s) | injuries & poisoni | 0.474748 |
| 655.1 | -0.11121 | 0.115561 | -0.96233 | 0.335883 | -0.3377 | 0.115288 | 0.004701 | Abnormality in fetal heart rate or rhythm | pregnancy compl | 0.473812 |
| 295.2 | 0.073411 | 0.076315 | 0.961949 | 0.336075 | -0.07616 | 0.222985 | 0.919201 | Paranoid disorders | mental disorders | 0.473563 |
| 292.5 | 0.061721 | 0.064173 | 0.961789 | 0.336156 | -0.06406 | 0.187499 | 0.515764 | Transient alteration of awareness | mental disorders | 0.47346 |
| 473 | 0.021406 | 0.022273 | 0.961059 | 0.336523 | -0.02225 | 0.065061 | 0.595711 | Diseases of the larynx and vocal cords | respiratory | 0.472986 |
| 227.2 | -0.08823 | 0.091983 | -0.95916 | 0.33748 | -0.26851 | 0.092058 | 0.295487 | Benign neoplasm of parathyroid gland | neoplasms | 0.471752 |
| 611.1 | -0.03185 | 0.033232 | -0.95845 | 0.337835 | -0.09699 | 0.033283 | 0.13635 | Abnormal mammogram | genitourinary | 0.471295 |
| 368.1 | 0.136141 | 0.142143 | 0.957773 | 0.338177 | -0.14245 | 0.414736 | 0.108553 | Amblyopia | sense organs | 0.470856 |
| 172 | -0.06355 | 0.066456 | -0.95628 | 0.33893 | -0.1938 | 0.066701 | 0.065359 | Skin cancer | neoplasms | 0.46989 |
| 379 | -0.02918 | 0.030558 | -0.955 | 0.33958 | -0.08907 | 0.030709 | 0.187821 | Other disorders of eye | sense organs | 0.469058 |
| 346.2 | 0.146836 | 0.153815 | 0.954629 | 0.339765 | -0.15464 | 0.448307 | 0.129465 | Nonspecific abnormal results of function study | neurological | 0.468821 |
| 385 | 0.07983 | 0.083866 | 0.951878 | 0.341159 | -0.08454 | 0.244204 | 0.712863 | Other disorders of middle ear and mastoid | sense organs | 0.467043 |
| 772.1 | -0.05391 | 0.056783 | -0.94949 | 0.342372 | -0.16521 | 0.057378 | 0.856406 | Muscular wasting and disuse atrophy | symptoms | 0.465502 |
| 261.41 | 0.124491 | 1.31E-01 | 0.946772 | 0.343755 | -0.13322 | 0.382207 | 0.698402 | Rickets or osteomalacia | endocrine/metab | 0.463751 |
| 395.1 | -0.0232 | 0.024511 | -0.94668 | 0.343803 | -0.07124 | 0.024836 | 0.797454 | Nonrheumatic mitral valve disorders | circulatory syster | 0.46369 |
| 346 | 0.107784 | 0.113892 | 0.946363 | 0.343963 | -0.11544 | 0.331009 | 0.253477 | Abnormal findings on study of brain and/or ner | neurological | 0.463488 |
| 149.2 | 0.173287 | 0.1837 | 0.943314 | 0.34552 | -0.18676 | 0.533332 | 0.237216 | Cancer of nasopharynx | neoplasms | 0.461527 |
| 426.25 | -0.22832 | 0.242091 | -0.9431 | 0.345628 | -0.70281 | 0.246172 | 0.036065 | Other heart block | circulatory syster | 0.461391 |
| 292.11 | -0.05007 | 0.05351 | -0.93569 | 0.349435 | -0.15495 | 0.054809 | 0.921928 | Aphasia | mental disorders | 0.456634 |
| 303 | 0.037324 | 0.039976 | 0.933648 | 0.350485 | -0.04103 | 0.115676 | 0.828749 | Psychogenic and somatoform disorders | mental disorders | 0.45533 |
| 599 | -0.01303 | 0.013953 | -0.93355 | 0.350536 | -0.04037 | 0.014322 | 0.698137 | Other symptoms/disorders or the urinary syste | genitourinary | 0.455267 |
| 389.3 | 0.074157 | 0.079483 | 0.932986 | 0.350827 | -0.08163 | 0.229942 | 0.953681 | Degenerative and vascular disorders of ear | sense organs | 0.454906 |
| 612.1 | -0.10842 | 0.116234 | -0.93275 | 0.350948 | -0.33623 | 0.119397 | 0.787688 | Galactorrhea | genitourinary | 0.454757 |
| 588.2 | 0.029895 | 0.032077 | 0.931969 | 0.351352 | -0.03297 | 0.092764 | 0.679957 | Secondary hyperparathyroidism (of renal origi | genitourinary | 0.454257 |
| 755.6 | -0.17789 | 0.190885 | -0.93191 | 0.351381 | -0.55202 | 0.196239 | 0.069865 | Other congenital anomalies of lower limb, inclu | congenital anom | 0.454222 |
| 728 | 0.055034 | 0.059279 | 0.928391 | 0.353205 | -0.06115 | 0.17122 | 0.62762 | Disorders of muscle, ligament, and fascia | musculoskeletal | 0.451973 |
| 695.2 | 0.131372 | 0.142377 | 0.922708 | 0.35616 | -0.14768 | 0.410425 | 0.26964 | Bullous dermatoses | dermatologic | 0.448355 |
| 285 | 0.02515 | 0.027273 | 0.922141 | 0.356455 | -0.0283 | 0.078604 | 0.069221 | Other anemias | hematopoietic | 0.447995 |
| 112.3 | 0.09253 | 0.100779 | 0.91815 | 0.35854 | -0.10499 | 0.290052 | 0.110602 | Candidiasis of skin and nails | infectious diseas | 0.445462 |
| 447.1 | -0.07203 | 0.078486 | -0.91777 | 0.358741 | -0.22586 | 0.081798 | 0.151132 | Stricture of artery | circulatory syster | 0.445219 |
| 195.3 | -0.12213 | 0.133103 | -0.91757 | 0.358845 | -0.38301 | 0.138746 | 0.011833 | Malignant neoplasm of head, face, and neck | neoplasms | 0.445094 |
| 472 | -0.0221 | 0.0241 | -0.91719 | 0.359046 | -0.06934 | 0.025131 | 0.714531 | Chronic pharyngitis and nasopharyngitis | respiratory | 0.44485 |
| 578.2 | 0.023891 | 0.026085 | 0.915874 | 0.359733 | -0.02724 | 0.075016 | 0.186684 | Blood in stool | digestive | 0.44402 |
| 459.9 | 0.018268 | 0.019948 | 0.915779 | 0.359783 | -0.02083 | 0.057365 | 0.581771 | Circulatory disease NEC | circulatory syster | 0.443959 |
| 495.2 | 0.025559 | 0.02798 | 0.913458 | 0.361002 | -0.02928 | 0.080398 | 0.751522 | Asthma with exacerbation | respiratory | 0.44249 |
| 250.42 | 0.012986 | 0.014241 | 0.9119 | 0.361822 | -0.01493 | 0.040898 | 0.363176 | Other abnormal glucose | endocrine/metab | 0.441506 |
| 389.5 | 0.117651 | 0.129197 | 0.910631 | 0.36249 | -0.13557 | 0.370872 | 0.079081 | Disorders of acoustic nerve | sense organs | 0.440704 |
| 149.5 | -0.14522 | 0.159604 | -0.90988 | 0.362883 | -0.45804 | 0.167597 | 0.005819 | Hx of malignant neoplasm of oral cavity and pl | neoplasms | 0.440233 |
| 912 | -0.05351 | 0.058862 | -0.90912 | 0.363289 | -0.16888 | 0.061855 | 0.187143 | Insect bite | injuries & poisoni | 0.439747 |
| 300.9 | 0.04028 | 0.044375 | 0.907707 | 0.364033 | -0.04669 | 0.127253 | 0.621529 | Posttraumatic stress disorder | mental disorders | 0.438859 |
| 280.1 | 0.022443 | 0.024762 | 0.906355 | 0.364748 | -0.02609 | 0.070976 | 0.396868 | Iron deficiency anemias, unspecified or not du | hematopoietic | 0.438007 |
| 287.1 | -0.15058 | 0.166135 | -0.90635 | 0.364749 | -0.47619 | 0.175042 | 0.036243 | Spontaneous ecchymoses | hematopoietic | 0.438005 |
| 592.11 | 0.020044 | 0.022118 | 0.906232 | 0.364813 | -0.02331 | 0.063396 | 0.444921 | Acute cystitis | genitourinary | 0.43793 |
| 707 | 0.024649 | 0.027312 | 0.902518 | 0.366782 | -0.02888 | 0.078179 | 0.721484 | Chronic ulcer of skin | dermatologic | 0.435593 |
| 586 | 0.015084 | 0.01673 | 0.901573 | 0.367284 | -0.01771 | 0.047874 | 0.567446 | Other disorders of the kidney and ureters | genitourinary | 0.434998 |

| phecode | beta | se | zval | pval | ci.lb | ci.ub | QEp | phenotype | category | minuslogp |
|---|---|---|---|---|---|---|---|---|---|---|
| 277.7 | 0.038304 | 0.042596 | 0.899248 | 0.368521 | -0.04518 | 0.12179 | 0.387241 | Dysmetabolic syndrome X | endocrine/metab | 0.433538 |
| 250.15 | 0.276372 | 0.307961 | 0.897425 | 0.369492 | -0.32722 | 0.879964 | 0.094446 | Diabetes type 1 with peripheral circulatory disc | endocrine/metab | 0.432395 |
| 634.3 | 0.143345 | 0.160395 | 0.893704 | 0.37148 | -0.17102 | 0.457713 | 0.221549 | Ectopic pregnancy | pregnancy compl | 0.430065 |
| 253.3 | 0.089891 | 0.100636 | 0.89323 | 0.371734 | -0.10735 | 0.287135 | 0.821287 | Diabetes insipidus | endocrine/metab | 0.429768 |
| 756.2 | -0.08625 | 0.096847 | -0.89062 | 0.373133 | -0.27607 | 0.103563 | 0.319622 | Pectus and other congenital anomalies of ribs/ | congenital anoma | 0.428136 |
| 790.6 | 0.013292 | 0.014939 | 0.88974 | 0.373605 | -0.01599 | 0.042572 | 0.400309 | Other abnormal blood chemistry | symptoms | 0.427587 |
| 429.3 | 0.027714 | 0.031156 | 0.889543 | 0.373711 | -0.03335 | 0.088778 | 0.124781 | Symptoms involving cardiovascular system | circulatory syster | 0.427464 |
| 174.2 | -0.36654 | 0.412061 | -0.88953 | 0.373719 | -1.17417 | 0.441085 | 0.008288 | Breast cancer [male] | neoplasms | 0.427455 |
| 306 | 0.012345 | 0.013886 | 0.889014 | 0.373996 | -0.01487 | 0.039561 | 0.911064 | Other mental disorder | mental disorders | 0.427134 |
| 117.1 | 0.22078 | 0.248485 | 0.888504 | 0.37427 | -0.26624 | 0.707802 | 0.38415 | Histoplasmosis | infectious diseas | 0.426816 |
| 578 | -0.01665 | 0.018736 | -0.88846 | 0.374296 | -0.05337 | 0.020076 | 0.255999 | Gastrointestinal hemorrhage | digestive | 0.426785 |
| 261.1 | 0.127176 | 0.143225 | 0.887946 | 0.37457 | -0.15354 | 0.407893 | 0.682535 | Vitamin A deficiency | endocrine/metab | 0.426467 |
| 70.2 | 0.054238 | 0.061199 | 8.86E-01 | 0.375483 | -0.06571 | 0.174187 | 0.261129 | Viral hepatitis B | infectious diseas | 0.42541 |
| 627.21 | -0.1128 | 0.127322 | -0.88591 | 0.375669 | -0.36234 | 0.136752 | 0.882727 | Symptomatic artificial menopause | genitourinary | 0.425195 |
| 817 | 0.042647 | 0.048149 | 0.885715 | 0.375771 | -0.05172 | 0.137018 | 0.674009 | Concussion | injuries & poisoni | 0.425076 |
| 741 | 0.024907 | 0.028138 | 0.885194 | 0.376052 | -0.03024 | 0.080056 | 0.135935 | Symptoms and disorders of the joints | musculoskeletal | 0.424752 |
| 627.3 | -0.02614 | 0.029618 | -0.88242 | 0.377549 | -0.08418 | 0.031914 | 0.727828 | Postmenopausal atrophic vaginitis | genitourinary | 0.423026 |
| 702.4 | 0.150631 | 0.170907 | 0.881362 | 0.378122 | -0.18434 | 0.485602 | 0.287102 | Degenerative skin disorders | dermatologic | 0.422368 |
| 276.12 | 0.018214 | 0.02071 | 0.879469 | 0.379147 | -0.02238 | 0.058805 | 0.533021 | Hyposmolality and/or hyponatremia | endocrine/metab | 0.421192 |
| 727.2 | 0.062124 | 0.070716 | 0.878502 | 0.379672 | -0.07648 | 0.200725 | 0.709623 | Bursitis disorders | musculoskeletal | 0.420592 |
| 634 | -0.03567 | 0.040773 | -0.87479 | 0.381688 | -0.11558 | 0.044246 | 0.715647 | Miscarriage; stillbirth | pregnancy compl | 0.418291 |
| 624.9 | 0.029379 | 0.033611 | 0.874106 | 0.382061 | -0.0365 | 0.095255 | 0.8946 | stress incontinence, female | genitourinary | 0.417868 |
| 290.11 | 0.084526 | 0.097562 | 0.866383 | 0.38628 | -0.10669 | 0.275745 | 0.229914 | Alzheimer's disease | mental disorders | 0.413097 |
| 289.8 | 0.041545 | 0.048114 | 0.863476 | 0.387876 | -0.05276 | 0.135846 | 0.766025 | Polycythemia, secondary | hematopoietic | 0.411307 |
| 1015 | 0.030937 | 0.035902 | 0.861707 | 0.388849 | -0.03943 | 0.101302 | 0.140389 | Effects of other external causes | other | 0.410219 |
| 599.6 | 0.045843 | 0.053326 | 0.859669 | 0.389972 | -0.05867 | 0.150359 | 0.820331 | Oliguria and anuria | genitourinary | 0.408967 |
| 613.9 | -0.0616 | 0.071726 | -0.85886 | 0.390416 | -0.20218 | 0.078978 | 0.498568 | Breast disorder NOS | genitourinary | 0.408472 |
| 165.1 | -0.07122 | 0.083081 | -0.85719 | 0.39134 | -0.23405 | 0.09162 | 0.024001 | Cancer of bronchus; lung | neoplasms | 0.407446 |
| 31.1 | 0.87507 | 1.021594 | 8.57E-01 | 0.391681 | -1.12722 | 2.877357 | 0.209436 | Leprosy | infectious diseas | 0.407068 |
| 446.7 | 0.223886 | 0.261677 | 0.855581 | 0.392229 | -0.28899 | 0.736764 | 0.699126 | Takayasu's disease | circulatory syster | 0.40646 |
| 473.4 | 0.021432 | 0.025071 | 0.854845 | 0.392637 | -0.02771 | 0.07057 | 0.910443 | Voice disturbance | respiratory | 0.406009 |
| 527.8 | -0.05365 | 0.062814 | -0.85409 | 0.393057 | -0.17676 | 0.069464 | 0.80303 | Other specified diseases of the salivary glands | digestive | 0.405544 |
| 291.1 | -0.1186 | 0.139104 | -0.8526 | 0.393879 | -0.39124 | 0.154038 | 0.306012 | Transient mental disorders due to conditions c | mental disorders | 0.404637 |
| 241.2 | -0.0267 | 0.031359 | -0.85152 | 0.394483 | -0.08817 | 0.03476 | 0.352723 | Nontoxic multinodular goiter | endocrine/metab | 0.403972 |
| 592.2 | -0.07372 | 0.086804 | -0.84929 | 0.395718 | -0.24385 | 0.09641 | 0.186494 | Urethritis and urethral syndrome | genitourinary | 0.402615 |
| 569.1 | 0.043063 | 0.050752 | 0.848512 | 0.396153 | -0.05641 | 0.142535 | 0.293694 | Toxic gastroenteritis and colitis | digestive | 0.402137 |
| 627.1 | 0.03368 | 0.039707 | 0.848208 | 0.396322 | -0.04414 | 0.111504 | 0.941577 | Postmenopausal bleeding | genitourinary | 0.401952 |
| 963 | -0.02189 | 0.02582 | -0.84786 | 0.396514 | -0.0725 | 0.028715 | 0.889474 | Poisoning by primarily systemic agents | injuries & poisoni | 0.401741 |
| 614.32 | 0.089483 | 0.105718 | 0.846433 | 0.397311 | -0.11772 | 0.296687 | 0.980252 | Chronic inflammatory pelvic disease | genitourinary | 0.400869 |
| 433.5 | 0.046347 | 0.054802 | 0.845724 | 0.397707 | -0.06106 | 0.153756 | 0.454072 | Cerebral aneurysm | circulatory syster | 0.400437 |
| 733.9 | 0.064633 | 0.076555 | 0.844262 | 0.398523 | -0.08541 | 0.214678 | 0.178214 | Chondromalacia | musculoskeletal | 0.399547 |
| 348.4 | 0.107954 | 0.127899 | 0.844059 | 0.398637 | -0.14272 | 0.358632 | 0.288241 | Cerebral cysts | neurological | 0.399423 |
| 447.7 | 0.033742 | 0.040047 | 0.842553 | 0.399479 | -0.04475 | 0.112232 | 0.731065 | Aortic ectasia | circulatory syster | 0.398506 |
| 194 | 0.128869 | 0.153122 | 0.841606 | 0.400008 | -0.17125 | 0.428982 | 0.229945 | Cancer of other endocrine glands | neoplasms | 0.397931 |
| 415.2 | 0.038065 | 0.045387 | 0.838676 | 0.401651 | -0.05089 | 0.127022 | 0.106745 | Chronic pulmonary heart disease | circulatory syster | 0.396151 |
| 792.1 | -0.03161 | 0.037722 | -0.83786 | 0.402108 | -0.10554 | 0.042328 | 0.83415 | Papanicolaou smear of cervix or vagina with a | genitourinary | 0.395657 |
| 117 | 0.032363 | 0.038651 | 0.837312 | 0.402417 | -0.04339 | 0.108116 | 0.70891 | Mycoses | infectious diseas | 0.395324 |
| 198.7 | 0.214312 | 0.256278 | 0.836249 | 0.403015 | -0.28798 | 0.716607 | 0.000724 | Secondary malignant neoplasm of skin | neoplasms | 0.394679 |

| phecode | beta | se | zval | pval | ci.lb | ci.ub | QEp | phenotype | category | minuslogp |
|---|---|---|---|---|---|---|---|---|---|---|
| 368 | -0.02306 | 0.027643 | -0.83423 | 0.404153 | -0.07724 | 0.031119 | 0.186753 | Visual disturbances | sense organs | 0.393455 |
| 250.4 | 0.011748 | 0.014094 | 0.833519 | 0.404552 | -0.01588 | 0.039371 | 0.420275 | Abnormal glucose | endocrine/metab | 0.393025 |
| 284.1 | 0.023892 | 0.028844 | 0.828302 | 0.4075 | -0.03264 | 0.080426 | 0.486096 | Pancytopenia | hematopoietic | 0.389873 |
| 509.2 | 0.072923 | 0.088066 | 0.828057 | 0.407639 | -0.09968 | 0.245529 | 0.061774 | Respiratory insufficiency | respiratory | 0.389725 |
| 164 | 0.155707 | 0.188245 | 0.827149 | 0.408153 | -0.21325 | 0.524661 | 0.15004 | Cancer of intrathoracic organs | neoplasms | 0.389177 |
| 599.5 | -0.02796 | 0.033814 | -0.82685 | 0.408323 | -0.09423 | 0.038315 | 0.077929 | Frequency of urination and polyuria | genitourinary | 0.388996 |
| 300.13 | 0.041383 | 0.050255 | 0.823461 | 0.410246 | -0.05712 | 0.139881 | 0.888672 | Phobia | mental disorders | 0.386956 |
| 942 | -0.05058 | 0.061439 | -0.82328 | 0.410349 | -0.171 | 0.069837 | 0.63386 | Infusion and transfusion reaction | injuries & poisoni | 0.386847 |
| 204.1 | -0.05823 | 0.070833 | -0.82209 | 0.411026 | -0.19706 | 0.080599 | 0.877304 | Lymphoid leukemia | neoplasms | 0.38613 |
| 459 | 0.014403 | 0.017546 | 0.820838 | 0.411738 | -0.01999 | 0.048793 | 0.436001 | Other disorders of circulatory system | circulatory syster | 0.385379 |
| 721.2 | 0.127702 | 0.155602 | 0.820696 | 0.411819 | -0.17727 | 0.432676 | 0.005279 | Spondylosis with myelopathy | musculoskeletal | 0.385293 |
| 627.4 | -0.13028 | 0.15902 | -0.81927 | 0.41263 | -0.44195 | 0.181392 | 0.031068 | Premenopausal menorrhagia | genitourinary | 0.384439 |
| 359.1 | -0.22006 | 0.268695 | -0.81898 | 0.412795 | -0.74669 | 0.306575 | 0.023443 | Muscular dystrophies | neurological | 0.384266 |
| 1008 | 0.053785 | 0.065707 | 0.81857 | 0.413032 | -0.075 | 0.182568 | 0.453866 | Crushing or internal injury to organs | other | 0.384017 |
| 800.4 | 0.065592 | 0.080218 | 0.817668 | 0.413547 | -0.09163 | 0.222816 | 0.279802 | Fracture of patella | injuries & poisoni | 0.383476 |
| 767 | 0.097449 | 0.119295 | 0.816871 | 0.414002 | -0.13637 | 0.331263 | 0.655006 | Cervicocranial/Cervicobrachial syndrome | symptoms | 0.382998 |
| 836 | 0.085361 | 0.104672 | 0.815507 | 0.414782 | -0.11979 | 0.290513 | 0.672475 | Traumatic arthropathy | injuries & poisoni | 0.38218 |
| 275.5 | 0.028527 | 0.035026 | 0.814447 | 0.415389 | -0.04012 | 0.097176 | 0.160339 | Disorders of calcium/phosphorus metabolism | endocrine/metab | 0.381545 |
| 38.3 | 0.027153 | 0.033438 | 8.12E-01 | 0.416772 | -0.03839 | 0.092691 | 0.700344 | Bacteremia | infectious diseas | 0.380101 |
| 301 | 0.064055 | 0.078928 | 0.81156 | 0.417044 | -0.09064 | 0.21875 | 0.710497 | Personality disorders | mental disorders | 0.379818 |
| 740.2 | 0.023607 | 0.02913 | 0.810411 | 0.417704 | -0.03349 | 0.080701 | 0.679199 | Osteoarthrosis, generalized | musculoskeletal | 0.379131 |
| 840.2 | 0.029245 | 0.036133 | 0.809357 | 0.41831 | -0.04157 | 0.100064 | 0.952637 | Rotator cuff (capsule) sprain | injuries & poisoni | 0.378502 |
| 598.4 | -0.12262 | 0.151567 | -0.80901 | 0.41851 | -0.41968 | 0.174446 | 0.130666 | Other cells and casts in urine | genitourinary | 0.378295 |
| 586.2 | 0.020342 | 0.02517 | 0.808196 | 0.418978 | -0.02899 | 0.069675 | 0.68899 | Cyst of kidney, acquired | genitourinary | 0.377809 |
| 377 | -0.03549 | 0.043933 | -0.80784 | 0.419181 | -0.1216 | 0.050616 | 0.96239 | Disorders of optic nerve and visual pathways | sense organs | 0.377598 |
| 480.12 | 0.084766 | 0.104939 | 0.80777 | 0.419223 | -0.12091 | 0.290442 | 0.351418 | Pseudomonal pneumonia | respiratory | 0.377555 |
| 480.11 | 0.031558 | 0.039093 | 0.807258 | 0.419518 | -0.04506 | 0.108178 | 0.863929 | Pneumococcal pneumonia | respiratory | 0.377249 |
| 618.5 | 0.220333 | 0.273589 | 0.805342 | 0.420623 | -0.31589 | 0.756558 | 0.021634 | Prolapse of vaginal vault after hysterectomy | genitourinary | 0.376107 |
| 782.6 | -0.05387 | 0.067108 | -0.8028 | 0.422093 | -0.1854 | 0.077656 | 0.086984 | Pallor and flushing | symptoms | 0.374592 |
| 110.12 | -0.05315 | 0.06623 | -0.80248 | 0.422277 | -0.18296 | 0.07666 | 0.050387 | Althete's foot | infectious diseas | 0.374402 |
| 348.2 | -0.0324 | 0.040379 | -0.80246 | 0.422288 | -0.11154 | 0.046739 | 0.901522 | Cerebral edema and compression of brain | neurological | 0.374391 |
| 300.8 | -0.05148 | 0.064165 | -0.80228 | 0.42239 | -0.17724 | 0.074282 | 0.989431 | Acute reaction to stress | mental disorders | 0.374286 |
| 795.8 | 0.058157 | 0.072532 | 0.801818 | 0.422658 | -0.084 | 0.200317 | 0.256829 | Abnormal tumor markers | symptoms | 0.374011 |
| 214.1 | 0.030292 | 0.037806 | 0.801254 | 0.422984 | -0.04381 | 0.10439 | 0.932772 | Lipoma of skin and subcutaneous tissue | neoplasms | 0.373676 |
| 499 | 0.121456 | 0.151639 | 0.800957 | 0.423156 | -0.17575 | 0.418663 | 0.921428 | Cystic fibrosis | respiratory | 0.373499 |
| 208 | 0.021177 | 0.026462 | 0.800294 | 0.423541 | -0.03069 | 0.073042 | 0.181363 | Benign neoplasm of colon | neoplasms | 0.373105 |
| 199 | -0.01998 | 0.024973 | -0.79995 | 0.423742 | -0.06892 | 0.028969 | 0.330871 | Neoplasm of uncertain behavior | neoplasms | 0.372898 |
| 751.22 | -0.06306 | 7.92E-02 | -0.79609 | 0.425982 | -0.21831 | 0.092191 | 0.488304 | Other specified congenital anomalies of kidney | congenital anoma | 0.370608 |
| 736 | 0.033401 | 0.042052 | 0.794269 | 0.427039 | -0.04902 | 0.115821 | 0.2845 | Other acquired deformities of limbs | musculoskeletal | 0.369532 |
| 976 | 0.199876 | 0.251648 | 0.794268 | 0.427039 | -0.29335 | 0.693097 | 0.433497 | Poisoning by agents primarily affecting skin & | injuries & poisoni | 0.369532 |
| 290 | 0.029986 | 0.038126 | 0.786487 | 0.431582 | -0.04474 | 0.104711 | 0.482991 | Delirium dementia and amnestic and other cog | mental disorders | 0.364936 |
| 430.2 | -0.04601 | 0.058589 | -0.78532 | 0.432265 | -0.16084 | 0.068821 | 0.590026 | Intracerebral hemorrhage | circulatory syster | 0.36425 |
| 523.32 | 0.344961 | 0.439387 | 0.785095 | 0.432398 | -0.51622 | 1.206144 | 0.669101 | Chronic periodontitis | digestive | 0.364117 |
| 165 | -0.06004 | 0.076537 | -0.78446 | 0.432769 | -0.21005 | 0.089969 | 0.043913 | Cancer within the respiratory system | neoplasms | 0.363744 |
| 561 | 0.010849 | 0.013834 | 0.784245 | 0.432896 | -0.01626 | 0.037962 | 0.580692 | Symptoms involving digestive system | digestive | 0.363616 |
| 564.9 | 0.020974 | 0.026752 | 0.784036 | 0.433019 | -0.03146 | 0.073407 | 0.246907 | Personal history of diseases of digestive syste | digestive | 0.363493 |
| 345.12 | 0.0447 | 0.05707 | 0.783256 | 0.433477 | -0.06715 | 0.156556 | 0.201161 | Partial epilepsy | neurological | 0.363034 |
| 426.4 | -0.24086 | 0.308295 | -0.78125 | 0.434653 | -0.8451 | 0.363391 | 0.056848 | Anomalous atrioventricular excitation | circulatory syster | 0.361857 |

| phecode | beta | se | zval | pval | ci.lb | ci.ub | QEp | phenotype | category | minuslogp |
|---|---|---|---|---|---|---|---|---|---|---|
| 597 | -0.03697 | 0.047688 | -0.77517 | 0.438238 | -0.13043 | 0.0565 | 0.86783 | Other disorders of urethra and urinary tract | genitourinary | 0.35829 |
| 189.1 | 0.048232 | 0.062413 | 0.772793 | 0.439645 | -0.07409 | 0.170558 | 0.337956 | Cancer of kidney and renal pelvis | neoplasms | 0.356898 |
| 709 | 0.031074 | 0.040273 | 0.771592 | 0.440356 | -0.04786 | 0.110007 | 0.267113 | Diffuse diseases of connective tissue | dermatologic | 0.356196 |
| 727.7 | 0.075278 | 0.097734 | 0.77023 | 0.441164 | -0.11628 | 0.266834 | 0.432687 | Contracture of tendon (sheath) | musculoskeletal | 0.3554 |
| 597.2 | -0.04108 | 0.053339 | -0.77022 | 0.441167 | -0.14563 | 0.06346 | 0.608061 | Urinary complications NEC | genitourinary | 0.355397 |
| 292.12 | -0.07263 | 0.094323 | -0.77002 | 0.44129 | -0.2575 | 0.11224 | 0.288095 | Symbolic dysfunction | mental disorders | 0.355275 |
| 278.3 | 0.075344 | 0.097874 | 0.769809 | 0.441413 | -0.11648 | 0.267173 | 0.29867 | Localized adiposity | endocrine/metab | 0.355154 |
| 963.1 | -0.02016 | 0.026209 | -0.76928 | 0.441725 | -0.07153 | 0.031207 | 0.897333 | Antineoplastic and immunosuppressive drugs | injuries & poisoni | 0.354848 |
| 560 | 0.018404 | 0.024078 | 0.764373 | 0.444645 | -0.02879 | 0.065596 | 0.57451 | Intestinal obstruction without mention of hernia | digestive | 0.351987 |
| 748 | 0.123447 | 0.161648 | 0.763679 | 0.445059 | -0.19338 | 0.440272 | 0.139646 | Anomalies of respiratory system, congenital | congenital anoma | 0.351583 |
| 495.11 | 0.147112 | 0.193169 | 0.761572 | 0.446316 | -0.23149 | 0.525717 | 0.155414 | Chronic obstructive asthma with exacerbation | respiratory | 0.350358 |
| 244.3 | -0.43222 | 0.56755 | -0.76155 | 0.446331 | -1.54459 | 0.680162 | 0.179838 | Iodine hypothyroidism | endocrine/metab | 0.350343 |
| 227.3 | -0.04763 | 0.062717 | -0.75946 | 0.447578 | -0.17055 | 0.075292 | 0.664934 | Benign neoplasm of pituitary gland and craniop | neoplasms | 0.349132 |
| 368.7 | -0.61971 | 0.817014 | -0.75851 | 0.448147 | -2.22103 | 0.981607 | 0.102927 | Disorders of accommodation | sense organs | 0.348579 |
| 535.8 | 0.029607 | 0.039035 | 0.758475 | 0.448167 | -0.0469 | 0.106114 | 0.491994 | Other specified gastritis | digestive | 0.34856 |
| 579 | 0.016156 | 0.021336 | 0.757223 | 0.448916 | -0.02566 | 0.057974 | 0.498899 | Other symptoms involving abdomen and pelvis | digestive | 0.347835 |
| 722.9 | 0.036092 | 0.047698 | 0.756691 | 0.449235 | -0.05739 | 0.129578 | 0.331407 | Other and unspecified disc disorder | musculoskeletal | 0.347526 |
| 381.1 | 0.055384 | 0.073454 | 0.753988 | 0.450857 | -0.08858 | 0.199352 | 0.013554 | Otitis media | sense organs | 0.345962 |
| 580.2 | 0.03634 | 0.04822 | 0.753635 | 0.451068 | -0.05817 | 0.130849 | 0.672232 | Nephrotic syndrome without mention of glome | genitourinary | 0.345758 |
| 198.6 | -0.04875 | 0.0647 | -0.75349 | 0.451155 | -0.17556 | 0.078058 | 0.124592 | Secondary malignancy of bone | neoplasms | 0.345675 |
| 297 | 0.038659 | 0.051318 | 0.753333 | 0.45125 | -0.06192 | 0.13924 | 0.883367 | Suicidal ideation or attempt | mental disorders | 0.345583 |
| 350.3 | -0.038 | 0.050474 | -0.75281 | 0.451562 | -0.13693 | 0.06093 | 0.604927 | Lack of coordination | neurological | 0.345283 |
| 792 | -0.02387 | 0.031744 | -0.75198 | 0.452066 | -0.08609 | 0.038347 | 0.80091 | Abnormal Papanicolaou smear of cervix and c | genitourinary | 0.344798 |
| 275.3 | 0.02135 | 0.028407 | 0.751569 | 0.45231 | -0.03433 | 0.077027 | 0.939045 | Disorders of magnesium metabolism | endocrine/metab | 0.344564 |
| 263 | -0.02119 | 0.028215 | -0.75103 | 0.452636 | -0.07649 | 0.034111 | 0.874467 | Other nutritional deficiency | endocrine/metab | 0.344251 |
| 732.7 | -0.17947 | 0.239296 | -7.50E-01 | 0.453248 | -0.64849 | 0.289537 | 0.901938 | Osteochondritis dissecans | musculoskeletal | 0.343664 |
| 750.1 | -0.03287 | 0.043863 | -0.74942 | 0.453605 | -0.11884 | 0.053098 | 0.828093 | Upper gastrointestinal congenital anomalies | congenital anoma | 0.343322 |
| 610.8 | -0.03673 | 0.049021 | -0.74922 | 0.453724 | -0.13281 | 0.059352 | 0.7591 | Other specified benign mammary dysplasias | genitourinary | 0.343208 |
| 772.4 | 0.069132 | 0.0924 | 0.748185 | 0.454348 | -0.11197 | 0.250233 | 0.356227 | Rhabdomyolysis | symptoms | 0.342611 |
| 359.2 | 0.031368 | 0.042033 | 0.746259 | 0.455511 | -0.05102 | 0.113751 | 0.622326 | Myopathy | neurological | 0.341501 |
| 195 | -0.02175 | 0.029162 | -0.74592 | 0.455714 | -0.07891 | 0.035404 | 0.16709 | Cancer, suspected or other | neoplasms | 0.341308 |
| 153.2 | 0.038558 | 0.051924 | 0.742588 | 0.457731 | -0.06321 | 0.140327 | 0.313473 | Colon cancer | neoplasms | 0.33939 |
| 757 | 0.131808 | 0.177638 | 0.742006 | 0.458084 | -0.21636 | 0.479972 | 0.07724 | Congenital anomalies of the integument | congenital anoma | 0.339055 |
| 750 | -0.0256 | 0.034585 | -0.74017 | 0.459195 | -0.09338 | 0.042187 | 0.544421 | Digestive congenital anomalies | congenital anoma | 0.338003 |
| 180.1 | 0.052464 | 0.071068 | 0.738217 | 0.460383 | -0.08683 | 0.191754 | 0.557313 | Cervical cancer | neoplasms | 0.336881 |
| 773 | 0.016585 | 0.022513 | 0.736685 | 0.461314 | -0.02754 | 0.060709 | 0.137673 | Pain in limb | symptoms | 0.336003 |
| 741.2 | 0.049506 | 0.06735 | 0.735059 | 0.462303 | -0.0825 | 0.18151 | 0.095622 | Stiffness of joint | musculoskeletal | 0.335073 |
| 276.42 | -0.0359 | 0.048917 | -0.73386 | 0.463034 | -0.13177 | 0.059978 | 0.488682 | Alkalosis | endocrine/metab | 0.334387 |
| 174.1 | 0.020258 | 0.027664 | 0.732294 | 0.463989 | -0.03396 | 0.074478 | 0.960702 | Breast cancer [female] | neoplasms | 0.333492 |
| 204.4 | 0.18338 | 0.250602 | 0.731757 | 0.464317 | -0.30779 | 0.674552 | 0.00051 | Multiple myeloma | neoplasms | 0.333186 |
| 8.7 | 0.105968 | 0.145134 | 7.30E-01 | 0.465303 | -0.17849 | 0.390426 | 0.794967 | Intestinal infection due to protozoa | infectious diseas | 0.332264 |
| 620 | -0.05566 | 0.076253 | -0.72996 | 0.465415 | -0.20511 | 0.093792 | 0.905534 | Dysplasia of female genital organs | genitourinary | 0.332159 |
| 531.5 | -0.3371 | 0.4619 | -0.72981 | 0.465507 | -1.24241 | 0.568208 | 0.001773 | Gastrojejunal ulcer | digestive | 0.332074 |
| 333.3 | 0.17744 | 0.243294 | 0.729321 | 0.465806 | -0.29941 | 0.654288 | 0.578139 | Tics and choreas | neurological | 0.331795 |
| 564.1 | -0.02667 | 0.036589 | -0.72898 | 0.466014 | -0.09839 | 0.04504 | 0.386599 | Irritable Bowel Syndrome | digestive | 0.331601 |
| 345 | 0.021087 | 0.028935 | 0.728766 | 0.466145 | -0.03563 | 0.077799 | 0.574978 | Epilepsy, recurrent seizures, convulsions | neurological | 0.331479 |
| 713.5 | 0.102005 | 0.140341 | 0.726837 | 0.467326 | -0.17306 | 0.377068 | 0.901489 | Arthropathy associated with neurological disor | musculoskeletal | 0.33038 |
| 704 | -0.01538 | 0.021248 | -0.72391 | 0.469123 | -0.05703 | 0.026263 | 0.609526 | Diseases of hair and hair follicles | dermatologic | 0.328713 |

| phecode | beta | se | zval | pval | ci.lb | ci.ub | QEp | phenotype | category | minuslogp |
|---|---|---|---|---|---|---|---|---|---|---|
| 288.1 | -0.01393 | 0.019296 | -0.72197 | 0.470312 | -0.05175 | 0.023888 | 0.635129 | Decreased white blood cell count | hematopoietic | 0.327614 |
| 819 | -0.02839 | 0.039358 | -0.72135 | 0.470695 | -0.10553 | 0.048749 | 0.995743 | Skull and face fracture and other intercranial ir | injuries & poisoni | 0.32726 |
| 202.23 | 0.195389 | 0.27102 | 0.72094 | 0.470946 | -0.3358 | 0.726578 | 0.15547 | Lymphosarcoma | neoplasms | 0.327029 |
| 939.1 | 0.069693 | 0.096747 | 0.720364 | 0.471301 | -0.11993 | 0.259314 | 0.566495 | Contact and allergic dermatitis of eyelid | dermatologic | 0.326702 |
| 427.8 | 0.025075 | 0.034844 | 0.719644 | 0.471744 | -0.04322 | 0.093368 | 0.519848 | Sinoatrial node dysfunction (Bradycardia) | circulatory syster | 0.326294 |
| 751.3 | 0.105553 | 0.146799 | 0.719031 | 0.472122 | -0.18217 | 0.393274 | 0.286492 | Obstructive genitourinary defect | congenital anoma | 0.325946 |
| 230 | 0.336301 | 0.467979 | 0.718623 | 0.472373 | -0.58092 | 1.253523 | 0.064704 | Kaposi's sarcoma | neoplasms | 0.325715 |
| 361.1 | -0.07979 | 0.111069 | -0.71841 | 0.472502 | -0.29748 | 0.137897 | 0.128256 | Retinal detachment with retinal defect | sense organs | 0.325597 |
| 513.4 | 0.161917 | 0.225411 | 0.718318 | 0.472561 | -0.27988 | 0.603715 | 0.007065 | Hyperventilation | respiratory | 0.325542 |
| 218 | 0.018562 | 0.025973 | 0.714649 | 0.474826 | -0.03234 | 0.069468 | 0.512381 | Benign neoplasm of uterus | neoplasms | 0.323465 |
| 561.1 | -0.01131 | 0.015844 | -0.71373 | 0.475396 | -0.04236 | 0.019745 | 0.397028 | Diarrhea | digestive | 0.322944 |
| 601.1 | -0.03477 | 0.048742 | -0.71335 | 0.475627 | -0.1303 | 0.060762 | 0.613061 | Prostatitis | genitourinary | 0.322733 |
| 626.15 | -0.10675 | 0.149666 | -0.71328 | 0.475675 | -0.40009 | 0.186587 | 0.113637 | Infertility, female, associated with anovulation | genitourinary | 0.322689 |
| 333.2 | -0.05741 | 0.080633 | -0.71203 | 0.476448 | -0.21545 | 0.100625 | 0.968379 | Myoclonus | neurological | 0.321985 |
| 353.2 | 0.093242 | 0.131372 | 0.709757 | 0.477855 | -0.16424 | 0.350726 | 0.679428 | Nerve root lesions | neurological | 0.320704 |
| 931 | -0.24409 | 0.344923 | -0.70767 | 0.479151 | -0.92013 | 0.431945 | 0.178944 | Contact dermatitis and other eczema due to pl | dermatologic | 0.319528 |
| 213 | 0.047206 | 0.066807 | 0.706604 | 0.479812 | -0.08373 | 0.178144 | 0.501832 | Benign neoplasm of bone and articular cartilag | neoplasms | 0.318928 |
| 514.2 | 0.029096 | 0.04118 | 0.706572 | 0.479832 | -0.05161 | 0.109807 | 0.031371 | Solitary pulmonary nodule | respiratory | 0.318911 |
| 355 | 0.040121 | 0.056885 | 0.705299 | 0.480624 | -0.07137 | 0.151613 | 0.534946 | Complex regional/central pain syndrome | neurological | 0.318194 |
| 242.2 | 0.093666 | 0.132818 | 0.705216 | 0.480676 | -0.16665 | 0.353984 | 0.473416 | Toxic multinodular goiter | endocrine/metab | 0.318148 |
| 426.21 | 0.033554 | 0.04781 | 0.701817 | 0.482793 | -0.06015 | 0.12726 | 0.799434 | First degree AV block | circulatory syster | 0.316239 |
| 189.2 | 0.034794 | 0.049622 | 0.701167 | 0.483199 | -0.06246 | 0.132051 | 0.359606 | Cancer of bladder | neoplasms | 0.315874 |
| 185 | -0.02172 | 0.031124 | -0.69771 | 0.48536 | -0.08272 | 0.039286 | 0.725541 | Cancer of prostate | neoplasms | 0.313936 |
| 783.1 | -0.04441 | 0.063709 | -0.69702 | 0.485789 | -0.16927 | 0.08046 | 0.55761 | Postprocedural fever | symptoms | 0.313552 |
| 759.1 | 0.138843 | 1.99E-01 | 0.696775 | 0.485944 | -0.25171 | 0.529395 | 0.358109 | Anomalies of endocrine glands, congenital | congenital anoma | 0.313414 |
| 577 | 0.017706 | 0.025419 | 0.696589 | 0.48606 | -0.03211 | 0.067526 | 0.924236 | Diseases of pancreas | digestive | 0.31331 |
| 270.3 | 0.023888 | 0.034443 | 0.693559 | 0.487959 | -0.04362 | 0.091395 | 0.795309 | Disorders of plasma protein metabolism | endocrine/metab | 0.311617 |
| 690 | -0.01809 | 0.026105 | -0.69308 | 0.488258 | -0.06926 | 0.033071 | 0.741333 | Erythematosquamous dermatosis | dermatologic | 0.311351 |
| 375.2 | 0.042186 | 0.060871 | 0.693042 | 0.488283 | -0.07712 | 0.161492 | 0.449297 | Epiphora | sense organs | 0.311328 |
| 735.2 | -0.03996 | 0.057904 | -0.69012 | 0.490118 | -0.15345 | 0.073528 | 0.160773 | Acquired toe deformities | musculoskeletal | 0.309699 |
| 174 | -0.01881 | 0.027363 | -0.68755 | 0.491737 | -0.07244 | 0.034817 | 0.940022 | Breast cancer | neoplasms | 0.308267 |
| 218.2 | 0.115036 | 0.167706 | 0.685939 | 0.492751 | -0.21366 | 0.443735 | 0.326546 | Other benign neoplasm of uterus | neoplasms | 0.307372 |
| 627 | -0.02319 | 0.033882 | -0.68453 | 0.493642 | -0.0896 | 0.043215 | 0.076159 | Menopausal and postmenopausal disorders | genitourinary | 0.306588 |
| 8.52 | 0.02967 | 0.043523 | 6.82E-01 | 0.49542 | -0.05563 | 0.114974 | 0.458211 | Intestinal infection due to C. difficile | infectious diseas | 0.305026 |
| 90.2 | -0.08251 | 0.121366 | -0.67983 | 0.496612 | -0.32038 | 0.155365 | 0.617205 | Gonococcal infections | infectious diseas | 0.303982 |
| 395.6 | 0.024522 | 0.036088 | 0.679497 | 0.496823 | -0.04621 | 0.095254 | 0.515937 | Heart valve replaced | circulatory syster | 0.303798 |
| 556.1 | 0.033443 | 0.049287 | 0.678525 | 0.497439 | -0.06316 | 0.130044 | 0.464466 | Ulceration of intestine | digestive | 0.30326 |
| 560.1 | 0.023417 | 0.034602 | 0.676747 | 0.498567 | -0.0444 | 0.091236 | 0.253871 | Paralytic ileus | digestive | 0.302277 |
| 752.2 | -0.04658 | 0.068953 | -0.67552 | 0.499343 | -0.18172 | 0.088566 | 0.612736 | Other specified congenital anomalies of nervou | congenital anoma | 0.301601 |
| 622.1 | -0.0304 | 0.045052 | -0.67486 | 0.499767 | -0.1187 | 0.057896 | 0.502198 | Polyp of corpus uteri | genitourinary | 0.301233 |
| 70.4 | -0.10548 | 0.156469 | -6.74E-01 | 0.500242 | -0.41215 | 0.201197 | 0.028857 | Chronic hepatitis | infectious diseas | 0.30082 |
| 536.8 | -0.02341 | 0.034777 | -0.67301 | 0.500943 | -0.09157 | 0.044756 | 0.2938 | Dyspepsia and other specified disorders of fur | digestive | 0.300211 |
| 696.3 | 0.072633 | 0.10803 | 0.672336 | 0.50137 | -0.1391 | 0.284368 | 0.658958 | Pityriasis | dermatologic | 0.299842 |
| 656.8 | 0.420888 | 0.630089 | 0.667982 | 0.504145 | -0.81406 | 1.655841 | 0.870375 | Perinatal jaundice | pregnancy compl | 0.297444 |
| 269 | 0.024979 | 0.037458 | 0.666859 | 0.504862 | -0.04844 | 0.098395 | 0.212115 | Proteinuria | endocrine/metab | 0.296827 |
| 256 | 0.032979 | 0.049465 | 0.666715 | 0.504954 | -0.06397 | 0.129929 | 0.659891 | Ovarian dysfunction | endocrine/metab | 0.296748 |
| 151 | 0.04626 | 0.069405 | 0.666519 | 0.505079 | -0.08977 | 0.18229 | 0.676065 | Cancer of stomach | neoplasms | 0.29664 |
| 550.2 | 0.013306 | 0.020028 | 0.664374 | 0.506451 | -0.02595 | 0.05256 | 0.448673 | Diaphragmatic hernia | digestive | 0.295463 |

| phecode | beta | se | zval | pval | ci.lb | ci.ub | QEp | phenotype | category | minuslogp |
|---|---|---|---|---|---|---|---|---|---|---|
| 573.5 | -0.0238 | 0.035827 | -0.66429 | 0.506505 | -0.09402 | 0.046421 | 0.749376 | Jaundice (not of newborn) | digestive | 0.295417 |
| 510.2 | 0.063897 | 0.096266 | 0.663753 | 0.506848 | -0.12478 | 0.252576 | 0.075378 | Lung transplant | respiratory | 0.295122 |
| 737 | -0.01822 | 0.027515 | -0.66223 | 0.507824 | -0.07215 | 0.035708 | 0.79206 | Curvature of spine | musculoskeletal | 0.294286 |
| 427 | 0.009095 | 0.01374 | 0.661944 | 0.508007 | -0.01783 | 0.036025 | 0.686553 | Cardiac dysrhythmias | circulatory syster | 0.29413 |
| 580.4 | 0.037394 | 0.056586 | 0.660832 | 0.50872 | -0.07351 | 0.148301 | 0.642695 | Renal sclerosis, NOS | genitourinary | 0.293521 |
| 244.1 | -0.02109 | 0.031922 | -0.66071 | 0.508796 | -0.08366 | 0.041474 | 0.396482 | Secondary hypothyroidism | endocrine/metab | 0.293456 |
| 750.21 | -0.08991 | 0.136166 | -0.66029 | 0.509065 | -0.35679 | 0.176971 | 0.088813 | Congenital anomalies of intestine | congenital anoma | 0.293226 |
| 519.1 | -0.1538 | 0.233717 | -0.65805 | 0.510506 | -0.61188 | 0.30428 | 0.014048 | Tracheostomy complications | respiratory | 0.291999 |
| 915 | -0.03928 | 0.059893 | -0.6559 | 0.511887 | -0.15667 | 0.078103 | 0.142825 | Superficial injury without mention of infection | injuries & poisoni | 0.290826 |
| 671 | -0.03802 | 0.058116 | -0.65421 | 0.512978 | -0.15192 | 0.075885 | 0.521664 | Venous/cerebrovascular complications emboli: | pregnancy compl | 0.289901 |
| 605 | -0.01751 | 0.026778 | -0.65401 | 0.513107 | -0.07 | 0.03497 | 0.277015 | Erectile dysfunction [ED] | genitourinary | 0.289792 |
| 614.54 | 0.0663 | 0.101432 | 0.653637 | 0.513346 | -0.1325 | 0.265103 | 0.592411 | Abscess or ulceration of vulva | genitourinary | 0.28959 |
| 709.5 | 0.084843 | 0.130627 | 0.649503 | 0.516013 | -0.17118 | 0.340866 | 0.205009 | Dermatomyositis | dermatologic | 0.287339 |
| 527 | 0.020181 | 0.031144 | 0.647994 | 0.516989 | -0.04086 | 0.081222 | 0.617072 | Diseases of the salivary glands | digestive | 0.286519 |
| 277.6 | 0.116422 | 0.179842 | 0.647356 | 0.517402 | -0.23606 | 0.468905 | 0.346688 | Other deficiencies of circulating enzymes | endocrine/metab | 0.286172 |
| 362.21 | 0.034185 | 0.052956 | 0.645537 | 0.518579 | -0.06961 | 0.137978 | 0.851496 | Macular degeneration, dry | sense organs | 0.285185 |
| 556 | 0.031412 | 0.04867 | 0.645392 | 0.518673 | -0.06398 | 0.126804 | 0.352577 | Ulceration of the lower GI tract | digestive | 0.285106 |
| 377.3 | -0.04078 | 0.063243 | -0.64485 | 0.519021 | -0.16474 | 0.083172 | 0.912585 | Optic neuritis/neuropathy | sense organs | 0.284815 |
| 211 | 0.018038 | 0.027995 | 0.644314 | 0.519372 | -0.03683 | 0.072907 | 0.792306 | Benign neoplasm of other parts of digestive sy | neoplasms | 0.284521 |
| 255.1 | 0.102342 | 0.159116 | 0.643194 | 0.520098 | -0.20952 | 0.414204 | 0.008515 | Adrenal hyperfunction | endocrine/metab | 0.283915 |
| 758.1 | 0.046202 | 0.071969 | 0.641967 | 0.520895 | -0.09486 | 0.187259 | 0.3173 | Chromosomal anomalies | congenital anoma | 0.28325 |
| 711.3 | 0.153773 | 0.240067 | 0.640542 | 0.52182 | -0.31675 | 0.624296 | 0.597855 | Behcet's syndrome | musculoskeletal | 0.282479 |
| 731.1 | 0.282165 | 0.440847 | 0.640053 | 0.522138 | -0.58188 | 1.14621 | 0.02766 | Osteitis deformans [Paget's disease of bone] | musculoskeletal | 0.282215 |
| 726.3 | -0.02105 | 0.032924 | -0.63941 | 0.522559 | -0.08558 | 0.043478 | 0.969214 | Bursitis | musculoskeletal | 0.281865 |
| 218.1 | 0.016613 | 0.026012 | 0.638671 | 0.523037 | -0.03437 | 0.067597 | 0.491204 | Uterine leiomyoma | neoplasms | 0.281468 |
| 599.1 | -0.02178 | 0.034135 | -0.63811 | 0.523405 | -0.08868 | 0.045122 | 0.844333 | Urinary obstruction | genitourinary | 0.281162 |
| 707.2 | 0.025264 | 0.039672 | 0.63682 | 0.524242 | -0.05249 | 0.10302 | 0.875798 | Chronic ulcer of leg or foot | dermatologic | 0.280468 |
| 381 | 0.029117 | 0.045751 | 0.636439 | 0.524491 | -0.06055 | 0.118787 | 0.069015 | Otitis media and Eustachian tube disorders | sense organs | 0.280262 |
| 528.5 | -0.05544 | 0.087157 | -0.63609 | 0.524716 | -0.22627 | 0.115385 | 0.140564 | Diseases of lips | digestive | 0.280075 |
| 579.2 | 0.020754 | 0.032652 | 0.635616 | 0.525026 | -0.04324 | 0.084751 | 0.858982 | Splenomegaly | digestive | 0.279819 |
| 636.1 | 0.065308 | 0.102843 | 0.635029 | 0.525409 | -0.13626 | 0.266876 | 0.676713 | Threatened premature labor | pregnancy compl | 0.279502 |
| 646 | -0.02329 | 0.036747 | -0.63392 | 0.526132 | -0.09532 | 0.048728 | 0.827783 | Other complications of pregnancy NEC | pregnancy compl | 0.278906 |
| 81.11 | 0.192405 | 0.303632 | 0.633678 | 0.526291 | -0.4027 | 0.787512 | 0.102194 | Acute graft-versus-host disease | infectious diseas | 0.278774 |
| 540.1 | 0.03359 | 0.05314 | 0.632109 | 0.527316 | -0.07056 | 0.137741 | 0.941553 | Appendicitis | digestive | 0.277929 |
| 594.3 | 0.053307 | 8.45E-02 | 0.631077 | 0.52799 | -0.11225 | 0.218865 | 0.272666 | Calculus of ureter | genitourinary | 0.277374 |
| 751.1 | -0.04989 | 0.079129 | -0.63049 | 0.528373 | -0.20498 | 0.1052 | 0.797927 | Congenital anomalies of genital organs | congenital anoma | 0.277059 |
| 427.12 | 0.019123 | 0.030343 | 0.630239 | 0.528538 | -0.04035 | 0.078595 | 0.897857 | Paroxysmal ventricular tachycardia | circulatory syster | 0.276924 |
| 381.2 | -0.02027 | 0.032162 | -0.63017 | 0.528581 | -0.0833 | 0.042768 | 0.605877 | Eustachian tube disorders | sense organs | 0.276889 |
| 686.2 | 0.048981 | 0.077728 | 0.630166 | 0.528586 | -0.10336 | 0.201325 | 0.193139 | Impetigo | dermatologic | 0.276884 |
| 575.6 | 0.034231 | 0.054466 | 0.628491 | 0.529683 | -0.07252 | 0.140983 | 0.628459 | Cholesterolosis of gallbladder | digestive | 0.275984 |
| 624.1 | 0.076658 | 0.122004 | 0.628318 | 0.529796 | -0.16247 | 0.315782 | 0.78546 | Dystrophy of female genital tract | genitourinary | 0.275892 |
| 701.1 | -0.02281 | 0.036396 | -0.62675 | 0.530822 | -0.09415 | 0.048523 | 0.440832 | Keratoderma, acquired | dermatologic | 0.275051 |
| 649 | -0.02648 | 0.042372 | -0.62486 | 0.532064 | -0.10952 | 0.056571 | 0.394281 | Other conditions or status of the mother compl | pregnancy compl | 0.274036 |
| 619.2 | 0.025762 | 0.041324 | 0.623404 | 0.533019 | -0.05523 | 0.106755 | 0.640119 | Disorders of uterus, NEC | genitourinary | 0.273257 |
| 599.3 | -0.01675 | 0.026891 | -0.62296 | 0.533308 | -0.06946 | 0.035954 | 0.253165 | Dysuria | genitourinary | 0.273022 |
| 368.2 | 0.026376 | 0.042389 | 0.622236 | 0.533787 | -0.0567 | 0.109456 | 0.480464 | Diplopia and disorders of binocular vision | sense organs | 0.272632 |
| 244.5 | -0.16129 | 0.259483 | -0.62157 | 0.534226 | -0.66986 | 0.347291 | 0.061795 | Congenital hypothyroidism | endocrine/metab | 0.272275 |
| 574.12 | 0.033422 | 0.054048 | 0.618373 | 0.53633 | -0.07251 | 0.139354 | 0.512941 | Cholelithiasis with other cholecystitis | digestive | 0.270568 |

| phecode | beta | se | zval | pval | ci.lb | ci.ub | QEp | phenotype | category | minuslogp |
|---|---|---|---|---|---|---|---|---|---|---|
| 274.11 | -0.02237 | 0.036187 | -0.61827 | 0.536394 | -0.0933 | 0.048552 | 0.683174 | Gouty arthropathy | endocrine/metab | 0.270516 |
| 681 | -0.01131 | 0.018335 | -0.617 | 0.537233 | -0.04725 | 0.024624 | 0.916339 | Superficial cellulitis and abscess | dermatologic | 0.269837 |
| 362.29 | 0.029609 | 0.048011 | 0.616716 | 0.537422 | -0.06449 | 0.123708 | 0.795592 | Macular degeneration (senile) of retina NOS | sense organs | 0.269685 |
| 292.3 | 0.015263 | 0.024756 | 0.616511 | 0.537557 | -0.03326 | 0.063784 | 0.309885 | Memory loss | mental disorders | 0.269575 |
| 289 | 0.012685 | 0.020595 | 0.615915 | 0.53795 | -0.02768 | 0.05305 | 0.443225 | Other diseases of blood and blood-forming org | hematopoietic | 0.269258 |
| 535.2 | 0.015246 | 0.024773 | 0.615432 | 0.53827 | -0.03331 | 0.063799 | 0.617575 | Atrophic gastritis | digestive | 0.269 |
| 654.2 | -0.15429 | 0.250892 | -0.61495 | 0.538586 | -0.64603 | 0.337452 | 0.125765 | Rhesus isoimmunization in pregnancy | pregnancy compl | 0.268745 |
| 960.3 | 0.301353 | 0.490219 | 0.614732 | 0.538732 | -6.59E-01 | 1.262164 | 0.919889 | Poisoning by antifungal antibiotics | injuries & poisoni | 0.268627 |
| 555.21 | 0.03003 | 0.04886 | 0.614616 | 0.538808 | -0.06573 | 0.125793 | 0.935849 | Ulcerative colitis (chronic) | digestive | 0.268566 |
| 442.8 | 0.037948 | 0.061984 | 0.61222 | 0.540392 | -0.08354 | 0.159435 | 0.83053 | Aneurysm of other specified artery | circulatory syster | 0.267291 |
| 303.4 | -0.19508 | 0.318748 | -0.61203 | 0.54052 | -0.81982 | 0.429653 | 0.001236 | Somatoform disorder | mental disorders | 0.267189 |
| 681.6 | 0.036102 | 0.059057 | 0.611306 | 0.540997 | -0.07965 | 0.151851 | 0.622249 | Cellulitis and abscess of foot, toe | dermatologic | 0.266805 |
| 743.22 | 0.044269 | 0.072552 | 0.610164 | 0.541753 | -0.09793 | 0.186468 | 0.480306 | Pathologic fracture of femur | musculoskeletal | 0.266199 |
| 756.3 | -0.1585 | 0.26003 | -0.60956 | 0.54215 | -0.66815 | 0.351144 | 0.129578 | Congenital anomalies of muscle, tendon, fasci | congenital anoma | 0.26588 |
| 701.2 | -0.01435 | 0.023619 | -0.60752 | 0.543504 | -0.06064 | 0.031943 | 0.312355 | Scar conditions and fibrosis of skin | dermatologic | 0.264797 |
| 575 | 0.014266 | 0.023517 | 0.606617 | 0.544105 | -0.03183 | 0.060357 | 0.559312 | Other biliary tract disease | digestive | 0.264317 |
| 452.8 | -0.05776 | 0.095325 | -0.60589 | 0.54459 | -0.24459 | 0.129078 | 0.703012 | Postphlebitic syndrome | circulatory syster | 0.263931 |
| 415.1 | 0.021177 | 0.034955 | 0.605849 | 0.544615 | -0.04733 | 0.089687 | 0.438742 | Acute pulmonary heart disease | circulatory syster | 0.26391 |
| 277 | -0.01325 | 0.021882 | -0.60548 | 0.544859 | -0.05614 | 0.029638 | 0.648013 | Other disorders of metabolism | endocrine/metab | 0.263716 |
| 728.7 | -0.02099 | 0.03474 | -0.60432 | 0.545631 | -0.08908 | 0.047095 | 0.278622 | Fasciitis | musculoskeletal | 0.263101 |
| 694.1 | 0.051616 | 0.085426 | 0.604215 | 0.545701 | -0.11582 | 0.219047 | 0.665625 | Vitiligo | dermatologic | 0.263045 |
| 386.3 | 0.04857 | 0.080418 | 0.603967 | 0.545866 | -0.10905 | 0.206186 | 0.703746 | Labyrinthitis | sense organs | 0.262914 |
| 429.9 | 0.058796 | 0.097667 | 0.601998 | 0.547175 | -0.13263 | 0.25022 | 0.099 | Cardiac complications, not elsewhere classifie | circulatory syster | 0.261874 |
| 342 | -0.03946 | 0.065593 | -0.60155 | 0.547476 | -0.16802 | 0.089102 | 0.26036 | Hemiplegia | neurological | 0.261635 |
| 270 | 0.020603 | 0.03434 | 0.599965 | 0.54853 | -0.0467 | 0.087909 | 0.545518 | Disorders of protein plasma/amino-acid transp | endocrine/metab | 0.2608 |
| 331 | -0.03404 | 0.056752 | -0.59987 | 0.548592 | -0.14527 | 0.077188 | 0.321058 | Other cerebral degenerations | neurological | 0.26075 |
| 619.5 | -0.0277 | 0.046243 | -0.59892 | 0.549229 | -0.11833 | 0.062939 | 0.372428 | Noninflammatory disorders of vulva and perine | genitourinary | 0.260247 |
| 586.1 | -0.0532 | 0.088852 | -0.59879 | 0.549311 | -0.22735 | 0.120942 | 0.722165 | Anatomical abnormatilies of kidney and ureter | genitourinary | 0.260182 |
| 747.2 | -0.05061 | 0.084651 | -0.59791 | 0.549903 | -0.21653 | 0.115299 | 0.132886 | Congenital anomalies of peripheral vascular sy | congenital anoma | 0.259714 |
| 756.21 | -0.05627 | 0.094298 | -0.59675 | 0.550673 | -0.24109 | 0.128549 | 0.300377 | Pectus excavatum | congenital anoma | 0.259107 |
| 288.11 | 0.018773 | 0.031504 | 0.595902 | 0.551241 | -0.04297 | 0.080521 | 0.200652 | Neutropenia | hematopoietic | 0.258659 |
| 620.1 | 0.053577 | 0.090114 | 0.594542 | 0.55215 | -0.12304 | 0.230197 | 0.676185 | Dysplasia of cervix | genitourinary | 0.257943 |
| 726.1 | 0.017393 | 0.029275 | 0.594147 | 0.552414 | -0.03998 | 0.074771 | 0.170791 | Enthesopathy | musculoskeletal | 0.257735 |
| 938.2 | -0.01753 | 0.029513 | -0.59398 | 0.552523 | -0.07538 | 0.040315 | 0.732658 | Chronic dermatitis due to solar radiation | dermatologic | 0.25765 |
| 376 | -0.06408 | 0.108057 | -0.59305 | 0.553145 | -0.27587 | 0.147705 | 0.337469 | Disorders of the orbit | sense organs | 0.257161 |
| 444 | 0.026302 | 0.04438 | 0.592646 | 0.553418 | -0.06068 | 0.113286 | 0.81575 | Arterial embolism and thrombosis | circulatory syster | 0.256946 |
| 875 | 0.057891 | 0.097759 | 0.592179 | 0.553731 | -0.13371 | 0.249496 | 0.825559 | Non-healing surgical wound | injuries & poisoni | 0.256702 |
| 530.12 | 0.046727 | 0.079365 | 0.588763 | 0.55602 | -0.10883 | 0.20228 | 0.116794 | Ulcer of esophagus | digestive | 0.254909 |
| 149.9 | 0.15385 | 0.262155 | 0.586866 | 0.557293 | -0.35996 | 0.667665 | 0.040341 | Cancer of of nasal cavities | neoplasms | 0.253916 |
| 262 | 0.04443 | 0.075805 | 0.586108 | 0.557803 | -0.10414 | 0.193004 | 0.03666 | Mineral deficiency NEC | endocrine/metab | 0.253519 |
| 362.7 | -0.12653 | 0.21607 | -0.58561 | 0.558136 | -0.55002 | 0.296956 | 0.031976 | Hereditary retinal dystrophies | sense organs | 0.25326 |
| 635 | -0.03246 | 0.055665 | -0.58314 | 0.559799 | -0.14156 | 0.076641 | 0.195519 | Hemorrhage during pregnancy; childbirth and | pregnancy compl | 0.251968 |
| 707.3 | 0.022063 | 0.037856 | 0.582823 | 0.560012 | -0.05213 | 0.09626 | 0.604172 | Chronic ulcer of unspecified site | dermatologic | 0.251802 |
| 578.9 | 0.017834 | 0.030695 | 0.580992 | 0.561246 | -0.04233 | 0.077995 | 0.643713 | Hemorrhage of gastrointestinal tract | digestive | 0.250847 |
| 172.21 | -0.01445 | 0.024884 | -0.58055 | 0.561547 | -0.06322 | 0.034325 | 0.884306 | Basal cell carcinoma | neoplasms | 0.250614 |
| 331.9 | -0.09084 | 0.157245 | -0.57772 | 0.563456 | -0.39904 | 0.217351 | 0.013965 | Cerebral degeneration, unspecified | neurological | 0.24914 |
| 714.1 | 0.019764 | 0.034217 | 0.577603 | 0.563532 | -0.0473 | 0.086829 | 0.861347 | Rheumatoid arthritis | musculoskeletal | 0.249081 |
| 362.4 | 0.028747 | 0.04986 | 0.576561 | 0.564236 | -0.06898 | 0.12647 | 0.265436 | Retinal vascular changes and abnomalities | sense organs | 0.248539 |

| phecode | beta | se | zval | pval | ci.lb | ci.ub | QEp | phenotype | category | minuslogp |
|---|---|---|---|---|---|---|---|---|---|---|
| 788 | -0.02356 | 0.040946 | -0.57546 | 0.56498 | -0.10381 | 0.056689 | 0.095684 | Syncope and collapse | symptoms | 0.247967 |
| 348.8 | 0.020818 | 0.036206 | 0.574991 | 0.565298 | -0.05014 | 0.091779 | 0.41195 | Encephalopathy, not elsewhere classified | neurological | 0.247723 |
| 246.2 | -0.04302 | 0.075004 | -0.57362 | 0.566225 | -0.19003 | 0.103982 | 0.696867 | Thyroid cyst | endocrine/metab | 0.247011 |
| 801 | 0.034523 | 0.060419 | 0.571395 | 0.567732 | -0.0839 | 0.152942 | 0.10291 | Fracture of ankle and foot | injuries & poisoni | 0.245857 |
| 910 | -0.06788 | 0.118838 | -0.57116 | 0.567889 | -0.30079 | 0.165042 | 0.888921 | Superficial injury, infected | injuries & poisoni | 0.245737 |
| 598 | -0.03512 | 0.061494 | -0.57113 | 0.567909 | -0.15565 | 0.085404 | 0.034691 | Abnormal findings on examination of urine | genitourinary | 0.245721 |
| 270.38 | 0.026461 | 0.04653 | 0.568683 | 0.569571 | -0.06474 | 0.117657 | 0.814578 | Other specified disorders of plasma protein me | endocrine/metab | 0.244452 |
| 714.2 | -0.1179 | 0.208394 | -0.56574 | 0.571574 | -0.52634 | 0.290549 | 0.196516 | Juvenile rheumatoid arthritis | musculoskeletal | 0.242928 |
| 325 | -0.11974 | 0.211787 | -0.56537 | 0.571825 | -0.53483 | 0.295357 | 0.326899 | Phlebitis and thrombophlebitis of intracranial v | neurological | 0.242737 |
| 415.11 | 0.019486 | 0.034962 | 0.557366 | 0.577277 | -0.04904 | 0.08801 | 0.448489 | Pulmonary embolism and infarction, acute | circulatory syster | 0.238616 |
| 323.8 | 0.045779 | 0.082304 | 0.556218 | 0.578062 | -0.11553 | 0.207091 | 0.199037 | Encephalitis, non-infectious | neurological | 0.238026 |
| 242.3 | -0.04244 | 0.076361 | -0.55582 | 0.578336 | -0.19211 | 0.107222 | 0.863677 | Exophthalmos | endocrine/metab | 0.23782 |
| 612.2 | -0.02762 | 0.049851 | -0.55408 | 0.579522 | -0.12533 | 0.070085 | 0.931899 | Hypertrophy of breast (Gynecomastia) | genitourinary | 0.23693 |
| 550.3 | 0.140215 | 0.253616 | 0.552863 | 0.580357 | -0.35686 | 0.637294 | 0.079704 | Femoral hernia | digestive | 0.236305 |
| 452.1 | 0.231672 | 0.419292 | 0.552531 | 0.580584 | -0.59013 | 1.053469 | 0.365347 | Iatrogenic pulmonary embolism and infarction | circulatory syster | 0.236135 |
| 656.6 | 0.164653 | 0.298702 | 0.551226 | 0.581479 | -0.42079 | 0.750099 | 0.582545 | Perinatal disorders of digestive system | pregnancy compl | 0.235466 |
| 271.9 | 0.172099 | 0.312477 | 0.550758 | 0.581799 | -0.44034 | 0.784542 | 0.015388 | Other disorders of carbohydrate transport and | endocrine/metab | 0.235227 |
| 259.3 | 0.303655 | 0.551402 | 0.550695 | 0.581843 | -0.77707 | 1.384383 | 0.914042 | Delay in sexual development and puberty NEC | endocrine/metab | 0.235194 |
| 290.16 | -0.04194 | 0.076269 | -0.54984 | 0.582432 | -0.19142 | 0.10755 | 0.935516 | Vascular dementia | mental disorders | 0.234755 |
| 189.21 | 0.086365 | 0.157286 | 0.549094 | 0.582941 | -0.22191 | 0.394641 | 0.03256 | Malignant neoplasm of bladder | neoplasms | 0.234375 |
| 289.9 | 0.021753 | 0.03967 | 0.548335 | 0.583462 | -0.056 | 0.099505 | 0.95801 | Abnormality of red blood cells | hematopoietic | 0.233987 |
| 870.4 | 0.067171 | 0.122514 | 0.548274 | 0.583504 | -0.17295 | 0.307294 | 0.589312 | Open wound of nose and sinus | injuries & poisoni | 0.233956 |
| 686.4 | -0.07049 | 0.12898 | -0.54651 | 0.584712 | -0.32329 | 0.182307 | 0.184604 | Pyogenic granuloma | dermatologic | 0.233058 |
| 724 | 0.019128 | 0.035053 | 0.54567 | 0.585293 | -0.04958 | 0.087831 | 0.229553 | Other and unspecified disorders of back | musculoskeletal | 0.232627 |
| 602 | 0.092721 | 0.169966 | 0.545528 | 0.585391 | -0.24041 | 0.425848 | 0.025444 | Other disorders of prostate | genitourinary | 0.232554 |
| 800 | 0.015012 | 0.02761 | 0.543712 | 0.58664 | -0.0391 | 0.069127 | 0.80303 | Fracture of lower limb | injuries & poisoni | 0.231629 |
| 285.22 | -0.02051 | 0.037824 | -0.54225 | 0.587644 | -0.09464 | 0.053623 | 0.857093 | Anemia in neoplastic disease | hematopoietic | 0.230886 |
| 514.1 | 0.02772 | 0.051172 | 0.541709 | 0.588019 | -0.07257 | 0.128015 | 0.29067 | Abnormal results of function study of pulmonar | respiratory | 0.230609 |
| 577.3 | 0.021882 | 0.0405 | 0.540299 | 0.588991 | -0.0575 | 0.10126 | 0.819334 | Cyst and pseudocyst of pancreas | digestive | 0.229892 |
| 691 | 0.025855 | 0.047975 | 0.538933 | 0.589933 | -0.06817 | 0.119885 | 0.603173 | Congenital anomalies of skin | dermatologic | 0.229197 |
| 612 | -0.02382 | 0.044229 | -0.53859 | 0.59017 | -0.11051 | 0.062865 | 0.833694 | Breast conditions, congenital or relating to hor | genitourinary | 0.229023 |
| 750.5 | -0.2611 | 0.485039 | -0.53832 | 0.590359 | -1.21176 | 0.689554 | 0.912392 | Congenital hypertrophic pyloric stenosis | congenital anoma | 0.228884 |
| 573.1 | 0.038063 | 0.071314 | 0.53374 | 0.593521 | -0.10171 | 0.177836 | 0.429959 | Chronic passive congestion of liver | digestive | 0.226564 |
| 988 | 0.214588 | 0.402332 | 0.53336 | 0.593784 | -0.57397 | 1.003145 | 0.313679 | Toxic effect of noxious substances eaten as fo | injuries & poisoni | 0.226371 |
| 513.31 | 0.028362 | 0.053291 | 0.532209 | 0.594582 | -0.07609 | 0.13281 | 0.477184 | Apnea | respiratory | 0.225789 |
| 807 | -0.03005 | 0.056498 | -0.53197 | 0.594749 | -0.14079 | 0.080679 | 0.2357 | Fracture of ribs | injuries & poisoni | 0.225666 |
| 804 | -0.03428 | 0.064498 | -0.5315 | 0.595073 | -0.16069 | 0.092133 | 0.250492 | Fracture of hand or wrist | injuries & poisoni | 0.22543 |
| 480.3 | -0.06366 | 0.119856 | -0.53115 | 0.595316 | -0.29858 | 0.171253 | 0.086376 | Pneumonia due to fungus (mycoses) | respiratory | 0.225253 |
| 505 | -0.02951 | 0.055583 | -0.53097 | 0.595439 | -0.13845 | 0.079427 | 0.475635 | Other pulmonary inflamation or edema | respiratory | 0.225162 |
| 184.11 | 0.05578 | 0.105132 | 0.530574 | 0.595714 | -0.15028 | 0.261836 | 0.079754 | Malignant neoplasm of ovary | neoplasms | 0.224962 |
| 716.2 | 0.071771 | 0.135404 | 0.530055 | 0.596074 | -0.19361 | 0.337157 | 0.803012 | Unspecified monoarthritis | musculoskeletal | 0.2247 |
| 657 | 0.185837 | 0.350718 | 0.529876 | 0.596198 | -0.50156 | 0.873232 | 0.976405 | Infections specific to the perinatal period | pregnancy compl | 0.22461 |
| 601.11 | -0.06218 | 0.11745 | -0.52943 | 0.596506 | -0.29238 | 0.168016 | 0.170786 | Acute prostatitis | genitourinary | 0.224385 |
| 716.8 | 0.193434 | 0.365846 | 0.528729 | 0.596993 | -0.52361 | 0.91048 | 0.893407 | Palindromic rheumatism | musculoskeletal | 0.224031 |
| 300.4 | 0.016089 | 0.030481 | 0.527835 | 0.597614 | -0.04365 | 0.075831 | 0.552166 | Dysthymic disorder | mental disorders | 0.223579 |
| 500 | 0.037357 | 0.070881 | 0.527043 | 0.598164 | -0.10157 | 0.176282 | 0.189836 | Lung disease due to external agents | respiratory | 0.22318 |
| 743.2 | 0.019225 | 0.036669 | 0.524299 | 0.600071 | -0.05264 | 0.091094 | 0.92577 | Pathologic fracture | musculoskeletal | 0.221798 |
| 259.1 | -0.17418 | 0.33222 | -0.52428 | 0.600081 | -0.82532 | 0.476962 | 0.619398 | Nonspecific abnormal results of other endocrir | endocrine/metab | 0.22179 |

| phecode | beta | se | zval | pval | ci.lb | ci.ub | QEp | phenotype | category | minuslogp |
|---|---|---|---|---|---|---|---|---|---|---|
| 727.5 | 0.023697 | 0.045236 | 0.523864 | 0.600373 | -0.06496 | 0.112358 | 0.68026 | Rupture of synovium | musculoskeletal | 0.221579 |
| 960.2 | -0.01889 | 0.036119 | -0.5229 | 0.601043 | -0.08968 | 0.051905 | 0.119245 | Allergy/adverse effect of penicillin | injuries & poisoni | 0.221095 |
| 282.8 | 0.028759 | 0.055031 | 0.522597 | 0.601255 | -0.0791 | 0.136617 | 0.597713 | Other hemoglobinopathies | hematopoietic | 0.220941 |
| 362.23 | -0.04034 | 0.077254 | -0.52212 | 0.601585 | -0.19175 | 0.111079 | 0.207968 | Cystoid macular degeneration of retina | sense organs | 0.220703 |
| 386 | 0.020121 | 0.038537 | 0.522121 | 0.601586 | -0.05541 | 0.095653 | 0.196758 | Vertiginous syndromes and other disorders of | sense organs | 0.220702 |
| 204.11 | -0.05434 | 0.104469 | -0.52019 | 0.602929 | -0.2591 | 0.150411 | 0.918557 | Lymphoid leukemia, acute | neoplasms | 0.219734 |
| 753 | -0.06248 | 0.120168 | -0.5199 | 0.603134 | -0.298 | 0.17305 | 0.282017 | Congenital anomalies of the eye | congenital anoma | 0.219586 |
| 603 | 0.026319 | 0.050899 | 0.517086 | 0.605096 | -0.07344 | 0.12608 | 0.859934 | Other disorders of testis | genitourinary | 0.218175 |
| 736.6 | 0.040571 | 0.078539 | 0.516569 | 0.605457 | -0.11336 | 0.194505 | 0.313044 | Unequal leg length (acquired) | musculoskeletal | 0.217917 |
| 362.3 | 0.054372 | 0.105353 | 0.516088 | 0.605793 | -0.15212 | 0.26086 | 0.073991 | Other nondiabetic retinopathy | sense organs | 0.217676 |
| 690.1 | -0.01356 | 0.0263 | -0.51567 | 0.606086 | -0.06511 | 0.037985 | 0.655485 | Seborrheic dermatitis | dermatologic | 0.217465 |
| 260.22 | -0.09321 | 0.181318 | -0.51408 | 0.607198 | -0.44859 | 0.262165 | 0.058115 | Nutritional marasmus | endocrine/metab | 0.21667 |
| 250.14 | 0.044819 | 0.087229 | 0.513807 | 0.607387 | -0.12615 | 0.215786 | 0.985567 | Type 1 diabetes with neurological manifestatio | endocrine/metab | 0.216535 |
| 800.3 | -0.05512 | 0.107686 | -0.51188 | 0.608732 | -0.26618 | 0.155938 | 0.085298 | Fracture of tibia and fibula | injuries & poisoni | 0.215574 |
| 394.7 | 0.023115 | 0.045169 | 0.511732 | 0.608838 | -0.06542 | 0.111645 | 0.42339 | Disease of tricuspid valve | circulatory syster | 0.215498 |
| 960.1 | -0.09361 | 0.18306 | -0.51138 | 0.609088 | -0.4524 | 0.265179 | 0.501072 | Adverse effects of antibacterials (not penicillins | injuries & poisoni | 0.21532 |
| 159.4 | -0.03892 | 0.076208 | -0.51071 | 0.609552 | -0.18829 | 0.110444 | 0.459187 | Malignant neoplasm of retroperitoneum and pe | neoplasms | 0.214989 |
| 623 | -0.02238 | 0.043928 | -0.5095 | 0.610404 | -0.10848 | 0.063716 | 0.850149 | Hypertrophy of female genital organs | genitourinary | 0.214383 |
| 537.1 | 0.042541 | 0.083527 | 0.509313 | 0.610533 | -0.12117 | 0.20625 | 0.622619 | Lesions of stomach and duodenum | digestive | 0.214291 |
| 531.2 | -0.01737 | 0.034119 | -0.50909 | 0.610687 | -0.08424 | 0.049502 | 0.688913 | Gastric ulcer | digestive | 0.214181 |
| 649.1 | 0.047166 | 0.092658 | 0.509032 | 0.61073 | -0.13444 | 0.228772 | 0.102477 | Diabetes or abnormal glucose tolerance comp | pregnancy compl | 0.214151 |
| 364.2 | 0.13582 | 0.267373 | 0.507981 | 0.611467 | -0.38822 | 0.659861 | 0.01898 | Corneal edema | sense organs | 0.213627 |
| 772.6 | 0.027797 | 5.47E-02 | 0.50789 | 0.61153 | -0.07947 | 0.135069 | 0.936202 | Facial weakness | symptoms | 0.213582 |
| 949.1 | 0.084581 | 0.167534 | 0.504857 | 0.613659 | -0.24378 | 0.412942 | 0.959007 | Diaper or napkin rash | injuries & poisoni | 0.212073 |
| 960 | 0.012385 | 0.024581 | 0.503849 | 0.614368 | -0.03579 | 0.060563 | 0.319087 | Poisoning by antibiotics | injuries & poisoni | 0.211572 |
| 615 | 0.029056 | 0.057767 | 0.50298 | 0.614978 | -0.08417 | 0.142277 | 0.108465 | Endometriosis | genitourinary | 0.21114 |
| 513.3 | 0.031029 | 0.062024 | 0.500275 | 0.616881 | -0.09054 | 0.152594 | 0.139897 | Hypoventilation | respiratory | 0.209798 |
| 681.7 | 0.018248 | 0.036481 | 0.500203 | 0.616932 | -0.05325 | 0.089748 | 0.689741 | Cellulitis and abscess of trunk | dermatologic | 0.209763 |
| 726.2 | 0.019092 | 0.038171 | 0.500179 | 0.616949 | -0.05572 | 0.093906 | 0.957768 | Synoviopathy | musculoskeletal | 0.209751 |
| 345.11 | -0.03533 | 0.070637 | -0.50014 | 0.616975 | -0.17378 | 0.103118 | 0.622104 | Generalized convulsive epilepsy | neurological | 0.209733 |
| 798 | -0.00697 | 0.013947 | -0.49956 | 0.617382 | -0.0343 | 0.020369 | 0.496911 | Malaise and fatigue | symptoms | 0.209446 |
| 626.11 | -0.03592 | 0.071973 | -0.49905 | 0.617747 | -0.17698 | 0.105147 | 0.060643 | Absent or infrequent menstruation | genitourinary | 0.209189 |
| 990 | -0.01103 | 0.022129 | -0.49834 | 0.618246 | -0.0544 | 0.032344 | 0.358726 | Effects radiation NOS | injuries & poisoni | 0.208839 |
| 614.1 | 0.042259 | 0.085492 | 0.494307 | 0.621089 | -0.1253 | 0.20982 | 0.345248 | Pelvic peritoneal adhesions, female (postopera | genitourinary | 0.206846 |
| 389.4 | -0.0229 | 0.046361 | -0.49404 | 0.621275 | -0.11377 | 0.067962 | 0.086008 | Tinnitus | sense organs | 0.206716 |
| 626.13 | 0.019373 | 0.039438 | 0.491219 | 0.623272 | -0.05792 | 0.096669 | 0.237307 | Irregular menstrual cycle | genitourinary | 0.205323 |
| 526.41 | 0.02413 | 0.04917 | 0.490742 | 0.623609 | -0.07224 | 0.120501 | 0.576877 | Temporomandibular joint disorder, unspecified | digestive | 0.205088 |
| 704.1 | -0.01388 | 0.028291 | -0.49074 | 0.623613 | -0.06933 | 0.041566 | 0.769224 | Alopecia | dermatologic | 0.205085 |
| 479 | 0.009562 | 0.019538 | 0.489396 | 0.624562 | -0.02873 | 0.047857 | 0.36455 | Other upper respiratory disease | respiratory | 0.204425 |
| 427.11 | -0.01913 | 0.039348 | -0.48611 | 0.626892 | -0.09625 | 0.057994 | 0.27431 | Paroxysmal supraventricular tachycardia | circulatory syster | 0.202807 |
| 361.2 | 0.167375 | 0.344621 | 0.485679 | 0.627195 | -0.50807 | 0.842821 | 0.011195 | Retinoschisis and retinal cysts | sense organs | 0.202597 |
| 420 | 0.012925 | 0.02665 | 0.485005 | 0.627673 | -0.03931 | 0.065158 | 0.506872 | Carditis | circulatory syster | 0.202267 |
| 674 | -0.04236 | 0.087356 | -0.48494 | 0.62772 | -0.21358 | 0.128853 | 0.790568 | Other complications of the puerperium NEC | pregnancy compl | 0.202234 |
| 980 | 0.015939 | 0.032896 | 0.48452 | 0.628017 | -0.04854 | 0.080413 | 0.708163 | Encounter for long-term (current) use of antibio | infectious diseas | 0.202029 |
| 145.5 | 0.179357 | 0.370718 | 0.48381 | 0.628521 | -0.54724 | 0.90595 | 0.052002 | Cancer of the mouth floor | neoplasms | 0.20168 |
| 345.3 | 0.01627 | 0.033661 | 0.483363 | 0.628838 | -0.0497 | 0.082244 | 0.377747 | Convulsions | neurological | 0.201461 |
| 687.2 | -0.01202 | 0.024925 | -0.48241 | 0.629515 | -0.06088 | 0.036828 | 0.378423 | Localized superficial swelling, mass, or lump | dermatologic | 0.200994 |
| 755.1 | 0.066928 | 0.138961 | 0.481635 | 0.630065 | -0.20543 | 0.339287 | 0.079118 | Congenital deformities of feet | congenital anoma | 0.200614 |

| phecode | beta | se | zval | pval | ci.lb | ci.ub | QEp | phenotype | category | minuslogp |
|---|---|---|---|---|---|---|---|---|---|---|
| 79 | 0.016764 | 0.034815 | 0.481529 | 0.63014 | -0.05147 | 0.085 | 0.017793 | Viral infection | infectious diseas | 0.200563 |
| 626.8 | -0.04708 | 0.097981 | -0.48048 | 0.630885 | -0.23912 | 0.144962 | 0.067771 | Infertility, female | genitourinary | 0.20005 |
| 425.11 | 0.044253 | 0.092198 | 0.479974 | 0.631246 | -0.13645 | 0.224957 | 0.96064 | Hypertrophic obstructive cardiomyopathy | circulatory syster | 0.199801 |
| 279.8 | 0.033579 | 0.069994 | 0.479746 | 0.631408 | -0.10361 | 0.170764 | 0.937111 | Other specified disorders involving the immune | endocrine/metab | 0.19969 |
| 110.11 | 0.021208 | 0.044213 | 0.479689 | 0.631449 | -0.06545 | 0.107864 | 0.059777 | Dermatophytosis of nail | infectious diseas | 0.199662 |
| 752.11 | -0.06041 | 0.12621 | -0.47861 | 0.632215 | -0.30777 | 0.186962 | 0.781758 | Spina bifida | congenital anoma | 0.199135 |
| 592.12 | 0.096513 | 0.201779 | 0.478311 | 0.632429 | -0.29897 | 0.491993 | 0.055452 | Chronic cystitis | genitourinary | 0.198988 |
| 427.22 | 0.014309 | 0.030026 | 0.47657 | 0.633668 | -0.04454 | 0.073159 | 0.81381 | Atrial flutter | circulatory syster | 0.198138 |
| 712 | 0.165394 | 0.347779 | 0.475572 | 0.634379 | -0.51624 | 0.847028 | 0.003193 | Infective connective tissue disorders | musculoskeletal | 0.197651 |
| 202.21 | -0.09482 | 0.199615 | -0.47502 | 0.634769 | -0.48606 | 0.296417 | 0.063547 | Nodular lymphoma | neoplasms | 0.197384 |
| 840.3 | -0.0268 | 0.05668 | -0.47291 | 0.63628 | -0.13789 | 0.084286 | 0.32393 | Joint/ligament sprain | injuries & poisoni | 0.196352 |
| 966 | 0.181649 | 0.384899 | 0.471939 | 0.63697 | -0.57274 | 0.936037 | 0.162376 | Poisoning by anticonvulsants and anti-Parkins | injuries & poisoni | 0.195881 |
| 158 | 0.042263 | 0.089636 | 0.471501 | 0.637283 | -0.13342 | 0.217947 | 0.048418 | Neoplasm of unspecified nature of digestive sy | neoplasms | 0.195668 |
| 292.1 | -0.01559 | 0.033082 | -0.47131 | 0.637419 | -0.08043 | 0.049248 | 0.870862 | Aphasia/speech disturbance | mental disorders | 0.195575 |
| 245.1 | -0.08166 | 0.173598 | -0.47039 | 0.63808 | -0.4219 | 0.258587 | 0.194803 | Thyroiditis, acute and subacute | endocrine/metab | 0.195125 |
| 452 | 0.024497 | 0.052167 | 0.469596 | 0.638644 | -0.07775 | 0.126743 | 0.018039 | Other venous embolism and thrombosis | circulatory syster | 0.194741 |
| 781.1 | -0.11157 | 0.237633 | -0.46951 | 0.638705 | -0.57732 | 0.354181 | 0.049696 | Loss of height | symptoms | 0.1947 |
| 368.4 | -0.03415 | 0.072792 | -0.46912 | 0.638981 | -0.17682 | 0.108522 | 0.177416 | Visual field defects | sense organs | 0.194512 |
| 756.22 | 0.209845 | 0.447446 | 0.468983 | 0.639081 | -0.66713 | 1.086822 | 0.323753 | Pectus carinatum | congenital anoma | 0.194444 |
| 619.4 | -0.0185 | 0.039509 | -0.46821 | 0.639633 | -0.09593 | 0.058937 | 0.107785 | Noninflammatory disorders of vagina | genitourinary | 0.194069 |
| 747.13 | 0.026057 | 0.055692 | 0.467874 | 0.639874 | -0.0831 | 0.135212 | 0.557203 | Congenital anomalies of great vessels | congenital anoma | 0.193905 |
| 285.8 | 0.108873 | 0.233634 | 0.465998 | 0.641217 | -0.34904 | 0.566787 | 0.084201 | Hemoglobinuria | hematopoietic | 0.192995 |
| 362.6 | 0.029001 | 0.062245 | 0.465919 | 0.641274 | -0.093 | 0.150998 | 0.892552 | Peripheral retinal degenerations | sense organs | 0.192957 |
| 130 | -0.08576 | 0.184392 | -0.46512 | 0.641844 | -0.44717 | 0.275637 | 0.233385 | Spirochetal infection | infectious diseas | 0.19257 |
| 741.6 | 0.096007 | 0.206469 | 0.464993 | 0.641936 | -0.30867 | 0.500679 | 0.509913 | Villonodular synovitis | musculoskeletal | 0.192508 |
| 70.1 | 0.052061 | 0.112038 | 4.65E-01 | 0.642168 | -0.16753 | 0.27165 | 0.628791 | Viral hepatitis A | infectious diseas | 0.192352 |
| 275 | 0.012755 | 0.027474 | 0.464252 | 0.642467 | -0.04109 | 0.066604 | 0.15087 | Disorders of mineral metabolism | endocrine/metab | 0.192149 |
| 281.11 | -0.19613 | 0.423032 | -0.46364 | 0.642906 | -1.02526 | 0.632993 | 3.41E-05 | Pernicious anemia | hematopoietic | 0.191853 |
| 790 | 0.01327 | 0.028629 | 0.463511 | 0.642998 | -0.04284 | 0.069383 | 0.324075 | Nonspecific findings on examination of blood | symptoms | 0.19179 |
| 530.13 | 0.028584 | 0.061697 | 0.463294 | 0.643154 | -0.09234 | 0.149508 | 0.428027 | Barrett's esophagus | digestive | 0.191685 |
| 610.3 | 0.053732 | 0.116035 | 0.46307 | 0.643315 | -0.17369 | 0.281157 | 0.112647 | Fibrosclerosis of breast | genitourinary | 0.191577 |
| 575.1 | -0.03016 | 0.065145 | -0.46293 | 0.643413 | -0.15784 | 0.097524 | 0.663782 | Cholangitis | digestive | 0.19151 |
| 465.2 | -0.00898 | 0.019412 | -0.46266 | 0.643608 | -0.04703 | 0.029066 | 0.918834 | Acute pharyngitis | respiratory | 0.191378 |
| 366.1 | 0.097716 | 0.21173 | 0.461512 | 0.644431 | -0.31727 | 0.5127 | 0.053278 | Nonsenile Cataract | sense organs | 0.190823 |
| 747 | -0.01346 | 0.029217 | -0.46059 | 0.645095 | -0.07072 | 0.043807 | 0.545245 | Cardiac and circulatory congenital anomalies | congenital anoma | 0.190376 |
| 500.2 | 0.169945 | 0.369041 | 0.460506 | 0.645153 | -0.55336 | 0.893252 | 0.015943 | Pneumoconiosis | respiratory | 0.190337 |
| 733.8 | 0.046985 | 0.102036 | 0.460472 | 0.645178 | -0.153 | 0.246971 | 0.238648 | Malunion and nonunion of fracture | musculoskeletal | 0.190321 |
| 598.9 | -0.03029 | 0.065811 | -0.46026 | 0.645326 | -0.15928 | 0.098696 | 0.024717 | Other nonspecific findings on examination of u | genitourinary | 0.190221 |
| 327.41 | -0.0176 | 0.038249 | -0.4601 | 0.645444 | -0.09257 | 0.057369 | 0.328838 | Organic or persistent insomnia | neurological | 0.190142 |
| 528.12 | -0.02956 | 0.064407 | -0.45897 | 0.646257 | -0.1558 | 0.096674 | 0.628895 | Oral aphthae | digestive | 0.189595 |
| 756.5 | -0.11868 | 0.259021 | -0.45819 | 0.646819 | -0.62635 | 0.388992 | 0.017686 | Congenital osteodystrophies | congenital anoma | 0.189217 |
| 245 | 0.020029 | 0.043823 | 0.457046 | 0.647638 | -0.06586 | 0.105921 | 0.400925 | Thyroiditis | endocrine/metab | 0.188668 |
| 379.9 | -0.01265 | 0.027676 | -0.45693 | 0.647721 | -0.06689 | 0.041598 | 0.633302 | Pain, swelling or discharge of eye | sense organs | 0.188612 |
| 696.4 | 0.014958 | 0.032773 | 0.456414 | 0.648092 | -0.04928 | 0.079191 | 0.965199 | Psoriasis | dermatologic | 0.188363 |
| 536.3 | 0.034713 | 0.07625 | 0.455255 | 0.648926 | -0.11473 | 0.184161 | 0.071737 | Gastroparesis | digestive | 0.187805 |
| 38.2 | -0.0297 | 0.065267 | -4.55E-01 | 0.649028 | -0.15763 | 0.098218 | 0.194184 | Gram positive septicemia | infectious diseas | 0.187737 |
| 174.3 | -0.07682 | 0.168976 | -0.4546 | 0.6494 | -0.408 | 0.254371 | 0.241547 | Neoplasm of uncertain behavior of breast | neoplasms | 0.187488 |
| 752 | -0.02653 | 0.058381 | -0.45438 | 0.649557 | -0.14095 | 0.087898 | 0.95886 | Nervous system congenital anomalies | congenital anoma | 0.187383 |

| phecode | beta | se | zval | pval | ci.lb | ci.ub | QEp | phenotype | category | minuslogp |
|---|---|---|---|---|---|---|---|---|---|---|
| 599.9 | 0.011334 | 0.025029 | 0.452846 | 0.650659 | -0.03772 | 0.060389 | 0.798615 | Other abnormality of urination | genitourinary | 0.186646 |
| 561.2 | 0.01654 | 0.036621 | 0.451669 | 0.651508 | -0.05523 | 0.088316 | 0.025213 | Flatulence | digestive | 0.18608 |
| 216.1 | 0.018435 | 0.041068 | 0.448887 | 0.653513 | -0.06206 | 0.098927 | 0.09737 | Screening for malignant neoplasms of the skin | neoplasms | 0.184746 |
| 364.5 | 0.075814 | 0.169319 | 0.447759 | 0.654327 | -0.25604 | 0.407673 | 0.039081 | Corneal dystrophy | sense organs | 0.184205 |
| 801.1 | 0.044575 | 0.099634 | 0.44739 | 0.654594 | -0.1507 | 0.239854 | 0.112996 | Fracture of foot | injuries & poisoni | 0.184028 |
| 751.12 | 0.068594 | 0.153442 | 0.447037 | 0.654848 | -0.23215 | 0.369336 | 0.549492 | Congenital anomalies of male genital organs | congenital anoma | 0.183859 |
| 859 | 0.015952 | 0.035775 | 0.445907 | 0.655664 | -0.05417 | 0.086071 | 0.281426 | Complication due to other implant and internal | injuries & poisoni | 0.183318 |
| 382 | -0.01099 | 0.024661 | -0.44559 | 0.655893 | -0.05932 | 0.037346 | 0.430289 | Otalgia | sense organs | 0.183167 |
| 275.6 | -0.01279 | 0.028789 | -0.44424 | 0.656872 | -0.06921 | 0.043636 | 0.226987 | Hypercalcemia | endocrine/metab | 0.18252 |
| 473.3 | -0.021 | 0.047323 | -0.44367 | 0.657282 | -0.11375 | 0.071756 | 0.887944 | Paralysis/spasm of vocal cords or larynx | respiratory | 0.182248 |
| 198.4 | -0.01792 | 0.040605 | -0.44133 | 0.658974 | -0.0975 | 0.061664 | 0.22819 | Secondary malignant neoplasm of liver | neoplasms | 0.181131 |
| 53.1 | 0.028379 | 0.064384 | 4.41E-01 | 0.659375 | -0.09781 | 0.15457 | 0.517175 | Herpes zoster with nervous system complicati | infectious diseas | 0.180867 |
| 550.6 | -0.02171 | 0.049523 | -0.43838 | 0.66111 | -0.11877 | 0.075354 | 0.950106 | Incisional hernia | digestive | 0.179727 |
| 270.32 | 0.036092 | 0.0827 | 0.436425 | 0.662528 | -0.126 | 0.198181 | 0.162034 | Paraproteinemia | endocrine/metab | 0.178796 |
| 686.1 | -0.04546 | 0.104185 | -0.43631 | 0.66261 | -0.24965 | 0.158741 | 0.110242 | Carbuncle and furuncle | dermatologic | 0.178742 |
| 609.1 | -0.07072 | 0.162346 | -0.43563 | 0.663107 | -0.38891 | 0.24747 | 0.173937 | Infertility, male | genitourinary | 0.178417 |
| 204.22 | 0.106237 | 0.244115 | 0.435194 | 0.663422 | -0.37222 | 0.584693 | 0.204029 | Myeloid leukemia, chronic | neoplasms | 0.17821 |
| 564 | 0.007577 | 0.01748 | 0.433449 | 0.664689 | -0.02668 | 0.041838 | 0.357969 | Functional digestive disorders | digestive | 0.177382 |
| 394 | -0.0116 | 0.026873 | -0.4315 | 0.666104 | -0.06427 | 0.041074 | 0.976328 | Rheumatic disease of the heart valves | circulatory syster | 0.176458 |
| 654 | -0.01647 | 0.038217 | -0.431 | 0.666471 | -0.09137 | 0.058432 | 0.430892 | Other and unspecified complications of birth; p | pregnancy compl | 0.176219 |
| 1.02E+03 | 0.016574 | 0.038517 | 0.430304 | 0.666975 | -0.05892 | 0.092066 | 0.191003 | other | other | 0.175891 |
| 713 | -0.07907 | 0.184165 | -0.42935 | 0.667665 | -0.44003 | 0.281885 | 0.066435 | Arthropathy associated with other disorders cl | musculoskeletal | 0.175441 |
| 204 | 0.018349 | 0.042784 | 0.42887 | 0.668018 | -0.06551 | 0.102203 | 0.277747 | Leukemia | neoplasms | 0.175212 |
| 272.13 | -0.00769 | 0.017929 | -0.42875 | 0.668108 | -0.04283 | 0.027454 | 0.502938 | Mixed hyperlipidemia | endocrine/metab | 0.175153 |
| 695.81 | 0.064563 | 0.150615 | 0.428661 | 0.66817 | -0.23064 | 0.359763 | 0.816697 | Erythema nodosum | dermatologic | 0.175113 |
| 860 | 0.02644 | 0.061898 | 0.427158 | 0.669264 | -0.09488 | 0.147759 | 0.410186 | Bone marrow or stem cell transplant | neoplasms | 0.174402 |
| 145 | 0.032964 | 0.077499 | 0.425343 | 0.670586 | -0.11893 | 0.184859 | 0.242684 | Cancer of mouth | neoplasms | 0.173545 |
| 180.3 | -0.04665 | 0.109687 | -0.42532 | 0.670603 | -0.26163 | 0.168331 | 0.018638 | Cervical intraepithelial neoplasia [CIN] [Cervic | neoplasms | 0.173535 |
| 965.2 | 0.138159 | 0.324852 | 0.425299 | 0.670619 | -0.49854 | 0.774858 | 0.379064 | Antirheumatics causing adverse effects in ther | injuries & poisoni | 0.173524 |
| 363.4 | 0.222831 | 5.25E-01 | 0.424658 | 0.671086 | -0.80562 | 1.251281 | 0.24454 | Choroidal degenerations | sense organs | 0.173222 |
| 374.1 | 0.025034 | 0.058959 | 0.424606 | 0.671124 | -0.09052 | 0.140592 | 0.959213 | Ectropion or entropion | sense organs | 0.173197 |
| 339 | -0.0165 | 0.038868 | -0.42447 | 0.671223 | -0.09268 | 0.059682 | 0.024556 | Other headache syndromes | neurological | 0.173133 |
| 601.12 | 0.064191 | 0.151237 | 0.424441 | 0.671244 | -0.23223 | 0.360611 | 0.101967 | Chronic prostatitis | genitourinary | 0.173119 |
| 795 | 0.020419 | 0.048166 | 0.423941 | 0.671609 | -0.07398 | 0.114823 | 0.486607 | Other and nonspecific abnormal cytological, hi | symptoms | 0.172884 |
| 370.31 | 0.058569 | 0.138247 | 0.423658 | 0.671815 | -0.21239 | 0.329529 | 0.299761 | Keratoconjunctivitis sicca | sense organs | 0.17275 |
| 378.1 | -0.01993 | 0.047055 | -0.42362 | 0.671842 | -0.11216 | 0.072293 | 0.583204 | Strabismus (not specified as paralytic) | sense organs | 0.172733 |
| 941 | 0.022553 | 0.053291 | 0.423207 | 0.672144 | -0.08189 | 0.127 | 0.175244 | Adverse reaction to serum or vaccine | injuries & poisoni | 0.172538 |
| 705 | 0.022693 | 0.053767 | 0.422072 | 0.672973 | -0.08269 | 0.128074 | 0.820441 | Disorders of sweat glands | dermatologic | 0.172003 |
| 596 | 0.009699 | 0.022986 | 0.421948 | 0.673063 | -0.03535 | 0.054751 | 0.270953 | Other disorders of bladder | genitourinary | 0.171944 |
| 781 | 0.008273 | 0.019623 | 0.421616 | 0.673305 | -0.03019 | 0.046734 | 0.178973 | Symptoms involving nervous and musculoskel | symptoms | 0.171788 |
| 286.9 | 0.014667 | 0.034889 | 0.420385 | 0.674204 | -0.05372 | 0.083049 | 0.72095 | Abnormal coagulation profile | hematopoietic | 0.171208 |
| 617 | -0.016 | 0.038256 | -0.41816 | 0.67583 | -0.09098 | 0.058984 | 0.173167 | Disorders secondary to childbirth, surgery, trau | genitourinary | 0.170163 |
| 291.4 | -0.02115 | 0.050585 | -0.41809 | 0.675881 | -0.12029 | 0.077995 | 0.607681 | Specific nonpsychotic mental disorders due to | mental disorders | 0.17013 |
| 727.4 | 0.019597 | 0.047052 | 0.416485 | 0.677055 | -0.07262 | 0.111817 | 0.234603 | Ganglion and cyst of synovium, tendon, and b | musculoskeletal | 0.169376 |
| 369.2 | -0.02355 | 0.056649 | -0.41577 | 0.677576 | -0.13458 | 0.087477 | 0.753476 | Eye infection, viral | sense organs | 0.169042 |
| 681.3 | 0.017652 | 0.04252 | 0.41514 | 0.678039 | -0.06569 | 0.10099 | 0.490306 | Cellulitis and abscess of arm/hand | dermatologic | 0.168745 |
| 681.5 | 0.027843 | 0.067142 | 0.414684 | 0.678373 | -0.10375 | 0.159439 | 0.030115 | Cellulitis and abscess of leg, except foot | dermatologic | 0.168531 |
| 90.3 | -0.08915 | 0.215194 | -0.41426 | 0.678681 | -0.51092 | 0.332625 | 0.425454 | Venereal diseases due to Chlamydia trachoma | infectious diseas | 0.168334 |

| phecode | beta | se | zval | pval | ci.lb | ci.ub | QEp | phenotype | category | minuslogp |
|---|---|---|---|---|---|---|---|---|---|---|
| 625 | -0.01613 | 0.039054 | -0.41315 | 0.679499 | -0.09268 | 0.060409 | 0.130815 | Pain and other symptoms associated with fem | genitourinary | 0.167811 |
| 694.3 | -0.09194 | 0.222578 | -0.41305 | 0.679567 | -0.52818 | 0.344308 | 0.182803 | Vascular disorders of skin | dermatologic | 0.167768 |
| 371.33 | 0.041804 | 0.101735 | 0.41091 | 0.681139 | -0.15759 | 0.241201 | 0.425511 | Noninfectious dermatoses of eyelid | sense organs | 0.166764 |
| 733.4 | 0.03919 | 0.095503 | 0.410353 | 0.681547 | -0.14799 | 0.226373 | 0.096635 | Aseptic necrosis of bone | musculoskeletal | 0.166504 |
| 414 | -0.01095 | 0.026758 | -0.40906 | 0.682494 | -0.06339 | 0.041499 | 0.827679 | Other forms of chronic heart disease | circulatory syster | 0.165901 |
| 386.2 | -0.01304 | 0.031919 | -0.40868 | 0.682778 | -0.07561 | 0.049516 | 0.557593 | Peripheral or central vertigo | sense organs | 0.165721 |
| 709.4 | 0.079658 | 0.195236 | 0.408009 | 0.683267 | -0.303 | 0.462314 | 0.172518 | Polymyositis | dermatologic | 0.165409 |
| 497 | 0.018811 | 0.046202 | 0.407154 | 0.683895 | -0.07174 | 0.109365 | 0.124817 | Bronchitis | respiratory | 0.16501 |
| 652 | -0.07541 | 0.185664 | -0.40614 | 0.684641 | -0.4393 | 0.28849 | 0.412416 | Malposition and malpresentation of fetus or ob | pregnancy compl | 0.164537 |
| 198.3 | -0.01761 | 0.043446 | -0.4054 | 0.685184 | -0.10277 | 0.06754 | 0.931766 | Secondary malignant neoplasm of digestive sy | neoplasms | 0.164193 |
| 696.42 | 0.026683 | 0.065948 | 0.404607 | 0.685767 | -0.10257 | 0.155939 | 0.333213 | Psoriatic arthropathy | dermatologic | 0.163824 |
| 739 | 0.024794 | 0.061315 | 0.404369 | 0.685941 | -0.09538 | 0.144968 | 0.924385 | Contracture of joint | musculoskeletal | 0.163713 |
| 255.2 | 0.017604 | 0.043665 | 0.403151 | 0.686837 | -0.06798 | 0.103187 | 0.568933 | Adrenal hypofunction | endocrine/metab | 0.163146 |
| 255.21 | 0.017604 | 0.043665 | 0.403151 | 0.686837 | -0.06798 | 0.103187 | 0.568933 | Glucocorticoid deficiency | endocrine/metab | 0.163146 |
| 433.8 | -0.01793 | 0.044592 | -0.4022 | 0.68754 | -0.10533 | 0.069464 | 0.345204 | Late effects of cerebrovascular disease | circulatory syster | 0.162702 |
| 306.1 | -0.03054 | 0.076035 | -0.40162 | 0.687966 | -0.17956 | 0.118488 | 0.234585 | Mental disorders durring/after pregnancy | mental disorders | 0.162433 |
| 478 | 0.01436 | 0.035824 | 0.400848 | 0.688532 | -0.05585 | 0.084575 | 0.962738 | Throat pain | respiratory | 0.162076 |
| 536 | -0.01162 | 0.029076 | -0.3998 | 0.689304 | -0.06861 | 0.045363 | 0.239472 | Disorders of function of stomach | digestive | 0.161589 |
| 374 | -0.00994 | 0.024975 | -0.39818 | 0.690496 | -0.05889 | 0.039005 | 0.536214 | Other disorders of eyelids | sense organs | 0.160839 |
| 608 | 0.013641 | 0.03426 | 0.398177 | 0.6905 | -0.05351 | 0.08079 | 0.739894 | Other disorders of male genital organs | genitourinary | 0.160837 |
| 586.12 | 0.043668 | 0.109789 | 0.397747 | 0.690817 | -0.17151 | 0.25885 | 0.765658 | Vesicoureteral reflux | genitourinary | 0.160637 |
| 334 | 0.039168 | 0.098481 | 0.397723 | 0.690834 | -0.15385 | 0.232188 | 0.006815 | Degenerative disease of the spinal cord | neurological | 0.160626 |
| 315.1 | 0.055034 | 0.138776 | 0.396567 | 0.691687 | -0.21696 | 0.327029 | 0.175133 | Learning disorder | mental disorders | 0.160091 |
| 471 | 0.019295 | 0.048689 | 0.396284 | 0.691895 | -0.07613 | 0.114723 | 0.968317 | Nasal polyps | respiratory | 0.15996 |
| 446.4 | 0.074209 | 0.187601 | 0.39557 | 0.692422 | -0.29348 | 0.4419 | 0.231319 | Wegener's granulomatosis | circulatory syster | 0.159629 |
| 751.11 | 0.039795 | 0.100601 | 0.395571 | 0.692422 | -0.15738 | 0.236969 | 0.899596 | Congenital anomalies of female genital organs | congenital anoma | 0.159629 |
| 528.7 | -0.06903 | 0.174598 | -0.39537 | 0.692571 | -0.41124 | 0.273175 | 0.180474 | Sialolithiasis | digestive | 0.159536 |
| 426.24 | 0.025819 | 0.065382 | 0.394901 | 0.692916 | -0.10233 | 0.153965 | 0.427039 | Atrioventricular block, complete | circulatory syster | 0.159319 |
| 580.1 | -0.02528 | 0.064097 | -0.39448 | 0.693227 | -0.15091 | 0.100343 | 0.931113 | Glomerulonephritis | genitourinary | 0.159124 |
| 293.1 | -0.00876 | 0.022204 | -0.39445 | 0.693247 | -0.05228 | 0.03476 | 0.648999 | Swelling, mass, or lump in head and neck [Spa | mental disorders | 0.159112 |
| 961 | 0.017855 | 0.045276 | 0.394348 | 0.693324 | -0.07089 | 0.106595 | 0.845194 | Poisoning by other anti-infectives | injuries & poisoni | 0.159064 |
| 772.3 | -0.01098 | 0.027867 | -0.39404 | 0.693552 | -0.0656 | 0.043638 | 0.76416 | Muscle weakness | symptoms | 0.158921 |
| 526.4 | 0.015268 | 0.038782 | 0.393698 | 0.693804 | -0.06074 | 0.09128 | 0.643724 | Temporomandibular joint disorders | digestive | 0.158763 |
| 274.1 | 0.011544 | 0.029407 | 0.392556 | 0.694647 | -0.04609 | 0.069182 | 0.732903 | Gout | endocrine/metab | 0.158236 |
| 564.8 | -0.01066 | 0.027181 | -0.39221 | 0.694905 | -0.06394 | 0.042614 | 0.753297 | Abnormal findings on exam of gastrointestinal | digestive | 0.158075 |
| 117.2 | -0.09497 | 0.242406 | -0.39177 | 0.695228 | -0.57007 | 0.380139 | 0.029144 | Coccidioidomycosis | infectious diseas | 0.157873 |
| 414.2 | -0.03011 | 0.076888 | -0.39158 | 0.695372 | -0.18081 | 0.12059 | 0.177498 | ASCVD | circulatory syster | 0.157783 |
| 622.2 | -0.03704 | 0.094769 | -0.39085 | 0.695906 | -0.22279 | 0.148704 | 0.156676 | Mucous polyp of cervix | genitourinary | 0.157449 |
| 159 | -0.01635 | 0.041852 | -0.39077 | 0.695966 | -0.09838 | 0.065674 | 0.395999 | Malignant neoplasm of other and ill-defined sit | neoplasms | 0.157412 |
| 381.3 | 0.086169 | 0.220694 | 0.390445 | 0.696207 | -0.34638 | 0.518722 | 0.001555 | Mastoiditis & related conditions | sense organs | 0.157261 |
| 655 | -0.03336 | 0.085527 | -0.3901 | 0.696462 | -0.20099 | 0.134266 | 0.001157 | Known or suspected fetal abnormality affecting | pregnancy compl | 0.157103 |
| 840.1 | 0.021266 | 0.054514 | 0.39009 | 0.69647 | -0.08558 | 0.128112 | 0.923847 | Muscle/tendon sprain | injuries & poisoni | 0.157098 |
| 79.1 | -0.01604 | 0.041371 | -0.38759 | 0.698316 | -0.09712 | 0.065051 | 0.1605 | Varicella infection | infectious diseas | 0.155948 |
| 695.8 | -0.01391 | 0.03592 | -0.38715 | 0.698644 | -0.08431 | 0.056496 | 0.405356 | Other specified erythematous conditions | dermatologic | 0.155744 |
| 323 | -0.05663 | 0.146628 | -0.38621 | 0.699345 | -0.34401 | 0.230757 | 0.044561 | Encephalitis | neurological | 0.155309 |
| 370.2 | 0.06415 | 0.166117 | 0.386173 | 0.699369 | -0.26143 | 0.389732 | 0.041639 | Superficial keratitis | sense organs | 0.155294 |
| 381.9 | -0.02746 | 0.071201 | -0.38565 | 0.699757 | -0.16701 | 0.112093 | 0.374275 | Otorrhea | sense organs | 0.155053 |
| 728.2 | -0.02925 | 0.075899 | -0.3854 | 0.699938 | -0.17801 | 0.119507 | 0.629865 | Laxity of ligament or hypermobility syndrome | musculoskeletal | 0.154941 |

| phecode | beta | se | zval | pval | ci.lb | ci.ub | QEp | phenotype | category | minuslogp |
|---|---|---|---|---|---|---|---|---|---|---|
| 723.1 | 0.047117 | 0.122478 | 0.384698 | 0.700461 | -0.19294 | 0.28717 | 0.100728 | Torticollis | musculoskeletal | 0.154616 |
| 375 | 0.018268 | 0.047571 | 0.384016 | 0.700967 | -0.07497 | 0.111506 | 0.759864 | Disorders of lacrimal system | sense organs | 0.154302 |
| 755.61 | -0.12445 | 0.324593 | -0.38342 | 0.701411 | -0.76064 | 0.511736 | 0.017459 | Congenital hip dysplasia and deformity | congenital anoma | 0.154027 |
| 301.2 | -0.05551 | 0.145105 | -0.38255 | 0.702053 | -0.33991 | 0.228891 | 0.320581 | Antisocial/borderline personality disorder | mental disorders | 0.15363 |
| 520.1 | 0.096738 | 0.253738 | 0.38125 | 0.703018 | -0.40058 | 0.594056 | 0.532748 | Hereditary disturbances in tooth structure | digestive | 0.153034 |
| 701.5 | -0.02089 | 0.054824 | -0.38103 | 0.703182 | -0.12834 | 0.086563 | 0.987086 | Abnormal granulation tissue | dermatologic | 0.152932 |
| 710.2 | 0.346915 | 0.91128 | 0.380689 | 0.703434 | -1.43916 | 2.13299 | 0.046867 | Periostitis | musculoskeletal | 0.152777 |
| 790.8 | -0.0209 | 0.055134 | -0.37914 | 0.704581 | -0.12896 | 0.087157 | 0.64933 | Elevated C-reactive protein (CRP) | symptoms | 0.152069 |
| 362.9 | 0.041726 | 0.110323 | 0.378214 | 0.705272 | -0.1745 | 0.257954 | 0.03535 | Retinal edema | sense organs | 0.151643 |
| 498 | -0.02056 | 0.054624 | -0.37635 | 0.706655 | -0.12762 | 0.086503 | 0.898398 | Acute bronchospasm | respiratory | 0.150793 |
| 394.3 | 0.080617 | 0.214399 | 0.376014 | 0.706906 | -0.3396 | 0.500831 | 0.098609 | Aortic valve disease | circulatory syster | 0.150638 |
| 473.1 | 0.029357 | 0.078869 | 0.372231 | 0.709721 | -0.12522 | 0.183938 | 0.508754 | Chronic laryngitis | respiratory | 0.148912 |
| 643.1 | -0.07609 | 0.204797 | -0.37155 | 0.71023 | -0.47749 | 0.325303 | 0.045357 | Hyperemesis gravidarum | pregnancy compl | 0.148601 |
| 871.4 | -0.03055 | 0.083119 | -0.36754 | 0.713213 | -0.19346 | 0.13236 | 0.698155 | Open wound of toe(s) | injuries & poisoni | 0.146781 |
| 723 | 0.012853 | 0.034992 | 0.367309 | 0.713388 | -0.05573 | 0.081435 | 0.385446 | Other disorders of cervical region | musculoskeletal | 0.146674 |
| 110.2 | -0.01913 | 0.052126 | -0.36703 | 0.713596 | -0.1213 | 0.083033 | 0.984003 | Dermatomycoses | infectious diseas | 0.146547 |
| 689 | -0.00589 | 0.01621 | -0.36331 | 0.71637 | -0.03766 | 0.025881 | 0.688823 | Disorder of skin and subcutaneous tissue NOS | dermatologic | 0.144863 |
| 258.1 | -0.03884 | 0.107344 | -0.36179 | 0.717509 | -0.24923 | 0.171555 | 0.844416 | Postablative ovarian failure | endocrine/metab | 0.144173 |
| 735.21 | 0.015319 | 0.042474 | 0.360675 | 0.718343 | -0.06793 | 0.098567 | 0.755301 | Hammer toe (acquired) | musculoskeletal | 0.143668 |
| 591 | 0.005809 | 0.016195 | 0.358699 | 0.719821 | -0.02593 | 0.03755 | 0.670846 | Urinary tract infection | genitourinary | 0.142776 |
| 287.32 | -0.01314 | 0.037079 | -0.35438 | 0.723055 | -0.08581 | 0.059534 | 0.64861 | Secondary thrombocytopenia | hematopoietic | 0.140829 |
| 586.3 | 0.040208 | 0.113504 | 0.35424 | 0.723159 | -0.18226 | 0.262672 | 0.478151 | Vascular disorders of kidney/hypertrophy | genitourinary | 0.140766 |
| 741.4 | 0.006851 | 0.019434 | 0.352523 | 0.724446 | -0.03124 | 0.044941 | 0.333987 | Joint effusions | musculoskeletal | 0.139994 |
| 610 | 0.022878 | 0.065043 | 0.351742 | 0.725032 | -0.1046 | 0.15036 | 0.022608 | Benign mammary dysplasias | genitourinary | 0.139643 |
| 451.2 | 0.037089 | 0.105643 | 0.351082 | 0.725527 | -0.16997 | 0.244146 | 0.129225 | Phlebitis and thrombophlebitis of lower extrem | circulatory syster | 0.139346 |
| 270.31 | 0.187118 | 0.533611 | 0.350664 | 0.72584 | -0.85874 | 1.232976 | 0.001674 | Polyclonal hypergammaglobulinemia | endocrine/metab | 0.139159 |
| 802 | -0.0813 | 0.231993 | -0.35046 | 0.725992 | -0.536 | 0.373393 | 0.021978 | Fracture of pelvis | injuries & poisoni | 0.139068 |
| 771 | -0.00794 | 0.022787 | -0.34832 | 0.727596 | -0.0526 | 0.036724 | 0.986031 | Musculoskeletal symptoms referable to limbs | symptoms | 0.138109 |
| 149.1 | 0.029142 | 0.083708 | 0.348136 | 0.727738 | -0.13492 | 0.193207 | 0.892935 | Cancer of oropharynx | neoplasms | 0.138025 |
| 221 | -0.05112 | 0.147096 | -0.34754 | 0.728188 | -0.33942 | 0.237182 | 0.968362 | Benign neoplasm of other female genital orgar | neoplasms | 0.137756 |
| 800.1 | 0.01665 | 0.047989 | 0.346961 | 0.72862 | -0.07741 | 0.110708 | 0.650303 | Fracture of neck of femur | injuries & poisoni | 0.137499 |
| 264.3 | -0.23392 | 0.676207 | -0.34593 | 0.729395 | -1.55926 | 1.091422 | 0.147184 | Delayed milestones | endocrine/metab | 0.137037 |
| 198.2 | -0.02194 | 0.063552 | -0.34529 | 0.729879 | -0.1465 | 0.102616 | 0.177756 | Secondary malignancy of respiratory organs | neoplasms | 0.136749 |
| 131 | 0.077478 | 0.226286 | 0.342391 | 0.732057 | -0.36603 | 0.520991 | 0.155322 | Protozoan infection | infectious diseas | 0.135455 |
| 172.1 | 0.120715 | 0.35332 | 0.341659 | 0.732608 | -0.57178 | 0.81321 | 0.005055 | Melanomas of skin, dx or hx | neoplasms | 0.135129 |
| 619.1 | -0.01317 | 0.038569 | -0.34141 | 0.732792 | -0.08876 | 0.062425 | 0.757665 | Noninflammatory disorders of ovary, fallopian t | genitourinary | 0.135019 |
| 772 | 0.016988 | 0.049917 | 0.340326 | 0.733611 | -0.08085 | 0.114824 | 0.176705 | Symptoms of the muscles | symptoms | 0.134534 |
| 315.2 | -0.05314 | 0.156182 | -0.34026 | 0.733658 | -0.35926 | 0.252969 | 0.062912 | Speech and language disorder | mental disorders | 0.134506 |
| 594.8 | 0.027942 | 0.082154 | 0.340117 | 0.733768 | -0.13308 | 0.188961 | 0.423125 | Renal colic | genitourinary | 0.134441 |
| 389.1 | -0.01702 | 0.050998 | -0.33383 | 0.738507 | -0.11698 | 0.082929 | 0.020936 | Sensorineural hearing loss | sense organs | 0.131646 |
| 359 | 0.015558 | 0.04668 | 0.333294 | 0.738912 | -0.07593 | 0.107049 | 0.464738 | Muscular dystrophies and other myopathies | neurological | 0.131407 |
| 614.4 | -0.03528 | 0.105989 | -0.33291 | 0.739205 | -0.24302 | 0.172451 | 0.180308 | Inflammatory diseases of uterus, except cervix | genitourinary | 0.131235 |
| 394.4 | -0.16358 | 0.491869 | -0.33257 | 0.739456 | -1.12763 | 0.800462 | 0.006225 | Acute rheumatic heart disease | circulatory syster | 0.131088 |
| 635.3 | -0.02377 | 0.071744 | -0.33138 | 0.740359 | -0.16439 | 0.116841 | 0.790994 | Placenta previa and abruptio placenta | pregnancy compl | 0.130558 |
| 711 | 0.023877 | 0.072519 | 0.329251 | 0.741966 | -0.11826 | 0.166012 | 0.445302 | Arthropathy associated with infections | musculoskeletal | 0.129616 |
| 41.2 | 0.014281 | 0.0436 | 3.28E-01 | 0.743263 | -0.07117 | 0.099736 | 0.218071 | Streptococcus infection | infectious diseas | 0.128857 |
| 429.2 | 0.0104 | 0.031817 | 0.32686 | 0.743774 | -0.05196 | 0.07276 | 0.570531 | Abnormal function study of cardiovascular sys | circulatory syster | 0.128559 |
| 370 | -0.02864 | 0.08766 | -0.32672 | 0.743878 | -0.20045 | 0.14317 | 0.05441 | Keratitis | sense organs | 0.128498 |

| phecode | beta | se | zval | pval | ci.lb | ci.ub | QEp | phenotype | category | minuslogp |
|---|---|---|---|---|---|---|---|---|---|---|
| 172.11 | 0.14948 | 0.460296 | 0.324749 | 0.745371 | -0.75268 | 1.051643 | 0.003997 | Melanomas of skin | neoplasms | 0.127627 |
| 519.9 | -0.00848 | 0.026156 | -0.32411 | 0.745854 | -0.05974 | 0.042788 | 0.778001 | Symptoms involving respiratory system and ot | respiratory | 0.127346 |
| 212 | -0.05243 | 0.161886 | -0.32387 | 0.746038 | -0.36972 | 0.264861 | 0.056981 | Benign neoplasm of respiratory and intrathora | neoplasms | 0.127239 |
| 857 | -0.01589 | 0.049184 | -0.3231 | 0.746618 | -0.11229 | 0.080507 | 0.542369 | Mechanical complication of unspecified genito | injuries & poisoni | 0.126902 |
| 202.24 | -0.04925 | 0.153463 | -0.3209 | 0.748288 | -0.35003 | 0.251537 | 0.529535 | Large cell lymphoma | neoplasms | 0.125931 |
| 609 | 0.043308 | 0.135544 | 0.319511 | 0.749339 | -0.22235 | 0.308969 | 0.222422 | Male infertility and abnormal spermatozoa | genitourinary | 0.125322 |
| 621 | -0.08441 | 0.265739 | -0.31764 | 0.750755 | -0.60525 | 0.436429 | 0.000186 | Endometrial hyperplasia | genitourinary | 0.124501 |
| 610.1 | -0.01362 | 0.042993 | -0.31685 | 0.751361 | -0.09789 | 0.070642 | 0.249021 | Cystic mastopathy | genitourinary | 0.124151 |
| 364.1 | -0.03482 | 0.110595 | -0.31486 | 0.752864 | -0.25159 | 0.18194 | 0.244532 | Corneal opacity | sense organs | 0.123283 |
| 286.1 | -0.04588 | 0.146036 | -0.31418 | 0.753383 | -0.33211 | 0.240343 | 0.104131 | Congenital coagulation defects | hematopoietic | 0.122984 |
| 733 | -0.00759 | 0.024222 | -0.31346 | 0.753931 | -0.05507 | 0.039882 | 0.36577 | Other disorders of bone and cartilage | musculoskeletal | 0.122668 |
| 704.8 | 0.01164 | 0.037146 | 0.313368 | 0.754001 | -0.06116 | 0.084445 | 0.324679 | Other specified diseases of hair and hair follicl | dermatologic | 0.122628 |
| 297.2 | -0.05714 | 0.183095 | -0.31207 | 0.75499 | -0.416 | 0.301722 | 0.146247 | Suicide or self-inflicted injury | mental disorders | 0.122059 |
| 728.71 | 0.050809 | 0.163042 | 0.311634 | 0.755319 | -0.26875 | 0.370365 | 0.060612 | Contracture of palmar fascia [Dupuytren's dise | musculoskeletal | 0.12187 |
| 245.21 | 0.013571 | 0.043762 | 0.310098 | 0.756486 | -0.0722 | 0.099343 | 0.45995 | Chronic lymphocytic thyroiditis | endocrine/metab | 0.121199 |
| 297.1 | 0.017348 | 0.056084 | 0.309314 | 0.757083 | -0.09257 | 0.12727 | 0.731081 | Suicidal ideation | mental disorders | 0.120857 |
| 253 | -0.01016 | 0.032897 | -0.3087 | 0.757548 | -0.07463 | 0.054322 | 0.680296 | Disorders of the pituitary gland and its hypotha | endocrine/metab | 0.12059 |
| 559 | 0.032544 | 0.105545 | 0.308342 | 0.757822 | -0.17432 | 0.239407 | 0.171111 | Ileostomy status | digestive | 0.120433 |
| 134.1 | 0.038174 | 0.124238 | 0.307264 | 0.758642 | -0.20533 | 0.281676 | 0.744974 | Intestinal helminthiases | infectious diseas | 0.119963 |
| 443.1 | -0.01302 | 0.04238 | -0.30714 | 0.758736 | -0.09608 | 0.070047 | 0.64366 | Raynaud's syndrome | circulatory syster | 0.119909 |
| 442.3 | -0.04161 | 0.135542 | -0.30701 | 0.758833 | -0.30727 | 0.224044 | 0.314514 | Aneurysm of artery of lower extremity | circulatory syster | 0.119854 |
| 458.1 | 0.009737 | 0.031804 | 0.306155 | 0.759487 | -0.0526 | 0.072073 | 0.430554 | Orthostatic hypotension | circulatory syster | 0.11948 |
| 41.12 | -0.03118 | 0.101869 | -3.06E-01 | 0.759521 | -0.23084 | 0.168477 | 0.062208 | Methicillin resistant Staphylococcus aureus | infectious diseas | 0.11946 |
| 332 | -0.06219 | 0.20333 | -0.30586 | 0.75971 | -0.46071 | 0.336329 | 0.029858 | Parkinson's disease | neurological | 0.119352 |
| 647 | -0.03924 | 0.128751 | -0.30479 | 0.760523 | -0.29159 | 0.213104 | 0.023833 | Infectious and parasitic complications affecting | pregnancy compl | 0.118888 |
| 800.2 | -0.05896 | 0.193789 | -0.30422 | 0.760957 | -0.43877 | 0.320864 | 0.040372 | Fracture of unspecified part of femur | injuries & poisoni | 0.11864 |
| 573.7 | -0.00828 | 0.027244 | -0.304 | 0.761125 | -0.06168 | 0.045114 | 0.734935 | Abnormal results of function study of liver | digestive | 0.118544 |
| 798.1 | 0.009316 | 0.030681 | 0.303643 | 0.7614 | -0.05082 | 0.06945 | 0.784064 | Chronic fatigue syndrome | symptoms | 0.118387 |
| 81.1 | 0.042312 | 0.140544 | 0.301057 | 0.763371 | -0.23315 | 0.317773 | 0.262192 | Graft-versus-host disease | infectious diseas | 0.117264 |
| 442.4 | 0.054234 | 0.180825 | 0.299927 | 0.764233 | -0.30018 | 0.408646 | 0.032862 | Arterial dissection | circulatory syster | 0.116774 |
| 198 | -0.01099 | 0.036844 | -0.29823 | 0.765531 | -0.0832 | 0.061224 | 0.137141 | Secondary malignant neoplasm | neoplasms | 0.116037 |
| 590 | 0.019636 | 0.065891 | 0.298003 | 0.765701 | -0.10951 | 0.148781 | 0.082376 | Pyelonephritis | genitourinary | 0.115941 |
| 741.1 | 0.044785 | 0.15058 | 0.297416 | 0.766149 | -0.25035 | 0.339916 | 0.495368 | Ankylosis of joint | musculoskeletal | 0.115687 |
| 372 | 0.011075 | 0.037348 | 0.296536 | 0.766821 | -0.06212 | 0.084275 | 0.199316 | Disorders of conjunctiva | sense organs | 0.115306 |
| 187.1 | 0.181738 | 0.613132 | 0.29641 | 0.766917 | -1.01998 | 1.383456 | 0.001098 | Malignant neoplasm of unspecified male genit | neoplasms | 0.115251 |
| 371.1 | 0.019012 | 0.064904 | 0.292927 | 0.769578 | -0.1082 | 0.146222 | 0.929419 | Uveitis, noninfectious or NOS | sense organs | 0.113748 |
| 170.2 | -0.01757 | 0.060068 | -0.29253 | 0.76988 | -0.1353 | 0.100159 | 0.353622 | Cancer of connective tissue | neoplasms | 0.113577 |
| 282.9 | 0.028182 | 0.097014 | 0.29049 | 0.771441 | -0.16196 | 0.218327 | 0.142841 | Other hereditary hemolytic anemias | hematopoietic | 0.112697 |
| 275.1 | -0.04693 | 0.1617 | -0.29023 | 0.771639 | -0.36386 | 0.269995 | 0.027319 | Disorders of iron metabolism | hematopoietic | 0.112586 |
| 259.4 | -0.14153 | 0.492118 | -0.2876 | 0.773654 | -1.10607 | 0.823001 | 0.218494 | Precocious sexual development and puberty N | endocrine/metab | 0.111453 |
| 742 | -0.0092 | 0.032091 | -0.28679 | 0.774273 | -0.0721 | 0.053694 | 0.837187 | Derangement of joint, non-traumatic | musculoskeletal | 0.111106 |
| 656.3 | -0.04532 | 0.158439 | -0.28601 | 0.774871 | -0.35585 | 0.26522 | 0.640236 | Endocrine and metabolic disturbances of fetus | pregnancy compl | 0.110771 |
| 287.2 | 0.117769 | 0.412011 | 0.285839 | 0.775001 | -0.68976 | 0.925295 | 0.851783 | Allergic purpura | hematopoietic | 0.110698 |
| 362.8 | -0.02343 | 0.082062 | -0.28554 | 0.775229 | -0.18427 | 0.137407 | 0.762419 | Retinal hemorrhage/ischemia | sense organs | 0.11057 |
| 79.2 | -0.03465 | 0.121985 | -0.28402 | 0.776398 | -0.27373 | 0.20444 | 0.208547 | Infectious mononucleosis | infectious diseas | 0.109915 |
| 974 | 0.070099 | 0.24972 | 0.280709 | 0.778934 | -0.41934 | 0.559541 | 0.088486 | Poisoning by water, mineral, and uric acid met | injuries & poisoni | 0.1085 |
| 579.8 | -0.01228 | 0.04386 | -0.2799 | 0.779553 | -0.09824 | 0.073687 | 0.161165 | Nonspecific abnormal findings in stool content | digestive | 0.108155 |
| 638 | 0.013018 | 0.046848 | 0.277875 | 0.781108 | -0.0788 | 0.104838 | 0.439407 | Other high-risk pregnancy | pregnancy compl | 0.107289 |

| phecode | beta | se | zval | pval | ci.lb | ci.ub | QEp | phenotype | category | minuslogp |
|---|---|---|---|---|---|---|---|---|---|---|
| 965 | 0.015585 | 0.056264 | 0.277003 | 0.781778 | -0.09469 | 0.12586 | 0.051867 | Poisoning by analgesics, antipyretics, and anti | injuries & poisoni | 0.106916 |
| 742.2 | -0.04854 | 0.176859 | -0.27443 | 0.783755 | -0.39517 | 0.298101 | 0.318254 | Pathological, developmental or recurrent disloc | musculoskeletal | 0.10582 |
| 535.6 | -0.0154 | 0.056723 | -0.27157 | 0.785953 | -0.12658 | 0.095771 | 0.863424 | Duodenitis | digestive | 0.104604 |
| 356 | 0.007612 | 0.028048 | 0.271383 | 0.786096 | -0.04736 | 0.062585 | 0.983103 | Hereditary and idiopathic peripheral neuropath | neurological | 0.104524 |
| 275.51 | 0.012841 | 0.047449 | 0.270622 | 0.786682 | -0.08016 | 0.105839 | 0.192353 | Hypocalcemia | endocrine/metab | 0.104201 |
| 286.3 | -0.01987 | 0.073511 | -0.2703 | 0.786927 | -0.16395 | 0.124209 | 0.747673 | Coagulation defects complicating pregnancy o | hematopoietic | 0.104066 |
| 340.1 | -0.01401 | 0.051856 | -0.27024 | 0.786977 | -0.11565 | 0.087623 | 0.208501 | Migrain with aura | neurological | 0.104038 |
| 313 | 0.009322 | 0.034536 | 0.26993 | 0.787214 | -0.05837 | 0.077013 | 0.538353 | Pervasive developmental disorders | mental disorders | 0.103907 |
| 603.2 | 0.021019 | 0.07811 | 0.269099 | 0.787854 | -0.13207 | 0.174112 | 0.554039 | Spermatocele | genitourinary | 0.103554 |
| 1001 | 0.011089 | 0.041249 | 0.268826 | 0.788063 | -0.06976 | 0.091935 | 0.92885 | Foreign body injury | other | 0.103439 |
| 971 | 0.092251 | 0.343332 | 0.268695 | 0.788164 | -0.58067 | 0.765169 | 0.140222 | Poisoning by drugs primarily affecting the auto | injuries & poisoni | 0.103383 |
| 555.2 | 0.010196 | 0.037974 | 0.268514 | 0.788304 | -0.06423 | 0.084623 | 0.927496 | Ulcerative colitis | digestive | 0.103306 |
| 290.3 | -0.01121 | 0.042017 | -0.2669 | 0.789548 | -0.09357 | 0.071138 | 0.773894 | Other persistent mental disorders due to condi | mental disorders | 0.102622 |
| 184.2 | 0.02498 | 0.093599 | 0.266886 | 0.789557 | -0.15847 | 0.208431 | 0.8268 | Cancer of other female genital organs (excludi | neoplasms | 0.102617 |
| 295 | 0.013252 | 0.049875 | 0.265711 | 0.790462 | -0.0845 | 0.111005 | 0.405568 | Schizophrenia and other psychotic disorders | mental disorders | 0.102119 |
| 458.2 | -0.01071 | 0.040526 | -0.26428 | 0.791562 | -0.09014 | 0.068719 | 0.989732 | Iatrogenic hypotension | circulatory syster | 0.101515 |
| 441.1 | 0.02921 | 0.110697 | 0.263871 | 0.791879 | -0.18775 | 0.246171 | 0.628176 | Acute vascular insufficiency of intestine | circulatory syster | 0.101341 |
| 530.9 | -0.01152 | 0.043647 | -0.26383 | 0.791911 | -0.09706 | 0.07403 | 0.413703 | Heartburn | digestive | 0.101324 |
| 661 | -0.03377 | 0.128125 | -0.26355 | 0.792124 | -0.28489 | 0.217352 | 0.010429 | Fetal distress and abnormal forces of labor | pregnancy compl | 0.101207 |
| 1000 | 0.024913 | 0.09504 | 0.262129 | 0.793222 | -0.16136 | 0.211189 | 0.19209 | Burns | other | 0.100605 |
| 272.14 | 0.068824 | 0.267989 | 0.256818 | 0.79732 | -0.45642 | 0.594072 | 0.212051 | Hyperchylomicronemia | endocrine/metab | 0.098368 |
| 289.4 | 0.005993 | 0.023403 | 0.256078 | 0.79789 | -0.03988 | 0.051863 | 0.313114 | Lymphadenitis | hematopoietic | 0.098057 |
| 686.5 | -0.026 | 0.101694 | -0.25568 | 0.798195 | -0.22532 | 0.173315 | 0.29488 | Pyoderma | dermatologic | 0.097891 |
| 184.1 | 0.020267 | 0.079886 | 0.253697 | 0.79973 | -0.13631 | 0.176841 | 0.191976 | Malignant neoplasm of ovary and other uterine | neoplasms | 0.097057 |
| 758 | 0.025774 | 0.102079 | 0.252495 | 0.800658 | -0.1743 | 0.225845 | 0.202294 | Chromosomal anomalies and genetic disorder | congenital anoma | 0.096553 |
| 597.1 | -0.01837 | 0.072798 | -0.25241 | 0.800728 | -0.16106 | 0.124306 | 0.801671 | Urethral stricture (not specified as infectious) | genitourinary | 0.096515 |
| 465 | -0.00429 | 0.017068 | -0.2511 | 0.801734 | -0.03774 | 2.92E-02 | 0.483434 | Acute upper respiratory infections of multiple c | respiratory | 0.09597 |
| 379.5 | 0.020012 | 0.079705 | 0.251074 | 0.801757 | -0.13621 | 0.17623 | 0.334743 | Disorders of iris and ciliary body | sense organs | 0.095957 |
| 368.9 | 0.00997 | 0.039757 | 0.250784 | 0.801981 | -0.06795 | 0.087893 | 0.600674 | Subjective visual disturbances | sense organs | 0.095836 |
| 286.8 | -0.00981 | 0.039114 | -0.25077 | 0.801993 | -0.08647 | 0.066853 | 0.83741 | Hypercoagulable state | hematopoietic | 0.095829 |
| 624.2 | 0.081173 | 0.324667 | 0.250018 | 0.802574 | -0.55516 | 0.717509 | 0.135474 | Atrophy of female genital tract | genitourinary | 0.095515 |
| 350.1 | 0.00786 | 0.031713 | 0.24785 | 0.80425 | -0.0543 | 0.070016 | 0.259224 | Abnormal involuntary movements | neurological | 0.094609 |
| 639 | 0.111088 | 0.451512 | 0.246036 | 0.805654 | -0.77386 | 0.996036 | 0.962259 | Complications following abortion or ectopic an | pregnancy compl | 0.093851 |
| 601.4 | 0.023637 | 0.096082 | 0.246008 | 0.805676 | -0.16468 | 0.211953 | 0.181896 | Balanoposthitis | genitourinary | 0.09384 |
| 384 | 0.018756 | 0.076305 | 0.245801 | 0.805836 | -0.1308 | 0.16831 | 0.499156 | Other disorders of tympanic membrane | sense organs | 0.093753 |
| 687.1 | 0.005981 | 0.024397 | 0.245174 | 0.806322 | -0.04184 | 0.053799 | 0.196077 | Rash and other nonspecific skin eruption | dermatologic | 0.093492 |
| 585.2 | 0.010166 | 0.041656 | 0.244057 | 0.807186 | -0.07148 | 0.091811 | 0.622691 | Renal failure NOS | genitourinary | 0.093026 |
| 706.8 | 0.013266 | 0.05457 | 0.243105 | 0.807924 | -0.09369 | 0.120221 | 0.06999 | Other specified diseases of sebaceous glands | dermatologic | 0.092629 |
| 453 | 0.014587 | 0.060386 | 0.241559 | 0.809122 | -0.10377 | 0.132942 | 0.322699 | Chronic venous hypertension | circulatory syster | 0.091986 |
| 756 | -0.0129 | 0.053891 | -0.23939 | 0.810805 | -0.11853 | 0.092723 | 0.244548 | Other congenital musculoskeletal anomalies | congenital anoma | 0.091084 |
| 555 | -0.00788 | 0.032919 | -0.23935 | 0.810831 | -0.0724 | 0.056641 | 0.967721 | Inflammatory bowel disease and other gastroe | digestive | 0.09107 |
| 303.3 | 0.010684 | 0.044777 | 0.238602 | 0.811415 | -0.07708 | 0.098445 | 0.630975 | Psychogenic disorder | mental disorders | 0.090757 |
| 368.3 | -0.04196 | 0.176017 | -0.23841 | 0.81156 | -0.38695 | 0.303023 | 0.10451 | Anisometropia | sense organs | 0.090679 |
| 277.51 | 0.013982 | 0.05937 | 0.235503 | 0.813818 | -0.10238 | 0.130346 | 0.589135 | Lipoprotein disorders | endocrine/metab | 0.089473 |
| 613 | -0.00817 | 0.034703 | -0.23534 | 0.813944 | -0.07618 | 0.059849 | 0.210784 | Other nonmalignant breast conditions | genitourinary | 0.089405 |
| 656 | 0.010632 | 0.045267 | 0.234871 | 0.814309 | -0.07809 | 0.099353 | 0.250523 | Other perinatal conditions of fetus or newborn | pregnancy compl | 0.089211 |
| 710.11 | 0.016643 | 0.071174 | 0.233838 | 0.81511 | -0.12286 | 0.156143 | 0.471496 | Acute osteomyelitis | musculoskeletal | 0.088784 |
| 271 | -0.00461 | 0.019813 | -0.23255 | 0.816111 | -0.04344 | 0.034225 | 0.319723 | Disorders of carbohydrate transport and metal | endocrine/metab | 0.088251 |

| phecode | beta | se | zval | pval | ci.lb | ci.ub | QEp | phenotype | category | minuslogp |
|---|---|---|---|---|---|---|---|---|---|---|
| 430.1 | 0.023832 | 0.102514 | 0.232474 | 0.81617 | -0.17709 | 0.224756 | 0.146177 | Subarachnoid hemorrhage | circulatory syster | 0.088219 |
| 317.11 | 0.034034 | 0.14664 | 0.232093 | 0.816466 | -0.25337 | 0.321443 | 0.007176 | Alcoholic liver damage | mental disorders | 0.088062 |
| 444.1 | 0.015638 | 0.0675 | 0.231668 | 0.816796 | -0.11666 | 0.147936 | 0.885686 | Arterial embolism and thrombosis of lower ext | circulatory syster | 0.087886 |
| 524 | -0.06273 | 0.272109 | -0.23053 | 0.817678 | -0.59605 | 0.470593 | 0.029203 | Dentofacial anomalies, including malocclusion | digestive | 0.087418 |
| 870 | 0.00544 | 0.023686 | 0.229659 | 0.818357 | -0.04098 | 0.051862 | 0.382971 | Open wounds of head; neck; and trunk | injuries & poisoni | 0.087057 |
| 198.5 | -0.01244 | 0.054729 | -0.22738 | 0.820125 | -0.11971 | 0.094822 | 0.85515 | Secondary malignancy of brain/spine | neoplasms | 0.08612 |
| 271.3 | -0.00432 | 0.019016 | -0.22729 | 0.820197 | -0.04159 | 0.032948 | 0.342139 | Intestinal disaccharidase deficiencies and disa | endocrine/metab | 0.086082 |
| 871 | 0.005335 | 0.023527 | 0.226766 | 0.820606 | -0.04078 | 0.051446 | 0.561298 | Open wounds of extremities | injuries & poisoni | 0.085865 |
| 709.2 | 0.00942 | 0.041793 | 0.225402 | 0.821667 | -0.07249 | 0.091332 | 0.306599 | Sicca syndrome | dermatologic | 0.085304 |
| 973 | 0.076639 | 0.342832 | 0.223547 | 0.82311 | -0.5953 | 0.748576 | 0.745552 | Poisoning by agents primarily affecting the gas | injuries & poisoni | 0.084542 |
| 557.1 | -0.03649 | 0.16362 | -0.223 | 0.823532 | -0.35718 | 0.284202 | 0.111734 | Celiac disease | digestive | 0.084319 |
| 250.11 | -0.03022 | 0.136328 | -0.22167 | 0.824574 | -0.29742 | 0.236978 | 0.959996 | Type 1 diabetes with ketoacidosis | endocrine/metab | 0.08377 |
| 711.1 | 0.016343 | 0.073956 | 0.220988 | 0.825102 | -0.12861 | 0.161294 | 0.446192 | Pyogenic arthritis | musculoskeletal | 0.083492 |
| 327.6 | 0.014192 | 0.06447 | 0.220129 | 0.825771 | -0.11217 | 0.140551 | 0.279381 | Circadian rhythm sleep disorder | neurological | 0.083141 |
| 686.3 | -0.03607 | 0.165081 | -0.21847 | 0.827062 | -0.35962 | 0.287487 | 0.201264 | Pilonidal cyst | dermatologic | 0.082462 |
| 626.14 | -0.01149 | 0.052759 | -0.21778 | 0.827604 | -0.1149 | 0.091916 | 0.292973 | Irregular menstrual bleeding | genitourinary | 0.082178 |
| 750.15 | 0.066145 | 0.30434 | 0.217339 | 0.827944 | -0.53035 | 0.662641 | 0.013287 | Congenital anomalies of stomach | congenital anoma | 0.081999 |
| 967 | 0.010201 | 0.04705 | 0.216809 | 0.828357 | -0.08201 | 0.102416 | 0.538374 | Adverse effects of sedatives or other central n | injuries & poisoni | 0.081782 |
| 245.2 | 0.009197 | 0.04249 | 0.216455 | 0.828633 | -0.07408 | 0.092476 | 0.481952 | Chronic thyroiditis | endocrine/metab | 0.081638 |
| 149 | -0.02326 | 0.107711 | -0.21596 | 0.829021 | -0.23437 | 0.187849 | 0.058846 | Cancer of larynx, pharynx, nasal cavities | neoplasms | 0.081435 |
| 594.2 | -0.01373 | 0.063607 | -0.21591 | 0.829054 | -0.1384 | 0.110934 | 0.610088 | Calculus of lower urinary tract | genitourinary | 0.081417 |
| 366.2 | 0.007684 | 0.035653 | 0.215518 | 0.829363 | -0.0622 | 0.077563 | 0.067388 | Senile cataract | sense organs | 0.081255 |
| 722.7 | -0.01466 | 0.068959 | -0.2126 | 0.831641 | -0.14982 | 0.120497 | 0.573669 | Intervertebral disc disorder with myelopathy | musculoskeletal | 0.080064 |
| 430.3 | -0.0217 | 0.102168 | -0.21244 | 0.831762 | -0.22195 | 0.178541 | 0.095874 | Subdural hemorrhage | circulatory syster | 0.080001 |
| 958 | 0.0161 | 0.075995 | 0.211856 | 0.832219 | -0.13285 | 0.165047 | 0.658455 | Certain early complications of trauma or proce | injuries & poisoni | 0.079762 |
| 643 | 0.033234 | 0.158066 | 0.210257 | 0.833467 | -0.27657 | 0.343038 | 0.002489 | Excessive vomiting in pregnancy | pregnancy compl | 0.079111 |
| 304 | -0.00462 | 0.02208 | -0.20914 | 0.834341 | -0.04789 | 0.038659 | 0.60128 | Adjustment reaction | mental disorders | 0.078657 |
| 388 | -0.01207 | 0.058094 | -0.2077 | 0.835466 | -0.12593 | 0.101797 | 0.036199 | Other disorders of ear | sense organs | 0.078071 |
| 430 | -0.00907 | 0.043828 | -0.20702 | 0.835993 | -0.09497 | 0.076828 | 0.553047 | Intracranial hemorrhage | circulatory syster | 0.077797 |
| 446.2 | 0.092378 | 0.44675 | 0.206778 | 0.836183 | -0.78324 | 0.967993 | 0.147789 | Acute febrile mucocutaneous lymph node sync | circulatory syster | 0.077699 |
| 281.13 | 0.027438 | 0.132705 | 0.206762 | 0.836196 | -0.23266 | 0.287536 | 0.321185 | Folate-deficiency anemia | hematopoietic | 0.077692 |
| 395.2 | -0.00894 | 0.043304 | -0.2065 | 0.836398 | -0.09382 | 0.075931 | 0.148533 | Nonrheumatic aortic valve disorders | circulatory syster | 0.077587 |
| 224 | -0.02434 | 0.118095 | -0.2061 | 0.836711 | -0.2558 | 0.207122 | 0.119472 | Benign neoplasm of eye | neoplasms | 0.077424 |
| 427.7 | 0.004189 | 0.020337 | 0.205997 | 0.836793 | -0.03567 | 0.044049 | 0.815783 | Tachycardia NOS | circulatory syster | 0.077382 |
| 433.11 | 0.014342 | 0.069906 | 0.205162 | 0.837445 | -0.12267 | 0.151355 | 0.361716 | Occlusion of cerebral arteries, with cerebral in | circulatory syster | 0.077044 |
| 447 | -0.00515 | 0.025206 | -0.2042 | 0.8382 | -0.05455 | 0.044256 | 0.722689 | Other disorders of arteries and arterioles | circulatory syster | 0.076652 |
| 753.2 | 0.027701 | 0.135951 | 0.203757 | 0.838544 | -0.23876 | 0.294161 | 0.995976 | Congenital anomalies of posterior segment of | congenital anoma | 0.076474 |
| 737.1 | -0.03222 | 0.159018 | -0.2026 | 0.839448 | -0.34389 | 0.279453 | 0.032681 | Kyphosis (acquired) | musculoskeletal | 0.076006 |
| 750.11 | -0.04678 | 0.231391 | -0.20216 | 0.83979 | -0.5003 | 0.406739 | 0.021383 | Esophageal atresia/tracheoesophageal fistula | congenital anoma | 0.075829 |
| 427.1 | -0.0043 | 0.021402 | -0.20069 | 0.840942 | -0.04624 | 0.037652 | 0.812972 | Paroxysmal tachycardia, unspecified | circulatory syster | 0.075234 |
| 789 | 0.002829 | 0.014172 | 0.19958 | 0.841809 | -0.02495 | 0.030606 | 0.316984 | Nausea and vomiting | symptoms | 0.074786 |
| 870.5 | -0.02447 | 0.122639 | -0.19955 | 0.841834 | -0.26484 | 0.215896 | 0.149722 | Open wound of lip and mouth | injuries & poisoni | 0.074774 |
| 290.1 | -0.00817 | 0.041142 | -0.19851 | 0.842644 | -0.0888 | 0.072469 | 0.270654 | Dementias | mental disorders | 0.074356 |
| 367.8 | -0.00981 | 0.049564 | -0.19793 | 0.843096 | -0.10695 | 0.087334 | 0.577736 | Hypermetropia | sense organs | 0.074123 |
| 593 | 0.006275 | 0.031865 | 0.19694 | 0.843875 | -0.05618 | 0.068729 | 0.159277 | Hematuria | genitourinary | 0.073722 |
| 8.6 | 0.010166 | 0.051778 | 1.96E-01 | 0.844352 | -0.09132 | 0.111648 | 0.720144 | Viral Enteritis | infectious diseas | 0.073476 |
| 611.3 | -0.0073 | 0.037668 | -0.19368 | 0.846428 | -0.08112 | 0.066532 | 0.168518 | Lump or mass in breast | genitourinary | 0.07241 |
| 636.2 | -0.03263 | 0.169264 | -0.19277 | 0.847143 | -0.36438 | 0.299123 | 0.202325 | Early onset of delivery | pregnancy compl | 0.072043 |

| phecode | beta | se | zval | pval | ci.lb | ci.ub | QEp | phenotype | category | minuslogp |
|---|---|---|---|---|---|---|---|---|---|---|
| 379.1 | -0.0374 | 0.194101 | -0.19268 | 0.847212 | -0.41783 | 0.343032 | 0.022521 | Scleritis and episcleritis | sense organs | 0.072008 |
| 250.41 | -0.00453 | 0.023716 | -0.19098 | 0.848539 | -0.05101 | 0.041954 | 0.532418 | Impaired fasting glucose | endocrine/metab | 0.071328 |
| 145.1 | 0.124569 | 0.65371 | 0.190557 | 0.848873 | -1.15668 | 1.405817 | 0.013367 | Cancer of lip | neoplasms | 0.071157 |
| 747.11 | 0.007006 | 0.037347 | 0.1876 | 0.85119 | -0.06619 | 0.080205 | 0.778074 | Cardiac shunt/ heart septal defect | congenital anoma | 0.069973 |
| 707.1 | -0.00866 | 0.046323 | -0.18703 | 0.851635 | -0.09945 | 0.082127 | 0.798828 | Decubitus ulcer | dermatologic | 0.069746 |
| 348.9 | -0.00568 | 0.030442 | -0.18656 | 0.852007 | -0.06535 | 0.053987 | 0.572507 | Other conditions of brain, NOS | neurological | 0.069557 |
| 369.5 | -0.00679 | 0.036467 | -0.1861 | 0.852363 | -0.07826 | 0.064688 | 0.257378 | Conjunctivitis, infectious | sense organs | 0.069375 |
| 704.2 | 0.035285 | 0.191211 | 0.184533 | 0.853595 | -0.33948 | 0.410051 | 0.008235 | Hirsutism | dermatologic | 0.068748 |
| 259.8 | 0.063482 | 0.345499 | 0.183739 | 0.854218 | -0.61368 | 0.740646 | 0.103998 | Polyglandular activity in multiple endocrine ade | endocrine/metab | 0.068431 |
| 761 | 0.007958 | 4.35E-02 | 0.18294 | 0.854845 | -0.07731 | 0.093223 | 0.011651 | Cervicalgia | symptoms | 0.068113 |
| 464 | -0.0036 | 0.019797 | -0.18205 | 0.855543 | -0.04241 | 0.035197 | 0.810428 | Acute sinusitis | respiratory | 0.067758 |
| 427.9 | 0.005848 | 0.032203 | 0.181591 | 0.855904 | -0.05727 | 0.068964 | 0.111593 | Palpitations | circulatory syster | 0.067575 |
| 780 | -0.00811 | 0.044924 | -0.18054 | 0.85673 | -0.09616 | 0.079939 | 0.687759 | Hypothermia/Chills | symptoms | 0.067156 |
| 626.12 | 0.006281 | 0.03516 | 0.178649 | 0.858213 | -0.06263 | 0.075193 | 0.620783 | Excessive or frequent menstruation | genitourinary | 0.066405 |
| 753.1 | 0.0583 | 0.328064 | 0.17771 | 0.858951 | -0.58469 | 0.701295 | 0.147788 | Congenital cataract and lens anomalies | congenital anoma | 0.066032 |
| 252 | 0.00485 | 0.027406 | 0.176968 | 0.859533 | -0.04887 | 0.058566 | 0.689803 | Disorders of parathyroid gland | endocrine/metab | 0.065737 |
| 727.8 | -0.09724 | 0.560802 | -0.17339 | 0.862348 | -1.19639 | 1.001917 | 0.002681 | Plica syndrome | musculoskeletal | 0.064317 |
| 227 | 0.006307 | 0.036575 | 0.172432 | 0.863098 | -0.06538 | 0.077992 | 0.603417 | Benign neoplasm of other endocrine glands ar | neoplasms | 0.06394 |
| 283 | 0.012473 | 0.072381 | 0.172321 | 0.863185 | -0.12939 | 0.154337 | 0.678465 | Acquired hemolytic anemias | hematopoietic | 0.063896 |
| 592.1 | 0.003454 | 0.020091 | 0.171929 | 0.863493 | -0.03592 | 0.042833 | 0.488351 | Cystitis | genitourinary | 0.063741 |
| 364.51 | -0.01628 | 0.095069 | -0.17125 | 0.86403 | -0.20261 | 0.170051 | 0.936046 | Fuchs' dystrophy | sense organs | 0.063471 |
| 365 | -0.00629 | 0.036804 | -0.1708 | 0.864382 | -0.07842 | 0.065849 | 0.132951 | Glaucoma | sense organs | 0.063294 |
| 597.8 | 0.043806 | 0.257677 | 0.170003 | 0.865008 | -0.46123 | 0.548844 | 0.018905 | Urethral hypermobility/ISD | genitourinary | 0.06298 |
| 279.11 | 0.007598 | 0.045 | 0.168853 | 0.865912 | -0.0806 | 0.095797 | 0.936837 | Deficiency of humoral immunity | endocrine/metab | 0.062526 |
| 440.1 | -0.02781 | 0.165098 | -0.16844 | 0.866238 | -0.35139 | 0.295777 | 0.035579 | Atherosclerosis of renal artery | circulatory syster | 0.062363 |
| 195.1 | -0.00613 | 0.036426 | -0.1682 | 0.866425 | -0.07752 | 0.065267 | 0.072802 | Malignant neoplasm, other | neoplasms | 0.062269 |
| 427.5 | -0.00713 | 0.042611 | -0.16734 | 0.867105 | -0.09065 | 0.076385 | 0.217982 | Arrhythmia (cardiac) NOS | circulatory syster | 0.061928 |
| 313.1 | -0.00842 | 0.050462 | -0.16685 | 0.867489 | -0.10732 | 0.090484 | 0.396821 | Attention deficit hyperactivity disorder | mental disorders | 0.061736 |
| 715.2 | -0.0353 | 0.212324 | -0.16624 | 0.867968 | -0.45144 | 0.38085 | 0.03615 | Ankylosing spondylitis | musculoskeletal | 0.061496 |
| 681.1 | -0.00867 | 0.05246 | -0.16535 | 0.868667 | -0.1115 | 0.094146 | 0.254161 | Cellulitis and abscess of fingers/toes | dermatologic | 0.061147 |
| 170.1 | 0.019679 | 0.119073 | 0.165266 | 0.868734 | -0.2137 | 0.253057 | 0.033505 | Bone cancer | neoplasms | 0.061113 |
| 313.3 | 0.019022 | 0.115446 | 0.164773 | 0.869123 | -0.20725 | 0.245291 | 0.570119 | Autism | mental disorders | 0.060919 |
| 601 | -0.00859 | 0.052173 | -0.16466 | 0.86921 | -0.11085 | 0.093667 | 0.412563 | Inflammatory diseases of prostate | genitourinary | 0.060875 |
| 210 | -0.018 | 0.109519 | -0.16435 | 0.869457 | -0.23265 | 0.196654 | 0.28977 | Benign neoplasm of lip, oral cavity, and pharyr | neoplasms | 0.060752 |
| 159.3 | 0.029201 | 0.180011 | 0.162219 | 0.871133 | -0.32361 | 0.382015 | 0.092357 | Malignant neoplasm of gallbladder and extrahe | neoplasms | 0.059915 |
| 637 | -0.01841 | 0.113627 | -0.16198 | 0.871323 | -0.24111 | 0.2043 | 0.846918 | Short gestation; low birth weight; and fetal grov | pregnancy compl | 0.059821 |
| 592.13 | -0.02314 | 0.143236 | -0.16152 | 0.871683 | -0.30387 | 0.257601 | 0.232971 | Chronic interstitial cystitis | genitourinary | 0.059642 |
| 353.1 | 0.00928 | 0.05776 | 0.16067 | 0.872353 | -0.10393 | 0.122487 | 0.449775 | Nerve plexus lesions | neurological | 0.059308 |
| 540.11 | 0.011094 | 0.069643 | 0.159292 | 0.873439 | -0.1254 | 0.147591 | 0.849002 | Acute appendicitis | digestive | 0.058768 |
| 803.2 | -0.00713 | 0.045592 | -0.15636 | 0.875752 | -0.09649 | 0.08223 | 0.916971 | Fracture of radius and ulna | injuries & poisoni | 0.057619 |
| 396 | 0.005886 | 0.037742 | 0.155949 | 0.876073 | -0.06809 | 0.07986 | 0.139427 | Abnormal heart sounds | circulatory syster | 0.057459 |
| 444.5 | 0.097256 | 0.624802 | 0.155659 | 0.876302 | -1.12733 | 1.321845 | 0.042695 | Atheroembolism | circulatory syster | 0.057346 |
| 386.9 | 0.003882 | 0.024952 | 0.155572 | 0.87637 | -0.04502 | 0.052787 | 0.220227 | Dizziness and giddiness (Light-headedness ar | sense organs | 0.057312 |
| 426.23 | -0.0282 | 0.182365 | -0.15463 | 0.877115 | -0.38563 | 0.329229 | 0.028957 | Second degree AV block | circulatory syster | 0.056944 |
| 443.8 | 0.015259 | 0.099114 | 0.153952 | 0.877647 | -0.179 | 0.209519 | 0.115744 | Other specified peripheral vascular diseases | circulatory syster | 0.05668 |
| 983 | -0.0528 | 0.344338 | -0.15333 | 0.878136 | -0.72769 | 0.622092 | 0.402838 | Toxic effect of corrosive aromatics, acids, and | injuries & poisoni | 0.056438 |
| 364.9 | -0.01874 | 0.122421 | -0.15312 | 0.878306 | -0.25869 | 0.221196 | 0.564417 | Cornea replaced by transplant | sense organs | 0.056354 |
| 369 | -0.00481 | 0.031945 | -0.15055 | 0.880331 | -0.06742 | 0.057801 | 0.338666 | Infection of the eye | sense organs | 0.055354 |

| phecode | beta | se | zval | pval | ci.lb | ci.ub | QEp | phenotype | category | minuslogp |
|---|---|---|---|---|---|---|---|---|---|---|
| 315 | -0.01454 | 0.096697 | -0.15039 | 0.880458 | -0.20406 | 0.17498 | 0.046696 | Develomental delays and disorders | mental disorders | 0.055292 |
| 189.12 | -0.03551 | 0.237622 | -0.14944 | 0.881205 | -0.50124 | 0.430221 | 0.020729 | Malignant neoplasm of renal pelvis | neoplasms | 0.054923 |
| 729.3 | 0.019538 | 0.132409 | 0.147555 | 0.882694 | -0.23998 | 0.279055 | 0.442455 | Panniculitis | musculoskeletal | 0.05419 |
| 191.11 | -0.00811 | 0.055555 | -0.14593 | 0.883975 | -0.11699 | 0.100779 | 0.92354 | Cancer of brain | neoplasms | 0.05356 |
| 426.32 | 0.006746 | 0.046413 | 0.145353 | 0.884432 | -0.08422 | 0.097715 | 0.474667 | Left bundle branch block | circulatory syster | 0.053335 |
| 709.3 | 0.010364 | 0.072769 | 0.142423 | 0.886746 | -0.13226 | 0.152988 | 0.514478 | Systemic sclerosis | dermatologic | 0.052201 |
| 701.4 | 0.005459 | 0.038564 | 0.141553 | 0.887433 | -0.07013 | 0.081043 | 0.672027 | Keloid scar | dermatologic | 0.051865 |
| 853 | 0.009132 | 0.064883 | 0.140752 | 0.888066 | -0.11804 | 0.136301 | 0.843523 | Complication of colostomy or enterostomy | injuries & poisoni | 0.051555 |
| 350.2 | 0.006522 | 0.046473 | 0.140342 | 0.88839 | -0.08456 | 0.097608 | 0.050252 | Abnormality of gait | neurological | 0.051396 |
| 703.1 | 0.007373 | 0.052713 | 0.139874 | 0.88876 | -0.09594 | 0.110688 | 0.236615 | Ingrowing nail | dermatologic | 0.051216 |
| 695 | -0.00485 | 0.034957 | -0.13884 | 0.889579 | -0.07337 | 0.063662 | 0.148272 | Erythematous conditions | dermatologic | 0.050815 |
| 706.2 | -0.00554 | 0.040277 | -0.13745 | 0.890678 | -0.08448 | 0.073405 | 0.184766 | Sebaceous cyst | dermatologic | 0.050279 |
| 477 | 0.007794 | 0.057453 | 0.135664 | 0.892087 | -0.10481 | 0.1204 | 0.077432 | Epistaxis or throat hemorrhage | respiratory | 0.049593 |
| 180 | -0.01102 | 0.081836 | -0.13462 | 0.892912 | -0.17141 | 0.149379 | 0.063789 | Cervical cancer and dysplasia | neoplasms | 0.049191 |
| 575.2 | -0.00603 | 0.044986 | -0.13411 | 0.893315 | -0.09421 | 0.082139 | 0.662275 | Obstruction of bile duct | digestive | 0.048995 |
| 383 | 0.017311 | 0.129091 | 0.134102 | 0.893322 | -0.2357 | 0.270326 | 0.510481 | Otosclerosis | sense organs | 0.048992 |
| 80 | 0.006653 | 0.0499 | 0.133331 | 0.893931 | -0.09115 | 0.104455 | 0.478873 | Postoperative infection | infectious diseas | 0.048696 |
| 701 | -0.00215 | 0.016123 | -0.13304 | 0.894161 | -0.03375 | 0.029456 | 0.492826 | Other hypertrophic and atrophic conditions of s | dermatologic | 0.048584 |
| 295.1 | -0.01093 | 0.08235 | -0.13277 | 0.894376 | -0.17234 | 0.150469 | 0.968239 | Schizophrenia | mental disorders | 0.04848 |
| 521.2 | 0.07381 | 0.557624 | 0.132365 | 0.894696 | -1.01911 | 1.166732 | 0.20334 | Dental abrasion, erosion and attrition | digestive | 0.048325 |
| 706.1 | -0.00767 | 0.058363 | -0.13147 | 0.895406 | -0.12206 | 0.106716 | 0.042252 | Acne | dermatologic | 0.04798 |
| 379.51 | 0.019583 | 0.151107 | 0.129596 | 0.896886 | -0.27658 | 0.315746 | 0.808023 | Pigmentary iris degeneration | sense organs | 0.047263 |
| 529 | 0.005647 | 0.044016 | 0.128289 | 0.89792 | -0.08062 | 0.091917 | 0.496418 | Diseases and other conditions of the tongue | digestive | 0.046762 |
| 531.3 | -0.0076 | 0.059439 | -0.1279 | 0.89823 | -0.1241 | 0.108896 | 0.888414 | Duodenal ulcer | digestive | 0.046612 |
| 727.1 | 0.004521 | 0.035403 | 0.127714 | 0.898375 | -0.06487 | 0.07391 | 0.122771 | Synovitis and tenosynovitis | musculoskeletal | 0.046542 |
| 614.33 | 0.030334 | 0.239377 | 0.126719 | 0.899162 | -0.43884 | 0.499503 | 0.04288 | Pelvic inflammatory disease, NOS | genitourinary | 0.046162 |
| 117.4 | 0.0085 | 0.067746 | 0.125472 | 0.90015 | -0.12428 | 0.14128 | 0.612583 | Aspergillosis | infectious diseas | 0.045685 |
| 512.3 | 0.009248 | 0.07398 | 0.125003 | 0.900521 | -0.13575 | 0.154246 | 0.542887 | Abnormal chest sounds | respiratory | 0.045506 |
| 374.3 | 0.005201 | 0.041788 | 0.124468 | 0.900945 | -0.0767 | 0.087105 | 0.394268 | Ptosis of eyelid | sense organs | 0.045302 |
| 289.5 | 0.007307 | 0.059589 | 0.122626 | 0.902403 | -0.10949 | 0.1241 | 0.614149 | Diseases of spleen | hematopoietic | 0.0446 |
| 716.3 | 0.091541 | 0.760024 | 0.120445 | 0.904131 | -1.39808 | 1.581161 | 1 | Kaschin-Beck disease | musculoskeletal | 0.043769 |
| 506 | -0.00618 | 0.051472 | -0.12003 | 0.904456 | -0.10706 | 0.094705 | 0.066151 | Empyema and pneumothorax | respiratory | 0.043613 |
| 38.1 | 0.004571 | 0.039312 | 1.16E-01 | 0.907444 | -0.07248 | 0.081621 | 0.810492 | Gram negative septicemia | infectious diseas | 0.04218 |
| 531.4 | -0.00369 | 0.032258 | -0.11454 | 0.908813 | -0.06692 | 0.05953 | 0.864062 | Peptic ulcer, site unspecified | digestive | 0.041525 |
| 972 | 0.009763 | 0.085395 | 0.114326 | 0.908979 | -0.15761 | 0.177134 | 0.248872 | Poisoning by agents primarily affecting the car | injuries & poisoni | 0.041446 |
| 938 | 0.003033 | 0.026604 | 0.11401 | 0.90923 | -0.04911 | 0.055175 | 0.659658 | Dermatitis due to solar radiation | dermatologic | 0.041326 |
| 324.1 | -0.1305 | 1.145207 | -0.11395 | 0.909275 | -2.37506 | 2.114064 | 1 | Jakob-Creutzfeldt disease | neurological | 0.041305 |
| 614.31 | 0.020747 | 0.185837 | 0.111641 | 0.911108 | -0.34349 | 0.384981 | 0.573819 | Acute inflammatory pelvic disease | genitourinary | 0.04043 |
| 627.22 | -0.00271 | 0.024298 | -0.11145 | 0.911258 | -0.05033 | 0.044914 | 0.448346 | Need for Hormone replacement therapy (postr | genitourinary | 0.040358 |
| 252.2 | 0.006665 | 0.059884 | 0.111295 | 0.911382 | -0.11071 | 0.124036 | 0.837498 | Hypoparathyroidism | endocrine/metab | 0.040299 |
| 709.6 | -0.01354 | 0.122671 | -0.11036 | 0.912124 | -0.25397 | 0.226894 | 0.104759 | Other specified diffuse diseases of connective | dermatologic | 0.039946 |
| 585.34 | -0.00383 | 0.034813 | -0.10994 | 0.912455 | -0.07206 | 0.064404 | 0.68446 | Chronic Kidney Disease, Stage IV | genitourinary | 0.039789 |
| 741.3 | -0.00574 | 0.052282 | -0.10975 | 0.912612 | -0.10821 | 0.096733 | 0.538981 | Difficulty in walking | musculoskeletal | 0.039714 |
| 370.3 | 0.016151 | 0.147273 | 0.109664 | 0.912676 | -0.2725 | 0.3048 | 0.022993 | Keratoconjunctivitis | sense organs | 0.039683 |
| 110 | 0.005584 | 0.051041 | 0.1094 | 0.912885 | -0.09446 | 0.105623 | 0.001203 | Dermatophytosis / Dermatomycosis | infectious diseas | 0.039584 |
| 256.4 | -0.00654 | 0.060193 | -0.10871 | 0.913435 | -0.12452 | 0.111433 | 0.363955 | Polycystic ovaries | endocrine/metab | 0.039323 |
| 743.4 | -0.00753 | 0.069706 | -0.10804 | 0.913961 | -0.14415 | 0.129091 | 0.387831 | Stress fracture | musculoskeletal | 0.039072 |
| 626.1 | -0.00462 | 0.042966 | -0.10751 | 0.914386 | -0.08883 | 0.079593 | 0.078681 | Irregular menstrual cycle/bleeding | genitourinary | 0.03887 |

| phecode | beta | se | zval | pval | ci.lb | ci.ub | QEp | phenotype | category | minuslogp |
|---|---|---|---|---|---|---|---|---|---|---|
| 415.21 | 0.007418 | 0.070075 | 0.105853 | 0.915699 | -0.12993 | 0.144762 | 0.139069 | Primary pulmonary hypertension | circulatory syster | 0.038247 |
| 184 | -0.00586 | 0.055393 | -0.10582 | 0.915727 | -0.11443 | 0.102707 | 0.356905 | Cancer of other female genital organs | neoplasms | 0.038234 |
| 560.4 | 0.005394 | 0.05156 | 0.104625 | 0.916673 | -0.09566 | 0.10645 | 0.201305 | Other intestinal obstruction | digestive | 0.037785 |
| 276.5 | -0.00203 | 0.019405 | -0.1046 | 0.916696 | -0.04006 | 0.036004 | 0.487969 | Hypovolemia | endocrine/metab | 0.037775 |
| 626 | 0.002392 | 0.022894 | 0.104488 | 0.916782 | -0.04248 | 0.047263 | 0.464638 | Disorders of menstruation and other abnormal | genitourinary | 0.037734 |
| 698 | -0.00601 | 0.05808 | -0.10354 | 0.917531 | -0.11985 | 0.107821 | 0.001475 | Pruritus and related conditions | dermatologic | 0.037379 |
| 701.6 | 0.012262 | 0.119342 | 0.102748 | 0.918163 | -0.22164 | 0.246169 | 0.235449 | Acquired acanthosis nigricans | dermatologic | 0.03708 |
| 749 | 0.008934 | 0.08746 | 0.102147 | 0.91864 | -0.16249 | 0.180353 | 0.408565 | Congenital anomalies of face and neck | congenital anoma | 0.036855 |
| 656.5 | 0.078413 | 0.772468 | 0.10151 | 0.919146 | -1.4356 | 1.592423 | 0.827345 | Hematological disorders of newborn | pregnancy compl | 0.036616 |
| 377.1 | -0.00619 | 0.063128 | -0.09804 | 0.921898 | -0.12992 | 0.117539 | 0.452153 | Optic atrophy | sense organs | 0.035317 |
| 255.3 | -0.01425 | 1.47E-01 | -0.09673 | 0.92294 | -0.3029 | 0.274409 | 0.87296 | Adrenogenital disorders | endocrine/metab | 0.034827 |
| 296.1 | -0.00289 | 0.030704 | -0.09396 | 0.92514 | -0.06306 | 0.057294 | 0.651121 | Bipolar | mental disorders | 0.033793 |
| 384.4 | -0.01205 | 0.128781 | -0.09358 | 0.92544 | -0.26446 | 0.240355 | 0.155071 | Perforation of tympanic membrane | sense organs | 0.033652 |
| 446 | -0.00491 | 0.05275 | -0.09309 | 0.925829 | -0.1083 | 0.098477 | 0.797662 | Polyarteritis nodosa and allied conditions | circulatory syster | 0.033469 |
| 628 | 0.003777 | 0.041298 | 0.091463 | 0.927125 | -0.07716 | 0.084719 | 0.273246 | Ovarian cyst | genitourinary | 0.032862 |
| 286.81 | 0.003934 | 0.043393 | 0.090664 | 0.927759 | -0.08111 | 0.088982 | 0.999112 | Primary hypercoagulable state | hematopoietic | 0.032565 |
| 558 | -0.00301 | 0.035332 | -0.0853 | 0.932019 | -0.07226 | 0.066236 | 0.185257 | Noninfectious gastroenteritis | digestive | 0.030575 |
| 750.22 | 0.006311 | 0.075785 | 0.083271 | 0.933636 | -0.14222 | 0.154846 | 0.716798 | Congenital anomaly of gallbladder, bile ducts, | congenital anoma | 0.029822 |
| 261.2 | -0.0032 | 0.040074 | -0.07993 | 0.936295 | -0.08175 | 0.075341 | 0.154511 | Vitamin B-complex deficiencies | endocrine/metab | 0.028587 |
| 370.1 | -0.02456 | 0.308494 | -0.07962 | 0.936542 | -0.6292 | 0.580076 | 0.000803 | Corneal ulcer | sense organs | 0.028473 |
| 54 | 0.001929 | 0.024268 | 7.95E-02 | 0.936639 | -0.04564 | 0.049493 | 0.977673 | Herpes simplex | infectious diseas | 0.028428 |
| 420.21 | -0.00851 | 0.107657 | -0.07903 | 0.937008 | -0.21951 | 0.202496 | 0.254744 | Acute pericarditis | circulatory syster | 0.028257 |
| 253.11 | -0.01456 | 0.186104 | -0.07822 | 0.937652 | -0.37931 | 0.3502 | 0.651576 | Acromegaly and gigantism | endocrine/metab | 0.027958 |
| 442 | 0.003312 | 4.23E-02 | 0.078217 | 0.937656 | -0.07969 | 0.086313 | 0.310602 | Other aneurysm | circulatory syster | 0.027957 |
| 790.1 | -0.00389 | 0.050574 | -0.07697 | 0.938649 | -0.10302 | 0.09523 | 0.562442 | Elevated sedimentation rate | symptoms | 0.027497 |
| 916 | 0.002361 | 0.030855 | 0.07652 | 0.939005 | -0.05811 | 0.062836 | 0.227519 | Contusion | injuries & poisoni | 0.027332 |
| 450 | -0.00337 | 0.044342 | -0.07591 | 0.939494 | -0.09027 | 0.083542 | 0.684839 | Noninfectious disorders of lymphatic channels | circulatory syster | 0.027106 |
| 735.22 | -0.06128 | 0.828537 | -0.07396 | 0.941042 | -1.68518 | 1.562623 | 0.083725 | Claw toe (acquired) | musculoskeletal | 0.026391 |
| 350 | 0.002159 | 0.029264 | 0.073774 | 0.94119 | -0.0552 | 0.059515 | 0.151248 | Abnormal movement | neurological | 0.026323 |
| 340 | 0.0028 | 0.038353 | 0.072994 | 0.941811 | -0.07237 | 0.077969 | 0.098955 | Migraine | neurological | 0.026036 |
| 706 | 0.003332 | 0.045884 | 0.072608 | 0.942118 | -0.0866 | 0.093263 | 0.007421 | Diseases of sebaceous glands | dermatologic | 0.025895 |
| 259 | 0.002491 | 0.034562 | 0.072074 | 0.942543 | -0.06525 | 0.070231 | 0.519778 | Other endocrine disorders | endocrine/metab | 0.025699 |
| 277.2 | 0.047406 | 0.658376 | 0.072004 | 0.942599 | -1.24299 | 1.3378 | 0.383643 | Other disorders of purine and pyrimidine meta | endocrine/metab | 0.025673 |
| 364.4 | 0.007675 | 0.107208 | 0.071594 | 0.942925 | -0.20245 | 0.217799 | 0.185341 | Corneal degenerations | sense organs | 0.025523 |
| 459.1 | 0.002861 | 0.040251 | 0.071068 | 0.943344 | -0.07603 | 0.081752 | 0.358699 | Hemorrhage NOS | circulatory syster | 0.02533 |
| 737.3 | -0.00209 | 0.029437 | -0.07091 | 0.943471 | -0.05978 | 0.055609 | 0.823706 | Kyphoscoliosis and scoliosis | musculoskeletal | 0.025271 |
| 512.8 | -0.00096 | 0.014019 | -0.06824 | 0.945595 | -0.02843 | 0.026521 | 0.945605 | Cough | respiratory | 0.024295 |
| 426.2 | 0.00226 | 0.033559 | 0.067349 | 0.946304 | -0.06351 | 0.068035 | 0.753797 | Atrioventricular [AV] block | circulatory syster | 0.023969 |
| 556.11 | -0.00513 | 0.07746 | -0.0662 | 0.94722 | -0.15695 | 0.14669 | 0.908704 | Angiodysplasia of intestine (without mention of | digestive | 0.023549 |
| 691.3 | 0.009483 | 0.144088 | 0.065812 | 0.947528 | -0.27292 | 0.29189 | 0.833985 | Congenital pigmentary anomalies of skin | dermatologic | 0.023408 |
| 191.1 | 0.003442 | 0.052501 | 0.065552 | 0.947735 | -0.09946 | 0.106343 | 0.740102 | Cancer of brain and nervous system | neoplasms | 0.023313 |
| 204.12 | 0.006129 | 0.094335 | 0.064967 | 0.9482 | -0.17876 | 0.191022 | 0.795035 | Lymphoid leukemia, chronic | neoplasms | 0.0231 |
| 255.11 | 0.009045 | 0.140059 | 0.06458 | 0.948509 | -0.26547 | 0.283556 | 0.206258 | Cushing's syndrome | endocrine/metab | 0.022959 |
| 524.3 | -0.02225 | 0.345421 | -0.06443 | 0.948632 | -0.69927 | 0.654759 | 0.018901 | Anomalies of tooth position/malocclusion | digestive | 0.022902 |
| 209 | -0.00439 | 0.068326 | -0.06425 | 0.94877 | -0.13831 | 0.129526 | 0.311245 | Neuroendocrine tumors | neoplasms | 0.022839 |
| 500.1 | 0.006669 | 0.104019 | 0.064114 | 0.94888 | -0.1972 | 0.210543 | 0.332537 | Extrinsic allergic alveolitis | respiratory | 0.022789 |
| 281.12 | -0.00819 | 0.130799 | -0.06262 | 0.950071 | -0.26455 | 0.248172 | 0.171056 | Other vitamin B12 deficiency anemia | hematopoietic | 0.022244 |
| 526 | -0.00184 | 0.029951 | -0.06158 | 0.9509 | -0.06055 | 0.056858 | 0.841904 | Diseases of the jaws | digestive | 0.021865 |

| phecode | beta | se | zval | pval | ci.lb | ci.ub | QEp | phenotype | category | minuslogp |
|---|---|---|---|---|---|---|---|---|---|---|
| 280.2 | -0.00217 | 0.035542 | -0.061 | 0.951355 | -0.07183 | 0.067493 | 0.269754 | Iron deficiency anemia secondary to blood loss | hematopoietic | 0.021657 |
| 389 | -0.00222 | 0.037338 | -0.05956 | 0.952503 | -0.0754 | 0.070957 | 0.022995 | Hearing loss | sense organs | 0.021133 |
| 599.2 | 0.001437 | 0.024193 | 0.059381 | 0.952649 | -0.04598 | 0.048853 | 0.426791 | Retention of urine | genitourinary | 0.021067 |
| 513.32 | 0.008877 | 0.149771 | 0.059269 | 0.952738 | -0.28467 | 0.302423 | 0.029205 | Orthopnea | respiratory | 0.021026 |
| 870.1 | 0.005219 | 0.088519 | 0.05896 | 0.952984 | -0.16827 | 0.178712 | 0.346389 | Open wound or laceration of eye or eyelid | injuries & poisoni | 0.020914 |
| 781.2 | 0.011841 | 0.203241 | 0.058259 | 0.953542 | -0.3865 | 0.410186 | 0.00171 | Abnormal posture | symptoms | 0.02066 |
| 530.15 | 0.006683 | 0.117768 | 0.056745 | 0.954748 | -0.22414 | 0.237505 | 0.610391 | Eosinophilic esophagitis | digestive | 0.020111 |
| 706.3 | 0.011587 | 0.214632 | 0.053986 | 0.956946 | -0.40908 | 0.432258 | 0.284502 | Seborrhea | dermatologic | 0.019112 |
| 696.41 | -0.00182 | 0.033875 | -0.05373 | 0.957148 | -0.06821 | 0.064574 | 0.774972 | Psoriasis vulgaris | dermatologic | 0.019021 |
| 509.3 | -0.0051 | 0.095546 | -0.05338 | 0.957429 | -0.19237 | 0.182166 | 0.080076 | Pulmonary insufficiency or respiratory failure fo | respiratory | 0.018893 |
| 876 | -0.00286 | 0.055814 | -0.05122 | 0.959152 | -0.11225 | 0.106535 | 0.7264 | Posttraumatic wound infection not elsewhere c | injuries & poisoni | 0.018112 |
| 1005 | -0.00072 | 0.014449 | -0.05015 | 0.960003 | -0.02904 | 0.027594 | 0.741236 | Other symptoms | other | 0.017727 |
| 703 | -0.00247 | 0.050285 | -0.04915 | 0.960802 | -0.10103 | 0.096085 | 0.05874 | Diseases of nail, NOS | dermatologic | 0.017366 |
| 770 | -0.00172 | 0.035092 | -0.0489 | 0.960999 | -0.0705 | 0.067063 | 0.059148 | Myalgia and myositis unspecified | symptoms | 0.017277 |
| 592 | -0.00096 | 0.019691 | -0.04856 | 0.961269 | -0.03955 | 0.037637 | 0.459808 | Cystitis and urethritis | genitourinary | 0.017155 |
| 426.8 | 0.003543 | 0.073297 | 0.048344 | 0.961442 | -0.14012 | 0.147203 | 0.242598 | Other cardiac conduction disorders | circulatory syster | 0.017077 |
| 644 | 0.003354 | 0.073869 | 0.045401 | 0.963787 | -0.14143 | 0.148135 | 0.221115 | Anemia during pregnancy | pregnancy compl | 0.016019 |
| 41.9 | 0.001988 | 0.045451 | 4.37E-02 | 0.965118 | -0.08709 | 0.091069 | 0.833977 | Infection with drug-resistant microorganisms | infectious diseas | 0.015419 |
| 727.6 | 0.00375 | 0.086078 | 0.043568 | 0.965249 | -0.16496 | 0.172459 | 0.151765 | Rupture of tendon, nontraumatic | musculoskeletal | 0.015361 |
| 293 | 0.001677 | 0.042308 | 0.039644 | 0.968377 | -0.08124 | 0.084599 | 0.413194 | Symptoms involving head and neck | mental disorders | 0.013956 |
| 759 | 0.002909 | 7.51E-02 | 0.038763 | 0.96908 | -0.1442 | 0.150022 | 0.194328 | Other and unspecified congenital anomalies | congenital anoma | 0.01364 |
| 395 | 0.001431 | 0.038291 | 0.037368 | 0.970192 | -0.07362 | 0.076479 | 0.073735 | Heart valve disorders | circulatory syster | 0.013142 |
| 913 | -0.00761 | 0.210355 | -0.03617 | 0.971147 | -0.4199 | 0.40468 | 0.02187 | Toxic effect of venom | injuries & poisoni | 0.012715 |
| 818 | 0.003026 | 0.085481 | 0.035399 | 0.971761 | -0.16451 | 0.170565 | 0.491664 | Intracranial hemorrhage (injury) | injuries & poisoni | 0.01244 |
| 427.61 | -0.00142 | 0.04064 | -0.03503 | 0.972058 | -0.08108 | 0.07823 | 0.625368 | Supraventricular premature beats | circulatory syster | 0.012308 |
| 668 | 0.014155 | 0.405352 | 0.03492 | 0.972143 | -0.78032 | 0.808631 | 0.19074 | Complications of the administration of anesthe | pregnancy compl | 0.01227 |
| 994.1 | 0.001648 | 0.047601 | 0.034627 | 0.972377 | -0.09165 | 0.094945 | 0.851198 | Systemic inflammatory response syndrome (S | injuries & poisoni | 0.012165 |
| 276.11 | -0.00127 | 0.037987 | -0.03339 | 0.973361 | -0.07572 | 0.073185 | 0.767696 | Hyperosmolality and/or hypernatremia | endocrine/metab | 0.011726 |
| 557 | -0.0015 | 0.045052 | -0.03321 | 0.973505 | -0.0898 | 0.086804 | 0.639642 | Intestinal malabsorption (non-celiac) | digestive | 0.011662 |
| 334.1 | 0.007764 | 0.233914 | 0.033192 | 0.973521 | -0.4507 | 0.466226 | 0.138852 | Spinocerebellar disease | neurological | 0.011655 |
| 695.3 | -0.00134 | 0.041401 | -0.03244 | 0.974125 | -0.08249 | 0.079801 | 0.293348 | Rosacea | dermatologic | 0.011385 |
| 170 | 0.002786 | 0.088523 | 0.03147 | 0.974894 | -0.17072 | 0.176288 | 0.020652 | Cancer of bone and connective tissue | neoplasms | 0.011042 |
| 528.4 | 0.005001 | 0.158967 | 0.031457 | 0.974905 | -0.30657 | 0.31657 | 0.890125 | Cysts of oral soft tissues | digestive | 0.011038 |
| 132 | 0.002826 | 0.091733 | 0.03081 | 0.975421 | -0.17697 | 0.18262 | 0.457204 | Infestation (lice, mites) | infectious diseas | 0.010808 |
| 540 | 0.001516 | 0.049558 | 0.030598 | 0.97559 | -0.09562 | 0.098648 | 0.972423 | Appendiceal conditions | digestive | 0.010733 |
| 427.3 | -0.00051 | 0.018391 | -0.02781 | 0.97781 | -0.03656 | 0.035533 | 0.727195 | Other specified cardiac dysrhythmias | circulatory syster | 0.009745 |
| 531 | -0.00065 | 0.024842 | -0.02611 | 0.979172 | -0.04934 | 0.04804 | 0.282988 | Peptic ulcer (excl. esophageal) | digestive | 0.009141 |
| 341 | 0.002879 | 0.111597 | 0.025796 | 0.97942 | -0.21585 | 0.221604 | 0.963658 | Other demyelinating diseases of central nervo | neurological | 0.009031 |
| 803 | -0.00077 | 0.029857 | -0.02571 | 0.979493 | -0.05929 | 0.05775 | 0.904517 | Fracture of upper limb | injuries & poisoni | 0.008999 |
| 550 | 0.000419 | 0.016365 | 0.025621 | 0.979559 | -0.03166 | 0.032495 | 0.612624 | Abdominal hernia | digestive | 0.008969 |
| 589 | -0.00111 | 0.043674 | -0.02548 | 0.979672 | -0.08671 | 0.084486 | 0.956288 | Abnormal results of function study of kidney | genitourinary | 0.008919 |
| 280 | 0.000455 | 0.018625 | 0.024441 | 0.980501 | -0.03605 | 0.03696 | 0.469078 | Iron deficiency anemias | hematopoietic | 0.008552 |
| 306.9 | 0.000993 | 0.040993 | 0.02423 | 0.980669 | -0.07935 | 0.081339 | 0.428489 | Tension headache | mental disorders | 0.008478 |
| 529.6 | -0.00259 | 0.114388 | -0.02268 | 0.981902 | -0.22679 | 0.221602 | 0.239356 | Glossodynia | digestive | 0.007932 |
| 327.72 | 0.003695 | 0.167884 | 0.022007 | 0.982442 | -0.32535 | 0.332741 | 0.077518 | Sleep related leg cramps | neurological | 0.007693 |
| 277.5 | 0.000924 | 0.046794 | 0.019752 | 0.984242 | -0.09079 | 0.092639 | 0.990118 | Other disorders of lipoid metabolism | endocrine/metab | 0.006898 |
| 750.2 | 0.002535 | 0.129086 | 0.019641 | 0.98433 | -0.25047 | 0.255539 | 0.049182 | Lower gastrointestinal congenital anomalies | congenital anoma | 0.006859 |
| 303.31 | -0.00112 | 0.05822 | -0.01925 | 0.984643 | -0.11523 | 0.112988 | 0.744089 | Gastrointestinal malfunction arising from ment | mental disorders | 0.006721 |

| phecode | beta | se | zval | pval | ci.lb | ci.ub | QEp | phenotype | category | minuslogp |
|---|---|---|---|---|---|---|---|---|---|---|
| 555.1 | 0.000778 | 0.045622 | 0.01706 | 0.986389 | -0.08864 | 0.090197 | 0.821657 | Regional enteritis | digestive | 0.005952 |
| 696 | 0.000536 | 0.031455 | 0.017032 | 0.986411 | -0.06112 | 0.062187 | 0.969716 | Psoriasis and related disorders | dermatologic | 0.005942 |
| 157 | 0.000774 | 0.054775 | 0.014137 | 0.988721 | -0.10658 | 0.108132 | 0.322888 | Pancreatic cancer | neoplasms | 0.004926 |
| 830 | -0.00032 | 0.025639 | -0.01245 | 0.990067 | -0.05057 | 0.049932 | 0.993635 | Dislocation | injuries & poisoni | 0.004335 |
| 252.1 | -0.00034 | 0.028502 | -0.01178 | 0.990597 | -0.0562 | 0.055526 | 0.550454 | Hyperparathyroidism | endocrine/metab | 0.004103 |
| 724.2 | 0.000527 | 0.045785 | 0.011516 | 0.990812 | -0.08921 | 0.090265 | 0.198475 | Disorders of coccyx | musculoskeletal | 0.004009 |
| 474 | 0.000514 | 0.045689 | 0.01125 | 0.991024 | -0.08903 | 0.090062 | 0.837316 | Acute and chronic tonsillitis | respiratory | 0.003916 |
| 281.1 | -0.00107 | 0.101926 | -0.01053 | 0.991598 | -0.20084 | 0.198698 | 0.09386 | Megaloblastic anemia | hematopoietic | 0.003664 |
| 724.1 | 0.000493 | 0.048666 | 0.010122 | 0.991924 | -0.09489 | 0.095876 | 0.123502 | Disorders of sacrum | musculoskeletal | 0.003522 |
| 588.1 | 0.001494 | 0.161051 | 0.009276 | 0.992599 | -0.31416 | 0.317147 | 0.251217 | Renal osteodystrophy | genitourinary | 0.003226 |
| 611.11 | 0.000697 | 0.076192 | 0.009152 | 0.992698 | -0.14864 | 0.15003 | 0.153046 | Mammographic microcalcification | genitourinary | 0.003183 |
| 110.1 | 0.000391 | 0.045583 | 0.008571 | 0.993161 | -0.08895 | 0.089732 | 0.007973 | Dermatophytosis | infectious diseas | 0.00298 |
| 283.1 | -0.00296 | 0.356655 | -0.0083 | 0.993381 | -0.70199 | 0.696072 | 0.001385 | Autoimmune hemolytic anemias | hematopoietic | 0.002884 |
| 327.5 | 0.000981 | 0.13997 | 0.007006 | 0.99441 | -0.27336 | 0.275317 | 0.056258 | Parasomnia | neurological | 0.002435 |
| 295.3 | -0.00062 | 0.090357 | -0.00681 | 0.994564 | -0.17771 | 0.17648 | 0.05369 | Psychosis | mental disorders | 0.002367 |
| 610.2 | -0.00059 | 0.133768 | -0.00442 | 0.996475 | -0.26277 | 0.26159 | 0.098138 | Fibroadenosis of breast | genitourinary | 0.001534 |
| 687.4 | -7.18E-05 | 0.017321 | -0.00414 | 0.996693 | -0.03402 | 0.033876 | 0.701356 | Disturbance of skin sensation | dermatologic | 0.001439 |
| 292.2 | -0.00018 | 0.048821 | -0.00371 | 0.99704 | -0.09587 | 0.095506 | 0.755346 | Mild cognitive impairment | mental disorders | 0.001287 |
| 474.2 | 0.000109 | 0.050825 | 0.00215 | 0.998284 | -0.0995 | 0.099724 | 0.515779 | Chronic tonsillitis and adenoiditis | respiratory | 0.000746 |
| 379.3 | -0.00031 | 0.150348 | -0.00206 | 0.998354 | -0.29499 | 0.294367 | 0.240333 | Aphakia and other disorders of lens | sense organs | 0.000715 |
| 580.12 | 0.000146 | 0.082078 | 0.001773 | 0.998585 | -0.16072 | 0.161016 | 0.919719 | Non-proliferative glomerulonephritis | genitourinary | 0.000615 |
| 286.12 | -0.00021 | 0.127905 | -0.00167 | 0.998665 | -0.2509 | 0.250475 | 0.795096 | Congenital deficiency of other clotting factors ( | hematopoietic | 0.00058 |
| 301.1 | 0.00019 | 0.283208 | 0.000672 | 0.999464 | -0.55489 | 0.555267 | 0.72625 | Schizoid personality disorder | mental disorders | 0.000233 |

| phecode | Coef. | Std.Err. | z | p_value_z | [0.025 | 0.975] | GIA | phenotype | category |
|---|---|---|---|---|---|---|---|---|---|
| 278.1 | 0.133218 | 0.026162 | 5.091985 | 3.54E-07 | 0.081941 | 0.184496 | EUR | Obesity | endocrine/metabolic |
| 317 | 0.230245 | 0.047992 | 4.797588 | 1.61E-06 | 0.136183 | 0.324307 | EUR | Alcohol-related disorders | mental disorders |
| 721 | 0.121378 | 0.025325 | 4.792815 | 1.64E-06 | 0.071742 | 0.171014 | EUR | Spondylosis and allied disorders | musculoskeletal |
| 278.11 | 0.160765 | 0.03418 | 4.703534 | 2.56E-06 | 0.093774 | 0.227756 | EUR | Morbid obesity | endocrine/metabolic |
| 150 | 0.713963 | 0.152959 | 4.667662 | 3.05E-06 | 0.414168 | 1.013757 | EUR | Cancer of esophagus | neoplasms |
| 228 | -0.12012 | 0.026233 | -4.5789 | 4.67E-06 | -0.17153 | -0.0687 | EUR | Hemangioma and lymphangioma, any site | neoplasms |
| 317.1 | 0.228683 | 0.053292 | 4.291097 | 1.78E-05 | 0.124232 | 0.333134 | EUR | Alcoholism | mental disorders |
| 401 | 0.097681 | 0.023235 | 4.204124 | 2.62E-05 | 0.052142 | 0.14322 | EUR | Hypertension | circulatory system |
| 281.11 | 0.681925 | 0.162936 | 4.185228 | 2.85E-05 | 0.362576 | 1.001274 | EUR | Pernicious anemia | hematopoietic |
| 721.1 | 0.103911 | 0.025692 | 4.044467 | 5.24E-05 | 0.053555 | 0.154266 | EUR | Spondylosis without myelopathy | musculoskeletal |
| 250 | 0.114965 | 0.02863 | 4.01553 | 5.93E-05 | 0.058851 | 0.171078 | EUR | Diabetes mellitus | endocrine/metabolic |
| 250.2 | 0.11687 | 0.029103 | 4.015743 | 5.93E-05 | 0.059829 | 0.17391 | EUR | Type 2 diabetes | endocrine/metabolic |
| 228.1 | -0.10984 | 0.027495 | -3.99499 | 6.47E-05 | -0.16373 | -0.05595 | EUR | Hemangioma of skin and subcutaneous tissue | neoplasms |
| 401.1 | 0.090212 | 0.023181 | 3.891583 | 9.96E-05 | 0.044777 | 0.135646 | EUR | Essential hypertension | circulatory system |
| 512.7 | 0.088159 | 0.022829 | 3.861679 | 0.000113 | 0.043415 | 0.132903 | EUR | Shortness of breath | respiratory |
| 539 | 0.22172 | 0.058866 | 3.766552 | 0.000166 | 0.106346 | 0.337095 | EUR | Bariatric surgery | digestive |
| 198.7 | -0.5447 | 0.14572 | -3.73798 | 0.000186 | -0.8303 | -0.25909 | EUR | Secondary malignant neoplasm of skin | neoplasms |
| 510.2 | 0.347812 | 0.095445 | 3.644123 | 0.000268 | 0.160744 | 0.53488 | EUR | Lung transplant | respiratory |
| 452 | 0.121707 | 0.033828 | 3.597797 | 0.000321 | 0.055405 | 0.188009 | EUR | Other venous embolism and thrombosis | circulatory system |
| 457.2 | 0.21917 | 0.061064 | 3.589167 | 0.000332 | 0.099486 | 0.338854 | EUR | Encounter for long-term (current) use of antiplatelets/antithrombotics | circulatory system |
| 279.1 | 0.122339 | 0.034729 | 3.522664 | 0.000427 | 0.054271 | 0.190407 | EUR | Immunity deficiency | endocrine/metabolic |
| 327.3 | 0.09108 | 0.025878 | 3.519635 | 0.000432 | 0.040361 | 0.141799 | EUR | Sleep apnea | neurological |
| 428.1 | 0.142 | 0.040587 | 3.498653 | 0.000468 | 0.062451 | 0.22155 | EUR | Congestive heart failure (CHF) NOS | circulatory system |
| 217.1 | -0.10844 | 0.031555 | -3.43669 | 0.000589 | -0.17029 | -0.0466 | EUR | Nevus, non-neoplastic | neoplasms |
| 597.8 | 0.589874 | 0.172348 | 3.422583 | 0.00062 | 0.252079 | 0.92767 | EUR | Urethral hypermobility/ISD | genitourinary |
| 274.2 | 0.26033 | 0.076509 | 3.4026 | 0.000667 | 0.110375 | 0.410286 | EUR | Crystal arthropathies | endocrine/metabolic |
| 411.4 | 0.097077 | 0.028557 | 3.399474 | 0.000675 | 0.041107 | 0.153047 | EUR | Coronary atherosclerosis | circulatory system |
| 274.21 | 0.2626 | 0.07778 | 3.376171 | 0.000735 | 0.110153 | 0.415047 | EUR | Chondrocalcinosis | endocrine/metabolic |
| 278 | 0.077191 | 0.022864 | 3.376061 | 0.000735 | 0.032378 | 0.122004 | EUR | Overweight, obesity and other hyperalimentation | endocrine/metabolic |
| 618.5 | 0.573122 | 0.169822 | 3.374833 | 0.000739 | 0.240277 | 0.905968 | EUR | Prolapse of vaginal vault after hysterectomy | genitourinary |
| 440.22 | 0.434841 | 0.130241 | 3.338747 | 0.000842 | 0.179574 | 0.690108 | EUR | Atherosclerosis of native arteries of the extremities with intermittent claudication | circulatory system |
| 389.1 | -0.11737 | 0.035194 | -3.33492 | 0.000853 | -0.18635 | -0.04839 | EUR | Sensorineural hearing loss | sense organs |
| 279 | 0.111433 | 0.033596 | 3.316895 | 0.00091 | 0.045587 | 0.17728 | EUR | Disorders involving the immune mechanism | endocrine/metabolic |
| 513 | 0.135222 | 0.04125 | 3.278111 | 0.001045 | 0.054374 | 0.21607 | EUR | Respiratory abnormalities | respiratory |
| 418 | 0.073241 | 0.02236 | 3.275535 | 0.001055 | 0.029416 | 0.117067 | EUR | Nonspecific chest pain | circulatory system |
| 217 | -0.1019 | 0.031146 | -3.27156 | 0.00107 | -0.16294 | -0.04085 | EUR | Vascular hamartomas and non-neoplastic nevi | neoplasms |
| 601.12 | -0.30989 | 0.095874 | -3.23232 | 0.001228 | -0.4978 | -0.12199 | EUR | Chronic prostatitis | genitourinary |
| 650 | -0.19142 | 0.059274 | -3.22949 | 0.00124 | -0.3076 | -0.07525 | EUR | Normal delivery | pregnancy complications |
| 529.6 | -0.38447 | 0.120187 | -3.19892 | 0.001379 | -0.62003 | -0.14891 | EUR | Glossodynia | digestive |
| 964.1 | 0.603011 | 0.189249 | 3.18634 | 0.001441 | 0.23209 | 0.973932 | EUR | Anticoagulants causing adverse effects | injuries & poisonings |
| 571 | 0.090295 | 0.028506 | 3.167611 | 0.001537 | 0.034425 | 0.146165 | EUR | Chronic liver disease and cirrhosis | digestive |
| 655 | -0.16214 | 0.051452 | -3.15134 | 0.001625 | -0.26299 | -0.0613 | EUR | Known or suspected fetal abnormality affecting management of mother | pregnancy complications |
| 504.1 | 0.275097 | 0.087543 | 3.142429 | 0.001676 | 0.103516 | 0.446678 | EUR | Idiopathic fibrosing alveolitis | respiratory |
| 756.5 | 0.680652 | 0.217032 | 3.136183 | 0.001712 | 0.255277 | 1.106027 | EUR | Congenital osteodystrophies | congenital anomalies |
| 510 | 0.101151 | 0.032456 | 3.116521 | 0.00183 | 0.037538 | 0.164764 | EUR | Other diseases of lung | respiratory |
| 655.1 | -0.21104 | 0.068218 | -3.09361 | 0.001977 | -0.34475 | -0.07733 | EUR | Abnormality in fetal heart rate or rhythm | pregnancy complications |
| 695.41 | 0.459062 | 0.148468 | 3.092004 | 0.001988 | 0.168071 | 0.750054 | EUR | Cutaneous lupus erythematosus | dermatologic |
| 503 | 0.130925 | 0.042412 | 3.087012 | 0.002022 | 0.0478 | 0.21405 | EUR | Pulmonary congestion and hypostasis | respiratory |
| 416 | 0.100235 | 0.03254 | 3.080404 | 0.002067 | 0.036459 | 0.164011 | EUR | Cardiomegaly | circulatory system |
| 442.11 | -0.31444 | 0.102777 | -3.05945 | 0.002217 | -0.51588 | -0.113 | EUR | Abdominal aortic aneurysm | circulatory system |
| 327.32 | 0.084981 | 0.028279 | 3.005154 | 0.002654 | 0.029556 | 0.140406 | EUR | Obstructive sleep apnea | neurological |
| 965.1 | 0.102967 | 0.034345 | 2.998072 | 0.002717 | 0.035653 | 0.170281 | EUR | Opiates and related narcotics causing adverse effects in therapeutic use | injuries & poisonings |
| 722.6 | 0.075662 | 0.025318 | 2.988457 | 0.002804 | 0.026039 | 0.125284 | EUR | Degeneration of intervertebral disc | musculoskeletal |
| 362.8 | -0.39379 | 0.131838 | -2.98689 | 0.002818 | -0.65218 | -0.13539 | EUR | Retinal hemorrhage/ischemia | sense organs |
| 701.1 | -0.13784 | 0.046264 | -2.97954 | 0.002887 | -0.22852 | -0.04717 | EUR | Keratoderma, acquired | dermatologic |
| 741.5 | 0.836823 | 0.281197 | 2.975928 | 0.002921 | 0.285686 | 1.38796 | EUR | Hemarthrosis | musculoskeletal |
| 599.5 | -0.0841 | 0.028264 | -2.97565 | 0.002924 | -0.1395 | -0.02871 | EUR | Frequency of urination and polyuria | genitourinary |
| 571.5 | 0.084748 | 0.028863 | 2.936219 | 0.003322 | 0.028177 | 0.141318 | EUR | Other chronic nonalcoholic liver disease | digestive |
| 676 | -0.33209 | 0.113111 | -2.93594 | 0.003325 | -0.55378 | -0.11039 | EUR | Other disorders of the breast associated with childbirth and disorders of lactation | pregnancy complications |

| phecode | Coef. | Std.Err. | z | p_value_z | [0.025 | 0.975] | GIA | phenotype | category |
|---|---|---|---|---|---|---|---|---|---|
| 440.1 | 0.489199 | 0.166746 | 2.933795 | 0.003348 | 0.162383 | 0.816016 | EUR | Atherosclerosis of renal artery | circulatory system |
| 531.1 | -0.30619 | 0.10481 | -2.92137 | 0.003485 | -0.51161 | -0.10076 | EUR | Hemorrhage from gastrointestinal ulcer | digestive |
| 415.2 | 0.144128 | 0.049374 | 2.919092 | 0.003511 | 0.047356 | 0.240899 | EUR | Chronic pulmonary heart disease | circulatory system |
| 110 | -0.08671 | 0.029806 | -2.90908 | 0.003625 | -0.14512 | -0.02829 | EUR | Dermatophytosis / Dermatomycosis | infectious diseases |
| 286.5 | 0.680254 | 0.235492 | 2.888654 | 0.003869 | 0.218699 | 1.14181 | EUR | Hemorrhagic disorder due to intrinsic circulating anticoagulants | hematopoietic |
| 426.7 | 0.078926 | 0.027349 | 2.885832 | 0.003904 | 0.025322 | 0.13253 | EUR | Abnormal electrocardiogram [ECG] [EKG] | circulatory system |
| 530 | 0.059822 | 0.020783 | 2.878399 | 0.003997 | 0.019088 | 0.100556 | EUR | Diseases of esophagus | digestive |
| 296.22 | 0.066401 | 0.023091 | 2.875548 | 0.004033 | 0.021142 | 0.111659 | EUR | Major depressive disorder | mental disorders |
| 187.2 | 0.398147 | 0.138509 | 2.874527 | 0.004046 | 0.126675 | 0.669619 | EUR | Malignant neoplasm of testis | neoplasms |
| 530.1 | 0.059681 | 0.020835 | 2.864379 | 0.004178 | 0.018844 | 0.100518 | EUR | Esophagitis, GERD and related diseases | digestive |
| 351 | 0.073098 | 0.025581 | 2.857472 | 0.00427 | 0.022959 | 0.123236 | EUR | Other peripheral nerve disorders | neurological |
| 561.2 | -0.08298 | 0.029126 | -2.84893 | 0.004387 | -0.14006 | -0.02589 | EUR | Flatulence | digestive |
| 513.8 | 0.146307 | 0.051767 | 2.82624 | 0.00471 | 0.044845 | 0.24777 | EUR | Disorders of diaphragm | respiratory |
| 512.3 | 0.374274 | 0.132523 | 2.824226 | 0.00474 | 0.114534 | 0.634013 | EUR | Abnormal chest sounds | respiratory |
| 571.81 | 0.232726 | 0.082586 | 2.81797 | 0.004833 | 0.070859 | 0.394592 | EUR | Portal hypertension | digestive |
| 703 | -0.12033 | 0.043016 | -2.79737 | 0.005152 | -0.20464 | -0.03602 | EUR | Diseases of nail, NOS | dermatologic |
| 401.2 | 0.089024 | 0.031905 | 2.790312 | 0.005266 | 0.026492 | 0.151556 | EUR | Hypertensive heart and/or renal disease | circulatory system |
| 706.2 | -0.09286 | 0.033309 | -2.78774 | 0.005308 | -0.15814 | -0.02757 | EUR | Sebaceous cyst | dermatologic |
| 334.1 | -0.42792 | 0.153627 | -2.78541 | 0.005346 | -0.72902 | -0.12681 | EUR | Spinocerebellar disease | neurological |
| 383 | -0.56324 | 0.202493 | -2.78152 | 0.00541 | -0.96012 | -0.16636 | EUR | Otosclerosis | sense organs |
| 303.4 | 0.332551 | 0.119585 | 2.780886 | 0.005421 | 0.09817 | 0.566933 | EUR | Somatoform disorder | mental disorders |
| 457 | 0.076264 | 0.027443 | 2.779052 | 0.005452 | 0.022478 | 0.130051 | EUR | Encounter for long-term (current) use of anticoagulants, antithrombotics, aspirin | circulatory system |
| 743.1 | -0.0929 | 0.033528 | -2.7709 | 0.00559 | -0.15862 | -0.02719 | EUR | Osteoporosis | musculoskeletal |
| 509.1 | 0.104334 | 0.037672 | 2.769572 | 0.005613 | 0.030499 | 0.178169 | EUR | Respiratory failure | respiratory |
| 1010 | -0.08664 | 0.031314 | -2.76689 | 0.005659 | -0.14802 | -0.02527 | EUR | Other tests | other |
| 502 | 0.142247 | 0.051501 | 2.76205 | 0.005744 | 0.041308 | 0.243186 | EUR | Postinflammatory pulmonary fibrosis | respiratory |
| 318 | 0.144992 | 0.053048 | 2.733225 | 0.006272 | 0.04102 | 0.248964 | EUR | Tobacco use disorder | mental disorders |
| 306.1 | -0.20743 | 0.0759 | -2.73291 | 0.006278 | -0.35619 | -0.05867 | EUR | Mental disorders durring/after pregnancy | mental disorders |
| 626.8 | -0.19912 | 0.073086 | -2.72442 | 0.006442 | -0.34236 | -0.05587 | EUR | Infertility, female | genitourinary |
| 441.2 | 0.563425 | 0.207166 | 2.71968 | 0.006535 | 0.157387 | 0.969463 | EUR | Chronic vascular insufficiency of intestine | circulatory system |
| 754.1 | 0.862037 | 0.31782 | 2.712343 | 0.006681 | 0.239121 | 1.484953 | EUR | Lumbosacral spondylolysis, congenital | congenital anomalies |
| 1014 | 1.414954 | 0.521683 | 2.712288 | 0.006682 | 0.392474 | 2.437434 | EUR | Effects of heat, cold and air pressure | other |
| 1004 | 0.178889 | 0.065992 | 2.710791 | 0.006712 | 0.049548 | 0.30823 | EUR | Other signs and symptoms involving emotional state | other |
| 312.3 | 0.555229 | 0.20492 | 2.709497 | 0.006739 | 0.153594 | 0.956865 | EUR | Impulse control disorder | mental disorders |
| 78 | -0.08765 | 0.032392 | -2.70595 | 0.006811 | -0.15114 | -0.02416 | EUR | Viral warts & HPV | infectious diseases |
| 285.1 | 0.083344 | 0.030806 | 2.705478 | 0.006821 | 0.022966 | 0.143723 | EUR | Acute posthemorrhagic anemia | hematopoietic |
| 611 | -0.08211 | 0.030385 | -2.70228 | 0.006887 | -0.14166 | -0.02256 | EUR | Abnormal findings on mammogram or breast exam | genitourinary |
| 338 | 0.055793 | 0.020657 | 2.700914 | 0.006915 | 0.015306 | 0.096281 | EUR | Pain | neurological |
| 71.1 | 0.313417 | 0.116206 | 2.697081 | 0.006995 | 0.085658 | 0.541177 | EUR | HIV infection, symptomatic | infectious diseases |
| 338.2 | 0.057218 | 0.021231 | 2.695014 | 0.007039 | 0.015606 | 0.098829 | EUR | Chronic pain | neurological |
| 722 | 0.064358 | 0.02396 | 2.686091 | 0.007229 | 0.017398 | 0.111319 | EUR | Intervertebral disc disorders | musculoskeletal |
| 962 | 0.168944 | 0.063134 | 2.675977 | 0.007451 | 0.045204 | 0.292684 | EUR | Poisoning by hormones and synthetic substitutes | injuries & poisonings |
| 731.1 | 1.072941 | 0.402603 | 2.665012 | 0.007699 | 0.283854 | 1.862028 | EUR | Osteitis deformans [Paget's disease of bone] | musculoskeletal |
| 250.22 | 0.137011 | 0.051618 | 2.65432 | 0.007947 | 0.035841 | 0.23818 | EUR | Type 2 diabetes with renal manifestations | endocrine/metabolic |
| 380.4 | -0.08293 | 0.031245 | -2.65432 | 0.007947 | -0.14417 | -0.0217 | EUR | Impacted cerumen | sense organs |
| 626.15 | -0.34633 | 0.130971 | -2.64435 | 0.008185 | -0.60303 | -0.08963 | EUR | Infertility, female, associated with anovulation | genitourinary |
| 411 | 0.072113 | 0.027279 | 2.643503 | 0.008205 | 0.018647 | 0.12558 | EUR | Ischemic Heart Disease | circulatory system |
| 765 | 0.10362 | 0.039382 | 2.631181 | 0.008509 | 0.026434 | 0.180806 | EUR | Cervical radiculitis | symptoms |
| 224.1 | -0.23294 | 0.088587 | -2.62952 | 0.008551 | -0.40657 | -0.05931 | EUR | Benign neoplasm of eye, uveal | neoplasms |
| 627 | -0.07383 | 0.02811 | -2.62658 | 0.008625 | -0.12893 | -0.01874 | EUR | Menopausal and postmenopausal disorders | genitourinary |
| 521 | 0.334941 | 0.127524 | 2.626506 | 0.008627 | 0.085 | 0.584883 | EUR | Diseases of hard tissues of teeth | digestive |
| 433.32 | 1.624659 | 0.62103 | 2.616072 | 0.008895 | 0.407463 | 2.841855 | EUR | Moyamoya disease | circulatory system |
| 110.1 | -0.08171 | 0.031245 | -2.61501 | 0.008923 | -0.14294 | -0.02047 | EUR | Dermatophytosis | infectious diseases |
| 446.2 | 1.116697 | 0.427441 | 2.612516 | 0.008988 | 0.278928 | 1.954466 | EUR | Acute febrile mucocutaneous lymph node syndrome (Kawasaki disease) | circulatory system |
| 613.7 | 0.221665 | 0.08487 | 2.611809 | 0.009006 | 0.055322 | 0.388008 | EUR | Other signs and symptoms in breast | genitourinary |
| 303.1 | 0.542012 | 0.208402 | 2.600799 | 0.009301 | 0.133551 | 0.950472 | EUR | Dissociative disorder | mental disorders |
| 521.1 | 0.350946 | 0.13537 | 2.592496 | 0.009528 | 0.085626 | 0.616267 | EUR | Dental caries | digestive |
| 706 | -0.06472 | 0.025018 | -2.58675 | 0.009688 | -0.11375 | -0.01568 | EUR | Diseases of sebaceous glands | dermatologic |
| 457.3 | 0.071729 | 0.027756 | 2.584239 | 0.009759 | 0.017327 | 0.12613 | EUR | Encounter for long-term (current) use of aspirin | circulatory system |

| phecode | Coef. | Std.Err. | z | p_value_z | [0.025 | 0.975] | GIA | phenotype | category |
|---|---|---|---|---|---|---|---|---|---|
| 512.2 | 0.095069 | 0.036883 | 2.577611 | 0.009949 | 0.02278 | 0.167358 | EUR | Painful respiration | respiratory |
| 292.5 | 0.243167 | 0.094411 | 2.575606 | 0.010006 | 0.058124 | 0.42821 | EUR | Transient alteration of awareness | mental disorders |
| 726.4 | 0.148168 | 0.057561 | 2.574131 | 0.010049 | 0.035352 | 0.260985 | EUR | Calcaneal spur; Exostosis NOS | musculoskeletal |
| 724.9 | 0.170665 | 0.066334 | 2.572826 | 0.010087 | 0.040653 | 0.300677 | EUR | Other unspecified back disorders | musculoskeletal |
| 506 | 0.112417 | 0.043711 | 2.571847 | 0.010116 | 0.026746 | 0.198088 | EUR | Empyema and pneumothorax | respiratory |
| 283.2 | 0.612351 | 0.23879 | 2.564397 | 0.010336 | 0.144332 | 1.08037 | EUR | Non-autoimmune hemolytic anemias | hematopoietic |
| 130 | -0.30459 | 0.118969 | -2.56028 | 0.010459 | -0.53777 | -0.07142 | EUR | Spirochetal infection | infectious diseases |
| 415 | 0.099864 | 0.039055 | 2.556976 | 0.010559 | 0.023317 | 0.176411 | EUR | Pulmonary heart disease | circulatory system |
| 875 | -0.39456 | 0.154318 | -2.55679 | 0.010564 | -0.69702 | -0.0921 | EUR | Non-healing surgical wound | injuries & poisonings |
| 327.4 | 0.062952 | 0.024627 | 2.556212 | 0.010582 | 0.014684 | 0.11122 | EUR | Insomnia | neurological |
| 250.3 | 0.118651 | 0.046423 | 2.555888 | 0.010592 | 0.027664 | 0.209637 | EUR | Insulin pump user | endocrine/metabolic |
| 717 | -0.31721 | 0.124367 | -2.55058 | 0.010754 | -0.56096 | -0.07345 | EUR | Polymyalgia Rheumatica | musculoskeletal |
| 288.11 | 0.100579 | 0.039565 | 2.542103 | 0.011019 | 0.023033 | 0.178126 | EUR | Neutropenia | hematopoietic |
| 145.1 | 0.917717 | 0.362463 | 2.531894 | 0.011345 | 0.207303 | 1.62813 | EUR | Cancer of lip | neoplasms |
| 259.8 | 0.776994 | 0.307016 | 2.530795 | 0.01138 | 0.175254 | 1.378734 | EUR | Polyglandular activity in multiple endocrine adenomatosis | endocrine/metabolic |
| 735 | -0.08157 | 0.03223 | -2.5307 | 0.011383 | -0.14474 | -0.0184 | EUR | Acquired foot deformities | musculoskeletal |
| 580 | 0.123918 | 0.049006 | 2.528624 | 0.011451 | 0.027868 | 0.219968 | EUR | Nephritis; nephrosis; renal sclerosis | genitourinary |
| 81.1 | 0.402112 | 0.159116 | 2.527165 | 0.011499 | 0.090251 | 0.713973 | EUR | Graft-versus-host disease | infectious diseases |
| 276.6 | 0.11963 | 0.04743 | 2.522251 | 0.011661 | 0.026669 | 0.212591 | EUR | Fluid overload | endocrine/metabolic |
| 580.31 | 0.177886 | 0.070638 | 2.518258 | 0.011794 | 0.039437 | 0.316334 | EUR | Nephritis and nephropathy in diseases classified elsewhere | genitourinary |
| 130.1 | -0.35658 | 0.141613 | -2.51801 | 0.011802 | -0.63414 | -0.07903 | EUR | Lyme disease | infectious diseases |
| 627.2 | -0.07426 | 0.029581 | -2.51042 | 0.012059 | -0.13224 | -0.01628 | EUR | Symptomatic menopause | genitourinary |
| 523.1 | 0.31626 | 0.126112 | 2.507773 | 0.012149 | 0.069085 | 0.563434 | EUR | Gingivitis | digestive |
| 525.2 | 1.565232 | 0.6244 | 2.506778 | 0.012184 | 0.34143 | 2.789033 | EUR | Atrophy of edentulous alveolar ridge | digestive |
| 514 | 0.057208 | 0.022881 | 2.500239 | 0.012411 | 0.012362 | 0.102055 | EUR | Abnormal findings examination of lungs | respiratory |
| 509 | 0.089509 | 0.036005 | 2.486046 | 0.012917 | 0.018941 | 0.160077 | EUR | Respiratory failure, insufficiency, arrest | respiratory |
| 665 | -0.14105 | 0.057008 | -2.47412 | 0.013356 | -0.25278 | -0.02931 | EUR | Obstetrical/birth trauma | pregnancy complications |
| 250.14 | 0.360972 | 0.145984 | 2.472691 | 0.01341 | 0.07485 | 0.647095 | EUR | Type 1 diabetes with neurological manifestations | endocrine/metabolic |
| 530.11 | 0.051716 | 0.020916 | 2.472575 | 0.013414 | 0.010722 | 0.09271 | EUR | GERD | digestive |
| 740.9 | 0.059734 | 0.024223 | 2.466009 | 0.013663 | 0.012258 | 0.107211 | EUR | Osteoarthrosis NOS | musculoskeletal |
| 716.9 | 0.063338 | 0.025734 | 2.461237 | 0.013846 | 0.0129 | 0.113776 | EUR | Arthropathy NOS | musculoskeletal |
| 253.4 | -0.36393 | 0.148257 | -2.45472 | 0.0141 | -0.65451 | -0.07335 | EUR | Anterior pituitary disorders | endocrine/metabolic |
| 507 | 0.070438 | 0.028698 | 2.454455 | 0.01411 | 0.014191 | 0.126686 | EUR | Pleurisy; pleural effusion | respiratory |
| 661 | -0.19379 | 0.07897 | -2.45401 | 0.014127 | -0.34857 | -0.03901 | EUR | Fetal distress and abnormal forces of labor | pregnancy complications |
| 562 | 0.068187 | 0.0278 | 2.45281 | 0.014175 | 0.013701 | 0.122674 | EUR | Diverticulosis and diverticulitis | digestive |
| 379.5 | -0.26187 | 0.106957 | -2.44839 | 0.01435 | -0.4715 | -0.05224 | EUR | Disorders of iris and ciliary body | sense organs |
| 117.3 | 1.438518 | 0.588772 | 2.44325 | 0.014556 | 0.284546 | 2.59249 | EUR | Blastomycotic infection | infectious diseases |
| 585.33 | 0.088614 | 0.036337 | 2.4387 | 0.01474 | 0.017396 | 0.159832 | EUR | Chronic Kidney Disease, Stage III | genitourinary |
| 590 | 0.13913 | 0.057088 | 2.437103 | 0.014805 | 0.027239 | 0.25102 | EUR | Pyelonephritis | genitourinary |
| 225.2 | 0.53077 | 0.217897 | 2.435876 | 0.014856 | 0.1037 | 0.957841 | EUR | Benign neoplasm of spinal cord, meninges | neoplasms |
| 327 | 0.054257 | 0.022407 | 2.42148 | 0.015457 | 0.010341 | 0.098173 | EUR | Sleep disorders | neurological |
| 741.1 | -0.50443 | 0.20891 | -2.4146 | 0.015752 | -0.91389 | -0.09498 | EUR | Ankylosis of joint | musculoskeletal |
| 571.8 | 0.16982 | 0.070382 | 2.412834 | 0.015829 | 0.031874 | 0.307765 | EUR | Liver abscess and sequelae of chronic liver disease | digestive |
| 580.3 | 0.131036 | 0.054933 | 2.385387 | 0.017061 | 0.02337 | 0.238702 | EUR | Nephritis and nephropathy without mention of glomerulonephritis | genitourinary |
| 250.12 | 0.3265 | 0.137138 | 2.380816 | 0.017274 | 0.057715 | 0.595286 | EUR | Type 1 diabetes with renal manifestations | endocrine/metabolic |
| 962.1 | 0.162386 | 0.068225 | 2.380154 | 0.017305 | 0.028667 | 0.296104 | EUR | Adrenal cortical steroids causing adverse effects in therapeutic use | injuries & poisonings |
| 313.2 | -0.50325 | 0.211774 | -2.37635 | 0.017485 | -0.91832 | -0.08818 | EUR | Tics and stuttering | mental disorders |
| 716 | 0.060517 | 0.025482 | 2.374917 | 0.017553 | 0.010574 | 0.110461 | EUR | Other arthropathies | musculoskeletal |
| 604 | 0.192763 | 0.081357 | 2.369357 | 0.017819 | 0.033307 | 0.35222 | EUR | Disorders of penis | genitourinary |
| 260.2 | 0.145084 | 0.061337 | 2.365371 | 0.018012 | 0.024866 | 0.265302 | EUR | severe protein-calorie malnutrition | endocrine/metabolic |
| 573.3 | 0.107066 | 0.04527 | 2.365028 | 0.018029 | 0.018337 | 0.195794 | EUR | Hepatomegaly | digestive |
| 284.2 | 0.779173 | 0.329784 | 2.362676 | 0.018144 | 0.132808 | 1.425538 | EUR | Constitutional aplastic anemia | hematopoietic |
| 624.2 | 0.491617 | 0.208445 | 2.3585 | 0.018349 | 0.083073 | 0.900162 | EUR | Atrophy of female genital tract | genitourinary |
| 495.11 | 0.412545 | 0.17516 | 2.355246 | 0.01851 | 0.069238 | 0.755852 | EUR | Chronic obstructive asthma with exacerbation | respiratory |
| 361.1 | -0.21667 | 0.092032 | -2.35435 | 0.018555 | -0.39705 | -0.0363 | EUR | Retinal detachment with retinal defect | sense organs |
| 694 | -0.05817 | 0.024817 | -2.34398 | 0.019079 | -0.10681 | -0.00953 | EUR | Dyschromia and Vitiligo | dermatologic |
| 1011 | 0.098665 | 0.042153 | 2.340651 | 0.01925 | 0.016047 | 0.181284 | EUR | Complications of surgical and medical procedures | other |
| 710.19 | 0.206519 | 0.088584 | 2.331336 | 0.019736 | 0.032898 | 0.38014 | EUR | Unspecified osteomyelitis | musculoskeletal |
| 426 | 0.059004 | 0.025321 | 2.33025 | 0.019793 | 0.009376 | 0.108632 | EUR | Cardiac conduction disorders | circulatory system |

| phecode | Coef. | Std.Err. | z | p_value_z | [0.025 | 0.975] | GIA | phenotype | category |
|---|---|---|---|---|---|---|---|---|---|
| 514.2 | 0.076812 | 0.032972 | 2.329621 | 0.019826 | 0.012188 | 0.141437 | EUR | Solitary pulmonary nodule | respiratory |
| 337 | 0.180601 | 0.077533 | 2.329336 | 0.019841 | 0.028639 | 0.332564 | EUR | Disorders of the autonomic nervous system | neurological |
| 216 | -0.05066 | 0.021798 | -2.3241 | 0.02012 | -0.09338 | -0.00794 | EUR | Benign neoplasm of skin | neoplasms |
| 728.1 | 0.581857 | 0.251356 | 2.314876 | 0.02062 | 0.089209 | 1.074505 | EUR | Muscular calcification and ossification | musculoskeletal |
| 610 | -0.10214 | 0.044354 | -2.30293 | 0.021283 | -0.18907 | -0.01521 | EUR | Benign mammary dysplasias | genitourinary |
| 380 | -0.20026 | 0.087188 | -2.29689 | 0.021625 | -0.37115 | -0.02938 | EUR | Disorders of external ear | sense organs |
| 110.12 | -0.12627 | 0.055176 | -2.28846 | 0.022111 | -0.23441 | -0.01813 | EUR | Althete's foot | infectious diseases |
| 276.14 | 0.090188 | 0.039455 | 2.285858 | 0.022263 | 0.012858 | 0.167518 | EUR | Hypopotassemia | endocrine/metabolic |
| 585.3 | 0.069911 | 0.030715 | 2.276142 | 0.022837 | 0.009711 | 0.130111 | EUR | Chronic renal failure [CKD] | genitourinary |
| 379.4 | 0.260416 | 0.114775 | 2.268926 | 0.023273 | 0.035461 | 0.485371 | EUR | Anomalies of pupillary function | sense organs |
| 761 | 0.05835 | 0.02573 | 2.267757 | 0.023344 | 0.00792 | 0.108781 | EUR | Cervicalgia | symptoms |
| 480.12 | 0.312951 | 0.138291 | 2.262994 | 0.023636 | 0.041906 | 0.583995 | EUR | Pseudomonal pneumonia | respiratory |
| 296.2 | 0.050126 | 0.022162 | 2.261747 | 0.023713 | 0.006688 | 0.093564 | EUR | Depression | mental disorders |
| 743.11 | -0.07714 | 0.034105 | -2.2617 | 0.023716 | -0.14398 | -0.01029 | EUR | Osteoporosis NOS | musculoskeletal |
| 331.9 | -0.24844 | 0.109967 | -2.25919 | 0.023872 | -0.46397 | -0.0329 | EUR | Cerebral degeneration, unspecified | neurological |
| 286.2 | 0.073419 | 0.032507 | 2.258554 | 0.023911 | 0.009706 | 0.137132 | EUR | Encounter for long-term (current) use of anticoagulants | hematopoietic |
| 626.11 | -0.1305 | 0.057831 | -2.25653 | 0.024037 | -0.24384 | -0.01715 | EUR | Absent or infrequent menstruation | genitourinary |
| 224 | -0.18649 | 0.082643 | -2.25654 | 0.024037 | -0.34846 | -0.02451 | EUR | Benign neoplasm of eye | neoplasms |
| 1013 | 0.087766 | 0.038956 | 2.252921 | 0.024264 | 0.011413 | 0.164119 | EUR | Asphyxia and hypoxemia | other |
| 694.2 | -0.05628 | 0.02504 | -2.2477 | 0.024595 | -0.10536 | -0.00721 | EUR | Other dyschromia | dermatologic |
| 151 | 0.284708 | 0.126925 | 2.243122 | 0.024889 | 0.03594 | 0.533477 | EUR | Cancer of stomach | neoplasms |
| 962.2 | 0.647089 | 0.289501 | 2.235188 | 0.025405 | 0.079678 | 1.2145 | EUR | Insulins and antidiabetic agents causing adverse effects in therapeutic use | injuries & poisonings |
| 349 | -0.11525 | 0.051613 | -2.23294 | 0.025553 | -0.21641 | -0.01409 | EUR | Other and unspecified disorders of the nervous system | neurological |
| 250.24 | 0.117377 | 0.052623 | 2.230537 | 0.025712 | 0.014238 | 0.220516 | EUR | Type 2 diabetes with neurological manifestations | endocrine/metabolic |
| 260.21 | 0.938726 | 0.42199 | 2.224523 | 0.026113 | 0.111641 | 1.76581 | EUR | Kwashiorkor | endocrine/metabolic |
| 535.9 | 0.108369 | 0.048749 | 2.223015 | 0.026215 | 0.012823 | 0.203915 | EUR | Gastritis and duodenitis, NOS | digestive |
| 222 | 0.450305 | 0.202599 | 2.222643 | 0.02624 | 0.053218 | 0.847391 | EUR | Benign neoplasm of male genital organs | neoplasms |
| 743.12 | -0.10254 | 0.046206 | -2.21917 | 0.026475 | -0.1931 | -0.01198 | EUR | Senile osteoporosis | musculoskeletal |
| 764 | 0.071375 | 0.032173 | 2.218447 | 0.026524 | 0.008316 | 0.134433 | EUR | Sciatica | symptoms |
| 450 | 0.159493 | 0.071917 | 2.217745 | 0.026572 | 0.018539 | 0.300446 | EUR | Noninfectious disorders of lymphatic channels | circulatory system |
| 285.2 | 0.079747 | 0.035993 | 2.215621 | 0.026717 | 0.009202 | 0.150292 | EUR | Anemia of chronic disease | hematopoietic |
| 715 | 0.097809 | 0.044216 | 2.21209 | 0.02696 | 0.011148 | 0.18447 | EUR | Other inflammatory spondylopathies | musculoskeletal |
| 170.1 | 0.196273 | 0.089039 | 2.204362 | 0.027499 | 0.021761 | 0.370786 | EUR | Bone cancer | neoplasms |
| 370.2 | -0.2455 | 0.111409 | -2.20356 | 0.027555 | -0.46385 | -0.02714 | EUR | Superficial keratitis | sense organs |
| 496.2 | 0.146589 | 0.066586 | 2.201506 | 0.0277 | 0.016083 | 0.277095 | EUR | Chronic bronchitis | respiratory |
| 428 | 0.080671 | 0.036705 | 2.197812 | 0.027962 | 0.00873 | 0.152613 | EUR | Congestive heart failure; nonhypertensive | circulatory system |
| 858 | 0.14625 | 0.066548 | 2.197662 | 0.027973 | 0.015818 | 0.276683 | EUR | Complication of internal orthopedic device | injuries & poisonings |
| 415.21 | 0.17717 | 0.080676 | 2.196081 | 0.028086 | 0.019049 | 0.335292 | EUR | Primary pulmonary hypertension | circulatory system |
| 603.2 | -0.23414 | 0.106715 | -2.19407 | 0.02823 | -0.4433 | -0.02498 | EUR | Spermatocele | genitourinary |
| 526.5 | 0.479462 | 0.218866 | 2.190667 | 0.028476 | 0.050493 | 0.90843 | EUR | Inflammatory conditions of jaw | digestive |
| 681.2 | 0.189478 | 0.086498 | 2.190546 | 0.028485 | 0.019945 | 0.359011 | EUR | Cellulitis and abscess of face/neck | dermatologic |
| 270.32 | -0.16955 | 0.077619 | -2.18441 | 0.028932 | -0.32168 | -0.01742 | EUR | Paraproteinemia | endocrine/metabolic |
| 504 | 0.14204 | 0.065048 | 2.183613 | 0.028991 | 0.014548 | 0.269533 | EUR | Other alveolar and parietoalveolar pneumonopathy | respiratory |
| 740.1 | 0.053573 | 0.024542 | 2.182919 | 0.029042 | 0.005472 | 0.101675 | EUR | Osteoarthritis; localized | musculoskeletal |
| 272.12 | 0.12135 | 0.055597 | 2.182673 | 0.02906 | 0.012382 | 0.230318 | EUR | Hyperglyceridemia | endocrine/metabolic |
| 740.3 | 0.17775 | 0.081535 | 2.180052 | 0.029254 | 0.017945 | 0.337555 | EUR | Osteoarthrosis involving more than one site, but not specified as generalized | musculoskeletal |
| 649 | -0.11164 | 0.051391 | -2.1724 | 0.029826 | -0.21237 | -0.01092 | EUR | Other conditions or status of the mother complicating pregnancy, childbirth, or the puerperium | pregnancy complications |
| 249 | 0.121213 | 0.055809 | 2.171941 | 0.02986 | 0.01183 | 0.230596 | EUR | Secondary diabetes mellitus | endocrine/metabolic |
| 516.1 | 0.169583 | 0.078129 | 2.170546 | 0.029965 | 0.016453 | 0.322713 | EUR | Hemoptysis | respiratory |
| 710 | 0.178966 | 0.082528 | 2.168556 | 0.030116 | 0.017215 | 0.340718 | EUR | Osteomyelitis, periostitis, and other infections involving bone | musculoskeletal |
| 402 | -0.06966 | 0.032143 | -2.16709 | 0.030228 | -0.13266 | -0.00666 | EUR | Elevated blood pressure reading without diagnosis of hypertension | circulatory system |
| 577.1 | 0.158366 | 0.07324 | 2.162295 | 0.030595 | 0.014819 | 0.301913 | EUR | Acute pancreatitis | digestive |
| 614 | -0.07982 | 0.036919 | -2.16197 | 0.030621 | -0.15218 | -0.00746 | EUR | Inflammatory diseases of female pelvic organs | genitourinary |
| 374.2 | 0.406072 | 0.187961 | 2.160402 | 0.030742 | 0.037675 | 0.774469 | EUR | Lagophthalmos | sense organs |
| 395.4 | 0.223688 | 0.103589 | 2.159389 | 0.03082 | 0.020658 | 0.426718 | EUR | Nonrheumatic pulmonary valve disorders | circulatory system |
| 323 | -0.23857 | 0.110715 | -2.15485 | 0.031173 | -0.45557 | -0.02158 | EUR | Encephalitis | neurological |
| 332 | -0.19112 | 0.088953 | -2.14856 | 0.031669 | -0.36547 | -0.01678 | EUR | Parkinson's disease | neurological |
| 797.1 | 0.170858 | 0.079528 | 2.1484 | 0.031682 | 0.014986 | 0.32673 | EUR | Cardiogenic shock | symptoms |
| 189.1 | 0.166359 | 0.07748 | 2.147114 | 0.031784 | 0.014501 | 0.318218 | EUR | Cancer of kidney and renal pelvis | neoplasms |

| phecode | Coef. | Std.Err. | z | p_value_z | [0.025 | 0.975] | GIA | phenotype | category |
|---|---|---|---|---|---|---|---|---|---|
| 295.3 | 0.160805 | 0.074964 | 2.14509 | 0.031946 | 0.013878 | 0.307733 | EUR | Psychosis | mental disorders |
| 710.11 | 0.269586 | 0.125815 | 2.142709 | 0.032136 | 0.022992 | 0.516179 | EUR | Acute osteomyelitis | musculoskeletal |
| 782.3 | 0.060154 | 0.0281 | 2.140754 | 0.032294 | 0.00508 | 0.115228 | EUR | Edema | symptoms |
| 736.3 | 0.532645 | 0.249227 | 2.137191 | 0.032582 | 0.04417 | 1.02112 | EUR | Acquired deformities of hip | musculoskeletal |
| 244.2 | -0.07476 | 0.035016 | -2.13509 | 0.032753 | -0.14339 | -0.00613 | EUR | Acquired hypothyroidism | endocrine/metabolic |
| 686.4 | -0.29579 | 0.138579 | -2.13444 | 0.032807 | -0.5674 | -0.02418 | EUR | Pyogenic granuloma | dermatologic |
| 285.21 | 0.114728 | 0.053798 | 2.132552 | 0.032961 | 0.009285 | 0.22017 | EUR | Anemia in chronic kidney disease | hematopoietic |
| 411.2 | 0.097275 | 0.045784 | 2.124654 | 0.033616 | 0.00754 | 0.187009 | EUR | Myocardial infarction | circulatory system |
| 724 | 0.073773 | 0.034854 | 2.116669 | 0.034288 | 0.005462 | 0.142085 | EUR | Other and unspecified disorders of back | musculoskeletal |
| 522.5 | 0.235556 | 0.111413 | 2.114268 | 0.034492 | 0.017191 | 0.453921 | EUR | Periapical abscess | digestive |
| 740 | 0.04961 | 0.023489 | 2.112067 | 0.034681 | 0.003573 | 0.095648 | EUR | Osteoarthrosis | musculoskeletal |
| 500.2 | 0.695643 | 0.329369 | 2.112046 | 0.034682 | 0.050091 | 1.341196 | EUR | Pneumoconiosis | respiratory |
| 246.7 | -0.10592 | 0.050336 | -2.10421 | 0.03536 | -0.20458 | -0.00726 | EUR | Abnormal results of function study of thyroid | endocrine/metabolic |
| 781.1 | 0.283996 | 0.13509 | 2.102276 | 0.035529 | 0.019225 | 0.548767 | EUR | Loss of height | symptoms |
| 362.31 | 0.415077 | 0.197746 | 2.099041 | 0.035813 | 0.027502 | 0.802652 | EUR | Separation of retinal layers | sense organs |
| 740.11 | 0.051572 | 0.024649 | 2.092244 | 0.036417 | 0.003261 | 0.099884 | EUR | Osteoarthrosis, localized, primary | musculoskeletal |
| 395.3 | 0.124998 | 0.059773 | 2.091205 | 0.03651 | 0.007845 | 0.242151 | EUR | Nonrheumatic tricuspid valve disorders | circulatory system |
| 727.8 | 0.558788 | 0.267574 | 2.088354 | 0.036766 | 0.034354 | 1.083223 | EUR | Plica syndrome | musculoskeletal |
| 870.2 | -0.33228 | 0.159197 | -2.08723 | 0.036867 | -0.6443 | -0.02026 | EUR | Open wound of ear | injuries & poisonings |
| 789.1 | 0.162718 | 0.078483 | 2.073285 | 0.038146 | 0.008894 | 0.316542 | EUR | Persistent vomiting | symptoms |
| 871.3 | -0.28796 | 0.138982 | -2.07193 | 0.038272 | -0.56036 | -0.01556 | EUR | Open wound of foot except toe(s) alone | injuries & poisonings |
| 695.9 | -0.18412 | 0.088966 | -2.06954 | 0.038495 | -0.35849 | -0.00975 | EUR | Unspecified erythematous condition | dermatologic |
| 743.13 | -0.12703 | 0.061387 | -2.06939 | 0.03851 | -0.24735 | -0.00672 | EUR | Other specified osteoporosis | musculoskeletal |
| 70 | 0.147199 | 0.071188 | 2.067762 | 0.038662 | 0.007674 | 0.286724 | EUR | Viral hepatitis | infectious diseases |
| 317.11 | 0.243205 | 0.117705 | 2.066228 | 0.038807 | 0.012508 | 0.473903 | EUR | Alcoholic liver damage | mental disorders |
| 189.12 | 0.430631 | 0.208451 | 2.065864 | 0.038841 | 0.022075 | 0.839186 | EUR | Malignant neoplasm of renal pelvis | neoplasms |
| 375.1 | -0.06947 | 0.033647 | -2.06452 | 0.038968 | -0.13541 | -0.00352 | EUR | Dry eyes | sense organs |
| 870.8 | 0.504383 | 0.245455 | 2.054888 | 0.03989 | 0.023299 | 0.985466 | EUR | Open wound of genital organs | injuries & poisonings |
| 264.1 | 0.864709 | 0.421818 | 2.049958 | 0.040369 | 0.037961 | 1.691456 | EUR | Short stature | endocrine/metabolic |
| 300.12 | 0.111185 | 0.054273 | 2.04862 | 0.040499 | 0.004812 | 0.217557 | EUR | Agorophobia, social phobia, and panic disorder | mental disorders |
| 513.32 | 0.29324 | 0.143267 | 2.046813 | 0.040676 | 0.012443 | 0.574038 | EUR | Orthopnea | respiratory |
| 508 | 0.053308 | 0.02606 | 2.045537 | 0.040802 | 0.00223 | 0.104385 | EUR | Pulmonary collapse; interstitial and compensatory emphysema | respiratory |
| 388 | -0.08288 | 0.040602 | -2.04119 | 0.041231 | -0.16245 | -0.0033 | EUR | Other disorders of ear | sense organs |
| 736.4 | 0.362716 | 0.177881 | 2.039102 | 0.04144 | 0.014077 | 0.711356 | EUR | Genu valgum or varum (acquired) | musculoskeletal |
| 610.2 | -0.25376 | 0.12479 | -2.03347 | 0.042005 | -0.49834 | -0.00917 | EUR | Fibroadenosis of breast | genitourinary |
| 352 | 0.14244 | 0.070088 | 2.032317 | 0.042122 | 0.005071 | 0.279809 | EUR | Disorders of other cranial nerves | neurological |
| 250.6 | 0.119994 | 0.059093 | 2.0306 | 0.042296 | 0.004174 | 0.235815 | EUR | Polyneuropathy in diabetes | endocrine/metabolic |
| 230 | 0.519515 | 0.255859 | 2.030474 | 0.042308 | 0.018041 | 1.02099 | EUR | Kaposi's sarcoma | neoplasms |
| 420.2 | 0.103871 | 0.051174 | 2.029775 | 0.042379 | 0.003572 | 0.20417 | EUR | Pericarditis | circulatory system |
| 528.11 | -0.19692 | 0.097039 | -2.02932 | 0.042425 | -0.38712 | -0.00673 | EUR | Stomatitis and mucositis (ulcerative) | digestive |
| 216.1 | -0.05835 | 0.028879 | -2.02042 | 0.04334 | -0.11495 | -0.00175 | EUR | Screening for malignant neoplasms of the skin | neoplasms |
| 300 | 0.042674 | 0.021143 | 2.018387 | 0.043551 | 0.001235 | 0.084112 | EUR | Anxiety disorders | mental disorders |
| 480.3 | 0.205474 | 0.101918 | 2.016067 | 0.043793 | 0.005718 | 0.40523 | EUR | Pneumonia due to fungus (mycoses) | respiratory |
| 797 | 0.11065 | 0.054928 | 2.014446 | 0.043963 | 0.002993 | 0.218307 | EUR | Shock | symptoms |
| 519.1 | 0.496252 | 0.246931 | 2.009677 | 0.044465 | 0.012276 | 0.980229 | EUR | Tracheostomy complications | respiratory |
| 448 | -0.15178 | 0.075802 | -2.00225 | 0.045258 | -0.30034 | -0.00321 | EUR | Disease of capillaries | circulatory system |
| 573.9 | 0.071445 | 0.035714 | 2.000492 | 0.045447 | 0.001447 | 0.141442 | EUR | Abnormal serum enzyme levels | digestive |
| 733.8 | 0.189499 | 0.094961 | 1.99556 | 0.045982 | 0.00338 | 0.375619 | EUR | Malunion and nonunion of fracture | musculoskeletal |
| 117.4 | 0.226009 | 0.11341 | 1.992853 | 0.046278 | 0.00373 | 0.448289 | EUR | Aspergillosis | infectious diseases |
| 495 | 0.050303 | 0.025293 | 1.988831 | 0.04672 | 0.00073 | 0.099876 | EUR | Asthma | respiratory |
| 172 | -0.05273 | 0.026558 | -1.9855 | 0.047089 | -0.10479 | -0.00068 | EUR | Skin cancer | neoplasms |
| 425.2 | 0.159511 | 0.080479 | 1.982024 | 0.047477 | 0.001775 | 0.317247 | EUR | Secondary/extrinsic cardiomyopathies | circulatory system |
| 226 | -0.29901 | 0.151976 | -1.96747 | 0.049129 | -0.59688 | -0.00114 | EUR | Benign neoplasm of thyroid glands | neoplasms |
| 364.5 | -0.19513 | 0.099697 | -1.95721 | 0.050323 | -0.39053 | 0.000275 | EUR | Corneal dystrophy | sense organs |
| 379.1 | 0.385578 | 0.197736 | 1.949966 | 0.05118 | -0.00198 | 0.773133 | EUR | Scleritis and episcleritis | sense organs |
| 433.12 | -0.24798 | 0.127483 | -1.94518 | 0.051753 | -0.49784 | 0.001885 | EUR | Cerebral atherosclerosis | circulatory system |
| 345.11 | -0.22848 | 0.117469 | -1.94504 | 0.05177 | -0.45872 | 0.001753 | EUR | Generalized convulsive epilepsy | neurological |
| 155 | 0.152347 | 0.078413 | 1.942886 | 0.05203 | -0.00134 | 0.306033 | EUR | Cancer of liver and intrahepatic bile duct | neoplasms |
| 366.2 | -0.05964 | 0.030781 | -1.93752 | 0.052682 | -0.11997 | 0.000691 | EUR | Senile cataract | sense organs |

| phecode | Coef. | Std.Err. | z | p_value_z | [0.025 | 0.975] | GIA | phenotype | category |
|---|---|---|---|---|---|---|---|---|---|
| 536.8 | -0.07879 | 0.040675 | -1.93693 | 0.052754 | -0.15851 | 0.000937 | EUR | Dyspepsia and other specified disorders of function of stomach | digestive |
| 396 | -0.07146 | 0.036896 | -1.93672 | 0.05278 | -0.14377 | 0.000858 | EUR | Abnormal heart sounds | circulatory system |
| 496 | 0.073806 | 0.038143 | 1.934988 | 0.052992 | -0.00095 | 0.148564 | EUR | Chronic airway obstruction | respiratory |
| 530.14 | 0.080163 | 0.041478 | 1.932677 | 0.053276 | -0.00113 | 0.161457 | EUR | Reflux esophagitis | digestive |
| 285 | 0.042022 | 0.021763 | 1.930868 | 0.053499 | -0.00063 | 0.084677 | EUR | Other anemias | hematopoietic |
| 653 | -0.13198 | 0.068577 | -1.9245 | 0.054292 | -0.26638 | 0.002432 | EUR | Problems associated with amniotic cavity and membranes | pregnancy complications |
| 601.11 | -0.18114 | 0.094632 | -1.91418 | 0.055598 | -0.36662 | 0.004333 | EUR | Acute prostatitis | genitourinary |
| 748 | 0.314824 | 0.165082 | 1.907073 | 0.056511 | -0.00873 | 0.63838 | EUR | Anomalies of respiratory system, congenital | congenital anomalies |
| 481 | 0.097586 | 0.051233 | 1.904754 | 0.056812 | -0.00283 | 0.198001 | EUR | Influenza | respiratory |
| 651 | -0.16071 | 0.084394 | -1.90434 | 0.056866 | -0.32612 | 0.004694 | EUR | Multiple gestation | pregnancy complications |
| 702 | -0.04425 | 0.02325 | -1.90303 | 0.057037 | -0.08982 | 0.001324 | EUR | Degenerative skin conditions and other dermatoses | dermatologic |
| 401.21 | 0.086866 | 0.045749 | 1.898747 | 0.057598 | -0.0028 | 0.176532 | EUR | Hypertensive heart disease | circulatory system |
| 495.1 | 0.165127 | 0.087025 | 1.897455 | 0.057768 | -0.00544 | 0.335693 | EUR | Chronic obstructive asthma | respiratory |
| 426.92 | 0.131128 | 0.069176 | 1.895582 | 0.058015 | -0.00445 | 0.26671 | EUR | Cardiac defibrillator in situ | circulatory system |
| 360 | -0.19681 | 0.103834 | -1.89539 | 0.058041 | -0.40032 | 0.006705 | EUR | Disorders of the globe | sense organs |
| 287.1 | -0.18547 | 0.097966 | -1.89317 | 0.058335 | -0.37748 | 0.006543 | EUR | Spontaneous ecchymoses | hematopoietic |
| 296 | 0.041458 | 0.021908 | 1.892397 | 0.058438 | -0.00148 | 0.084396 | EUR | Mood disorders | mental disorders |
| 681.5 | 0.093857 | 0.049639 | 1.890809 | 0.05865 | -0.00343 | 0.191147 | EUR | Cellulitis and abscess of leg, except foot | dermatologic |
| 286.1 | -0.26983 | 0.142713 | -1.89075 | 0.058658 | -0.54955 | 0.009878 | EUR | Congenital coagulation defects | hematopoietic |
| 585.1 | 0.058796 | 0.031162 | 1.88681 | 0.059186 | -0.00228 | 0.119872 | EUR | Acute renal failure | genitourinary |
| 379.2 | -0.06746 | 0.03594 | -1.87689 | 0.060534 | -0.1379 | 0.002986 | EUR | Disorders of vitreous body | sense organs |
| 714.2 | -0.31895 | 0.16997 | -1.87653 | 0.060582 | -0.65209 | 0.014181 | EUR | Juvenile rheumatoid arthritis | musculoskeletal |
| 414.2 | -0.24477 | 0.130488 | -1.87578 | 0.060686 | -0.50052 | 0.010985 | EUR | ASCVD | circulatory system |
| 446.6 | 0.605993 | 0.323386 | 1.873901 | 0.060944 | -0.02783 | 1.239817 | EUR | Polyarteritis nodosa | circulatory system |
| 253.3 | 0.296535 | 0.158383 | 1.872264 | 0.06117 | -0.01389 | 0.606959 | EUR | Diabetes insipidus | endocrine/metabolic |
| 327.1 | 0.079174 | 0.042369 | 1.868666 | 0.061669 | -0.00387 | 0.162216 | EUR | Hypersomnia | neurological |
| 378.5 | -0.26934 | 0.144361 | -1.86575 | 0.062077 | -0.55229 | 0.013601 | EUR | Paralytic strabismus | sense organs |
| 513.4 | 0.275644 | 0.14777 | 1.865355 | 0.062132 | -0.01398 | 0.565269 | EUR | Hyperventilation | respiratory |
| 261.1 | -0.46529 | 0.249675 | -1.86358 | 0.06238 | -0.95465 | 0.024063 | EUR | Vitamin A deficiency | endocrine/metabolic |
| 604.1 | 0.528142 | 0.283877 | 1.860457 | 0.062821 | -0.02825 | 1.084531 | EUR | Redundant prepuce and phimosis/BXO | genitourinary |
| 290.1 | 0.126726 | 0.068244 | 1.856946 | 0.063319 | -0.00703 | 0.260483 | EUR | Dementias | mental disorders |
| 557.1 | -0.15071 | 0.081268 | -1.85448 | 0.063671 | -0.30999 | 0.008573 | EUR | Celiac disease | digestive |
| 394.7 | 0.144818 | 0.078302 | 1.849479 | 0.064389 | -0.00865 | 0.298288 | EUR | Disease of tricuspid valve | circulatory system |
| 601.8 | -0.46637 | 0.253058 | -1.84293 | 0.065339 | -0.96235 | 0.029615 | EUR | Other inflammatory disorders of male genital organs | genitourinary |
| 611.1 | -0.06214 | 0.033763 | -1.84043 | 0.065705 | -0.12831 | 0.004036 | EUR | Abnormal mammogram | genitourinary |
| 301.2 | 0.31874 | 0.173596 | 1.836106 | 0.066342 | -0.0215 | 0.658981 | EUR | Antisocial/borderline personality disorder | mental disorders |
| 580.1 | 0.227927 | 0.124974 | 1.823795 | 0.068183 | -0.01702 | 0.472871 | EUR | Glomerulonephritis | genitourinary |
| 594 | 0.058852 | 0.032271 | 1.82366 | 0.068203 | -0.0044 | 0.122103 | EUR | Urinary calculus | genitourinary |
| 707.3 | 0.114921 | 0.063056 | 1.822524 | 0.068376 | -0.00867 | 0.238509 | EUR | Chronic ulcer of unspecified site | dermatologic |
| 81.11 | 0.486897 | 0.267644 | 1.819197 | 0.068881 | -0.03768 | 1.01147 | EUR | Acute graft-versus-host disease | infectious diseases |
| 722.9 | 0.093767 | 0.052018 | 1.802587 | 0.071453 | -0.00819 | 0.19572 | EUR | Other and unspecified disc disorder | musculoskeletal |
| 501 | 0.109529 | 0.060827 | 1.80068 | 0.071753 | -0.00969 | 0.228748 | EUR | Pneumonitis due to inhalation of food or vomitus | respiratory |
| 841 | -0.0632 | 0.035106 | -1.80014 | 0.071838 | -0.132 | 0.005611 | EUR | Sprains and strains of back and neck | injuries & poisonings |
| 170 | 0.105227 | 0.058467 | 1.799776 | 0.071896 | -0.00937 | 0.219819 | EUR | Cancer of bone and connective tissue | neoplasms |
| 519.2 | 0.221293 | 0.123062 | 1.79822 | 0.072142 | -0.0199 | 0.462491 | EUR | Respiratory complications | respiratory |
| 742.2 | 0.305527 | 0.170031 | 1.796891 | 0.072353 | -0.02773 | 0.63878 | EUR | Pathological, developmental or recurrent dislocation | musculoskeletal |
| 988 | -2.29167 | 1.275532 | -1.79664 | 0.072393 | -4.79167 | 0.208325 | EUR | Toxic effect of noxious substances eaten as food | injuries & poisonings |
| 599.4 | 0.066248 | 0.036895 | 1.795568 | 0.072563 | -0.00607 | 0.138561 | EUR | Urinary incontinence | genitourinary |
| 610.1 | -0.09047 | 0.050563 | -1.78926 | 0.073574 | -0.18957 | 0.008631 | EUR | Cystic mastopathy | genitourinary |
| 227.1 | 0.195436 | 0.109655 | 1.782287 | 0.074702 | -0.01948 | 0.410356 | EUR | Benign neoplasm of adrenal gland | neoplasms |
| 604.3 | 0.236554 | 0.132761 | 1.7818 | 0.074782 | -0.02365 | 0.496761 | EUR | Peyronie's disease | genitourinary |
| 626.4 | -0.20155 | 0.113368 | -1.77786 | 0.075427 | -0.42375 | 0.020645 | EUR | Premenstrual tension syndromes | genitourinary |
| 645 | -0.13417 | 0.075513 | -1.77677 | 0.075606 | -0.28217 | 0.013833 | EUR | Late pregnancy and failed induction | pregnancy complications |
| 159.3 | 0.308016 | 0.173636 | 1.773915 | 0.076077 | -0.0323 | 0.648337 | EUR | Malignant neoplasm of gallbladder and extrahepatic bile ducts | neoplasms |
| 695.2 | 0.307824 | 0.174347 | 1.76558 | 0.077466 | -0.03389 | 0.649539 | EUR | Bullous dermatoses | dermatologic |
| 524.3 | 0.440458 | 0.249716 | 1.763834 | 0.07776 | -0.04898 | 0.929893 | EUR | Anomalies of tooth position/malocclusion | digestive |
| 496.1 | 0.13155 | 0.074628 | 1.762748 | 0.077943 | -0.01472 | 0.277819 | EUR | Emphysema | respiratory |
| 279.2 | -0.24401 | 0.138506 | -1.76174 | 0.078113 | -0.51548 | 0.027455 | EUR | Autoimmune disease NEC | endocrine/metabolic |
| 579.8 | 0.075777 | 0.043095 | 1.758376 | 0.078684 | -0.00869 | 0.160242 | EUR | Nonspecific abnormal findings in stool contents | digestive |

| phecode | Coef. | Std.Err. | z | p_value_z | [0.025 | 0.975] | GIA | phenotype | category |
|---|---|---|---|---|---|---|---|---|---|
| 536.3 | 0.140152 | 0.079864 | 1.754886 | 0.079279 | -0.01638 | 0.296683 | EUR | Gastroparesis | digestive |
| 556.11 | 0.234201 | 0.133674 | 1.752035 | 0.079768 | -0.02779 | 0.496196 | EUR | Angiodysplasia of intestine (without mention of hemorrhage) | digestive |
| 260.6 | -0.10237 | 0.058468 | -1.75082 | 0.079976 | -0.21696 | 0.012228 | EUR | Anorexia | endocrine/metabolic |
| 256 | 0.125599 | 0.07188 | 1.747351 | 0.080576 | -0.01528 | 0.266481 | EUR | Ovarian dysfunction | endocrine/metabolic |
| 315.2 | -0.19403 | 0.111059 | -1.7471 | 0.08062 | -0.4117 | 0.02364 | EUR | Speech and language disorder | mental disorders |
| 364.1 | -0.22459 | 0.128564 | -1.74693 | 0.080649 | -0.47658 | 0.027388 | EUR | Corneal opacity | sense organs |
| 8.7 | -0.33949 | 0.194437 | -1.74602 | 8.08E-02 | -0.72058 | 0.041598 | EUR | Intestinal infection due to protozoa | infectious diseases |
| 585.32 | 0.105395 | 0.060442 | 1.743728 | 0.081207 | -0.01307 | 0.22386 | EUR | End stage renal disease | genitourinary |
| 526.9 | 0.263014 | 0.150946 | 1.742437 | 0.081432 | -0.03283 | 0.558863 | EUR | Jaw disease NOS | digestive |
| 770 | 0.04534 | 0.026059 | 1.739904 | 0.081876 | -0.00573 | 0.096414 | EUR | Myalgia and myositis unspecified | symptoms |
| 872 | 0.319621 | 0.184248 | 1.734731 | 0.082788 | -0.0415 | 0.680741 | EUR | Traumatic amputation | injuries & poisonings |
| 480 | 0.051645 | 0.029774 | 1.734542 | 0.082822 | -0.00671 | 0.110001 | EUR | Pneumonia | respiratory |
| 527.1 | -0.39727 | 0.229299 | -1.73255 | 0.083176 | -0.84669 | 0.052146 | EUR | Hypertrophy of salivary gland | digestive |
| 986 | -1.23268 | 0.71163 | -1.73219 | 0.083239 | -2.62745 | 0.162087 | EUR | Toxic effect of carbon monoxide | injuries & poisonings |
| 550.6 | -0.13836 | 0.079922 | -1.73113 | 0.083428 | -0.295 | 0.018289 | EUR | Incisional hernia | digestive |
| 535 | 0.055323 | 0.031975 | 1.730175 | 0.083599 | -0.00735 | 0.117994 | EUR | Gastritis and duodenitis | digestive |
| 256.1 | 0.564306 | 0.326493 | 1.728383 | 0.08392 | -0.07561 | 1.204221 | EUR | Hyperestrogenism | endocrine/metabolic |
| 681.1 | -0.09168 | 0.053326 | -1.71918 | 0.085582 | -0.19619 | 0.01284 | EUR | Cellulitis and abscess of fingers/toes | dermatologic |
| 526.1 | -0.63133 | 0.367557 | -1.71765 | 0.085861 | -1.35173 | 0.089065 | EUR | Cysts of the jaws | digestive |
| 994 | 0.060972 | 0.035499 | 1.717564 | 0.085876 | -0.00861 | 0.13055 | EUR | Sepsis and SIRS | injuries & poisonings |
| 634.1 | -0.15792 | 0.091949 | -1.71743 | 0.0859 | -0.33813 | 0.0223 | EUR | Missed abortion/Hydatidiform mole | pregnancy complications |
| 702.2 | -0.04082 | 0.023897 | -1.70827 | 0.087587 | -0.08766 | 0.006015 | EUR | Seborrheic keratosis | dermatologic |
| 200 | 0.089447 | 0.052407 | 1.706781 | 0.087863 | -0.01327 | 0.192162 | EUR | Myeloproliferative disease | neoplasms |
| 428.2 | 0.099313 | 0.05823 | 1.705525 | 0.088097 | -0.01482 | 0.213442 | EUR | Heart failure NOS | circulatory system |
| 635 | -0.1168 | 0.068641 | -1.70167 | 0.088818 | -0.25134 | 0.01773 | EUR | Hemorrhage during pregnancy; childbirth and postpartum | pregnancy complications |
| 427.5 | -0.0753 | 0.044315 | -1.69918 | 0.089285 | -0.16215 | 0.011557 | EUR | Arrhythmia (cardiac) NOS | circulatory system |
| 370 | -0.12916 | 0.076023 | -1.69898 | 0.089323 | -0.27816 | 0.019841 | EUR | Keratitis | sense organs |
| 261.4 | -0.04299 | 0.025302 | -1.69888 | 0.089341 | -0.09258 | 0.006606 | EUR | Vitamin D deficiency | endocrine/metabolic |
| 731 | 0.106627 | 0.062852 | 1.696482 | 0.089795 | -0.01656 | 0.229814 | EUR | Osteitis deformans and osteopathies associated with other disorders classified elsewhere | musculoskeletal |
| 277.4 | -0.09873 | 0.058255 | -1.69472 | 0.090129 | -0.2129 | 0.015452 | EUR | Disorders of bilirubin excretion | endocrine/metabolic |
| 512.9 | 0.040597 | 0.023957 | 1.694608 | 0.09015 | -0.00636 | 0.087551 | EUR | Other dyspnea | respiratory |
| 411.41 | -0.27005 | 0.159429 | -1.69384 | 0.090295 | -0.58252 | 0.042427 | EUR | Aneurysm and dissection of heart | circulatory system |
| 440 | 0.064823 | 0.03827 | 1.693826 | 0.090298 | -0.01019 | 0.139831 | EUR | Atherosclerosis | circulatory system |
| 429.1 | 0.099954 | 0.059017 | 1.693654 | 0.090331 | -0.01572 | 0.215625 | EUR | Heart transplant/surgery | circulatory system |
| 714 | 0.077875 | 0.046008 | 1.692639 | 0.090524 | -0.0123 | 0.16805 | EUR | Rheumatoid arthritis and other inflammatory polyarthropathies | musculoskeletal |
| 526.42 | 0.171425 | 0.101331 | 1.691743 | 0.090695 | -0.02718 | 0.37003 | EUR | Arthralgia/ankylosis of temporomandibular joint | digestive |
| 713 | -0.23872 | 0.141182 | -1.69084 | 0.090867 | -0.51543 | 0.037995 | EUR | Arthropathy associated with other disorders classified elsewhere | musculoskeletal |
| 187 | 0.204552 | 0.121089 | 1.689272 | 0.091167 | -0.03278 | 0.441881 | EUR | Cancer of other male genital organs | neoplasms |
| 573 | 0.052814 | 0.031267 | 1.68912 | 0.091196 | -0.00847 | 0.114097 | EUR | Other disorders of liver | digestive |
| 362.7 | 0.375413 | 0.222398 | 1.688022 | 0.091407 | -0.06048 | 0.811305 | EUR | Hereditary retinal dystrophies | sense organs |
| 526.8 | 0.586505 | 0.348447 | 1.683197 | 0.092337 | -0.09644 | 1.269449 | EUR | Exostosis of jaw | digestive |
| 710.1 | 0.1407 | 0.083839 | 1.678219 | 0.093304 | -0.02362 | 0.305021 | EUR | Osteomyelitis | musculoskeletal |
| 755.3 | 0.425417 | 0.253546 | 1.67787 | 0.093373 | -0.07152 | 0.922358 | EUR | Congenital anomaly of fingers/toes | congenital anomalies |
| 339 | 0.042235 | 0.025191 | 1.676593 | 0.093622 | -0.00714 | 0.091609 | EUR | Other headache syndromes | neurological |
| 295 | 0.1106 | 0.066021 | 1.675219 | 0.093891 | -0.0188 | 0.24 | EUR | Schizophrenia and other psychotic disorders | mental disorders |
| 300.1 | 0.035784 | 0.021361 | 1.675199 | 0.093895 | -0.00608 | 0.077651 | EUR | Anxiety disorder | mental disorders |
| 707.2 | 0.114904 | 0.068605 | 1.674859 | 0.093962 | -0.01956 | 0.249368 | EUR | Chronic ulcer of leg or foot | dermatologic |
| 452.2 | 0.06902 | 0.041281 | 1.671964 | 0.094531 | -0.01189 | 0.149929 | EUR | Deep vein thrombosis [DVT] | circulatory system |
| 599.3 | -0.04829 | 0.028887 | -1.67171 | 0.094582 | -0.10491 | 0.008327 | EUR | Dysuria | genitourinary |
| 90.2 | -0.33422 | 0.200381 | -1.6679 | 0.095335 | -0.72696 | 0.058524 | EUR | Gonococcal infections | infectious diseases |
| 536 | -0.06104 | 0.036654 | -1.66545 | 0.095823 | -0.13288 | 0.010795 | EUR | Disorders of function of stomach | digestive |
| 411.3 | 0.075642 | 0.045473 | 1.663436 | 0.096225 | -0.01348 | 0.164768 | EUR | Angina pectoris | circulatory system |
| 528.5 | -0.11686 | 0.07026 | -1.66318 | 0.096277 | -0.25456 | 0.020852 | EUR | Diseases of lips | digestive |
| 575.7 | 0.107418 | 0.064684 | 1.660649 | 0.096784 | -0.01936 | 0.234197 | EUR | Other disorders of gallbladder | digestive |
| 579 | 0.057786 | 0.034843 | 1.658446 | 0.097227 | -0.01051 | 0.126077 | EUR | Other symptoms involving abdomen and pelvis | digestive |
| 626.14 | 0.113955 | 0.068725 | 1.658139 | 0.097289 | -0.02074 | 0.248654 | EUR | Irregular menstrual bleeding | genitourinary |
| 270.1 | -0.17735 | 0.107092 | -1.65606 | 0.097709 | -0.38725 | 0.032546 | EUR | Disturbances of amino-acid transport | endocrine/metabolic |
| 272 | 0.038862 | 0.023534 | 1.651301 | 0.098677 | -0.00726 | 0.084989 | EUR | Disorders of lipoid metabolism | endocrine/metabolic |
| 706.1 | -0.06211 | 0.03768 | -1.64826 | 0.099299 | -0.13596 | 0.011745 | EUR | Acne | dermatologic |

| phecode | Coef. | Std.Err. | z | p_value_z | [0.025 | 0.975] | GIA | phenotype | category |
|---|---|---|---|---|---|---|---|---|---|
| 738.4 | 0.081032 | 0.049179 | 1.647702 | 0.099414 | -0.01536 | 0.17742 | EUR | Acquired spondylolisthesis | musculoskeletal |
| 292 | 0.047426 | 0.028794 | 1.647104 | 0.099537 | -0.00901 | 0.103861 | EUR | Neurological disorders | mental disorders |
| 70.4 | -0.20399 | 0.124035 | -1.6446 | 0.100052 | -0.44709 | 0.039116 | EUR | Chronic hepatitis | infectious diseases |
| 355.1 | 0.106706 | 0.065043 | 1.640545 | 0.100892 | -0.02078 | 0.234188 | EUR | Chronic pain syndrome | neurological |
| 916 | 0.052387 | 0.031996 | 1.637273 | 0.101573 | -0.01032 | 0.115099 | EUR | Contusion | injuries & poisonings |
| 530.2 | 0.07738 | 0.047281 | 1.6366 | 0.101714 | -0.01529 | 0.17005 | EUR | Esophageal bleeding (varices/hemorrhage) | digestive |
| 286.7 | 0.088243 | 0.054012 | 1.633762 | 0.102309 | -0.01762 | 0.194104 | EUR | Other and unspecified coagulation defects | hematopoietic |
| 187.8 | -0.66301 | 0.40601 | -1.63298 | 0.102473 | -1.45877 | 0.132758 | EUR | Neoplasm of uncertain behavior of male genital organs | neoplasms |
| 742.1 | 0.329257 | 0.201847 | 1.631216 | 0.102845 | -0.06636 | 0.724871 | EUR | Loose body in joint | musculoskeletal |
| 687.2 | 0.052992 | 0.032541 | 1.628445 | 0.103431 | -0.01079 | 0.116771 | EUR | Localized superficial swelling, mass, or lump | dermatologic |
| 757 | -0.24045 | 0.147936 | -1.62536 | 0.104087 | -0.5304 | 0.049501 | EUR | Congenital anomalies of the integument | congenital anomalies |
| 272.1 | 0.038248 | 0.023536 | 1.625091 | 0.104143 | -0.00788 | 0.084379 | EUR | Hyperlipidemia | endocrine/metabolic |
| 669 | -0.12353 | 0.076031 | -1.62471 | 0.104225 | -0.27254 | 0.02549 | EUR | Complications of labor and delivery NEC | pregnancy complications |
| 722.1 | 0.052904 | 0.032591 | 1.623261 | 0.104534 | -0.01097 | 0.116783 | EUR | Displacement of intervertebral disc | musculoskeletal |
| 440.9 | 0.06998 | 0.043148 | 1.621868 | 0.104832 | -0.01459 | 0.154547 | EUR | Atherosclerosis of aorta | circulatory system |
| 420.22 | -0.20962 | 0.129373 | -1.6203 | 0.105167 | -0.46319 | 0.043943 | EUR | Chronic pericarditis | circulatory system |
| 509.3 | -0.17272 | 0.106835 | -1.61673 | 0.105937 | -0.38212 | 0.03667 | EUR | Pulmonary insufficiency or respiratory failure following trauma and surgery | respiratory |
| 272.11 | -0.04043 | 0.0251 | -1.6106 | 0.107266 | -0.08962 | 0.008769 | EUR | Hypercholesterolemia | endocrine/metabolic |
| 229 | -0.12085 | 0.075174 | -1.60758 | 0.107927 | -0.26819 | 0.02649 | EUR | Benign neoplasm of unspecified sites | neoplasms |
| 359 | 0.113404 | 0.070577 | 1.606804 | 0.108097 | -0.02493 | 0.251732 | EUR | Muscular dystrophies and other myopathies | neurological |
| 70.3 | 0.157911 | 0.098291 | 1.606562 | 0.10815 | -0.03474 | 0.350559 | EUR | Viral hepatitis C | infectious diseases |
| 483 | 0.067658 | 0.042148 | 1.605255 | 0.108438 | -0.01495 | 0.150267 | EUR | Acute bronchitis and bronchiolitis | respiratory |
| 509.8 | 0.110677 | 0.068988 | 1.604306 | 0.108647 | -0.02454 | 0.245891 | EUR | Dependence on respirator [Ventilator] or supplemental oxygen | respiratory |
| 564.1 | -0.05846 | 0.036487 | -1.60223 | 0.109105 | -0.12997 | 0.013053 | EUR | Irritable Bowel Syndrome | digestive |
| 316.1 | -0.10422 | 0.065045 | -1.60223 | 0.109105 | -0.2317 | 0.023269 | EUR | Polyneuropathy due to drugs | mental disorders |
| 276.41 | 0.070715 | 0.044394 | 1.592894 | 0.111184 | -0.0163 | 0.157726 | EUR | Acidosis | endocrine/metabolic |
| 580.32 | 0.110073 | 0.069128 | 1.592323 | 0.111312 | -0.02541 | 0.245561 | EUR | Nephritis and nephropathy with pathological lesion | genitourinary |
| 531.4 | -0.08573 | 0.053988 | -1.58786 | 0.112317 | -0.19154 | 0.020089 | EUR | Peptic ulcer, site unspecified | digestive |
| 331.1 | -0.16231 | 0.102441 | -1.58442 | 0.113097 | -0.36309 | 0.038471 | EUR | Hydrocephalus | neurological |
| 381.3 | -0.22937 | 0.144844 | -1.58357 | 0.113293 | -0.51326 | 0.054519 | EUR | Mastoiditis & related conditions | sense organs |
| 562.1 | 0.04453 | 0.028145 | 1.582195 | 0.113605 | -0.01063 | 0.099692 | EUR | Diverticulosis | digestive |
| 741 | 0.040809 | 0.025819 | 1.580579 | 0.113974 | -0.0098 | 0.091413 | EUR | Symptoms and disorders of the joints | musculoskeletal |
| 1015 | 0.055511 | 0.035195 | 1.577263 | 0.114735 | -0.01347 | 0.124491 | EUR | Effects of other external causes | other |
| 255.2 | 0.109019 | 0.069227 | 1.574803 | 0.115302 | -0.02666 | 0.244702 | EUR | Adrenal hypofunction | endocrine/metabolic |
| 255.21 | 0.109019 | 0.069227 | 1.574803 | 0.115302 | -0.02666 | 0.244702 | EUR | Glucocorticoid deficiency | endocrine/metabolic |
| 724.8 | 0.102029 | 0.0649 | 1.572105 | 0.115926 | -0.02517 | 0.22923 | EUR | Other symptoms referable to back | musculoskeletal |
| 599.6 | 0.177757 | 0.113198 | 1.570322 | 0.11634 | -0.04411 | 0.399622 | EUR | Oliguria and anuria | genitourinary |
| 636 | -0.10169 | 0.064815 | -1.56896 | 0.116657 | -0.22873 | 0.025343 | EUR | Early or threatened labor; hemorrhage in early pregnancy | pregnancy complications |
| 729 | 0.042865 | 0.02736 | 1.566686 | 0.117188 | -0.01076 | 0.09649 | EUR | Other disorders of soft tissues | musculoskeletal |
| 522 | 0.170387 | 0.108826 | 1.565683 | 0.117423 | -0.04291 | 0.383682 | EUR | Diseases of pulp and periapical tissues | digestive |
| 594.3 | -0.11291 | 0.072208 | -1.56361 | 0.117909 | -0.25443 | 0.02862 | EUR | Calculus of ureter | genitourinary |
| 994.2 | 0.056355 | 0.036048 | 1.563344 | 0.117972 | -0.0143 | 0.127007 | EUR | Sepsis | injuries & poisonings |
| 617 | -0.06167 | 0.039466 | -1.56252 | 0.118165 | -0.13902 | 0.015685 | EUR | Disorders secondary to childbirth, surgery, trauma | genitourinary |
| 737.1 | -0.15174 | 0.097257 | -1.56018 | 0.118717 | -0.34236 | 0.038882 | EUR | Kyphosis (acquired) | musculoskeletal |
| 281.9 | 0.09974 | 0.063936 | 1.55999 | 0.118762 | -0.02557 | 0.225053 | EUR | Deficiency anemias | hematopoietic |
| 302.1 | 0.110624 | 0.071099 | 1.5559 | 0.119732 | -0.02873 | 0.249976 | EUR | Decreased libido | mental disorders |
| 622 | 0.086386 | 0.055637 | 1.552676 | 0.1205 | -0.02266 | 0.195431 | EUR | Polyp of female genital organs | genitourinary |
| 771.1 | 0.043235 | 0.027857 | 1.552027 | 0.120656 | -0.01136 | 0.097835 | EUR | Swelling of limb | symptoms |
| 733 | -0.04276 | 0.027565 | -1.5513 | 0.12083 | -0.09679 | 0.011265 | EUR | Other disorders of bone and cartilage | musculoskeletal |
| 316 | 0.058042 | 0.037494 | 1.548052 | 0.12161 | -0.01544 | 0.131528 | EUR | Substance addiction and disorders | mental disorders |
| 614.5 | -0.05866 | 0.037938 | -1.54625 | 0.122045 | -0.13302 | 0.015696 | EUR | Inflammatory disease of cervix, vagina, and vulva | genitourinary |
| 255 | 0.07865 | 0.050925 | 1.544409 | 0.122489 | -0.02116 | 0.178462 | EUR | Disorders of adrenal glands | endocrine/metabolic |
| 71 | 0.164664 | 0.106668 | 1.543713 | 0.122658 | -0.0444 | 0.373729 | EUR | Human immunodeficiency virus [HIV] disease | infectious diseases |
| 443.9 | 0.084237 | 0.05459 | 1.543088 | 0.122809 | -0.02276 | 0.191232 | EUR | Peripheral vascular disease, unspecified | circulatory system |
| 586.4 | -0.07187 | 0.046678 | -1.53966 | 0.123643 | -0.16336 | 0.019619 | EUR | Stricture/obstruction of ureter | genitourinary |
| 420.3 | -0.13054 | 0.084907 | -1.53747 | 0.124178 | -0.29696 | 0.035873 | EUR | Endocarditis | circulatory system |
| 254 | 0.438001 | 0.285296 | 1.53525 | 0.124723 | -0.12117 | 0.997171 | EUR | Diseases of thymus gland | endocrine/metabolic |
| 939 | -0.03916 | 0.02557 | -1.53153 | 0.125638 | -0.08928 | 0.010955 | EUR | Atopic/contact dermatitis due to other or unspecified | dermatologic |
| 250.1 | 0.118112 | 0.077233 | 1.52931 | 0.126188 | -0.03326 | 0.269485 | EUR | Type 1 diabetes | endocrine/metabolic |

| phecode | Coef. | Std.Err. | z | p_value_z | [0.025 | 0.975] | GIA | phenotype | category |
|---|---|---|---|---|---|---|---|---|---|
| 760 | 0.032842 | 0.021493 | 1.528074 | 0.126494 | -0.00928 | 0.074967 | EUR | Back pain | symptoms |
| 598.4 | 0.275837 | 0.180646 | 1.526944 | 0.126775 | -0.07822 | 0.629897 | EUR | Other cells and casts in urine | genitourinary |
| 210 | -0.18483 | 0.121338 | -1.52325 | 0.127696 | -0.42265 | 0.05299 | EUR | Benign neoplasm of lip, oral cavity, and pharynx | neoplasms |
| 276.42 | -0.14476 | 0.095091 | -1.52238 | 0.127915 | -0.33114 | 0.04161 | EUR | Alkalosis | endocrine/metabolic |
| 745 | 0.031745 | 0.020888 | 1.519801 | 0.128561 | -0.00919 | 0.072684 | EUR | Pain in joint | musculoskeletal |
| 614.52 | -0.06718 | 0.044209 | -1.51963 | 0.128604 | -0.15383 | 0.019467 | EUR | Vaginitis and vulvovaginitis | genitourinary |
| 751.2 | 0.085283 | 0.056154 | 1.518733 | 0.12883 | -0.02478 | 0.195344 | EUR | Congenital anomalies of urinary system | congenital anomalies |
| 586.3 | -0.29177 | 0.192144 | -1.51852 | 0.128883 | -0.66837 | 0.08482 | EUR | Vascular disorders of kidney/hypertrophy | genitourinary |
| 1009 | 0.060455 | 0.039828 | 1.51789 | 0.129042 | -0.01761 | 0.138517 | EUR | Injury, NOS | other |
| 691 | -0.10944 | 0.072099 | -1.51787 | 0.129047 | -0.25075 | 0.031874 | EUR | Congenital anomalies of skin | dermatologic |
| 358 | -0.13631 | 0.089867 | -1.51682 | 0.129312 | -0.31245 | 0.039824 | EUR | Myoneural disorders | neurological |
| 442.3 | 0.314929 | 0.20774 | 1.515972 | 0.129527 | -0.09224 | 0.722092 | EUR | Aneurysm of artery of lower extremity | circulatory system |
| 110.11 | -0.05735 | 0.037922 | -1.51227 | 0.130464 | -0.13167 | 0.016977 | EUR | Dermatophytosis of nail | infectious diseases |
| 145 | 0.124939 | 0.082647 | 1.511718 | 0.130606 | -0.03705 | 0.286925 | EUR | Cancer of mouth | neoplasms |
| 427.8 | 0.084716 | 0.056185 | 1.507812 | 0.131603 | -0.0254 | 0.194836 | EUR | Sinoatrial node dysfunction (Bradycardia) | circulatory system |
| 346.3 | -0.32854 | 0.21798 | -1.50719 | 0.131763 | -0.75577 | 0.098697 | EUR | Nonspecific abnormal findings in cerebrospinal fluid | neurological |
| 426.31 | 0.094487 | 0.062745 | 1.505883 | 0.132097 | -0.02849 | 0.217465 | EUR | Right bundle branch block | circulatory system |
| 38.3 | 0.088973 | 0.059097 | 1.505545 | 1.32E-01 | -0.02685 | 0.204801 | EUR | Bacteremia | infectious diseases |
| 851 | 0.092184 | 0.061309 | 1.503612 | 0.132681 | -0.02798 | 0.212347 | EUR | Complications of transplants and reattached limbs | injuries & poisonings |
| 803.1 | 0.121995 | 0.081208 | 1.502256 | 0.133031 | -0.03717 | 0.28116 | EUR | Fracture of humerus | injuries & poisonings |
| 756.3 | -0.2019 | 0.134524 | -1.50084 | 0.133396 | -0.46556 | 0.061763 | EUR | Congenital anomalies of muscle, tendon, fascia, and connective tissue | congenital anomalies |
| 327.72 | -0.18868 | 0.125729 | -1.50071 | 0.133431 | -0.43511 | 0.057742 | EUR | Sleep related leg cramps | neurological |
| 415.1 | 0.086321 | 0.057551 | 1.499917 | 0.133636 | -0.02648 | 0.199119 | EUR | Acute pulmonary heart disease | circulatory system |
| 415.11 | 0.086321 | 0.057551 | 1.499917 | 0.133636 | -0.02648 | 0.199119 | EUR | Pulmonary embolism and infarction, acute | circulatory system |
| 479 | 0.037545 | 0.025075 | 1.497324 | 0.134309 | -0.0116 | 0.086692 | EUR | Other upper respiratory disease | respiratory |
| 753 | -0.19149 | 0.127966 | -1.49644 | 0.134539 | -0.4423 | 0.059315 | EUR | Congenital anomalies of the eye | congenital anomalies |
| 384.1 | 0.556629 | 0.372256 | 1.495286 | 0.13484 | -0.17298 | 1.286237 | EUR | Myringitis | sense organs |
| 395.6 | 0.08307 | 0.055609 | 1.49383 | 0.13522 | -0.02592 | 0.192062 | EUR | Heart valve replaced | circulatory system |
| 433.3 | 0.056075 | 0.03756 | 1.492954 | 0.135449 | -0.01754 | 0.129692 | EUR | Cerebral ischemia | circulatory system |
| 522.1 | -1.81022 | 1.213987 | -1.49114 | 0.135926 | -4.18959 | 0.569151 | EUR | Pulpitis and necrosis of tooth pulp | digestive |
| 440.2 | 0.129553 | 0.087254 | 1.48477 | 0.137605 | -0.04146 | 0.300568 | EUR | Atherosclerosis of the extremities | circulatory system |
| 362.23 | -0.11904 | 0.080214 | -1.48404 | 0.137798 | -0.27626 | 0.038176 | EUR | Cystoid macular degeneration of retina | sense organs |
| 941 | 0.106987 | 0.072228 | 1.481239 | 0.138543 | -0.03458 | 0.248551 | EUR | Adverse reaction to serum or vaccine | injuries & poisonings |
| 370.3 | -0.18799 | 0.126967 | -1.48063 | 0.138705 | -0.43684 | 0.060859 | EUR | Keratoconjunctivitis | sense organs |
| 750.15 | -0.3137 | 0.211957 | -1.48004 | 0.138863 | -0.72913 | 0.101723 | EUR | Congenital anomalies of stomach | congenital anomalies |
| 751.21 | 0.095413 | 0.064498 | 1.479307 | 0.139058 | -0.031 | 0.221827 | EUR | Cystic kidney disease | congenital anomalies |
| 290 | 0.079838 | 0.054002 | 1.478422 | 0.139295 | -0.026 | 0.18568 | EUR | Delirium dementia and amnestic and other cognitive disorders | mental disorders |
| 41.21 | 0.306858 | 0.207569 | 1.478343 | 0.139316 | -0.09997 | 0.713685 | EUR | Rheumatic fever / chorea | infectious diseases |
| 613.1 | -0.12275 | 0.083231 | -1.4748 | 0.140268 | -0.28588 | 0.040381 | EUR | Inflammatory disease of breast | genitourinary |
| 800.4 | -0.16666 | 0.113193 | -1.47239 | 0.140915 | -0.38852 | 0.05519 | EUR | Fracture of patella | injuries & poisonings |
| 643.1 | -0.22507 | 0.153385 | -1.46732 | 0.142289 | -0.5257 | 0.075565 | EUR | Hyperemesis gravidarum | pregnancy complications |
| 335 | 0.173027 | 0.118024 | 1.466034 | 0.142639 | -0.0583 | 0.404349 | EUR | Multiple sclerosis | neurological |
| 362 | -0.05355 | 0.036571 | -1.46437 | 0.143092 | -0.12523 | 0.018124 | EUR | Other retinal disorders | sense organs |
| 428.4 | 0.073656 | 0.050435 | 1.460415 | 0.144176 | -0.02519 | 0.172507 | EUR | Heart failure with preserved EF [Diastolic heart failure] | circulatory system |
| 250.7 | 0.119118 | 0.081591 | 1.459941 | 0.144306 | -0.0408 | 0.279034 | EUR | Diabetic retinopathy | endocrine/metabolic |
| 427.21 | 0.048039 | 0.033045 | 1.453732 | 0.146021 | -0.01673 | 0.112806 | EUR | Atrial fibrillation | circulatory system |
| 611.11 | -0.11527 | 0.079487 | -1.45014 | 0.147019 | -0.27106 | 0.040524 | EUR | Mammographic microcalcification | genitourinary |
| 989 | 0.355152 | 0.245021 | 1.449476 | 0.147205 | -0.12508 | 0.835384 | EUR | Toxic effect of other substances, chiefly nonmedicinal as to source | injuries & poisonings |
| 555 | 0.065414 | 0.04513 | 1.449446 | 0.147213 | -0.02304 | 0.153868 | EUR | Inflammatory bowel disease and other gastroenteritis and colitis | digestive |
| 728.71 | 0.100205 | 0.069285 | 1.446259 | 0.148105 | -0.03559 | 0.236001 | EUR | Contracture of palmar fascia [Dupuytren's disease] | musculoskeletal |
| 426.91 | 0.077389 | 0.053595 | 1.443959 | 0.14875 | -0.02766 | 0.182433 | EUR | Cardiac pacemaker in situ | circulatory system |
| 596.1 | -0.13497 | 0.093582 | -1.4423 | 0.149218 | -0.31839 | 0.048444 | EUR | Bladder neck obstruction | genitourinary |
| 425.12 | 0.189372 | 0.131406 | 1.441123 | 0.14955 | -0.06818 | 0.446922 | EUR | Other hypertrophic cardiomyopathy | circulatory system |
| 289.1 | 0.657283 | 0.456386 | 1.440191 | 0.149813 | -0.23722 | 1.551783 | EUR | Myelofibrosis | hematopoietic |
| 981 | 0.620727 | 0.431102 | 1.43986 | 0.149907 | -0.22422 | 1.465673 | EUR | Toxic effect of (non-ethyl) alcohol and petroleum and other solvents | injuries & poisonings |
| 305.21 | -0.26125 | 0.181632 | -1.43834 | 0.150338 | -0.61724 | 0.094744 | EUR | Anorexia nervosa | mental disorders |
| 578.2 | 0.062609 | 0.043538 | 1.43804 | 0.150423 | -0.02272 | 0.147942 | EUR | Blood in stool | digestive |
| 379.3 | 0.221794 | 0.154378 | 1.436693 | 0.150805 | -0.08078 | 0.524369 | EUR | Aphakia and other disorders of lens | sense organs |
| 634.3 | 0.264753 | 0.184412 | 1.435662 | 0.151098 | -0.09669 | 0.626194 | EUR | Ectopic pregnancy | pregnancy complications |

| phecode | Coef. | Std.Err. | z | p_value_z | [0.025 | 0.975] | GIA | phenotype | category |
|---|---|---|---|---|---|---|---|---|---|
| 528.1 | -0.0841 | 0.058675 | -1.43332 | 0.151767 | -0.1991 | 0.030901 | EUR | Stomatitis and mucositis | digestive |
| 627.5 | -0.13061 | 0.091181 | -1.43246 | 0.152012 | -0.30932 | 0.048098 | EUR | Premature menopause and other ovarian failure | genitourinary |
| 972 | -0.16005 | 0.112071 | -1.42807 | 0.15327 | -0.3797 | 0.059609 | EUR | Poisoning by agents primarily affecting the cardiovascular system | injuries & poisonings |
| 580.14 | 0.375239 | 0.263166 | 1.425864 | 0.153908 | -0.14056 | 0.891034 | EUR | Chronic glomerulonephritis, NOS | genitourinary |
| 592.13 | -0.16757 | 0.117642 | -1.42438 | 0.154335 | -0.39814 | 0.063007 | EUR | Chronic interstitial cystitis | genitourinary |
| 471 | 0.102417 | 0.071981 | 1.422827 | 0.154786 | -0.03866 | 0.243497 | EUR | Nasal polyps | respiratory |
| 654.2 | -0.20302 | 0.142861 | -1.42107 | 0.155296 | -0.48302 | 0.076987 | EUR | Rhesus isoimmunization in pregnancy | pregnancy complications |
| 618.1 | 0.090489 | 0.063771 | 1.418966 | 0.155909 | -0.0345 | 0.215478 | EUR | Prolapse of vaginal walls | genitourinary |
| 636.8 | -0.36515 | 0.257389 | -1.41865 | 0.156 | -0.86962 | 0.139327 | EUR | Cervical incompetence | pregnancy complications |
| 587 | 0.102711 | 0.073069 | 1.405667 | 0.159823 | -0.0405 | 0.245924 | EUR | Kidney replaced by transpant | genitourinary |
| 41.1 | 0.081454 | 0.058088 | 1.40225 | 0.160841 | -0.0324 | 0.195305 | EUR | Staphylococcus infections | infectious diseases |
| 756.2 | -0.19138 | 0.136635 | -1.40063 | 0.161324 | -0.45917 | 0.076424 | EUR | Pectus and other congenital anomalies of ribs/sternum | congenital anomalies |
| 622.2 | 0.130956 | 0.093723 | 1.397263 | 0.162334 | -0.05274 | 0.314649 | EUR | Mucous polyp of cervix | genitourinary |
| 635.2 | -0.1101 | 0.078843 | -1.39638 | 0.1626 | -0.26463 | 0.044435 | EUR | Antepartum hemorrhage, abruptio placentae, and placenta previa | pregnancy complications |
| 701.6 | 0.199875 | 0.143169 | 1.396077 | 0.162691 | -0.08073 | 0.480481 | EUR | Acquired acanthosis nigricans | dermatologic |
| 592.12 | -0.21028 | 0.150785 | -1.3946 | 0.163137 | -0.50582 | 0.085249 | EUR | Chronic cystitis | genitourinary |
| 297.2 | 0.2441 | 0.175196 | 1.393296 | 0.16353 | -0.09928 | 0.587478 | EUR | Suicide or self-inflicted injury | mental disorders |
| 155.1 | 0.123622 | 0.088835 | 1.391593 | 0.164046 | -0.05049 | 0.297734 | EUR | Malignant neoplasm of liver, primary | neoplasms |
| 428.3 | 0.070471 | 0.050668 | 1.390834 | 0.164276 | -0.02884 | 0.169778 | EUR | Heart failure with reduced EF [Systolic or combined heart failure] | circulatory system |
| 735.22 | 0.830787 | 0.598788 | 1.387448 | 0.165305 | -0.34282 | 2.004389 | EUR | Claw toe (acquired) | musculoskeletal |
| 627.4 | -0.16591 | 0.119759 | -1.38534 | 0.165948 | -0.40063 | 0.068816 | EUR | Premenopausal menorrhagia | genitourinary |
| 323.8 | -0.16778 | 0.121241 | -1.38383 | 0.166409 | -0.4054 | 0.06985 | EUR | Encephalitis, non-infectious | neurological |
| 214.1 | 0.080172 | 0.058007 | 1.382111 | 0.166938 | -0.03352 | 0.193863 | EUR | Lipoma of skin and subcutaneous tissue | neoplasms |
| 573.1 | 0.206057 | 0.149331 | 1.379865 | 0.167628 | -0.08663 | 0.498741 | EUR | Chronic passive congestion of liver | digestive |
| 362.9 | -0.12689 | 0.091963 | -1.37979 | 0.167651 | -0.30713 | 0.053354 | EUR | Retinal edema | sense organs |
| 365.5 | 0.344642 | 0.249881 | 1.379221 | 0.167827 | -0.14512 | 0.8344 | EUR | Pseudoexfoliation glaucoma | sense organs |
| 805 | 0.080064 | 0.05808 | 1.3785 | 0.168049 | -0.03377 | 0.193899 | EUR | Fracture of vertebral column without mention of spinal cord injury | injuries & poisonings |
| 1019 | 0.055729 | 0.04049 | 1.376375 | 0.168706 | -0.02363 | 0.135088 | EUR | Other ill-defined and unknown causes of morbidity and mortality | other |
| 509.2 | -0.14768 | 0.107457 | -1.3743 | 0.169347 | -0.35829 | 0.062933 | EUR | Respiratory insufficiency | respiratory |
| 704.1 | -0.05967 | 0.043437 | -1.37375 | 0.169519 | -0.14481 | 0.025463 | EUR | Alopecia | dermatologic |
| 643 | -0.11245 | 0.082012 | -1.37115 | 0.170329 | -0.27319 | 0.04829 | EUR | Excessive vomiting in pregnancy | pregnancy complications |
| 38 | 0.048183 | 0.035185 | 1.369412 | 1.71E-01 | -0.02078 | 0.117144 | EUR | Septicemia | infectious diseases |
| 791 | 0.11327 | 0.082754 | 1.368749 | 0.171078 | -0.04893 | 0.275466 | EUR | Gangrene | symptoms |
| 362.29 | 0.107124 | 0.078316 | 1.367834 | 0.171364 | -0.04637 | 0.260621 | EUR | Macular degeneration (senile) of retina NOS | sense organs |
| 480.11 | -0.09241 | 0.067581 | -1.36738 | 0.171506 | -0.22487 | 0.040048 | EUR | Pneumococcal pneumonia | respiratory |
| 528.12 | -0.1415 | 0.103764 | -1.36371 | 0.172659 | -0.34488 | 0.06187 | EUR | Oral aphthae | digestive |
| 772 | -0.06574 | 0.048233 | -1.36301 | 0.17288 | -0.16028 | 0.028793 | EUR | Symptoms of the muscles | symptoms |
| 559 | -0.13034 | 0.095812 | -1.36039 | 0.173707 | -0.31813 | 0.057446 | EUR | Ileostomy status | digestive |
| 622.1 | 0.086674 | 0.063805 | 1.358429 | 0.174328 | -0.03838 | 0.211729 | EUR | Polyp of corpus uteri | genitourinary |
| 874 | 0.397477 | 0.292739 | 1.357784 | 0.174532 | -0.17628 | 0.971235 | EUR | Complication of amputation stump | injuries & poisonings |
| 368.7 | 1.14503 | 0.846479 | 1.352699 | 0.176152 | -0.51404 | 2.804098 | EUR | Disorders of accommodation | sense organs |
| 189.11 | 0.107033 | 0.079142 | 1.352421 | 0.176241 | -0.04808 | 0.262148 | EUR | Malignant neoplasm of kidney, except pelvis | neoplasms |
| 245.1 | -0.32036 | 0.236957 | -1.352 | 0.176377 | -0.78479 | 0.144062 | EUR | Thyroiditis, acute and subacute | endocrine/metabolic |
| 306.9 | 0.09047 | 0.067083 | 1.348634 | 0.177455 | -0.04101 | 0.22195 | EUR | Tension headache | mental disorders |
| 497 | 0.05464 | 0.040566 | 1.346957 | 0.177994 | -0.02487 | 0.134147 | EUR | Bronchitis | respiratory |
| 379 | -0.04198 | 0.031174 | -1.34679 | 0.178049 | -0.10308 | 0.019115 | EUR | Other disorders of eye | sense organs |
| 325 | -0.41896 | 0.311498 | -1.34499 | 0.17863 | -1.02948 | 0.191564 | EUR | Phlebitis and thrombophlebitis of intracranial venous sinuses | neurological |
| 704.12 | -0.13761 | 0.102411 | -1.34372 | 0.179039 | -0.33833 | 0.06311 | EUR | Telogen effluvium | dermatologic |
| 816 | 0.40933 | 0.304922 | 1.342408 | 0.179464 | -0.18831 | 1.006967 | EUR | Cerebral laceration and contusion | injuries & poisonings |
| 555.2 | 0.070853 | 0.052804 | 1.341811 | 0.179657 | -0.03264 | 0.174348 | EUR | Ulcerative colitis | digestive |
| 204.3 | 0.679792 | 0.506661 | 1.34171 | 0.17969 | -0.31325 | 1.672831 | EUR | Monocytic leukemia | neoplasms |
| 611.3 | -0.05028 | 0.037475 | -1.34161 | 0.179724 | -0.12373 | 0.023173 | EUR | Lump or mass in breast | genitourinary |
| 496.3 | 0.077079 | 0.057476 | 1.341067 | 0.179899 | -0.03557 | 0.18973 | EUR | Bronchiectasis | respiratory |
| 426.3 | 0.064927 | 0.04851 | 1.338418 | 0.18076 | -0.03015 | 0.160006 | EUR | Bundle branch block | circulatory system |
| 324 | -0.13266 | 0.099453 | -1.33385 | 0.182253 | -0.32758 | 0.062269 | EUR | Other CNS infection and poliomyelitis | neurological |
| 389.2 | -0.12079 | 0.09064 | -1.33265 | 0.182647 | -0.29844 | 0.056859 | EUR | Conductive hearing loss | sense organs |
| 577 | 0.054904 | 0.041212 | 1.332213 | 0.18279 | -0.02587 | 0.135679 | EUR | Diseases of pancreas | digestive |
| 994.21 | 0.093036 | 0.069966 | 1.329732 | 0.183606 | -0.04409 | 0.230167 | EUR | Septic shock | injuries & poisonings |
| 367 | -0.0399 | 0.030035 | -1.32845 | 0.184029 | -0.09877 | 0.018967 | EUR | Disorders of refraction and accommodation; blindness and low vision | sense organs |

| phecode | Coef. | Std.Err. | z | p_value_z | [0.025 | 0.975] | GIA | phenotype | category |
|---|---|---|---|---|---|---|---|---|---|
| 741.4 | 0.041005 | 0.030902 | 1.32694 | 0.184529 | -0.01956 | 0.101572 | EUR | Joint effusions | musculoskeletal |
| 588.1 | 0.389878 | 0.294629 | 1.323285 | 0.185741 | -0.18758 | 0.967341 | EUR | Renal osteodystrophy | genitourinary |
| 550.4 | 0.073561 | 0.055645 | 1.321976 | 0.186176 | -0.0355 | 0.182623 | EUR | Umbilical hernia | digestive |
| 174.2 | -0.24047 | 0.181902 | -1.32198 | 0.186176 | -0.59699 | 0.116051 | EUR | Breast cancer [male] | neoplasms |
| 184.11 | -0.11904 | 0.09011 | -1.321 | 0.186502 | -0.29565 | 0.057577 | EUR | Malignant neoplasm of ovary | neoplasms |
| 686.3 | -0.23139 | 0.175174 | -1.3209 | 0.186535 | -0.57472 | 0.111948 | EUR | Pilonidal cyst | dermatologic |
| 305.2 | -0.12427 | 0.09417 | -1.31966 | 0.18695 | -0.30884 | 0.060298 | EUR | Eating disorder | mental disorders |
| 575.8 | -0.08294 | 0.062963 | -1.31728 | 0.187744 | -0.20634 | 0.040465 | EUR | Other disorders of biliary tract | digestive |
| 371 | -0.04024 | 0.030553 | -1.31715 | 0.187788 | -0.10012 | 0.01964 | EUR | Inflammation of the eye | sense organs |
| 722.7 | -0.14631 | 0.111121 | -1.31668 | 0.187945 | -0.3641 | 0.071482 | EUR | Intervertebral disc disorder with myelopathy | musculoskeletal |
| 755 | 0.108319 | 0.08232 | 1.315827 | 0.188232 | -0.05303 | 0.269663 | EUR | Congenital anomalies of limbs | congenital anomalies |
| 149 | 0.108761 | 0.082957 | 1.31105 | 0.189841 | -0.05383 | 0.271354 | EUR | Cancer of larynx, pharynx, nasal cavities | neoplasms |
| 352.2 | 0.122357 | 0.093377 | 1.310347 | 0.190079 | -0.06066 | 0.305373 | EUR | Facial nerve disorders [CN7] | neurological |
| 270.33 | -0.16766 | 0.128098 | -1.30887 | 0.190578 | -0.41873 | 0.083404 | EUR | Amyloidosis | endocrine/metabolic |
| 704.2 | -0.14274 | 0.109269 | -1.30632 | 0.191443 | -0.3569 | 0.071423 | EUR | Hirsutism | dermatologic |
| 636.3 | -0.11012 | 0.084315 | -1.30603 | 0.191542 | -0.27537 | 0.055136 | EUR | Hemorrhage in early pregnancy | pregnancy complications |
| 613 | -0.04616 | 0.03537 | -1.305 | 0.191893 | -0.11548 | 0.023166 | EUR | Other nonmalignant breast conditions | genitourinary |
| 715.2 | -0.14438 | 0.110751 | -1.30369 | 0.19234 | -0.36145 | 0.072683 | EUR | Ankylosing spondylitis | musculoskeletal |
| 509.5 | 0.403787 | 0.310617 | 1.299952 | 0.193618 | -0.20501 | 1.012586 | EUR | Respiratory arrest | respiratory |
| 172.22 | -0.05685 | 0.043783 | -1.29854 | 0.194102 | -0.14267 | 0.028959 | EUR | Squamous cell carcinoma | neoplasms |
| 619.4 | -0.048 | 0.037009 | -1.29704 | 0.194619 | -0.12054 | 0.024534 | EUR | Noninflammatory disorders of vagina | genitourinary |
| 363.3 | 0.17214 | 0.133363 | 1.290762 | 0.196786 | -0.08925 | 0.433527 | EUR | Chorioretinal scars | sense organs |
| 567 | 0.078932 | 0.061184 | 1.290079 | 0.197023 | -0.04099 | 0.198849 | EUR | Peritonitis and retroperitoneal infections | digestive |
| 389.3 | -0.14712 | 0.114225 | -1.28798 | 0.197753 | -0.371 | 0.076757 | EUR | Degenerative and vascular disorders of ear | sense organs |
| 795.82 | -0.20971 | 0.162998 | -1.28656 | 0.198247 | -0.52918 | 0.109763 | EUR | Elevated cancer antigen 125 [CA 125] | symptoms |
| 79.9 | -0.06947 | 0.054038 | -1.28557 | 0.198594 | -0.17538 | 0.036443 | EUR | Viremia, NOS | infectious diseases |
| 755.6 | 0.167053 | 0.130259 | 1.282468 | 0.199678 | -0.08825 | 0.422357 | EUR | Other congenital anomalies of lower limb, including pelvic girdle | congenital anomalies |
| 199 | -0.03383 | 0.026434 | -1.27993 | 0.200568 | -0.08564 | 0.017976 | EUR | Neoplasm of uncertain behavior | neoplasms |
| 401.3 | 0.051121 | 0.039973 | 1.278906 | 0.20093 | -0.02722 | 0.129466 | EUR | Other hypertensive complications | circulatory system |
| 912 | -0.07197 | 0.056353 | -1.2772 | 0.20153 | -0.18242 | 0.038475 | EUR | Insect bite | injuries & poisonings |
| 750.14 | 0.16131 | 0.126437 | 1.275822 | 0.202019 | -0.0865 | 0.409121 | EUR | Congenital anomalies of esophagus | congenital anomalies |
| 173 | -0.03487 | 0.027385 | -1.27346 | 0.202853 | -0.08855 | 0.0188 | EUR | Neoplasm of uncertain behavior of skin | neoplasms |
| 563 | -0.03198 | 0.02514 | -1.27212 | 0.203329 | -0.08126 | 0.017292 | EUR | Constipation | digestive |
| 420.1 | 0.18544 | 0.146052 | 1.269679 | 0.204199 | -0.10082 | 0.471697 | EUR | Myocarditis | circulatory system |
| 420 | 0.056916 | 0.044843 | 1.269208 | 0.204367 | -0.03098 | 0.144807 | EUR | Carditis | circulatory system |
| 253.1 | -0.12941 | 0.101987 | -1.2689 | 0.204476 | -0.3293 | 0.07048 | EUR | Pituitary hyperfunction | endocrine/metabolic |
| 586.11 | 0.436924 | 0.344383 | 1.268713 | 0.204543 | -0.23806 | 1.111902 | EUR | Small kidney | genitourinary |
| 333 | -0.06038 | 0.047645 | -1.26725 | 0.205065 | -0.15376 | 0.033004 | EUR | Extrapyramidal disease and abnormal movement disorders | neurological |
| 783 | 0.03513 | 0.027791 | 1.264108 | 0.206191 | -0.01934 | 0.089599 | EUR | Fever of unknown origin | symptoms |
| 610.4 | -0.11554 | 0.091514 | -1.26259 | 0.206735 | -0.29491 | 0.063819 | EUR | Benign neoplasm of breast | genitourinary |
| 556 | 0.078218 | 0.061964 | 1.2623 | 0.206841 | -0.04323 | 0.199666 | EUR | Ulceration of the lower GI tract | digestive |
| 706.3 | 0.254305 | 0.201561 | 1.261679 | 0.207064 | -0.14075 | 0.649358 | EUR | Seborrhea | dermatologic |
| 377.1 | -0.13941 | 0.110506 | -1.26159 | 0.207095 | -0.356 | 0.077174 | EUR | Optic atrophy | sense organs |
| 427.11 | 0.05062 | 0.040144 | 1.260971 | 0.207319 | -0.02806 | 0.129301 | EUR | Paroxysmal supraventricular tachycardia | circulatory system |
| 573.5 | -0.07445 | 0.059069 | -1.2604 | 0.207526 | -0.19022 | 0.041323 | EUR | Jaundice (not of newborn) | digestive |
| 695.22 | 0.300462 | 0.238406 | 1.260294 | 0.207563 | -0.16681 | 0.767728 | EUR | Pemphigus and pemphigoid | dermatologic |
| 242.1 | -0.12054 | 0.095746 | -1.25898 | 0.208039 | -0.3082 | 0.067117 | EUR | Graves' disease | endocrine/metabolic |
| 524 | 0.249822 | 0.198669 | 1.257479 | 0.20858 | -0.13956 | 0.639206 | EUR | Dentofacial anomalies, including malocclusion | digestive |
| 976 | 0.546162 | 0.436652 | 1.250795 | 0.211009 | -0.30966 | 1.401985 | EUR | Poisoning by agents primarily affecting skin & mucous membrane, ophthalmological, otorhinolaryngological, & dental drugs | injuries & poisonings |
| 447.1 | 0.124491 | 0.099568 | 1.250314 | 0.211185 | -0.07066 | 0.31964 | EUR | Stricture of artery | circulatory system |
| 340 | 0.035123 | 0.028099 | 1.24996 | 0.211314 | -0.01995 | 0.090196 | EUR | Migraine | neurological |
| 286 | 0.04366 | 0.034993 | 1.247649 | 0.21216 | -0.02493 | 0.112245 | EUR | Coagulation defects | hematopoietic |
| 198.6 | 0.072432 | 0.058099 | 1.246699 | 0.212508 | -0.04144 | 0.186305 | EUR | Secondary malignancy of bone | neoplasms |
| 610.8 | -0.09463 | 0.076009 | -1.24496 | 0.213146 | -0.2436 | 0.054347 | EUR | Other specified benign mammary dysplasias | genitourinary |
| 596.5 | 0.054726 | 0.044021 | 1.243175 | 0.213803 | -0.03155 | 0.141005 | EUR | Functional disorders of bladder | genitourinary |
| 477 | 0.067734 | 0.054498 | 1.242882 | 0.213911 | -0.03908 | 0.174548 | EUR | Epistaxis or throat hemorrhage | respiratory |
| 798.1 | 0.057092 | 0.045937 | 1.242832 | 0.21393 | -0.03294 | 0.147128 | EUR | Chronic fatigue syndrome | symptoms |
| 585.34 | 0.085001 | 0.068417 | 1.242392 | 0.214092 | -0.04909 | 0.219097 | EUR | Chronic Kidney Disease, Stage IV | genitourinary |
| 158 | 0.083155 | 0.06694 | 1.242217 | 0.214157 | -0.04805 | 0.214355 | EUR | Neoplasm of unspecified nature of digestive system | neoplasms |

| phecode | Coef. | Std.Err. | z | p_value_z | [0.025 | 0.975] | GIA | phenotype | category |
|---|---|---|---|---|---|---|---|---|---|
| 433.2 | 0.057295 | 0.04621 | 1.239894 | 0.215015 | -0.03327 | 0.147865 | EUR | Occlusion of cerebral arteries | circulatory system |
| 302 | 0.079841 | 0.064431 | 1.23918 | 0.215279 | -0.04644 | 0.206124 | EUR | Sexual and gender identity disorders | mental disorders |
| 270.11 | -0.14926 | 0.120562 | -1.23807 | 0.21569 | -0.38556 | 0.087033 | EUR | Disturbances of sulphur-bearing amino-acid metabolism | endocrine/metabolic |
| 736.2 | -0.14634 | 0.11859 | -1.23398 | 0.217212 | -0.37877 | 0.086095 | EUR | Acquired deformities of finger | musculoskeletal |
| 626 | 0.042223 | 0.034266 | 1.232212 | 0.21787 | -0.02494 | 0.109382 | EUR | Disorders of menstruation and other abnormal bleeding from female genital tract | genitourinary |
| 411.8 | 0.077386 | 0.062818 | 1.231894 | 0.217989 | -0.04574 | 0.200508 | EUR | Other chronic ischemic heart disease, unspecified | circulatory system |
| 262 | -0.06934 | 0.056317 | -1.23118 | 0.218255 | -0.17971 | 0.041042 | EUR | Mineral deficiency NEC | endocrine/metabolic |
| 374.1 | -0.1167 | 0.09486 | -1.23025 | 0.218602 | -0.30262 | 0.06922 | EUR | Ectropion or entropion | sense organs |
| 372 | 0.067097 | 0.054554 | 1.229917 | 0.218728 | -0.03983 | 0.174022 | EUR | Disorders of conjunctiva | sense organs |
| 818.2 | 0.361058 | 0.294097 | 1.227686 | 0.219565 | -0.21536 | 0.937477 | EUR | Subarachnoid hemorrhage (injury) | injuries & poisonings |
| 200.1 | 0.118529 | 0.096648 | 1.226397 | 0.220049 | -0.0709 | 0.307955 | EUR | Polycythemia vera | neoplasms |
| 172.11 | 0.065145 | 0.053122 | 1.226325 | 0.220076 | -0.03897 | 0.169263 | EUR | Melanomas of skin | neoplasms |
| 698 | -0.04085 | 0.033347 | -1.22495 | 0.220594 | -0.10621 | 0.02451 | EUR | Pruritus and related conditions | dermatologic |
| 635.3 | -0.1288 | 0.105241 | -1.22387 | 0.221003 | -0.33507 | 0.077468 | EUR | Placenta previa and abruptio placenta | pregnancy complications |
| 251.1 | 0.087923 | 0.071928 | 1.22238 | 0.221564 | -0.05305 | 0.2289 | EUR | Hypoglycemia | endocrine/metabolic |
| 359.1 | 0.261414 | 0.214012 | 1.221495 | 0.221899 | -0.15804 | 0.680869 | EUR | Muscular dystrophies | neurological |
| 695.7 | 0.086155 | 0.070678 | 1.218982 | 0.222851 | -0.05237 | 0.224681 | EUR | Prurigo and Lichen | dermatologic |
| 374.6 | 0.098225 | 0.080722 | 1.216827 | 0.22367 | -0.05999 | 0.256438 | EUR | Dermatochalasis | sense organs |
| 260.3 | 0.091091 | 0.075063 | 1.213533 | 0.224926 | -0.05603 | 0.238212 | EUR | Adult failure to thrive | endocrine/metabolic |
| 331 | -0.0902 | 0.074419 | -1.2121 | 0.225472 | -0.23606 | 0.055655 | EUR | Other cerebral degenerations | neurological |
| 288.3 | 0.108456 | 0.089688 | 1.209268 | 0.22656 | -0.06733 | 0.284241 | EUR | Eosinophilia | hematopoietic |
| 433.6 | -0.25068 | 0.208267 | -1.20363 | 0.228734 | -0.65887 | 0.15752 | EUR | Acute, but ill-defined cerebrovascular disease | circulatory system |
| 270 | -0.061 | 0.050698 | -1.20314 | 0.228924 | -0.16036 | 0.03837 | EUR | Disorders of protein plasma/amino-acid transport and metabolism | endocrine/metabolic |
| 290.12 | -0.20356 | 0.169363 | -1.20191 | 0.2294 | -0.53551 | 0.128387 | EUR | Dementia with cerebral degenerations | mental disorders |
| 276.4 | 0.050421 | 0.042054 | 1.198953 | 0.230546 | -0.032 | 0.132845 | EUR | Acid-base balance disorder | endocrine/metabolic |
| 394.1 | -0.12682 | 0.106036 | -1.19604 | 0.231682 | -0.33465 | 0.081004 | EUR | Mitral valve stenosis and aortic valve stenosis | circulatory system |
| 969 | -0.17818 | 0.14923 | -1.19402 | 0.232471 | -0.47067 | 0.114302 | EUR | Poisoning by psychotropic agents | injuries & poisonings |
| 289.8 | 0.092493 | 0.077475 | 1.193837 | 0.232542 | -0.05936 | 0.244342 | EUR | Polycythemia, secondary | hematopoietic |
| 300.11 | -0.03622 | 0.030397 | -1.19147 | 0.23347 | -0.0958 | 0.02336 | EUR | Generalized anxiety disorder | mental disorders |
| 456 | 0.049694 | 0.041727 | 1.19092 | 0.233685 | -0.03209 | 0.131478 | EUR | Chronic venous insufficiency [CVI] | circulatory system |
| 174.3 | -0.2643 | 0.222046 | -1.19028 | 0.233936 | -0.6995 | 0.170905 | EUR | Neoplasm of uncertain behavior of breast | neoplasms |
| 41.8 | -0.10733 | 0.090204 | -1.18989 | 0.23409 | -0.28413 | 0.069464 | EUR | H. pylori | infectious diseases |
| 367.1 | -0.05755 | 0.048583 | -1.18447 | 0.236228 | -0.15277 | 0.037676 | EUR | Myopia | sense organs |
| 229.1 | 0.511921 | 0.432277 | 1.184241 | 0.236318 | -0.33533 | 1.359169 | EUR | Benign neoplasm of lymph nodes | neoplasms |
| 426.24 | 0.0921 | 0.077835 | 1.183285 | 0.236696 | -0.06045 | 0.244653 | EUR | Atrioventricular block, complete | circulatory system |
| 654.1 | 0.11753 | 0.099364 | 1.182823 | 0.236879 | -0.07722 | 0.31228 | EUR | Abnormality of organs and soft tissues of pelvis complicating pregnancy, childbirth, or the puerperium | pregnancy complications |
| 264.3 | -1.34567 | 1.138621 | -1.18184 | 0.237268 | -3.57733 | 0.885984 | EUR | Delayed milestones | endocrine/metabolic |
| 754 | 0.0915 | 0.077433 | 1.181663 | 0.23734 | -0.06027 | 0.243267 | EUR | Congenital musculoskeletal deformities of spine | congenital anomalies |
| 706.8 | -0.04662 | 0.039546 | -1.1789 | 0.238438 | -0.12413 | 0.030888 | EUR | Other specified diseases of sebaceous glands | dermatologic |
| 788 | 0.039246 | 0.0333 | 1.178546 | 0.238579 | -0.02602 | 0.104512 | EUR | Syncope and collapse | symptoms |
| 530.13 | 0.088717 | 0.075297 | 1.178216 | 0.238711 | -0.05886 | 0.236297 | EUR | Barrett's esophagus | digestive |
| 958.1 | -0.29444 | 0.249918 | -1.17815 | 0.238738 | -0.78427 | 0.19539 | EUR | Postoperative shock | injuries & poisonings |
| 686.5 | -0.20733 | 0.176274 | -1.17619 | 0.239518 | -0.55282 | 0.138159 | EUR | Pyoderma | dermatologic |
| 652 | -0.24643 | 0.209881 | -1.17413 | 0.240343 | -0.65779 | 0.164931 | EUR | Malposition and malpresentation of fetus or obstruction | pregnancy complications |
| 451 | 0.083103 | 0.070873 | 1.172567 | 0.24097 | -0.05581 | 0.222012 | EUR | Phlebitis and thrombophlebitis | circulatory system |
| 251.8 | 0.375351 | 0.320176 | 1.172326 | 0.241066 | -0.25218 | 1.002884 | EUR | Abnormality of secretion of glucagon or gastrin | endocrine/metabolic |
| 609.1 | -0.19828 | 0.169352 | -1.17079 | 0.241682 | -0.5302 | 0.133648 | EUR | Infertility, male | genitourinary |
| 743.21 | -0.13182 | 0.112659 | -1.1701 | 0.241961 | -0.35263 | 0.088985 | EUR | Pathologic fracture of vertebrae | musculoskeletal |
| 618 | 0.063614 | 0.054471 | 1.167853 | 0.242866 | -0.04315 | 0.170375 | EUR | Genital prolapse | genitourinary |
| 961.1 | -0.04298 | 0.036803 | -1.16771 | 0.242925 | -0.11511 | 0.029158 | EUR | Poisoning/allergy of sulfonamides | injuries & poisonings |
| 426.23 | -0.12592 | 0.107872 | -1.16735 | 0.243068 | -0.33735 | 0.085501 | EUR | Second degree AV block | circulatory system |
| 681.6 | 0.108287 | 0.092764 | 1.167333 | 0.243076 | -0.07353 | 0.290101 | EUR | Cellulitis and abscess of foot, toe | dermatologic |
| 277 | -0.04141 | 0.035484 | -1.167 | 0.24321 | -0.11096 | 0.028138 | EUR | Other disorders of metabolism | endocrine/metabolic |
| 781 | 0.036894 | 0.03163 | 1.166434 | 0.243439 | -0.0251 | 0.098887 | EUR | Symptoms involving nervous and musculoskeletal systems | symptoms |
| 394.4 | -0.70167 | 0.601913 | -1.16573 | 0.243723 | -1.8814 | 0.478058 | EUR | Acute rheumatic heart disease | circulatory system |
| 389 | -0.02948 | 0.025303 | -1.16502 | 0.244011 | -0.07907 | 0.020114 | EUR | Hearing loss | sense organs |
| 585.2 | 0.094052 | 0.08124 | 1.157706 | 0.246984 | -0.06518 | 0.253281 | EUR | Renal failure NOS | genitourinary |
| 440.21 | 0.282568 | 0.244126 | 1.157464 | 0.247083 | -0.19591 | 0.761046 | EUR | Atherosclerosis of native arteries of the extremities with ulceration or gangrene | circulatory system |
| 337.1 | 0.15076 | 0.130305 | 1.156982 | 0.24728 | -0.10463 | 0.406153 | EUR | Peripheral autonomic neuropathy | neurological |

| phecode | Coef. | Std.Err. | z | p_value_z | [0.025 | 0.975] | GIA | phenotype | category |
|---|---|---|---|---|---|---|---|---|---|
| 274 | 0.050547 | 0.043708 | 1.156457 | 0.247494 | -0.03512 | 0.136214 | EUR | Gout and other crystal arthropathies | endocrine/metabolic |
| 756.21 | -0.156 | 0.135584 | -1.15055 | 0.249917 | -0.42174 | 0.109744 | EUR | Pectus excavatum | congenital anomalies |
| 418.1 | 0.067304 | 0.058603 | 1.148487 | 0.250768 | -0.04755 | 0.182164 | EUR | Precordial pain | circulatory system |
| 751.1 | -0.12857 | 0.111977 | -1.14814 | 0.250909 | -0.34804 | 0.090905 | EUR | Congenital anomalies of genital organs | congenital anomalies |
| 741.2 | -0.05959 | 0.052021 | -1.14542 | 0.252036 | -0.16154 | 0.042373 | EUR | Stiffness of joint | musculoskeletal |
| 180.1 | 0.134006 | 0.117033 | 1.145022 | 0.2522 | -0.09538 | 0.363387 | EUR | Cervical cancer | neoplasms |
| 528 | -0.04327 | 0.037801 | -1.14478 | 0.252301 | -0.11736 | 0.030814 | EUR | Diseases of the oral soft tissues, excluding lesions specific for gingiva and tongue | digestive |
| 599.2 | -0.03843 | 0.033575 | -1.1447 | 0.252333 | -0.10424 | 0.027372 | EUR | Retention of urine | genitourinary |
| 451.2 | -0.12264 | 0.107167 | -1.14437 | 0.25247 | -0.33268 | 0.087404 | EUR | Phlebitis and thrombophlebitis of lower extremities | circulatory system |
| 967 | 0.08335 | 0.072934 | 1.142819 | 0.253114 | -0.0596 | 0.226298 | EUR | Adverse effects of sedatives or other central nervous system depressants and anesthetics | injuries & poisonings |
| 194 | 0.20623 | 0.180462 | 1.142791 | 0.253126 | -0.14747 | 0.559928 | EUR | Cancer of other endocrine glands | neoplasms |
| 723.1 | -0.11626 | 0.101779 | -1.14227 | 0.25334 | -0.31574 | 0.083223 | EUR | Torticollis | musculoskeletal |
| 740.12 | 0.071469 | 0.062677 | 1.140274 | 0.254172 | -0.05138 | 0.194315 | EUR | Osteoarthrosis, localized, secondary | musculoskeletal |
| 300.3 | -0.10484 | 0.092158 | -1.13756 | 0.255303 | -0.28546 | 0.075791 | EUR | Obsessive-compulsive disorders | mental disorders |
| 371.3 | -0.04021 | 0.035392 | -1.13598 | 0.255964 | -0.10957 | 0.029163 | EUR | Inflammation of eyelids | sense organs |
| 727.1 | -0.03516 | 0.030967 | -1.13543 | 0.256196 | -0.09585 | 0.025533 | EUR | Synovitis and tenosynovitis | musculoskeletal |
| 619 | -0.03243 | 0.028568 | -1.13534 | 0.256233 | -0.08843 | 0.023558 | EUR | Noninflammatory female genital disorders | genitourinary |
| 285.22 | 0.06741 | 0.059458 | 1.133743 | 0.256902 | -0.04913 | 0.183945 | EUR | Anemia in neoplastic disease | hematopoietic |
| 523.3 | 0.136304 | 0.120597 | 1.130242 | 0.258374 | -0.10006 | 0.372669 | EUR | Periodontitis (acute or chronic) | digestive |
| 256.4 | 0.092712 | 0.082159 | 1.128442 | 0.259133 | -0.06832 | 0.253741 | EUR | Polycystic ovaries | endocrine/metabolic |
| 371.21 | -0.06572 | 0.058355 | -1.12626 | 0.260056 | -0.1801 | 0.048651 | EUR | Allergic conjunctivitis | sense organs |
| 720 | 0.035747 | 0.031759 | 1.125587 | 0.26034 | -0.0265 | 0.097993 | EUR | Spinal stenosis | musculoskeletal |
| 523.31 | 0.136564 | 0.121362 | 1.125265 | 0.260477 | -0.1013 | 0.374429 | EUR | Acute periodontitis | digestive |
| 619.3 | -0.05353 | 0.047735 | -1.12147 | 0.262088 | -0.14709 | 0.040026 | EUR | Noninflammatory disorders of cervix | genitourinary |
| 250.25 | 0.073141 | 0.065288 | 1.12029 | 0.26259 | -0.05482 | 0.201102 | EUR | Diabetes type 2 with peripheral circulatory disorders | endocrine/metabolic |
| 782.6 | 0.061673 | 0.055108 | 1.119122 | 0.263088 | -0.04634 | 0.169683 | EUR | Pallor and flushing | symptoms |
| 289.3 | -0.06286 | 0.056192 | -1.11868 | 0.263277 | -0.173 | 0.047274 | EUR | Personal history of diseases of blood and blood-forming organs | hematopoietic |
| 433 | 0.034358 | 0.030721 | 1.118381 | 0.263404 | -0.02585 | 0.09457 | EUR | Cerebrovascular disease | circulatory system |
| 743.9 | -0.03088 | 0.027657 | -1.11654 | 0.26419 | -0.08509 | 0.023327 | EUR | Osteopenia or other disorder of bone and cartilage | musculoskeletal |
| 362.5 | 0.39687 | 0.356601 | 1.112926 | 0.26574 | -0.30205 | 1.095794 | EUR | Toxic maculopathy of retina | sense organs |
| 513.3 | 0.073742 | 0.066304 | 1.112186 | 0.266058 | -0.05621 | 0.203695 | EUR | Hypoventilation | respiratory |
| 287.31 | -0.1246 | 0.112041 | -1.11207 | 0.26611 | -0.34419 | 0.095 | EUR | Primary thrombocytopenia | hematopoietic |
| 860 | 0.098748 | 0.088909 | 1.110669 | 0.266711 | -0.07551 | 0.273006 | EUR | Bone marrow or stem cell transplant | neoplasms |
| 255.12 | 0.186529 | 0.168663 | 1.10593 | 0.268757 | -0.14404 | 0.517103 | EUR | Hyperaldosteronism | endocrine/metabolic |
| 980 | 0.068053 | 0.061544 | 1.105754 | 0.268833 | -0.05257 | 0.188677 | EUR | Encounter for long-term (current) use of antibiotics | infectious diseases |
| 270.35 | -0.2199 | 0.198887 | -1.10568 | 0.268865 | -0.60972 | 0.169906 | EUR | Macroglobulinemia | endocrine/metabolic |
| 521.4 | -0.87317 | 0.790612 | -1.10443 | 0.269408 | -2.42274 | 0.676397 | EUR | Tooth complications likely association with other diseases | digestive |
| 946 | 0.095207 | 0.086724 | 1.097815 | 0.272285 | -0.07477 | 0.265183 | EUR | Anaphylactic shock NOS | injuries & poisonings |
| 642 | -0.08292 | 0.075567 | -1.09734 | 0.272494 | -0.23103 | 0.065186 | EUR | Hypertension complicating pregnancy, childbirth, and the puerperium | pregnancy complications |
| 426.9 | 0.052217 | 0.047684 | 1.095058 | 0.273491 | -0.04124 | 0.145677 | EUR | Cardiac pacemaker/device in situ | circulatory system |
| 281 | 0.058651 | 0.053642 | 1.093389 | 0.274223 | -0.04648 | 0.163787 | EUR | Other deficiency anemia | hematopoietic |
| 751.3 | 0.231027 | 0.21157 | 1.091966 | 0.274848 | -0.18364 | 0.645697 | EUR | Obstructive genitourinary defect | congenital anomalies |
| 614.4 | -0.20011 | 0.183441 | -1.09087 | 0.275331 | -0.55965 | 0.159428 | EUR | Inflammatory diseases of uterus, except cervix | genitourinary |
| 500.1 | 0.193241 | 0.177168 | 1.090722 | 0.275395 | -0.154 | 0.540485 | EUR | Extrinsic allergic alveolitis | respiratory |
| 270.12 | -0.66588 | 0.611567 | -1.08881 | 0.276236 | -1.86453 | 0.532766 | EUR | Phenylketonuria [PKU] | endocrine/metabolic |
| 425.1 | 0.051758 | 0.047711 | 1.084823 | 0.278 | -0.04175 | 0.145269 | EUR | Primary/intrinsic cardiomyopathies | circulatory system |
| 977 | 0.029858 | 0.02755 | 1.083763 | 0.27847 | -0.02414 | 0.083854 | EUR | Personal history of allergy to medicinal agents | injuries & poisonings |
| 443 | 0.043507 | 0.040146 | 1.083701 | 0.278497 | -0.03518 | 0.122192 | EUR | Peripheral vascular disease | circulatory system |
| 694.3 | -0.27201 | 0.251123 | -1.08316 | 0.278739 | -0.7642 | 0.220186 | EUR | Vascular disorders of skin | dermatologic |
| 618.2 | 0.1117 | 0.103379 | 1.080487 | 0.279926 | -0.09092 | 0.314319 | EUR | Uterine/Uterovaginal prolapse | genitourinary |
| 278.4 | 0.042909 | 0.039761 | 1.079176 | 0.280509 | -0.03502 | 0.120838 | EUR | Abnormal weight gain | endocrine/metabolic |
| 433.31 | 0.043735 | 0.040552 | 1.078493 | 0.280814 | -0.03575 | 0.123217 | EUR | Transient cerebral ischemia | circulatory system |
| 53 | 0.050954 | 0.047255 | 1.078273 | 0.280912 | -0.04166 | 0.143571 | EUR | Herpes zoster | infectious diseases |
| 575.6 | -0.10153 | 0.094327 | -1.07632 | 0.281785 | -0.2864 | 0.083352 | EUR | Cholesterolosis of gallbladder | digestive |
| 334.2 | -0.2697 | 0.250972 | -1.07461 | 0.282552 | -0.76159 | 0.2222 | EUR | Anterior horn cell disease | neurological |
| 427.2 | 0.034787 | 0.032377 | 1.074428 | 0.282631 | -0.02867 | 0.098245 | EUR | Atrial fibrillation and flutter | circulatory system |
| 571.51 | 0.075046 | 0.069886 | 1.073839 | 0.282895 | -0.06193 | 0.212019 | EUR | Cirrhosis of liver without mention of alcohol | digestive |
| 465.2 | -0.03192 | 0.029778 | -1.07203 | 0.283706 | -0.09029 | 0.026441 | EUR | Acute pharyngitis | respiratory |
| 281.13 | -0.18847 | 0.175921 | -1.07134 | 0.284016 | -0.53327 | 0.156328 | EUR | Folate-deficiency anemia | hematopoietic |

| phecode | Coef. | Std.Err. | z | p_value_z | [0.025 | 0.975] | GIA | phenotype | category |
|---|---|---|---|---|---|---|---|---|---|
| 535.1 | 0.084738 | 0.079101 | 1.071273 | 0.284047 | -0.0703 | 0.239773 | EUR | Acute gastritis | digestive |
| 350.3 | -0.0855 | 0.07986 | -1.0706 | 0.284351 | -0.24202 | 0.071025 | EUR | Lack of coordination | neurological |
| 795 | 0.081356 | 0.076161 | 1.068224 | 0.28542 | -0.06792 | 0.230628 | EUR | Other and nonspecific abnormal cytological, histological and immunological findings | symptoms |
| 803.2 | -0.07461 | 0.069905 | -1.0673 | 0.285838 | -0.21162 | 0.062402 | EUR | Fracture of radius and ulna | injuries & poisonings |
| 242.3 | -0.14161 | 0.132938 | -1.06525 | 0.286763 | -0.40217 | 0.118941 | EUR | Exophthalmos | endocrine/metabolic |
| 275.2 | 0.289886 | 0.272621 | 1.063329 | 0.287633 | -0.24444 | 0.824214 | EUR | Disorders of copper metabolism | endocrine/metabolic |
| 184.1 | -0.09055 | 0.0852 | -1.06276 | 0.287892 | -0.25754 | 0.076442 | EUR | Malignant neoplasm of ovary and other uterine adnexa | neoplasms |
| 284 | 0.050559 | 0.047589 | 1.062406 | 0.288052 | -0.04271 | 0.143832 | EUR | Aplastic anemia | hematopoietic |
| 180 | 0.072596 | 0.068555 | 1.058941 | 0.289627 | -0.06177 | 0.206962 | EUR | Cervical cancer and dysplasia | neoplasms |
| 857 | 0.084329 | 0.079898 | 1.055457 | 0.291216 | -0.07227 | 0.240926 | EUR | Mechanical complication of unspecified genitourinary device, implant, and graft | injuries & poisonings |
| 350.5 | -0.13509 | 0.128297 | -1.05292 | 0.292377 | -0.38654 | 0.116371 | EUR | Abnormal reflex | neurological |
| 512 | 0.021803 | 0.020723 | 1.052127 | 0.292741 | -0.01881 | 0.062419 | EUR | Other symptoms of respiratory system | respiratory |
| 579.2 | 0.059461 | 0.056521 | 1.052014 | 0.292793 | -0.05132 | 0.170239 | EUR | Splenomegaly | digestive |
| 512.1 | 0.052801 | 0.050285 | 1.050034 | 0.293703 | -0.04576 | 0.151357 | EUR | Wheezing | respiratory |
| 987 | -0.54156 | 0.517403 | -1.04669 | 0.295241 | -1.55565 | 0.472529 | EUR | Toxic effect of other gases, fumes, or vapors | injuries & poisonings |
| 859 | -0.06416 | 0.06133 | -1.04623 | 0.295456 | -0.18437 | 0.056039 | EUR | Complication due to other implant and internal device | injuries & poisonings |
| 707 | 0.047999 | 0.045886 | 1.046062 | 0.295532 | -0.04193 | 0.137933 | EUR | Chronic ulcer of skin | dermatologic |
| 573.7 | 0.04944 | 0.047336 | 1.04446 | 0.296273 | -0.04334 | 0.142216 | EUR | Abnormal results of function study of liver | digestive |
| 175 | -0.05121 | 0.049077 | -1.04344 | 0.296743 | -0.1474 | 0.04498 | EUR | Acquired absence of breast | neoplasms |
| 695.4 | 0.074948 | 0.07198 | 1.041243 | 0.297763 | -0.06613 | 0.216026 | EUR | Lupus (localized and systemic) | dermatologic |
| 474.1 | -0.12668 | 0.121671 | -1.04115 | 0.297806 | -0.36515 | 0.111793 | EUR | Acute tonsillitis | respiratory |
| 526.4 | 0.061037 | 0.058675 | 1.040252 | 0.298223 | -0.05396 | 0.176039 | EUR | Temporomandibular joint disorders | digestive |
| 458 | 0.033759 | 0.032491 | 1.03902 | 0.298795 | -0.02992 | 0.097439 | EUR | Hypotension | circulatory system |
| 384.4 | -0.13737 | 0.132261 | -1.03862 | 0.298982 | -0.3966 | 0.121858 | EUR | Perforation of tympanic membrane | sense organs |
| 363.4 | 0.402161 | 0.388238 | 1.03586 | 0.300267 | -0.35877 | 1.163093 | EUR | Choroidal degenerations | sense organs |
| 368.5 | 0.317126 | 0.306489 | 1.034706 | 0.300806 | -0.28358 | 0.917834 | EUR | Color vision deficiencies | sense organs |
| 455 | -0.0242 | 0.023402 | -1.03416 | 0.301063 | -0.07007 | 0.021666 | EUR | Hemorrhoids | circulatory system |
| 721.8 | 0.075797 | 0.073379 | 1.032948 | 0.301628 | -0.06802 | 0.219618 | EUR | Other allied disorders of spine | musculoskeletal |
| 709.2 | 0.068574 | 0.066409 | 1.032608 | 0.301787 | -0.06158 | 0.198733 | EUR | Sicca syndrome | dermatologic |
| 636.2 | 0.210966 | 0.204386 | 1.032193 | 0.301982 | -0.18962 | 0.611556 | EUR | Early onset of delivery | pregnancy complications |
| 182 | -0.09053 | 0.087732 | -1.03192 | 0.302111 | -0.26248 | 0.081419 | EUR | Malignant neoplasm of uterus | neoplasms |
| 433.21 | 0.048464 | 0.046997 | 1.031208 | 0.302443 | -0.04365 | 0.140577 | EUR | Cerebral artery occlusion, with cerebral infarction | circulatory system |
| 965 | 0.047389 | 0.046167 | 1.026464 | 0.304673 | -0.0431 | 0.137875 | EUR | Poisoning by analgesics, antipyretics, and antirheumatics | injuries & poisonings |
| 565 | 0.031721 | 0.030905 | 1.026384 | 0.304711 | -0.02885 | 0.092294 | EUR | Anal and rectal conditions | digestive |
| 282.9 | -0.10338 | 0.100753 | -1.02602 | 0.304881 | -0.30085 | 0.094098 | EUR | Other hereditary hemolytic anemias | hematopoietic |
| 696.3 | -0.18058 | 0.176009 | -1.02599 | 0.304897 | -0.52555 | 0.164388 | EUR | Pityriasis | dermatologic |
| 149.1 | -0.12945 | 0.126187 | -1.02585 | 0.304964 | -0.37677 | 0.117873 | EUR | Cancer of oropharynx | neoplasms |
| 444.2 | -0.34787 | 0.339468 | -1.02475 | 0.305481 | -1.01321 | 0.317475 | EUR | Embolism and thrombosis of abdominal aorta | circulatory system |
| 202.21 | -0.13566 | 0.13261 | -1.02296 | 0.306327 | -0.39557 | 0.124257 | EUR | Nodular lymphoma | neoplasms |
| 801 | 0.050547 | 0.049476 | 1.021634 | 0.306954 | -0.04643 | 0.147519 | EUR | Fracture of ankle and foot | injuries & poisonings |
| 802 | -0.13473 | 0.13213 | -1.01971 | 0.307865 | -0.3937 | 0.124235 | EUR | Fracture of pelvis | injuries & poisonings |
| 172.21 | -0.0346 | 0.033935 | -1.01969 | 0.307873 | -0.10112 | 0.031908 | EUR | Basal cell carcinoma | neoplasms |
| 585.4 | 0.044739 | 0.043905 | 1.018997 | 0.308204 | -0.04131 | 0.130792 | EUR | Chronic kidney disease, Stage I or II | genitourinary |
| 535.8 | 0.066714 | 0.06554 | 1.017899 | 0.308726 | -0.06174 | 0.19517 | EUR | Other specified gastritis | digestive |
| 227.3 | -0.09832 | 0.09673 | -1.01643 | 0.309424 | -0.28791 | 0.091268 | EUR | Benign neoplasm of pituitary gland and craniopharyngeal duct (pouch) | neoplasms |
| 750.11 | -0.1193 | 0.117433 | -1.01591 | 0.309673 | -0.34946 | 0.110863 | EUR | Esophageal atresia/tracheoesophageal fistula | congenital anomalies |
| 540.11 | 0.100797 | 0.09927 | 1.015382 | 0.309924 | -0.09377 | 0.295362 | EUR | Acute appendicitis | digestive |
| 204.11 | -0.17234 | 0.169791 | -1.01501 | 0.310102 | -0.50512 | 0.160445 | EUR | Lymphoid leukemia, acute | neoplasms |
| 656.8 | 0.775528 | 0.764648 | 1.014229 | 0.310473 | -0.72315 | 2.27421 | EUR | Perinatal jaundice | pregnancy complications |
| 710.2 | -0.77183 | 0.761213 | -1.01395 | 0.310607 | -2.26378 | 0.720119 | EUR | Periostitis | musculoskeletal |
| 333.3 | 0.300251 | 0.296225 | 1.013591 | 0.310778 | -0.28034 | 0.880842 | EUR | Tics and choreas | neurological |
| 214 | 0.045278 | 0.044727 | 1.01231 | 0.31139 | -0.04239 | 0.132941 | EUR | Lipoma | neoplasms |
| 575.1 | -0.10926 | 0.108229 | -1.00956 | 0.312704 | -0.32139 | 0.102861 | EUR | Cholangitis | digestive |
| 189.4 | -0.13448 | 0.133446 | -1.00775 | 0.313573 | -0.39603 | 0.127068 | EUR | Malignant neoplasm of other urinary organs | neoplasms |
| 792 | 0.0494 | 0.049036 | 1.007418 | 0.313734 | -0.04671 | 0.14551 | EUR | Abnormal Papanicolaou smear of cervix and cervical HPV | genitourinary |
| 594.1 | 0.034005 | 0.033812 | 1.005715 | 0.314553 | -0.03227 | 0.100276 | EUR | Calculus of kidney | genitourinary |
| 352.1 | 0.103456 | 0.102904 | 1.005363 | 0.314722 | -0.09823 | 0.305144 | EUR | Trigeminal nerve disorders [CN5] | neurological |
| 624 | -0.05238 | 0.052174 | -1.00393 | 0.31541 | -0.15464 | 0.04988 | EUR | Symptoms involving female genital tract | genitourinary |
| 856 | 0.18825 | 0.188006 | 1.001302 | 0.316681 | -0.18023 | 0.556735 | EUR | Vascular complications of surgery and medical procedures | injuries & poisonings |

| phecode | Coef. | Std.Err. | z | p_value_z | [0.025 | 0.975] | GIA | phenotype | category |
|---|---|---|---|---|---|---|---|---|---|
| 496.21 | 0.098124 | 0.098149 | 0.999744 | 0.317435 | -0.09424 | 0.290493 | EUR | Obstructive chronic bronchitis | respiratory |
| 459.7 | 0.052579 | 0.052648 | 0.998684 | 0.317948 | -0.05061 | 0.155768 | EUR | Blood vessel replaced | circulatory system |
| 385 | 0.142391 | 0.142917 | 0.996316 | 0.319097 | -0.13772 | 0.422504 | EUR | Other disorders of middle ear and mastoid | sense organs |
| 714.1 | 0.055827 | 0.056094 | 0.995247 | 0.319616 | -0.05411 | 0.165769 | EUR | Rheumatoid arthritis | musculoskeletal |
| 31 | 0.122341 | 0.123202 | 0.993015 | 3.21E-01 | -0.11913 | 0.363812 | EUR | Diseases due to other mycobacteria | infectious diseases |
| 81.12 | 0.211877 | 0.213486 | 0.992466 | 0.32097 | -0.20655 | 0.630302 | EUR | Chronic graft-versus-host disease | infectious diseases |
| 773 | -0.0209 | 0.021069 | -0.99218 | 0.32111 | -0.0622 | 0.02039 | EUR | Pain in limb | symptoms |
| 218.1 | 0.042243 | 0.042578 | 0.99214 | 0.321129 | -0.04121 | 0.125694 | EUR | Uterine leiomyoma | neoplasms |
| 870.6 | 0.301766 | 0.304881 | 0.989784 | 0.322279 | -0.29579 | 0.899321 | EUR | Open wound of neck | injuries & poisonings |
| 350.1 | -0.0341 | 0.034509 | -0.98816 | 0.323076 | -0.10174 | 0.033536 | EUR | Abnormal involuntary movements | neurological |
| 303 | 0.059684 | 0.060454 | 0.987278 | 0.323506 | -0.0588 | 0.178171 | EUR | Psychogenic and somatoform disorders | mental disorders |
| 149.3 | 0.444894 | 0.451189 | 0.986048 | 0.32411 | -0.43942 | 1.329209 | EUR | Cancer of hypopharynx | neoplasms |
| 367.9 | 0.052074 | 0.052857 | 0.985178 | 0.324537 | -0.05152 | 0.155672 | EUR | Blindness and low vision | sense organs |
| 560 | 0.038571 | 0.03919 | 0.984223 | 0.325006 | -0.03824 | 0.115381 | EUR | Intestinal obstruction without mention of hernia | digestive |
| 446.9 | -0.11272 | 0.114545 | -0.9841 | 0.325067 | -0.33723 | 0.11178 | EUR | Arteritis NOS | circulatory system |
| 464 | 0.028719 | 0.029218 | 0.982928 | 0.325643 | -0.02855 | 0.085985 | EUR | Acute sinusitis | respiratory |
| 574 | 0.039349 | 0.040152 | 0.979999 | 0.327086 | -0.03935 | 0.118046 | EUR | Cholelithiasis and cholecystitis | digestive |
| 715.1 | 0.050866 | 0.051931 | 0.979486 | 0.32734 | -0.05092 | 0.152649 | EUR | Sacroiliitis NEC | musculoskeletal |
| 286.12 | -0.18204 | 0.185858 | -0.97945 | 0.327358 | -0.54631 | 0.182236 | EUR | Congenital deficiency of other clotting factors (including factor VII) | hematopoietic |
| 442.4 | -0.14575 | 0.149021 | -0.97805 | 0.32805 | -0.43782 | 0.146326 | EUR | Arterial dissection | circulatory system |
| 454.11 | 0.051648 | 0.052812 | 0.977963 | 0.328093 | -0.05186 | 0.155158 | EUR | Varicose veins of lower extremity, symptomtic | circulatory system |
| 574.12 | -0.08667 | 0.088656 | -0.97761 | 0.328266 | -0.26043 | 0.087091 | EUR | Cholelithiasis with other cholecystitis | digestive |
| 585 | 0.02589 | 0.026515 | 0.976434 | 0.32885 | -0.02608 | 0.077858 | EUR | Renal failure | genitourinary |
| 530.12 | 0.068236 | 0.069885 | 0.97641 | 0.328861 | -0.06874 | 0.205208 | EUR | Ulcer of esophagus | digestive |
| 1007 | -0.16448 | 0.168462 | -0.97636 | 0.328886 | -0.49466 | 0.1657 | EUR | Injury to blood vessels | other |
| 381.9 | -0.09186 | 0.094243 | -0.97468 | 0.32972 | -0.27657 | 0.092856 | EUR | Otorrhea | sense organs |
| 613.9 | -0.11203 | 0.114964 | -0.97448 | 0.329821 | -0.33735 | 0.113295 | EUR | Breast disorder NOS | genitourinary |
| 574.11 | -0.11434 | 0.117461 | -0.97344 | 0.330335 | -0.34456 | 0.115878 | EUR | Cholelithiasis with acute cholecystitis | digestive |
| 930 | 0.0391 | 0.040186 | 0.972971 | 0.330568 | -0.03966 | 0.117863 | EUR | Allergic reaction to food | injuries & poisonings |
| 756.1 | -0.21344 | 0.219576 | -0.97204 | 0.33103 | -0.6438 | 0.216924 | EUR | Congenital anomalies of abdominal wall; diaphram | congenital anomalies |
| 327.41 | -0.03883 | 0.040101 | -0.96829 | 0.3329 | -0.11743 | 0.039768 | EUR | Organic or persistent insomnia | neurological |
| 654 | -0.05217 | 0.053909 | -0.96767 | 0.333208 | -0.15783 | 0.053493 | EUR | Other and unspecified complications of birth; puerperium affecting management of mother | pregnancy complications |
| 1008 | 0.116672 | 0.120756 | 0.966178 | 0.333955 | -0.12001 | 0.353349 | EUR | Crushing or internal injury to organs | other |
| 803.3 | -0.09624 | 0.099633 | -0.96597 | 0.33406 | -0.29152 | 0.099034 | EUR | Fracture of clavicle or scapula | injuries & poisonings |
| 276.5 | -0.0313 | 0.03244 | -0.96477 | 0.334659 | -0.09488 | 0.032284 | EUR | Hypovolemia | endocrine/metabolic |
| 379.51 | -0.23875 | 0.247531 | -0.96452 | 0.334786 | -0.7239 | 0.246403 | EUR | Pigmentary iris degeneration | sense organs |
| 81 | 0.06022 | 0.062455 | 0.964212 | 0.33494 | -0.06219 | 0.18263 | EUR | Infection/inflammation of internal prosthetic device; implant; and graft | infectious diseases |
| 722.8 | 0.070701 | 0.073523 | 0.96161 | 0.336245 | -0.0734 | 0.214803 | EUR | Postlaminectomy syndrome | musculoskeletal |
| 609.11 | 0.236077 | 0.245981 | 0.959737 | 0.337188 | -0.24604 | 0.718191 | EUR | Azoospermia and oligospermia | genitourinary |
| 8.5 | -0.0552 | 0.057515 | -0.95966 | 3.37E-01 | -0.16792 | 0.057532 | EUR | Bacterial enteritis | infectious diseases |
| 285.8 | 0.232368 | 0.242371 | 0.95873 | 0.337695 | -0.24267 | 0.707406 | EUR | Hemoglobinuria | hematopoietic |
| 636.1 | -0.15385 | 0.1606 | -0.95796 | 0.338084 | -0.46862 | 0.160922 | EUR | Threatened premature labor | pregnancy complications |
| 704 | -0.02804 | 0.029283 | -0.95763 | 0.338247 | -0.08543 | 0.029351 | EUR | Diseases of hair and hair follicles | dermatologic |
| 191.1 | -0.07506 | 0.078541 | -0.95567 | 0.33924 | -0.229 | 0.078878 | EUR | Cancer of brain and nervous system | neoplasms |
| 619.5 | -0.06594 | 0.069153 | -0.95359 | 0.340292 | -0.20148 | 0.069594 | EUR | Noninflammatory disorders of vulva and perineum | genitourinary |
| 527 | 0.048103 | 0.050445 | 0.953587 | 0.340293 | -0.05077 | 0.146973 | EUR | Diseases of the salivary glands | digestive |
| 195.1 | -0.02625 | 0.027529 | -0.95338 | 0.340397 | -0.0802 | 0.02771 | EUR | Malignant neoplasm, other | neoplasms |
| 301.1 | 0.315307 | 0.331472 | 0.951233 | 0.341486 | -0.33437 | 0.964979 | EUR | Schizoid personality disorder | mental disorders |
| 367.2 | -0.04233 | 0.04454 | -0.95043 | 0.341891 | -0.12963 | 0.044965 | EUR | Astigmatism | sense organs |
| 817 | 0.062786 | 0.066069 | 0.95031 | 0.341955 | -0.06671 | 0.192279 | EUR | Concussion | injuries & poisonings |
| 385.3 | 0.20223 | 0.213663 | 0.946494 | 0.343897 | -0.21654 | 0.621001 | EUR | Cholesteatoma | sense organs |
| 359.2 | 0.068957 | 0.072871 | 0.94629 | 0.344 | -0.07387 | 0.211782 | EUR | Myopathy | neurological |
| 174 | -0.03938 | 0.041651 | -0.94553 | 0.344389 | -0.12102 | 0.042252 | EUR | Breast cancer | neoplasms |
| 1100 | -0.06762 | 0.071561 | -0.94496 | 0.34468 | -0.20788 | 0.072635 | EUR | Family history | other |
| 686.1 | -0.0923 | 0.097773 | -0.94404 | 0.34515 | -0.28393 | 0.099331 | EUR | Carbuncle and furuncle | dermatologic |
| 275.53 | 0.054456 | 0.057835 | 0.941582 | 0.346406 | -0.0589 | 0.16781 | EUR | Disorders of phosphorus metabolism | endocrine/metabolic |
| 614.3 | -0.12706 | 0.134994 | -0.94122 | 0.346591 | -0.39164 | 0.137524 | EUR | Pelvic inflammatory disease (PID) | genitourinary |
| 499 | 0.181012 | 0.192687 | 0.939409 | 0.347521 | -0.19665 | 0.55867 | EUR | Cystic fibrosis | respiratory |
| 870.5 | 0.104861 | 0.111676 | 0.938979 | 0.347741 | -0.11402 | 0.323741 | EUR | Open wound of lip and mouth | injuries & poisonings |

| phecode | Coef. | Std.Err. | z | p_value_z | [0.025 | 0.975] | GIA | phenotype | category |
|---|---|---|---|---|---|---|---|---|---|
| 426.4 | 0.167294 | 0.178539 | 0.937015 | 0.348751 | -0.18264 | 0.517224 | EUR | Anomalous atrioventricular excitation | circulatory system |
| 871.2 | -0.05871 | 0.062743 | -0.93576 | 0.349398 | -0.18169 | 0.064262 | EUR | Open wound of finger(s) | injuries & poisonings |
| 327.71 | 0.059144 | 0.063228 | 0.935418 | 0.349573 | -0.06478 | 0.183068 | EUR | Restless legs syndrome | neurological |
| 753.1 | 0.30224 | 0.323881 | 0.933181 | 0.350727 | -0.33256 | 0.937035 | EUR | Congenital cataract and lens anomalies | congenital anomalies |
| 840.3 | 0.05726 | 0.061549 | 0.930314 | 0.352209 | -0.06337 | 0.177893 | EUR | Joint/ligament sprain | injuries & poisonings |
| 609.2 | 0.127703 | 0.137483 | 0.928867 | 0.352958 | -0.14176 | 0.397165 | EUR | Abnormal spermatozoa | genitourinary |
| 195 | -0.0246 | 0.026503 | -0.92807 | 0.353371 | -0.07654 | 0.027348 | EUR | Cancer, suspected or other | neoplasms |
| 427.4 | 0.079822 | 0.086501 | 0.922786 | 0.356119 | -0.08972 | 0.24936 | EUR | Cardiac arrest and ventricular fibrillation | circulatory system |
| 255.11 | -0.15322 | 0.16634 | -0.92112 | 0.356986 | -0.47924 | 0.172801 | EUR | Cushing's syndrome | endocrine/metabolic |
| 345.1 | -0.06259 | 0.0681 | -0.91905 | 0.35807 | -0.19606 | 0.070886 | EUR | Epilepsy | neurological |
| 280.2 | 0.044882 | 0.048927 | 0.917308 | 0.358981 | -0.05101 | 0.140777 | EUR | Iron deficiency anemia secondary to blood loss (chronic) | hematopoietic |
| 634 | -0.05347 | 0.058314 | -0.91701 | 0.359138 | -0.16777 | 0.060819 | EUR | Miscarriage; stillbirth | pregnancy complications |
| 696.4 | 0.044936 | 0.049081 | 0.915546 | 0.359905 | -0.05126 | 0.141134 | EUR | Psoriasis | dermatologic |
| 271 | -0.02305 | 0.025244 | -0.91321 | 0.361131 | -0.07253 | 0.026425 | EUR | Disorders of carbohydrate transport and metabolism | endocrine/metabolic |
| 198.2 | 0.056189 | 0.061563 | 0.91271 | 0.361395 | -0.06447 | 0.17685 | EUR | Secondary malignancy of respiratory organs | neoplasms |
| 41.12 | 0.082724 | 0.090896 | 0.910095 | 0.362772 | -0.09543 | 0.260876 | EUR | Methicillin resistant Staphylococcus aureus | infectious diseases |
| 938.1 | -0.09843 | 0.108249 | -0.90928 | 0.363203 | -0.31059 | 0.113736 | EUR | Acute dermatitis due to solar radiation | dermatologic |
| 223 | 0.110902 | 0.122017 | 0.908906 | 0.3634 | -0.12825 | 0.35005 | EUR | Benign neoplasm of kidney and other urinary organs | neoplasms |
| 218 | 0.03858 | 0.042456 | 0.908693 | 0.363512 | -0.04463 | 0.121793 | EUR | Benign neoplasm of uterus | neoplasms |
| 53.1 | -0.08999 | 0.099081 | -0.90824 | 0.363753 | -0.28418 | 0.104206 | EUR | Herpes zoster with nervous system complications | infectious diseases |
| 947 | 0.046608 | 0.051344 | 0.907774 | 0.363998 | -0.05402 | 0.14724 | EUR | Urticaria | dermatologic |
| 170.2 | 0.063283 | 0.069731 | 0.907534 | 0.364124 | -0.07339 | 0.199953 | EUR | Cancer of connective tissue | neoplasms |
| 536.7 | -0.09314 | 0.10277 | -0.90632 | 0.364764 | -0.29457 | 0.108283 | EUR | Complications of gastrostomy, colostomy and enterostomy | digestive |
| 580.2 | 0.086706 | 0.095861 | 0.904499 | 0.365731 | -0.10118 | 0.274591 | EUR | Nephrotic syndrome without mention of glomerulonephritis | genitourinary |
| 781.2 | -0.08309 | 0.091914 | -0.90398 | 0.366007 | -0.26324 | 0.09706 | EUR | Abnormal posture | symptoms |
| 401.22 | 0.034837 | 0.038602 | 0.902475 | 0.366804 | -0.04082 | 0.110495 | EUR | Hypertensive chronic kidney disease | circulatory system |
| 289 | 0.030332 | 0.033623 | 0.902111 | 0.366998 | -0.03557 | 0.096231 | EUR | Other diseases of blood and blood-forming organs | hematopoietic |
| 427.6 | 0.035256 | 0.039178 | 0.899911 | 0.368168 | -0.04153 | 0.112043 | EUR | Premature beats | circulatory system |
| 110.13 | -0.05093 | 0.056772 | -0.89701 | 0.369714 | -0.1622 | 0.060347 | EUR | Dermatophytosis of the body | infectious diseases |
| 715.3 | 0.151071 | 0.168761 | 0.895182 | 0.37069 | -0.17969 | 0.481836 | EUR | Spinal enthesopathy | musculoskeletal |
| 705 | -0.07889 | 0.088305 | -0.89337 | 0.371661 | -0.25196 | 0.094185 | EUR | Disorders of sweat glands | dermatologic |
| 701.3 | -0.08208 | 0.091992 | -0.8923 | 0.372231 | -0.26239 | 0.098217 | EUR | Circumscribed scleroderma | dermatologic |
| 702.1 | -0.02483 | 0.027873 | -0.89073 | 0.373073 | -0.07946 | 0.029802 | EUR | Actinic keratosis | dermatologic |
| 353 | 0.059152 | 0.066471 | 0.889887 | 0.373526 | -0.07113 | 0.189432 | EUR | Nerve root and plexus disorders | neurological |
| 290.2 | 0.075289 | 0.084673 | 0.889178 | 0.373907 | -0.09067 | 0.241245 | EUR | Delirium due to conditions classified elsewhere | mental disorders |
| 743 | -0.0229 | 0.025796 | -0.88759 | 0.37476 | -0.07346 | 0.027663 | EUR | Osteoporosis, osteopenia and pathological fracture | musculoskeletal |
| 588 | 0.053058 | 0.059846 | 0.886566 | 0.375312 | -0.06424 | 0.170354 | EUR | Disorders resulting from impaired renal function | genitourinary |
| 626.12 | 0.050511 | 0.057139 | 0.884009 | 0.376692 | -0.06148 | 0.162501 | EUR | Excessive or frequent menstruation | genitourinary |
| 365.2 | -0.08639 | 0.097818 | -0.8832 | 0.377128 | -0.27811 | 0.105327 | EUR | Primary angle-closure glaucoma | sense organs |
| 368.9 | 0.055721 | 0.063097 | 0.883099 | 0.377183 | -0.06795 | 0.179389 | EUR | Subjective visual disturbances | sense organs |
| 430 | 0.063566 | 0.072255 | 0.87975 | 0.378994 | -0.07805 | 0.205183 | EUR | Intracranial hemorrhage | circulatory system |
| 342 | -0.09293 | 0.105718 | -0.87903 | 0.379386 | -0.30013 | 0.114274 | EUR | Hemiplegia | neurological |
| 361 | -0.05619 | 0.064074 | -0.87695 | 0.380513 | -0.18177 | 0.069393 | EUR | Retinal detachments and defects | sense organs |
| 756.22 | -0.65655 | 0.749312 | -0.8762 | 0.380919 | -2.12517 | 0.812074 | EUR | Pectus carinatum | congenital anomalies |
| 346.2 | -0.13485 | 0.153988 | -0.87572 | 0.381181 | -0.43666 | 0.16696 | EUR | Nonspecific abnormal results of function study of brain and central nervous system | neurological |
| 618.6 | -0.1981 | 0.22722 | -0.87186 | 0.383285 | -0.64345 | 0.24724 | EUR | Vaginal enterocele, congenital or acquired | genitourinary |
| 527.7 | 0.053511 | 0.061425 | 0.871159 | 0.383667 | -0.06688 | 0.1739 | EUR | Disturbance of salivary secretion | digestive |
| 283.21 | 0.420799 | 0.4838 | 0.86978 | 0.384421 | -0.52743 | 1.36903 | EUR | Hemolytic-uremic syndrome | hematopoietic |
| 738 | 0.035235 | 0.040531 | 0.869349 | 0.384656 | -0.0442 | 0.114674 | EUR | Other acquired musculoskeletal deformity | musculoskeletal |
| 149.5 | 0.093067 | 0.107067 | 0.869233 | 0.384719 | -0.11678 | 0.302915 | EUR | Hx of malignant neoplasm of oral cavity and pharynx | neoplasms |
| 911 | -0.09939 | 0.114527 | -0.86781 | 0.385498 | -0.32386 | 0.125081 | EUR | Blister | injuries & poisonings |
| 261 | -0.02051 | 0.023652 | -0.867 | 0.38594 | -0.06686 | 0.025851 | EUR | Vitamin deficiency | endocrine/metabolic |
| 255.22 | -0.92098 | 1.064298 | -0.86534 | 0.386851 | -3.00697 | 1.165003 | EUR | Mineralocorticoid deficiency | endocrine/metabolic |
| 550.2 | 0.026679 | 0.030977 | 0.861267 | 0.389091 | -0.03403 | 0.087393 | EUR | Diaphragmatic hernia | digestive |
| 756 | -0.06708 | 0.077925 | -0.86077 | 0.389363 | -0.21981 | 0.085654 | EUR | Other congenital musculoskeletal anomalies | congenital anomalies |
| 577.2 | 0.086728 | 0.100975 | 0.858905 | 0.390393 | -0.11118 | 0.284636 | EUR | Chronic pancreatitis | digestive |
| 750.13 | -0.08453 | 0.098603 | -0.85729 | 0.391286 | -0.27779 | 0.108728 | EUR | Congenital anomalies of mouth/tongue | congenital anomalies |
| 733.6 | 0.069557 | 0.081296 | 0.855595 | 0.392222 | -0.08978 | 0.228895 | EUR | Costochondritis | musculoskeletal |
| 612.3 | 0.194477 | 0.227641 | 0.854312 | 0.392932 | -0.25169 | 0.640645 | EUR | Congenital anomalies of breast | genitourinary |

| phecode | Coef. | Std.Err. | z | p_value_z | [0.025 | 0.975] | GIA | phenotype | category |
|---|---|---|---|---|---|---|---|---|---|
| 528.3 | 0.16201 | 0.189848 | 0.853369 | 0.393455 | -0.21008 | 0.534105 | EUR | Cellulitis and abscess of oral soft tissues | digestive |
| 628 | 0.040655 | 0.047671 | 0.852823 | 0.393757 | -0.05278 | 0.134088 | EUR | Ovarian cyst | genitourinary |
| 350.2 | -0.03076 | 0.03608 | -0.85245 | 0.393963 | -0.10147 | 0.039959 | EUR | Abnormality of gait | neurological |
| 523.32 | -1.02122 | 1.198092 | -0.85237 | 0.394008 | -3.36944 | 1.326998 | EUR | Chronic periodontitis | digestive |
| 364.41 | 0.166572 | 0.195568 | 0.851735 | 0.394361 | -0.21673 | 0.549878 | EUR | Keratoconus | sense organs |
| 975 | 0.216756 | 0.256004 | 0.846688 | 0.397169 | -0.285 | 0.718515 | EUR | Poisoning by agents primarily acting on the smooth and skeletal muscles and respiratory system | injuries & poisonings |
| 454 | 0.0329 | 0.038884 | 0.846103 | 0.397495 | -0.04331 | 0.10911 | EUR | Varicose veins | circulatory system |
| 259.1 | -0.28347 | 0.335159 | -0.84576 | 0.397686 | -0.94037 | 0.373435 | EUR | Nonspecific abnormal results of other endocrine function study | endocrine/metabolic |
| 334 | -0.05555 | 0.065709 | -0.8454 | 0.397887 | -0.18434 | 0.073237 | EUR | Degenerative disease of the spinal cord | neurological |
| 253.5 | 0.24218 | 0.286871 | 0.844213 | 0.39855 | -0.32008 | 0.804437 | EUR | Pituitary dwarfism | endocrine/metabolic |
| 525 | 0.076542 | 0.090809 | 0.842893 | 0.399288 | -0.10144 | 0.254524 | EUR | Other diseases of the teeth and supporting structures | digestive |
| 271.3 | -0.02129 | 0.025353 | -0.83957 | 0.40115 | -0.07098 | 0.028406 | EUR | Intestinal disaccharidase deficiencies and disaccharide malabsorption | endocrine/metabolic |
| 286.6 | 0.088615 | 0.105604 | 0.839124 | 0.4014 | -0.11837 | 0.295596 | EUR | Defibrination syndrome | hematopoietic |
| 189 | 0.049636 | 0.059198 | 0.838461 | 0.401772 | -0.06639 | 0.165662 | EUR | Cancer of urinary organs (incl. kidney and bladder) | neoplasms |
| 362.2 | -0.03634 | 0.043488 | -0.83568 | 0.403333 | -0.12158 | 0.048893 | EUR | Degeneration of macula and posterior pole of retina | sense organs |
| 353.1 | 0.067033 | 0.080292 | 0.834869 | 0.403792 | -0.09034 | 0.224402 | EUR | Nerve plexus lesions | neurological |
| 134 | 0.138452 | 0.165898 | 0.83456 | 0.403966 | -0.1867 | 0.463607 | EUR | Helminthiases | infectious diseases |
| 480.5 | 0.075074 | 0.090378 | 0.830674 | 0.406158 | -0.10206 | 0.252211 | EUR | Bronchopneumonia and lung abscess | respiratory |
| 783.1 | -0.08173 | 0.098396 | -0.83058 | 0.40621 | -0.27458 | 0.111126 | EUR | Postprocedural fever | symptoms |
| 279.11 | 0.051955 | 0.06262 | 0.829682 | 0.406719 | -0.07078 | 0.174688 | EUR | Deficiency of humoral immunity | endocrine/metabolic |
| 627.3 | -0.0376 | 0.045438 | -0.82754 | 0.407929 | -0.12666 | 0.051455 | EUR | Postmenopausal atrophic vaginitis | genitourinary |
| 365.11 | 0.065433 | 0.079119 | 0.827023 | 0.408224 | -0.08964 | 0.220503 | EUR | Primary open angle glaucoma | sense organs |
| 362.27 | -0.06938 | 0.084007 | -0.82593 | 0.408841 | -0.23404 | 0.095266 | EUR | Drusen (degenerative) of retina | sense organs |
| 709.6 | 0.098408 | 0.11921 | 0.825506 | 0.409085 | -0.13524 | 0.332055 | EUR | Other specified diffuse diseases of connective tissue | dermatologic |
| 286.11 | 0.216379 | 0.262149 | 0.825402 | 0.409143 | -0.29742 | 0.730181 | EUR | Von willebrand's disease | hematopoietic |
| 174.1 | -0.03474 | 0.04215 | -0.82413 | 0.409868 | -0.11735 | 0.047875 | EUR | Breast cancer [female] | neoplasms |
| 444.1 | -0.11156 | 0.135467 | -0.82351 | 0.41022 | -0.37707 | 0.153952 | EUR | Arterial embolism and thrombosis of lower extremity artery | circulatory system |
| 338.1 | 0.018468 | 0.022435 | 0.823204 | 0.410392 | -0.0255 | 0.06244 | EUR | Acute pain | neurological |
| 560.2 | 0.061027 | 0.074211 | 0.822347 | 0.410879 | -0.08442 | 0.206477 | EUR | Impaction of intestine | digestive |
| 193 | -0.05288 | 0.064337 | -0.82189 | 0.411138 | -0.17898 | 0.07322 | EUR | Thyroid cancer | neoplasms |
| 343 | 0.167039 | 0.203241 | 0.821875 | 0.411148 | -0.23131 | 0.565385 | EUR | Infantile cerebral palsy | neurological |
| 240 | -0.05384 | 0.065519 | -0.8217 | 0.41125 | -0.18225 | 0.074578 | EUR | Simple and unspecified goiter | endocrine/metabolic |
| 389.4 | -0.03008 | 0.036773 | -0.81793 | 0.413399 | -0.10215 | 0.041996 | EUR | Tinnitus | sense organs |
| 41.9 | -0.0701 | 0.085714 | -0.81786 | 0.413439 | -0.2381 | 0.097895 | EUR | Infection with drug-resistant microorganisms | infectious diseases |
| 637 | -0.14579 | 0.178357 | -0.81743 | 0.413683 | -0.49537 | 0.20378 | EUR | Short gestation; low birth weight; and fetal growth retardation | pregnancy complications |
| 153.2 | -0.0486 | 0.059512 | -0.81663 | 0.414139 | -0.16524 | 0.068042 | EUR | Colon cancer | neoplasms |
| 368.2 | 0.053332 | 0.065307 | 0.816624 | 0.414143 | -0.07467 | 0.181332 | EUR | Diplopia and disorders of binocular vision | sense organs |
| 755.61 | 0.145807 | 0.178862 | 0.815197 | 0.414959 | -0.20475 | 0.49637 | EUR | Congenital hip dysplasia and deformity | congenital anomalies |
| 578 | -0.02487 | 0.030579 | -0.81337 | 0.416005 | -0.08481 | 0.035062 | EUR | Gastrointestinal hemorrhage | digestive |
| 537.1 | 0.144221 | 0.177362 | 0.813148 | 0.416133 | -0.2034 | 0.491844 | EUR | Lesions of stomach and duodenum | digestive |
| 433.1 | 0.034297 | 0.042223 | 0.812298 | 0.416621 | -0.04846 | 0.117052 | EUR | Occlusion and stenosis of precerebral arteries | circulatory system |
| 701.4 | 0.04996 | 0.061577 | 0.81134 | 0.417171 | -0.07073 | 0.170648 | EUR | Keloid scar | dermatologic |
| 41.11 | 0.069979 | 0.086267 | 0.811196 | 0.417253 | -0.0991 | 0.239059 | EUR | Methicillin sensitive Staphylococcus aureus | infectious diseases |
| 870.1 | -0.10627 | 0.131049 | -0.81093 | 0.417408 | -0.36312 | 0.150581 | EUR | Open wound or laceration of eye or eyelid | injuries & poisonings |
| 277.2 | -0.87587 | 1.080796 | -0.81039 | 0.417715 | -2.99419 | 1.242454 | EUR | Other disorders of purine and pyrimidine metabolism | endocrine/metabolic |
| 785 | -0.01674 | 0.020656 | -0.8102 | 0.417823 | -0.05722 | 0.023749 | EUR | Abdominal pain | symptoms |
| 476 | -0.01909 | 0.023588 | -0.80932 | 0.418329 | -0.06532 | 0.027142 | EUR | Allergic rhinitis | respiratory |
| 480.1 | 0.039803 | 0.049192 | 0.809134 | 0.418438 | -0.05661 | 0.136218 | EUR | Bacterial pneumonia | respiratory |
| 473 | 0.027939 | 0.03455 | 0.808653 | 0.418715 | -0.03978 | 0.095656 | EUR | Diseases of the larynx and vocal cords | respiratory |
| 557 | 0.053286 | 0.065957 | 0.807887 | 0.419156 | -0.07599 | 0.182559 | EUR | Intestinal malabsorption (non-celiac) | digestive |
| 913 | -0.09025 | 0.111743 | -0.80769 | 0.419268 | -0.30926 | 0.128758 | EUR | Toxic effect of venom | injuries & poisonings |
| 716.3 | 0.833031 | 1.032155 | 0.80708 | 0.419621 | -1.18995 | 2.856017 | EUR | Kaschin-Beck disease | musculoskeletal |
| 381 | -0.02834 | 0.035146 | -0.80637 | 0.420029 | -0.09723 | 0.040545 | EUR | Otitis media and Eustachian tube disorders | sense organs |
| 371.9 | 0.428059 | 0.532026 | 0.804583 | 0.42106 | -0.61469 | 1.47081 | EUR | Chronic inflammatory disorders of orbit | sense organs |
| 876 | -0.07412 | 0.092132 | -0.80448 | 0.421122 | -0.25469 | 0.106457 | EUR | Posttraumatic wound infection not elsewhere classified | injuries & poisonings |
| 724.1 | 0.033841 | 0.042146 | 0.802956 | 0.422 | -0.04876 | 0.116446 | EUR | Disorders of sacrum | musculoskeletal |
| 836 | 0.124515 | 0.155132 | 0.802635 | 0.422186 | -0.17954 | 0.428569 | EUR | Traumatic arthropathy | injuries & poisonings |
| 759 | 0.065781 | 0.081969 | 0.802514 | 0.422256 | -0.09487 | 0.226437 | EUR | Other and unspecified congenital anomalies | congenital anomalies |
| 366.3 | 0.451593 | 0.562798 | 0.802407 | 0.422318 | -0.65147 | 1.554657 | EUR | Traumatic cataract | sense organs |

| phecode | Coef. | Std.Err. | z | p_value_z | [0.025 | 0.975] | GIA | phenotype | category |
|---|---|---|---|---|---|---|---|---|---|
| 842 | -0.0262 | 0.032677 | -0.80194 | 0.422589 | -0.09025 | 0.037841 | EUR | Other sprains and strains | injuries & poisonings |
| 571.6 | -0.11206 | 0.139893 | -0.80101 | 0.423123 | -0.38624 | 0.162129 | EUR | Primary biliary cirrhosis | digestive |
| 657 | -0.47808 | 0.59733 | -0.80037 | 0.423497 | -1.64883 | 0.692661 | EUR | Infections specific to the perinatal period | pregnancy complications |
| 117 | 0.051405 | 0.064337 | 0.798993 | 0.424295 | -0.07469 | 0.177504 | EUR | Mycoses | infectious diseases |
| 949.1 | 0.259541 | 0.325527 | 0.797294 | 0.42528 | -0.37848 | 0.897562 | EUR | Diaper or napkin rash | injuries & poisonings |
| 695.42 | 0.058328 | 0.073171 | 0.797151 | 0.425363 | -0.08508 | 0.201741 | EUR | Systemic lupus erythematosus | dermatologic |
| 355 | 0.067305 | 0.084649 | 0.795106 | 0.426552 | -0.0986 | 0.233215 | EUR | Complex regional/central pain syndrome | neurological |
| 246.2 | -0.09481 | 0.119479 | -0.79353 | 0.427467 | -0.32899 | 0.139364 | EUR | Thyroid cyst | endocrine/metabolic |
| 694.1 | 0.114071 | 0.1438 | 0.793263 | 0.427625 | -0.16777 | 0.395915 | EUR | Vitiligo | dermatologic |
| 710.3 | 0.578337 | 0.729154 | 0.793162 | 0.427684 | -0.85078 | 2.007453 | EUR | Osteopathy resulting from poliomyelitis | musculoskeletal |
| 758 | -0.08442 | 0.1067 | -0.79116 | 0.428853 | -0.29354 | 0.124712 | EUR | Chromosomal anomalies and genetic disorders | congenital anomalies |
| 575 | -0.03157 | 0.040035 | -0.78854 | 0.430379 | -0.11004 | 0.046898 | EUR | Other biliary tract disease | digestive |
| 530.3 | -0.0513 | 0.065073 | -0.7883 | 0.430524 | -0.17884 | 0.076244 | EUR | Stricture and stenosis of esophagus | digestive |
| 364.51 | -0.09707 | 0.123244 | -0.78762 | 0.430921 | -0.33862 | 0.144485 | EUR | Fuchs' dystrophy | sense organs |
| 733.4 | 0.072402 | 0.092036 | 0.786667 | 0.431477 | -0.10799 | 0.252789 | EUR | Aseptic necrosis of bone | musculoskeletal |
| 386.1 | 0.081975 | 0.104221 | 0.786546 | 0.431548 | -0.12229 | 0.286244 | EUR | Meniere's disease | sense organs |
| 627.1 | 0.050853 | 0.064781 | 0.784999 | 0.432454 | -0.07612 | 0.177823 | EUR | Postmenopausal bleeding | genitourinary |
| 31.1 | -0.87133 | 1.111019 | -0.78427 | 4.33E-01 | -3.04889 | 1.306223 | EUR | Leprosy | infectious diseases |
| 346.1 | 0.04449 | 0.056807 | 0.783183 | 0.43352 | -0.06685 | 0.15583 | EUR | Nonspecific abnormal findings on radiological and other examination of skull and head | neurological |
| 751 | 0.039204 | 0.050109 | 0.782384 | 0.433989 | -0.05901 | 0.137415 | EUR | Genitourinary congenital anomalies | congenital anomalies |
| 612.1 | 0.140195 | 0.179274 | 0.782015 | 0.434206 | -0.21118 | 0.491566 | EUR | Galactorrhea | genitourinary |
| 795.8 | -0.0682 | 0.087308 | -0.78118 | 0.434697 | -0.23932 | 0.102917 | EUR | Abnormal tumor markers | symptoms |
| 270.31 | -0.22251 | 0.284867 | -0.78111 | 0.434735 | -0.78084 | 0.335815 | EUR | Polyclonal hypergammaglobulinemia | endocrine/metabolic |
| 180.3 | 0.061341 | 0.07855 | 0.780915 | 0.434853 | -0.09261 | 0.215295 | EUR | Cervical intraepithelial neoplasia [CIN] [Cervical dysplasia] | neoplasms |
| 560.1 | -0.04738 | 0.060689 | -0.78075 | 0.434951 | -0.16633 | 0.071565 | EUR | Paralytic ileus | digestive |
| 521.2 | 0.747782 | 0.958985 | 0.779764 | 0.43553 | -1.13179 | 2.627358 | EUR | Dental abrasion, erosion and attrition | digestive |
| 287.32 | 0.049847 | 0.064076 | 0.777937 | 0.436606 | -0.07574 | 0.175435 | EUR | Secondary thrombocytopenia | hematopoietic |
| 727.6 | 0.057614 | 0.074132 | 0.777187 | 0.437048 | -0.08768 | 0.202911 | EUR | Rupture of tendon, nontraumatic | musculoskeletal |
| 442.2 | -0.17761 | 0.228858 | -0.77605 | 0.437717 | -0.62616 | 0.270947 | EUR | Aneurysm of iliac artery | circulatory system |
| 447.7 | 0.046715 | 0.060256 | 0.775282 | 0.438173 | -0.07138 | 0.164815 | EUR | Aortic ectasia | circulatory system |
| 790.8 | 0.065465 | 0.084624 | 0.773599 | 0.439168 | -0.10039 | 0.231325 | EUR | Elevated C-reactive protein (CRP) | symptoms |
| 556.1 | 0.052353 | 0.067754 | 0.772693 | 0.439704 | -0.08044 | 0.185149 | EUR | Ulceration of intestine | digestive |
| 965.2 | 0.420093 | 0.543776 | 0.772548 | 0.43979 | -0.64569 | 1.485874 | EUR | Antirheumatics causing adverse effects in therapeutic use | injuries & poisonings |
| 369.5 | -0.03059 | 0.039611 | -0.77231 | 0.439929 | -0.10823 | 0.047044 | EUR | Conjunctivitis, infectious | sense organs |
| 711.2 | -0.16619 | 0.215232 | -0.77214 | 0.440033 | -0.58804 | 0.255659 | EUR | Reiter's disease | musculoskeletal |
| 527.2 | 0.086465 | 0.11212 | 0.77118 | 0.4406 | -0.13329 | 0.306216 | EUR | Sialoadenitis | digestive |
| 754.2 | 0.065977 | 0.085578 | 0.770952 | 0.440735 | -0.10175 | 0.233706 | EUR | Spondylolisthesis, congenital | congenital anomalies |
| 191.11 | -0.06222 | 0.081034 | -0.76785 | 0.442578 | -0.22105 | 0.096602 | EUR | Cancer of brain | neoplasms |
| 695.21 | 0.249876 | 0.326149 | 0.76614 | 0.443593 | -0.38936 | 0.889116 | EUR | Dermatitis herpetiformis | dermatologic |
| 251 | 0.234788 | 0.306512 | 0.765999 | 0.443677 | -0.36596 | 0.83554 | EUR | Other disorders of pancreatic internal secretion | endocrine/metabolic |
| 381.1 | -0.03575 | 0.046683 | -0.76589 | 0.443745 | -0.12725 | 0.055743 | EUR | Otitis media | sense organs |
| 164 | -0.14297 | 0.186672 | -0.76588 | 0.443748 | -0.50884 | 0.222902 | EUR | Cancer of intrathoracic organs | neoplasms |
| 772.3 | 0.03441 | 0.044961 | 0.765334 | 0.444073 | -0.05371 | 0.122533 | EUR | Muscle weakness | symptoms |
| 364.2 | -0.12537 | 0.163826 | -0.76527 | 0.444112 | -0.44646 | 0.195722 | EUR | Corneal edema | sense organs |
| 605 | -0.0295 | 0.038555 | -0.76517 | 0.444171 | -0.10507 | 0.046066 | EUR | Erectile dysfunction [ED] | genitourinary |
| 345.12 | -0.06742 | 0.088332 | -0.76329 | 0.445289 | -0.24055 | 0.105705 | EUR | Partial epilepsy | neurological |
| 601 | -0.04567 | 0.059936 | -0.76194 | 0.446095 | -0.16314 | 0.071805 | EUR | Inflammatory diseases of prostate | genitourinary |
| 520.2 | 0.310549 | 0.407881 | 0.761371 | 0.446435 | -0.48888 | 1.109982 | EUR | Disturbances in tooth eruption | digestive |
| 711.1 | 0.101828 | 0.133992 | 0.75996 | 0.447278 | -0.16079 | 0.364447 | EUR | Pyogenic arthritis | musculoskeletal |
| 780 | -0.05953 | 0.078402 | -0.75935 | 0.447645 | -0.2132 | 0.094131 | EUR | Hypothermia/Chills | symptoms |
| 535.6 | 0.068557 | 0.090431 | 0.758119 | 0.44838 | -0.10868 | 0.245799 | EUR | Duodenitis | digestive |
| 303.31 | 0.068253 | 0.090268 | 0.756107 | 0.449585 | -0.10867 | 0.245176 | EUR | Gastrointestinal malfunction arising from mental factors | mental disorders |
| 270.21 | 0.218822 | 0.289465 | 0.755955 | 0.449676 | -0.34852 | 0.786163 | EUR | Disorders of urea cycle metabolism | endocrine/metabolic |
| 446.3 | 0.237463 | 0.31557 | 0.752489 | 0.451757 | -0.38104 | 0.855968 | EUR | Hypersensitivity angiitis | circulatory system |
| 296.1 | -0.03691 | 0.04931 | -0.74845 | 0.454192 | -0.13355 | 0.05974 | EUR | Bipolar | mental disorders |
| 250.15 | 0.243573 | 0.325649 | 0.747962 | 0.454483 | -0.39469 | 0.881833 | EUR | Diabetes type 1 with peripheral circulatory disorders | endocrine/metabolic |
| 803.21 | -0.11246 | 0.150809 | -0.74573 | 0.455828 | -0.40804 | 0.183117 | EUR | Colles' fracture | injuries & poisonings |
| 189.21 | -0.06523 | 0.08748 | -0.7457 | 0.455847 | -0.23669 | 0.106223 | EUR | Malignant neoplasm of bladder | neoplasms |
| 261.41 | 0.147934 | 0.198435 | 0.745504 | 0.455967 | -0.24099 | 0.53686 | EUR | Rickets or osteomalacia | endocrine/metabolic |

| phecode | Coef. | Std.Err. | z | p_value_z | [0.025 | 0.975] | GIA | phenotype | category |
|---|---|---|---|---|---|---|---|---|---|
| 395 | 0.022789 | 0.030627 | 0.744084 | 0.456825 | -0.03724 | 0.082816 | EUR | Heart valve disorders | circulatory system |
| 426.32 | 0.056399 | 0.075913 | 0.742942 | 0.457517 | -0.09239 | 0.205186 | EUR | Left bundle branch block | circulatory system |
| 500 | 0.070001 | 0.094239 | 0.742802 | 0.457602 | -0.1147 | 0.254706 | EUR | Lung disease due to external agents | respiratory |
| 426.22 | -0.17024 | 0.229621 | -0.74138 | 0.45846 | -0.62029 | 0.279811 | EUR | Mobitz II AV block | circulatory system |
| 742.9 | 0.043588 | 0.058795 | 0.741353 | 0.45848 | -0.07165 | 0.158823 | EUR | Other derangement of joint | musculoskeletal |
| 184.2 | 0.111787 | 0.150866 | 0.740968 | 0.458713 | -0.1839 | 0.407479 | EUR | Cancer of other female genital organs (excluding uterus and ovary) | neoplasms |
| 174.11 | 0.031521 | 0.042551 | 0.740801 | 0.458814 | -0.05188 | 0.114919 | EUR | Malignant neoplasm of female breast | neoplasms |
| 261.3 | 0.27048 | 0.367522 | 0.735956 | 0.461758 | -0.44985 | 0.990809 | EUR | Vitamin C deficiencies | endocrine/metabolic |
| 245 | -0.0324 | 0.044036 | -0.73581 | 0.461847 | -0.11871 | 0.053906 | EUR | Thyroiditis | endocrine/metabolic |
| 938.2 | -0.02987 | 0.040589 | -0.7358 | 0.461854 | -0.10942 | 0.049688 | EUR | Chronic dermatitis due to solar radiation | dermatologic |
| 291.8 | 0.035362 | 0.048104 | 0.735115 | 0.46227 | -0.05892 | 0.129643 | EUR | Alteration of consciousness | mental disorders |
| 385.5 | 0.174612 | 0.237689 | 0.734624 | 0.462569 | -0.29125 | 0.640473 | EUR | Tympanosclerosis and middle ear disease related to otitis media | sense organs |
| 290.16 | 0.096848 | 0.131883 | 0.734352 | 0.462734 | -0.16164 | 0.355333 | EUR | Vascular dementia | mental disorders |
| 470 | -0.0291 | 0.039634 | -0.73419 | 0.462831 | -0.10678 | 0.048582 | EUR | Septal Deviations/Turbinate Hypertrophy | respiratory |
| 599.1 | -0.03818 | 0.052075 | -0.73324 | 0.46341 | -0.14025 | 0.063881 | EUR | Urinary obstruction | genitourinary |
| 327.5 | 0.061741 | 0.084383 | 0.731679 | 0.464364 | -0.10365 | 0.227129 | EUR | Parasomnia | neurological |
| 458.2 | 0.054869 | 0.074992 | 0.731667 | 0.464372 | -0.09211 | 0.201851 | EUR | Iatrogenic hypotension | circulatory system |
| 250.11 | 0.156719 | 0.214626 | 0.730194 | 0.465271 | -0.26394 | 0.577378 | EUR | Type 1 diabetes with ketoacidosis | endocrine/metabolic |
| 320 | -0.07207 | 0.098784 | -0.7296 | 0.465636 | -0.26569 | 0.121541 | EUR | Meningitis | neurological |
| 758.1 | -0.08116 | 0.111375 | -0.72875 | 0.466157 | -0.29946 | 0.137127 | EUR | Chromosomal anomalies | congenital anomalies |
| 750.21 | -0.07965 | 0.109374 | -0.72828 | 0.466445 | -0.29402 | 0.134715 | EUR | Congenital anomalies of intestine | congenital anomalies |
| 952 | -0.1332 | 0.183031 | -0.72773 | 0.466778 | -0.49193 | 0.225536 | EUR | Spinal cord injury without evidence of spinal bone injury | injuries & poisonings |
| 800.3 | 0.062715 | 0.086552 | 0.724596 | 0.4687 | -0.10692 | 0.232355 | EUR | Fracture of tibia and fibula | injuries & poisonings |
| 578.9 | 0.039542 | 0.054682 | 0.723131 | 0.469599 | -0.06763 | 0.146717 | EUR | Hemorrhage of gastrointestinal tract | digestive |
| 735.3 | -0.0371 | 0.051321 | -0.72298 | 0.469692 | -0.13769 | 0.063484 | EUR | Hallux valgus (Bunion) | musculoskeletal |
| 647 | -0.07265 | 0.100564 | -0.72241 | 0.470045 | -0.26975 | 0.124454 | EUR | Infectious and parasitic complications affecting pregnancy | pregnancy complications |
| 357 | 0.020191 | 0.02801 | 0.720873 | 0.470988 | -0.03471 | 0.075089 | EUR | Inflammatory and toxic neuropathy | neurological |
| 313.3 | 0.118019 | 0.163835 | 0.720351 | 0.471309 | -0.20309 | 0.43913 | EUR | Autism | mental disorders |
| 292.11 | -0.06251 | 0.086822 | -0.71999 | 0.471533 | -0.23268 | 0.107657 | EUR | Aphasia | mental disorders |
| 441.1 | -0.14156 | 0.196725 | -0.71956 | 0.471796 | -0.52713 | 0.244019 | EUR | Acute vascular insufficiency of intestine | circulatory system |
| 276.1 | 0.018925 | 0.02631 | 0.719331 | 0.471937 | -0.03264 | 0.070491 | EUR | Electrolyte imbalance | endocrine/metabolic |
| 250.42 | 0.016442 | 0.022864 | 0.719141 | 0.472054 | -0.02837 | 0.061255 | EUR | Other abnormal glucose | endocrine/metabolic |
| 772.1 | 0.069955 | 0.097314 | 0.718856 | 0.47223 | -0.12078 | 0.260686 | EUR | Muscular wasting and disuse atrophy | symptoms |
| 800.1 | -0.05433 | 0.075866 | -0.71614 | 0.473905 | -0.20303 | 0.094364 | EUR | Fracture of neck of femur | injuries & poisonings |
| 870.3 | -0.04513 | 0.063033 | -0.71598 | 0.474001 | -0.16867 | 0.078412 | EUR | Other open wound of head and face | injuries & poisonings |
| 315.1 | -0.13178 | 0.184093 | -0.71581 | 0.474106 | -0.49259 | 0.22904 | EUR | Learning disorder | mental disorders |
| 284.1 | 0.03491 | 0.048887 | 0.714091 | 0.475171 | -0.06091 | 0.130726 | EUR | Pancytopenia | hematopoietic |
| 705.1 | 0.094999 | 0.133381 | 0.712242 | 0.476315 | -0.16642 | 0.356421 | EUR | Dyshidrosis | dermatologic |
| 603 | -0.05575 | 0.078345 | -0.71159 | 0.476721 | -0.2093 | 0.097804 | EUR | Other disorders of testis | genitourinary |
| 736 | 0.044132 | 0.062043 | 0.711305 | 0.476895 | -0.07747 | 0.165734 | EUR | Other acquired deformities of limbs | musculoskeletal |
| 370.31 | -0.11618 | 0.163454 | -0.7108 | 0.477207 | -0.43655 | 0.204181 | EUR | Keratoconjunctivitis sicca | sense organs |
| 290.11 | -0.07116 | 0.100134 | -0.71065 | 0.477301 | -0.26742 | 0.125098 | EUR | Alzheimer's disease | mental disorders |
| 198.1 | -0.02897 | 0.040772 | -0.71052 | 0.477384 | -0.10888 | 0.050943 | EUR | Secondary malignancy of lymph nodes | neoplasms |
| 741.6 | -0.19409 | 0.273536 | -0.70954 | 0.477987 | -0.73021 | 0.342035 | EUR | Villonodular synovitis | musculoskeletal |
| 41.2 | 0.042119 | 0.05939 | 0.70919 | 0.478206 | -0.07428 | 0.15852 | EUR | Streptococcus infection | infectious diseases |
| 767 | -0.10268 | 0.144892 | -0.70867 | 0.478531 | -0.38666 | 0.181303 | EUR | Cervicocranial/Cervicobrachial syndrome | symptoms |
| 516 | 0.050317 | 0.071182 | 0.706878 | 0.479642 | -0.0892 | 0.189831 | EUR | Abnormal sputum | respiratory |
| 687.1 | -0.01793 | 0.025388 | -0.70618 | 0.480073 | -0.06769 | 0.03183 | EUR | Rash and other nonspecific skin eruption | dermatologic |
| 735.1 | -0.05062 | 0.071708 | -0.70595 | 0.480217 | -0.19117 | 0.089923 | EUR | Flat foot | musculoskeletal |
| 562.2 | 0.042806 | 0.060728 | 0.70487 | 0.480891 | -0.07622 | 0.161831 | EUR | Diverticulitis | digestive |
| 705.3 | -0.13043 | 0.185067 | -0.70476 | 0.480958 | -0.49315 | 0.232296 | EUR | Hidradenitis | dermatologic |
| 241.2 | 0.028418 | 0.040587 | 0.700185 | 0.483811 | -0.05113 | 0.107967 | EUR | Nontoxic multinodular goiter | endocrine/metabolic |
| 604.2 | 0.411741 | 0.588164 | 0.700045 | 0.483899 | -0.74104 | 1.564521 | EUR | Vascular disorders of penis | genitourinary |
| 766 | 0.028136 | 0.0402 | 0.699909 | 0.483984 | -0.05065 | 0.106927 | EUR | Neuralgia, neuritis, and radiculitis NOS | symptoms |
| 204.2 | 0.078319 | 0.112 | 0.699272 | 0.484382 | -0.1412 | 0.297835 | EUR | Myeloid leukemia | neoplasms |
| 593 | -0.02289 | 0.032738 | -0.69927 | 0.484386 | -0.08706 | 0.041273 | EUR | Hematuria | genitourinary |
| 592.11 | 0.024828 | 0.035525 | 0.698896 | 0.484617 | -0.0448 | 0.094457 | EUR | Acute cystitis | genitourinary |
| 695.81 | -0.19654 | 0.282933 | -0.69467 | 0.487264 | -0.75108 | 0.357994 | EUR | Erythema nodosum | dermatologic |
| 452.8 | -0.10197 | 0.146977 | -0.69377 | 0.487826 | -0.39004 | 0.186101 | EUR | Postphlebitic syndrome | circulatory system |

| phecode | Coef. | Std.Err. | z | p_value_z | [0.025 | 0.975] | GIA | phenotype | category |
|---|---|---|---|---|---|---|---|---|---|
| 221 | -0.14008 | 0.202056 | -0.6933 | 0.488124 | -0.53611 | 0.255937 | EUR | Benign neoplasm of other female genital organs | neoplasms |
| 736.6 | 0.078973 | 0.113938 | 0.693125 | 0.488231 | -0.14434 | 0.302288 | EUR | Unequal leg length (acquired) | musculoskeletal |
| 371.2 | -0.03695 | 0.053445 | -0.69136 | 0.489338 | -0.1417 | 0.0678 | EUR | Conjunctivitis, noninfectious | sense organs |
| 598 | 0.029289 | 0.042391 | 0.690913 | 0.48962 | -0.0538 | 0.112374 | EUR | Abnormal findings on examination of urine | genitourinary |
| 530.6 | -0.14438 | 0.208998 | -0.69082 | 0.489678 | -0.55401 | 0.265248 | EUR | Diverticulum of esophagus, acquired | digestive |
| 250.21 | 0.111772 | 0.162008 | 0.689917 | 0.490246 | -0.20576 | 0.429302 | EUR | Type 2 diabetes with ketoacidosis | endocrine/metabolic |
| 281.1 | 0.062351 | 0.090418 | 0.68958 | 0.490459 | -0.11487 | 0.239567 | EUR | Megaloblastic anemia | hematopoietic |
| 531 | -0.02795 | 0.040582 | -0.6887 | 0.491011 | -0.10749 | 0.05159 | EUR | Peptic ulcer (excl. esophageal) | digestive |
| 642.1 | -0.07708 | 0.112024 | -0.68803 | 0.491434 | -0.29664 | 0.142488 | EUR | Preeclampsia and eclampsia | pregnancy complications |
| 348.8 | 0.04084 | 0.059472 | 0.686698 | 0.492273 | -0.07572 | 0.157404 | EUR | Encephalopathy, not elsewhere classified | neurological |
| 687.3 | -0.05292 | 0.077156 | -0.68594 | 0.492752 | -0.20415 | 0.098299 | EUR | Changes in skin texture | dermatologic |
| 592.21 | 0.191943 | 0.280105 | 0.685253 | 0.493184 | -0.35705 | 0.74094 | EUR | Urethral syndrome | genitourinary |
| 132 | 0.094979 | 0.13865 | 0.685029 | 0.493326 | -0.17677 | 0.366727 | EUR | Infestation (lice, mites) | infectious diseases |
| 729.7 | 0.172413 | 0.251912 | 0.684418 | 0.493711 | -0.32132 | 0.666151 | EUR | Nontraumatic compartment syndrome | musculoskeletal |
| 275.51 | -0.04042 | 0.059084 | -0.68405 | 0.493941 | -0.15622 | 0.075386 | EUR | Hypocalcemia | endocrine/metabolic |
| 425.8 | -0.13985 | 0.204786 | -0.68292 | 0.494658 | -0.54123 | 0.261521 | EUR | Other cardiomyopathy | circulatory system |
| 473.1 | 0.084646 | 0.123996 | 0.682646 | 0.494831 | -0.15838 | 0.327674 | EUR | Chronic laryngitis | respiratory |
| 327.6 | 0.065151 | 0.095469 | 0.682431 | 0.494967 | -0.12196 | 0.252265 | EUR | Circadian rhythm sleep disorder | neurological |
| 353.2 | -0.12767 | 0.18729 | -0.68165 | 0.495461 | -0.49475 | 0.239415 | EUR | Nerve root lesions | neurological |
| 602 | -0.06792 | 0.099663 | -0.68151 | 0.495552 | -0.26326 | 0.127416 | EUR | Other disorders of prostate | genitourinary |
| 710.12 | 0.097623 | 0.143402 | 0.68076 | 0.496023 | -0.18344 | 0.378686 | EUR | Chronic osteomyelitis | musculoskeletal |
| 614.53 | -0.13479 | 0.198065 | -0.68053 | 0.496168 | -0.52299 | 0.253411 | EUR | Cyst or abscess of Bartholin's gland | genitourinary |
| 371.33 | -0.09309 | 0.137809 | -0.67552 | 0.499346 | -0.36319 | 0.177008 | EUR | Noninfectious dermatoses of eyelid | sense organs |
| 942 | 0.06835 | 0.101245 | 0.6751 | 0.499612 | -0.13009 | 0.266786 | EUR | Infusion and transfusion reaction | injuries & poisonings |
| 41.4 | 0.044183 | 0.065544 | 0.6741 | 0.500248 | -0.08428 | 0.172647 | EUR | E. coli | infectious diseases |
| 620.1 | -0.09054 | 0.134433 | -0.67348 | 0.500645 | -0.35402 | 0.172947 | EUR | Dysplasia of cervix | genitourinary |
| 444.5 | -0.31787 | 0.471988 | -0.67346 | 0.500652 | -1.24295 | 0.607213 | EUR | Atheroembolism | circulatory system |
| 564.8 | 0.029103 | 0.04322 | 0.673361 | 0.500717 | -0.05561 | 0.113812 | EUR | Abnormal findings on exam of gastrointestinal tract/ abdominal area | digestive |
| 580.11 | 0.16195 | 0.240565 | 0.673204 | 0.500817 | -0.30955 | 0.633449 | EUR | Proliferative glomerulonephritis | genitourinary |
| 772.2 | 0.030505 | 0.04532 | 0.67311 | 0.500877 | -0.05832 | 0.119331 | EUR | Spasm of muscle | symptoms |
| 526.3 | -0.13895 | 0.206664 | -0.67237 | 0.50135 | -0.54401 | 0.2661 | EUR | Anomalies of jaw size/symmetry | digestive |
| 292.4 | 0.029073 | 0.04325 | 0.672217 | 0.501445 | -0.0557 | 0.113842 | EUR | Altered mental status | mental disorders |
| 153.3 | 0.04363 | 0.06497 | 0.671548 | 0.501871 | -0.08371 | 0.170968 | EUR | Malignant neoplasm of rectum, rectosigmoid junction, and anus | neoplasms |
| 276 | 0.016071 | 0.023954 | 0.6709 | 0.502284 | -0.03088 | 0.06302 | EUR | Disorders of fluid, electrolyte, and acid-base balance | endocrine/metabolic |
| 426.21 | -0.04958 | 0.07442 | -0.66621 | 0.505275 | -0.19544 | 0.096281 | EUR | First degree AV block | circulatory system |
| 270.38 | 0.0542 | 0.081435 | 0.665564 | 0.50569 | -0.10541 | 0.213811 | EUR | Other specified disorders of plasma protein metabolism | endocrine/metabolic |
| 963 | 0.026753 | 0.040248 | 0.664695 | 0.506246 | -0.05213 | 0.105638 | EUR | Poisoning by primarily systemic agents | injuries & poisonings |
| 550 | 0.016901 | 0.025477 | 0.663395 | 0.507078 | -0.03303 | 0.066835 | EUR | Abdominal hernia | digestive |
| 574.1 | 0.027763 | 0.04192 | 0.662283 | 0.50779 | -0.0544 | 0.109925 | EUR | Cholelithiasis | digestive |
| 726.2 | 0.03864 | 0.058513 | 0.660358 | 0.509024 | -0.07604 | 0.153324 | EUR | Synoviopathy | musculoskeletal |
| 364.4 | -0.07905 | 0.119822 | -0.6597 | 0.509443 | -0.31389 | 0.155799 | EUR | Corneal degenerations | sense organs |
| 292.12 | -0.09963 | 0.151305 | -0.65845 | 0.510248 | -0.39618 | 0.196926 | EUR | Symbolic dysfunction | mental disorders |
| 495.2 | 0.028523 | 0.04335 | 0.657962 | 0.510563 | -0.05644 | 0.113488 | EUR | Asthma with exacerbation | respiratory |
| 313.1 | 0.034861 | 0.052993 | 0.657841 | 0.51064 | -0.069 | 0.138726 | EUR | Attention deficit hyperactivity disorder | mental disorders |
| 963.1 | 0.026818 | 0.04082 | 0.656985 | 0.511191 | -0.05319 | 0.106825 | EUR | Antineoplastic and immunosuppressive drugs causing adverse effects | injuries & poisonings |
| 704.8 | -0.02429 | 0.036981 | -0.65689 | 0.511249 | -0.09677 | 0.048189 | EUR | Other specified diseases of hair and hair follicles | dermatologic |
| 771.2 | -0.03422 | 0.052163 | -0.65594 | 0.511863 | -0.13645 | 0.068022 | EUR | Cramp of limb | symptoms |
| 600 | -0.02424 | 0.036998 | -0.65508 | 0.512417 | -0.09675 | 0.048278 | EUR | Hyperplasia of prostate | genitourinary |
| 853 | -0.06832 | 0.104655 | -0.65285 | 0.513856 | -0.27344 | 0.136797 | EUR | Complication of colostomy or enterostomy | injuries & poisonings |
| 292.3 | 0.026145 | 0.040099 | 0.652007 | 0.514397 | -0.05245 | 0.104738 | EUR | Memory loss | mental disorders |
| 561 | 0.014103 | 0.02164 | 0.651693 | 0.514599 | -0.02831 | 0.056517 | EUR | Symptoms involving digestive system | digestive |
| 361.2 | -0.15583 | 0.239194 | -0.65147 | 0.514743 | -0.62464 | 0.312984 | EUR | Retinoschisis and retinal cysts | sense organs |
| 732 | 0.111497 | 0.171369 | 0.650625 | 0.515289 | -0.22438 | 0.447374 | EUR | Osteochondropathies | musculoskeletal |
| 187.1 | 0.189268 | 0.291475 | 0.649347 | 0.516114 | -0.38201 | 0.760548 | EUR | Malignant neoplasm of unspecified male genital organ | neoplasms |
| 368.4 | -0.0512 | 0.079236 | -0.64617 | 0.518167 | -0.2065 | 0.1041 | EUR | Visual field defects | sense organs |
| 960.3 | 0.680588 | 1.05399 | 0.645725 | 0.518457 | -1.38519 | 2.74637 | EUR | Poisoning by antifungal antibiotics | injuries & poisonings |
| 695.3 | -0.02858 | 0.044323 | -0.64485 | 0.519026 | -0.11545 | 0.05829 | EUR | Rosacea | dermatologic |
| 739 | 0.058914 | 0.091389 | 0.644649 | 0.519155 | -0.1202 | 0.238032 | EUR | Contracture of joint | musculoskeletal |
| 709 | 0.031691 | 0.049229 | 0.643746 | 0.51974 | -0.0648 | 0.128178 | EUR | Diffuse diseases of connective tissue | dermatologic |

| phecode | Coef. | Std.Err. | z | p_value_z | [0.025 | 0.975] | GIA | phenotype | category |
|---|---|---|---|---|---|---|---|---|---|
| 312 | 0.067641 | 0.105184 | 0.643071 | 0.520178 | -0.13852 | 0.273799 | EUR | Conduct disorders | mental disorders |
| 614.51 | -0.03924 | 0.061025 | -0.64304 | 0.520201 | -0.15885 | 0.080365 | EUR | Cervicitis and endocervicitis | genitourinary |
| 287 | -0.0205 | 0.031886 | -0.64299 | 0.520229 | -0.083 | 0.041993 | EUR | Purpura and other hemorrhagic conditions | hematopoietic |
| 840 | -0.01696 | 0.026426 | -0.64192 | 0.520926 | -0.06876 | 0.034831 | EUR | Sprains and strains | injuries & poisonings |
| 735.23 | 0.081547 | 0.127139 | 0.641405 | 0.521259 | -0.16764 | 0.330735 | EUR | Hallux rigidus | musculoskeletal |
| 703.1 | -0.0353 | 0.055086 | -0.64088 | 0.521598 | -0.14327 | 0.072663 | EUR | Ingrowing nail | dermatologic |
| 593.1 | 0.035172 | 0.0549 | 0.640664 | 0.521741 | -0.07243 | 0.142774 | EUR | Gross hematuria | genitourinary |
| 145.4 | 0.167444 | 0.261374 | 0.640632 | 0.521762 | -0.34484 | 0.679728 | EUR | Cancer of the gums | neoplasms |
| 589 | 0.045172 | 0.070588 | 0.639937 | 0.522213 | -0.09318 | 0.183521 | EUR | Abnormal results of function study of kidney | genitourinary |
| 1001 | -0.04392 | 0.068658 | -0.63975 | 0.522333 | -0.17849 | 0.090643 | EUR | Foreign body injury | other |
| 594.8 | -0.06684 | 0.104616 | -0.63891 | 0.522883 | -0.27188 | 0.138203 | EUR | Renal colic | genitourinary |
| 964 | 0.08914 | 0.139519 | 0.638908 | 0.522883 | -0.18431 | 0.362593 | EUR | Poisoning by agents primarily affecting blood constituents | injuries & poisonings |
| 609 | 0.07148 | 0.112574 | 0.634962 | 0.525453 | -0.14916 | 0.292122 | EUR | Male infertility and abnormal spermatozoa | genitourinary |
| 369 | -0.02354 | 0.037135 | -0.63391 | 0.526142 | -0.09632 | 0.049243 | EUR | Infection of the eye | sense organs |
| 283.1 | 0.124015 | 0.19567 | 0.633797 | 0.526214 | -0.25949 | 0.50752 | EUR | Autoimmune hemolytic anemias | hematopoietic |
| 386.3 | 0.079344 | 0.126167 | 0.62888 | 0.529428 | -0.16794 | 0.326627 | EUR | Labyrinthitis | sense organs |
| 442.1 | -0.03669 | 0.058357 | -0.62866 | 0.529574 | -0.15106 | 0.077691 | EUR | Aortic aneurysm | circulatory system |
| 578.8 | -0.02364 | 0.037682 | -0.62741 | 0.530392 | -0.0975 | 0.050214 | EUR | Hemorrhage of rectum and anus | digestive |
| 709.3 | -0.07146 | 0.114621 | -0.62344 | 0.532996 | -0.29611 | 0.153194 | EUR | Systemic sclerosis | dermatologic |
| 427.22 | 0.029276 | 0.04704 | 0.622363 | 0.533703 | -0.06292 | 0.121473 | EUR | Atrial flutter | circulatory system |
| 595 | -0.03021 | 0.048703 | -0.62031 | 0.535055 | -0.12567 | 0.065245 | EUR | Hydronephrosis | genitourinary |
| 201 | 0.074456 | 0.120631 | 0.617221 | 0.537089 | -0.16198 | 0.31089 | EUR | Hodgkin's disease | neoplasms |
| 204 | 0.040047 | 0.065301 | 0.613262 | 0.539703 | -0.08794 | 0.168034 | EUR | Leukemia | neoplasms |
| 614.31 | -0.15188 | 0.247792 | -0.61292 | 0.53993 | -0.63754 | 0.333787 | EUR | Acute inflammatory pelvic disease | genitourinary |
| 531.2 | -0.0344 | 0.056144 | -0.61279 | 0.540017 | -0.14444 | 0.075636 | EUR | Gastric ulcer | digestive |
| 426.8 | -0.06529 | 0.106842 | -0.61109 | 0.541142 | -0.2747 | 0.144116 | EUR | Other cardiac conduction disorders | circulatory system |
| 823 | -0.61171 | 1.002036 | -0.61047 | 0.541554 | -2.57566 | 1.352246 | EUR | Torus fracture | injuries & poisonings |
| 276.8 | 0.07985 | 0.130806 | 0.610445 | 0.541567 | -0.17652 | 0.336224 | EUR | Polydipsia | endocrine/metabolic |
| 245.2 | -0.02796 | 0.045885 | -0.60926 | 0.542352 | -0.11789 | 0.061977 | EUR | Chronic thyroiditis | endocrine/metabolic |
| 638 | -0.03424 | 0.056278 | -0.60835 | 0.542956 | -0.14454 | 0.076067 | EUR | Other high-risk pregnancy | pregnancy complications |
| 241.1 | 0.021407 | 0.035203 | 0.608099 | 0.543122 | -0.04759 | 0.090404 | EUR | Nontoxic uninodular goiter | endocrine/metabolic |
| 149.4 | -0.13081 | 0.215539 | -0.6069 | 0.543917 | -0.55326 | 0.291638 | EUR | Cancer of larynx | neoplasms |
| 208 | -0.01469 | 0.024233 | -0.60622 | 0.54437 | -0.06219 | 0.032806 | EUR | Benign neoplasm of colon | neoplasms |
| 258.1 | -0.10387 | 0.171447 | -0.60586 | 0.544605 | -0.4399 | 0.232156 | EUR | Postablative ovarian failure | endocrine/metabolic |
| 282.8 | 0.052731 | 0.087056 | 0.60572 | 0.544701 | -0.11789 | 0.223357 | EUR | Other hemoglobinopathies | hematopoietic |
| 696 | 0.028608 | 0.047338 | 0.60433 | 0.545624 | -0.06417 | 0.121389 | EUR | Psoriasis and related disorders | dermatologic |
| 282.5 | 0.297555 | 0.493354 | 0.603126 | 0.546425 | -0.6694 | 1.264511 | EUR | Sickle cell anemia | hematopoietic |
| 345.3 | -0.03052 | 0.050648 | -0.60255 | 0.546807 | -0.12979 | 0.068751 | EUR | Convulsions | neurological |
| 691.3 | -0.13829 | 0.230022 | -0.6012 | 0.547705 | -0.58913 | 0.312545 | EUR | Congenital pigmentary anomalies of skin | dermatologic |
| 79 | -0.01444 | 0.024042 | -0.60049 | 0.548178 | -0.06156 | 0.032685 | EUR | Viral infection | infectious diseases |
| 454.1 | 0.026506 | 0.044225 | 0.599337 | 0.548948 | -0.06017 | 0.113186 | EUR | Varicose veins of lower extremity | circulatory system |
| 528.41 | 0.236608 | 0.395227 | 0.598663 | 0.549398 | -0.53802 | 1.011238 | EUR | Cyst of the salivary gland | digestive |
| 274.11 | -0.03695 | 0.061856 | -0.59734 | 0.550282 | -0.15818 | 0.084287 | EUR | Gouty arthropathy | endocrine/metabolic |
| 202.23 | -0.16557 | 0.277499 | -0.59665 | 0.550741 | -0.70946 | 0.378318 | EUR | Lymphosarcoma | neoplasms |
| 759.1 | 0.206704 | 0.347322 | 0.595136 | 0.551753 | -0.47403 | 0.887442 | EUR | Anomalies of endocrine glands, congenital | congenital anomalies |
| 540.1 | 0.048706 | 0.081937 | 0.594435 | 0.552221 | -0.11189 | 0.2093 | EUR | Appendicitis | digestive |
| 345 | -0.02806 | 0.047286 | -0.59348 | 0.552861 | -0.12074 | 0.064616 | EUR | Epilepsy, recurrent seizures, convulsions | neurological |
| 732.1 | 0.110226 | 0.185998 | 0.592623 | 0.553433 | -0.25432 | 0.474775 | EUR | Juvenile osteochondrosis | musculoskeletal |
| 244.5 | -0.11081 | 0.188126 | -0.58902 | 0.555848 | -0.47953 | 0.257909 | EUR | Congenital hypothyroidism | endocrine/metabolic |
| 614.33 | -0.11418 | 0.194345 | -0.5875 | 0.556868 | -0.49509 | 0.266732 | EUR | Pelvic inflammatory disease, NOS | genitourinary |
| 382 | -0.02201 | 0.037509 | -0.58685 | 0.557304 | -0.09553 | 0.051504 | EUR | Otalgia | sense organs |
| 215 | 0.028723 | 0.048993 | 0.58627 | 0.557694 | -0.0673 | 0.124747 | EUR | Other benign neoplasm of connective and other soft tissue | neoplasms |
| 327.31 | 0.041388 | 0.070815 | 0.584451 | 0.558917 | -0.09741 | 0.180182 | EUR | Central/nonobstructive sleep apnea | neurological |
| 647.3 | -0.16448 | 0.282211 | -0.58283 | 0.560006 | -0.7176 | 0.388641 | EUR | Major puerperal infection | pregnancy complications |
| 204.12 | 0.077041 | 0.132649 | 0.580789 | 0.561383 | -0.18295 | 0.337027 | EUR | Lymphoid leukemia, chronic | neoplasms |
| 458.1 | 0.028648 | 0.049394 | 0.579987 | 0.561923 | -0.06816 | 0.125458 | EUR | Orthostatic hypotension | circulatory system |
| 430.1 | 0.082793 | 0.14291 | 0.579334 | 0.562364 | -0.19731 | 0.362891 | EUR | Subarachnoid hemorrhage | circulatory system |
| 244.3 | -0.30222 | 0.522338 | -0.57859 | 0.562866 | -1.32598 | 0.721544 | EUR | Iodine hypothyroidism | endocrine/metabolic |
| 723 | 0.030715 | 0.053344 | 0.57579 | 0.564757 | -0.07384 | 0.135266 | EUR | Other disorders of cervical region | musculoskeletal |

| phecode | Coef. | Std.Err. | z | p_value_z | [0.025 | 0.975] | GIA | phenotype | category |
|---|---|---|---|---|---|---|---|---|---|
| 386.21 | 0.179118 | 0.311381 | 0.575236 | 0.565132 | -0.43118 | 0.789414 | EUR | Central origin vertigo | sense organs |
| 429.2 | -0.02956 | 0.051519 | -0.57385 | 0.566069 | -0.13054 | 0.071411 | EUR | Abnormal function study of cardiovascular system | circulatory system |
| 656 | 0.041906 | 0.073093 | 0.573328 | 0.566423 | -0.10135 | 0.185165 | EUR | Other perinatal conditions of fetus or newborn | pregnancy complications |
| 972.6 | -0.1377 | 0.240359 | -0.57288 | 0.566726 | -0.60879 | 0.333398 | EUR | Antihypertensive agents causing adverse effects | injuries & poisonings |
| 598.9 | 0.024608 | 0.04296 | 0.572812 | 0.566772 | -0.05959 | 0.108807 | EUR | Other nonspecific findings on examination of urine | genitourinary |
| 459 | 0.016225 | 0.028449 | 0.57032 | 0.56846 | -0.03953 | 0.071985 | EUR | Other disorders of circulatory system | circulatory system |
| 159.2 | -0.07574 | 0.132864 | -0.5701 | 0.568613 | -0.33615 | 0.184663 | EUR | Malignant neoplasm of small intestine, including duodenum | neoplasms |
| 225 | 0.036472 | 0.064035 | 0.569569 | 0.56897 | -0.08903 | 0.161978 | EUR | Benign neoplasm of brain and other parts of nervous system | neoplasms |
| 726.1 | -0.01542 | 0.027166 | -0.56768 | 0.570254 | -0.06867 | 0.037823 | EUR | Enthesopathy | musculoskeletal |
| 313 | 0.02834 | 0.049966 | 0.567198 | 0.57058 | -0.06959 | 0.126271 | EUR | Pervasive developmental disorders | mental disorders |
| 202.24 | -0.13185 | 0.232455 | -0.56719 | 0.570588 | -0.58745 | 0.323759 | EUR | Large cell lymphoma | neoplasms |
| 360.2 | -0.07604 | 0.134376 | -0.56591 | 0.571457 | -0.33942 | 0.187328 | EUR | Progressive myopia | sense organs |
| 376 | 0.095523 | 0.169222 | 0.56448 | 0.572427 | -0.23615 | 0.427192 | EUR | Disorders of the orbit | sense organs |
| 599 | -0.01232 | 0.021846 | -0.56391 | 0.572818 | -0.05514 | 0.030498 | EUR | Other symptoms/disorders or the urinary system | genitourinary |
| 772.6 | -0.05548 | 0.098387 | -0.56387 | 0.572844 | -0.24831 | 0.137357 | EUR | Facial weakness | symptoms |
| 364 | -0.0377 | 0.066992 | -0.56277 | 0.57359 | -0.169 | 0.093601 | EUR | Corneal opacity and other disorders of cornea | sense organs |
| 972.2 | 0.08957 | 0.159798 | 0.560522 | 0.575123 | -0.22363 | 0.402768 | EUR | Antilipemic and antiarteriosclerotic drugs causing adverse effects in therapeutic use | injuries & poisonings |
| 558 | 0.018579 | 0.033147 | 0.560519 | 0.575126 | -0.04639 | 0.083546 | EUR | Noninfectious gastroenteritis | digestive |
| 523 | 0.045657 | 0.081549 | 0.559876 | 0.575564 | -0.11418 | 0.205491 | EUR | Gingival and periodontal diseases | digestive |
| 453 | 0.05483 | 0.098024 | 0.559354 | 0.57592 | -0.13729 | 0.246954 | EUR | Chronic venous hypertension | circulatory system |
| 304 | -0.01935 | 0.034587 | -0.55934 | 0.57593 | -0.08713 | 0.048443 | EUR | Adjustment reaction | mental disorders |
| 426.2 | -0.02903 | 0.052023 | -0.55804 | 0.576817 | -0.13099 | 0.072932 | EUR | Atrioventricular [AV] block | circulatory system |
| 427.7 | -0.01843 | 0.03322 | -0.55464 | 0.579143 | -0.08354 | 0.046685 | EUR | Tachycardia NOS | circulatory system |
| 110.2 | -0.0435 | 0.078468 | -0.55433 | 0.579354 | -0.19729 | 0.110297 | EUR | Dermatomycoses | infectious diseases |
| 695.8 | 0.029802 | 0.054019 | 0.551688 | 0.581162 | -0.07607 | 0.135677 | EUR | Other specified erythematous conditions | dermatologic |
| 8 | -0.02129 | 0.038674 | -0.55038 | 5.82E-01 | -0.09708 | 0.054514 | EUR | Intestinal infection | infectious diseases |
| 378.1 | 0.040563 | 0.07443 | 0.54498 | 0.585767 | -0.10532 | 0.186443 | EUR | Strabismus (not specified as paralytic) | sense organs |
| 696.41 | 0.027603 | 0.050744 | 0.543968 | 0.586463 | -0.07185 | 0.127061 | EUR | Psoriasis vulgaris | dermatologic |
| 427.9 | -0.01518 | 0.027912 | -0.54387 | 0.586533 | -0.06989 | 0.039526 | EUR | Palpitations | circulatory system |
| 960 | -0.01373 | 0.025313 | -0.54228 | 0.587629 | -0.06334 | 0.035886 | EUR | Poisoning by antibiotics | injuries & poisonings |
| 446.7 | -0.20679 | 0.382045 | -0.54128 | 0.588314 | -0.95559 | 0.542 | EUR | Takayasu's disease | circulatory system |
| 286.3 | -0.05377 | 0.09958 | -0.53998 | 0.589209 | -0.24894 | 0.141402 | EUR | Coagulation defects complicating pregnancy or postpartum | hematopoietic |
| 459.1 | 0.036162 | 0.067063 | 0.539217 | 0.589737 | -0.09528 | 0.167603 | EUR | Hemorrhage NOS | circulatory system |
| 425 | 0.024511 | 0.045474 | 0.539016 | 0.589876 | -0.06462 | 0.113638 | EUR | Cardiomyopathy | circulatory system |
| 378 | 0.030737 | 0.057165 | 0.53769 | 0.590791 | -0.0813 | 0.14278 | EUR | Strabismus and other disorders of binocular eye movements | sense organs |
| 687.4 | -0.01443 | 0.026866 | -0.53725 | 0.591097 | -0.06709 | 0.038222 | EUR | Disturbance of skin sensation | dermatologic |
| 771 | -0.01984 | 0.036944 | -0.53705 | 0.591232 | -0.09225 | 0.052569 | EUR | Musculoskeletal symptoms referable to limbs | symptoms |
| 292.2 | 0.04099 | 0.076345 | 0.536908 | 0.591331 | -0.10864 | 0.190623 | EUR | Mild cognitive impairment | mental disorders |
| 132.1 | 0.164871 | 0.307129 | 0.536814 | 0.591396 | -0.43709 | 0.766833 | EUR | Pediculosis and phthirus infestation | infectious diseases |
| 727.7 | -0.07911 | 0.147417 | -0.53664 | 0.591514 | -0.36804 | 0.209822 | EUR | Contracture of tendon (sheath) | musculoskeletal |
| 362.21 | 0.045025 | 0.083926 | 0.536485 | 0.591623 | -0.11947 | 0.209518 | EUR | Macular degeneration, dry | sense organs |
| 855 | 0.086735 | 0.161708 | 0.536365 | 0.591706 | -0.23021 | 0.403676 | EUR | Complication of nervous system device, implant, and graft | injuries & poisonings |
| 54 | 0.02014 | 0.037658 | 0.534807 | 0.592783 | -0.05367 | 0.093949 | EUR | Herpes simplex | infectious diseases |
| 131 | -0.18788 | 0.351904 | -0.53389 | 0.593417 | -0.8776 | 0.50184 | EUR | Protozoan infection | infectious diseases |
| 368.91 | 0.078025 | 0.146315 | 0.533272 | 0.593845 | -0.20875 | 0.364797 | EUR | Psychophysical visual disturbances | sense organs |
| 394 | 0.023988 | 0.045064 | 0.53231 | 0.594511 | -0.06434 | 0.112311 | EUR | Rheumatic disease of the heart valves | circulatory system |
| 601.4 | 0.09916 | 0.18636 | 0.53209 | 0.594664 | -0.2661 | 0.46442 | EUR | Balanoposthitis | genitourinary |
| 374.3 | 0.033502 | 0.06325 | 0.529682 | 0.596332 | -0.09047 | 0.15747 | EUR | Ptosis of eyelid | sense organs |
| 287.3 | -0.01828 | 0.034533 | -0.5294 | 0.596528 | -0.08596 | 0.049401 | EUR | Thrombocytopenia | hematopoietic |
| 701 | -0.01302 | 0.024652 | -0.52808 | 0.597445 | -0.06134 | 0.035299 | EUR | Other hypertrophic and atrophic conditions of skin | dermatologic |
| 264.9 | 0.078637 | 0.148997 | 0.527777 | 0.597654 | -0.21339 | 0.370666 | EUR | Lack of normal physiological development, unspecified | endocrine/metabolic |
| 447 | 0.022022 | 0.041792 | 0.526935 | 0.598239 | -0.05989 | 0.103932 | EUR | Other disorders of arteries and arterioles | circulatory system |
| 530.5 | -0.03288 | 0.062458 | -0.52639 | 0.59862 | -0.15529 | 0.089539 | EUR | Disorders of esophageal motility | digestive |
| 958 | -0.07335 | 0.139648 | -0.52526 | 0.599401 | -0.34706 | 0.200354 | EUR | Certain early complications of trauma or procedure | injuries & poisonings |
| 272.14 | 0.206479 | 0.393316 | 0.52497 | 0.599604 | -0.56441 | 0.977364 | EUR | Hyperchylomicronemia | endocrine/metabolic |
| 195.3 | 0.039134 | 0.074908 | 0.522432 | 0.601369 | -0.10768 | 0.185952 | EUR | Malignant neoplasm of head, face, and neck | neoplasms |
| 530.7 | 0.134056 | 0.256806 | 0.522011 | 0.601663 | -0.36928 | 0.637387 | EUR | Gastroesophageal laceration-hemorrhage syndrome | digestive |
| 257.1 | -0.02607 | 0.05003 | -0.52118 | 0.602239 | -0.12413 | 0.071982 | EUR | Testicular hypofunction | endocrine/metabolic |
| 728.7 | 0.019837 | 0.038094 | 0.520727 | 0.602557 | -0.05483 | 0.0945 | EUR | Fasciitis | musculoskeletal |

| phecode | Coef. | Std.Err. | z | p_value_z | [0.025 | 0.975] | GIA | phenotype | category |
|---|---|---|---|---|---|---|---|---|---|
| 204.1 | 0.05447 | 0.104604 | 0.520724 | 0.602559 | -0.15055 | 0.25949 | EUR | Lymphoid leukemia | neoplasms |
| 626.2 | 0.038361 | 0.073824 | 0.519629 | 0.603322 | -0.10633 | 0.183054 | EUR | Dysmenorrhea | genitourinary |
| 755.4 | 0.144287 | 0.277832 | 0.519333 | 0.603529 | -0.40025 | 0.688829 | EUR | Congenital anomalies of upper limb, including shoulder girdle | congenital anomalies |
| 709.7 | 0.036208 | 0.069808 | 0.518675 | 0.603987 | -0.10061 | 0.173029 | EUR | Unspecified diffuse connective tissue disease | dermatologic |
| 639 | -0.4032 | 0.777776 | -0.5184 | 0.604176 | -1.92762 | 1.12121 | EUR | Complications following abortion or ectopic and molar pregnancies | pregnancy complications |
| 612.2 | -0.0403 | 0.077849 | -0.51767 | 0.604688 | -0.19288 | 0.112281 | EUR | Hypertrophy of breast (Gynecomastia) | genitourinary |
| 625.1 | -0.03363 | 0.065088 | -0.51675 | 0.605329 | -0.16121 | 0.093936 | EUR | Dyspareunia | genitourinary |
| 315.3 | 0.094995 | 0.183845 | 0.516712 | 0.605357 | -0.26533 | 0.455324 | EUR | Mental retardation | mental disorders |
| 627.21 | -0.10674 | 0.206807 | -0.51615 | 0.605752 | -0.51208 | 0.298591 | EUR | Symptomatic artificial menopause | genitourinary |
| 227.2 | 0.043537 | 0.084641 | 0.514368 | 0.606995 | -0.12236 | 0.20943 | EUR | Benign neoplasm of parathyroid gland | neoplasms |
| 750.2 | 0.044063 | 0.086327 | 0.510419 | 0.609758 | -0.12513 | 0.213261 | EUR | Lower gastrointestinal congenital anomalies | congenital anomalies |
| 191 | -0.03308 | 0.064932 | -0.50951 | 0.610394 | -0.16035 | 0.094181 | EUR | Manlignant and unknown neoplasms of brain and nervous system | neoplasms |
| 790.6 | 0.01197 | 0.0236 | 0.507224 | 0.611998 | -0.03428 | 0.058225 | EUR | Other abnormal blood chemistry | symptoms |
| 347 | -0.0982 | 0.193835 | -0.50661 | 0.612428 | -0.47811 | 0.281711 | EUR | Cataplexy and narcolepsy | neurological |
| 531.5 | -0.09298 | 0.183809 | -0.50584 | 0.612968 | -0.45324 | 0.267281 | EUR | Gastrojejunal ulcer | digestive |
| 255.1 | -0.05561 | 0.110007 | -0.50549 | 0.613215 | -0.27122 | 0.160002 | EUR | Adrenal hyperfunction | endocrine/metabolic |
| 218.2 | 0.161363 | 0.319459 | 0.505115 | 0.613478 | -0.46476 | 0.787492 | EUR | Other benign neoplasm of uterus | neoplasms |
| 586.1 | -0.07505 | 0.148931 | -0.50396 | 0.614292 | -0.36695 | 0.216844 | EUR | Anatomical abnormatilies of kidney and ureters | genitourinary |
| 840.1 | 0.045408 | 0.090368 | 0.502476 | 0.615333 | -0.13171 | 0.222526 | EUR | Muscle/tendon sprain | injuries & poisonings |
| 528.7 | 0.088059 | 0.175545 | 0.501634 | 0.615925 | -0.256 | 0.432121 | EUR | Sialolithiasis | digestive |
| 716.2 | 0.112968 | 0.225286 | 0.501443 | 0.616059 | -0.32858 | 0.554521 | EUR | Unspecified monoarthritis | musculoskeletal |
| 474 | -0.03431 | 0.068561 | -0.50043 | 0.616775 | -0.16869 | 0.100067 | EUR | Acute and chronic tonsillitis | respiratory |
| 621 | -0.06493 | 0.129944 | -0.49964 | 0.617326 | -0.31961 | 0.18976 | EUR | Endometrial hyperplasia | genitourinary |
| 368.1 | 0.063041 | 0.126571 | 0.498065 | 0.618438 | -0.18503 | 0.311116 | EUR | Amblyopia | sense organs |
| 250.23 | 0.034533 | 0.069614 | 0.496068 | 0.619847 | -0.10191 | 0.170973 | EUR | Type 2 diabetes with ophthalmic manifestations | endocrine/metabolic |
| 285.3 | 0.208802 | 0.422069 | 0.49471 | 0.620805 | -0.61844 | 1.036043 | EUR | Sideroblastic anemia | hematopoietic |
| 386 | 0.021021 | 0.042505 | 0.494566 | 0.620906 | -0.06229 | 0.104329 | EUR | Vertiginous syndromes and other disorders of vestibular system | sense organs |
| 257 | -0.02466 | 0.049973 | -0.4934 | 0.621733 | -0.1226 | 0.073289 | EUR | Testicular dysfunction | endocrine/metabolic |
| 145.5 | 0.126111 | 0.256241 | 0.492156 | 0.622609 | -0.37611 | 0.628334 | EUR | Cancer of the mouth floor | neoplasms |
| 198.3 | -0.03466 | 0.07045 | -0.49205 | 0.622685 | -0.17275 | 0.103415 | EUR | Secondary malignant neoplasm of digestive systems | neoplasms |
| 626.13 | -0.02302 | 0.046795 | -0.492 | 0.622719 | -0.11474 | 0.068693 | EUR | Irregular menstrual cycle | genitourinary |
| 625 | 0.017719 | 0.036108 | 0.490715 | 0.623628 | -0.05305 | 0.088489 | EUR | Pain and other symptoms associated with female genital organs | genitourinary |
| 573.2 | 0.047872 | 0.097581 | 0.490583 | 0.623721 | -0.14338 | 0.239128 | EUR | Liver replaced by transplant | digestive |
| 529.1 | 0.070448 | 0.14372 | 0.490176 | 0.624009 | -0.21124 | 0.352133 | EUR | Glossitis | digestive |
| 626.21 | -0.17673 | 0.361825 | -0.48843 | 0.625245 | -0.88589 | 0.532437 | EUR | Mittelschmerz | genitourinary |
| 798 | -0.01056 | 0.021638 | -0.48789 | 0.625625 | -0.05297 | 0.031853 | EUR | Malaise and fatigue | symptoms |
| 430.2 | 0.053038 | 0.108808 | 0.487448 | 0.625941 | -0.16022 | 0.266299 | EUR | Intracerebral hemorrhage | circulatory system |
| 716.1 | 0.055154 | 0.113913 | 0.484176 | 0.628261 | -0.16811 | 0.278419 | EUR | Unspecified polyarthropathy or polyarthritis | musculoskeletal |
| 752.11 | -0.09925 | 0.204994 | -0.48414 | 0.628283 | -0.50103 | 0.302533 | EUR | Spina bifida | congenital anomalies |
| 724.2 | 0.021001 | 0.043439 | 0.483454 | 0.628773 | -0.06414 | 0.106139 | EUR | Disorders of coccyx | musculoskeletal |
| 574.2 | -0.04019 | 0.083334 | -0.48232 | 0.629581 | -0.20352 | 0.123138 | EUR | Calculus of bile duct | digestive |
| 433.8 | 0.037932 | 0.078727 | 0.48182 | 0.629934 | -0.11637 | 0.192234 | EUR | Late effects of cerebrovascular disease | circulatory system |
| 751.22 | -0.06149 | 0.12801 | -0.48035 | 0.630975 | -0.31239 | 0.189405 | EUR | Other specified congenital anomalies of kidney | congenital anomalies |
| 830 | 0.018373 | 0.038521 | 0.476971 | 0.633383 | -0.05713 | 0.093873 | EUR | Dislocation | injuries & poisonings |
| 705.8 | 0.024392 | 0.051186 | 0.476532 | 0.633696 | -0.07593 | 0.124715 | EUR | Hyperhidrosis | dermatologic |
| 395.1 | 0.018084 | 0.038024 | 0.475596 | 0.634362 | -0.05644 | 0.092611 | EUR | Nonrheumatic mitral valve disorders | circulatory system |
| 608 | 0.025278 | 0.053335 | 0.473952 | 0.635534 | -0.07926 | 0.129814 | EUR | Other disorders of male genital organs | genitourinary |
| 202.22 | -0.04939 | 0.10457 | -0.4723 | 0.636711 | -0.25434 | 0.155565 | EUR | Reticulosarcoma | neoplasms |
| 288.1 | 0.014615 | 0.030946 | 0.472279 | 0.636728 | -0.04604 | 0.075268 | EUR | Decreased white blood cell count | hematopoietic |
| 333.8 | -0.08267 | 0.175379 | -0.47136 | 0.637383 | -0.4264 | 0.26107 | EUR | Other degenerative diseases of the basal ganglia | neurological |
| 172.1 | 0.023522 | 0.049984 | 0.470594 | 0.63793 | -0.07444 | 0.121488 | EUR | Melanomas of skin, dx or hx | neoplasms |
| 750.1 | -0.03018 | 0.064918 | -0.46495 | 0.641964 | -0.15742 | 0.097053 | EUR | Upper gastrointestinal congenital anomalies | congenital anomalies |
| 1002 | -0.01618 | 0.034842 | -0.46434 | 0.642403 | -0.08447 | 0.05211 | EUR | Symptoms concerning nutrition, metabolism, and development | other |
| 427.42 | 0.047362 | 0.102116 | 0.463806 | 0.642787 | -0.15278 | 0.247505 | EUR | Cardiac arrest | circulatory system |
| 686.2 | 0.036637 | 0.079255 | 0.462263 | 0.643893 | -0.1187 | 0.191974 | EUR | Impetigo | dermatologic |
| 411.1 | 0.034727 | 0.075232 | 0.461603 | 0.644366 | -0.11272 | 0.18218 | EUR | Unstable angina (intermediate coronary syndrome) | circulatory system |
| 550.5 | 0.022501 | 0.048756 | 0.461504 | 0.644437 | -0.07306 | 0.118061 | EUR | Ventral hernia | digestive |
| 433.11 | -0.04452 | 0.096483 | -0.4614 | 0.644514 | -0.23362 | 0.144587 | EUR | Occlusion of cerebral arteries, with cerebral infarction | circulatory system |
| 286.4 | 0.044093 | 0.095602 | 0.461211 | 0.644647 | -0.14328 | 0.231469 | EUR | Acquired coagulation factor deficiency | hematopoietic |

| phecode | Coef. | Std.Err. | z | p_value_z | [0.025 | 0.975] | GIA | phenotype | category |
|---|---|---|---|---|---|---|---|---|---|
| 871.4 | 0.059253 | 0.128569 | 0.460862 | 0.644898 | -0.19274 | 0.311243 | EUR | Open wound of toe(s) | injuries & poisonings |
| 80 | 0.031835 | 0.06925 | 0.459715 | 0.645721 | -0.10389 | 0.167563 | EUR | Postoperative infection | infectious diseases |
| 979 | 0.015758 | 0.03432 | 0.459155 | 0.646123 | -0.05151 | 0.083025 | EUR | Adverse drug events and drug allergies | injuries & poisonings |
| 686 | -0.01643 | 0.035807 | -0.45888 | 0.646321 | -0.08661 | 0.053749 | EUR | Other local infections of skin and subcutaneous tissue | dermatologic |
| 907 | 0.051536 | 0.112701 | 0.457277 | 0.647472 | -0.16935 | 0.272426 | EUR | Injuries to the nervous system | injuries & poisonings |
| 727.5 | 0.031405 | 0.069034 | 0.454925 | 0.649163 | -0.1039 | 0.166708 | EUR | Rupture of synovium | musculoskeletal |
| 565.1 | 0.022003 | 0.048372 | 0.454879 | 0.649196 | -0.0728 | 0.116811 | EUR | Anal and rectal polyp | digestive |
| 259.4 | 0.16976 | 0.373685 | 0.454287 | 0.649623 | -0.56265 | 0.90217 | EUR | Precocious sexual development and puberty NEC | endocrine/metabolic |
| 747.13 | 0.039779 | 0.088057 | 0.451745 | 0.651453 | -0.13281 | 0.212368 | EUR | Congenital anomalies of great vessels | congenital anomalies |
| 291 | 0.018749 | 0.041874 | 0.447747 | 0.654336 | -0.06332 | 0.100822 | EUR | Other specified nonpsychotic and/or transient mental disorders | mental disorders |
| 290.13 | 0.085926 | 0.192274 | 0.446896 | 0.65495 | -0.29092 | 0.462776 | EUR | Senile dementia | mental disorders |
| 568 | 0.019223 | 0.043195 | 0.445032 | 0.656297 | -0.06544 | 0.103883 | EUR | Other disorders of peritoneum | digestive |
| 444 | -0.03606 | 0.081429 | -0.44289 | 0.657847 | -0.19566 | 0.123535 | EUR | Arterial embolism and thrombosis | circulatory system |
| 804 | 0.025632 | 0.058056 | 0.441517 | 0.658839 | -0.08815 | 0.139419 | EUR | Fracture of hand or wrist | injuries & poisonings |
| 378.2 | -0.06091 | 0.138176 | -0.44082 | 0.659341 | -0.33173 | 0.209908 | EUR | Nystagmus and other irregular eye movements | sense organs |
| 603.1 | 0.04342 | 0.098948 | 0.438818 | 0.660794 | -0.15051 | 0.237354 | EUR | Hydrocele | genitourinary |
| 990 | 0.015033 | 0.034266 | 0.438731 | 0.660857 | -0.05213 | 0.082193 | EUR | Effects radiation NOS | injuries & poisonings |
| 133 | 0.100756 | 0.229801 | 0.438447 | 0.661062 | -0.34965 | 0.551158 | EUR | Arthropod-borne diseases | infectious diseases |
| 263 | -0.01995 | 0.045504 | -0.43838 | 0.661112 | -0.10913 | 0.069238 | EUR | Other nutritional deficiency | endocrine/metabolic |
| 289.4 | 0.013753 | 0.031381 | 0.438256 | 0.661201 | -0.04775 | 0.075258 | EUR | Lymphadenitis | hematopoietic |
| 736.5 | 0.189703 | 0.434306 | 0.436795 | 0.66226 | -0.66152 | 1.040927 | EUR | Acquired deformities of knee | musculoskeletal |
| 962.3 | -0.07485 | 0.171807 | -0.43564 | 0.663101 | -0.41158 | 0.26189 | EUR | Hormones and synthetic substitutes causing adverse effects in therapeutic use | injuries & poisonings |
| 597.1 | -0.05095 | 0.117305 | -0.43434 | 0.664042 | -0.28086 | 0.178964 | EUR | Urethral stricture (not specified as infectious) | genitourinary |
| 227 | 0.02454 | 0.056626 | 0.43336 | 0.664753 | -0.08645 | 0.135525 | EUR | Benign neoplasm of other endocrine glands and related structures | neoplasms |
| 763 | 0.01319 | 0.030484 | 0.432684 | 0.665244 | -0.04656 | 0.072937 | EUR | Thoracic or lumbosacral neuritis or radiculitis, unspecified | symptoms |
| 275.3 | 0.022378 | 0.05177 | 0.432258 | 0.665554 | -0.07909 | 0.123845 | EUR | Disorders of magnesium metabolism | endocrine/metabolic |
| 292.1 | -0.02317 | 0.05378 | -0.43092 | 0.66653 | -0.12858 | 0.082232 | EUR | Aphasia/speech disturbance | mental disorders |
| 727 | -0.01087 | 0.025237 | -0.43085 | 0.666576 | -0.06034 | 0.03859 | EUR | Other disorders of synovium, tendon, and bursa | musculoskeletal |
| 145.2 | 0.045015 | 0.104704 | 0.429926 | 0.66725 | -0.1602 | 0.250231 | EUR | Cancer of tongue | neoplasms |
| 599.8 | -0.01802 | 0.042167 | -0.42738 | 0.669104 | -0.10067 | 0.064625 | EUR | Other symptoms involving urinary system | genitourinary |
| 185 | 0.019655 | 0.046412 | 0.423504 | 0.671927 | -0.07131 | 0.11062 | EUR | Cancer of prostate | neoplasms |
| 1000 | -0.04405 | 0.10404 | -0.42339 | 0.672014 | -0.24796 | 0.159866 | EUR | Burns | other |
| 572 | 0.019274 | 0.045958 | 0.419388 | 0.674933 | -0.0708 | 0.10935 | EUR | Ascites (non malignant) | digestive |
| 681.7 | -0.02582 | 0.061582 | -0.41925 | 0.675035 | -0.14652 | 0.09488 | EUR | Cellulitis and abscess of trunk | dermatologic |
| 726 | 0.009758 | 0.023397 | 0.417058 | 0.676636 | -0.0361 | 0.055616 | EUR | Peripheral enthesopathies and allied syndromes | musculoskeletal |
| 333.4 | -0.03706 | 0.089167 | -0.41567 | 0.677651 | -0.21183 | 0.1377 | EUR | Torsion dystonia | neurological |
| 380.1 | -0.02096 | 0.050476 | -0.41523 | 0.677975 | -0.11989 | 0.077972 | EUR | Otitis externa | sense organs |
| 658 | 0.068743 | 0.166159 | 0.413717 | 0.679082 | -0.25692 | 0.394408 | EUR | Maternal complication of pregnancy affecting fetus or newborn | pregnancy complications |
| 729.1 | -0.15646 | 0.379098 | -0.41272 | 0.679811 | -0.89948 | 0.586557 | EUR | Rheumatism, unspecified and fibrositis | musculoskeletal |
| 602.3 | -0.09239 | 0.223915 | -0.41261 | 0.679893 | -0.53125 | 0.346476 | EUR | Dysplasia of prostate | genitourinary |
| 278.3 | 0.056741 | 0.137573 | 0.412441 | 0.680017 | -0.2129 | 0.326379 | EUR | Localized adiposity | endocrine/metabolic |
| 519.8 | -0.0181 | 0.044051 | -0.41085 | 0.681186 | -0.10444 | 0.068241 | EUR | Other diseases of respiratory system, NEC | respiratory |
| 276.13 | 0.015863 | 0.038624 | 0.410706 | 0.681288 | -0.05984 | 0.091565 | EUR | Hyperpotassemia | endocrine/metabolic |
| 301 | 0.051315 | 0.125187 | 0.409905 | 0.681876 | -0.19405 | 0.296677 | EUR | Personality disorders | mental disorders |
| 394.3 | -0.06732 | 0.164284 | -0.40979 | 0.681956 | -0.38931 | 0.254668 | EUR | Aortic valve disease | circulatory system |
| 286.9 | 0.023923 | 0.058391 | 0.409707 | 0.682021 | -0.09052 | 0.138367 | EUR | Abnormal coagulation profile | hematopoietic |
| 550.3 | -0.08484 | 0.207728 | -0.40844 | 0.682954 | -0.49198 | 0.322296 | EUR | Femoral hernia | digestive |
| 246 | -0.01424 | 0.035024 | -0.4067 | 0.684229 | -0.08289 | 0.054402 | EUR | Other disorders of thyroid | endocrine/metabolic |
| 580.13 | 0.104819 | 0.257748 | 0.406671 | 0.68425 | -0.40036 | 0.609996 | EUR | Acute glomerulonephritis, NOS | genitourinary |
| 592.1 | 0.012926 | 0.031853 | 0.405806 | 0.684885 | -0.0495 | 0.075357 | EUR | Cystitis | genitourinary |
| 626.1 | 0.015126 | 0.037356 | 0.404902 | 0.685549 | -0.05809 | 0.088342 | EUR | Irregular menstrual cycle/bleeding | genitourinary |
| 915 | 0.021237 | 0.05269 | 0.403062 | 0.686903 | -0.08203 | 0.124507 | EUR | Superficial injury without mention of infection | injuries & poisonings |
| 938 | -0.01497 | 0.037256 | -0.40194 | 0.687726 | -0.08799 | 0.058045 | EUR | Dermatitis due to solar radiation | dermatologic |
| 212 | -0.05537 | 0.138061 | -0.40107 | 0.688369 | -0.32597 | 0.215223 | EUR | Benign neoplasm of respiratory and intrathoracic organs | neoplasms |
| 386.9 | -0.01045 | 0.026283 | -0.39748 | 0.69101 | -0.06196 | 0.041067 | EUR | Dizziness and giddiness (Light-headedness and vertigo) | sense organs |
| 368.3 | 0.063907 | 0.161305 | 0.396189 | 0.691966 | -0.25225 | 0.38006 | EUR | Anisometropia | sense organs |
| 720.1 | 0.015592 | 0.039474 | 0.394993 | 0.692848 | -0.06178 | 0.09296 | EUR | Spinal stenosis of lumbar region | musculoskeletal |
| 38.2 | -0.04085 | 0.103452 | -0.39489 | 6.93E-01 | -0.24361 | 0.161909 | EUR | Gram positive septicemia | infectious diseases |
| 367.4 | -0.01838 | 0.046726 | -0.39343 | 0.694001 | -0.10996 | 0.073198 | EUR | Presbyopia | sense organs |

| phecode | Coef. | Std.Err. | z | p_value_z | [0.025 | 0.975] | GIA | phenotype | category |
|---|---|---|---|---|---|---|---|---|---|
| 241 | 0.01233 | 0.031413 | 0.392508 | 0.694683 | -0.04924 | 0.073898 | EUR | Nontoxic nodular goiter | endocrine/metabolic |
| 747.11 | 0.022746 | 0.05802 | 0.392045 | 0.695025 | -0.09097 | 0.136464 | EUR | Cardiac shunt/ heart septal defect | congenital anomalies |
| 691.1 | 0.171139 | 0.437531 | 0.391146 | 0.695689 | -0.68641 | 1.028683 | EUR | Ichthyosis congenita | dermatologic |
| 446.5 | -0.06682 | 0.171029 | -0.3907 | 0.69602 | -0.40203 | 0.268389 | EUR | Giant cell arteritis | circulatory system |
| 172.2 | -0.01072 | 0.027452 | -0.39051 | 0.696162 | -0.06452 | 0.043084 | EUR | Other non-epithelial cancer of skin | neoplasms |
| 540 | 0.029101 | 0.074546 | 0.39038 | 0.696255 | -0.11701 | 0.175209 | EUR | Appendiceal conditions | digestive |
| 668 | -0.14049 | 0.360389 | -0.38983 | 0.696663 | -0.84684 | 0.565859 | EUR | Complications of the administration of anesthetic or other sedation in labor and delivery | pregnancy complications |
| 649.1 | -0.03879 | 0.099735 | -0.38892 | 0.697334 | -0.23427 | 0.156687 | EUR | Diabetes or abnormal glucose tolerance complicating pregnancy | pregnancy complications |
| 253.11 | -0.0949 | 0.245582 | -0.38643 | 0.699176 | -0.57623 | 0.38643 | EUR | Acromegaly and gigantism | endocrine/metabolic |
| 646 | -0.02069 | 0.053839 | -0.38435 | 0.700718 | -0.12621 | 0.084829 | EUR | Other complications of pregnancy NEC | pregnancy complications |
| 809 | 0.008518 | 0.02238 | 0.380617 | 0.703487 | -0.03535 | 0.052381 | EUR | Fracture of unspecified bones | injuries & poisonings |
| 707.1 | 0.032275 | 0.084828 | 0.380476 | 0.703592 | -0.13399 | 0.198536 | EUR | Decubitus ulcer | dermatologic |
| 593.2 | -0.01703 | 0.044755 | -0.38046 | 0.703608 | -0.10475 | 0.070691 | EUR | Microscopic hematuria | genitourinary |
| 840.2 | 0.021584 | 0.056833 | 0.379772 | 0.704115 | -0.08981 | 0.132975 | EUR | Rotator cuff (capsule) sprain | injuries & poisonings |
| 530.9 | -0.02139 | 0.056478 | -0.37878 | 0.704852 | -0.13209 | 0.089303 | EUR | Heartburn | digestive |
| 250.41 | -0.01415 | 0.037369 | -0.3787 | 0.704908 | -0.08739 | 0.059089 | EUR | Impaired fasting glucose | endocrine/metabolic |
| 740.2 | -0.01813 | 0.047884 | -0.37856 | 0.705016 | -0.11198 | 0.075725 | EUR | Osteoarthrosis, generalized | musculoskeletal |
| 442.8 | -0.03993 | 0.105597 | -0.37812 | 0.70534 | -0.24689 | 0.167038 | EUR | Aneurysm of other specified artery | circulatory system |
| 369.2 | 0.033898 | 0.089671 | 0.378025 | 0.705412 | -0.14185 | 0.209649 | EUR | Eye infection, viral | sense organs |
| 264.2 | -0.07242 | 0.192101 | -0.37698 | 0.706188 | -0.44893 | 0.304092 | EUR | Failure to thrive (childhood) | endocrine/metabolic |
| 614.1 | -0.04886 | 0.13074 | -0.37373 | 0.708602 | -0.30511 | 0.207383 | EUR | Pelvic peritoneal adhesions, female (postoperative) (postinfection) | genitourinary |
| 871.1 | 0.032157 | 0.086644 | 0.371138 | 0.710535 | -0.13766 | 0.201976 | EUR | Open wound of hand except finger(s) | injuries & poisonings |
| 264 | 0.044348 | 0.120578 | 0.367795 | 0.713026 | -0.19198 | 0.280676 | EUR | Lack of normal physiological development | endocrine/metabolic |
| 473.3 | 0.026199 | 0.071864 | 0.364563 | 0.715438 | -0.11465 | 0.16705 | EUR | Paralysis/spasm of vocal cords or larynx | respiratory |
| 931 | 0.057246 | 0.157036 | 0.364539 | 0.715455 | -0.25054 | 0.365032 | EUR | Contact dermatitis and other eczema due to plants [except food] | dermatologic |
| 531.3 | -0.03494 | 0.09596 | -0.36412 | 0.715766 | -0.22302 | 0.153136 | EUR | Duodenal ulcer | digestive |
| 367.8 | 0.029717 | 0.081628 | 0.364058 | 0.715815 | -0.13027 | 0.189706 | EUR | Hypermetropia | sense organs |
| 427.3 | -0.01043 | 0.028842 | -0.36147 | 0.717748 | -0.06696 | 0.046104 | EUR | Other specified cardiac dysrhythmias | circulatory system |
| 149.9 | -0.06854 | 0.189657 | -0.36137 | 0.717823 | -0.44026 | 0.303184 | EUR | Cancer of of nasal cavities | neoplasms |
| 446 | -0.03118 | 0.086283 | -0.36136 | 0.717831 | -0.20029 | 0.137932 | EUR | Polyarteritis nodosa and allied conditions | circulatory system |
| 564.9 | -0.01113 | 0.030819 | -0.36103 | 0.718076 | -0.07153 | 0.049278 | EUR | Personal history of diseases of digestive system | digestive |
| 225.1 | -0.02337 | 0.06475 | -0.36087 | 0.7182 | -0.15027 | 0.103541 | EUR | Benign neoplasm of brain, cranial nerves, meninges | neoplasms |
| 79.2 | 0.048849 | 0.135436 | 0.360683 | 0.718336 | -0.2166 | 0.314299 | EUR | Infectious mononucleosis | infectious diseases |
| 513.31 | 0.030775 | 0.085535 | 0.359796 | 0.718999 | -0.13687 | 0.198421 | EUR | Apnea | respiratory |
| 749.2 | 0.083516 | 0.23257 | 0.359099 | 0.719521 | -0.37231 | 0.539345 | EUR | Congenital anomalies of skull and face bones | congenital anomalies |
| 441 | 0.039021 | 0.108946 | 0.358167 | 0.720218 | -0.17451 | 0.252552 | EUR | Vascular insufficiency of intestine | circulatory system |
| 478 | -0.01989 | 0.055926 | -0.35556 | 0.722167 | -0.1295 | 0.089728 | EUR | Throat pain | respiratory |
| 514.1 | 0.030467 | 0.086042 | 0.354092 | 0.72327 | -0.13817 | 0.199107 | EUR | Abnormal results of function study of pulmonary system | respiratory |
| 696.42 | 0.035707 | 0.101407 | 0.352119 | 0.724749 | -0.16305 | 0.234461 | EUR | Psoriatic arthropathy | dermatologic |
| 592.2 | -0.03435 | 0.097611 | -0.35187 | 0.724934 | -0.22566 | 0.156968 | EUR | Urethritis and urethral syndrome | genitourinary |
| 790.1 | 0.031079 | 0.088387 | 0.35162 | 0.725123 | -0.14216 | 0.204315 | EUR | Elevated sedimentation rate | symptoms |
| 569.2 | 0.02063 | 0.059043 | 0.349398 | 0.726791 | -0.09509 | 0.136352 | EUR | Gastrointestinal complications | digestive |
| 793 | -0.02943 | 0.084234 | -0.34939 | 0.726798 | -0.19453 | 0.135666 | EUR | Nonspecific abnormal findings on radiological and other examination of musculoskeletal system | symptoms |
| 323.2 | -0.09437 | 0.270286 | -0.34915 | 0.726974 | -0.62412 | 0.435379 | EUR | Acute (transverse) myelitis | neurological |
| 610.3 | -0.03466 | 0.099294 | -0.34905 | 0.727049 | -0.22927 | 0.159954 | EUR | Fibrosclerosis of breast | genitourinary |
| 252.1 | 0.01599 | 0.045911 | 0.348283 | 0.727628 | -0.07399 | 0.105973 | EUR | Hyperparathyroidism | endocrine/metabolic |
| 356 | 0.015525 | 0.044805 | 0.346504 | 0.728964 | -0.07229 | 0.10334 | EUR | Hereditary and idiopathic peripheral neuropathy | neurological |
| 701.5 | -0.02873 | 0.083026 | -0.34598 | 0.729358 | -0.19145 | 0.134003 | EUR | Abnormal granulation tissue | dermatologic |
| 145.3 | 0.058879 | 0.170699 | 0.344932 | 0.730146 | -0.27568 | 0.393443 | EUR | Cancer of major salivary glands | neoplasms |
| 695 | -0.0104 | 0.030186 | -0.34469 | 0.730331 | -0.06957 | 0.048759 | EUR | Erythematous conditions | dermatologic |
| 528.4 | -0.11256 | 0.327335 | -0.34387 | 0.730947 | -0.75412 | 0.529006 | EUR | Cysts of oral soft tissues | digestive |
| 573.6 | 0.014519 | 0.042276 | 0.343437 | 0.73127 | -0.06834 | 0.097378 | EUR | Nonspecific elevation of levels of transaminase or lactic acid dehydrogenase [LDH] | digestive |
| 289.9 | 0.022755 | 0.06685 | 0.340391 | 0.733562 | -0.10827 | 0.153778 | EUR | Abnormality of red blood cells | hematopoietic |
| 276.11 | 0.025198 | 0.074149 | 0.339825 | 0.733989 | -0.12013 | 0.170528 | EUR | Hyperosmolality and/or hypernatremia | endocrine/metabolic |
| 619.2 | 0.021551 | 0.063677 | 0.338449 | 0.735025 | -0.10325 | 0.146355 | EUR | Disorders of uterus, NEC | genitourinary |
| 647.1 | 0.041725 | 0.123291 | 0.338425 | 0.735043 | -0.19992 | 0.28337 | EUR | Infections of genitourinary tract during pregnancy | pregnancy complications |
| 599.9 | 0.013468 | 0.039946 | 0.337146 | 0.736007 | -0.06482 | 0.09176 | EUR | Other abnormality of urination | genitourinary |
| 530.15 | -0.03693 | 0.111174 | -0.33217 | 0.739763 | -0.25483 | 0.180969 | EUR | Eosinophilic esophagitis | digestive |
| 743.22 | 0.031444 | 0.094663 | 0.332166 | 0.739764 | -0.15409 | 0.21698 | EUR | Pathologic fracture of femur | musculoskeletal |

| phecode | Coef. | Std.Err. | z | p_value_z | [0.025 | 0.975] | GIA | phenotype | category |
|---|---|---|---|---|---|---|---|---|---|
| 597 | 0.025032 | 0.075658 | 0.33086 | 0.74075 | -0.12325 | 0.173319 | EUR | Other disorders of urethra and urinary tract | genitourinary |
| 475.9 | -0.01158 | 0.035081 | -0.33017 | 0.741273 | -0.08034 | 0.057175 | EUR | Postnasal drip | respiratory |
| 252 | 0.014575 | 0.044158 | 0.33006 | 0.741354 | -0.07197 | 0.101123 | EUR | Disorders of parathyroid gland | endocrine/metabolic |
| 90 | 0.023123 | 0.070085 | 0.329919 | 0.741461 | -0.11424 | 0.160488 | EUR | Sexually transmitted infections (not HIV or hepatitis) | infectious diseases |
| 429 | -0.00921 | 0.027995 | -0.32894 | 0.742204 | -0.06408 | 0.045661 | EUR | Ill-defined descriptions and complications of heart disease | circulatory system |
| 159.4 | -0.03865 | 0.11775 | -0.32824 | 0.742727 | -0.26944 | 0.192134 | EUR | Malignant neoplasm of retroperitoneum and peritoneum | neoplasms |
| 244.4 | -0.00838 | 0.025546 | -0.32805 | 0.742874 | -0.05845 | 0.041689 | EUR | Hypothyroidism NOS | endocrine/metabolic |
| 362.4 | -0.02772 | 0.084574 | -0.32771 | 0.74313 | -0.19348 | 0.138046 | EUR | Retinal vascular changes and abnomalities | sense organs |
| 366.1 | -0.05287 | 0.161761 | -0.32683 | 0.743797 | -0.36991 | 0.264177 | EUR | Nonsenile Cataract | sense organs |
| 350.6 | 0.0183 | 0.056202 | 0.325604 | 0.744724 | -0.09185 | 0.128454 | EUR | Disturbances of sensation of smell and taste | neurological |
| 250.4 | 0.007297 | 0.022415 | 0.325552 | 0.744763 | -0.03663 | 0.051229 | EUR | Abnormal glucose | endocrine/metabolic |
| 751.12 | -0.0756 | 0.232314 | -0.32542 | 0.744862 | -0.53093 | 0.379727 | EUR | Congenital anomalies of male genital organs | congenital anomalies |
| 41 | -0.00927 | 0.028548 | -0.32476 | 7.45E-01 | -0.06522 | 0.046682 | EUR | Bacterial infection NOS | infectious diseases |
| 381.11 | -0.01749 | 0.054401 | -0.32147 | 0.747855 | -0.12411 | 0.089135 | EUR | Suppurative and unspecified otitis media | sense organs |
| 198.4 | -0.01998 | 0.062434 | -0.32005 | 0.748929 | -0.14235 | 0.102386 | EUR | Secondary malignant neoplasm of liver | neoplasms |
| 535.2 | -0.01292 | 0.040607 | -0.31823 | 0.750313 | -0.09251 | 0.066666 | EUR | Atrophic gastritis | digestive |
| 259 | -0.01638 | 0.051484 | -0.31818 | 0.750347 | -0.11729 | 0.084526 | EUR | Other endocrine disorders | endocrine/metabolic |
| 870.4 | 0.051173 | 0.160842 | 0.318158 | 0.750365 | -0.26407 | 0.366418 | EUR | Open wound of nose and sinus | injuries & poisonings |
| 789 | 0.006974 | 0.021963 | 0.317541 | 0.750833 | -0.03607 | 0.05002 | EUR | Nausea and vomiting | symptoms |
| 8.52 | -0.02426 | 0.077074 | -0.31479 | 7.53E-01 | -0.17532 | 0.126799 | EUR | Intestinal infection due to C. difficile | infectious diseases |
| 350 | -0.00867 | 0.027594 | -0.31405 | 0.753485 | -0.06275 | 0.045417 | EUR | Abnormal movement | neurological |
| 577.3 | 0.020356 | 0.064824 | 0.314025 | 0.753502 | -0.1067 | 0.147409 | EUR | Cyst and pseudocyst of pancreas | digestive |
| 375 | -0.0239 | 0.076225 | -0.31355 | 0.753867 | -0.1733 | 0.125499 | EUR | Disorders of lacrimal system | sense organs |
| 575.2 | -0.02479 | 0.079144 | -0.31325 | 0.754094 | -0.17991 | 0.130328 | EUR | Obstruction of bile duct | digestive |
| 972.1 | 0.092157 | 0.294844 | 0.312561 | 0.754614 | -0.48573 | 0.67004 | EUR | Cardiac rhythm regulators causing adverse effects in therapeutic use | injuries & poisonings |
| 362.1 | -0.11744 | 0.37599 | -0.31234 | 0.754783 | -0.85436 | 0.61949 | EUR | Retinopathy of prematurity | sense organs |
| 957 | 0.096295 | 0.308956 | 0.31168 | 0.755284 | -0.50925 | 0.701838 | EUR | Injury to other and unspecified nerves | injuries & poisonings |
| 287.2 | 0.136254 | 0.43736 | 0.311537 | 0.755392 | -0.72096 | 0.993463 | EUR | Allergic purpura | hematopoietic |
| 270.2 | 0.075163 | 0.243445 | 0.308746 | 0.757515 | -0.40198 | 0.552305 | EUR | Disorders of amino-acid metabolism | endocrine/metabolic |
| 275.11 | -0.06758 | 0.219252 | -0.30824 | 0.757898 | -0.49731 | 0.362144 | EUR | Hereditary hemochromatosis | hematopoietic |
| 555.1 | 0.018835 | 0.061244 | 0.307547 | 0.758427 | -0.1012 | 0.13887 | EUR | Regional enteritis | digestive |
| 743.2 | 0.017557 | 0.057211 | 0.306889 | 0.758928 | -0.09457 | 0.129689 | EUR | Pathologic fracture | musculoskeletal |
| 269 | -0.01443 | 0.047379 | -0.30454 | 0.760713 | -0.10729 | 0.078432 | EUR | Proteinuria | endocrine/metabolic |
| 253 | 0.016389 | 0.054287 | 0.301898 | 0.76273 | -0.09001 | 0.122789 | EUR | Disorders of the pituitary gland and its hypothalamic control | endocrine/metabolic |
| 282 | 0.019893 | 0.066076 | 0.301068 | 0.763362 | -0.10961 | 0.149399 | EUR | Hereditary hemolytic anemias | hematopoietic |
| 512.8 | 0.006621 | 0.022009 | 0.300836 | 0.763539 | -0.03652 | 0.049759 | EUR | Cough | respiratory |
| 755.1 | -0.03252 | 0.108566 | -0.29952 | 0.764544 | -0.2453 | 0.180268 | EUR | Congenital deformities of feet | congenital anomalies |
| 965.3 | 0.110017 | 0.368484 | 0.298566 | 0.765271 | -0.6122 | 0.832233 | EUR | Salicylates causing adverse effects in therapeutic use | injuries & poisonings |
| 580.12 | 0.053369 | 0.179288 | 0.297675 | 0.765952 | -0.29803 | 0.404766 | EUR | Non-proliferative glomerulonephritis | genitourinary |
| 528.6 | 0.057957 | 0.197172 | 0.29394 | 0.768803 | -0.32849 | 0.444407 | EUR | Leukoplakia of oral mucosa | digestive |
| 711.3 | -0.08929 | 0.304256 | -0.29349 | 0.769151 | -0.68563 | 0.507036 | EUR | Behcet's syndrome | musculoskeletal |
| 371.1 | -0.03242 | 0.111135 | -0.29174 | 0.770484 | -0.25024 | 0.185399 | EUR | Uveitis, noninfectious or NOS | sense organs |
| 735.21 | 0.019112 | 0.065558 | 0.291529 | 0.770647 | -0.10938 | 0.147603 | EUR | Hammer toe (acquired) | musculoskeletal |
| 286.8 | 0.016943 | 0.058224 | 0.291002 | 0.77105 | -0.09717 | 0.131061 | EUR | Hypercoagulable state | hematopoietic |
| 727.2 | 0.030718 | 0.106238 | 0.289144 | 0.772471 | -0.1775 | 0.23894 | EUR | Bursitis disorders | musculoskeletal |
| 275.6 | 0.013786 | 0.047714 | 0.288939 | 0.772628 | -0.07973 | 0.107304 | EUR | Hypercalcemia | endocrine/metabolic |
| 800.2 | -0.03935 | 0.136385 | -0.28851 | 0.772954 | -0.30666 | 0.227961 | EUR | Fracture of unspecified part of femur | injuries & poisonings |
| 702.4 | -0.07185 | 0.249729 | -0.28772 | 0.773559 | -0.56131 | 0.417608 | EUR | Degenerative skin disorders | dermatologic |
| 747.1 | -0.01242 | 0.043224 | -0.28742 | 0.773791 | -0.09714 | 0.072294 | EUR | Cardiac congenital anomalies | congenital anomalies |
| 365.1 | 0.017593 | 0.0613 | 0.286989 | 0.77412 | -0.10255 | 0.137739 | EUR | Open-angle glaucoma | sense organs |
| 198 | 0.009223 | 0.032154 | 0.286826 | 0.774246 | -0.0538 | 0.072244 | EUR | Secondary malignant neoplasm | neoplasms |
| 213 | 0.029959 | 0.104701 | 0.286136 | 0.774774 | -0.17525 | 0.23517 | EUR | Benign neoplasm of bone and articular cartilage | neoplasms |
| 974 | 0.084022 | 0.295194 | 0.284634 | 0.775924 | -0.49455 | 0.662591 | EUR | Poisoning by water, mineral, and uric acid metabolism drugs | injuries & poisonings |
| 136 | -0.00852 | 0.02995 | -0.28462 | 0.775935 | -0.06723 | 0.050177 | EUR | Other infectious and parasitic diseases | infectious diseases |
| 465 | -0.0064 | 0.022504 | -0.28458 | 0.775967 | -0.05051 | 0.037703 | EUR | Acute upper respiratory infections of multiple or unspecified sites | respiratory |
| 271.9 | -0.04777 | 0.168057 | -0.28422 | 0.776238 | -0.37715 | 0.28162 | EUR | Other disorders of carbohydrate transport and metabolism | endocrine/metabolic |
| 752 | 0.024846 | 0.087416 | 0.284224 | 0.776239 | -0.14649 | 0.196178 | EUR | Nervous system congenital anomalies | congenital anomalies |
| 818 | 0.041476 | 0.1461 | 0.283889 | 0.776495 | -0.24487 | 0.327826 | EUR | Intracranial hemorrhage (injury) | injuries & poisonings |
| 446.8 | -0.06767 | 0.238817 | -0.28337 | 0.776896 | -0.53575 | 0.4004 | EUR | Thrombotic microangiopathy | circulatory system |

| phecode | Coef. | Std.Err. | z | p_value_z | [0.025 | 0.975] | GIA | phenotype | category |
|---|---|---|---|---|---|---|---|---|---|
| 960.1 | -0.07519 | 0.266021 | -0.28265 | 0.777446 | -0.59658 | 0.446202 | EUR | Adverse effects of antibacterials (not penicillins) | injuries & poisonings |
| 293.1 | 0.009938 | 0.035287 | 0.281639 | 0.778221 | -0.05922 | 0.0791 | EUR | Swelling, mass, or lump in head and neck [Space-occupying lesion, intracranial NOS] | mental disorders |
| 729.3 | 0.057591 | 0.204612 | 0.281464 | 0.778355 | -0.34344 | 0.458623 | EUR | Panniculitis | musculoskeletal |
| 360.3 | 0.1028 | 0.367484 | 0.279741 | 0.779676 | -0.61746 | 0.823057 | EUR | Hypotony of eye | sense organs |
| 681 | -0.00815 | 0.029124 | -0.27974 | 0.779677 | -0.06523 | 0.048934 | EUR | Superficial cellulitis and abscess | dermatologic |
| 277.6 | 0.076629 | 0.275011 | 0.27864 | 0.780521 | -0.46238 | 0.615642 | EUR | Other deficiencies of circulating enzymes | endocrine/metabolic |
| 260.7 | -0.05906 | 0.213562 | -0.27654 | 0.78213 | -0.47763 | 0.359515 | EUR | Polyphagia | endocrine/metabolic |
| 429.9 | 0.024396 | 0.088223 | 0.27653 | 0.782141 | -0.14852 | 0.19731 | EUR | Cardiac complications, not elsewhere classified | circulatory system |
| 741.3 | -0.02436 | 0.088359 | -0.27565 | 0.782815 | -0.19754 | 0.148825 | EUR | Difficulty in walking | musculoskeletal |
| 596 | 0.009956 | 0.036244 | 0.274696 | 0.78355 | -0.06108 | 0.080993 | EUR | Other disorders of bladder | genitourinary |
| 569.1 | 0.022884 | 0.083405 | 0.274375 | 0.783797 | -0.14059 | 0.186356 | EUR | Toxic gastroenteritis and colitis | digestive |
| 790.9 | -0.04522 | 0.165214 | -0.27369 | 0.78432 | -0.36903 | 0.278596 | EUR | Abnormal arterial blood gases | symptoms |
| 300.9 | 0.019243 | 0.07046 | 0.273099 | 0.784777 | -0.11886 | 0.157343 | EUR | Posttraumatic stress disorder | mental disorders |
| 8.6 | -0.02494 | 0.091783 | -0.2717 | 7.86E-01 | -0.20483 | 0.154955 | EUR | Viral Enteritis | infectious diseases |
| 112.3 | -0.02528 | 0.093083 | -0.27163 | 0.785903 | -0.20772 | 0.157155 | EUR | Candidiasis of skin and nails | infectious diseases |
| 527.8 | 0.029039 | 0.10707 | 0.271219 | 0.786222 | -0.18081 | 0.238892 | EUR | Other specified diseases of the salivary glands | digestive |
| 242.2 | -0.06641 | 0.248386 | -0.26738 | 0.789175 | -0.55324 | 0.420414 | EUR | Toxic multinodular goiter | endocrine/metabolic |
| 984 | 0.11089 | 0.414823 | 0.267319 | 0.789224 | -0.70215 | 0.923929 | EUR | Toxic effect of lead and its compounds (including fumes) | injuries & poisonings |
| 443.1 | -0.01608 | 0.060177 | -0.26715 | 0.789357 | -0.13402 | 0.101869 | EUR | Raynaud's syndrome | circulatory system |
| 752.1 | -0.04324 | 0.163785 | -0.26399 | 0.791784 | -0.36425 | 0.277774 | EUR | Neural tube defects | congenital anomalies |
| 701.2 | -0.00901 | 0.034174 | -0.26372 | 0.791993 | -0.07599 | 0.057967 | EUR | Scar conditions and fibrosis of skin | dermatologic |
| 807 | -0.02379 | 0.090388 | -0.26325 | 0.792358 | -0.20095 | 0.153362 | EUR | Fracture of ribs | injuries & poisonings |
| 374 | -0.01058 | 0.040527 | -0.26109 | 0.794025 | -0.09001 | 0.068851 | EUR | Other disorders of eyelids | sense organs |
| 184 | -0.01989 | 0.076517 | -0.25992 | 0.794926 | -0.16986 | 0.130083 | EUR | Cancer of other female genital organs | neoplasms |
| 270.3 | 0.014922 | 0.057431 | 0.259832 | 0.794994 | -0.09764 | 0.127485 | EUR | Disorders of plasma protein metabolism | endocrine/metabolic |
| 377.3 | -0.02547 | 0.098112 | -0.25956 | 0.7952 | -0.21776 | 0.166829 | EUR | Optic neuritis/neuropathy | sense organs |
| 220 | 0.026664 | 0.102962 | 0.25897 | 0.795658 | -0.17514 | 0.228466 | EUR | Benign neoplasm of ovary | neoplasms |
| 204.4 | 0.02611 | 0.101097 | 0.258269 | 0.796199 | -0.17204 | 0.224256 | EUR | Multiple myeloma | neoplasms |
| 983 | 0.129336 | 0.501149 | 0.258079 | 0.796346 | -0.8529 | 1.111569 | EUR | Toxic effect of corrosive aromatics, acids, and caustic alkalis | injuries & poisonings |
| 443.8 | -0.02851 | 0.111302 | -0.25614 | 0.797839 | -0.24666 | 0.189638 | EUR | Other specified peripheral vascular diseases | circulatory system |
| 198.5 | 0.023005 | 0.090444 | 0.254362 | 0.799216 | -0.15426 | 0.200271 | EUR | Secondary malignancy of brain/spine | neoplasms |
| 910 | 0.049079 | 0.193225 | 0.254001 | 0.799495 | -0.32964 | 0.427794 | EUR | Superficial injury, infected | injuries & poisonings |
| 733.9 | 0.0186 | 0.073322 | 0.253677 | 0.799745 | -0.12511 | 0.162308 | EUR | Chondromalacia | musculoskeletal |
| 289.5 | 0.025677 | 0.101349 | 0.253357 | 0.799992 | -0.17296 | 0.224318 | EUR | Diseases of spleen | hematopoietic |
| 279.7 | 0.008947 | 0.035325 | 0.253271 | 0.800059 | -0.06029 | 0.078183 | EUR | Other immunological findings | endocrine/metabolic |
| 613.5 | -0.01211 | 0.047833 | -0.25324 | 0.800085 | -0.10586 | 0.081638 | EUR | Mastodynia | genitourinary |
| 619.1 | -0.01532 | 0.060594 | -0.25288 | 0.800361 | -0.13408 | 0.103438 | EUR | Noninflammatory disorders of ovary, fallopian tube, and broad ligament | genitourinary |
| 295.2 | -0.03454 | 0.137559 | -0.2511 | 0.801734 | -0.30415 | 0.235068 | EUR | Paranoid disorders | mental disorders |
| 297.1 | 0.021839 | 0.087575 | 0.249378 | 0.803069 | -0.1498 | 0.193483 | EUR | Suicidal ideation | mental disorders |
| 117.1 | 0.077027 | 0.313577 | 0.24564 | 0.805961 | -0.53757 | 0.691627 | EUR | Histoplasmosis | infectious diseases |
| 588.2 | -0.01518 | 0.061816 | -0.24558 | 0.806009 | -0.13634 | 0.105976 | EUR | Secondary hyperparathyroidism (of renal origin) | genitourinary |
| 344 | -0.02433 | 0.099202 | -0.24524 | 0.806273 | -0.21876 | 0.170105 | EUR | Other paralytic syndromes | neurological |
| 90.3 | -0.08679 | 0.35433 | -0.24494 | 0.806503 | -0.78126 | 0.607684 | EUR | Venereal diseases due to Chlamydia trachomatis | infectious diseases |
| 1005 | -0.00549 | 0.022468 | -0.24454 | 0.806812 | -0.04953 | 0.038542 | EUR | Other symptoms | other |
| 244.1 | 0.01204 | 0.049245 | 0.244495 | 0.806848 | -0.08448 | 0.108558 | EUR | Secondary hypothyroidism | endocrine/metabolic |
| 733.2 | -0.03202 | 0.131232 | -0.24398 | 0.807247 | -0.28923 | 0.225192 | EUR | Cyst of bone | musculoskeletal |
| 526.41 | -0.01738 | 0.071392 | -0.24346 | 0.807647 | -0.15731 | 0.122544 | EUR | Temporomandibular joint disorder, unspecified | digestive |
| 281.12 | 0.030839 | 0.126834 | 0.243143 | 0.807895 | -0.21775 | 0.27943 | EUR | Other vitamin B12 deficiency anemia | hematopoietic |
| 300.13 | 0.018113 | 0.074641 | 0.24267 | 0.808261 | -0.12818 | 0.164407 | EUR | Phobia | mental disorders |
| 624.1 | -0.04199 | 0.17445 | -0.24068 | 0.809799 | -0.3839 | 0.299928 | EUR | Dystrophy of female genital tract | genitourinary |
| 612 | -0.01632 | 0.068363 | -0.23868 | 0.811354 | -0.15031 | 0.117672 | EUR | Breast conditions, congenital or relating to hormones | genitourinary |
| 586.12 | -0.03927 | 0.165675 | -0.23705 | 0.812622 | -0.36399 | 0.285444 | EUR | Vesicoureteral reflux | genitourinary |
| 697 | 0.02663 | 0.112491 | 0.236731 | 0.812866 | -0.19385 | 0.247109 | EUR | Sarcoidosis | dermatologic |
| 1006 | 0.065323 | 0.276142 | 0.236556 | 0.813001 | -0.4759 | 0.606551 | EUR | Crushing injury | other |
| 749 | -0.02459 | 0.104181 | -0.23599 | 0.813443 | -0.22878 | 0.179606 | EUR | Congenital anomalies of face and neck | congenital anomalies |
| 568.1 | -0.0114 | 0.048374 | -0.23563 | 0.813721 | -0.10621 | 0.083413 | EUR | Peritoneal adhesions (postoperative) (postinfection) | digestive |
| 287.4 | -0.04803 | 0.204808 | -0.2345 | 0.814594 | -0.44944 | 0.353388 | EUR | Qualitative platelet defects | hematopoietic |
| 252.2 | -0.02241 | 0.095766 | -0.23402 | 0.81497 | -0.21011 | 0.165288 | EUR | Hypoparathyroidism | endocrine/metabolic |
| 172.3 | -0.0116 | 0.049651 | -0.23371 | 0.81521 | -0.10892 | 0.08571 | EUR | Carcinoma in situ of skin | neoplasms |

| phecode | Coef. | Std.Err. | z | p_value_z | [0.025 | 0.975] | GIA | phenotype | category |
|---|---|---|---|---|---|---|---|---|---|
| 624.9 | -0.01252 | 0.054052 | -0.23158 | 0.816868 | -0.11846 | 0.093423 | EUR | stress incontinence, female | genitourinary |
| 592 | 0.007115 | 0.030949 | 0.229893 | 0.818175 | -0.05354 | 0.067774 | EUR | Cystitis and urethritis | genitourinary |
| 949 | -0.00615 | 0.026811 | -0.2294 | 0.818557 | -0.0587 | 0.046397 | EUR | Allergies, other | injuries & poisonings |
| 277.7 | -0.01669 | 0.072902 | -0.22888 | 0.818965 | -0.15957 | 0.126199 | EUR | Dysmetabolic syndrome X | endocrine/metabolic |
| 362.22 | 0.028153 | 0.123112 | 0.22868 | 0.819118 | -0.21314 | 0.269448 | EUR | Macular degeneration, wet | sense organs |
| 561.1 | -0.00573 | 0.025151 | -0.22771 | 0.819871 | -0.05502 | 0.043568 | EUR | Diarrhea | digestive |
| 204.21 | 0.029673 | 0.130514 | 0.227352 | 0.82015 | -0.22613 | 0.285475 | EUR | Myeloid leukemia, acute | neoplasms |
| 250.13 | 0.033285 | 0.147501 | 0.225658 | 0.821467 | -0.25581 | 0.322382 | EUR | Type 1 diabetes with ophthalmic manifestations | endocrine/metabolic |
| 615 | 0.013387 | 0.059583 | 0.22468 | 0.822228 | -0.10339 | 0.130169 | EUR | Endometriosis | genitourinary |
| 134.1 | -0.05053 | 0.22494 | -0.22463 | 0.822269 | -0.4914 | 0.390347 | EUR | Intestinal helminthiases | infectious diseases |
| 656.6 | 0.166611 | 0.746227 | 0.223271 | 0.823325 | -1.29597 | 1.629189 | EUR | Perinatal disorders of digestive system | pregnancy complications |
| 242 | -0.01272 | 0.057032 | -0.22307 | 0.823478 | -0.1245 | 0.099058 | EUR | Thyrotoxicosis with or without goiter | endocrine/metabolic |
| 346 | -0.0323 | 0.144888 | -0.22293 | 0.823589 | -0.31628 | 0.251675 | EUR | Abnormal findings on study of brain and/or nervous system | neurological |
| 742 | -0.01047 | 0.047035 | -0.22251 | 0.823917 | -0.10265 | 0.081722 | EUR | Derangement of joint, non-traumatic | musculoskeletal |
| 790 | 0.007526 | 0.033841 | 0.222407 | 0.823997 | -0.0588 | 0.073853 | EUR | Nonspecific findings on examination of blood | symptoms |
| 520.1 | 0.168199 | 0.757829 | 0.221949 | 0.824354 | -1.31712 | 1.653517 | EUR | Hereditary disturbances in tooth structure | digestive |
| 153 | 0.011296 | 0.051074 | 0.221174 | 0.824957 | -0.08881 | 0.1114 | EUR | Colorectal cancer | neoplasms |
| 743.4 | 0.022735 | 0.104304 | 0.217973 | 0.82745 | -0.1817 | 0.227166 | EUR | Stress fracture | musculoskeletal |
| 994.1 | -0.02033 | 0.093287 | -0.21789 | 0.827515 | -0.20317 | 0.162513 | EUR | Systemic inflammatory response syndrome (SIRS) | injuries & poisonings |
| 601.3 | 0.025222 | 0.116402 | 0.216679 | 0.828458 | -0.20292 | 0.253365 | EUR | Orchitis and epididymitis | genitourinary |
| 961 | 0.015937 | 0.073747 | 0.216104 | 0.828907 | -0.1286 | 0.160479 | EUR | Poisoning by other anti-infectives | injuries & poisonings |
| 537 | 0.0068 | 0.031516 | 0.215776 | 0.829163 | -0.05497 | 0.068571 | EUR | Other disorders of stomach and duodenum | digestive |
| 592.3 | -0.124 | 0.576005 | -0.21528 | 0.829548 | -1.25295 | 1.004946 | EUR | Urethral stricture due to infecton | genitourinary |
| 681.3 | -0.01503 | 0.069994 | -0.21478 | 0.829936 | -0.15222 | 0.122152 | EUR | Cellulitis and abscess of arm/hand | dermatologic |
| 452.1 | -0.12213 | 0.571312 | -0.21377 | 0.830727 | -1.24188 | 0.997621 | EUR | Iatrogenic pulmonary embolism and infarction | circulatory system |
| 348.7 | -0.02047 | 0.096307 | -0.21253 | 0.831694 | -0.20923 | 0.168289 | EUR | Coma | neurological |
| 327.7 | 0.010101 | 0.04772 | 0.211665 | 0.832368 | -0.08343 | 0.103629 | EUR | Sleep related movement disorders | neurological |
| 277.8 | -0.16362 | 0.783281 | -0.20889 | 0.834533 | -1.69882 | 1.371582 | EUR | Carnitine deficiencies | endocrine/metabolic |
| 525.1 | -0.04286 | 0.206284 | -0.20776 | 0.83542 | -0.44717 | 0.361453 | EUR | Loss of teeth or edentulism | digestive |
| 427 | -0.00444 | 0.021449 | -0.20713 | 0.835904 | -0.04648 | 0.037597 | EUR | Cardiac dysrhythmias | circulatory system |
| 280 | -0.00638 | 0.030885 | -0.20653 | 0.836375 | -0.06691 | 0.054155 | EUR | Iron deficiency anemias | hematopoietic |
| 112 | -0.00762 | 0.037061 | -0.20552 | 0.837166 | -0.08025 | 0.065021 | EUR | Candidiasis | infectious diseases |
| 656.3 | -0.05403 | 0.263877 | -0.20476 | 0.83776 | -0.57122 | 0.463157 | EUR | Endocrine and metabolic disturbances of fetus and newborn | pregnancy complications |
| 334.21 | -0.07075 | 0.347944 | -0.20333 | 0.83888 | -0.7527 | 0.611212 | EUR | Amyotrophic Lateral Sclerosis | neurological |
| 275.5 | 0.008122 | 0.040164 | 0.202221 | 0.839744 | -0.0706 | 0.086842 | EUR | Disorders of calcium/phosphorus metabolism | endocrine/metabolic |
| 204.22 | -0.03948 | 0.1967 | -0.20069 | 0.840941 | -0.425 | 0.34605 | EUR | Myeloid leukemia, chronic | neoplasms |
| 255.3 | -0.04496 | 0.224453 | -0.2003 | 0.841245 | -0.48488 | 0.394962 | EUR | Adrenogenital disorders | endocrine/metabolic |
| 366 | -0.0057 | 0.028472 | -0.20024 | 0.84129 | -0.06151 | 0.050103 | EUR | Cataract | sense organs |
| 259.3 | 0.185882 | 0.932119 | 0.199419 | 0.841935 | -1.64104 | 2.012801 | EUR | Delay in sexual development and puberty NEC | endocrine/metabolic |
| 793.2 | -0.00922 | 0.046313 | -0.19907 | 0.842208 | -0.09999 | 0.081553 | EUR | Nonspecific abnormal findings on radiological and other examination of other intrathoracic organs (echocardiogram, etc) | symptoms |
| 377 | 0.014482 | 0.073025 | 0.198315 | 0.842798 | -0.12864 | 0.157608 | EUR | Disorders of optic nerve and visual pathways | sense organs |
| 442 | 0.009604 | 0.04879 | 0.196845 | 0.843949 | -0.08602 | 0.105231 | EUR | Other aneurysm | circulatory system |
| 340.1 | -0.00879 | 0.044922 | -0.19567 | 0.844869 | -0.09684 | 0.079255 | EUR | Migrain with aura | neurological |
| 362.6 | 0.018703 | 0.096043 | 0.194733 | 0.845602 | -0.16954 | 0.206943 | EUR | Peripheral retinal degenerations | sense organs |
| 623 | 0.013294 | 0.06837 | 0.194436 | 0.845834 | -0.12071 | 0.147295 | EUR | Hypertrophy of female genital organs | genitourinary |
| 769 | 0.046348 | 0.238881 | 0.19402 | 0.84616 | -0.42185 | 0.514545 | EUR | Nonallopathic lesions NEC | symptoms |
| 300.8 | -0.02085 | 0.107672 | -0.19362 | 0.846472 | -0.23188 | 0.190185 | EUR | Acute reaction to stress | mental disorders |
| 532 | 0.00564 | 0.029277 | 0.192654 | 0.84723 | -0.05174 | 0.063023 | EUR | Dysphagia | digestive |
| 333.2 | -0.02459 | 0.12844 | -0.19142 | 0.8482 | -0.27632 | 0.227152 | EUR | Myoclonus | neurological |
| 472 | 0.006961 | 0.036436 | 0.191037 | 0.848497 | -0.06445 | 0.078375 | EUR | Chronic pharyngitis and nasopharyngitis | respiratory |
| 261.2 | 0.007211 | 0.037831 | 0.190617 | 0.848826 | -0.06694 | 0.081359 | EUR | Vitamin B-complex deficiencies | endocrine/metabolic |
| 443.7 | -0.01903 | 0.101082 | -0.18828 | 0.850655 | -0.21715 | 0.179085 | EUR | Peripheral angiopathy in diseases classified elsewhere | circulatory system |
| 560.4 | 0.009886 | 0.053245 | 0.185672 | 0.852702 | -0.09447 | 0.114244 | EUR | Other intestinal obstruction | digestive |
| 199.4 | -0.03825 | 0.207301 | -0.18451 | 0.853609 | -0.44455 | 0.368052 | EUR | Neurofibromatosis | neoplasms |
| 8.51 | -0.16179 | 0.878834 | -0.1841 | 8.54E-01 | -1.88427 | 1.560693 | EUR | Intestinal e.coli | infectious diseases |
| 165.1 | -0.01295 | 0.070385 | -0.18405 | 0.853977 | -0.15091 | 0.124999 | EUR | Cancer of bronchus; lung | neoplasms |
| 560.3 | 0.027013 | 0.147087 | 0.18365 | 0.854288 | -0.26127 | 0.315298 | EUR | Peritoneal or intestinal adhesions | digestive |
| 971 | 0.067849 | 0.370567 | 0.183094 | 0.854724 | -0.65845 | 0.794147 | EUR | Poisoning by drugs primarily affecting the autonomic nervous system | injuries & poisonings |
| 573.4 | -0.01989 | 0.108999 | -0.18248 | 0.855208 | -0.23352 | 0.193744 | EUR | Acute and subacute necrosis of liver | digestive |

| phecode | Coef. | Std.Err. | z | p_value_z | [0.025 | 0.975] | GIA | phenotype | category |
|---|---|---|---|---|---|---|---|---|---|
| 459.9 | 0.005895 | 0.032385 | 0.182031 | 0.855558 | -0.05758 | 0.069368 | EUR | Circulatory disease NEC | circulatory system |
| 752.2 | -0.01867 | 0.104005 | -0.17948 | 0.857558 | -0.22251 | 0.185179 | EUR | Other specified congenital anomalies of nervous system | congenital anomalies |
| 189.2 | -0.01531 | 0.08546 | -0.17918 | 0.857798 | -0.18281 | 0.152186 | EUR | Cancer of bladder | neoplasms |
| 750.5 | -0.11057 | 0.619104 | -0.17859 | 0.858259 | -1.32399 | 1.102855 | EUR | Congenital hypertrophic pyloric stenosis | congenital anomalies |
| 753.2 | 0.031673 | 0.177726 | 0.178212 | 0.858556 | -0.31666 | 0.380009 | EUR | Congenital anomalies of posterior segment of eye | congenital anomalies |
| 244 | -0.00444 | 0.025063 | -0.17731 | 0.859264 | -0.05357 | 0.044679 | EUR | Hypothyroidism | endocrine/metabolic |
| 689 | -0.00431 | 0.024355 | -0.17714 | 0.859396 | -0.05205 | 0.043421 | EUR | Disorder of skin and subcutaneous tissue NOS | dermatologic |
| 519 | 0.005522 | 0.031759 | 0.173877 | 0.861962 | -0.05673 | 0.06777 | EUR | Other diseases of respiratory system, not elsewhere classified | respiratory |
| 303.3 | 0.011663 | 0.067157 | 0.173674 | 0.862122 | -0.11996 | 0.14329 | EUR | Psychogenic disorder | mental disorders |
| 79.1 | 0.006487 | 0.037373 | 0.17358 | 0.862196 | -0.06676 | 0.079737 | EUR | Varicella infection | infectious diseases |
| 259.2 | -0.03694 | 0.215011 | -0.1718 | 0.863598 | -0.45835 | 0.384475 | EUR | Carcinoid syndrome | endocrine/metabolic |
| 117.2 | 0.03182 | 0.185291 | 0.171732 | 0.863648 | -0.33134 | 0.394984 | EUR | Coccidioidomycosis | infectious diseases |
| 288 | 0.004891 | 0.028552 | 0.171304 | 0.863985 | -0.05107 | 0.060853 | EUR | Diseases of white blood cells | hematopoietic |
| 818.1 | -0.04161 | 0.243144 | -0.17112 | 0.864128 | -0.51816 | 0.434946 | EUR | Subdural hemorrhage (injury) | injuries & poisonings |
| 800 | -0.00753 | 0.044109 | -0.1707 | 0.864459 | -0.09398 | 0.078922 | EUR | Fracture of lower limb | injuries & poisonings |
| 288.2 | 0.00507 | 0.029945 | 0.169301 | 0.86556 | -0.05362 | 0.063761 | EUR | Elevated white blood cell count | hematopoietic |
| 290.3 | 0.01119 | 0.066374 | 0.168589 | 0.86612 | -0.1189 | 0.14128 | EUR | Other persistent mental disorders due to conditions classified elsewhere | mental disorders |
| 712 | 0.036478 | 0.219584 | 0.166124 | 0.868059 | -0.3939 | 0.466854 | EUR | Infective connective tissue disorders | musculoskeletal |
| 270.34 | -0.05516 | 0.332108 | -0.1661 | 0.86808 | -0.70608 | 0.595758 | EUR | Alpha-1-antitrypsin deficiency | endocrine/metabolic |
| 277.5 | -0.01222 | 0.073734 | -0.1658 | 0.868318 | -0.15674 | 0.132291 | EUR | Other disorders of lipoid metabolism | endocrine/metabolic |
| 333.1 | 0.011092 | 0.067794 | 0.163612 | 0.870036 | -0.12178 | 0.143966 | EUR | Essential tremor | neurological |
| 427.61 | 0.010543 | 0.06464 | 0.163106 | 0.870435 | -0.11615 | 0.137235 | EUR | Supraventricular premature beats | circulatory system |
| 578.1 | -0.01554 | 0.096974 | -0.16029 | 0.872656 | -0.20561 | 0.174522 | EUR | Hematemesis | digestive |
| 474.2 | -0.01209 | 0.07595 | -0.15923 | 0.873484 | -0.16095 | 0.136766 | EUR | Chronic tonsillitis and adenoiditis | respiratory |
| 795.81 | 0.021143 | 0.133772 | 0.158049 | 0.874418 | -0.24105 | 0.283331 | EUR | Elevated carcinoembryonic antigen [CEA] | symptoms |
| 315 | -0.01036 | 0.06692 | -0.15484 | 0.87695 | -0.14152 | 0.120799 | EUR | Develomental delays and disorders | mental disorders |
| 277.1 | -0.05553 | 0.359795 | -0.15434 | 0.877344 | -0.76071 | 0.649655 | EUR | Disorders of porphyrin metabolism | endocrine/metabolic |
| 365 | 0.006093 | 0.039651 | 0.15366 | 0.877878 | -0.07162 | 0.083807 | EUR | Glaucoma | sense organs |
| 190 | -0.02316 | 0.151362 | -0.15299 | 0.878403 | -0.31982 | 0.273506 | EUR | Cancer of eye | neoplasms |
| 564 | -0.00361 | 0.024059 | -0.15012 | 0.880666 | -0.05077 | 0.043542 | EUR | Functional digestive disorders | digestive |
| 599.7 | 0.03269 | 0.218209 | 0.149811 | 0.880914 | -0.39499 | 0.460373 | EUR | Urethral discharge | genitourinary |
| 425.11 | 0.022575 | 0.151085 | 0.149422 | 0.88122 | -0.27355 | 0.318697 | EUR | Hypertrophic obstructive cardiomyopathy | circulatory system |
| 704.11 | 0.025275 | 0.169235 | 0.14935 | 0.881277 | -0.30642 | 0.356969 | EUR | Alopecia Areata | dermatologic |
| 295.1 | -0.02215 | 0.150029 | -0.14767 | 0.882606 | -0.31621 | 0.271898 | EUR | Schizophrenia | mental disorders |
| 835 | -0.00662 | 0.04511 | -0.14676 | 0.883322 | -0.09504 | 0.081795 | EUR | Internal derangement of knee | injuries & poisonings |
| 737 | -0.00602 | 0.041476 | -0.14514 | 0.884598 | -0.08731 | 0.075271 | EUR | Curvature of spine | musculoskeletal |
| 291.4 | -0.01137 | 0.078333 | -0.1451 | 0.884635 | -0.1649 | 0.142164 | EUR | Specific nonpsychotic mental disorders due to brain damage | mental disorders |
| 737.3 | 0.006346 | 0.044007 | 0.144196 | 0.885345 | -0.07991 | 0.092597 | EUR | Kyphoscoliosis and scoliosis | musculoskeletal |
| 275.1 | -0.01553 | 0.10842 | -0.14325 | 0.886096 | -0.22803 | 0.196968 | EUR | Disorders of iron metabolism | hematopoietic |
| 794 | -0.01798 | 0.12596 | -0.14273 | 0.886502 | -0.26485 | 0.228898 | EUR | Abnormal results of other function studies (bladder, pancreas, placenta, spleen, etc) | symptoms |
| 627.22 | -0.00507 | 0.036324 | -0.13963 | 0.888954 | -0.07627 | 0.066122 | EUR | Need for Hormone replacement therapy (postmenopausal) | genitourinary |
| 671 | -0.01192 | 0.085397 | -0.13961 | 0.888967 | -0.1793 | 0.155453 | EUR | Venous/cerebrovascular complications embolism in pregnancy and the puerperium | pregnancy complications |
| 674 | -0.01782 | 0.130453 | -0.13661 | 0.891338 | -0.2735 | 0.237862 | EUR | Other complications of the puerperium NEC | pregnancy complications |
| 381.2 | -0.00621 | 0.045487 | -0.13656 | 0.891379 | -0.09536 | 0.082941 | EUR | Eustachian tube disorders | sense organs |
| 751.11 | 0.019523 | 0.143077 | 0.136449 | 0.891466 | -0.2609 | 0.299949 | EUR | Congenital anomalies of female genital organs | congenital anomalies |
| 411.9 | -0.01197 | 0.087808 | -0.13637 | 0.891525 | -0.18408 | 0.160126 | EUR | Other acute and subacute forms of ischemic heart disease | circulatory system |
| 275 | 0.004121 | 0.030271 | 0.136133 | 0.891716 | -0.05521 | 0.063451 | EUR | Disorders of mineral metabolism | endocrine/metabolic |
| 475 | -0.00355 | 0.026315 | -0.13494 | 0.892658 | -0.05513 | 0.048026 | EUR | Chronic sinusitis | respiratory |
| 614.32 | 0.02551 | 0.190121 | 0.134175 | 0.893264 | -0.34712 | 0.398139 | EUR | Chronic inflammatory pelvic disease | genitourinary |
| 159 | -0.00854 | 0.064189 | -0.13309 | 0.894121 | -0.13435 | 0.117265 | EUR | Malignant neoplasm of other and ill-defined sites within the digestive organs and peritoneum | neoplasms |
| 644 | -0.01075 | 0.081468 | -0.13192 | 0.89505 | -0.17042 | 0.148927 | EUR | Anemia during pregnancy | pregnancy complications |
| 722.3 | 0.041036 | 0.312149 | 0.131463 | 0.895409 | -0.57076 | 0.652836 | EUR | Schmorl's nodes | musculoskeletal |
| 985 | 0.034391 | 0.26171 | 0.131411 | 0.895451 | -0.47855 | 0.547334 | EUR | Toxic effect of other metals | injuries & poisonings |
| 202 | -0.00775 | 0.059035 | -0.13131 | 0.89553 | -0.12346 | 0.107954 | EUR | Cancer of other lymphoid, histiocytic tissue | neoplasms |
| 386.2 | 0.006529 | 0.050197 | 0.130057 | 0.896522 | -0.09186 | 0.104914 | EUR | Peripheral or central vertigo | sense organs |
| 165 | -0.00854 | 0.066568 | -0.12835 | 0.897872 | -0.13901 | 0.121927 | EUR | Cancer within the respiratory system | neoplasms |
| 871 | -0.00468 | 0.037102 | -0.12609 | 0.89966 | -0.0774 | 0.068041 | EUR | Open wounds of extremities | injuries & poisonings |
| 750.22 | 0.015523 | 0.123297 | 0.125901 | 0.89981 | -0.22613 | 0.257181 | EUR | Congenital anomaly of gallbladder, bile ducts, liver, pancreas | congenital anomalies |
| 505 | 0.011616 | 0.092383 | 0.125737 | 0.89994 | -0.16945 | 0.192683 | EUR | Other pulmonary inflamation or edema | respiratory |

| phecode | Coef. | Std.Err. | z | p_value_z | [0.025 | 0.975] | GIA | phenotype | category |
|---|---|---|---|---|---|---|---|---|---|
| 458.9 | 0.004682 | 0.037611 | 0.124488 | 0.900929 | -0.06903 | 0.078398 | EUR | Hypotension NOS | circulatory system |
| 250.5 | -0.02747 | 0.220868 | -0.12435 | 0.901037 | -0.46036 | 0.405428 | EUR | Glycosuria or Acetonuria | endocrine/metabolic |
| 260.1 | 0.010356 | 0.083645 | 0.123806 | 0.901469 | -0.15358 | 0.174296 | EUR | Cachexia | endocrine/metabolic |
| 735.2 | -0.00636 | 0.053098 | -0.11977 | 0.904661 | -0.11043 | 0.09771 | EUR | Acquired toe deformities | musculoskeletal |
| 202.2 | -0.00766 | 0.064313 | -0.1191 | 0.905192 | -0.13371 | 0.11839 | EUR | Non-Hodgkins lymphoma | neoplasms |
| 696.2 | 0.034881 | 0.295978 | 0.117851 | 0.906186 | -0.54523 | 0.614988 | EUR | Parapsoriasis | dermatologic |
| 586.2 | -0.00484 | 0.041096 | -0.11777 | 0.906248 | -0.08539 | 0.075707 | EUR | Cyst of kidney, acquired | genitourinary |
| 280.1 | 0.003823 | 0.032591 | 0.11729 | 0.90663 | -0.06005 | 0.067699 | EUR | Iron deficiency anemias, unspecified or not due to blood loss | hematopoietic |
| 728.2 | 0.013066 | 0.111539 | 0.117139 | 0.90675 | -0.20555 | 0.231679 | EUR | Laxity of ligament or hypermobility syndrome | musculoskeletal |
| 690.1 | -0.00453 | 0.039451 | -0.11478 | 0.908621 | -0.08185 | 0.072795 | EUR | Seborrheic dermatitis | dermatologic |
| 713.5 | 0.026576 | 0.232308 | 0.1144 | 0.908921 | -0.42874 | 0.481891 | EUR | Arthropathy associated with neurological disorders | musculoskeletal |
| 149.2 | 0.023415 | 0.204988 | 0.114226 | 0.909058 | -0.37835 | 0.425184 | EUR | Cancer of nasopharynx | neoplasms |
| 958.2 | 0.039579 | 0.346932 | 0.114084 | 0.909171 | -0.6404 | 0.719554 | EUR | Traumatic and surgical subcutaneous emphysema | injuries & poisonings |
| 211 | -0.00484 | 0.042823 | -0.113 | 0.910028 | -0.08877 | 0.079092 | EUR | Benign neoplasm of other parts of digestive system | neoplasms |
| 747 | -0.00442 | 0.039225 | -0.11267 | 0.91029 | -0.0813 | 0.072461 | EUR | Cardiac and circulatory congenital anomalies | congenital anomalies |
| 277.51 | 0.010191 | 0.090928 | 0.112079 | 0.91076 | -0.16802 | 0.188407 | EUR | Lipoprotein disorders | endocrine/metabolic |
| 362.26 | -0.00681 | 0.061553 | -0.11063 | 0.911906 | -0.12745 | 0.113832 | EUR | Macular puckering of retina | sense organs |
| 258 | -0.02144 | 0.195444 | -0.10967 | 0.912667 | -0.4045 | 0.361628 | EUR | Iatrogenic endocrine disorders | endocrine/metabolic |
| 870 | 0.004045 | 0.036955 | 0.109458 | 0.912839 | -0.06838 | 0.076475 | EUR | Open wounds of head; neck; and trunk | injuries & poisonings |
| 433.5 | 0.011255 | 0.105688 | 0.106493 | 0.915191 | -0.19589 | 0.2184 | EUR | Cerebral aneurysm | circulatory system |
| 242.31 | 0.028397 | 0.268964 | 0.105581 | 0.915915 | -0.49876 | 0.555557 | EUR | Thyrotoxic exophthalmos | endocrine/metabolic |
| 430.3 | 0.011106 | 0.105341 | 0.105427 | 0.916037 | -0.19536 | 0.217571 | EUR | Subdural hemorrhage | circulatory system |
| 716.8 | -0.05348 | 0.511801 | -0.1045 | 0.916773 | -1.05659 | 0.949628 | EUR | Palindromic rheumatism | musculoskeletal |
| 279.8 | 0.010934 | 0.106235 | 0.102925 | 0.918022 | -0.19728 | 0.219151 | EUR | Other specified disorders involving the immune mechanism | endocrine/metabolic |
| 427.12 | 0.005183 | 0.050476 | 0.102683 | 0.918214 | -0.09375 | 0.104114 | EUR | Paroxysmal ventricular tachycardia | circulatory system |
| 747.2 | -0.00922 | 0.089841 | -0.10265 | 0.91824 | -0.18531 | 0.166862 | EUR | Congenital anomalies of peripheral vascular system | congenital anomalies |
| 157 | -0.00813 | 0.080028 | -0.10159 | 0.919085 | -0.16498 | 0.148723 | EUR | Pancreatic cancer | neoplasms |
| 368 | 0.002962 | 0.02969 | 0.099756 | 0.920538 | -0.05523 | 0.061152 | EUR | Visual disturbances | sense organs |
| 580.4 | -0.01039 | 0.105153 | -0.09885 | 0.921254 | -0.21649 | 0.195701 | EUR | Renal sclerosis, NOS | genitourinary |
| 429.3 | -0.00306 | 0.030972 | -0.09866 | 0.92141 | -0.06376 | 0.057648 | EUR | Symptoms involving cardiovascular system | circulatory system |
| 939.1 | 0.013863 | 0.143632 | 0.096515 | 0.923111 | -0.26765 | 0.295376 | EUR | Contact and allergic dermatitis of eyelid | dermatologic |
| 601.1 | -0.00679 | 0.070891 | -0.0958 | 0.92368 | -0.14574 | 0.132153 | EUR | Prostatitis | genitourinary |
| 732.7 | -0.03455 | 0.366343 | -0.09431 | 0.924862 | -0.75257 | 0.683469 | EUR | Osteochondritis dissecans | musculoskeletal |
| 348.2 | 0.006085 | 0.065489 | 0.092923 | 0.925965 | -0.12227 | 0.134442 | EUR | Cerebral edema and compression of brain | neurological |
| 520 | 0.031317 | 0.337272 | 0.092853 | 0.92602 | -0.62972 | 0.692357 | EUR | Disorders of tooth development | digestive |
| 272.9 | 0.009806 | 0.105727 | 0.092748 | 0.926104 | -0.19741 | 0.217026 | EUR | Unspecified disorder of lipoid metabolism | endocrine/metabolic |
| 389.5 | -0.00914 | 0.098715 | -0.09259 | 0.926227 | -0.20262 | 0.184337 | EUR | Disorders of acoustic nerve | sense organs |
| 245.21 | -0.00426 | 0.0461 | -0.09247 | 0.926324 | -0.09462 | 0.086091 | EUR | Chronic lymphocytic thyroiditis | endocrine/metabolic |
| 801.1 | -0.00783 | 0.084948 | -0.09216 | 0.926569 | -0.17432 | 0.158666 | EUR | Fracture of foot | injuries & poisonings |
| 737.2 | 0.024978 | 0.27343 | 0.091351 | 0.927213 | -0.51093 | 0.560891 | EUR | Lordosis (acquired) | musculoskeletal |
| 395.2 | 0.003835 | 0.042197 | 0.090876 | 0.927591 | -0.07887 | 0.08654 | EUR | Nonrheumatic aortic valve disorders | circulatory system |
| 575.9 | -0.00751 | 0.082889 | -0.09059 | 0.927818 | -0.16997 | 0.15495 | EUR | Nonspecific abnormal findings on radiological and other examination of biliary tract | digestive |
| 569 | -0.0025 | 0.027749 | -0.08994 | 0.928337 | -0.05688 | 0.051891 | EUR | Other disorders of intestine | digestive |
| 348.9 | 0.004454 | 0.049535 | 0.089923 | 0.928348 | -0.09263 | 0.10154 | EUR | Other conditions of brain, NOS | neurological |
| 480.2 | -0.00666 | 0.07415 | -0.08988 | 0.928381 | -0.152 | 0.138666 | EUR | Viral pneumonia | respiratory |
| 498 | -0.00751 | 0.084145 | -0.08924 | 0.928893 | -0.17243 | 0.157413 | EUR | Acute bronchospasm | respiratory |
| 721.2 | -0.00679 | 0.077714 | -0.08743 | 0.930326 | -0.15911 | 0.145521 | EUR | Spondylosis with myelopathy | musculoskeletal |
| 597.2 | 0.007743 | 0.088803 | 0.087195 | 0.930517 | -0.16631 | 0.181793 | EUR | Urinary complications NEC | genitourinary |
| 473.4 | 0.003321 | 0.038574 | 0.086093 | 0.931392 | -0.07228 | 0.078924 | EUR | Voice disturbance | respiratory |
| 253.2 | 0.010294 | 0.121468 | 0.08475 | 0.93246 | -0.22778 | 0.248367 | EUR | Pituitary hypofunction | endocrine/metabolic |
| 363 | 0.008744 | 0.105703 | 0.082718 | 0.934076 | -0.19843 | 0.215918 | EUR | Chorioretinal inflammations, scars, and other disorders of choroid | sense organs |
| 727.4 | 0.003949 | 0.047772 | 0.082672 | 0.934112 | -0.08968 | 0.09758 | EUR | Ganglion and cyst of synovium, tendon, and bursa | musculoskeletal |
| 70.1 | 0.01432 | 0.173548 | 0.082515 | 0.934237 | -0.32583 | 0.354469 | EUR | Viral hepatitis A | infectious diseases |
| 364.9 | -0.01626 | 0.201057 | -0.08087 | 0.935544 | -0.41032 | 0.377805 | EUR | Cornea replaced by transplant | sense organs |
| 480.13 | -0.01469 | 0.18432 | -0.07967 | 0.936496 | -0.37595 | 0.346575 | EUR | MRSA pneumonia | respiratory |
| 555.21 | 0.005322 | 0.067432 | 0.078919 | 0.937097 | -0.12684 | 0.137485 | EUR | Ulcerative colitis (chronic) | digestive |
| 291.1 | 0.014869 | 0.189802 | 0.078338 | 0.937559 | -0.35714 | 0.386874 | EUR | Transient mental disorders due to conditions classified elsewhere | mental disorders |
| 341 | 0.013336 | 0.171187 | 0.077906 | 0.937903 | -0.32218 | 0.348856 | EUR | Other demyelinating diseases of central nervous system | neurological |
| 529 | -0.00536 | 0.070898 | -0.07566 | 0.939686 | -0.14432 | 0.133592 | EUR | Diseases and other conditions of the tongue | digestive |

| phecode | Coef. | Std.Err. | z | p_value_z | [0.025 | 0.975] | GIA | phenotype | category |
|---|---|---|---|---|---|---|---|---|---|
| 427.41 | -0.00904 | 0.120905 | -0.07474 | 0.940419 | -0.24601 | 0.227932 | EUR | Ventricular fibrillation and flutter | circulatory system |
| 854 | 0.004601 | 0.063778 | 0.072145 | 0.942486 | -0.1204 | 0.129603 | EUR | Complications of cardiac/vascular device, implant, and graft | injuries & poisonings |
| 742.8 | -0.00535 | 0.074138 | -0.07211 | 0.942514 | -0.15065 | 0.139962 | EUR | Articular cartilage disorder | musculoskeletal |
| 1012 | -0.00889 | 0.124409 | -0.07142 | 0.94306 | -0.25272 | 0.234952 | EUR | Late effect | other |
| 297 | 0.005742 | 0.081384 | 0.070553 | 0.943754 | -0.15377 | 0.165251 | EUR | Suicidal ideation or attempt | mental disorders |
| 550.1 | 0.003255 | 0.046784 | 0.069585 | 0.944524 | -0.08844 | 0.09495 | EUR | Inguinal hernia | digestive |
| 283 | -0.00899 | 0.129406 | -0.06949 | 0.944599 | -0.26262 | 0.244639 | EUR | Acquired hemolytic anemias | hematopoietic |
| 749.1 | 0.020247 | 0.29164 | 0.069426 | 0.944651 | -0.55136 | 0.591852 | EUR | Cleft palate | congenital anomalies |
| 695.1 | -0.01553 | 0.223814 | -0.06938 | 0.944685 | -0.4542 | 0.423138 | EUR | Toxic erythema | dermatologic |
| 736.1 | -0.01553 | 0.225589 | -0.06883 | 0.945127 | -0.45767 | 0.426619 | EUR | Acquired deformities of forearm | musculoskeletal |
| 348 | 0.002959 | 0.04366 | 0.067779 | 0.945962 | -0.08261 | 0.088531 | EUR | Other conditions of brain | neurological |
| 358.1 | 0.011428 | 0.169164 | 0.067557 | 0.946138 | -0.32013 | 0.342983 | EUR | Myasthenia gravis | neurological |
| 370.1 | 0.012312 | 0.184663 | 0.066673 | 0.946842 | -0.34962 | 0.374245 | EUR | Corneal ulcer | sense organs |
| 690 | -0.00253 | 0.0392 | -0.06448 | 0.948591 | -0.07936 | 0.074302 | EUR | Erythematosquamous dermatosis | dermatologic |
| 197 | 0.001382 | 0.021648 | 0.063849 | 0.949091 | -0.04105 | 0.043811 | EUR | Chemotherapy | neoplasms |
| 379.9 | 0.002752 | 0.045451 | 0.060541 | 0.951725 | -0.08633 | 0.091835 | EUR | Pain, swelling or discharge of eye | sense organs |
| 286.81 | -0.00378 | 0.063044 | -0.06 | 0.952159 | -0.12735 | 0.119781 | EUR | Primary hypercoagulable state | hematopoietic |
| 209 | -0.00644 | 0.107585 | -0.05982 | 0.952303 | -0.2173 | 0.204427 | EUR | Neuroendocrine tumors | neoplasms |
| 384 | -0.00616 | 0.105287 | -0.05853 | 0.953324 | -0.21252 | 0.200196 | EUR | Other disorders of tympanic membrane | sense organs |
| 414 | 0.002576 | 0.044036 | 0.058491 | 0.953357 | -0.08373 | 0.088884 | EUR | Other forms of chronic heart disease | circulatory system |
| 362.3 | 0.006199 | 0.106196 | 0.058369 | 0.953455 | -0.20194 | 0.214339 | EUR | Other nondiabetic retinopathy | sense organs |
| 420.21 | 0.007476 | 0.129155 | 0.057881 | 0.953844 | -0.24566 | 0.260615 | EUR | Acute pericarditis | circulatory system |
| 772.4 | 0.008928 | 0.159228 | 0.056072 | 0.955284 | -0.30315 | 0.32101 | EUR | Rhabdomyolysis | symptoms |
| 293 | -0.00287 | 0.05124 | -0.05607 | 0.95529 | -0.1033 | 0.097555 | EUR | Symptoms involving head and neck | mental disorders |
| 260 | 0.002144 | 0.040049 | 0.053539 | 0.957302 | -0.07635 | 0.08064 | EUR | Protein-calorie malnutrition | endocrine/metabolic |
| 260.22 | 0.009636 | 0.180763 | 0.053307 | 0.957487 | -0.34465 | 0.363926 | EUR | Nutritional marasmus | endocrine/metabolic |
| 446.4 | 0.012096 | 0.228391 | 0.05296 | 0.957764 | -0.43554 | 0.459734 | EUR | Wegener's granulomatosis | circulatory system |
| 574.3 | -0.00445 | 0.084625 | -0.05256 | 0.958085 | -0.17031 | 0.161414 | EUR | Cholecystitis without cholelithiasis | digestive |
| 272.13 | -0.00151 | 0.028858 | -0.0522 | 0.958366 | -0.05807 | 0.055054 | EUR | Mixed hyperlipidemia | endocrine/metabolic |
| 792.1 | -0.00296 | 0.056768 | -0.05216 | 0.9584 | -0.11422 | 0.108302 | EUR | Papanicolaou smear of cervix or vagina with atypical squamous cells | genitourinary |
| 526 | 0.002374 | 0.045828 | 0.051804 | 0.958685 | -0.08745 | 0.092195 | EUR | Diseases of the jaws | digestive |
| 803 | -0.00229 | 0.044979 | -0.05087 | 0.95943 | -0.09044 | 0.085868 | EUR | Fracture of upper limb | injuries & poisonings |
| 394.2 | 0.003026 | 0.061421 | 0.04927 | 0.960704 | -0.11736 | 0.123409 | EUR | Mitral valve disease | circulatory system |
| 850 | 0.003035 | 0.064861 | 0.046792 | 0.962679 | -0.12409 | 0.13016 | EUR | Hemorrhage or hematoma complicating a procedure | injuries & poisonings |
| 70.2 | 0.005808 | 0.124265 | 0.04674 | 0.962721 | -0.23775 | 0.249362 | EUR | Viral hepatitis B | infectious diseases |
| 591 | -0.00118 | 0.025769 | -0.04562 | 0.963614 | -0.05168 | 0.049331 | EUR | Urinary tract infection | genitourinary |
| 700 | -0.00287 | 0.063403 | -0.04531 | 0.963862 | -0.12714 | 0.121395 | EUR | Corns and callosities | dermatologic |
| 613.8 | -0.00233 | 0.052092 | -0.04464 | 0.964392 | -0.10442 | 0.099773 | EUR | Other specified disorders of breast | genitourinary |
| 292.6 | -0.00521 | 0.12391 | -0.04207 | 0.966442 | -0.24807 | 0.237647 | EUR | Hallucinations | mental disorders |
| 196 | 0.000962 | 0.023164 | 0.041514 | 0.966886 | -0.04444 | 0.046362 | EUR | Radiotherapy | neoplasms |
| 300.4 | -0.00188 | 0.047072 | -0.04001 | 0.968082 | -0.09414 | 0.090376 | EUR | Dysthymic disorder | mental disorders |
| 687 | -0.00111 | 0.028005 | -0.03967 | 0.968357 | -0.056 | 0.053778 | EUR | Symptoms affecting skin | dermatologic |
| 796 | -0.0018 | 0.045658 | -0.03952 | 0.968478 | -0.09129 | 0.087683 | EUR | Elevated prostate specific antigen [PSA] | genitourinary |
| 614.54 | -0.0067 | 0.178775 | -0.03745 | 0.970126 | -0.35709 | 0.343697 | EUR | Abscess or ulceration of vulva | genitourinary |
| 973 | -0.02056 | 0.589731 | -0.03487 | 0.972187 | -1.17641 | 1.13529 | EUR | Poisoning by agents primarily affecting the gastrointestinal system | injuries & poisonings |
| 586 | 0.000929 | 0.027488 | 0.033778 | 0.973054 | -0.05295 | 0.054804 | EUR | Other disorders of the kidney and ureters | genitourinary |
| 427.1 | 0.001129 | 0.033452 | 0.033748 | 0.973078 | -0.06444 | 0.066693 | EUR | Paroxysmal tachycardia, unspecified | circulatory system |
| 594.2 | -0.00334 | 0.101078 | -0.03306 | 0.973626 | -0.20145 | 0.194767 | EUR | Calculus of lower urinary tract | genitourinary |
| 750 | 0.001605 | 0.052314 | 0.030686 | 0.97552 | -0.10093 | 0.104139 | EUR | Digestive congenital anomalies | congenital anomalies |
| 726.3 | 0.001475 | 0.049517 | 0.029791 | 0.976234 | -0.09558 | 0.098527 | EUR | Bursitis | musculoskeletal |
| 348.4 | 0.003972 | 0.135503 | 0.029311 | 0.976617 | -0.26161 | 0.269553 | EUR | Cerebral cysts | neurological |
| 709.4 | -0.00766 | 0.262708 | -0.02916 | 0.976733 | -0.52256 | 0.507237 | EUR | Polymyositis | dermatologic |
| 711 | 0.002885 | 0.104935 | 0.02749 | 0.978069 | -0.20278 | 0.208554 | EUR | Arthropathy associated with infections | musculoskeletal |
| 519.9 | 0.001162 | 0.043399 | 0.026774 | 0.97864 | -0.0839 | 0.086223 | EUR | Symptoms involving respiratory system and other chest symptoms | respiratory |
| 960.2 | 0.000622 | 0.029977 | 0.020757 | 0.983439 | -0.05813 | 0.059377 | EUR | Allergy/adverse effect of penicillin | injuries & poisonings |
| 728 | 0.001688 | 0.0923 | 0.018286 | 0.985411 | -0.17922 | 0.182592 | EUR | Disorders of muscle, ligament, and fascia | musculoskeletal |
| 276.12 | 0.000632 | 0.035792 | 0.017646 | 0.985921 | -0.06952 | 0.070783 | EUR | Hyposmolality and/or hyponatremia | endocrine/metabolic |
| 286.13 | 0.00503 | 0.285281 | 0.01763 | 0.985934 | -0.55411 | 0.564169 | EUR | Congenital factor VIII disorder | hematopoietic |
| 253.7 | -0.00152 | 0.087054 | -0.01741 | 0.986111 | -0.17214 | 0.169107 | EUR | Other disorders of neurohypophysis | endocrine/metabolic |

| phecode | Coef. | Std.Err. | z | p_value_z | [0.025 | 0.975] | GIA | phenotype | category |
|---|---|---|---|---|---|---|---|---|---|
| 10 | 0.002131 | 0.126093 | 0.016898 | 9.87E-01 | -0.24501 | 0.249268 | EUR | Tuberculosis | infectious diseases |
| 255.13 | 0.021274 | 1.267836 | 0.01678 | 0.986612 | -2.46364 | 2.506186 | EUR | Medulloadrenal hyperfunction | endocrine/metabolic |
| 306 | 0.000349 | 0.021965 | 0.015888 | 0.987323 | -0.0427 | 0.0434 | EUR | Other mental disorder | mental disorders |
| 709.5 | 0.002842 | 0.179854 | 0.015804 | 0.987391 | -0.34966 | 0.355349 | EUR | Dermatomyositis | dermatologic |
| 375.2 | -0.00129 | 0.081842 | -0.01573 | 0.987448 | -0.16169 | 0.159119 | EUR | Epiphora | sense organs |
| 274.1 | 0.00077 | 0.04967 | 0.015501 | 0.987633 | -0.09658 | 0.098122 | EUR | Gout | endocrine/metabolic |
| 465.4 | -0.00124 | 0.080751 | -0.01535 | 0.98775 | -0.15951 | 0.157029 | EUR | Acute laryngitis and tracheitis | respiratory |
| 38.1 | 0.00103 | 0.068958 | 0.014933 | 9.88E-01 | -0.13413 | 0.136186 | EUR | Gram negative septicemia | infectious diseases |
| 585.31 | 0.000801 | 0.054385 | 0.014726 | 0.988251 | -0.10579 | 0.107394 | EUR | Renal dialysis | genitourinary |
| 426.25 | 0.001912 | 0.133792 | 0.014293 | 0.988596 | -0.26031 | 0.264139 | EUR | Other heart block | circulatory system |
| 70.9 | -0.00091 | 0.067349 | -0.01358 | 0.989166 | -0.13292 | 0.131088 | EUR | Hepatitis NOS | infectious diseases |
| 620 | 0.001122 | 0.119148 | 0.009416 | 0.992487 | -0.2324 | 0.234647 | EUR | Dysplasia of female genital organs | genitourinary |
| 819 | 0.000552 | 0.06038 | 0.009145 | 0.992703 | -0.11779 | 0.118895 | EUR | Skull and face fracture and other intercranial injury | injuries & poisonings |
| 966 | 0.001669 | 0.443351 | 0.003765 | 0.996996 | -0.86728 | 0.870622 | EUR | Poisoning by anticonvulsants and anti-Parkinsonism drugs | injuries & poisonings |
| 656.2 | 0.001275 | 0.493602 | 0.002584 | 0.997939 | -0.96617 | 0.968718 | EUR | Respiratory conditions of fetus and newborn | pregnancy complications |
| 747.12 | -0.00015 | 0.071623 | -0.00209 | 0.998335 | -0.14053 | 0.140229 | EUR | Valvular heart disease/ heart chambers | congenital anomalies |
