# Supplementary material for: Polygenic scores for tobacco use provide insights into systemic health risks in a diverse EHR-linked biobank in Los Angeles
Source: Transl Psychiatry. 2024 Jan 18;14:38. doi: 10.1038/s41398-024-02743-z (PMC10796315; doi:10.1038/s41398-024-02743-z)
Supplement: Supplementary file 4 — Supplementary material Part 2 [file 41398_2024_2743_MOESM4_ESM.pdf]

Quantile	Coef.	Std.Err.	z	P>|z|	[0.025	0.975]	phecode	Smoking Histor	or	or_lower_ci	or_upper_ci	phenotype
2	0.003838	0.050713	0.075675	9.40E-01	-0.095559	0.103234	278.1	all	1.003845375	0.9088647371	1.108750826	Obesity
3	0.164329	0.04967	3.308406	9.38E-04	0.066977	0.261681	278.1	all	1.178602011	1.069270885	1.29911206	Obesity
4	0.191586	0.04956	3.865708	1.11E-04	0.094449	0.288722	278.1	all	1.211168989	1.09905311	1.334720625	Obesity
5	0.275738	0.04915	5.61017	2.02E-08	0.179407	0.37207	278.1	all	1.317502633	1.196507624	1.450734529	Obesity
2	0.044565	0.08908	0.500286	6.17E-01	-0.130028	0.219159	278.1	smokers	1.045572937	0.8780708446	1.245029221	Obesity
3	0.112361	0.087061	1.290593	1.97E-01	-0.058276	0.282998	278.1	smokers	1.118916717	0.943389536	1.327102508	Obesity
4	0.233316	0.085472	2.729721	6.34E-03	0.065793	0.400839	278.1	smokers	1.262780455	1.068005617	1.493076864	Obesity
5	0.158136	0.084782	1.865219	6.22E-02	-0.008033	0.324305	278.1	smokers	1.171325484	0.9919991783	1.383069079	Obesity
2	-0.024439	0.061867	-0.395021	6.93E-01	-0.145695	0.096818	278.1	never smokers	0.9758572144	0.8644213115	1.101659853	Obesity
3	0.178675	0.060655	2.94576	3.22E-03	0.059793	0.297556	278.1	never smokers	1.195632101	1.061616769	1.346563781	Obesity
4	0.146535	0.061205	2.394165	1.67E-02	0.026575	0.266494	278.1	never smokers	1.157815454	1.026931264	1.305379757	Obesity
5	0.323672	0.060706	5.331753	9.73E-08	0.20469	0.442655	278.1	never smokers	1.382193873	1.227144591	1.556835134	Obesity
2	0.060156	0.083029	0.724517	4.69E-01	-0.102579	0.222891	317	all	1.062002206	0.9025068489	1.249684351	Alcohol Related Disorders
3	0.114164	0.081872	1.39442	1.63E-01	-0.046302	0.27463	317	all	1.120935943	0.9547535831	1.316043649	Alcohol Related Disorders
4	0.14942	0.081282	1.838293	6.60E-02	-0.00989	0.30873	317	all	1.161160574	0.9901587452	1.361694663	Alcohol Related Disorders
5	0.331898	0.078638	4.220568	2.44E-05	0.17777	0.486026	317	all	1.393610693	1.194550543	1.625842268	Alcohol Related Disorders
2	-0.05487	0.117757	-0.465964	6.41E-01	-0.28567	0.175929	317	smokers	0.946608199	0.7515105735	1.192353399	Alcohol Related Disorders
3	0.07488	0.113284	0.660992	5.09E-01	-0.147153	0.296913	317	smokers	1.077754813	0.8631619035	1.345698219	Alcohol Related Disorders
4	-0.043004	0.114628	-0.37516	7.08E-01	-0.267672	0.181664	317	smokers	0.9579075584	0.765158712	1.199211191	Alcohol Related Disorders
5	0.108429	0.110009	0.985632	3.24E-01	-0.107186	0.324043	317	smokers	1.114525774	0.8983585628	1.382706762	Alcohol Related Disorders
2	0.113096	0.119021	0.950224	3.42E-01	-0.12018	0.346372	317	never smokers	1.119739423	0.8867608054	1.413928499	Alcohol Related Disorders
3	0.03311	0.121626	0.272224	7.85E-01	-0.205273	0.271493	317	never smokers	1.033664236	0.814424948	1.311921688	Alcohol Related Disorders
4	0.216821	0.117171	1.850473	6.42E-02	-0.012829	0.446472	317	never smokers	1.242121742	0.9872529408	1.562788929	Alcohol Related Disorders
5	0.386099	0.114721	3.365558	7.64E-04	0.16125	0.610947	317	never smokers	1.471230315	1.174978677	1.842175113	Alcohol Related Disorders
2	0.083422	0.114189	0.730559	4.65E-01	-0.140385	0.307229	165.1	all	1.087000426	0.8690235969	1.359652293	Lung Cancer
3	0.132885	0.112847	1.177564	2.39E-01	-0.088292	0.354062	165.1	all	1.142118647	0.9154935136	1.424843524	Lung Cancer
4	0.013369	0.116296	0.114958	9.08E-01	-0.214566	0.241304	165.1	all	1.013458765	0.8068915551	1.272907941	Lung Cancer
5	0.069135	0.115643	0.597834	5.50E-01	-0.15752	0.295791	165.1	all	1.071580863	0.8542597282	1.344189192	Lung Cancer
2	0.044932	0.162265	0.276906	7.82E-01	-0.273102	0.362966	165.1	smokers	1.045956732	0.7610151601	1.43758698	Lung Cancer
3	0.243666	0.154625	1.575856	1.15E-01	-0.059393	0.546725	165.1	smokers	1.275918103	0.9423363582	1.727585899	Lung Cancer
4	0.038054	0.160849	0.236582	8.13E-01	-0.277204	0.353312	165.1	smokers	1.038787326	0.7578998698	1.423775292	Lung Cancer
5	0.11764	0.156387	0.752236	4.52E-01	-0.188872	0.424152	165.1	smokers	1.124839096	0.8278924701	1.528293877	Lung Cancer
2	0.080711	0.161667	0.499242	6.18E-01	-0.236151	0.397573	165.1	never smokers	1.084057559	0.7896614261	1.488208429	Lung Cancer
3	-0.079049	0.169257	-0.467037	6.40E-01	-0.410787	0.252688	165.1	never smokers	0.9239946476	0.6631281629	1.28748152	Lung Cancer
4	-0.092631	0.170937	-0.541903	5.88E-01	-0.427663	0.2424	165.1	never smokers	0.9115297927	0.6520311123	1.274303812	Lung Cancer
5	-0.156199	0.178317	-0.875959	3.81E-01	-0.505694	0.193297	165.1	never smokers	0.855388951	0.6030868879	1.213243073	Lung Cancer

id.exposure	id.outcome	outcome	exposure	method	nsnp	b	se	pval	Lower	Upper
ukb-b-9405	ieu-b-142	Cigarettes smoked per day || id:	Waist circumference	MR Egger	354	0.2741497268	0.08960154622	0.00238592461	0.09853069625	0.4497687574
ukb-b-9405	ieu-b-142	Cigarettes smoked per day || id:	Waist circumference	Weighted medi	354	0.448117121	0.037719681	1.50E-32	0.3741865463	0.5220476958
ukb-b-9405	ieu-b-142	Cigarettes smoked per day || id:	Waist circumference	Inverse varianc	354	0.4511343936	0.0314887711	1.49E-46	0.3894164022	0.5128523849
ukb-b-9405	ieu-b-142	Cigarettes smoked per day || id:	Waist circumference	Simple mode	354	0.5220078374	0.1310167226	8.22E-05	0.2652150612	0.7788006136
ukb-b-9405	ieu-b-142	Cigarettes smoked per day || id:	Waist circumference	Weighted mode	354	0.5540653322	0.09653984117	2.05E-08	0.3648472435	0.7432834208
ukb-b-19953	ieu-b-142	Cigarettes smoked per day || id:	Body mass index	MR Egger	430	0.2046630127	0.06768810718	0.00264793439	0.07199432266	0.3373317028
ukb-b-19953	ieu-b-142	Cigarettes smoked per day || id:	Body mass index	Weighted medi	430	0.3511669783	0.02943765723	8.34E-33	0.2934691701	0.4088647864
ukb-b-19953	ieu-b-142	Cigarettes smoked per day || id:	Body mass index	Inverse varianc	430	0.3551980315	0.02504452269	1.17E-45	0.306110767	0.404285296
ukb-b-19953	ieu-b-142	Cigarettes smoked per day || id:	Body mass index	Simple mode	430	0.4843041676	0.1072896744	8.23E-06	0.2740164058	0.6945919293
ukb-b-19953	ieu-b-142	Cigarettes smoked per day || id:	Body mass index	Weighted mode	430	0.4290785262	0.08454273134	5.78E-07	0.2633747728	0.5947822796
**REVERSE DIRECTION**										
**id.exposure**	**id.outcome**	**outcome**	**exposure**	**method**	**nsnp**	**b**	**se**	**pval**	**Lower**	**Upper**
ieu-b-142	ukb-b-9405	Waist circumference	Cigarettes smoked per day || ic	MR Egger	22	-0.03696704743	0.03270202039	2.72E-01	-0.1010630074	0.02712891253
ieu-b-142	ukb-b-9405	Waist circumference	Cigarettes smoked per day || ic	Weighted mediar	22	0.0119501802	0.01328593298	0.3684070946	-0.01409024844	0.03799060884
ieu-b-142	ukb-b-9405	Waist circumference	Cigarettes smoked per day || ic	Inverse variance	22	0.04472660161	0.02200973562	0.04214069218	0.001587519793	0.08786568343
ieu-b-142	ukb-b-9405	Waist circumference	Cigarettes smoked per day || ic	Simple mode	22	0.1319009835	0.04993650265	0.0152643439	0.03402543834	0.2297765287
ieu-b-142	ukb-b-9405	Waist circumference	Cigarettes smoked per day || ic	Weighted mode	22	0.000286467916	0.01080535755	0.9790995618	-0.02089203287	0.02146496871
ieu-b-142	ukb-b-19953	Body mass index	Cigarettes smoked per day || ic	MR Egger	22	-0.04274165623	0.03721013405	0.2642507127	-0.115673519	0.03019020651
ieu-b-142	ukb-b-19953	Body mass index	Cigarettes smoked per day || ic	Weighted mediar	22	0.000899117347	0.01500598675	0.952221547	-0.02851261668	0.03031085138
ieu-b-142	ukb-b-19953	Body mass index	Cigarettes smoked per day || ic	Inverse variance	22	0.04517289476	0.02462039268	0.06653895132	-0.00308307489	0.0934288644
ieu-b-142	ukb-b-19953	Body mass index	Cigarettes smoked per day || ic	Simple mode	22	0.1306854554	0.0594371208	0.03924346467	0.01418869864	0.2471822122
ieu-b-142	ukb-b-19953	Body mass index	Cigarettes smoked per day || ic	Weighted mode	22	-0.00012287103	0.01208497762	0.9919838162	-0.02380942717	0.0235636851
